# Multi-Omic Profiling of *Melophlus* Sponges Reveals Diverse Metabolomic and Microbiome Architectures that Are Non-overlapping with Ecological Neighbors

**DOI:** 10.3390/md18020124

**Published:** 2020-02-19

**Authors:** Ipsita Mohanty, Sheila Podell, Jason S. Biggs, Neha Garg, Eric E. Allen, Vinayak Agarwal

**Affiliations:** 1 School of Chemistry and Biochemistry, Georgia Institute of Technology, Atlanta, GA 30332, USA; mohantyipsita92@gatech.edu (I.M.); neha.garg@chemistry.gatech.edu (N.G.); 2 Marine Biology Research Division, Scripps Institution of Oceanography, University of California, San Diego, La Jolla, CA 92093, USA; spodell@ucsd.edu (S.P.); eallen@ucsd.edu (E.E.A.); 3 University of Guam Marine Laboratory, UOG Station, Mangilao 96913, Guam; biggs.js@gmail.com; 4 Division of Biological Sciences, University of California, San Diego, La Jolla, CA 92093, USA; 5 School of Biological Sciences, Georgia Institute of Technology, Atlanta, GA 30332, USA

**Keywords:** sponge, natural product, glycosylation, metabolomics, metagenomics

## Abstract

Marine sponge holobionts, defined as filter-feeding sponge hosts together with their associated microbiomes, are prolific sources of natural products. The inventory of natural products that have been isolated from marine sponges is extensive. Here, using untargeted mass spectrometry, we demonstrate that sponges harbor a far greater diversity of low-abundance natural products that have evaded discovery. While these low-abundance natural products may not be feasible to isolate, insights into their chemical structures can be gleaned by careful curation of mass fragmentation spectra. Sponges are also some of the most complex, multi-organismal holobiont communities in the oceans. We overlay sponge metabolomes with their microbiome structures and detailed metagenomic characterization to discover candidate gene clusters that encode production of sponge-derived natural products. The multi-omic profiling strategy for sponges that we describe here enables quantitative comparison of sponge metabolomes and microbiomes to address, among other questions, the ecological relevance of sponge natural products and for the phylochemical assignment of previously undescribed sponge identities.

## 1. Introduction

Sponges are filter-feeding sessile invertebrates that are present at all latitudes in the oceans. High rates of water filtration, up to several thousand times the sponge volume per day, make sponges primary players in the benthic-pelagic nutrient recirculation [1,2]. Sponges host a diverse bacterial microbiome and among high-microbial-abundance sponges such as those discussed in this study, the microbial population can comprise up to 35% weight of the sponge [3,4]. In addition to bacteria, sponges also host eukaryotic partners such as fungi and dinoflagellates [5]. Thus, from a molecular perspective, sponges can be conceptualized as a community of multiple interacting partners, referred to here as holobionts. In the marine environment, sponge holobionts are among the richest sources of specialized metabolites, colloquially termed natural products. The complexity of sponge natural product chemical structures has offered title compounds for total synthesis and catalyst development, and several sponge-derived molecules have progressed through pharmaceutical development pipelines to be approved for clinical use [6,7]. Uncoupled from these motivations, natural products are postulated to serve core ecological functions such as defending the sponge against predation and infection and shaping the structure of the benthic community [8]. Using next-generation sequencing and assembly of bacterial genomes from metagenomic data, it is well established that members of the sponge microbiome are responsible for the biogenesis of several sponge-derived natural products [9].

Traditionally, sponge natural products have been inventoried using isolation of individual molecules and structural elucidation using a combination of experimental and theoretical approaches. These efforts have built a foundation of more than 11,000 sponge-derived natural product structures [10]. The application of untargeted mass spectrometry has revealed that the natural product diversity from sponges has been severely underappreciated [11]. The analytical challenge, now, is two-fold: first, to gain structural insights into metabolites that due to their lesser abundance and the complexity of the sponge metabolomes will remain inaccessible to traditional spectroscopic characterization; second, to advance the characterization of sponge extracts from disjointed descriptions of individual natural products to an overall metabolomic description of the holobiont community. These two advances, when realized, will allow for comparative analyses to determine which fractions of sponge metabolomes stay conserved, or diverge, in response to ecological, pharmacological, and/or other metrics. Furthermore, sponge metabolomes could then be overlaid with the holobiont microbial community structures to determine how the overall holobiont community shapes, and responds to, differences in sponge metabolomes. Correlative conservation between metabolomic features and microbiome structures aid in metagenomic mining to search for natural product biosynthetic gene loci in the sponge holobiont. 

In this study, we couple the metabolomic and metagenomic characterizations of marine sponges of the genus *Melophlus* that were collected in Guam (Apra Harbor). The *Melophlus* genus is typified by the large barrel-shaped sponge *Melophlus sarasinorum* (previously called *Asteropus sarasinorum*) that is widely distributed in the Indo-Pacific with natural product isolation studies realized from Indonesia, Palau, Guam, and the Solomon Islands. In this study, DNA barcoding, microbiome composition, and metabolomic comparison places an additional, morphologically unrecognizable sponge species within the *Melophlus* genus. We demonstrate that the chemical diversity of natural products in the *Melophlus* metabolome far exceeds the current inventory. Using metagenomics, we discover possible genetic routes for the production of characteristic *Melophlus* natural products. Aided by structural insights from mass spectrometric fragmentation, we detect the presence of new natural product classes previously unknown from *Melophlus* sponges. Finally, we compare and contrast the microbiome and metabolome architectures of dominant and abundant sponge genera in close geographical neighborhoods of each other to demonstrate that a shared ecological environment does not necessarily translate to metabolomic and microbiological overlap. Our findings demonstrate the applicability of contemporary omic technologies in providing a detailed chemical, genetic, and microbiological characterization of sponge holobionts.

## 2. Results

### 2.1. Sponge Phylogeny and Microbiome

**Figure 1:**
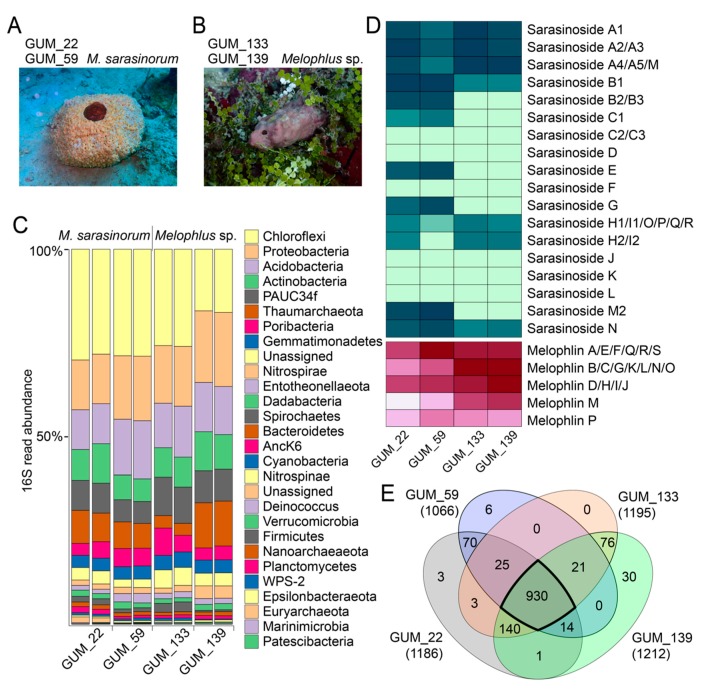
Metabolomes and microbiomes of *Melophlus* sponges. Morphology of (**A**) *Melophlus sarasinorum* and (**B**) *Melophlus* sp. sponges collected in Apra Harbor, Guam. (**C**) Microbiome composition, at the phylum level, for sponge specimens used in this study. Each sample was sequenced in duplicate. (**D**) Relative abundances of sarasinosides and melophlin congeners among sponge specimens used in this study. Darker color denotes higher abundance. (**E**) Overlap of metabolomic features among sponge specimens used in this study. Features that are shared by all four *Melophlus* specimens used in this study are highlighted.

The sponge *M. sarasinorum* was identified by numerous small ostia distributed around a large central osculum on a yellow, round ultrastructure at the Apra Harbor in Guam (Figure 1A). Two biological replicate specimens were collected, GUM_22 and GUM_59. Specimens were frozen dry, for chemical analyses, and in preserving solution RNA*later*, for DNA extraction. Sponge specimens were barcoded by Sanger sequencing of PCR amplicons corresponding to the internal transcribed spacer-2 (ITS-2) region between 5.8S and 28S rRNA encoding genes, and the 5’ terminus of the 28S rRNA encoding gene (Figure S1). BLAST against the GenBank nr database identified that sequences with the highest similarity corresponded to the *Melophlus* genera [12,13]. 

A different sponge morphology, for which biological replicates GUM_133 and GUM_139 were collected, was not immediately recognizable (Figure 1B). The GUM_133 and GUM_139 28S rRNA gene amplicons, only 40% similar to GUM_22 and GUM_59, had the greatest similarity to *Theonella* genus sequences. However, the ITS-2 amplicon sequences were identical between the two groups, GUM_22/GUM_59 and GUM_133/GUM_139 (Table S1). Next, we determined the microbiome composition of all sponge specimens in duplicate, using next-generation sequencing of the v4 region of the 16S rRNA gene PCR amplicons and differentiated amplicon sequence variants (ASVs). Taxonomic assignment to the phylum level reveals that microbiome architectures between the GUM_22/GUM_59 and GUM_133/GUM_139 specimens were highly similar, with Chloroflexi, Proteobacteria, Acidobacteria, and Actinobacteria being the dominant bacterial phyla (Figure 1C). Given the high microbiome and metabolomic similarity (vide infra) between the two groups of sponges, we propose that GUM_133 and GUM_139 may constitute a single species among the *Melophlus* genus. 

### 2.2. Overall Metabolomic Description

Untargeted liquid chromatography/high-resolution time-of-flight mass spectrometry (LC/MS) data were acquired with no prior derivatization or fractionation of organic extracts generated from sponge tissues. Each sponge extract was analyzed in duplicate. Of the *Melophlus*-derived natural products, two natural product families dominate. These are the glycosylated sterol molecules grouped as sarasinosides, and the polyketide/fatty acid tetramate conjugates- melophlins [14,15,16,17,18,19,20]. Manually curating previously described sarasinosides and melophlins, first, we dereplicated identities of already known congeners (Tables S2 and S3). Nearly all known sarasinosides and melophlins could be identified. Together with the LC/MS datasets, MS^2^ fragmentation spectra corresponding to sarasinoside and melophlin congeners were deposited to the Global Natural Products Social Molecular Networking (GNPS) database [21]. 

Next, metabolites detected in the LC/MS datasets were extracted as ‘features’ using the feature detection and alignment tool MZmine2 [22]. Extracted features comprise ion *m/z*s conjoined with MS^2^ spectra, and information corresponding to retention time and ion abundance is appended to them. Features were ‘deisotoped’, in that, only the most abundant isotopic ion is preserved in a feature. Using feature mining, we compared the relative abundances of known sarasinoside and melophlin congeners among the four sponge specimens (Figure 1D). The *M. sarasinorum* samples GUM_22/GUM_59 harbor a greater abundance and diversity of sarasinoside congeners while the *Melophlus* sp. samples GUM_133/GUM_139 were enriched for melophlins. In this approach, isomeric sarasinosides and melophlin congeners are grouped together as they cannot be easily differentiated based on MS^2^ fragmentation data alone. The overall metabolomes of *M. sarasinorum* and *Melophlus* sp. sponges were highly similar. Of the 1319 total unique features detected, 930 features (70.5%) are shared among the four sponge specimens (Figure 1E). Only 79 (6.0%) and 106 (8.0%) features were unique to *M. sarasinorum* and *Melophlus* sp. specimens, respectively. Of the 70 features that are shared among *M. sarasinorum* samples only, multiple features correspond to sarasinoside congeners, and of the 76 features shared among the *Melophlus* sp. samples only, features corresponding to melophlins can be detected (Table S4). 

### 2.3. Sarasinoside Chemical Diversity

**Figure 2:**
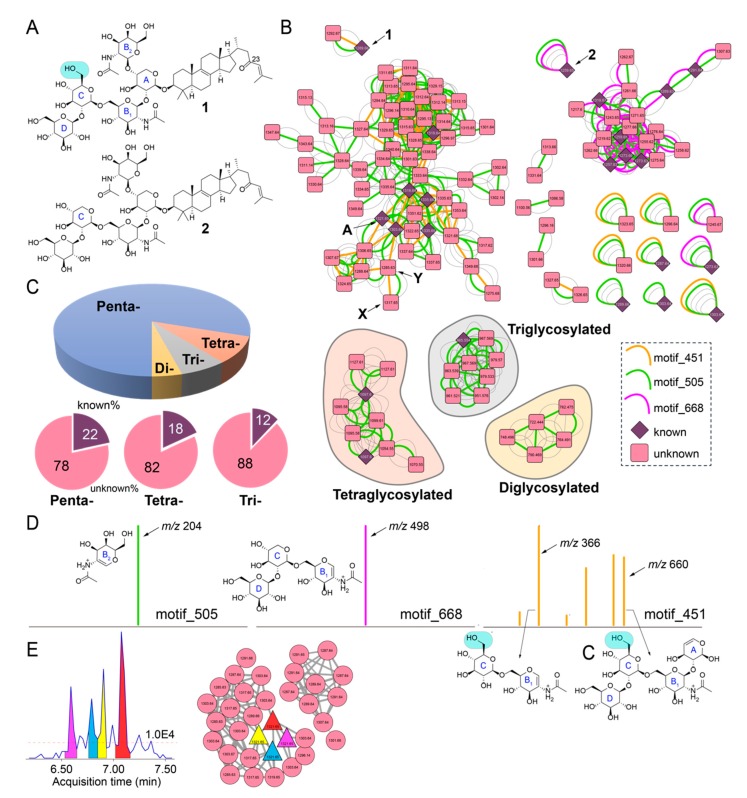
Sarasinoside chemical diversity revealed by mass spectrometry. (**A**) Chemical structures of sarasinoside A1 (**1**) and sarasinoside B1 (**2**). The glycosidic rings are labeled, note the difference in ring ‘C’ between the two structures. (**B**) Sarasinoside molecular network. The motif_451, motif_505, and motif_668 are shown in color. Subnetworks corresponding to tetra-, tri-, and diglycosylated sarasinosides are labeled. Relevance of nodes labeled **A**, **X**, and **Y** is described in text. (**C**) Distribution of nodes according to glycosylation pattern, and distribution of nodes for which chemical identities can be dereplicated (known) and for which identities are not known (unknown) according to glycosylation pattern. (**D**) Structural annotation of ions comprising motif_505 (left), and motif_668 (middle), and motif_451 (right). (**E**) Extracted ion chromatogram generated by MZmine2 demonstrating four features corresponding to *m/z* 1321.65. The red dashed line represents the MS^1^ threshold below which all features were discarded. Feature-based molecular networking (FBMN) demonstrating the clustering of the four features with other nodes corresponding to sarasinoside congeners.

The detection of sarasinoside A1 (**1**, Figure 2A) from *M. sarasinorum* has extensive precedent. To verify the identity of the natural product from *Melophlus* sp., we chromatographically isolated it from GUM_133 and acquired ^1^H and ^13^C NMR spectra. Comparison to literature data established the identity of the molecule isolated from GUM_133 as **1** (Figures S2 and S3; Table S5). Molecule **1** is representative of a polyglycosylated sterol (glycosyl moieties: A-xylose, B_1_-N-acetylglucosamine, B_2_-N-acetylgalactosamine, C,D-glucose). Ions observed in the MS^2^ spectra for **1**, which is dominated by glycosidic fragments, were structurally annotated (Figure S4). For sarasinoside B1 (**2**, Figure 2A), among other sarasinosides, the ‘C’ glycosyl moiety is changed from glucose to xylose, as compared to **1**. The C_23_-oxo group is invariant among sarasinosides. 

Next, we queried the chemical diversity of sarasinoside congeners detected in the LC/MS datasets using MS^2^ spectral matching-based molecular networking [21]. To connect nodes that remain disjointed by spectral overlap alone, spectral matching was supplemented with determination of spectral motifs by MS2LDA [23]. Here, MS2LDA motifs represent substructures characterized by discrete fragments and neutral losses present in MS^2^ spectra. Hence, nodes that are not connected in classical molecular network but contain a common substructure are searched using MS2LDA. A molecular network for sarasinoside congeners in which parent ions are represented as nodes that are connected by overlap of fragmentation spectra, as well as conservation of MS2LDA spectral motifs, is illustrated in Figure 2B. Nodes corresponding to **1** and **2** are labeled. Nodes for previously known sarasinoside congeners are shown as diamonds, and nodes for molecules that could not be dereplicated are shown as rounded squares. Based on previously described sarasinoside congeners, accurate mass, and manual annotation of MS^2^ fragmentation spectra, the network is divided among pentaglycosylated (unshaded), tetraglycosylated (shaded in brown), triglycosylated (gray), and diglycosylated sarasinosides (yellow). It is clear that pentaglycosylated congeners dominate the sarasinoside inventory (Figure 2C). Within each of these groups, the fraction of congeners that have been described in literature is minor. None of the diglycosylated sarasinosides were previously reported.

The node corresponding to **1** demonstrates the presence of multiple MS2LDA motifs (Figure 2B). Of these, motif_505, highlighted as a green edge, is conserved for each node in the sarasinoside molecular network (Figure 2B). The motif_505 is comprised of a single ion, *m/z* 204, which corresponds to the N-acetylhexose fragment generated by the cleavage of the glycosidic bond between A and B_2_ sugars (Figure 2D, left; complete inventory of MS2LDA motifs is provided as supplementary information). Conservation of motif_505 demonstrates that the A-B_2_ glycosylation pattern is conserved among all sarasinosides. Note that motif_505 now allows us to collate all sarasinoside nodes, including singletons that were otherwise disjointed by spectral overlap alone.

Nodes corresponding to **1** and **2** differ in the presence of motif_451 (brown edge, present only for **1**) and motif_668 (magenta edge, present only for **2**). Motifs _451 and _668 divide the pentaglycosylated sarasinosides into two subnetworks. The motif_668 comprises of a single MS^2^ fragment ion, *m/z* 498, the structure of which is rationalized to be the B_1_-C-D glycosidic chain for **2** (Figure 2D, middle). As the C glycosyl ring differs between **1** and **2** (glucose and xylose, respectively, Figure 2A), motif_668 is not conserved for **1**. Hence, nodes demonstrating the presence of motif_668 should possess the glycosidic pattern identical to **2**, and not **1**. Structural annotation of motif_451 reveals the presence of ions corresponding to the A-B_1_-C-D and B_1_-C glycosidic fragments in which the C ring corresponds to glucose (Figure 2D, right). Hence, motif_451 allows for determination of the glycosylation pattern for these nodes to be identical to that for **1**. It is instructive to observe that only a single methoxy group differentiates **1** and **2**, which is then differentially propagated by motifs _668 and _451 to classify pentaglycosylated sarasinoside into two distinct families.

The analyses presented above condenses parent ions within a 0.01 Da range into a single node. For instance, the node labeled ‘A’ in Figure 2B is comprised of 28 MS^2^ spectra spanning a 0.75 min retention time window (Table S6). Rather than a single sarasinoside congener, it is likely that the spread of retention time represents the presence of isomers. By combining classical molecular networking with feature mining by MZmine2, feature-based molecular networking (FBMN) allows for the separation of isomeric molecules as distinct features, because features incorporate an additional metric of retention time [24]. FBMN reveals that the sarasinoside chemical diversity extends even further than that illustrated in Figure 2B. For example, at least four distinct features corresponding to *m/z* 1321.65 can be detected (Figure 2E). Organizing these features in a molecular network reveals that they indeed cluster together, along with other sarasinoside congeners.

**Figure 3:**
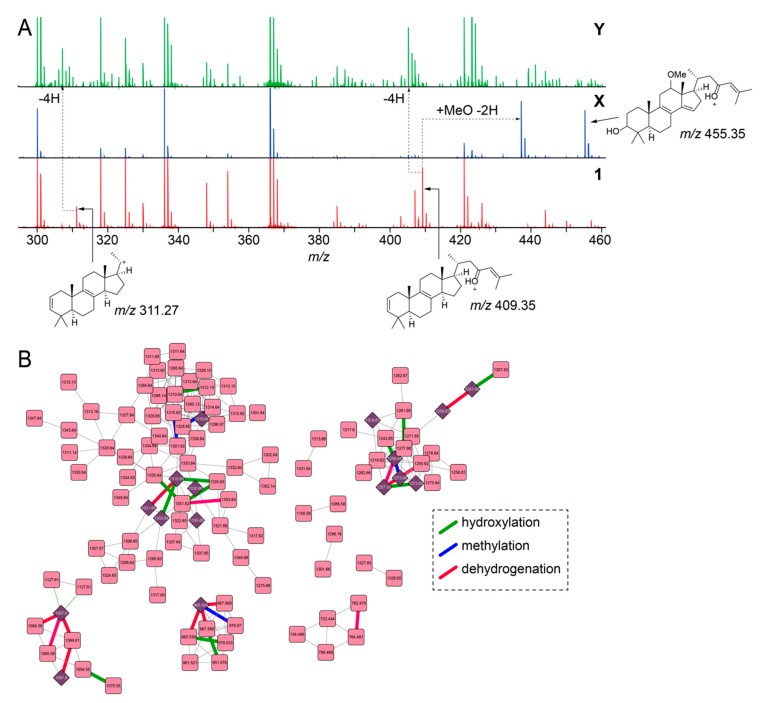
Characterization of low-abundance sarasinoside congeners. (**A**) Comparison of MS^2^ fragmentation spectra corresponding to nodes for **1**, **X**, and **Y** (as labeled in Figure 2B). Chemical structures for two principal fragment ions, *m/z* 311.27 and *m/z* 409.35 that are detected in the MS^2^ fragmentation spectra for **1** are shown. The ion m/z 455.35 for X denotes the methoxylated, dehydrogenated sterol, as compared to **1**, with the 3’-OH preserved. (**B**) Sarasinoside molecular network in which edges corresponding to characteristic mass differences, such as hydroxylation (green), methylation (blue), and dehydrogenation (red) are highlighted. Compared to the molecular network in Figure 2B, singleton nodes and edges corresponding to MS2LDA motifs are omitted for clarity.

Next, we focused on the determination of putative structures of unknown sarasinoside congeners. Illustratively, node labeled **X** in Figure 2B, [M + H]^1+^ *m/z* 1317.65, possesses an identical glycosylation pattern as **1** revealed by the conservation of motif_451 and motif_505. The mass increase of 27.99 Da between **X** and **1** corresponds to the addition of a methoxy group and a dehydrogenation as compared to **1** (+ MeO(− H) − 2H = 27.99 Da), as is evident by the comparison of the MS^2^ fragmentation ions for **1** and the sarasinoside congener corresponding to node **X** (Figure 3A). The sterol core of **1** is likely derived from the C_30_-demethylation of lanosterol which introduces the Δ^14,15^ unsaturation, as is observed in the structure of sarasinoside A3 (vide infra, Table S2). We posit that methoxylation at C_12_, as is observed in the structure of sarasinoside F, will furnish the natural product corresponding to node **X**. Likewise, for the node labeled **Y** in Figure 2B, [M + H]^1+^ *m/z* 1285.63, the glycosylation pattern is conserved as for **1**. The molecular formula thus predicted can only be satisfied by an unprecedented two addition dehydrogenations on the sterol core as compared to **1** (Figure 3A). Rather than traversing each node individually, we asked whether structural differences can be curated on the basis of accurate mass differences. We could indeed traverse nodes in the sarasinoside molecular network based on mass differences corresponding to common modifications such as hydroxylation (15.99 Da), methylation (14.01 Da), and dehydrogenation (2.01 Da) (Figure 3B). In light of the characterization of the glycosylation patterns, a convergent theme emerges in which tailoring of the sterol core emerges as the principal driver of sarasinoside chemical diversity.

### 2.4. Putative Sterol Biosynthetic Gene Clusters in the Sponge Microbiome

**Figure 4:**
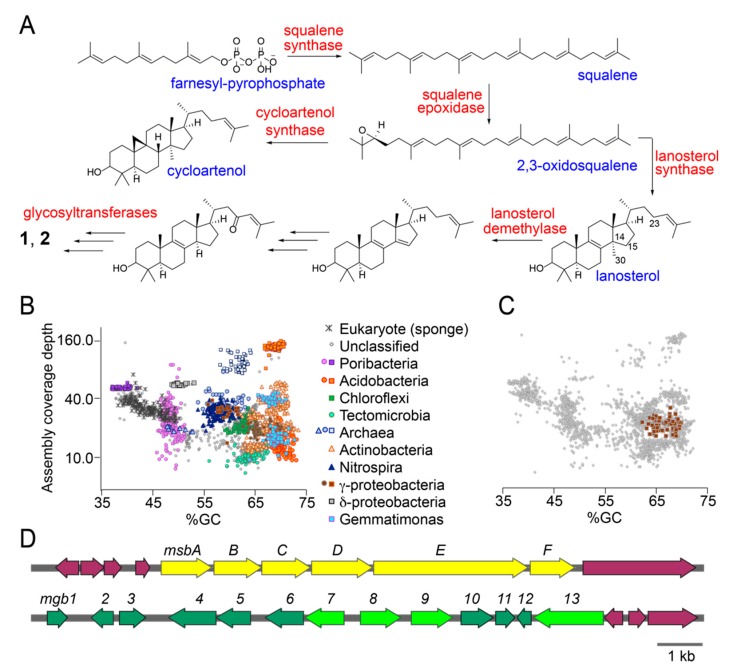
Sarasinoside biosynthetic potential in the *M. sarasinorum* metagenome. (**A**) Sterol biosynthetic scheme with key enzymes (in red) and intermediates (in blue). Note that 2,3-oxidosqualene can be transformed to either cycloartenol or lanosterol. Only lanosterol is relevant as a biosynthetic intermediate for the elaboration of sarasinosides. (**B**) All GUM_22 metagenomic scaffolds greater than 10 kb in length are displayed in grey. Colored points represent phylogenetically assigned scaffolds classified by amino acid sequence similarity of multiple predicted proteins to sequences from the GenBank nr database. (**C**) Tight clustering of the metagenomic contigs for the *γ*-proteobacterium that harbors the *msb* and *mgb* gene loci. (**D**) Metagenomic scaffolds bearing the *mgb* and *msb* loci. Protein products of neighboring genes, in purple, do not bear homology to sterol biosynthetic genes or sugar biosynthesis/transfer genes.

Next, we searched for the sarasinoside biosynthetic potential in the *M. sarasinorum* holobiont. Supported by literature, a hypothetical enzymatic scheme furnishing sarasinosides is illustrated in Figure 4A [25,26]. In this scheme, enzyme squalene synthase converts two molecules of farnesyl pyrophosphate to squalene. Squalene epoxidase then furnishes 2,3-oxidosqualene followed by conversion to lanosterol by lanosterol synthase. Alternatively, 2,3-oxidosqualene can also be converted to cycloartenol by cycloartenol synthase. Specific for sarasinosides, C_30_-lanosterol is likely demethylated by lanosterol demethylase which also introduces the Δ^14,15^ unsaturation. Redox tailoring to establish the C_23_-oxo modification and reduction of the Δ^14,15^ unsaturation furnishes the aglycone which is then glycosylated to yield **1** and **2**. The catalytic sequence for C_30_-demethylation and redox tailoring of the tetracyclic core can be reversed. Indeed, sterols bearing the C_23_-oxo moiety without C_30_-demethylation are detected from other marine sources [27,28]. In the event that the Δ^14,15^ unsaturation is not reduced, the aglycone corresponding to sarasinosides A3, B3, and C3 is produced (Table S2). Enzymatic glycosylation is expected to proceed via glycosyltransferase enzymes that employ sugar-diphosphate nucleosidic substrates. We used enzymes in this predictive biosynthetic scheme as diagnostic elements to mine the *M. sarasinorum* metagenome. 

Total DNA isolated from the GUM_22 holobiont was packaged into a 500 bp Illumina TruSeq DNA library and sequenced using the 2 × 150 bp paired end protocol on an Illumina HiSeq 2500 sequencer. Adaptor trimming and quality filtering yielded 105,560,704 high quality reads which were assembled into 58,828 scaffolds. Scaffolds were phylogenetically binned according to procedures that we have described previously [29,30]. Scaffold bins were organized into distinct clusters according to their phylogenetic derivation, which included sponge host as well as associated microbial taxa. Immediately, we could observe the clustering of scaffolds derived from different microbial taxa on a coverage (y-axis) vs %GC content (x-axis) plot (Figure 4B). Predicted protein functional annotations were obtained with Prokka [31]. Hidden Markov Model based searches were performed to identify candidate sterol biosynthetic enzymes using PFAM database patterns PF00494 (squalene synthase), PF08491 (squalene epoxidase), and PF13243 and PF13249 (squalene cyclase) [32]. Sequences encoding glycosyltransferase candidate enzymes were detected using PFAM database pattern PF00535 and CAZy classified search patterns for glucose, xylose, and N-acetylhexose transferases [33]. For each of these search criteria, individually, multiple genes were detected in the GUM_22 metagenome. For instance, 109 candidate squalene synthases and 32 squalene epoxidases were detected, along with 592 N-acetylhexose and 473 glucose/xylose glycosyltransferases. To sort the large number of potential hits that were dispersed across the metagenome, we queried whether multiple candidate sterol biosynthetic genes and glycosyltransferase-encoding genes were present together, within a single microbial taxon. Only a solitary *γ*-proteobacterium satisfied this criterion, the tight grouping of metagenomic scaffolds for which is demonstrated in Figure 4C. The metagenomically assembled genome (MAG) of this *γ*-proteobacterial symbiont, divided among 57 scaffolds, is judged by CheckM to be 95% complete [34]. The full length 16S rRNA gene sequence recovered from the MAG demonstrates its closest sequenced relatives to be uncultivated sponge symbionts. The sterol biosynthetic genes, annotated within the *Melophlus*-derived sterol biosynthesis (*msb*) gene cluster and glycosyltransferases, present in the *Melophlus*-derived glycosyl biosynthesis (*mgb*) gene cluster, can be identified from metagenomic scaffolds derived from this *γ*-proteobacterium (Figure 4D).

Within the *msb* locus, gene *msbE* encodes a bi-functional squalene epoxidase/sterol cyclase. Sequence alignment identifies a key valine residue in the C-terminal MsbE cyclase domain that allows for its annotation as a lanosterol cyclase, rather than an oxidosqualene cyclase (Figure 4A) [25]. Residues involved in coordinating the flavin cofactor can be discerned in the N-terminal epoxidase domain. Gene *msbC* encodes an integral membrane sterol reductase, homologs of which catalyze the reduction of the Δ^14,15^ unsaturation while protein product of gene *msbD* demonstrates high homology to the lanosterol C_30_-demethylase. Overlapping genes *msbA* and *msbB* encode a NAD(P)-dependent oxidoreductase and a Rieske oxygenase, respectively. Homologs of MsbA and MsbB have been demonstrated to catalyze redox tailoring of the sterol scaffold, such as oxidative demethylation of 4,4-gemdimethyl [35]. We posit that, among other activities, MsbA and MsbB could participate in the installation of the C_23_-oxo moiety. Gene *msbF* encodes a SAM-dependent sterol methyltransferase that could participate in the biosynthesis of methoxylated sarasinoside congeners such as sarasinoside F, H2, I2 (Table S2). Genes at either terminus of the *msb* gene locus described here bear no resemblance to sterol biosynthetic genes. Separated from the *msb* locus is the *mgb* locus. Genes *mgb7–9*,*13* all encode glycosyltransferases while genes *mgb2*,*4–6* encode enzymes involve in the biosynthesis and tailoring of sugar-diphosphate nucleosides. Gene *mgb1* encodes a transcriptional regulator. Other genes, such as *mgb10* and *mgb12* that encode SAM-dependent methyltransferases, have no readily discernable roles in sarasinoside biosynthesis. 

### 2.5. Mining for Other Glycosylated Molecules 

The abundance of glycosyltransferases identified in the *M. sarasinorum* metagenome translates to the *Melophlus* metabolomes being exceptionally enriched for glycosylated molecules. The MS2LDA motif_505, which corresponds to the *m/z* 204 fragment ion for N-acetylhexose moieties of **1** (Figure 2), alone, could be detected in 692 (28.7%) of the 2405 nodes in the molecular network for *Melophlus* LC/MS data used in this study. Glycosylated molecules are distributed across all four sponge specimens ranging in parent mass *m/z*s from 393.20 to 1889.98, implying that a wide diversity of structures undergo glycosylation in *Melophlus* holobionts.

**Figure 5:**
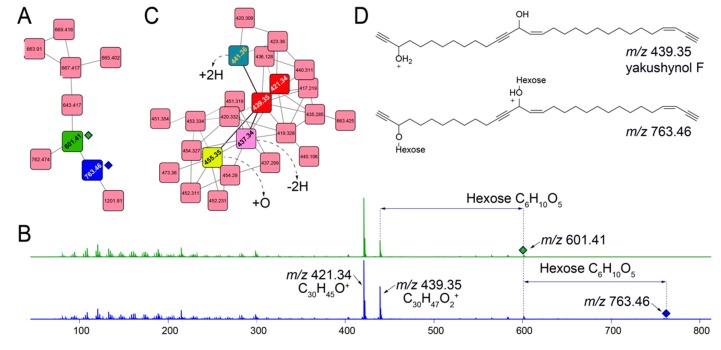
Other glycosylated molecules present in *Melophlus* metabolomes. (**A**) Network containing nodes for *m/z* 601.41 (in green) and *m/z* 763.46 (in blue) that correspond to mono- and diglycosylated C_30_ polyacetylinic natural products, respectively. (**B**) MS^2^ fragmentation spectra for *m/z* 601.41 (in green, top) and *m/z* 763.46 (in blue, bottom) demonstrating the neutral losses for one, and two hexose sugar moieties, respectively. The position of the parent ions is marked by diamonds. Two major fragment ions are detected, *m/z* 421.34 and *m/z* 439.35 that are annotated to the molecular formulae as labeled. The low-abundance fragment ions between 100 and 300 Da are characteristic of long alkyl chains, which, when fragmented, generate ions separated by methylene (14 Da) units. (**C**) Subnetwork containing nodes for *m/z* 421.34 and *m/z* 439.35 (in red). Progressing from *m/z* 439.35, connecting nodes can be annotated by rationalizing modifications, such as hydroxylation (+O, node in yellow), dehydrogenation (−2H, node in pink), and reduction (+2H, node in green). (**D**) Mining the MarinLit database for m/z 439.35 leads to the identification of yakushynol F as a potential structure which is supported by fragmentation pattern characteristic of long alkyl chains. Glycosylation at either, and both hydroxyls of yakushynol F will lead to products corresponding to *m/z* 601.41 and *m/z* 763.46, respectively.

Querying for other glycosylated molecules presents interesting results. For instance, for the subnetwork illustrated in Figure 5A, MS^2^ spectra for ions *m/z* 601.41 (green node) and *m/z* 763.46 (blue node) demonstrate neutral losses corresponding to one and two hexose moieties, respectively (Figure 5B). The presence of evenly distributed lower abundance fragmentation ions between 100 and 300 Da that differ by 14 Da points towards a long, linear alkyl chain. Higher abundance fragment ions, *m/z* 421.34 and *m/z* 439.35 correspond to the molecular formulae C_30_H_45_O^+^ and C_30_H_47_O_2_^+^, respectively, representing the aglycone structures for glycosylated parent ions *m/z* 601.41 and *m/z* 763.46. Mining the *Melophlus* metabolomes for *m/z* 421.34 and *m/z* 439.35 lead to the identification of another dedicated subnetwork in which molecular ions corresponding to these aglycone substructures are present (Figure 5C, nodes in red). Dereplicating these molecules using the MarinLit repository lead to hits to sponge-derived polyacetylenes with *m/z* 439.35 neatly corresponding to yakushynol F (Figure 5D) [36]. While the positions of the unsaturations can undoubtedly differ, this is the first instance in which polyacetylinic natural products have been detected from *Melophlus* sponges and to the best of our knowledge, glycosylated polyacetylenes from marine sponges overall [37]. Polyacetylinic natural products are widespread in marine metabolomes and based on accurate mass differences, other nodes in the subnetwork illustrated in Figure 5C can be rationalized as modifications such as hydroxylation (node 455.35), dehydrogenation (node 437.34), and reduction (node 441.36) of the C_30_ yakushynol F-like core structure. 

### 2.6. Diversity of Melophlin Natural Products

**Figure 6:**
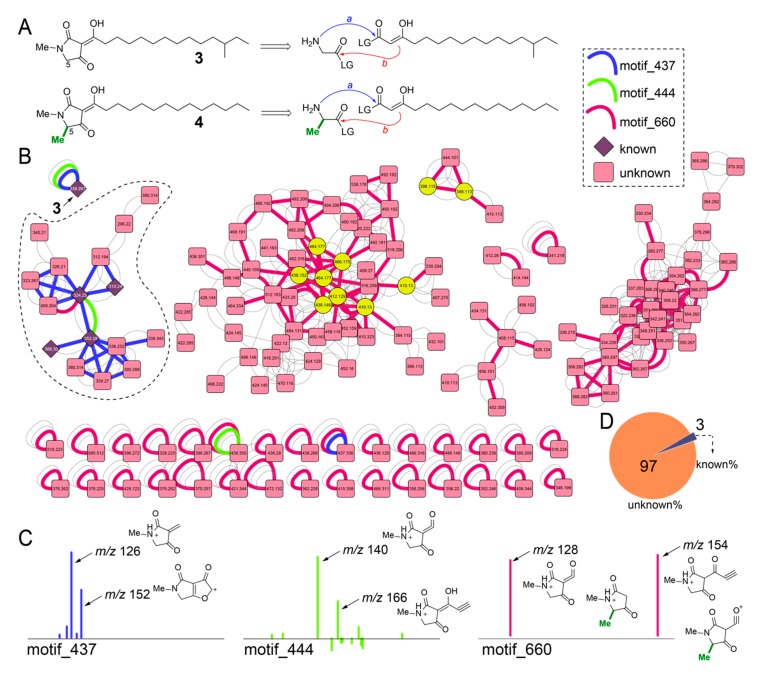
Inventory of melophlin congeners in sponge extracts. (**A**) Chemical structure of isomers **3** and **4**. Note the difference in methylation state at C5 between **3** and **4**. A retrobiosynthetic reaction sequence is shown in which the amino acid (glycine or alanine) primary amine condenses with the activated β-keto acid (shown as the enolate tautomer) progressing to Dieckmann cyclization which will furnish the tetramate heterocyclic core structure for melophlins. (**B**) Molecular network for melophlin congeners. The three MS2LDA motifs are highlighted as curved connecting edges with nodes corresponding to previously known melophlin congeners shown as diamonds and those corresponding to unknown brominated congeners shown as yellow circles. (**C**) Structural annotation of MS2 fragment ions comprising of motif_437, motif_444, and motif_660. Note that all fragment ions observed here constitute the tetramate heterocycle. (**D**) Distribution of known and unknown melophlin congeners quantified from panel B.

We inventoried the diversity of the other class of natural products previously described from *Melophlus* sponges, the long alkyl chain tetramates-melophlins. To verify the identity of melophlins from *Melophlus* sp. samples GUM_133/GUM_139, we isolated the most abundant melophlin congener present in the GUM_139 extract and acquired ^1^H and ^13^C NMR data (Figures S5 and S6). Comparison to literature data established that the isolated melophlin corresponded to melophlin I (**3**, Figure 6A) [20]. Melophlin congeners can be divided among two families, one with and the other without methylation at C_5_, such as the isomeric melophlin J (**4**, Table S3). Biosynthetically, this difference likely translates to the tetramate heterocycle being derived from the condensation of glycine (Figure 6A, top) or alanine (Figure 6A, bottom) primary amine with the activated carboxylic acid of a β-keto acid (path a, Figure 6A) followed by Dieckmann cyclization of the enolate with the aminoacyl activated carboxylic acid (path b) [38,39,40,41]. Spectroscopically, C_5_-methylated and C_5_-demethyl melophlins can be distinguished by ^13^C shifts at 62.7 and 57.7 ppm, respectively (in CDCl_3_) [20].

The MS^2^ spectra for **3** was structurally annotated (Figure S7). In the molecular network, node corresponding to **3** occurs as a singleton and is associated with motif_437 (blue edge, Figure 6B) and motif_444 (green edge). The dominant MS^2^ product ions comprising motifs _437 and _444 were annotated (Figure 6C). Determining the presence of these two motifs in the molecular network identified a subnetwork (dashed) in which nodes corresponding to other melophlin congeners were detected (Figure 6B, nodes corresponding to known melophlins shown as diamonds). In this subnetwork, a third MS2LDA motif, motif_660 was identified (red edge, Figure 6B). The motif_660 is comprised of only two MS^2^ product ions that can be structurally annotated to be derived from both the C_5_-methylated and C_5_-desmethyl melophlins (Figure 6C). The motif_660 connects numerous other nodes in the network, including singletons that also possess motif_437 and motif_444, demonstrating that the structural diversity of melophlins, just as for sarasinosides, is far greater than that appreciated previously (Figure 6D). Within these potentially novel melophlin congeners are brominated derivatives with the bromine atoms predicated upon the alkyl chain, as determined by annotation of the fragmentation spectra (yellow nodes, Figure 6B). 

### 2.7. Microbiome and Metabolome Comparison of Melophlus Sponges to Neighboring Sponges

**Figure 7:**
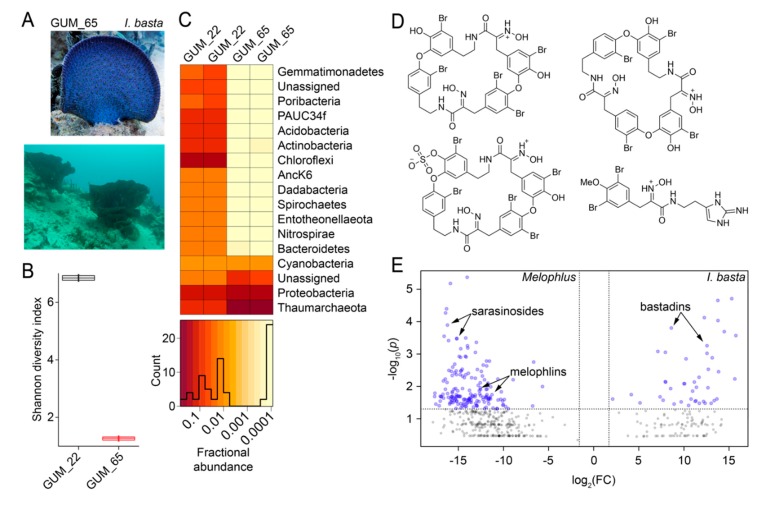
Microbiome and metabolome divergence between sponge geographical neighbors. (**A**) Morphology of *Ianthella basta* (top, specimen GUM_65) and the presence of *I. basta* sponges on the Apra harbor seafloor (bottom). (**B**) Shannon diversity indices for *M. sarasinorum* GUM_22 and *I. basta* GUM_65. (**C**) Relative abundance heatmap for microbiome amplicon sequence variants (ASVs) at the phyla level for technical replicates of GUM_22 and GUM_65. The histogram demonstrates ASV abundances. (**D**) Representative polybrominated natural products detected in the *I. basta* metabolomes dereplicated using MarinLit. (**E**) Comparative two-dimensional distribution of metabolomic features mined using MZmine2 for the four *Melophlus* specimens used in this study (on left) against four biological replicates of *I. basta* (on right). Each sponge specimen was analyzed by LC/MS in duplicate. On the x-axis, on a log_2_ scale, is plotted the mean ratio fold-change (FC) for each metabolomic feature identified above a common MS^1^ abundance threshold. The y-axis represents the statistical significance *p*-value of the ratio fold-change for each metabolite (plotted on a log_10_ scale). Metabolomic features with *p*-value > 0.05 are colored grey.

Next, we aimed to decipher whether *Melophlus* sponges share any of their microbiome or metabolomic architecture with their geographical neighbors. In Apra Harbor (Guam), together with *M. sarasinorum*, the ‘elephant ear’ sponge *Ianthella basta* is abundantly present (Figure 7A). The microbiome of the *I. basta* specimen GUM_65 was sequenced in duplicate and compared to that of *M. sarasinorum* specimen GUM_22. The *I. basta* microbiome, with a mean Shannon diversity index of 1.25 is less diverse than that of *M. sarasinorum* (mean diversity index 6.85, Figure 7B). Individual components of the microbiome are also divergent, with the *I. basta* biome being dominated by Thaumarchaeota with lesser relative abundance of Chloroflexi, Actinobacteria, and Acidobacteria phyla that dominate the *M. sarasinorum* microbiome (Figure 7C). The holobiont architectural divergence is reflected in the respective metabolomes as well. The *I. basta* metabolome, just as for *Melophlus*, have been extensively mined to reveal a rich tapestry of brominated alkaloids collectively called the bastadins (Figure 7D) [42,43]. The metabolomes of four *I. basta* biological replicates were compared to metabolomes of the four *Melophlus* specimens used in this study. A comparative plot reveals that no metabolomic features are shared between *Melophlus* and *I. basta* (Figure 7D). In this analysis, features contributing the most to metabolomic divergence are members of natural product families dedicated to respective sponge taxa, sarasinosides and melophlins for *Melophlus* and bastadins for *I. basta*. 

## 3. Discussion

While *Melophlus* sponges have been extensively mined for natural products, this is the first untargeted metabolomic characterization of their natural product profiles, their microbiome architectures, and their metagenomes. Contemporary omics tools are making it abundantly clear that the chemical diversity and biosynthetic potential for marine sponges far exceeds prior characterizations [11]. A paired multi-omic profiling of marine sponge holobionts provides several advances. Overlapping metabolomic and microbiome architectures allow for phylochemical insight into identities of novel sponges to assist phylogenetic assignments, especially in cases of non-concerted evolution of the rRNA genes as we observe for the *Melophlus* genus here [44]. Overall, grossly different sponge morphologies for species in the *Melophlus* genus do not correspond to different microbiomes and different metabolomes. We have previously recorded similar observation for the *Lamellodysidsea* genus of marine sponges [29]. How and why the microbiome structure is selected for at the genus level in marine sponges is presently not clear. 

A multi-omic profiling strategy also enables biosynthetic insights. For sarasinosides, chemical diversity is largely realized via modifications on the sterol core demonstrating the participation of promiscuous redox catalysts. Extensive redox tailoring of the sterol tetracyclic core is broadly observed in marine sponges [45]. Accurate mass differences between key MS^2^ fragment ions allows for rapidly curating what these redox modifications are, as is illustrated for representative sarasinoside congeners in Figure 3. The complexity of the sponge holobiont complicates the discovery of these modifying enzymes. It is plausible that a central sterol core that is delivered by a sponge symbiont is modified by other members of the holobiont community. Thus, in this study, we have focused on the discovery of biogenetic routes for the delivery of the sterol core structure. While sterols are ubiquitous in sponge metabolomes, the *msb* locus represents, to the best of our knowledge, the very first microbially encoded sponge-derived gene cluster that likely encodes for sterol production. Sterol biosynthetic genes can be dispersed in bacterial genomes, as is also the case for the γ-proteobacterial symbiont from which the *msb* locus is derived [25]. A squalene synthase encoding gene, an enzyme which converts farnesyl pyrophosphate to squalene (Figure 4A), is missing from the *msb* locus and is indeed located on a different metagenomic scaffold derived from this symbiont. Lesser variability is observed in the sarasinoside glycosylation pattern. The xylose-glycosyltransferase which installs the ‘A’ sugar ring is likely tolerant towards a large diversity of sterol structures. The B_1_ and B_2_ N-acetylhexose sugars remains invariant. Promiscuity for the sugar nucleosidic substrate (glucose or xylose) is apparent for the C-sugar glycosyltransferase. In addition to a diversity of marine sources, glycosylated sterols are widely present in plant metabolomes, where, due to their amphipathic structures, they are postulated to serve roles in modulating membrane permeabilization [46,47].

Another primary advance afforded by multi-omic profiling of sponges are the insights gained into the overall metabolomic architecture of sponges. Instructive to observe is the enrichment of distinct natural product classes. The motif_505, which corresponds to the presence of N-acetylhexose sugars (Figure 2D), is conserved for greater than 25% of all ions detected in the *Melophlus* LC/MS datasets. The holobiont metagenome then reveals nearly 600 N-acetylhexose glycosyltransferases to be present. While undoubtedly some of these glycosyltransferases will be involved in primary metabolism, the abundance of glycosylated metabolites and the number of candidate glycosyltransferase enzymes detected in *Melophlus* is unprecedented. Along the same lines, our prior metabolomic profiling of the Indo-Pacific sponge *Lamellodysidea herbacea* found the sponge to be exceptionally rich in the diversity of polyhalogenated phenols, leading us to posit that the sponge holobiont harbors promiscuous halogenating enzymes [48]. The rules that govern *Melophlus* sponges’ preponderance towards glycosylated natural products while *Lamellodysidea* sponges select for polyhalogenated phenols are presently not clear. These two examples are in complete contrast to our metabolomic description of the Floridian sponge *Smenospongia aurea*, the metabolome of which, while being very diverse, is not enriched in one particular natural product class [49]. 

Organizing of sponge extracts as a collection of metabolomic features allows for quantitative comparisons to address questions of choice. For instance, here, we ask whether geographical neighbors that experience identical predatory and infectious pressure use shared metabolites to combat ecological stressors? Comparison of the *Melophlus* metabolomes with that of *I. basta*, another sponge that is co-dominant on the Apra Harbor seafloor, reveals no metabolomic overlap. Crucially, this analysis includes not only abundant natural products in respective sponges that have been described spectroscopically, but also lesser abundance metabolites that are outside the purview of traditional isolation-based characterization. If sponges do not share or conserve metabolites to address comparable ecological challenges, do each of these different natural product classes represent independent defense mechanisms? It is likely that the role of sponge natural products extends beyond the simple hypothesis of predator deterrence to include recruitment and maintenance of specific commensal associations, and other, as yet unidentified roles in holobiont physiology. While ecological insight is an obvious starting point, metabolomic overlap can be used to address other questions as well such as to discern identities of metabolites in different sponge extracts leading to similar pharmacological assay readouts. Geographic proximity also does not translate to overlapping microbiomes. While Proteobacteria are abundant in both high- and low-microbial-abundance sponges, high-microbial-abundance sponges such as *M. sarasinorum* are enriched in Chloroflexi while low-microbial-abundance sponges such as *I. basta* are enriched in archaeal symbionts (Figure 7B,C), a general trend that has been observed previously [50]. 

Our study design used less than 5 g of wet tissue collected for each sponge specimen. The multi-omic workflows described here present a starting point to prioritize sponge specimens for larger collections for targeted deliverables. Geographically mapping paired multi-omic data from biodiversity hotspots will also democratize the addressing of a greater diversity of scientific questions from a single resource.

## 4. Materials and Methods 

### 4.1. DNA Extraction from Sponge Tissues

The DNA extraction protocol used in this study was adapted from our previous reports [29,30]. Sponge tissue frozen in RNA*later* was thawed overnight at 4 °C and rinsed thrice with Tris-EDTA (TE) buffer (10 mM Tris-HCl pH 7.5, 1 mM EDTA-Na). Sponge tissue was homogenized using sterile scalpel and treated with lysozyme at 30 °C in TE buffer for 2 h, followed by treatment with proteinase K at 65 °C for 30 min. Impurities were extracted twice with buffered phenol:chloroform:isoamyl alcohol and DNA precipitated by addition of equal volume of ice cold isopropanol. DNA was pelleted by centrifugation, washed twice with 70% EtOH, dissolved in TE buffer, and further column purified using Qiagen DNA purification kit using manufacturer’s directions.

### 4.2. Molecular Determination of Sponge Phylogeny

The hypervariable ITS-2 region of the ribosomal gene was amplified from metagenomic DNA using Q5 high fidelity DNA polymerase with the forward primer SP58bF (5′-AATCATCGAGTCTTTGAACG-3′) and reverse primer SP28cR (5′-CTTTTCACCTTTCCCTCA-3′), as described previously [51]. The thermocycling conditions were as follows: initial denaturation of 30 s at 98 °C, 35 cycles each of 10 s at 98 °C, 30 s at 45 °C, and 1 min at 72 °C, and a final extension of 2 min at 72 °C. The PCR products were purified and concentrated using Zymo DNA clean and concentrator kit and clone libraries were generated using ligation-independent TA cloning with the pGEM-T Easy Vector System kit. Three clones for each sponge amplicon were Sanger sequenced and reads were filtered manually to remove the nucleotides from the pGEM-T Easy vector. Filtered sequences were used to search the GenBank nr/nt database using Basic Local Alignment Search Tool (BLAST). The D3-D5 region of the 28S rRNA gene was amplified using the primers NL4F (5′-GACCCGAAAGATGGTGAACTA-3′) and NL4R (5′-ACCTTGGAGACCTGATGCG-3′) [12]. The thermocycling conditions were as follows: initial denaturation of 2 min at 95 °C, 35 cycles each of 30 s at 95 °C, 30 s at 55 °C, 70 s at 72 °C, and a final extension of 2 min at 72 °C in a 25 µL reaction volume using GoTaq DNA polymerase. Purification, assembly into vectors, and Sanger sequencing proceeded as described above.

### 4.3. 16S rRNA Gene Amplification, Sequencing, and Data Analysis

Next-generation sequencing of the v4 region of the 16S rRNA gene was used to inventory sponge-associated microbiomes. Illumina MiSeq sequencing of the dual-indexed PCR amplicons was carried out as previously described [52]. The v4 primers 515F and 806R were used with both forward and reverse primers barcoded and appended with Illumina-specific adaptors according to established procedures [53]. The PCR reactions contained 1 µL 20 ng/µL template DNA, 0.5 µL each 20 µM forward and reverse primer, 0.5 µL 10 nM dNTPs, 0.25 µL of Q5 high fidelity DNA polymerase, reaction buffer, and molecular biology grade water to adjust the volume to 25 µL. The thermocycling conditions were as follows: initial denaturation of 30 s at 98 °C, 35 cycles each of 30 s at 98 °C, 30 s at 50 °C, 20 s at 72 °C, and a final extension of 2 min at 72 °C. Amplicons were purified and concentrated using Zymo DNA clean and concentrator kit, pooled in equimolar concentrations, and sequenced on an Illumina MiSeq platform using a 500 cycle kit with 10% PhiX DNA to increase read diversity. The raw sequences were imported into QIIME2 (v.2017.7; https://docs.qiime2.org/2019.7/) using the qiime tools import script with the input format as PairedEndFastqManifestPhred33, and the sequences were demultiplexed using the qiime demux script [54]. DADA2 software was used to obtain a set of observed sequence variants (SVs) [55]. Based on quality scores, the forward and reverse reads were truncated at 150 bp using the qiime dada2 denoise script. Taxonomy was assigned using the SILVA pre-trained classifier (silva-119-99-515-806-nb-classifier) using the qiime feature classifier plug-in [56]. Taxonomic distributions of the samples were calculated using the qiime taxa barplot script.

The α-diversity metrics associated with the *M. sarasinorum* (GUM_22) and *I. basta* (GUM_65) were computed using the qiime diversity core metrics script. The Shannon diversity index was used to the compare the microbial diversity as it takes both abundance and richness into consideration. The relative abundances of the different taxonomic phyla associated were represented through a heatmap. The reads corresponding to each individual phylum in a sample were normalized by dividing the value by the sum of the reads of all phyla in the given sample. The negative logarithm of the normalized values was computed to reduce the spread of the data, and the values were imported to R studio in a .csv file format. The corresponding heatmap was generated in RStudio using the following script:

#x<-read.csv(“Final XIC area.csv”, row.names = 1, check.names = FALSE)

#y<- data.matrix(x)

#pal<-choose_palette()

#heatmap.2(y, trace = ‘none’, margins = c(10,12), Rowv = FALSE, Colv = FALSE, col = pal, cexRow = 1.0,lwid = c(2,10),lhei = c(2,10), density.info = ‘none’, key.par = list(mar = c(4,4,4,10)))\

### 4.4. Sponge Chemical Extraction and Mass Spectrometry Data Acquisition

Sponge tissues were frozen and lyophilized to dryness. Dried tissues were soaked in 1:1 v/v DCM/MeOH (1 mL solvent/100 mg dry sponge tissue) for 48 h at room temperature. The DCM/MeOH extract was clarified by centrifugation, dried, resuspended in MeOH, and directly analyzed using an Agilent 1290 Infinity II UHPLC coupled to a Bruker ImpactII ultra-high-resolution Qq-TOF mass spectrometer equipped with an electron spray ionization source. A Kinetex 1.7 μm C_18_ reversed-phase UHPLC column (50 × 2.1 mm) was employed for chromatographic separation. MS spectra were acquired in positive ionization mode from m/z 50–2000 Da. An active exclusion of ‘2’ spectra was employed, implying, that an MS^1^ ion would not be selected for fragmentation after two consecutive MS^2^ spectra had been recorded for it in a 0.5 min time window. For acquiring MS^2^ data, eight most intense ions per MS^1^ spectra were selected. Chromatography solvent A: water + 0.1% v/v formic acid, solvent B: MeCN + 0.1% v/v formic acid. Flow rate was held constant at 0.5 mL/min throughout. The elution profile employed was: 5% solvent B for 3 min, a linear gradient from 5% to 50% B in 5 min, 50% B for 2 min, from 50% to 100% B in 5 min, 100% B for 3 min, from 100% to 5% B in 1 min, 5% B for 1 min, from 5% to 100% B in 1 min, 100% B for 3 min, from 100% to 5% B in 1 min, 5% B for 5 min.

### 4.5. Molecular Networking

Molecular networks were generated using the GNPS online workflow [21]. The MS^2^ raw data were clustered with MS-Cluster with a parent mass tolerance of 0.01 Da and a MS^2^ fragment ion tolerance of 0.05 Da to create consensus spectra. The edges were filtered to have a cosine score above 0.7 and more than 4 MS^2^ matched peaks were used. Edges between nodes were preserved if both nodes were within each other’s top 10 most similar nodes. The molecular network thus created was visualized using Cytoscape 3.6.1. The molecular networking job processed by the classical molecular networking workflow in GNPS was reanalyzed by MS2LDA using default parameters [23]. For feature-based molecular networking, raw data files were processed using MZmine2 for feature detection [22]. The MS^1^ data was filtered by assigning a threshold level for noise detection at 10,000, MS^2^ spectra were filtered using a noise level threshold of 500. The following are the parameters applied to the filtered data: chromatogram builder (minimum time span: 0.1 min; minimum intensity of the highest data point in the chromatogram: 1500; *m/z* tolerance: 15 ppm); chromatogram deconvolution (local minimum search, *m/z* range for MS^2^ scan pairing: 0.025 Da; retention time range for MS^2^ scan pairing: 0.2 min); isotopic peaks grouper (*m/z* tolerance: 15 ppm; retention time tolerance absolute: 0.1 min; maximum charge: 3; representative isotope: most intense); join aligner (*m/z* tolerance: 15 ppm; retention time tolerance: 0.1 min); feature list rows filter (*m/z* range: 1280 to 1350 Da; MS/MS filter; reset peak number ID); remove duplicate filter (retention time tolerance absolute: 0.1; *m/z* tolerance: 5 ppm); peak finder (intensity tolerance: 0.1; retention time tolerance absolute: 0.1; *m/z* tolerance: 15 ppm). The two output files of MZmine2 were a feature table with ion intensities (.csv file format) and a list of MS^2^ spectra (mgf file). These files were exported with MZmine2 to the GNPS feature based networking workflow [24]. The parameters were set with the precursor ion mass tolerance of 0.01 Da and the fragment ion mass tolerance of 0.05 Da. The cosine score was set at 0.7 and a minimum of 6 matched fragment ions were needed to generate the network. The network was visualized as before in Cytoscape 3.6.1 and used to generate the Venn diagram illustrating the metabolomic overlap between M. *sarasinorum* (samples GUM_22 and GUM_59) and *Melophlus sp*. (samples GUM_133 and GUM_139). Plot for comparison of *Melophlus* and *I. basta* metabolomes were generated by MetaboAnalyst using features extracted by MZmine2 [57]. The volcano plot was generated at a fold-change threshold of 3 and a p-value threshold of 0.05, after inter quartile data filtering and pareto scaling of the data.

### 4.6. Isolation and Structural Characterization of ***1*** and ***3***

Sponge extracts, generated as described above, were chromatographed on a Luna 5 μm C_18_ reversed-phase LC column (250×10 mm) using chromatography solvent A: water + 0.05% v/v trifluoracetic acid (TFA), solvent B: MeCN + 0.05% v/v TFA. Flow rate was held constant at 2 mL/min throughout. The elution profile employed for isolation of **1** was: 5% solvent B for 5 min, a linear gradient from 5% to 40% B in 5 min, from 40% to 60% B in 20 min, from 60% to 100% B in 5 min, 100% B for 3 min, from 100% to 5% B in 1 min, 5% B for 1 min, from 5% to 100% B in 1 min, 100% B for 3 min, from 100% to 5% B in 1 min, 5% B for 5 min. The elution profile employed for isolation of **3** was: 5% solvent B for 5 min, a linear gradient from 5% to 50% B in 5 min, 50% B for 10 min, from 50% to 90% B in 2 min, from 90% to 100% B in 22 min, 100% B for 5 min, from 100% to 5% B in 1 min, 5% B for 1 min, from 5% to 100% B in 1 min, 100% B for 1 min, from 100% to 5% B in 1 min, 5% B for 5 min. An additional isocratic purification step was employed for **3**, 93% solvent B for 45 min. Solvents were removed in vacuo to afford dried molecules. Molecules were dissolved **1** in CD_3_OD and **3** in CDCl_3_ followed by NMR data acquisition using an 800 MHz Bruker Avance III HD spectrometer.

### 4.7. Quantification of Relative Abundances of Sarasinosides and Melophlins

The abundance of sarasinoside and melophlin congeners was calculated by the area under the extracted ion chromatogram (EIC) generated as a part of the feature table processing in MZmine2. Identical parameters as described above for feature table generation were used, except that the *m/z* range was changed to 250–1335 Da in the feature list rows filter module. Areas under the EIC for known sarasinoside and melophlin congeners were tabulated and normalized separately for sarasinosides and melophlins. These values were then converted into a negative logarithmic scale. Normalized logarithmic areas for absent congeners were assigned a value of 6 (since the negative logarithm of 0 is undefined). The value 6 was chosed based on the least abundant sarasinoside which had a negative logarithmic value of 4.41. Therefore, 6 is a fair estimation, when based on the logarithmic scale, to denote the abundance of sarasinosides that are not detected in the sponge metabolome. The script used to generate the heatmap in RStudio is described above.

### 4.8. Metagenomic Sequencing, Assembly, and Mining

Illumina HiSeq 2500 reads (2 × 150 paired end) were processed using Trimmomatic version 0.339 [58], then assembled using IDBA-UD version 1.1.117 [59]. Scaffolds were binned based on percent GC, nucleotide composition, coverage depth, and taxonomic classification by DarkHorse version 2.0 [60], as previously described [61]. Reads were mapped to binned scaffold groups using Bowtie2, version 2.218 [62], and coverage depth was determined with the idxstats module of samtools version 0.1.191 [63]. Second-round, targeted assemblies were performed to obtain MAGs from individual binned read subsets using Celera Assembler version 8.3 [64]. MAG completeness was assessed using CheckM version 1.07 [34].

## 5. Database Deposition Information

The mass spectrometry data collected were deposited in the MassIVE repository with the MassIVE ID# MSV000084824. Sponge 28S rRNA and ITS-2 rRNA amplicon data, 16S amplicon data, and metagenomic sequencing data were deposited to GenBank under BioProject accession number PRJNA602901. Sequences for the *msb* and *mgb* gene loci have been deposited to Genbank under accession numbers MT026193 and MT026194, respectively.

## Supplementary Materials

The following are available online at https://www.mdpi.com/1660-3397/18/2/124/s1, Figure S1: Molecular determination of sponge phylogeny, Figure S2: ^1^H-NMR spectra for **1** in CD_3_OD, Figure S3: ^13^C-NMR spectra for **1** in CD_3_OD, Figure S4: Structural annotation of the MS^2^ spectra for **1**, Figure S5: ^1^H-NMR for **3** in CDCl_3_, Figure S6: ^13^C-NMR for **3** in CDCl_3_, Figure S7: Structural annotation of the MS^2^ spectra for **3**, Table S1: Percentage identity matrix for *Melophlus* sponges as generated by EMBL-EBI Clustal Omega, Table S2: Sarasinoside congeners detected in this study and deposited to GNPS, Table S3: Melophlins congeners detected in this study and deposited to GNPS, Table S4: Table representing representative features that are shared among sponge specimens used in this study as denoted in Figure 1E in the main text, Table S5: ^13^C and ^1^H NMR data for **1** in CD_3_OD, Table S6: Table of retention times and precursor intensities for parent ion *m/z* 1321.65 condensed into a single node in molecular networking, Table S7: ^13^C and ^1^H NMR data for **3** in CDCl_3_.
