# Supplementary material for: Multi-Omic Profiling of Melophlus Sponges Reveals Diverse Metabolomic and Microbiome Architectures that Are Non-overlapping with Ecological Neighbors
Source: Mar Drugs. 2020 Feb 19;18(2):124. doi: 10.3390/md18020124 (PMC7074536; doi:10.3390/md18020124)
Supplement: Supplementary file 1 [file marinedrugs-18-00124-s001.pdf]

SUPPLEMENTARY INFORMATION FOR:

Multi-omic profiling of *Melophlus* sponges reveals diverse metabolomic and microbiome architectures that are non-overlapping with ecological neighbors

Ipsita Mohanty, Sheila Podell, Jason S. Biggs, Neha Garg, Eric E. Allen, Vinayak Agarwal

SUPPLEMENTARY FIGURES

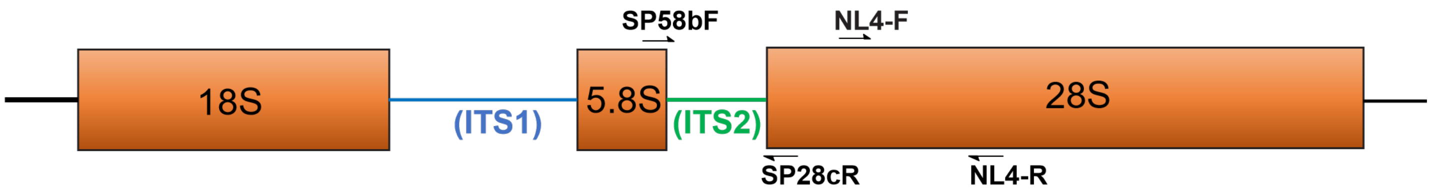

Fig. S1: **Molecular determination of sponge phylogeny**. The hypervariable internal transcribed spacer region (ITS-2) and the D3-D5 region of the large subunit (28S) of the ribosomal gene were used as the barcode for the sponge phylogeny determination. The forward primer SP58bF (5'-AATCATCGAGTCTTTGAACG-3') and reverse primer SP28cR (5'-CTTTTCACCTTTCCCTCA-3') pair was used to amplify the ITS-2 region. The NL4_F (5'-GACCCGAAAGATGGTGAACTA-3') and NL4_R (5'-ACCTTGGAGACCTGATGCG-3') primer pair was selected for amplifying the D3-D5 region on the 28S ribosomal RNA gene.

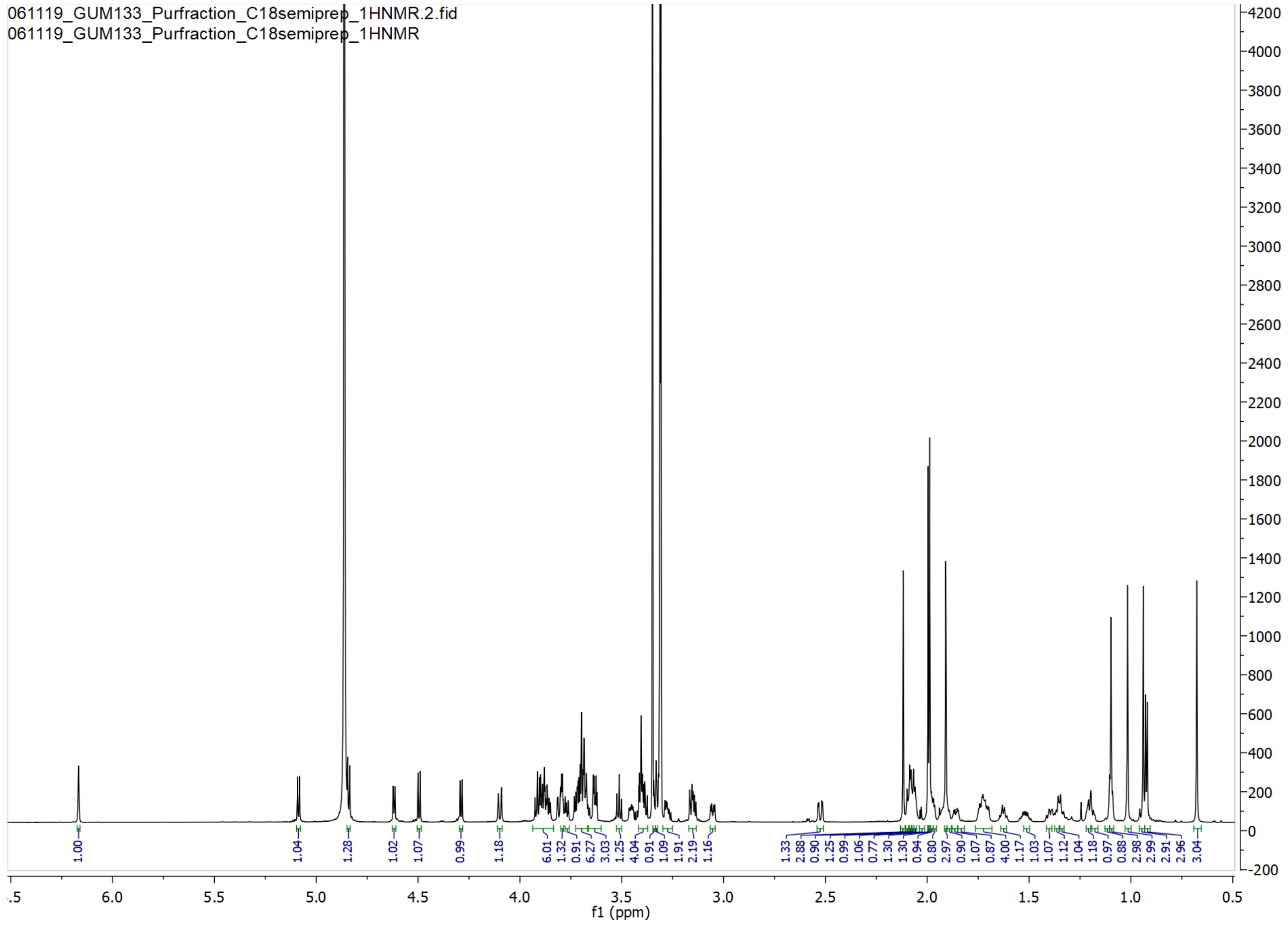

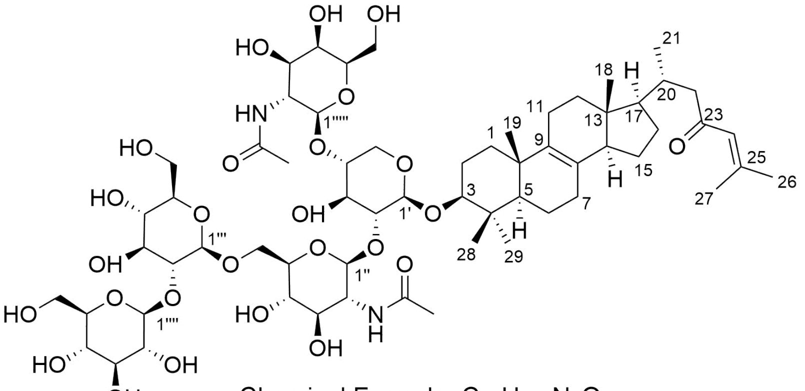

Chemical Formula: $C_{62}H_{100}N_2O_{26}$

Fig S2: ^{1}H-NMR spectra for **1** in CD_3OD.

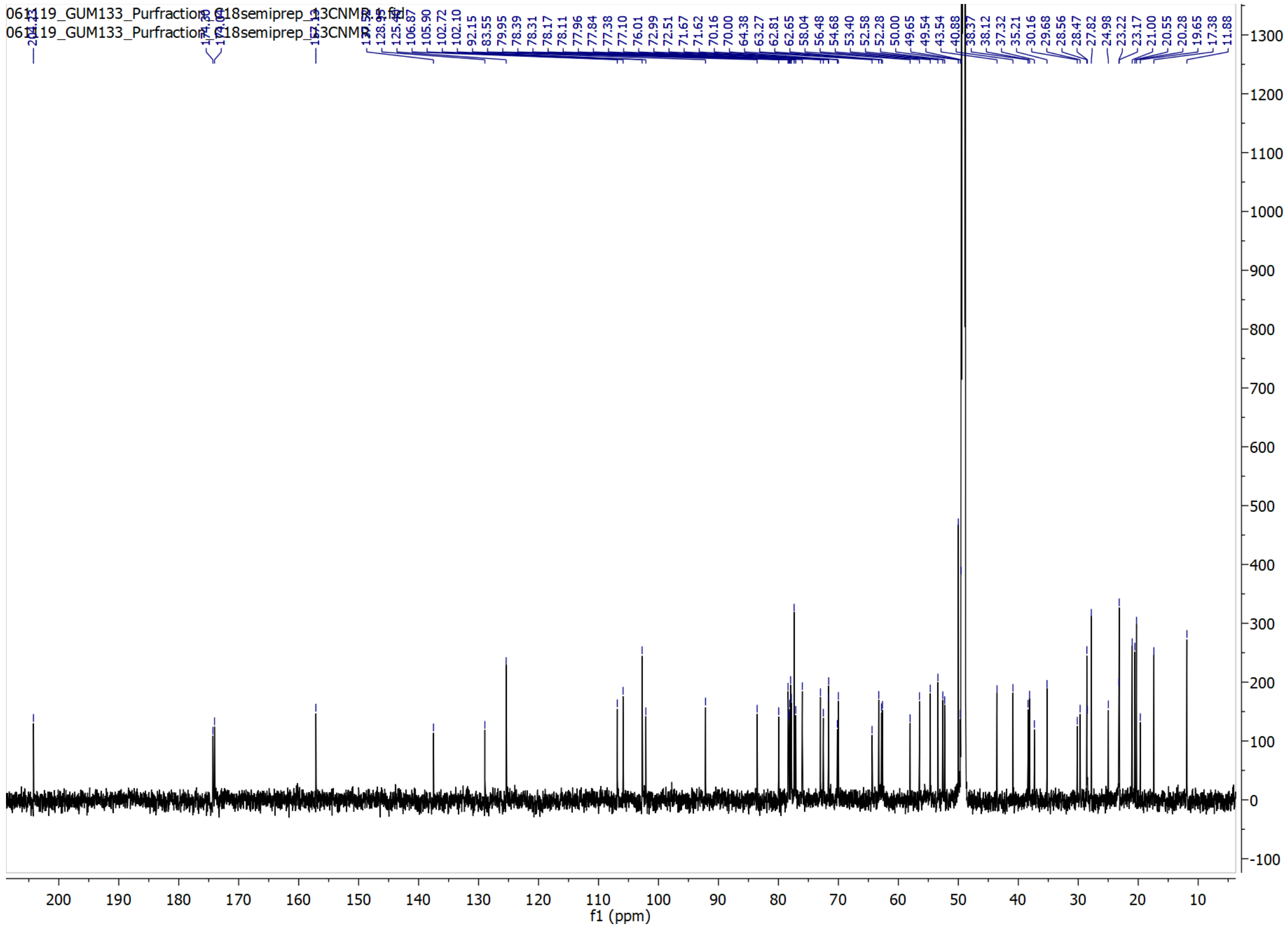

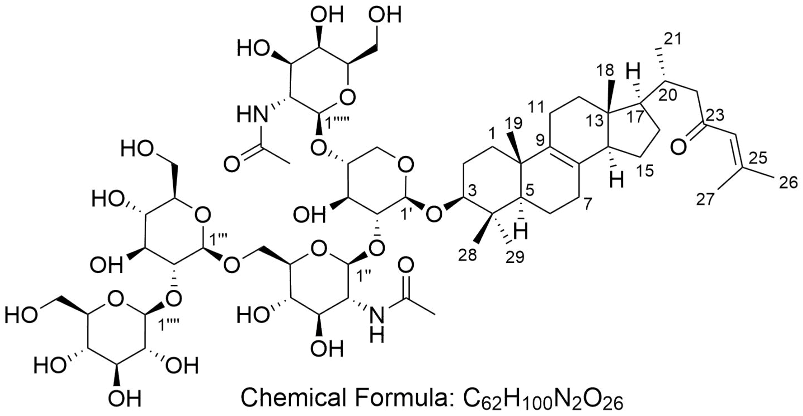

Fig S3: ^{13}C-NMR spectra for **1** in CD_3OD.

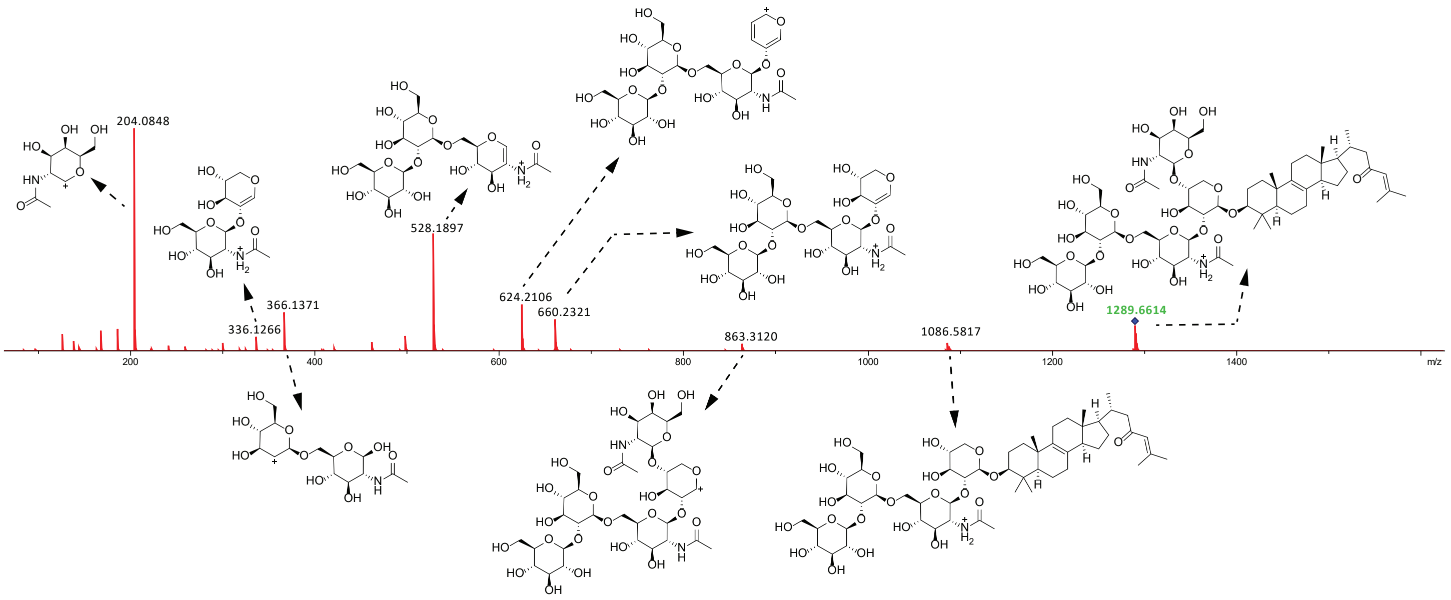

Fig. S4: Structural annotation of the MS^2 spectra for **1**.

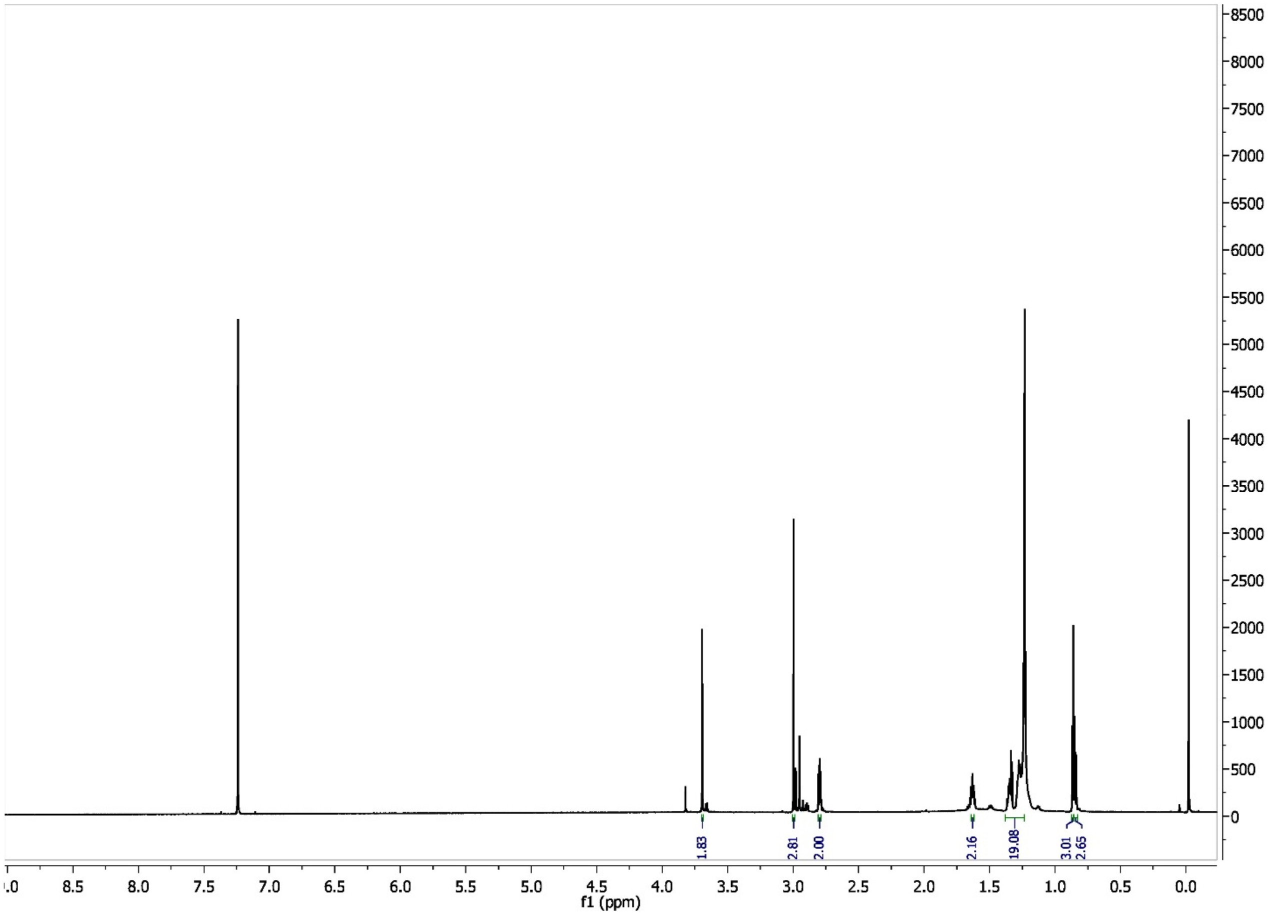

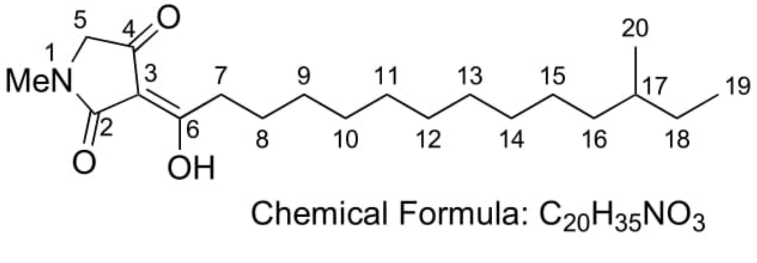

Fig. S5: ^{1}H-NMR for **3** in $CDCl_3$.

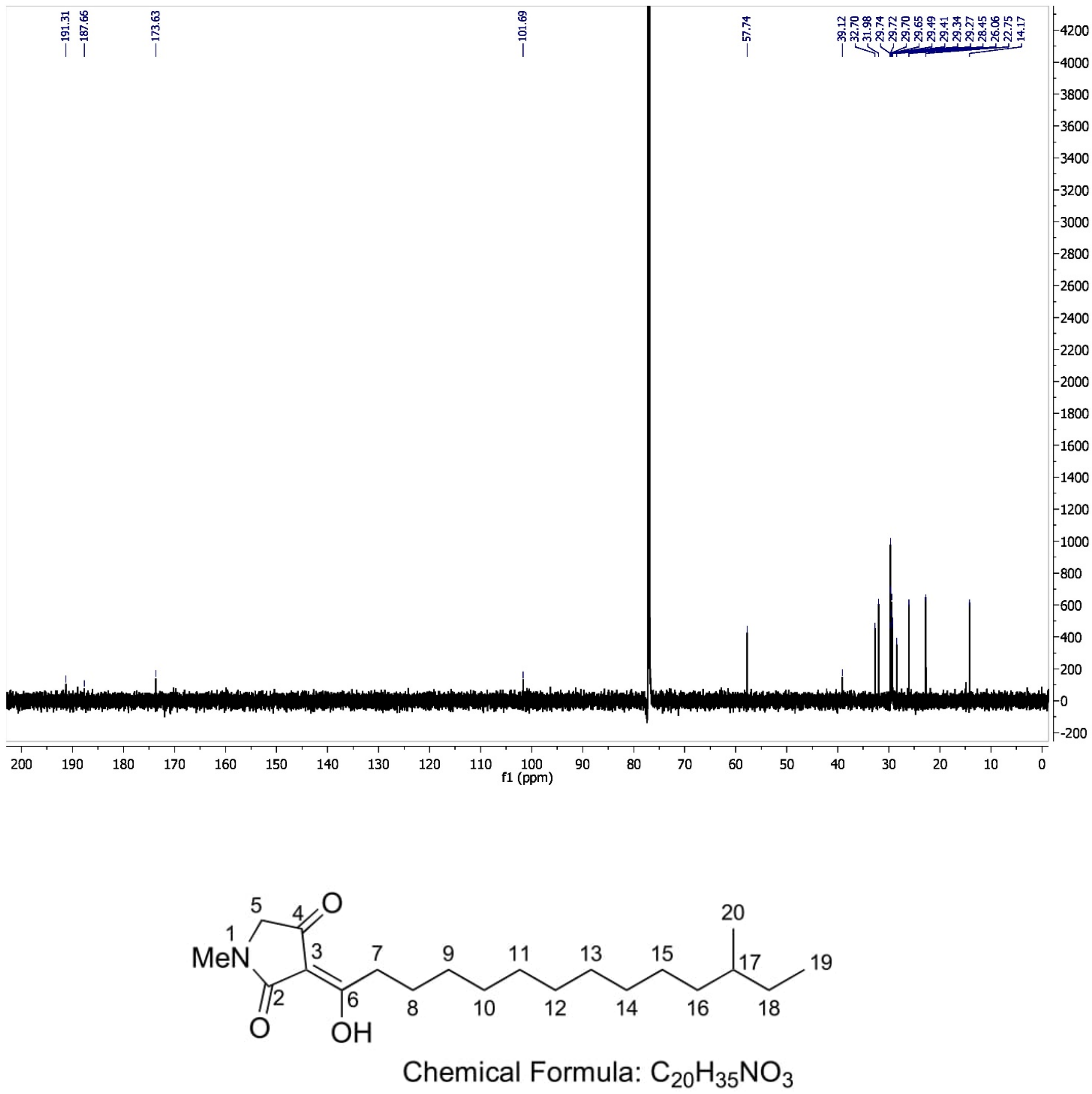

Fig. S6: ^{13}C-NMR for **3** in $CDCl_3$.

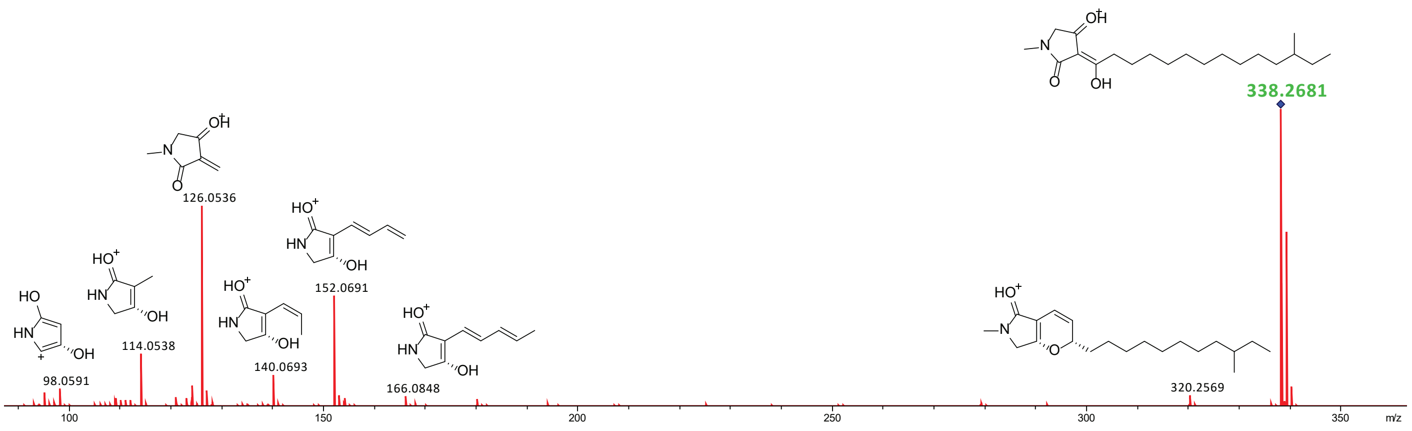

Fig. S7: Structural annotation of the MS^2 spectra for **3**.

SUPPLEMENTARY TABLES

Table S1: Percentage identity matrix for Melophlus sponges as generated by EMBL-EBI Clustal Omega [1]

	Sponge_ID	GUM_22	GUM_59	GUM_133	GUM_139
ITS-2	GUM_22	100.00	100.00	100.00	100.00
	GUM_59	100.00	100.00	100.00	100.00
	GUM_133	100.00	100.00	100.00	100.00
	GUM_139	100.00	100.00	100.00	100.00
	Sponge_ID	GUM_22	GUM_59	GUM_133	GUM_139
28S rRNA	GUM_22	100.00	100.00	43.85	44.28
	GUM_59	100.00	100.00	43.85	44.28
	GUM_133	43.85	43.85	100.00	99.70
	GUM_139	44.28	44.28	99.70	100.00

Table S2: Sarasinoside congeners detected in this study and deposited to GNPS [ref: 2-9]

**Natural product**	**Chemical structure**	**Exact mass**	**Reference**	**GNPS SpectrumID**
Sarasinoside A1	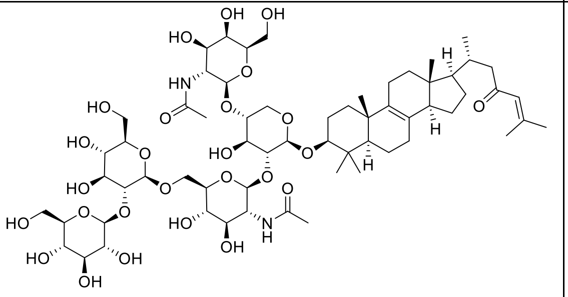	1288.6564	2	CMSLIB00005436303
Sarasinoside A2	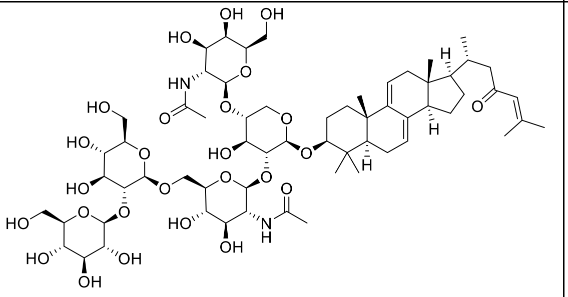	1286.6408	3	CCMSLIB00005436473
Sarasinoside A3	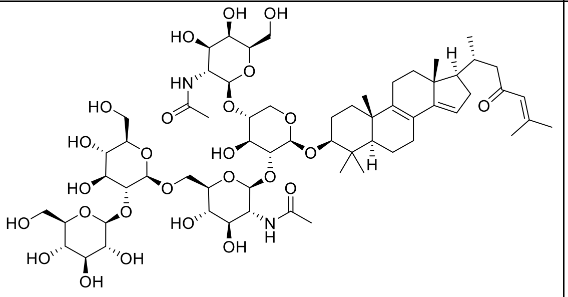	1286.6408	3	CCMSLIB00005436473
Sarasinoside A4	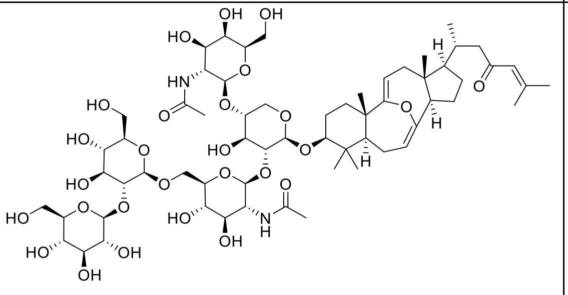	1302.6357	4	CCMSLIB00005436474

Sarasinoside A5	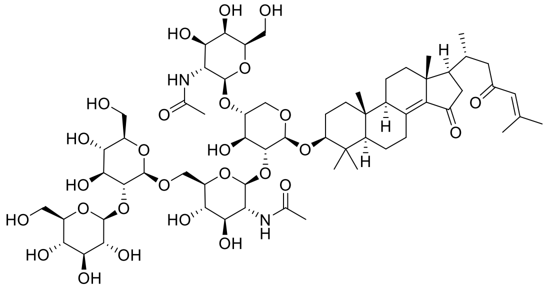	1302.6357	4	CCMSLIB00005436474
Sarasinoside B1	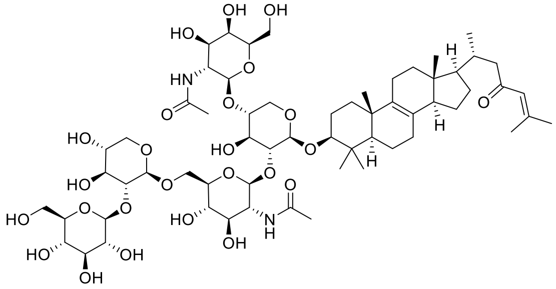	1258.6459	3	CCMSLIB00005436458
Sarasinoside B2	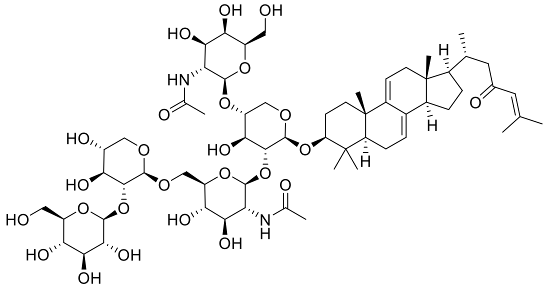	1256.6302	3	CCMSLIB00005436475
Sarasinoside B3	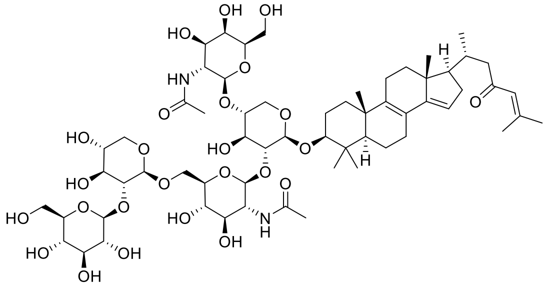	1256.6302	3	CCMSLIB00005436475
Sarasioside C1	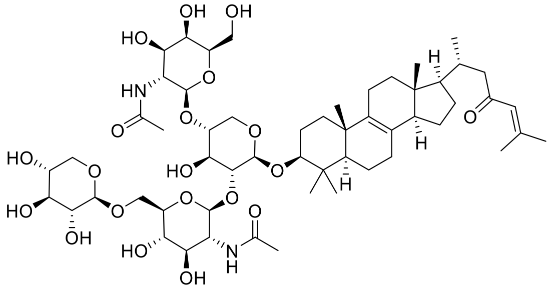	1096.5930	3	CCMSLIB00005436467

Sarasinoside C2	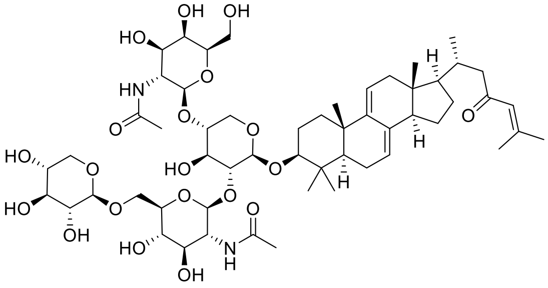	1094.5774	3	Not detected in datasets used in this study
Sarasinoside C3	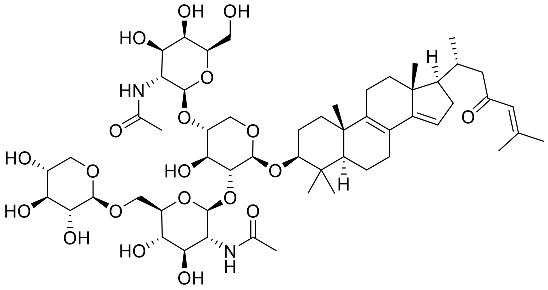	1094.5774	3	Not detected in datasets used in this study
Sarasinoside D	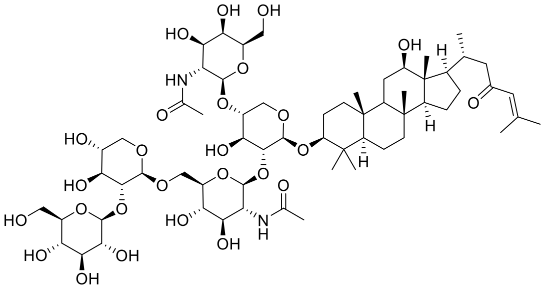	1290.6721	5	Not detected in datasets used in this study
Sarasinoside E	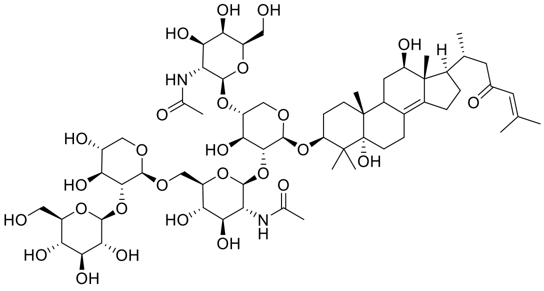	1290.6357	5	CCMSLIB00005436468
Sarasinoside F	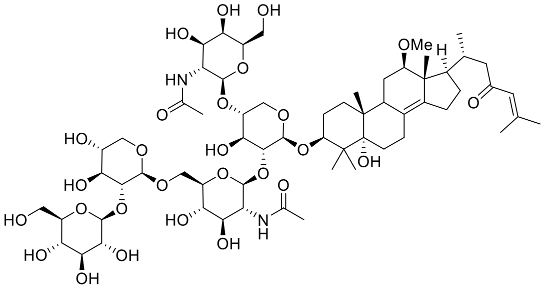	1304.6513	5	Not detected in datasets used in this study

Sarasinoside G	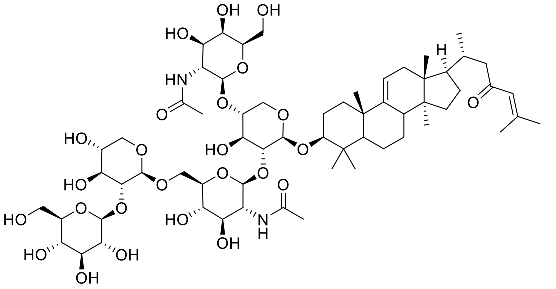	1272.6615	5	CCMSLIB00005436476
Sarasinoside H1	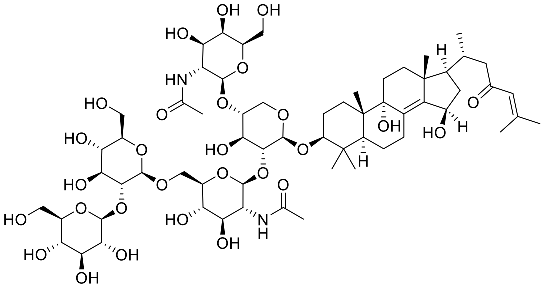	1320.6463	6	CCMSLIB00005436481
Sarasinoside H2	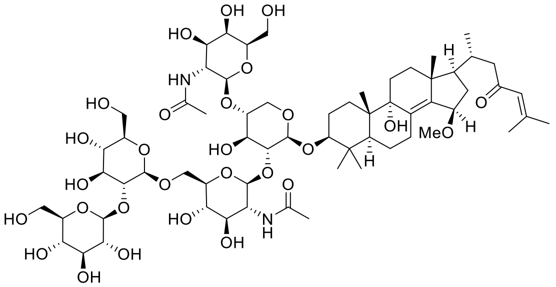	1334.6619	6	CCMSLIB00005436480
Sarasinoside I1	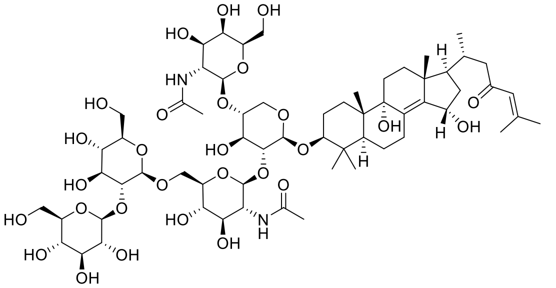	1320.6463	6	CCMSLIB00005436481
Sarasinoside I2	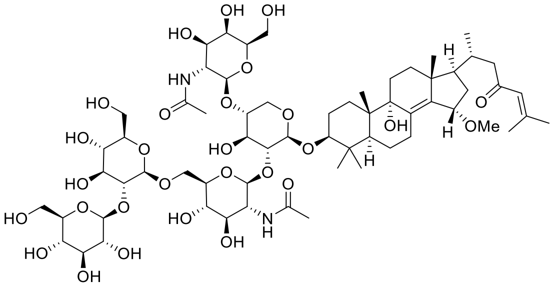	1334.6619	6	CCMSLIB00005436480

Sarasinoside J	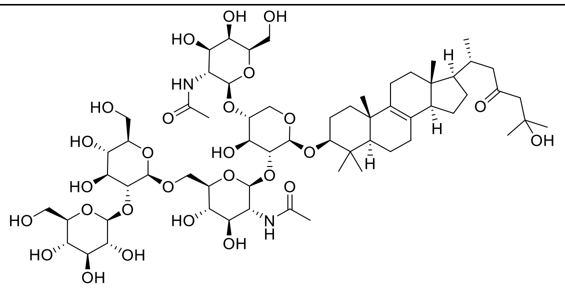	1306.6670	7	Not detected in datasets used in this study
Sarasinoside K	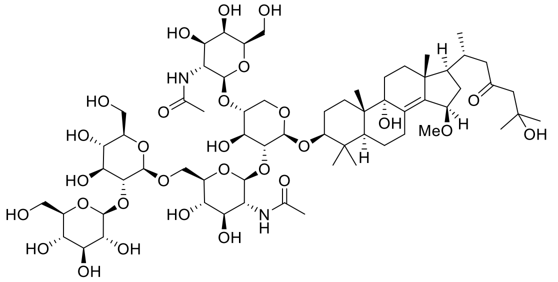	1352.6725	7	Not detected in datasets used in this study
Sarasinoside L	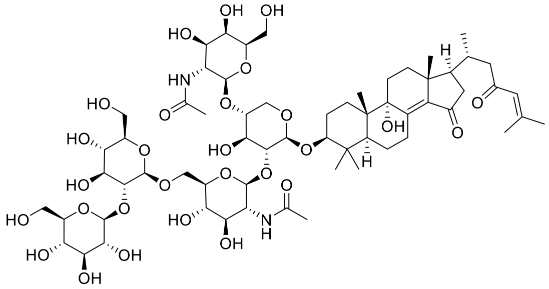	1318.6306	7	CCMSLIB00005436477
Sarasinoside M	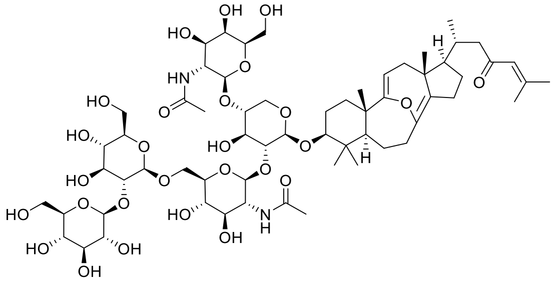	1302.6357	7	CCMSLIB00005436474
Sarasinoside M2	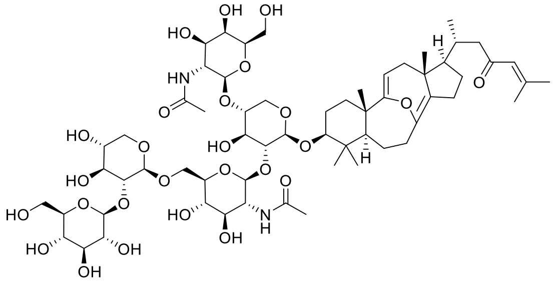	1272.6251	9	CCMSLIB00005436478

Sarasinoside N	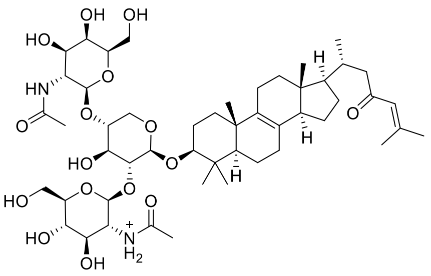	964.5508	8	CCMSLIB00005436461
Sarasinoside O	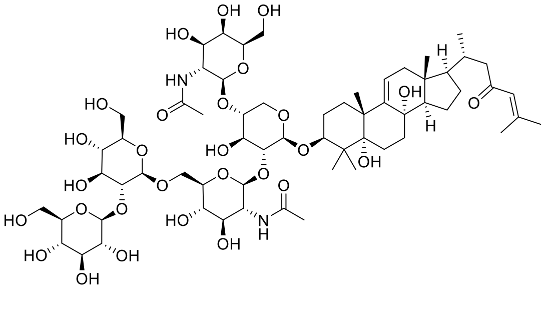	1320.6463	8	CCMSLIB00005436481
Sarasinoside P	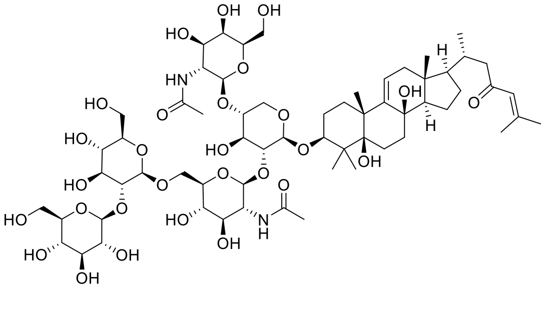	1320.6463	8	CCMSLIB00005436481
Sarasinoside Q	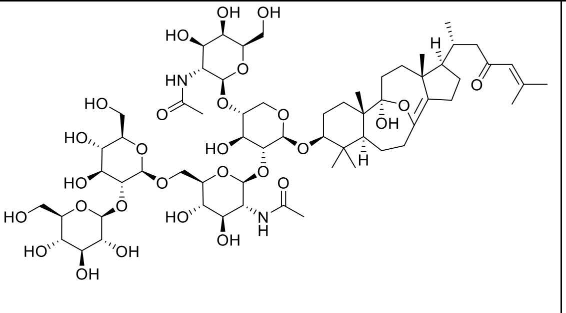	1320.6463	8	CCMSLIB00005436481
Sarasinoside R	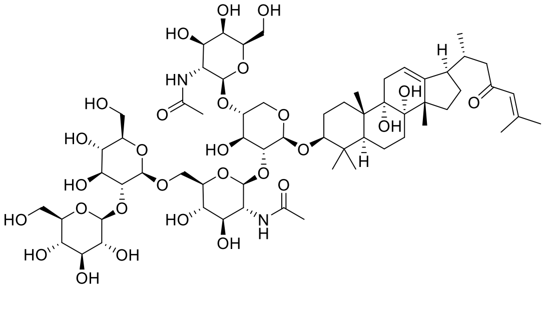	1320.6463	8	CCMSLIB00005436481

Table S3: Melophlins congeners detected in this study and deposited to GNPS [ref: 10-12]

**Natural product**	**Chemical structure**	**Exact mass**	**Reference**	**GNPS SpectrumID**
Melophlin A	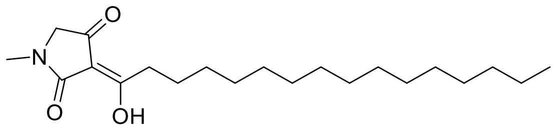	351.2773	10	CCMSLIB00005436504
Melophlin B	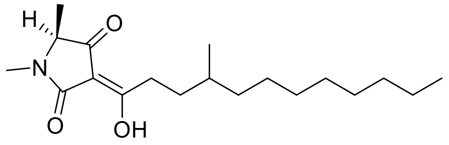	323.246	10	CCMSLIB00005436512
Melophlin C	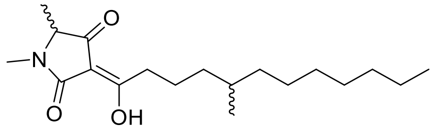	323.246	11	CCMSLIB00005436512
Melophlin D	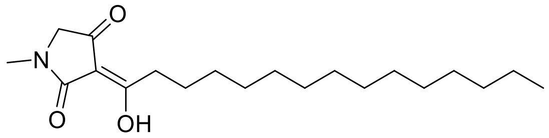	337.2617	11	CCMSLIB00005436513
Melophlin E	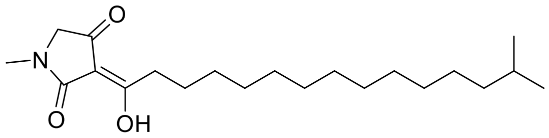	351.2773	11	CCMSLIB00005436504
Melophlin F	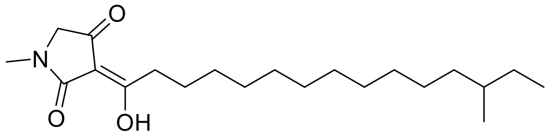	351.2773	11	CCMSLIB00005436504
Melophlin G	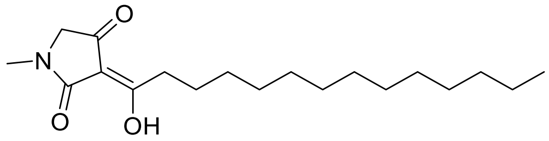	323.246	11	CCMSLIB00005436512
Melophlin H	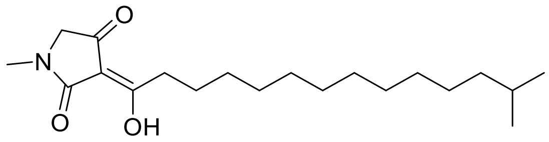	337.2617	11	CCMSLIB00005436513

Melophlin I	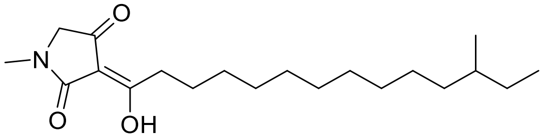	337.2617	11	CCMSLIB00005436513
Melophlin J	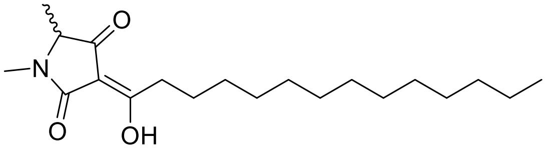	337.2617	11	CCMSLIB00005436513
Melophlin K	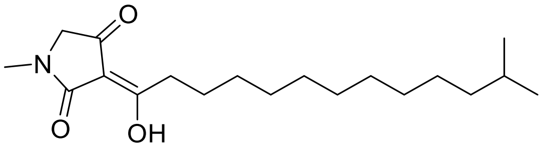	323.246	11	CCMSLIB00005436512
Melophlin L	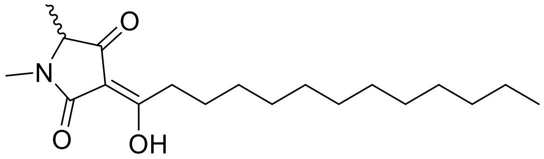	323.246	11	CCMSLIB00005436512
Melophlin M	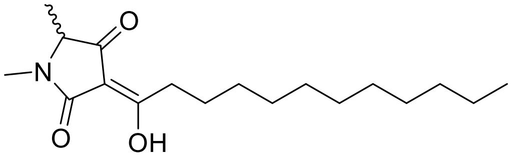	309.2304	11	CCMSLIB00005436514
Melophlin N	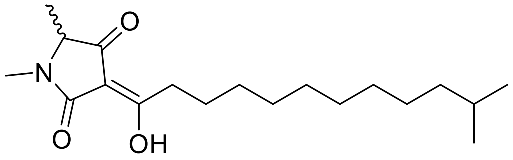	323.246	11	CCMSLIB00005436512
Melophlin O	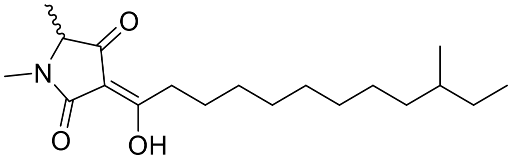	323.246	11	CCMSLIB00005436512
Melophlin P	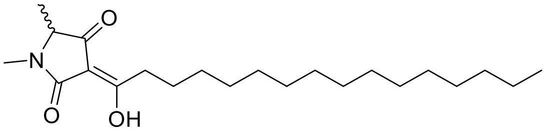	365.293	12	CCMSLIB00005436515
Melophlin Q	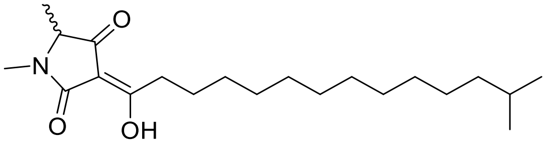	351.2773	12	CCMSLIB00005436504

Melophlin R	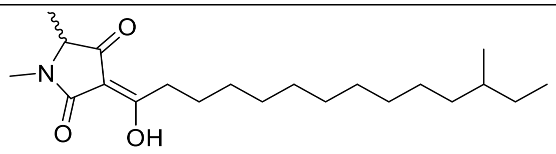	351.2773	12	CCMSLIB00005436504
Melophlin S	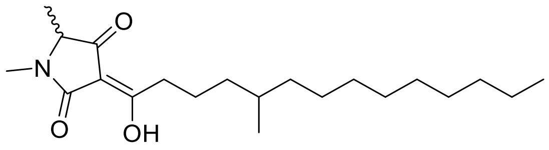	351.2773	12	CCMSLIB00005436504

Table S4: Table representing representative features that are shared among sponge specimens used in this study as denoted in Fig. 1E in the main text

Sponge code	Total number of features	Sarasinosides and Melophlins Features (*m/z*)
GUM_22, GUM_59, GUM_133, GUM_139	930	1321.651, 1319.6469, 1303.6422, 1291.6422, 1289.6624, 1287.6479, 1259.6532, 1257.638, 965.5576, 366.2992, 352.2841, 338.2687, 324.2533, 310.2369
GUM_22, GUM_59	70	1291.6415, 1273.6682, 1273.6321
GUM_133, GUM_139	76	324.2522

Table S5: ^{13}C and ^{1}H NMR data for **1** in CD_3OD

**Sterol**		
**Position**	**δ_C**	**δ_H (mult.)**
1	37.32	1.21 (m)
		1.7 (m)
2	28.47	1.73 (m)
		1.82 (m)
3	92.16	3.05
4	40.88	
5	49.65	1.12 (m)
6	19.65	1.51 (m)
		1.72 (m)
7	29.86	1.74 (m)
		1.98 (m)
8	128.95	
9	137.52	
10	38.12	
11	21.0	1.97 (m)
		2.07 (m)
12	38.37	1.40 (m)
		1.86 (m)
13	43.54	
14	56.48	2.06 (m)
15	24.98	1.36 (m)
		1.63 (m)
16	30.16	1.34 (m)
		1.89 (m)
17	53.4	1.17 (m)
18	11.88	0.66 (s)
19	20.55	1.01 (s)
20	35.21	2.10 (m)
21	20.28	0.91 (d)
22	52.28	2.52
		2.09 (m)
23	204.23	
24	125.4	6.17 (s)
25	157.13	
26	50.0	2.12 (s)
27	28.56	1.91 (s)
28	17.38	0.92 (d)
29	27.82	1.10 (s)
**Xylose**		
**Position**	**δ_C**	**δ_H (mult.)**
1’	106.87	4.29 (d)

2’	78.31	4.10 (d)
3’	52.58	3.51 (m)
4’	79.95	3.68 (m)
5’	64.38	3.14 (m)
		3.88 (m)
**N-acetylglucosamine**		
1’’	102.1	4.82 (m)
2’’	58.04	3.68 (m)
3’’	77.1	3.41 (m)
4’’	72.51	3.16 (m)
5’’	78.11	3.63 (m)
6’’	70	3.91 (m)
		3.77 (m)
Ac	23.17	2.00 (s)
	174.3	
**Glucose-I**		
1’’’	102.72	5.08 (d)
2’’’	83.55	3.34 (m)
3’’’	77.96	3.68 (m)
4’’’	71.67	3.28 (m)
5’’’	77.38	3.41 (m)
6’’’	62.65	3.70 (m)
		3.83 (m)
**Glucose-II**		
1’’’’	105.9	4.62 (d)
2’’’’	76.01	3.40 (m)
3’’’’	77.84	3.40 (m)
4’’’’	71.62	3.29 (m)
5’’’’	78.17	3.27 (m)
6’’’’	62.81	3.71 (m)
		3.91 (m)
**N-acetylgalactosamine**		
1’’’’’	102.72	4.49 (d)
2’’’’’	54.68	3.85 (m)
3’’’’’	72.99	3.63 (m)
4’’’’’	70.16	3.78 (m)
5’’’’’	78.39	3.63 (m)
6’’’’’	63.27	3.72 (m)
		3.88 (m)
Ac	23.33	1.99 (s)
	174.04	

Table S6: Table of retention times and precursor intensities for parent ion *m/z* 1321.65 condensed into a single node in molecular networking

Scan number	Dataset	Precursor intensity	Retention time (min)	Parent Mass
1779	GUM22_2	9417	6.53	1321.640
1787	GUM22_2	11148	6.55	1321.640
1802	GUM139_2	21563	6.58	1321.650
1799	GUM22_1	53127	6.59	1321.650
1800	GUM139_1	35206	6.60	1321.650
1801	GUM133_1	44109	6.60	1321.650
1808	GUM133_2	39361	6.61	1321.650
1808	GUM133_1	32753	6.62	1321.650
1811	GUM22_1	20689	6.63	1321.650
1819	GUM139_2	24469	6.64	1321.650
1818	GUM133_2	21968	6.64	1321.650
1828	GUM139_2	23563	6.67	1321.650
1836	GUM139_1	29602	6.71	1321.650
1843	GUM133_1	19952	6.73	1321.650
1865	GUM22_1	44040	6.80	1321.650
1885	GUM139_1	33586	6.87	1321.650
1889	GUM139_1	37377	6.88	1321.650
1925	GUM133_2	58829	6.98	1321.650
1934	GUM133_2	55569	7.01	1321.650
1936	GUM139_2	67727	7.01	1321.650
1937	GUM22_2	22207	7.03	1321.650
1946	GUM139_2	44898	7.04	1321.650
1945	GUM22_2	30922	7.06	1321.650
1961	GUM133_1	193956	7.11	1321.650
1962	GUM139_1	104484	7.11	1321.650
1969	GUM133_1	56838	7.13	1321.650
2012	GUM22_2	11673	7.27	1321.650
2012	GUM22_1	17322	7.27	1321.650

Table S7: ^{13}C and ^{1}H NMR data for **3** in $CDCl_3$

**Position**	**δ_C**	**δ_H (mult.)**
1		
2	173.63	
3	101.69	
4	191.31	
5	57.74	3.69 (s)
6	187.66	
7	31.98	2.80 (t)
8	26.06	1.63 (m)
9-15	29.27-29.71	1.12 – 1.39 (m)
16	39.12	1.12 – 1.39 (m)
17	32.7	1.12 – 1.39 (m)
18	29.74	1.12 – 1.39 (m)
19	14.17	0.86 (t)
20	22.75	0.84 (d)
N-CH_3	28.45	3.00 (s)

SUPPLEMENTARY REFERENCES

1. Madeira, F.; Park, Y. M.; Lee, J.; Buso, N.; Gur, T.; Madhusoodanan, N.; Basutkar, P.; Tivey, A. R. N.; Potter, S. C.; Finn, R. D.; Lopez, R., The EMBL-EBI search and sequence analysis tools APIs in 2019. *Nucleic Acids Res.* **2019,** 47, (W1), 636-641.

2. Kitagawa, I.; Kobayashi, M.; Okamoto, Y.; Yoshikawa, M.; Hamamoto, Y., Structures of sarasinosides A1, B1, and C1; new norlanostane-triterpenoid oligoglycosides from the Palauan marine sponge Asteropus sarasinosum. *Chem. Pharm. Bull.* **1987,** 35, (12), 5036-5039.

3. Motomasa, K.; Yoshihiro, O.; Isao, K., Marine Natural Products. XXVIII. The Structures of Sarasinosides A1, A2, A3, B1, B2, B3, C1, C2, and C3, Nine New Norlanostane-Triterpenoidal Oligoglycosides from the Palauan Marine Sponge Asteropus sarasinosum. *Chem. Pharm. Bull.* **1991,** 39, (11), 2867-2877.

4. Santalova, E. A.; Denisenko, V. A.; Dmitrenok, P. S.; Berdyshev, D. V.; Stonik, V. A., Two New Sarasinosides from the Sponge Melophlus Sarasinorum. *Nat. Prod. Commun.* **2006,** 1, (4), 265-271.

5. Espada, A.; Jiménez, C.; Rodríguez, J.; Crews, P.; Riguera, R., Sarasinosides D-G: four new triterpenoid saponins from the sponge asteropus sarasinosum. *Tetrahedron* **1992,** 48, (40), 8685-8696.

6. Lee, H.-S.; Seo, Y.; Cho, K. W.; Rho, J.-R.; Shin, J.; Paul, V. J., New Triterpenoid Saponins from the Sponge Melophlus isis. *J. Nat. Prod.* **2000,** 63, (7), 915-919.

7. Dai, H.-F.; Edrada, R. A.; Ebel, R.; Nimtz, M.; Wray, V.; Proksch, P., Norlanostane Triterpenoidal Saponins from the Marine Sponge Melophlus sarassinorum. *J. Nat. Prod.* **2005,** 68, (8), 1231-1237.

8. Lee, J.-H.; Jeon, J.-e.; Lee, Y.-J.; Lee, H.-S.; Sim, C. J.; Oh, K.-B.; Shin, J., Nortriterpene Glycosides of the Sarasinoside Class from the Sponge Lipastrotethya sp. *J. Nat. Prod.* **2012,** 75, (7), 1365-1372.

9. Puilingi, C. G.; Kudo, Y.; Cho, Y.; Konoki, K.; Yotsu-Yamashita, M., A new sarasinoside congener, sarasinoside M2, from a marine sponge collected in the Solomon Islands. *Biosci. Biotechnol. Biochem.* **2017,** 81, (2), 222-225.

10. Aoki, S.; Higuchi, K.; Ye, Y.; Satari, R.; Kobayashi, M., Melophlins A and B, Novel Tetramic Acids Reversing the Phenotype of ras-Transformed Cells, from the Marine Sponge Melophlus sarassinorum. *Tetrahedron* **2000,** 56, (13), 1833-1836.

11. Wang, C. Y.; Wang, B. G.; Wiryowidagdo, S.; Wray, V.; van Soest, R.; Steube, K. G.; Guan, H. S.; Proksch, P.; Ebel, R., Melophlins C-O, thirteen novel tetramic acids from the marine sponge Melophlus sarassinorum. *J. Nat. Prod.* **2003,** 66, (1), 51-56.

12. Xu, J.; Hasegawa, M.; Harada, K.; Kobayashi, H.; Nagai, H.; Namikoshi, M., Melophlins P, Q, R, and S: four new tetramic acid derivatives, from two Palauan marine sponges of the genus Melophlus. *Chem. Pharm. Bull.* **2006,** 54, (6), 852-854.

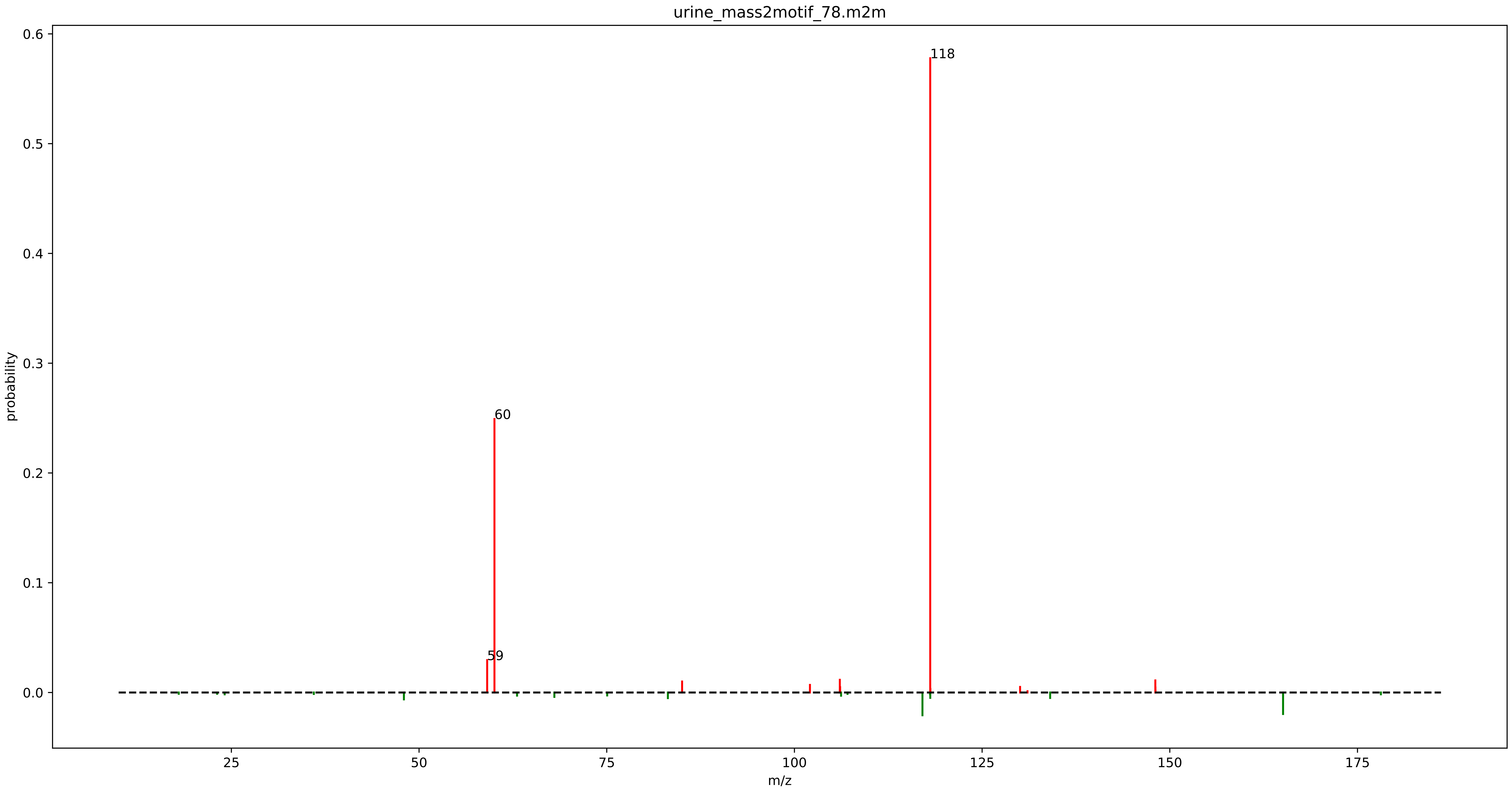

urine_mass2motif_78.m2m
probability
m/z
118
60
59
0.6
0.5
0.4
0.3
0.2
0.1
0.0
25
50
75
100
125
150
175

urine_mass2motif_78.m2m

fragment_118.0875	: 0.58
fragment_60.0425	: 0.25
fragment_59.0725	: 0.03
loss_117.0525	: 0.02
loss_165.0875	: 0.02
fragment_106.0475	: 0.01
fragment_148.0725	: 0.01
fragment_85.0375	: 0.01
fragment_102.0675	: 0.01
loss_47.9825	: 0.01
loss_83.1425	: 0.01
fragment_130.0575	: 0.01

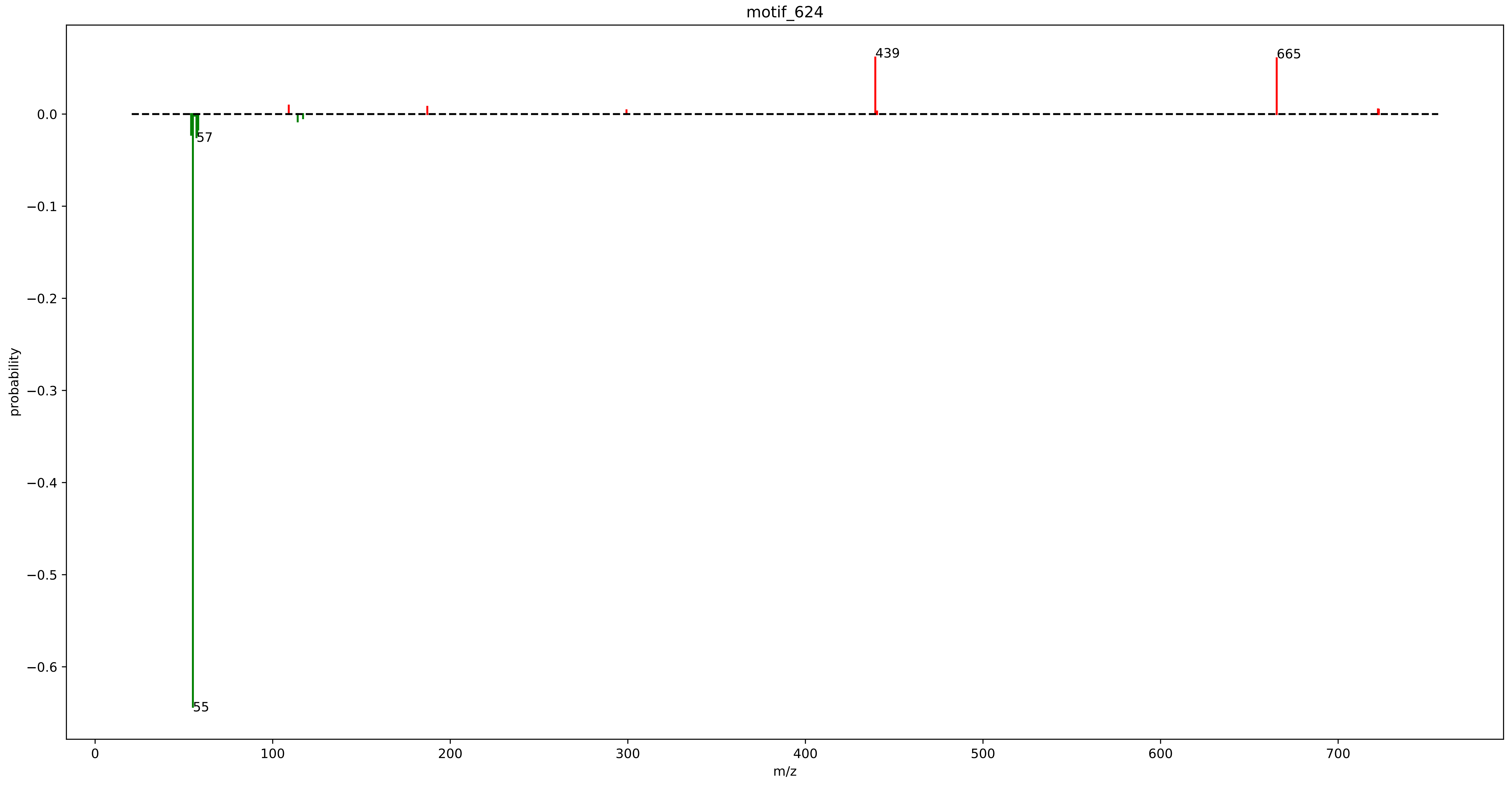
motif_624
439
665
57
55
0.0
−0.1
−0.2
−0.3
−0.4
−0.5
−0.6
probability
0
100
200
300
400
500
600
700
m/z

motif_624

loss_55.0625	: 0.64
fragment_439.3575	: 0.06
fragment_665.4025	: 0.06
loss_57.0775	: 0.03
loss_57.5675	: 0.02
loss_54.0575	: 0.02
loss_58.0675	: 0.02
fragment_109.0475	: 0.01
loss_114.0825	: 0.01
fragment_187.0675	: 0.01
fragment_722.4175	: 0.01

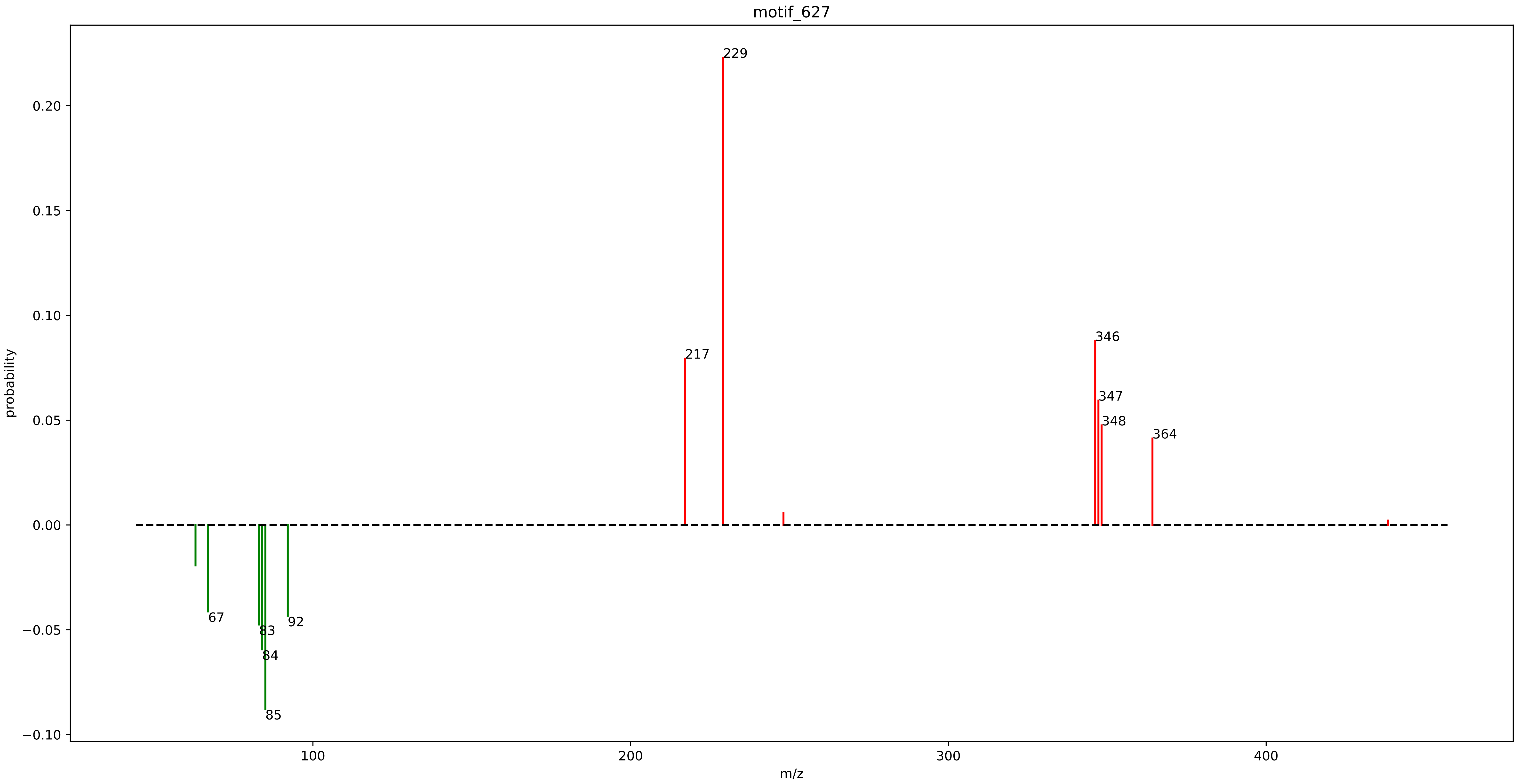

motif_627
probability
m/z
0.20
0.15
0.10
0.05
0.00
−0.05
−0.10
100
200
300
400
229
217
346
347
348
364
67
83
84
85
92

motif_627

fragment_229.1025	: 0.22
loss_85.0275	: 0.09
fragment_346.2225	: 0.09
fragment_217.1175	: 0.08
fragment_347.2275	: 0.06
loss_84.0225	: 0.06
fragment_348.2225	: 0.05
loss_83.0275	: 0.05
loss_92.0625	: 0.04
loss_67.0125	: 0.04
fragment_364.2325	: 0.04
loss_63.0375	: 0.02
fragment_248.0875	: 0.01

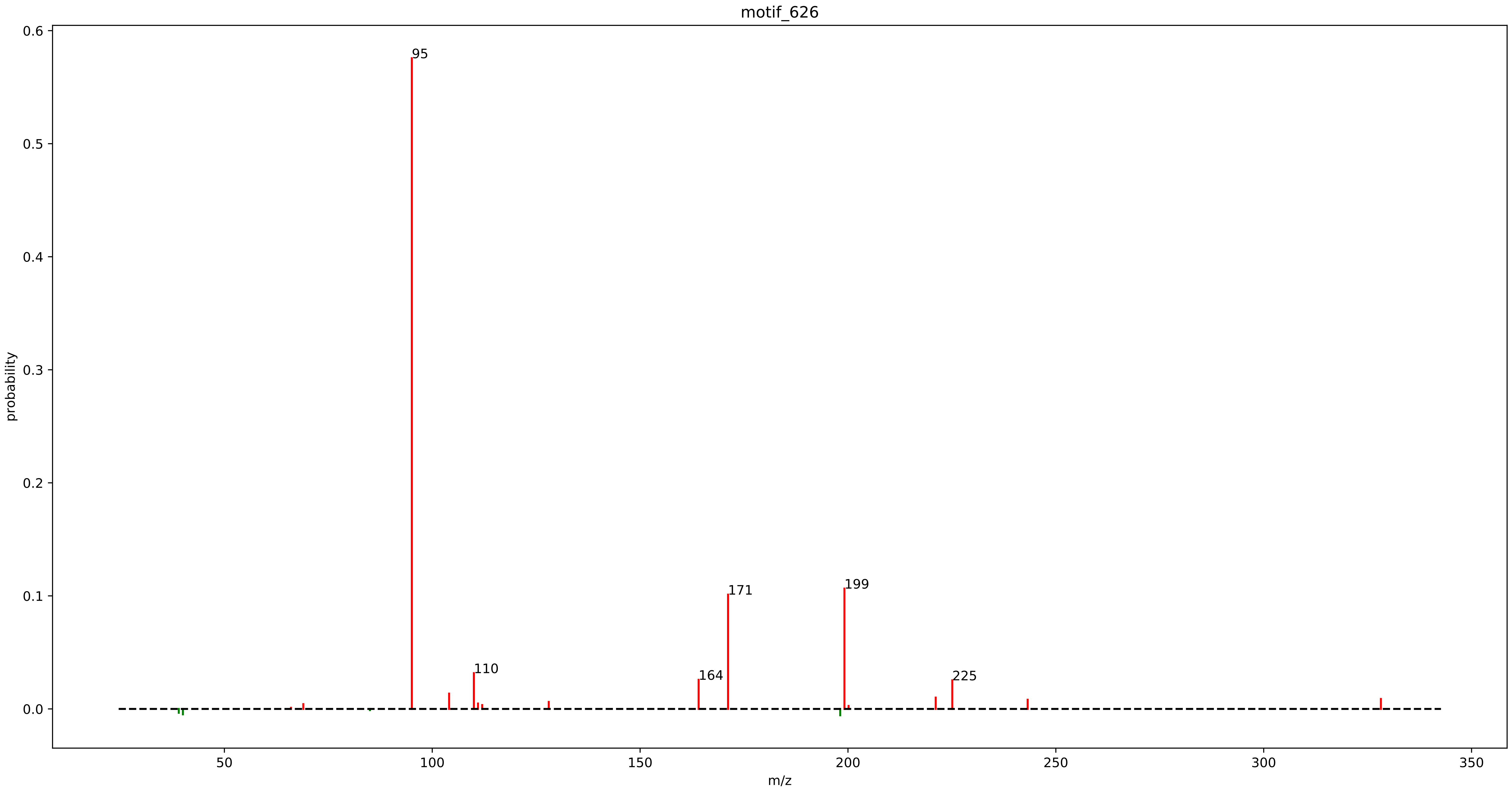
motif_626
probability
m/z
0.0
0.1
0.2
0.3
0.4
0.5
0.6
50
100
150
200
250
300
350
95
110
164
171
199
225

motif_626

fragment_95.0825	: 0.58
fragment_199.1425	: 0.11
fragment_171.1475	: 0.10
fragment_110.0075	: 0.03
fragment_164.0725	: 0.03
fragment_225.0875	: 0.03
fragment_104.0525	: 0.01
fragment_221.0975	: 0.01
fragment_328.1875	: 0.01
fragment_243.2125	: 0.01
fragment_128.0175	: 0.01
loss_198.1375	: 0.01

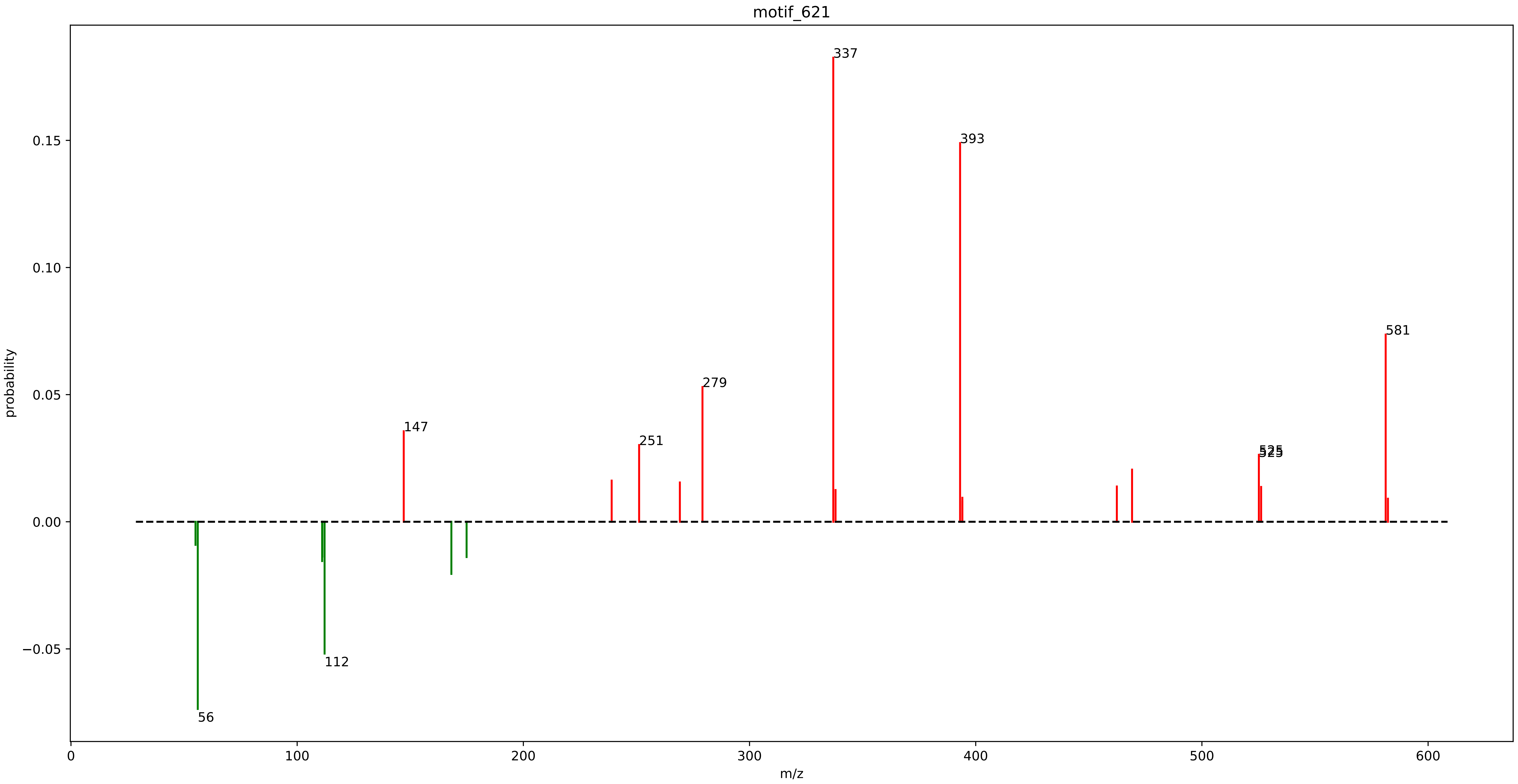

motif_621
probability
m/z
337
393
581
279
147
251
525
525
56
112
0.15
0.10
0.05
0.00
−0.05
0
100
200
300
400
500
600

motif_621

fragment_337.0225	: 0.18
fragment_393.0875	: 0.15
fragment_581.2425	: 0.07
loss_56.0575	: 0.07
fragment_279.1925	: 0.05
loss_112.1225	: 0.05
fragment_147.1175	: 0.04
fragment_251.1825	: 0.03
fragment_525.1825	: 0.03
fragment_525.1775	: 0.03
fragment_469.1175	: 0.02
loss_168.1825	: 0.02
fragment_239.0475	: 0.02
fragment_269.1975	: 0.02
loss_111.0425	: 0.02
loss_174.9075	: 0.01
fragment_462.3925	: 0.01
loss_111.1175	: 0.01
fragment_526.1825	: 0.01
fragment_338.0275	: 0.01
fragment_394.0925	: 0.01
fragment_582.2475	: 0.01
loss_55.0575	: 0.01
fragment_394.0875	: 0.01

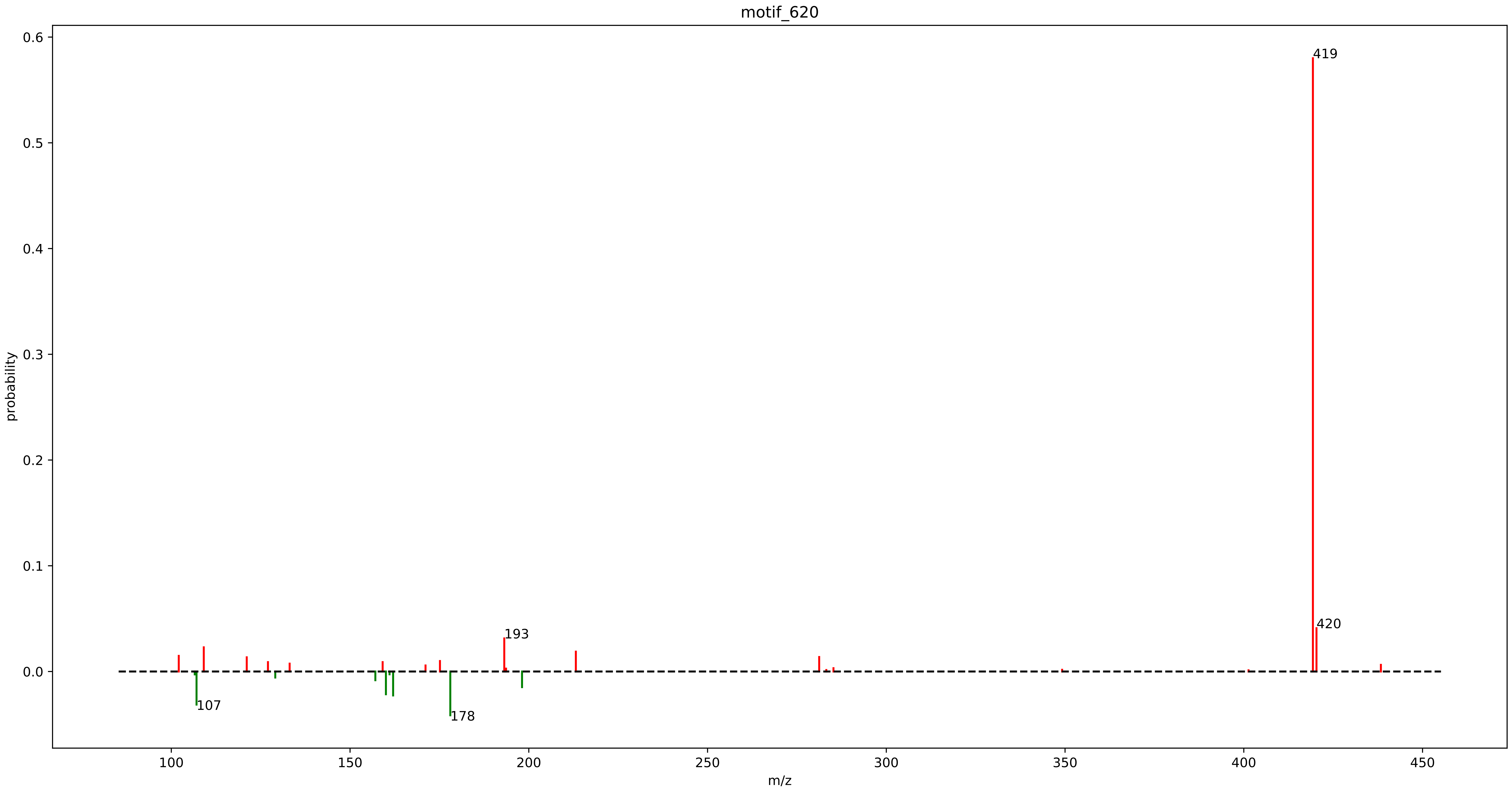

motif_620
419
420
193
107
178
probability
m/z
0.6
0.5
0.4
0.3
0.2
0.1
0.0
100
150
200
250
300
350
400
450

motif_620

fragment_419.3325	: 0.58
loss_178.0425	: 0.04
fragment_420.3325	: 0.04
loss_107.0575	: 0.03
fragment_193.1375	: 0.03
fragment_109.0825	: 0.02
loss_162.0475	: 0.02
loss_160.0325	: 0.02
fragment_213.1625	: 0.02
loss_198.1075	: 0.01
fragment_102.0875	: 0.01
fragment_281.2275	: 0.01
fragment_121.1025	: 0.01
fragment_175.1475	: 0.01
fragment_159.1175	: 0.01
fragment_127.0375	: 0.01
loss_157.0775	: 0.01
fragment_133.1025	: 0.01
fragment_438.3475	: 0.01
loss_129.0825	: 0.01
fragment_171.1125	: 0.01

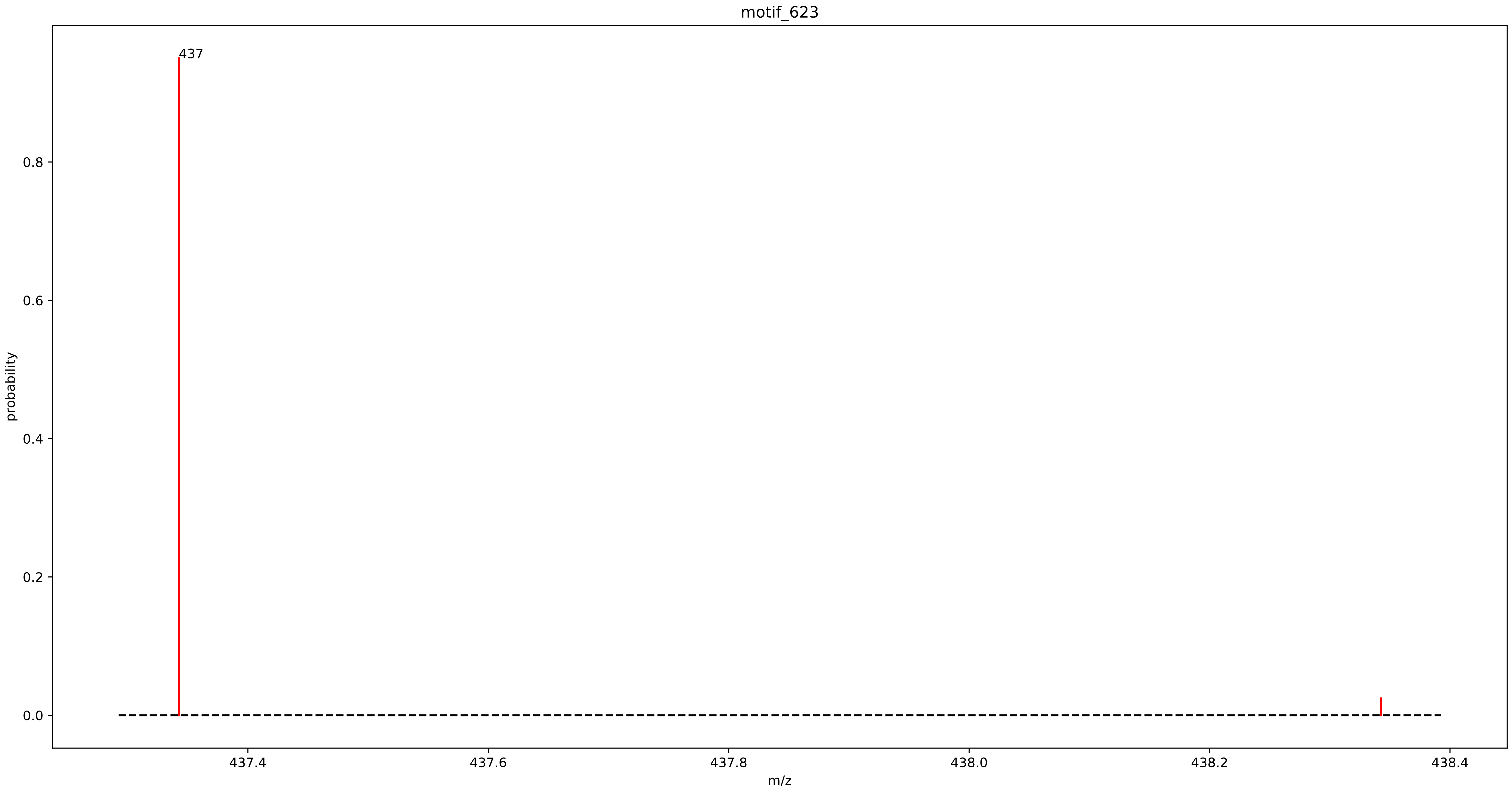
motif_623
437
probability
0.0
0.2
0.4
0.6
0.8
437.4
437.6
437.8
438.0
438.2
438.4
m/z

motif_623

fragment_437.3425	: 0.95
fragment_438.3425	: 0.02

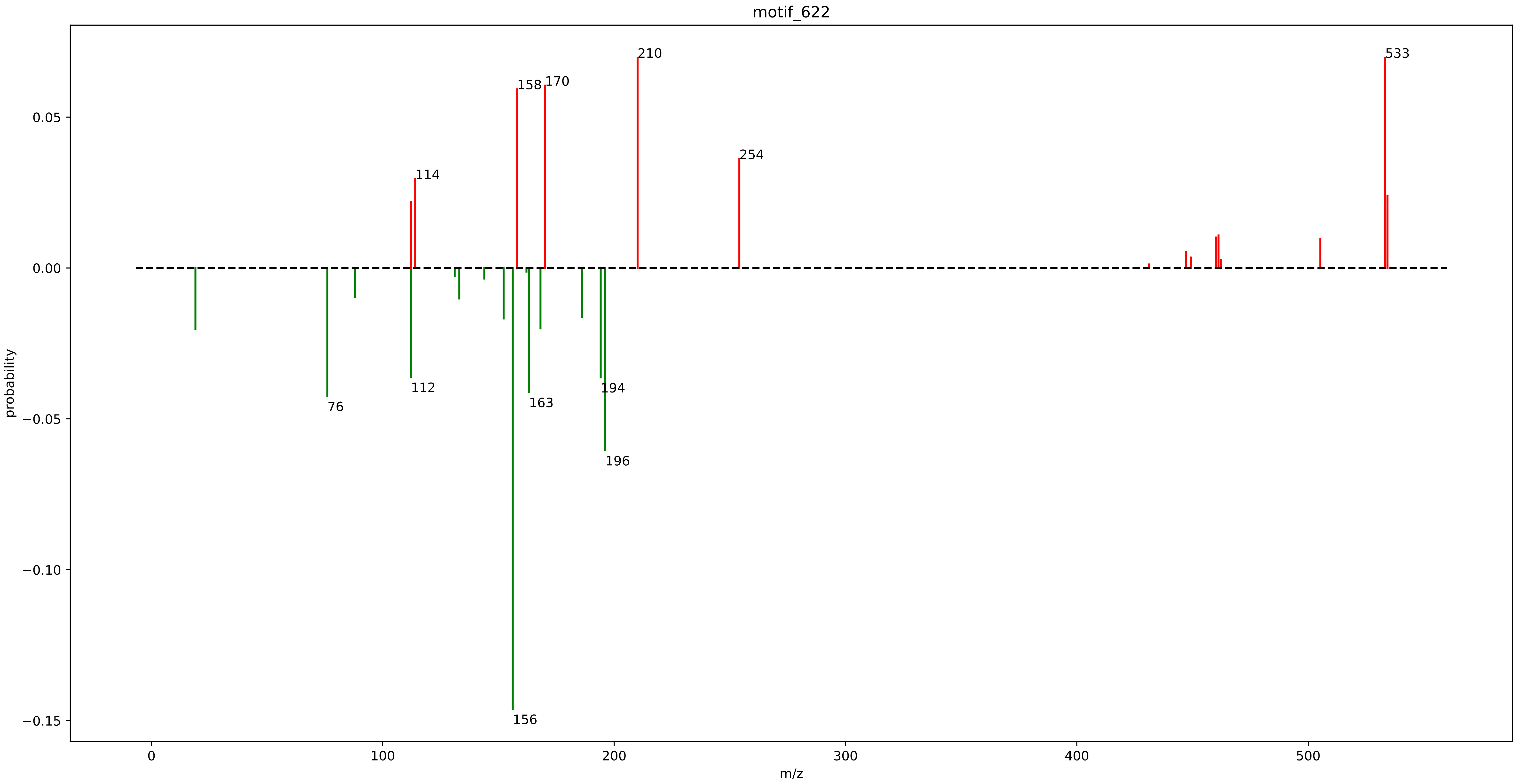
motif_622
probability
m/z
0.05
0.00
−0.05
−0.10
−0.15
0
100
200
300
400
500
114
158
170
210
254
533
76
112
163
194
196
156

motif_622

loss_156.1475	: 0.15
fragment_210.1125	: 0.07
fragment_533.2575	: 0.07
fragment_170.0825	: 0.06
loss_196.1825	: 0.06
fragment_158.0825	: 0.06
loss_76.0425	: 0.04
loss_163.1775	: 0.04
loss_194.1575	: 0.04
loss_112.1425	: 0.04
fragment_254.1175	: 0.04
fragment_114.0575	: 0.03
fragment_534.2575	: 0.02
fragment_112.0725	: 0.02
loss_19.0025	: 0.02
loss_168.1475	: 0.02
loss_152.2175	: 0.02
loss_186.1625	: 0.02
fragment_461.2325	: 0.01
fragment_460.2275	: 0.01
loss_133.0475	: 0.01
fragment_505.2225	: 0.01
loss_88.0475	: 0.01
fragment_447.2175	: 0.01

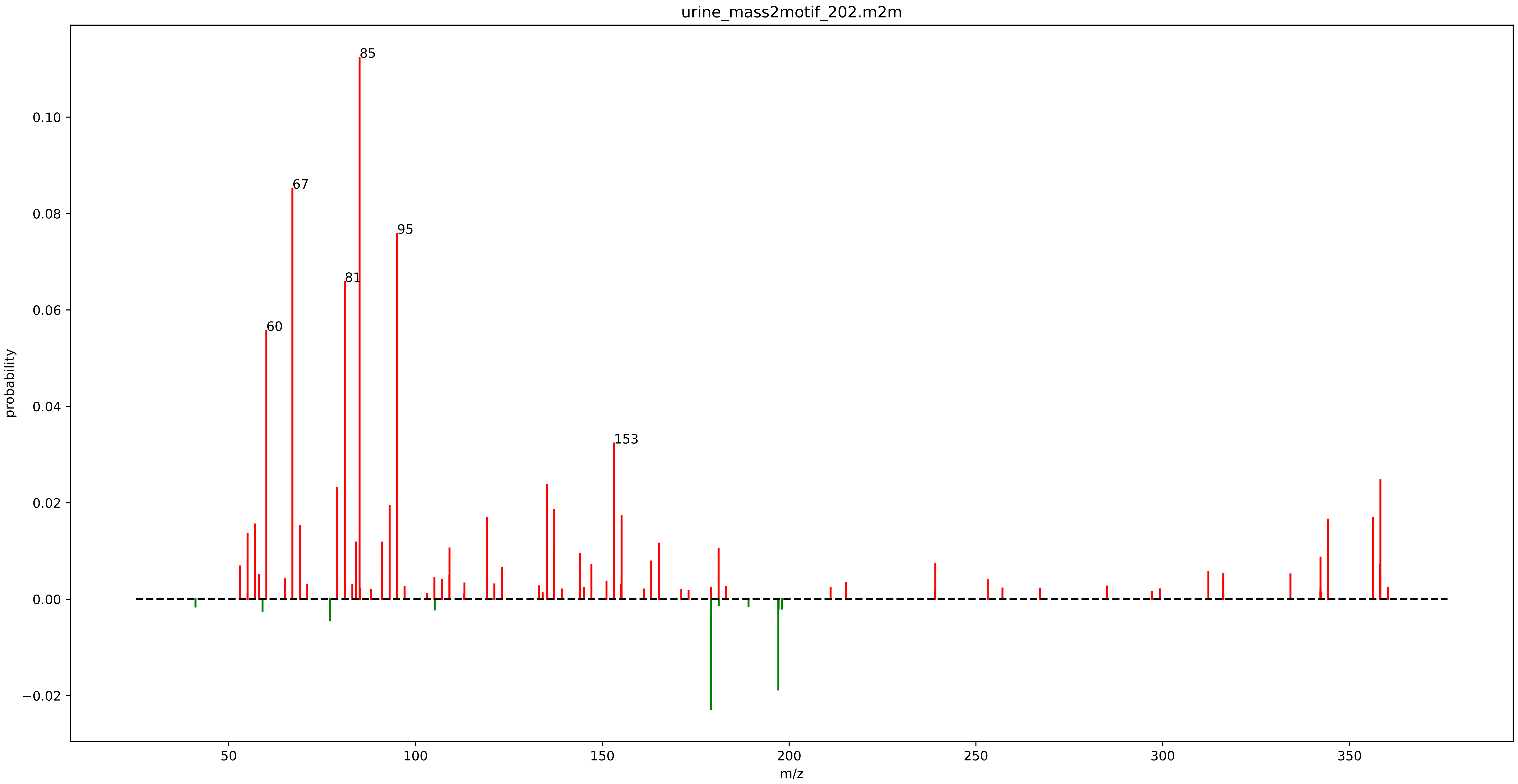
urine_mass2motif_202.m2m
probability
m/z
85
67
95
81
60
153
0.10
0.08
0.06
0.04
0.02
0.00
−0.02
50
100
150
200
250
300
350

urine_mass2motif_202.m2m

fragment_85.0275	: 0.11
fragment_67.0525	: 0.09
fragment_95.0875	: 0.08
fragment_81.0675	: 0.07
fragment_60.0825	: 0.06
fragment_153.1275	: 0.03
fragment_358.2575	: 0.02
fragment_135.1175	: 0.02
fragment_79.0525	: 0.02
loss_179.1125	: 0.02
fragment_93.0675	: 0.02
loss_197.1275	: 0.02
fragment_137.1325	: 0.02
fragment_155.1425	: 0.02
fragment_119.0875	: 0.02
fragment_356.2425	: 0.02
fragment_344.2075	: 0.02
fragment_57.0325	: 0.02
fragment_69.0725	: 0.02
fragment_55.0525	: 0.01
fragment_84.0825	: 0.01
fragment_91.0525	: 0.01
fragment_165.0925	: 0.01
fragment_137.0975	: 0.01
fragment_109.1025	: 0.01
fragment_181.1225	: 0.01
fragment_144.1025	: 0.01
fragment_342.2275	: 0.01
fragment_163.1125	: 0.01
fragment_137.0575	: 0.01
fragment_239.1275	: 0.01
fragment_147.0775	: 0.01
fragment_358.2175	: 0.01
fragment_53.0375	: 0.01
fragment_123.1175	: 0.01
fragment_344.2425	: 0.01
loss_179.1525	: 0.01
fragment_312.2175	: 0.01
fragment_316.2125	: 0.01
fragment_334.1825	: 0.01
fragment_58.0675	: 0.01

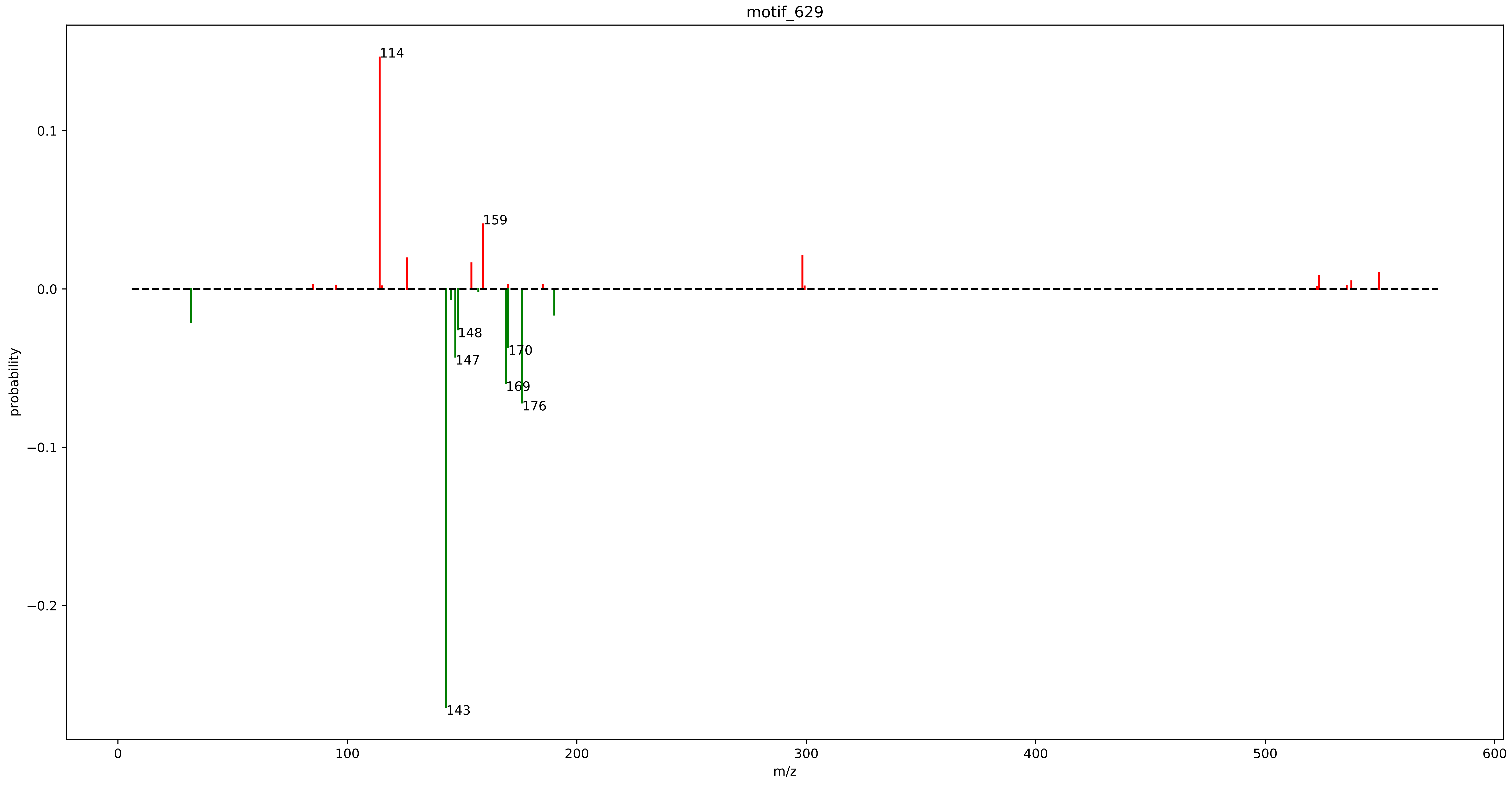
motif_629
114
159
148
147
170
169
176
143
0.1
0.0
−0.1
−0.2
probability
0
100
200
300
400
500
600
m/z

motif_629

loss_143.0575	: 0.26
fragment_114.0575	: 0.15
loss_176.1575	: 0.07
loss_169.0725	: 0.06
loss_147.0675	: 0.04
fragment_159.0925	: 0.04
loss_170.0675	: 0.04
loss_148.1225	: 0.03
loss_176.1525	: 0.02
loss_31.8925	: 0.02
fragment_298.3075	: 0.02
fragment_126.0575	: 0.02
loss_190.1675	: 0.02
fragment_154.0525	: 0.02
fragment_549.4975	: 0.01
fragment_523.4875	: 0.01
loss_169.0675	: 0.01
loss_145.0725	: 0.01

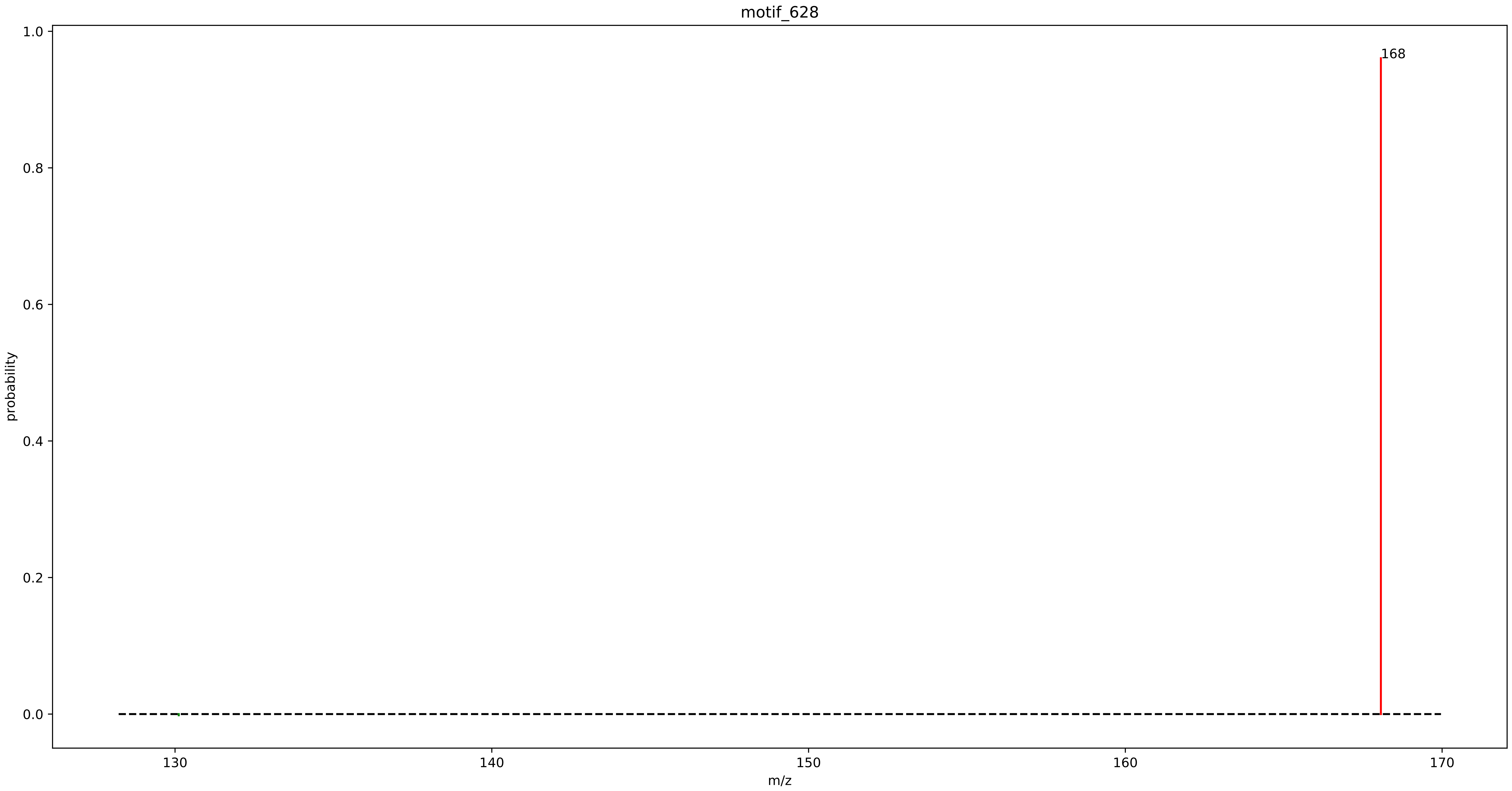

motif_628
168
probability
m/z
1.0
0.8
0.6
0.4
0.2
0.0
130
140
150
160
170

motif_628

fragment_168.0675 : 0.96

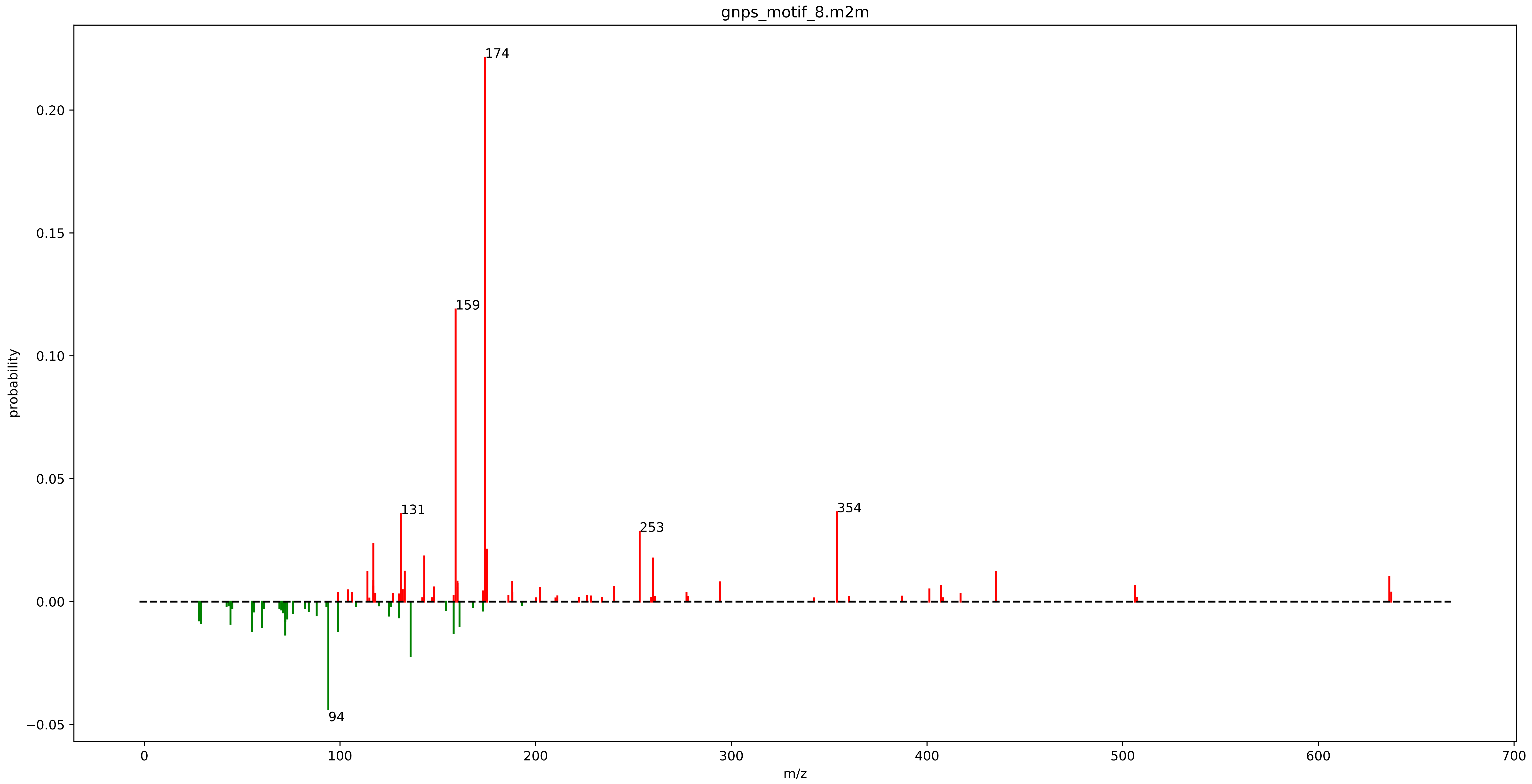
gnps_motif_8.m2m
probability
m/z
0.20
0.15
0.10
0.05
0.00
−0.05
0
100
200
300
400
500
600
700
174
159
131
253
354
94

gnps_motif_8.m2m

fragment_174.0925	: 0.22
fragment_159.0675	: 0.12
loss_94.0525	: 0.04
fragment_354.0525	: 0.04
fragment_131.0725	: 0.04
fragment_253.1225	: 0.03
fragment_117.0475	: 0.02
loss_136.0725	: 0.02
fragment_175.0925	: 0.02
fragment_143.0725	: 0.02
fragment_259.9975	: 0.02
loss_72.0425	: 0.01
loss_158.0675	: 0.01
fragment_133.0775	: 0.01
loss_99.0725	: 0.01
fragment_435.1575	: 0.01
fragment_114.0375	: 0.01
loss_55.0075	: 0.01
loss_60.0825	: 0.01
loss_161.0825	: 0.01
fragment_636.2625	: 0.01
loss_44.0475	: 0.01
loss_29.0275	: 0.01
fragment_117.0425	: 0.01
fragment_160.0425	: 0.01
fragment_188.0825	: 0.01
loss_72.0725	: 0.01
fragment_294.1075	: 0.01
loss_27.9975	: 0.01
fragment_160.0825	: 0.01
loss_73.0525	: 0.01
loss_60.0425	: 0.01
loss_130.0725	: 0.01
fragment_407.1575	: 0.01
fragment_506.1875	: 0.01
fragment_117.0575	: 0.01
fragment_240.1225	: 0.01
fragment_148.0375	: 0.01
loss_125.1225	: 0.01
loss_88.0775	: 0.01
fragment_202.0975	: 0.01
fragment_160.0725	: 0.01

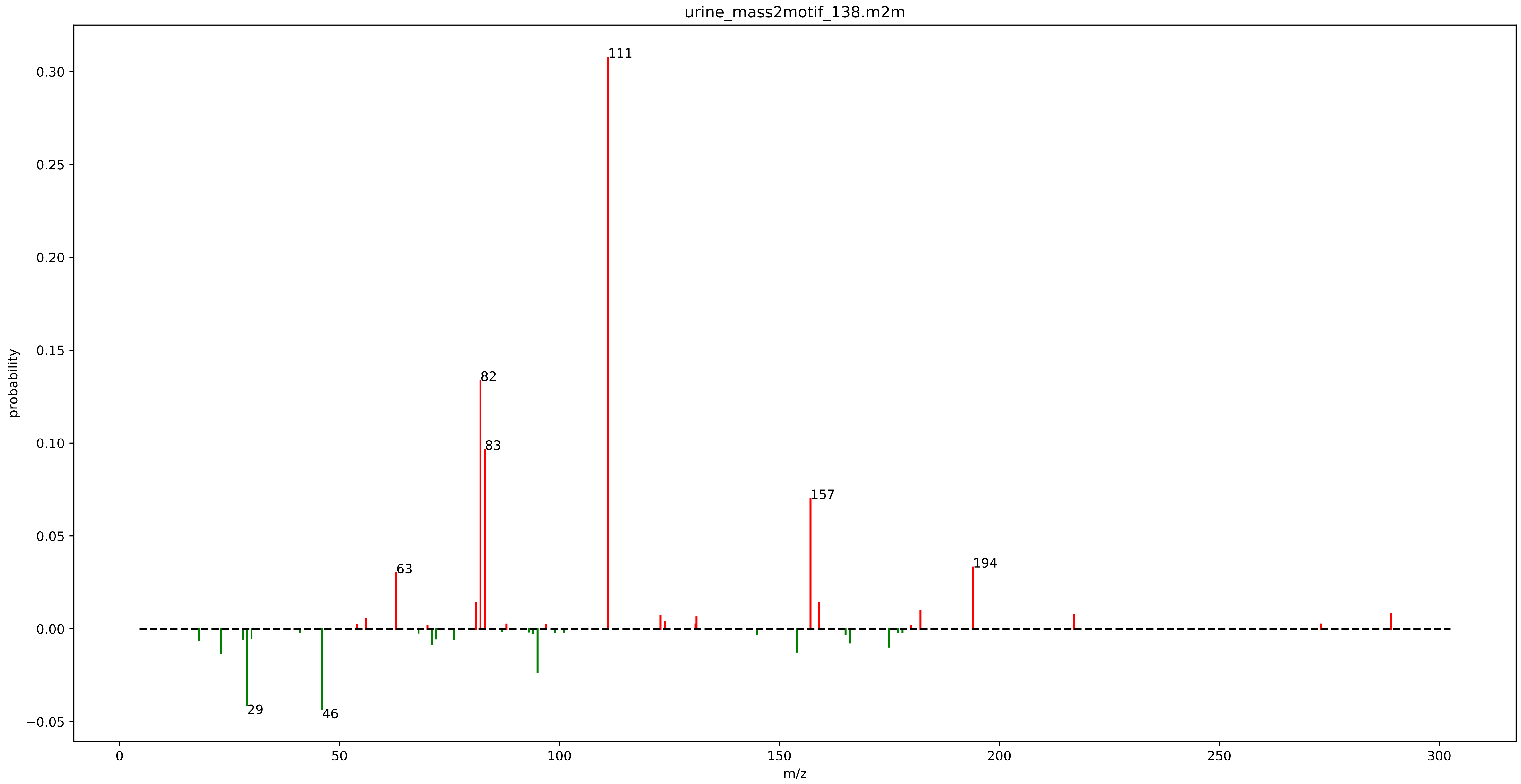
urine_mass2motif_138.m2m
probability
m/z
0.30
0.25
0.20
0.15
0.10
0.05
0.00
−0.05
0
50
100
150
200
250
300
111
82
83
157
63
194
29
46

urine_mass2motif_138.m2m

fragment_111.0575	: 0.31
fragment_82.0525	: 0.13
fragment_83.0625	: 0.10
fragment_157.0625	: 0.07
loss_46.0775	: 0.04
loss_29.0025	: 0.04
fragment_193.9975	: 0.03
fragment_62.9275	: 0.03
loss_95.0475	: 0.02
fragment_81.0425	: 0.01
fragment_159.0275	: 0.01
loss_23.0175	: 0.01
loss_154.0825	: 0.01
fragment_111.0925	: 0.01
loss_174.9825	: 0.01
fragment_182.0625	: 0.01
loss_71.0025	: 0.01
fragment_289.0475	: 0.01
loss_166.0825	: 0.01
fragment_217.0175	: 0.01
fragment_122.9625	: 0.01
loss_28.9925	: 0.01
loss_29.0225	: 0.01
fragment_131.1625	: 0.01
loss_18.0825	: 0.01
loss_76.0175	: 0.01
fragment_289.0425	: 0.01
fragment_56.0475	: 0.01
loss_27.9875	: 0.01
loss_72.0325	: 0.01
loss_30.0125	: 0.01

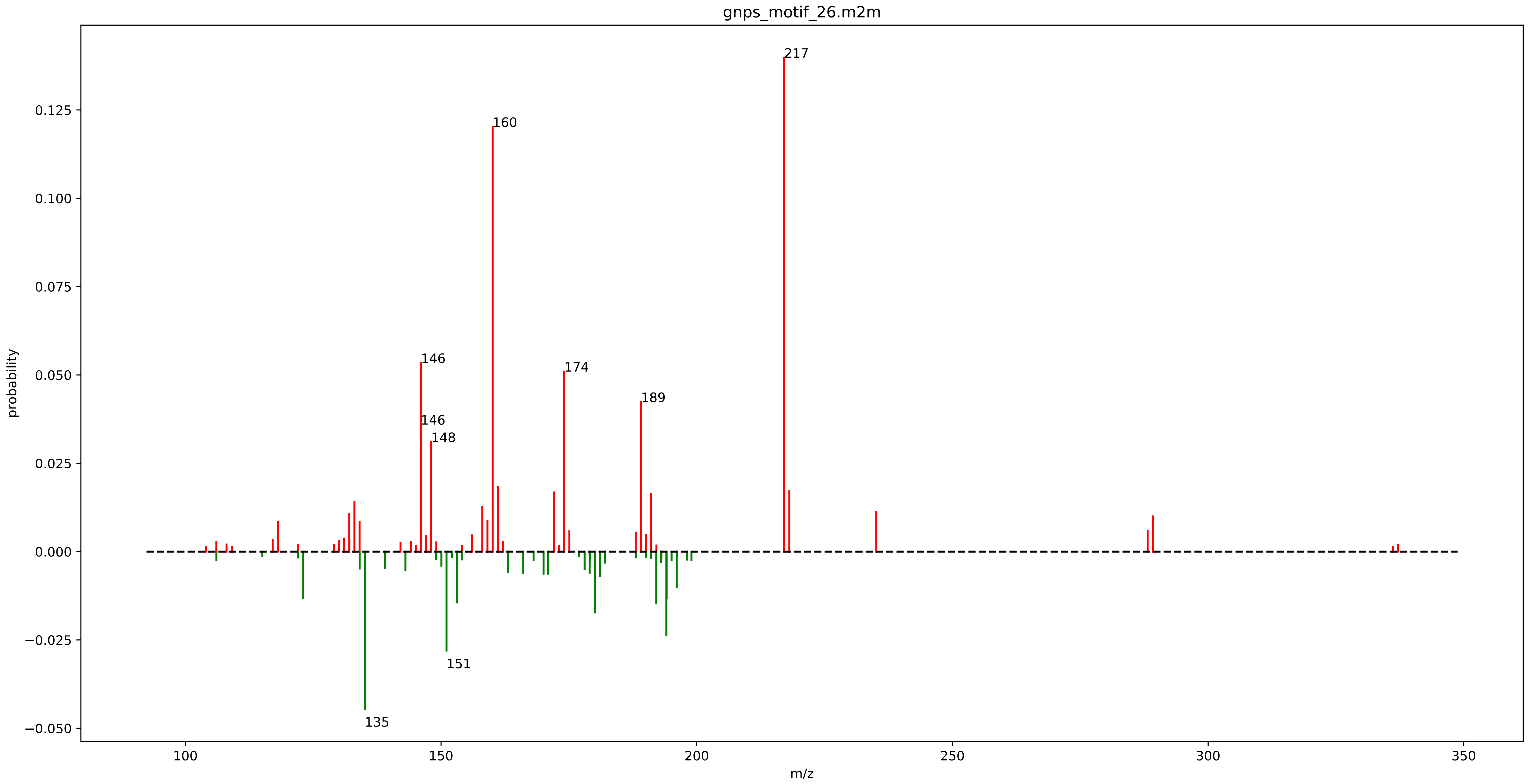
gnps_motif_26.m2m
probability
m/z
0.125
0.100
0.075
0.050
0.025
0.000
−0.025
−0.050
100
150
200
250
300
350
217
160
146
174
189
146
148
151
135

gnps_motif_26.m2m

fragment_217.0975	: 0.14
fragment_160.0775	: 0.12
fragment_146.0625	: 0.05
fragment_174.0925	: 0.05
loss_135.0675	: 0.04
fragment_189.1025	: 0.04
fragment_146.0225	: 0.04
fragment_148.0775	: 0.03
loss_151.0625	: 0.03
loss_194.0675	: 0.02
fragment_161.0825	: 0.02
loss_180.0875	: 0.02
fragment_218.1025	: 0.02
fragment_172.0775	: 0.02
fragment_191.1175	: 0.02
loss_192.0875	: 0.01
loss_153.0775	: 0.01
fragment_133.0525	: 0.01
loss_194.1075	: 0.01
loss_123.0675	: 0.01
fragment_158.0625	: 0.01
fragment_235.1075	: 0.01
fragment_132.0425	: 0.01
loss_196.0825	: 0.01
fragment_289.1775	: 0.01
loss_180.0525	: 0.01
fragment_159.0675	: 0.01
fragment_134.0575	: 0.01
fragment_118.0675	: 0.01
loss_181.0725	: 0.01
loss_170.9625	: 0.01
loss_170.0475	: 0.01
loss_166.0725	: 0.01
loss_179.0575	: 0.01
fragment_288.1725	: 0.01
loss_163.0625	: 0.01
fragment_175.0925	: 0.01
fragment_188.0725	: 0.01
loss_143.0375	: 0.01

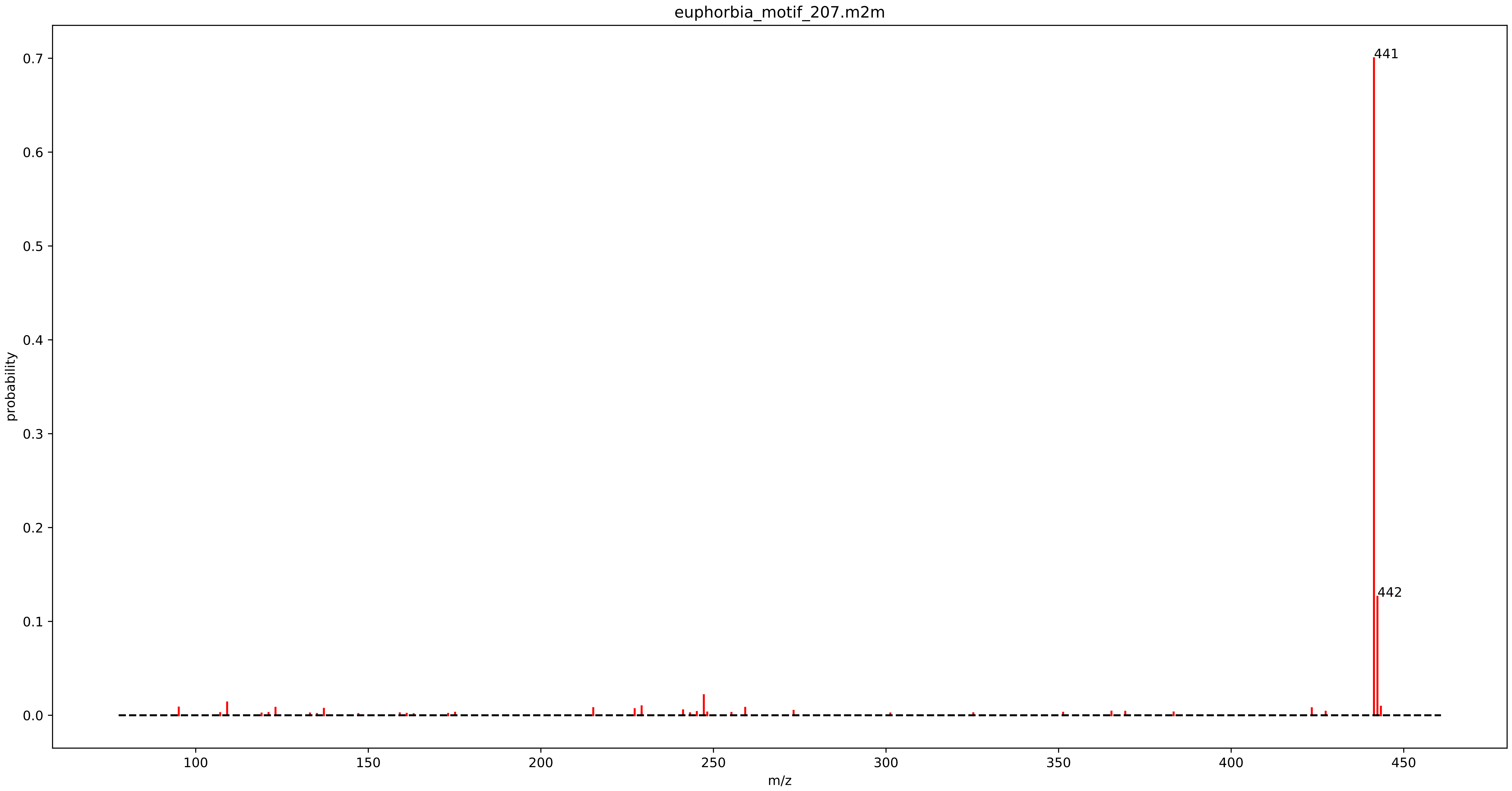

euphorbia_motif_207.m2m
441
442
probability
m/z
0.0
0.1
0.2
0.3
0.4
0.5
0.6
0.7
100
150
200
250
300
350
400
450

euphorbia_motif_207.m2m

fragment_441.3725	: 0.70
fragment_442.3775	: 0.13
fragment_247.2075	: 0.02
fragment_109.1025	: 0.01
fragment_229.1925	: 0.01
fragment_443.3775	: 0.01
fragment_95.0875	: 0.01
fragment_123.1175	: 0.01
fragment_259.2025	: 0.01
fragment_215.1775	: 0.01
fragment_423.3625	: 0.01
fragment_137.1325	: 0.01
fragment_227.1775	: 0.01
fragment_241.1925	: 0.01

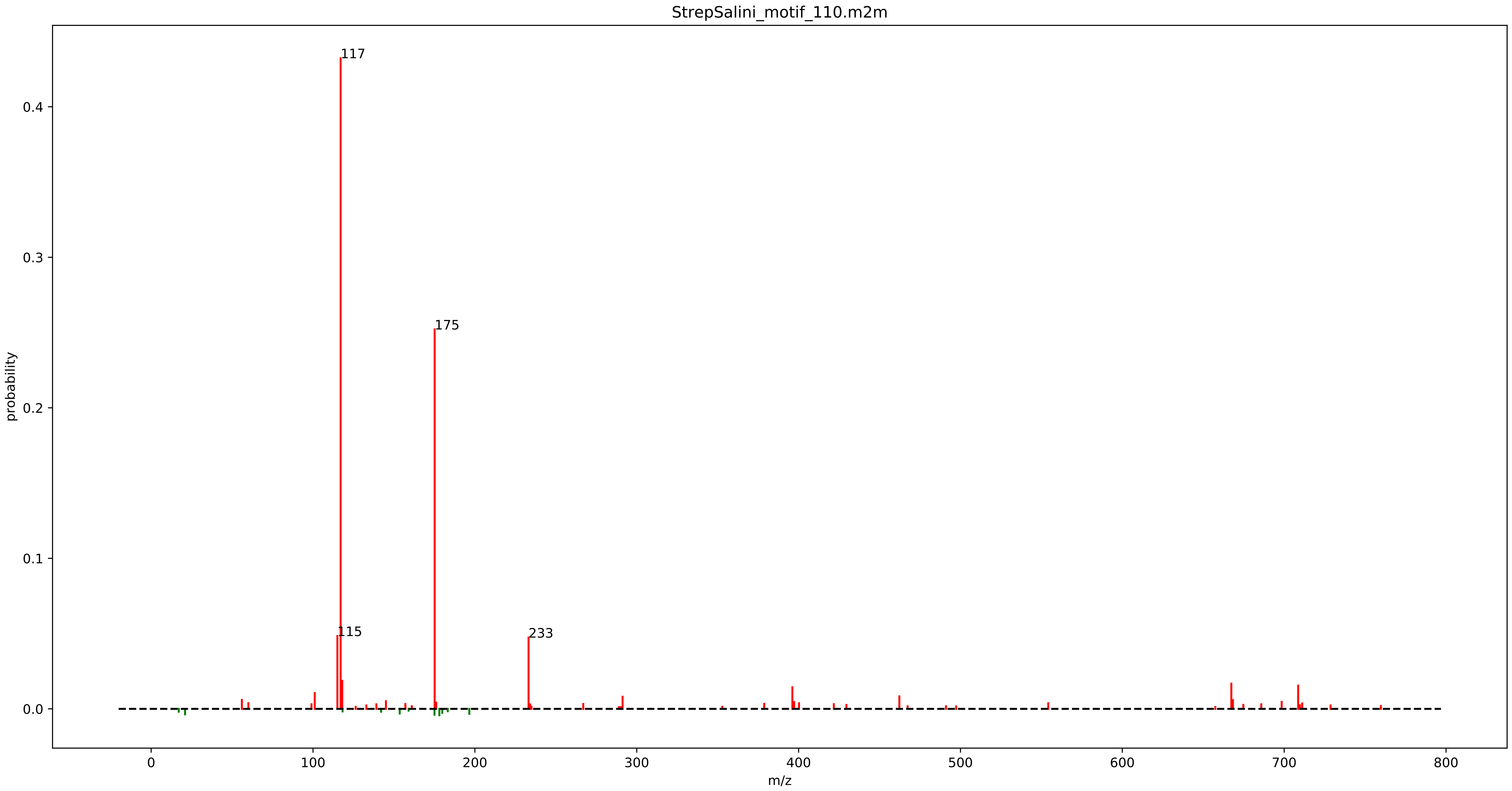
StrepSalini_motif_110.m2m
117
175
115
233
probability
0.4
0.3
0.2
0.1
0.0
0
100
200
300
400
500
600
700
800
m/z

StrepSalini_motif_110.m2m

fragment_117.0500	: 0.43
fragment_175.1500	: 0.25
fragment_115.0500	: 0.05
fragment_233.1500	: 0.05
fragment_118.0500	: 0.02
fragment_667.3500	: 0.02
fragment_708.6500	: 0.02
fragment_396.1500	: 0.01
fragment_101.0500	: 0.01
fragment_462.2500	: 0.01
fragment_291.2500	: 0.01
fragment_56.0500	: 0.01
fragment_668.3500	: 0.01
fragment_145.0500	: 0.01

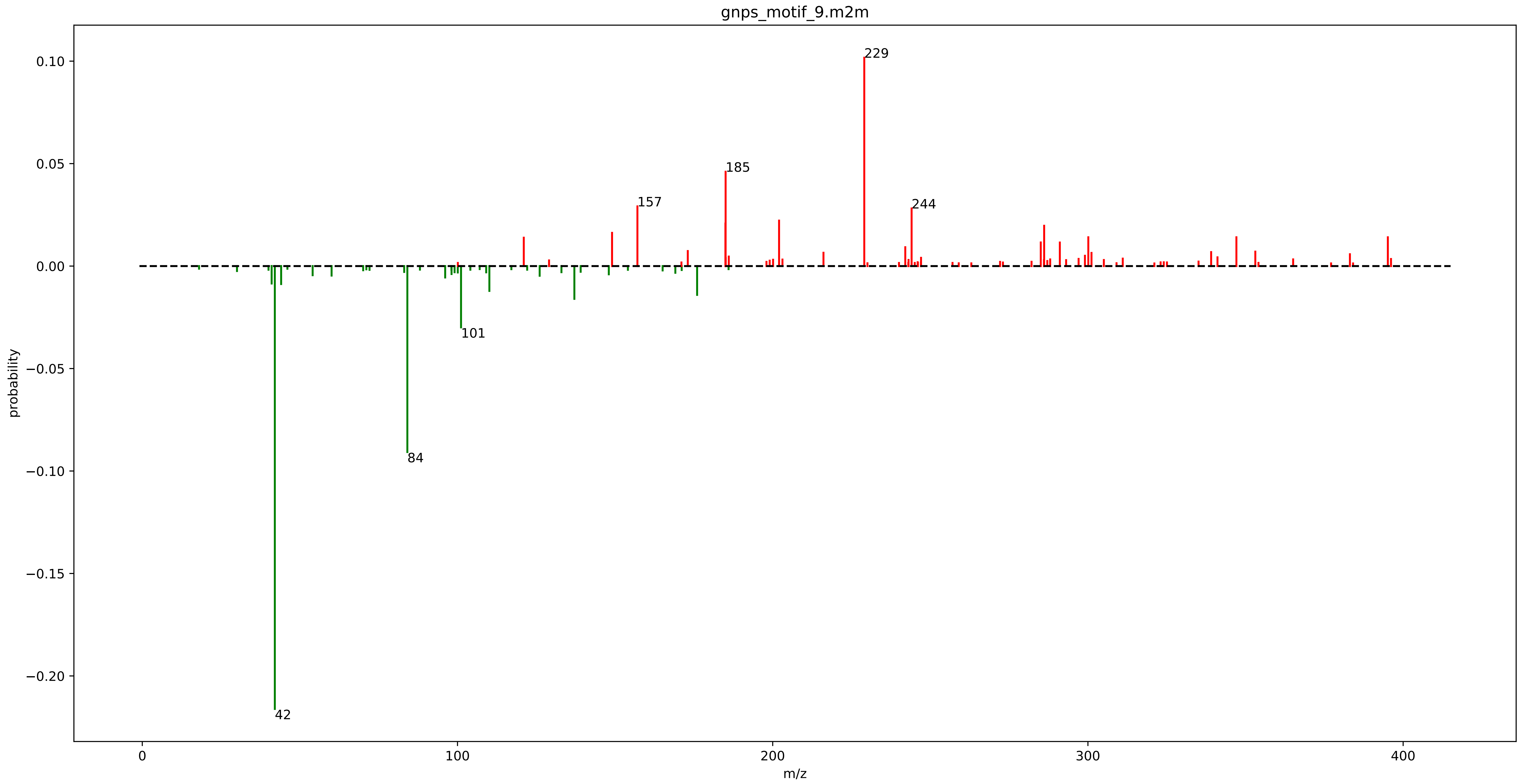

gnps_motif_9.m2m
probability
m/z
0.10
0.05
0.00
−0.05
−0.10
−0.15
−0.20
0
100
200
300
400
229
185
157
244
101
84
42

gnps_motif_9.m2m

loss_42.0475	: 0.22
fragment_229.0475	: 0.10
loss_84.0925	: 0.09
fragment_185.0575	: 0.05
loss_101.1225	: 0.03
fragment_157.0775	: 0.03
fragment_244.0625	: 0.03
fragment_202.0175	: 0.02
fragment_184.9925	: 0.02
fragment_286.1125	: 0.02
fragment_149.0325	: 0.02
loss_137.0775	: 0.02
loss_176.0175	: 0.01
fragment_347.1025	: 0.01
fragment_395.1475	: 0.01
fragment_300.1225	: 0.01
fragment_121.0275	: 0.01
loss_110.1125	: 0.01
fragment_285.0375	: 0.01
fragment_291.0875	: 0.01
fragment_242.0575	: 0.01
loss_44.0525	: 0.01
loss_41.0425	: 0.01
fragment_173.0575	: 0.01
fragment_353.1025	: 0.01
fragment_339.0875	: 0.01
fragment_216.1025	: 0.01
fragment_301.1325	: 0.01
loss_41.0375	: 0.01
fragment_383.1125	: 0.01
loss_96.0925	: 0.01
fragment_299.0525	: 0.01

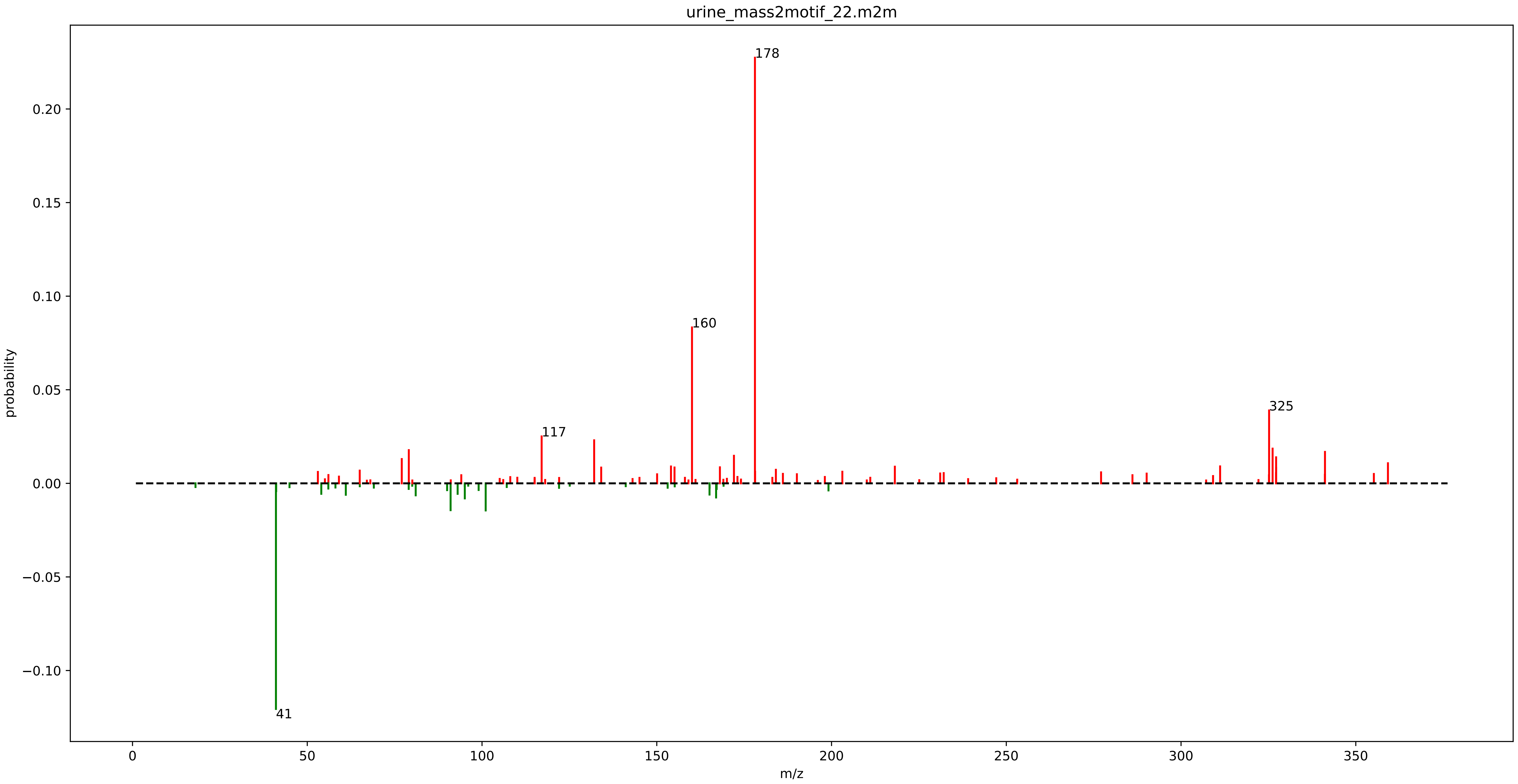
urine_mass2motif_22.m2m
probability
m/z
178
160
117
325
41
0.20
0.15
0.10
0.05
0.00
−0.05
−0.10
0
50
100
150
200
250
300
350

urine_mass2motif_22.m2m

fragment_178.0875	: 0.23
loss_41.0375	: 0.12
fragment_160.0775	: 0.08
fragment_325.1925	: 0.04
fragment_117.0575	: 0.03
fragment_132.0825	: 0.02
fragment_326.1975	: 0.02
fragment_79.0525	: 0.02
fragment_341.1825	: 0.02
fragment_172.0775	: 0.01
loss_101.0475	: 0.01
loss_91.0075	: 0.01
fragment_327.2025	: 0.01
fragment_77.0375	: 0.01
fragment_359.1975	: 0.01
fragment_311.1775	: 0.01
fragment_154.0625	: 0.01
fragment_218.1175	: 0.01
fragment_168.0625	: 0.01
fragment_155.0725	: 0.01
fragment_134.0975	: 0.01
fragment_327.2075	: 0.01
loss_95.0725	: 0.01
loss_166.9725	: 0.01
fragment_184.0775	: 0.01
fragment_65.0075	: 0.01
loss_81.0225	: 0.01
fragment_203.0925	: 0.01
fragment_178.1325	: 0.01
loss_61.0275	: 0.01
fragment_53.0375	: 0.01
loss_165.0875	: 0.01
fragment_277.1175	: 0.01
loss_93.0225	: 0.01
loss_54.0125	: 0.01
fragment_232.0925	: 0.01
fragment_327.1675	: 0.01
fragment_231.0875	: 0.01
fragment_290.1625	: 0.01
fragment_186.0875	: 0.01
fragment_355.1675	: 0.01

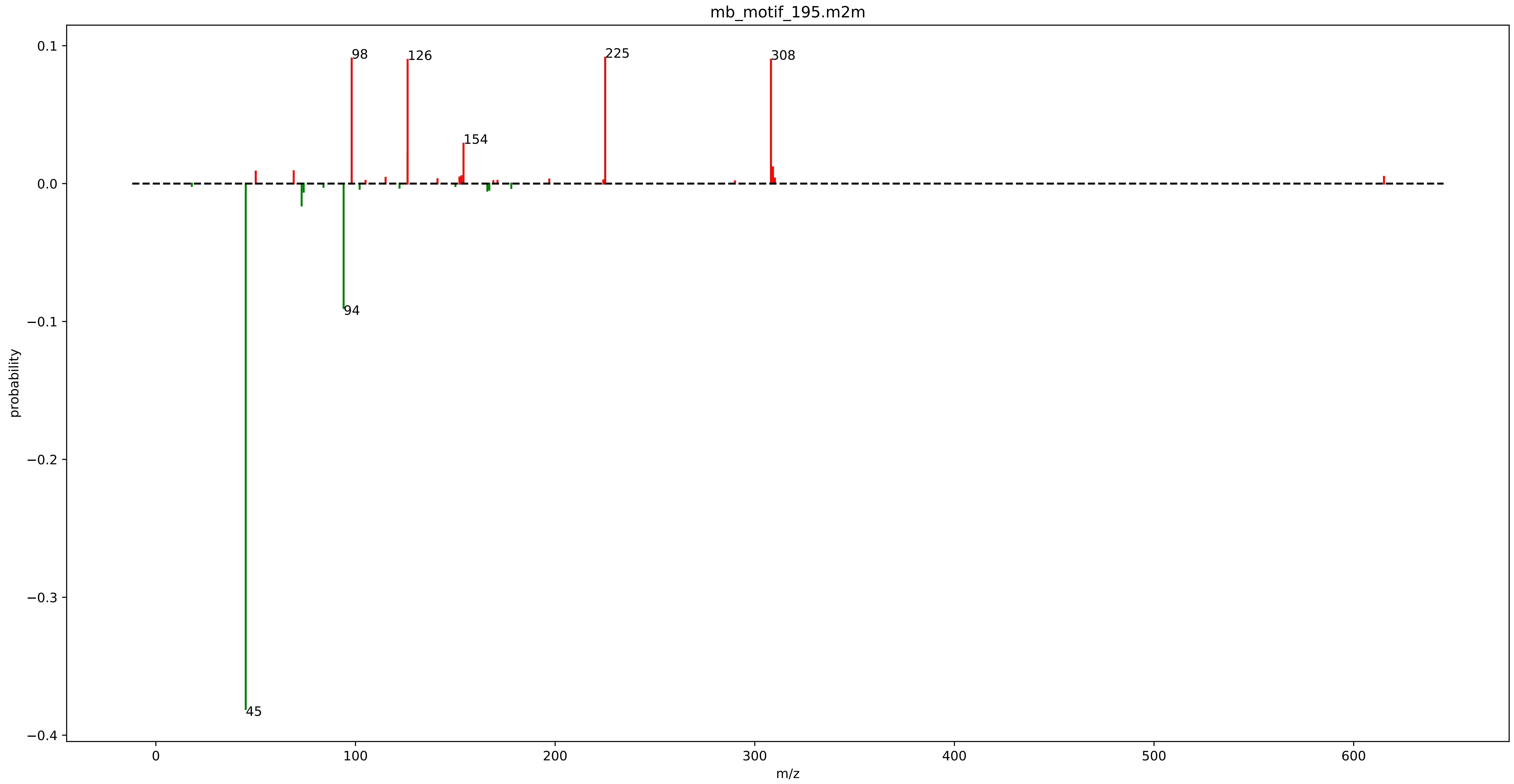
mb_motif_195.m2m
probability
m/z
0.1
0.0
−0.1
−0.2
−0.3
−0.4
0
100
200
300
400
500
600
98
126
225
308
154
94
45

mb_motif_195.m2m

loss_45.0225	: 0.38
fragment_225.0525	: 0.09
fragment_98.0625	: 0.09
loss_94.0425	: 0.09
fragment_308.0925	: 0.09
fragment_126.0925	: 0.09
fragment_154.0875	: 0.03
fragment_126.0575	: 0.02
loss_73.0175	: 0.02
fragment_309.0925	: 0.01
fragment_69.0325	: 0.01
fragment_50.0125	: 0.01
loss_74.0475	: 0.01
fragment_153.0675	: 0.01
loss_166.0275	: 0.01

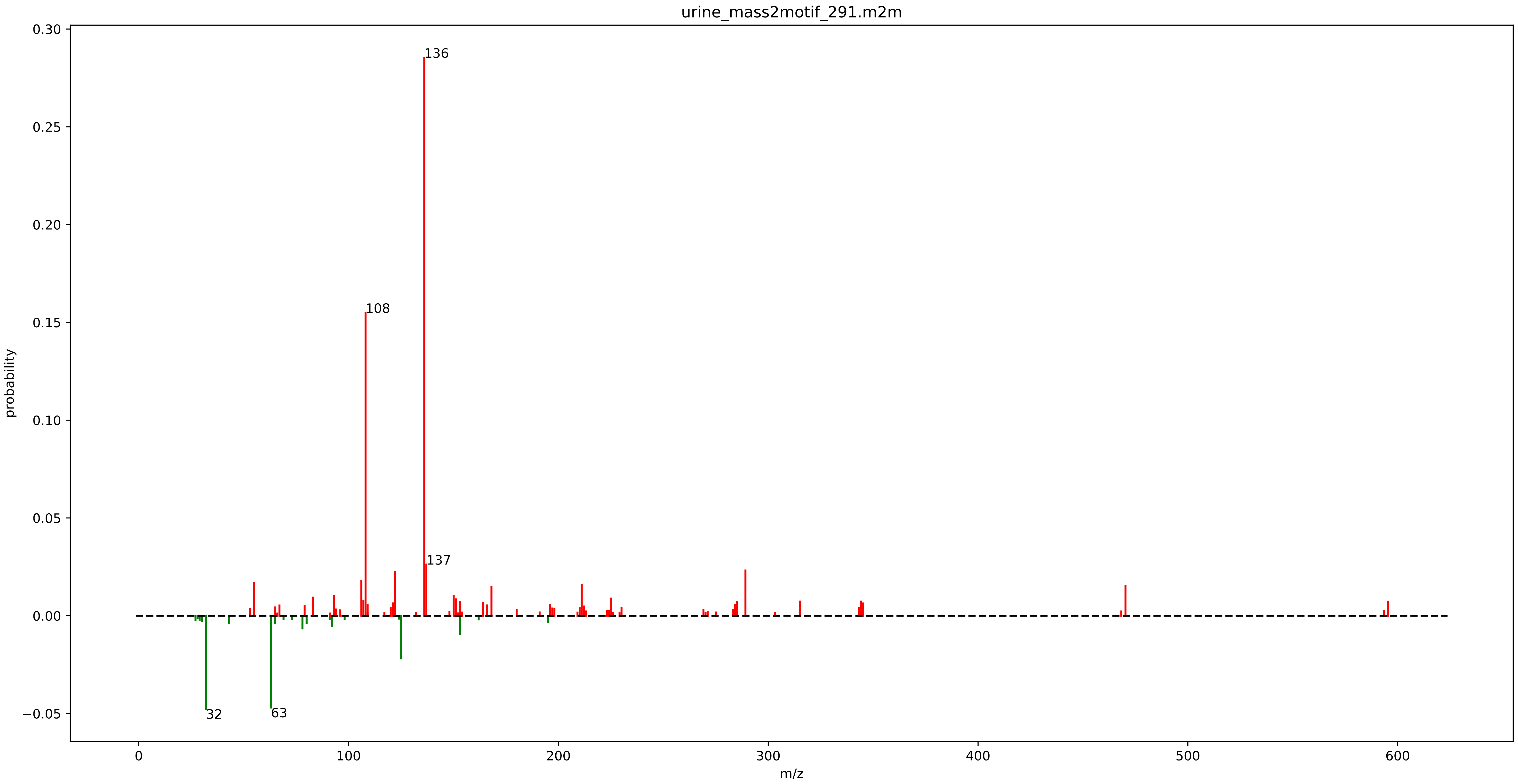
urine_mass2motif_291.m2m
probability
m/z
0.30
0.25
0.20
0.15
0.10
0.05
0.00
−0.05
0
100
200
300
400
500
600
136
108
137
32
63

urine_mass2motif_291.m2m

fragment_136.0775	: 0.29
fragment_108.0825	: 0.15
loss_32.0275	: 0.05
loss_62.9875	: 0.05
fragment_137.0825	: 0.03
fragment_289.1525	: 0.02
fragment_122.0625	: 0.02
loss_125.0825	: 0.02
fragment_106.0675	: 0.02
fragment_55.0525	: 0.02
fragment_211.1225	: 0.02
fragment_470.2625	: 0.02
fragment_168.1025	: 0.01
fragment_93.0575	: 0.01
fragment_150.0925	: 0.01
loss_153.0775	: 0.01
fragment_83.0875	: 0.01
fragment_225.1375	: 0.01
fragment_151.0975	: 0.01
fragment_107.0725	: 0.01

fragment_344.1725	: 0.01
fragment_315.2025	: 0.01
fragment_595.3525	: 0.01
fragment_153.1025	: 0.01
fragment_285.1625	: 0.01
fragment_164.0725	: 0.01
loss_78.0125	: 0.01
fragment_121.0875	: 0.01
fragment_345.1775	: 0.01
fragment_284.1375	: 0.01
fragment_196.0975	: 0.01
fragment_109.0875	: 0.01
fragment_166.0875	: 0.01
loss_91.9925	: 0.01
fragment_67.0525	: 0.01
fragment_79.0525	: 0.01

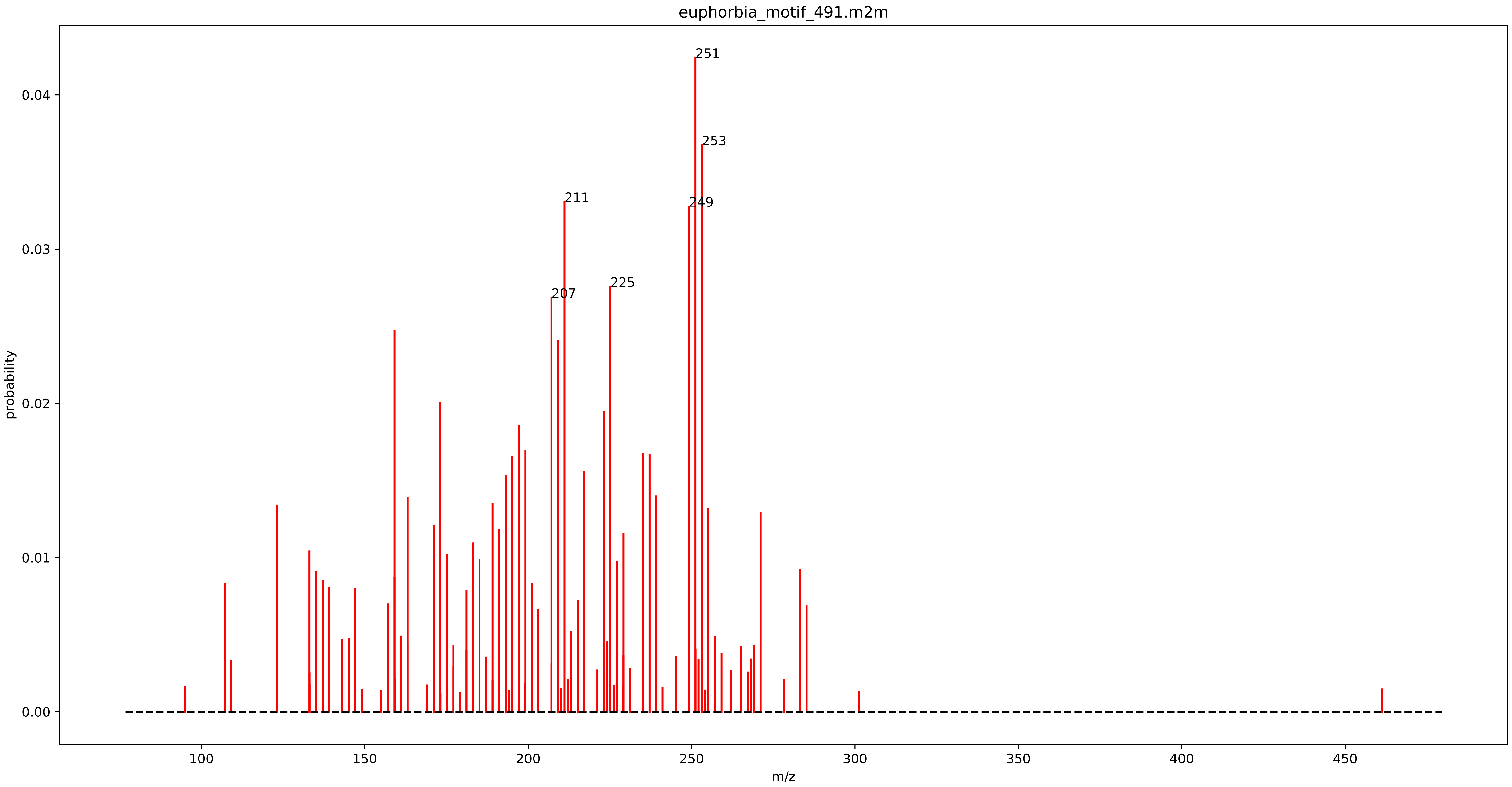

euphorbia_motif_491.m2m
probability
m/z
0.00
0.01
0.02
0.03
0.04
100
150
200
250
300
350
400
450
251
253
211
249
225
207

euphorbia_motif_491.m2m

fragment_251.1425 : 0.04
fragment_253.1225 : 0.04
fragment_211.1125 : 0.03
fragment_249.1625 : 0.03
fragment_225.1275 : 0.03
fragment_207.1175 : 0.03
fragment_159.0825 : 0.02
fragment_209.1325 : 0.02
fragment_209.0975 : 0.02
fragment_173.0975 : 0.02
fragment_223.1125 : 0.02
fragment_197.1325 : 0.02
fragment_253.1575 : 0.02
fragment_199.1125 : 0.02
fragment_235.1125 : 0.02
fragment_237.1275 : 0.02
fragment_195.1175 : 0.02
fragment_217.1225 : 0.02
fragment_193.0875 : 0.02
fragment_239.1075 : 0.01
fragment_163.1125 : 0.01
fragment_189.0925 : 0.01
fragment_123.0775 : 0.01
fragment_255.1375 : 0.01
fragment_271.1325 : 0.01
fragment_197.0975 : 0.01
fragment_171.0825 : 0.01
fragment_191.1075 : 0.01
fragment_229.1225 : 0.01
fragment_183.1175 : 0.01
fragment_133.0675 : 0.01
fragment_175.0725 : 0.01
fragment_185.0975 : 0.01
fragment_227.1075 : 0.01
fragment_227.1425 : 0.01
fragment_123.0425 : 0.01
fragment_283.1675 : 0.01
fragment_135.0775 : 0.01
fragment_175.0775 : 0.01
fragment_159.0775 : 0.01
fragment_137.0975 : 0.01
fragment_107.0875 : 0.01
fragment_201.0925 : 0.01
fragment_139.1125 : 0.01
fragment_147.0825 : 0.01
fragment_181.1025 : 0.01
fragment_171.0775 : 0.01
fragment_197.0925 : 0.01
fragment_215.1075 : 0.01
fragment_157.1025 : 0.01
fragment_285.1875 : 0.01
fragment_203.1075 : 0.01
fragment_235.1475 : 0.01
fragment_193.1025 : 0.01
fragment_171.1125 : 0.01
fragment_239.1775 : 0.01
fragment_135.0825 : 0.01
fragment_213.0925 : 0.01

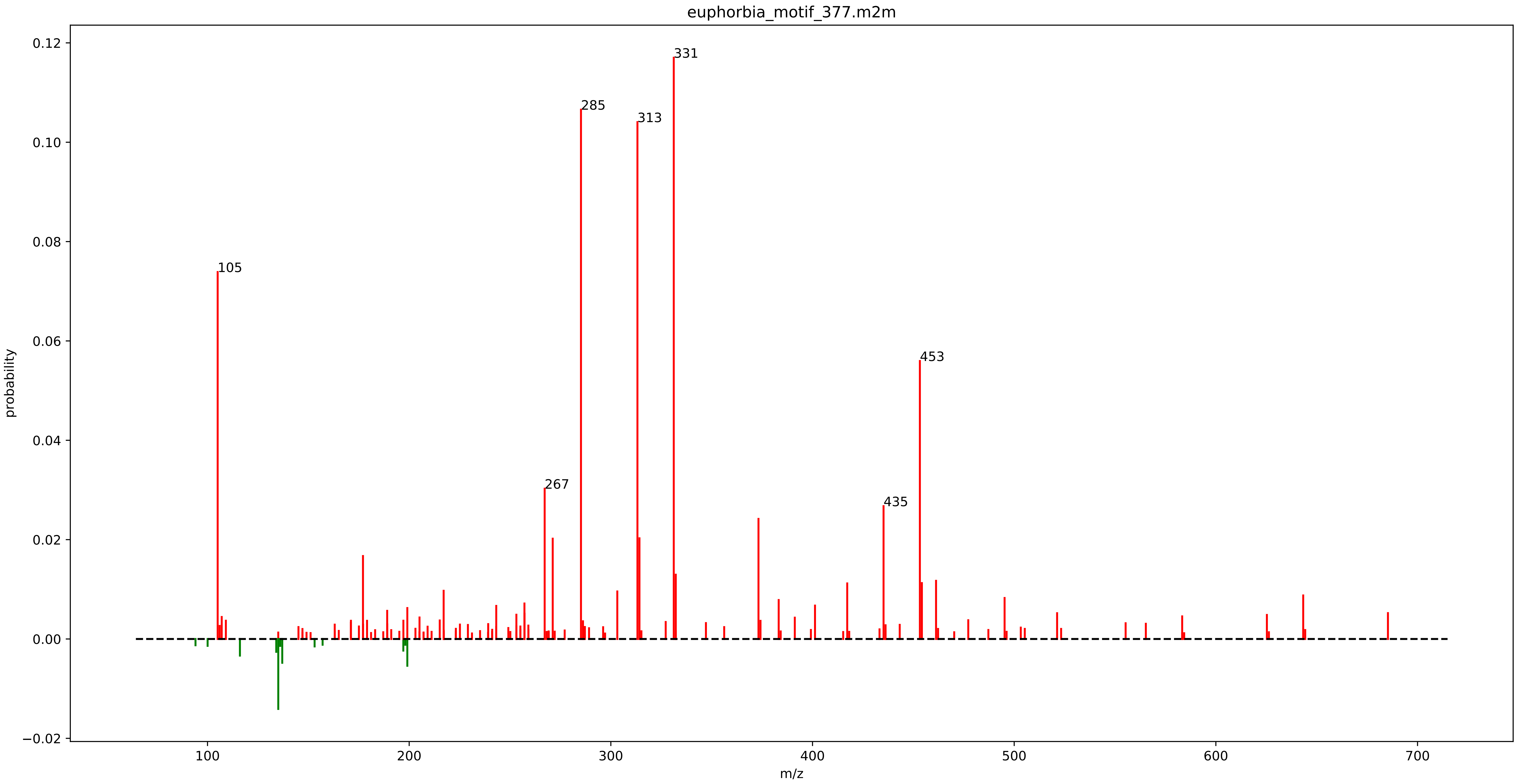
euphorbia_motif_377.m2m
probability
m/z
105
267
285
313
331
435
453
0.12
0.10
0.08
0.06
0.04
0.02
0.00
−0.02
100
200
300
400
500
600
700

euphorbia_motif_377.m2m

fragment_331.1925	: 0.12
fragment_285.1875	: 0.11
fragment_313.1825	: 0.10
fragment_105.0325	: 0.07
fragment_453.2325	: 0.06
fragment_267.1775	: 0.03
fragment_435.2175	: 0.03
fragment_373.2025	: 0.02
fragment_314.1875	: 0.02
fragment_271.1725	: 0.02
fragment_177.0925	: 0.02
loss_135.0875	: 0.01
fragment_332.1975	: 0.01
fragment_461.2525	: 0.01
fragment_454.2325	: 0.01
fragment_417.2075	: 0.01
fragment_217.0875	: 0.01
fragment_303.1975	: 0.01
fragment_643.3125	: 0.01
fragment_495.2375	: 0.01
fragment_383.2225	: 0.01
fragment_257.1525	: 0.01
fragment_401.2325	: 0.01
fragment_243.1775	: 0.01
fragment_199.0775	: 0.01
fragment_189.0925	: 0.01
loss_199.0775	: 0.01
fragment_685.3275	: 0.01
fragment_521.2775	: 0.01
fragment_257.1925	: 0.01

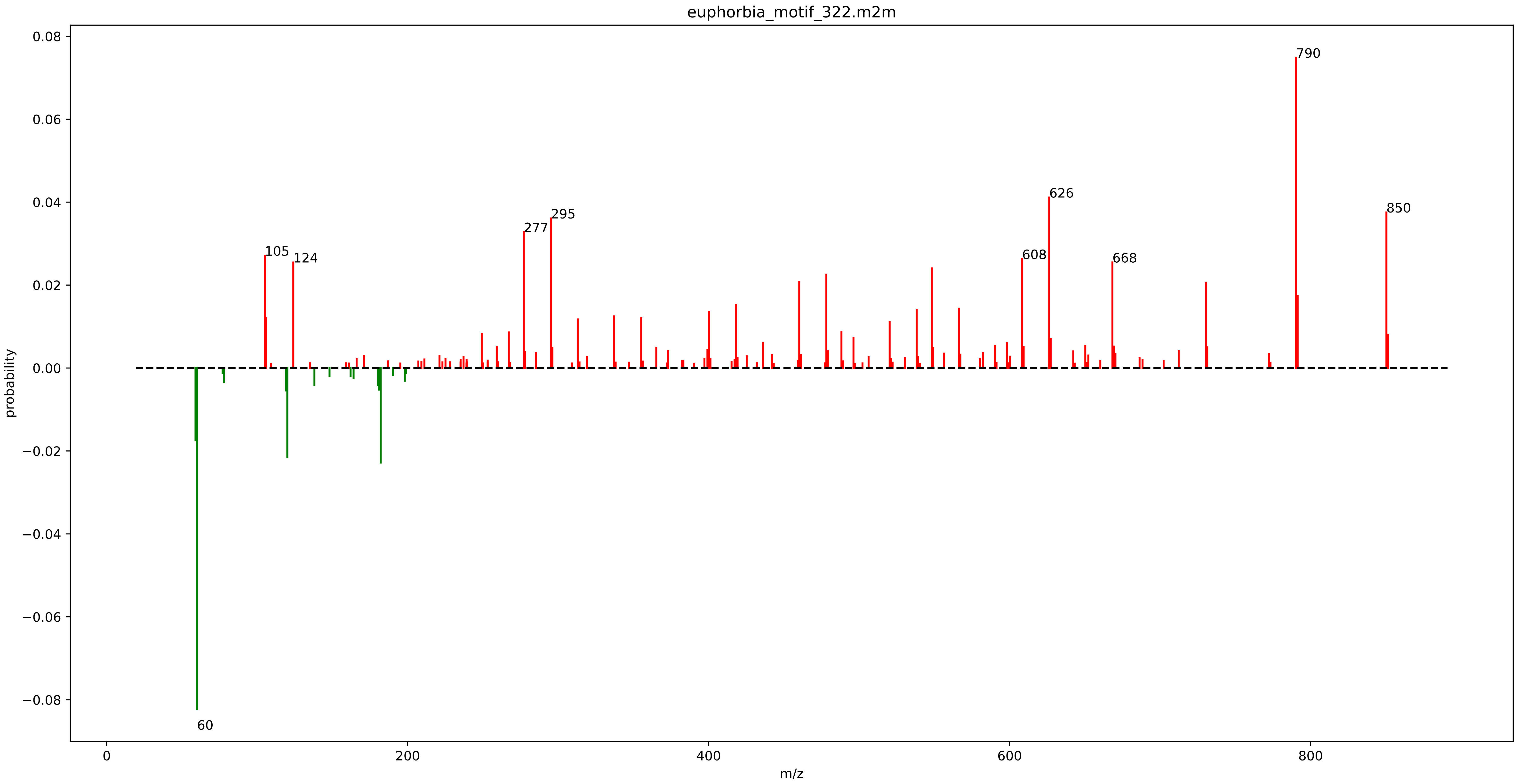

euphorbia_motif_322.m2m
probability
m/z
0.08
0.06
0.04
0.02
0.00
−0.02
−0.04
−0.06
−0.08
0
200
400
600
800
790
626
850
295
277
105
124
608
668
60

euphorbia_motif_322.m2m

loss_60.0175	: 0.08
fragment_790.3475	: 0.07
fragment_626.2975	: 0.04
fragment_850.3675	: 0.04
fragment_295.1675	: 0.04
fragment_277.1575	: 0.03
fragment_105.0325	: 0.03
fragment_608.2875	: 0.03
fragment_668.3075	: 0.03
fragment_124.0375	: 0.03
fragment_548.2625	: 0.02
loss_182.0575	: 0.02
fragment_478.2225	: 0.02
loss_120.0425	: 0.02
fragment_460.2125	: 0.02
fragment_730.3275	: 0.02
loss_59.0175	: 0.02
fragment_791.3475	: 0.02
fragment_418.2025	: 0.02
fragment_566.2725	: 0.01
fragment_538.2425	: 0.01
fragment_400.1875	: 0.01
fragment_337.1775	: 0.01
fragment_355.1875	: 0.01
fragment_106.0275	: 0.01
fragment_313.1775	: 0.01
fragment_520.2325	: 0.01
fragment_488.2425	: 0.01
fragment_267.1725	: 0.01
fragment_249.1625	: 0.01
fragment_851.3725	: 0.01
fragment_496.2325	: 0.01
fragment_627.2975	: 0.01
fragment_436.2125	: 0.01
fragment_598.2625	: 0.01
loss_119.0375	: 0.01
fragment_650.2925	: 0.01
fragment_590.2775	: 0.01
loss_181.0575	: 0.01
fragment_669.3075	: 0.01
fragment_259.1475	: 0.01
fragment_609.2875	: 0.01
fragment_731.3275	: 0.01

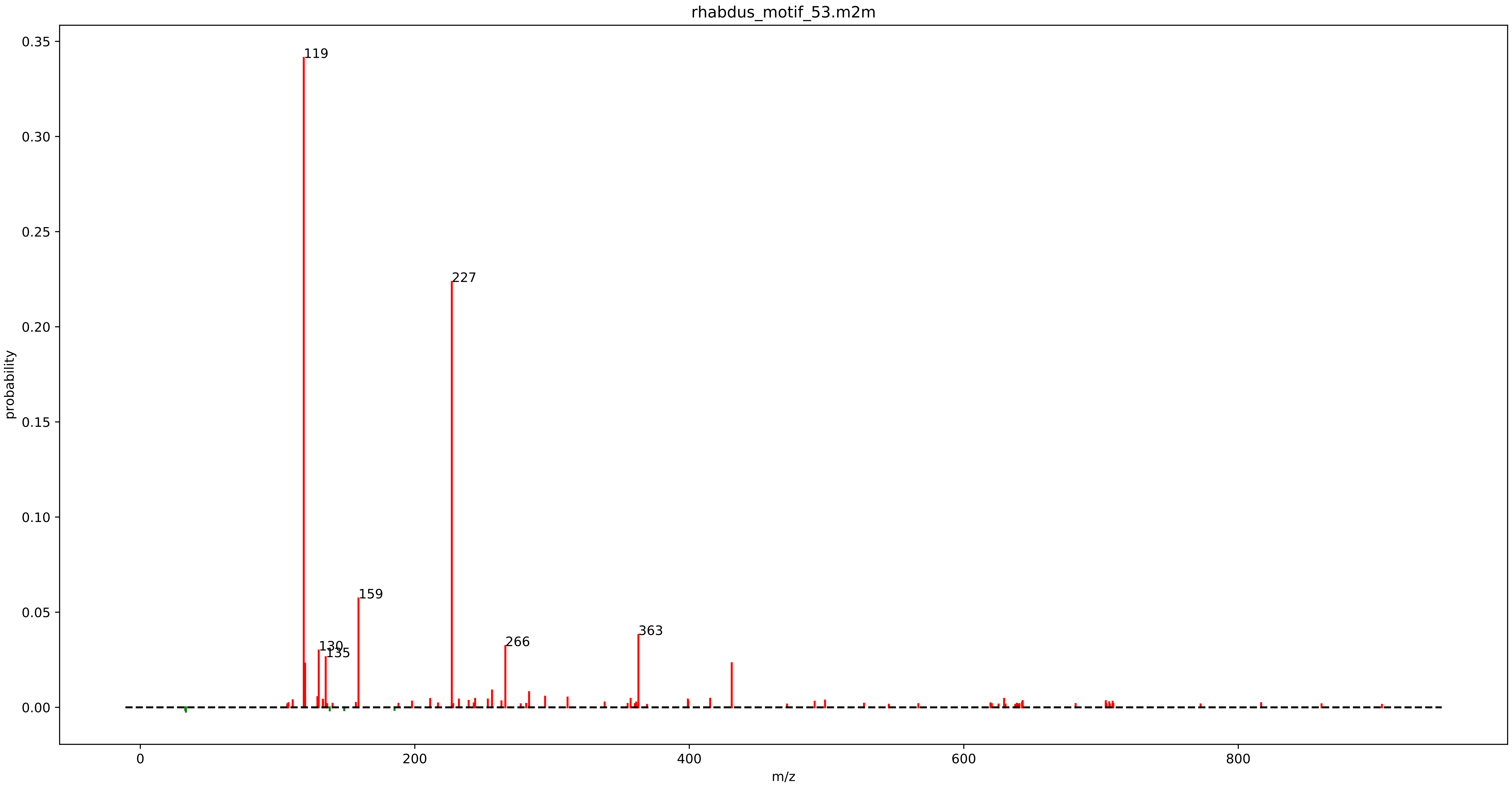

rhabdus_motif_53.m2m
probability
m/z
0.35
0.30
0.25
0.20
0.15
0.10
0.05
0.00
0
200
400
600
800
119
227
159
130
135
266
363

rhabdus_motif_53.m2m

fragment_119.0825	: 0.34
fragment_226.9525	: 0.22
fragment_158.9625	: 0.06
fragment_362.9275	: 0.04
fragment_265.9625	: 0.03
fragment_129.9875	: 0.03
fragment_135.0775	: 0.03
fragment_430.9125	: 0.02
fragment_120.0875	: 0.02
fragment_256.2625	: 0.01
fragment_283.2625	: 0.01
fragment_294.9375	: 0.01
fragment_129.0525	: 0.01
fragment_311.2925	: 0.01

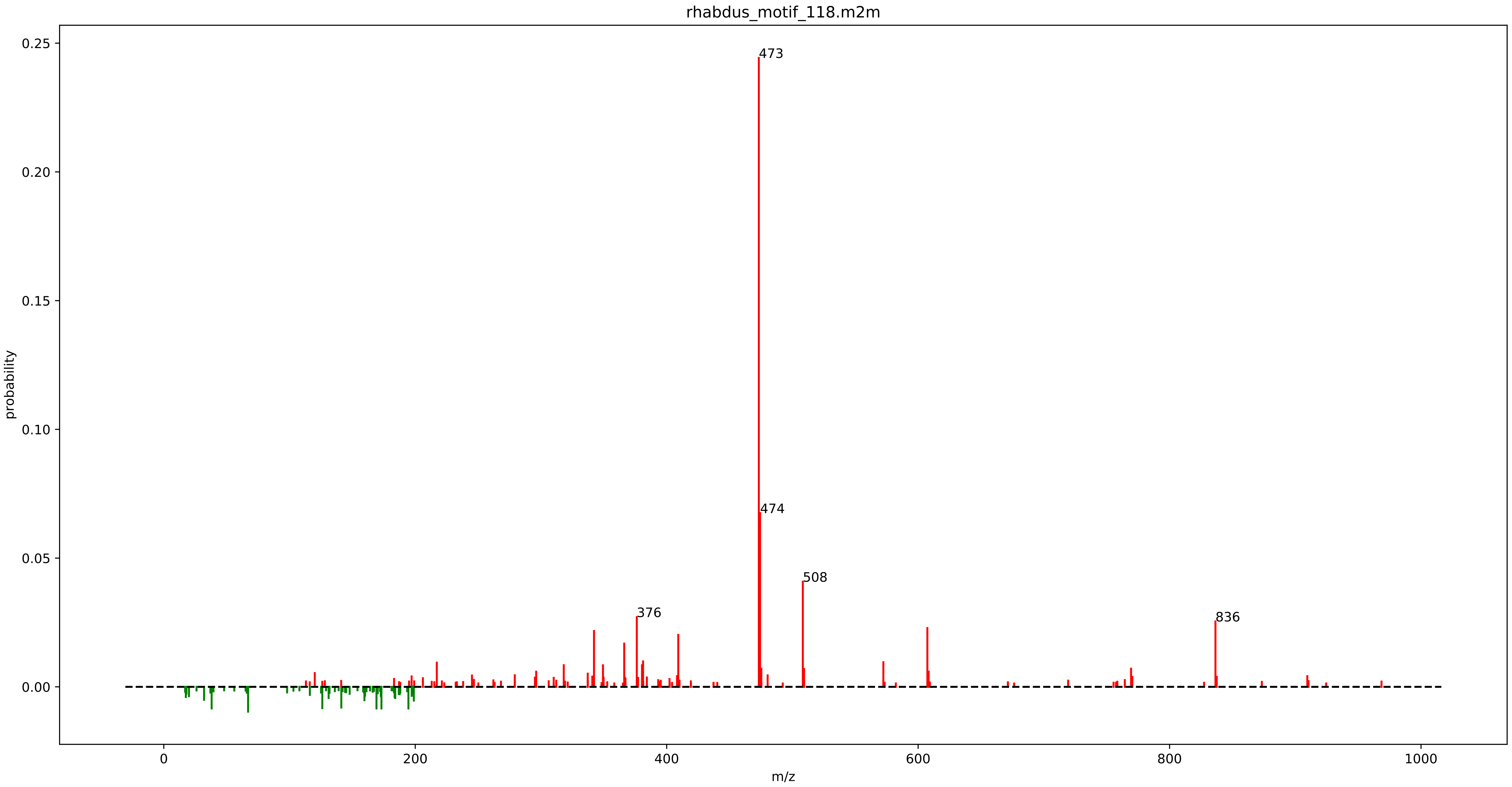

rhabdus_motif_118.m2m
473
474
508
376
836
probability
0.25
0.20
0.15
0.10
0.05
0.00
0
200
400
600
800
1000
m/z

rhabdus_motif_118.m2m

fragment_473.2775	: 0.24
fragment_474.2775	: 0.07
fragment_508.2775	: 0.04
fragment_376.2225	: 0.03
fragment_836.4875	: 0.03
fragment_607.3425	: 0.02
fragment_342.1825	: 0.02
fragment_409.2075	: 0.02
fragment_366.2075	: 0.02
fragment_381.2125	: 0.01
loss_67.0325	: 0.01
fragment_572.3425	: 0.01
fragment_217.1325	: 0.01
loss_173.1125	: 0.01
loss_38.0425	: 0.01
fragment_380.3525	: 0.01
loss_194.5675	: 0.01
loss_169.1825	: 0.01
loss_169.1575	: 0.01
fragment_349.3175	: 0.01
fragment_318.1225	: 0.01
loss_126.0725	: 0.01
loss_141.1925	: 0.01
fragment_769.3675	: 0.01
fragment_475.2825	: 0.01
fragment_509.2775	: 0.01
fragment_608.3475	: 0.01
fragment_296.1975	: 0.01
fragment_120.0775	: 0.01
loss_198.8625	: 0.01
loss_159.5725	: 0.01
loss_67.0725	: 0.01
fragment_337.1825	: 0.01
loss_32.0325	: 0.01

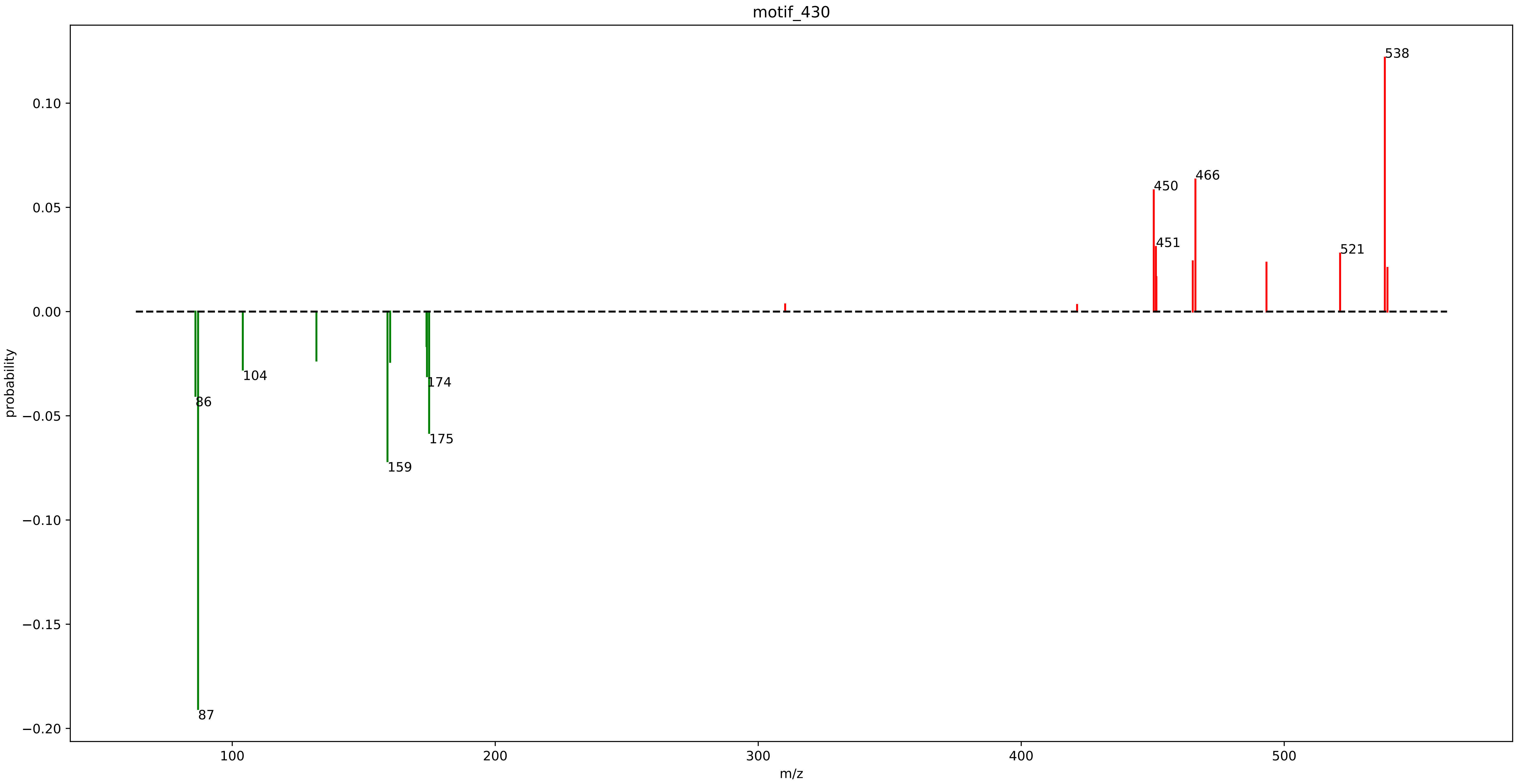

motif_430
probability
m/z
0.10
0.05
0.00
−0.05
−0.10
−0.15
−0.20
100
200
300
400
500
538
466
450
451
521
86
87
104
159
174
175

motif_430

loss_87.0075	: 0.19
fragment_538.2575	: 0.12
loss_159.0275	: 0.07
fragment_466.2325	: 0.06
fragment_450.3925	: 0.06
loss_174.8675	: 0.06
loss_86.0025	: 0.04
fragment_451.2125	: 0.03
loss_174.0475	: 0.03
loss_104.0075	: 0.03
fragment_521.2525	: 0.03
loss_160.0375	: 0.02
fragment_465.2275	: 0.02
loss_132.0025	: 0.02
fragment_493.2575	: 0.02
fragment_539.2625	: 0.02
fragment_451.3975	: 0.02
loss_173.8625	: 0.02

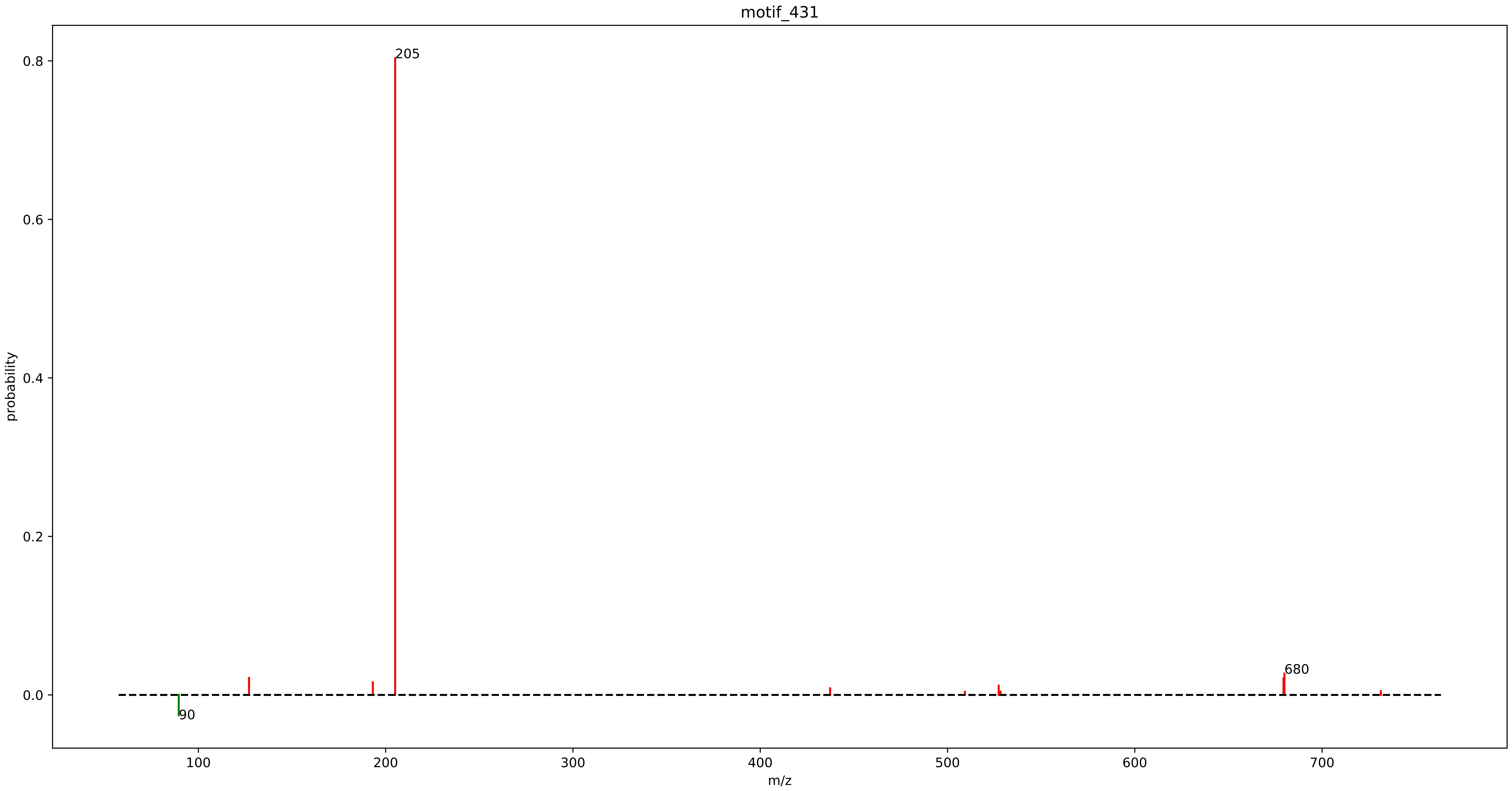

motif_431
205
680
90
probability
0.8
0.6
0.4
0.2
0.0
100
200
300
400
500
600
700
m/z

motif_431

fragment_205.0725	: 0.80
fragment_679.8975	: 0.03
loss_89.5275	: 0.03
fragment_127.0375	: 0.02
fragment_679.4025	: 0.02
fragment_193.1575	: 0.02
fragment_527.3325	: 0.01
fragment_437.3325	: 0.01

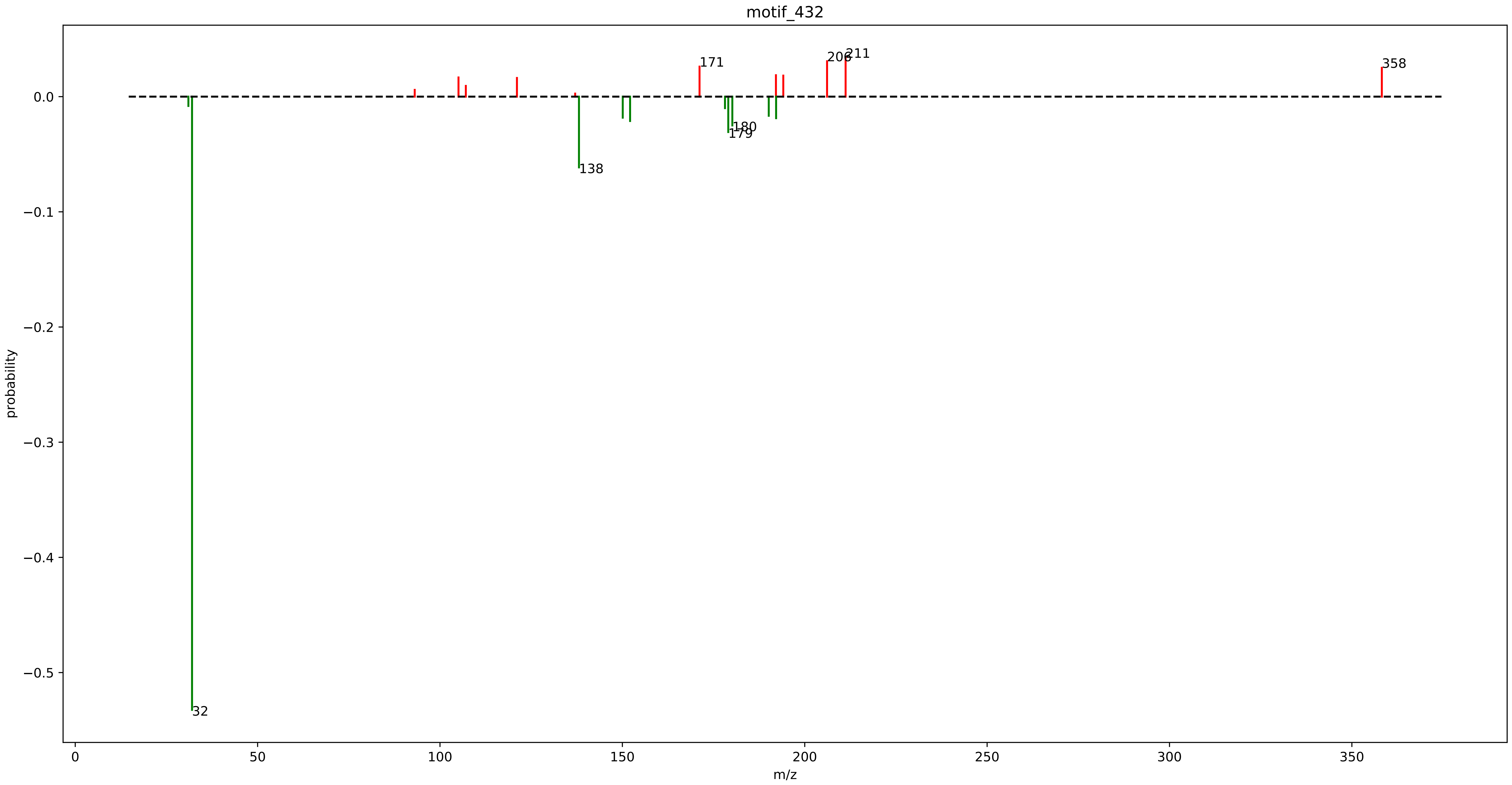

motif_432
probability
m/z
0.0
−0.1
−0.2
−0.3
−0.4
−0.5
0
50
100
150
200
250
300
350
32
138
171
179
180
206
211
358

motif_432

loss_32.0225	: 0.53
loss_138.1025	: 0.06
fragment_211.2025	: 0.03
loss_179.0325	: 0.03
fragment_206.1175	: 0.03
fragment_171.1575	: 0.03
fragment_358.2125	: 0.03
loss_180.1475	: 0.03
loss_152.1175	: 0.02
loss_192.1475	: 0.02
fragment_192.1025	: 0.02
loss_150.1025	: 0.02
fragment_194.1175	: 0.02
fragment_105.0725	: 0.02
loss_190.1325	: 0.02
fragment_121.0975	: 0.02
loss_178.1325	: 0.01
fragment_107.0825	: 0.01
loss_31.0225	: 0.01
fragment_93.0725	: 0.01

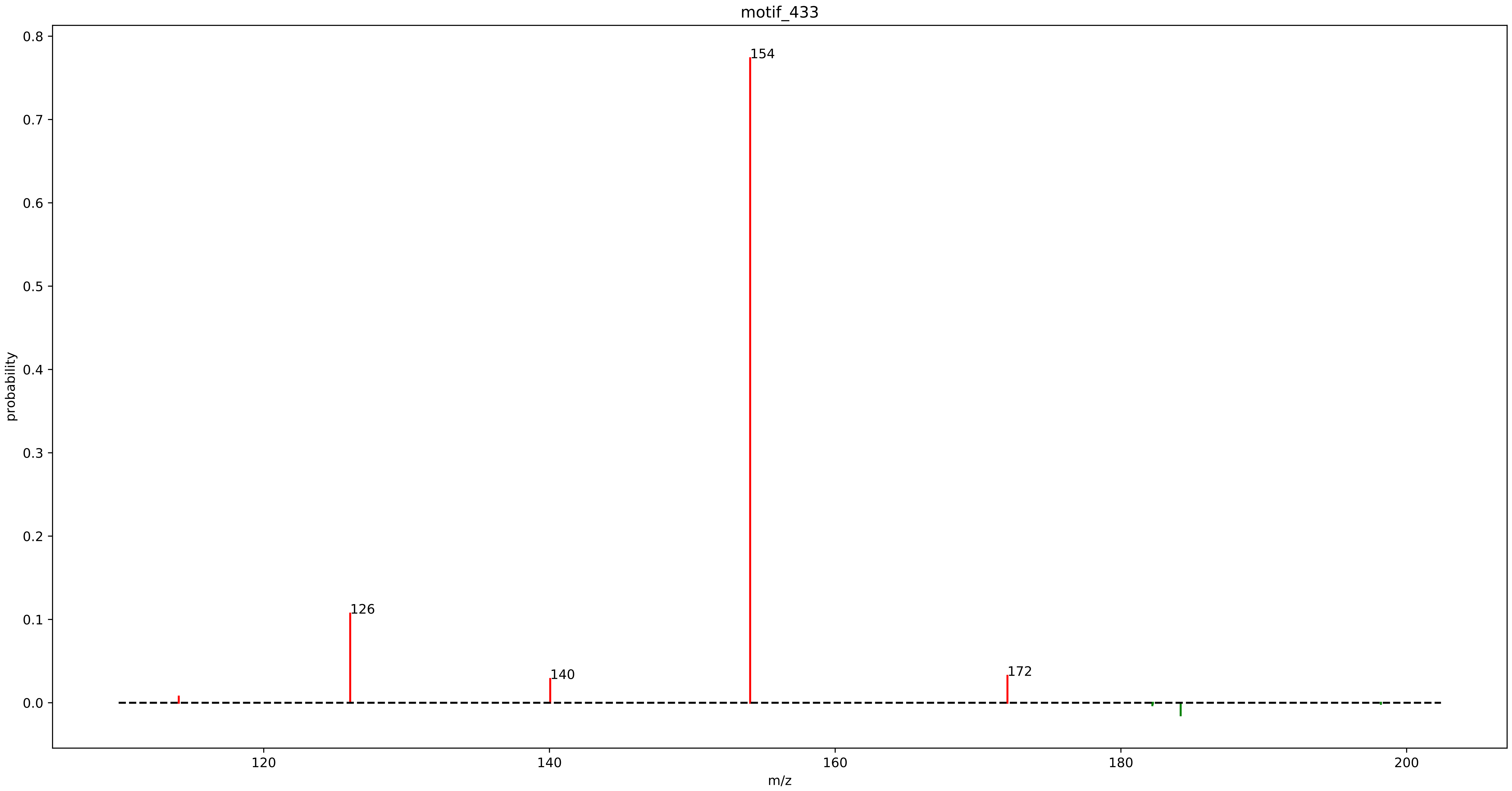

motif_433
probability
m/z
154
126
140
172
0.8
0.7
0.6
0.5
0.4
0.3
0.2
0.1
0.0
120
140
160
180
200

motif_433

fragment_154.0475	: 0.77
fragment_126.0525	: 0.11
fragment_172.0575	: 0.03
fragment_140.0525	: 0.03
loss_184.1825	: 0.02
fragment_114.0525	: 0.01

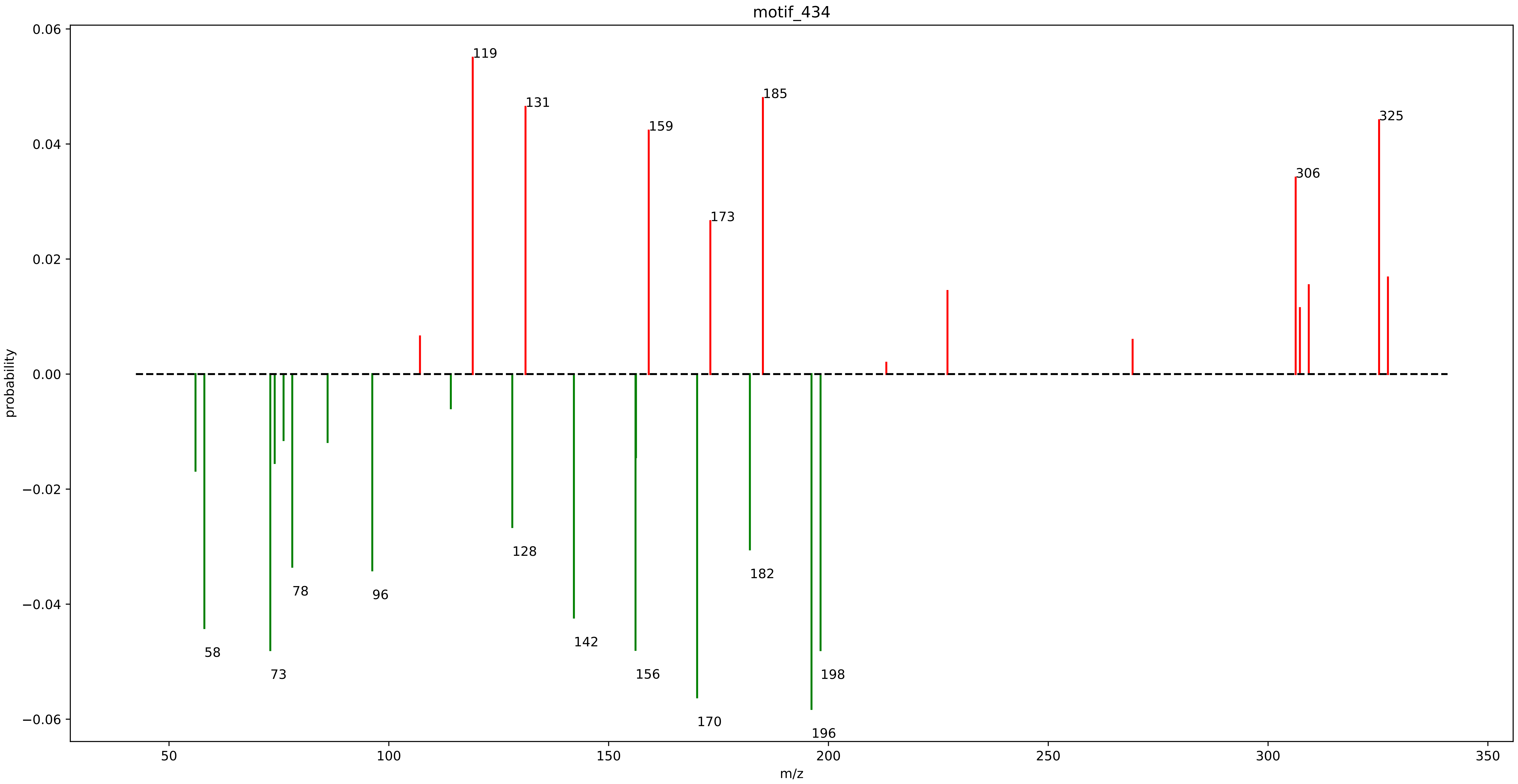
motif_434
probability
m/z
0.06
0.04
0.02
0.00
−0.02
−0.04
−0.06
50
100
150
200
250
300
350
119
131
159
173
185
306
325
58
73
78
96
128
142
156
170
182
196
198

motif_434

loss_196.1425	: 0.06
loss_170.1275	: 0.06
fragment_119.0825	: 0.06
fragment_185.0875	: 0.05
loss_198.2025	: 0.05
loss_73.0375	: 0.05
loss_156.1125	: 0.05
fragment_131.0875	: 0.05
loss_58.0375	: 0.04
fragment_325.2525	: 0.04
loss_142.0975	: 0.04
fragment_159.1175	: 0.04
fragment_306.2775	: 0.03
loss_96.2325	: 0.03
loss_78.0325	: 0.03
loss_182.1275	: 0.03
fragment_173.1325	: 0.03
loss_128.0825	: 0.03
loss_56.0225	: 0.02
fragment_327.2675	: 0.02
loss_74.0375	: 0.02
fragment_309.2575	: 0.02
fragment_227.0725	: 0.01
loss_156.2175	: 0.01
loss_86.0725	: 0.01
fragment_307.2425	: 0.01
loss_76.0525	: 0.01
fragment_107.0825	: 0.01
fragment_269.1875	: 0.01
loss_114.1025	: 0.01

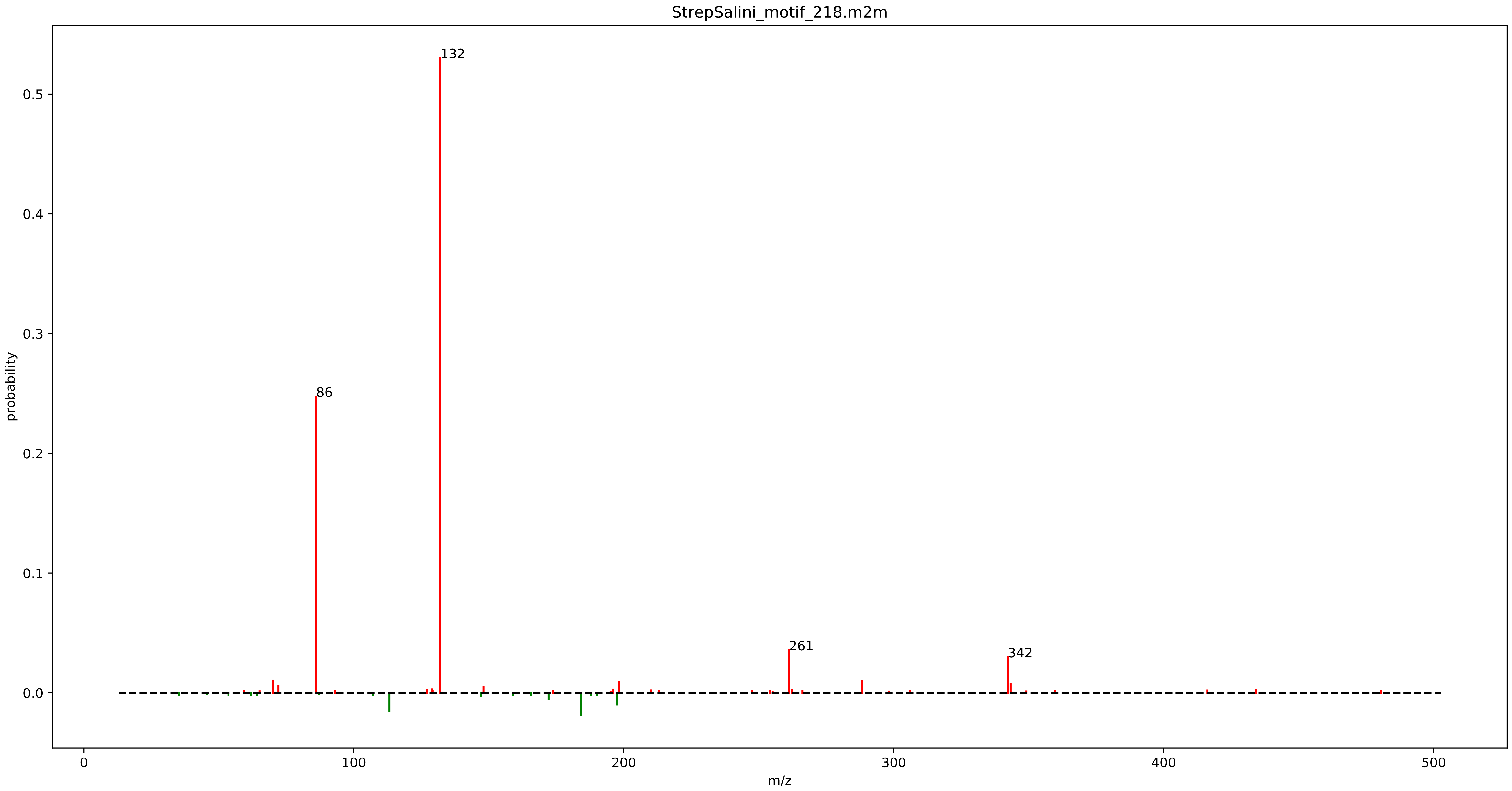

StrepSalini_motif_218.m2m
probability
m/z
0.5
0.4
0.3
0.2
0.1
0.0
0
100
200
300
400
500
132
86
261
342

StrepSalini_motif_218.m2m

fragment_132.0500	: 0.53
fragment_86.0500	: 0.25
fragment_261.1500	: 0.04
fragment_342.2525	: 0.03
loss_184.0500	: 0.02
loss_113.1500	: 0.02
fragment_70.0500	: 0.01
fragment_288.1500	: 0.01
loss_197.5500	: 0.01
fragment_198.1500	: 0.01
fragment_343.2500	: 0.01
fragment_72.0500	: 0.01
loss_172.1525	: 0.01

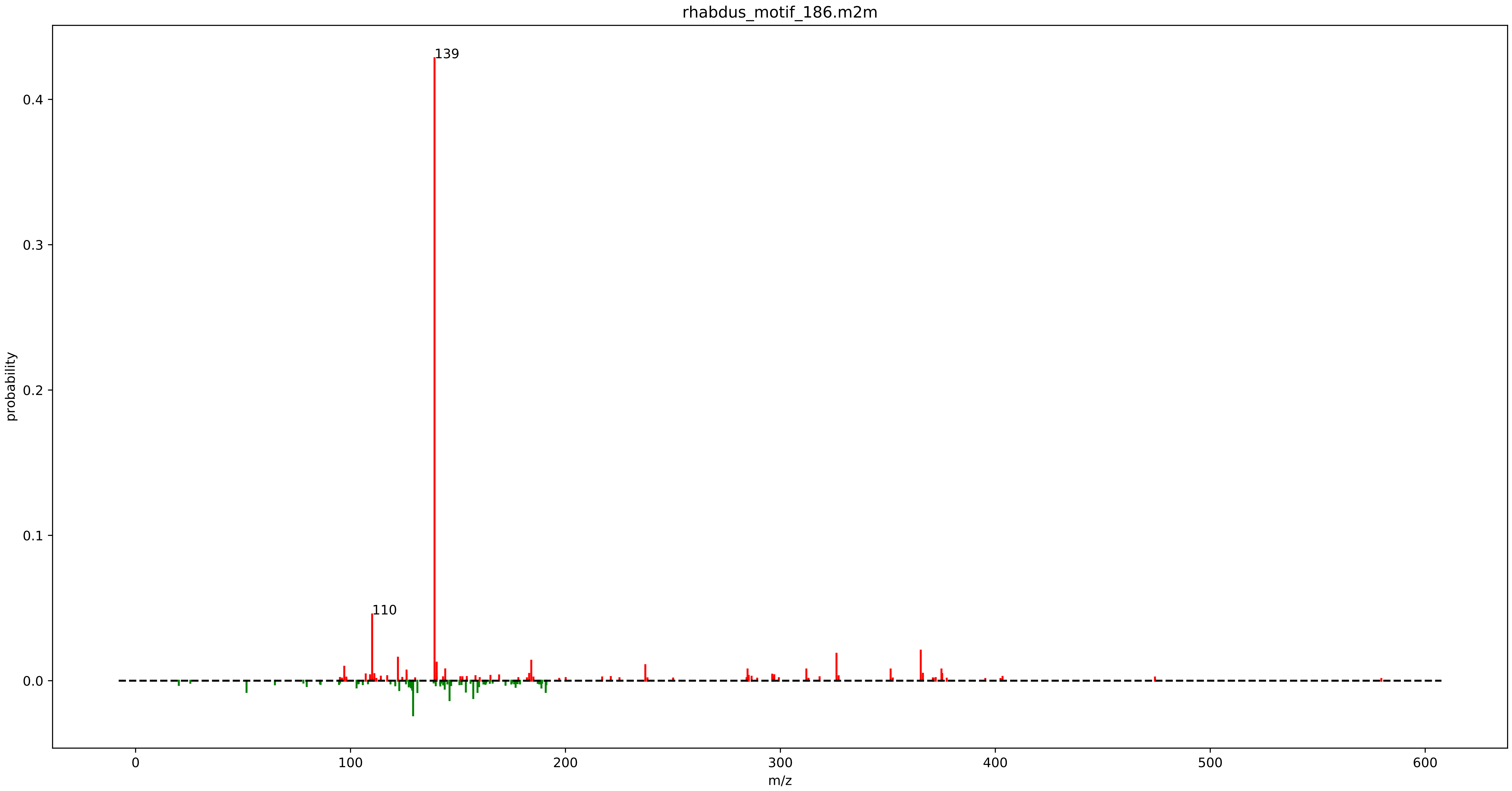

rhabdus_motif_186.m2m
139
110
0.4
0.3
0.2
0.1
0.0
probability
0
100
200
300
400
500
600
m/z

rhabdus_motif_186.m2m

fragment_139.0875	: 0.43
fragment_110.0575	: 0.05
loss_129.1175	: 0.02
fragment_365.3025	: 0.02
fragment_326.0975	: 0.02
fragment_122.0575	: 0.02
fragment_184.0975	: 0.01
loss_146.0725	: 0.01
fragment_140.0875	: 0.01
loss_157.1075	: 0.01
fragment_237.1575	: 0.01
fragment_97.0875	: 0.01
loss_129.1125	: 0.01
loss_131.1125	: 0.01
fragment_144.0625	: 0.01
loss_159.0775	: 0.01
loss_190.8225	: 0.01
fragment_284.7175	: 0.01
fragment_312.0775	: 0.01
fragment_351.2875	: 0.01
loss_51.6175	: 0.01
fragment_374.9375	: 0.01
loss_153.6625	: 0.01
fragment_126.0625	: 0.01
loss_122.6825	: 0.01
loss_128.6675	: 0.01
loss_143.8225	: 0.01

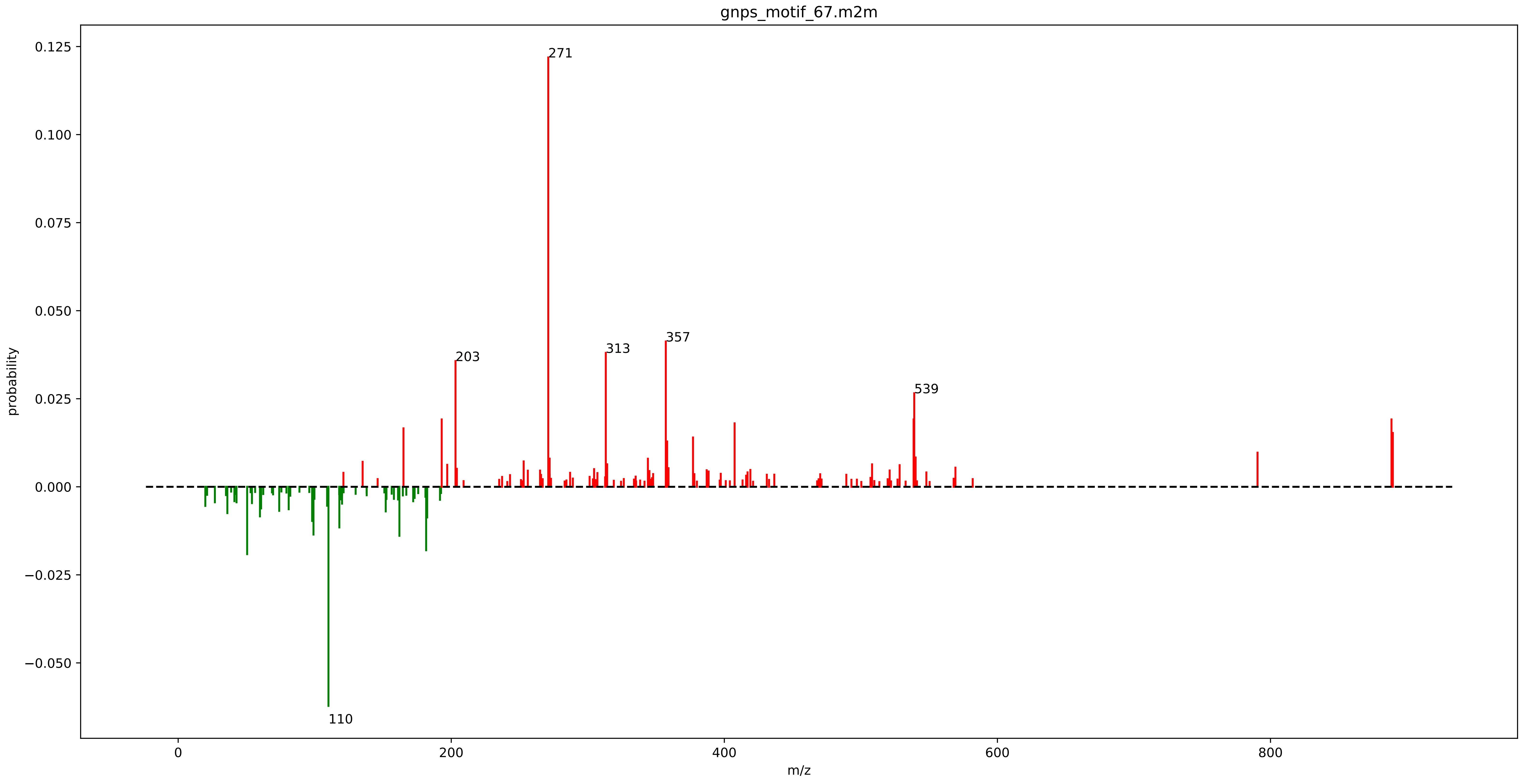

gnps_motif_67.m2m
probability
m/z
0.125
0.100
0.075
0.050
0.025
0.000
−0.025
−0.050
0
200
400
600
800
271
203
313
357
539
110

gnps_motif_67.m2m

fragment_271.0575	: 0.12
loss_110.0725	: 0.06
fragment_357.1325	: 0.04
fragment_313.1925	: 0.04
fragment_203.1175	: 0.04
fragment_539.0975	: 0.03
fragment_538.6225	: 0.02
loss_50.5175	: 0.02
fragment_888.6125	: 0.02
fragment_193.0075	: 0.02
loss_181.6175	: 0.02
fragment_407.5275	: 0.02
fragment_165.0175	: 0.02
fragment_889.6175	: 0.02
fragment_377.0675	: 0.01
loss_162.0325	: 0.01
loss_99.1025	: 0.01
fragment_358.1325	: 0.01
loss_118.0425	: 0.01
fragment_790.5125	: 0.01
loss_98.1025	: 0.01
loss_182.3925	: 0.01
loss_59.8875	: 0.01
fragment_540.0975	: 0.01
fragment_272.0675	: 0.01
fragment_344.0225	: 0.01
loss_36.0225	: 0.01
fragment_253.0475	: 0.01
fragment_135.1025	: 0.01
loss_152.0125	: 0.01
loss_74.0075	: 0.01
fragment_314.1925	: 0.01
fragment_508.2025	: 0.01
loss_80.9425	: 0.01
fragment_197.0525	: 0.01
loss_60.7525	: 0.01
fragment_528.3875	: 0.01
fragment_569.2575	: 0.01
loss_19.8875	: 0.01
loss_109.0725	: 0.01
fragment_359.1225	: 0.01
fragment_204.1225	: 0.01
fragment_304.6275	: 0.01

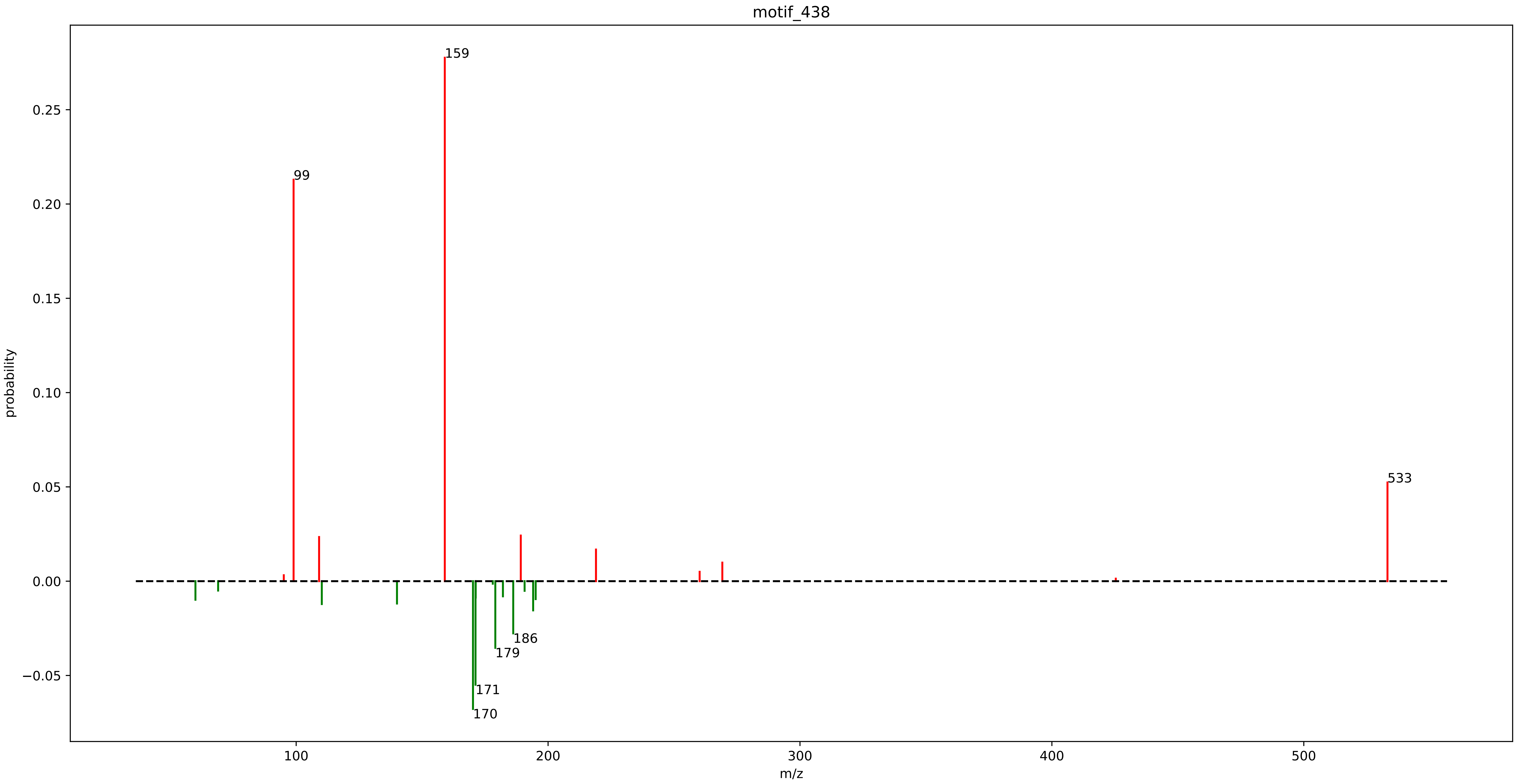

motif_438
probability
m/z
0.25
0.20
0.15
0.10
0.05
0.00
−0.05
100
200
300
400
500
159
99
533
186
179
171
170

motif_438

fragment_158.9975	: 0.28
fragment_98.9725	: 0.21
loss_170.1925	: 0.07
loss_171.1975	: 0.05
fragment_533.2175	: 0.05
loss_179.0575	: 0.04
loss_186.1675	: 0.03
fragment_189.1625	: 0.02
fragment_109.0975	: 0.02
fragment_219.0175	: 0.02
loss_194.0725	: 0.02
loss_110.1725	: 0.01
loss_140.0225	: 0.01
loss_60.0125	: 0.01
fragment_269.1725	: 0.01
loss_195.0775	: 0.01
loss_171.2675	: 0.01
loss_182.0775	: 0.01
loss_190.6825	: 0.01

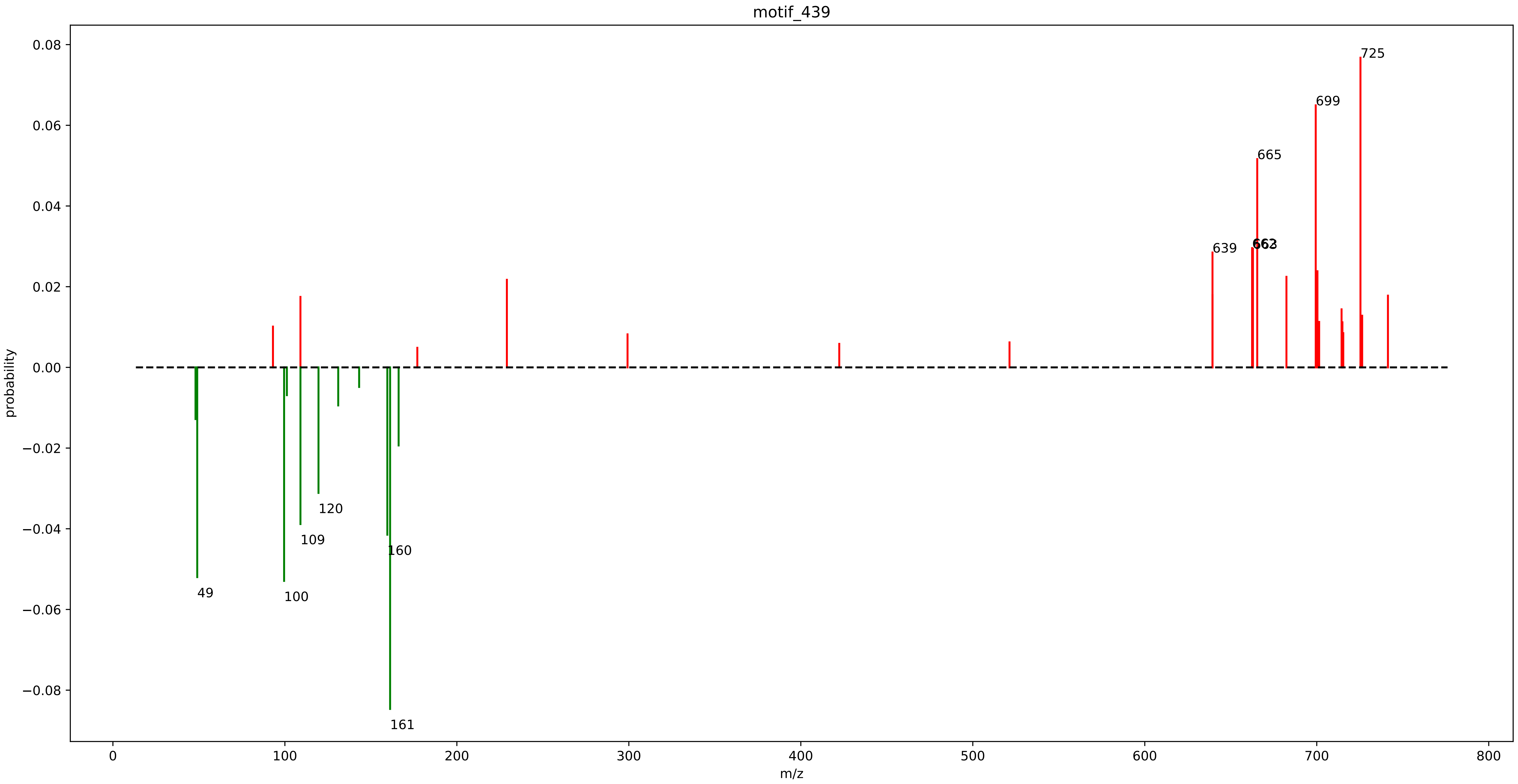
motif_439
probability
m/z
0.08
0.06
0.04
0.02
0.00
−0.02
−0.04
−0.06
−0.08
0
100
200
300
400
500
600
700
800
725
699
665
639
49
100
109
120
160
161

motif_439

loss_161.1825	: 0.08
fragment_725.4125	: 0.08
fragment_699.3975	: 0.06
loss_99.5425	: 0.05
loss_49.0325	: 0.05
fragment_665.3925	: 0.05
loss_159.5625	: 0.04
loss_109.0525	: 0.04
loss_119.5375	: 0.03
fragment_662.3975	: 0.03
fragment_662.8975	: 0.03
fragment_639.3725	: 0.03
fragment_700.4025	: 0.02
fragment_682.3725	: 0.02
fragment_229.0825	: 0.02
loss_166.1325	: 0.02
fragment_639.3775	: 0.02
fragment_741.3925	: 0.02
fragment_109.0425	: 0.02
fragment_714.4275	: 0.01
loss_48.0275	: 0.01
fragment_726.4125	: 0.01
fragment_701.4025	: 0.01
fragment_714.9275	: 0.01
fragment_93.0675	: 0.01
loss_131.0175	: 0.01
fragment_715.4275	: 0.01
fragment_299.2375	: 0.01
loss_101.1625	: 0.01
fragment_521.3475	: 0.01
fragment_422.3525	: 0.01

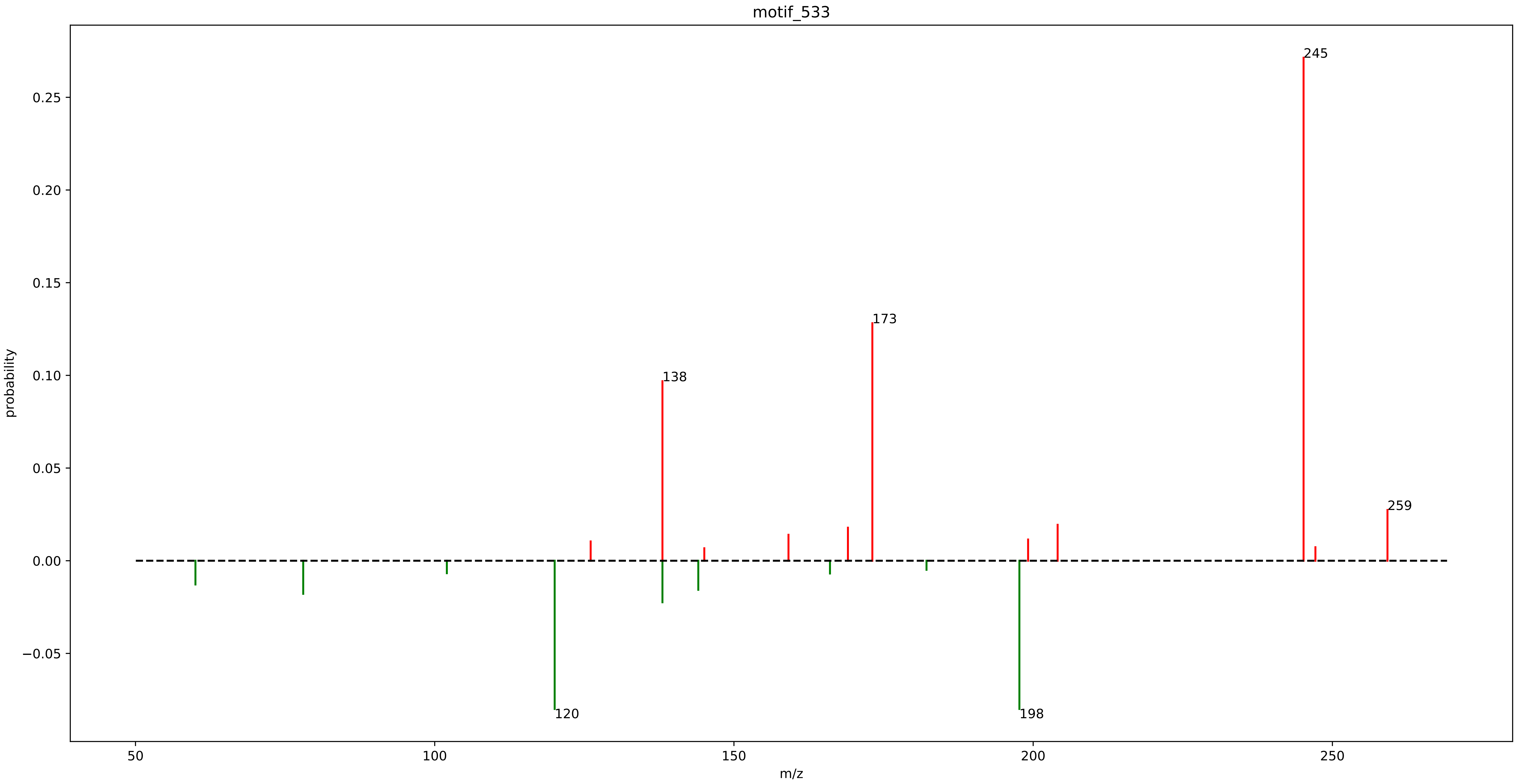

motif_533
probability
m/z
0.25
0.20
0.15
0.10
0.05
0.00
−0.05
50
100
150
200
250
245
173
138
259
120
198

motif_533

fragment_245.1825	: 0.27
fragment_173.1275	: 0.13
fragment_138.0525	: 0.10
loss_197.6975	: 0.08
loss_120.0425	: 0.08
fragment_259.2025	: 0.03
loss_138.0525	: 0.02
fragment_204.0875	: 0.02
loss_78.0275	: 0.02
fragment_169.0475	: 0.02
loss_144.0425	: 0.02
fragment_159.1125	: 0.01
loss_60.0175	: 0.01
fragment_199.1525	: 0.01
fragment_126.0575	: 0.01
fragment_247.1625	: 0.01
loss_166.0475	: 0.01
loss_102.0275	: 0.01
fragment_145.0475	: 0.01

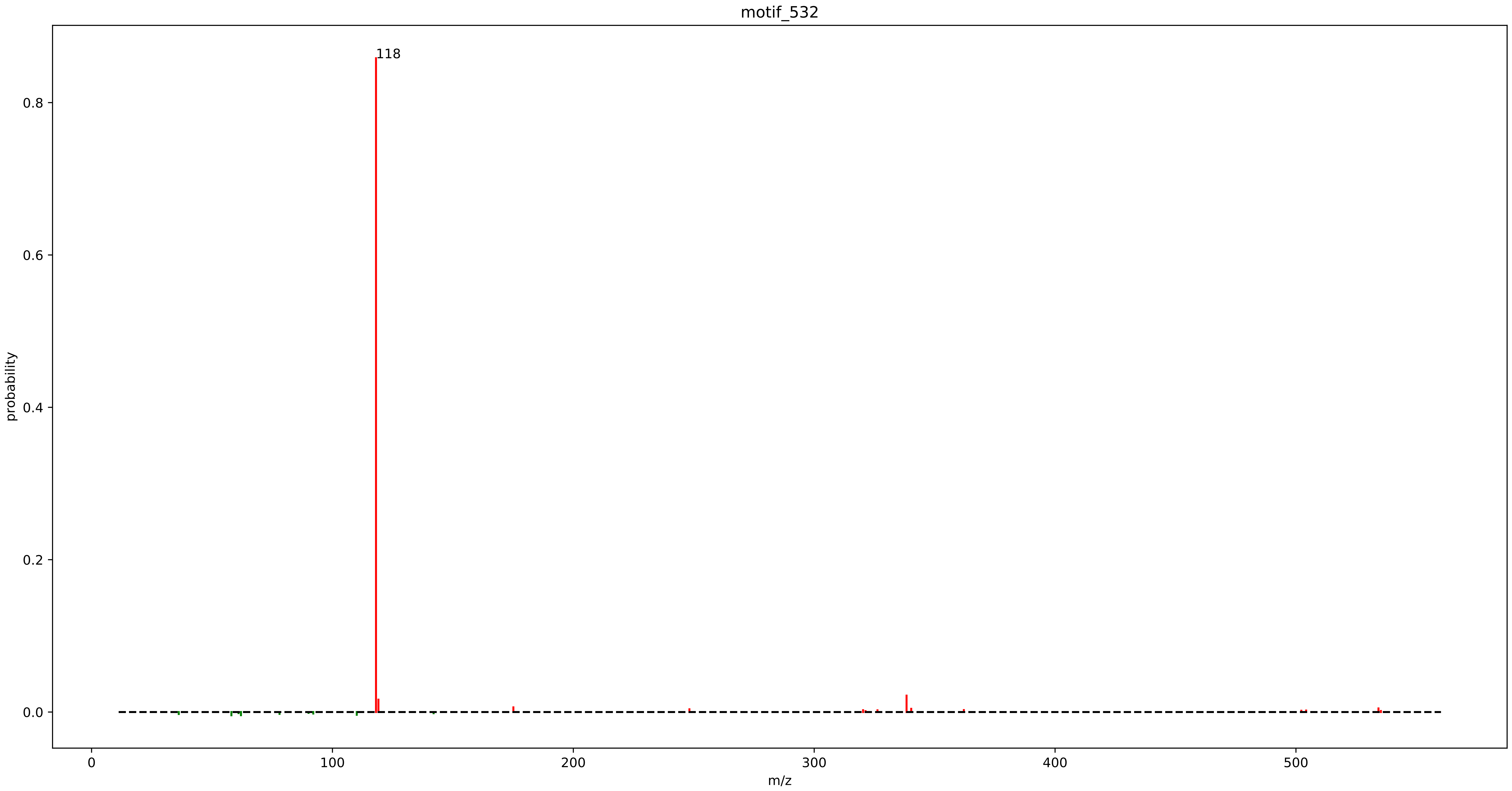
motif_532
118
probability
0.0
0.2
0.4
0.6
0.8
m/z
0
100
200
300
400
500

motif_532

fragment_118.0825	: 0.86
fragment_338.3425	: 0.02
fragment_119.0875	: 0.02
fragment_175.1125	: 0.01

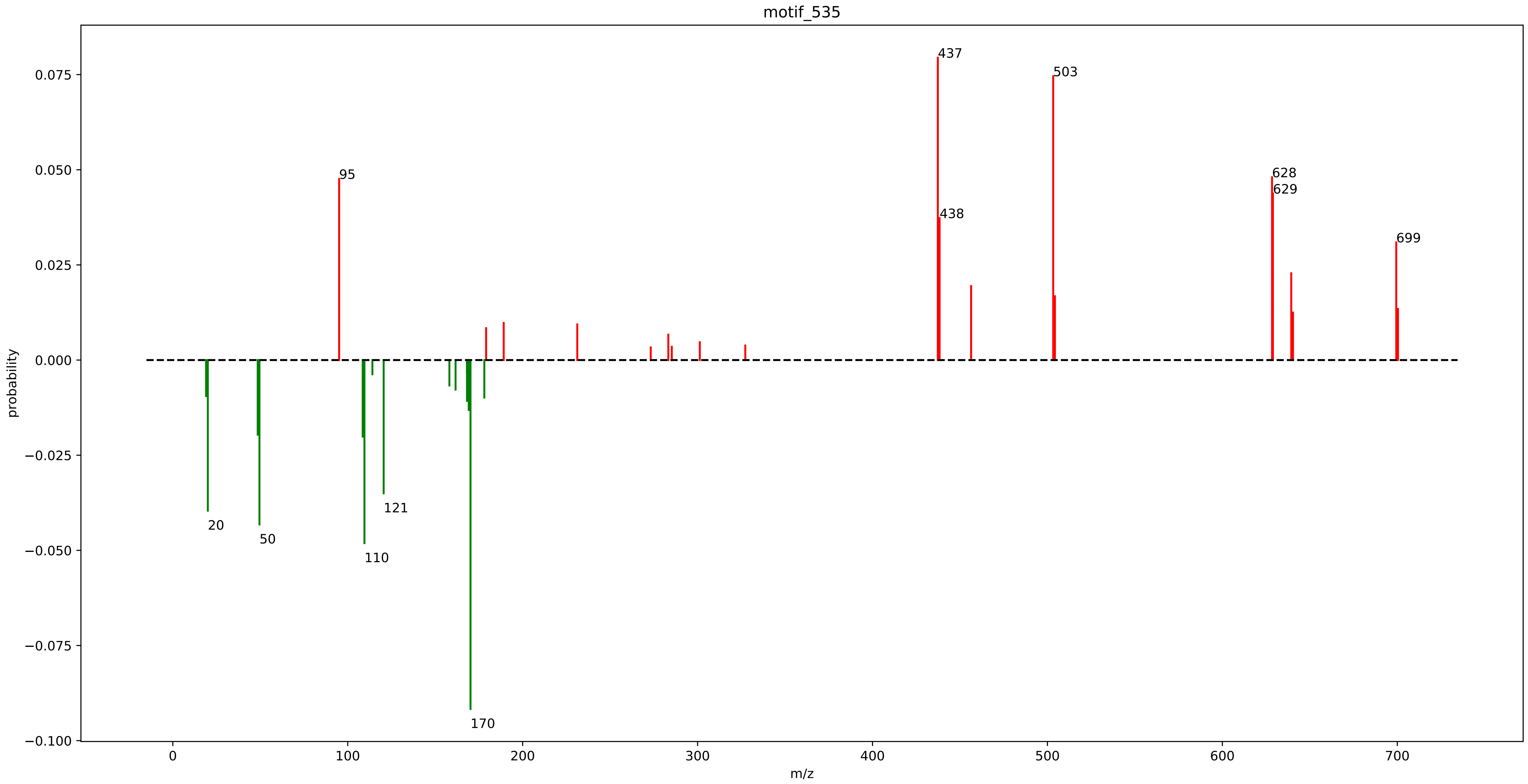

motif_535
probability
m/z
0.075
0.050
0.025
0.000
−0.025
−0.050
−0.075
−0.100
0
100
200
300
400
500
600
700
437
503
95
628
629
438
699
20
50
110
121
170

motif_535

loss_170.1825	: 0.09
fragment_437.3375	: 0.08
fragment_503.2875	: 0.07
loss_109.5525	: 0.05
fragment_628.3925	: 0.05
fragment_95.0825	: 0.05
fragment_628.8925	: 0.04
loss_49.5325	: 0.04
loss_20.0225	: 0.04
fragment_438.3425	: 0.04
loss_120.5375	: 0.04
fragment_699.3975	: 0.03
fragment_639.3775	: 0.02
loss_108.5525	: 0.02
loss_48.5325	: 0.02
fragment_456.3575	: 0.02
fragment_504.2875	: 0.02
fragment_700.3975	: 0.01
loss_169.1775	: 0.01
fragment_640.3775	: 0.01
loss_168.1825	: 0.01
loss_178.0825	: 0.01
fragment_189.1625	: 0.01
loss_19.0225	: 0.01
fragment_231.2125	: 0.01
fragment_179.1075	: 0.01
loss_161.6475	: 0.01
fragment_283.2575	: 0.01
loss_158.1125	: 0.01

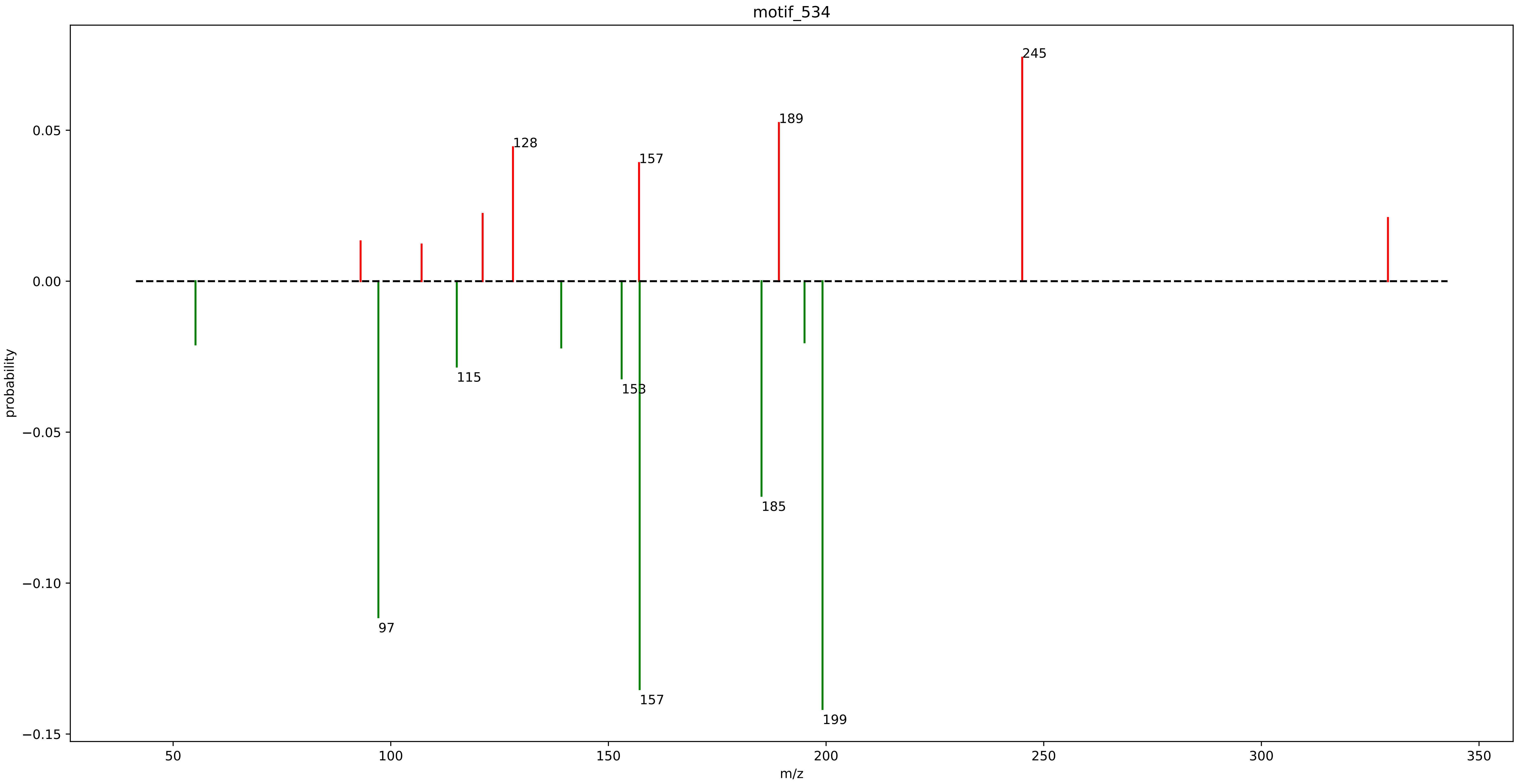

motif_534
probability
m/z
0.05
0.00
−0.05
−0.10
−0.15
50
100
150
200
250
300
350
245
189
128
157
115
153
97
157
185
199

motif_534

loss_199.1825	: 0.14
loss_157.1725	: 0.14
loss_97.1525	: 0.11
fragment_245.0525	: 0.07
loss_185.1675	: 0.07
fragment_189.1625	: 0.05
fragment_128.0725	: 0.04
fragment_157.0325	: 0.04
loss_153.0375	: 0.03
loss_115.1625	: 0.03
fragment_121.1025	: 0.02
loss_139.1625	: 0.02
loss_55.1425	: 0.02
fragment_329.0725	: 0.02
loss_195.0475	: 0.02
fragment_93.0675	: 0.01
fragment_107.0825	: 0.01

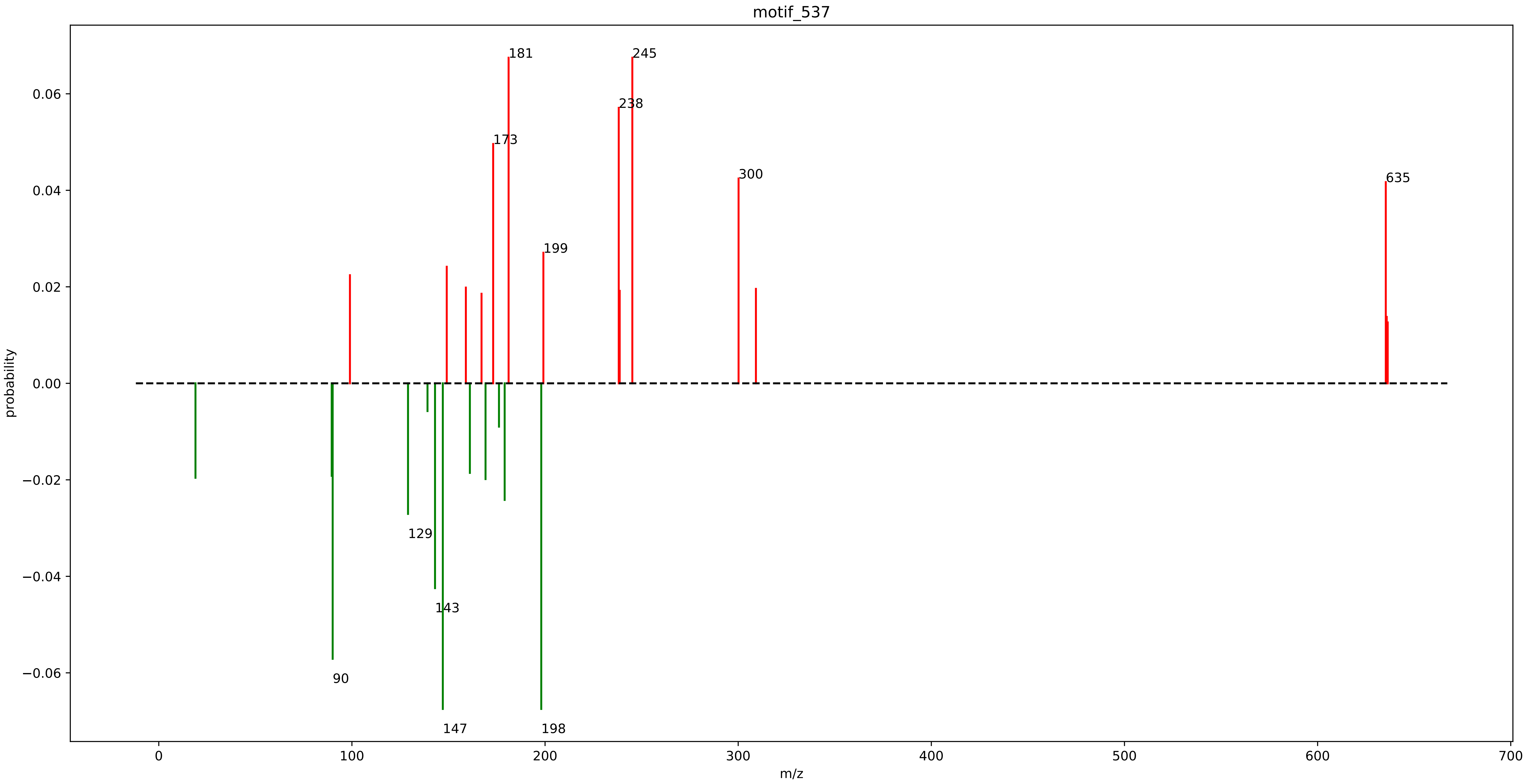

motif_537
probability
m/z
0.06
0.04
0.02
0.00
−0.02
−0.04
−0.06
0
100
200
300
400
500
600
700
181
245
238
173
300
635
199
129
143
90
147
198

motif_537

loss_198.0325	: 0.07
loss_147.0575	: 0.07
fragment_181.1225	: 0.07
fragment_245.1875	: 0.07
fragment_238.1475	: 0.06
loss_90.0325	: 0.06
fragment_173.1275	: 0.05
loss_143.0275	: 0.04
fragment_300.1925	: 0.04
fragment_635.2975	: 0.04
fragment_199.1325	: 0.03
loss_129.0475	: 0.03
loss_179.0825	: 0.02
fragment_149.0975	: 0.02
fragment_98.9725	: 0.02
fragment_158.9975	: 0.02
loss_169.1825	: 0.02
loss_18.9975	: 0.02
fragment_309.1775	: 0.02
fragment_238.6475	: 0.02
loss_89.5275	: 0.02
fragment_167.1075	: 0.02
loss_161.0725	: 0.02
fragment_635.7975	: 0.01
fragment_636.3025	: 0.01
loss_176.1425	: 0.01
loss_139.1325	: 0.01

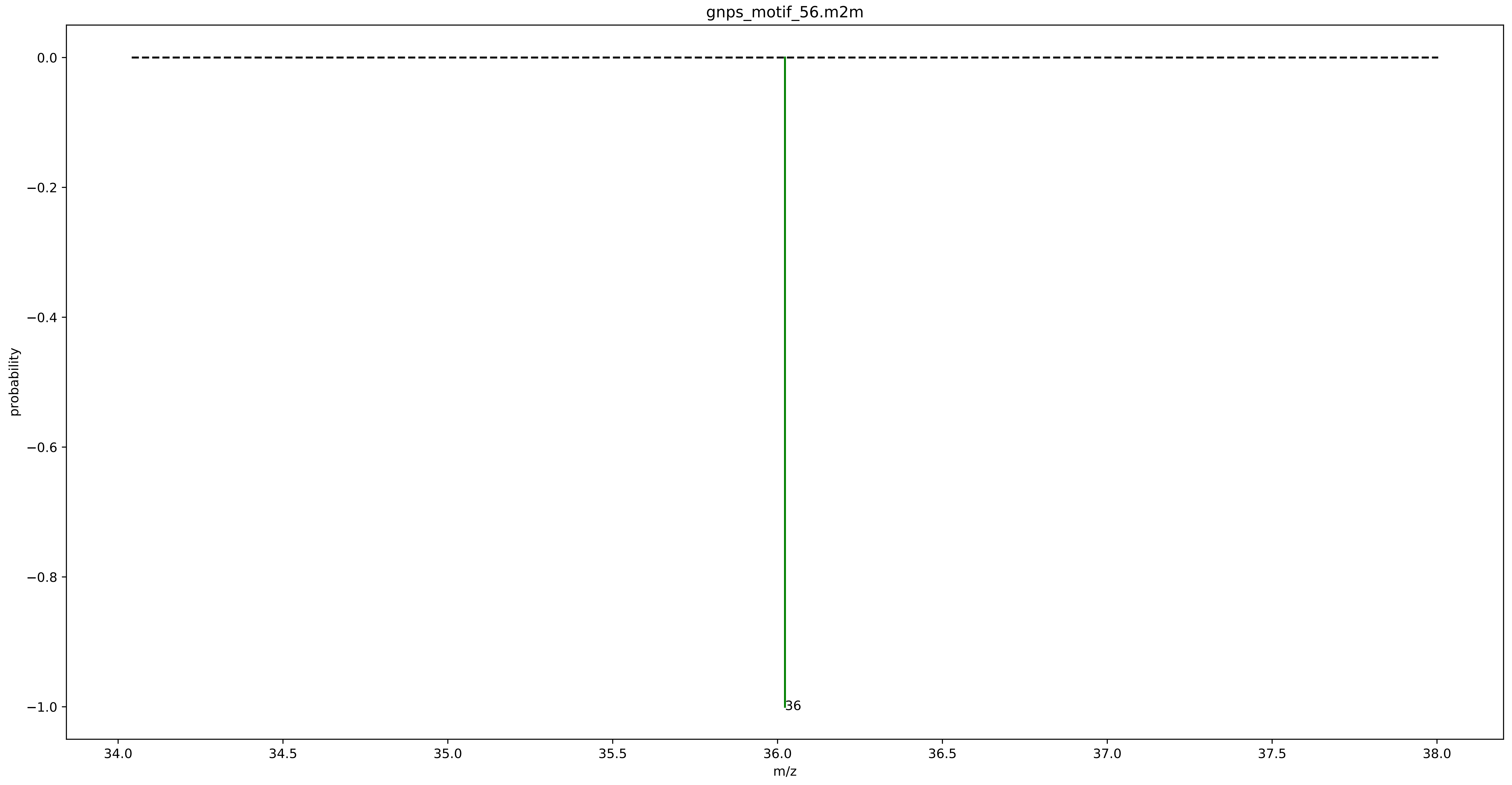
gnps_motif_56.m2m
0.0
−0.2
−0.4
−0.6
−0.8
−1.0
probability
36
34.0
34.5
35.0
35.5
36.0
36.5
37.0
37.5
38.0
m/z

gnps_motif_56.m2m

loss_36.0225 : 1.00

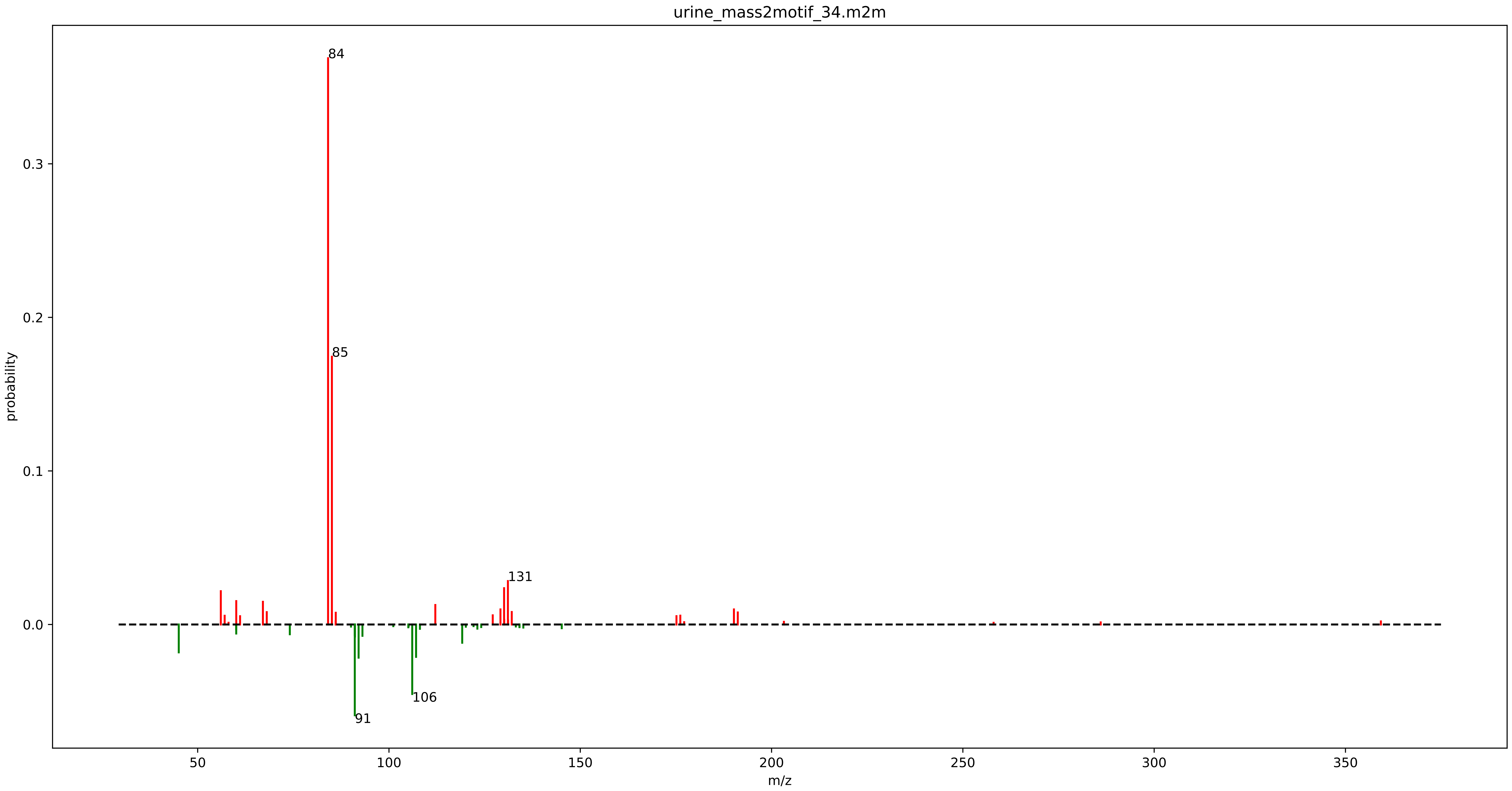

urine_mass2motif_34.m2m
probability
m/z
0.3
0.2
0.1
0.0
50
100
150
200
250
300
350
84
85
131
106
91

urine_mass2motif_34.m2m

fragment_84.0825	: 0.37
fragment_85.0825	: 0.17
loss_91.0625	: 0.06
loss_106.0775	: 0.05
fragment_131.0875	: 0.03
fragment_130.0875	: 0.02
fragment_56.0475	: 0.02
loss_92.0625	: 0.02
loss_107.0825	: 0.02
loss_45.0575	: 0.02
fragment_60.0825	: 0.02
fragment_67.0525	: 0.01
fragment_112.1125	: 0.01
loss_119.1425	: 0.01
fragment_129.1375	: 0.01
fragment_190.1625	: 0.01
fragment_132.0925	: 0.01
fragment_68.0575	: 0.01
fragment_191.1675	: 0.01
loss_91.1075	: 0.01
fragment_86.0875	: 0.01
loss_93.0675	: 0.01
loss_91.0575	: 0.01
fragment_85.0775	: 0.01
loss_74.0825	: 0.01
fragment_127.1225	: 0.01
loss_60.0775	: 0.01
fragment_176.1475	: 0.01
fragment_57.0525	: 0.01
fragment_175.1425	: 0.01
fragment_61.0825	: 0.01

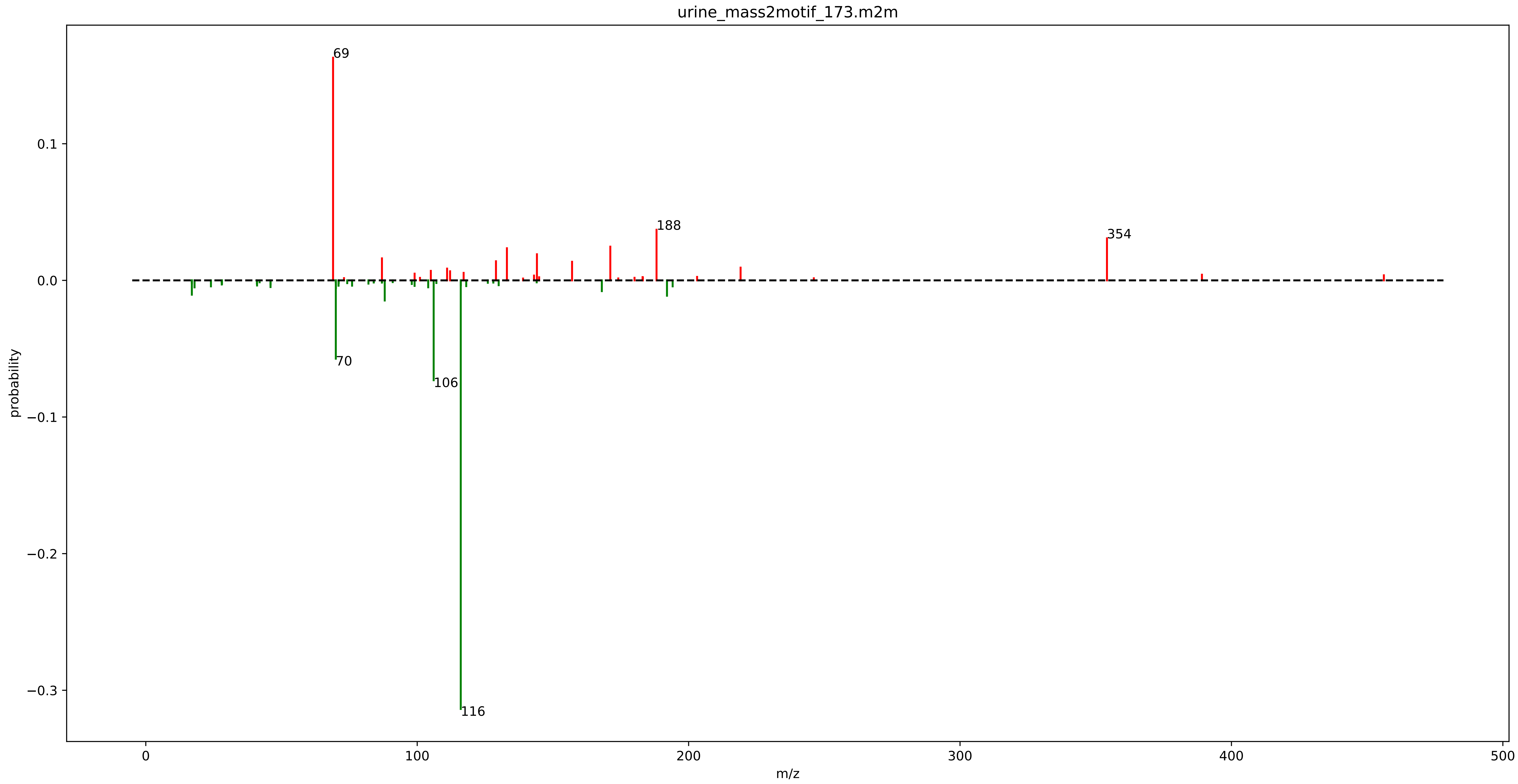

urine_mass2motif_173.m2m
69
188
354
70
106
116
0.1
0.0
−0.1
−0.2
−0.3
probability
0
100
200
300
400
500
m/z

urine_mass2motif_173.m2m

loss_116.0425	: 0.31
fragment_68.9975	: 0.16
loss_106.0625	: 0.07
loss_70.0075	: 0.06
fragment_188.1775	: 0.04
fragment_354.1375	: 0.03
fragment_171.1475	: 0.02
fragment_133.0525	: 0.02
fragment_144.1125	: 0.02
fragment_87.0075	: 0.02
loss_88.0325	: 0.01
fragment_129.0175	: 0.01
fragment_157.0525	: 0.01
loss_192.0275	: 0.01
loss_16.9675	: 0.01
fragment_219.1475	: 0.01
fragment_111.0075	: 0.01
loss_168.0225	: 0.01
fragment_105.0175	: 0.01
fragment_112.1125	: 0.01
loss_70.0475	: 0.01
fragment_117.1025	: 0.01
loss_17.9625	: 0.01
loss_104.0525	: 0.01

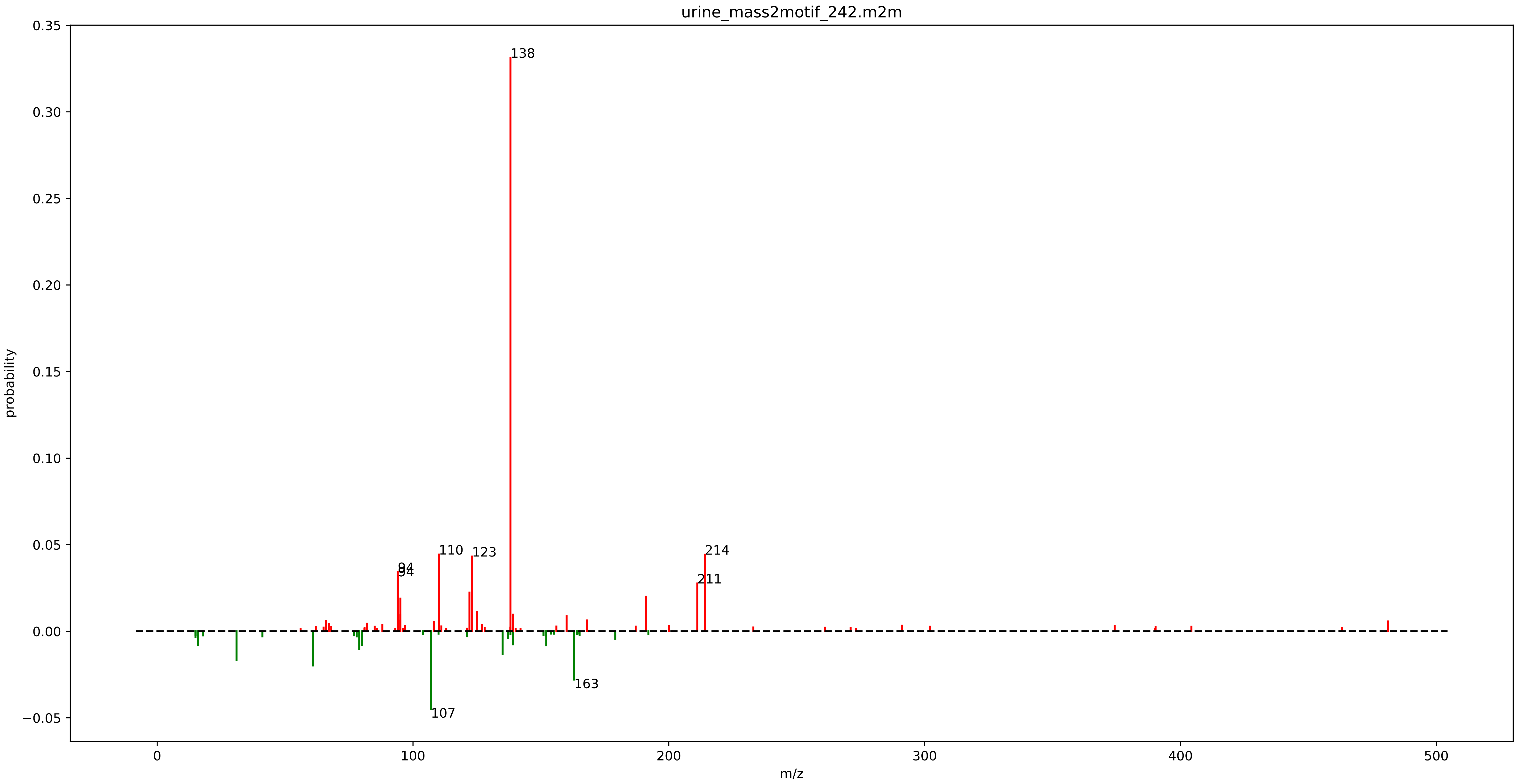
urine_mass2motif_242.m2m
probability
m/z
0.35
0.30
0.25
0.20
0.15
0.10
0.05
0.00
−0.05
0
100
200
300
400
500
138
110
123
214
211
94
94
107
163

urine_mass2motif_242.m2m

fragment_138.0925	: 0.33
loss_106.9975	: 0.04
fragment_110.0975	: 0.04
fragment_214.0825	: 0.04
fragment_123.0675	: 0.04
fragment_94.0425	: 0.03
fragment_94.0675	: 0.03
loss_163.0275	: 0.03
fragment_211.1325	: 0.03
fragment_122.0625	: 0.02
fragment_191.0775	: 0.02
loss_60.9925	: 0.02
fragment_95.0725	: 0.02
loss_31.0175	: 0.02
loss_135.0325	: 0.01
fragment_125.0075	: 0.01
loss_79.0025	: 0.01
fragment_139.0975	: 0.01
fragment_95.0475	: 0.01
fragment_160.0525	: 0.01
loss_152.0775	: 0.01
loss_16.0325	: 0.01
loss_80.0675	: 0.01
loss_139.0975	: 0.01
loss_107.0225	: 0.01
fragment_168.0625	: 0.01
fragment_95.0425	: 0.01
fragment_66.0475	: 0.01
fragment_481.0875	: 0.01
loss_163.0025	: 0.01
fragment_108.0825	: 0.01

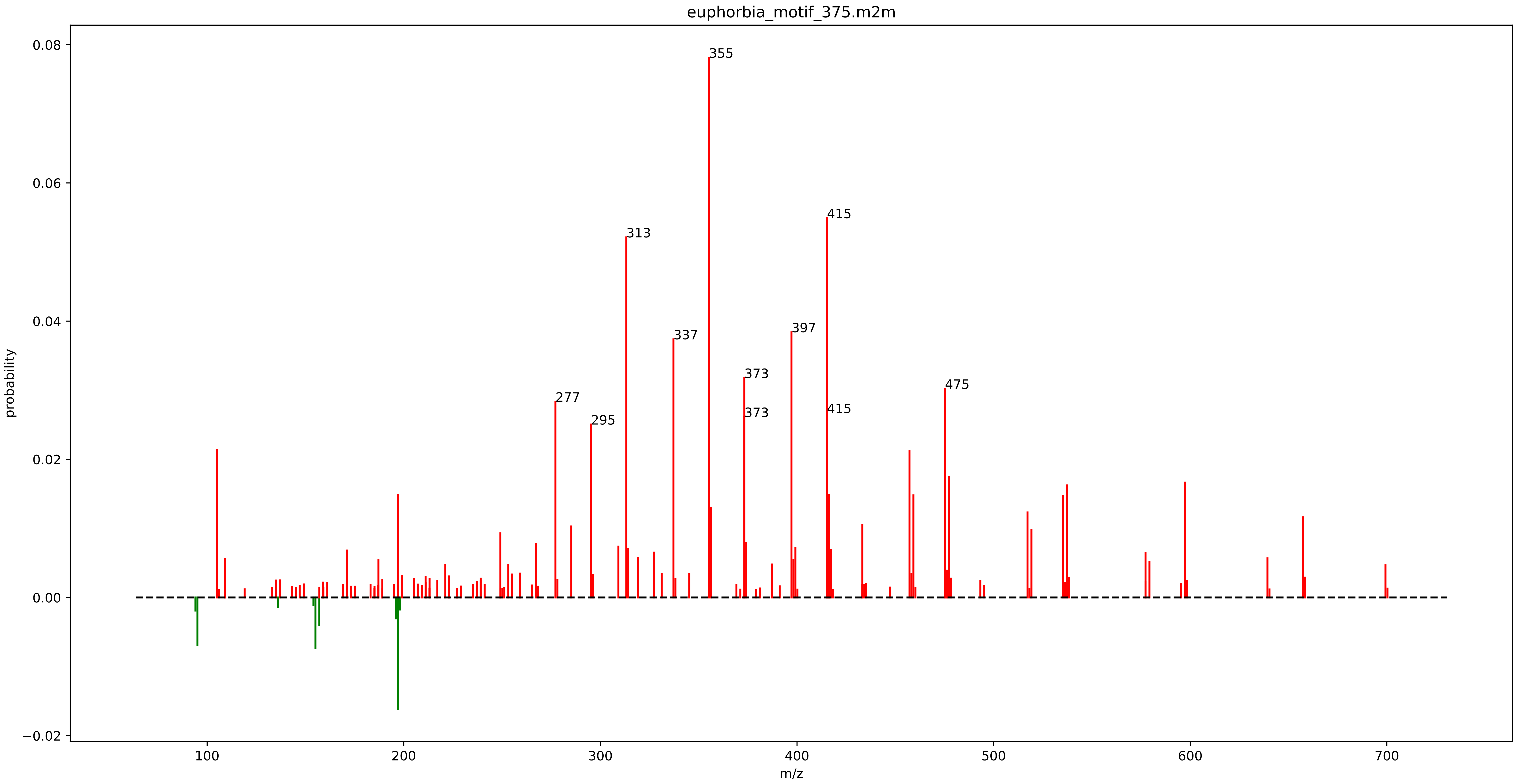
euphorbia_motif_375.m2m
probability
m/z
0.08
0.06
0.04
0.02
0.00
−0.02
100
200
300
400
500
600
700
355
415
313
337
397
373
475
373
415
277
295

euphorbia_motif_375.m2m

fragment_355.1875	: 0.08
fragment_415.2125	: 0.05
fragment_313.1775	: 0.05
fragment_397.1975	: 0.04
fragment_337.1775	: 0.04
fragment_373.1975	: 0.03
fragment_475.2325	: 0.03
fragment_277.1575	: 0.03
fragment_415.2075	: 0.03
fragment_373.2025	: 0.03
fragment_295.1675	: 0.03
fragment_105.0325	: 0.02
fragment_457.2225	: 0.02
fragment_477.2275	: 0.02
fragment_597.2675	: 0.02
fragment_537.2475	: 0.02
loss_197.0875	: 0.02
fragment_416.2125	: 0.01
fragment_197.1175	: 0.01
fragment_459.2125	: 0.01
fragment_535.2525	: 0.01
fragment_356.1925	: 0.01
fragment_517.2425	: 0.01
fragment_657.2875	: 0.01
fragment_433.2225	: 0.01
fragment_285.1825	: 0.01
fragment_519.2375	: 0.01
fragment_249.1625	: 0.01
fragment_475.2275	: 0.01
fragment_374.2025	: 0.01
fragment_267.1725	: 0.01
fragment_309.1825	: 0.01
loss_155.0825	: 0.01
fragment_399.1925	: 0.01
fragment_314.1825	: 0.01
loss_95.0625	: 0.01
fragment_417.2025	: 0.01
fragment_171.1175	: 0.01
fragment_327.1975	: 0.01
fragment_577.2625	: 0.01
loss_197.0925	: 0.01
fragment_319.1675	: 0.01
fragment_639.2775	: 0.01
fragment_109.1025	: 0.01
fragment_398.2025	: 0.01
fragment_187.1125	: 0.01
fragment_579.2575	: 0.01

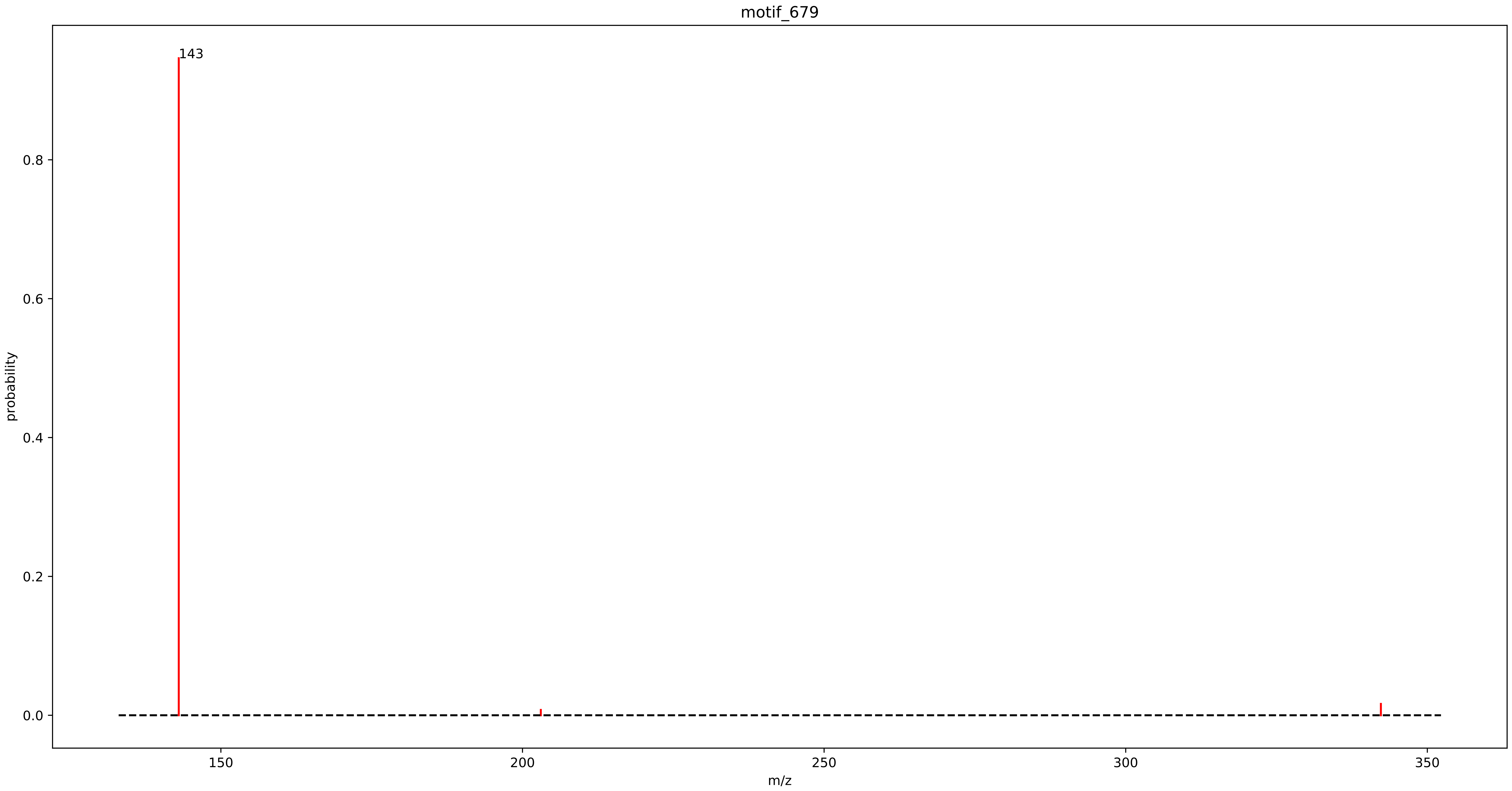

motif_679
143
0.0
0.2
0.4
0.6
0.8
probability
150
200
250
300
350
m/z

motif_679

fragment_143.0175	: 0.95
fragment_342.3025	: 0.02
fragment_203.0375	: 0.01

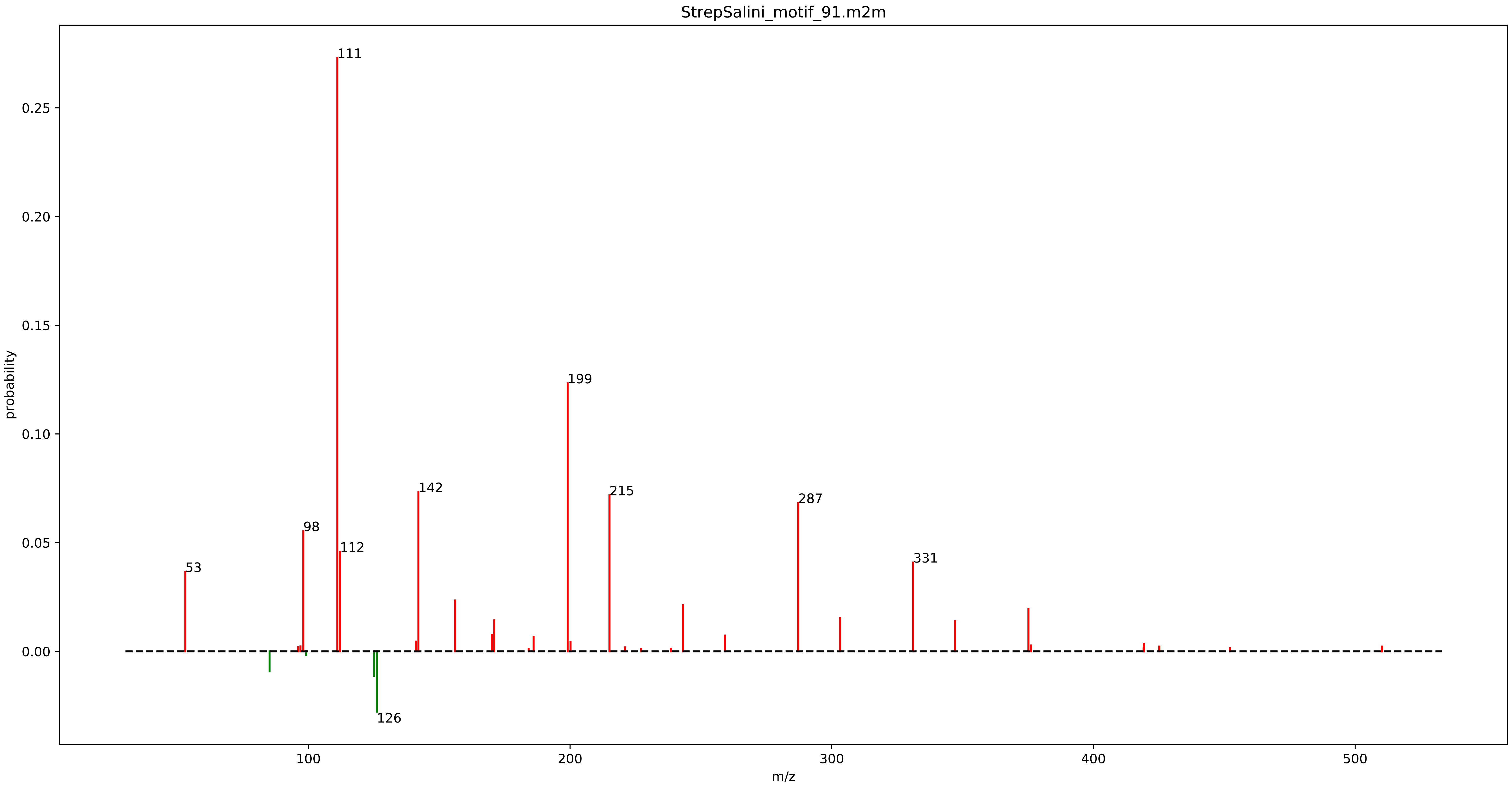

StrepSalini_motif_91.m2m
probability
m/z
0.25
0.20
0.15
0.10
0.05
0.00
100
200
300
400
500
111
199
142
215
287
98
112
53
331
126

StrepSalini_motif_91.m2m

fragment_111.0500	: 0.27
fragment_199.0500	: 0.12
fragment_142.0525	: 0.07
fragment_215.0500	: 0.07
fragment_287.1500	: 0.07
fragment_98.0500	: 0.06
fragment_112.0500	: 0.05
fragment_331.1500	: 0.04
fragment_52.9500	: 0.04
loss_126.1525	: 0.03
fragment_156.0500	: 0.02
fragment_243.1525	: 0.02
fragment_375.1500	: 0.02
fragment_303.1500	: 0.02
fragment_171.0500	: 0.01
fragment_347.1500	: 0.01
loss_125.1525	: 0.01
loss_85.1525	: 0.01
fragment_170.0500	: 0.01
fragment_259.1500	: 0.01
fragment_186.0500	: 0.01

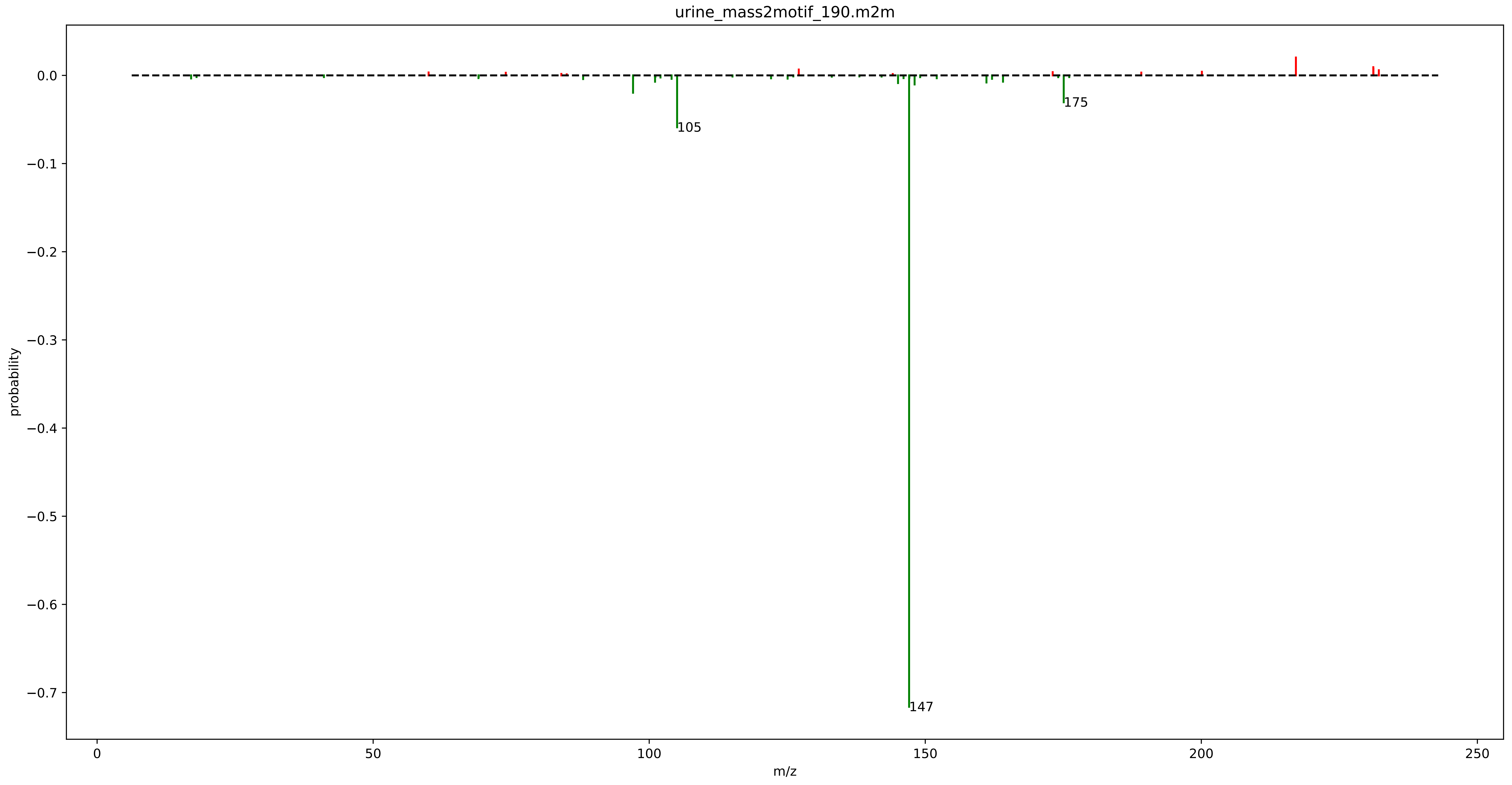

urine_mass2motif_190.m2m
probability
m/z
0.0
−0.1
−0.2
−0.3
−0.4
−0.5
−0.6
−0.7
0
50
100
150
200
250
105
147
175

urine_mass2motif_190.m2m

loss_147.0725	: 0.72
loss_105.0525	: 0.06
loss_175.0775	: 0.03
fragment_217.1325	: 0.02
loss_97.0725	: 0.02
loss_148.0675	: 0.01
fragment_231.1475	: 0.01
loss_145.0675	: 0.01
loss_161.0825	: 0.01
fragment_231.1725	: 0.01
loss_101.0575	: 0.01
loss_164.0825	: 0.01
fragment_127.0875	: 0.01
fragment_232.1525	: 0.01

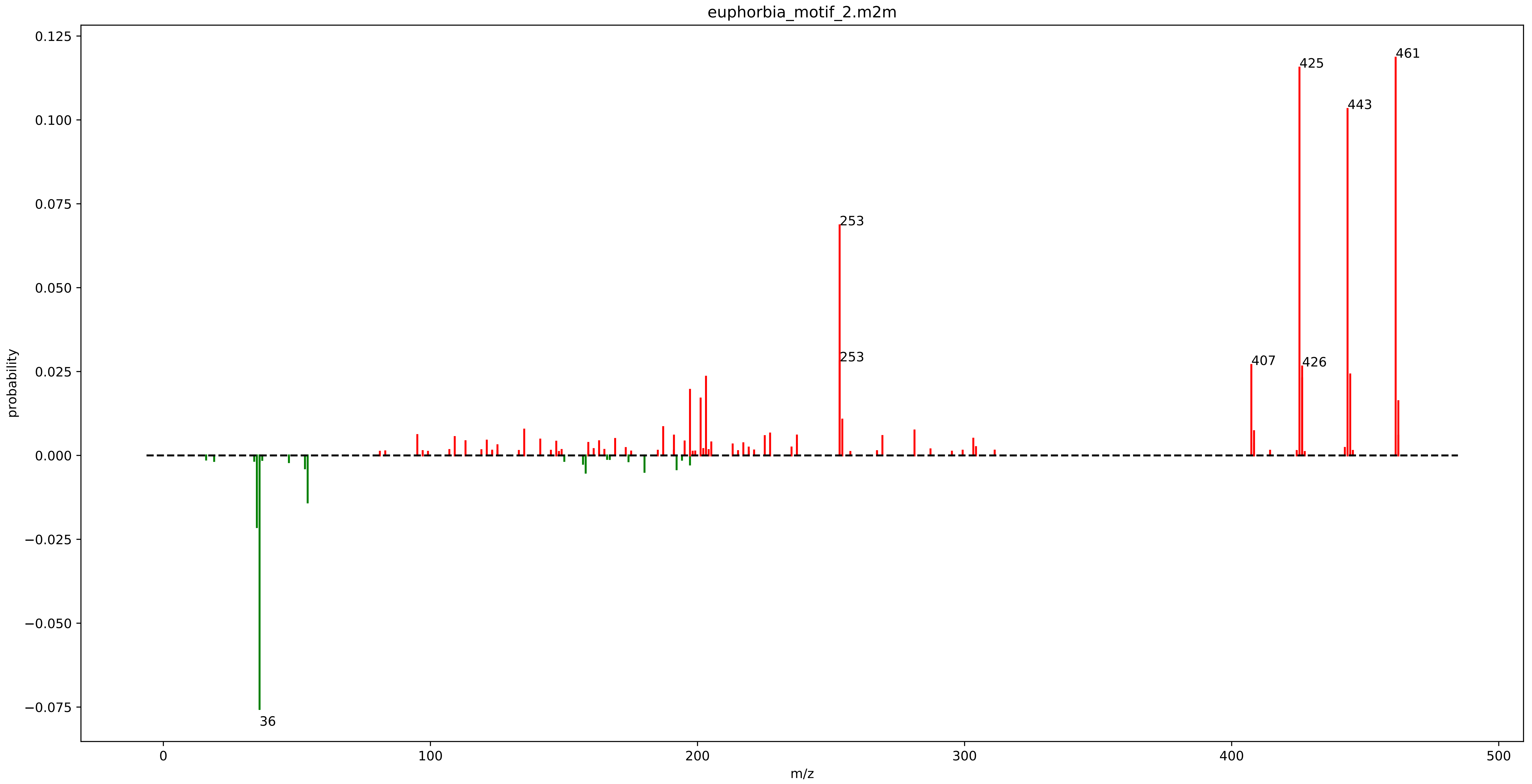

euphorbia_motif_2.m2m
probability
m/z
0.125
0.100
0.075
0.050
0.025
0.000
−0.025
−0.050
−0.075
0
100
200
300
400
500
461
425
443
253
253
407
426
36

euphorbia_motif_2.m2m

fragment_461.3975	: 0.12
fragment_425.3825	: 0.12
fragment_443.3875	: 0.10
loss_36.0225	: 0.08
fragment_253.2125	: 0.07
fragment_253.2175	: 0.03
fragment_407.3675	: 0.03
fragment_426.3825	: 0.03
fragment_407.3625	: 0.02
fragment_444.3875	: 0.02
fragment_203.1775	: 0.02
fragment_426.3775	: 0.02
loss_35.0175	: 0.02
fragment_197.1875	: 0.02
fragment_201.1625	: 0.02
fragment_462.4025	: 0.02
loss_54.0325	: 0.01
fragment_254.2175	: 0.01
fragment_187.1475	: 0.01
fragment_135.1175	: 0.01
fragment_281.2425	: 0.01
fragment_408.3725	: 0.01
fragment_227.1775	: 0.01
fragment_95.0875	: 0.01
fragment_237.2175	: 0.01
fragment_191.1775	: 0.01
fragment_462.3975	: 0.01
fragment_269.2275	: 0.01
fragment_225.1825	: 0.01
fragment_109.1025	: 0.01
loss_158.1325	: 0.01

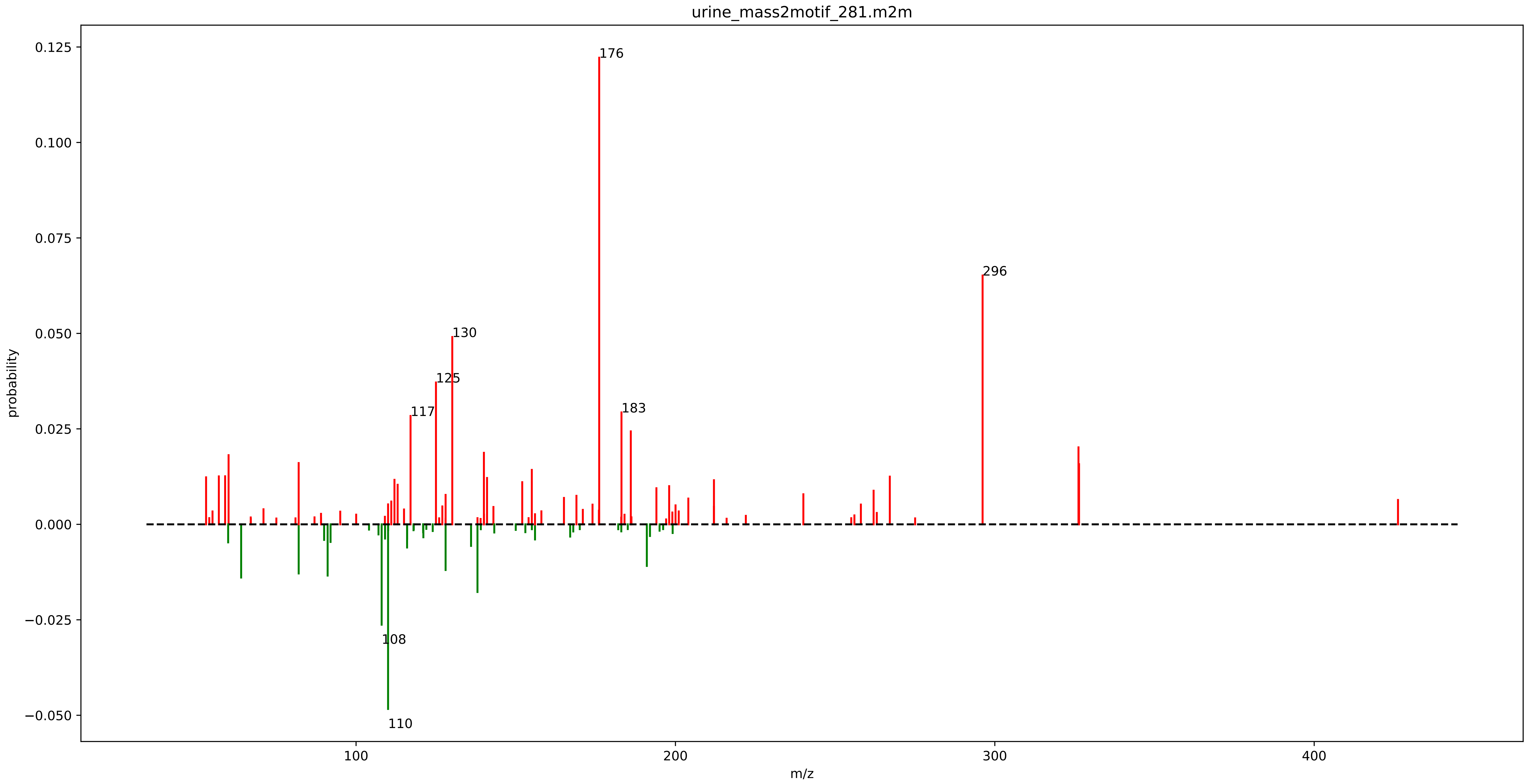

urine_mass2motif_281.m2m
probability
m/z
0.125
0.100
0.075
0.050
0.025
0.000
−0.025
−0.050
100
200
300
400
176
296
130
125
117
183
108
110

urine_mass2motif_281.m2m

fragment_176.1275	: 0.12
fragment_296.1725	: 0.07
fragment_130.1225	: 0.05
loss_110.0225	: 0.05
fragment_125.0175	: 0.04
fragment_183.1025	: 0.03
fragment_117.0575	: 0.03
loss_108.0075	: 0.03
fragment_186.0225	: 0.02
fragment_326.1975	: 0.02
fragment_140.0275	: 0.02
fragment_60.0825	: 0.02
loss_138.0125	: 0.02
fragment_82.0275	: 0.02
fragment_326.3775	: 0.02
fragment_155.0275	: 0.01
loss_64.0175	: 0.01
loss_91.0975	: 0.01
loss_82.0325	: 0.01
fragment_58.9975	: 0.01
fragment_57.0325	: 0.01
fragment_267.1225	: 0.01
fragment_53.0375	: 0.01
fragment_141.0125	: 0.01
loss_128.0325	: 0.01
fragment_112.0225	: 0.01
fragment_212.0575	: 0.01
fragment_152.0275	: 0.01
loss_191.0425	: 0.01
fragment_113.0175	: 0.01
fragment_198.0325	: 0.01
fragment_194.0425	: 0.01
fragment_262.0475	: 0.01
fragment_240.0525	: 0.01
fragment_128.0325	: 0.01
fragment_169.0025	: 0.01
fragment_165.0925	: 0.01
fragment_204.0325	: 0.01
fragment_426.2425	: 0.01
loss_115.9725	: 0.01
fragment_111.0275	: 0.01
loss_136.0025	: 0.01
fragment_110.0225	: 0.01
fragment_258.0625	: 0.01
fragment_174.0375	: 0.01

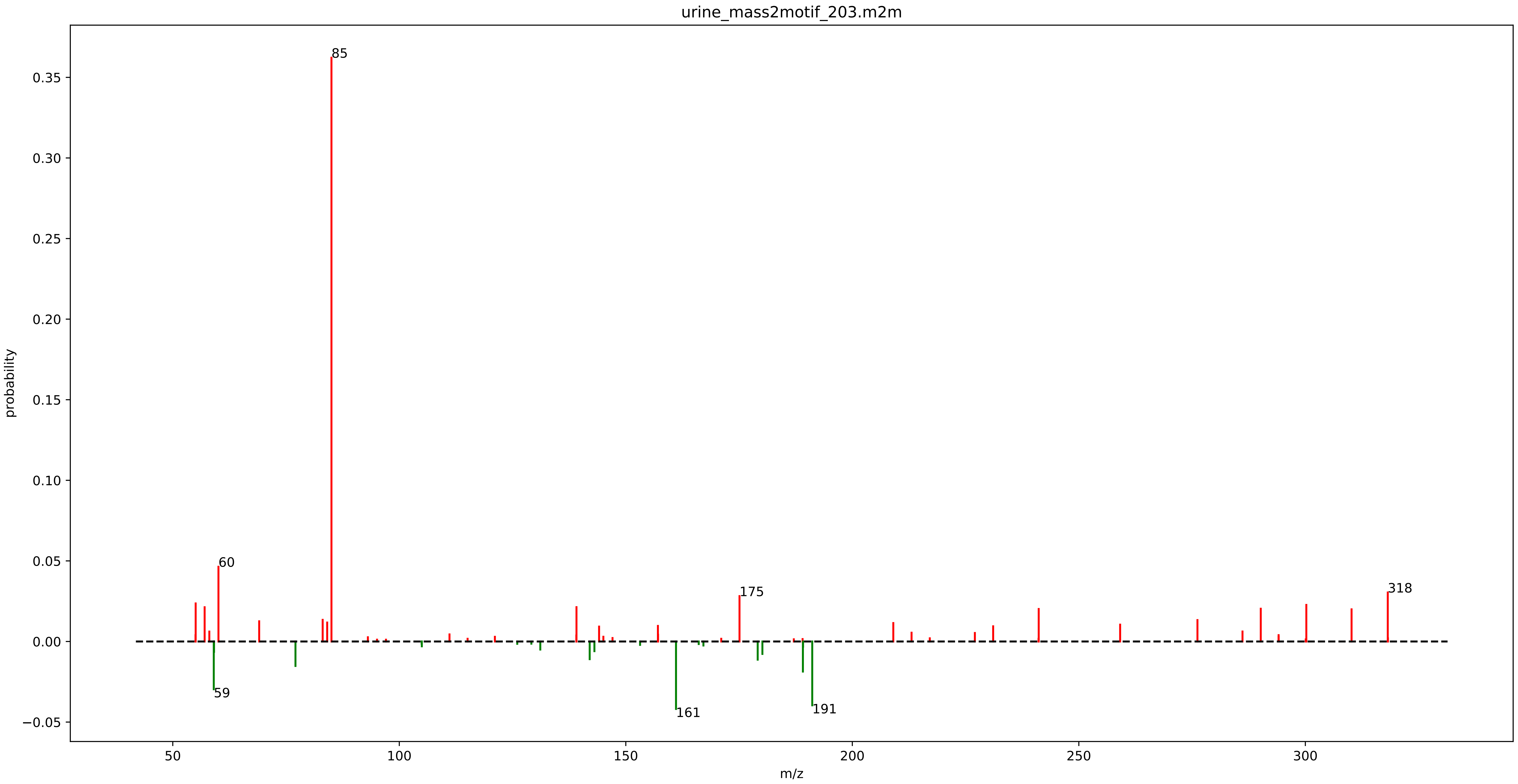
urine_mass2motif_203.m2m
probability
m/z
85
60
59
175
161
191
318
0.35
0.30
0.25
0.20
0.15
0.10
0.05
0.00
−0.05
50
100
150
200
250
300

urine_mass2motif_203.m2m

fragment_85.0275	: 0.36
fragment_60.0825	: 0.05
loss_161.0825	: 0.04
loss_191.1525	: 0.04
fragment_318.1925	: 0.03
loss_59.0475	: 0.03
fragment_175.0975	: 0.03
fragment_55.0525	: 0.02
fragment_300.2175	: 0.02
fragment_139.1125	: 0.02
fragment_57.0325	: 0.02
fragment_290.1575	: 0.02
fragment_241.1425	: 0.02
fragment_310.2025	: 0.02
loss_189.0925	: 0.02
loss_77.0825	: 0.02
fragment_83.0875	: 0.01
fragment_276.1825	: 0.01
fragment_69.0725	: 0.01
fragment_84.0825	: 0.01
fragment_209.0475	: 0.01
loss_179.1125	: 0.01
loss_142.0275	: 0.01
fragment_259.1175	: 0.01
fragment_157.0875	: 0.01
fragment_231.0875	: 0.01
fragment_144.1025	: 0.01
loss_180.1525	: 0.01
loss_59.1075	: 0.01
fragment_286.1275	: 0.01
fragment_58.0675	: 0.01
loss_143.0725	: 0.01
fragment_213.0775	: 0.01
fragment_227.0525	: 0.01

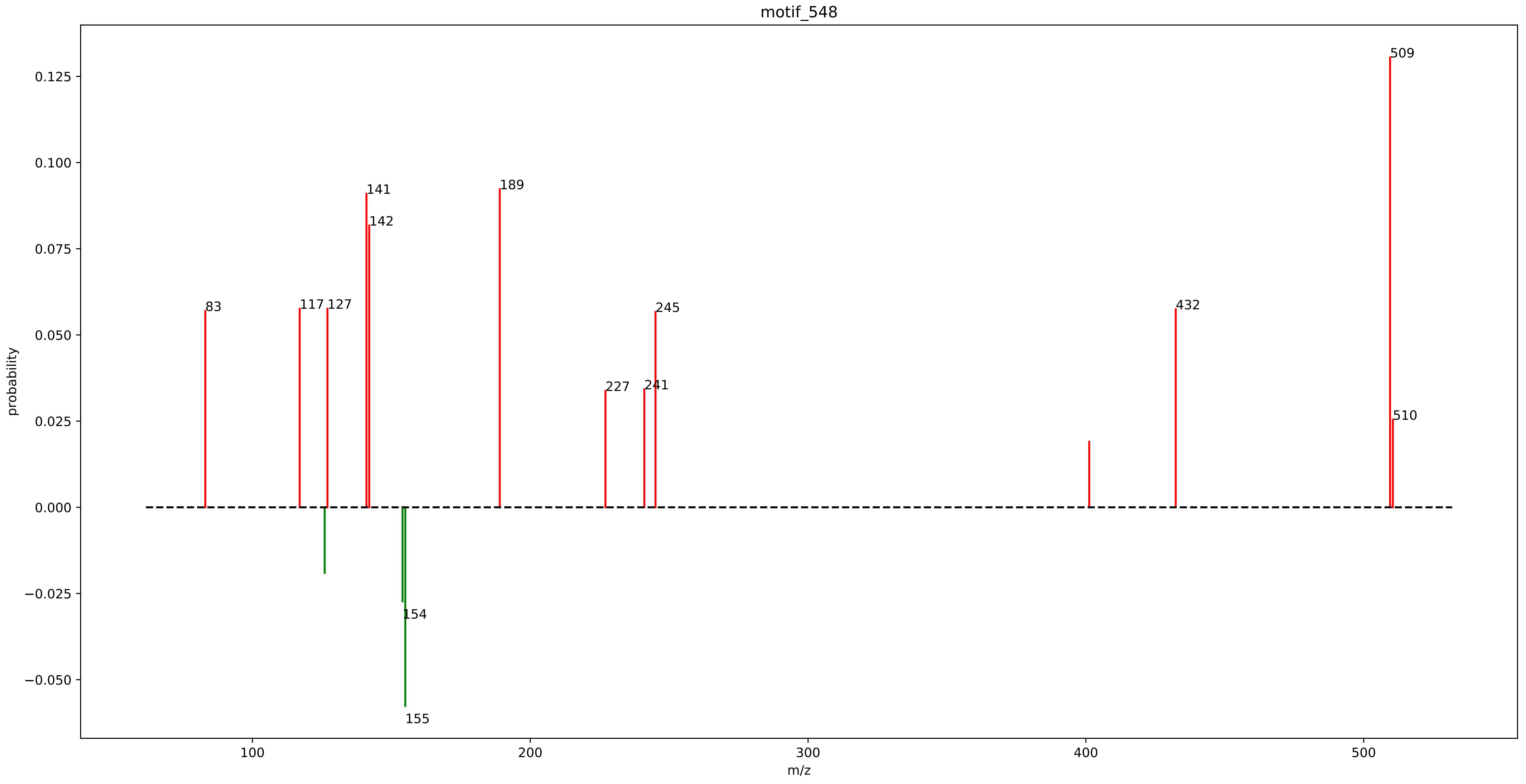
motif_548
probability
m/z
509
141
189
142
83
117
127
245
432
227
241
510
154
155
0.125
0.100
0.075
0.050
0.025
0.000
−0.025
−0.050
100
200
300
400
500

motif_548

fragment_509.4925	: 0.13
fragment_189.0275	: 0.09
fragment_141.0475	: 0.09
fragment_142.0525	: 0.08
fragment_117.0075	: 0.06
fragment_127.0125	: 0.06
loss_155.0275	: 0.06
fragment_432.3425	: 0.06
fragment_83.0475	: 0.06
fragment_245.0925	: 0.06
fragment_241.0625	: 0.03
fragment_227.0675	: 0.03
loss_154.0275	: 0.03
fragment_510.4925	: 0.03
fragment_401.2075	: 0.02
loss_126.0075	: 0.02

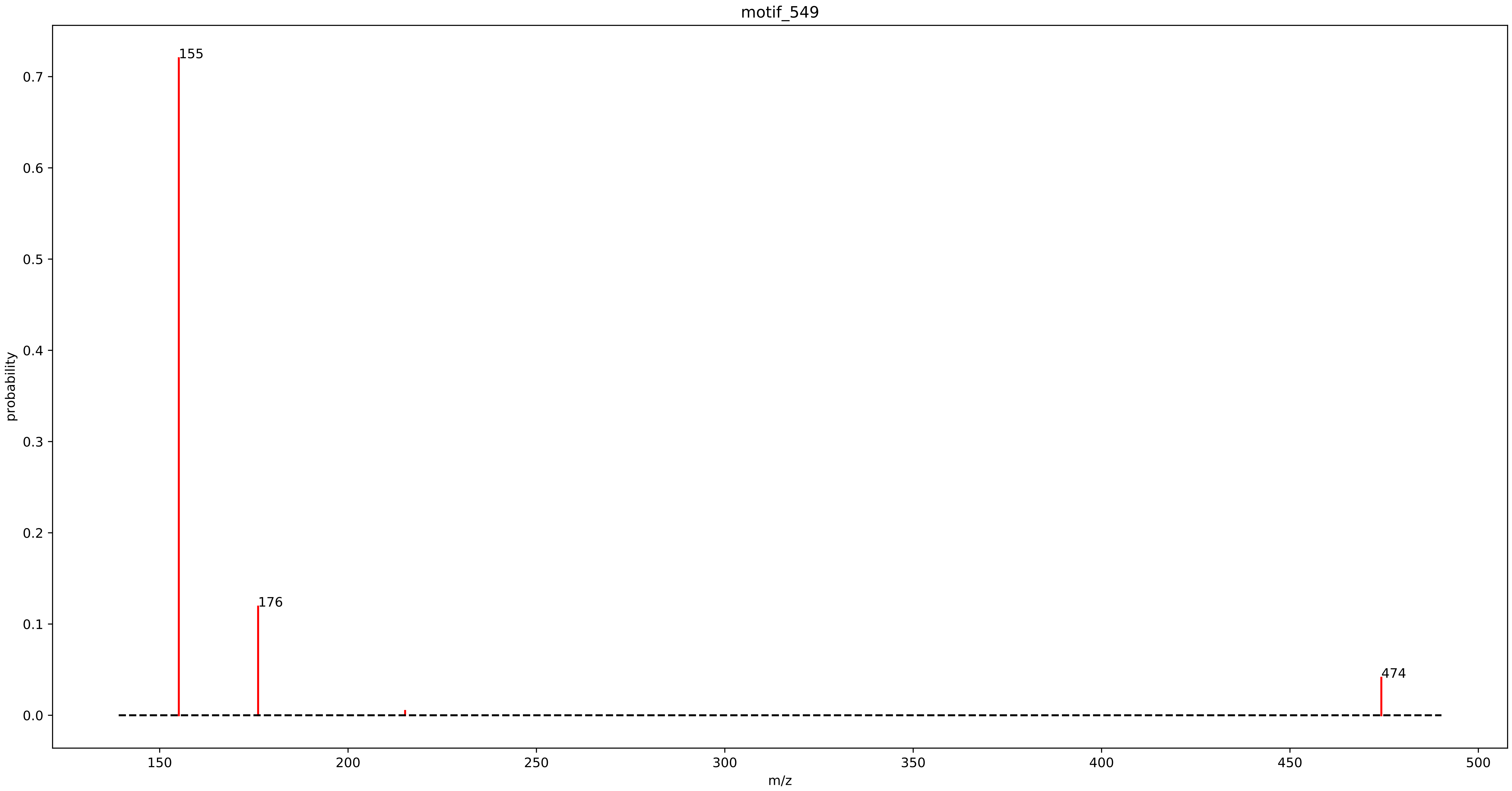

motif_549
155
176
474
0.7
0.6
0.5
0.4
0.3
0.2
0.1
0.0
probability
150
200
250
300
350
400
450
500
m/z

motif_549

fragment_155.0825	: 0.72
fragment_176.1275	: 0.12
fragment_474.2525	: 0.04

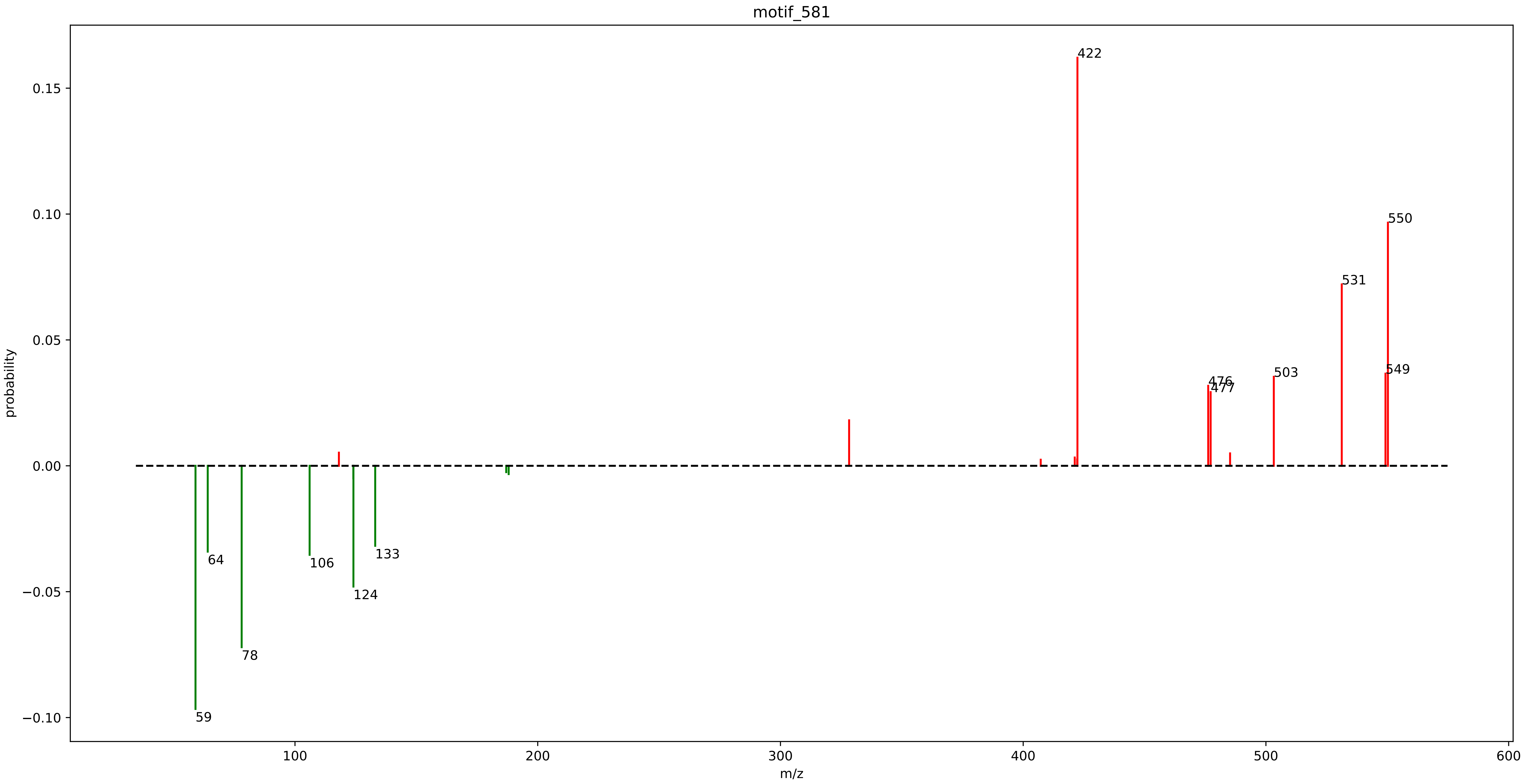
motif_581
probability
m/z
0.15
0.10
0.05
0.00
−0.05
−0.10
100
200
300
400
500
600
422
550
531
503
476
477
549
59
64
78
106
124
133

motif_581

fragment_422.3475	: 0.16
fragment_550.2575	: 0.10
loss_59.0125	: 0.10
fragment_531.2375	: 0.07
loss_78.0275	: 0.07
loss_124.0725	: 0.05
fragment_549.2525	: 0.04
fragment_503.2425	: 0.04
loss_106.0225	: 0.04
loss_64.0475	: 0.03
fragment_476.2225	: 0.03
loss_133.0475	: 0.03
fragment_477.2275	: 0.03
fragment_328.2825	: 0.02
fragment_118.0875	: 0.01

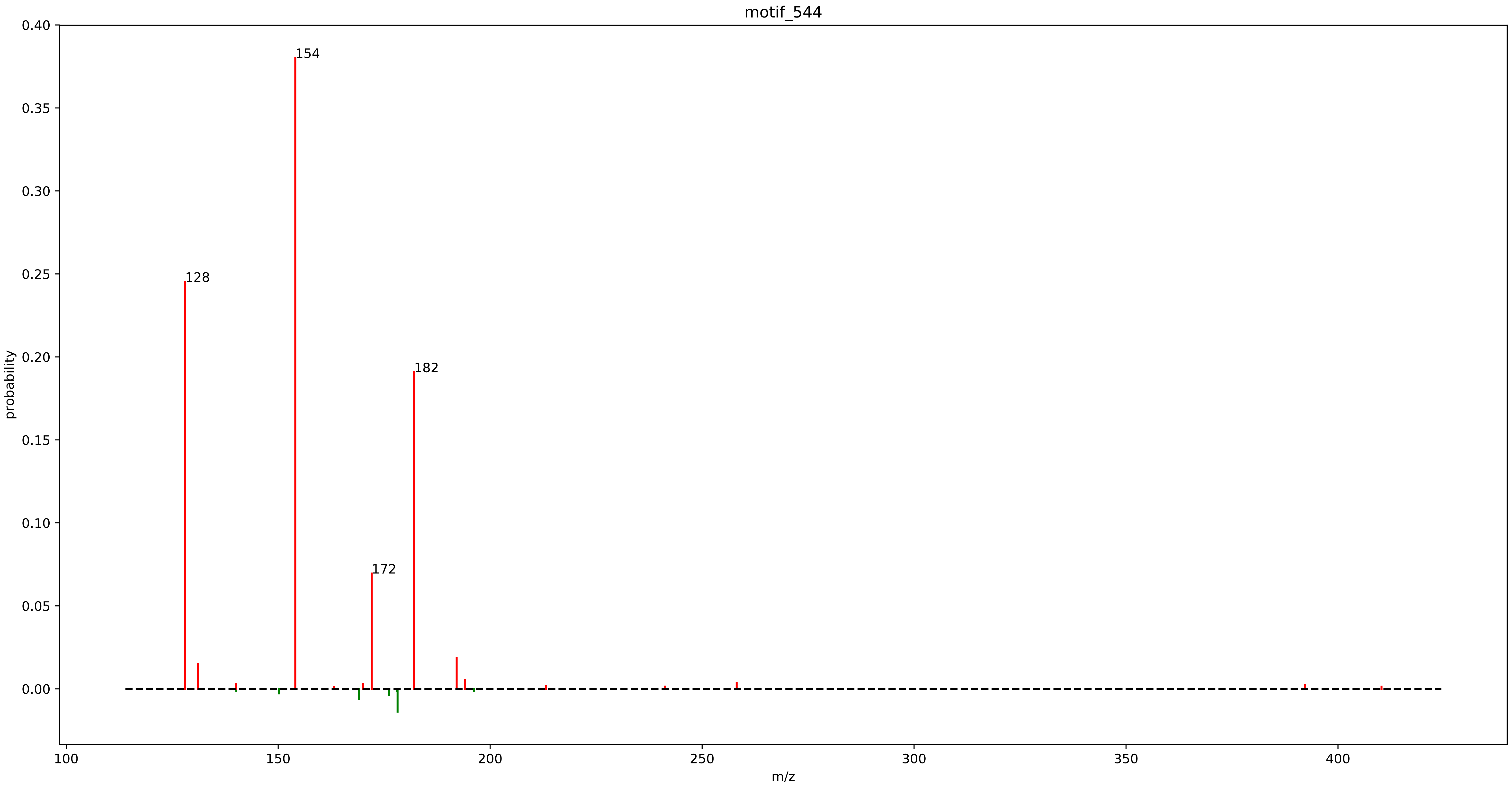
motif_544
probability
m/z
0.40
0.35
0.30
0.25
0.20
0.15
0.10
0.05
0.00
100
150
200
250
300
350
400
154
128
182
172

motif_544

fragment_154.0475	: 0.38
fragment_128.0725	: 0.25
fragment_182.0825	: 0.19
fragment_172.0575	: 0.07
fragment_192.1025	: 0.02
fragment_131.0875	: 0.02
loss_178.1675	: 0.01
loss_169.0725	: 0.01
fragment_194.1175	: 0.01

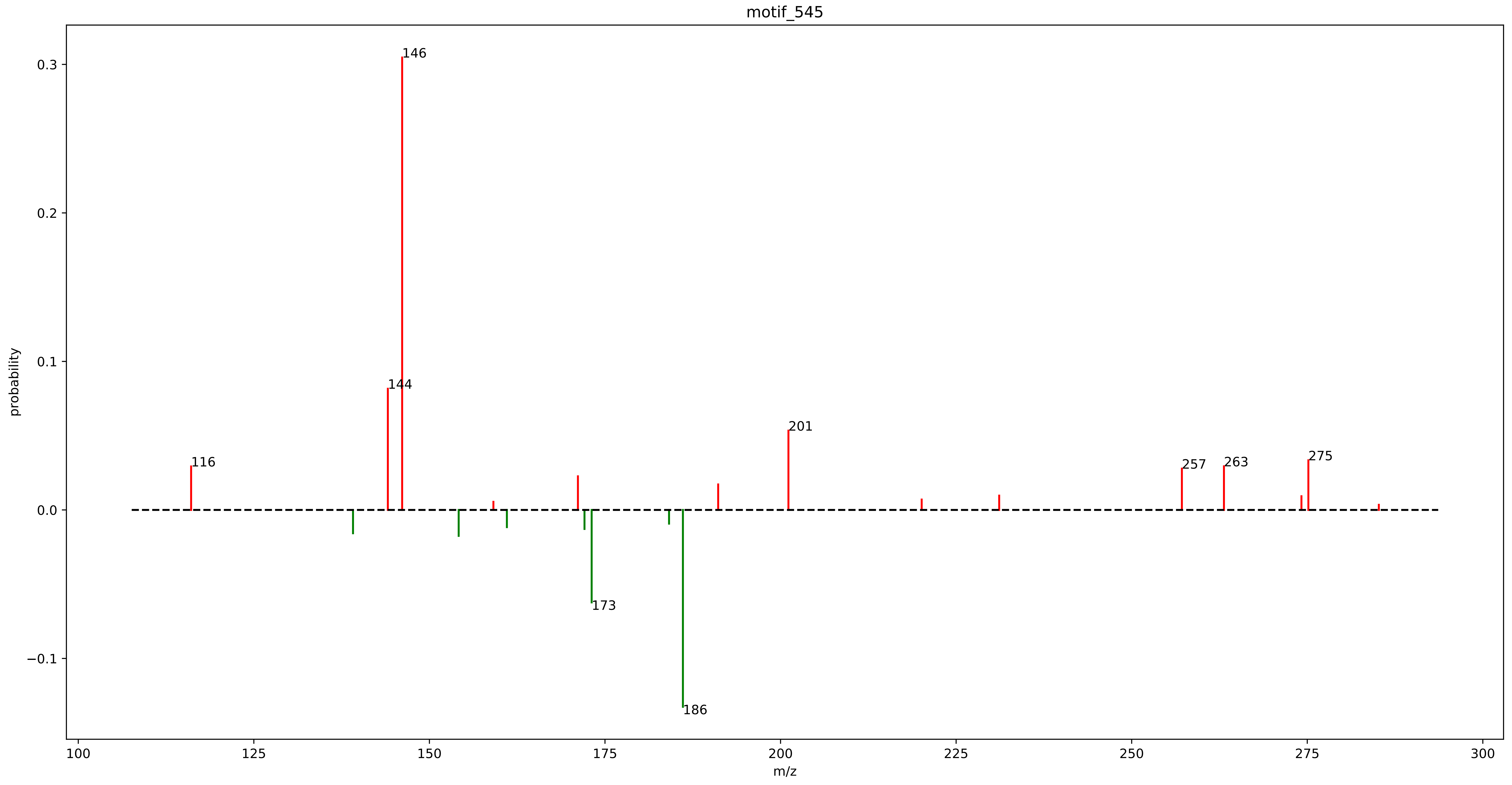
motif_545
probability
m/z
0.3
0.2
0.1
0.0
−0.1
100
125
150
175
200
225
250
275
300
146
144
116
201
257
263
275
173
186

motif_545

fragment_146.1175	: 0.30
loss_186.0975	: 0.13
fragment_144.0825	: 0.08
loss_173.1025	: 0.06
fragment_201.1225	: 0.05
fragment_275.1575	: 0.03
fragment_263.1375	: 0.03
fragment_116.0675	: 0.03
fragment_257.1475	: 0.03
fragment_171.1475	: 0.02
loss_154.1675	: 0.02
fragment_191.1175	: 0.02
loss_139.1225	: 0.02
loss_172.0825	: 0.01
loss_161.0325	: 0.01
fragment_231.1325	: 0.01
loss_184.1225	: 0.01
fragment_274.1725	: 0.01
fragment_220.0975	: 0.01
fragment_159.1125	: 0.01

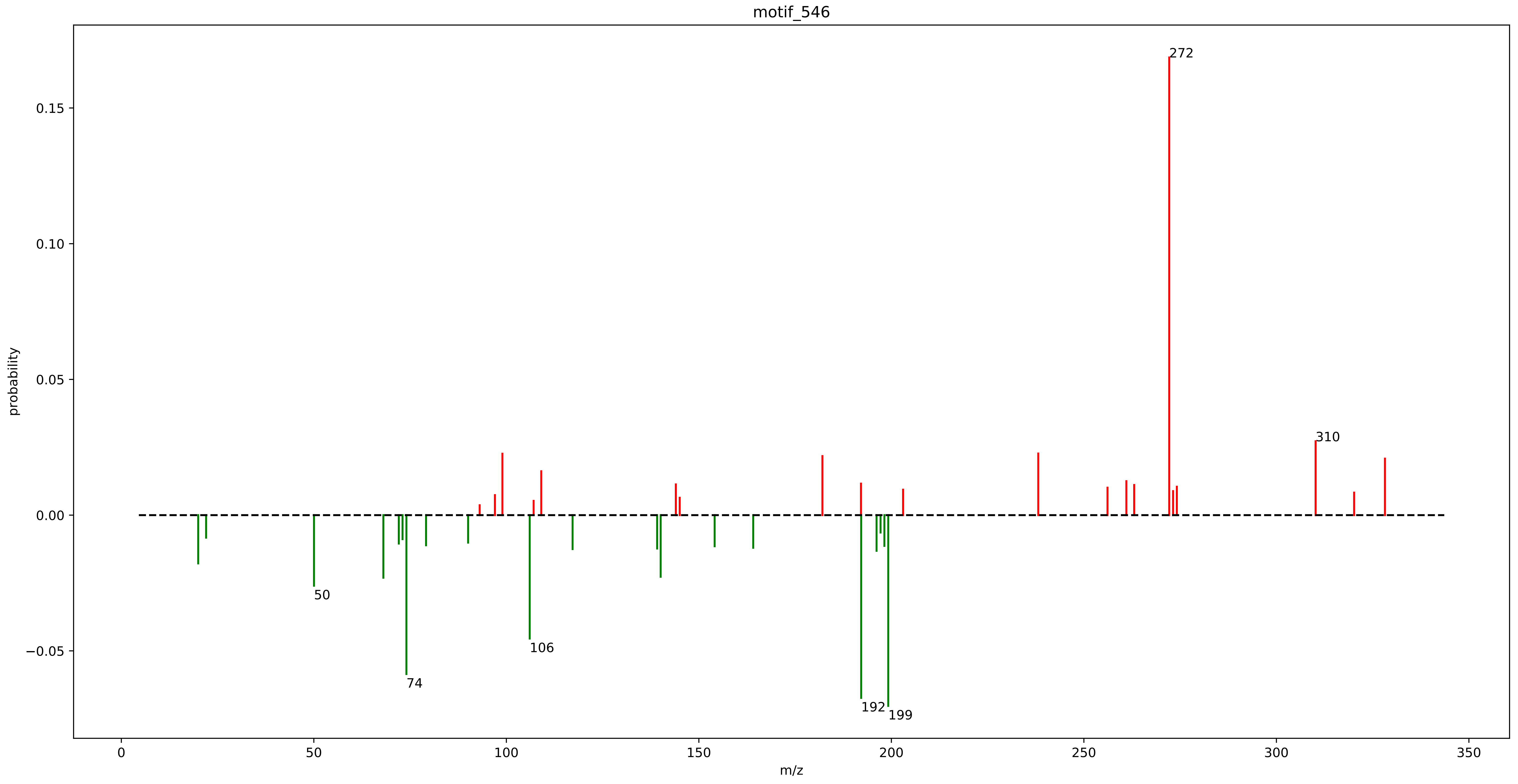

motif_546
272
310
50
74
106
192
199
0.15
0.10
0.05
0.00
−0.05
probability
0
50
100
150
200
250
300
350
m/z

motif_546

fragment_272.1625	: 0.17
loss_199.1825	: 0.07
loss_192.1525	: 0.07
loss_74.0375	: 0.06
loss_106.0625	: 0.05
fragment_310.1775	: 0.03
loss_50.0375	: 0.03
loss_68.0475	: 0.02
fragment_238.1475	: 0.02
loss_140.0775	: 0.02
fragment_98.9725	: 0.02
fragment_182.0825	: 0.02
fragment_328.1925	: 0.02
loss_19.9625	: 0.02
fragment_109.0625	: 0.02
loss_196.1475	: 0.01
loss_117.1975	: 0.01
fragment_261.0275	: 0.01
loss_139.1625	: 0.01
loss_164.1175	: 0.01
fragment_192.1025	: 0.01
loss_154.0975	: 0.01
fragment_144.0175	: 0.01
loss_198.1825	: 0.01
loss_79.1425	: 0.01
fragment_263.0575	: 0.01
loss_72.0575	: 0.01
fragment_274.1425	: 0.01
fragment_256.1325	: 0.01
loss_90.0675	: 0.01
fragment_203.0375	: 0.01
loss_73.0325	: 0.01
fragment_273.1675	: 0.01
loss_22.0175	: 0.01
fragment_320.1825	: 0.01
fragment_97.0275	: 0.01
loss_197.1875	: 0.01
fragment_145.0175	: 0.01
fragment_107.0825	: 0.01

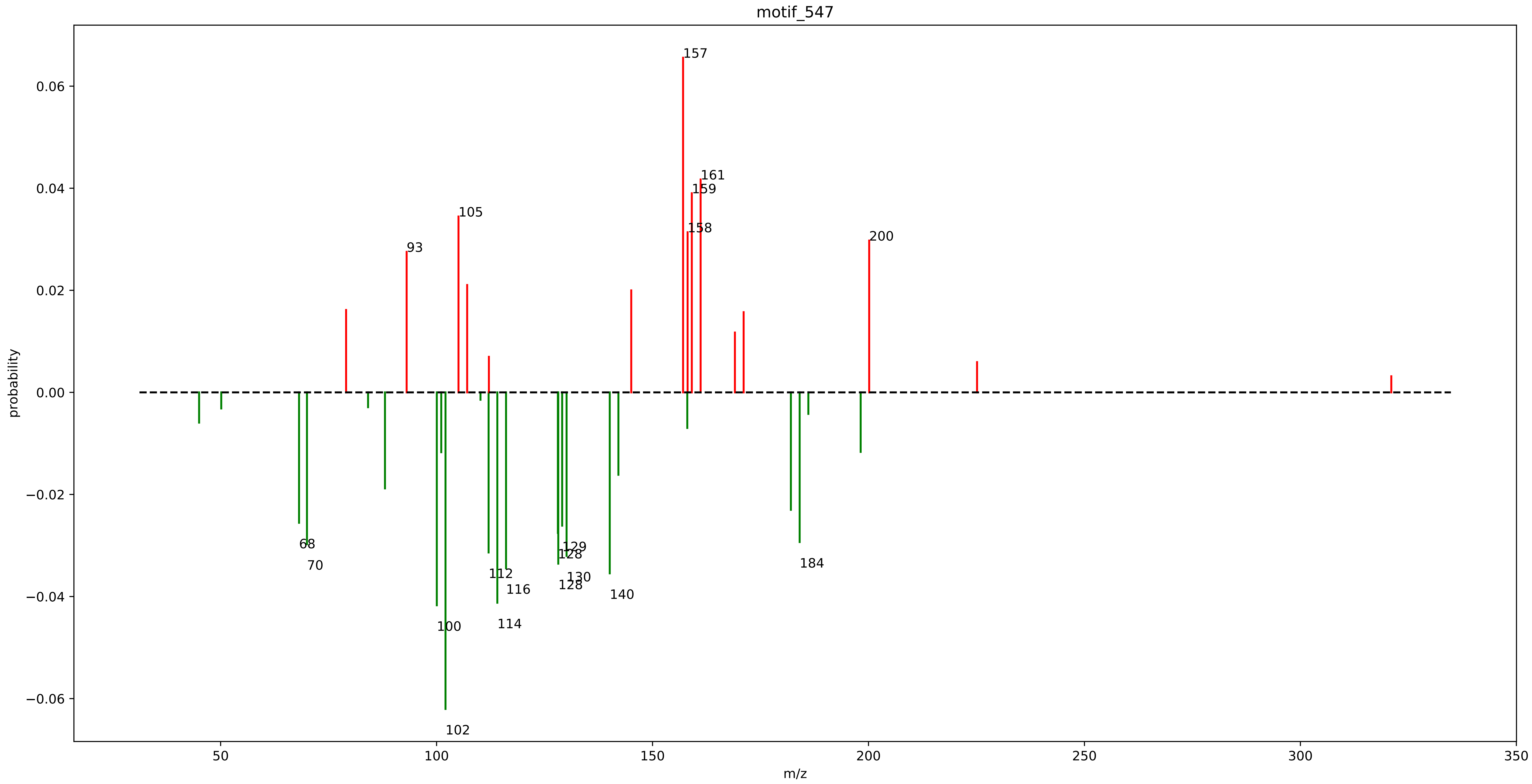

motif_547
probability
m/z
0.06
0.04
0.02
0.00
−0.02
−0.04
−0.06
50
100
150
200
250
300
350
157
161
159
105
158
200
93
68
70
112
116
114
100
102
129
128
130
128
140
184

motif_547

fragment_157.0725	: 0.07
loss_102.0675	: 0.06
loss_100.0525	: 0.04
fragment_161.1325	: 0.04
loss_114.0675	: 0.04
fragment_159.0925	: 0.04
loss_140.0925	: 0.04
loss_116.0825	: 0.03
fragment_105.0725	: 0.03
loss_128.1775	: 0.03
loss_130.0975	: 0.03
loss_112.0425	: 0.03
fragment_158.1175	: 0.03
fragment_200.1625	: 0.03
loss_69.9925	: 0.03
loss_184.0625	: 0.03
loss_128.0825	: 0.03
fragment_93.0675	: 0.03
loss_129.0775	: 0.03
loss_68.1575	: 0.03
loss_182.0325	: 0.02
fragment_107.0825	: 0.02
fragment_145.0725	: 0.02
loss_88.0525	: 0.02
fragment_79.0525	: 0.02
loss_142.0975	: 0.02
fragment_171.0925	: 0.02
fragment_169.0725	: 0.01
loss_101.0825	: 0.01
loss_198.1875	: 0.01
fragment_112.1125	: 0.01
loss_158.0475	: 0.01
loss_45.0175	: 0.01
fragment_225.1375	: 0.01

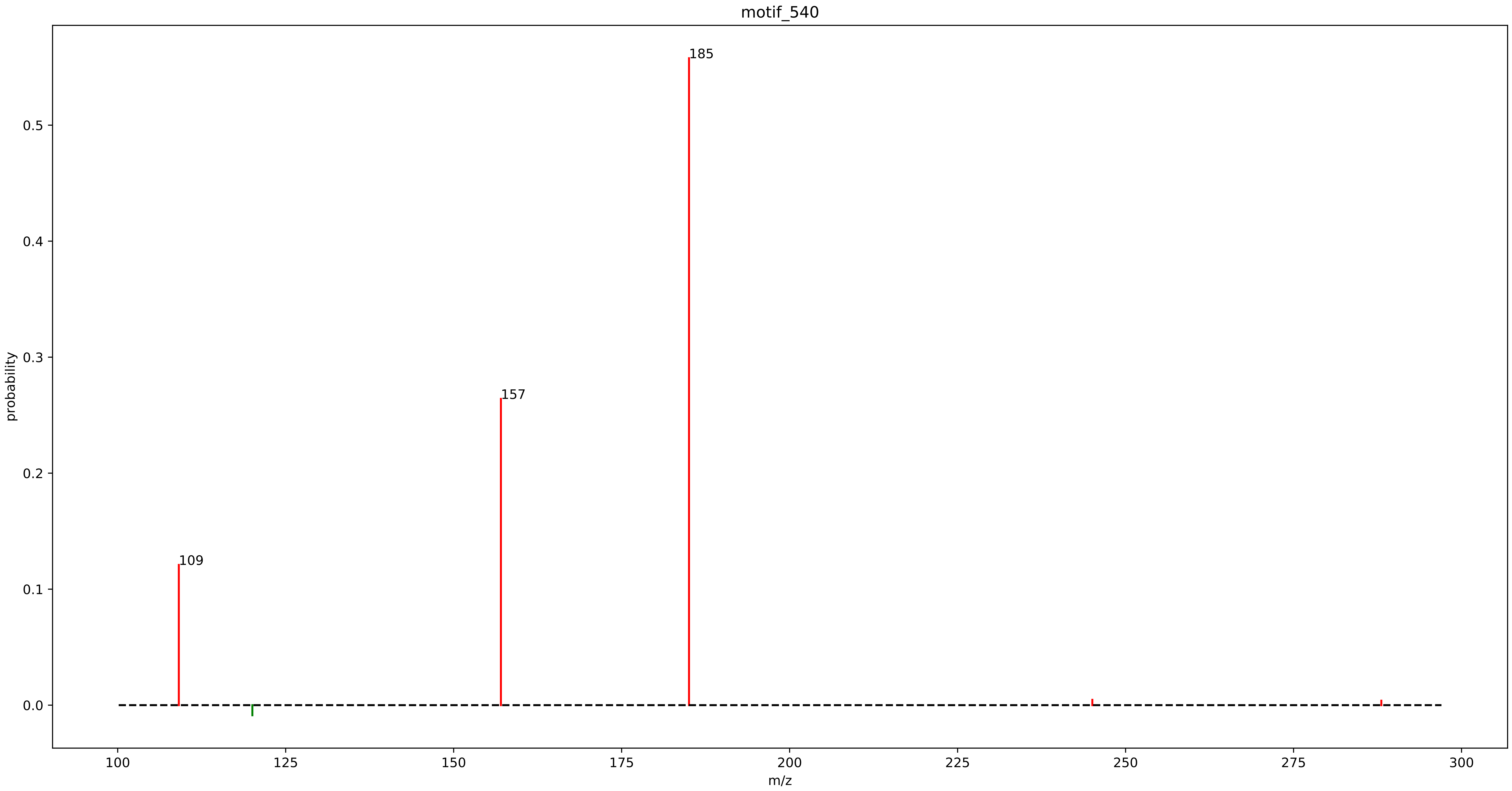

motif_540
185
157
109
probability
0.5
0.4
0.3
0.2
0.1
0.0
100
125
150
175
200
225
250
275
300
m/z

motif_540

fragment_185.0275	: 0.56
fragment_157.0325	: 0.26
fragment_109.0975	: 0.12
loss_120.0425	: 0.01

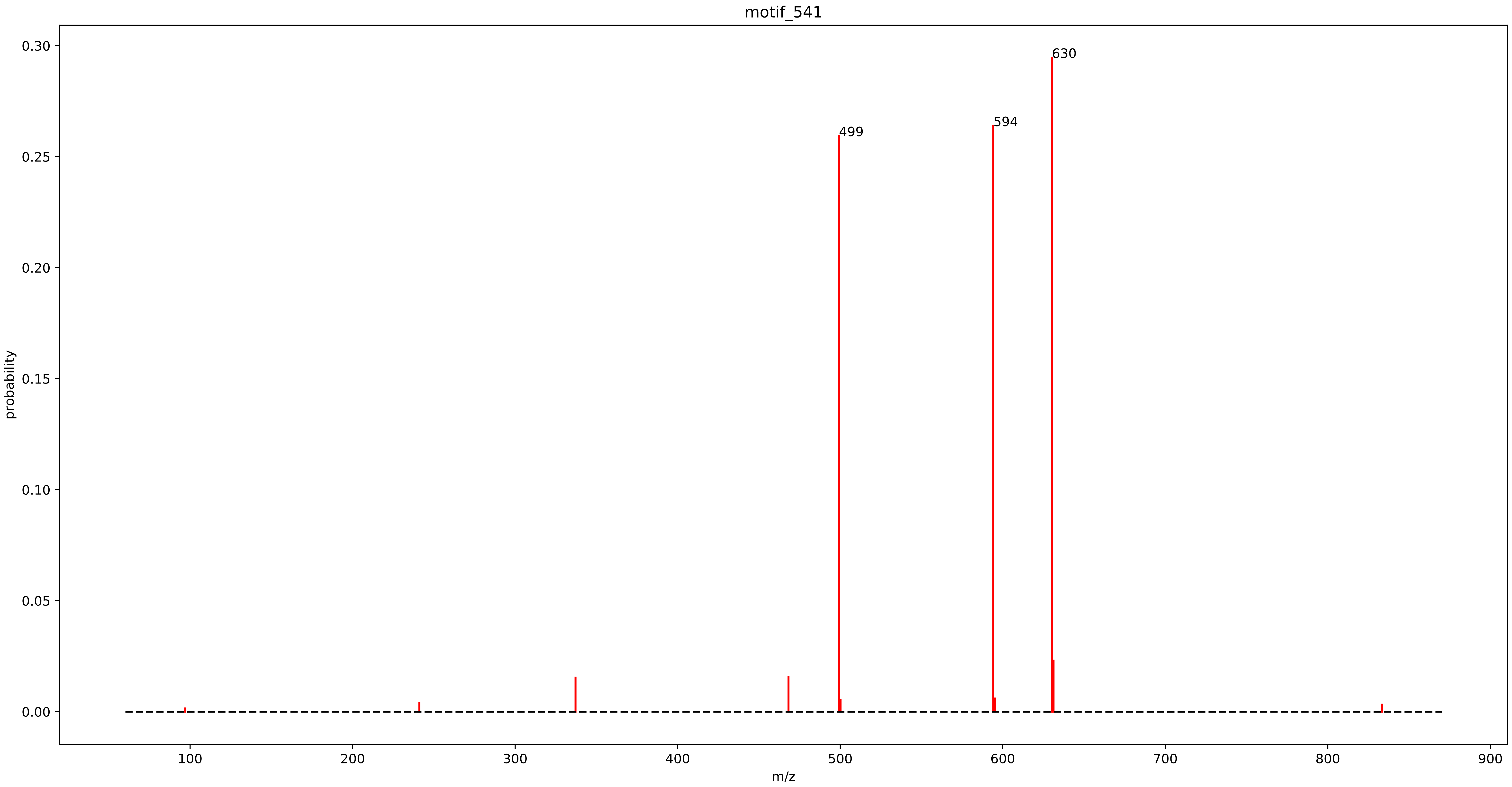
motif_541
probability
m/z
630
594
499
0.30
0.25
0.20
0.15
0.10
0.05
0.00
100
200
300
400
500
600
700
800
900

motif_541

fragment_630.2225	: 0.29
fragment_594.2025	: 0.26
fragment_499.1825	: 0.26
fragment_631.2275	: 0.02
fragment_468.1725	: 0.02
fragment_337.1325	: 0.02
fragment_595.2075	: 0.01
fragment_500.1875	: 0.01

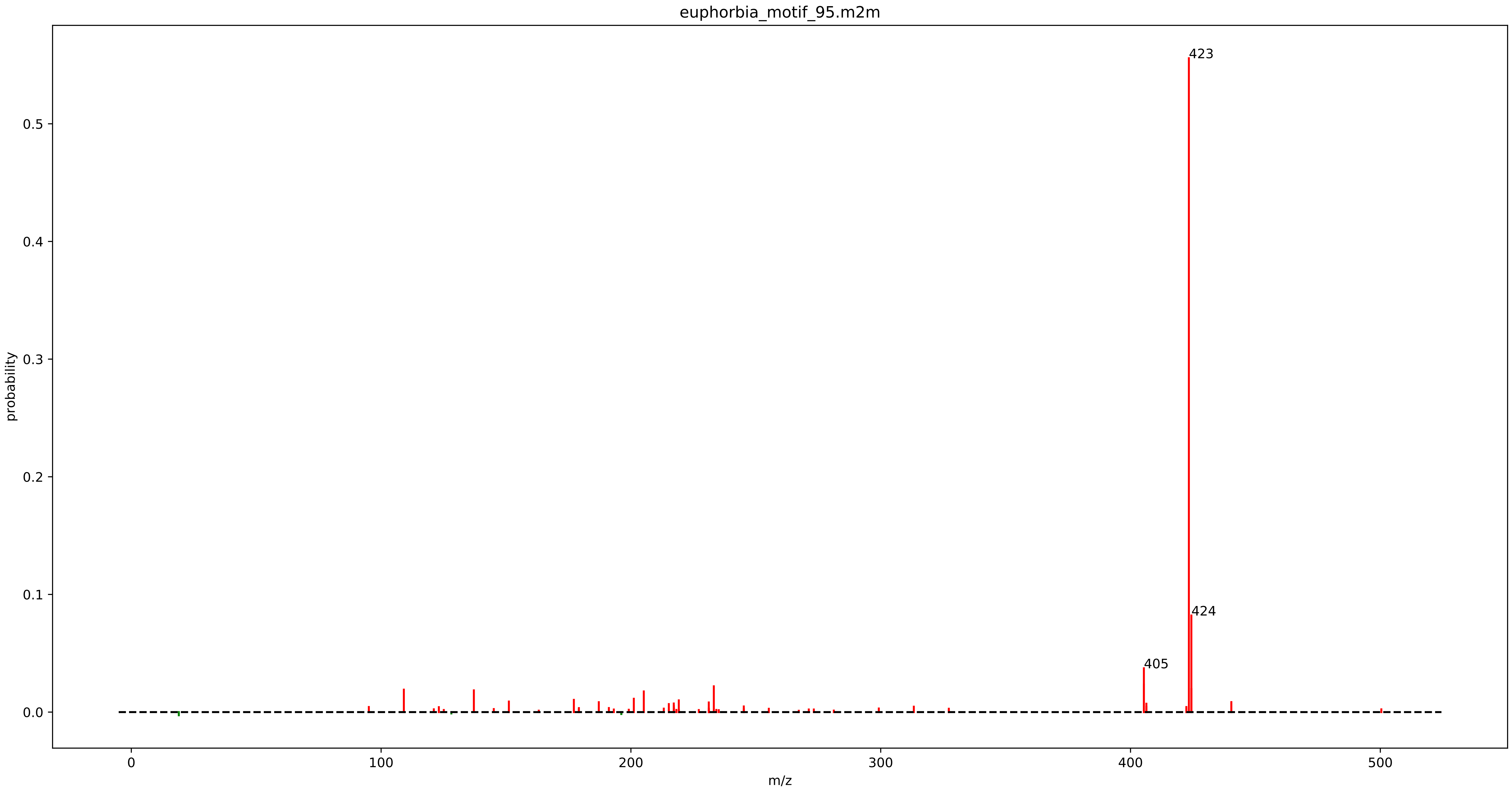
euphorbia_motif_95.m2m
423
424
405
probability
m/z
0.0
0.1
0.2
0.3
0.4
0.5
0
100
200
300
400
500

euphorbia_motif_95.m2m

fragment_423.3625	: 0.56
fragment_424.3625	: 0.08
fragment_405.3525	: 0.04
fragment_405.3475	: 0.02
fragment_233.1875	: 0.02
fragment_424.3675	: 0.02
fragment_109.1025	: 0.02
fragment_137.1325	: 0.02
fragment_205.1575	: 0.02
fragment_201.1625	: 0.01
fragment_177.1625	: 0.01
fragment_219.1725	: 0.01
fragment_151.1475	: 0.01
fragment_440.3625	: 0.01
fragment_187.1475	: 0.01
fragment_231.1725	: 0.01
fragment_440.3575	: 0.01
fragment_217.1575	: 0.01
fragment_406.3525	: 0.01
fragment_215.1775	: 0.01
fragment_217.1925	: 0.01

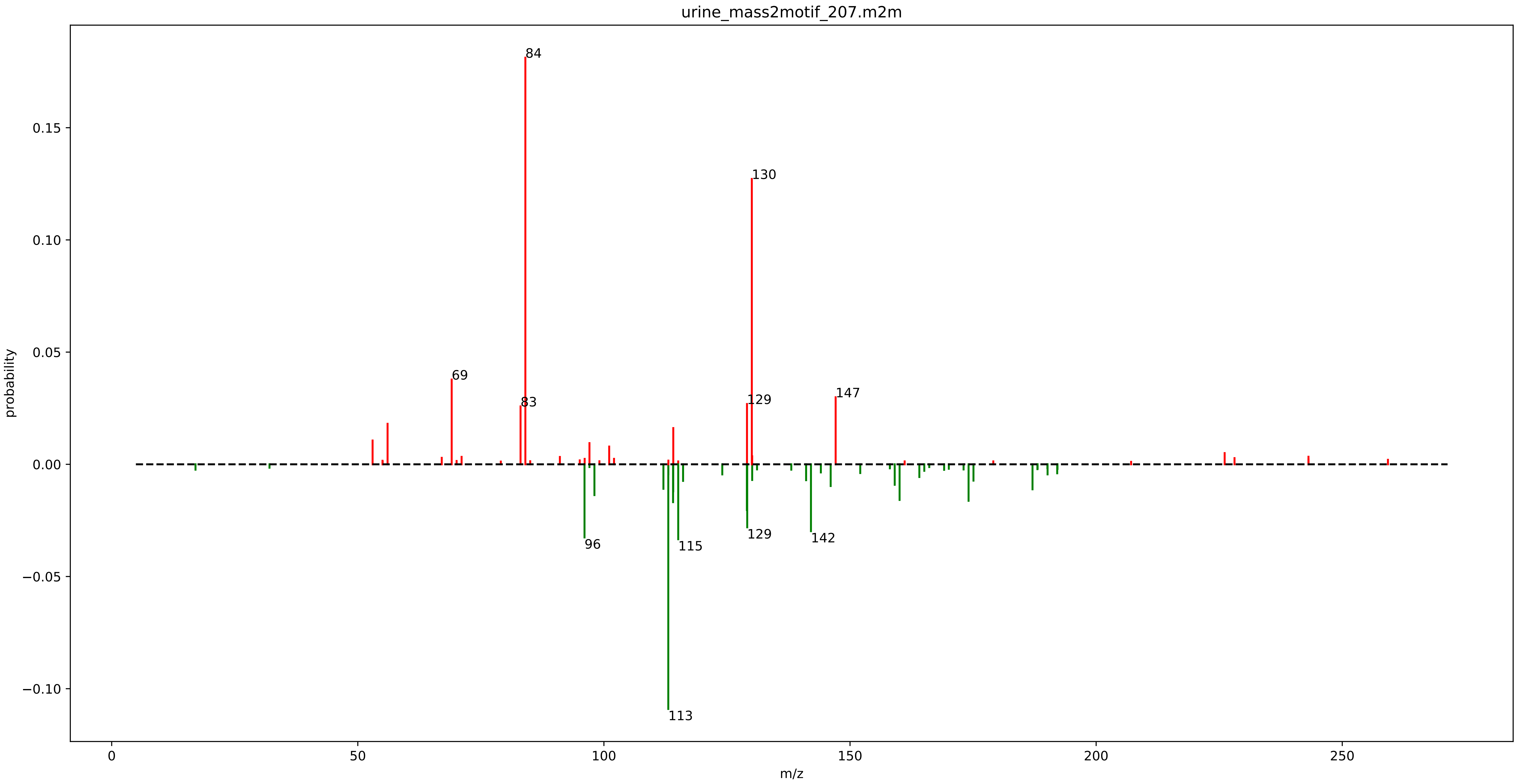
urine_mass2motif_207.m2m
probability
m/z
0.15
0.10
0.05
0.00
−0.05
−0.10
0
50
100
150
200
250
84
130
69
83
129
147
96
115
129
142
113

urine_mass2motif_207.m2m

fragment_84.0425	: 0.18
fragment_130.0475	: 0.13
loss_113.0775	: 0.11
fragment_69.0725	: 0.04
loss_115.0975	: 0.03
loss_96.0575	: 0.03
fragment_147.0775	: 0.03
loss_142.0625	: 0.03
loss_129.1125	: 0.03
fragment_129.0675	: 0.03
fragment_83.0625	: 0.03
loss_129.0425	: 0.02
fragment_56.0475	: 0.02
loss_114.0475	: 0.02
loss_174.0825	: 0.02
fragment_114.0925	: 0.02
loss_160.0675	: 0.02
loss_98.0725	: 0.01
loss_114.0825	: 0.01
loss_187.0725	: 0.01
loss_112.0875	: 0.01
fragment_53.0025	: 0.01
loss_146.0675	: 0.01
fragment_97.0625	: 0.01
loss_159.0775	: 0.01
fragment_101.0725	: 0.01
loss_116.0825	: 0.01
loss_175.0775	: 0.01
loss_141.0775	: 0.01
loss_130.1225	: 0.01
loss_164.0825	: 0.01

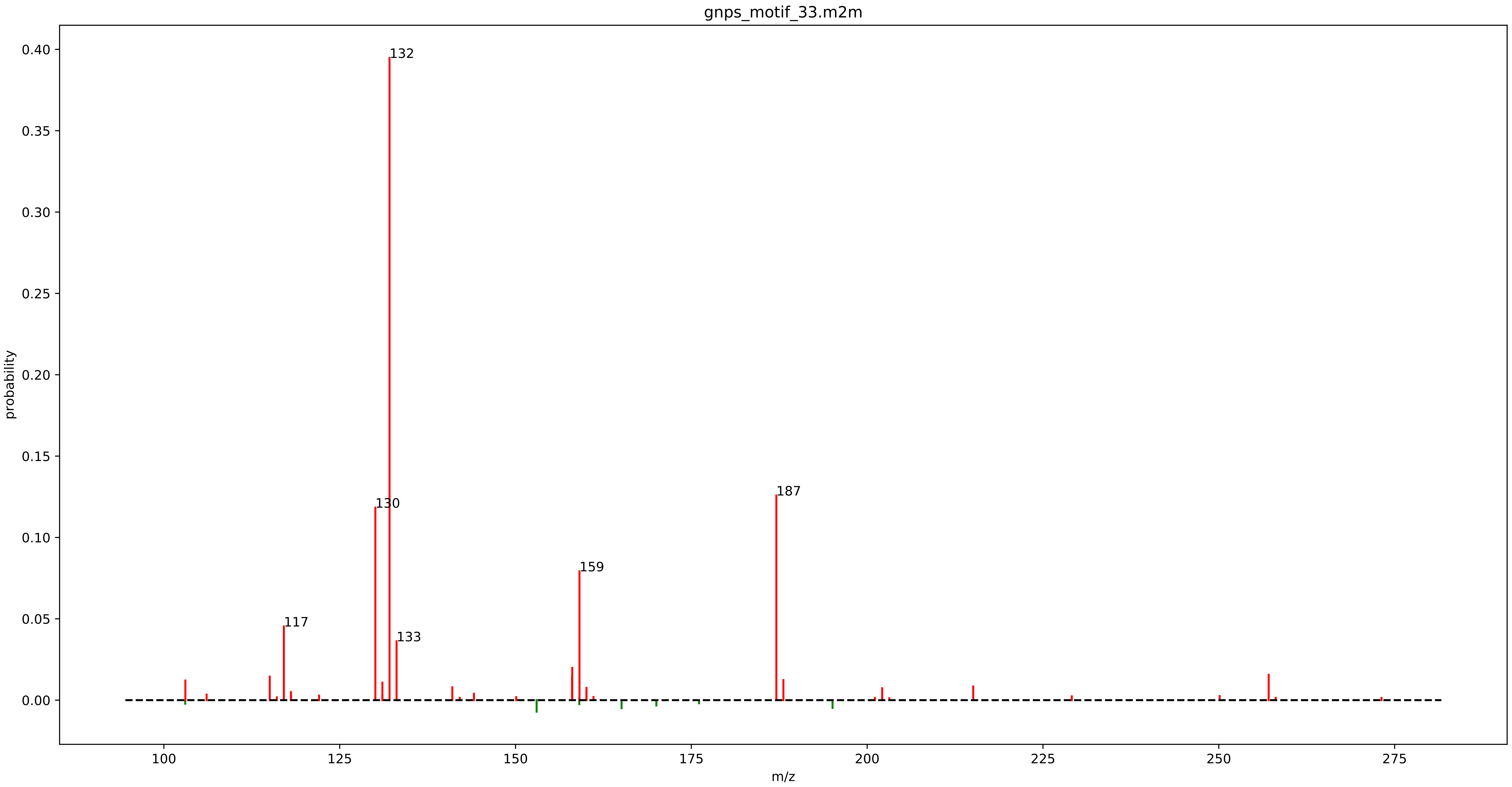

gnps_motif_33.m2m
probability
m/z
0.40
0.35
0.30
0.25
0.20
0.15
0.10
0.05
0.00
100
125
150
175
200
225
250
275
132
130
187
159
117
133

gnps_motif_33.m2m

fragment_132.0825	: 0.39
fragment_187.0875	: 0.13
fragment_130.0675	: 0.12
fragment_159.0925	: 0.08
fragment_117.0575	: 0.05
fragment_133.0825	: 0.04
fragment_158.0625	: 0.02
fragment_257.0925	: 0.02
fragment_115.0525	: 0.01
fragment_158.0375	: 0.01
fragment_188.0875	: 0.01
fragment_103.0525	: 0.01
fragment_131.0675	: 0.01
fragment_215.0825	: 0.01
fragment_141.0125	: 0.01
fragment_160.0925	: 0.01
fragment_202.1325	: 0.01
loss_152.9975	: 0.01
fragment_160.0775	: 0.01

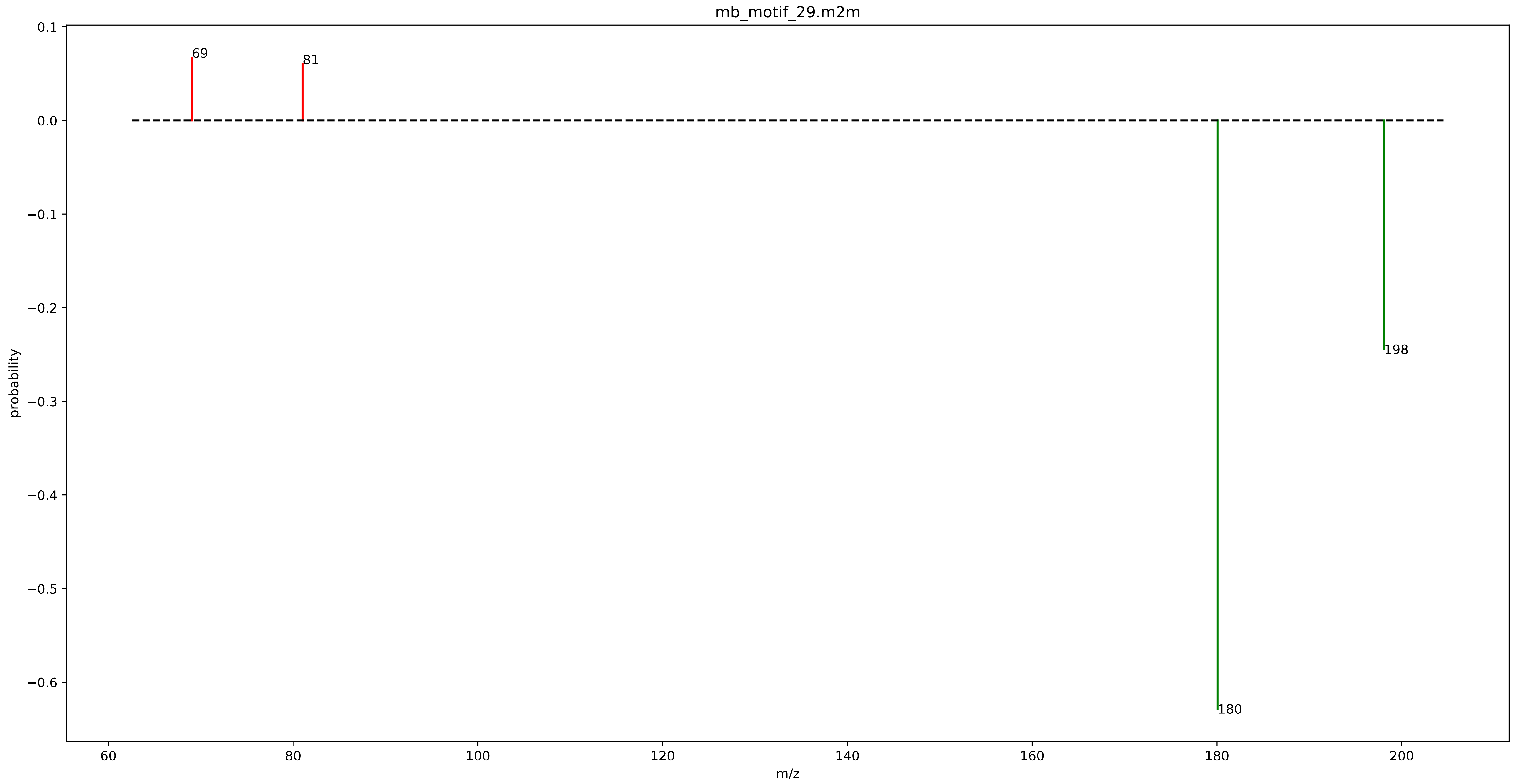
mb_motif_29.m2m
probability
m/z
69
81
180
198
0.1
0.0
−0.1
−0.2
−0.3
−0.4
−0.5
−0.6
60
80
100
120
140
160
180
200

mb_motif_29.m2m

loss_180.0625	: 0.63
loss_198.0775	: 0.24
fragment_69.0325	: 0.07
fragment_81.0325	: 0.06

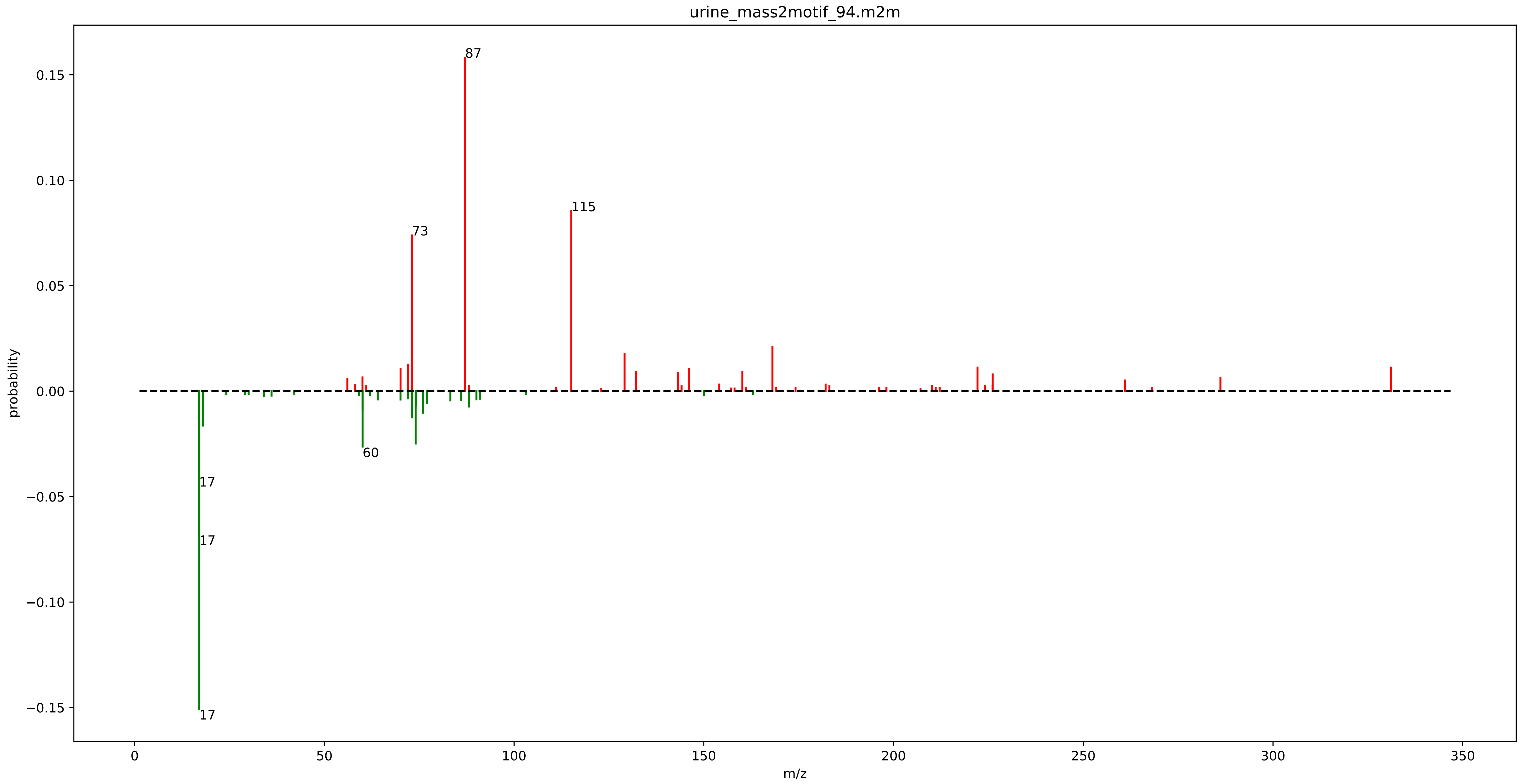

urine_mass2motif_94.m2m
probability
m/z
87
115
73
60
17
17
17
0.15
0.10
0.05
0.00
−0.05
−0.10
−0.15
0
50
100
150
200
250
300
350

urine_mass2motif_94.m2m

fragment_87.0975	: 0.16
loss_17.0175	: 0.15
fragment_115.0925	: 0.09
fragment_73.0825	: 0.07
loss_17.0225	: 0.07
loss_16.9775	: 0.04
loss_60.0775	: 0.03
loss_74.0575	: 0.02
fragment_168.0775	: 0.02
fragment_129.1125	: 0.02
loss_18.0675	: 0.02
fragment_72.0425	: 0.01
loss_73.0525	: 0.01
fragment_73.0475	: 0.01
fragment_222.1275	: 0.01
fragment_331.0925	: 0.01
fragment_70.0725	: 0.01
fragment_146.1375	: 0.01
loss_76.0625	: 0.01
fragment_87.0425	: 0.01
loss_18.0225	: 0.01
fragment_132.1225	: 0.01
fragment_160.1525	: 0.01
fragment_143.1275	: 0.01
loss_60.0675	: 0.01
fragment_226.1225	: 0.01
loss_74.0475	: 0.01
loss_88.0675	: 0.01
loss_74.0925	: 0.01
fragment_60.0425	: 0.01
fragment_286.1425	: 0.01
fragment_56.0575	: 0.01
loss_77.0625	: 0.01
fragment_73.0775	: 0.01
fragment_261.0525	: 0.01

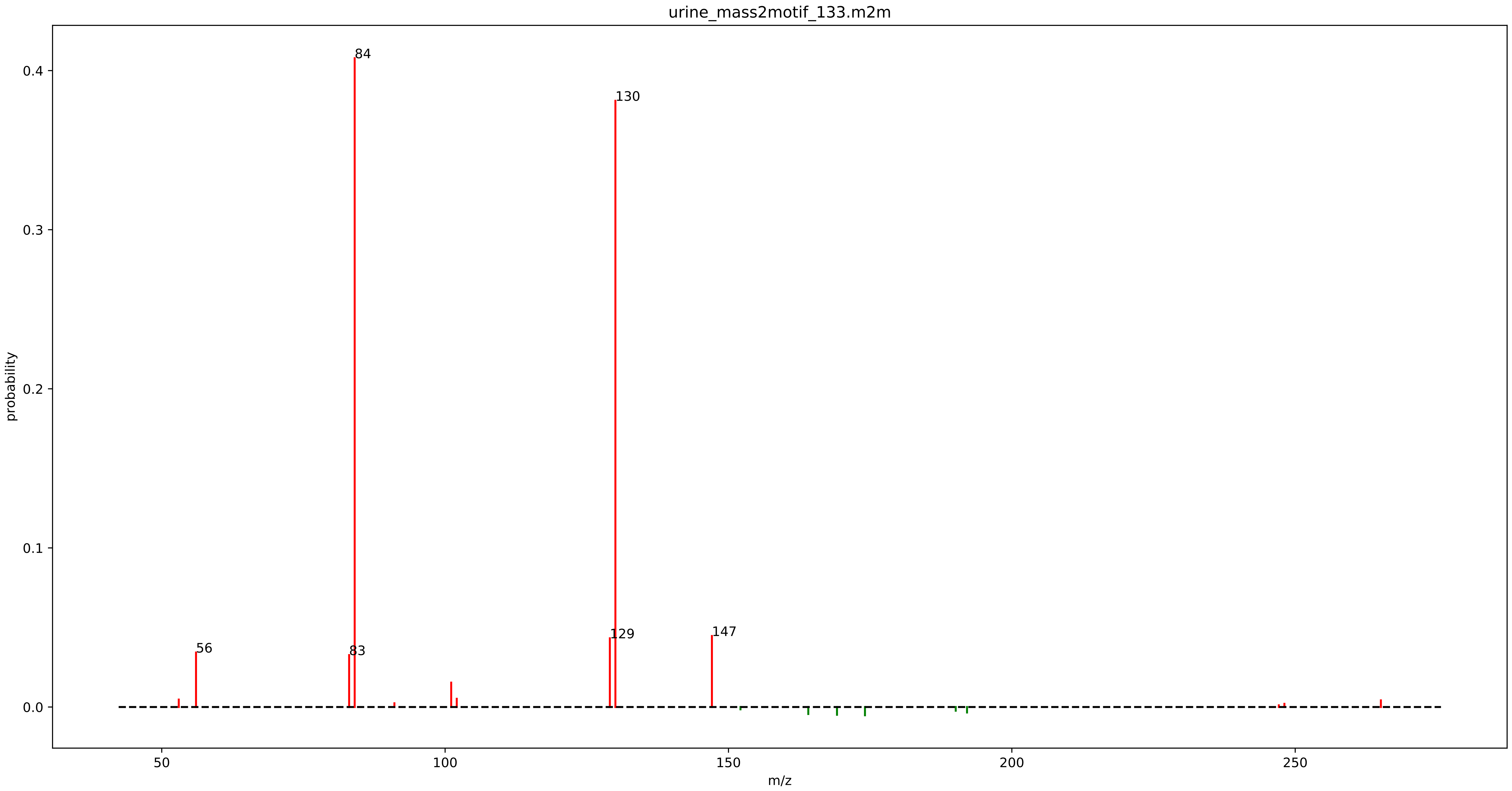
urine_mass2motif_133.m2m
probability
m/z
0.0
0.1
0.2
0.3
0.4
50
100
150
200
250
56
83
84
129
130
147

urine_mass2motif_133.m2m

fragment_84.0425	: 0.41
fragment_130.0475	: 0.38
fragment_147.0775	: 0.04
fragment_129.0675	: 0.04
fragment_56.0475	: 0.03
fragment_83.0625	: 0.03
fragment_101.0725	: 0.02
loss_174.0825	: 0.01
fragment_102.0525	: 0.01

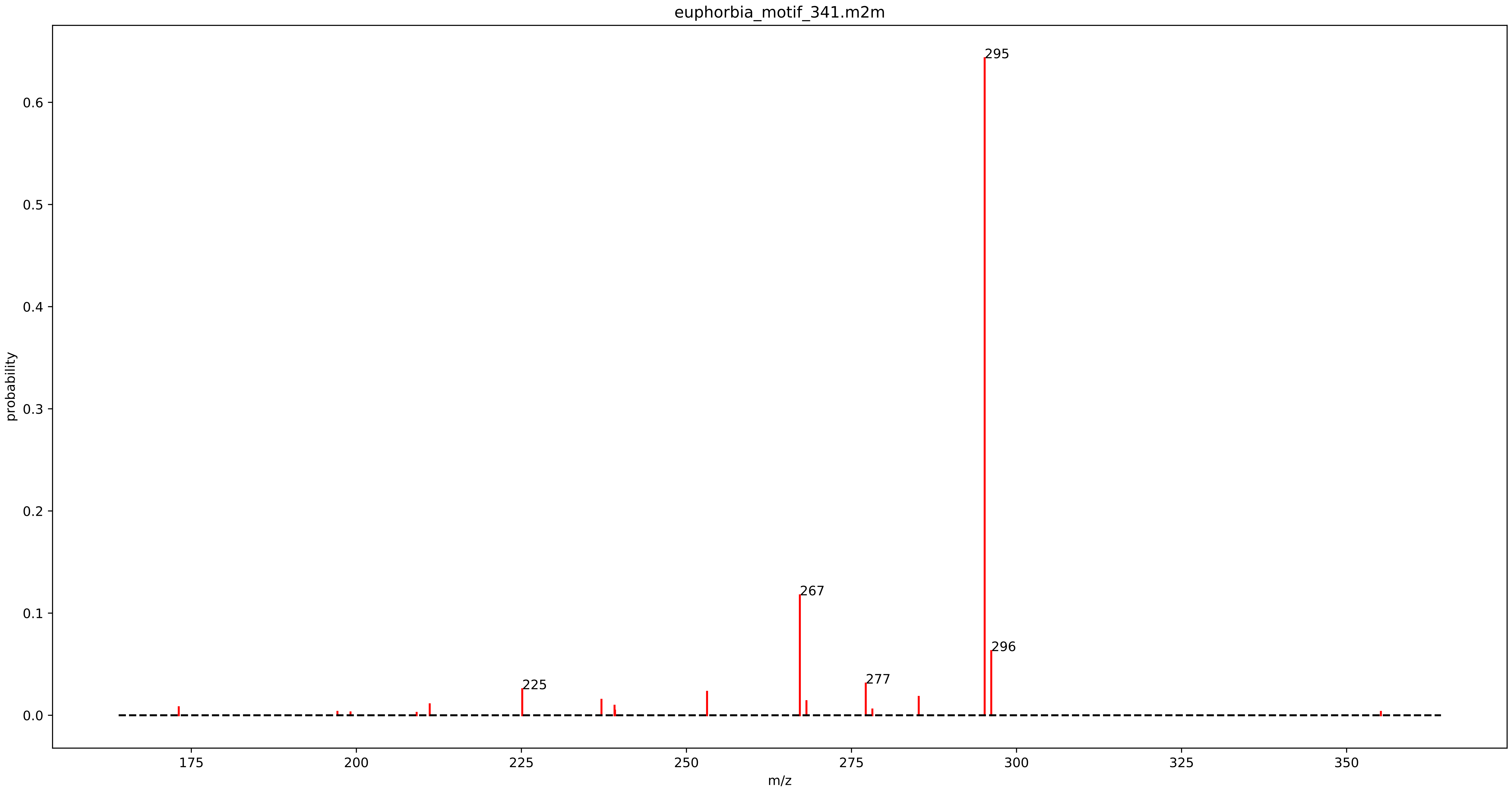

euphorbia_motif_341.m2m
probability
m/z
0.0
0.1
0.2
0.3
0.4
0.5
0.6
175
200
225
250
275
300
325
350
295
267
296
277
225

euphorbia_motif_341.m2m

fragment_295.1725	: 0.64
fragment_267.1775	: 0.12
fragment_296.1725	: 0.06
fragment_277.1625	: 0.03
fragment_225.1275	: 0.03
fragment_253.1225	: 0.02
fragment_285.1875	: 0.02
fragment_237.1275	: 0.02
fragment_268.1775	: 0.01
fragment_211.1125	: 0.01
fragment_239.1075	: 0.01
fragment_173.0975	: 0.01
fragment_278.1625	: 0.01

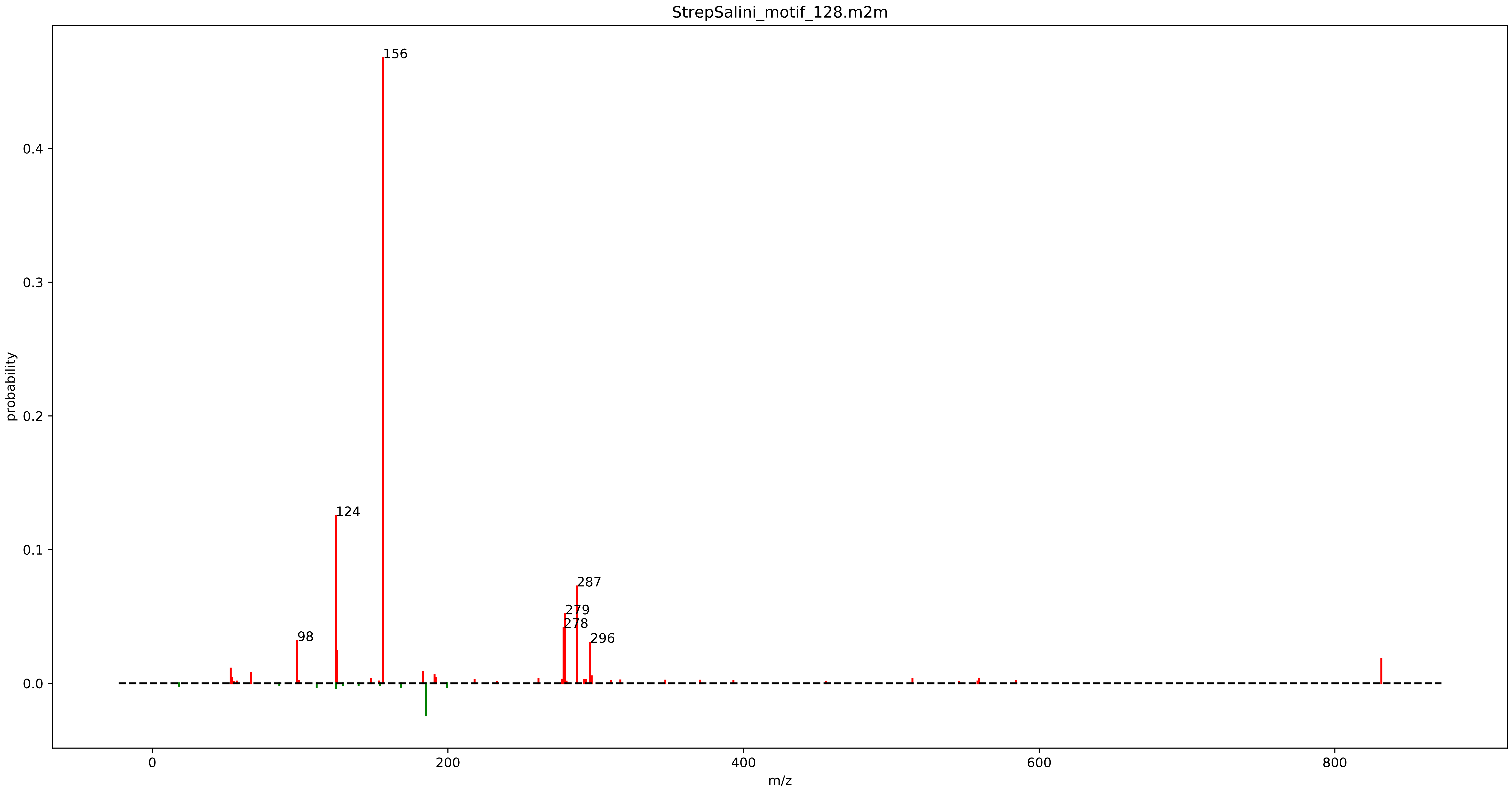

StrepSalini_motif_128.m2m
probability
m/z
156
124
98
287
279
278
296
0.0
0.1
0.2
0.3
0.4
0
200
400
600
800

StrepSalini_motif_128.m2m

fragment_156.0500	: 0.47
fragment_124.0500	: 0.13
fragment_287.1500	: 0.07
fragment_279.2525	: 0.05
fragment_278.2500	: 0.04
fragment_98.0500	: 0.03
fragment_296.2500	: 0.03
fragment_125.0500	: 0.02
loss_185.1500	: 0.02
fragment_831.4500	: 0.02
fragment_53.0500	: 0.01
fragment_183.0500	: 0.01
fragment_66.9500	: 0.01
fragment_190.9500	: 0.01
fragment_297.2500	: 0.01

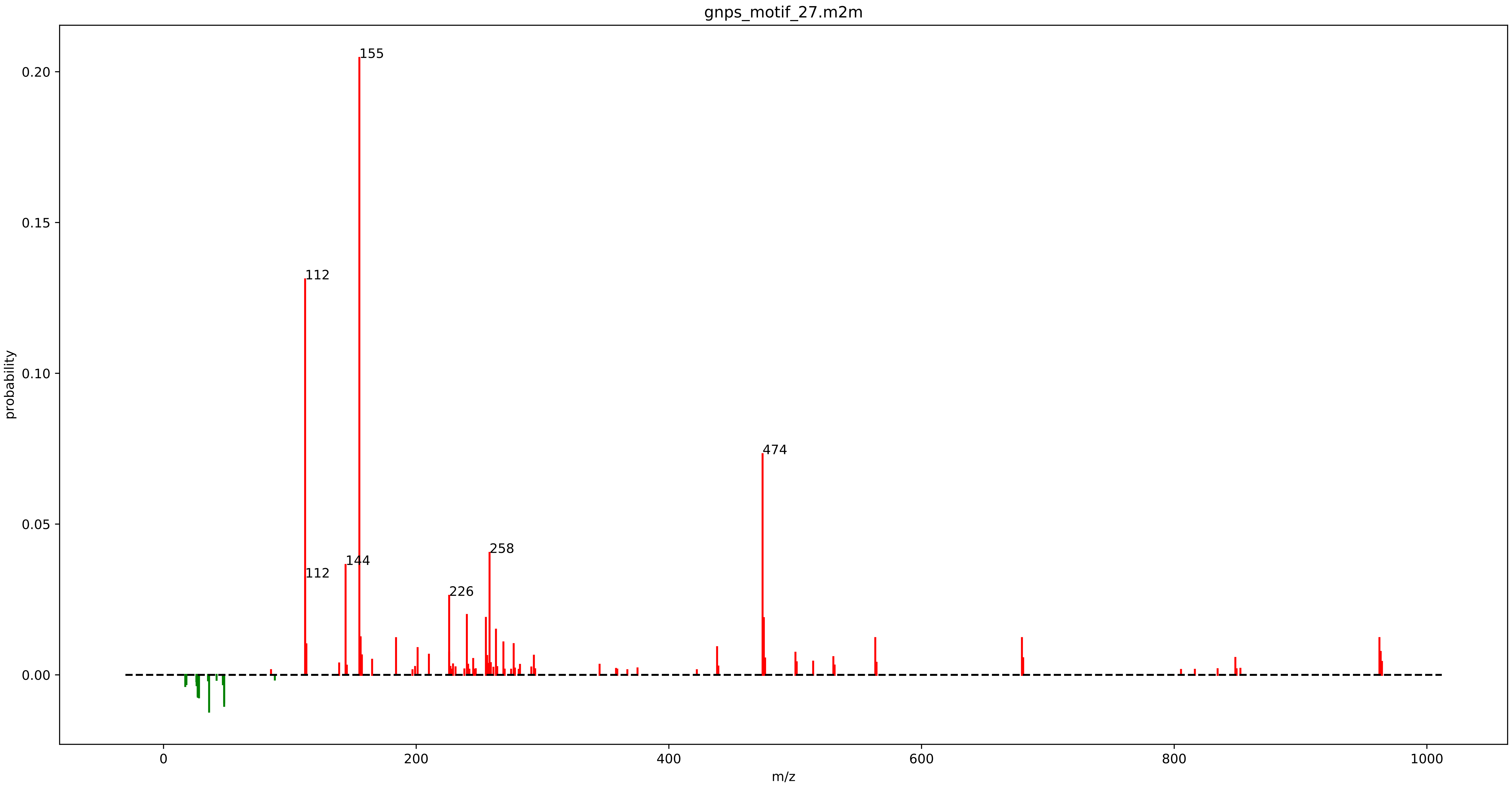

gnps_motif_27.m2m
probability
m/z
0.20
0.15
0.10
0.05
0.00
0
200
400
600
800
1000
155
112
474
258
144
112
226

gnps_motif_27.m2m

fragment_155.0175	: 0.20
fragment_112.0775	: 0.13
fragment_474.1725	: 0.07
fragment_258.0625	: 0.04
fragment_144.1025	: 0.04
fragment_112.1125	: 0.03
fragment_226.0875	: 0.03
fragment_240.1025	: 0.02
fragment_255.1725	: 0.02
fragment_475.1775	: 0.02
fragment_263.1425	: 0.01
fragment_156.0225	: 0.01
fragment_563.3775	: 0.01
fragment_679.4625	: 0.01
loss_36.0825	: 0.01
fragment_962.4425	: 0.01
fragment_184.0425	: 0.01
fragment_269.0275	: 0.01
loss_48.0025	: 0.01
fragment_277.1575	: 0.01
fragment_113.0775	: 0.01
fragment_438.1725	: 0.01
loss_48.0075	: 0.01
fragment_201.1275	: 0.01
fragment_963.4475	: 0.01
loss_28.0975	: 0.01
fragment_226.0925	: 0.01
fragment_500.1825	: 0.01
loss_27.1375	: 0.01
loss_27.0075	: 0.01
fragment_210.0575	: 0.01
fragment_157.0125	: 0.01
fragment_293.1525	: 0.01
loss_28.0075	: 0.01
fragment_256.1725	: 0.01
fragment_530.1925	: 0.01
fragment_848.3925	: 0.01
fragment_680.4525	: 0.01
fragment_476.1775	: 0.01
fragment_245.1125	: 0.01
fragment_165.1025	: 0.01

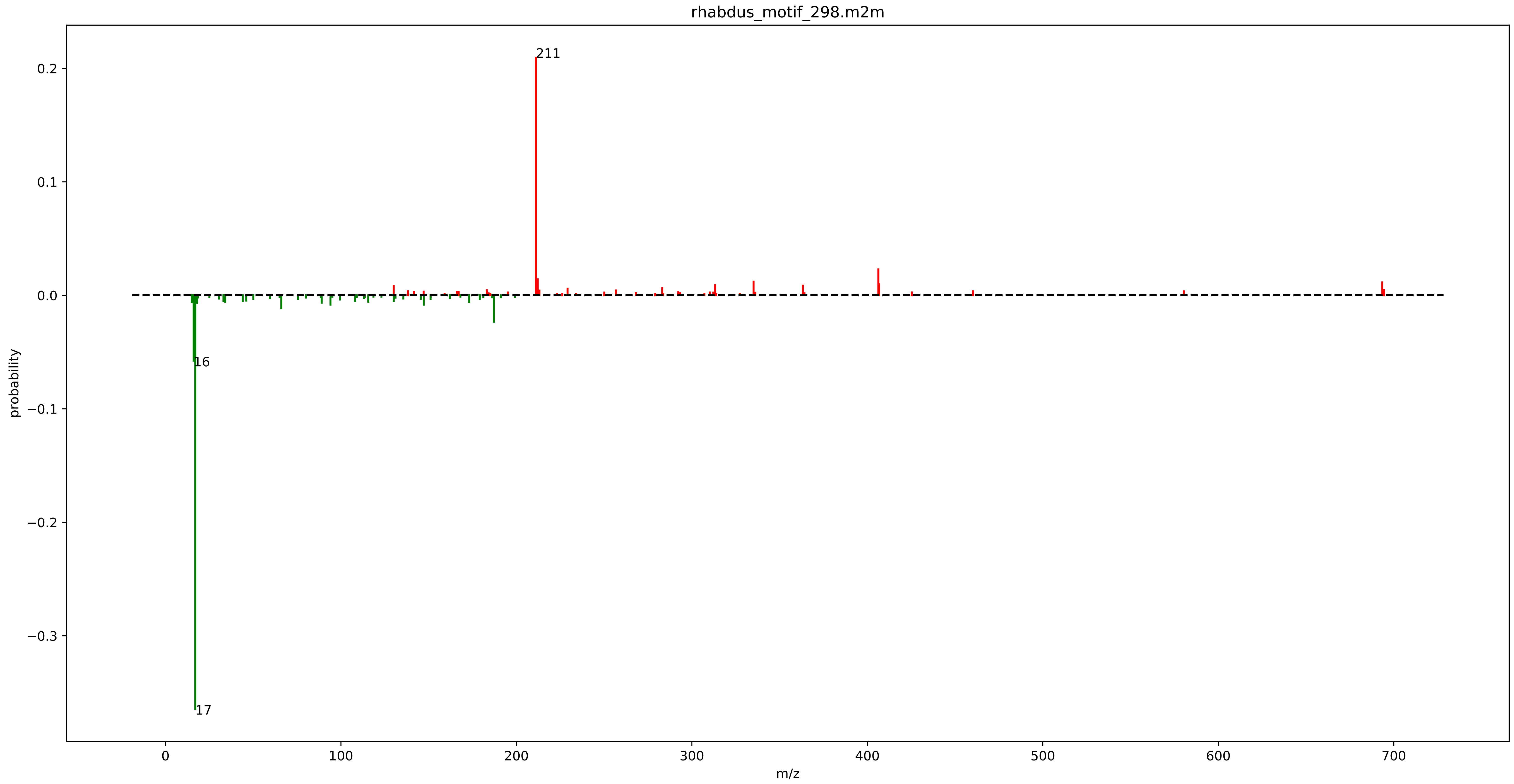

rhabdus_motif_298.m2m
211
16
17
0.2
0.1
0.0
−0.1
−0.2
−0.3
probability
0
100
200
300
400
500
600
700
m/z

rhabdus_motif_298.m2m

loss_17.0275	: 0.36
fragment_211.1425	: 0.21
loss_16.0225	: 0.06
loss_187.1425	: 0.02
fragment_406.2675	: 0.02
fragment_212.1475	: 0.01
fragment_335.1475	: 0.01
loss_66.0275	: 0.01
fragment_693.3975	: 0.01
fragment_406.7675	: 0.01
fragment_313.2275	: 0.01
fragment_363.1425	: 0.01
fragment_130.0475	: 0.01
loss_147.1225	: 0.01
loss_94.0225	: 0.01
loss_18.0325	: 0.01
loss_89.0025	: 0.01
fragment_283.1425	: 0.01
loss_15.0175	: 0.01
loss_173.0975	: 0.01
fragment_229.1275	: 0.01
loss_34.0525	: 0.01
loss_115.5925	: 0.01
loss_44.0425	: 0.01
loss_108.0375	: 0.01

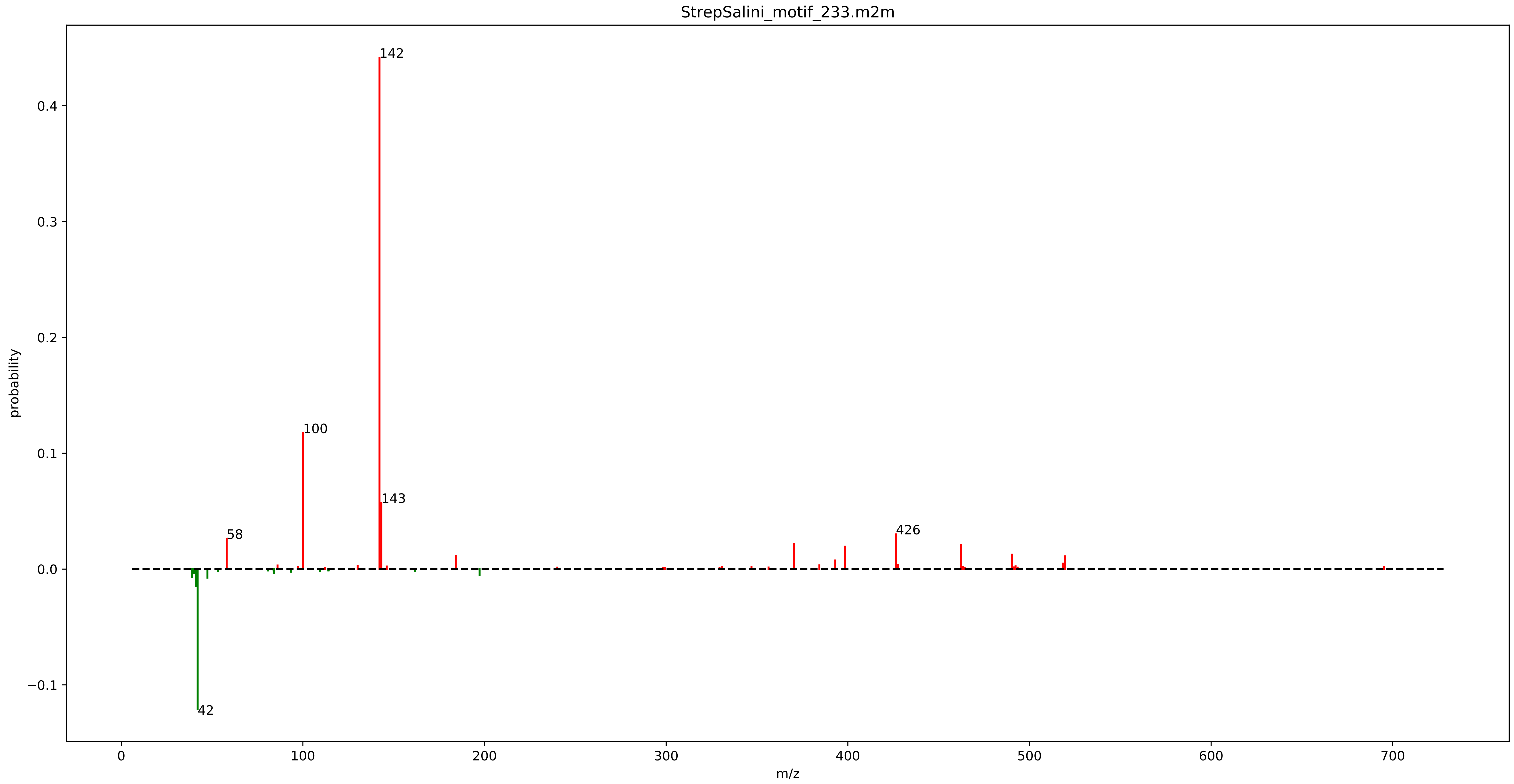

StrepSalini_motif_233.m2m
probability
m/z
142
100
143
58
426
42
0.4
0.3
0.2
0.1
0.0
−0.1
0
100
200
300
400
500
600
700

StrepSalini_motif_233.m2m

fragment_142.1500	: 0.44
loss_42.0500	: 0.12
fragment_100.1500	: 0.12
fragment_143.1525	: 0.06
fragment_426.4500	: 0.03
fragment_58.0500	: 0.03
fragment_370.3500	: 0.02
fragment_462.3500	: 0.02
fragment_398.3525	: 0.02
loss_41.0500	: 0.01
fragment_490.3525	: 0.01
fragment_184.1500	: 0.01
fragment_519.4500	: 0.01
loss_47.4500	: 0.01
fragment_393.0500	: 0.01
loss_38.8500	: 0.01
fragment_490.4500	: 0.01
loss_197.2525	: 0.01

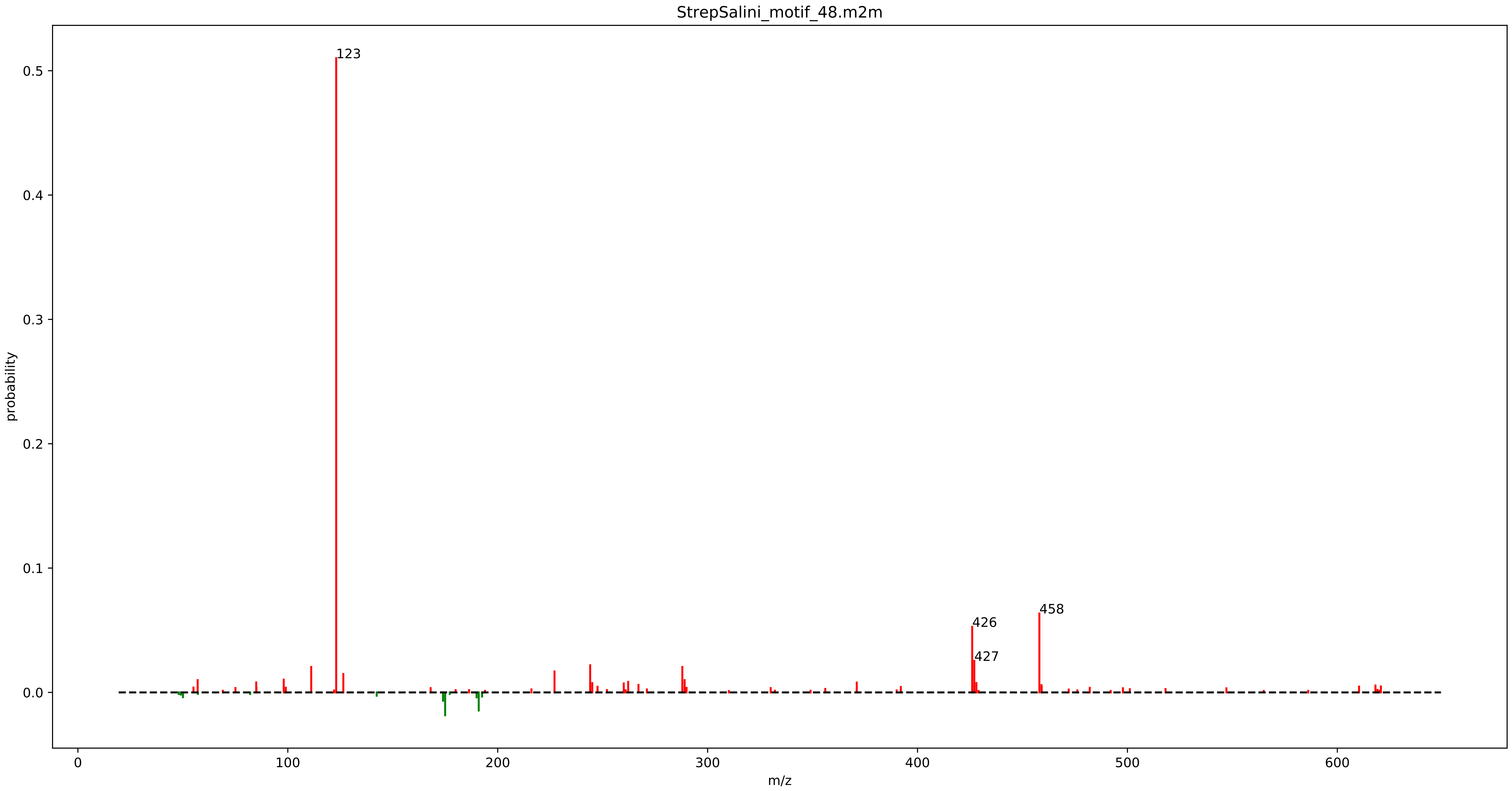
StrepSalini_motif_48.m2m
probability
m/z
123
426
427
458
0.0
0.1
0.2
0.3
0.4
0.5
0
100
200
300
400
500
600

StrepSalini_motif_48.m2m

fragment_123.0500	: 0.51
fragment_458.0500	: 0.06
fragment_426.0500	: 0.05
fragment_427.0500	: 0.03
fragment_244.0500	: 0.02
fragment_287.9500	: 0.02
fragment_111.1500	: 0.02
loss_174.9500	: 0.02
fragment_227.0500	: 0.02
fragment_126.4500	: 0.01
loss_190.9500	: 0.01
fragment_98.0500	: 0.01
fragment_57.0500	: 0.01
fragment_289.0500	: 0.01
fragment_262.1500	: 0.01
fragment_84.9500	: 0.01
fragment_371.0500	: 0.01
fragment_428.0500	: 0.01
fragment_245.0525	: 0.01
fragment_260.0500	: 0.01
loss_173.9500	: 0.01
fragment_267.0500	: 0.01
fragment_459.0500	: 0.01
fragment_618.1500	: 0.01

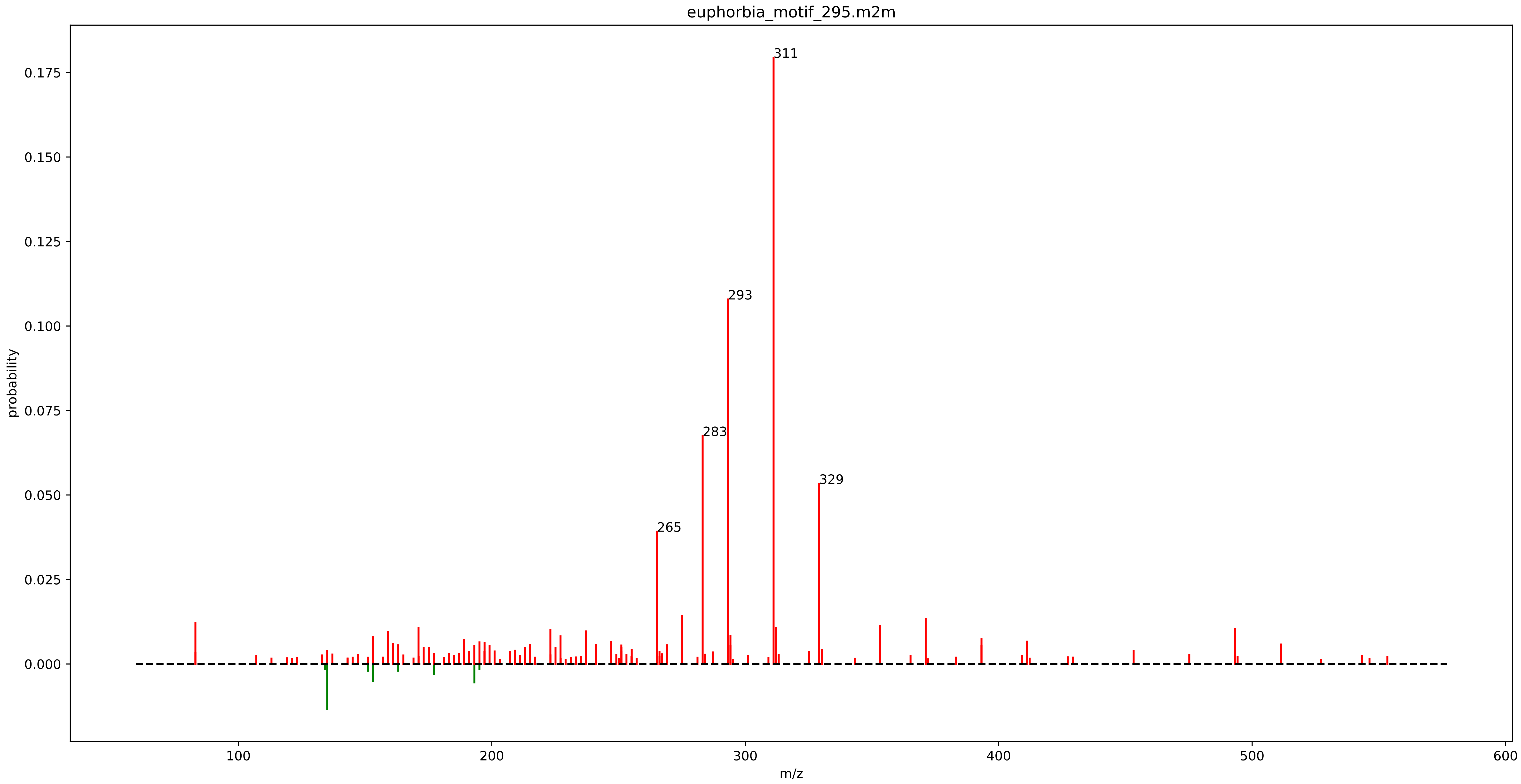
euphorbia_motif_295.m2m
probability
m/z
0.175
0.150
0.125
0.100
0.075
0.050
0.025
0.000
100
200
300
400
500
600
311
293
283
329
265

euphorbia_motif_295.m2m

fragment_311.1675	: 0.18
fragment_293.1575	: 0.11
fragment_283.1725	: 0.07
fragment_329.1775	: 0.05
fragment_265.1625	: 0.04
fragment_265.1575	: 0.01
fragment_275.1425	: 0.01
loss_135.0875	: 0.01
fragment_371.1875	: 0.01
fragment_83.0475	: 0.01
fragment_353.1775	: 0.01
fragment_171.0825	: 0.01
fragment_312.1675	: 0.01
fragment_493.2625	: 0.01
fragment_312.1725	: 0.01
fragment_223.1125	: 0.01
fragment_237.1275	: 0.01
fragment_159.0825	: 0.01
fragment_294.1575	: 0.01
fragment_227.1075	: 0.01
fragment_153.0925	: 0.01
fragment_393.2125	: 0.01
fragment_189.0925	: 0.01
fragment_237.1675	: 0.01
fragment_411.2225	: 0.01
fragment_247.1525	: 0.01
fragment_195.1175	: 0.01
fragment_197.1325	: 0.01
fragment_411.2175	: 0.01
fragment_161.0975	: 0.01
fragment_511.3425	: 0.01
fragment_241.1225	: 0.01
fragment_215.1075	: 0.01
fragment_163.0775	: 0.01
fragment_269.1575	: 0.01
fragment_251.1075	: 0.01
loss_193.1275	: 0.01
fragment_193.0875	: 0.01
fragment_294.1625	: 0.01
fragment_199.1125	: 0.01
fragment_393.2075	: 0.01
fragment_247.1475	: 0.01
loss_153.0975	: 0.01

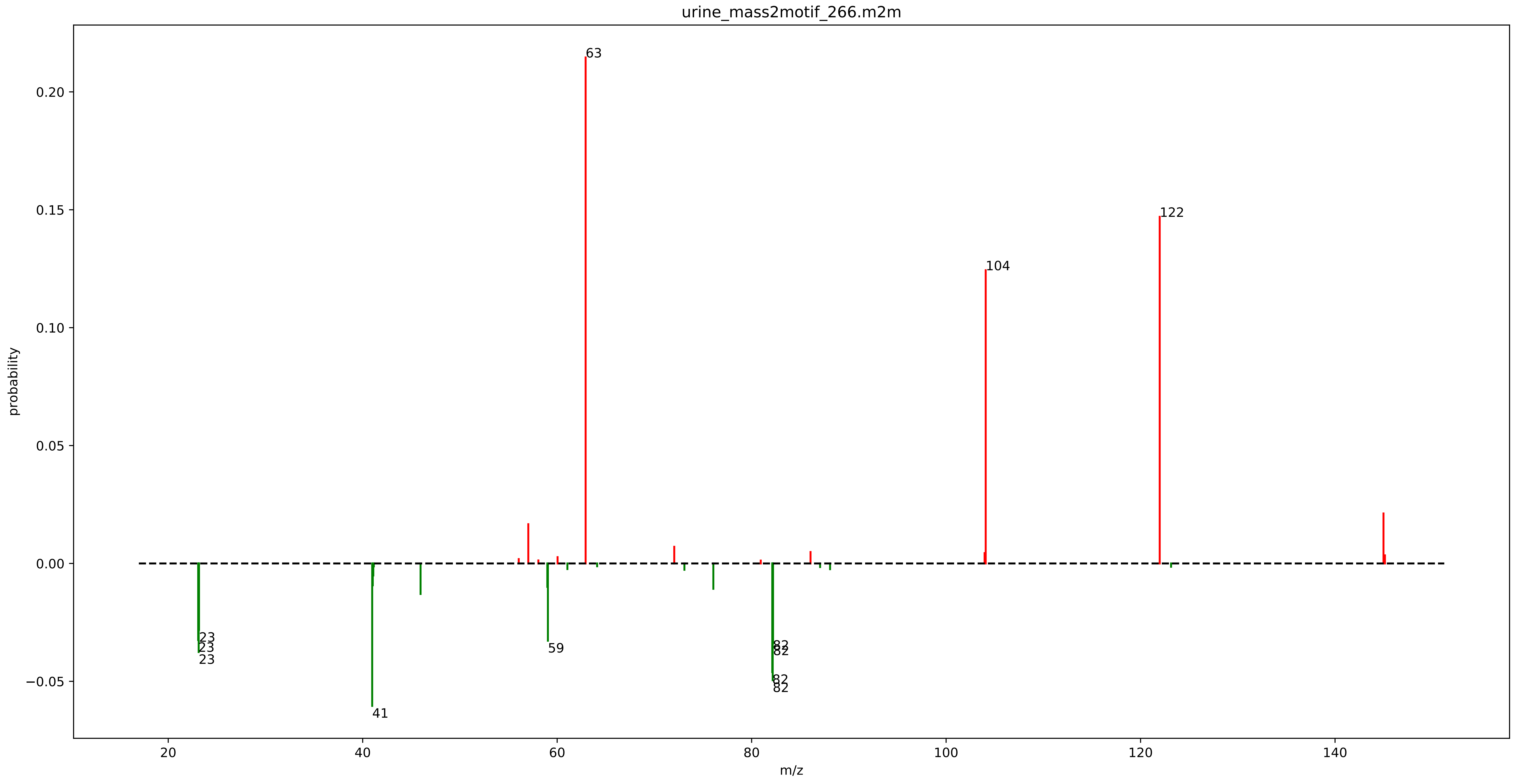

urine_mass2motif_266.m2m
probability
m/z
0.20
0.15
0.10
0.05
0.00
−0.05
20
40
60
80
100
120
140
63
122
104
23
23
23
41
59
82
82
82
82

urine_mass2motif_266.m2m

fragment_62.9275 : 0.21
fragment_121.9675 : 0.15
fragment_104.0725 : 0.12
loss_40.9775 : 0.06
loss_82.1675 : 0.05
loss_82.1225 : 0.05
loss_23.1325 : 0.04
loss_82.2025 : 0.03
loss_59.0475 : 0.03
loss_23.0825 : 0.03
loss_82.1775 : 0.03
loss_23.1675 : 0.03
loss_23.1425 : 0.02
fragment_144.9825 : 0.02
fragment_57.0325 : 0.02
loss_45.9525 : 0.01
loss_76.0625 : 0.01
loss_58.9875 : 0.01
loss_41.0375 : 0.01
fragment_72.0425 : 0.01
loss_82.1375 : 0.01
loss_41.0925 : 0.01

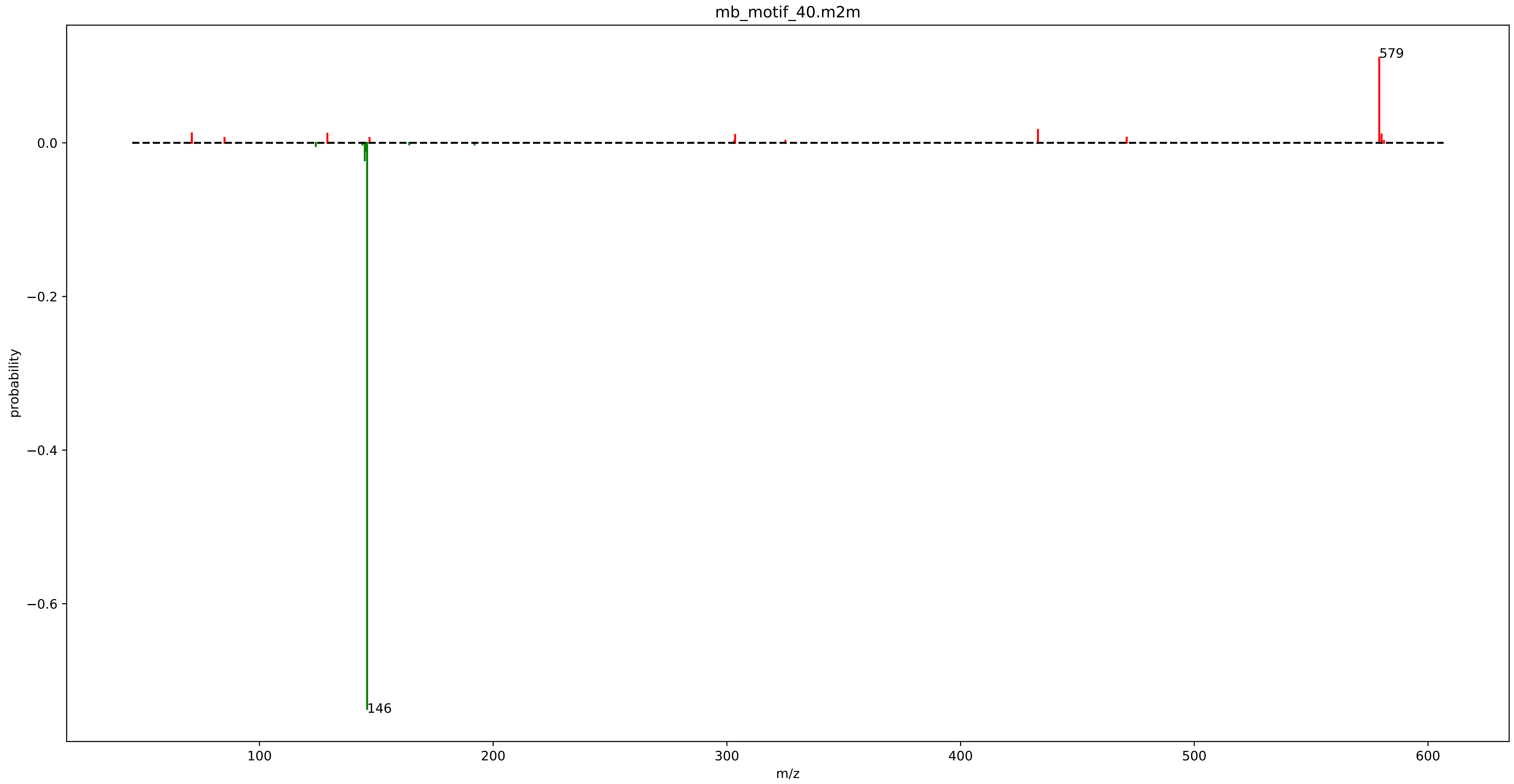

mb_motif_40.m2m
579
146
0.0
−0.2
−0.4
−0.6
probability
100
200
300
400
500
600
m/z

mb_motif_40.m2m

loss_146.0575	: 0.74
fragment_579.1725	: 0.11
loss_145.0525	: 0.02
fragment_433.1075	: 0.02
fragment_71.0525	: 0.01
fragment_129.0525	: 0.01
fragment_580.1725	: 0.01
loss_145.5875	: 0.01
fragment_303.5225	: 0.01
fragment_471.0925	: 0.01
fragment_147.0675	: 0.01
fragment_85.0225	: 0.01

gnps_motif_62.m2m

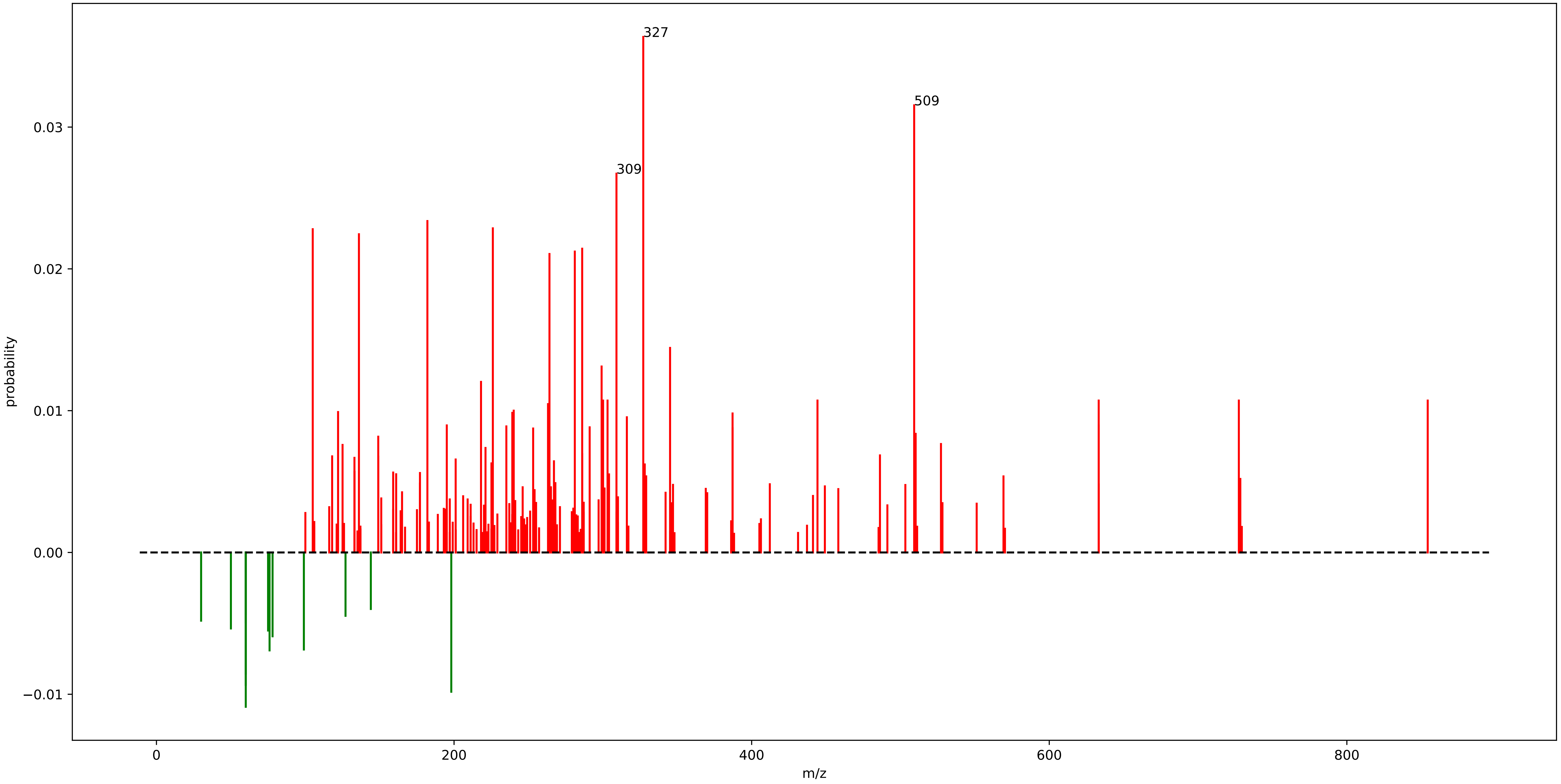

gnps_motif_62.m2m

fragment_327.1575	: 0.04
fragment_509.2175	: 0.03
fragment_309.1475	: 0.03
fragment_182.0825	: 0.02
fragment_226.0725	: 0.02
fragment_105.0325	: 0.02
fragment_136.0775	: 0.02
fragment_286.1075	: 0.02
fragment_281.1525	: 0.02
fragment_264.1225	: 0.02
fragment_345.1725	: 0.01
fragment_299.1625	: 0.01
fragment_218.1175	: 0.01
loss_60.0225	: 0.01
fragment_633.2275	: 0.01
fragment_727.4075	: 0.01
fragment_300.1575	: 0.01
fragment_444.2375	: 0.01
fragment_854.3375	: 0.01
fragment_303.1525	: 0.01
fragment_263.1425	: 0.01
fragment_240.1025	: 0.01
fragment_122.0575	: 0.01
fragment_239.1425	: 0.01
loss_198.1375	: 0.01
fragment_387.1675	: 0.01
fragment_316.1325	: 0.01
loss_60.0125	: 0.01
fragment_195.1175	: 0.01
fragment_633.2425	: 0.01
fragment_235.1475	: 0.01
fragment_291.1375	: 0.01
fragment_387.1825	: 0.01
fragment_253.1575	: 0.01
loss_60.0325	: 0.01
fragment_510.2175	: 0.01
fragment_149.0575	: 0.01
fragment_527.2275	: 0.01
fragment_125.0725	: 0.01
fragment_221.1325	: 0.01
fragment_286.1525	: 0.01
loss_76.0175	: 0.01
fragment_486.2225	: 0.01
loss_99.0725	: 0.01
fragment_118.0675	: 0.01
fragment_149.0975	: 0.01
fragment_133.0625	: 0.01
fragment_201.0825	: 0.01
fragment_267.1375	: 0.01
fragment_225.1275	: 0.01
fragment_328.1625	: 0.01
loss_78.0325	: 0.01
fragment_133.1025	: 0.01
fragment_159.0875	: 0.01
fragment_177.0925	: 0.01
fragment_161.0975	: 0.01
fragment_304.1525	: 0.01
loss_75.0125	: 0.01
fragment_329.1275	: 0.01
fragment_569.2375	: 0.01
loss_50.0375	: 0.01
fragment_728.4075	: 0.01

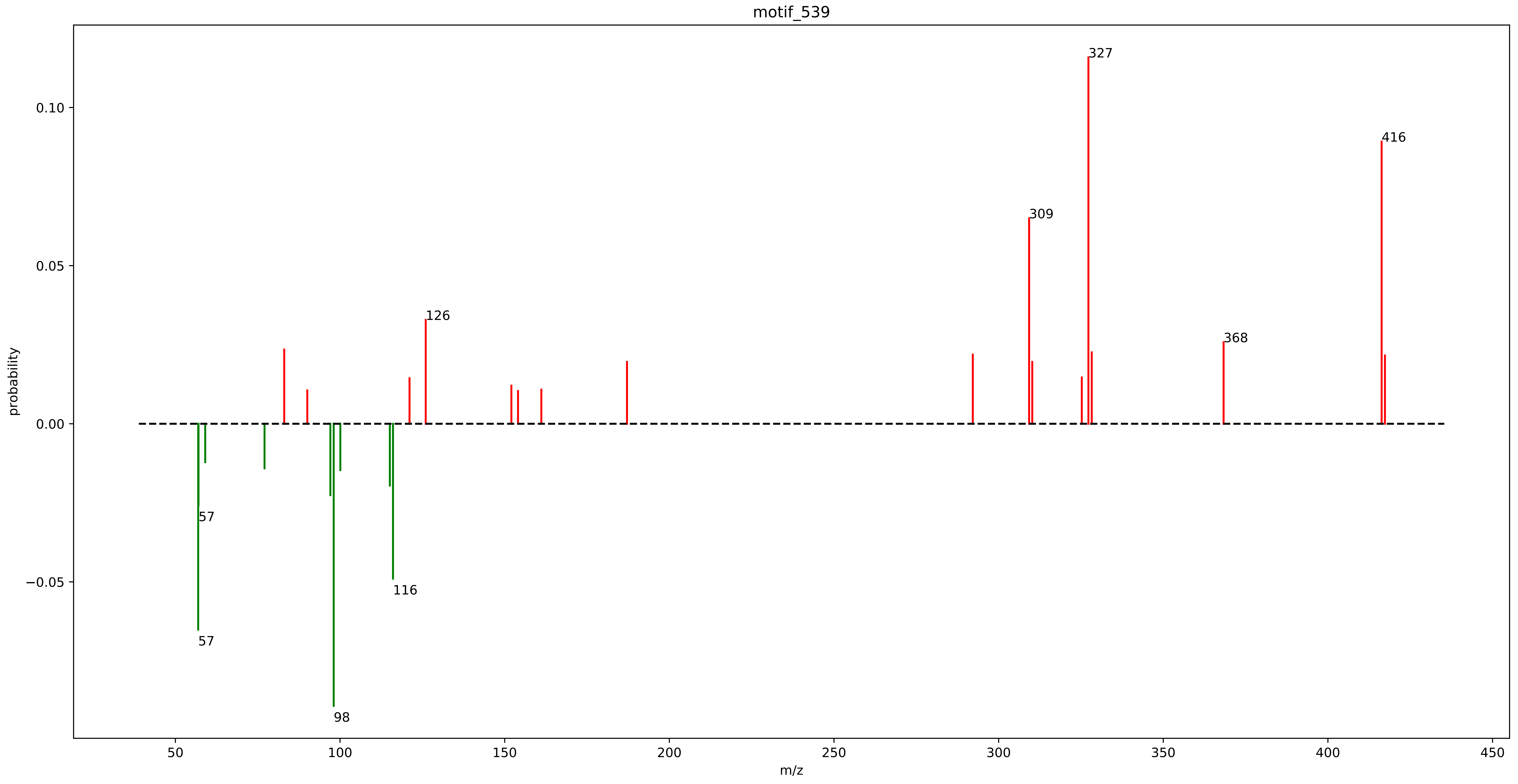

motif_539
probability
m/z
0.10
0.05
0.00
−0.05
50
100
150
200
250
300
350
400
450
327
416
309
126
368
57
57
116
98

motif_539

fragment_327.2675	: 0.12
loss_98.0725	: 0.09
fragment_416.3125	: 0.09
loss_56.9225	: 0.07
fragment_309.2575	: 0.06
loss_116.0825	: 0.05
fragment_126.0175	: 0.03
fragment_368.3175	: 0.03
loss_57.0225	: 0.03
fragment_83.0475	: 0.02
fragment_328.2725	: 0.02
loss_97.0675	: 0.02
fragment_292.1575	: 0.02
fragment_417.3175	: 0.02
fragment_310.2125	: 0.02
loss_115.1275	: 0.02
fragment_187.1475	: 0.02
loss_100.0875	: 0.01
fragment_325.2525	: 0.01
fragment_121.0975	: 0.01
loss_77.0675	: 0.01
loss_59.0575	: 0.01
fragment_152.0325	: 0.01
fragment_161.1325	: 0.01
fragment_90.0575	: 0.01
fragment_154.0475	: 0.01

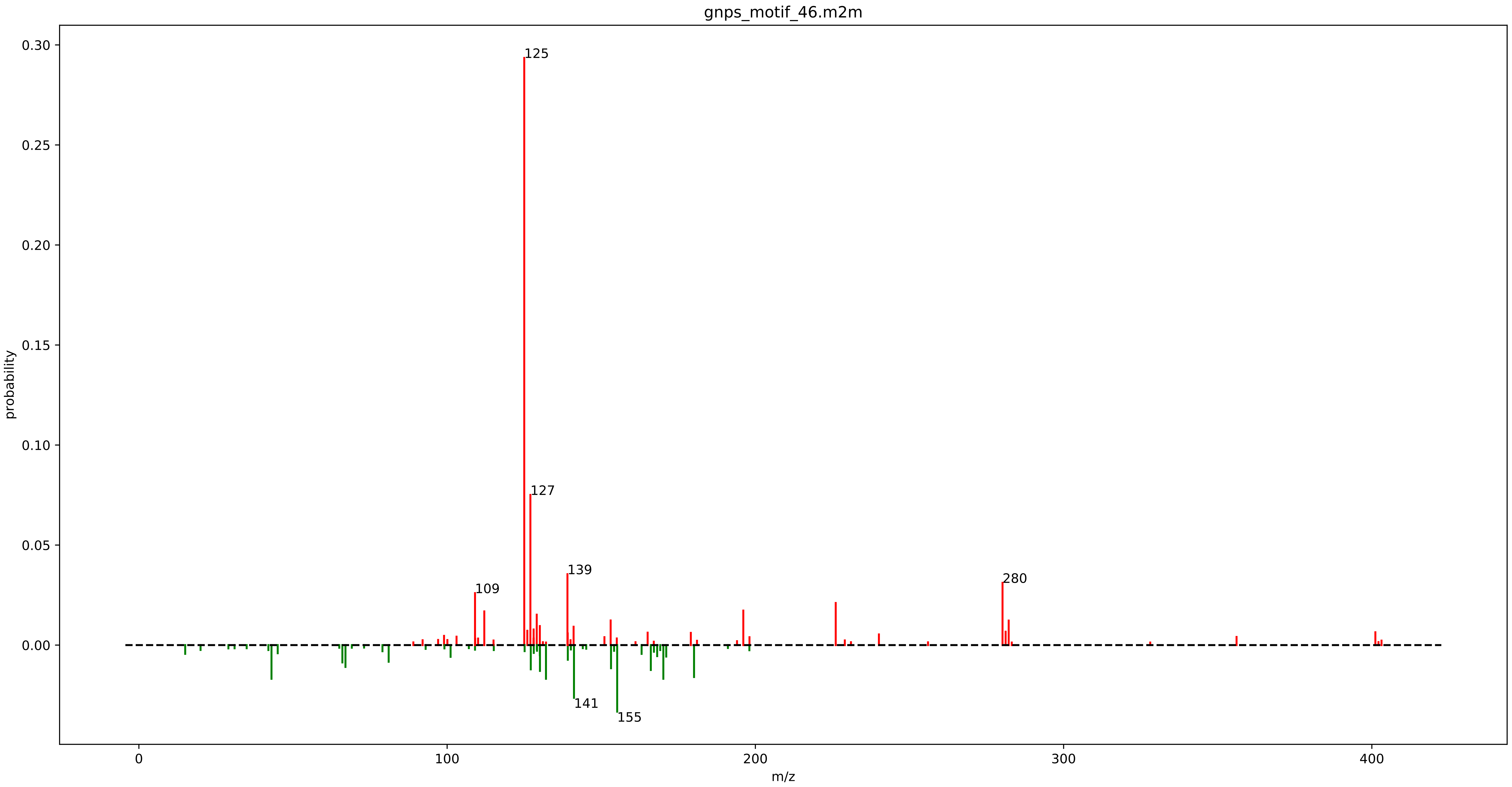
gnps_motif_46.m2m
probability
m/z
0.30
0.25
0.20
0.15
0.10
0.05
0.00
0
100
200
300
400
125
127
139
109
280
141
155

gnps_motif_46.m2m

fragment_125.0175	: 0.29
fragment_127.0125	: 0.08
fragment_139.0325	: 0.04
loss_155.1675	: 0.03
fragment_280.1825	: 0.03
loss_141.1525	: 0.03
fragment_109.0475	: 0.03
fragment_226.0675	: 0.02
fragment_196.0875	: 0.02
loss_132.0675	: 0.02
fragment_112.0575	: 0.02
loss_43.0175	: 0.02
loss_170.1425	: 0.02
loss_180.1025	: 0.02
fragment_129.0675	: 0.02
loss_130.1125	: 0.01
loss_166.0825	: 0.01
fragment_153.0475	: 0.01
fragment_282.1825	: 0.01
loss_127.1375	: 0.01
loss_153.1675	: 0.01
loss_67.0175	: 0.01
fragment_130.0775	: 0.01
fragment_141.0275	: 0.01
loss_66.0125	: 0.01
loss_81.0325	: 0.01
fragment_128.0625	: 0.01
loss_139.1525	: 0.01
fragment_126.0175	: 0.01
fragment_281.1875	: 0.01
fragment_401.1425	: 0.01
fragment_165.0475	: 0.01
fragment_179.0625	: 0.01
loss_101.1225	: 0.01
fragment_139.1225	: 0.01
loss_171.0825	: 0.01
loss_168.1425	: 0.01
fragment_240.0775	: 0.01
loss_130.0725	: 0.01

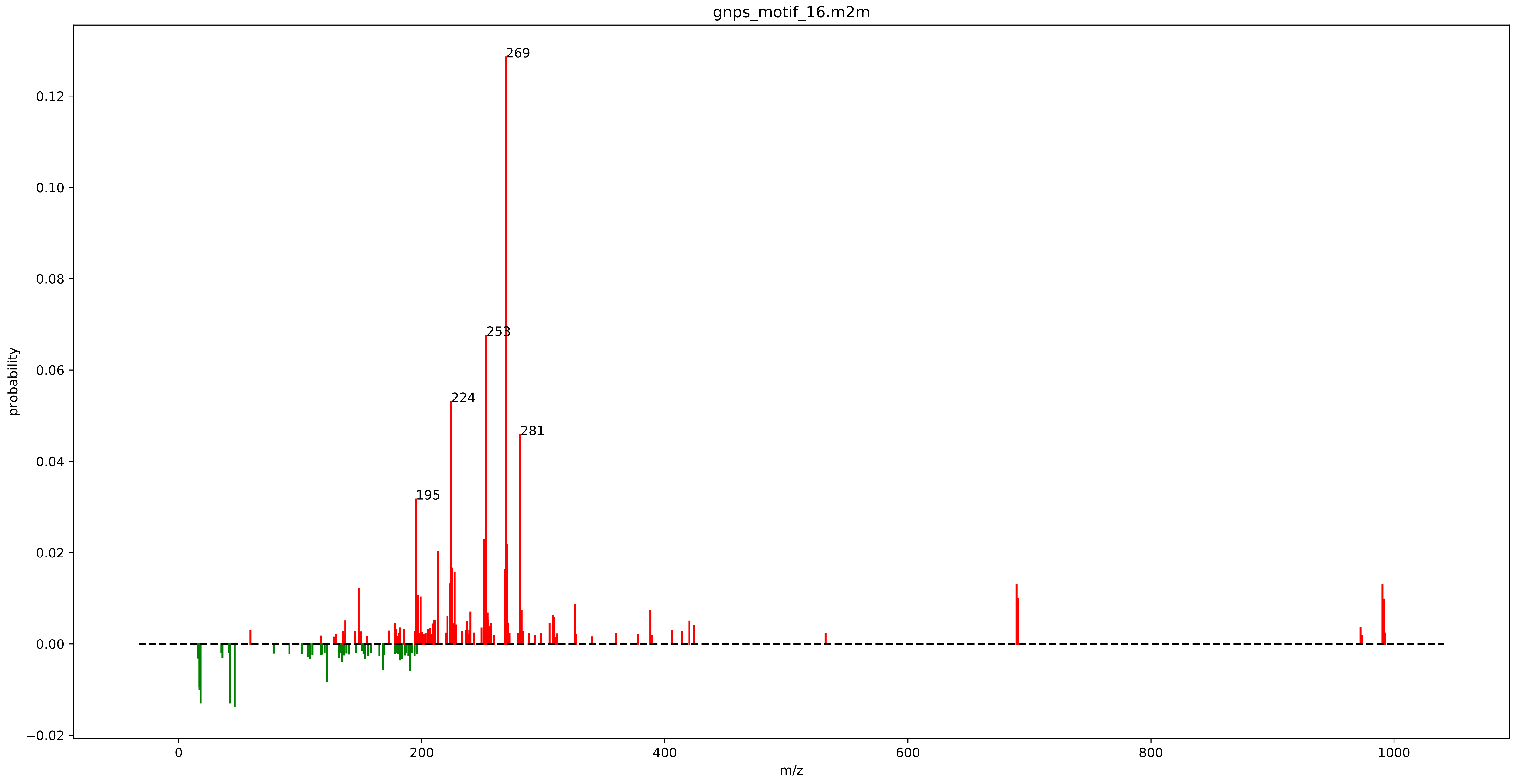

gnps_motif_16.m2m
probability
m/z
269
253
224
281
195
0.12
0.10
0.08
0.06
0.04
0.02
0.00
−0.02
0
200
400
600
800
1000

gnps_motif_16.m2m

fragment_269.0825	: 0.13
fragment_253.0875	: 0.07
fragment_224.0825	: 0.05
fragment_281.0825	: 0.05
fragment_195.0825	: 0.03
fragment_251.0725	: 0.02
fragment_270.0875	: 0.02
fragment_213.0925	: 0.02
fragment_225.0925	: 0.02
fragment_268.0725	: 0.02
fragment_227.0725	: 0.02
loss_46.0425	: 0.01
fragment_223.0775	: 0.01
fragment_689.4475	: 0.01
fragment_990.5425	: 0.01
loss_18.0075	: 0.01
loss_42.0075	: 0.01
fragment_148.1125	: 0.01
fragment_197.0975	: 0.01
fragment_199.0775	: 0.01
fragment_690.4425	: 0.01
loss_17.0075	: 0.01
fragment_991.5425	: 0.01
loss_17.0025	: 0.01
fragment_326.1075	: 0.01
loss_122.0125	: 0.01
fragment_282.0875	: 0.01
fragment_388.1525	: 0.01
fragment_240.0775	: 0.01
fragment_254.0875	: 0.01
fragment_308.1275	: 0.01
fragment_221.0975	: 0.01
fragment_326.1025	: 0.01
loss_190.0825	: 0.01
fragment_309.0775	: 0.01
loss_168.0625	: 0.01
fragment_254.0575	: 0.01
fragment_210.0675	: 0.01

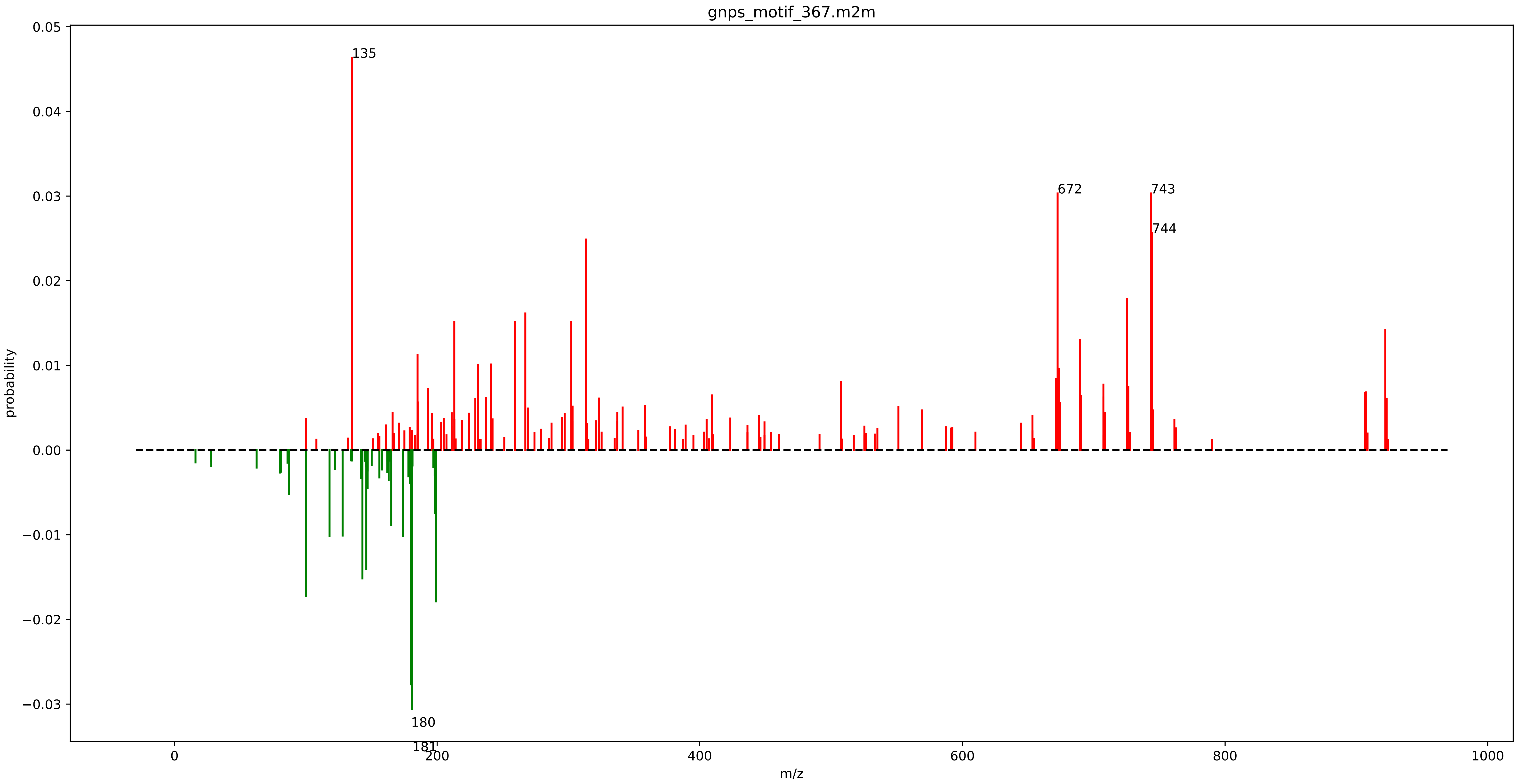

gnps_motif_367.m2m
probability
m/z
135
672
743
744
180
181
0.05
0.04
0.03
0.02
0.01
0.00
−0.01
−0.02
−0.03
0
200
400
600
800
1000

gnps_motif_367.m2m

fragment_135.0575	: 0.05
loss_181.0975	: 0.03
fragment_672.4425	: 0.03
fragment_743.3975	: 0.03
loss_180.0925	: 0.03
fragment_744.4025	: 0.03
fragment_313.1675	: 0.02
fragment_743.4025	: 0.02
loss_181.0925	: 0.02
fragment_725.3875	: 0.02
loss_199.1075	: 0.02
loss_100.0575	: 0.02
fragment_267.1775	: 0.02
loss_143.1275	: 0.02
fragment_302.1025	: 0.02
fragment_259.0925	: 0.02
fragment_213.0875	: 0.02
fragment_922.0075	: 0.01
loss_146.0625	: 0.01
fragment_689.3675	: 0.01
fragment_185.0925	: 0.01
loss_174.0575	: 0.01
fragment_241.0825	: 0.01
loss_118.0675	: 0.01
fragment_231.0975	: 0.01
loss_128.0525	: 0.01
fragment_673.4375	: 0.01
loss_165.0725	: 0.01
fragment_671.3525	: 0.01
fragment_231.0675	: 0.01
fragment_507.3575	: 0.01
fragment_707.3775	: 0.01
loss_198.1075	: 0.01
fragment_726.3875	: 0.01
fragment_193.0925	: 0.01
fragment_672.3575	: 0.01
fragment_907.4875	: 0.01
fragment_906.4875	: 0.01
fragment_671.3575	: 0.01
fragment_409.1775	: 0.01
fragment_690.3675	: 0.01
fragment_673.4425	: 0.01
fragment_707.3725	: 0.01
fragment_237.1225	: 0.01
fragment_323.2375	: 0.01
fragment_923.0125	: 0.01
fragment_229.1125	: 0.01
fragment_185.1675	: 0.01
fragment_674.4475	: 0.01
fragment_358.1675	: 0.01
loss_87.0675	: 0.01
fragment_303.1275	: 0.01
fragment_551.2725	: 0.01
fragment_341.2375	: 0.01

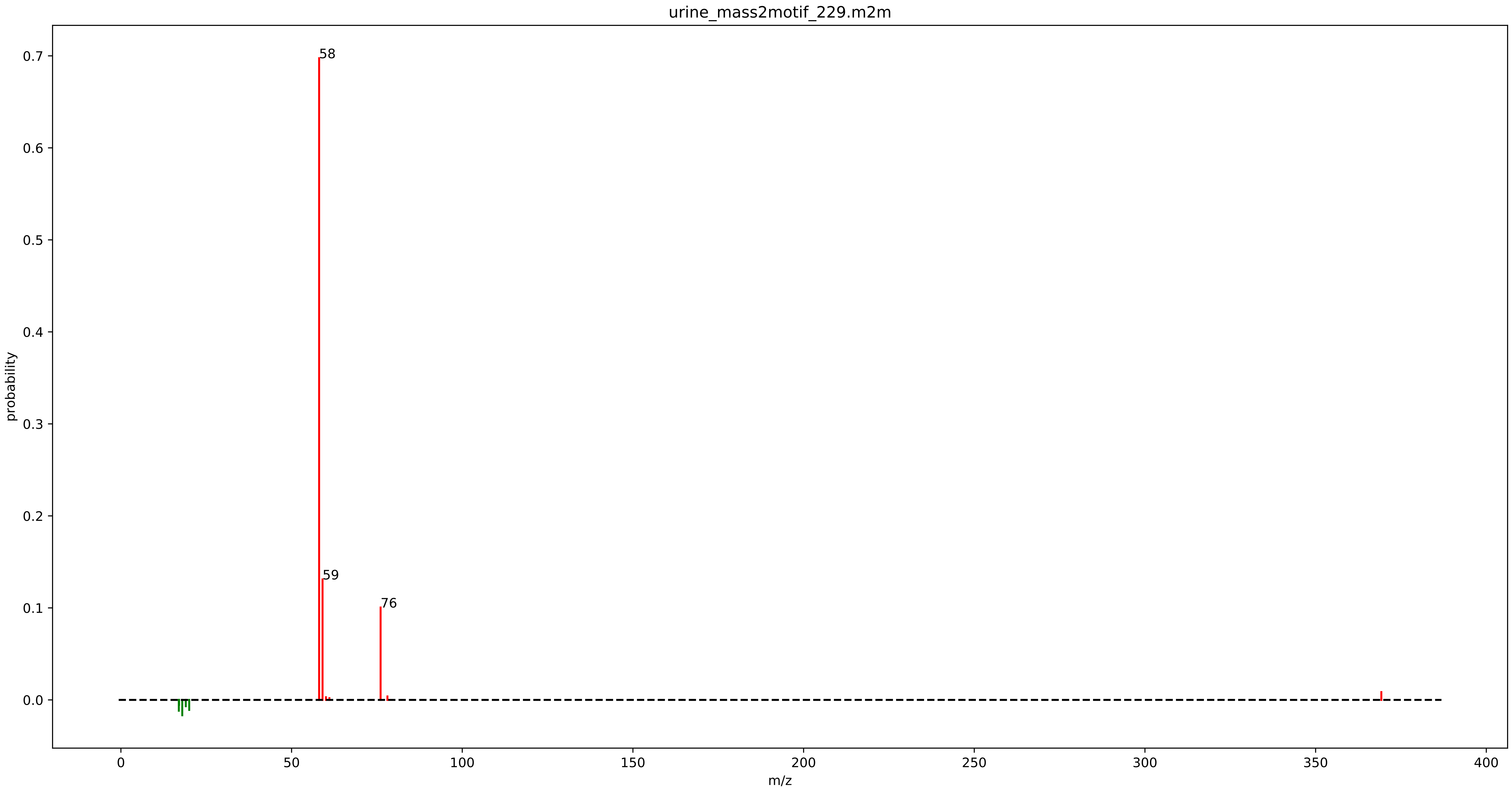

urine_mass2motif_229.m2m
probability
m/z
58
59
76
0.7
0.6
0.5
0.4
0.3
0.2
0.1
0.0
0
50
100
150
200
250
300
350
400

urine_mass2motif_229.m2m

fragment_58.0675	: 0.70
fragment_59.0725	: 0.13
fragment_76.0775	: 0.10
loss_17.9725	: 0.02
loss_16.9675	: 0.01
loss_20.0125	: 0.01
fragment_369.2525	: 0.01
loss_19.0075	: 0.01
loss_17.0375	: 0.01

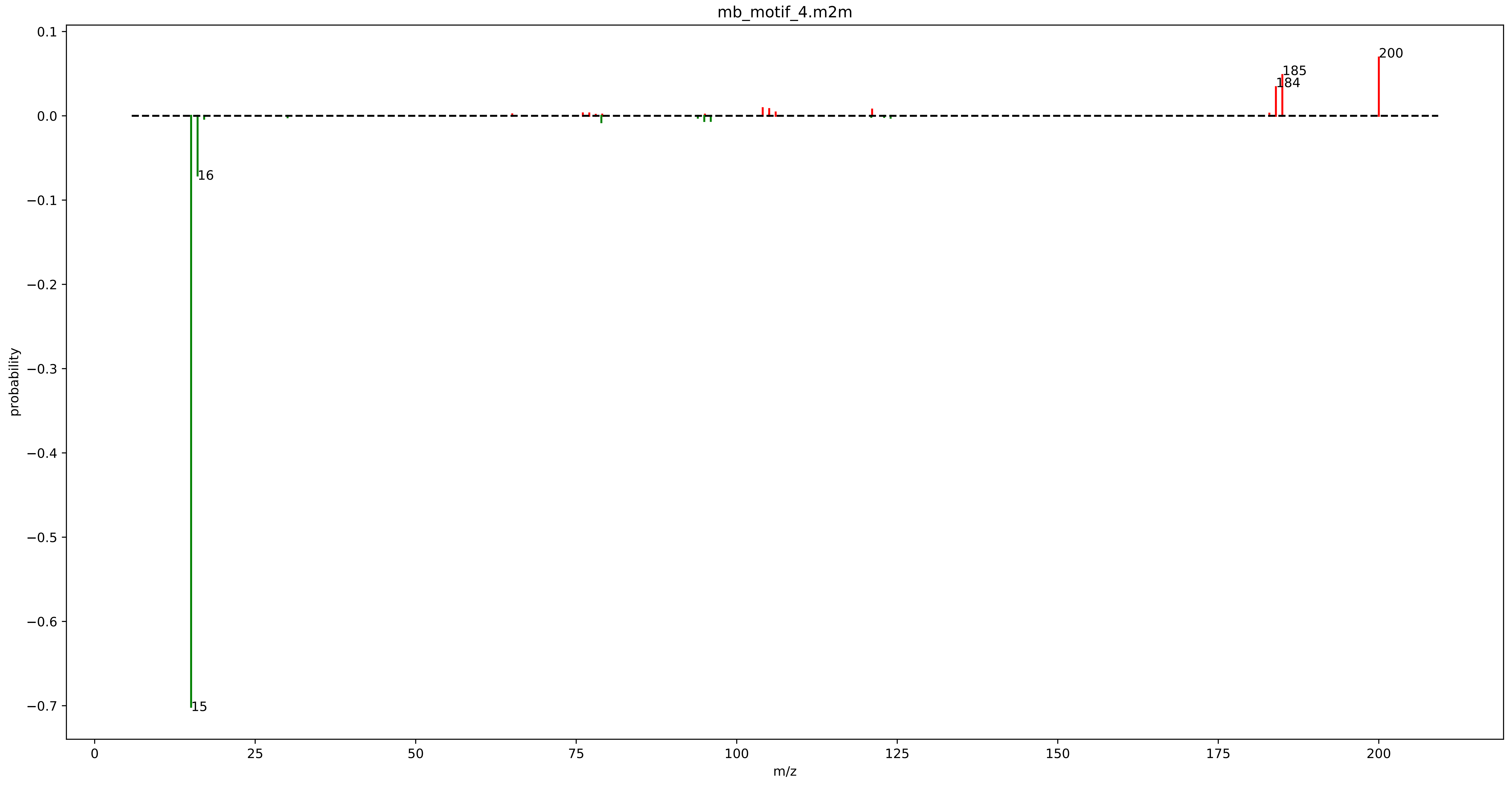

mb_motif_4.m2m
probability
m/z
0.1
0.0
−0.1
−0.2
−0.3
−0.4
−0.5
−0.6
−0.7
0
25
50
75
100
125
150
175
200
200
185
184
16
15

mb_motif_4.m2m

loss_15.0225	: 0.70
loss_16.0325	: 0.07
fragment_200.0075	: 0.07
fragment_184.9775	: 0.05
fragment_183.9775	: 0.03
fragment_104.0475	: 0.01
fragment_105.0575	: 0.01
loss_78.9175	: 0.01
fragment_121.0875	: 0.01
loss_94.9475	: 0.01
loss_95.9575	: 0.01

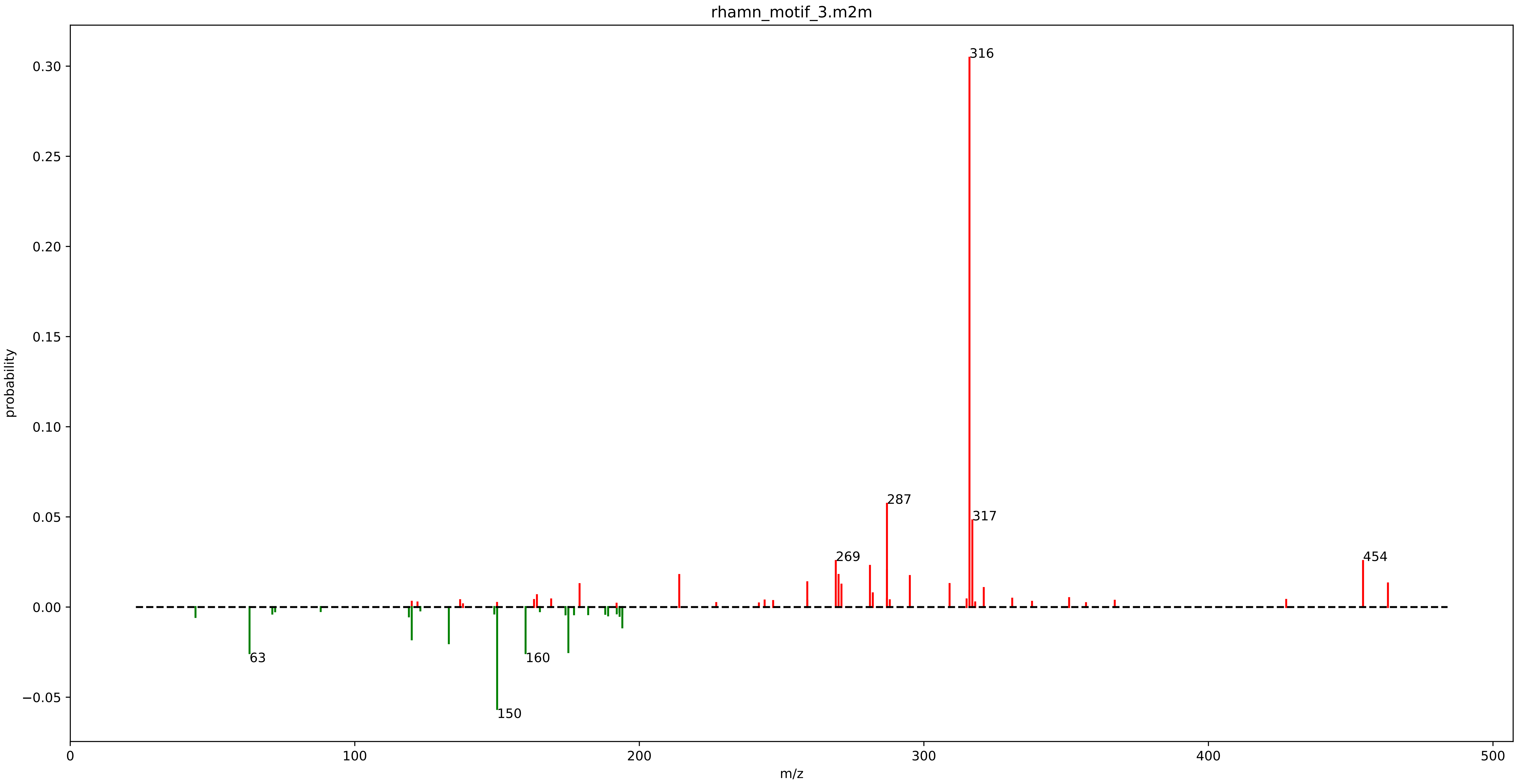

rhamn_motif_3.m2m
probability
m/z
0.30
0.25
0.20
0.15
0.10
0.05
0.00
−0.05
0
100
200
300
400
500
316
287
317
269
454
63
160
150

rhamn_motif_3.m2m

fragment_316.0225	: 0.30
fragment_287.0225	: 0.06
loss_150.0275	: 0.06
fragment_317.0275	: 0.05
loss_63.0125	: 0.03
fragment_454.3375	: 0.03
fragment_269.0375	: 0.03
loss_160.0225	: 0.03
loss_175.0575	: 0.02
fragment_281.0475	: 0.02
fragment_287.0175	: 0.02
loss_133.0525	: 0.02
loss_120.0175	: 0.02
fragment_270.0175	: 0.02
fragment_214.0275	: 0.02
fragment_295.0625	: 0.02
fragment_259.0225	: 0.01
fragment_463.0875	: 0.01
fragment_309.0325	: 0.01
fragment_178.9975	: 0.01
fragment_271.0325	: 0.01
loss_194.0225	: 0.01
fragment_321.0425	: 0.01
fragment_317.0225	: 0.01
fragment_316.0125	: 0.01
fragment_282.0525	: 0.01
fragment_164.0075	: 0.01
loss_44.0225	: 0.01
loss_119.0175	: 0.01

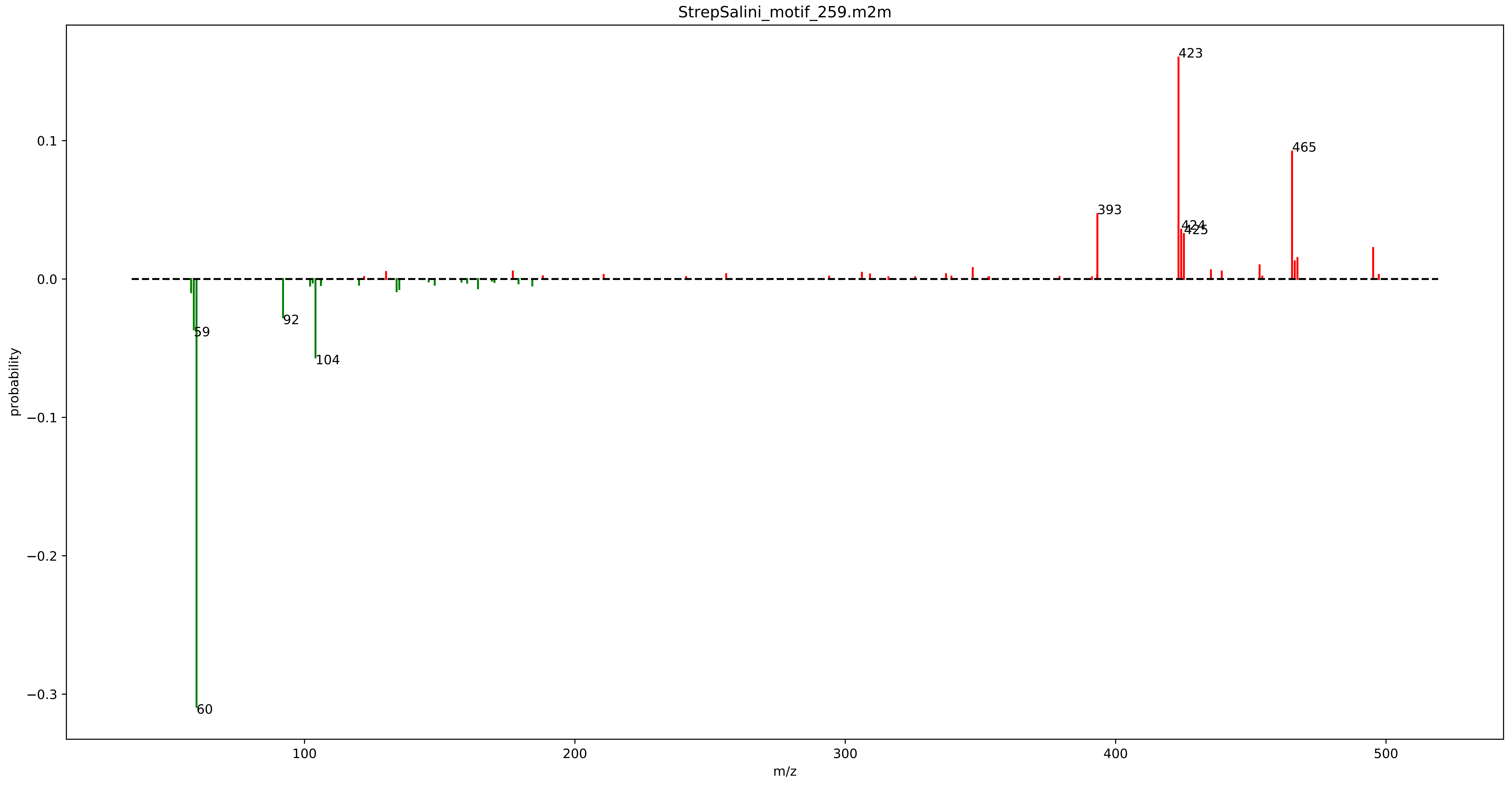

StrepSalini_motif_259.m2m
probability
m/z
0.1
0.0
−0.1
−0.2
−0.3
100
200
300
400
500
423
465
393
424
425
59
92
104
60

StrepSalini_motif_259.m2m

loss_60.0500	: 0.31
fragment_423.2500	: 0.16
fragment_465.2500	: 0.09
loss_104.0500	: 0.06
fragment_393.2500	: 0.05
loss_59.0500	: 0.04
fragment_424.2500	: 0.04
fragment_425.2500	: 0.03
loss_92.0500	: 0.03
fragment_495.2500	: 0.02
fragment_467.2500	: 0.02
fragment_466.2500	: 0.01
fragment_453.2500	: 0.01
loss_58.0475	: 0.01
loss_134.0500	: 0.01
fragment_347.1500	: 0.01
loss_135.0500	: 0.01
loss_164.1500	: 0.01
fragment_435.2500	: 0.01
fragment_177.0525	: 0.01
fragment_439.2500	: 0.01
fragment_130.1500	: 0.01

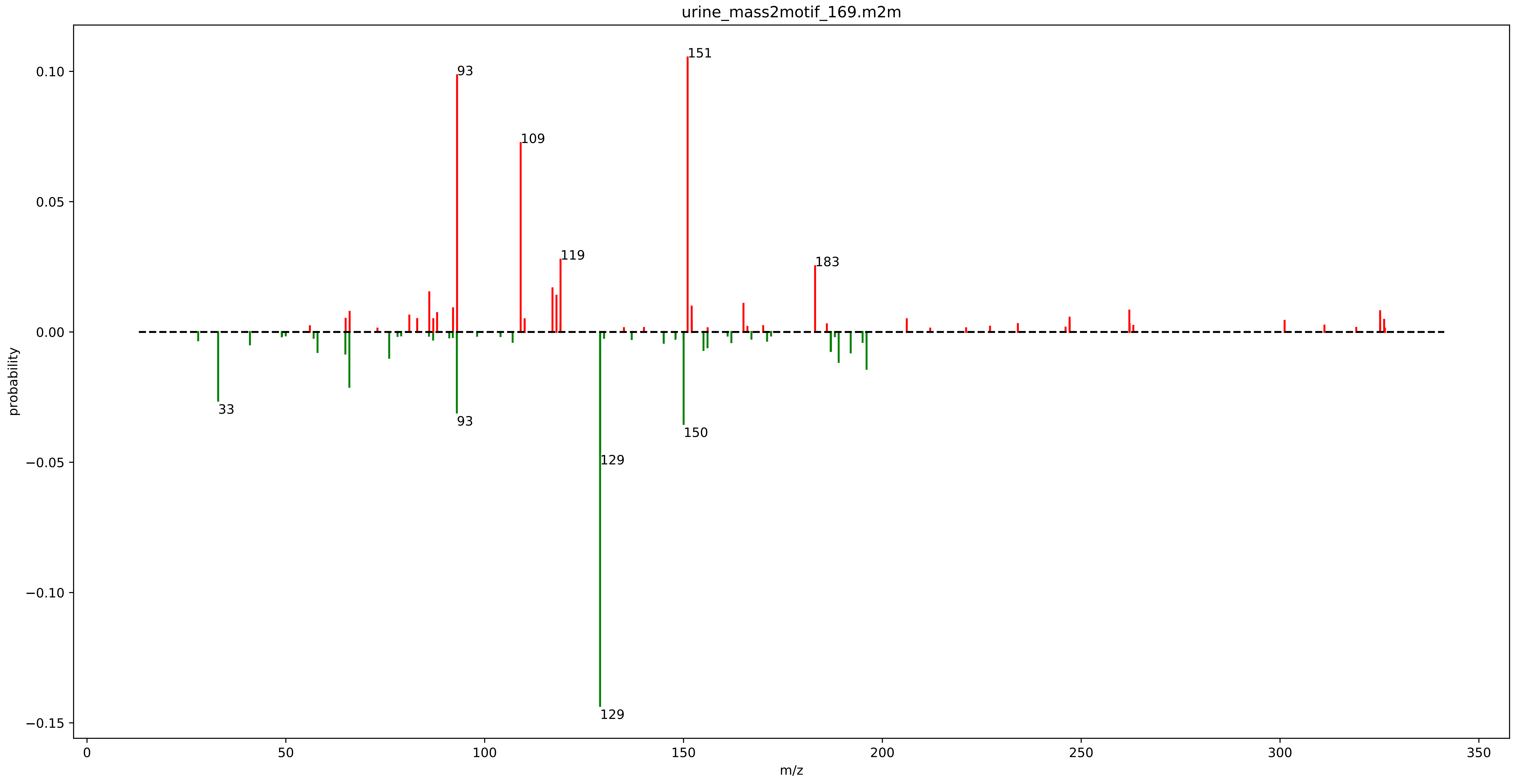

urine_mass2motif_169.m2m
probability
m/z
0.10
0.05
0.00
−0.05
−0.10
−0.15
0
50
100
150
200
250
300
350
93
109
119
151
183
33
93
129
150
129

urine_mass2motif_169.m2m

loss_129.0225	: 0.14
fragment_151.0325	: 0.11
fragment_93.0575	: 0.10
fragment_109.0475	: 0.07
loss_129.0425	: 0.05
loss_150.0275	: 0.04
loss_93.0025	: 0.03
fragment_119.0725	: 0.03
loss_32.9825	: 0.03
fragment_183.0875	: 0.03
loss_65.9775	: 0.02
fragment_117.0475	: 0.02
fragment_86.0725	: 0.02
loss_196.0275	: 0.01
fragment_118.0525	: 0.01
loss_189.0175	: 0.01
fragment_165.0775	: 0.01
loss_75.9925	: 0.01
fragment_152.0525	: 0.01
fragment_92.0475	: 0.01
loss_64.9675	: 0.01
fragment_262.1025	: 0.01
fragment_325.1675	: 0.01
loss_192.0375	: 0.01
fragment_66.0475	: 0.01
loss_57.9725	: 0.01
loss_187.0075	: 0.01
fragment_88.0375	: 0.01
loss_187.0725	: 0.01
loss_155.0225	: 0.01
fragment_81.0525	: 0.01
loss_156.0325	: 0.01
fragment_247.0775	: 0.01
fragment_65.0375	: 0.01

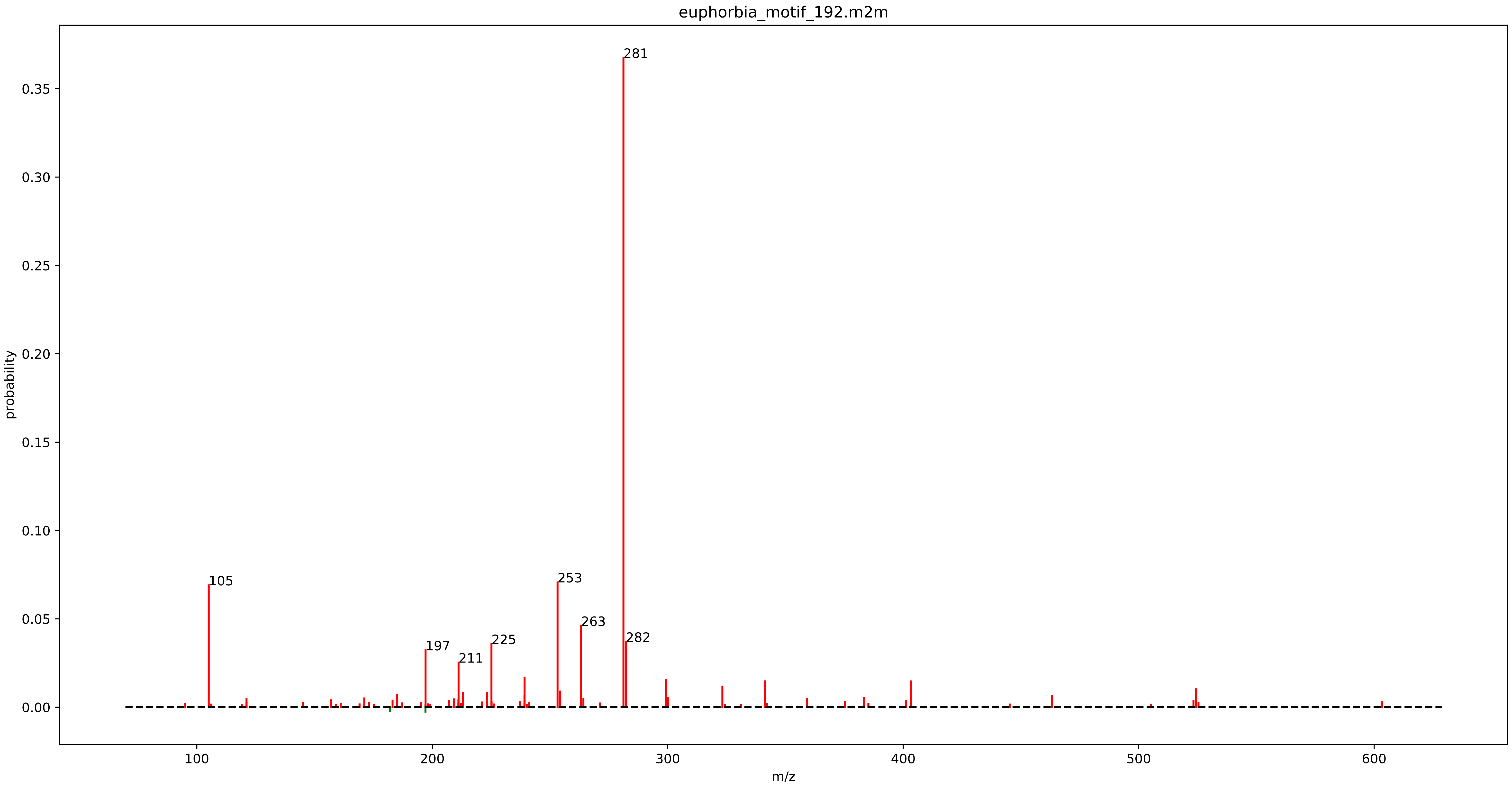

euphorbia_motif_192.m2m
probability
m/z
0.35
0.30
0.25
0.20
0.15
0.10
0.05
0.00
100
200
300
400
500
600
281
105
253
263
197
225
211
282

euphorbia_motif_192.m2m

fragment_281.1875	: 0.37
fragment_253.1925	: 0.07
fragment_105.0325	: 0.07
fragment_263.1775	: 0.05
fragment_282.1925	: 0.04
fragment_225.1275	: 0.04
fragment_197.1325	: 0.03
fragment_211.1475	: 0.03
fragment_239.1775	: 0.02
fragment_299.1975	: 0.02
fragment_341.2125	: 0.01
fragment_403.2275	: 0.01
fragment_299.2025	: 0.01
fragment_323.2025	: 0.01
fragment_524.4075	: 0.01
fragment_254.1975	: 0.01
fragment_223.1475	: 0.01
fragment_213.1275	: 0.01
fragment_185.0975	: 0.01
fragment_341.2075	: 0.01
fragment_463.2425	: 0.01
fragment_239.1425	: 0.01
fragment_463.2475	: 0.01
fragment_383.2225	: 0.01
fragment_300.2025	: 0.01
fragment_403.2225	: 0.01

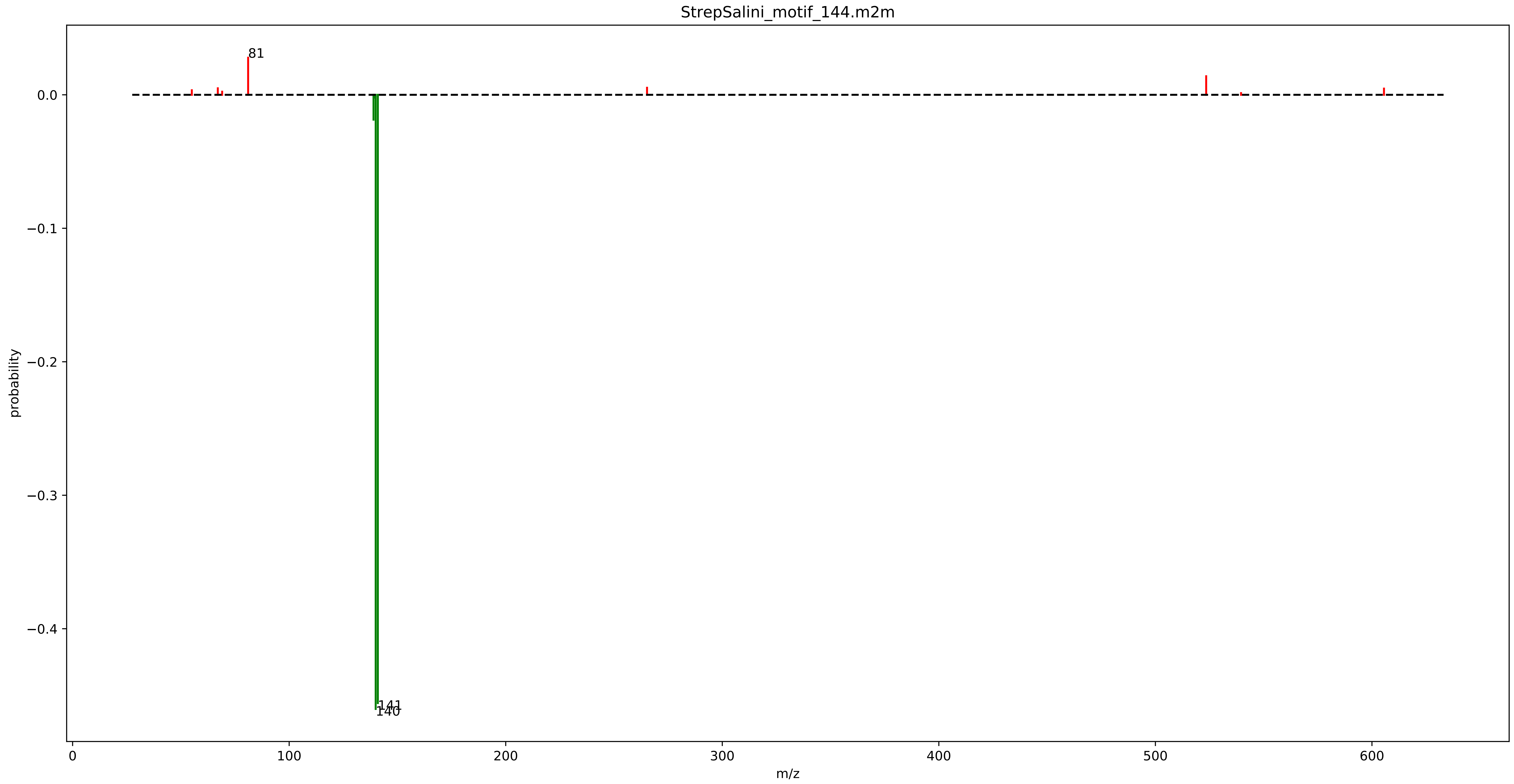

StrepSalini_motif_144.m2m
81
141
140
0.0
−0.1
−0.2
−0.3
−0.4
probability
0
100
200
300
400
500
600
m/z

StrepSalini_motif_144.m2m

loss_139.9500	: 0.46
loss_140.9500	: 0.46
fragment_81.0500	: 0.03
loss_138.9500	: 0.02
fragment_523.4500	: 0.01
fragment_265.2525	: 0.01

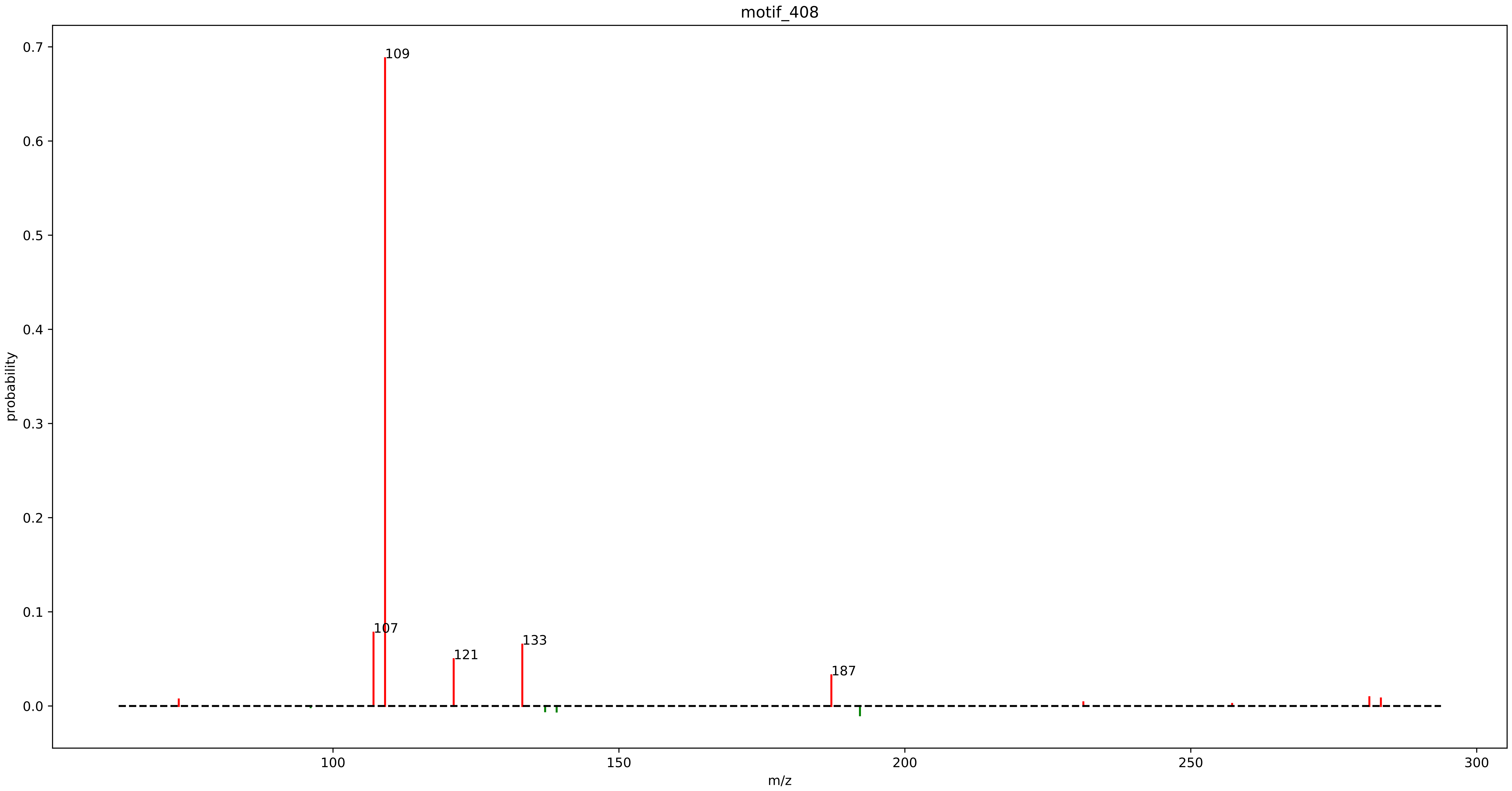

motif_408
0.7
0.6
0.5
0.4
0.3
0.2
0.1
0.0
probability
109
107
121
133
187
100
150
200
250
300
m/z

motif_408

fragment_109.1025	: 0.69
fragment_107.0825	: 0.08
fragment_133.1025	: 0.07
fragment_121.1025	: 0.05
fragment_187.1475	: 0.03
loss_192.1475	: 0.01
fragment_281.2275	: 0.01
fragment_283.2425	: 0.01
fragment_73.0275	: 0.01
loss_139.1075	: 0.01
loss_137.0925	: 0.01

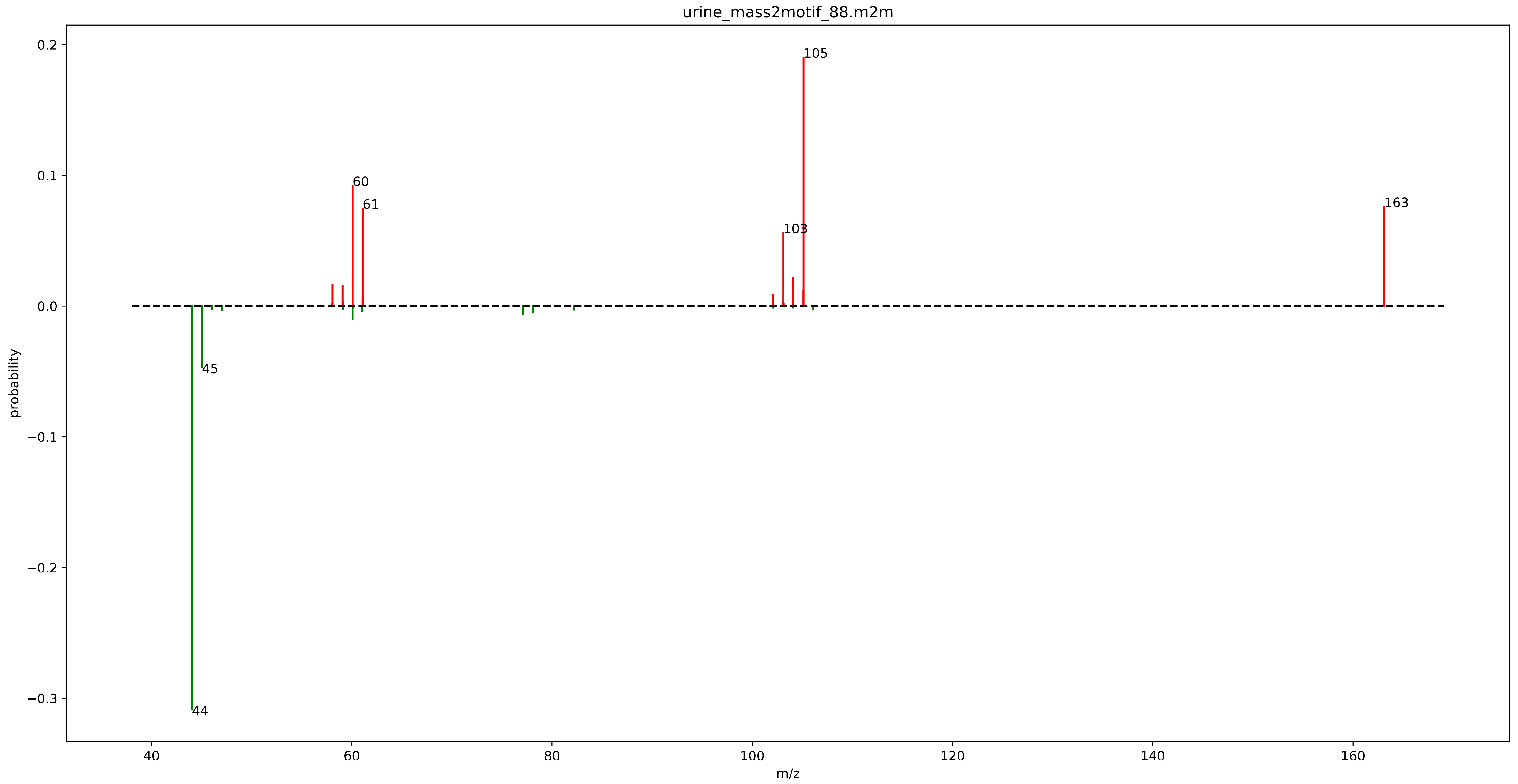

urine_mass2motif_88.m2m
probability
m/z
0.2
0.1
0.0
−0.1
−0.2
−0.3
40
60
80
100
120
140
160
105
60
61
163
103
45
44

urine_mass2motif_88.m2m

loss_44.0275	: 0.31
fragment_105.1125	: 0.19
fragment_60.0825	: 0.09
fragment_163.1175	: 0.08
fragment_61.0825	: 0.07
fragment_103.0925	: 0.06
loss_45.0325	: 0.05
fragment_104.0425	: 0.02
fragment_58.0675	: 0.02
fragment_59.0675	: 0.02
fragment_105.1025	: 0.01
loss_60.0775	: 0.01
fragment_102.0925	: 0.01
loss_60.0675	: 0.01
fragment_61.0775	: 0.01
loss_77.0825	: 0.01
loss_77.0775	: 0.01

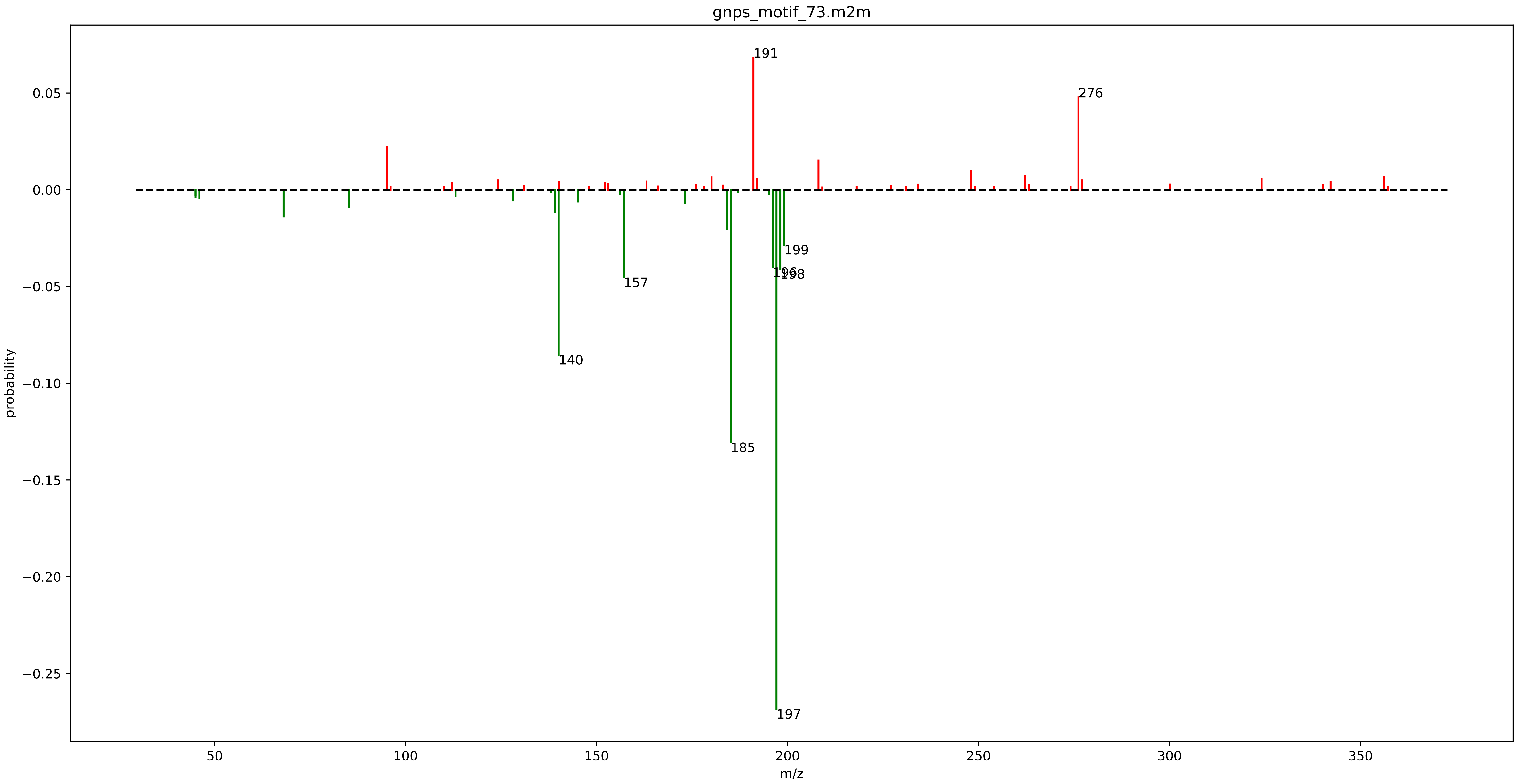

gnps_motif_73.m2m
probability
m/z
0.05
0.00
−0.05
−0.10
−0.15
−0.20
−0.25
50
100
150
200
250
300
350
191
276
199
196
198
157
140
185
197

gnps_motif_73.m2m

loss_197.1075	: 0.27
loss_185.1075	: 0.13
loss_140.0825	: 0.09
fragment_191.0725	: 0.07
fragment_276.1575	: 0.05
loss_157.1125	: 0.05
loss_198.1125	: 0.04
loss_196.1025	: 0.04
loss_199.1225	: 0.03
fragment_95.0875	: 0.02
loss_184.1025	: 0.02
fragment_208.0975	: 0.02
loss_68.0625	: 0.01
loss_139.0775	: 0.01
fragment_248.0925	: 0.01
loss_85.0875	: 0.01
fragment_262.1075	: 0.01
loss_173.1025	: 0.01
fragment_356.1875	: 0.01
fragment_180.1025	: 0.01
loss_145.1125	: 0.01
fragment_324.1225	: 0.01
loss_128.0825	: 0.01
fragment_192.0725	: 0.01

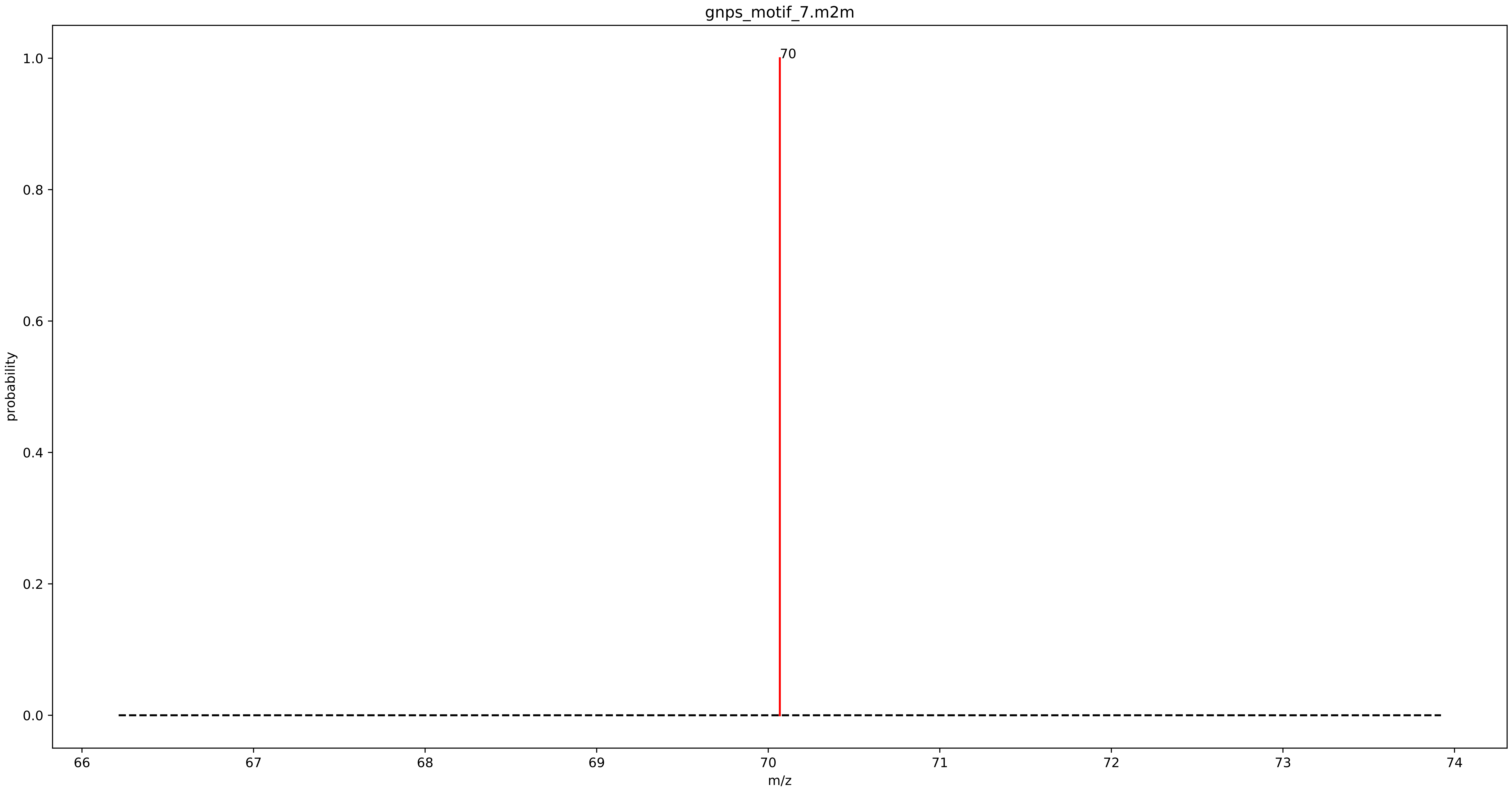
gnps_motif_7.m2m
70
1.0
0.8
0.6
0.4
0.2
0.0
probability
66
67
68
69
70
71
72
73
74
m/z

gnps_motif_7.m2m

fragment_70.0675 : 1.00

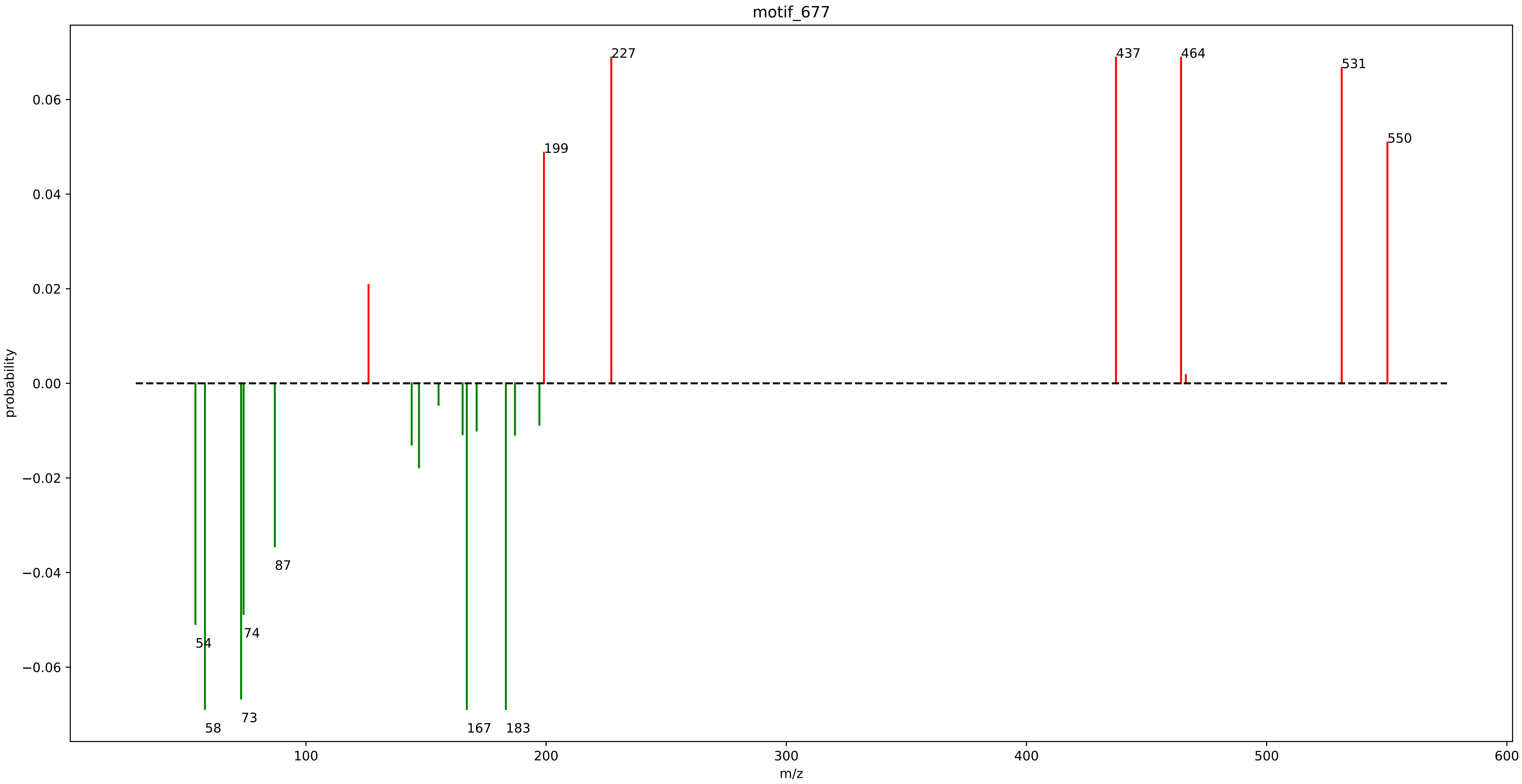

motif_677
probability
m/z
0.06
0.04
0.02
0.00
−0.02
−0.04
−0.06
100
200
300
400
500
600
227
437
464
531
550
199
87
54
74
58
73
167
183

motif_677

fragment_437.2625	: 0.07
loss_166.9775	: 0.07
fragment_227.1175	: 0.07
loss_183.2325	: 0.07
fragment_464.3375	: 0.07
loss_57.9525	: 0.07
fragment_531.2375	: 0.07
loss_73.0025	: 0.07
loss_53.9825	: 0.05
fragment_550.2575	: 0.05
fragment_199.0875	: 0.05
loss_74.0375	: 0.05
loss_87.0475	: 0.03
fragment_126.0525	: 0.02
loss_147.0675	: 0.02
loss_144.0225	: 0.01
loss_187.0275	: 0.01
loss_165.2225	: 0.01
loss_171.0675	: 0.01
loss_197.2125	: 0.01

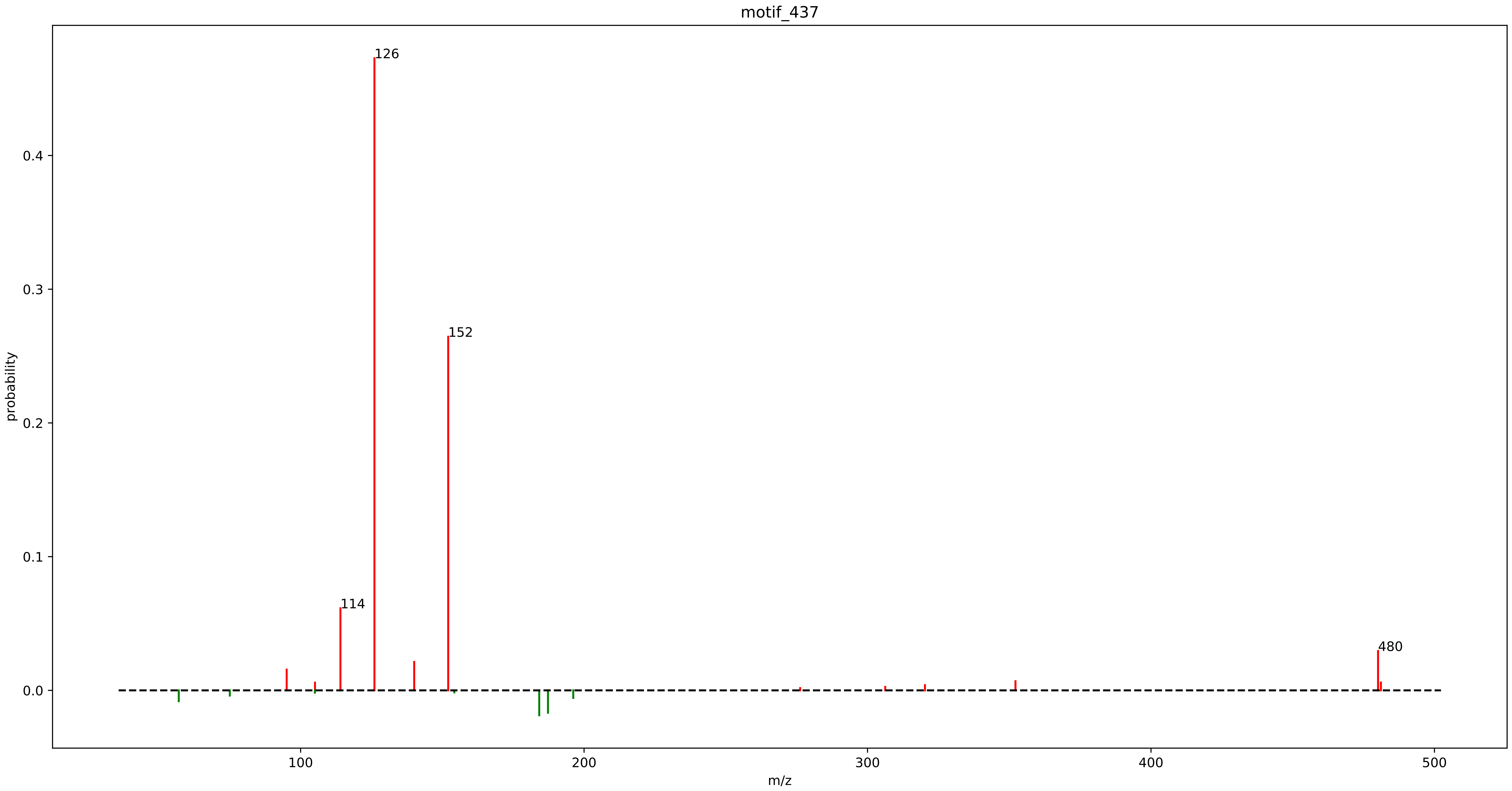

motif_437
126
152
114
480
probability
0.4
0.3
0.2
0.1
0.0
100
200
300
400
500
m/z

motif_437

fragment_126.0525	: 0.47
fragment_152.0725	: 0.26
fragment_114.0525	: 0.06
fragment_480.1025	: 0.03
fragment_140.0725	: 0.02
loss_184.1825	: 0.02
loss_187.2725	: 0.02
fragment_95.0825	: 0.02
loss_57.0175	: 0.01
fragment_352.1875	: 0.01
fragment_481.1025	: 0.01
fragment_105.0725	: 0.01
loss_196.1825	: 0.01

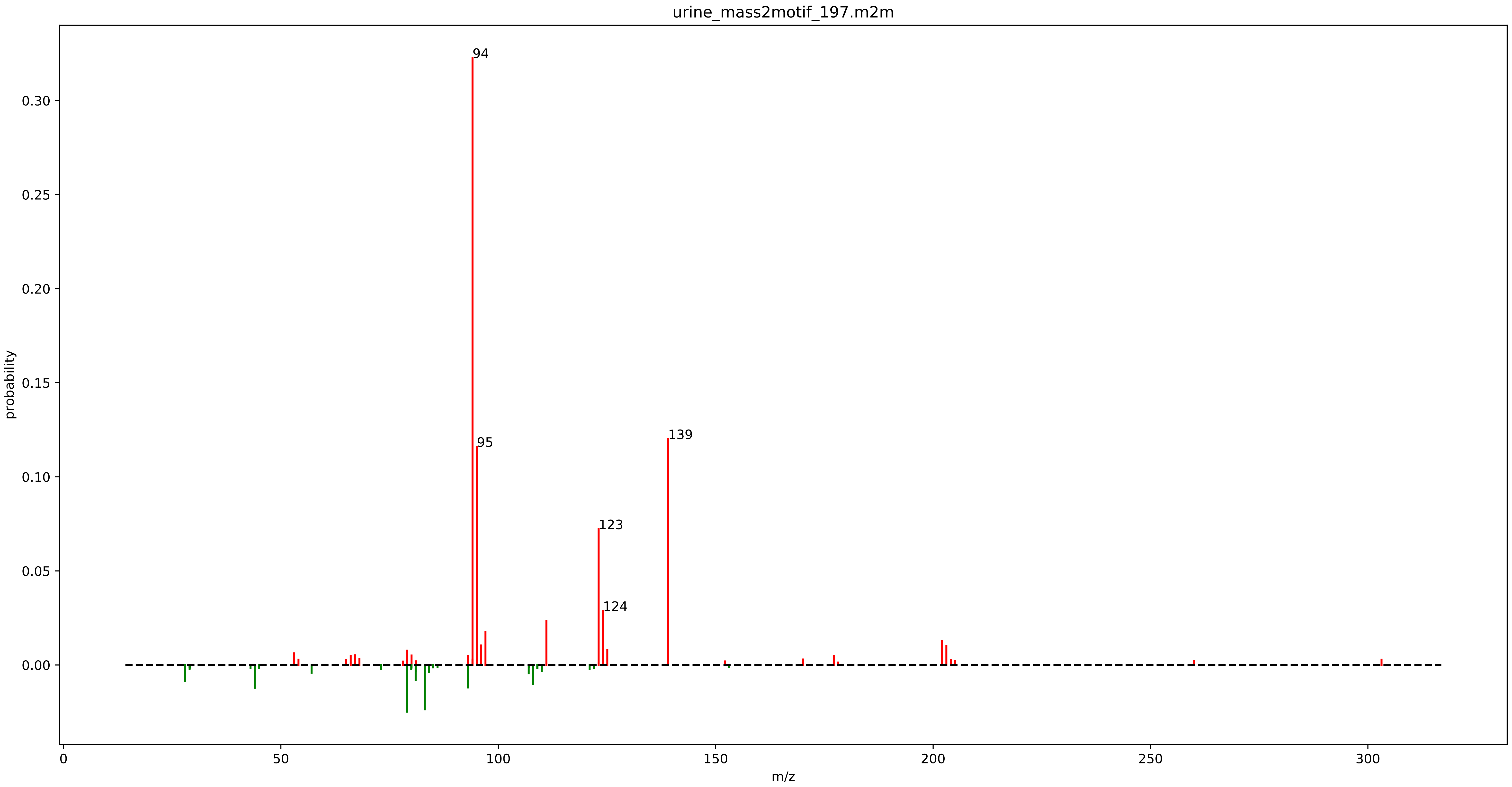

urine_mass2motif_197.m2m
probability
m/z
94
95
139
123
124
0.30
0.25
0.20
0.15
0.10
0.05
0.00
0
50
100
150
200
250
300

urine_mass2motif_197.m2m

fragment_94.0675	: 0.32
fragment_139.0625	: 0.12
fragment_95.0675	: 0.12
fragment_123.0675	: 0.07
fragment_124.0725	: 0.03
loss_78.9825	: 0.02
loss_83.0725	: 0.02
fragment_111.0625	: 0.02
fragment_95.0725	: 0.02
fragment_97.0475	: 0.02
fragment_202.0525	: 0.01
loss_43.9825	: 0.01
loss_93.0575	: 0.01
fragment_96.0425	: 0.01
fragment_203.0525	: 0.01
loss_107.9875	: 0.01
loss_27.9875	: 0.01
fragment_125.0775	: 0.01
loss_80.9825	: 0.01
fragment_79.0425	: 0.01
loss_79.0375	: 0.01
fragment_53.0375	: 0.01
fragment_67.0525	: 0.01
fragment_96.0775	: 0.01
fragment_80.0425	: 0.01

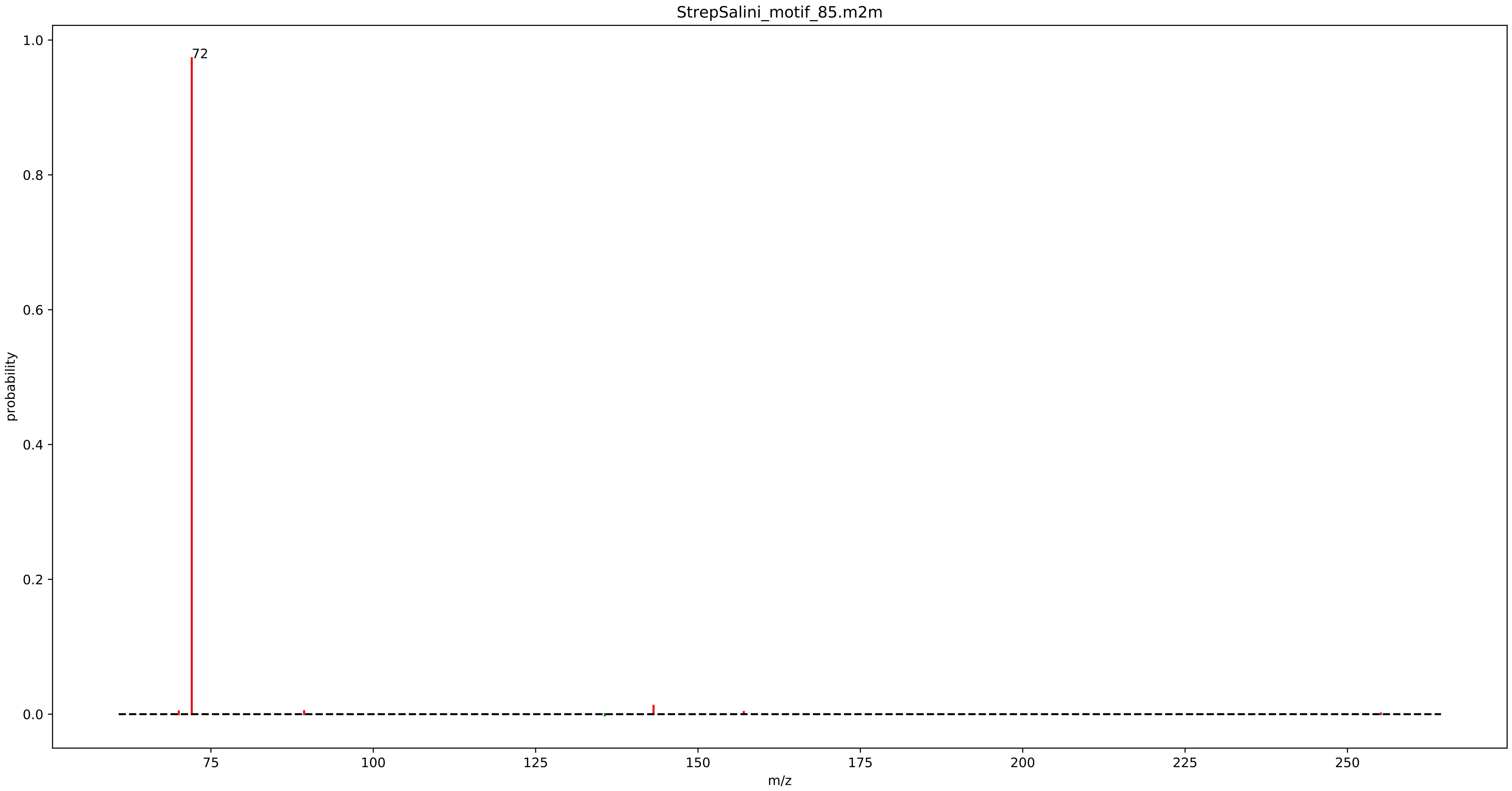
StrepSalini_motif_85.m2m
72
probability
m/z
1.0
0.8
0.6
0.4
0.2
0.0
75
100
125
150
175
200
225
250

StrepSalini_motif_85.m2m

fragment_72.0500	: 0.97
fragment_143.1525	: 0.01

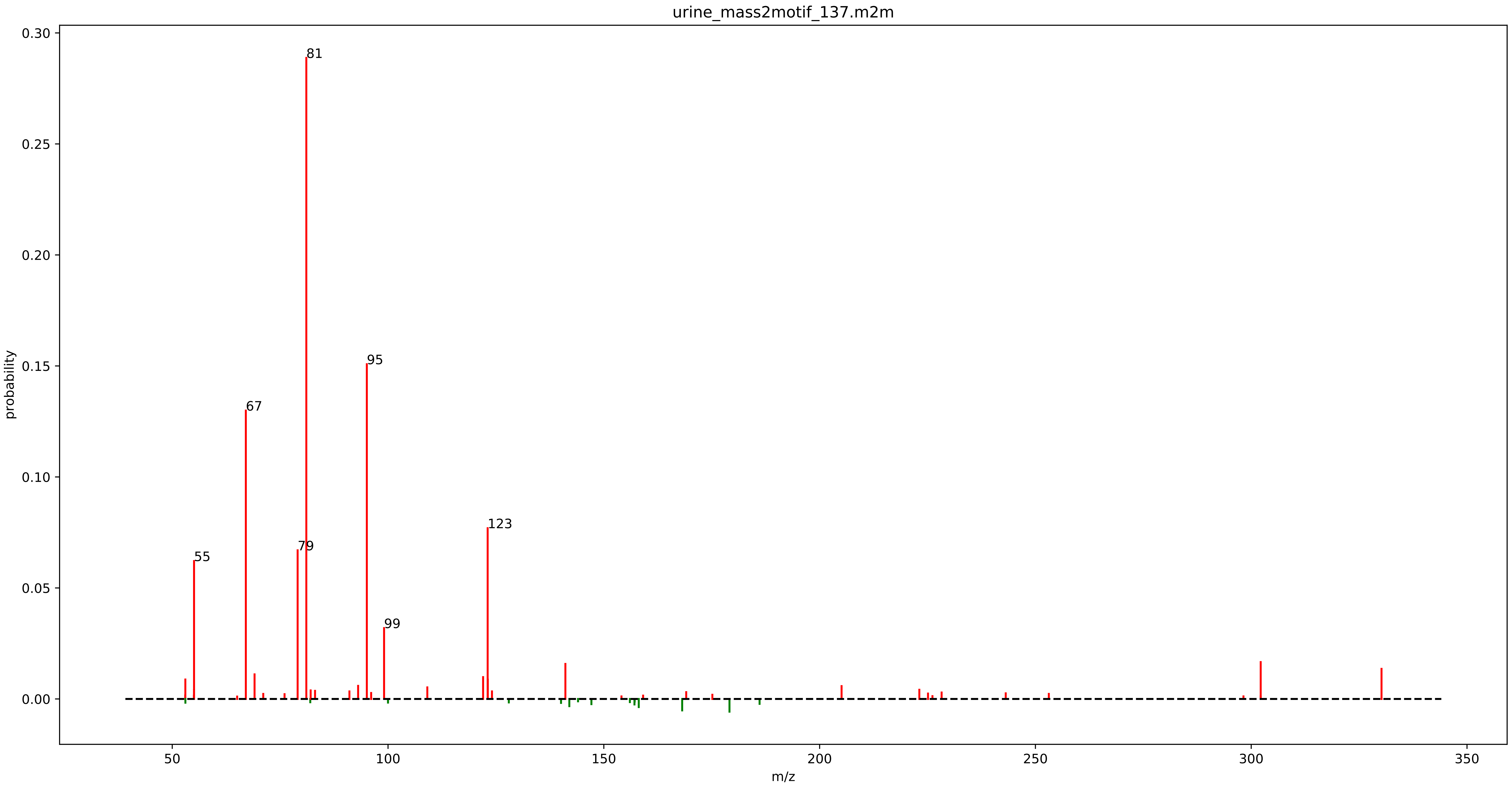

urine_mass2motif_137.m2m
probability
m/z
0.30
0.25
0.20
0.15
0.10
0.05
0.00
50
100
150
200
250
300
350
81
95
67
79
55
123
99

urine_mass2motif_137.m2m

fragment_81.0675	: 0.29
fragment_95.0875	: 0.15
fragment_67.0525	: 0.13
fragment_123.0825	: 0.08
fragment_79.0525	: 0.07
fragment_55.0525	: 0.06
fragment_99.0825	: 0.03
fragment_302.1975	: 0.02
fragment_141.0925	: 0.02
fragment_330.1925	: 0.01
fragment_69.0725	: 0.01
fragment_122.0275	: 0.01
fragment_53.0375	: 0.01
fragment_123.1175	: 0.01
fragment_93.0675	: 0.01
fragment_205.0875	: 0.01
loss_179.1125	: 0.01
fragment_109.1025	: 0.01
loss_168.1525	: 0.01

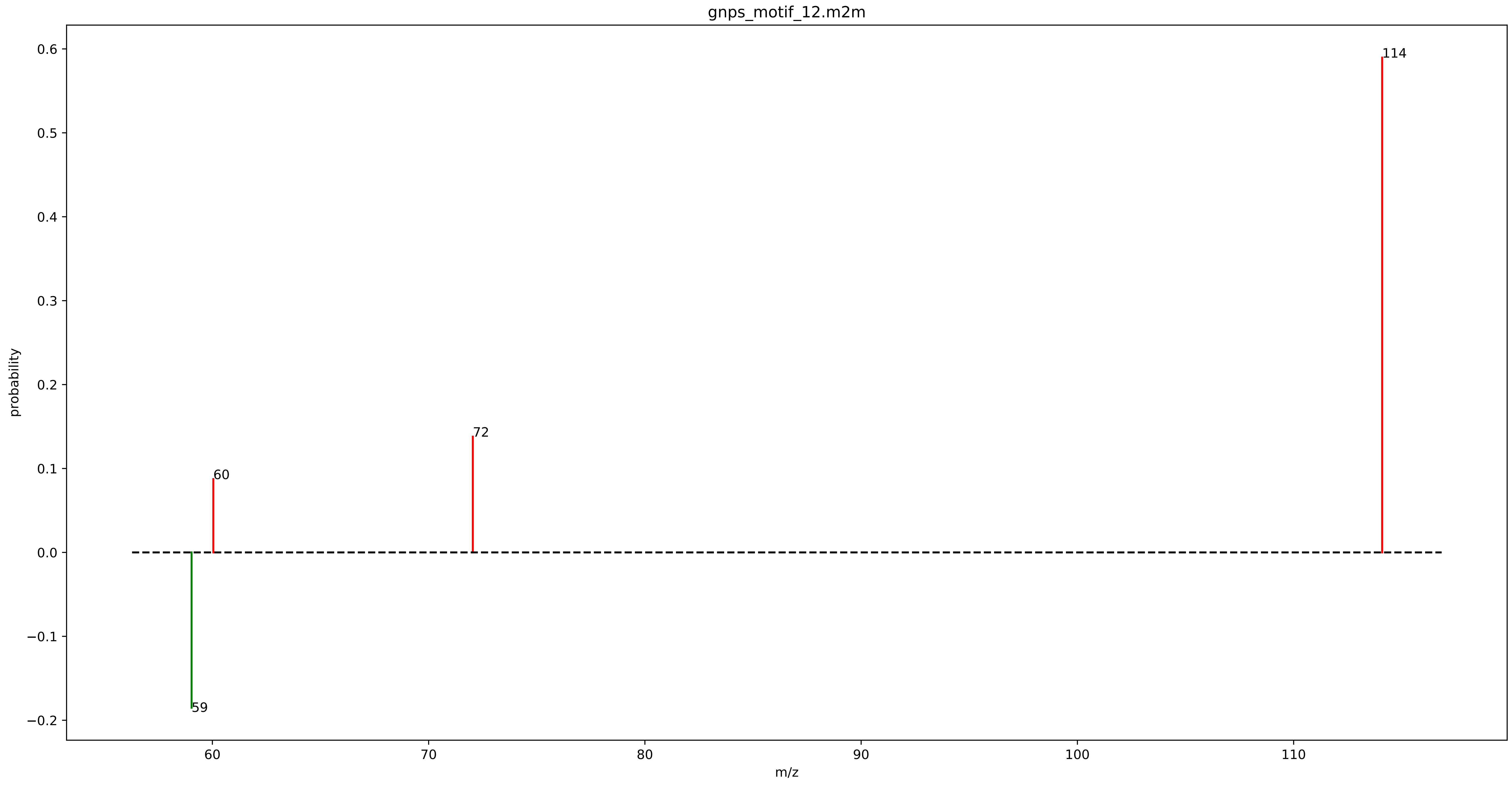

gnps_motif_12.m2m
probability
m/z
0.6
0.5
0.4
0.3
0.2
0.1
0.0
−0.1
−0.2
60
70
80
90
100
110
114
72
60
59

gnps_motif_12.m2m

fragment_114.0925	: 0.59
loss_59.0375	: 0.19
fragment_72.0425	: 0.14
fragment_60.0425	: 0.09

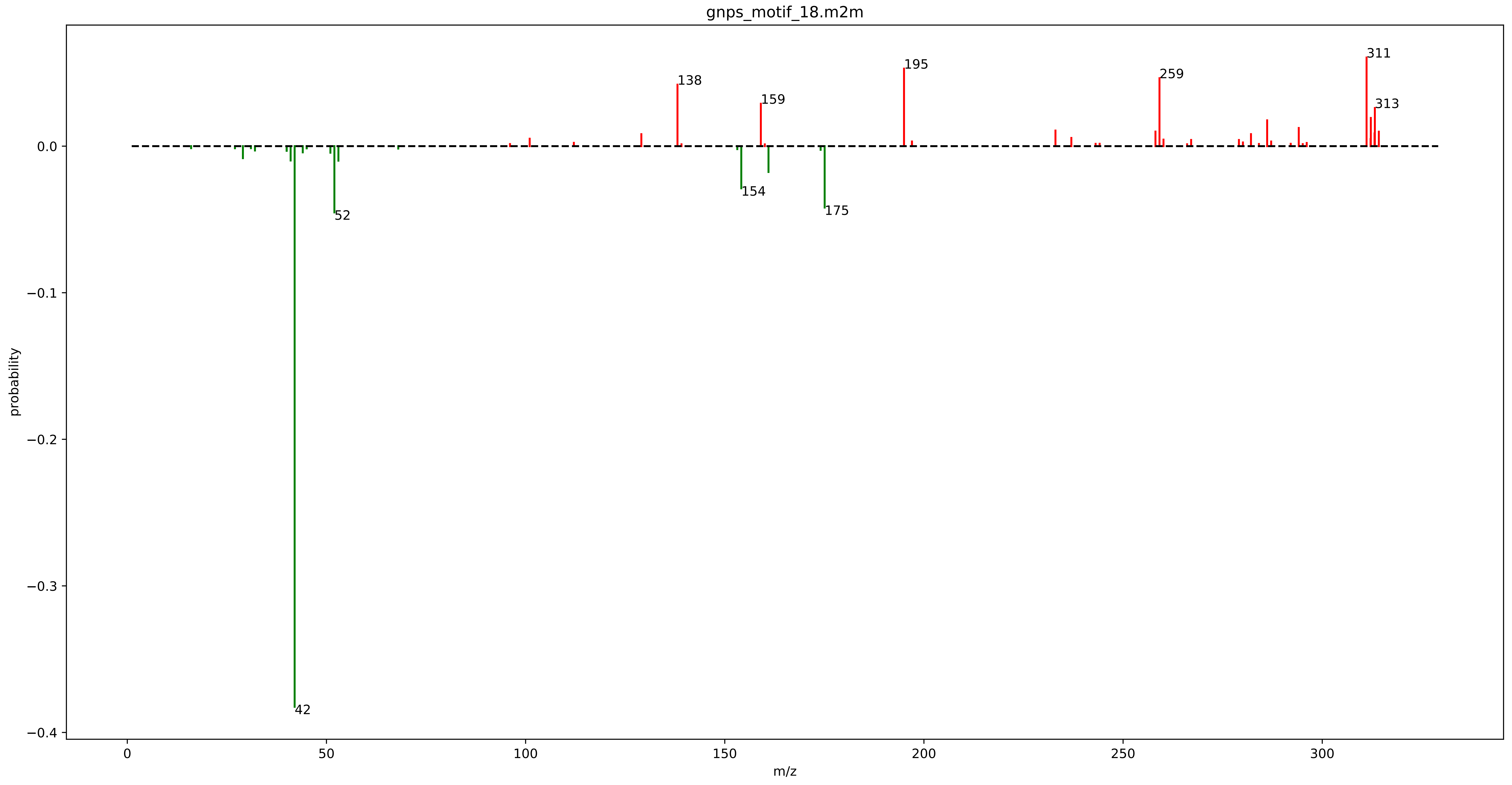

gnps_motif_18.m2m
probability
m/z
0.0
−0.1
−0.2
−0.3
−0.4
0
50
100
150
200
250
300
311
195
259
138
159
313
52
154
175
42

gnps_motif_18.m2m

loss_42.0125	: 0.38
fragment_311.1275	: 0.06
fragment_195.0025	: 0.05
fragment_259.1375	: 0.05
loss_51.9925	: 0.05
fragment_138.1275	: 0.04
loss_175.0775	: 0.04
fragment_159.0575	: 0.03
loss_154.1475	: 0.03
fragment_313.2075	: 0.03
fragment_312.1975	: 0.02
loss_160.9775	: 0.02
fragment_286.1675	: 0.02
fragment_294.1025	: 0.01
fragment_233.0225	: 0.01
fragment_258.1275	: 0.01
loss_53.0025	: 0.01
fragment_314.2075	: 0.01
loss_41.0075	: 0.01
fragment_313.1275	: 0.01
fragment_282.1075	: 0.01
loss_29.0275	: 0.01
fragment_129.0575	: 0.01
fragment_237.0125	: 0.01
fragment_101.0225	: 0.01

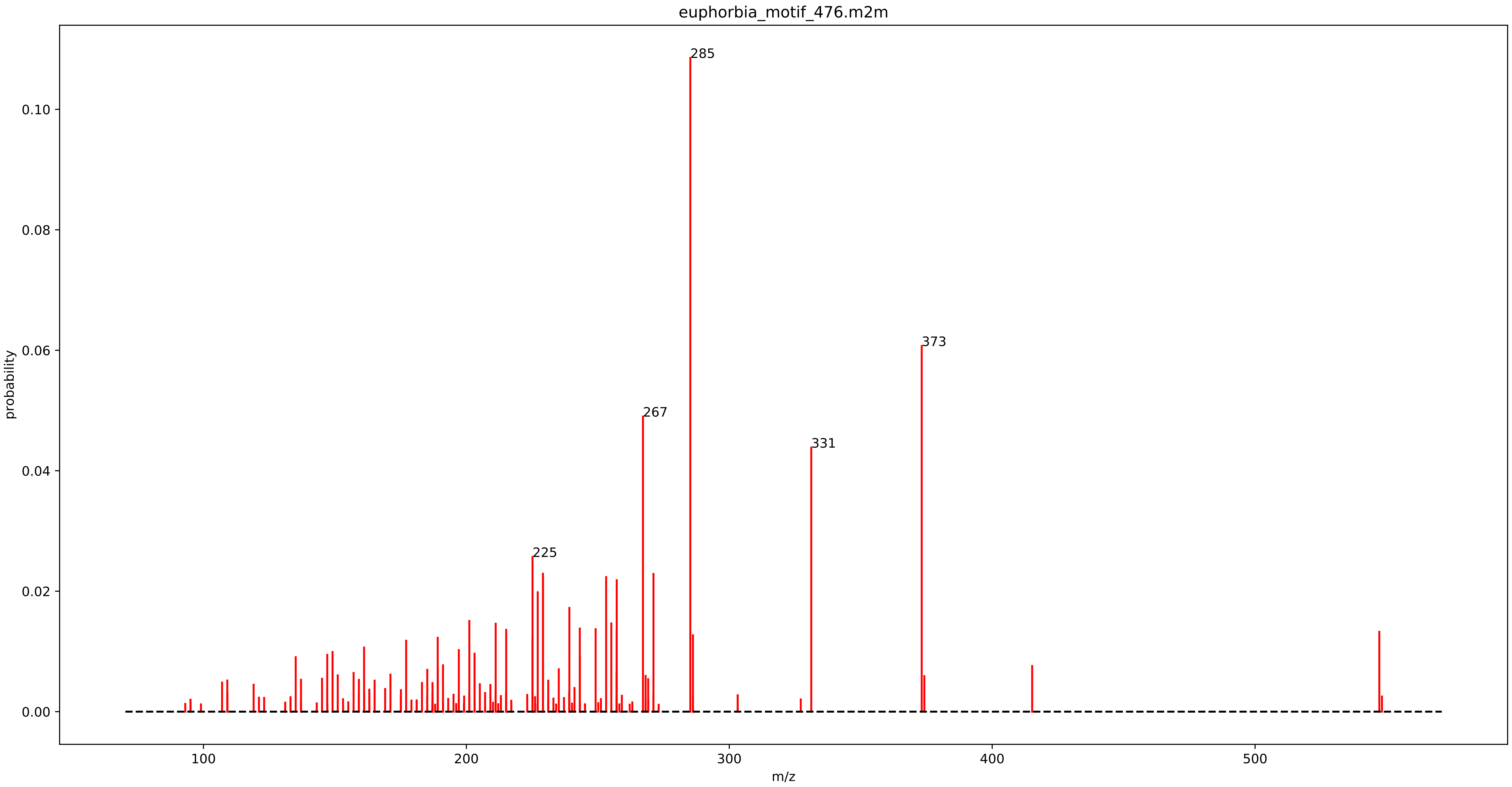

euphorbia_motif_476.m2m
probability
m/z
0.00
0.02
0.04
0.06
0.08
0.10
100
200
300
400
500
285
373
267
331
225

euphorbia_motif_476.m2m

fragment_285.1825	: 0.11
fragment_373.1975	: 0.06
fragment_267.1725	: 0.05
fragment_331.1875	: 0.04
fragment_225.1625	: 0.03
fragment_229.1225	: 0.02
fragment_271.1675	: 0.02
fragment_253.1575	: 0.02
fragment_229.1575	: 0.02
fragment_257.1875	: 0.02
fragment_227.1425	: 0.02
fragment_239.1775	: 0.02
fragment_201.1275	: 0.02
fragment_255.1375	: 0.01
fragment_211.1475	: 0.01
fragment_243.1375	: 0.01
fragment_249.1625	: 0.01
fragment_215.1425	: 0.01
fragment_257.1525	: 0.01
fragment_547.2575	: 0.01
fragment_286.1875	: 0.01
fragment_189.0925	: 0.01
fragment_225.1275	: 0.01
fragment_177.0925	: 0.01
fragment_161.0925	: 0.01
fragment_197.1325	: 0.01
fragment_149.0975	: 0.01
fragment_203.1075	: 0.01
fragment_147.0825	: 0.01
fragment_243.1725	: 0.01
fragment_135.0825	: 0.01
fragment_191.1075	: 0.01
fragment_415.2075	: 0.01
fragment_189.1275	: 0.01
fragment_235.1475	: 0.01
fragment_185.1325	: 0.01
fragment_257.1175	: 0.01
fragment_157.1025	: 0.01
fragment_171.1175	: 0.01
fragment_151.0725	: 0.01
fragment_268.1775	: 0.01
fragment_374.2025	: 0.01
fragment_145.1025	: 0.01
fragment_189.0875	: 0.01
fragment_269.1525	: 0.01
fragment_137.0975	: 0.01
fragment_159.1175	: 0.01
fragment_109.0625	: 0.01
fragment_165.0925	: 0.01
fragment_231.1375	: 0.01

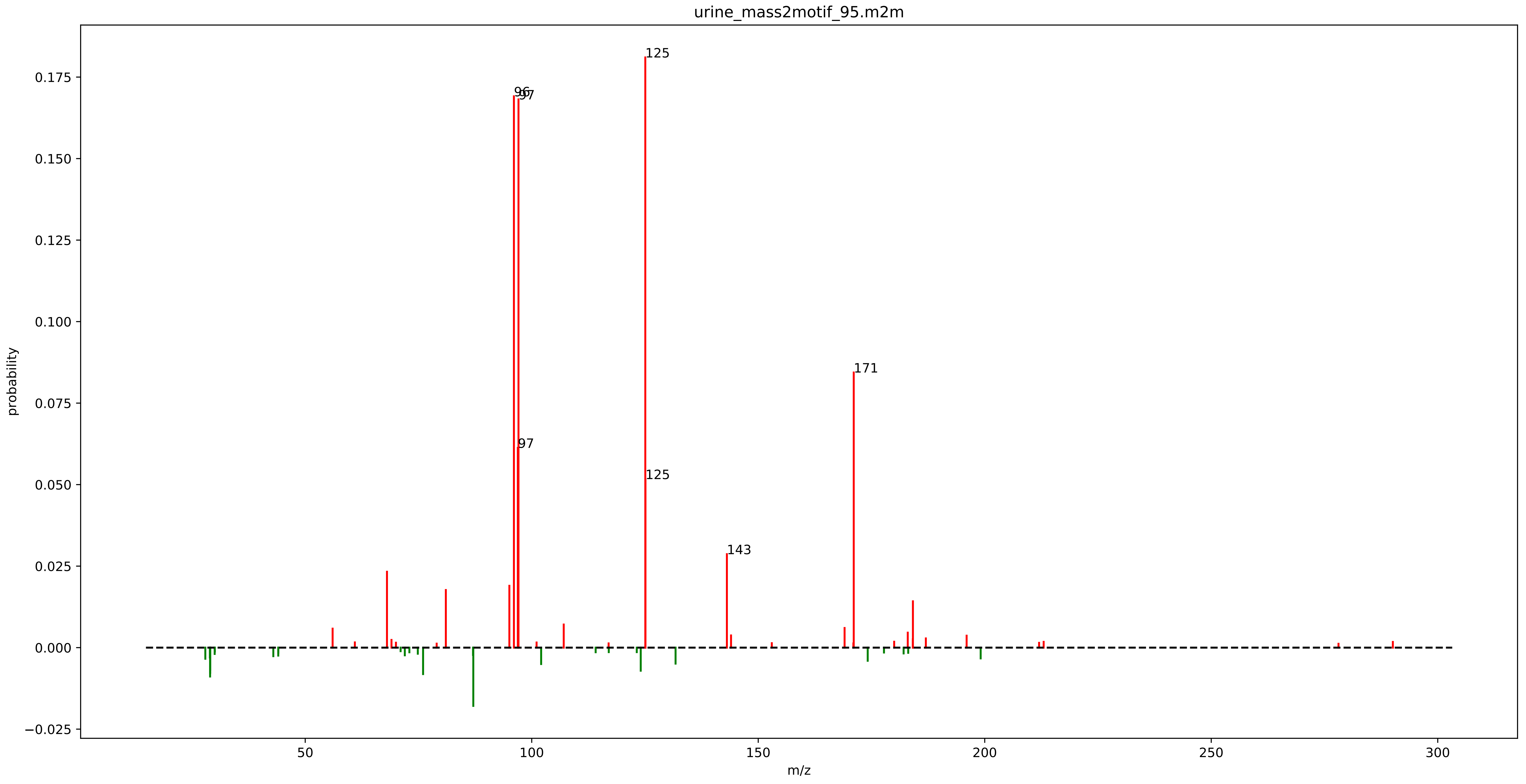
urine_mass2motif_95.m2m
probability
m/z
0.175
0.150
0.125
0.100
0.075
0.050
0.025
0.000
−0.025
50
100
150
200
250
300
125
96
97
171
97
125
143

urine_mass2motif_95.m2m

fragment_125.0725	: 0.18
fragment_96.0675	: 0.17
fragment_97.0775	: 0.17
fragment_171.0825	: 0.08
fragment_96.9275	: 0.06
fragment_125.1075	: 0.05
fragment_143.0825	: 0.03
fragment_68.0475	: 0.02
fragment_95.0625	: 0.02
loss_87.1025	: 0.02
fragment_81.0425	: 0.02
fragment_97.0375	: 0.02
fragment_184.1475	: 0.01
loss_29.0025	: 0.01
loss_76.0175	: 0.01
loss_29.0225	: 0.01
fragment_107.0625	: 0.01
loss_124.0625	: 0.01
loss_29.0375	: 0.01
fragment_169.0625	: 0.01
fragment_56.0475	: 0.01
loss_102.0775	: 0.01

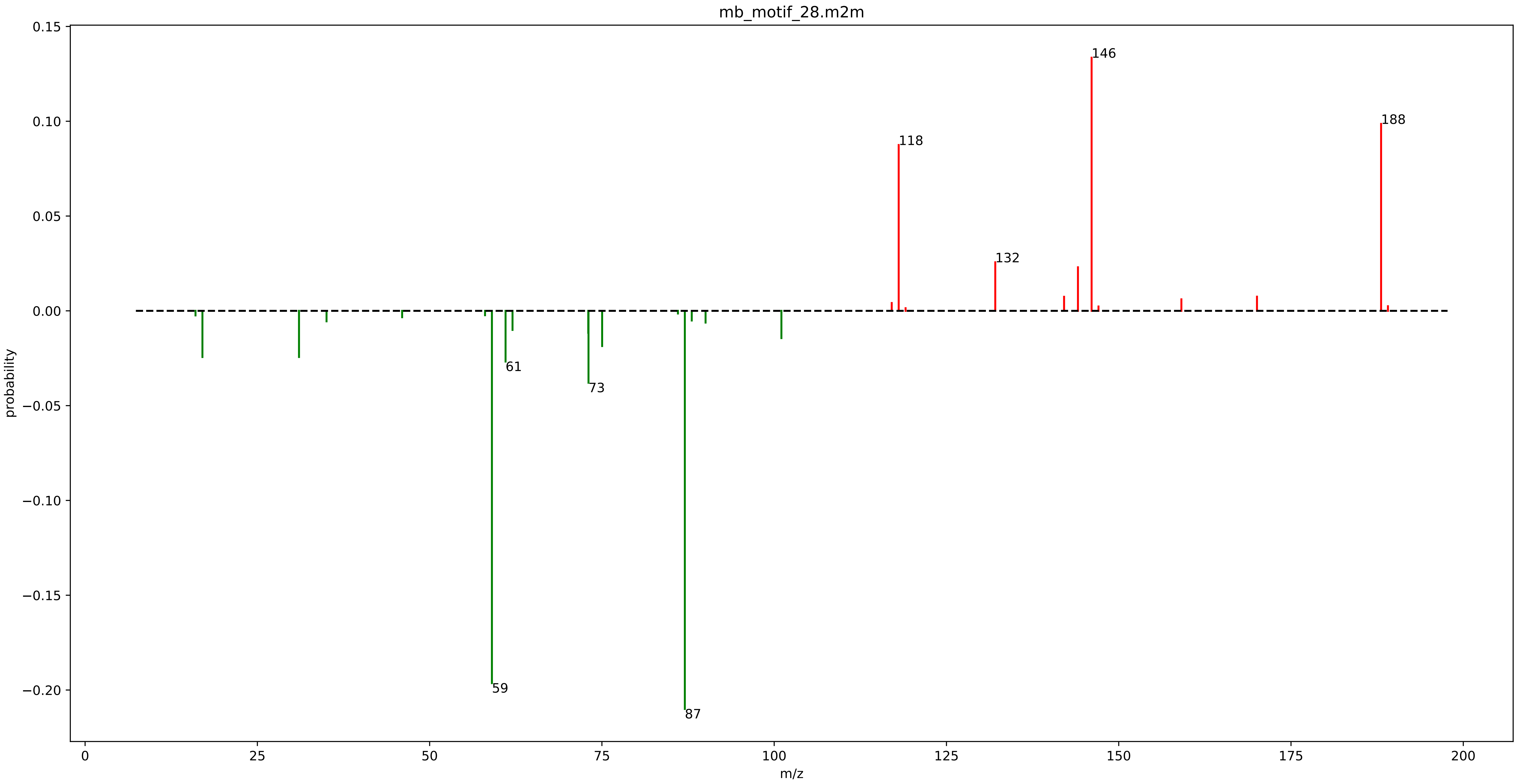

mb_motif_28.m2m
probability
m/z
0.15
0.10
0.05
0.00
−0.05
−0.10
−0.15
−0.20
0
25
50
75
100
125
150
175
200
146
188
118
132
61
73
59
87

mb_motif_28.m2m

loss_87.0325	: 0.21
loss_59.0375	: 0.20
fragment_146.0625	: 0.13
fragment_188.0725	: 0.10
fragment_118.0675	: 0.09
loss_73.0525	: 0.04
loss_61.0175	: 0.03
fragment_132.0825	: 0.03
loss_31.0425	: 0.02
loss_17.0275	: 0.02
fragment_144.0825	: 0.02
loss_75.0325	: 0.02
loss_101.0475	: 0.01
loss_73.0175	: 0.01
loss_62.0275	: 0.01
fragment_170.0625	: 0.01
fragment_142.0675	: 0.01
loss_90.0425	: 0.01
fragment_159.0925	: 0.01
loss_35.0375	: 0.01
loss_88.0375	: 0.01

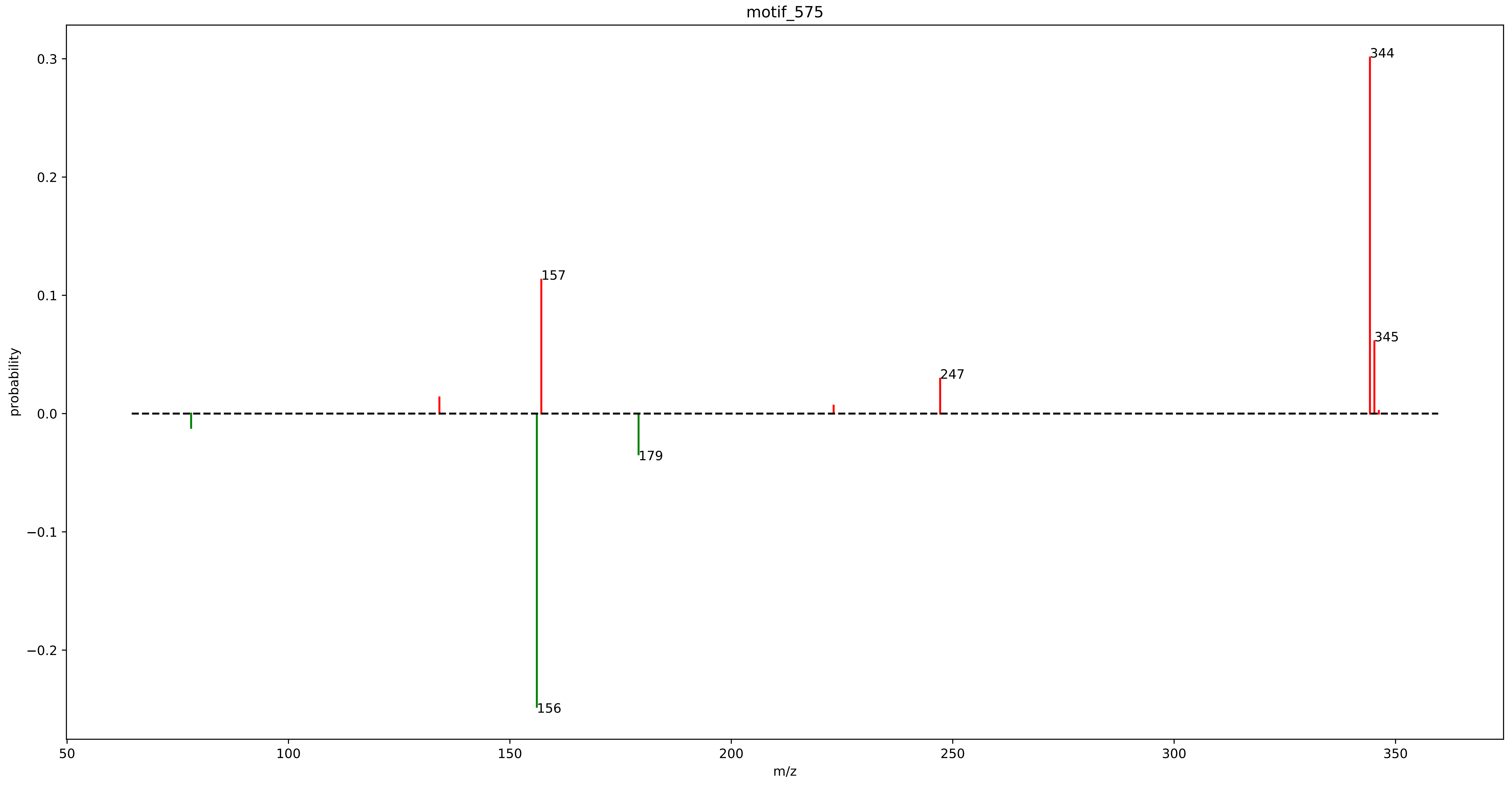

motif_575
probability
m/z
0.3
0.2
0.1
0.0
−0.1
−0.2
50
100
150
200
250
300
350
344
157
345
247
179
156

motif_575

fragment_344.2175	: 0.30
loss_156.0875	: 0.25
fragment_157.0975	: 0.11
fragment_345.2225	: 0.06
loss_179.0575	: 0.03
fragment_247.1625	: 0.03
fragment_134.0825	: 0.01
loss_78.0125	: 0.01
fragment_223.1075	: 0.01

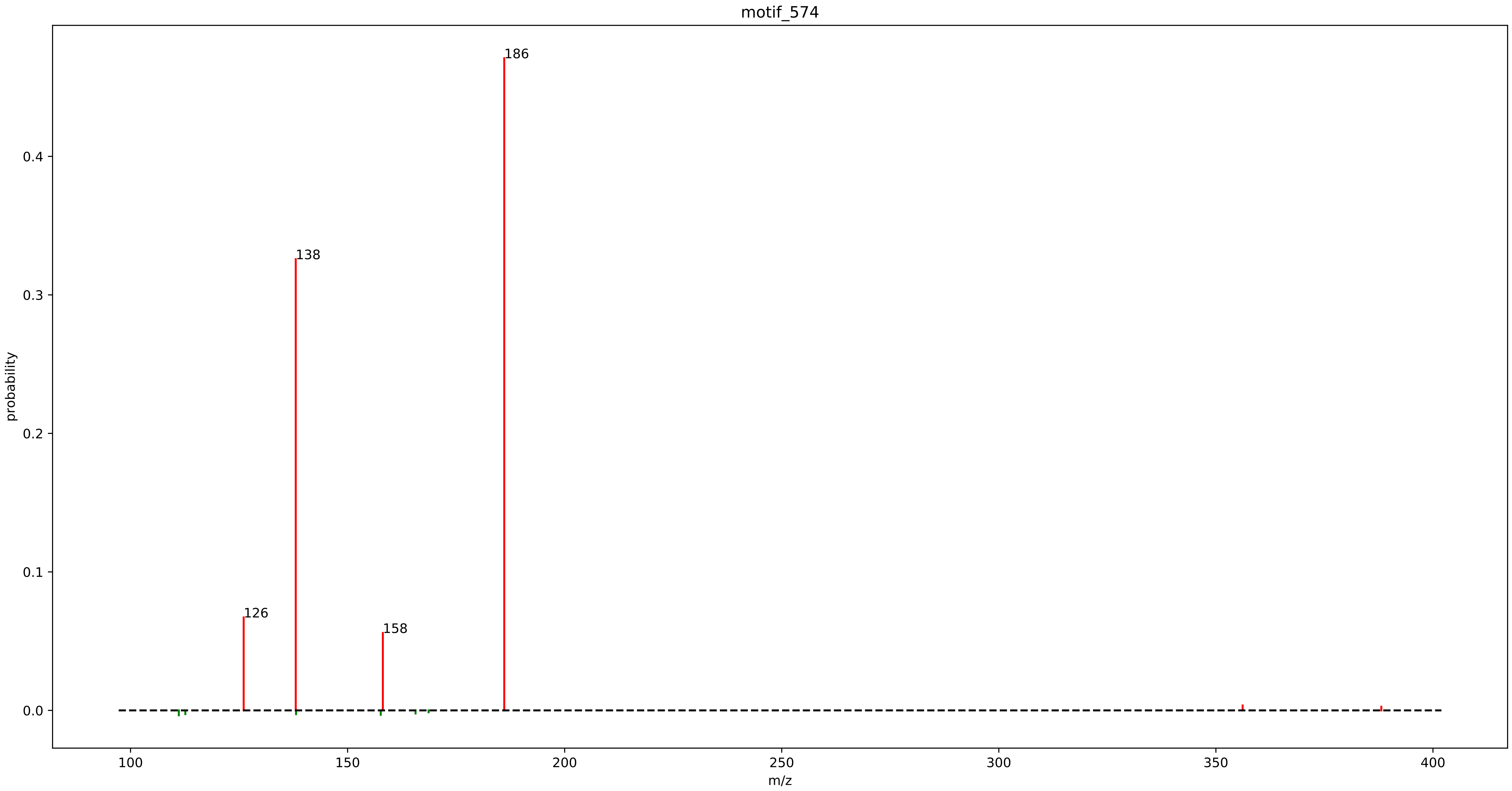

motif_574
186
138
126
158
0.4
0.3
0.2
0.1
0.0
probability
100
150
200
250
300
350
400
m/z

motif_574

fragment_186.0775	: 0.47
fragment_138.0525	: 0.33
fragment_126.0575	: 0.07
fragment_158.1175	: 0.06

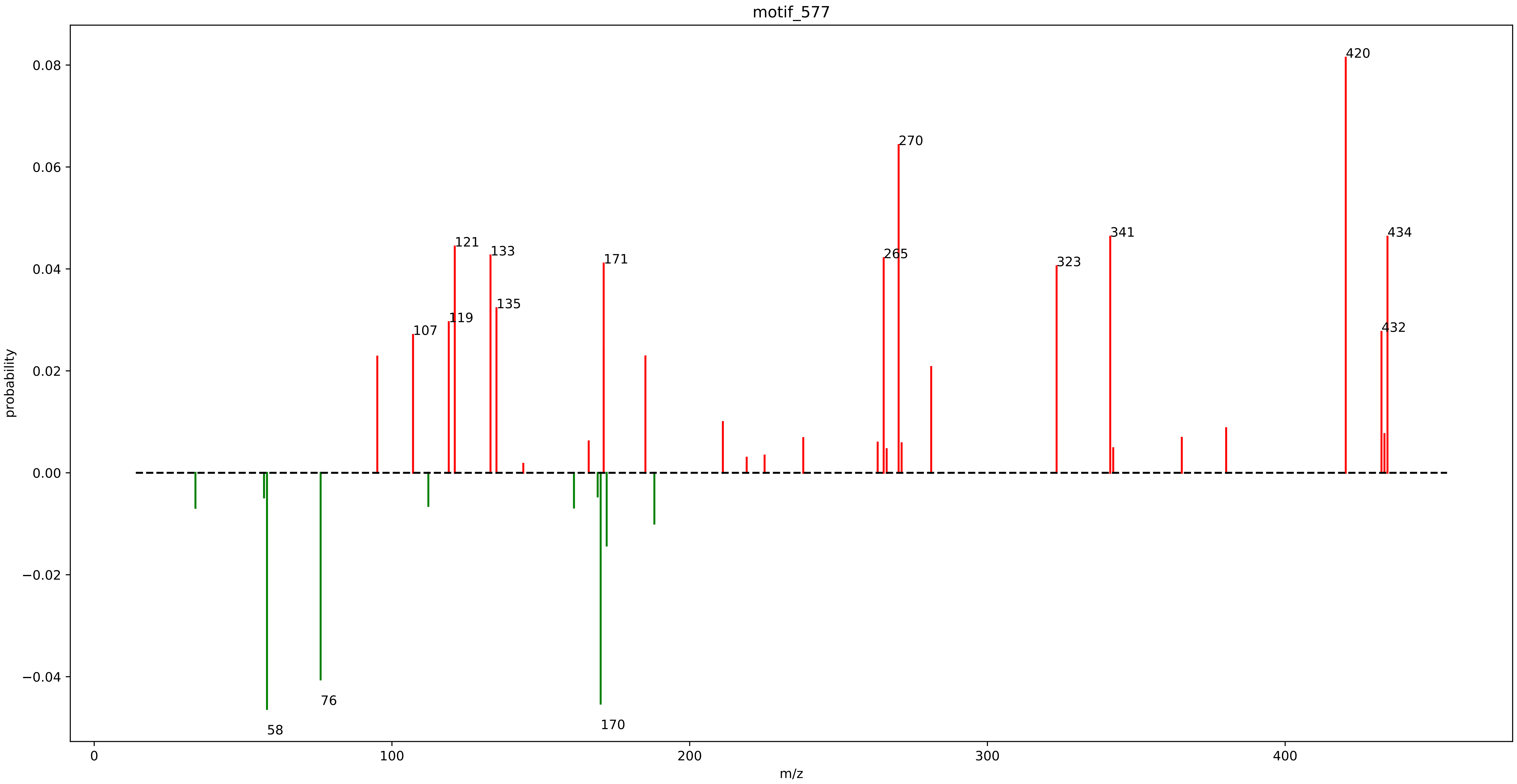

motif_577
probability
m/z
0.08
0.06
0.04
0.02
0.00
−0.02
−0.04
0
100
200
300
400
420
270
341
434
121
133
171
265
323
135
119
107
432
58
76
170

motif_577

fragment_420.3475	: 0.08
fragment_270.1775	: 0.06
fragment_341.2475	: 0.05
loss_58.0375	: 0.05
fragment_434.3625	: 0.05
loss_170.1025	: 0.05
fragment_121.1025	: 0.04
fragment_133.1025	: 0.04
fragment_265.1525	: 0.04
fragment_171.1125	: 0.04
fragment_323.2375	: 0.04
loss_76.0525	: 0.04
fragment_135.1075	: 0.03
fragment_119.0825	: 0.03
fragment_432.3475	: 0.03
fragment_107.0825	: 0.03
fragment_171.1175	: 0.02
fragment_185.1325	: 0.02
fragment_95.0825	: 0.02
fragment_281.1125	: 0.02
loss_172.1225	: 0.01
fragment_211.1475	: 0.01
loss_188.1375	: 0.01
fragment_380.1825	: 0.01
fragment_433.3475	: 0.01
loss_34.0025	: 0.01
fragment_365.2825	: 0.01
loss_161.1375	: 0.01
fragment_238.1475	: 0.01
loss_112.2275	: 0.01
fragment_166.0875	: 0.01
fragment_263.1375	: 0.01
fragment_271.1825	: 0.01

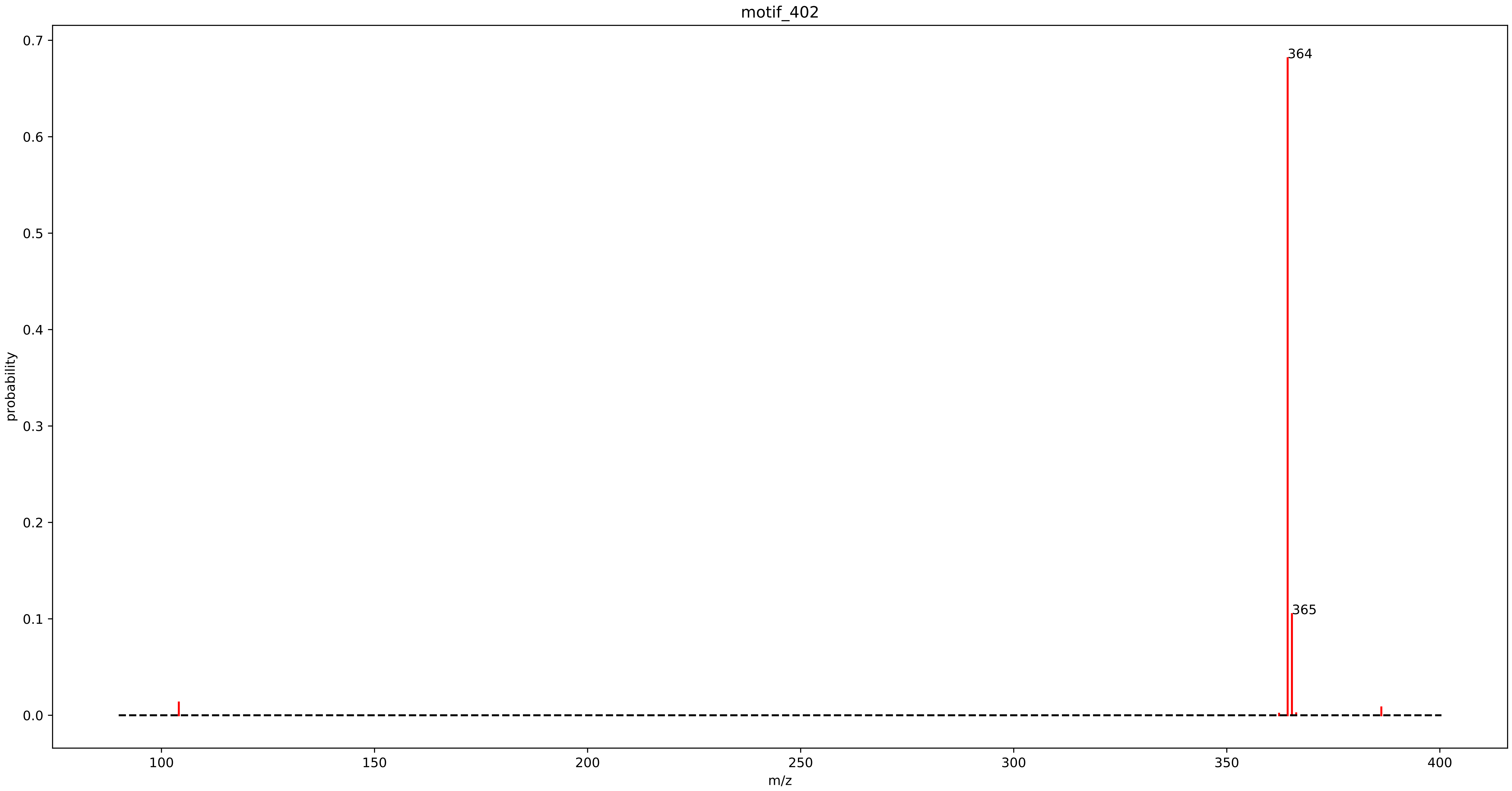

motif_402
364
365
probability
m/z
0.0
0.1
0.2
0.3
0.4
0.5
0.6
0.7
100
150
200
250
300
350
400

motif_402

fragment_364.2825	: 0.68
fragment_365.2875	: 0.10
fragment_104.0725	: 0.01
fragment_386.2675	: 0.01

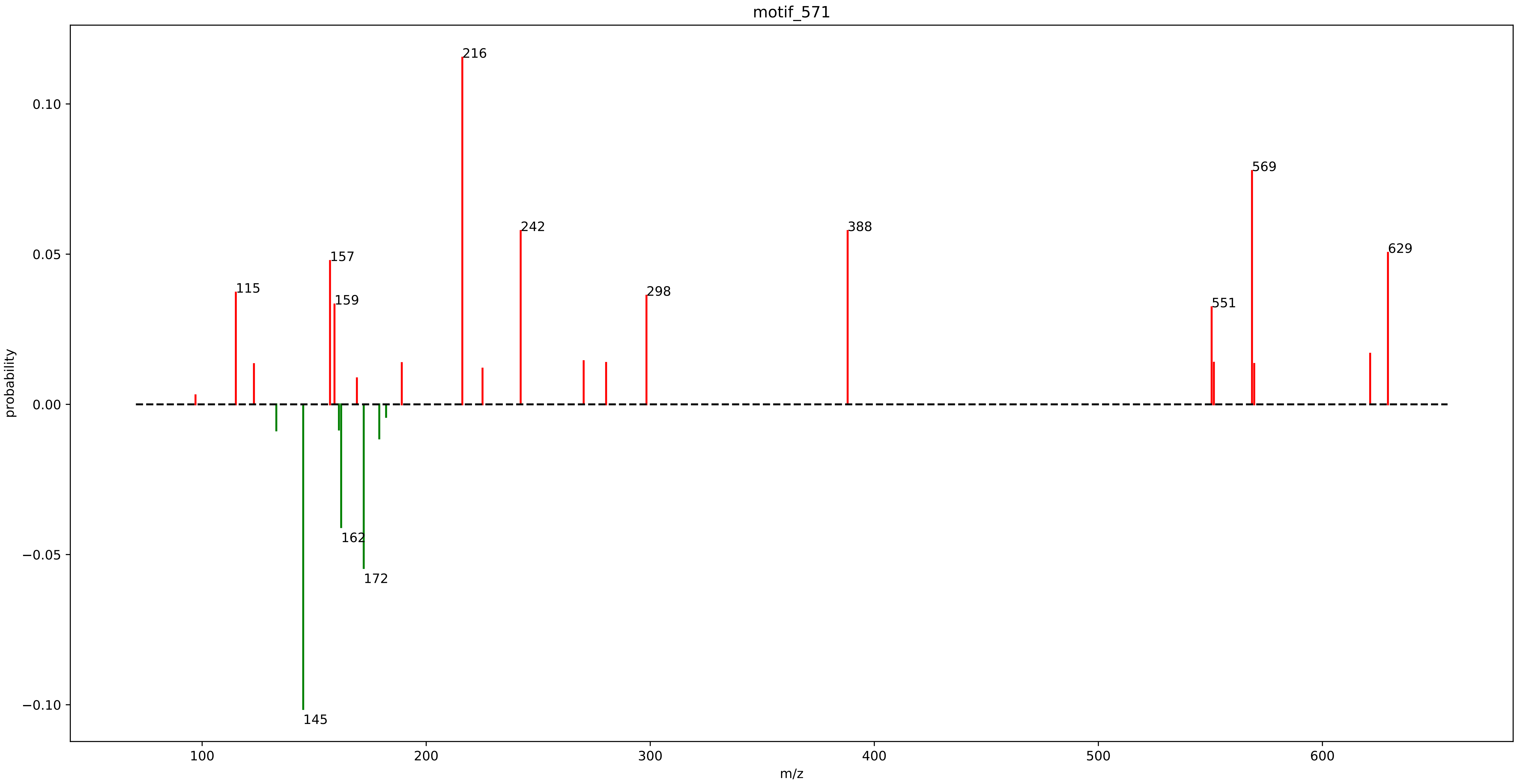

motif_571
0.10
0.05
0.00
−0.05
−0.10
probability
100
200
300
400
500
600
m/z
216
569
242
388
629
157
115
159
298
551
162
172
145

motif_571

fragment_216.1225	: 0.12
loss_145.1075	: 0.10
fragment_568.5675	: 0.08
fragment_388.0975	: 0.06
fragment_242.1625	: 0.06
loss_172.1175	: 0.05
fragment_629.2425	: 0.05
fragment_157.0725	: 0.05
loss_162.0475	: 0.04
fragment_115.0375	: 0.04
fragment_298.3075	: 0.04
fragment_159.0625	: 0.03
fragment_550.5525	: 0.03
fragment_621.3025	: 0.02
fragment_270.2775	: 0.01
fragment_551.5575	: 0.01
fragment_280.2975	: 0.01
fragment_189.1025	: 0.01
fragment_569.5675	: 0.01
fragment_123.1175	: 0.01
fragment_225.1375	: 0.01
loss_179.0575	: 0.01
loss_133.1075	: 0.01
fragment_169.0725	: 0.01
loss_161.0475	: 0.01

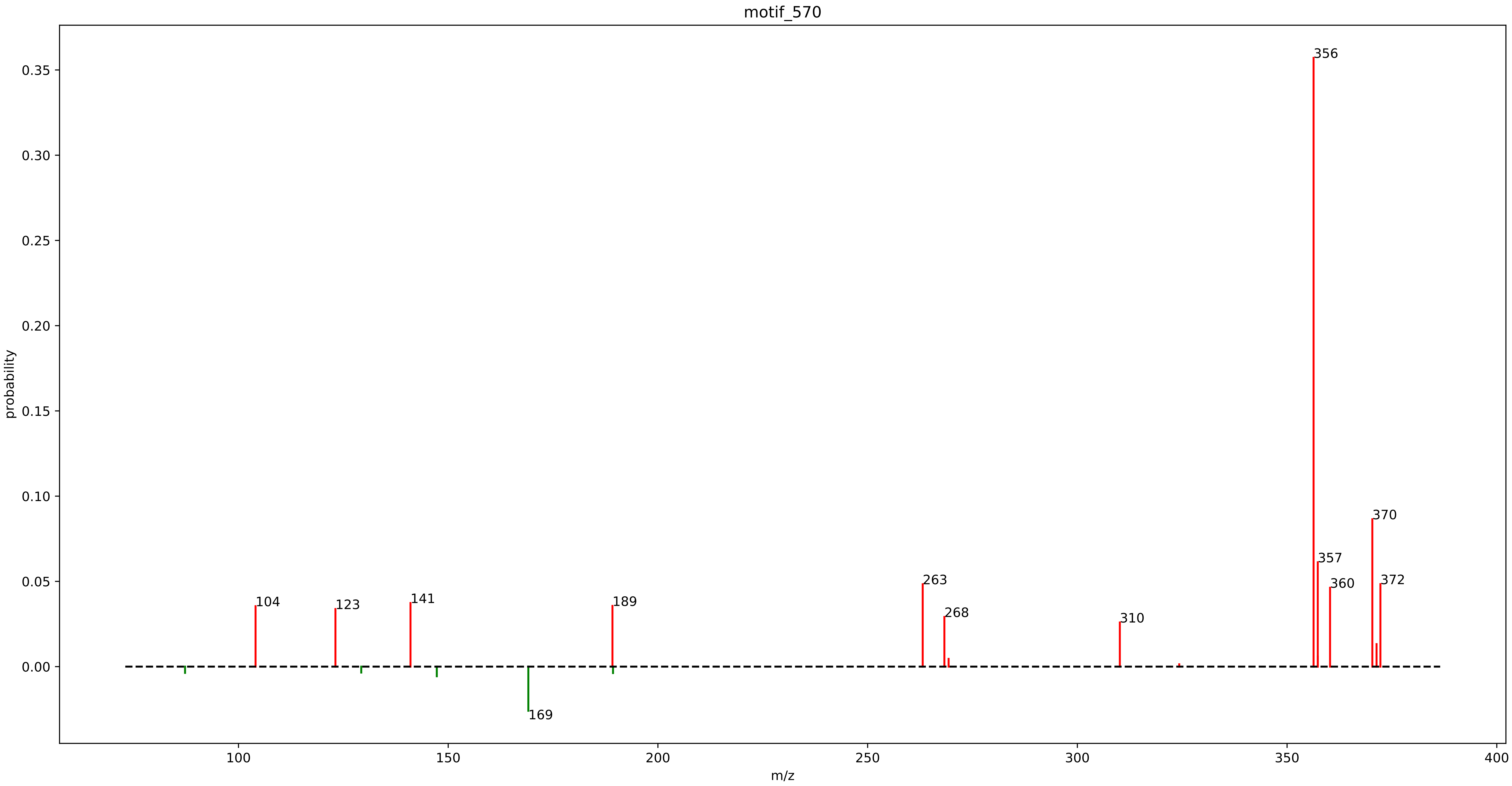

motif_570
probability
m/z
0.35
0.30
0.25
0.20
0.15
0.10
0.05
0.00
100
150
200
250
300
350
400
356
370
357
263
360
372
104
123
141
189
268
310
169

motif_570

fragment_356.3175	: 0.36
fragment_370.3275	: 0.09
fragment_357.3175	: 0.06
fragment_372.2525	: 0.05
fragment_263.1275	: 0.05
fragment_360.2475	: 0.05
fragment_141.0125	: 0.04
fragment_189.1625	: 0.04
fragment_104.0725	: 0.04
fragment_123.1175	: 0.03
fragment_268.2975	: 0.03
loss_169.1075	: 0.03
fragment_310.1275	: 0.03
fragment_371.3325	: 0.01
fragment_357.3225	: 0.01
loss_147.2825	: 0.01

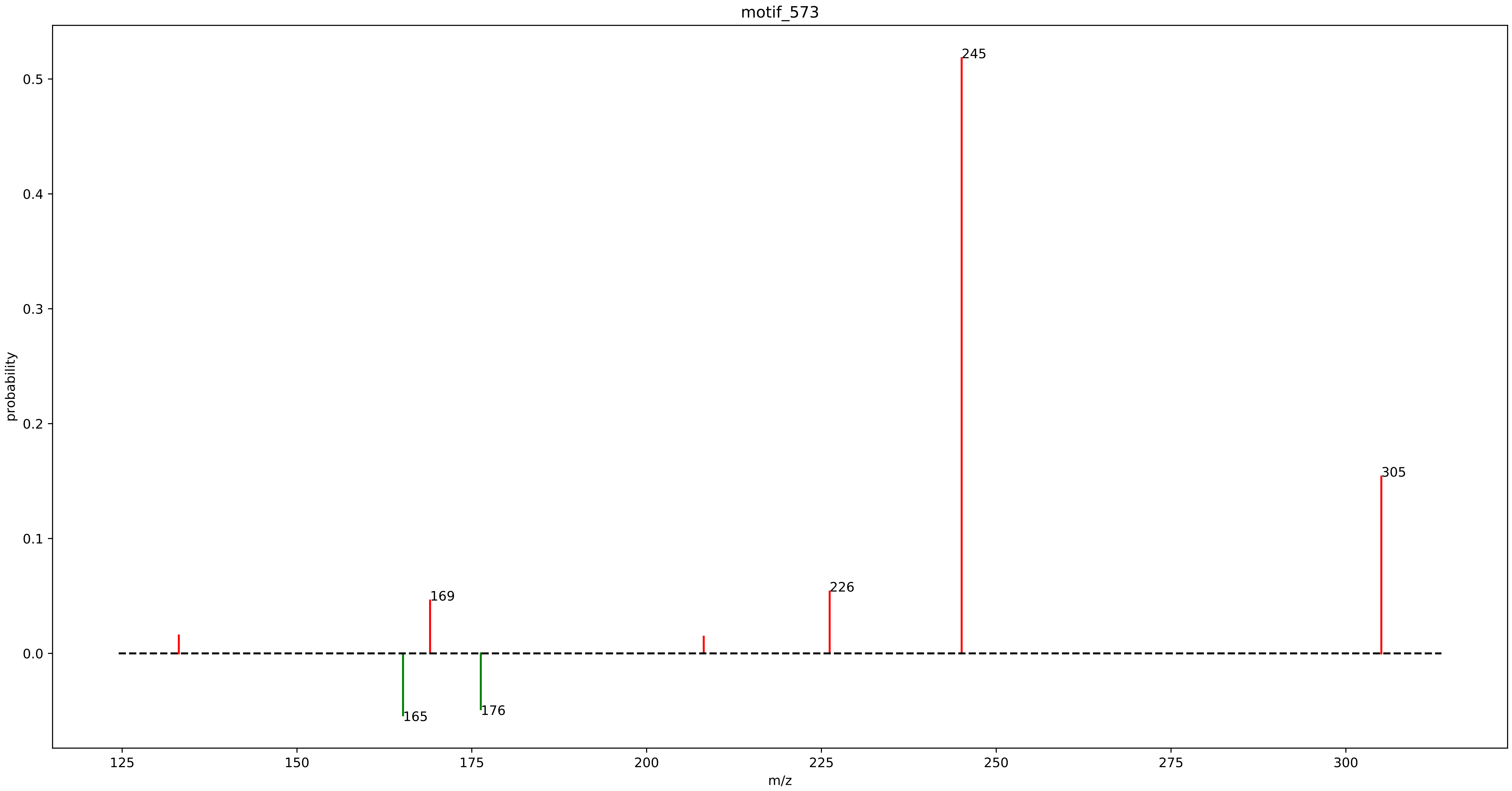

motif_573
probability
m/z
245
305
169
226
165
176
0.5
0.4
0.3
0.2
0.1
0.0
125
150
175
200
225
250
275
300

motif_573

fragment_245.0525	: 0.52
fragment_305.0725	: 0.15
loss_165.1675	: 0.05
fragment_226.1775	: 0.05
loss_176.2875	: 0.05
fragment_169.0325	: 0.05
fragment_133.1025	: 0.02
fragment_208.1675	: 0.01

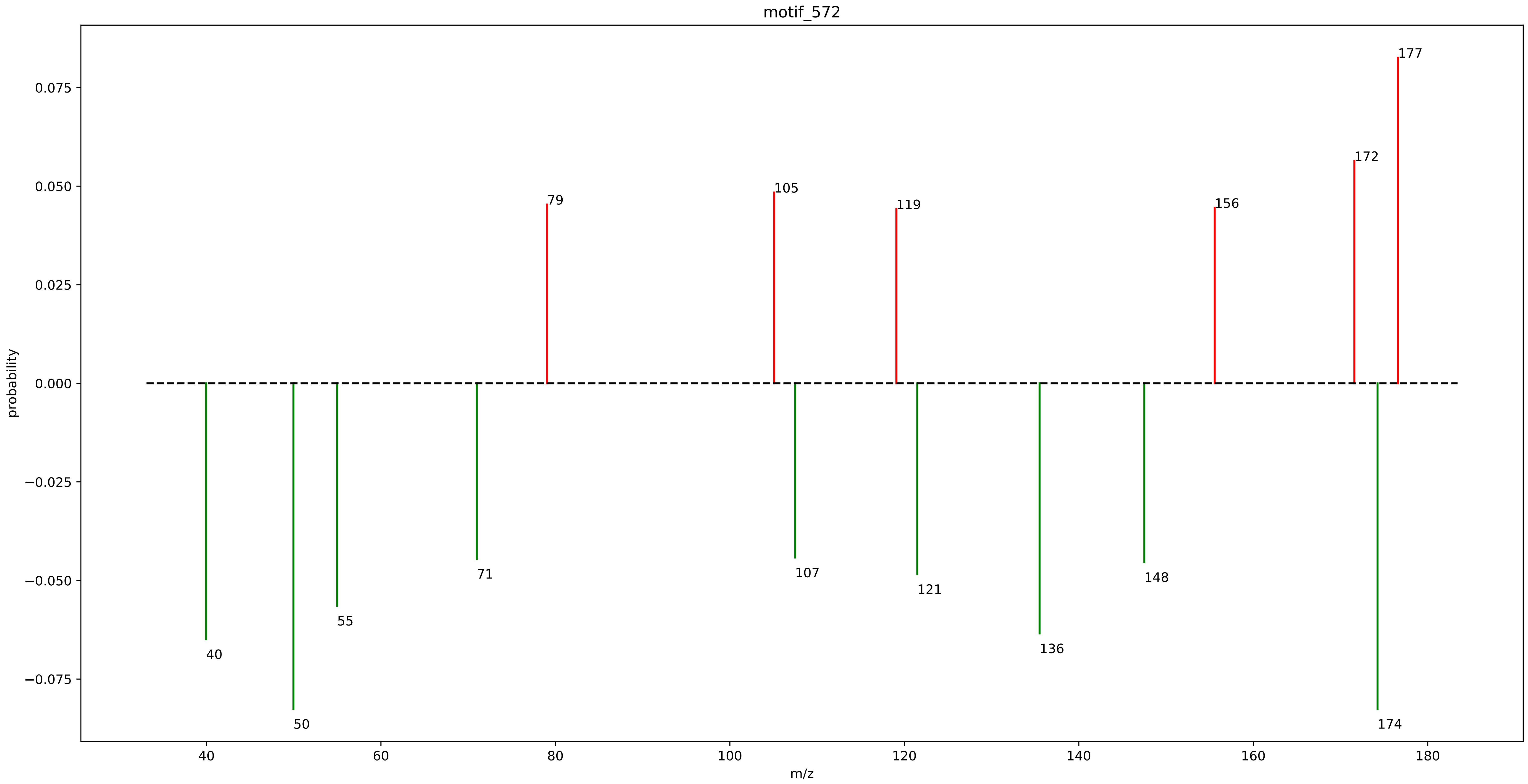

motif_572
probability
m/z
0.075
0.050
0.025
0.000
−0.025
−0.050
−0.075
40
60
80
100
120
140
160
180
79
105
119
156
172
177
40
50
55
71
107
121
136
148
174

motif_572

loss_49.9675	: 0.08
fragment_176.5875	: 0.08
loss_174.2375	: 0.08
loss_39.9525	: 0.06
loss_135.5025	: 0.06
fragment_171.5825	: 0.06
loss_54.9775	: 0.06
fragment_105.0725	: 0.05
loss_121.4875	: 0.05
loss_147.5025	: 0.05
fragment_79.0525	: 0.05
loss_70.9875	: 0.04
fragment_155.5675	: 0.04
fragment_119.0825	: 0.04
loss_107.4725	: 0.04

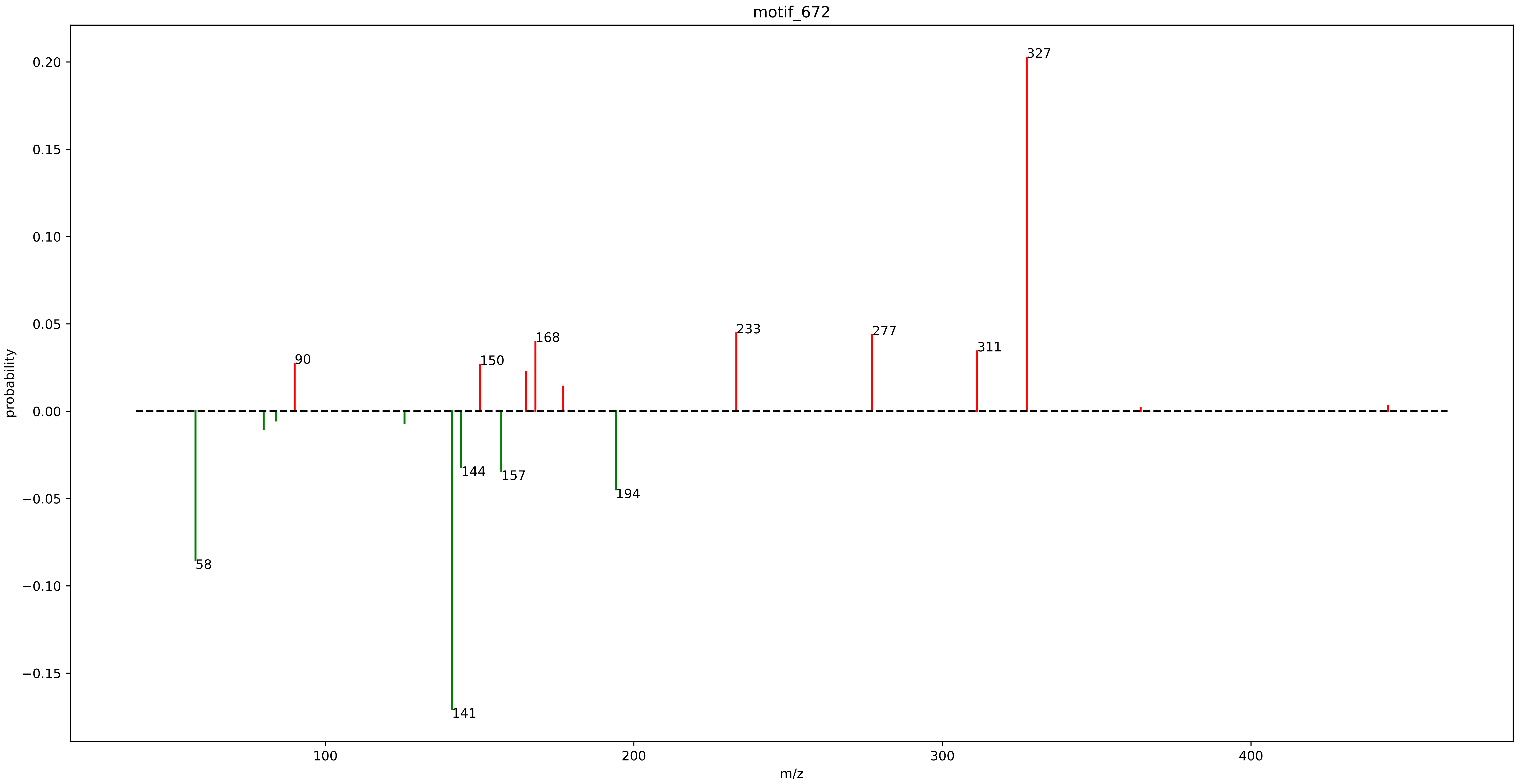

motif_672
0.20
0.15
0.10
0.05
0.00
−0.05
−0.10
−0.15
probability
100
200
300
400
m/z
327
233
277
311
168
150
90
144
157
194
58
141

motif_672

fragment_327.2875	: 0.20
loss_141.0175	: 0.17
loss_57.9175	: 0.09
loss_194.1125	: 0.04
fragment_233.1875	: 0.04
fragment_277.2125	: 0.04
fragment_168.0775	: 0.04
loss_157.0375	: 0.03
fragment_311.2675	: 0.03
loss_144.0425	: 0.03
fragment_90.0575	: 0.03
fragment_150.0675	: 0.03
fragment_165.0875	: 0.02
fragment_177.1125	: 0.01
loss_80.0125	: 0.01
loss_125.6525	: 0.01
loss_83.9375	: 0.01

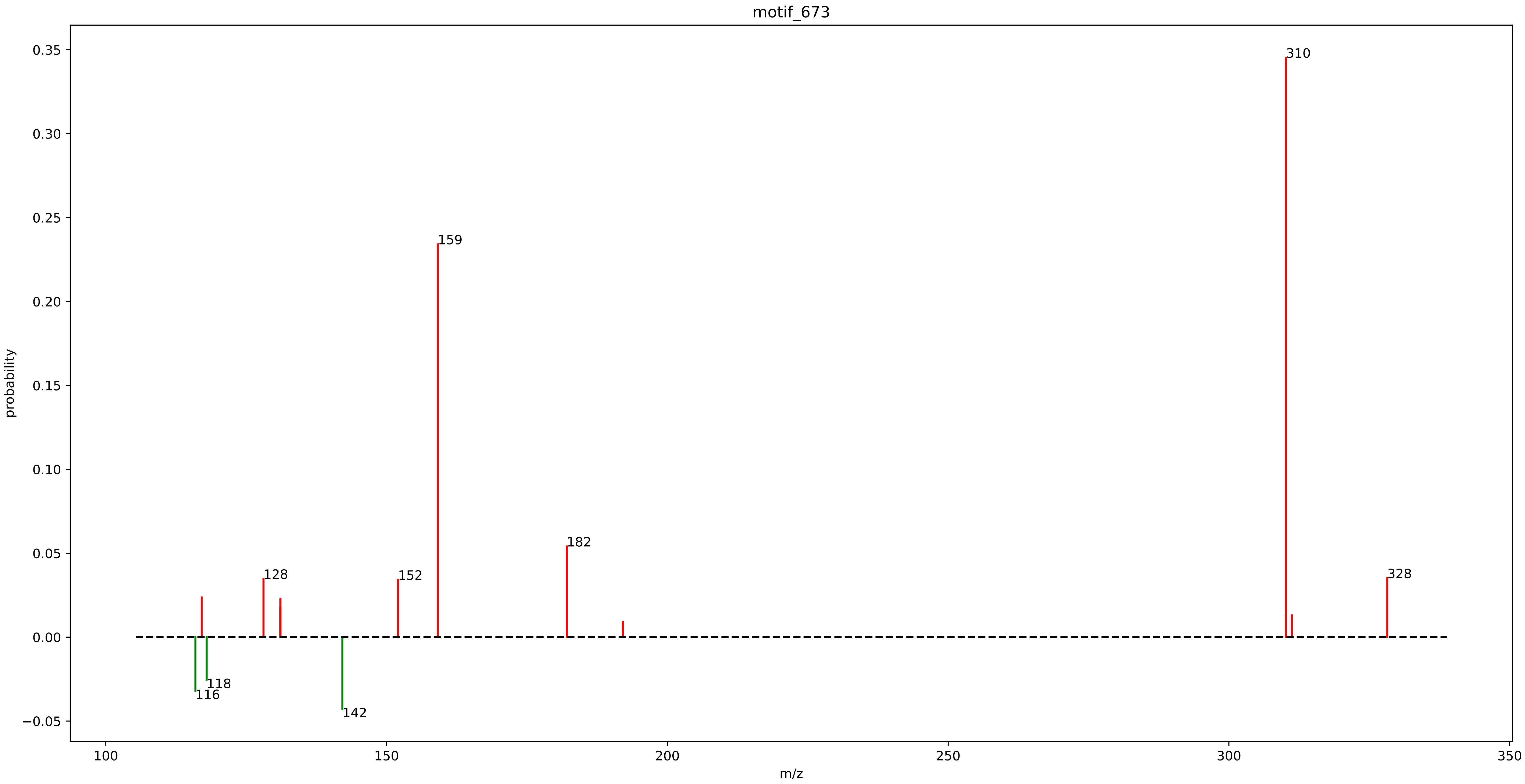

motif_673
0.35
0.30
0.25
0.20
0.15
0.10
0.05
0.00
−0.05
probability
100
150
200
250
300
350
m/z
310
159
182
128
152
328
116
118
142

motif_673

fragment_310.1775	: 0.35
fragment_159.1175	: 0.23
fragment_182.0825	: 0.05
loss_142.1275	: 0.04
fragment_328.1925	: 0.04
fragment_128.0675	: 0.03
fragment_152.0325	: 0.03
loss_115.9475	: 0.03
loss_117.9425	: 0.03
fragment_117.0675	: 0.02
fragment_131.0825	: 0.02
fragment_131.0875	: 0.02
fragment_311.1825	: 0.01
fragment_192.1025	: 0.01

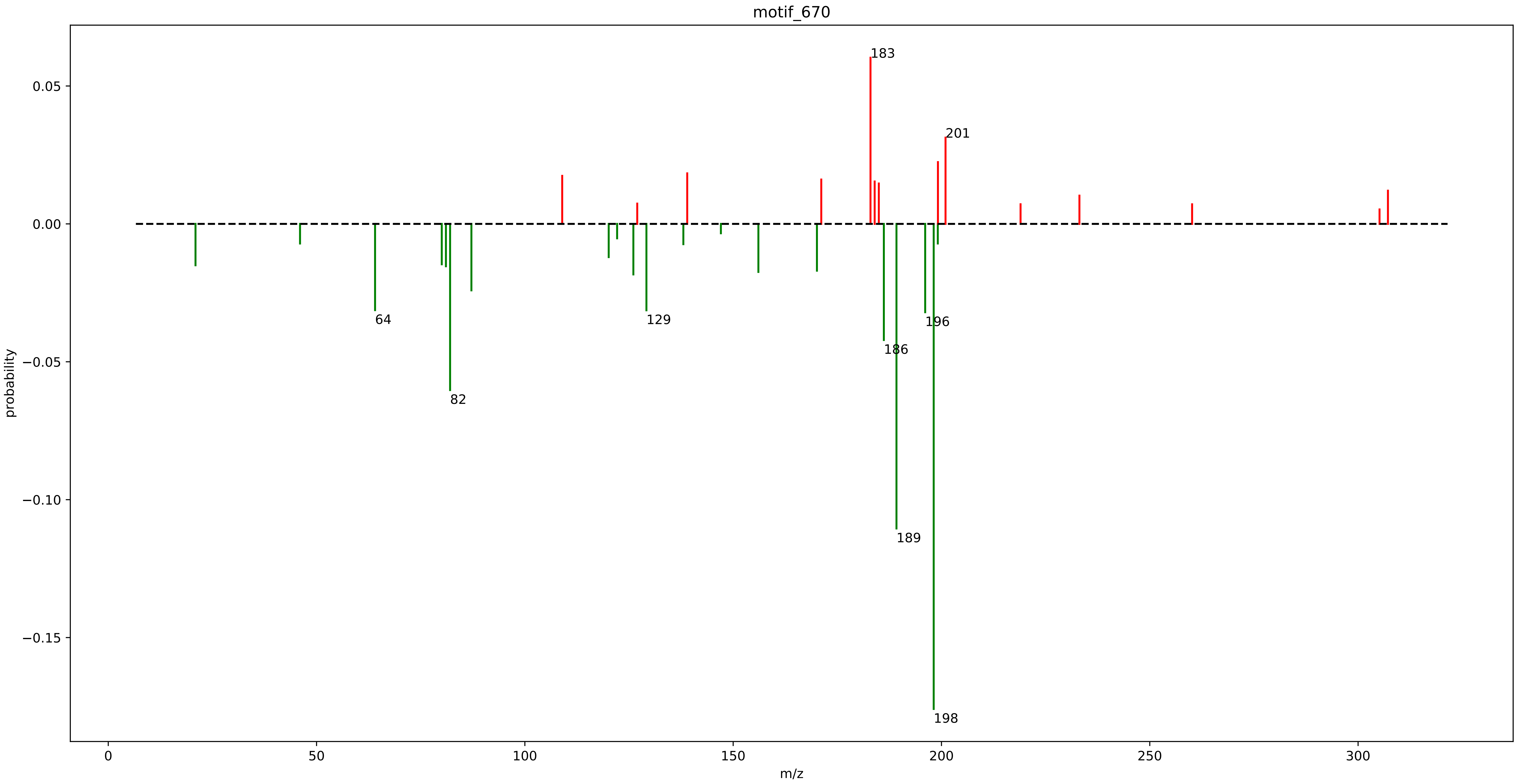
motif_670
probability
m/z
0.05
0.00
−0.05
−0.10
−0.15
0
50
100
150
200
250
300
183
201
64
82
129
196
186
189
198

motif_670

loss_198.1325	: 0.18
loss_189.1975	: 0.11
loss_82.0525	: 0.06
fragment_182.9625	: 0.06
loss_186.1675	: 0.04
loss_196.0875	: 0.03
loss_129.1775	: 0.03
fragment_200.9725	: 0.03
loss_64.0425	: 0.03
loss_87.1675	: 0.02
fragment_199.1525	: 0.02
loss_126.0425	: 0.02
fragment_138.9725	: 0.02
fragment_108.9625	: 0.02
loss_156.0525	: 0.02
loss_170.1075	: 0.02
fragment_171.1475	: 0.02
fragment_183.9625	: 0.02
loss_81.0525	: 0.02
loss_20.9425	: 0.02
loss_80.0525	: 0.01
fragment_184.9625	: 0.01
loss_120.1225	: 0.01
fragment_307.1675	: 0.01
fragment_233.1125	: 0.01
fragment_126.9725	: 0.01
loss_138.0425	: 0.01
loss_46.0325	: 0.01
fragment_218.9825	: 0.01
loss_199.1175	: 0.01
fragment_260.1575	: 0.01
fragment_305.1475	: 0.01
loss_122.1425	: 0.01

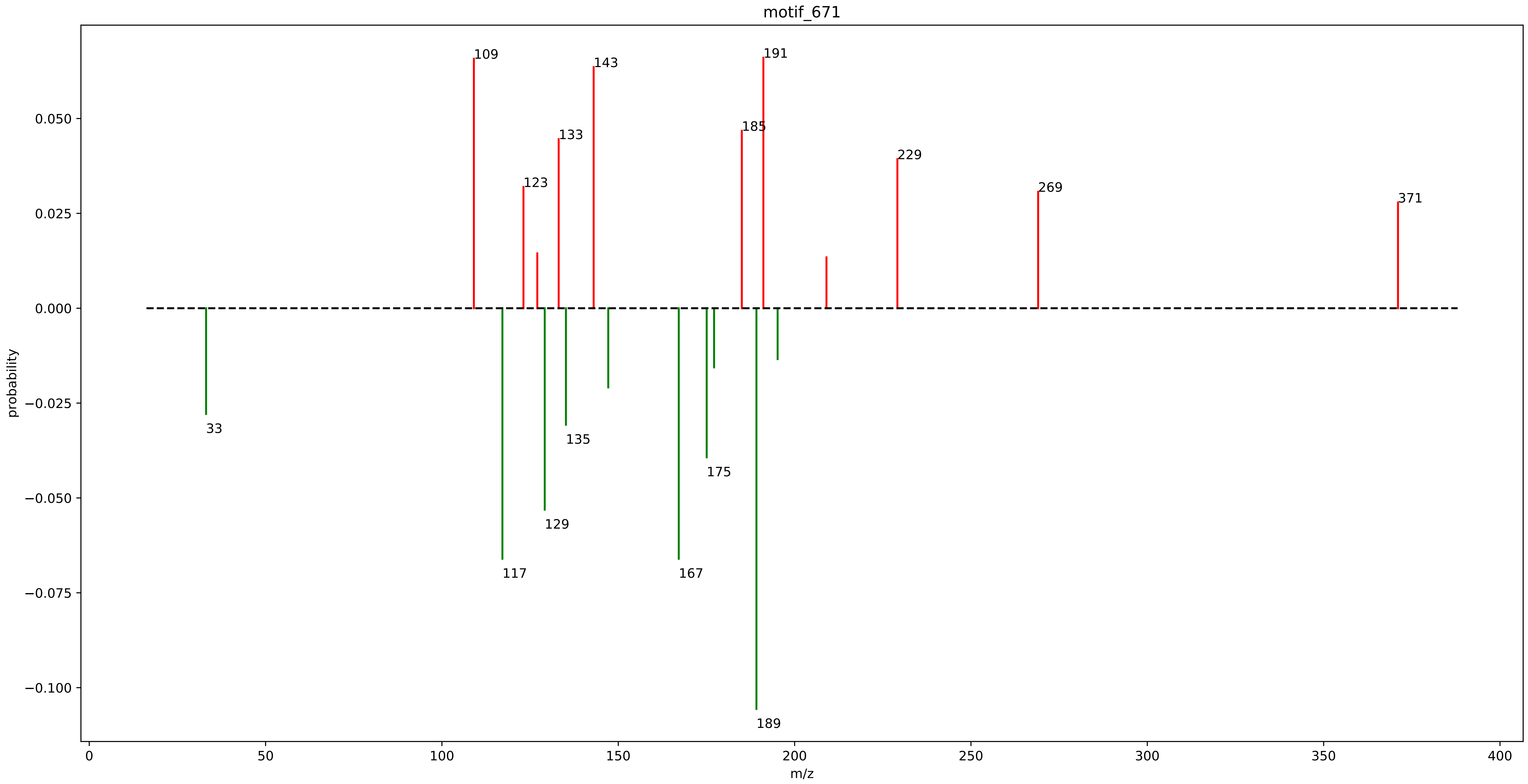
motif_671
probability
m/z
0.050
0.025
0.000
−0.025
−0.050
−0.075
−0.100
0
50
100
150
200
250
300
350
400
109
123
133
143
185
191
229
269
371
33
117
129
135
167
175
189

motif_671

loss_189.1725	: 0.11
fragment_191.1425	: 0.07
loss_167.1525	: 0.07
loss_117.1475	: 0.07
fragment_109.0575	: 0.07
fragment_143.0175	: 0.06
loss_129.1475	: 0.05
fragment_185.0275	: 0.05
fragment_133.1025	: 0.04
loss_175.0725	: 0.04
fragment_229.1375	: 0.04
fragment_123.1175	: 0.03
loss_135.1625	: 0.03
fragment_269.0475	: 0.03
loss_33.1275	: 0.03
fragment_371.0825	: 0.03
loss_147.1575	: 0.02
loss_177.1675	: 0.02
fragment_127.0375	: 0.01
loss_195.1825	: 0.01
fragment_209.0275	: 0.01

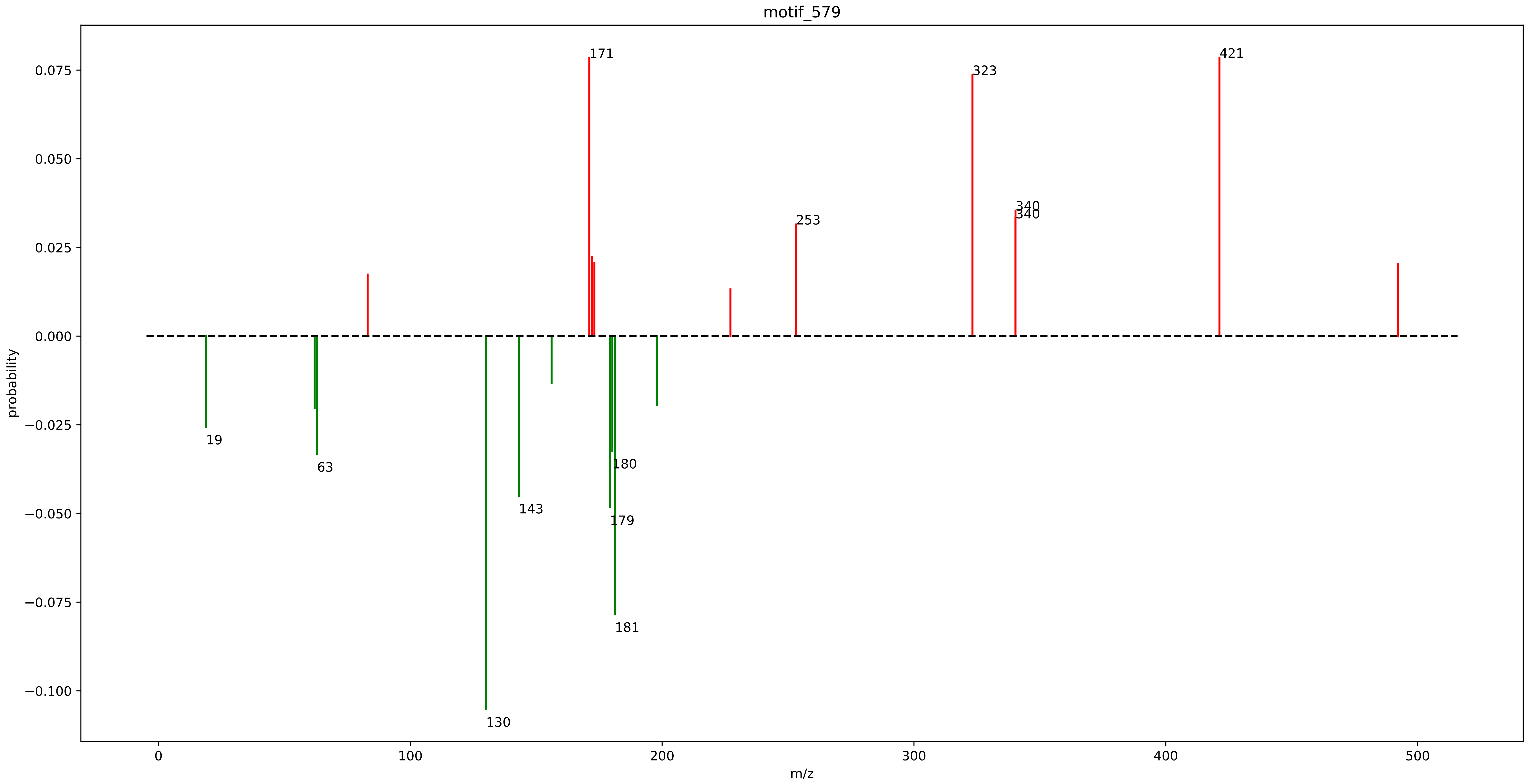

motif_579
171
323
421
340
340
253
19
63
180
143
179
181
130
0.075
0.050
0.025
0.000
−0.025
−0.050
−0.075
−0.100
probability
0
100
200
300
400
500
m/z

motif_579

loss_130.0975	: 0.11
fragment_421.3075	: 0.08
fragment_171.0925	: 0.08
loss_181.2325	: 0.08
fragment_323.2325	: 0.07
loss_179.2375	: 0.05
loss_143.1025	: 0.04
fragment_340.3225	: 0.04
loss_62.9575	: 0.03
fragment_340.2875	: 0.03
loss_180.2325	: 0.03
fragment_253.1325	: 0.03
loss_18.8975	: 0.03
fragment_172.0975	: 0.02
fragment_173.1075	: 0.02
fragment_492.2125	: 0.02
loss_62.0175	: 0.02
loss_197.9125	: 0.02
fragment_83.0475	: 0.02
loss_197.9275	: 0.02
loss_156.1125	: 0.01
fragment_227.1175	: 0.01

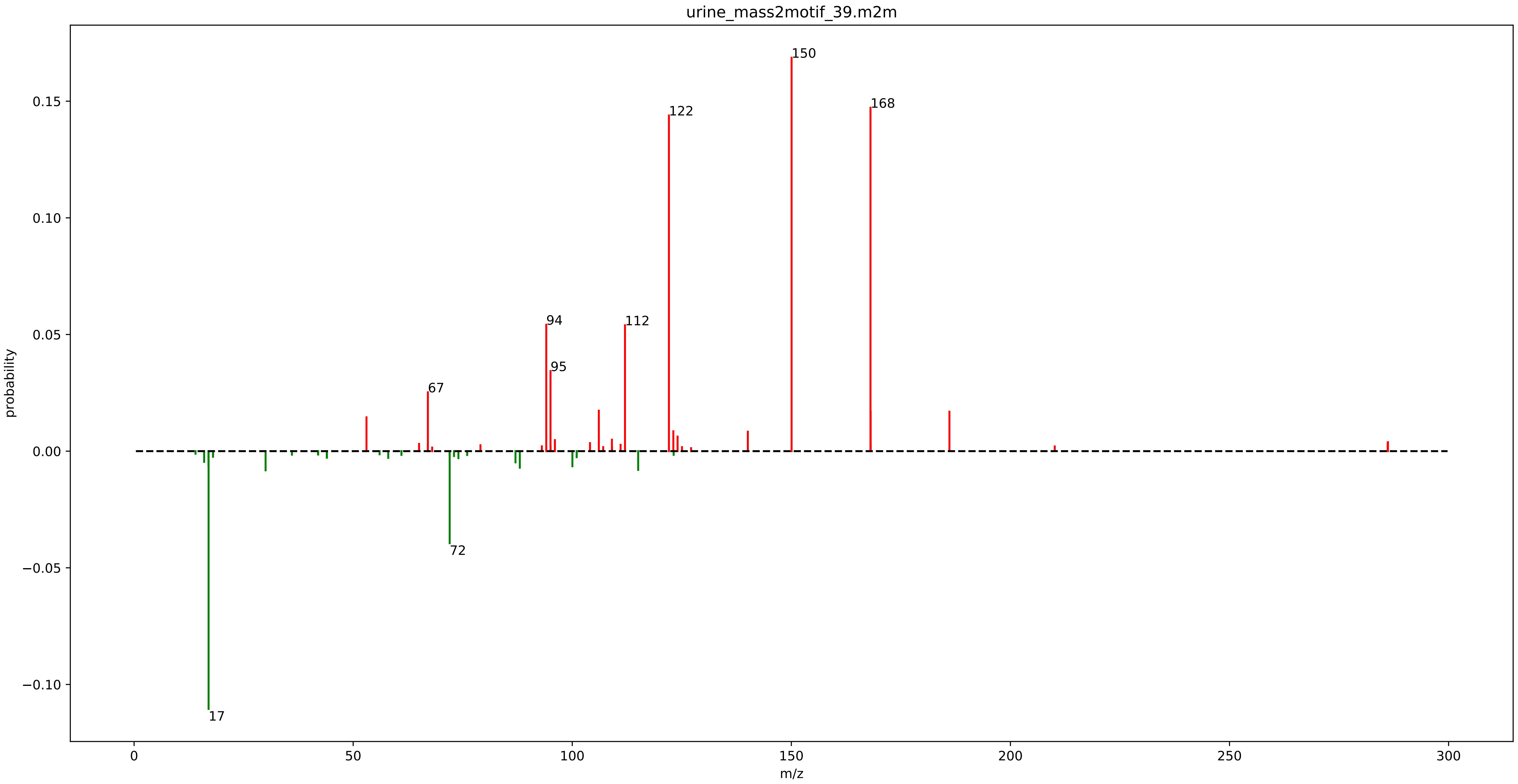
urine_mass2motif_39.m2m
probability
m/z
0.15
0.10
0.05
0.00
−0.05
−0.10
0
50
100
150
200
250
300
150
168
122
94
112
95
67
72
17

urine_mass2motif_39.m2m

fragment_150.0575	: 0.17
fragment_168.0625	: 0.15
fragment_122.0625	: 0.14
loss_17.0025	: 0.11
fragment_94.0675	: 0.05
fragment_112.0375	: 0.05
loss_72.0225	: 0.04
fragment_95.0375	: 0.03
fragment_67.0525	: 0.03
fragment_106.0675	: 0.02
fragment_186.0775	: 0.02
fragment_168.1025	: 0.02
fragment_53.0375	: 0.01
fragment_123.0675	: 0.01
fragment_140.0725	: 0.01
loss_30.0125	: 0.01
loss_115.0425	: 0.01
loss_88.0325	: 0.01
loss_100.0325	: 0.01
fragment_124.0375	: 0.01
loss_16.9775	: 0.01

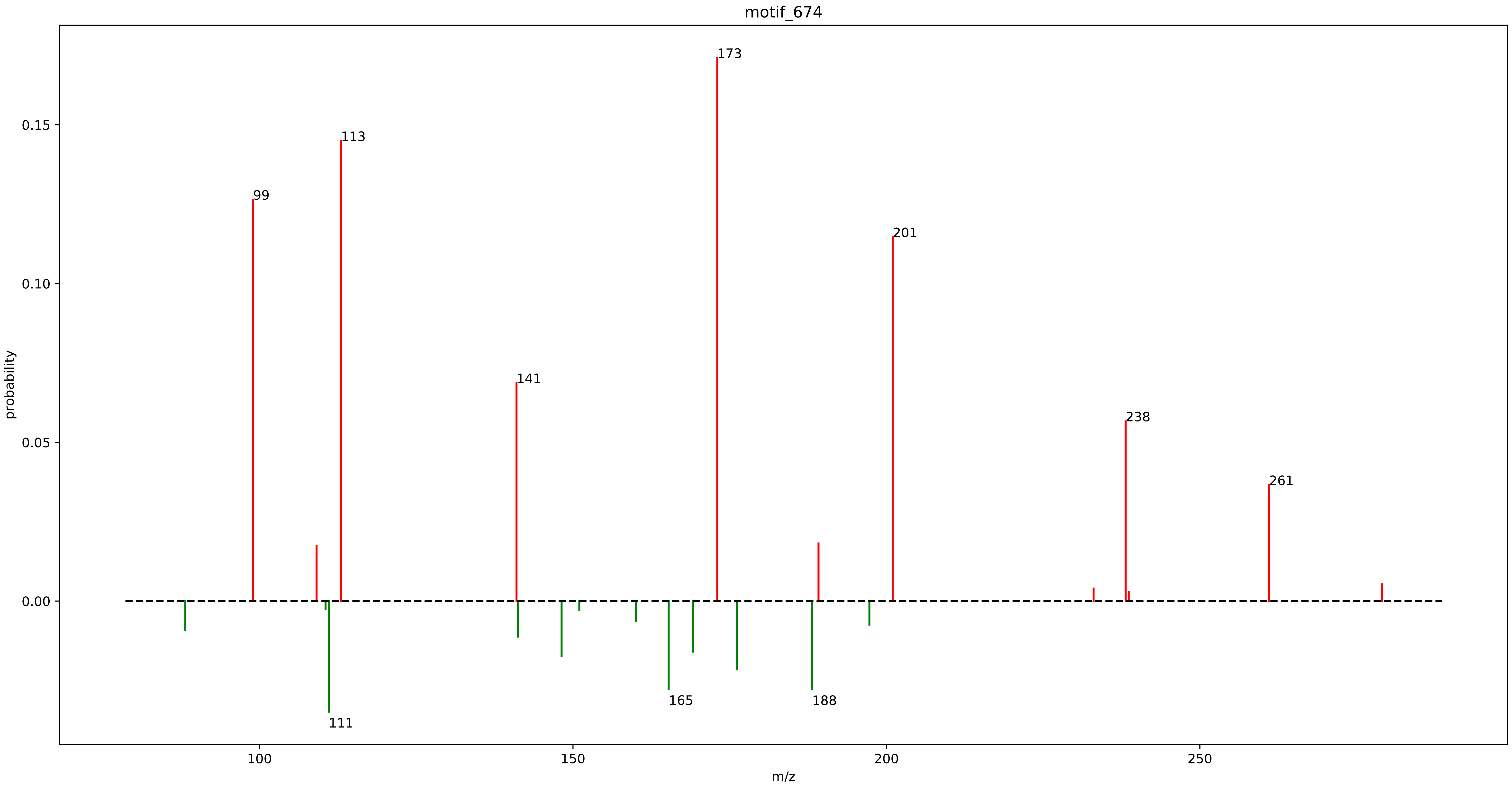

motif_674
probability
m/z
0.15
0.10
0.05
0.00
100
150
200
250
99
113
141
173
201
238
261
111
165
188

motif_674

fragment_173.0125	: 0.17
fragment_112.9875	: 0.14
fragment_98.9725	: 0.13
fragment_201.0075	: 0.11
fragment_140.9875	: 0.07
fragment_238.1475	: 0.06
fragment_261.0275	: 0.04
loss_111.0375	: 0.03
loss_188.1425	: 0.03
loss_165.2575	: 0.03
loss_176.1725	: 0.02
fragment_189.1625	: 0.02
fragment_109.0975	: 0.02
loss_148.1775	: 0.02
loss_169.1825	: 0.02
loss_141.1875	: 0.01
loss_88.1525	: 0.01
loss_197.2825	: 0.01
loss_160.0225	: 0.01
fragment_279.0375	: 0.01

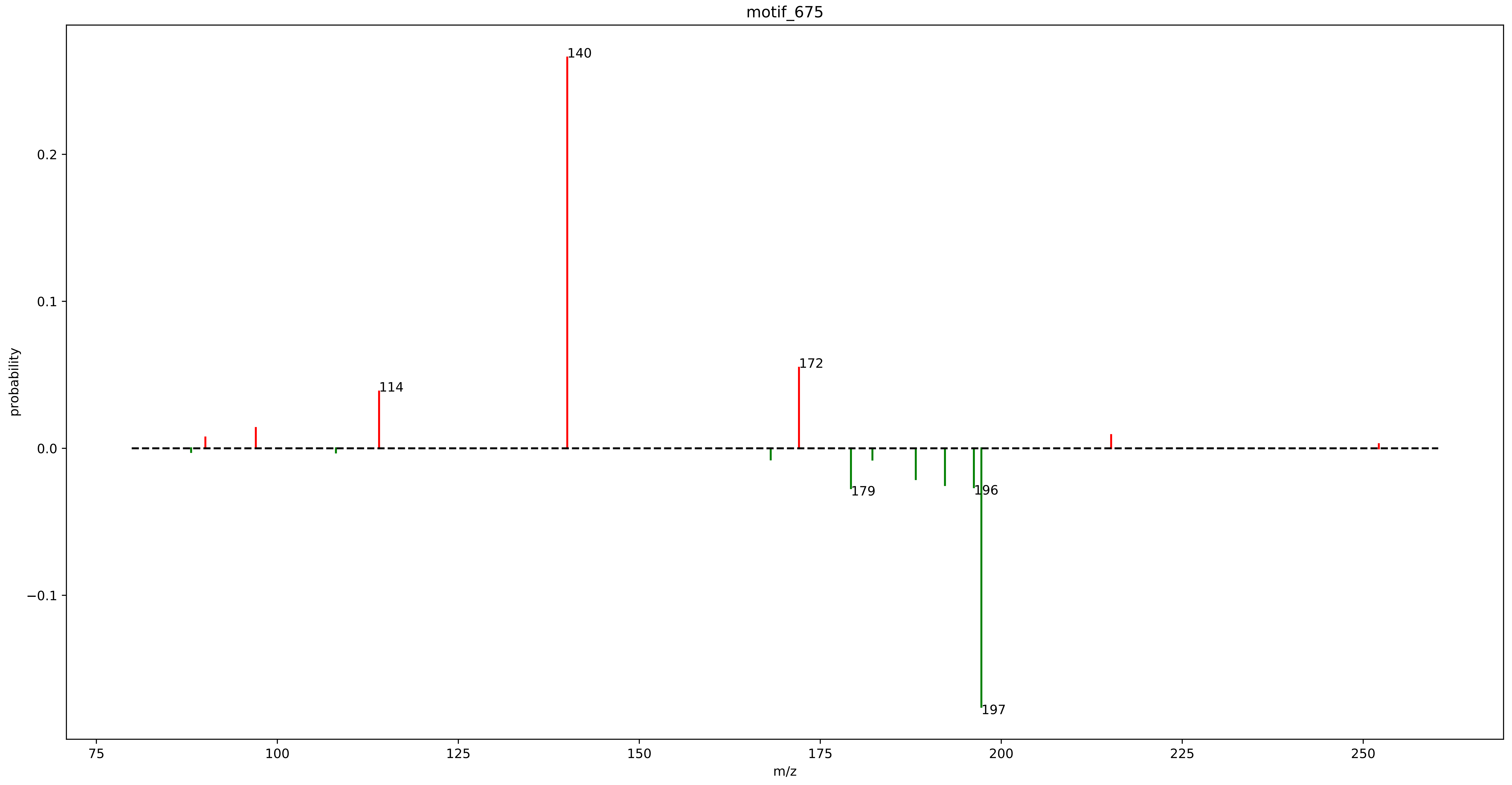

motif_675
140
172
114
179
196
197
probability
0.2
0.1
0.0
−0.1
75
100
125
150
175
200
225
250
m/z

motif_675

fragment_140.0475	: 0.27
loss_197.2525	: 0.18
fragment_172.0575	: 0.05
fragment_114.0525	: 0.04
loss_179.2375	: 0.03
loss_196.2175	: 0.03
loss_192.2225	: 0.02
loss_188.1925	: 0.02
fragment_97.0275	: 0.01
fragment_215.1775	: 0.01
loss_182.2025	: 0.01
loss_168.1525	: 0.01
fragment_90.0575	: 0.01

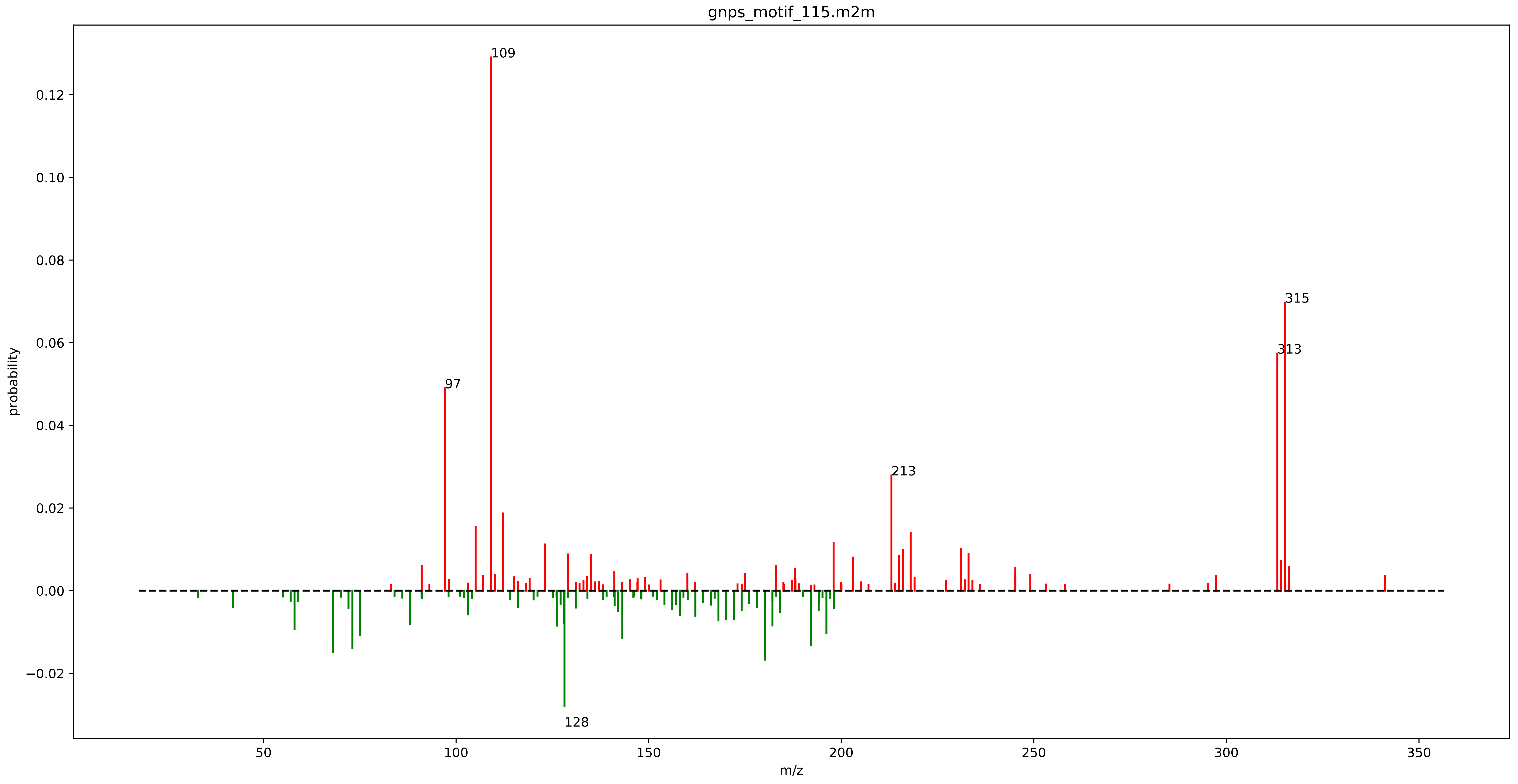

gnps_motif_115.m2m
probability
m/z
109
97
315
313
213
128
0.12
0.10
0.08
0.06
0.04
0.02
0.00
−0.02
50
100
150
200
250
300
350

gnps_motif_115.m2m

fragment_109.0675	: 0.13
fragment_315.2325	: 0.07
fragment_313.2175	: 0.06
fragment_97.0675	: 0.05
fragment_213.0325	: 0.03
loss_128.1325	: 0.03
fragment_112.1125	: 0.02
loss_180.1525	: 0.02
fragment_105.0725	: 0.02
loss_68.0275	: 0.01
fragment_218.0225	: 0.01
loss_73.0675	: 0.01
loss_192.1525	: 0.01
fragment_198.0075	: 0.01
loss_143.1525	: 0.01
fragment_123.0825	: 0.01
loss_75.0425	: 0.01
loss_196.1475	: 0.01
fragment_231.0675	: 0.01
fragment_216.0425	: 0.01
loss_58.0425	: 0.01
fragment_233.0475	: 0.01
fragment_129.0725	: 0.01
fragment_135.0825	: 0.01
fragment_215.0275	: 0.01
loss_126.1325	: 0.01
loss_182.1275	: 0.01
loss_88.0175	: 0.01
fragment_203.0675	: 0.01
loss_128.0825	: 0.01
fragment_314.2175	: 0.01
loss_168.1125	: 0.01
loss_172.1075	: 0.01
loss_170.1325	: 0.01
loss_162.1225	: 0.01
fragment_91.0575	: 0.01
fragment_182.9825	: 0.01
loss_158.1775	: 0.01
loss_103.0375	: 0.01
fragment_316.2375	: 0.01
fragment_245.1875	: 0.01
fragment_188.0475	: 0.01
loss_184.1475	: 0.01

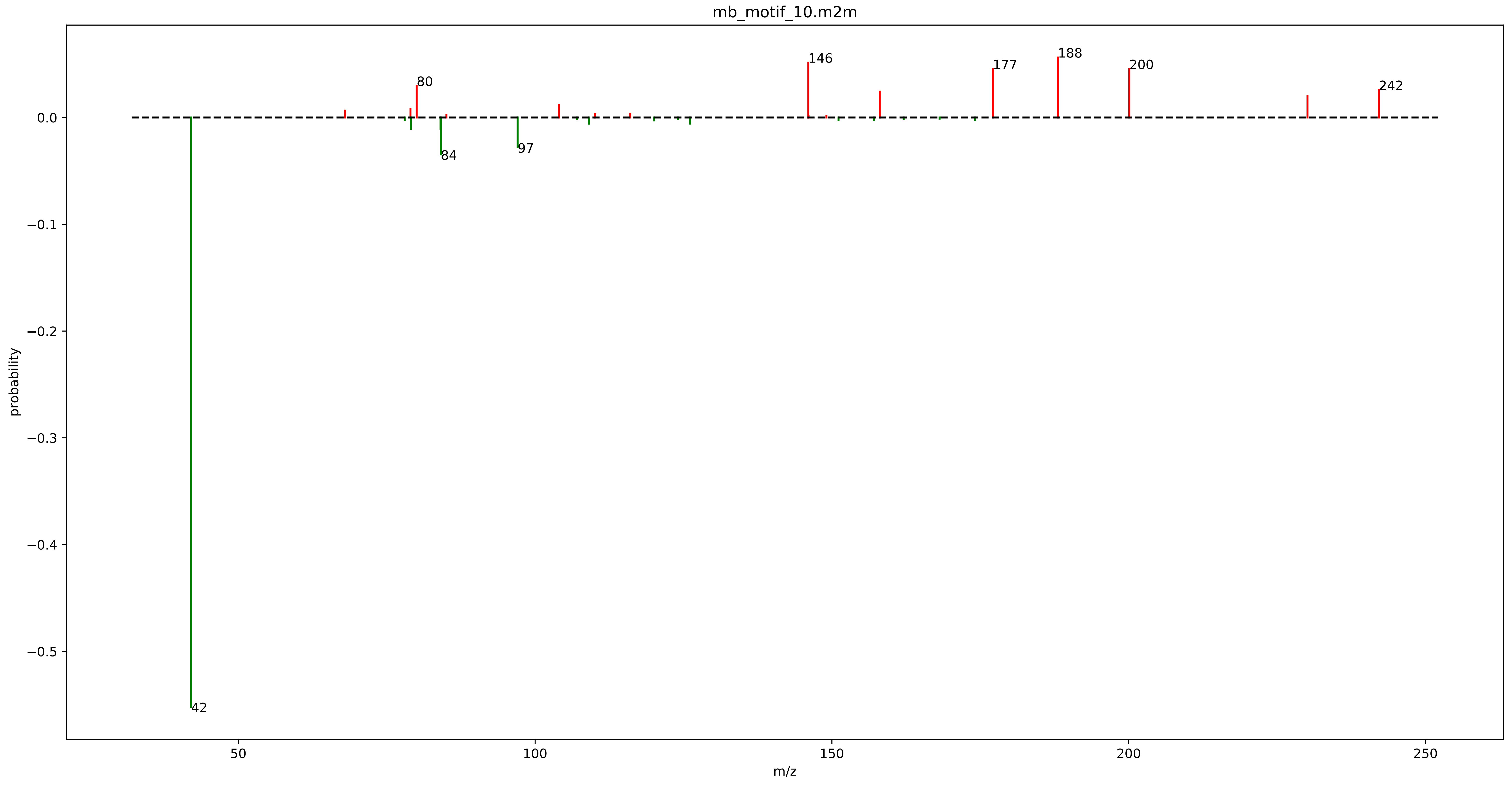

mb_motif_10.m2m
probability
m/z
0.0
−0.1
−0.2
−0.3
−0.4
−0.5
50
100
150
200
250
80
146
177
188
200
242
84
97
42

mb_motif_10.m2m

loss_42.0475	: 0.55
fragment_188.0725	: 0.06
fragment_146.0225	: 0.05
fragment_200.0975	: 0.05
fragment_177.1025	: 0.05
loss_84.0925	: 0.03
fragment_80.0475	: 0.03
loss_97.0525	: 0.03
fragment_242.1425	: 0.03
fragment_158.0475	: 0.02
fragment_230.1175	: 0.02
fragment_104.0025	: 0.01
loss_79.0425	: 0.01
loss_84.0675	: 0.01
fragment_79.0075	: 0.01
fragment_68.0225	: 0.01
loss_109.0625	: 0.01
loss_126.1175	: 0.01

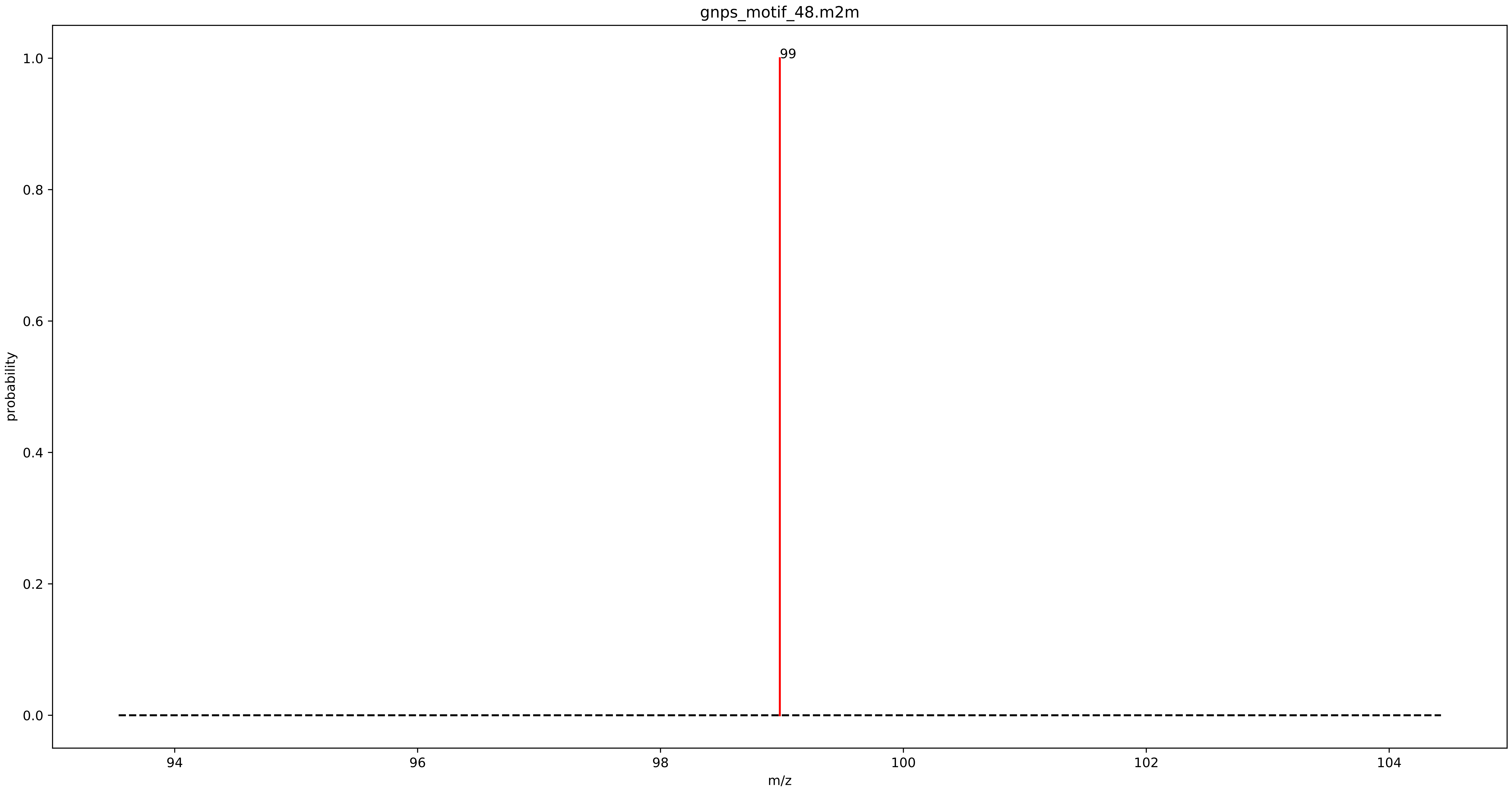

gnps_motif_48.m2m
99
1.0
0.8
0.6
0.4
0.2
0.0
probability
94
96
98
100
102
104
m/z

gnps_motif_48.m2m

fragment_98.9825 : 1.00

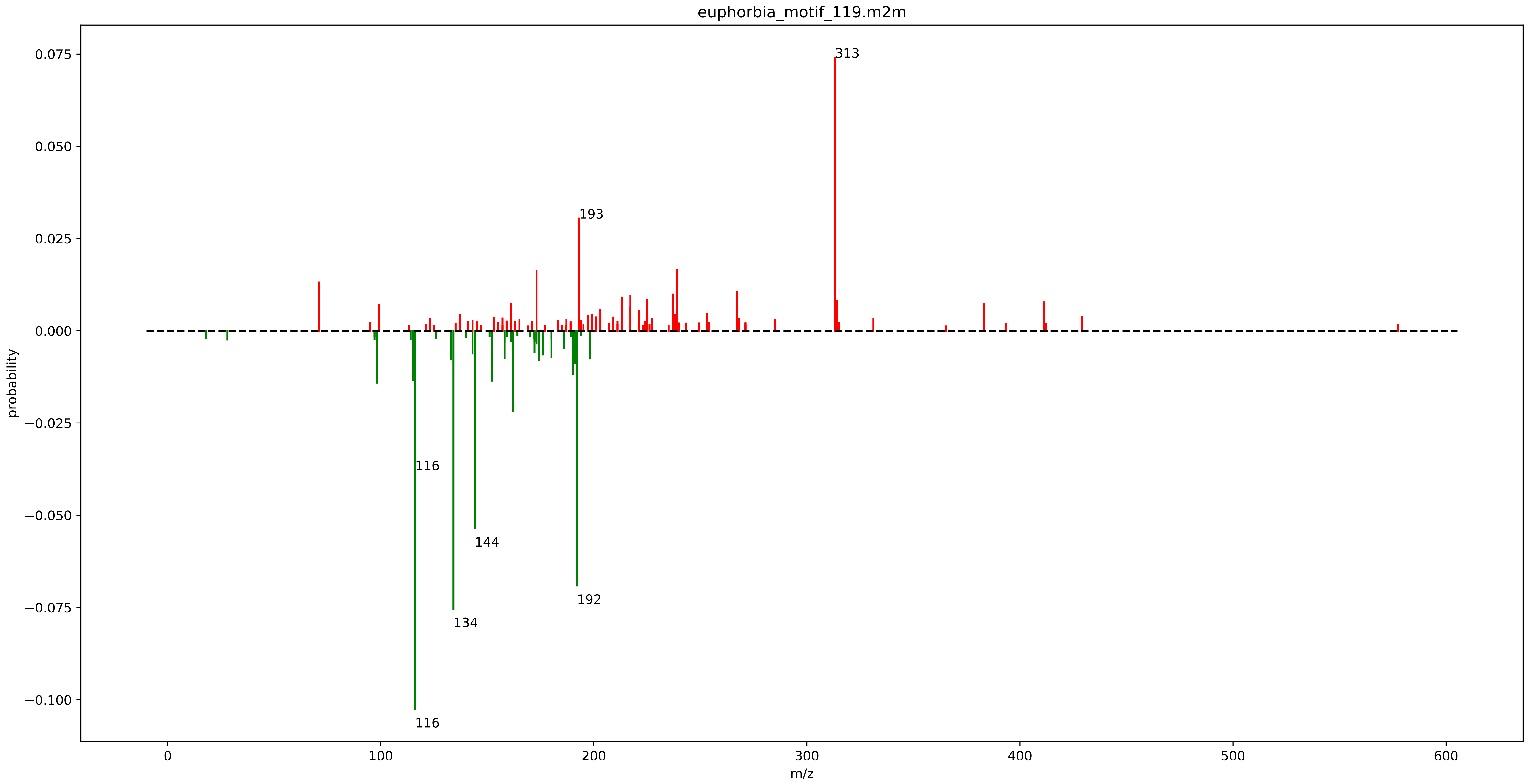

euphorbia_motif_119.m2m
probability
m/z
0.075
0.050
0.025
0.000
−0.025
−0.050
−0.075
−0.100
0
100
200
300
400
500
600
313
193
116
144
192
134
116

euphorbia_motif_119.m2m

loss_116.0825	: 0.10	loss_191.0775	: 0.01
loss_134.0975	: 0.08	fragment_225.1225	: 0.01
fragment_313.1775	: 0.07	fragment_314.1825	: 0.01
loss_192.0775	: 0.07	loss_174.1275	: 0.01
loss_144.0775	: 0.05	loss_133.0925	: 0.01
loss_116.0875	: 0.03	fragment_411.2525	: 0.01
fragment_193.0825	: 0.03	loss_198.1275	: 0.01
loss_162.0925	: 0.02	loss_158.1325	: 0.01
fragment_239.1425	: 0.02	fragment_161.0925	: 0.01
fragment_173.0975	: 0.02	fragment_383.2175	: 0.01
loss_98.0725	: 0.01	loss_180.0975	: 0.01
loss_98.0775	: 0.01	fragment_99.0775	: 0.01
loss_152.1075	: 0.01	loss_144.0825	: 0.01
loss_115.0825	: 0.01	loss_176.1425	: 0.01
fragment_71.0875	: 0.01	fragment_217.1175	: 0.01
loss_190.1225	: 0.01	loss_143.0775	: 0.01
fragment_267.1725	: 0.01	loss_172.1125	: 0.01
fragment_237.1275	: 0.01	fragment_411.2475	: 0.01
fragment_217.1225	: 0.01	fragment_203.1075	: 0.01
fragment_213.1275	: 0.01	fragment_221.1325	: 0.01

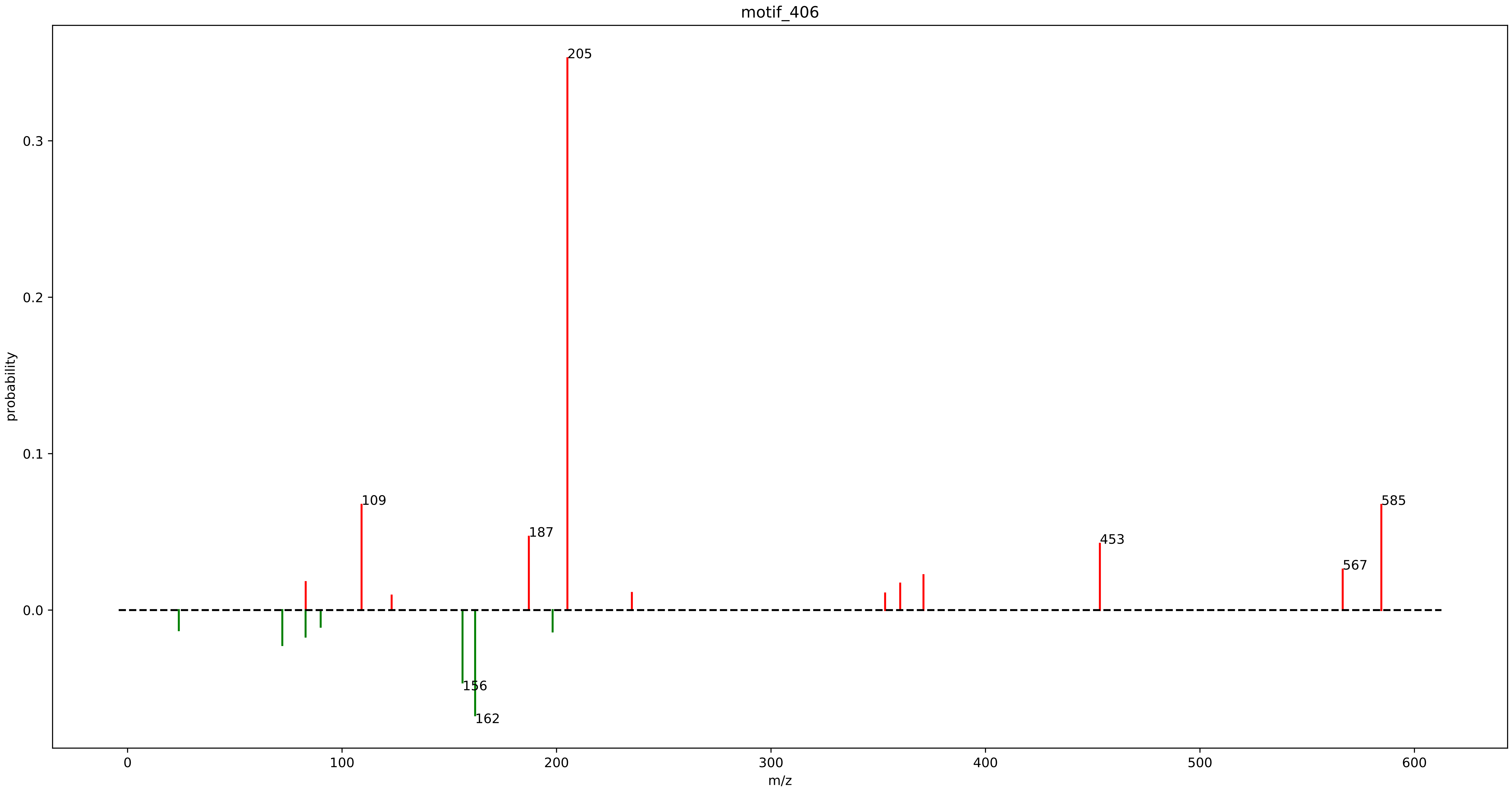

motif_406
205
109
187
453
567
585
156
162
0.3
0.2
0.1
0.0
probability
0
100
200
300
400
500
600
m/z

motif_406

fragment_205.0675	: 0.35
fragment_109.0925	: 0.07
loss_162.0475	: 0.07
fragment_584.5625	: 0.07
fragment_187.0925	: 0.05
loss_156.1675	: 0.05
fragment_453.3325	: 0.04
fragment_566.5475	: 0.03
loss_72.1425	: 0.02
fragment_371.0825	: 0.02
fragment_83.0475	: 0.02
fragment_360.2325	: 0.02
loss_82.9925	: 0.02
loss_198.1775	: 0.01
loss_23.8925	: 0.01
fragment_235.1275	: 0.01
loss_90.0325	: 0.01
fragment_353.1975	: 0.01
fragment_123.1175	: 0.01

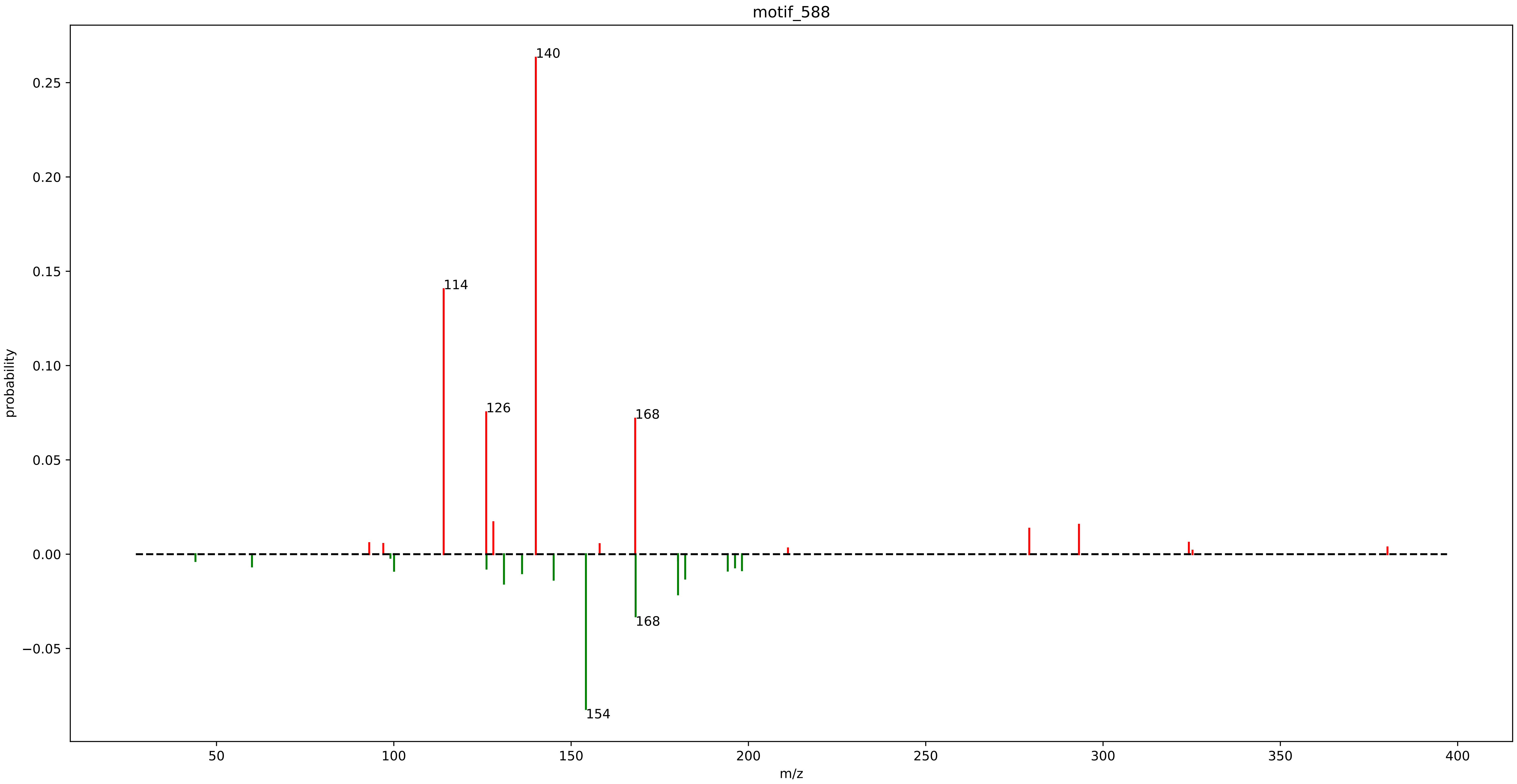

motif_588
probability
m/z
140
114
126
168
168
154
0.25
0.20
0.15
0.10
0.05
0.00
−0.05
50
100
150
200
250
300
350
400

motif_588

fragment_140.0375	: 0.26
fragment_114.0525	: 0.14
loss_154.1725	: 0.08
fragment_126.0525	: 0.08
fragment_168.0625	: 0.07
loss_168.1825	: 0.03
loss_180.1475	: 0.02
fragment_128.0675	: 0.02
fragment_293.2075	: 0.02
loss_131.0575	: 0.02
loss_145.0725	: 0.01
fragment_279.1925	: 0.01
loss_182.1675	: 0.01
loss_136.1625	: 0.01
loss_100.0725	: 0.01
loss_194.1675	: 0.01
loss_198.1725	: 0.01
loss_126.1375	: 0.01
loss_196.2175	: 0.01
loss_59.9975	: 0.01
fragment_324.1975	: 0.01
fragment_93.0675	: 0.01
fragment_97.0275	: 0.01
fragment_158.0425	: 0.01

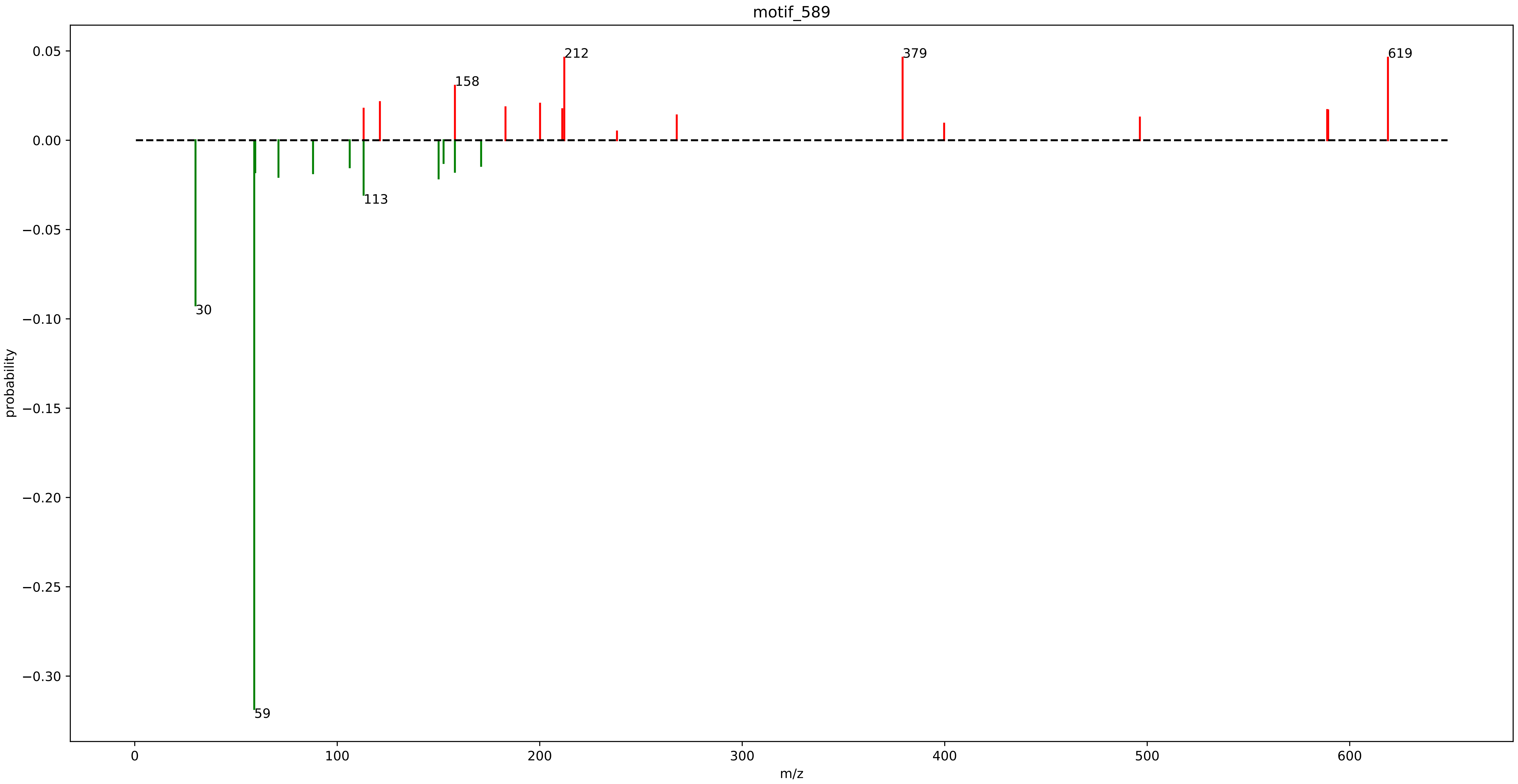

motif_589
probability
m/z
0.05
0.00
−0.05
−0.10
−0.15
−0.20
−0.25
−0.30
0
100
200
300
400
500
600
30
59
113
158
212
379
619

motif_589

loss_59.0125	: 0.32
loss_30.0075	: 0.09
fragment_212.1325	: 0.05
fragment_379.1975	: 0.05
fragment_618.8825	: 0.05
loss_113.0225	: 0.03
fragment_158.1175	: 0.03
loss_150.0675	: 0.02
fragment_121.0725	: 0.02
fragment_200.1625	: 0.02
loss_70.9775	: 0.02
fragment_183.0925	: 0.02
loss_88.0525	: 0.02
loss_59.5175	: 0.02
fragment_113.0325	: 0.02
loss_158.1075	: 0.02
fragment_211.1225	: 0.02
fragment_588.8725	: 0.02
fragment_589.3725	: 0.02
loss_106.1675	: 0.02
loss_171.0575	: 0.01
fragment_267.6625	: 0.01
loss_152.5125	: 0.01
fragment_496.3775	: 0.01
fragment_399.7075	: 0.01

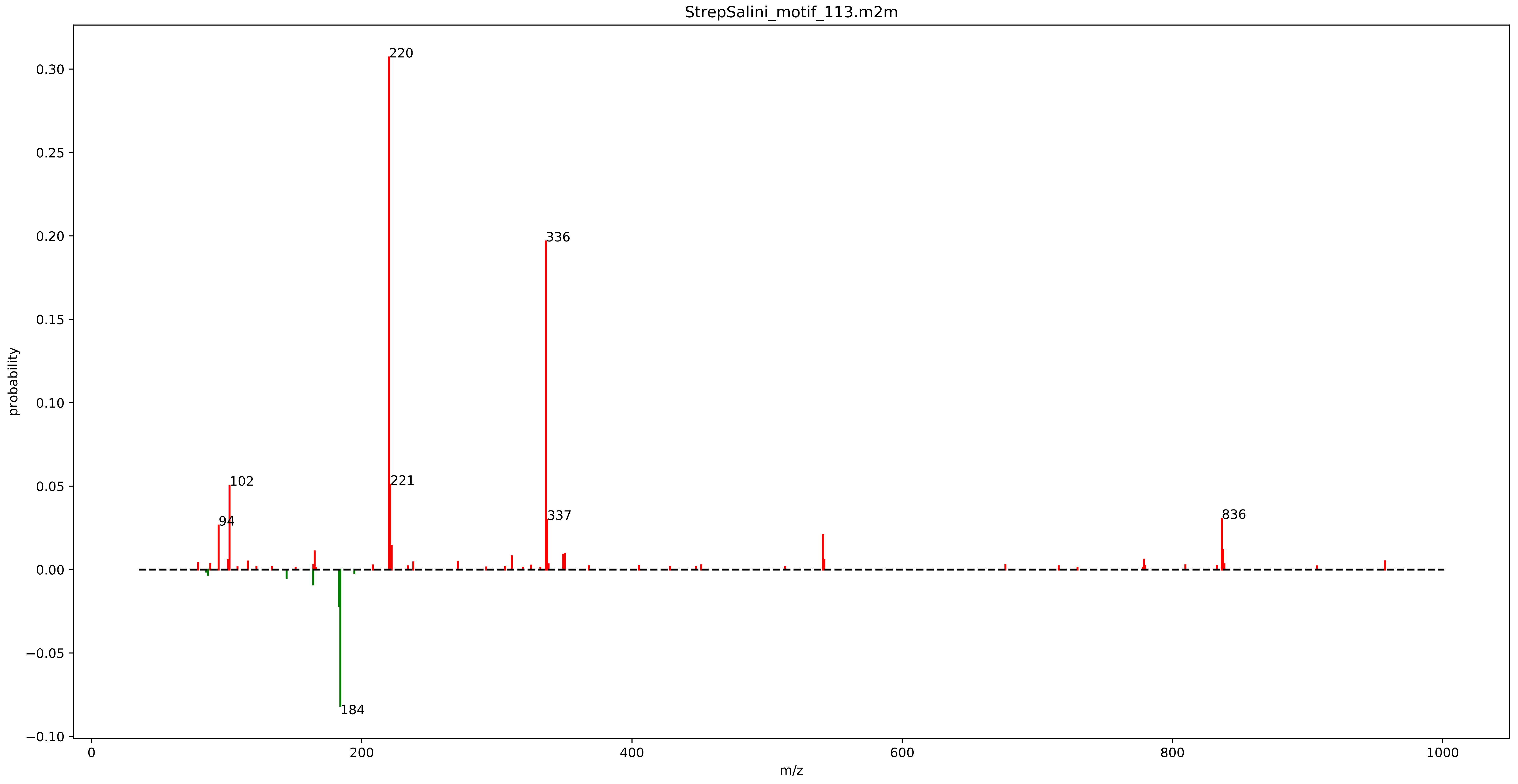

StrepSalini_motif_113.m2m
probability
m/z
220
336
102
221
94
337
836
184
0.30
0.25
0.20
0.15
0.10
0.05
0.00
−0.05
−0.10
0
200
400
600
800
1000

StrepSalini_motif_113.m2m

fragment_220.1500	: 0.31
fragment_336.2525	: 0.20
loss_184.1525	: 0.08
fragment_221.1500	: 0.05
fragment_102.1500	: 0.05
fragment_836.4500	: 0.03
fragment_337.2525	: 0.03
fragment_94.0500	: 0.03
loss_183.1525	: 0.02
fragment_541.3500	: 0.02
fragment_222.1500	: 0.01
fragment_837.4500	: 0.01
fragment_165.1500	: 0.01
fragment_350.2525	: 0.01
loss_164.0500	: 0.01
fragment_349.0500	: 0.01
fragment_311.0500	: 0.01
fragment_778.8500	: 0.01
fragment_101.0500	: 0.01
fragment_542.3500	: 0.01

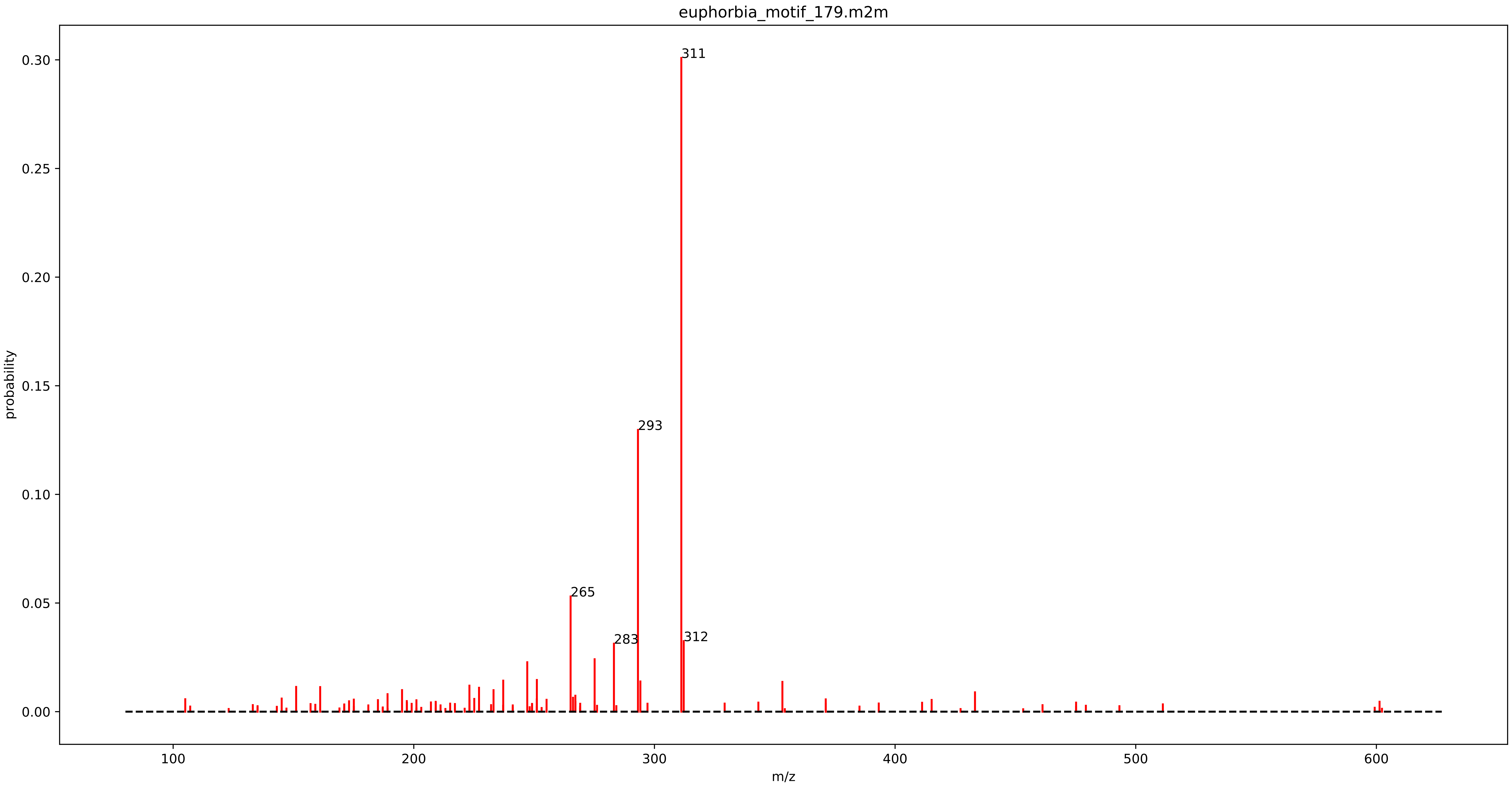

euphorbia_motif_179.m2m
probability
m/z
311
293
265
283
312
0.30
0.25
0.20
0.15
0.10
0.05
0.00
100
200
300
400
500
600

euphorbia_motif_179.m2m

fragment_311.1675	: 0.30
fragment_293.1525	: 0.13
fragment_265.1575	: 0.05
fragment_312.1675	: 0.03
fragment_283.1675	: 0.03
fragment_275.1425	: 0.02
fragment_247.1475	: 0.02
fragment_251.1425	: 0.01
fragment_237.1625	: 0.01
fragment_294.1575	: 0.01
fragment_353.1725	: 0.01
fragment_223.1125	: 0.01
fragment_151.1125	: 0.01
fragment_161.0975	: 0.01
fragment_227.1075	: 0.01
fragment_195.1175	: 0.01
fragment_233.1325	: 0.01
fragment_433.2025	: 0.01
fragment_189.0925	: 0.01
fragment_267.1375	: 0.01
fragment_266.1625	: 0.01
fragment_145.1025	: 0.01
fragment_225.1275	: 0.01
fragment_105.0325	: 0.01
fragment_371.1825	: 0.01
fragment_251.1075	: 0.01
fragment_175.0775	: 0.01
fragment_255.1725	: 0.01
fragment_415.1925	: 0.01
fragment_185.0975	: 0.01
fragment_201.0925	: 0.01
fragment_223.1475	: 0.01

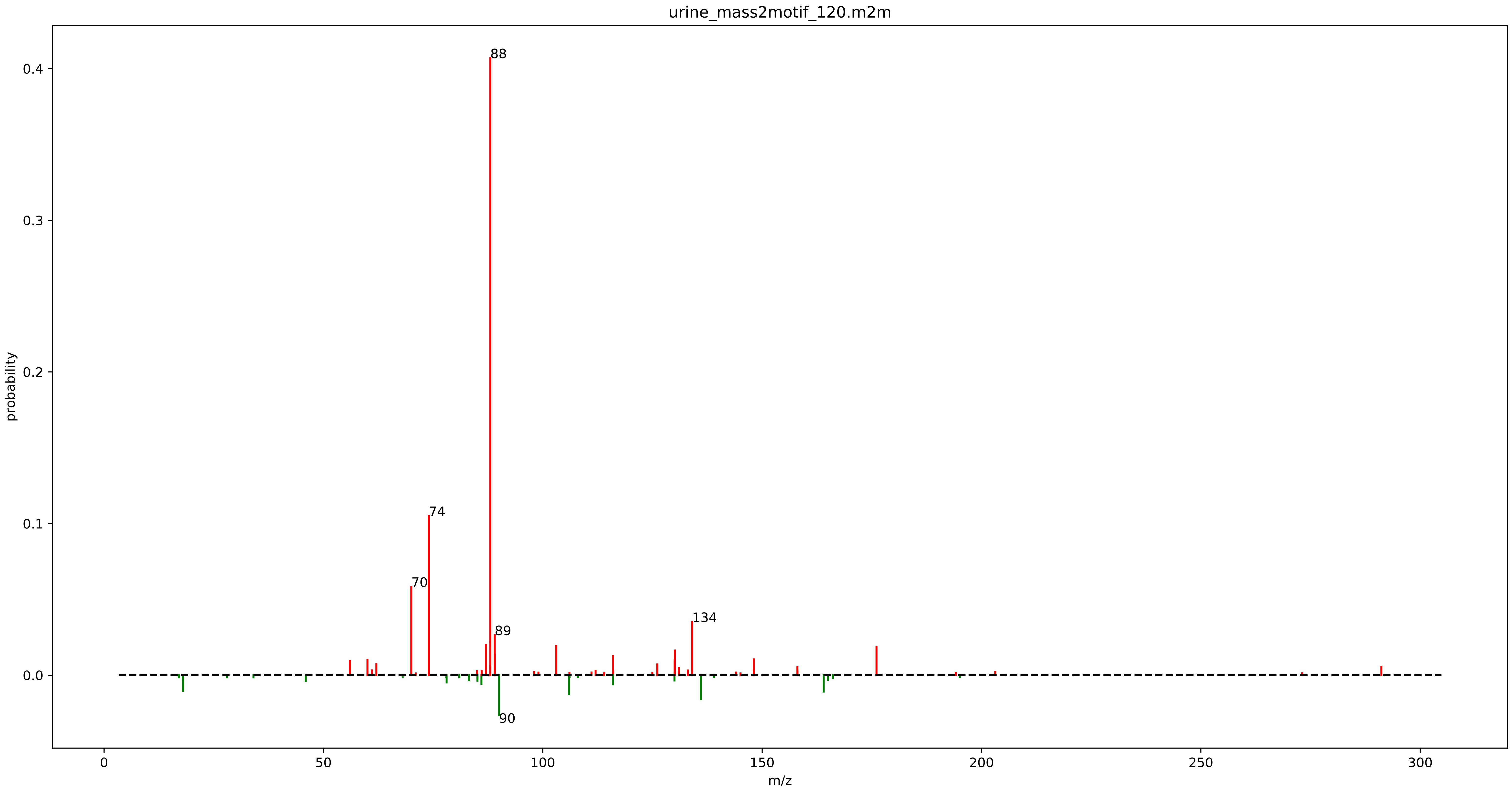

urine_mass2motif_120.m2m
probability
m/z
0.4
0.3
0.2
0.1
0.0
0
50
100
150
200
250
300
88
74
70
89
134
90

urine_mass2motif_120.m2m

fragment_88.0375	: 0.41
fragment_74.0225	: 0.10
fragment_70.0275	: 0.06
fragment_134.0475	: 0.04
loss_90.0125	: 0.03
fragment_89.0425	: 0.03
fragment_87.0575	: 0.02
fragment_103.0575	: 0.02
fragment_176.0675	: 0.02
fragment_130.0875	: 0.02
loss_136.0175	: 0.02
fragment_89.0475	: 0.01
fragment_116.0325	: 0.01
loss_105.9975	: 0.01
loss_164.0125	: 0.01
loss_17.9925	: 0.01
fragment_148.0975	: 0.01
fragment_60.0425	: 0.01
fragment_130.0475	: 0.01
fragment_56.0475	: 0.01
fragment_62.0625	: 0.01
fragment_126.1025	: 0.01
loss_116.0125	: 0.01
loss_86.0175	: 0.01
fragment_291.1325	: 0.01
fragment_88.0625	: 0.01
fragment_158.0425	: 0.01

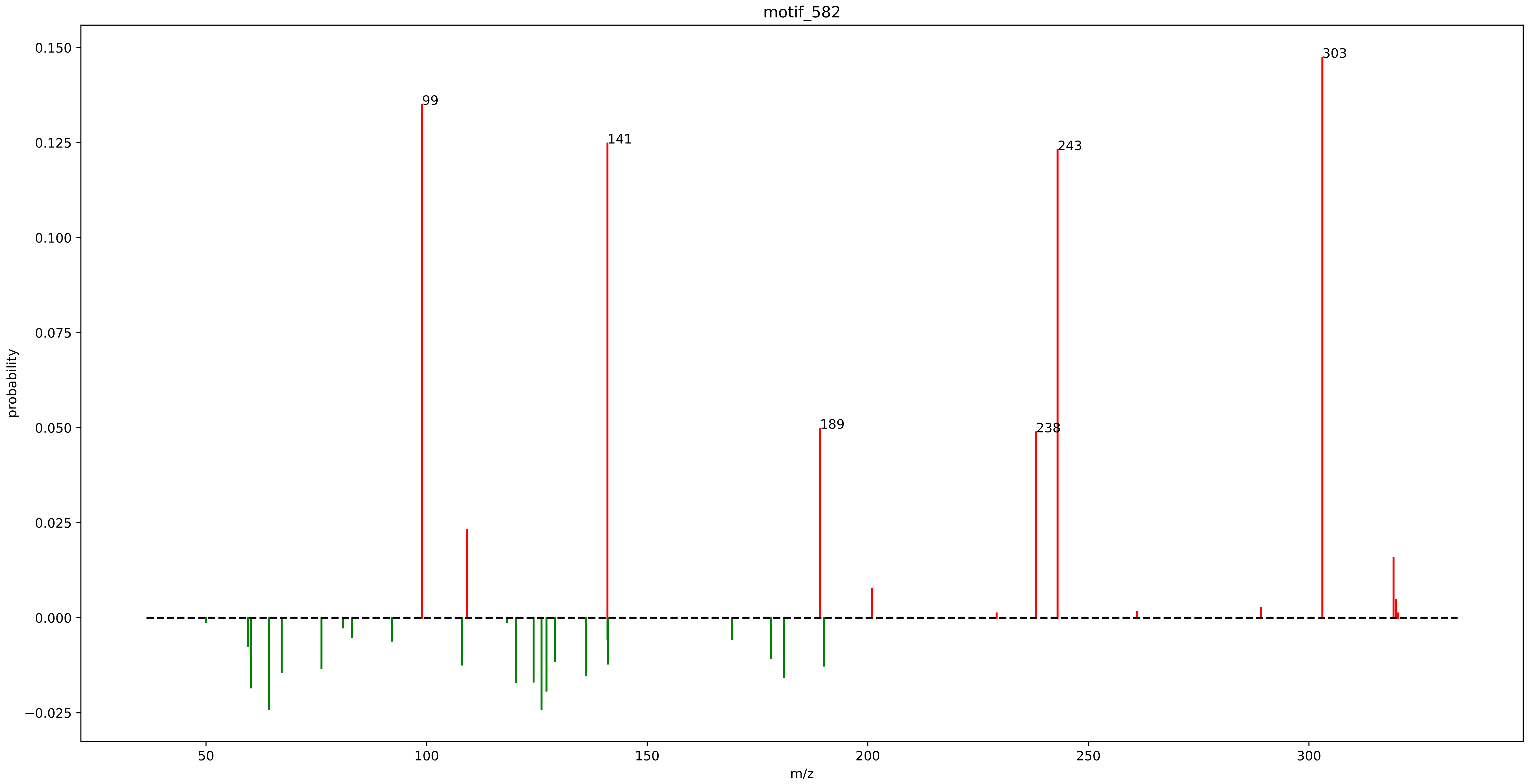
motif_582
0.150
0.125
0.100
0.075
0.050
0.025
0.000
−0.025
probability
50
100
150
200
250
300
m/z
99
141
189
238
243
303

motif_582

fragment_303.0375	: 0.15
fragment_98.9725	: 0.13
fragment_140.9875	: 0.12
fragment_243.0175	: 0.12
fragment_189.1625	: 0.05
fragment_238.1475	: 0.05
loss_64.2175	: 0.02
loss_126.0375	: 0.02
fragment_109.0975	: 0.02
loss_127.1725	: 0.02
loss_60.1675	: 0.02
loss_120.1925	: 0.02
loss_124.2375	: 0.02
fragment_319.1725	: 0.02
loss_181.0275	: 0.02
loss_136.1775	: 0.02
loss_67.1525	: 0.01
loss_76.1575	: 0.01
loss_190.0325	: 0.01
loss_108.0275	: 0.01
loss_141.0475	: 0.01
loss_129.1075	: 0.01
loss_178.0925	: 0.01
fragment_201.0075	: 0.01
loss_59.5175	: 0.01
loss_92.1325	: 0.01
loss_169.1825	: 0.01
loss_140.9975	: 0.01

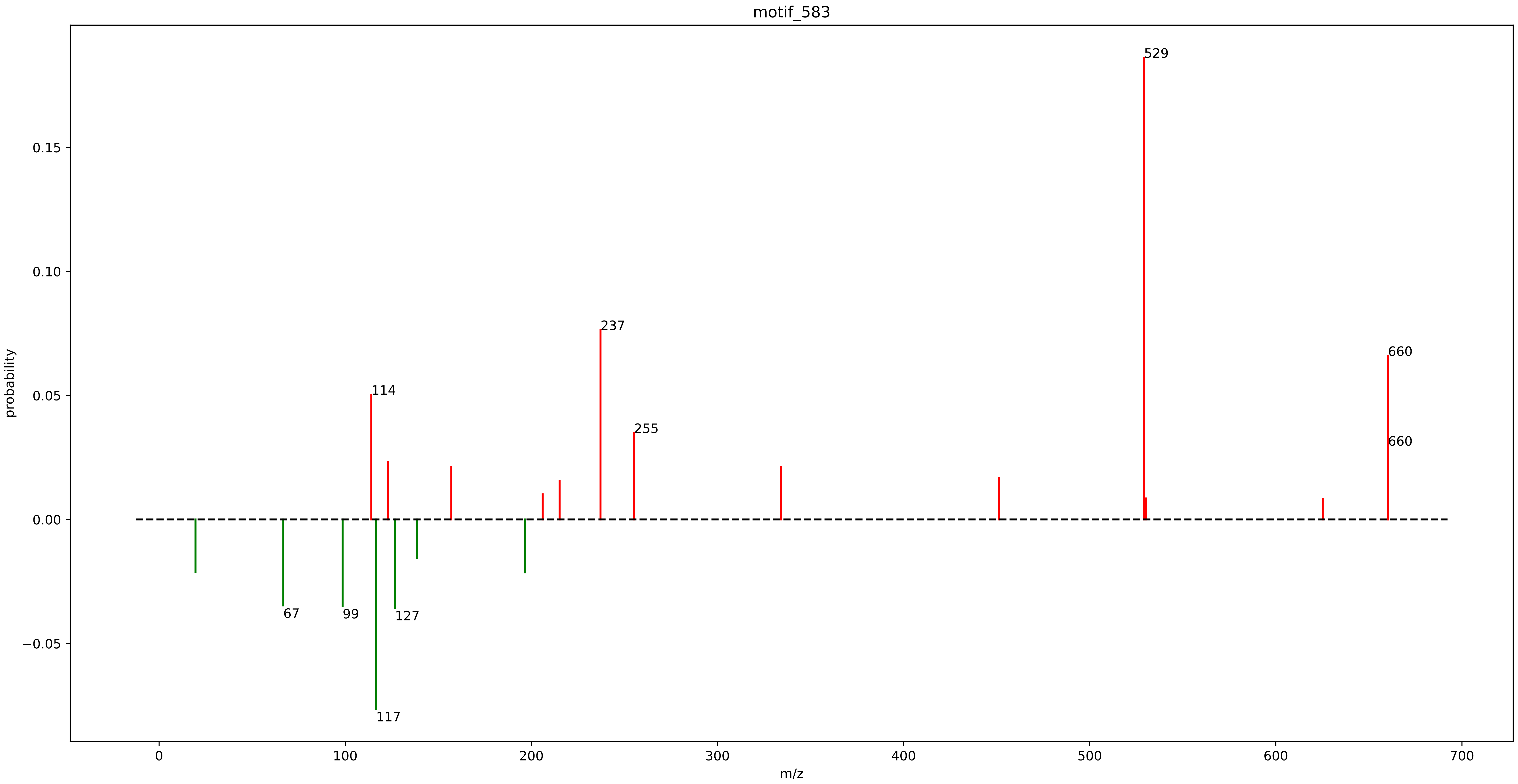

motif_583
probability
m/z
0.15
0.10
0.05
0.00
−0.05
0
100
200
300
400
500
600
700
529
237
660
114
255
660
67
99
127
117

motif_583

fragment_529.1975	: 0.19
loss_116.6225	: 0.08
fragment_237.1675	: 0.08
fragment_660.2375	: 0.07
fragment_114.0525	: 0.05
loss_126.7525	: 0.04
loss_98.6125	: 0.03
fragment_255.1825	: 0.03
loss_66.7325	: 0.03
fragment_660.2325	: 0.03
fragment_123.1175	: 0.02
loss_196.7575	: 0.02
fragment_157.0325	: 0.02
loss_19.5575	: 0.02
fragment_334.2375	: 0.02
fragment_451.3625	: 0.02
loss_138.5925	: 0.02
fragment_215.1975	: 0.02
fragment_206.0925	: 0.01
fragment_530.1975	: 0.01
fragment_625.2175	: 0.01

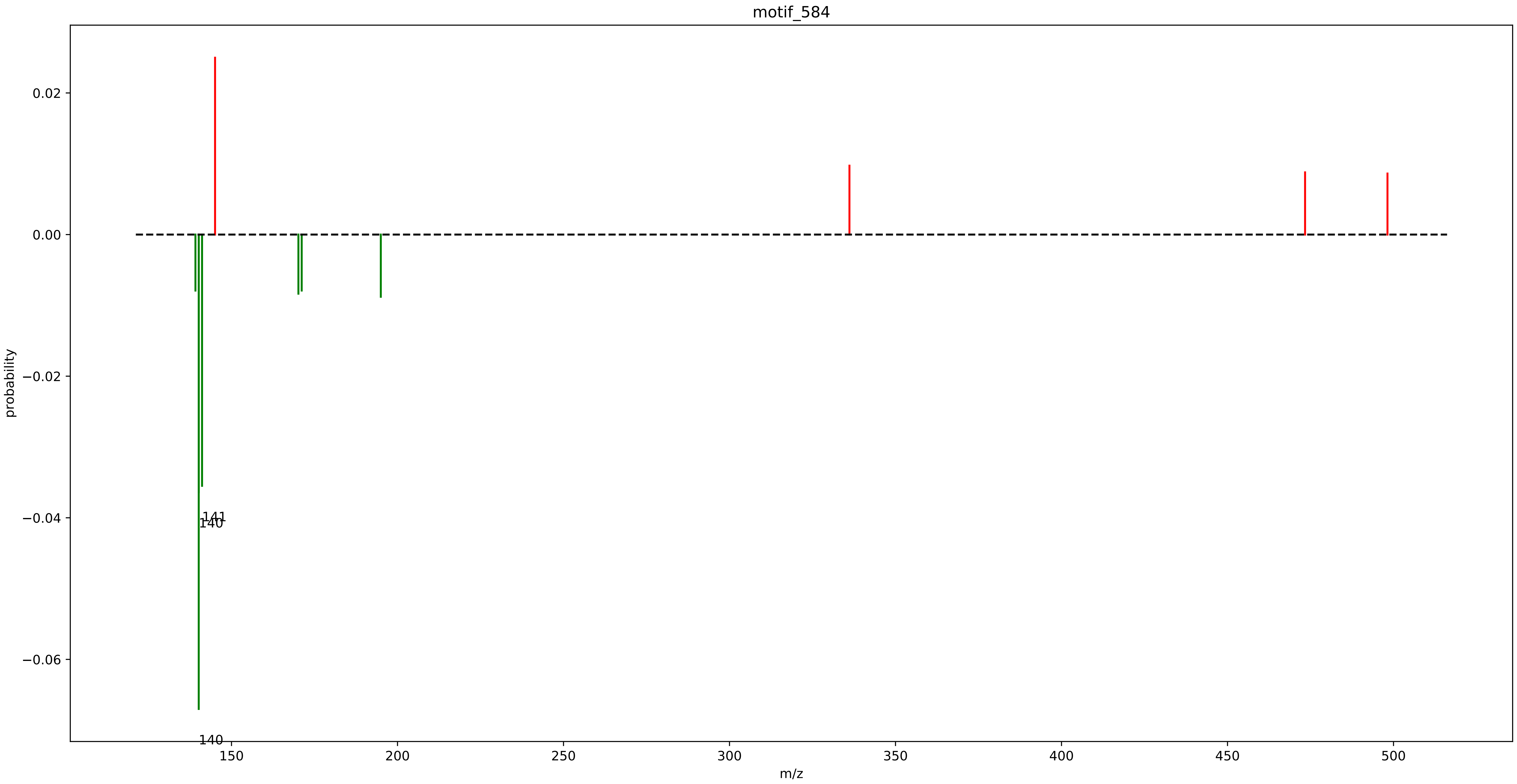

motif_584
probability
m/z
0.02
0.00
−0.02
−0.04
−0.06
150
200
250
300
350
400
450
500
141
140
140

motif_584

loss_140.1275	: 0.07
loss_140.1425	: 0.04
loss_141.1325	: 0.04
fragment_145.0475	: 0.02
fragment_336.1275	: 0.01
loss_194.9725	: 0.01
fragment_473.3625	: 0.01
fragment_498.1825	: 0.01
loss_170.1525	: 0.01
loss_139.1375	: 0.01
loss_171.1425	: 0.01

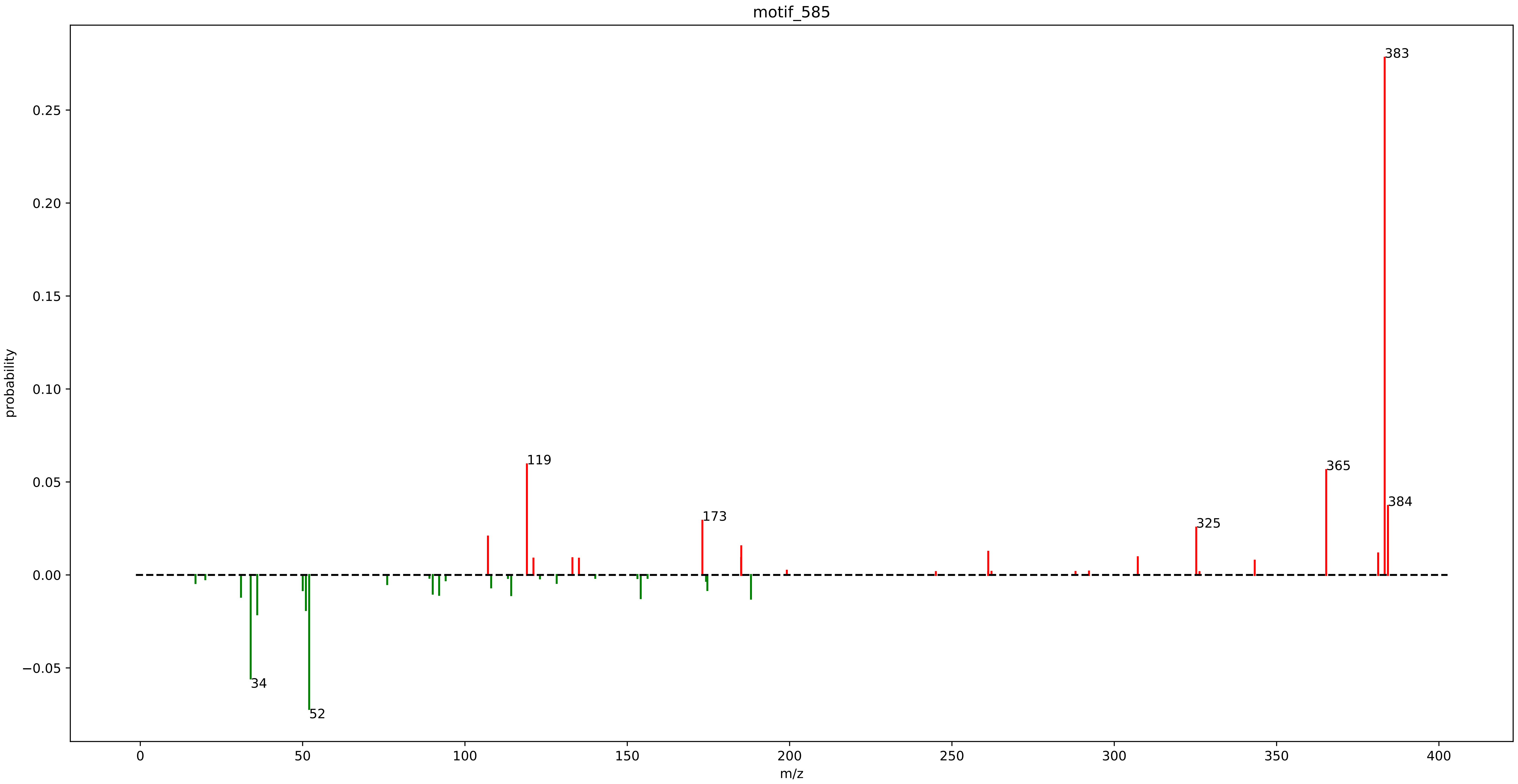

motif_585
probability
m/z
383
119
365
384
173
325
34
52
0.25
0.20
0.15
0.10
0.05
0.00
−0.05
0
50
100
150
200
250
300
350
400

motif_585

fragment_383.2925	: 0.28
loss_52.0175	: 0.07
fragment_119.0825	: 0.06
fragment_365.2825	: 0.06
loss_34.0025	: 0.06
fragment_384.2975	: 0.04
fragment_173.1325	: 0.03
fragment_325.2525	: 0.03
loss_36.0175	: 0.02
fragment_107.0825	: 0.02
loss_51.0125	: 0.02
fragment_185.1125	: 0.02
loss_188.1025	: 0.01
fragment_261.1825	: 0.01
loss_154.1325	: 0.01
loss_31.0225	: 0.01
fragment_381.2775	: 0.01
loss_114.2425	: 0.01
loss_92.0475	: 0.01
loss_90.0675	: 0.01
fragment_307.2425	: 0.01
fragment_185.0675	: 0.01
fragment_133.1025	: 0.01
fragment_121.0975	: 0.01
fragment_135.1125	: 0.01
loss_50.0325	: 0.01
loss_174.6725	: 0.01
fragment_343.2625	: 0.01
loss_108.0775	: 0.01

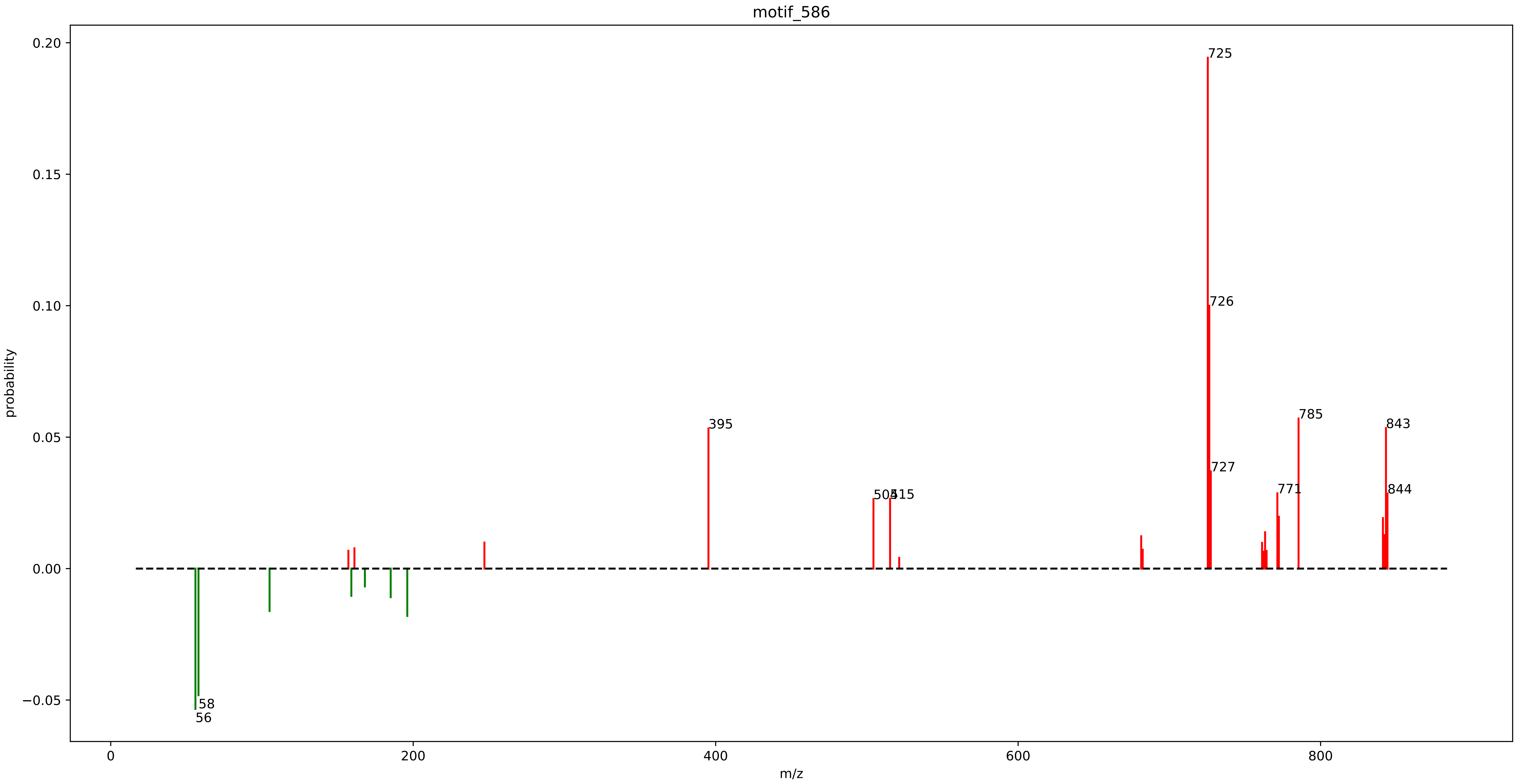

motif_586
probability
m/z
0.20
0.15
0.10
0.05
0.00
−0.05
0
200
400
600
800
725
726
727
395
505
515
785
843
844
771
58
56

motif_586

fragment_725.4125	: 0.19
fragment_726.4125	: 0.10
fragment_785.4325	: 0.06
fragment_843.2275	: 0.05
loss_56.0025	: 0.05
fragment_395.2175	: 0.05
loss_58.0175	: 0.05
fragment_727.4125	: 0.04
fragment_771.4175	: 0.03
fragment_844.2275	: 0.03
fragment_515.3375	: 0.03
fragment_504.3375	: 0.03
fragment_772.4225	: 0.02
fragment_841.2275	: 0.02
loss_196.0825	: 0.02
loss_105.0175	: 0.02
fragment_763.2975	: 0.01
fragment_842.2275	: 0.01
fragment_681.3775	: 0.01
loss_185.1125	: 0.01
loss_159.0975	: 0.01
fragment_247.0825	: 0.01
fragment_761.3025	: 0.01
fragment_161.1325	: 0.01
fragment_682.3775	: 0.01
loss_168.0525	: 0.01
fragment_157.1325	: 0.01
fragment_764.3025	: 0.01
fragment_762.3025	: 0.01

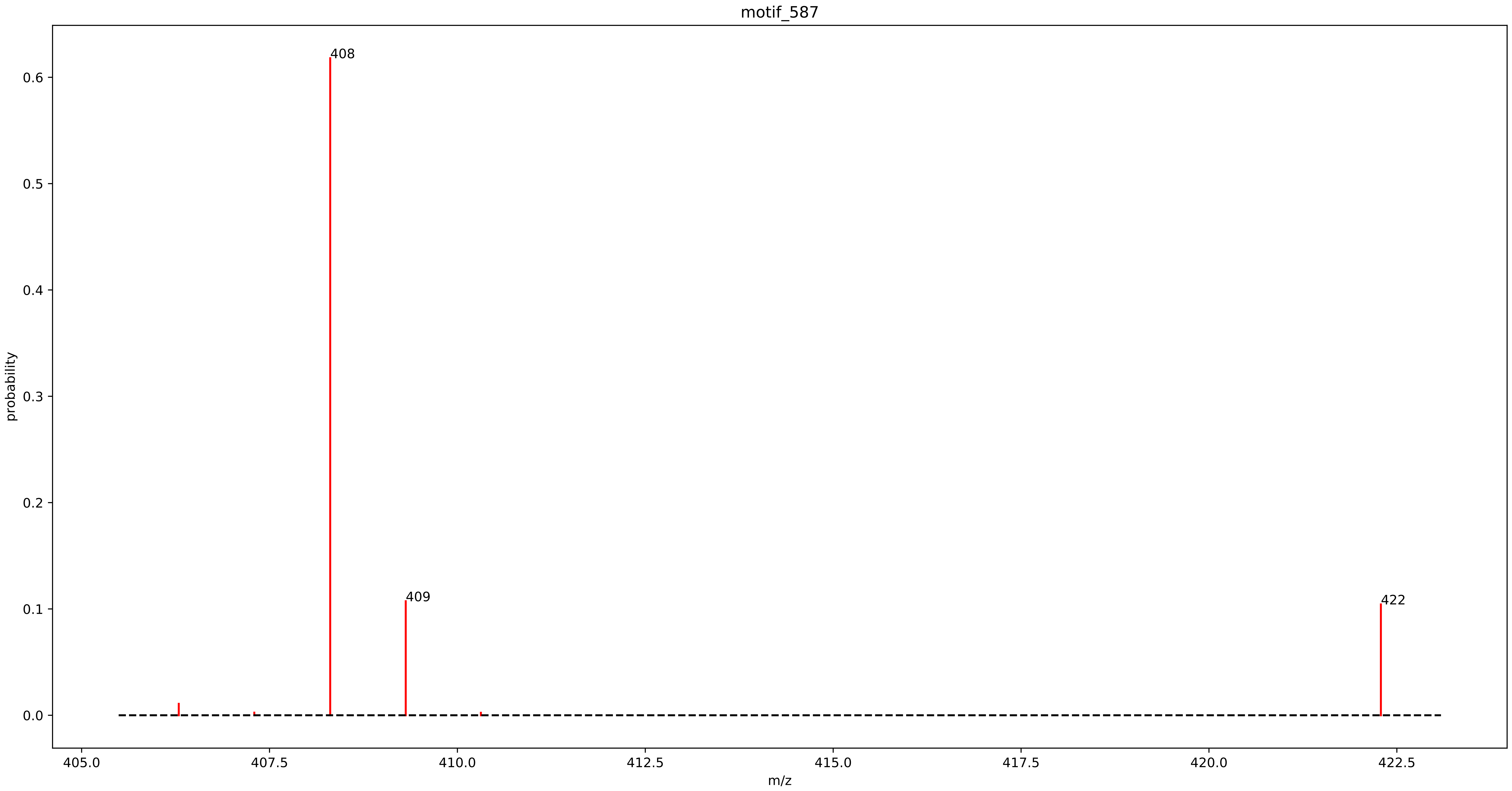

motif_587
probability
m/z
408
409
422
0.0
0.1
0.2
0.3
0.4
0.5
0.6
405.0
407.5
410.0
412.5
415.0
417.5
420.0
422.5

motif_587

fragment_408.3075	: 0.62
fragment_409.3125	: 0.11
fragment_422.2875	: 0.10
fragment_406.2925	: 0.01

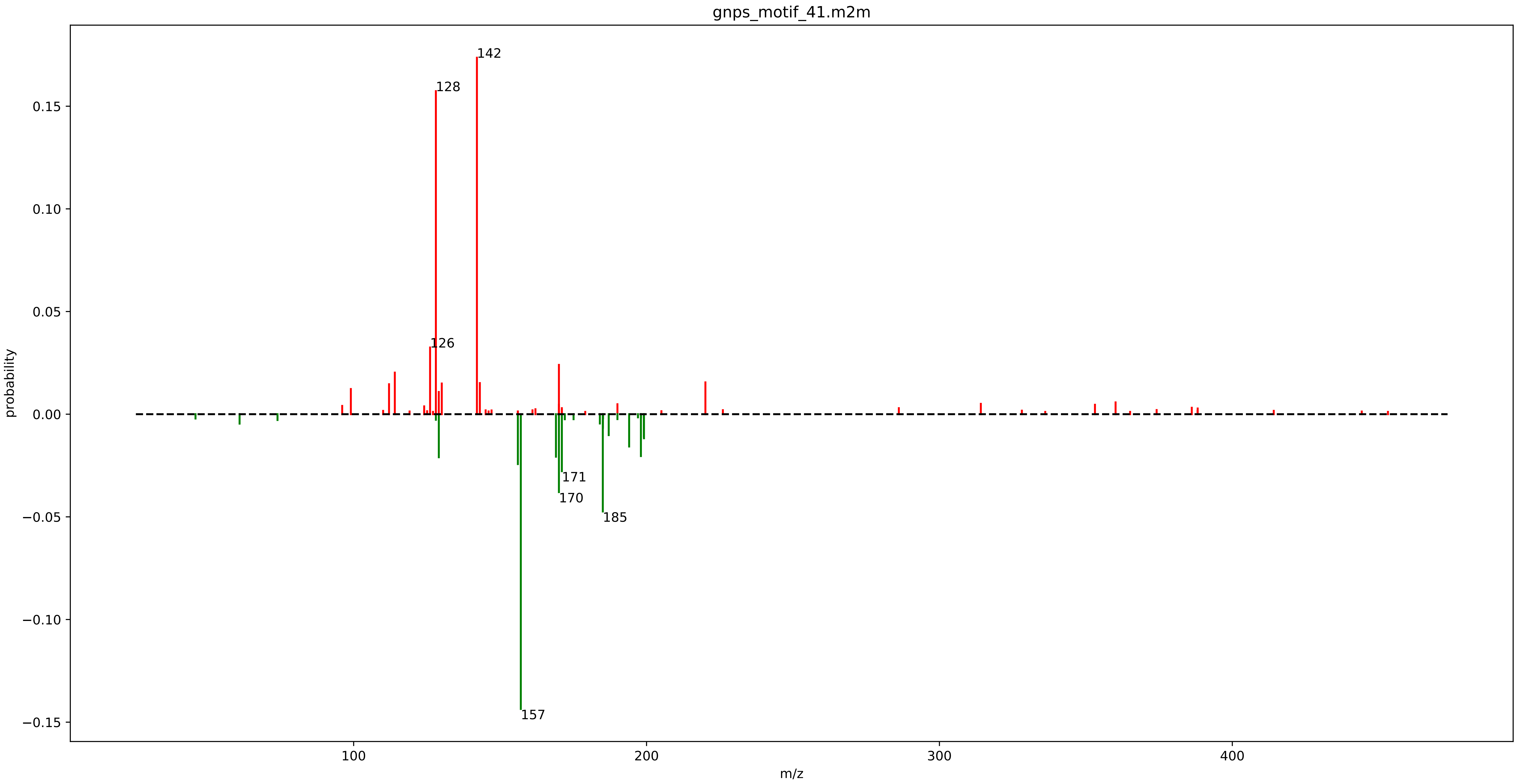

gnps_motif_41.m2m
probability
m/z
0.15
0.10
0.05
0.00
−0.05
−0.10
−0.15
100
200
300
400
142
128
126
171
170
185
157

gnps_motif_41.m2m

fragment_142.0875	: 0.17
fragment_128.0725	: 0.16
loss_157.0725	: 0.14
loss_185.0675	: 0.05
loss_170.0825	: 0.04
fragment_126.0925	: 0.03
loss_171.0875	: 0.03
loss_156.0675	: 0.02
fragment_170.0825	: 0.02
loss_129.0775	: 0.02
loss_169.0725	: 0.02
loss_198.0775	: 0.02
fragment_114.0525	: 0.02
loss_194.0575	: 0.02
fragment_220.0975	: 0.02
fragment_143.0875	: 0.02
fragment_130.0875	: 0.01
fragment_112.0775	: 0.01
fragment_99.0425	: 0.01
loss_199.1225	: 0.01
loss_199.0825	: 0.01
fragment_129.0675	: 0.01
loss_187.0825	: 0.01
loss_185.1075	: 0.01
fragment_360.1425	: 0.01
fragment_314.1375	: 0.01

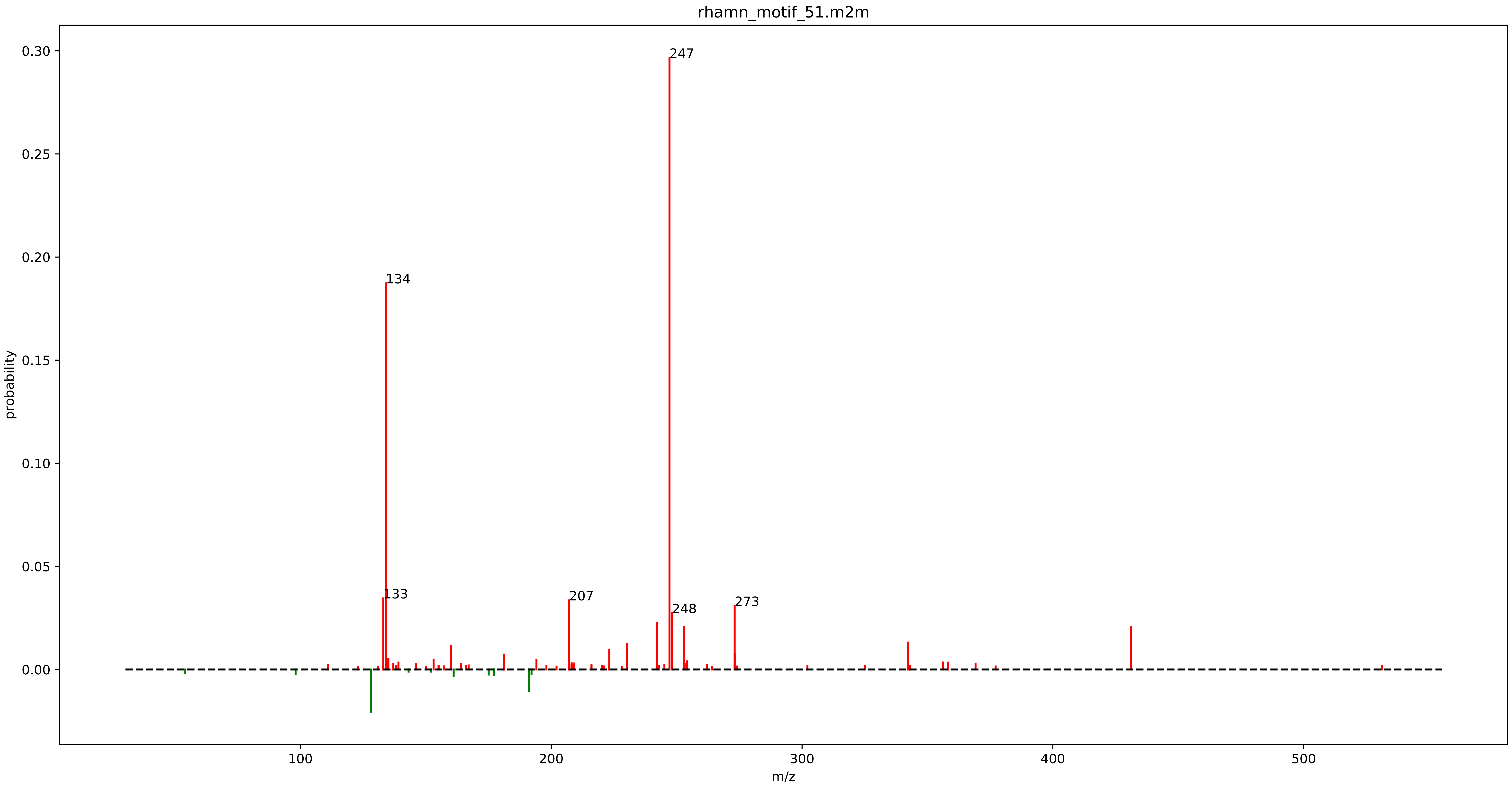

rhamn_motif_51.m2m
probability
m/z
0.30
0.25
0.20
0.15
0.10
0.05
0.00
100
200
300
400
500
247
134
133
207
248
273

rhamn_motif_51.m2m

fragment_247.1475	: 0.30
fragment_134.0625	: 0.19
fragment_133.0525	: 0.03
fragment_207.1125	: 0.03
fragment_273.1225	: 0.03
fragment_248.1475	: 0.03
fragment_242.1175	: 0.02
fragment_431.2325	: 0.02
loss_128.2425	: 0.02
fragment_253.0475	: 0.02
fragment_342.1825	: 0.01
fragment_230.1225	: 0.01
fragment_230.1175	: 0.01
fragment_160.0425	: 0.01
fragment_342.1775	: 0.01
fragment_342.1425	: 0.01
loss_191.1325	: 0.01
fragment_160.0375	: 0.01
fragment_223.1425	: 0.01
fragment_207.1175	: 0.01
fragment_273.1275	: 0.01
fragment_181.0975	: 0.01
fragment_134.0675	: 0.01
fragment_207.1075	: 0.01
fragment_342.1375	: 0.01
fragment_135.0675	: 0.01
fragment_342.1925	: 0.01

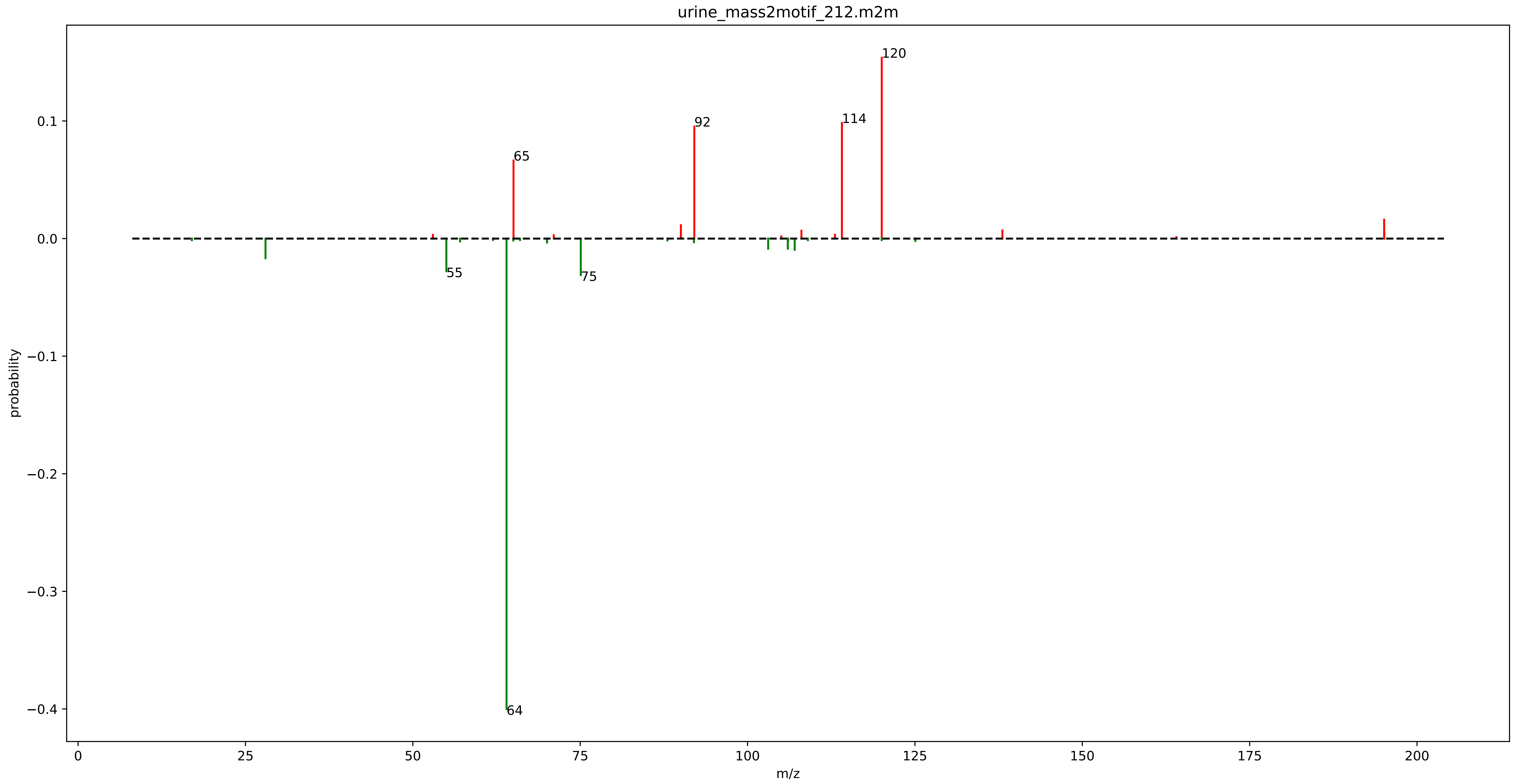
urine_mass2motif_212.m2m
probability
m/z
0.1
0.0
−0.1
−0.2
−0.3
−0.4
0
25
50
75
100
125
150
175
200
120
92
114
65
55
75
64

urine_mass2motif_212.m2m

loss_63.9975	: 0.40
fragment_120.0425	: 0.15
fragment_114.0925	: 0.10
fragment_92.0475	: 0.10
fragment_65.0375	: 0.07
loss_75.0875	: 0.03
loss_55.0075	: 0.03
loss_27.9925	: 0.02
fragment_195.0875	: 0.02
fragment_90.0375	: 0.01
loss_107.0375	: 0.01
loss_103.0775	: 0.01
loss_106.0075	: 0.01
fragment_138.0675	: 0.01
fragment_108.0475	: 0.01
fragment_195.1125	: 0.01

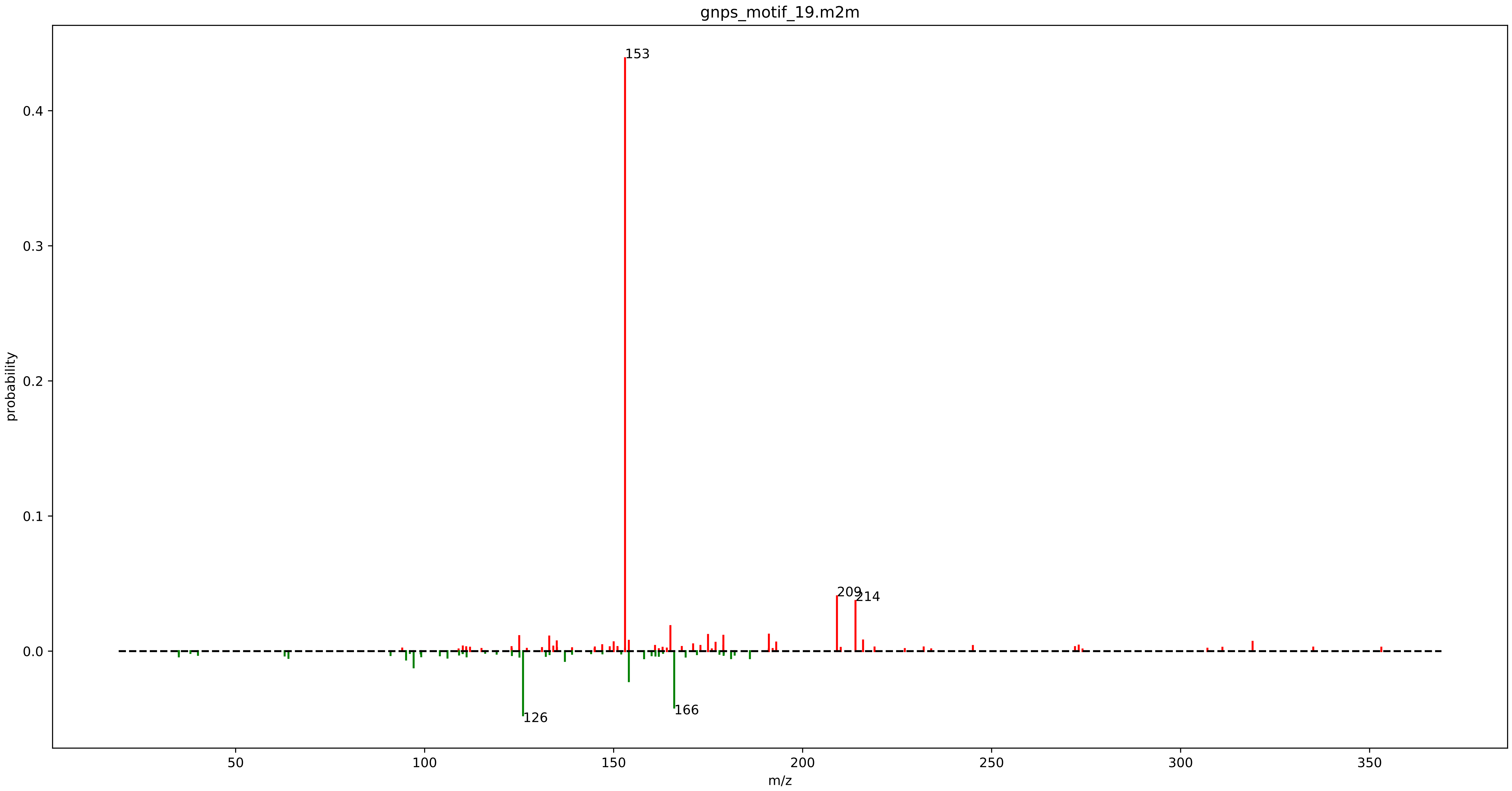
gnps_motif_19.m2m
153
209
214
126
166
probability
0.4
0.3
0.2
0.1
0.0
50
100
150
200
250
300
350
m/z

gnps_motif_19.m2m

fragment_153.0175	: 0.44
loss_126.0325	: 0.05
loss_166.0275	: 0.04
fragment_209.0825	: 0.04
fragment_213.9825	: 0.04
loss_154.0275	: 0.02
fragment_165.0175	: 0.02
fragment_191.0725	: 0.01
fragment_174.9725	: 0.01
loss_97.0875	: 0.01
fragment_179.0325	: 0.01
fragment_125.0225	: 0.01
fragment_132.9625	: 0.01
fragment_215.9875	: 0.01
fragment_154.0225	: 0.01
fragment_134.9575	: 0.01
loss_137.1075	: 0.01
fragment_319.0425	: 0.01
fragment_150.0125	: 0.01
fragment_193.0125	: 0.01
loss_95.0925	: 0.01
fragment_176.9675	: 0.01
loss_158.0575	: 0.01
loss_186.0525	: 0.01
loss_181.0725	: 0.01
fragment_171.0275	: 0.01

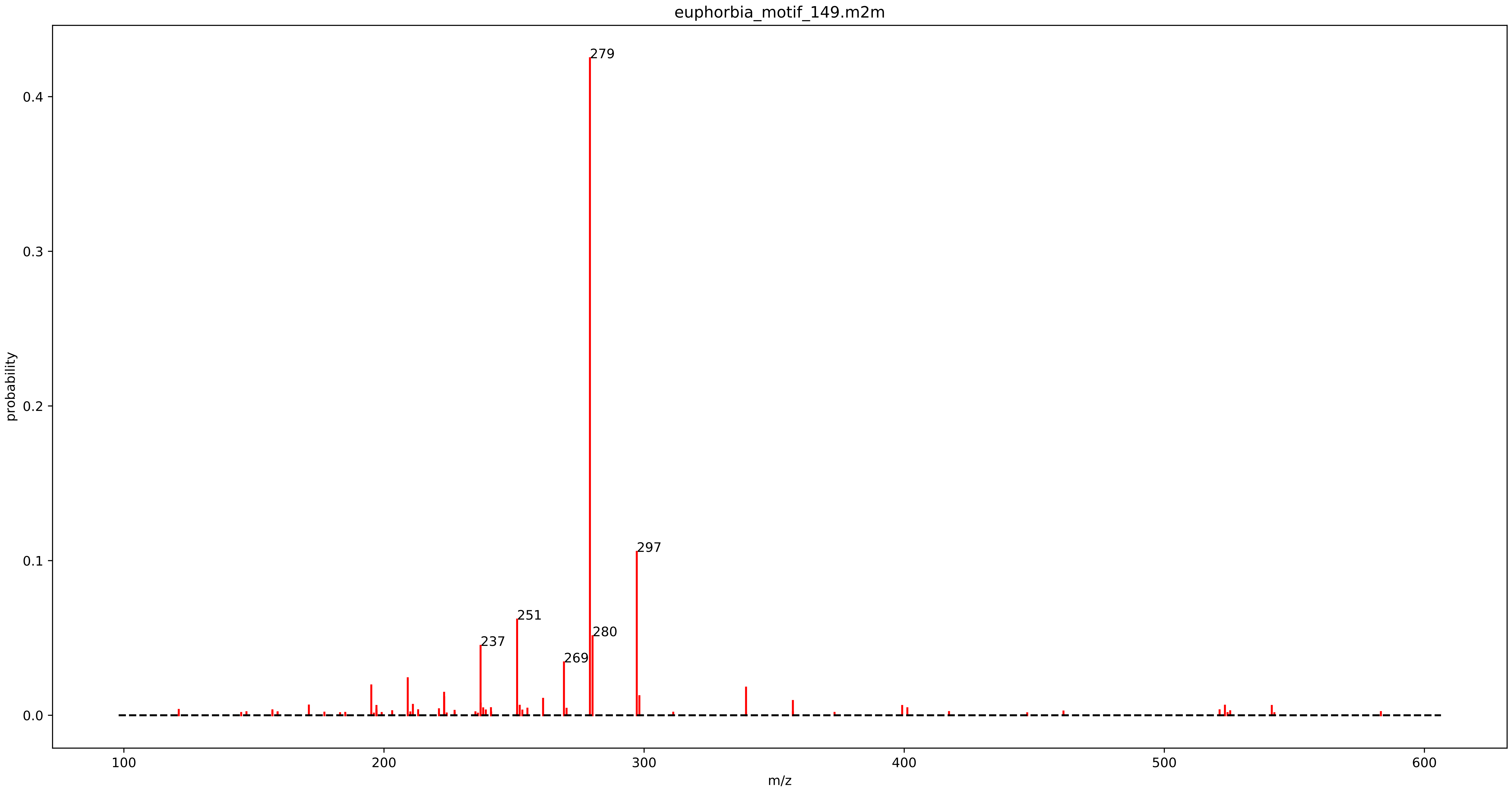

euphorbia_motif_149.m2m
probability
m/z
0.0
0.1
0.2
0.3
0.4
100
200
300
400
500
600
279
297
251
237
280
269

euphorbia_motif_149.m2m

fragment_279.1725	: 0.42
fragment_297.1825	: 0.11
fragment_251.1775	: 0.06
fragment_280.1775	: 0.05
fragment_237.1275	: 0.04
fragment_269.1875	: 0.03
fragment_209.1325	: 0.02
fragment_195.1175	: 0.02
fragment_339.1925	: 0.02
fragment_223.1125	: 0.01
fragment_298.1875	: 0.01
fragment_223.1475	: 0.01
fragment_261.1625	: 0.01
fragment_357.2025	: 0.01
fragment_237.1625	: 0.01
fragment_211.1125	: 0.01
fragment_171.1175	: 0.01
fragment_523.3025	: 0.01
fragment_252.1825	: 0.01
fragment_541.3175	: 0.01
fragment_197.0975	: 0.01
fragment_399.2175	: 0.01

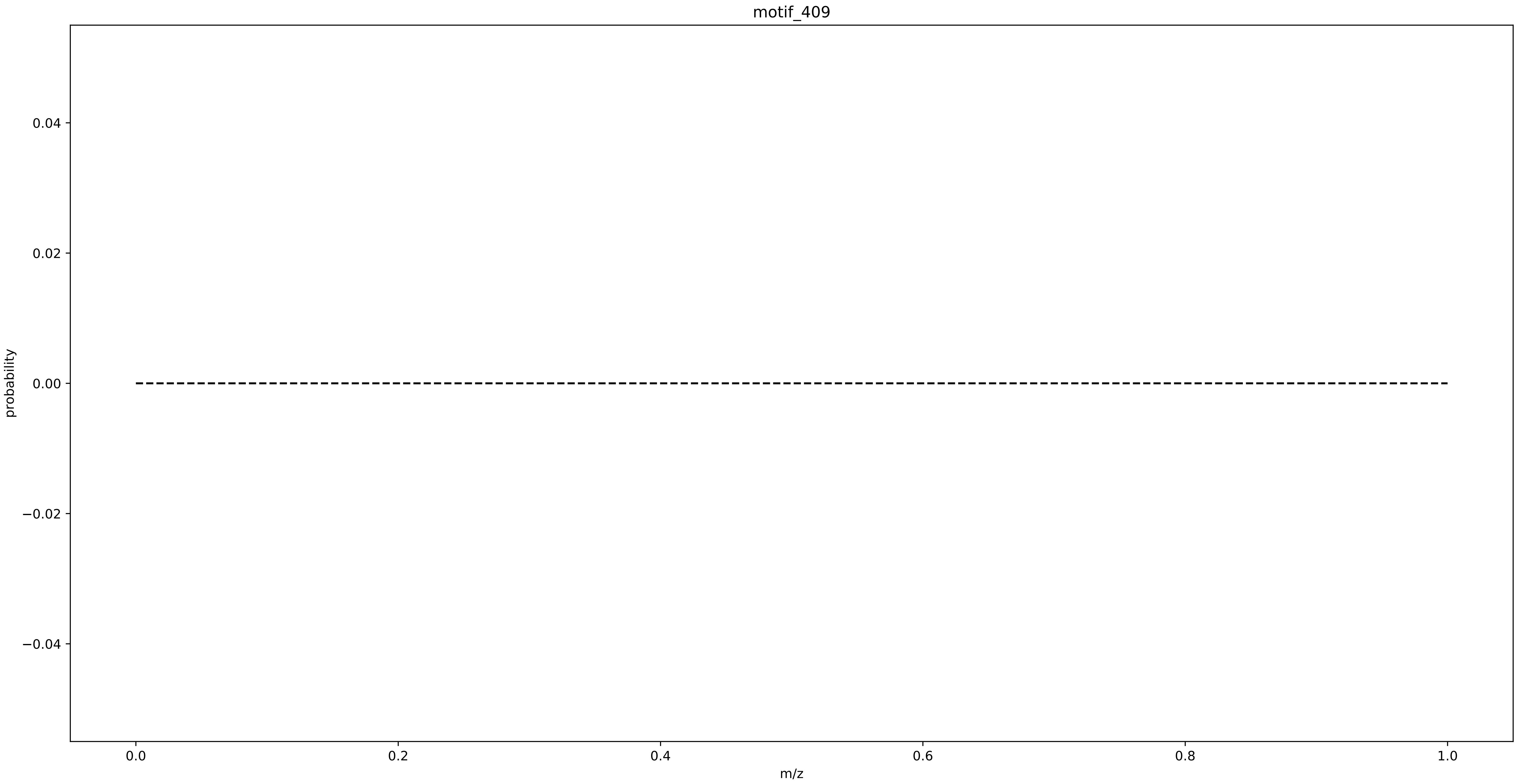

motif_409
probability
0.04
0.02
0.00
−0.02
−0.04
0.0
0.2
0.4
0.6
0.8
1.0
m/z

motif_409

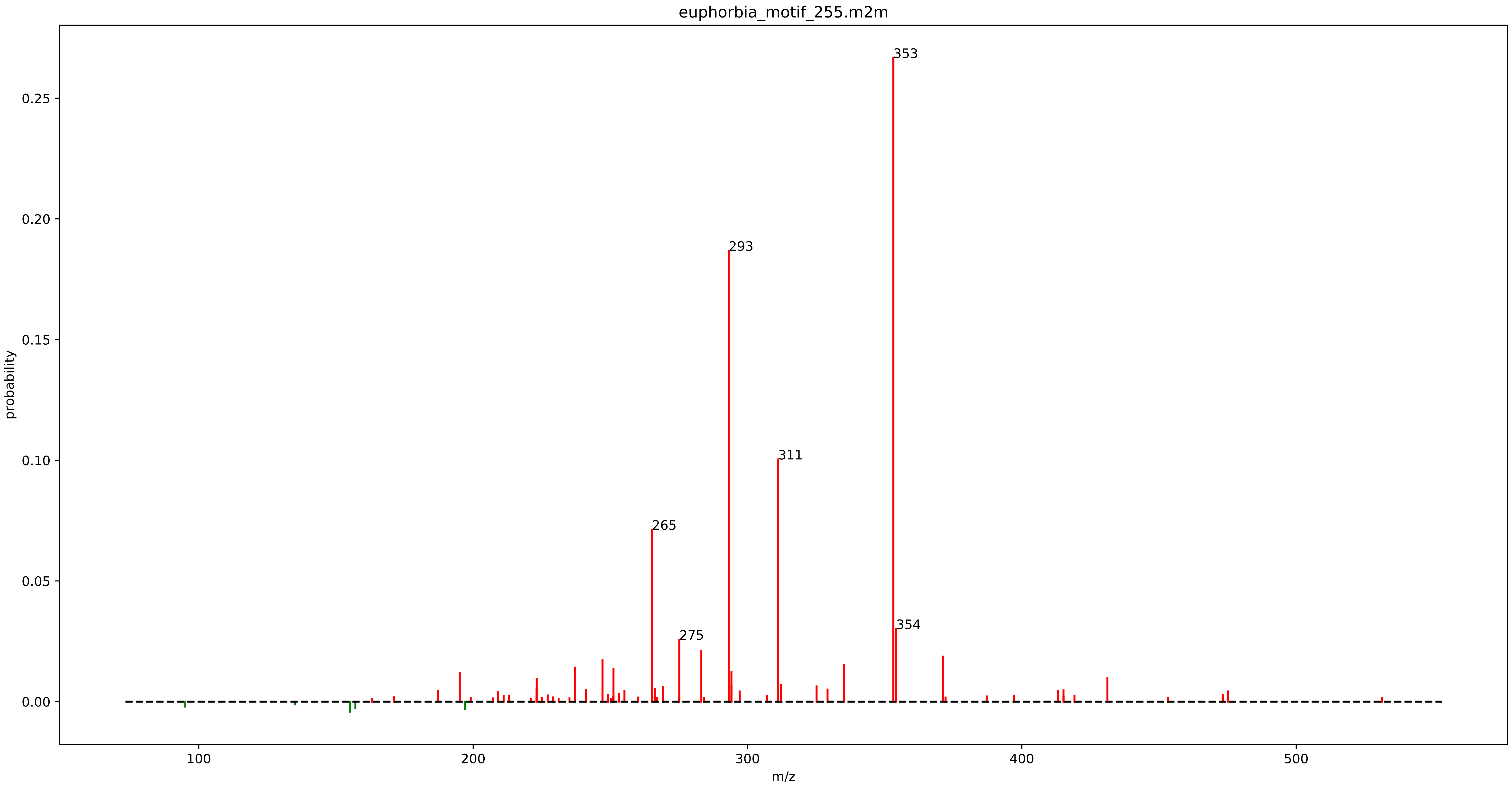

euphorbia_motif_255.m2m
probability
m/z
0.25
0.20
0.15
0.10
0.05
0.00
100
200
300
400
500
353
293
311
265
354
275

euphorbia_motif_255.m2m

fragment_353.1725	: 0.27
fragment_293.1525	: 0.19
fragment_311.1625	: 0.10
fragment_265.1575	: 0.07
fragment_354.1775	: 0.03
fragment_275.1425	: 0.03
fragment_283.1675	: 0.02
fragment_371.1825	: 0.02
fragment_247.1475	: 0.02
fragment_335.1625	: 0.02
fragment_237.1275	: 0.01
fragment_251.1425	: 0.01
fragment_294.1525	: 0.01
fragment_195.1175	: 0.01
fragment_431.2025	: 0.01
fragment_223.1475	: 0.01
fragment_354.1725	: 0.01
fragment_312.1675	: 0.01
fragment_294.1575	: 0.01
fragment_325.1775	: 0.01
fragment_269.1525	: 0.01
fragment_237.1625	: 0.01
fragment_266.1625	: 0.01
fragment_223.1125	: 0.01

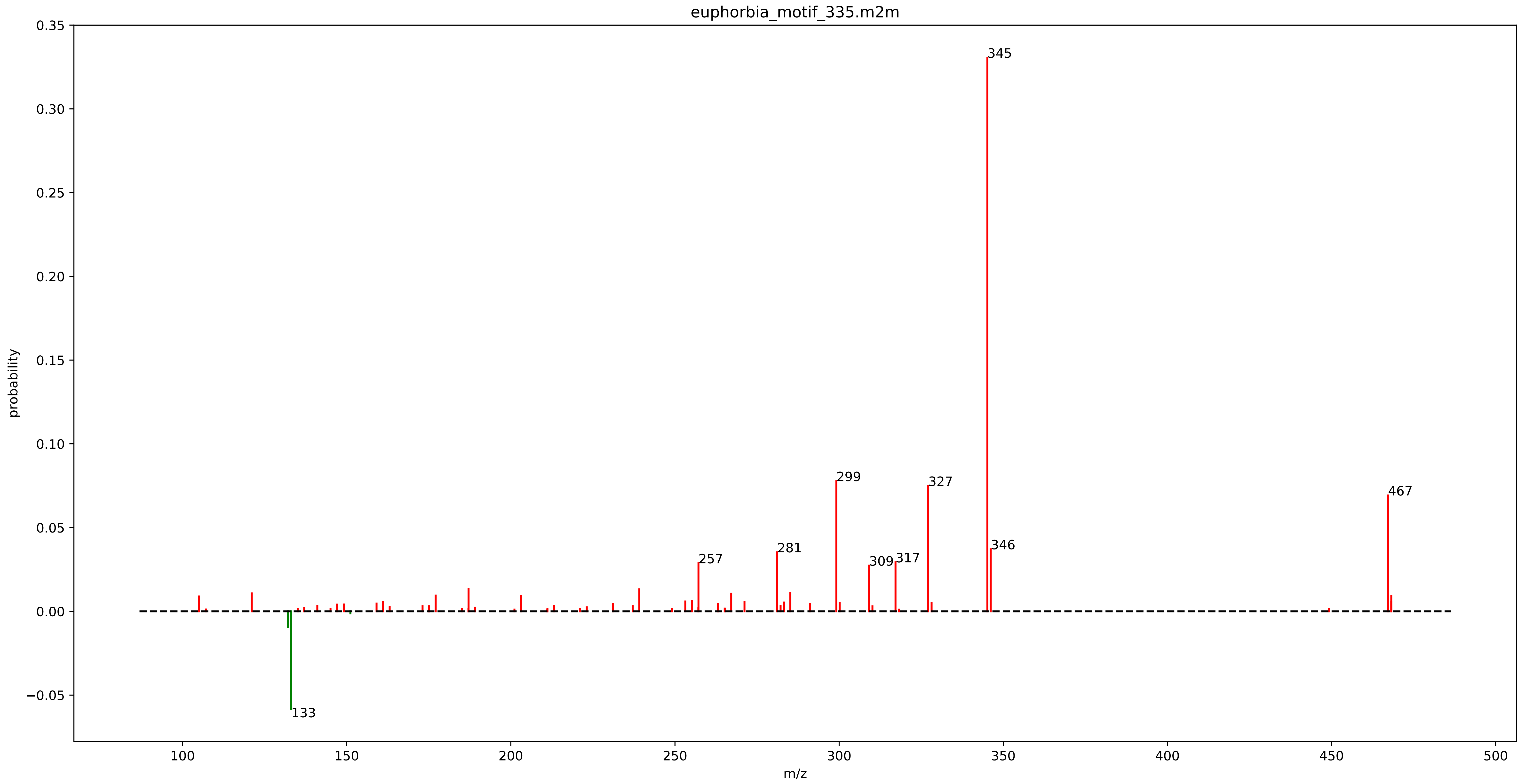
euphorbia_motif_335.m2m
probability
m/z
0.35
0.30
0.25
0.20
0.15
0.10
0.05
0.00
−0.05
100
150
200
250
300
350
400
450
500
345
299
327
467
346
257
281
309
317
133

euphorbia_motif_335.m2m

fragment_345.1675	: 0.33
fragment_299.1625	: 0.08
fragment_327.1575	: 0.07
fragment_467.2075	: 0.07
loss_133.1125	: 0.06
fragment_346.1725	: 0.04
fragment_281.1525	: 0.04
fragment_317.1725	: 0.03
fragment_257.1525	: 0.03
fragment_309.1475	: 0.03
fragment_187.1125	: 0.01
fragment_239.1425	: 0.01
fragment_285.1475	: 0.01
fragment_121.0625	: 0.01
fragment_267.1375	: 0.01
fragment_177.0925	: 0.01
loss_132.1075	: 0.01
fragment_468.2125	: 0.01
fragment_203.1075	: 0.01
fragment_105.0325	: 0.01
fragment_255.1375	: 0.01
fragment_253.1575	: 0.01
fragment_161.0975	: 0.01
fragment_468.2075	: 0.01
fragment_271.1675	: 0.01
fragment_283.1675	: 0.01
fragment_300.1675	: 0.01
fragment_328.1575	: 0.01

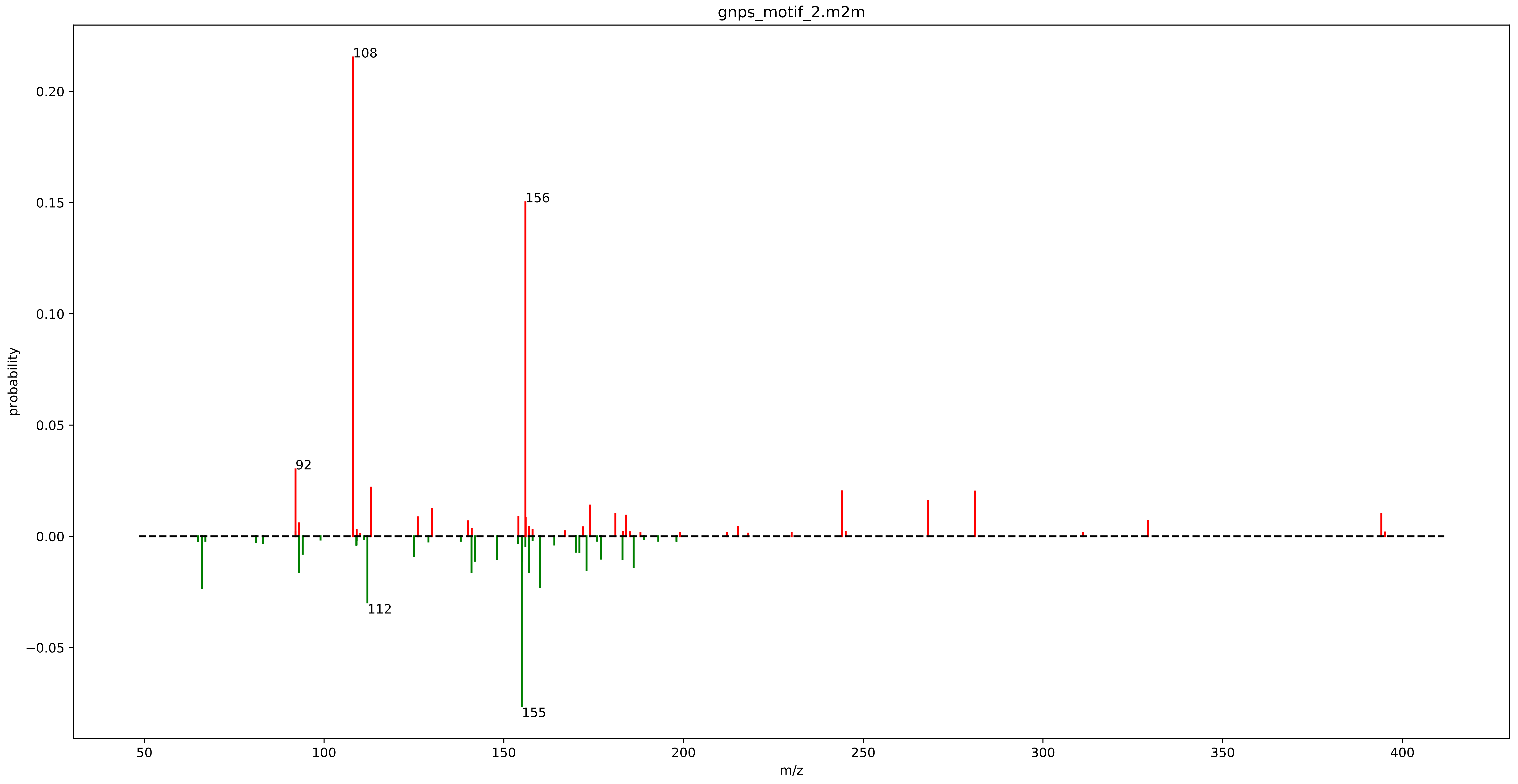
gnps_motif_2.m2m
probability
m/z
0.20
0.15
0.10
0.05
0.00
−0.05
50
100
150
200
250
300
350
400
108
156
92
112
155

gnps_motif_2.m2m

fragment_108.0425	: 0.22
fragment_156.0125	: 0.15
loss_155.0025	: 0.08
fragment_92.0475	: 0.03
loss_112.0625	: 0.03
loss_65.9775	: 0.02
loss_160.0325	: 0.02
fragment_113.0725	: 0.02
fragment_244.1125	: 0.02
fragment_281.0725	: 0.02
loss_93.0575	: 0.02
loss_157.0175	: 0.02
loss_141.0225	: 0.02
fragment_268.0725	: 0.02
loss_173.0225	: 0.02
fragment_174.0325	: 0.01
loss_186.1275	: 0.01
fragment_130.0425	: 0.01
loss_155.0675	: 0.01
loss_142.0225	: 0.01
loss_157.0275	: 0.01
loss_183.0325	: 0.01
fragment_394.1375	: 0.01
loss_148.0875	: 0.01
fragment_181.0425	: 0.01
loss_177.0075	: 0.01
fragment_184.0875	: 0.01
loss_125.0575	: 0.01
fragment_154.0625	: 0.01
fragment_126.0675	: 0.01
fragment_156.0775	: 0.01
loss_94.0525	: 0.01
loss_171.0325	: 0.01
fragment_329.1375	: 0.01
loss_170.0275	: 0.01
fragment_140.0425	: 0.01
fragment_93.0575	: 0.01

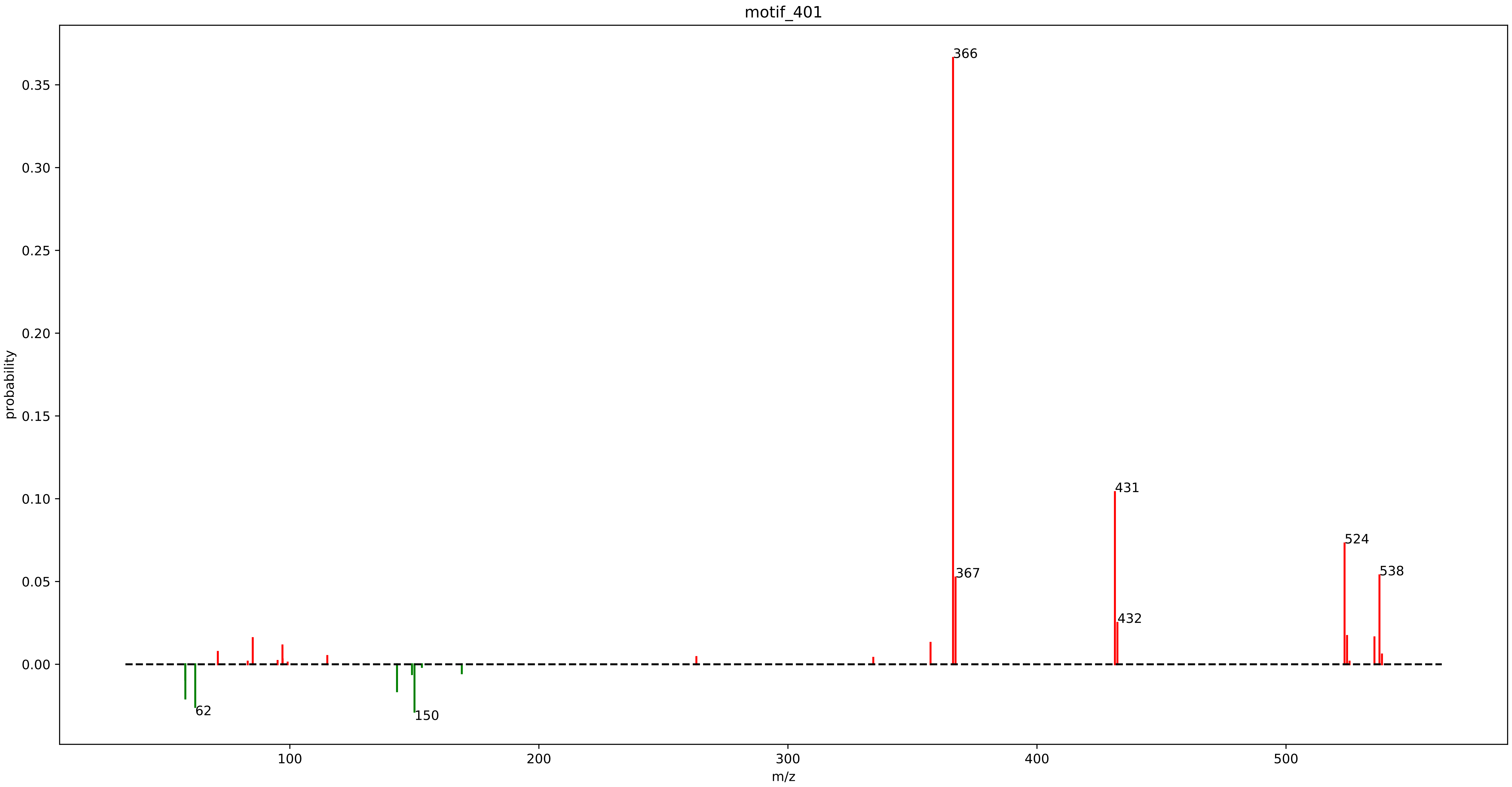

motif_401
366
367
431
432
524
538
62
150
0.35
0.30
0.25
0.20
0.15
0.10
0.05
0.00
probability
100
200
300
400
500
m/z

motif_401

fragment_366.2975	: 0.37
fragment_431.2975	: 0.10
fragment_523.5075	: 0.07
fragment_537.5225	: 0.05
fragment_367.3025	: 0.05
loss_150.0475	: 0.03
loss_62.0125	: 0.03
fragment_432.3025	: 0.03
loss_58.0375	: 0.02
fragment_524.5125	: 0.02
loss_143.0325	: 0.02
fragment_535.5075	: 0.02
fragment_85.1025	: 0.02
fragment_357.2625	: 0.01
fragment_97.0275	: 0.01
fragment_97.0375	: 0.01
loss_58.0025	: 0.01
fragment_71.0825	: 0.01
fragment_538.5275	: 0.01
loss_149.0475	: 0.01
loss_169.0475	: 0.01

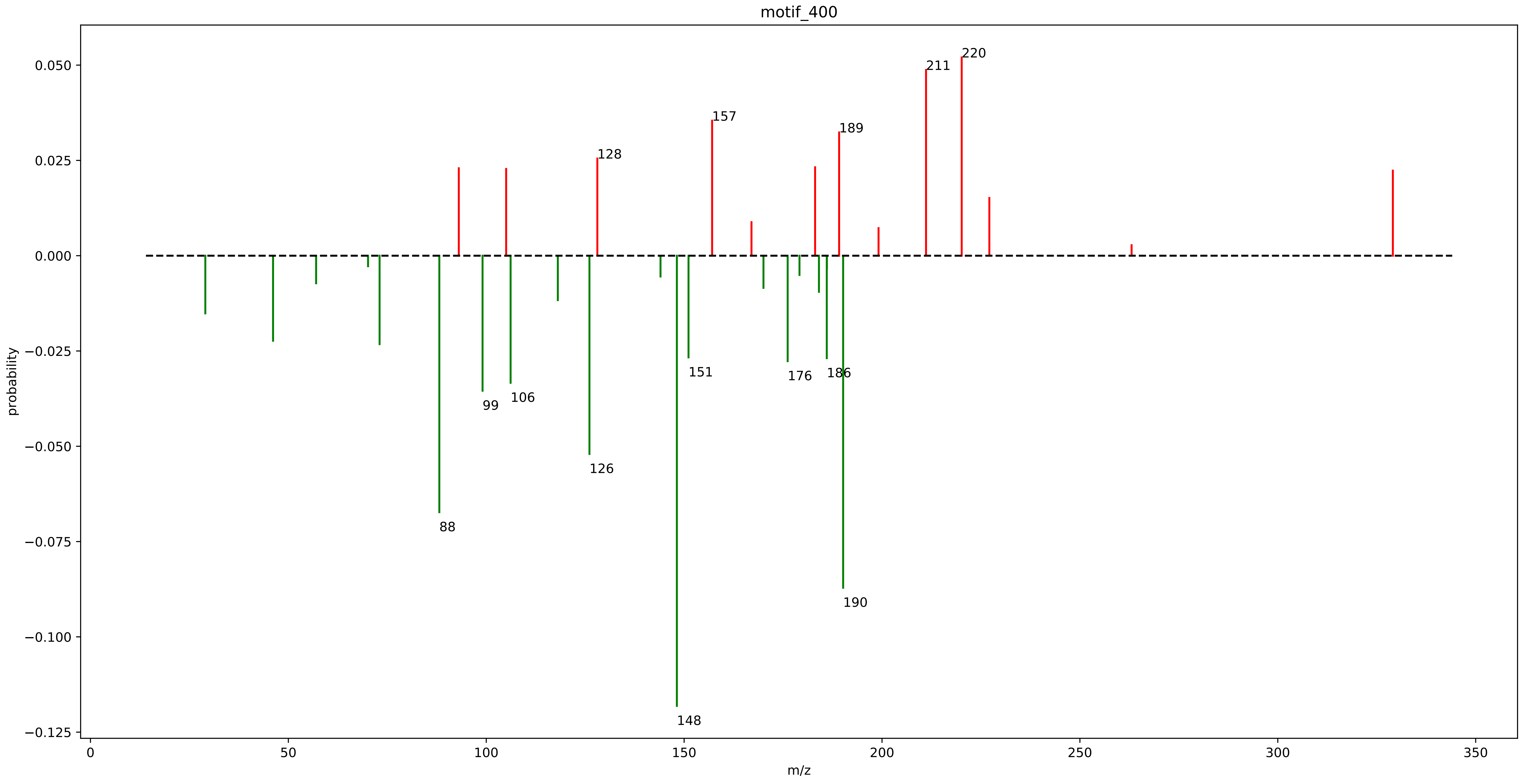

motif_400
probability
m/z
0.050
0.025
0.000
−0.025
−0.050
−0.075
−0.100
−0.125
0
50
100
150
200
250
300
350
220
211
157
189
128
88
99
106
126
148
151
176
186
190

motif_400

loss_148.1675	: 0.12
loss_190.1775	: 0.09
loss_88.1475	: 0.07
loss_126.0775	: 0.05
fragment_220.1325	: 0.05
fragment_211.1225	: 0.05
fragment_157.0725	: 0.04
loss_99.0675	: 0.04
loss_106.1575	: 0.03
fragment_189.1625	: 0.03
loss_176.1625	: 0.03
loss_186.0425	: 0.03
loss_151.1075	: 0.03
fragment_128.0725	: 0.03
fragment_183.0925	: 0.02
loss_73.0525	: 0.02
fragment_93.0675	: 0.02
fragment_105.0325	: 0.02
loss_46.1375	: 0.02
fragment_329.0725	: 0.02
loss_29.0225	: 0.02
fragment_227.1175	: 0.02
loss_118.0875	: 0.01
loss_184.0625	: 0.01
fragment_167.0175	: 0.01
loss_170.0475	: 0.01
fragment_199.1225	: 0.01
loss_57.0175	: 0.01
loss_144.0325	: 0.01
loss_179.1475	: 0.01

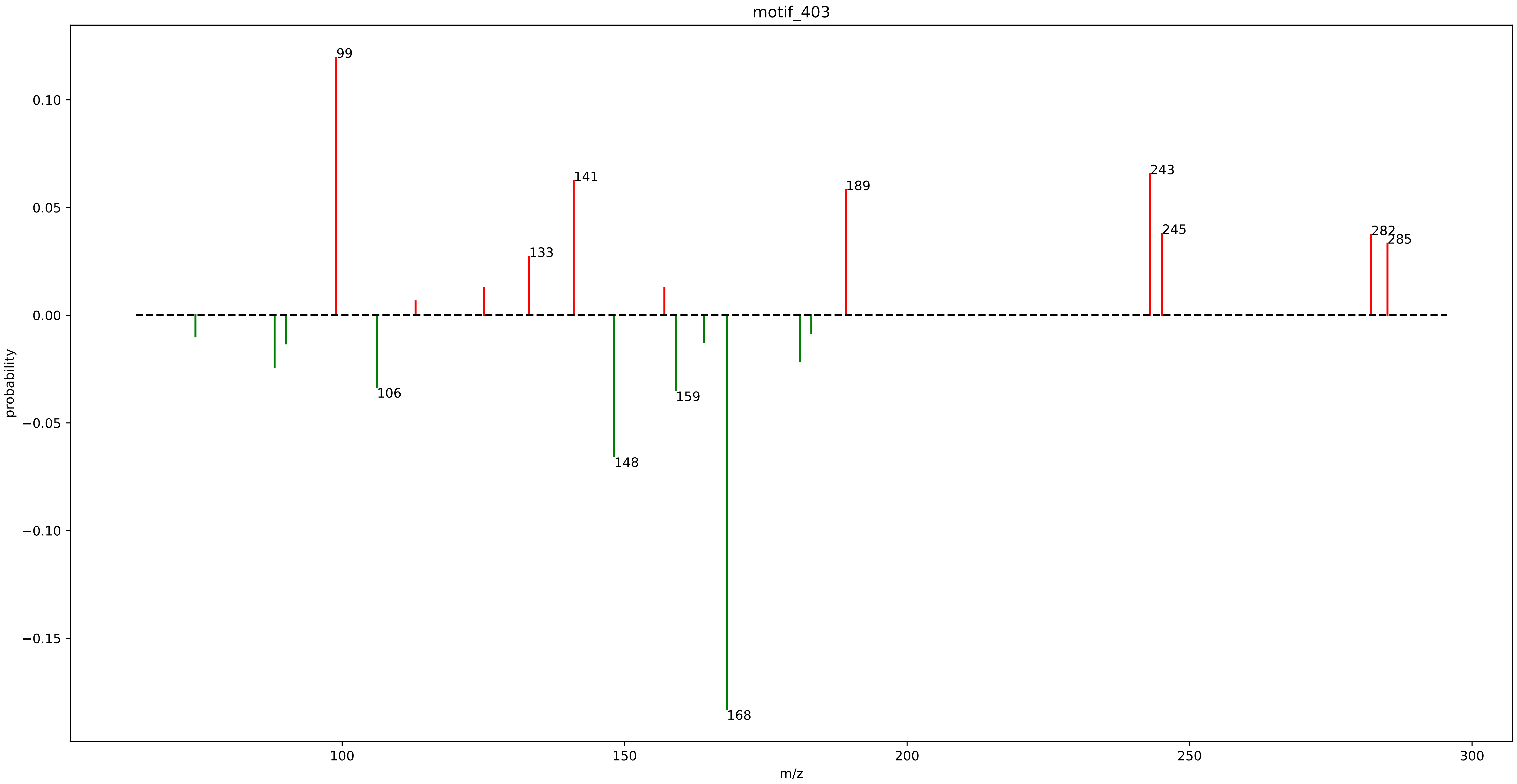

motif_403
probability
m/z
0.10
0.05
0.00
−0.05
−0.10
−0.15
100
150
200
250
300
99
141
189
243
245
133
282
285
106
159
148
168

motif_403

loss_168.0875	: 0.18
fragment_98.9725	: 0.12
loss_148.1775	: 0.07
fragment_243.0175	: 0.07
fragment_140.9875	: 0.06
fragment_189.1625	: 0.06
fragment_245.1275	: 0.04
fragment_282.1475	: 0.04
loss_159.0525	: 0.03
loss_106.1625	: 0.03
fragment_285.0275	: 0.03
fragment_133.1025	: 0.03
loss_88.0425	: 0.02
loss_181.0275	: 0.02
loss_90.0675	: 0.01
fragment_125.1075	: 0.01
loss_164.0075	: 0.01
fragment_157.0325	: 0.01
loss_74.0325	: 0.01
loss_183.0525	: 0.01
fragment_112.9925	: 0.01
fragment_140.9825	: 0.01

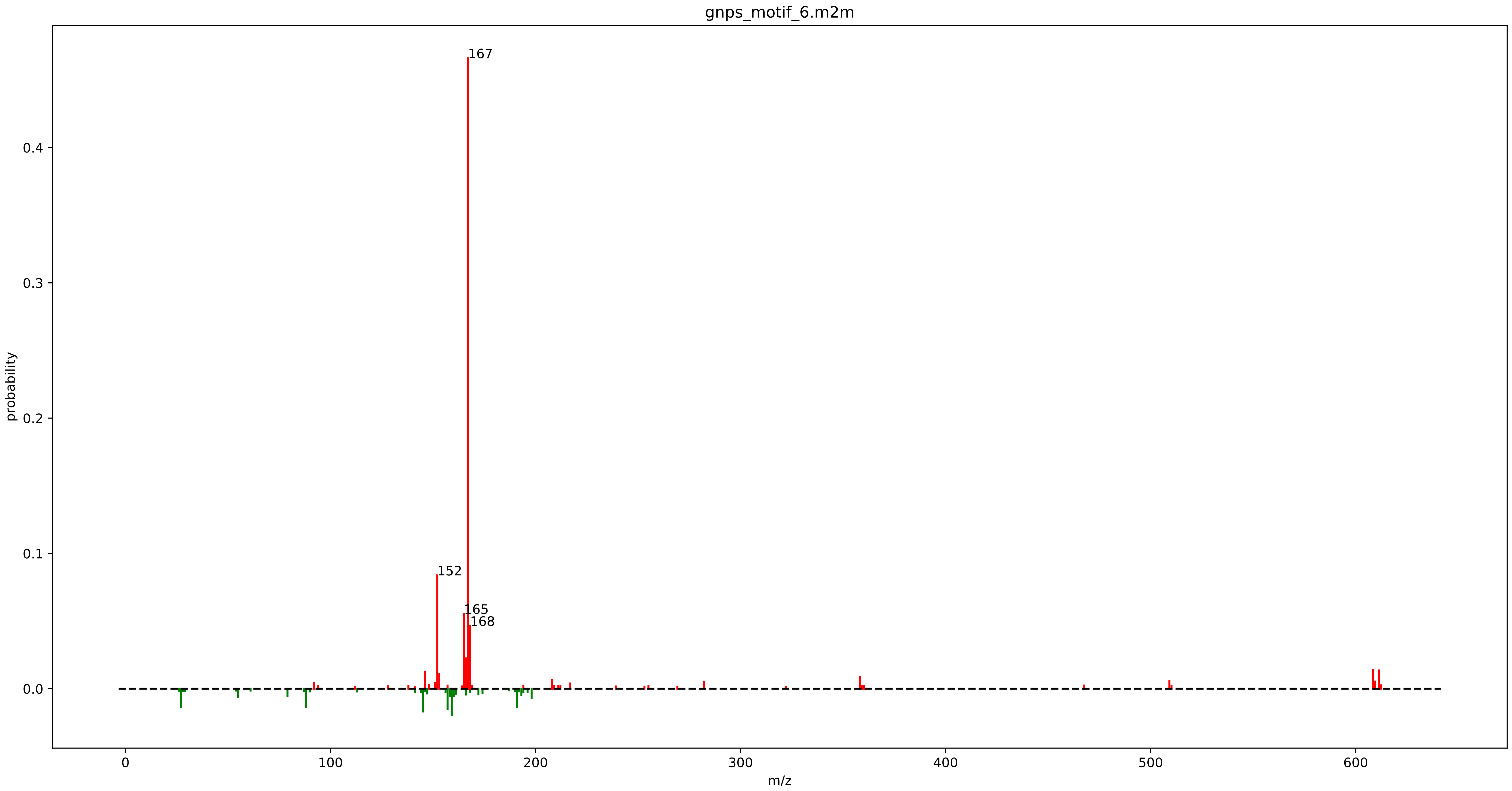

gnps_motif_6.m2m
probability
m/z
167
152
165
168
0.4
0.3
0.2
0.1
0.0
0
100
200
300
400
500
600

gnps_motif_6.m2m

fragment_167.0875	: 0.47
fragment_152.0625	: 0.08
fragment_165.0675	: 0.06
fragment_168.0875	: 0.05
fragment_166.0775	: 0.02
loss_159.1275	: 0.02
loss_145.1125	: 0.02
loss_157.1125	: 0.02
loss_191.0675	: 0.01
fragment_608.4025	: 0.01
loss_87.9775	: 0.01
loss_27.0125	: 0.01
fragment_611.2875	: 0.01
fragment_146.0725	: 0.01
fragment_153.0675	: 0.01
fragment_358.1575	: 0.01
loss_198.1025	: 0.01
fragment_168.0925	: 0.01
fragment_208.1325	: 0.01
loss_55.0325	: 0.01
fragment_509.1425	: 0.01
loss_160.1325	: 0.01
loss_79.0375	: 0.01
loss_158.1175	: 0.01
fragment_609.4075	: 0.01

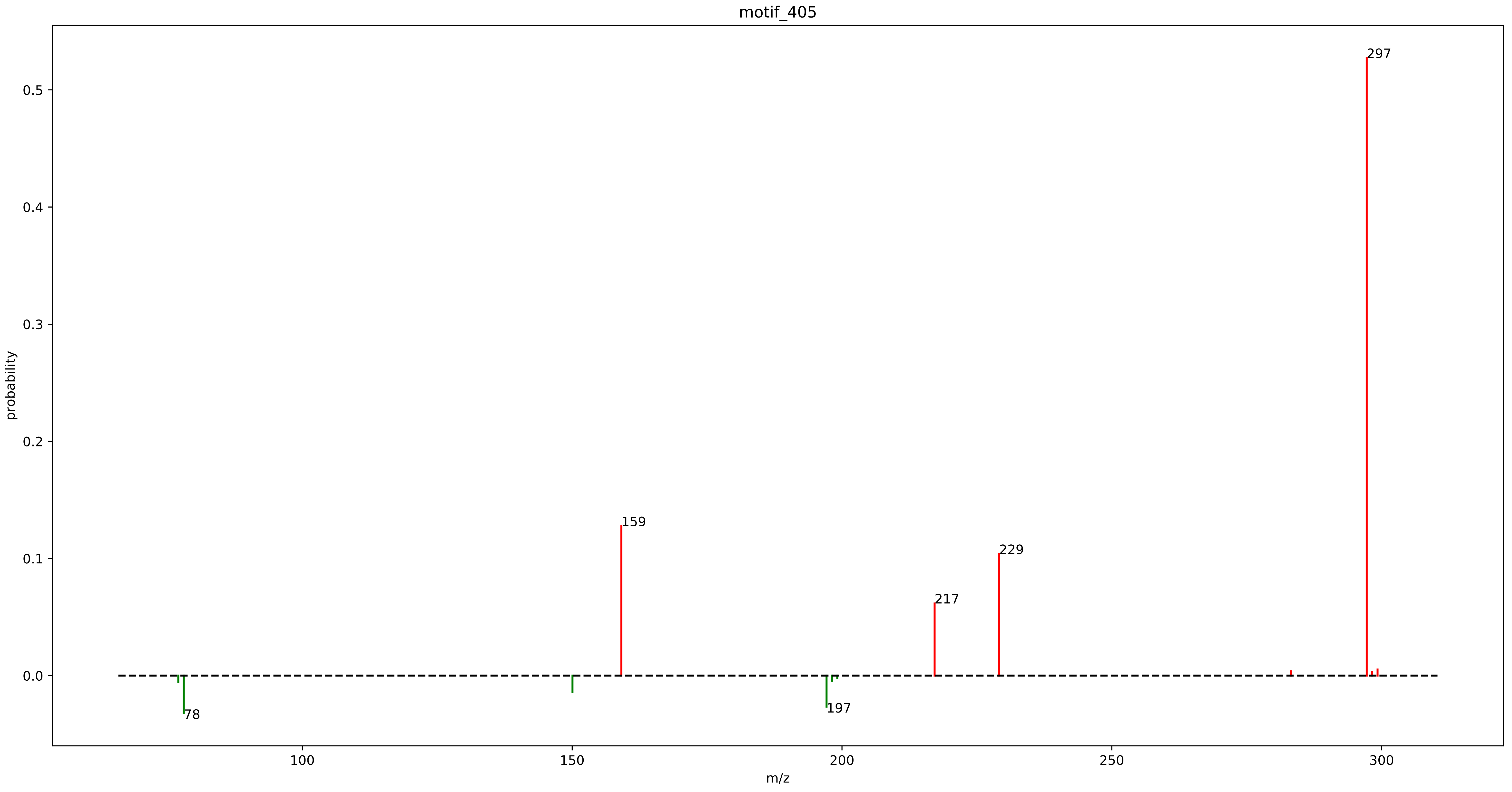

motif_405
297
159
229
217
78
197
0.5
0.4
0.3
0.2
0.1
0.0
probability
100
150
200
250
300
m/z

motif_405

fragment_297.2225	: 0.53
fragment_159.1175	: 0.13
fragment_229.1125	: 0.10
fragment_217.1575	: 0.06
loss_78.0325	: 0.03
loss_197.1375	: 0.03
loss_150.0625	: 0.01
loss_77.0275	: 0.01
fragment_299.2375	: 0.01

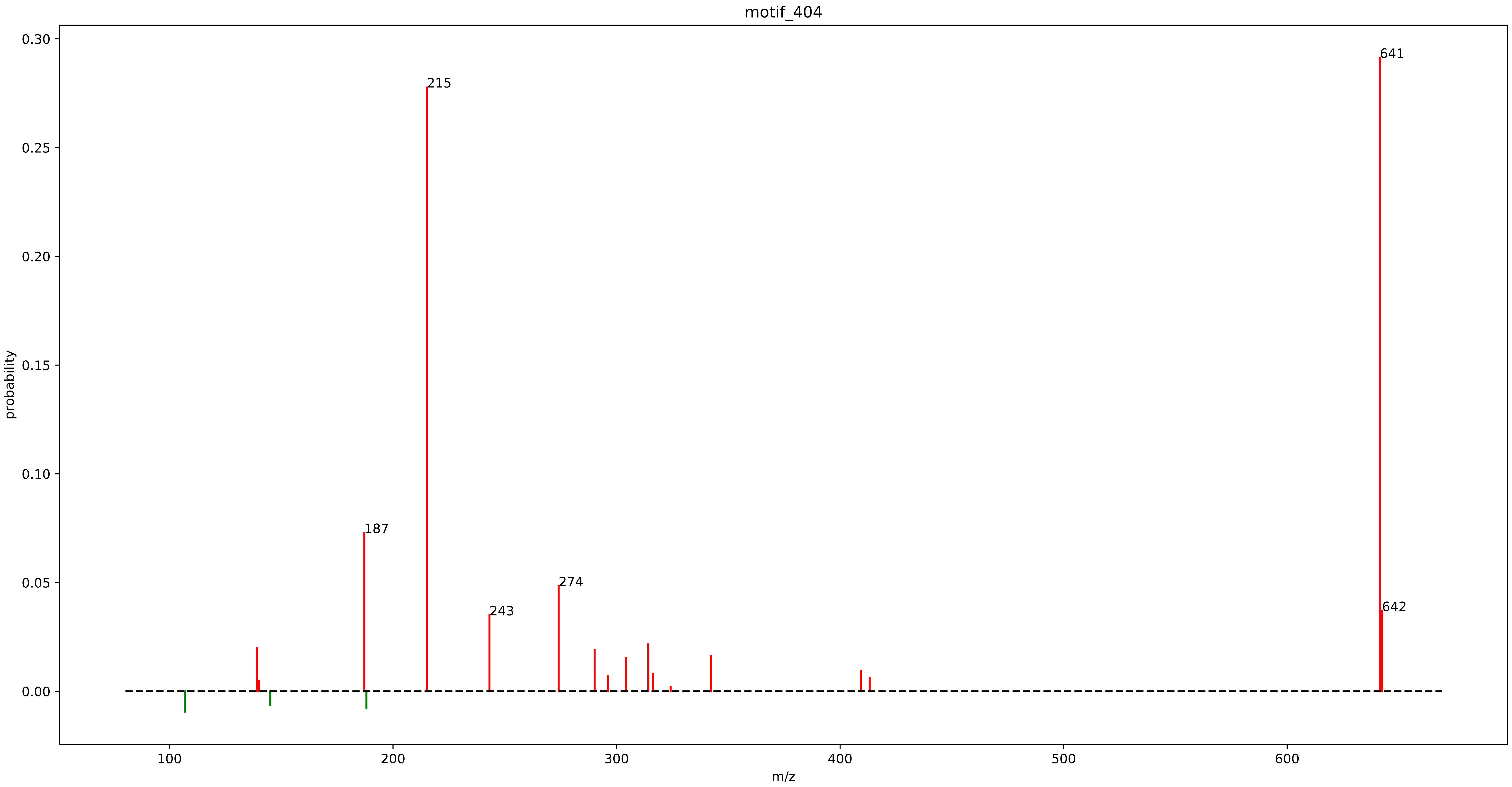

motif_404
probability
m/z
0.30
0.25
0.20
0.15
0.10
0.05
0.00
100
200
300
400
500
600
641
215
187
274
243
642

motif_404

fragment_641.4025	: 0.29
fragment_215.1375	: 0.28
fragment_187.1425	: 0.07
fragment_274.0875	: 0.05
fragment_642.4075	: 0.04
fragment_243.1425	: 0.03
fragment_314.2075	: 0.02
fragment_139.1325	: 0.02
fragment_290.1725	: 0.02
fragment_342.2025	: 0.02
fragment_304.1875	: 0.02
loss_107.0575	: 0.01
fragment_409.2775	: 0.01
fragment_316.2225	: 0.01
loss_188.1125	: 0.01
fragment_296.1975	: 0.01
loss_145.1075	: 0.01
fragment_413.2375	: 0.01

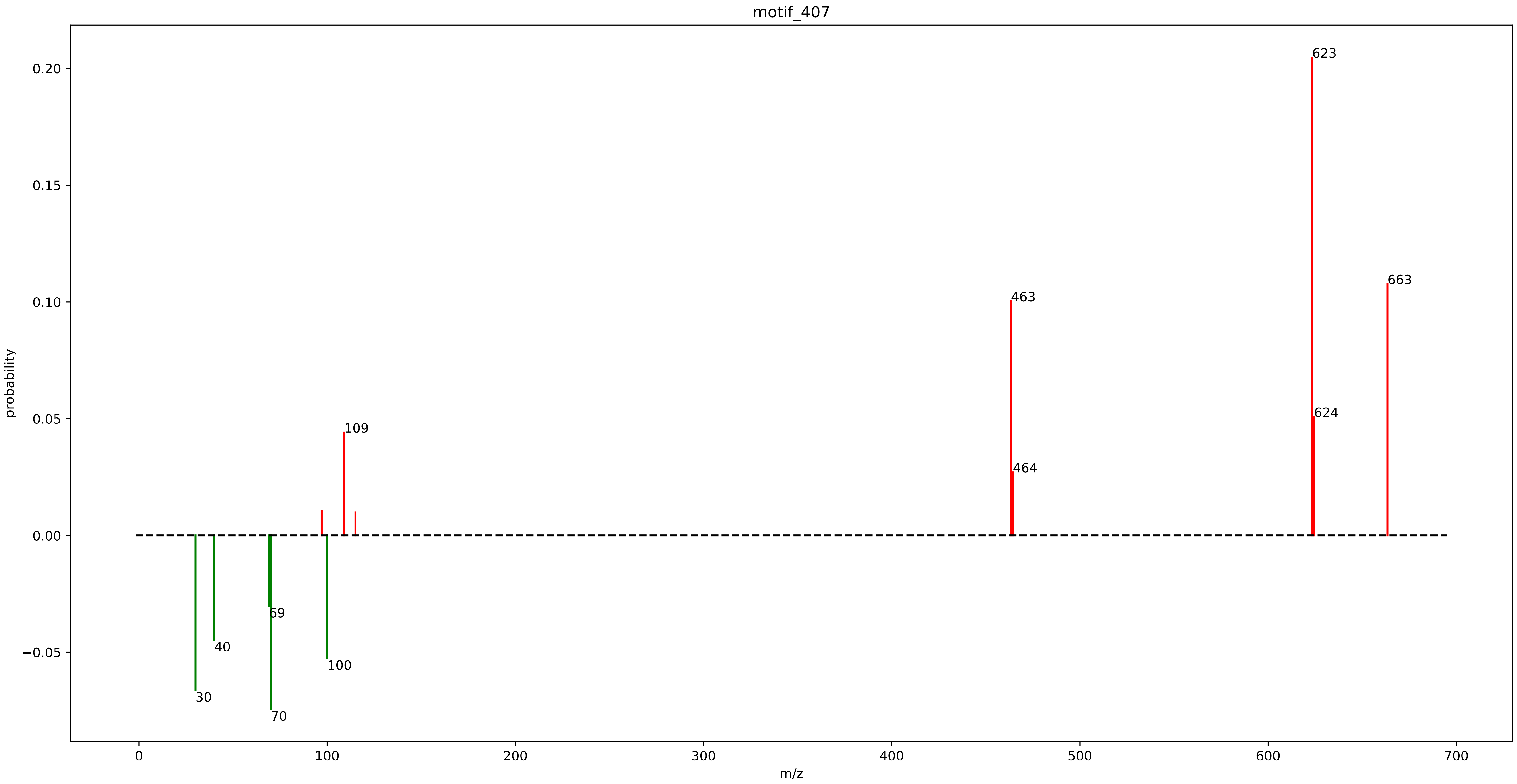

motif_407
probability
m/z
0.20
0.15
0.10
0.05
0.00
−0.05
0
100
200
300
400
500
600
700
623
663
463
624
109
464
69
40
100
30
70

motif_407

fragment_623.3825	: 0.20
fragment_663.4125	: 0.11
fragment_463.3625	: 0.10
loss_70.0375	: 0.07
loss_30.0075	: 0.07
loss_100.0475	: 0.05
fragment_624.3825	: 0.05
loss_40.0275	: 0.04
fragment_109.0425	: 0.04
loss_69.0375	: 0.03
fragment_464.3625	: 0.03
fragment_97.0275	: 0.01
fragment_115.0375	: 0.01

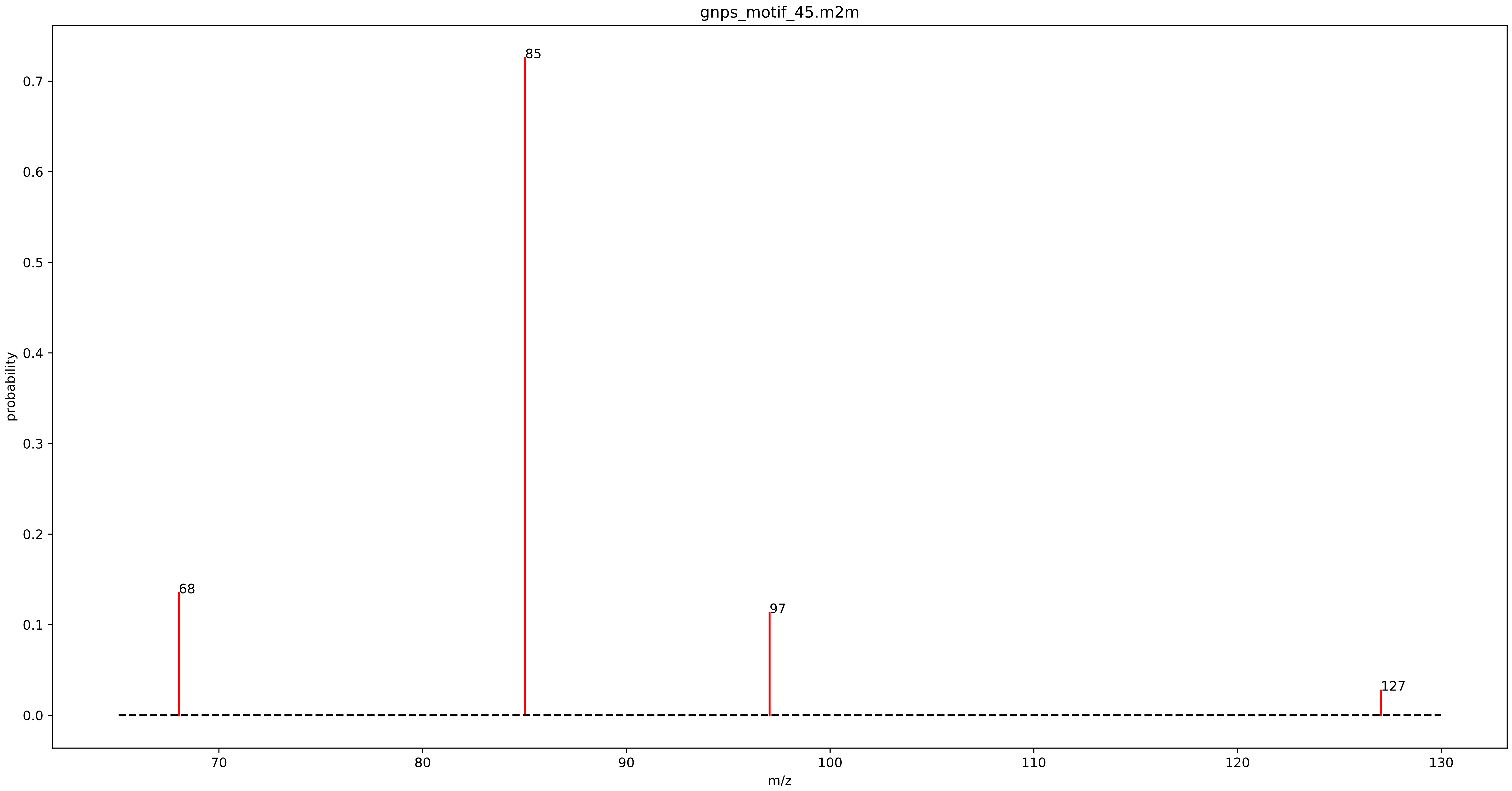
gnps_motif_45.m2m
probability
m/z
85
68
97
127
0.0
0.1
0.2
0.3
0.4
0.5
0.6
0.7
70
80
90
100
110
120
130

gnps_motif_45.m2m

fragment_85.0275	: 0.73
fragment_68.0275	: 0.13
fragment_97.0275	: 0.11
fragment_127.0375	: 0.03

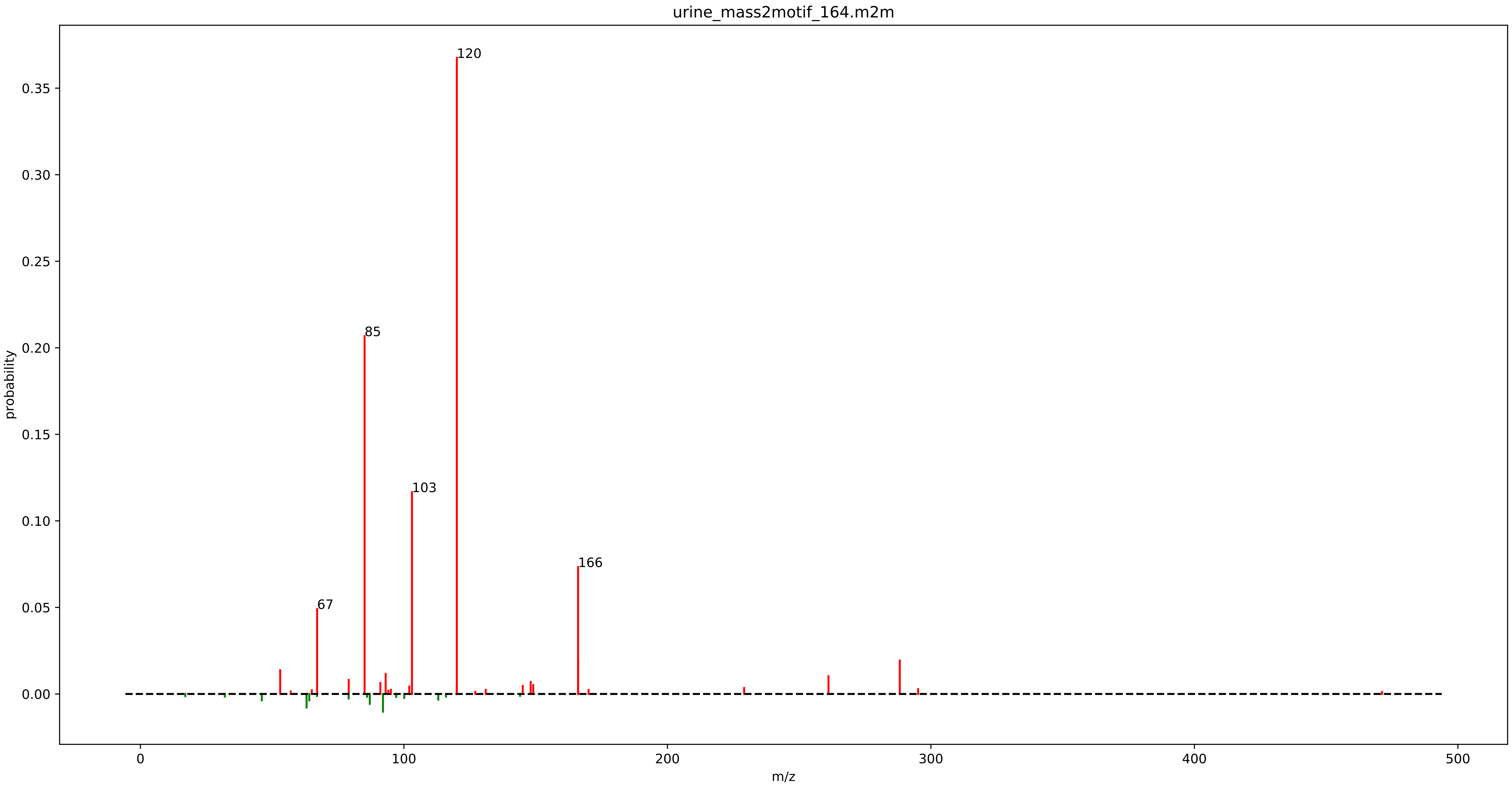

urine_mass2motif_164.m2m
probability
m/z
120
85
103
166
67
0.35
0.30
0.25
0.20
0.15
0.10
0.05
0.00
0
100
200
300
400
500

urine_mass2motif_164.m2m

fragment_120.0825	: 0.37
fragment_85.0625	: 0.21
fragment_103.0525	: 0.12
fragment_166.0875	: 0.07
fragment_67.0525	: 0.05
fragment_288.1825	: 0.02
fragment_166.1075	: 0.02
fragment_53.0375	: 0.01
fragment_93.0675	: 0.01
fragment_261.1225	: 0.01
loss_92.0475	: 0.01
fragment_79.0525	: 0.01
loss_63.0525	: 0.01
fragment_148.0975	: 0.01
fragment_91.0525	: 0.01
loss_87.0425	: 0.01
fragment_149.0625	: 0.01

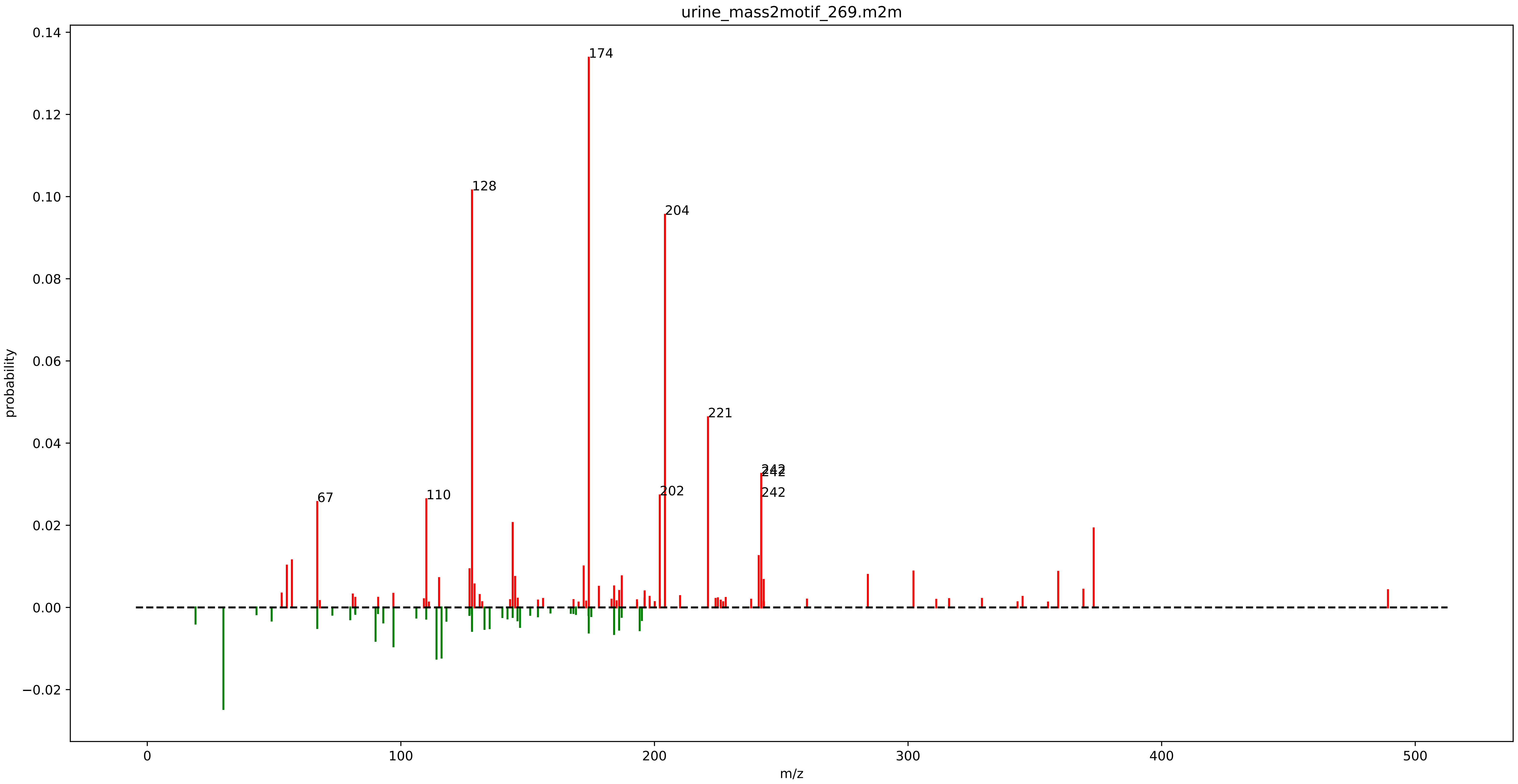

urine_mass2motif_269.m2m
probability
m/z
0.14
0.12
0.10
0.08
0.06
0.04
0.02
0.00
−0.02
0
100
200
300
400
500
174
128
204
221
242
242
242
202
67
110

urine_mass2motif_269.m2m

fragment_174.0925	: 0.13	fragment_127.0625	: 0.01	loss_67.0175	: 0.01
fragment_128.0475	: 0.10	fragment_302.1375	: 0.01		
fragment_204.1225	: 0.10	fragment_359.1925	: 0.01		
fragment_221.0975	: 0.05	loss_90.0125	: 0.01		
fragment_242.1025	: 0.03	fragment_284.1475	: 0.01		
fragment_242.1175	: 0.03	fragment_187.0975	: 0.01		
fragment_202.0825	: 0.03	fragment_145.0475	: 0.01		
fragment_242.0775	: 0.03	fragment_115.0525	: 0.01		
fragment_110.0325	: 0.03	fragment_243.0825	: 0.01		
fragment_67.0175	: 0.03	loss_184.0675	: 0.01		
loss_30.0125	: 0.02	loss_174.0825	: 0.01		
fragment_144.0825	: 0.02	loss_128.0475	: 0.01		
fragment_373.1925	: 0.02	fragment_129.0475	: 0.01		
fragment_241.1075	: 0.01	loss_194.1625	: 0.01		
loss_114.0475	: 0.01	loss_186.0625	: 0.01		
loss_116.0425	: 0.01	loss_132.9675	: 0.01		
fragment_57.0325	: 0.01	fragment_184.0775	: 0.01		
fragment_55.0275	: 0.01	loss_128.0325	: 0.01		
fragment_172.0775	: 0.01	loss_135.0075	: 0.01		
loss_97.0625	: 0.01	fragment_178.0775	: 0.01		

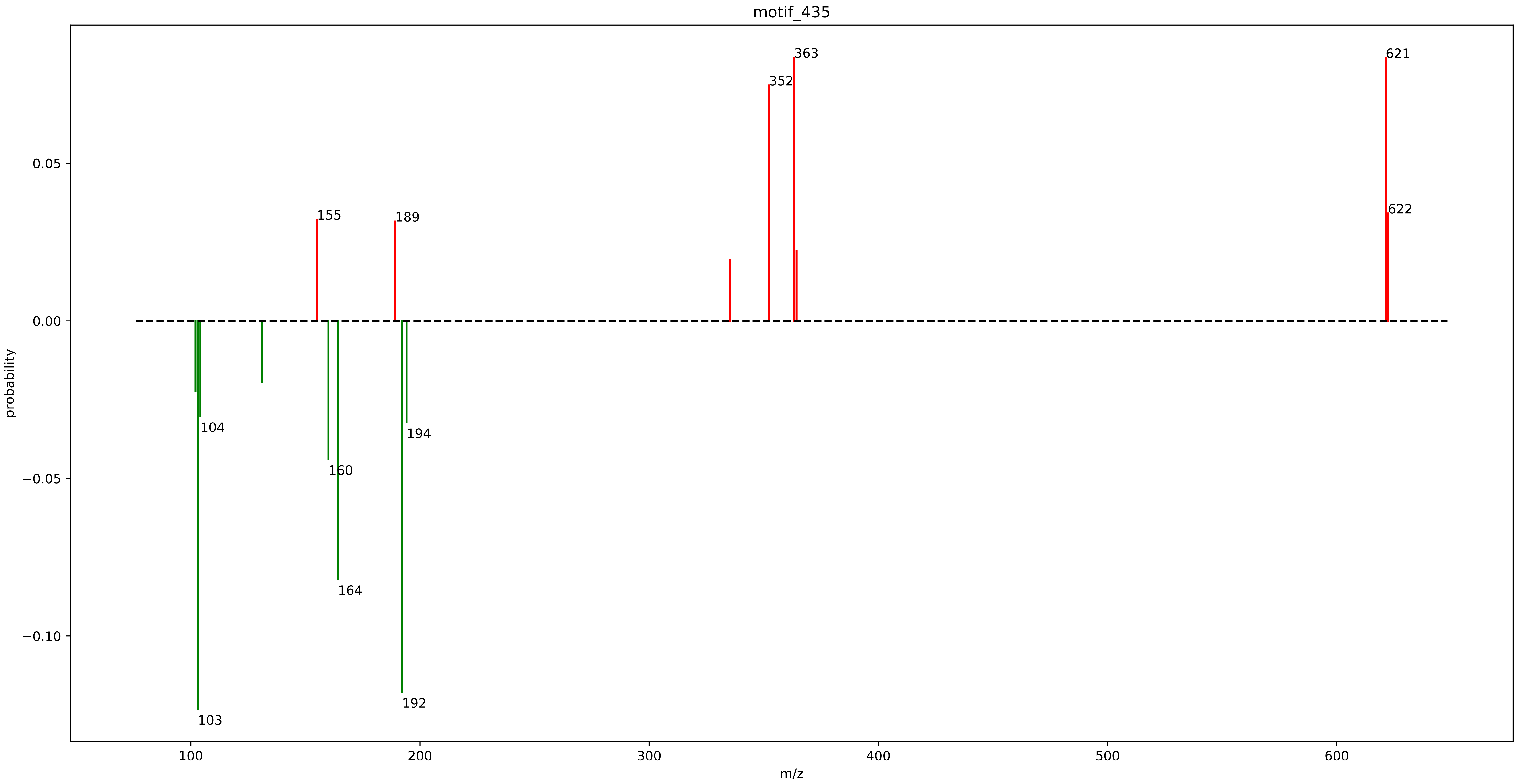

motif_435
probability
m/z
0.05
0.00
−0.05
−0.10
100
200
300
400
500
600
155
189
352
363
621
622
104
103
160
164
194
192

motif_435

loss_103.0625	: 0.12
loss_192.1575	: 0.12
fragment_363.2625	: 0.08
fragment_621.3125	: 0.08
loss_164.1625	: 0.08
fragment_352.2875	: 0.07
loss_160.0275	: 0.04
fragment_622.3175	: 0.03
loss_194.1725	: 0.03
fragment_155.0175	: 0.03
fragment_189.1625	: 0.03
loss_104.1375	: 0.03
fragment_364.2675	: 0.02
loss_102.0575	: 0.02
loss_131.0575	: 0.02
fragment_335.2675	: 0.02

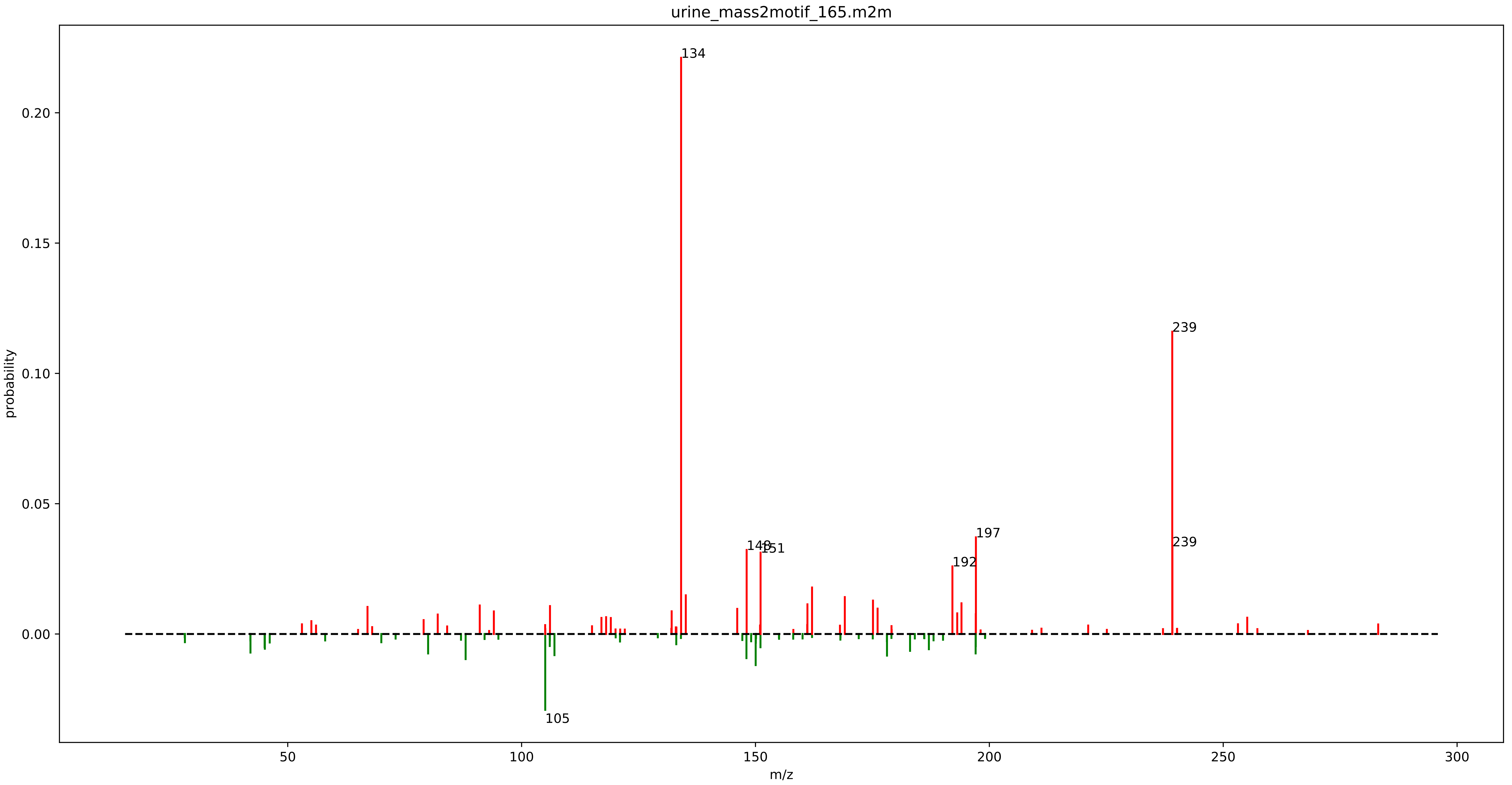

urine_mass2motif_165.m2m
probability
m/z
0.20
0.15
0.10
0.05
0.00
50
100
150
200
250
300
134
239
197
239
148
151
192
105

urine_mass2motif_165.m2m

fragment_134.0975	: 0.22
fragment_239.1025	: 0.12
fragment_197.1275	: 0.04
fragment_239.1375	: 0.03
fragment_148.1125	: 0.03
fragment_151.0875	: 0.03
loss_105.0525	: 0.03
fragment_192.1025	: 0.03
fragment_162.0925	: 0.02
fragment_135.1025	: 0.01
fragment_169.0975	: 0.01
fragment_175.1225	: 0.01
loss_150.0425	: 0.01
fragment_194.0425	: 0.01
fragment_161.1075	: 0.01
fragment_91.0525	: 0.01
fragment_106.0675	: 0.01
fragment_67.0525	: 0.01
fragment_176.1075	: 0.01
fragment_146.0975	: 0.01
loss_88.0325	: 0.01
loss_148.0675	: 0.01
fragment_132.0825	: 0.01
fragment_94.0675	: 0.01
loss_178.1175	: 0.01
loss_107.0225	: 0.01
fragment_193.1325	: 0.01
fragment_197.0925	: 0.01
fragment_82.0675	: 0.01
loss_80.0125	: 0.01
loss_197.0675	: 0.01
loss_42.0325	: 0.01
loss_183.0475	: 0.01
fragment_118.0675	: 0.01
fragment_255.1325	: 0.01
fragment_117.0675	: 0.01
fragment_119.0725	: 0.01
loss_187.0725	: 0.01
loss_45.1075	: 0.01
fragment_79.0525	: 0.01
loss_151.0675	: 0.01
loss_45.0575	: 0.01

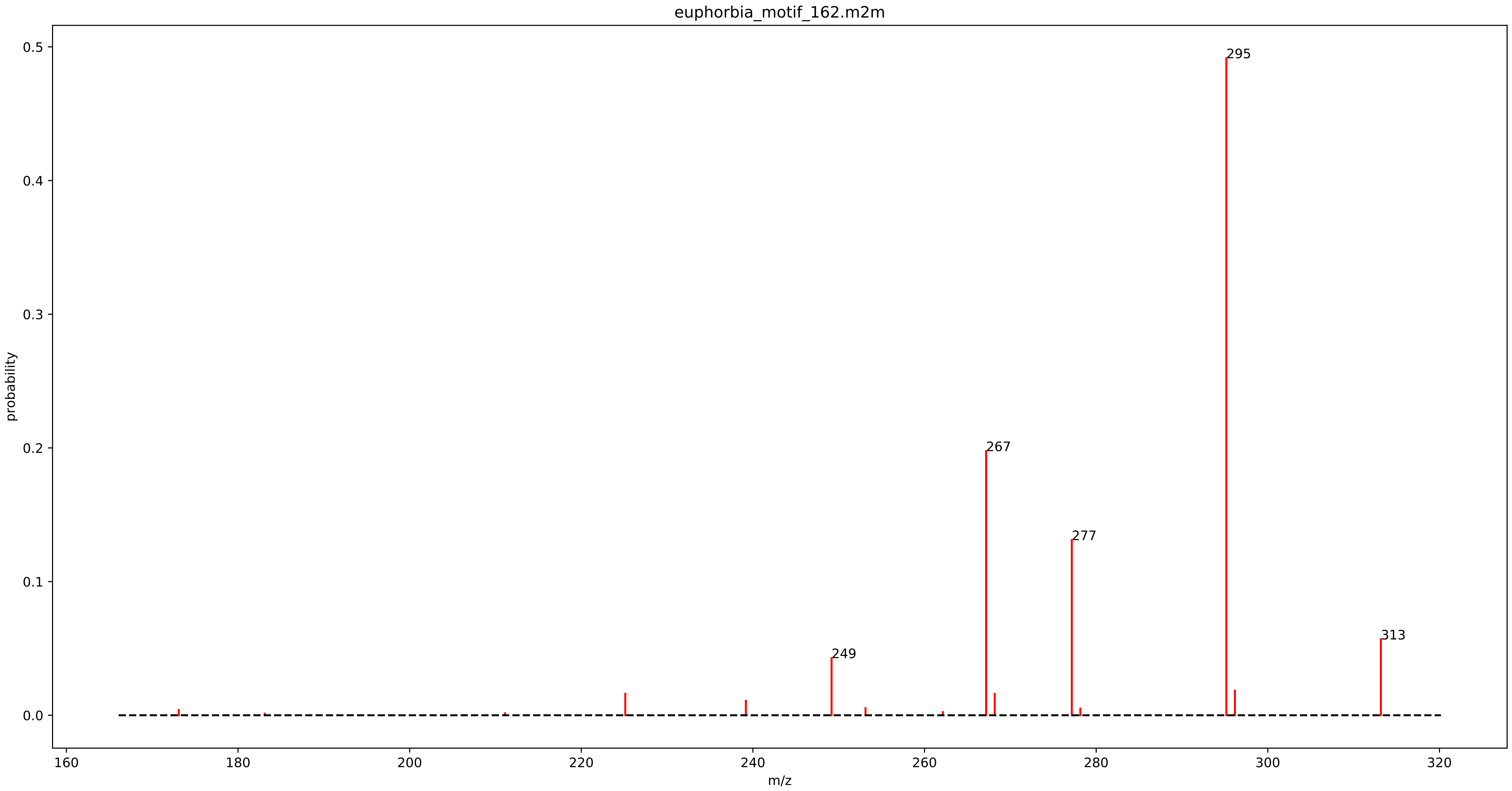

euphorbia_motif_162.m2m
probability
m/z
0.0
0.1
0.2
0.3
0.4
0.5
160
180
200
220
240
260
280
300
320
295
267
277
249
313

euphorbia_motif_162.m2m

fragment_295.1675	: 0.49
fragment_267.1725	: 0.20
fragment_277.1575	: 0.13
fragment_313.1775	: 0.06
fragment_249.1625	: 0.04
fragment_296.1675	: 0.02
fragment_225.1275	: 0.02
fragment_268.1775	: 0.02
fragment_239.1775	: 0.01
fragment_253.1175	: 0.01

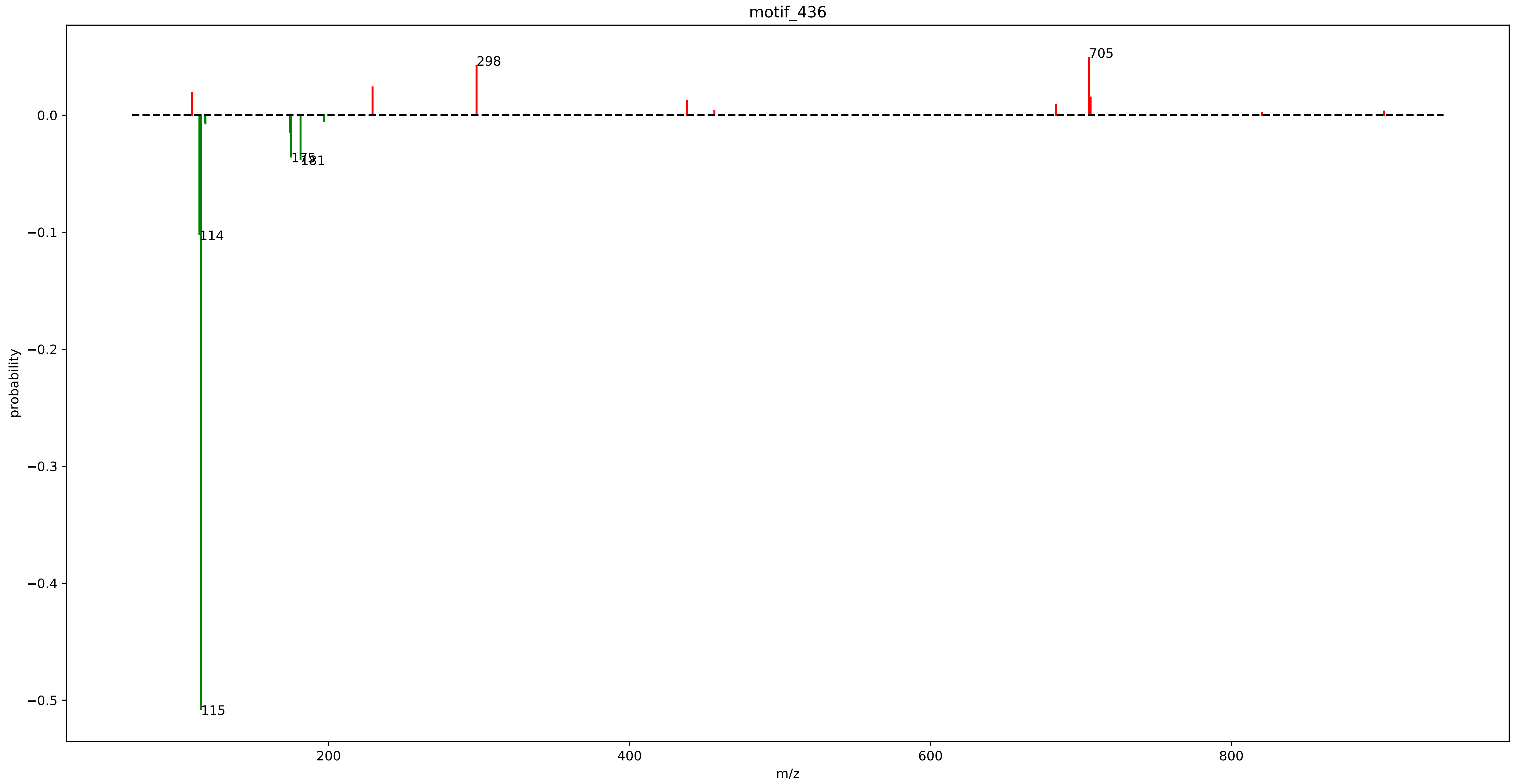

motif_436
probability
m/z
0.0
−0.1
−0.2
−0.3
−0.4
−0.5
200
400
600
800
298
705
175
181
114
115

motif_436

loss_115.0825	: 0.51
loss_114.0775	: 0.10
fragment_705.3975	: 0.05
fragment_298.3075	: 0.04
loss_181.3125	: 0.04
loss_175.1025	: 0.04
fragment_229.1825	: 0.02
fragment_109.0425	: 0.02
fragment_706.4025	: 0.02
loss_174.1025	: 0.01
fragment_438.3425	: 0.01
fragment_109.0575	: 0.01
fragment_683.4175	: 0.01
loss_118.0925	: 0.01
loss_117.5875	: 0.01

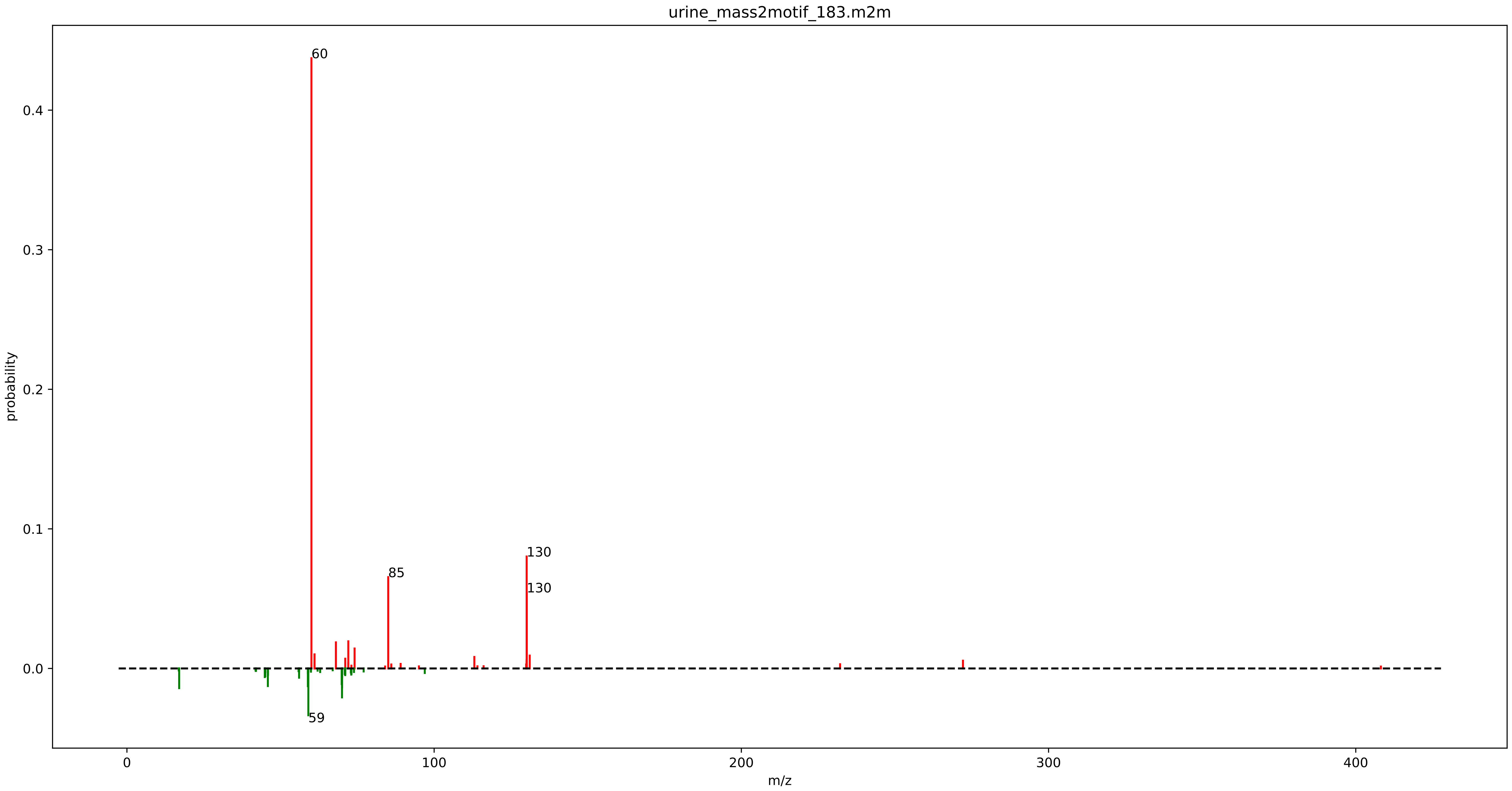
urine_mass2motif_183.m2m
probability
m/z
0.4
0.3
0.2
0.1
0.0
0
100
200
300
400
60
85
130
130
59

urine_mass2motif_183.m2m

fragment_60.0575	: 0.44
fragment_130.1075	: 0.08
fragment_85.0525	: 0.07
fragment_130.1575	: 0.05
loss_59.0475	: 0.03
loss_70.0075	: 0.02
fragment_72.0625	: 0.02
fragment_68.0225	: 0.02
fragment_74.0975	: 0.01
loss_16.9975	: 0.01
loss_70.0325	: 0.01
loss_58.8975	: 0.01
loss_45.8775	: 0.01
loss_69.9025	: 0.01
fragment_61.0575	: 0.01
fragment_61.0525	: 0.01
fragment_131.1125	: 0.01
fragment_113.0825	: 0.01
fragment_71.0625	: 0.01
loss_56.0375	: 0.01
loss_70.0625	: 0.01
loss_44.8725	: 0.01
fragment_130.0975	: 0.01
loss_45.0325	: 0.01
fragment_272.1375	: 0.01

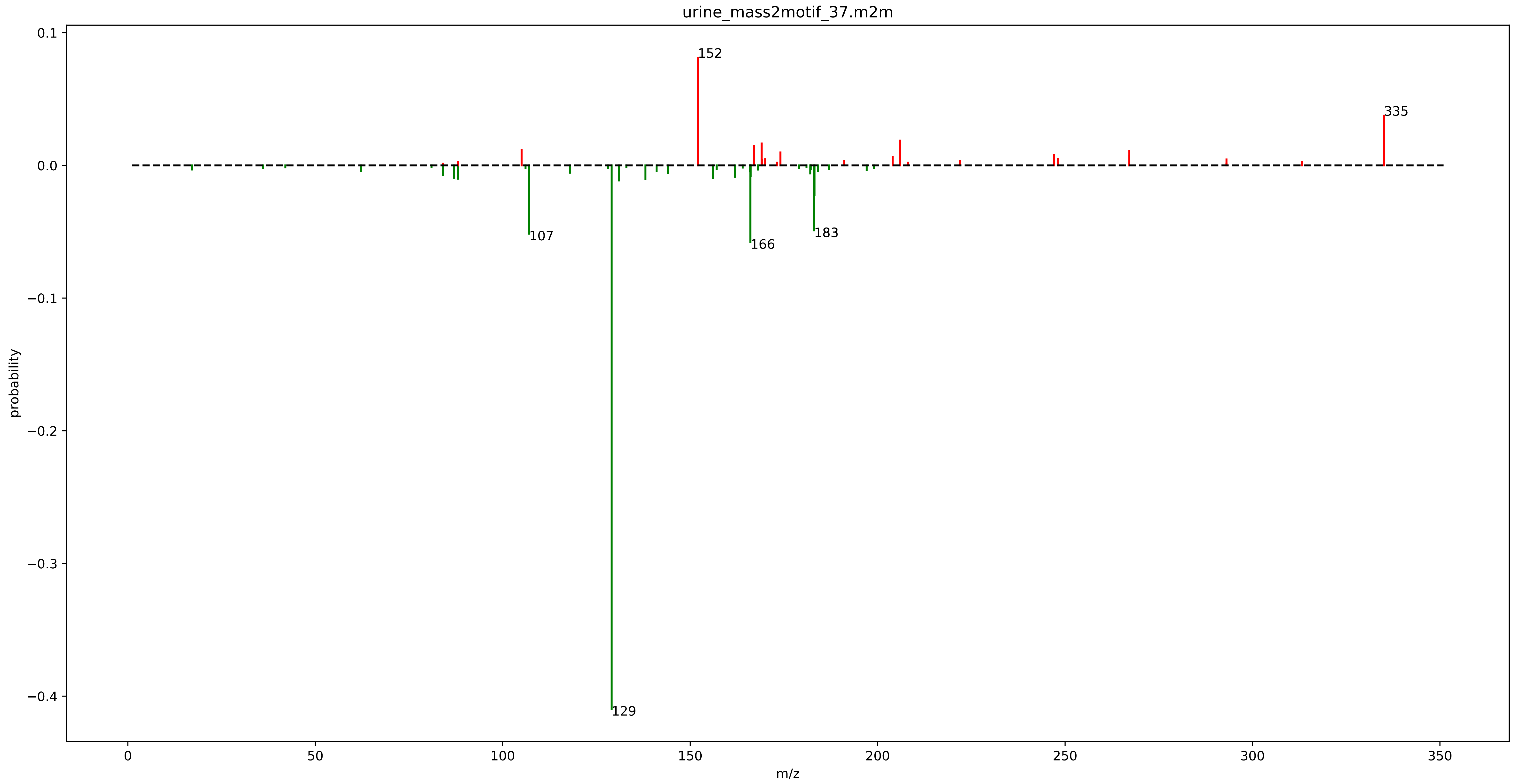

urine_mass2motif_37.m2m
152
335
107
166
183
129
probability
m/z
0.1
0.0
−0.1
−0.2
−0.3
−0.4
0
50
100
150
200
250
300
350

urine_mass2motif_37.m2m

loss_129.0425	: 0.41
fragment_152.0325	: 0.08
loss_166.0625	: 0.06
loss_107.0575	: 0.05
loss_183.0475	: 0.05
fragment_335.0675	: 0.04
loss_183.1625	: 0.02
fragment_206.0275	: 0.02
fragment_169.0625	: 0.02
fragment_167.0275	: 0.01
fragment_105.0375	: 0.01
loss_131.0675	: 0.01
fragment_267.1375	: 0.01
loss_138.0675	: 0.01
loss_88.0325	: 0.01
fragment_174.0775	: 0.01
loss_156.0775	: 0.01
loss_87.0425	: 0.01
loss_162.0325	: 0.01
fragment_247.0525	: 0.01
loss_166.1375	: 0.01
fragment_267.1325	: 0.01
loss_84.0225	: 0.01
fragment_204.0075	: 0.01
loss_182.0375	: 0.01
loss_144.0725	: 0.01
loss_118.0025	: 0.01

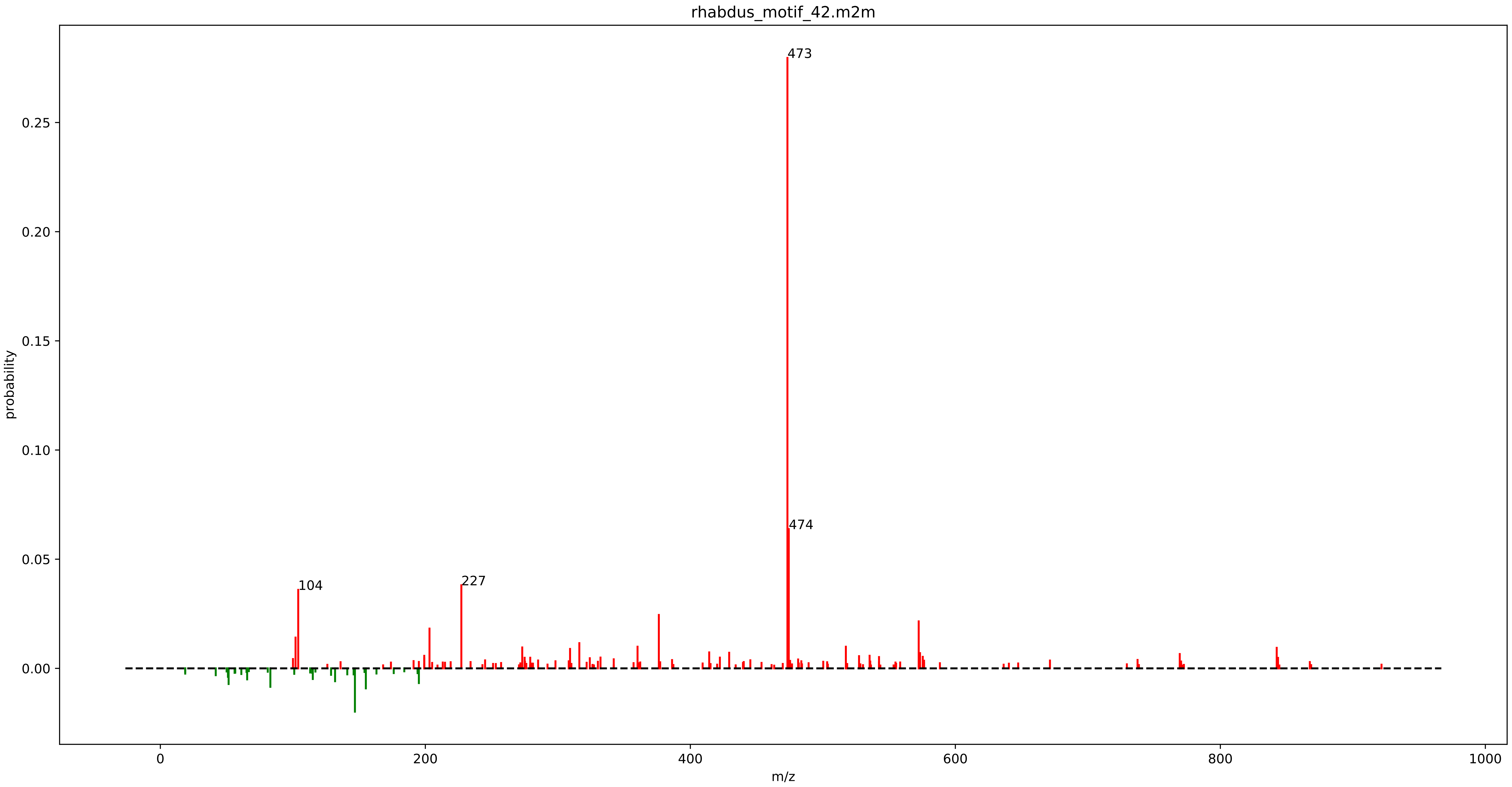

rhabdus_motif_42.m2m
probability
m/z
0.00
0.05
0.10
0.15
0.20
0.25
0
200
400
600
800
1000
473
474
104
227

rhabdus_motif_42.m2m

fragment_473.2725	: 0.28
fragment_474.2775	: 0.06
fragment_227.1775	: 0.04
fragment_104.0675	: 0.04
fragment_376.2225	: 0.02
fragment_572.3425	: 0.02
loss_146.8575	: 0.02
fragment_203.1375	: 0.02
fragment_102.0375	: 0.01
fragment_316.2225	: 0.01
fragment_360.1475	: 0.01
fragment_517.3725	: 0.01
fragment_273.1275	: 0.01
fragment_842.5375	: 0.01
loss_155.1025	: 0.01
fragment_309.2175	: 0.01
loss_83.0375	: 0.01
fragment_414.2375	: 0.01
loss_51.5275	: 0.01
fragment_429.3075	: 0.01
fragment_573.3475	: 0.01
loss_195.1225	: 0.01
fragment_769.3575	: 0.01
loss_131.8775	: 0.01
fragment_535.2825	: 0.01
fragment_199.1775	: 0.01
fragment_527.3225	: 0.01
fragment_575.4125	: 0.01
fragment_542.3925	: 0.01
loss_65.5275	: 0.01

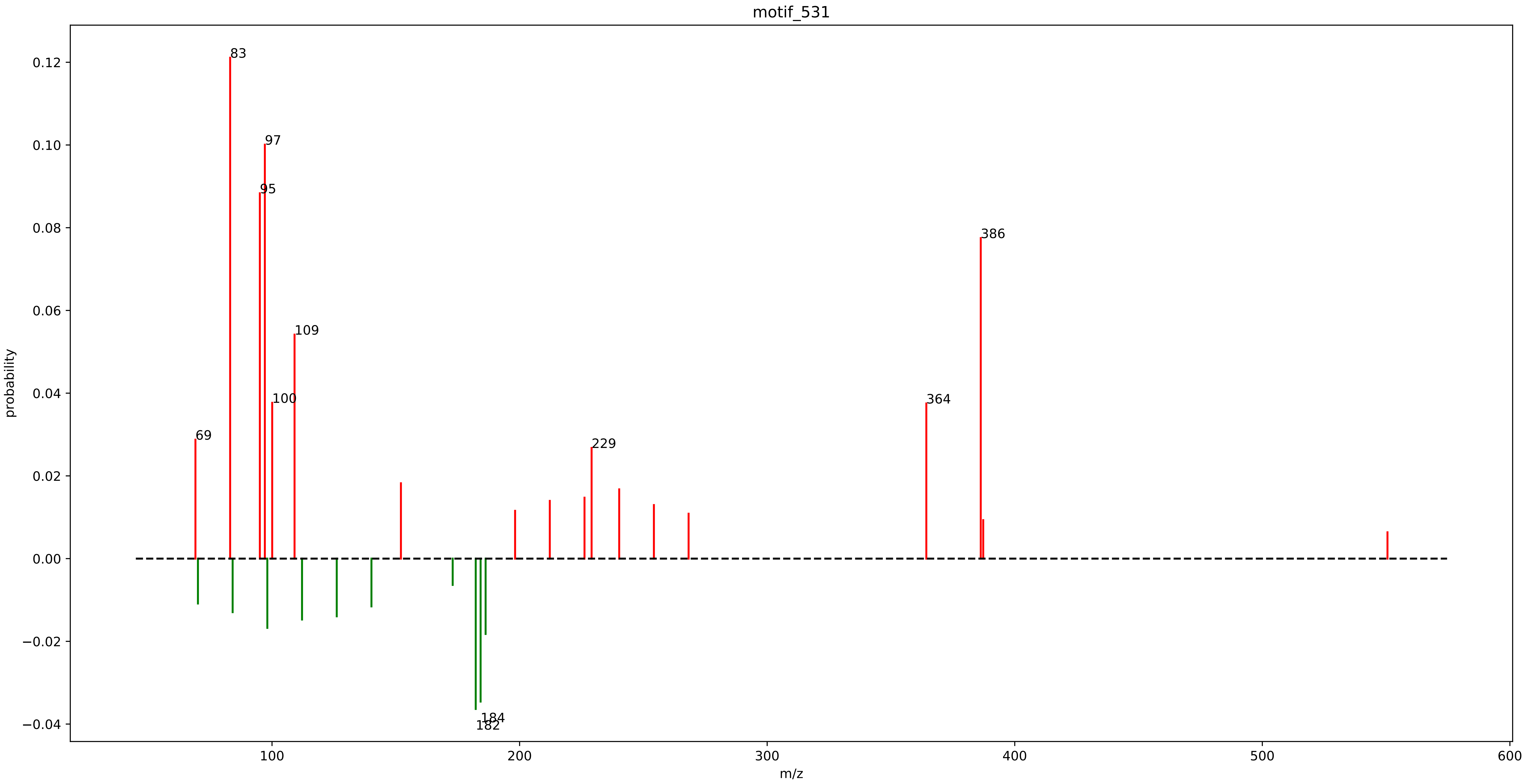

motif_531
0.12
0.10
0.08
0.06
0.04
0.02
0.00
−0.02
−0.04
probability
83
97
95
109
100
69
229
364
386
184
182
100
200
300
400
500
600
m/z

motif_531

fragment_83.0825	: 0.12
fragment_97.1025	: 0.10
fragment_95.0825	: 0.09
fragment_386.2675	: 0.08
fragment_109.0975	: 0.05
fragment_100.0725	: 0.04
fragment_364.2875	: 0.04
loss_182.2775	: 0.04
loss_184.2525	: 0.03
fragment_69.0675	: 0.03
fragment_229.0825	: 0.03
loss_186.2725	: 0.02
fragment_152.0675	: 0.02
fragment_240.2325	: 0.02
loss_98.1075	: 0.02
loss_112.1225	: 0.01
fragment_226.2175	: 0.01
loss_126.1425	: 0.01
fragment_212.1975	: 0.01
fragment_254.2475	: 0.01
loss_84.0925	: 0.01
loss_140.1575	: 0.01
fragment_198.1825	: 0.01
fragment_268.2625	: 0.01
loss_70.0775	: 0.01
fragment_387.2675	: 0.01
loss_172.9725	: 0.01
fragment_550.5525	: 0.01

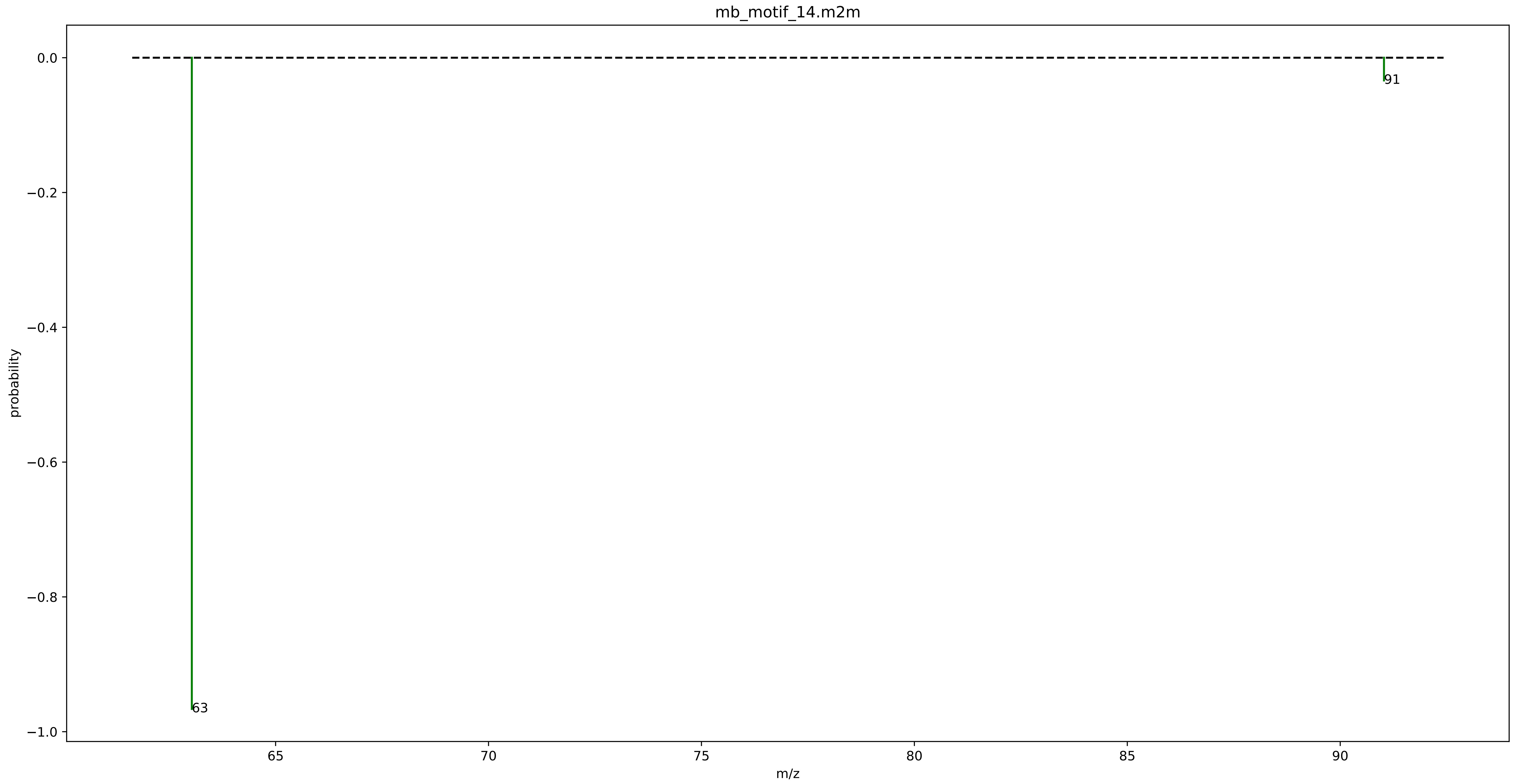

mb_motif_14.m2m
0.0
−0.2
−0.4
−0.6
−0.8
−1.0
probability
63
91
65
70
75
80
85
90
m/z

mb_motif_14.m2m

loss_63.0325	: 0.97
loss_91.0275	: 0.03

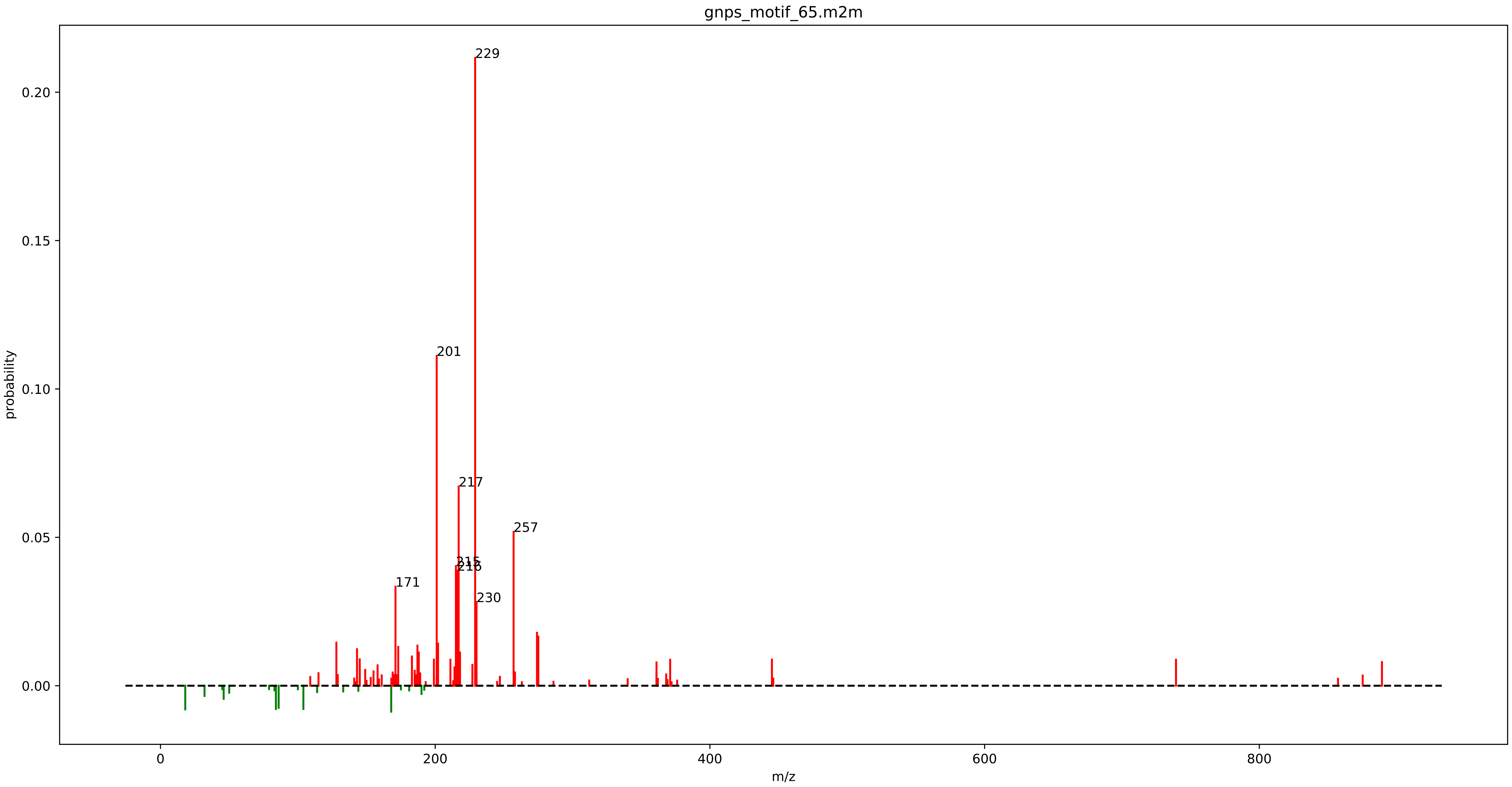

gnps_motif_65.m2m
probability
m/z
0.20
0.15
0.10
0.05
0.00
0
200
400
600
800
229
201
217
257
215
216
171
230

gnps_motif_65.m2m

fragment_229.0875	: 0.21
fragment_201.0925	: 0.11
fragment_217.0875	: 0.07
fragment_257.0825	: 0.05
fragment_215.0725	: 0.04
fragment_216.0775	: 0.04
fragment_171.0825	: 0.03
fragment_230.0875	: 0.03
fragment_274.1075	: 0.02
fragment_275.0925	: 0.02
fragment_128.0625	: 0.01
fragment_202.0925	: 0.01
fragment_187.0775	: 0.01
fragment_173.0975	: 0.01
fragment_143.0875	: 0.01
fragment_218.0875	: 0.01
fragment_188.0825	: 0.01
fragment_183.0825	: 0.01
fragment_145.0625	: 0.01
fragment_199.0775	: 0.01
fragment_445.1625	: 0.01
fragment_211.0775	: 0.01
loss_167.9875	: 0.01
fragment_739.3475	: 0.01
fragment_371.0725	: 0.01
fragment_889.3025	: 0.01
loss_18.0375	: 0.01
loss_104.0475	: 0.01
loss_84.0325	: 0.01
fragment_361.1325	: 0.01
loss_86.0375	: 0.01
fragment_227.0825	: 0.01
fragment_158.0725	: 0.01
fragment_214.0625	: 0.01
fragment_145.1025	: 0.01
fragment_149.0425	: 0.01
fragment_185.0975	: 0.01

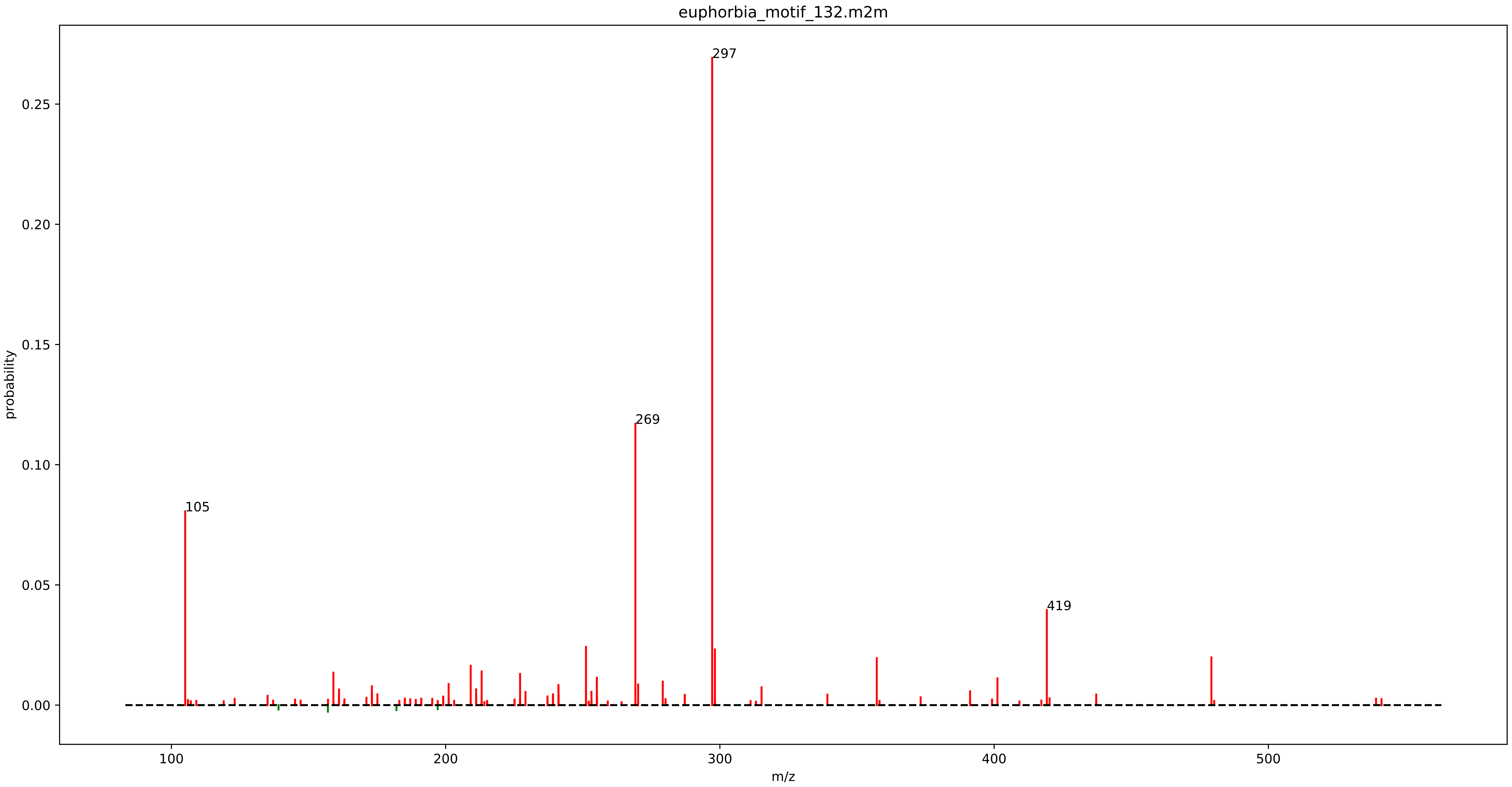

euphorbia_motif_132.m2m
probability
m/z
0.25
0.20
0.15
0.10
0.05
0.00
100
200
300
400
500
297
269
105
419

euphorbia_motif_132.m2m

fragment_297.1825	: 0.27
fragment_269.1875	: 0.12
fragment_105.0325	: 0.08
fragment_419.2175	: 0.04
fragment_251.1775	: 0.02
fragment_298.1875	: 0.02
fragment_479.2375	: 0.02
fragment_357.2025	: 0.02
fragment_209.1325	: 0.02
fragment_213.1275	: 0.01
fragment_159.0775	: 0.01
fragment_227.1425	: 0.01
fragment_255.1375	: 0.01
fragment_401.2075	: 0.01
fragment_279.1725	: 0.01
fragment_201.0875	: 0.01
fragment_270.1925	: 0.01
fragment_241.1225	: 0.01
fragment_173.0925	: 0.01
fragment_241.1575	: 0.01
fragment_315.1925	: 0.01
fragment_211.1075	: 0.01
fragment_161.1325	: 0.01
fragment_173.1325	: 0.01
fragment_391.2275	: 0.01
fragment_251.1825	: 0.01
fragment_253.1575	: 0.01
fragment_229.1175	: 0.01

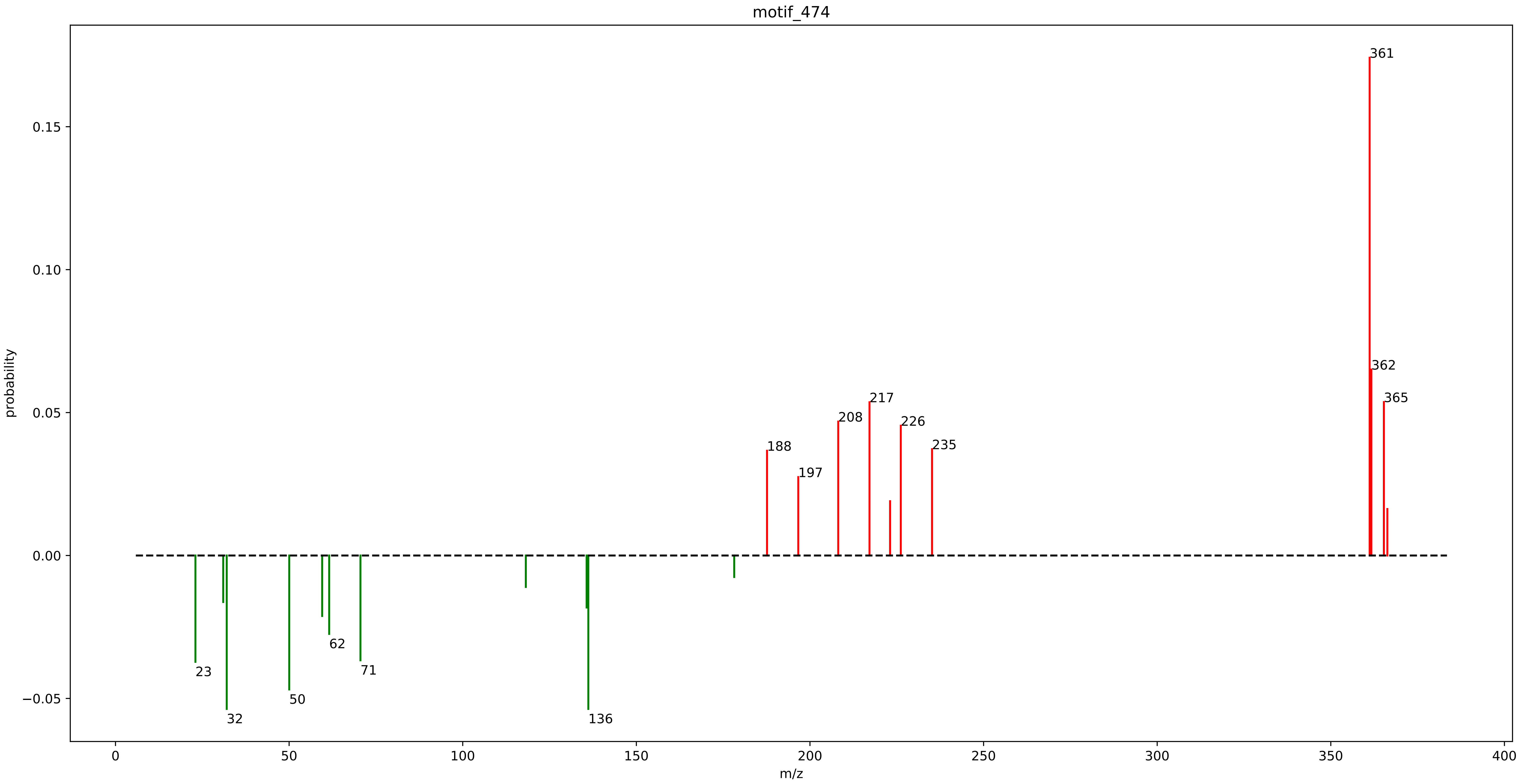

motif_474
probability
m/z
0.15
0.10
0.05
0.00
−0.05
0
50
100
150
200
250
300
350
400
361
362
365
217
208
226
188
235
197
23
32
50
62
71
136

motif_474

fragment_361.1825	: 0.17
fragment_361.6875	: 0.07
fragment_365.2825	: 0.05
loss_32.0275	: 0.05
loss_136.1775	: 0.05
fragment_217.1475	: 0.05
fragment_208.1425	: 0.05
loss_50.0325	: 0.05
fragment_226.1525	: 0.05
fragment_235.1575	: 0.04
loss_23.0175	: 0.04
loss_70.5425	: 0.04
fragment_187.6325	: 0.04
fragment_196.6325	: 0.03
loss_61.5375	: 0.03
loss_59.5175	: 0.02
fragment_223.0725	: 0.02
loss_135.6725	: 0.02
loss_31.0225	: 0.02
fragment_366.2875	: 0.02
loss_118.1675	: 0.01
loss_178.1875	: 0.01

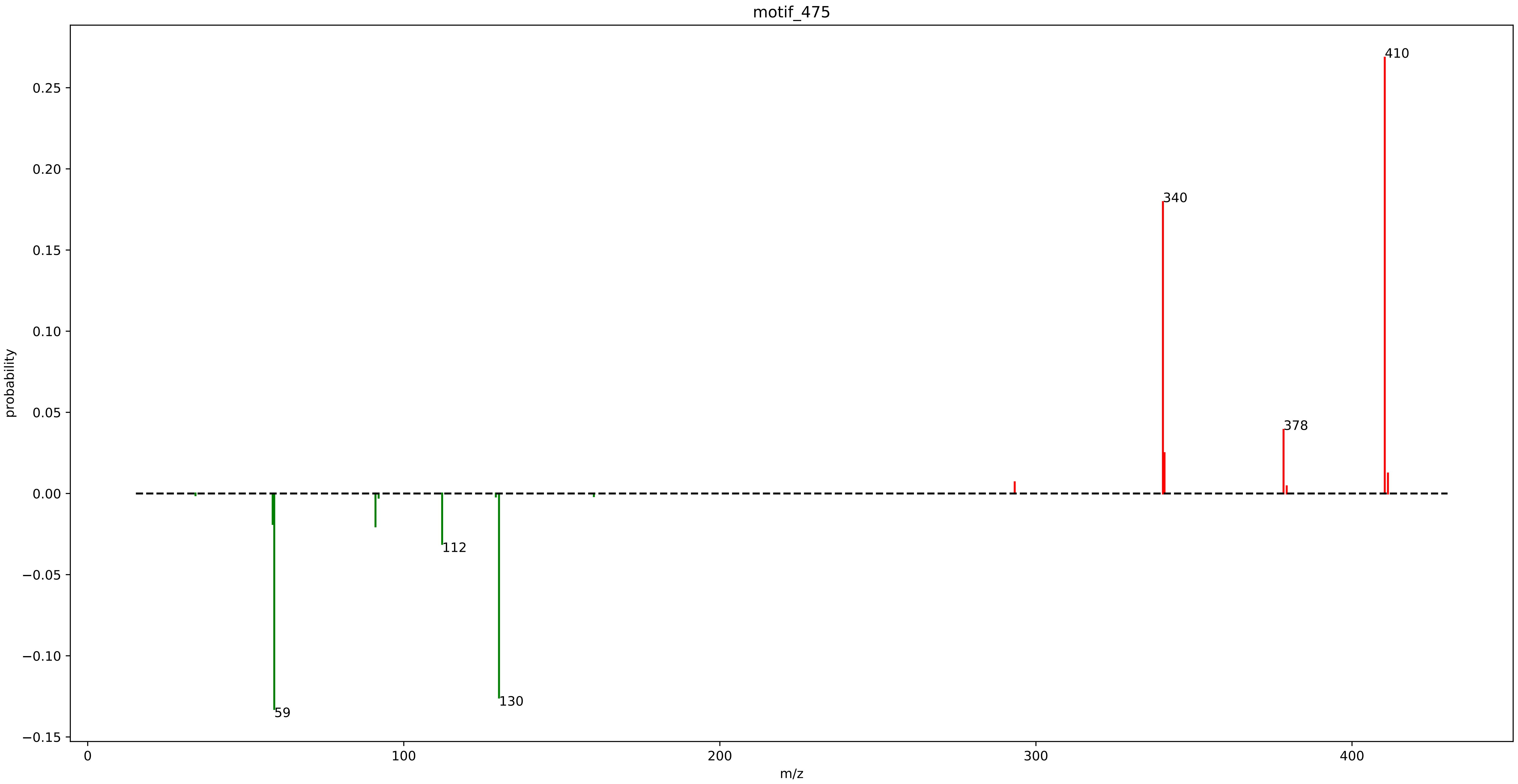

motif_475
probability
m/z
0.25
0.20
0.15
0.10
0.05
0.00
−0.05
−0.10
−0.15
0
100
200
300
400
410
340
378
112
59
130

motif_475

fragment_410.3625	: 0.27
fragment_340.1775	: 0.18
loss_59.0225	: 0.13
loss_130.1325	: 0.13
fragment_378.3375	: 0.04
loss_112.1375	: 0.03
fragment_340.6825	: 0.02
loss_91.0475	: 0.02
loss_58.5175	: 0.02
fragment_411.3675	: 0.01
fragment_293.2825	: 0.01

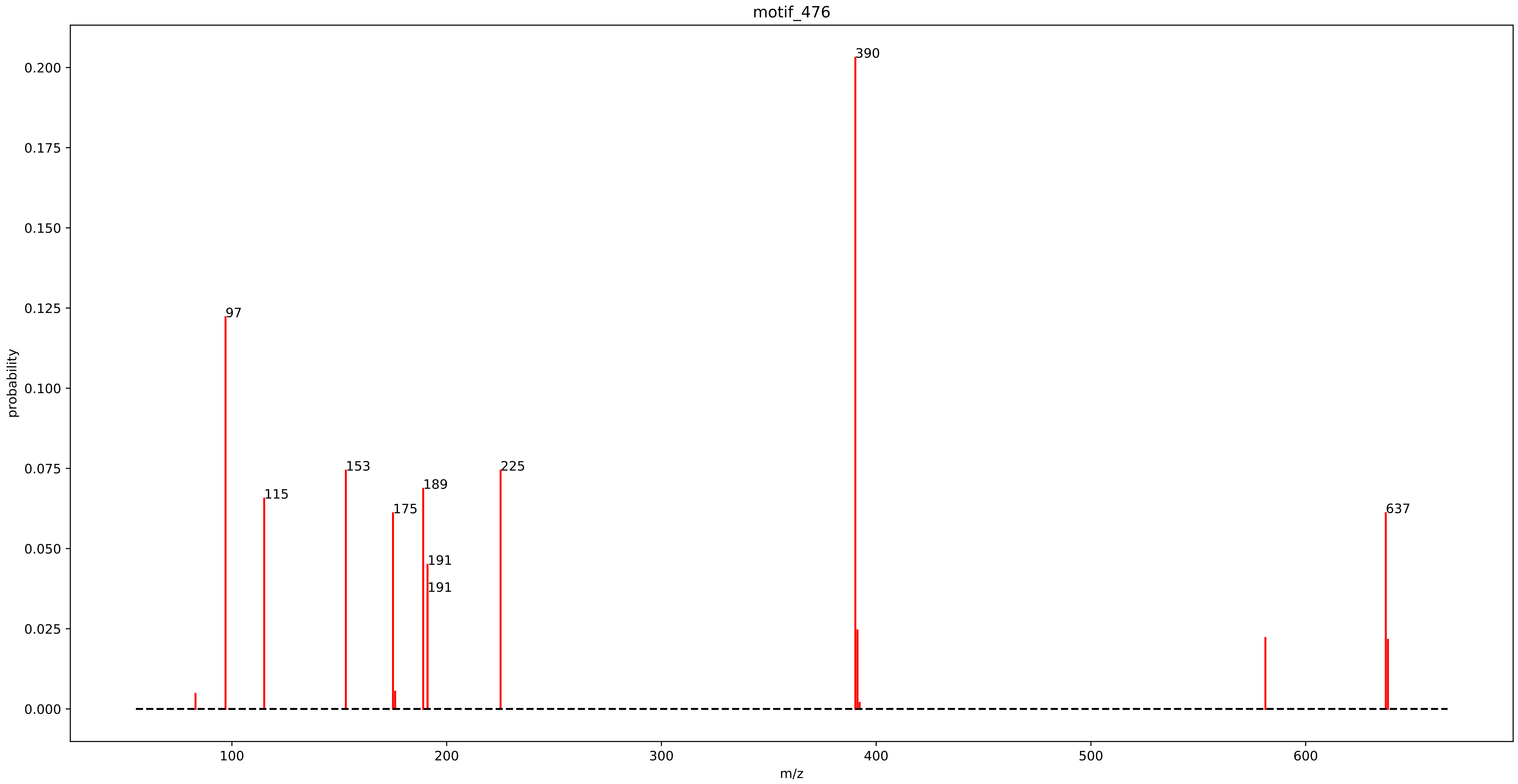

motif_476
probability
m/z
390
97
115
153
175
189
191
191
225
637
0.200
0.175
0.150
0.125
0.100
0.075
0.050
0.025
0.000
100
200
300
400
500
600

motif_476

fragment_390.2975	: 0.20
fragment_97.0275	: 0.12
fragment_225.0975	: 0.07
fragment_153.0525	: 0.07
fragment_189.0725	: 0.07
fragment_115.0375	: 0.07
fragment_637.3025	: 0.06
fragment_175.0125	: 0.06
fragment_191.0925	: 0.04
fragment_191.0875	: 0.04
fragment_391.3025	: 0.02
fragment_581.2425	: 0.02
fragment_638.3075	: 0.02
fragment_176.0175	: 0.01

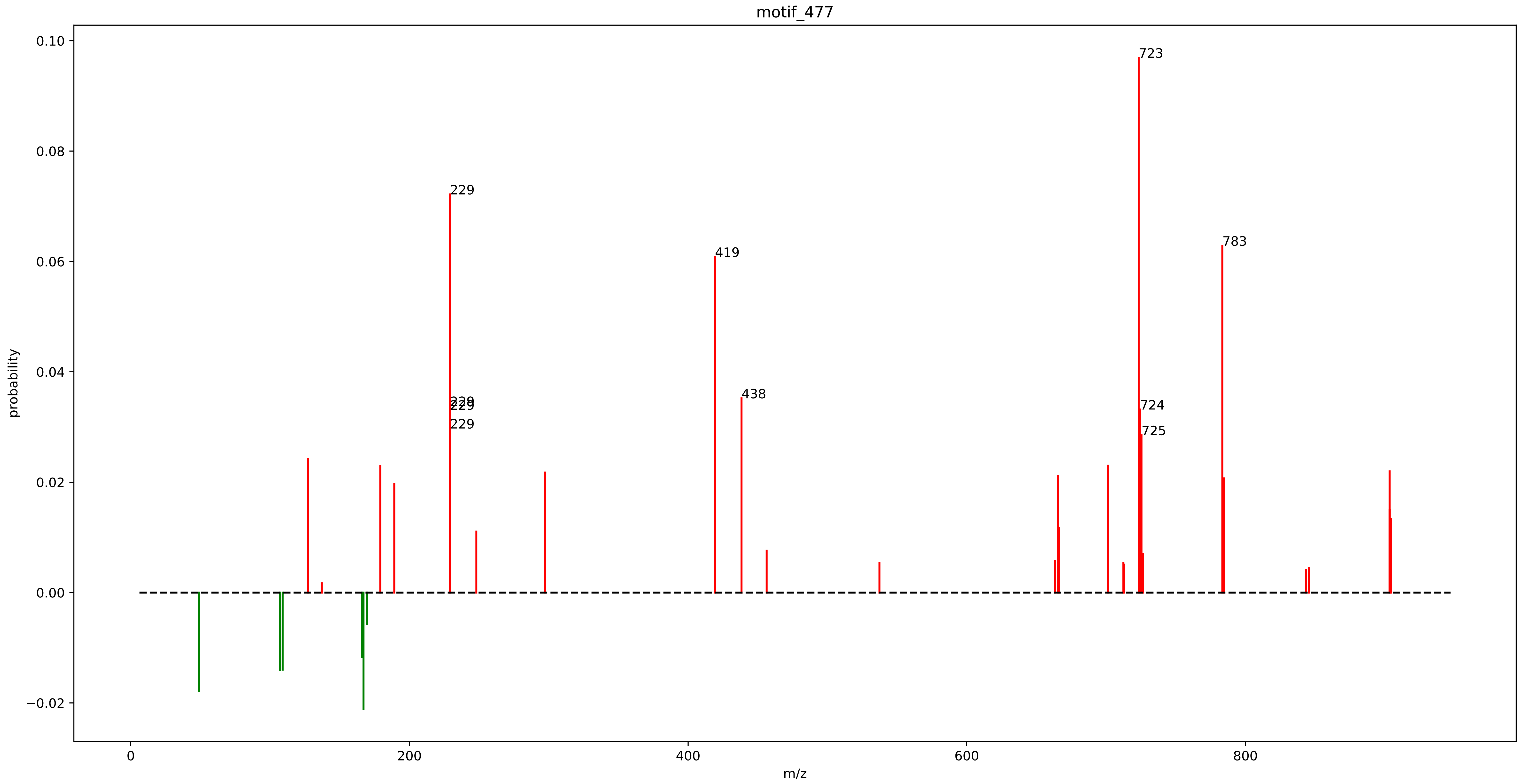

motif_477
probability
m/z
229
229
229
229
419
438
723
724
725
783
0.10
0.08
0.06
0.04
0.02
0.00
−0.02
0
200
400
600
800

motif_477

fragment_723.3975	: 0.10
fragment_229.1325	: 0.07
fragment_783.4175	: 0.06
fragment_419.3325	: 0.06
fragment_438.3475	: 0.04
fragment_229.1425	: 0.03
fragment_724.3975	: 0.03
fragment_229.1225	: 0.03
fragment_229.0925	: 0.03
fragment_725.4075	: 0.03
fragment_127.0375	: 0.02
fragment_701.4275	: 0.02
fragment_179.1075	: 0.02
fragment_229.1075	: 0.02
fragment_903.4575	: 0.02
fragment_297.2225	: 0.02
fragment_665.3925	: 0.02
loss_167.0575	: 0.02
fragment_784.4175	: 0.02
fragment_189.1625	: 0.02
loss_49.0325	: 0.02
fragment_903.4625	: 0.01
loss_107.0375	: 0.01
loss_109.0525	: 0.01
fragment_904.4625	: 0.01
fragment_666.3925	: 0.01
loss_166.0525	: 0.01
fragment_248.0825	: 0.01
fragment_456.3575	: 0.01
fragment_726.4125	: 0.01
fragment_663.3775	: 0.01
loss_169.5775	: 0.01
fragment_537.3425	: 0.01
fragment_712.4125	: 0.01
fragment_712.9125	: 0.01

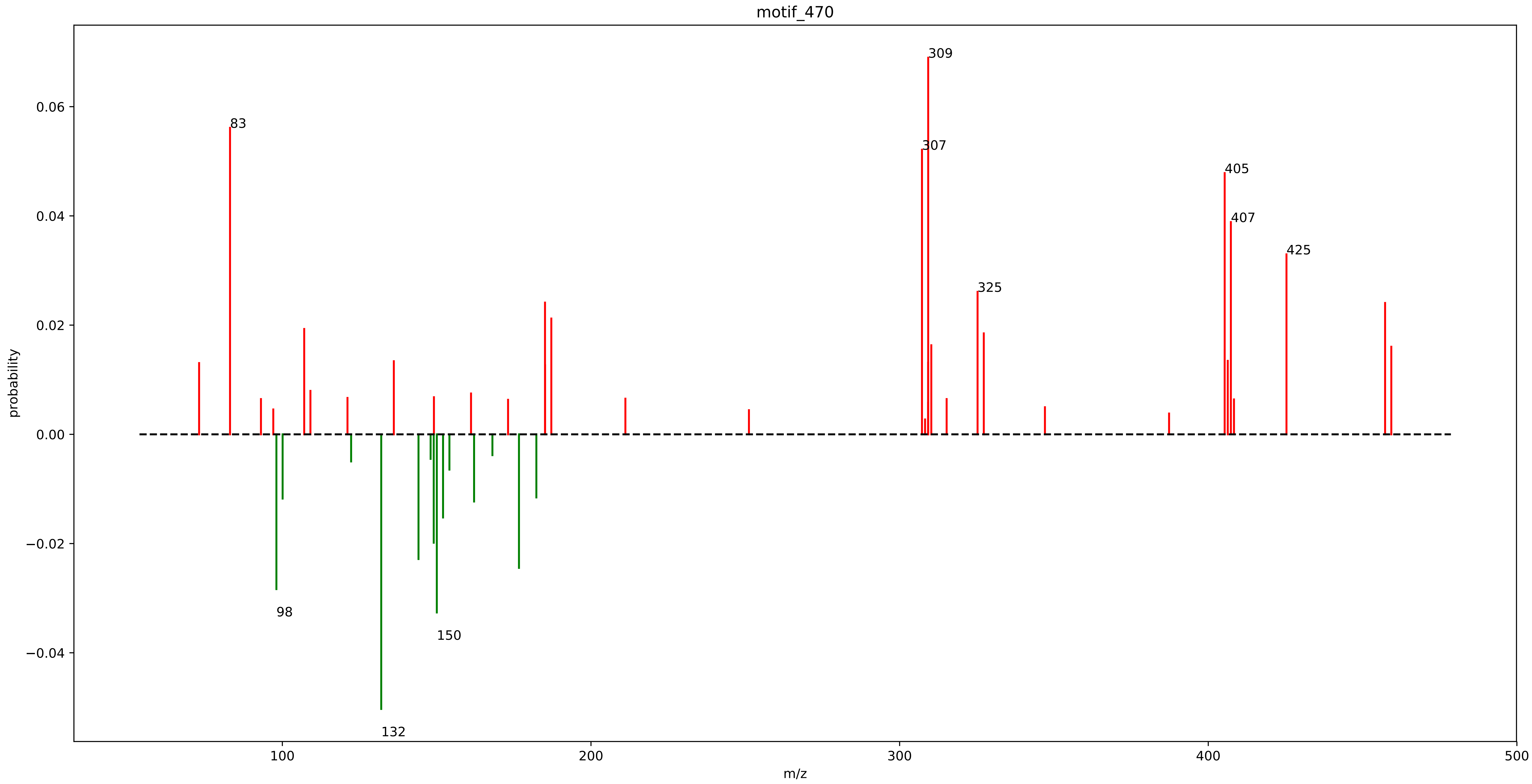

motif_470
0.06
0.04
0.02
0.00
−0.02
−0.04
probability
100
200
300
400
500
m/z
83
309
307
405
407
425
325
98
150
132

motif_470

fragment_309.2575	: 0.07
fragment_83.0475	: 0.06
fragment_307.2425	: 0.05
loss_132.0375	: 0.05
fragment_405.3175	: 0.05
fragment_407.3275	: 0.04
fragment_425.3425	: 0.03
loss_150.0475	: 0.03
loss_98.0725	: 0.03
fragment_325.2525	: 0.03
loss_176.6675	: 0.02
fragment_185.1025	: 0.02
fragment_457.2925	: 0.02
loss_144.1125	: 0.02
fragment_187.1475	: 0.02
loss_149.0475	: 0.02
fragment_107.0825	: 0.02
fragment_327.2675	: 0.02
fragment_310.2625	: 0.02
fragment_459.3075	: 0.02
fragment_405.3125	: 0.02
loss_152.0675	: 0.02
fragment_406.3175	: 0.01
fragment_136.1175	: 0.01
fragment_309.2375	: 0.01
fragment_73.0275	: 0.01
fragment_185.1325	: 0.01
loss_162.1225	: 0.01
loss_100.0875	: 0.01
loss_182.3025	: 0.01
fragment_109.0975	: 0.01
fragment_161.1325	: 0.01
fragment_149.1125	: 0.01
fragment_121.1025	: 0.01
fragment_211.1475	: 0.01
fragment_93.0675	: 0.01
fragment_315.2325	: 0.01
loss_154.1325	: 0.01
fragment_408.3325	: 0.01
fragment_173.1325	: 0.01
fragment_309.2325	: 0.01

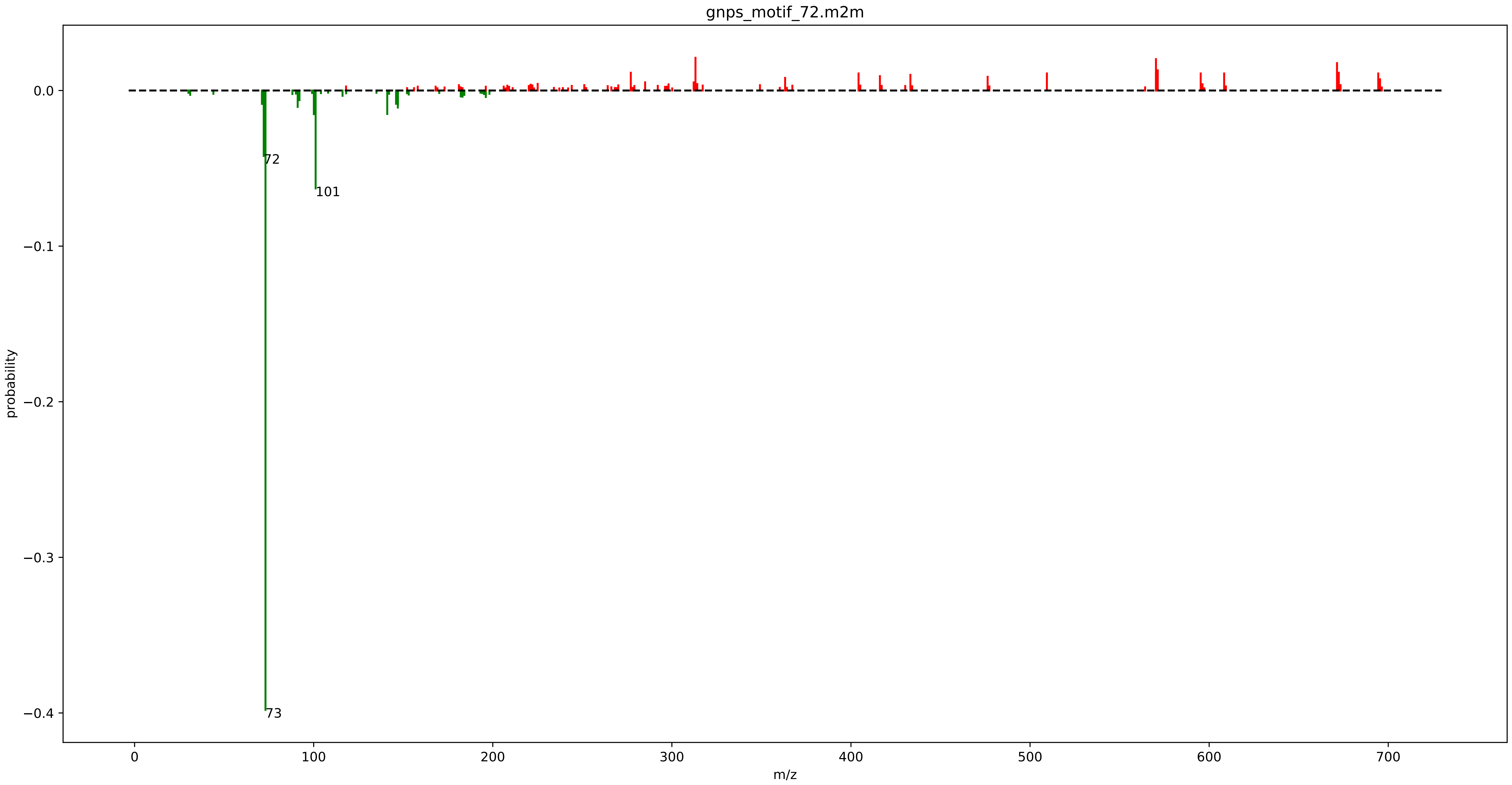

gnps_motif_72.m2m
probability
m/z
0.0
−0.1
−0.2
−0.3
−0.4
0
100
200
300
400
500
600
700
72
101
73

gnps_motif_72.m2m

loss_73.0875	: 0.40
loss_101.0825	: 0.06
loss_72.0875	: 0.04
fragment_313.1775	: 0.02
fragment_570.3075	: 0.02
fragment_671.3925	: 0.02
loss_100.0825	: 0.02
loss_141.0775	: 0.02
fragment_571.3075	: 0.01
fragment_672.3925	: 0.01
fragment_277.0675	: 0.01
loss_146.9775	: 0.01
fragment_509.4025	: 0.01
fragment_694.4175	: 0.01
fragment_570.3025	: 0.01
fragment_595.2775	: 0.01
fragment_404.2325	: 0.01
fragment_608.3675	: 0.01
loss_91.0575	: 0.01
fragment_433.1875	: 0.01
fragment_416.1575	: 0.01
fragment_476.3125	: 0.01
loss_147.0675	: 0.01
loss_71.0925	: 0.01
loss_145.9825	: 0.01
fragment_363.2275	: 0.01
fragment_695.4225	: 0.01
loss_92.0625	: 0.01
fragment_285.0625	: 0.01
fragment_312.1675	: 0.01

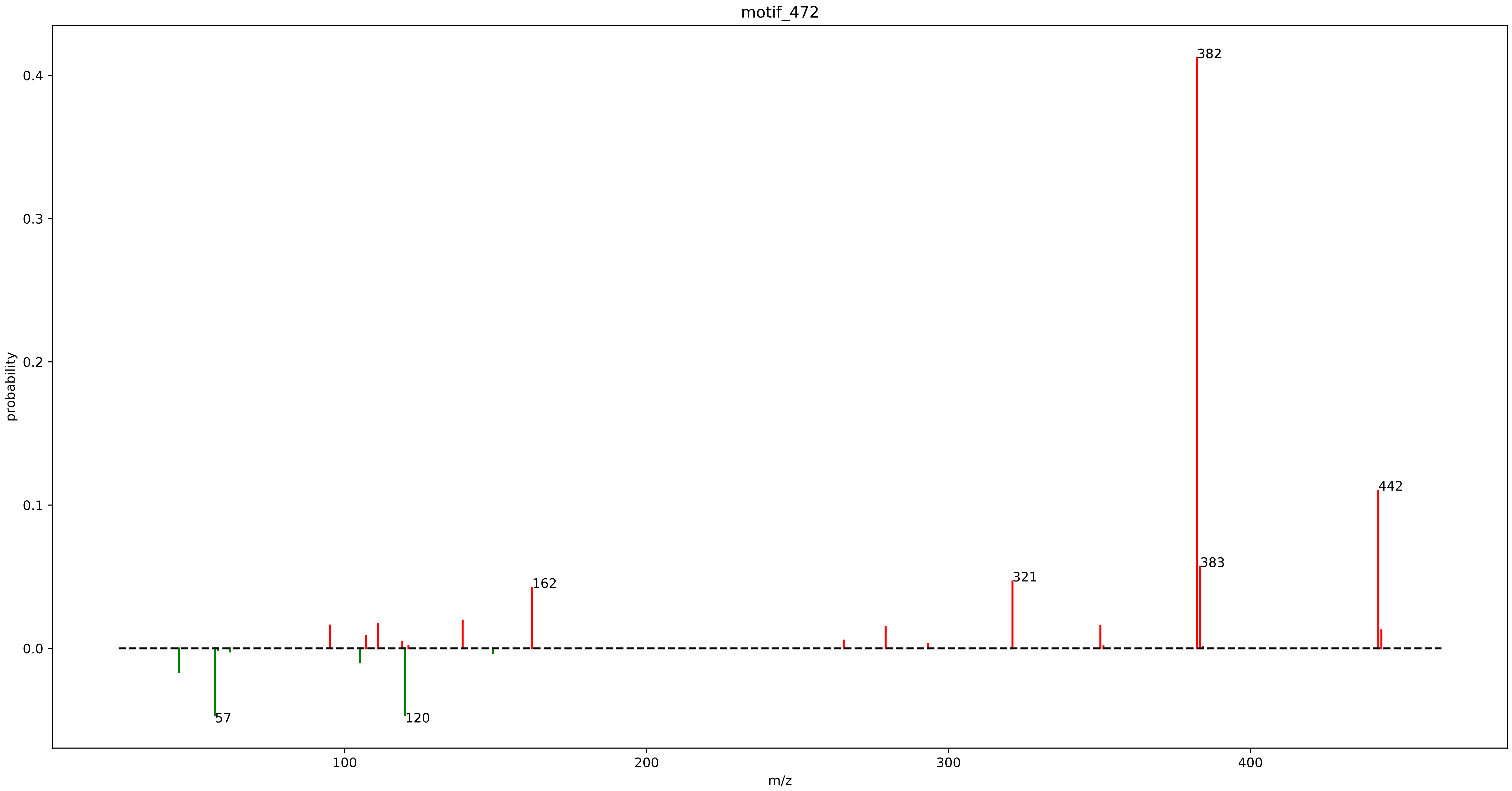

motif_472
probability
m/z
0.0
0.1
0.2
0.3
0.4
100
200
300
400
382
442
383
321
162
57
120

motif_472

fragment_382.3325	: 0.41
fragment_442.3525	: 0.11
fragment_383.3325	: 0.06
loss_57.0225	: 0.05
fragment_321.1775	: 0.05
loss_120.0425	: 0.05
fragment_162.0875	: 0.04
fragment_139.0725	: 0.02
fragment_111.0825	: 0.02
loss_45.0525	: 0.02
fragment_95.0825	: 0.02
fragment_350.3025	: 0.02
fragment_279.1675	: 0.02
fragment_443.3575	: 0.01
loss_105.0775	: 0.01
fragment_107.0825	: 0.01
fragment_265.2525	: 0.01

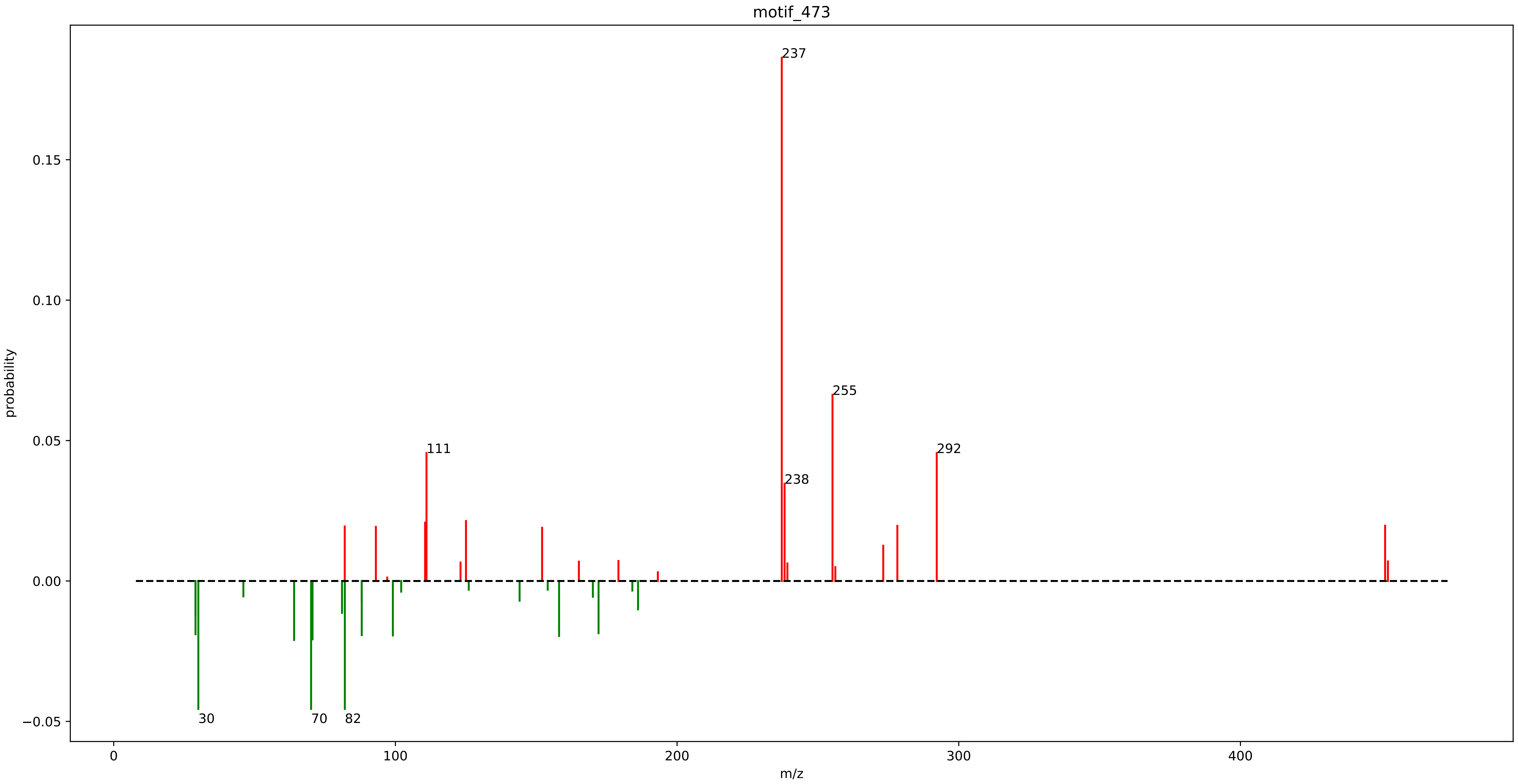

motif_473
237
255
111
292
238
30
70
82
probability
m/z
0.15
0.10
0.05
0.00
−0.05
0
100
200
300
400

motif_473

fragment_237.1675	: 0.19
fragment_255.1775	: 0.07
fragment_111.0425	: 0.05
loss_70.0775	: 0.05
loss_30.0425	: 0.05
loss_82.0525	: 0.05
fragment_292.1825	: 0.05
fragment_238.1725	: 0.03
fragment_125.0575	: 0.02
loss_64.0425	: 0.02
loss_70.5775	: 0.02
fragment_110.5425	: 0.02
fragment_451.3625	: 0.02
fragment_278.1975	: 0.02
loss_158.1075	: 0.02
fragment_82.0125	: 0.02
loss_99.1075	: 0.02
loss_88.0525	: 0.02
fragment_93.0725	: 0.02
fragment_152.0825	: 0.02
loss_29.0425	: 0.02
loss_172.1225	: 0.02
fragment_273.1925	: 0.01
loss_29.0375	: 0.01
loss_81.0525	: 0.01
loss_186.1425	: 0.01
loss_88.0325	: 0.01
fragment_179.1775	: 0.01
loss_144.0925	: 0.01
fragment_452.3675	: 0.01
fragment_165.1475	: 0.01
fragment_123.1175	: 0.01
fragment_239.1675	: 0.01
loss_170.1075	: 0.01
loss_46.0325	: 0.01

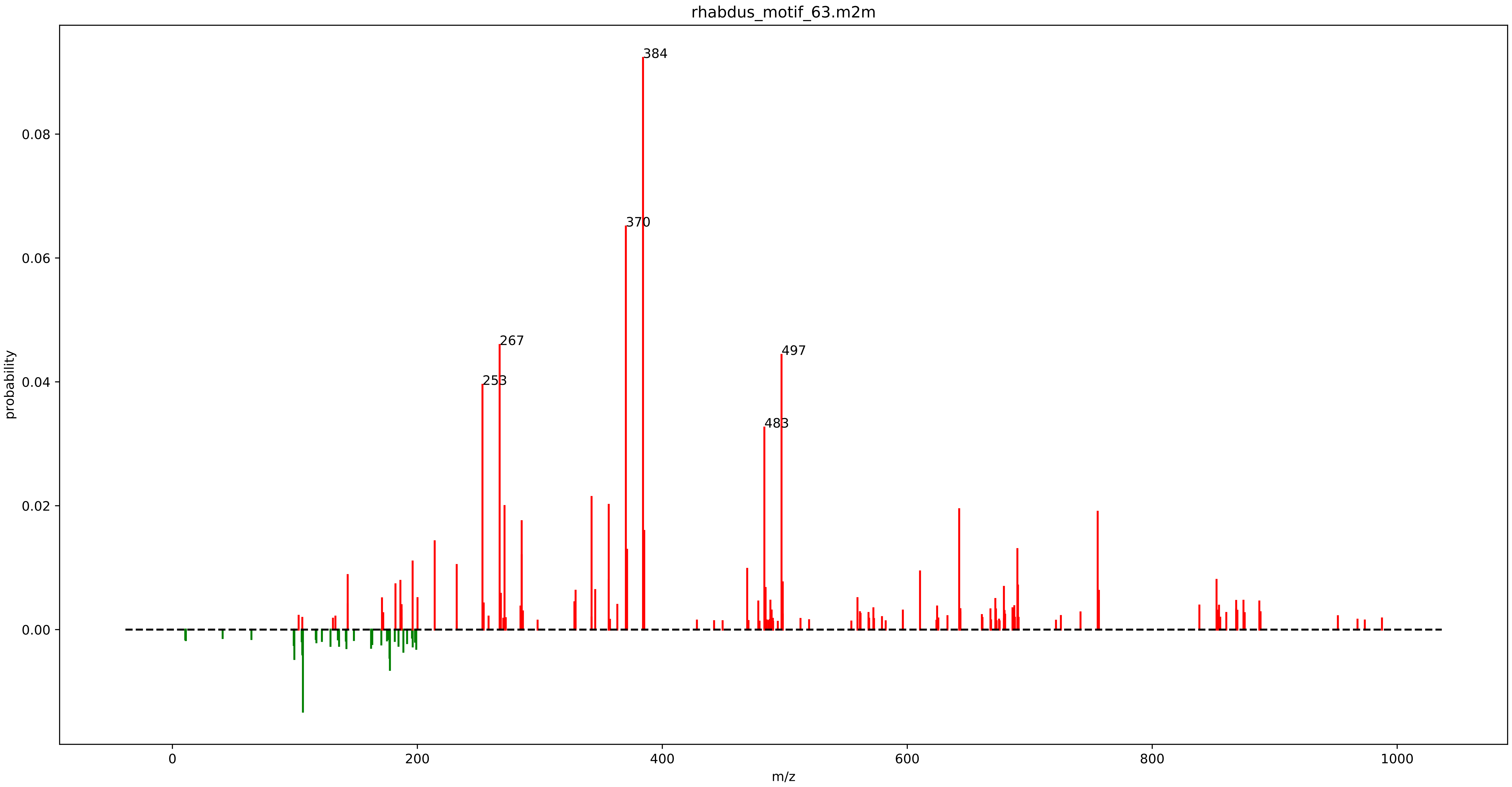

rhabdus_motif_63.m2m
probability
m/z
0.08
0.06
0.04
0.02
0.00
0
200
400
600
800
1000
384
370
267
253
497
483

rhabdus_motif_63.m2m

fragment_384.2475	: 0.09
fragment_370.2325	: 0.07
fragment_267.1725	: 0.05
fragment_497.3325	: 0.04
fragment_253.1525	: 0.04
fragment_483.3175	: 0.03
fragment_342.2375	: 0.02
fragment_356.2525	: 0.02
fragment_271.1675	: 0.02
fragment_642.3825	: 0.02
fragment_755.4675	: 0.02
fragment_285.1775	: 0.02
fragment_385.2525	: 0.02
fragment_214.1425	: 0.01
loss_106.5675	: 0.01
fragment_689.9025	: 0.01
fragment_371.2375	: 0.01
fragment_285.1825	: 0.01
fragment_196.1325	: 0.01
fragment_232.1275	: 0.01
fragment_469.3025	: 0.01
fragment_610.4175	: 0.01
fragment_143.1125	: 0.01
fragment_852.5175	: 0.01
fragment_356.2175	: 0.01
fragment_186.1475	: 0.01
fragment_498.3375	: 0.01
fragment_182.1175	: 0.01
fragment_690.4025	: 0.01
fragment_678.9075	: 0.01
fragment_484.3225	: 0.01
loss_177.5925	: 0.01
fragment_345.2125	: 0.01
fragment_329.1825	: 0.01
fragment_756.4675	: 0.01
fragment_268.1725	: 0.01
fragment_200.1275	: 0.01
fragment_559.3425	: 0.01
fragment_171.1125	: 0.01

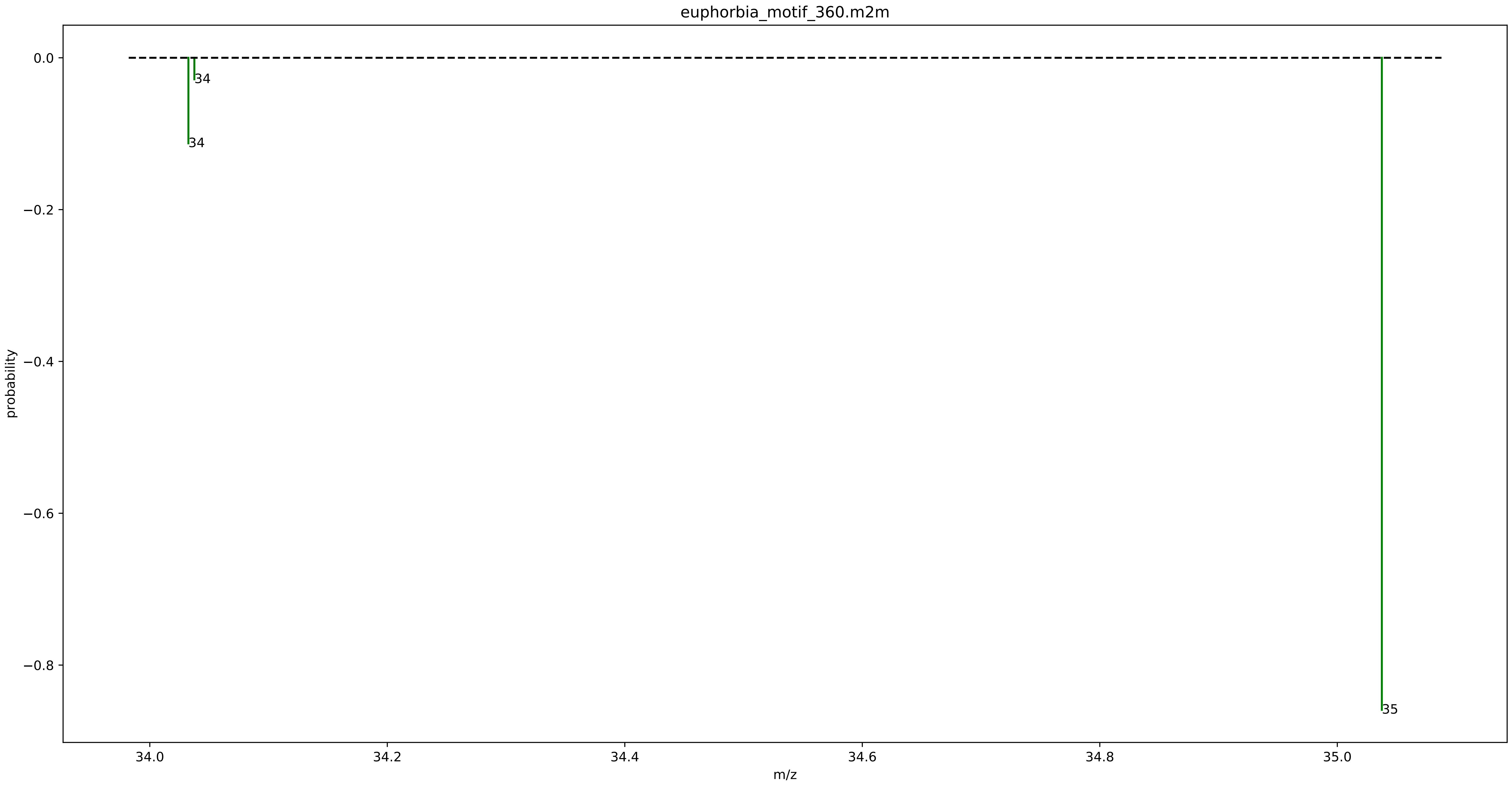

euphorbia_motif_360.m2m
0.0
−0.2
−0.4
−0.6
−0.8
probability
34
34
35
34.0
34.2
34.4
34.6
34.8
35.0
m/z

euphorbia_motif_360.m2m

loss_35.0375	: 0.86
loss_34.0325	: 0.11
loss_34.0375	: 0.03

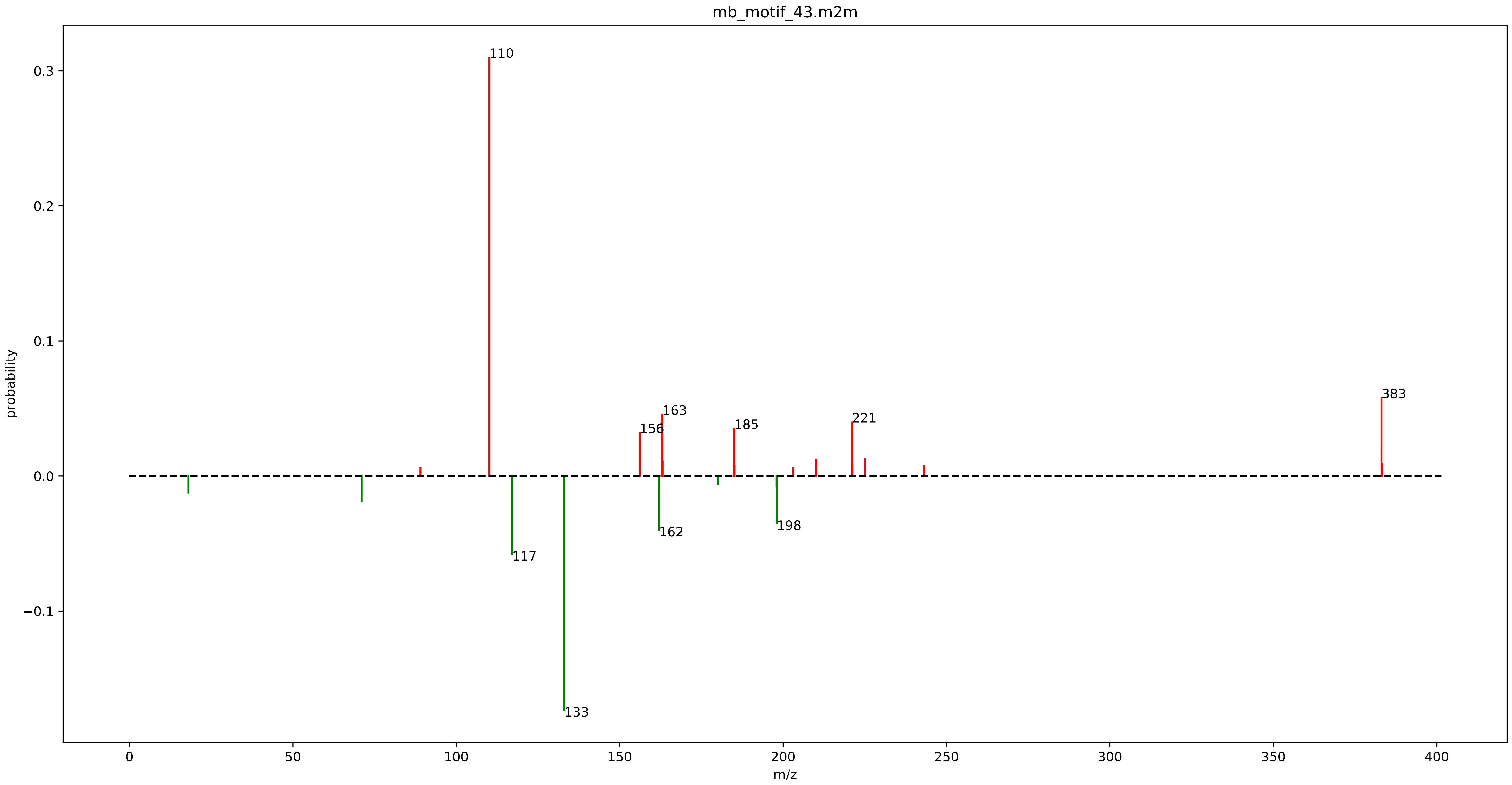

mb_motif_43.m2m
probability
m/z
0.3
0.2
0.1
0.0
−0.1
0
50
100
150
200
250
300
350
400
110
156
163
185
221
383
117
133
162
198

mb_motif_43.m2m

fragment_110.0725	: 0.31
loss_133.0375	: 0.17
loss_117.0425	: 0.06
fragment_383.0825	: 0.06
fragment_163.0375	: 0.05
fragment_221.0475	: 0.04
loss_162.0325	: 0.04
loss_198.0425	: 0.03
fragment_185.0275	: 0.03
fragment_156.0775	: 0.03
fragment_383.0775	: 0.02
loss_71.0375	: 0.02
fragment_225.0975	: 0.01
loss_18.0125	: 0.01
fragment_210.0925	: 0.01
fragment_163.0975	: 0.01
fragment_383.1875	: 0.01
fragment_221.1325	: 0.01
loss_161.9475	: 0.01
fragment_221.0425	: 0.01
fragment_185.0975	: 0.01
loss_197.9775	: 0.01
fragment_243.1075	: 0.01
fragment_185.0225	: 0.01
loss_198.0525	: 0.01
fragment_203.0375	: 0.01
loss_180.0425	: 0.01
fragment_89.0375	: 0.01

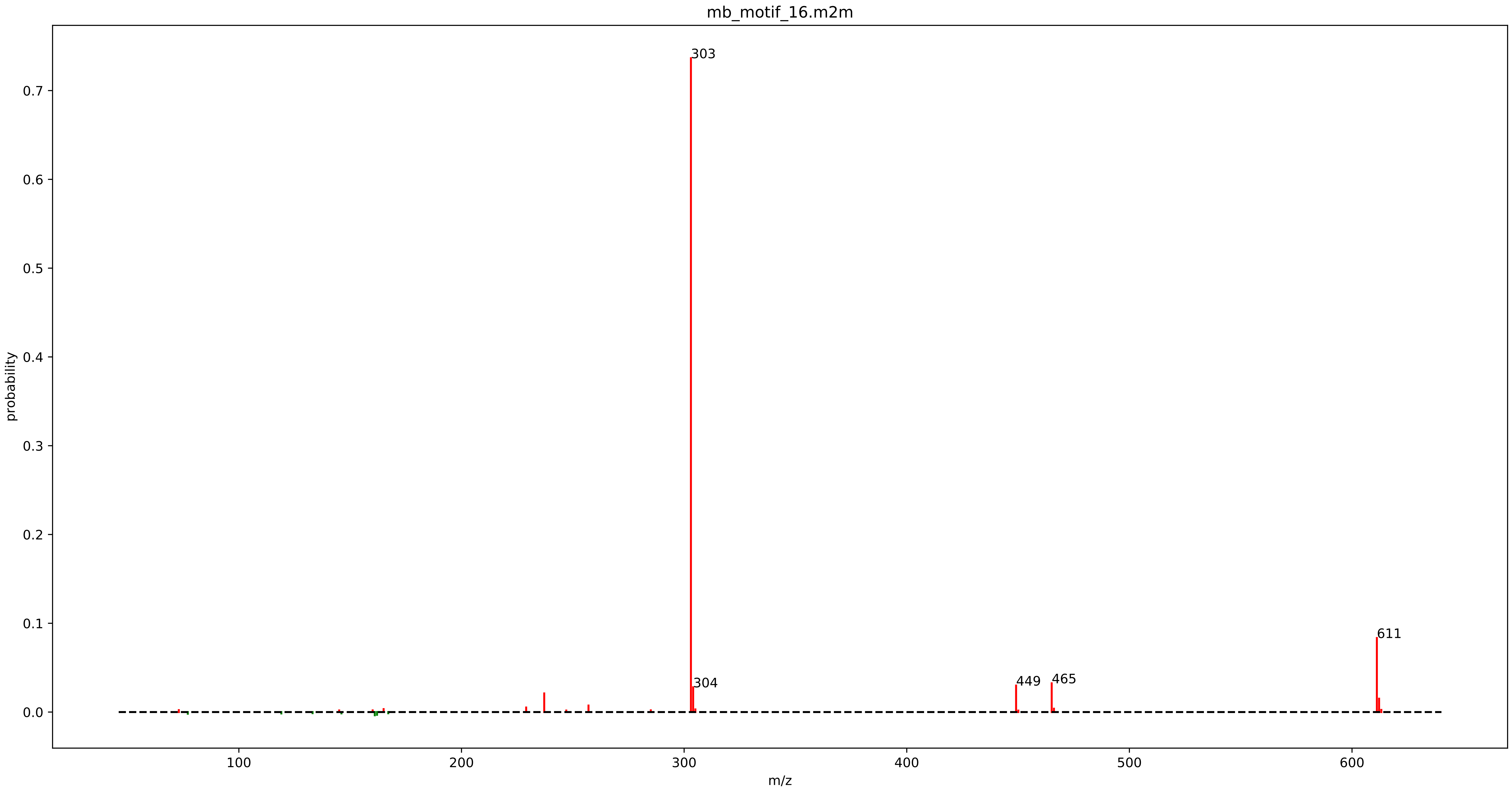

mb_motif_16.m2m
probability
m/z
303
304
449
465
611
0.0
0.1
0.2
0.3
0.4
0.5
0.6
0.7
100
200
300
400
500
600

mb_motif_16.m2m

fragment_303.0525	: 0.74
fragment_611.1625	: 0.08
fragment_465.1025	: 0.03
fragment_449.1075	: 0.03
fragment_304.0525	: 0.03
fragment_237.1575	: 0.02
fragment_612.1625	: 0.02
fragment_257.0425	: 0.01
fragment_229.0475	: 0.01

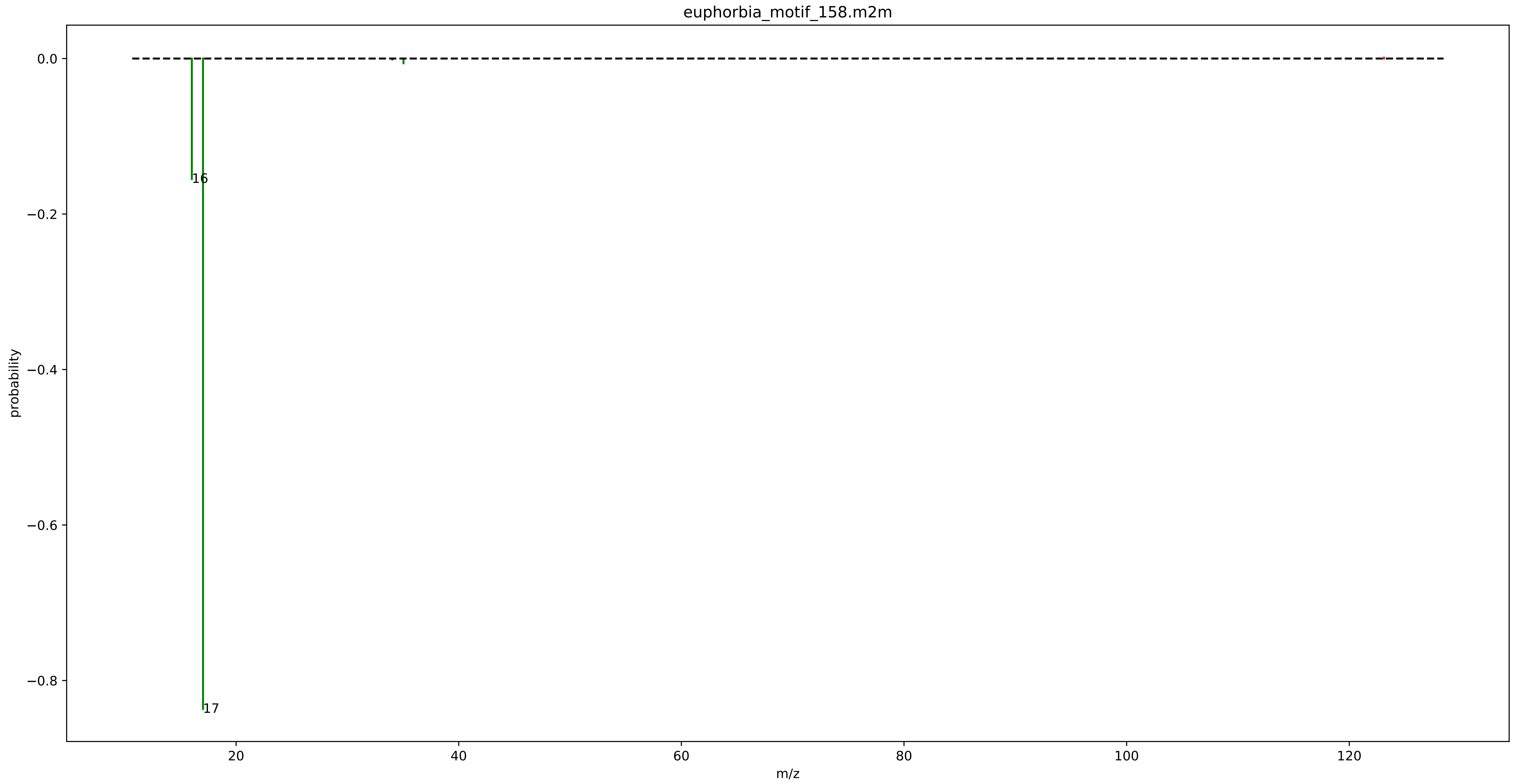

euphorbia_motif_158.m2m
0.0
−0.2
−0.4
−0.6
−0.8
probability
16
17
20
40
60
80
100
120
m/z

euphorbia_motif_158.m2m

loss_17.0275	: 0.84
loss_16.0225	: 0.15
loss_35.0375	: 0.01

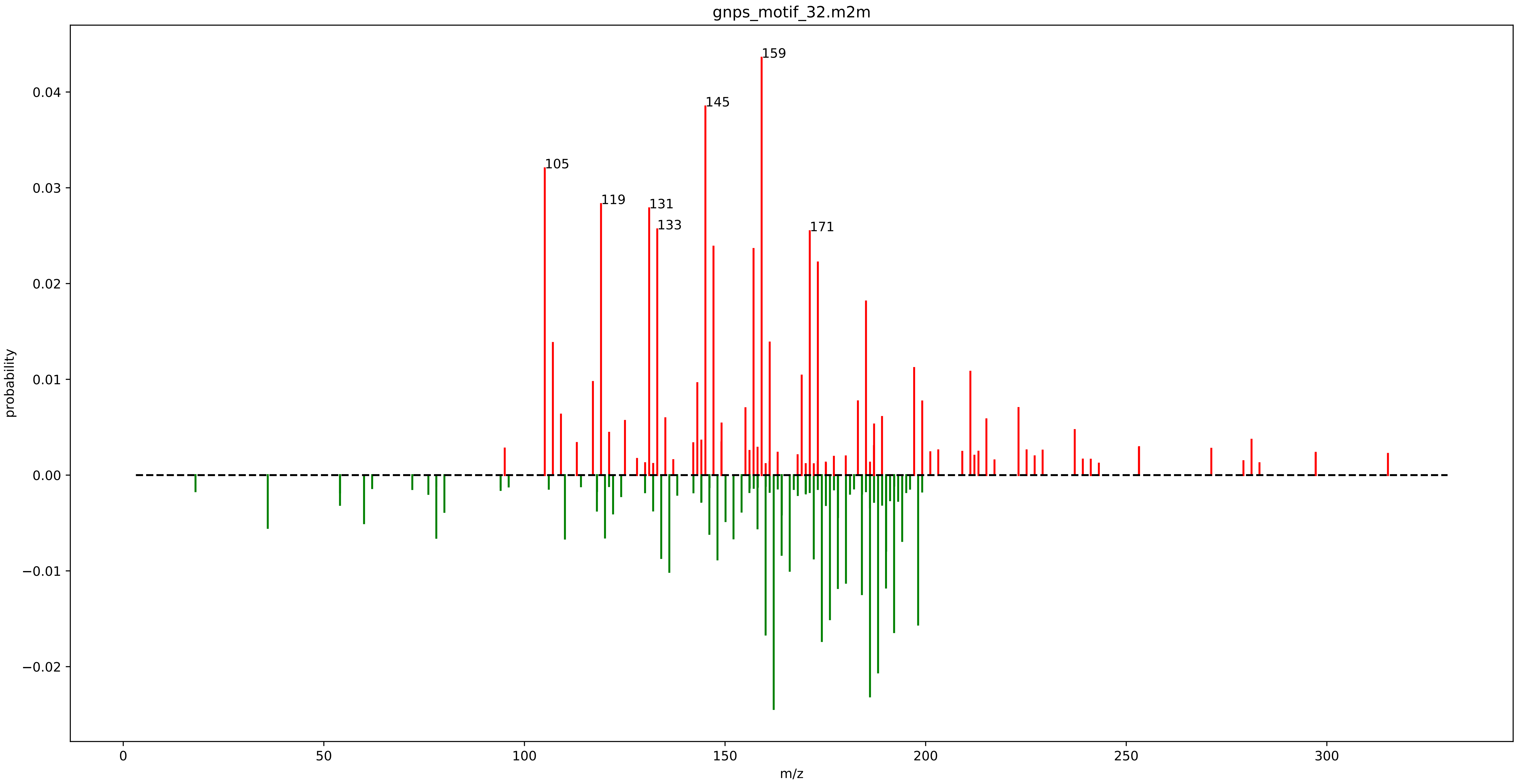
gnps_motif_32.m2m
probability
m/z
0.04
0.03
0.02
0.01
0.00
−0.01
−0.02
0
50
100
150
200
250
300
105
119
131
133
145
159
171

gnps_motif_32.m2m

fragment_159.1175	: 0.04
fragment_145.1025	: 0.04
fragment_105.0675	: 0.03
fragment_119.0875	: 0.03
fragment_131.0875	: 0.03
fragment_133.1025	: 0.03
fragment_171.1175	: 0.03
loss_162.1275	: 0.02
fragment_147.1175	: 0.02
fragment_157.1025	: 0.02
loss_186.1275	: 0.02
fragment_173.1325	: 0.02
loss_188.1425	: 0.02
fragment_185.1325	: 0.02
loss_174.1275	: 0.02
loss_160.1125	: 0.02
loss_192.1375	: 0.02
loss_198.1275	: 0.02
loss_176.1425	: 0.02
fragment_161.1325	: 0.01
fragment_107.0875	: 0.01
loss_184.1125	: 0.01
loss_178.1225	: 0.01
loss_190.1225	: 0.01
loss_180.1375	: 0.01
fragment_197.1325	: 0.01
fragment_211.1475	: 0.01
fragment_169.1025	: 0.01
loss_136.1125	: 0.01
loss_166.1225	: 0.01
fragment_117.0675	: 0.01
fragment_143.0875	: 0.01
loss_148.1125	: 0.01
loss_172.1125	: 0.01
loss_134.0925	: 0.01
loss_164.1075	: 0.01
loss_190.1575	: 0.01
fragment_183.1175	: 0.01
fragment_199.1475	: 0.01
fragment_223.1475	: 0.01
fragment_155.0875	: 0.01
loss_194.1525	: 0.01
loss_110.0925	: 0.01
loss_152.1075	: 0.01
loss_78.0325	: 0.01
loss_120.0775	: 0.01
fragment_109.1025	: 0.01
loss_146.0925	: 0.01
fragment_189.1275	: 0.01
fragment_135.1175	: 0.01
fragment_215.1425	: 0.01
fragment_125.0575	: 0.01
loss_158.0925	: 0.01
loss_36.0225	: 0.01
fragment_149.1325	: 0.01
fragment_187.1475	: 0.01
loss_60.0225	: 0.01

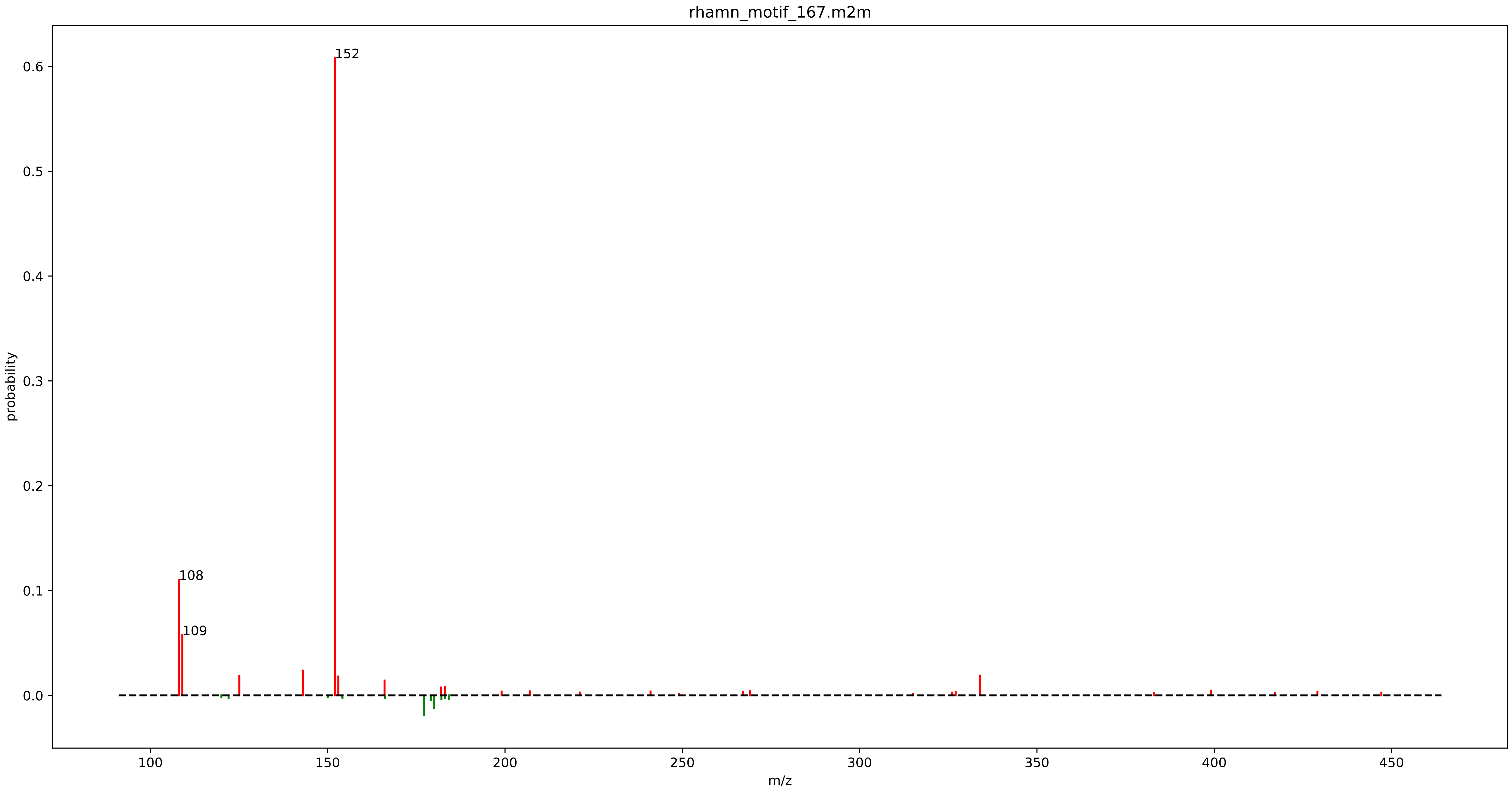

rhamn_motif_167.m2m
probability
m/z
152
108
109
0.6
0.5
0.4
0.3
0.2
0.1
0.0
100
150
200
250
300
350
400
450

rhamn_motif_167.m2m

fragment_152.0125	: 0.61
fragment_108.0225	: 0.11
fragment_109.0275	: 0.06
fragment_143.0375	: 0.02
fragment_334.0075	: 0.02
loss_177.2275	: 0.02
fragment_125.0975	: 0.02
fragment_153.0125	: 0.02
fragment_166.0275	: 0.01
loss_180.0575	: 0.01
fragment_183.0275	: 0.01
fragment_182.0175	: 0.01

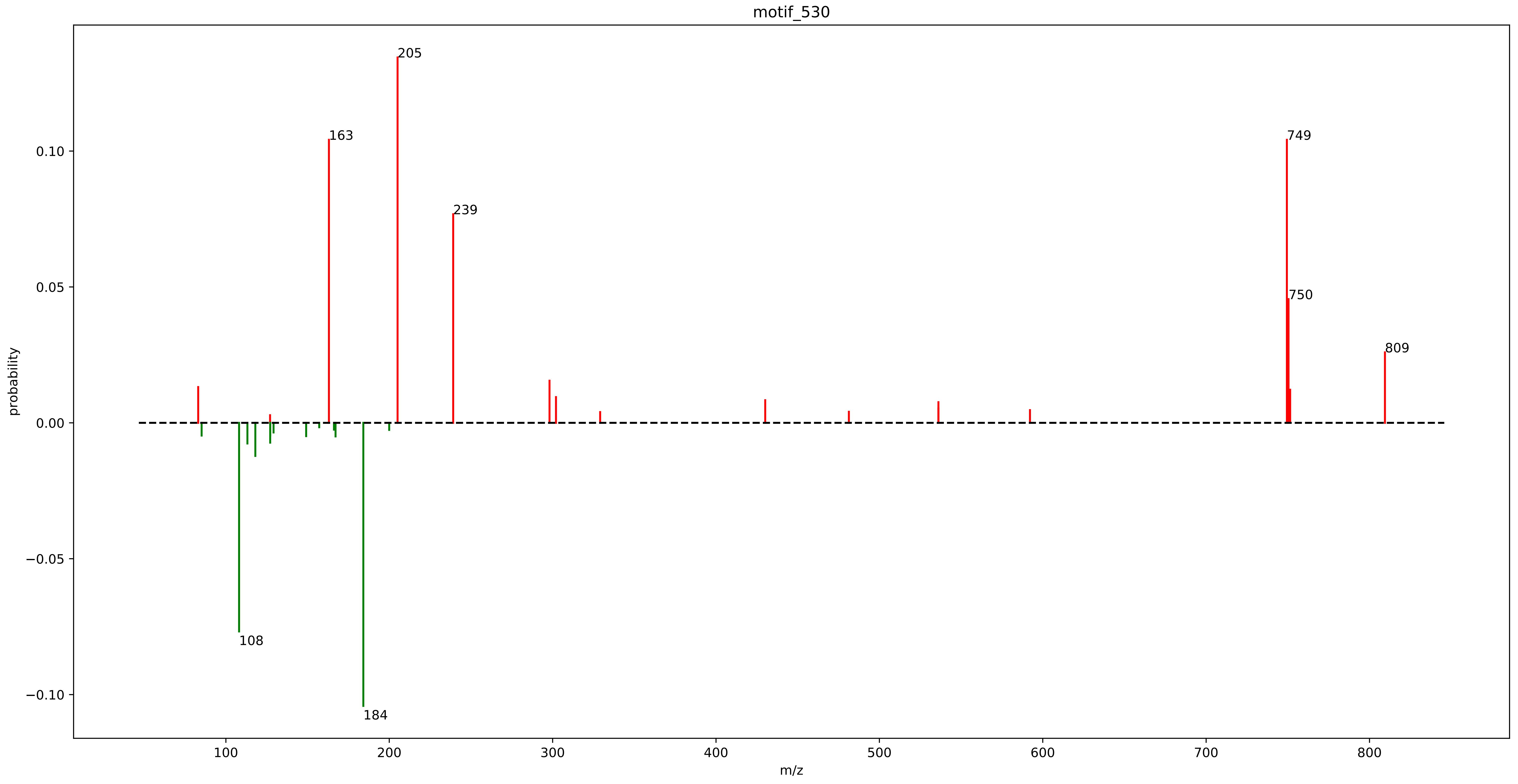

motif_530
205
163
749
239
750
809
108
184
0.10
0.05
0.00
−0.05
−0.10
probability
100
200
300
400
500
600
700
800
m/z

motif_530

fragment_205.0825	: 0.13
fragment_163.0725	: 0.10
loss_184.1275	: 0.10
fragment_749.4225	: 0.10
fragment_239.1225	: 0.08
loss_108.0775	: 0.08
fragment_750.4275	: 0.05
fragment_809.4475	: 0.03
fragment_298.0875	: 0.02
fragment_83.0475	: 0.01
fragment_751.4325	: 0.01
loss_118.0275	: 0.01
fragment_302.0525	: 0.01
fragment_430.1175	: 0.01
loss_113.1725	: 0.01
fragment_536.1325	: 0.01
loss_127.1175	: 0.01
fragment_536.1275	: 0.01

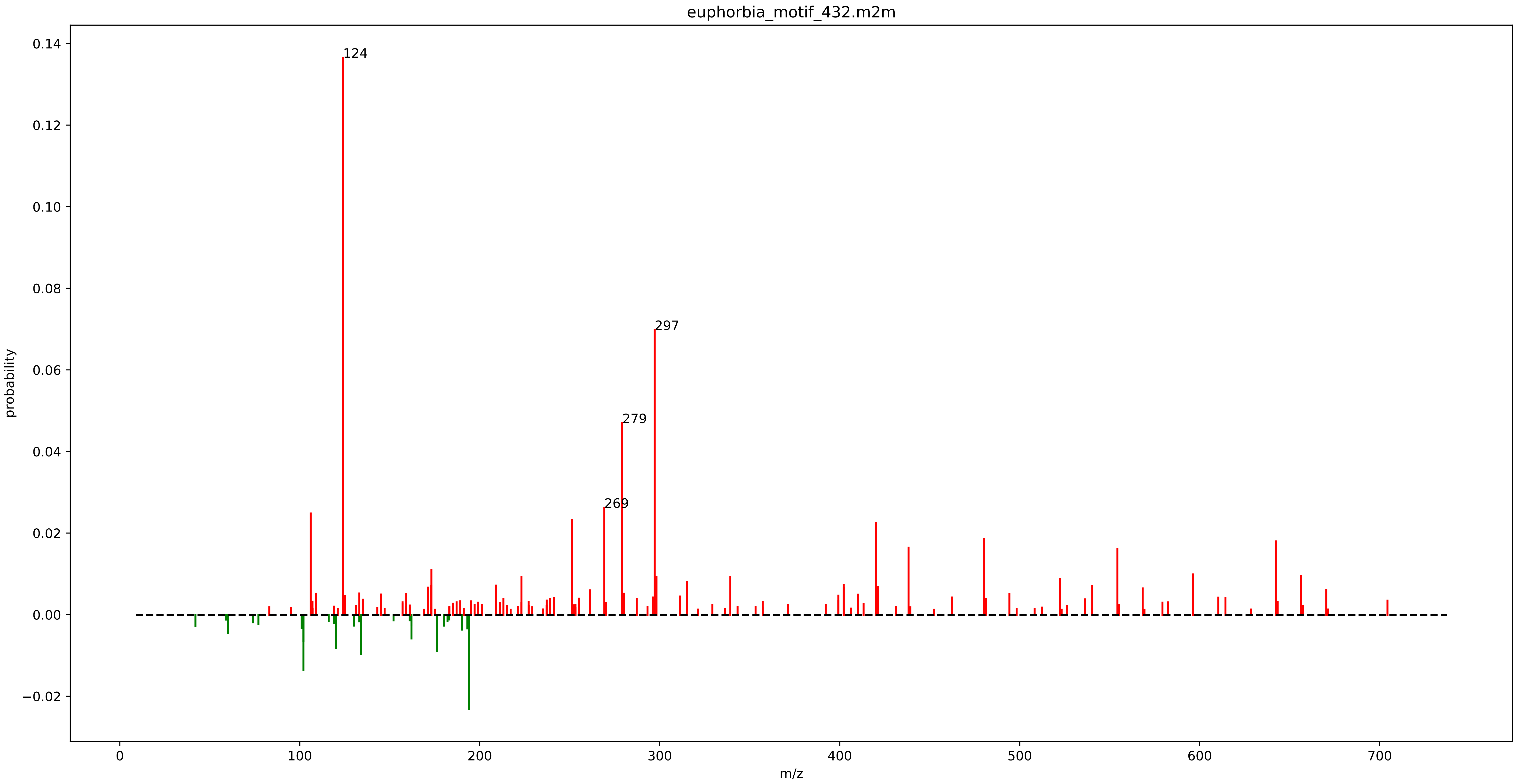

euphorbia_motif_432.m2m
probability
m/z
124
297
279
269
0.14
0.12
0.10
0.08
0.06
0.04
0.02
0.00
−0.02
0
100
200
300
400
500
600
700

euphorbia_motif_432.m2m

fragment_124.0375	: 0.14
fragment_297.1825	: 0.07
fragment_279.1725	: 0.05
fragment_269.1875	: 0.03
fragment_106.0275	: 0.02
fragment_251.1775	: 0.02
loss_194.0825	: 0.02
fragment_420.2125	: 0.02
fragment_420.2175	: 0.02
fragment_480.2375	: 0.02
fragment_642.2875	: 0.02
fragment_438.2275	: 0.02
fragment_554.2725	: 0.02
loss_102.0375	: 0.01
fragment_173.1325	: 0.01
fragment_596.2825	: 0.01
loss_134.0625	: 0.01
fragment_656.3025	: 0.01
fragment_223.1475	: 0.01
fragment_298.1875	: 0.01
fragment_339.1925	: 0.01
loss_176.0725	: 0.01
fragment_522.2475	: 0.01
loss_120.0425	: 0.01
fragment_315.1925	: 0.01
fragment_402.2075	: 0.01
fragment_209.1325	: 0.01
fragment_173.0975	: 0.01
fragment_540.2575	: 0.01
fragment_421.2175	: 0.01
fragment_171.1175	: 0.01
fragment_402.2025	: 0.01
loss_102.0325	: 0.01
fragment_568.2875	: 0.01
fragment_670.3175	: 0.01
fragment_261.1625	: 0.01
loss_162.0575	: 0.01
fragment_133.1025	: 0.01
fragment_280.1775	: 0.01
fragment_109.1025	: 0.01
fragment_494.2525	: 0.01
fragment_159.1175	: 0.01

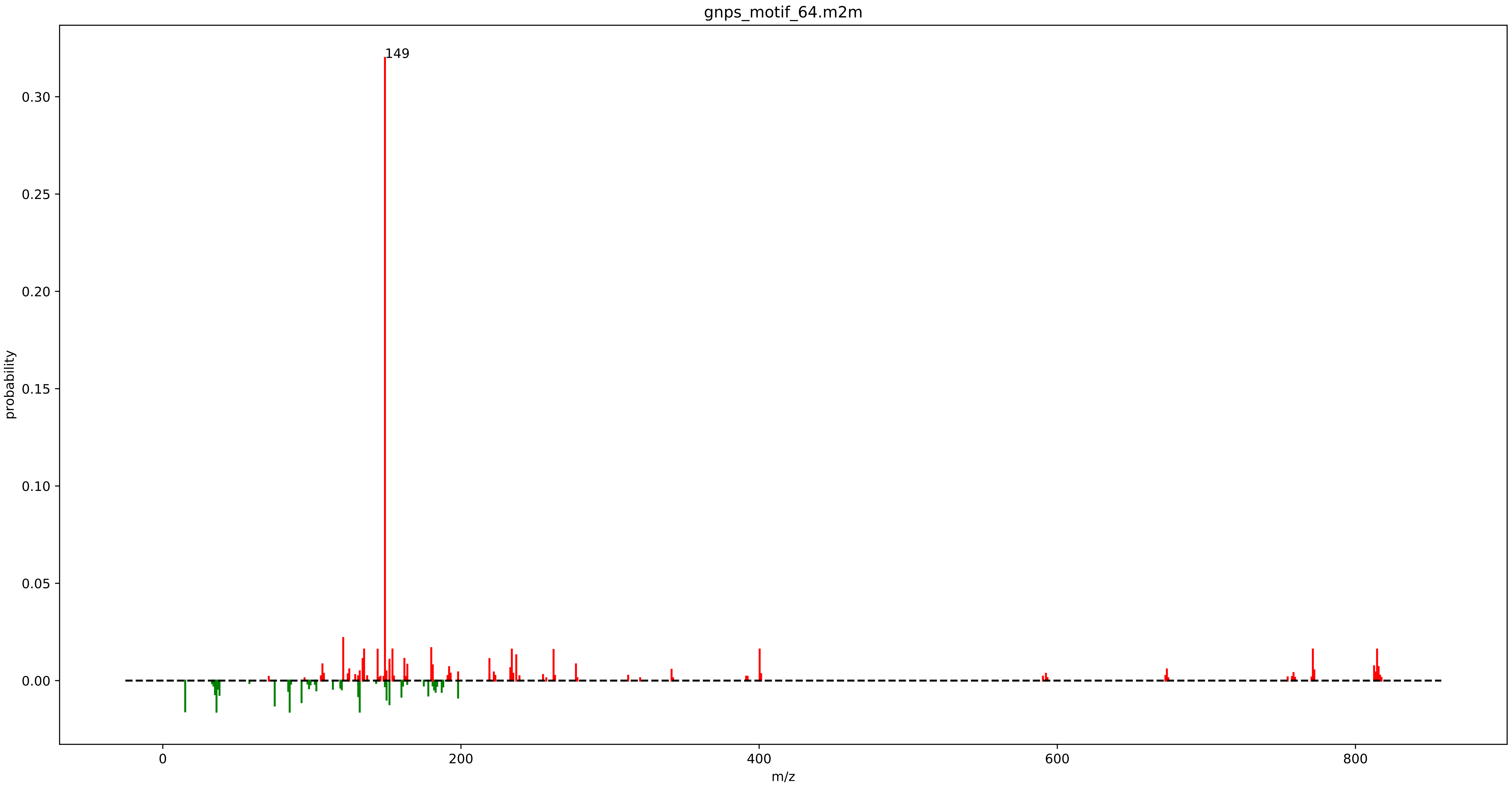

gnps_motif_64.m2m
149
probability
0.30
0.25
0.20
0.15
0.10
0.05
0.00
0
200
400
600
800
m/z

gnps_motif_64.m2m

fragment_149.0225 : 0.32
fragment_121.0275 : 0.02
fragment_180.0775 : 0.02
fragment_154.0475 : 0.02
fragment_814.5025 : 0.02
loss_85.1075 : 0.02
loss_132.0875 : 0.02
loss_36.0225 : 0.02
fragment_771.4325 : 0.02
fragment_400.3425 : 0.02
fragment_234.1175 : 0.02
fragment_135.0275 : 0.02
fragment_144.1025 : 0.02
loss_15.0225 : 0.02
fragment_262.1125 : 0.02
fragment_237.0925 : 0.01
loss_75.0675 : 0.01
loss_152.0475 : 0.01
fragment_162.0625 : 0.01
fragment_134.0425 : 0.01
fragment_219.0875 : 0.01
loss_93.0775 : 0.01
fragment_152.0525 : 0.01
loss_150.1025 : 0.01
loss_198.0775 : 0.01
fragment_277.1325 : 0.01
fragment_107.0625 : 0.01
fragment_152.0775 : 0.01
loss_160.0825 : 0.01
fragment_164.0475 : 0.01
loss_131.0925 : 0.01
fragment_181.0725 : 0.01
loss_178.0975 : 0.01
fragment_812.4925 : 0.01
loss_38.0375 : 0.01
loss_35.0175 : 0.01
fragment_815.5125 : 0.01
fragment_192.0425 : 0.01
fragment_134.0675 : 0.01
fragment_233.1075 : 0.01
fragment_125.0725 : 0.01
loss_187.0975 : 0.01
fragment_673.5175 : 0.01
loss_183.0225 : 0.01
fragment_341.2625 : 0.01
fragment_772.3975 : 0.01
loss_84.1425 : 0.01

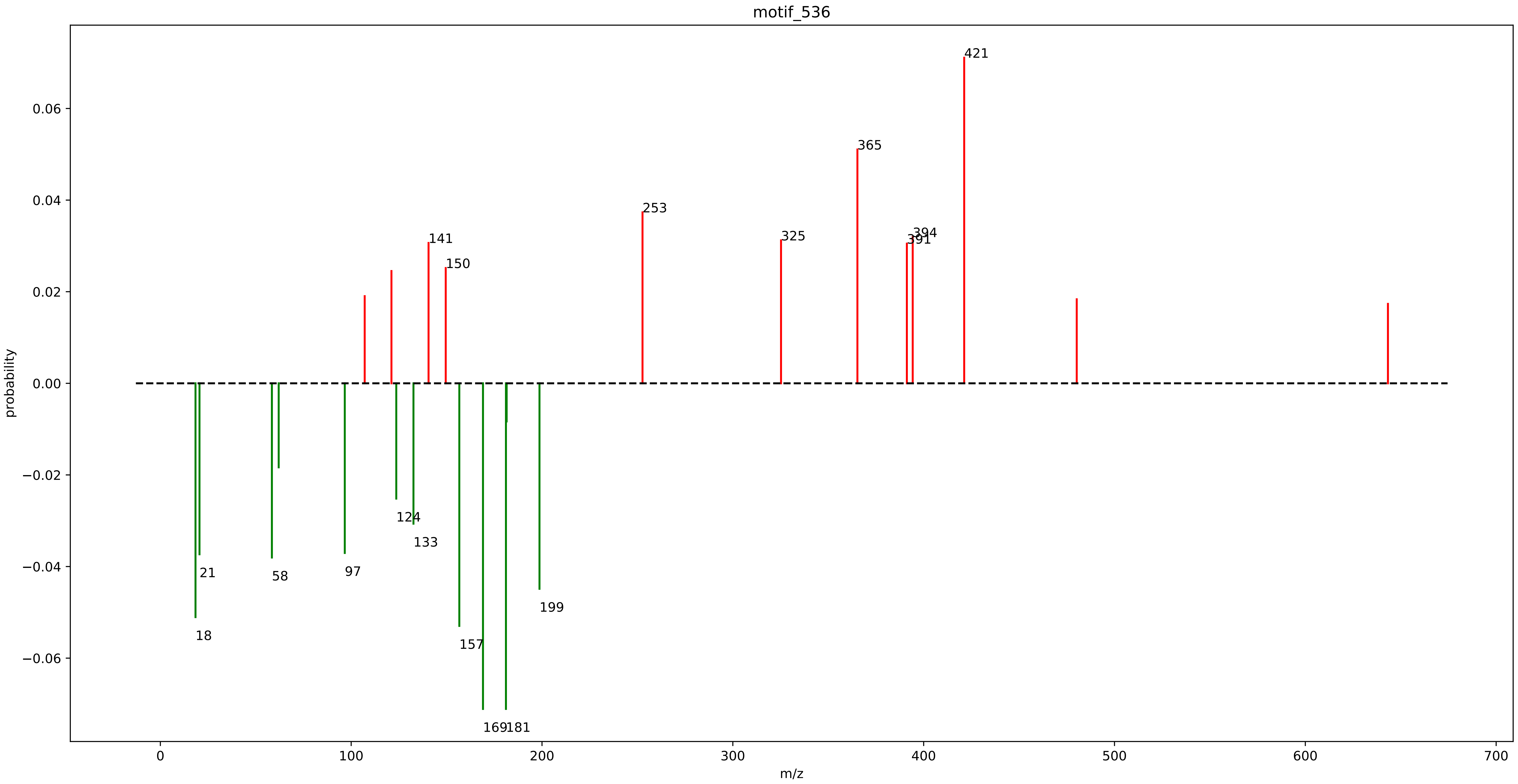

motif_536
probability
m/z
0.06
0.04
0.02
0.00
−0.02
−0.04
−0.06
0
100
200
300
400
500
600
700
421
365
253
141
325
394
391
150
124
133
21
58
97
18
157
199
169
181

motif_536

loss_169.0725	: 0.07
loss_181.1025	: 0.07
fragment_421.2375	: 0.07
loss_156.6675	: 0.05
loss_18.4225	: 0.05
fragment_365.2825	: 0.05
loss_198.6775	: 0.04
loss_58.4525	: 0.04
loss_20.5125	: 0.04
fragment_252.6675	: 0.04
loss_96.6425	: 0.04
fragment_394.2575	: 0.03
fragment_325.2525	: 0.03
fragment_140.5425	: 0.03
loss_132.6375	: 0.03
fragment_391.1975	: 0.03
loss_123.6325	: 0.03
fragment_149.5475	: 0.03
fragment_121.1025	: 0.02
fragment_107.0825	: 0.02
fragment_480.2125	: 0.02
loss_62.0125	: 0.02
fragment_643.2975	: 0.02
loss_181.4725	: 0.01

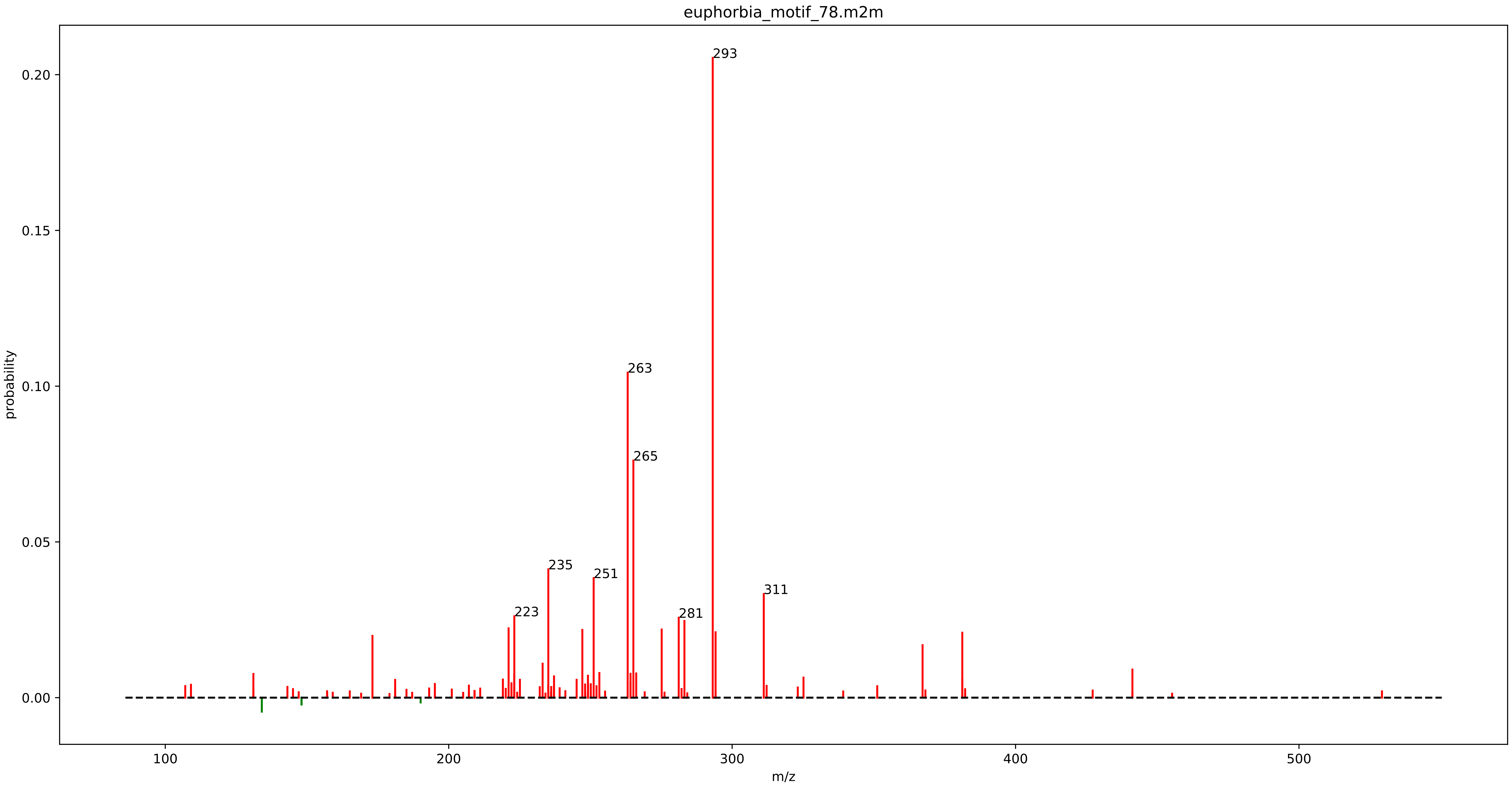
euphorbia_motif_78.m2m
probability
m/z
0.20
0.15
0.10
0.05
0.00
100
200
300
400
500
293
263
265
235
251
223
281
311

euphorbia_motif_78.m2m

fragment_293.1525	: 0.21
fragment_263.1425	: 0.10
fragment_265.1575	: 0.08
fragment_235.1475	: 0.04
fragment_251.1425	: 0.04
fragment_311.1625	: 0.03
fragment_223.1475	: 0.03
fragment_281.1525	: 0.03
fragment_283.1675	: 0.02
fragment_221.1325	: 0.02
fragment_275.1425	: 0.02
fragment_247.1475	: 0.02
fragment_294.1575	: 0.02
fragment_381.2075	: 0.02
fragment_173.0975	: 0.02
fragment_367.1925	: 0.02
fragment_233.1325	: 0.01
fragment_441.2275	: 0.01
fragment_253.1575	: 0.01
fragment_266.1625	: 0.01
fragment_264.1475	: 0.01
fragment_131.0825	: 0.01
fragment_223.1125	: 0.01
fragment_249.1275	: 0.01
fragment_237.1625	: 0.01
fragment_325.1775	: 0.01
fragment_381.2025	: 0.01
fragment_219.1175	: 0.01
fragment_245.1325	: 0.01
fragment_225.1275	: 0.01
fragment_181.1025	: 0.01

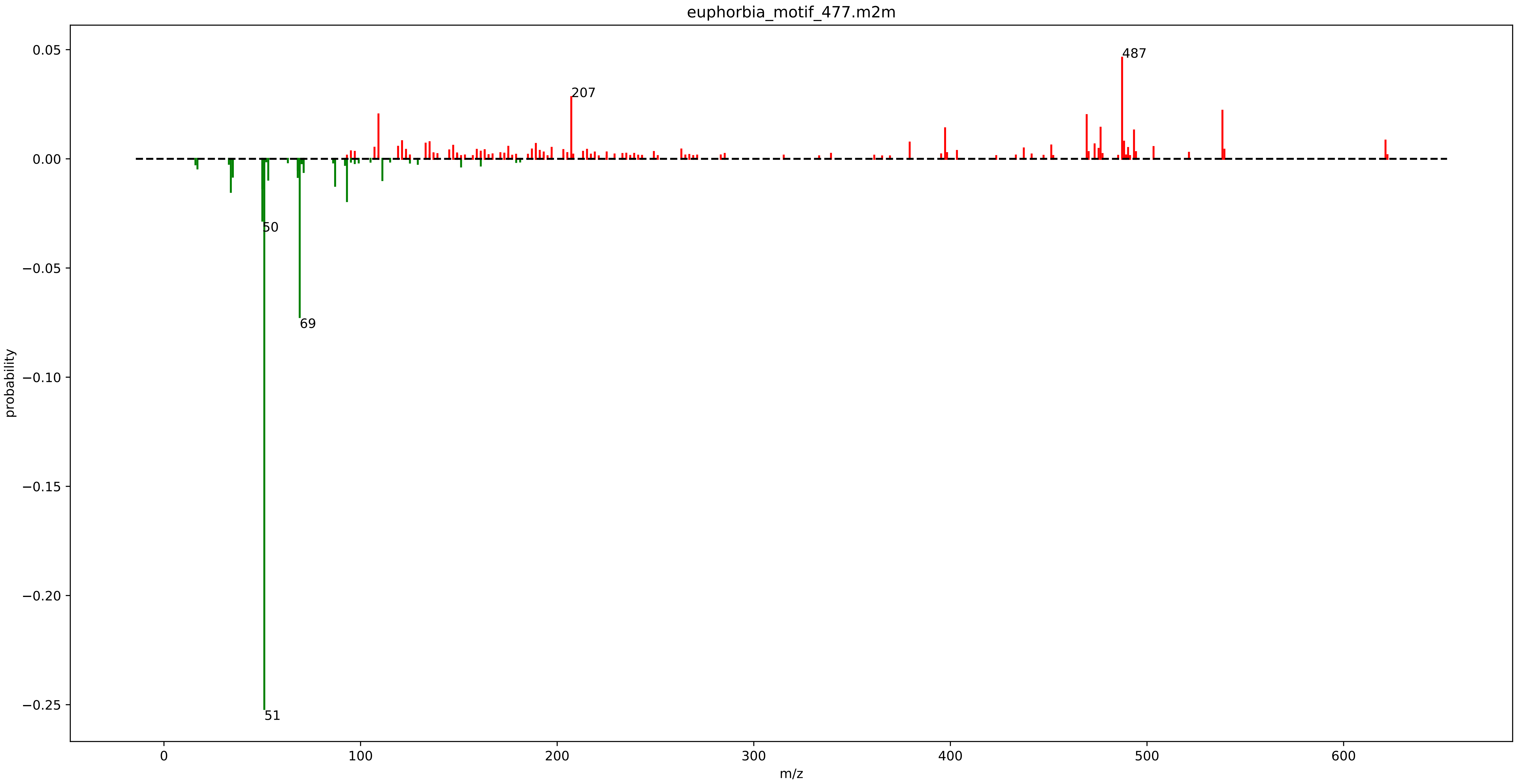

euphorbia_motif_477.m2m
0.05
0.00
−0.05
−0.10
−0.15
−0.20
−0.25
probability
207
487
50
69
51
0
100
200
300
400
500
600
m/z

euphorbia_motif_477.m2m

loss_51.0375	: 0.25
loss_69.0475	: 0.07
fragment_487.3325	: 0.05
loss_50.0325	: 0.03
fragment_207.1725	: 0.03
fragment_538.3625	: 0.02
fragment_109.0975	: 0.02
fragment_469.3225	: 0.02
loss_93.0825	: 0.02
loss_34.0225	: 0.02
fragment_476.3875	: 0.01
fragment_397.3025	: 0.01
loss_50.0375	: 0.01
fragment_493.3875	: 0.01
loss_87.0575	: 0.01
loss_111.0925	: 0.01
loss_53.0525	: 0.01
fragment_621.3075	: 0.01
loss_68.0475	: 0.01
loss_35.0475	: 0.01
fragment_121.0975	: 0.01
fragment_488.3325	: 0.01
fragment_135.1125	: 0.01
fragment_379.2925	: 0.01
fragment_133.0975	: 0.01
fragment_189.1575	: 0.01
fragment_473.3525	: 0.01
fragment_451.3125	: 0.01
loss_71.0625	: 0.01
fragment_147.1125	: 0.01
fragment_119.0825	: 0.01
fragment_175.1475	: 0.01
fragment_503.3275	: 0.01
fragment_107.0825	: 0.01
fragment_197.1925	: 0.01

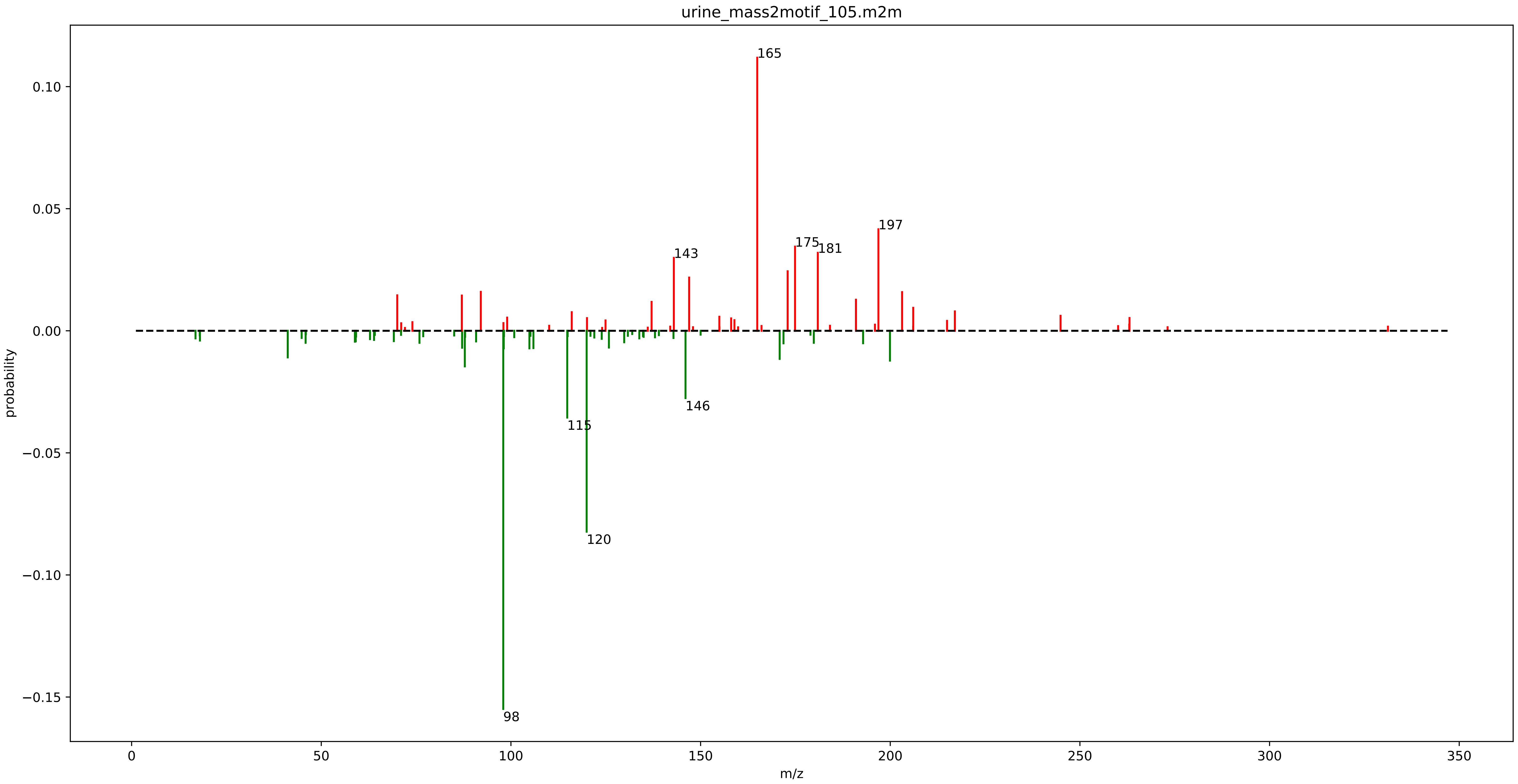
urine_mass2motif_105.m2m
probability
m/z
0.10
0.05
0.00
−0.05
−0.10
−0.15
0
50
100
150
200
250
300
350
165
197
175
181
143
146
115
120
98

urine_mass2motif_105.m2m

loss_97.9775	: 0.15
fragment_164.9325	: 0.11
loss_119.9575	: 0.08
fragment_196.8775	: 0.04
loss_114.8425	: 0.04
fragment_174.8975	: 0.03
fragment_180.9025	: 0.03
fragment_142.9475	: 0.03
loss_146.0225	: 0.03
fragment_172.9525	: 0.02
fragment_146.9825	: 0.02
fragment_92.0575	: 0.02
fragment_203.1225	: 0.02
loss_87.8425	: 0.01
fragment_70.0275	: 0.01
fragment_87.0575	: 0.01
fragment_190.9625	: 0.01
loss_199.9225	: 0.01
fragment_137.0825	: 0.01
loss_170.8475	: 0.01
loss_41.1575	: 0.01
fragment_206.0575	: 0.01
fragment_217.0375	: 0.01
fragment_116.0325	: 0.01
loss_104.8675	: 0.01
loss_98.1075	: 0.01
loss_105.9275	: 0.01
loss_87.1325	: 0.01
loss_125.8275	: 0.01
fragment_244.8975	: 0.01
fragment_154.9425	: 0.01
fragment_99.0075	: 0.01
fragment_263.0775	: 0.01
fragment_120.0525	: 0.01
loss_171.8525	: 0.01
loss_192.8425	: 0.01
fragment_158.0575	: 0.01

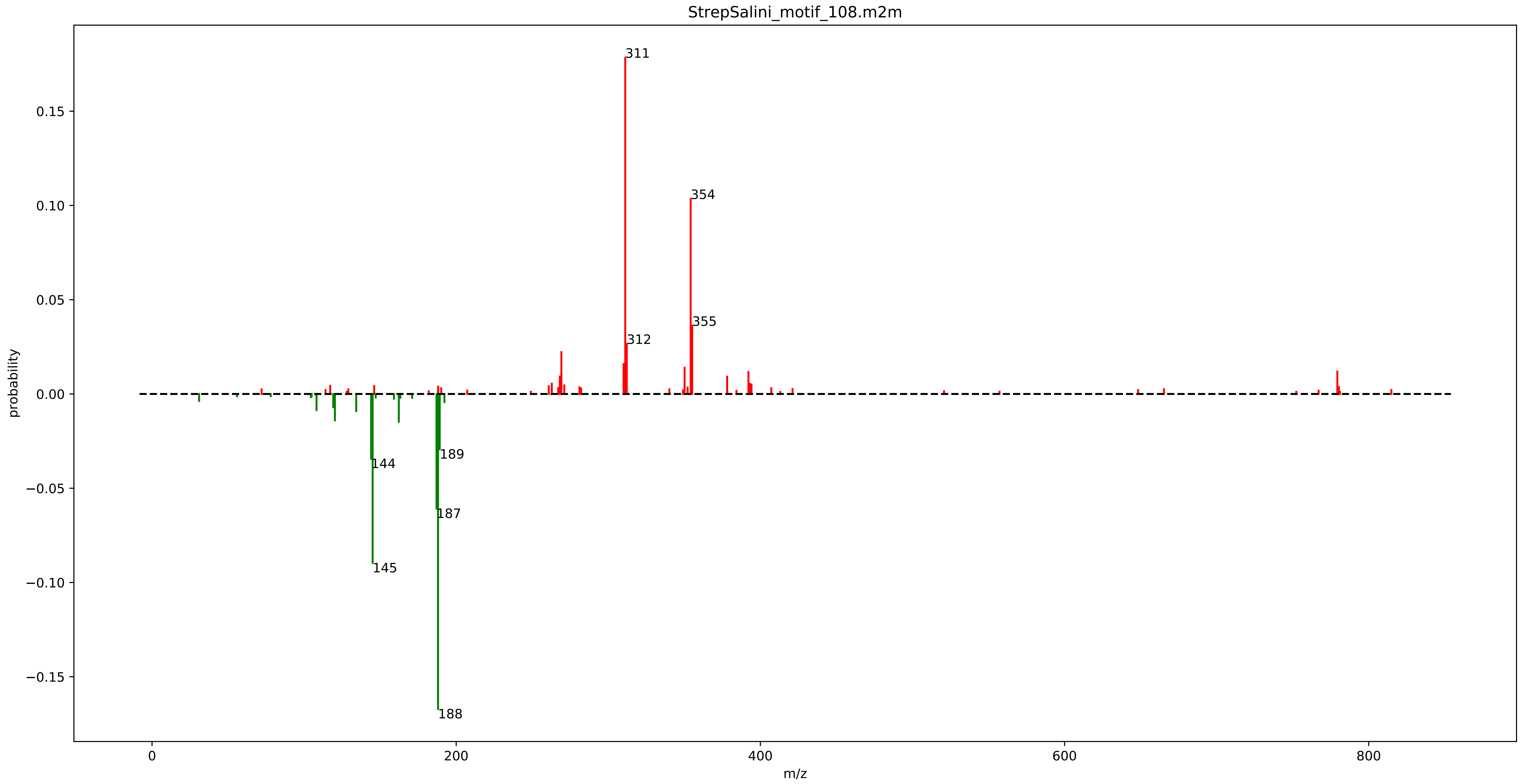

StrepSalini_motif_108.m2m
probability
m/z
0.15
0.10
0.05
0.00
−0.05
−0.10
−0.15
0
200
400
600
800
311
354
355
312
144
145
189
187
188

StrepSalini_motif_108.m2m

fragment_311.1500	: 0.18
loss_188.0500	: 0.17
fragment_354.1500	: 0.10
loss_145.0500	: 0.09
loss_187.0500	: 0.06
fragment_355.1500	: 0.04
loss_144.0525	: 0.03
loss_189.1500	: 0.03
fragment_312.1500	: 0.03
fragment_269.1500	: 0.02
fragment_310.0500	: 0.02
loss_162.2500	: 0.01
loss_120.2500	: 0.01
fragment_350.1500	: 0.01
fragment_779.3500	: 0.01
fragment_392.1500	: 0.01
fragment_268.1525	: 0.01
fragment_378.1500	: 0.01
loss_134.2500	: 0.01
loss_108.1525	: 0.01
loss_119.1500	: 0.01
fragment_262.8500	: 0.01
fragment_393.1500	: 0.01

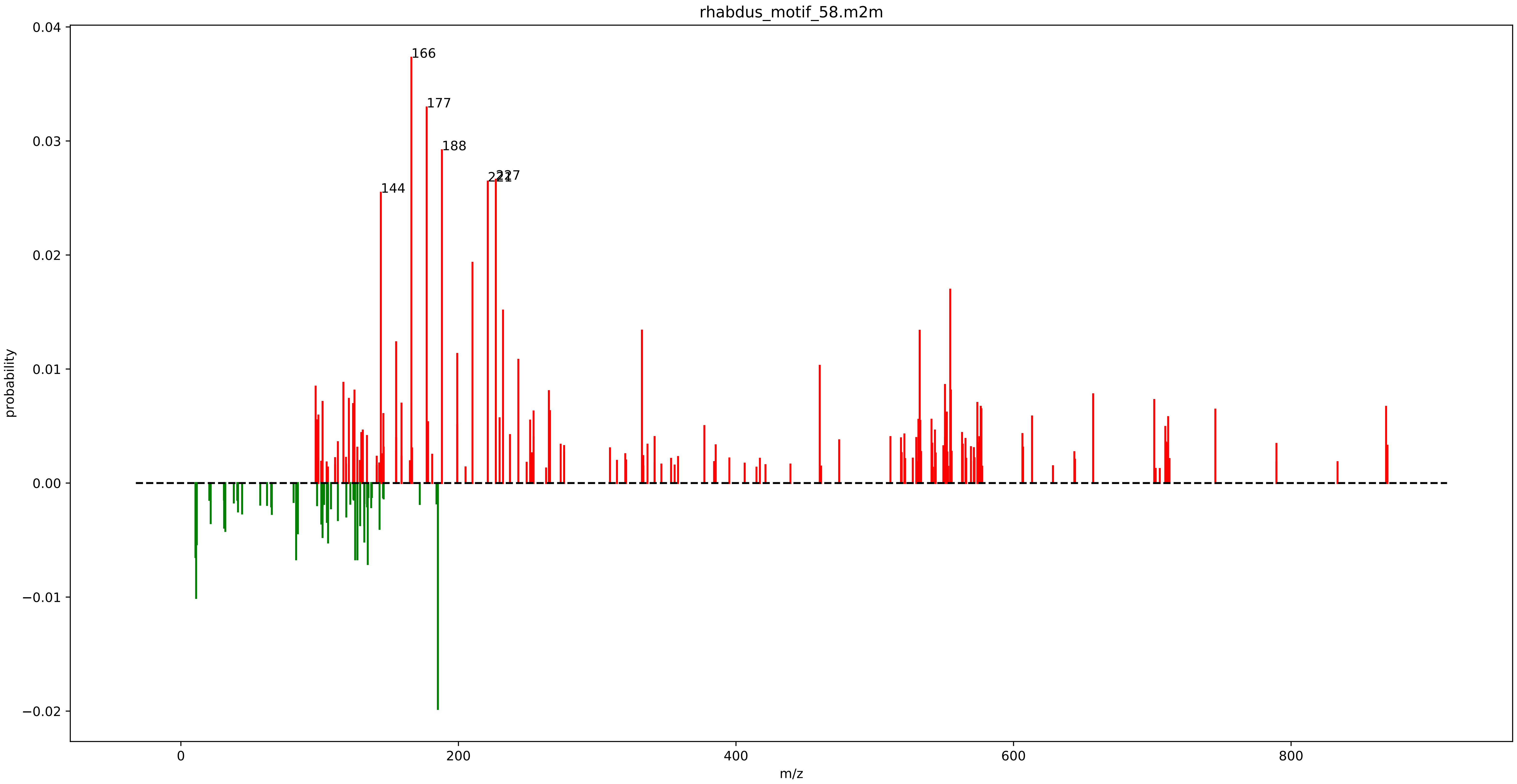

rhabdus_motif_58.m2m
probability
m/z
0.04
0.03
0.02
0.01
0.00
−0.01
−0.02
0
200
400
600
800
166
177
188
221
227
144

rhabdus_motif_58.m2m

fragment_166.0875 : 0.04
fragment_177.1125 : 0.03
fragment_188.1025 : 0.03
fragment_226.9525 : 0.03
fragment_221.1375 : 0.03
fragment_144.0775 : 0.03
loss_185.1775 : 0.02
fragment_210.1175 : 0.02
fragment_554.3725 : 0.02
fragment_232.1275 : 0.02
fragment_332.2225 : 0.01
fragment_532.3575 : 0.01
fragment_155.1075 : 0.01
fragment_199.1275 : 0.01
fragment_243.1525 : 0.01
fragment_460.3425 : 0.01
loss_10.9875 : 0.01
fragment_117.0875 : 0.01
fragment_550.5975 : 0.01
fragment_97.0975 : 0.01
fragment_554.8725 : 0.01
fragment_125.0725 : 0.01
fragment_265.1625 : 0.01
fragment_657.3675 : 0.01
fragment_121.0625 : 0.01
fragment_701.3925 : 0.01
fragment_102.0675 : 0.01
loss_134.6525 : 0.01
fragment_573.9075 : 0.01
fragment_158.9625 : 0.01
fragment_124.0775 : 0.01
fragment_868.4625 : 0.01
fragment_576.3775 : 0.01
fragment_576.3675 : 0.01
loss_83.0525 : 0.01
loss_125.5725 : 0.01
loss_127.1875 : 0.01
loss_10.4875 : 0.01
fragment_576.8875 : 0.01
fragment_745.4175 : 0.01
fragment_265.9625 : 0.01
fragment_254.1425 : 0.01
fragment_551.8925 : 0.01
fragment_145.9275 : 0.01
fragment_99.1125 : 0.01
fragment_613.3425 : 0.01
fragment_711.4175 : 0.01
fragment_229.6525 : 0.01
fragment_540.9025 : 0.01
fragment_531.4325 : 0.01
fragment_97.9675 : 0.01
fragment_251.6625 : 0.01
fragment_532.8575 : 0.01
loss_11.4925 : 0.01
fragment_178.1175 : 0.01
loss_106.0675 : 0.01
loss_132.1775 : 0.01
fragment_199.1225 : 0.01

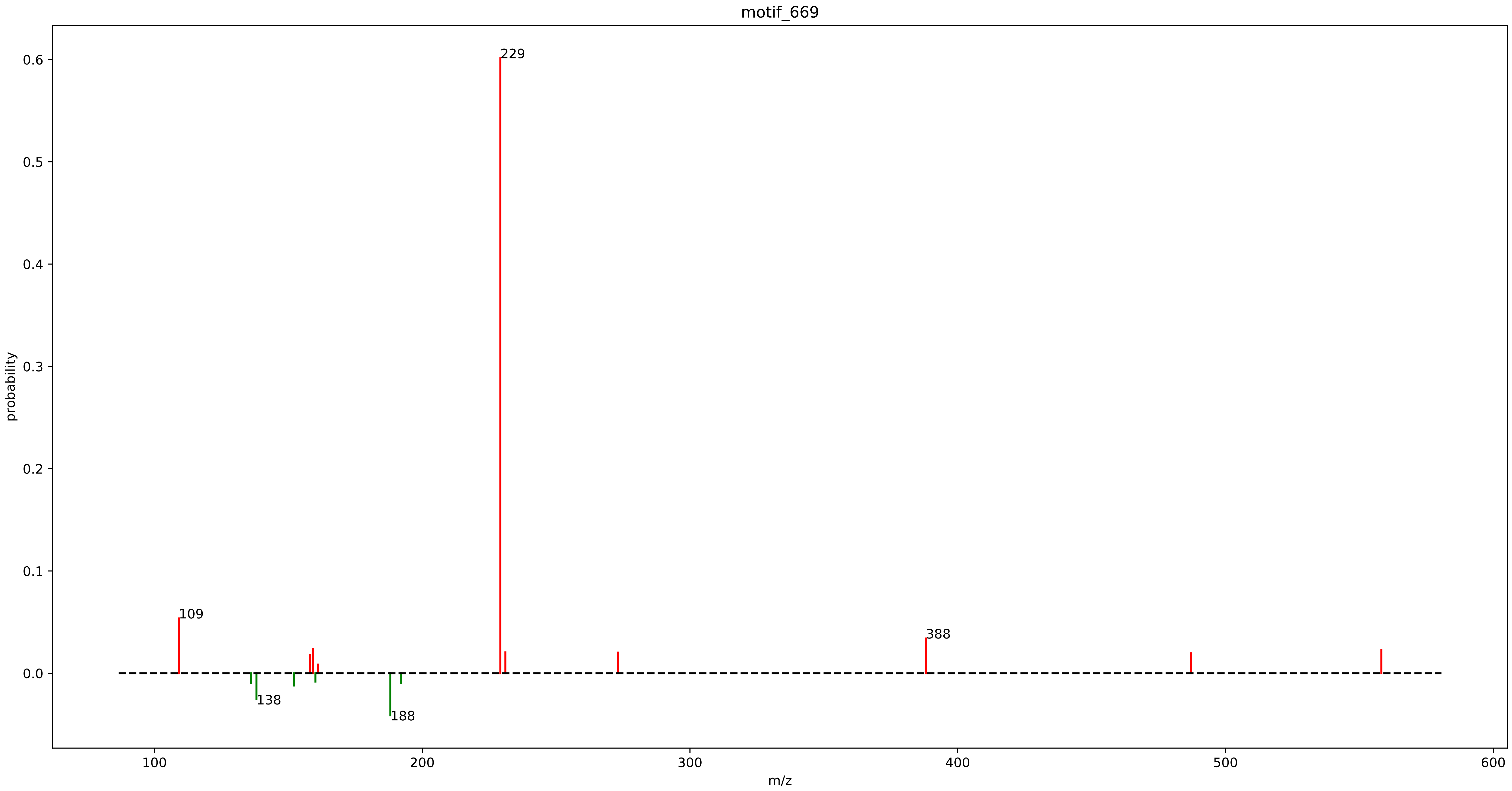
motif_669
probability
m/z
229
109
388
138
188
0.6
0.5
0.4
0.3
0.2
0.1
0.0
100
200
300
400
500
600

motif_669

fragment_229.1925	: 0.60
fragment_109.0975	: 0.05
loss_188.1175	: 0.04
fragment_388.0975	: 0.03
loss_138.1025	: 0.03
fragment_159.1175	: 0.02
fragment_558.2075	: 0.02
fragment_231.0575	: 0.02
fragment_273.0725	: 0.02
fragment_487.1675	: 0.02
fragment_158.0425	: 0.02
loss_152.1175	: 0.01
loss_192.1475	: 0.01
loss_136.0875	: 0.01
fragment_161.1325	: 0.01
loss_160.1225	: 0.01

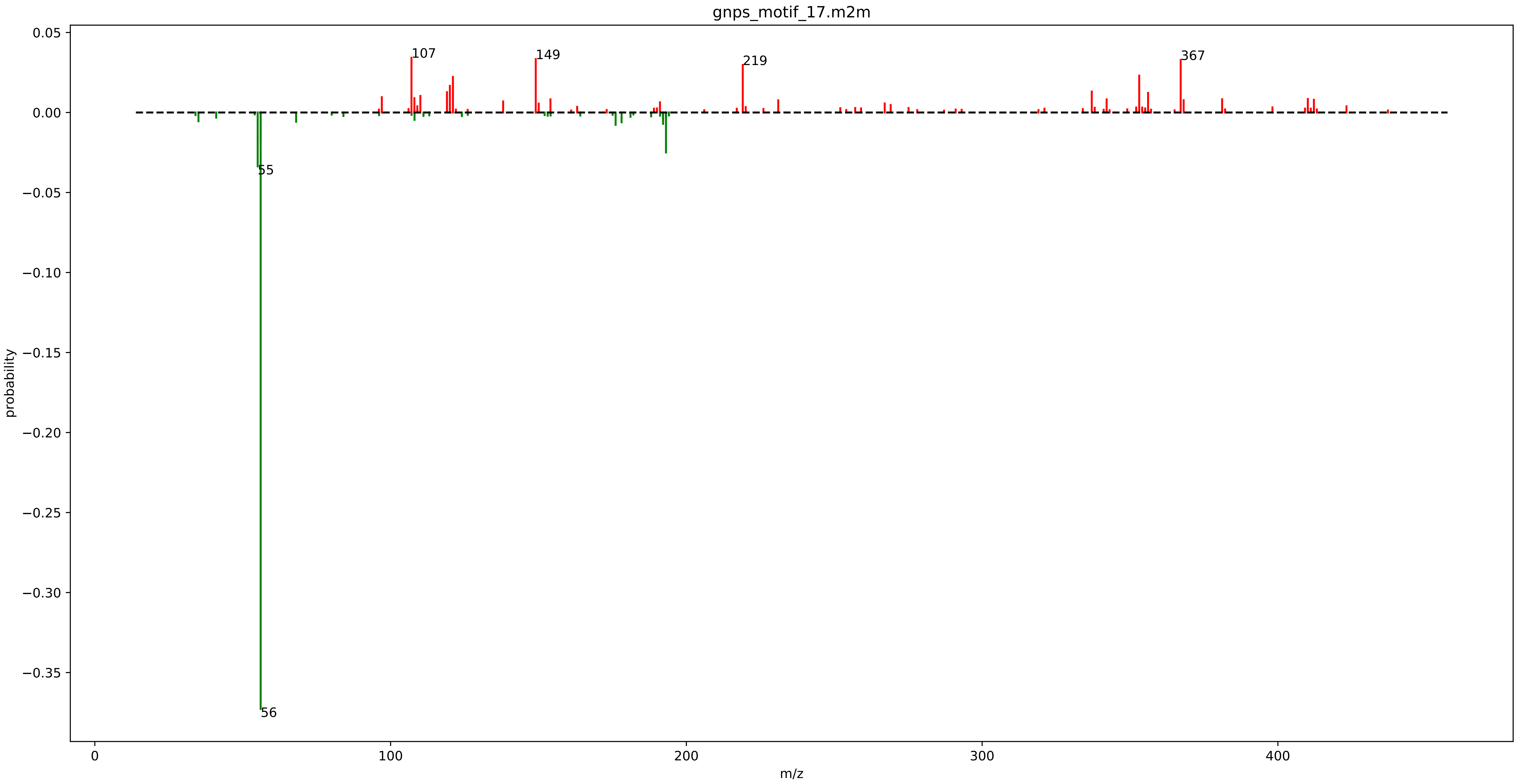

gnps_motif_17.m2m
probability
m/z
107
149
219
367
55
56
0.05
0.00
−0.05
−0.10
−0.15
−0.20
−0.25
−0.30
−0.35
0
100
200
300
400

gnps_motif_17.m2m

loss_56.0625	: 0.37
fragment_107.0625	: 0.03
loss_55.0575	: 0.03
fragment_149.0725	: 0.03
fragment_367.1325	: 0.03
fragment_219.0675	: 0.03
loss_193.1125	: 0.02
loss_55.0625	: 0.02
fragment_353.1125	: 0.02
fragment_121.0775	: 0.02
loss_56.0575	: 0.02
fragment_120.0425	: 0.02
fragment_337.0725	: 0.01
fragment_119.0625	: 0.01
fragment_356.1125	: 0.01
fragment_110.0625	: 0.01
fragment_97.0275	: 0.01
fragment_108.0425	: 0.01
fragment_410.1225	: 0.01
fragment_381.1475	: 0.01
fragment_154.0475	: 0.01
fragment_342.0975	: 0.01
fragment_412.1775	: 0.01
loss_176.0825	: 0.01
fragment_368.1375	: 0.01
fragment_231.0675	: 0.01
loss_192.1275	: 0.01
fragment_138.0525	: 0.01
fragment_191.0725	: 0.01
loss_178.0975	: 0.01
loss_68.0625	: 0.01
fragment_267.0625	: 0.01
fragment_150.0775	: 0.01
loss_35.0375	: 0.01

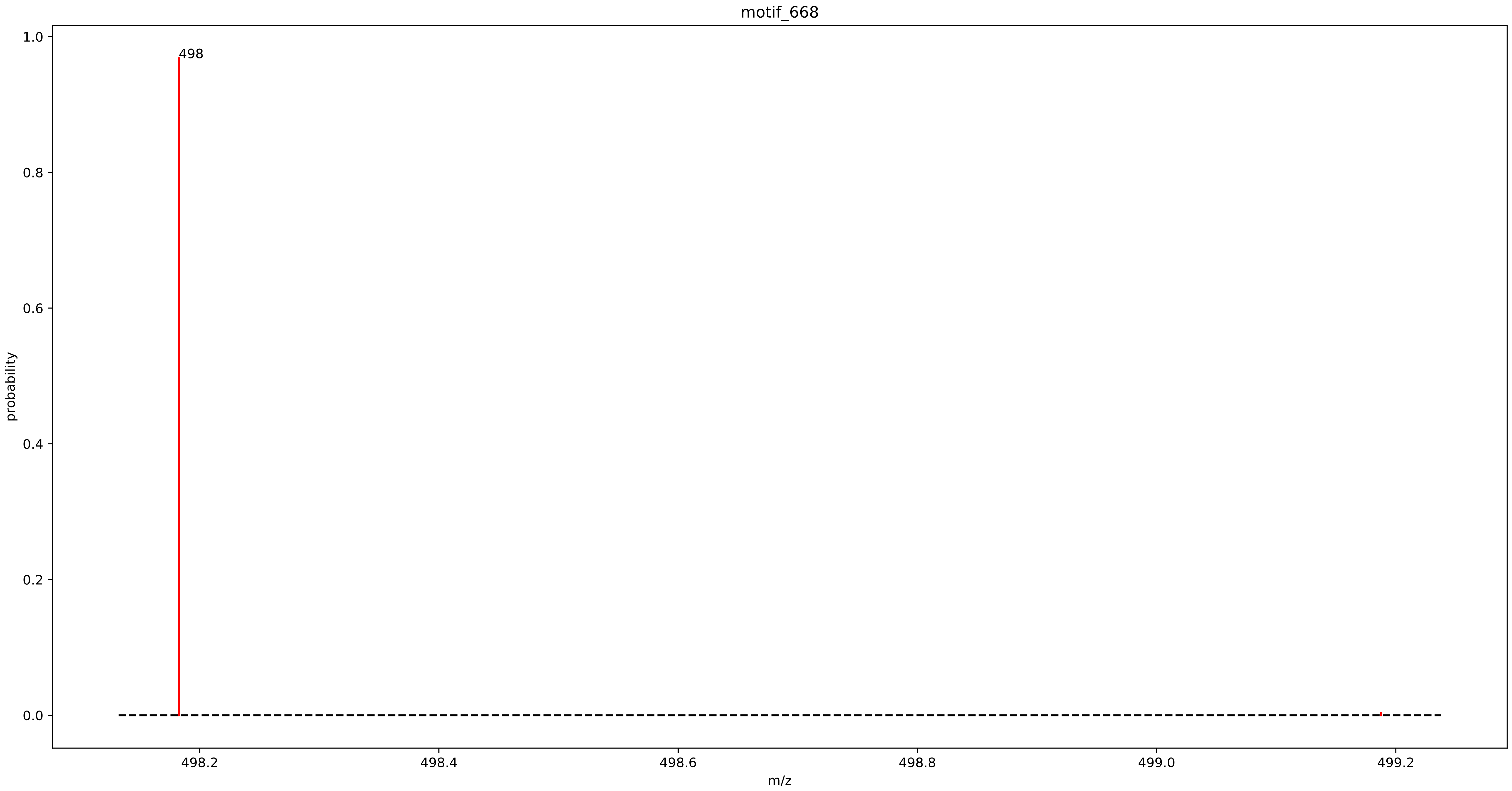

motif_668
498
probability
m/z
1.0
0.8
0.6
0.4
0.2
0.0
498.2
498.4
498.6
498.8
499.0
499.2

motif_668

fragment_498.1825 : 0.97

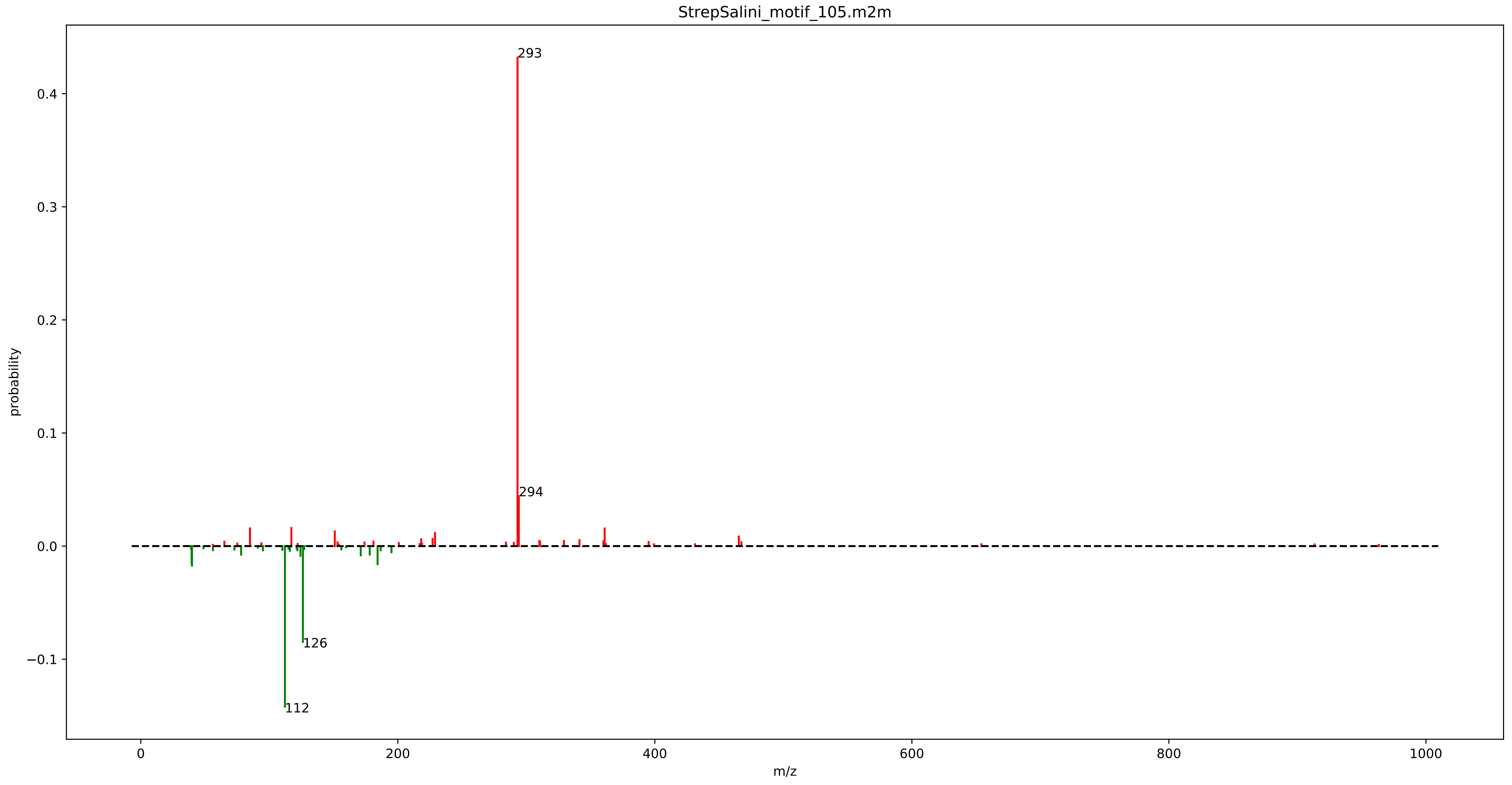

StrepSalini_motif_105.m2m
probability
m/z
0.4
0.3
0.2
0.1
0.0
−0.1
0
200
400
600
800
1000
293
294
126
112

StrepSalini_motif_105.m2m

fragment_293.1500	: 0.43
loss_112.1500	: 0.14
loss_126.1525	: 0.08
fragment_294.1500	: 0.04
loss_39.8500	: 0.02
fragment_117.1500	: 0.02
loss_184.2525	: 0.02
fragment_84.9500	: 0.02
fragment_360.9500	: 0.02
loss_39.5500	: 0.02
fragment_150.9500	: 0.01
fragment_228.9500	: 0.01
loss_124.2500	: 0.01
fragment_465.3500	: 0.01
loss_171.1500	: 0.01
loss_78.0500	: 0.01
loss_178.1500	: 0.01
fragment_227.0500	: 0.01
fragment_218.1500	: 0.01
loss_195.0525	: 0.01
fragment_341.3500	: 0.01

rhabdus_motif_295.m2m

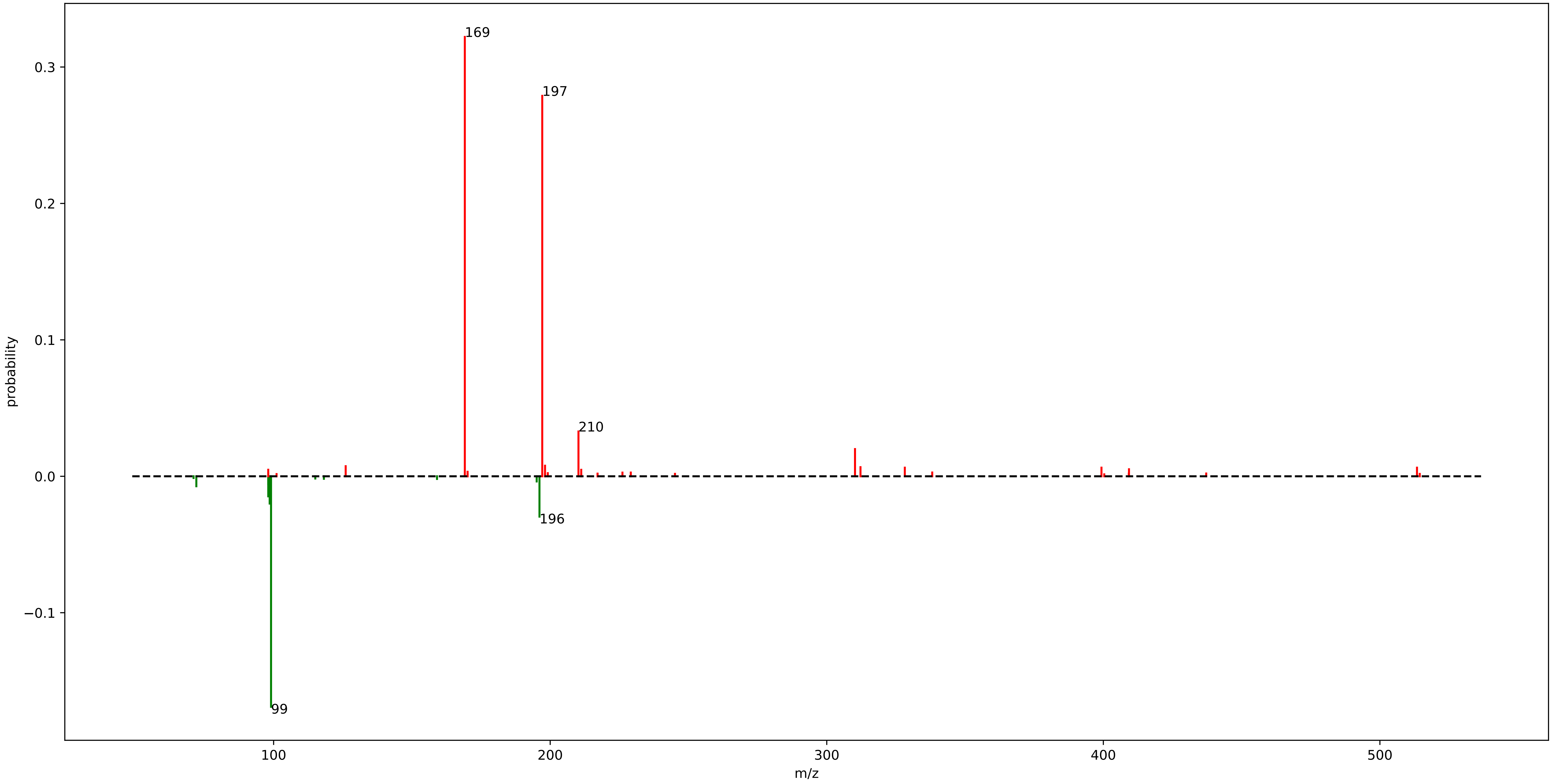

rhabdus_motif_295.m2m

fragment_169.1325	: 0.32
fragment_197.1275	: 0.28
loss_99.0675	: 0.17
fragment_210.2225	: 0.03
loss_196.1225	: 0.03
loss_98.5675	: 0.02
fragment_310.2125	: 0.02
loss_98.0625	: 0.01
fragment_198.1325	: 0.01
fragment_126.0525	: 0.01
loss_72.0575	: 0.01
fragment_312.1925	: 0.01
fragment_513.4125	: 0.01
fragment_399.3275	: 0.01
fragment_328.2225	: 0.01
loss_98.0675	: 0.01
fragment_409.2775	: 0.01

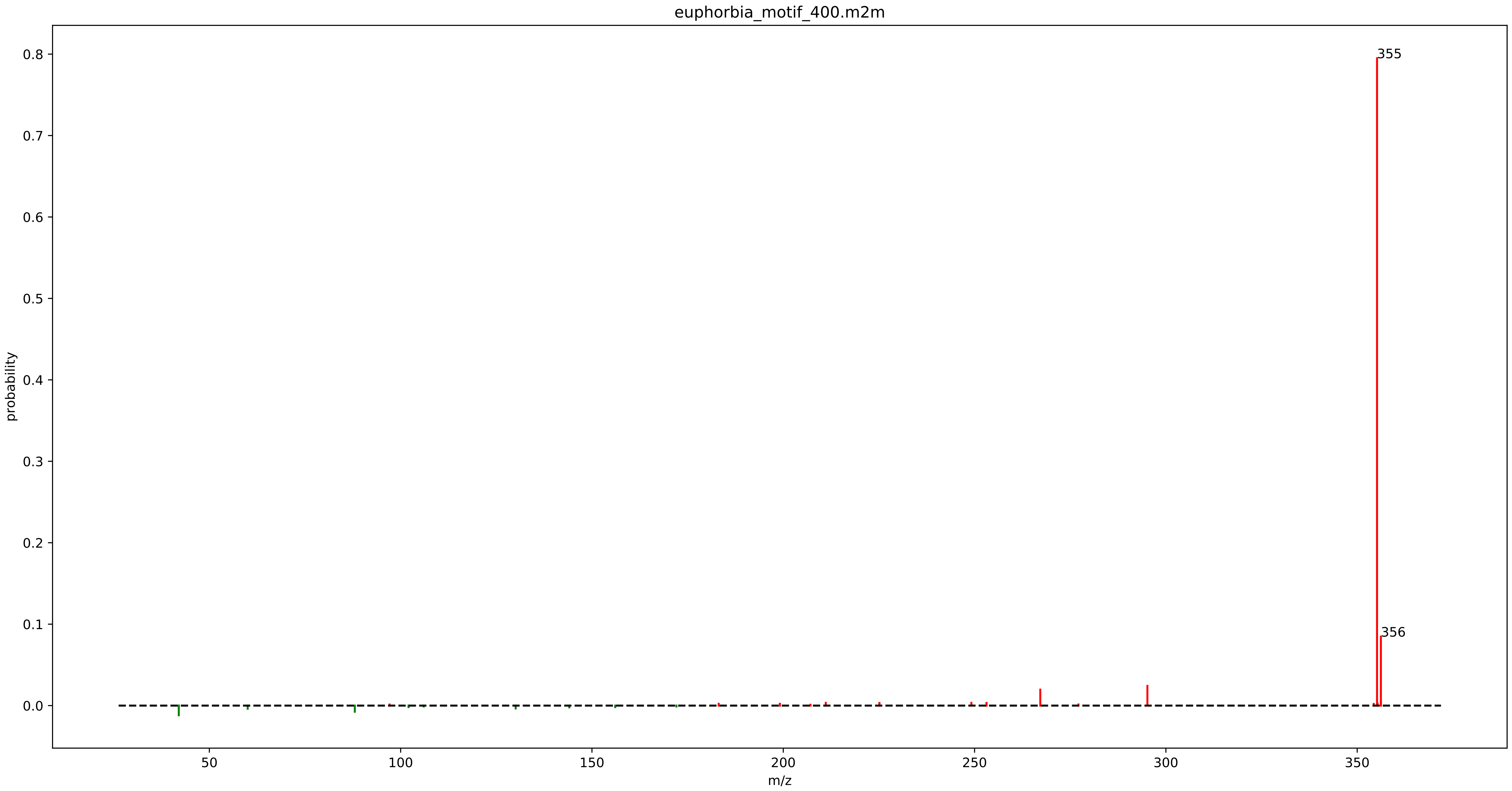

euphorbia_motif_400.m2m
probability
m/z
355
356
0.8
0.7
0.6
0.5
0.4
0.3
0.2
0.1
0.0
50
100
150
200
250
300
350

euphorbia_motif_400.m2m

fragment_355.1875	: 0.79
fragment_356.1925	: 0.08
fragment_295.1675	: 0.02
fragment_267.1725	: 0.02
loss_42.0125	: 0.01
loss_88.0175	: 0.01

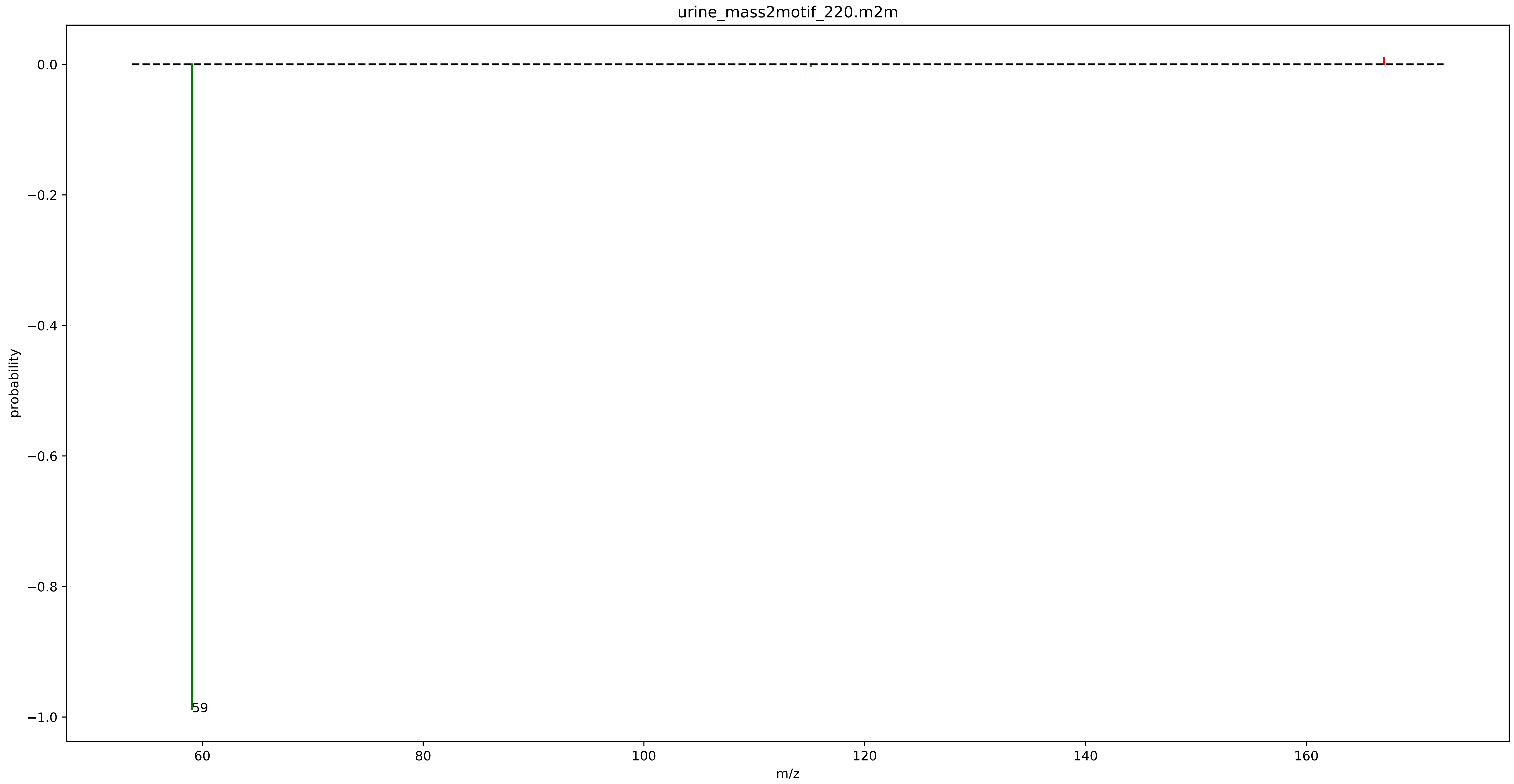

urine_mass2motif_220.m2m
0.0
−0.2
−0.4
−0.6
−0.8
−1.0
probability
59
60
80
100
120
140
160
m/z

urine_mass2motif_220.m2m

loss_59.0475	: 0.99
fragment_167.0325	: 0.01

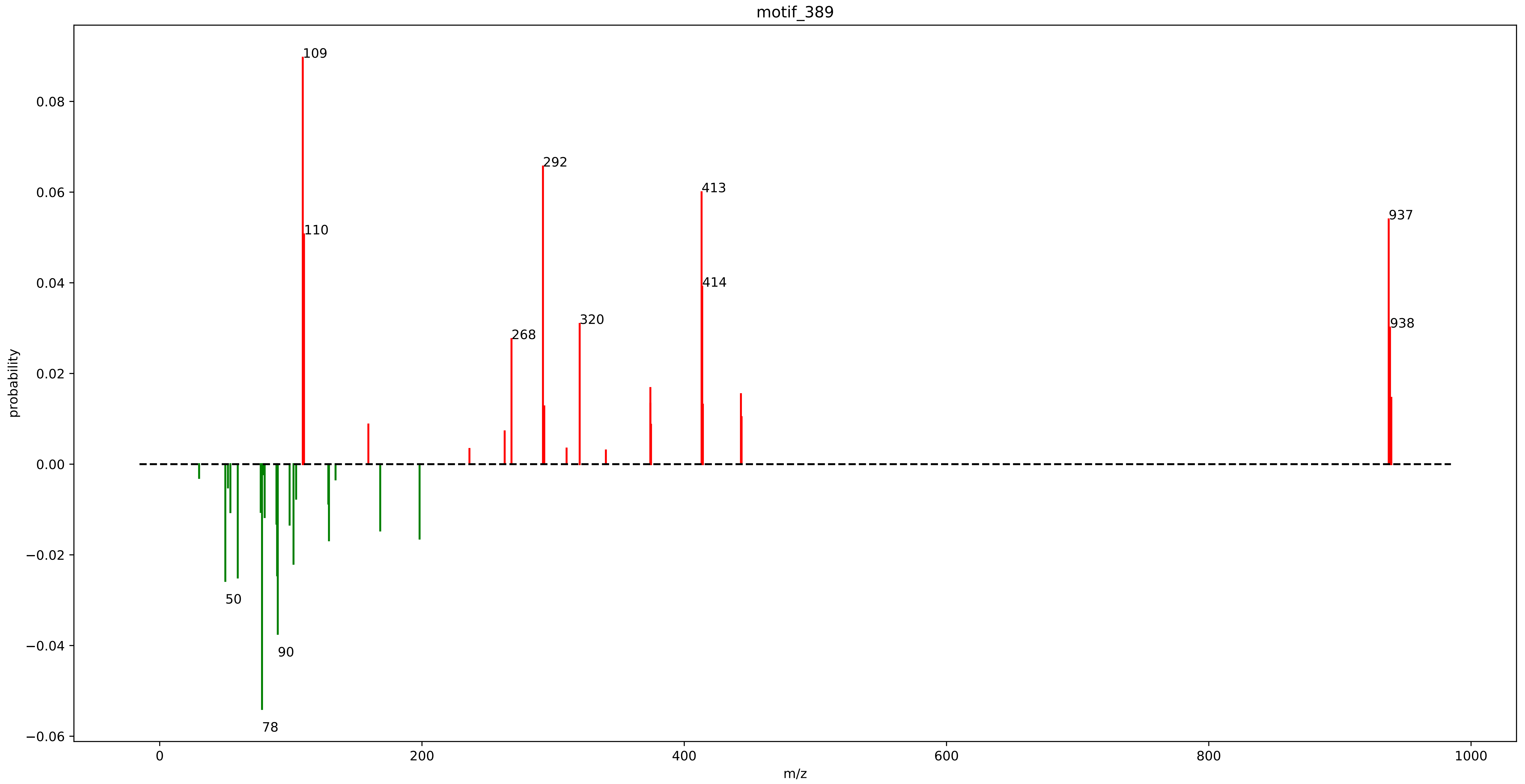

motif_389
probability
m/z
0.08
0.06
0.04
0.02
0.00
−0.02
−0.04
−0.06
0
200
400
600
800
1000
109
110
292
413
414
937
938
268
320
50
90
78

motif_389

fragment_109.0975	: 0.09
fragment_292.2625	: 0.07
fragment_413.2175	: 0.06
fragment_937.1975	: 0.05
loss_78.0275	: 0.05
fragment_110.0575	: 0.05
fragment_413.7175	: 0.04
loss_90.0325	: 0.04
fragment_320.2575	: 0.03
fragment_938.2025	: 0.03
fragment_268.2625	: 0.03
loss_50.0375	: 0.03
loss_59.5175	: 0.02
loss_89.5275	: 0.02
loss_102.0325	: 0.02
fragment_374.1725	: 0.02
loss_129.0725	: 0.02
loss_198.1775	: 0.02
fragment_443.2275	: 0.02
fragment_939.2025	: 0.01
loss_168.1625	: 0.01
loss_99.0575	: 0.01
fragment_374.1775	: 0.01
fragment_414.2175	: 0.01
loss_89.0275	: 0.01
fragment_293.2675	: 0.01
loss_80.0475	: 0.01
loss_53.9075	: 0.01
loss_77.0275	: 0.01
fragment_443.7275	: 0.01
fragment_159.1175	: 0.01
fragment_374.6775	: 0.01
loss_128.5725	: 0.01
loss_104.0475	: 0.01
fragment_263.0625	: 0.01
loss_52.0525	: 0.01

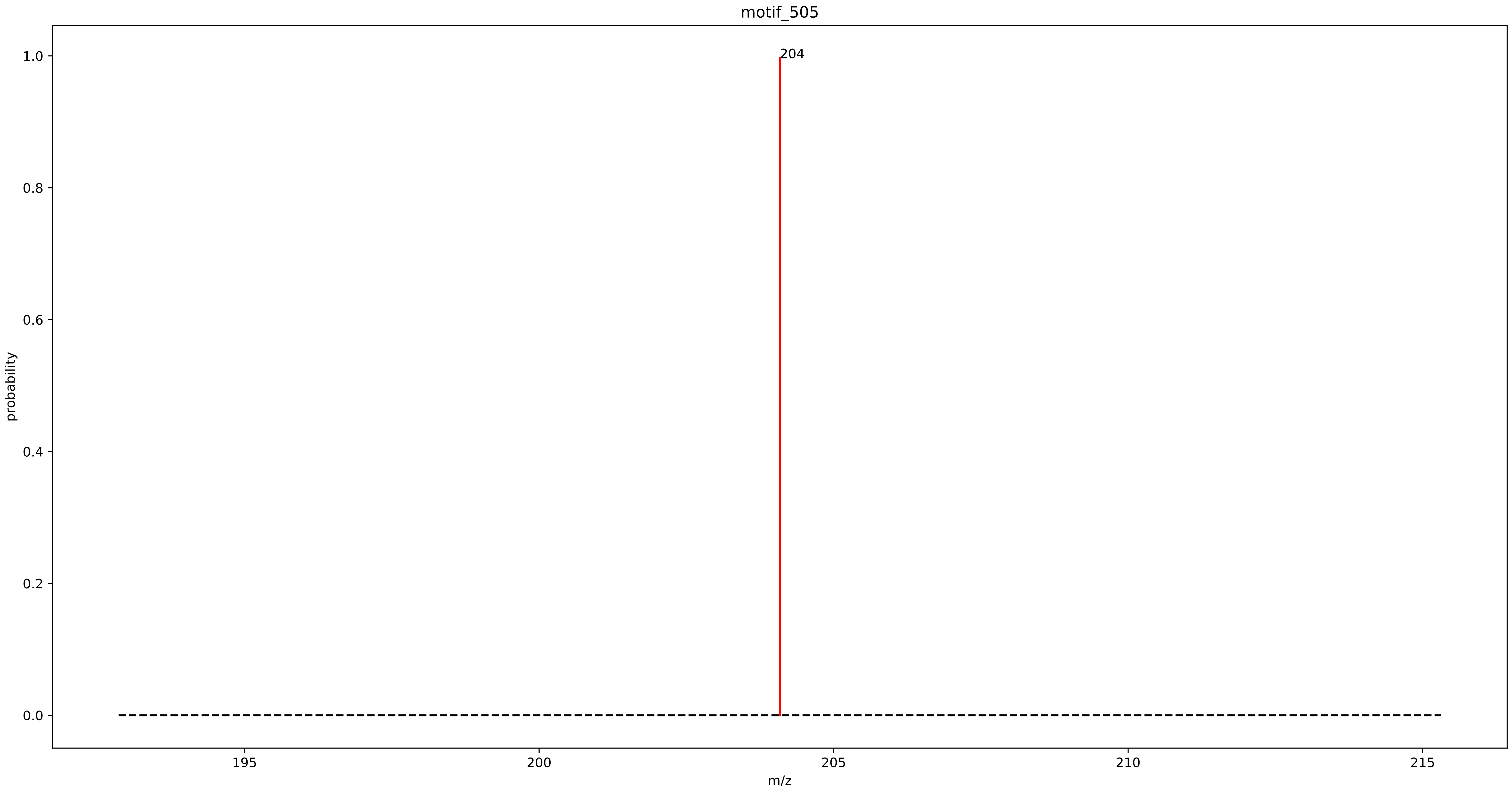
motif_505
204
1.0
0.8
0.6
0.4
0.2
0.0
probability
195
200
205
210
215
m/z

motif_505

fragment_204.0875 : 1.00

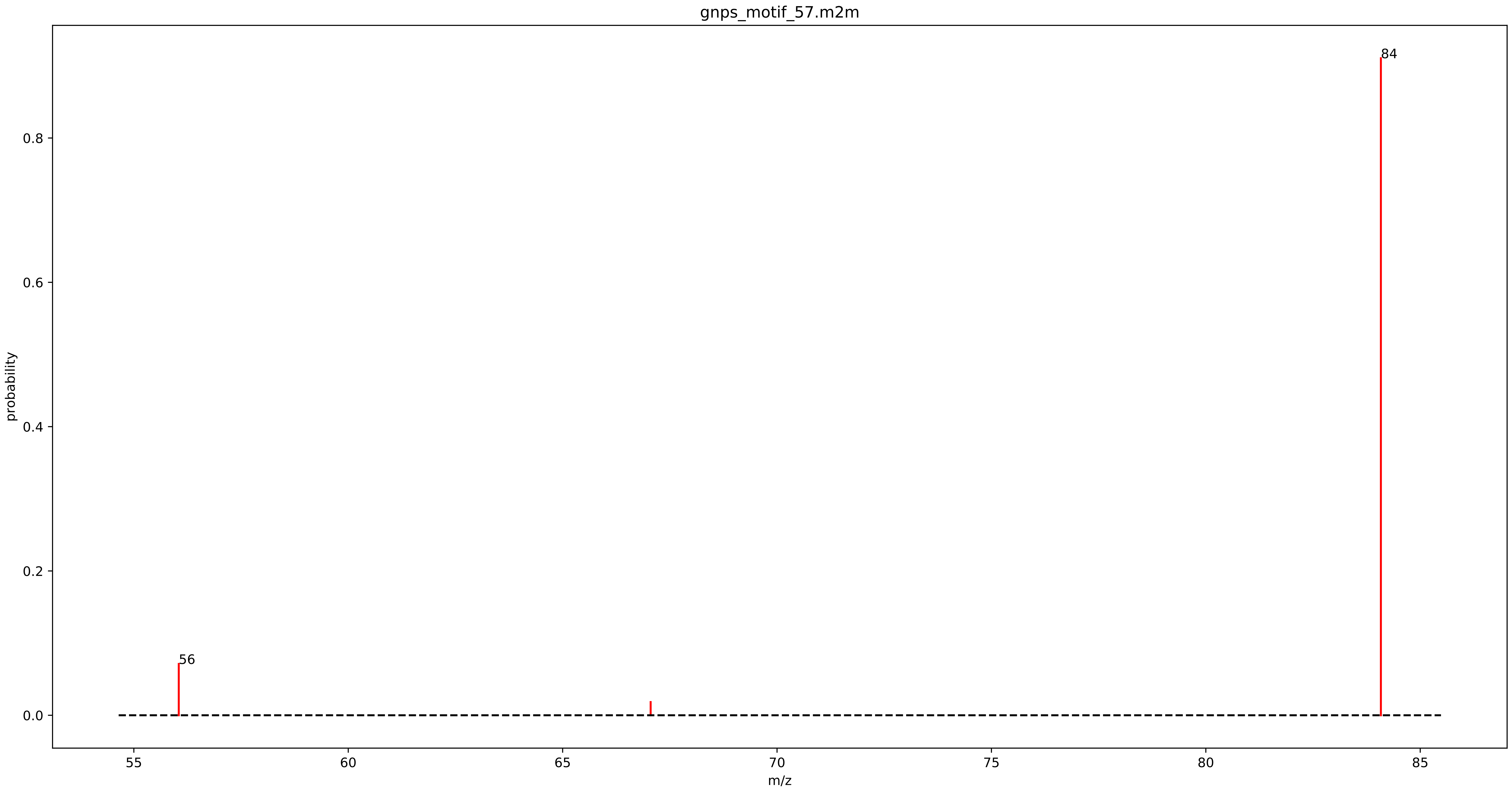
gnps_motif_57.m2m
probability
m/z
84
56
0.0
0.2
0.4
0.6
0.8
55
60
65
70
75
80
85

gnps_motif_57.m2m

fragment_84.0825	: 0.91
fragment_56.0475	: 0.07
fragment_67.0525	: 0.02

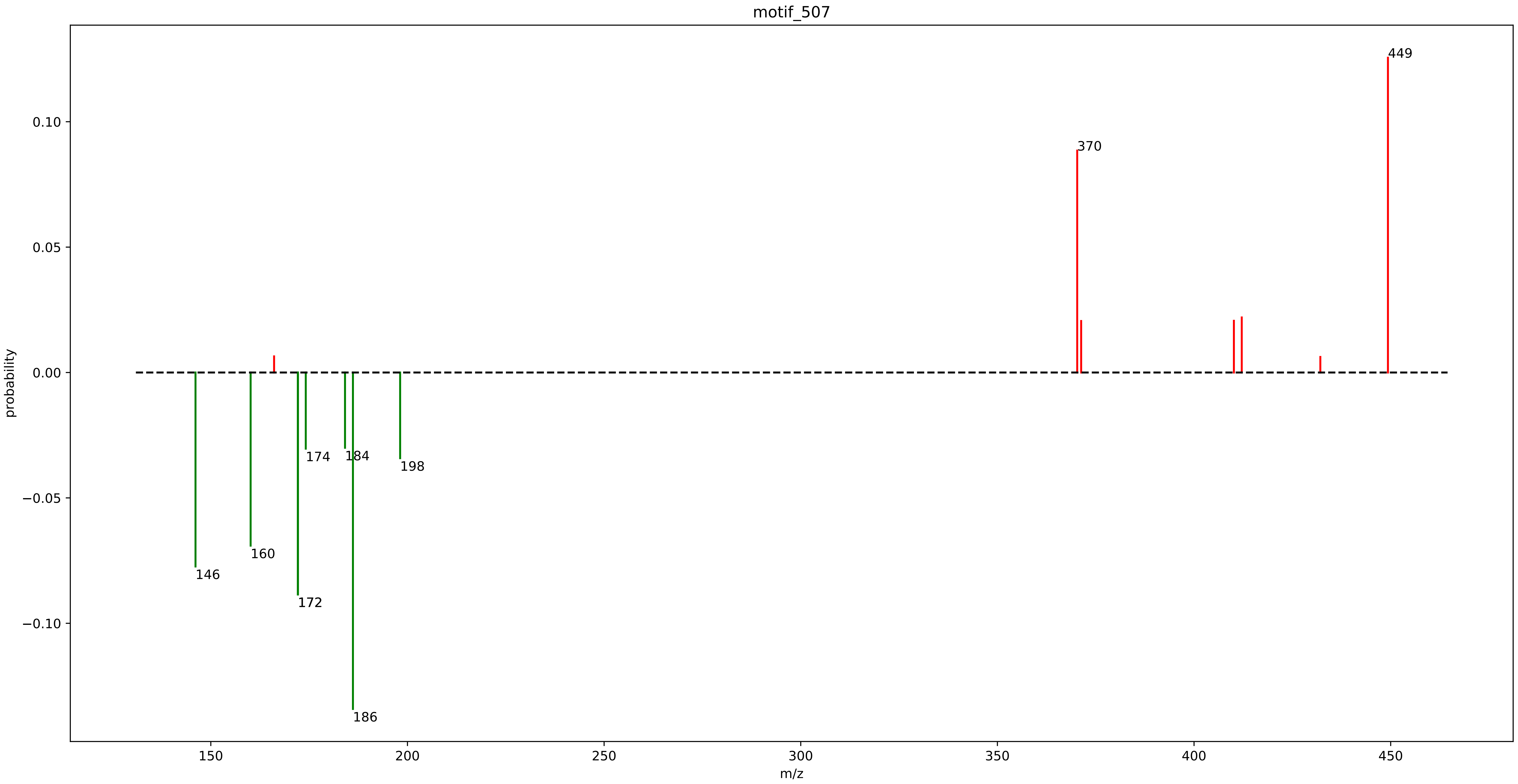
motif_507
probability
m/z
0.10
0.05
0.00
−0.05
−0.10
150
200
250
300
350
400
450
449
370
146
160
172
174
184
186
198

motif_507

loss_186.1375	: 0.13
fragment_449.3025	: 0.13
fragment_370.2925	: 0.09
loss_172.1625	: 0.09
loss_172.1225	: 0.09
loss_146.1075	: 0.08
loss_160.1225	: 0.07
loss_198.1375	: 0.03
loss_174.1425	: 0.03
loss_184.1225	: 0.03
fragment_412.1325	: 0.02
fragment_410.1325	: 0.02
fragment_371.2975	: 0.02
fragment_166.0875	: 0.01
fragment_432.1125	: 0.01

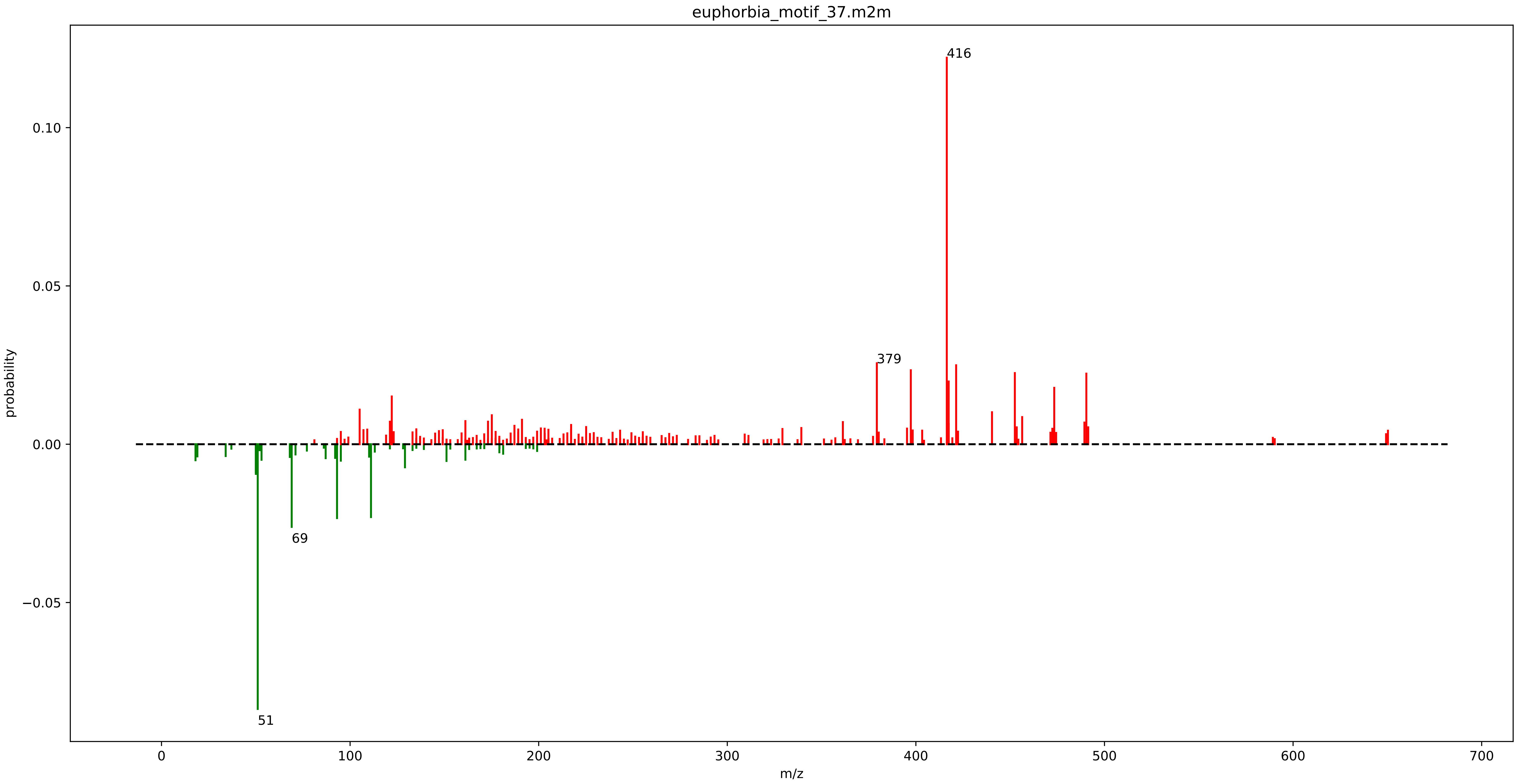

euphorbia_motif_37.m2m
416
379
69
51
0.10
0.05
0.00
−0.05
probability
0
100
200
300
400
500
600
700
m/z

euphorbia_motif_37.m2m

fragment_416.3875	: 0.12
loss_51.0275	: 0.08
loss_69.0375	: 0.03
fragment_379.3025	: 0.03
fragment_421.3475	: 0.02
fragment_397.3125	: 0.02
loss_93.0775	: 0.02
loss_111.0875	: 0.02
fragment_452.4775	: 0.02
fragment_490.3875	: 0.02
fragment_417.3925	: 0.02
fragment_473.3625	: 0.02
fragment_122.0975	: 0.02
fragment_105.0675	: 0.01
fragment_440.3625	: 0.01
loss_50.0275	: 0.01
fragment_175.1475	: 0.01
loss_50.0225	: 0.01
fragment_456.3575	: 0.01
fragment_191.1425	: 0.01
fragment_161.1325	: 0.01
loss_129.0975	: 0.01
fragment_173.1325	: 0.01
fragment_121.1025	: 0.01
fragment_361.2875	: 0.01
fragment_489.3575	: 0.01
fragment_417.3875	: 0.01
fragment_217.1975	: 0.01
fragment_187.1475	: 0.01
fragment_225.1625	: 0.01
fragment_491.3825	: 0.01
fragment_453.4825	: 0.01
loss_151.1175	: 0.01
loss_95.0925	: 0.01
fragment_339.2675	: 0.01
loss_18.0325	: 0.01

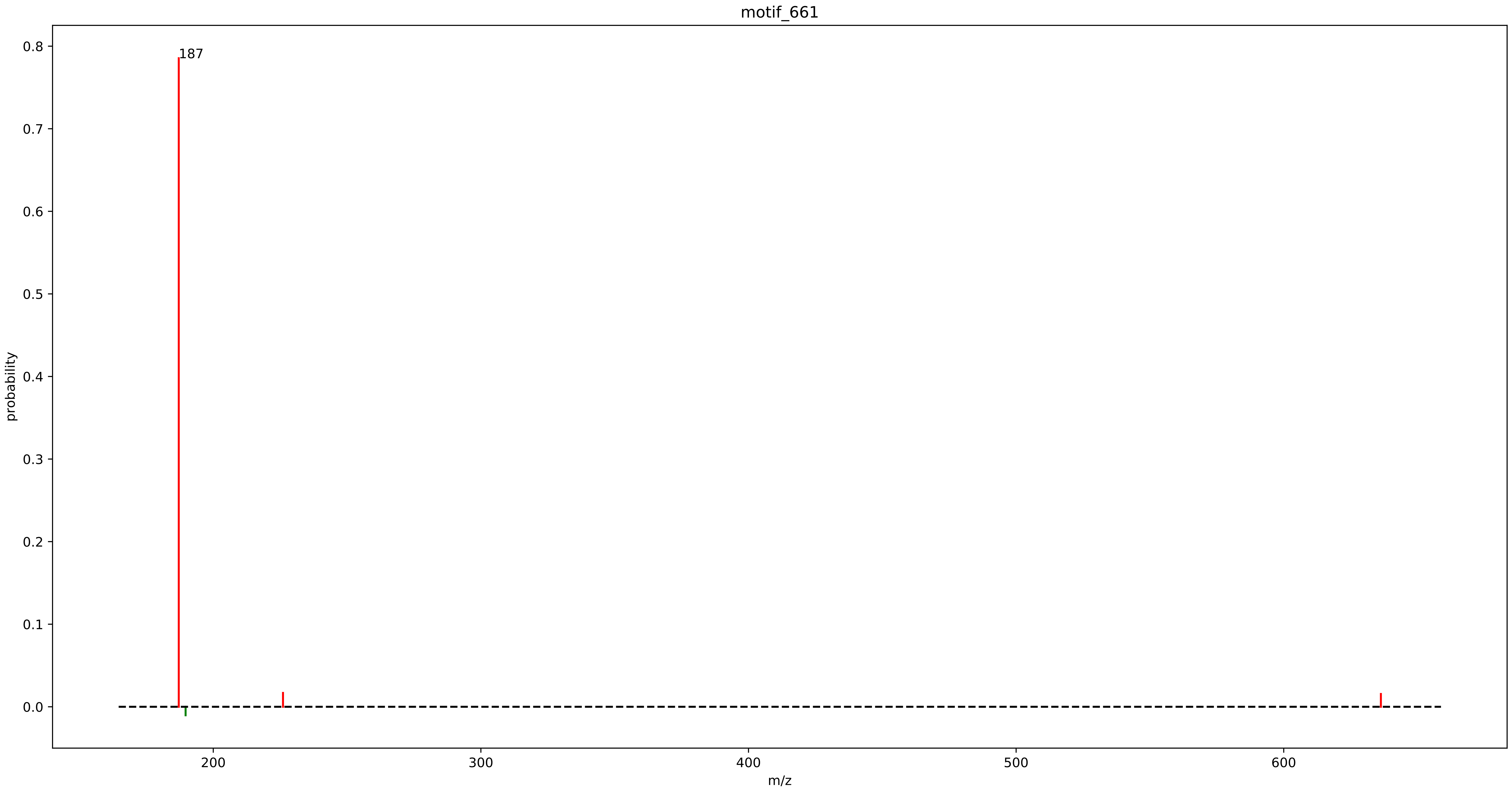

motif_661
187
probability
0.8
0.7
0.6
0.5
0.4
0.3
0.2
0.1
0.0
200
300
400
500
600
m/z

motif_661

fragment_187.1075	: 0.79
fragment_226.0575	: 0.02
fragment_636.3025	: 0.02
loss_189.6625	: 0.01

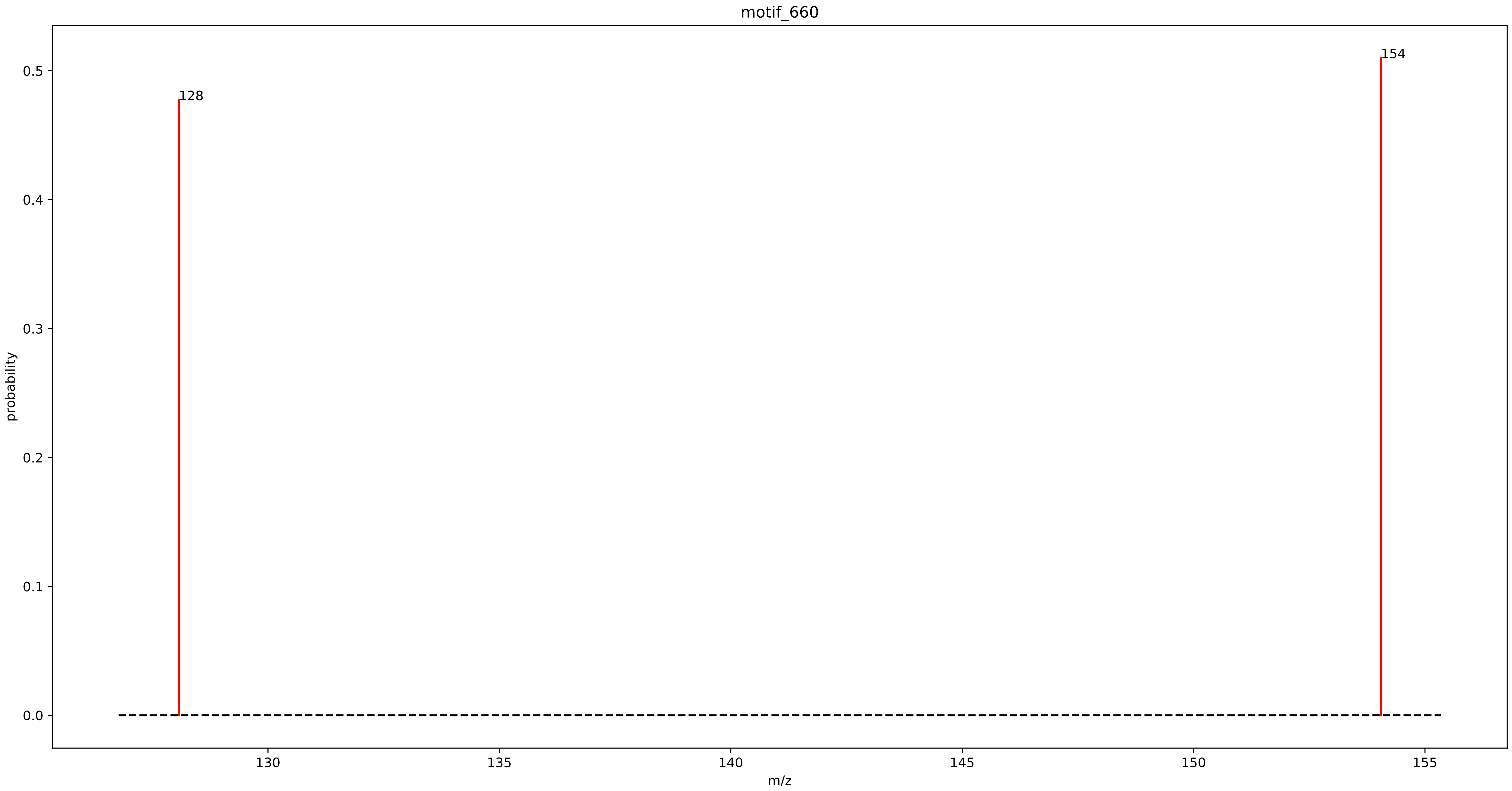

motif_660
128
154
probability
m/z
0.0
0.1
0.2
0.3
0.4
0.5
130
135
140
145
150
155

motif_660

fragment_154.0475	: 0.51
fragment_128.0725	: 0.48

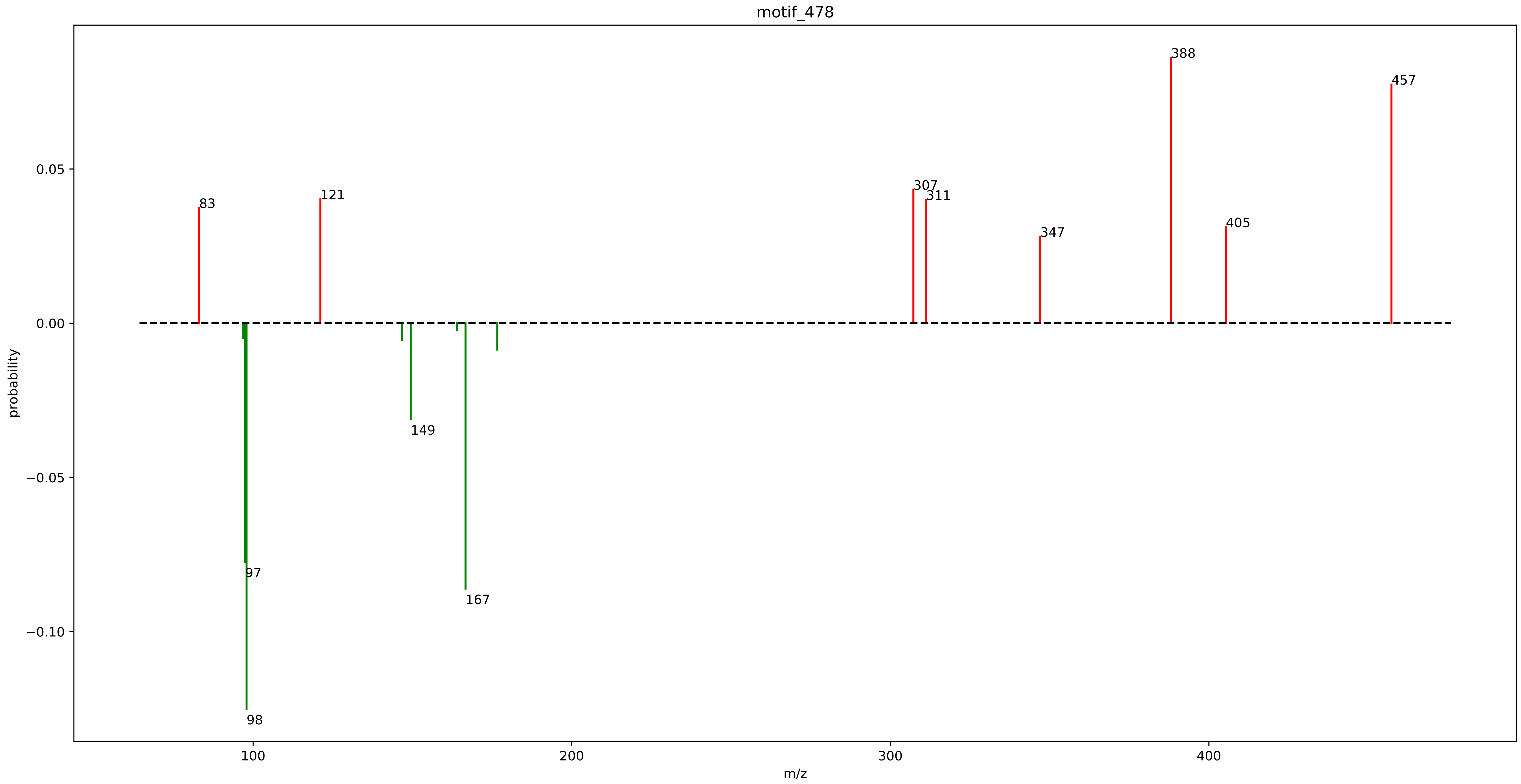
motif_478
probability
m/z
0.05
0.00
−0.05
−0.10
100
200
300
400
83
121
307
311
347
388
405
457
97
98
149
167

motif_478

loss_97.9375	: 0.13
loss_166.6675	: 0.09
fragment_388.1075	: 0.09
loss_97.4825	: 0.08
fragment_457.2975	: 0.08
fragment_307.2425	: 0.04
fragment_121.0975	: 0.04
fragment_311.2725	: 0.04
fragment_83.0475	: 0.04
loss_149.4625	: 0.03
fragment_405.3175	: 0.03
fragment_347.0825	: 0.03
loss_176.6475	: 0.01
loss_146.6375	: 0.01

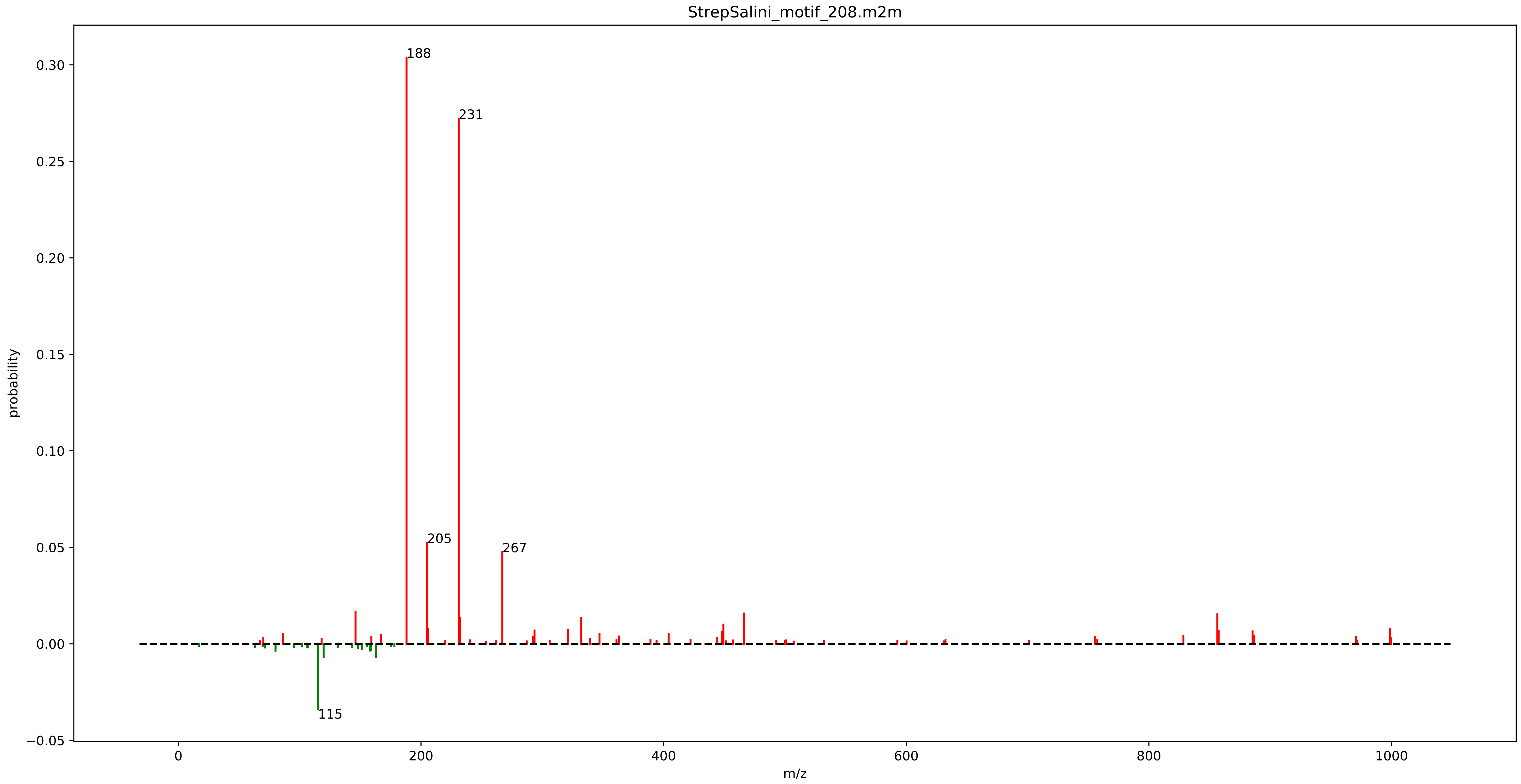

StrepSalini_motif_208.m2m
probability
m/z
188
231
205
267
115
0.30
0.25
0.20
0.15
0.10
0.05
0.00
−0.05
0
200
400
600
800
1000

StrepSalini_motif_208.m2m

fragment_188.0500	: 0.30
fragment_231.0500	: 0.27
fragment_205.0500	: 0.05
fragment_267.0500	: 0.05
loss_115.0500	: 0.03
fragment_146.0500	: 0.02
fragment_466.1500	: 0.02
fragment_856.4500	: 0.02
fragment_232.0500	: 0.01
fragment_332.1500	: 0.01
fragment_449.2500	: 0.01
fragment_998.5500	: 0.01
fragment_206.0500	: 0.01
fragment_232.1500	: 0.01
fragment_321.0500	: 0.01
fragment_293.5500	: 0.01
loss_119.7500	: 0.01
fragment_857.4500	: 0.01
loss_163.1500	: 0.01
fragment_885.4525	: 0.01
fragment_448.1500	: 0.01
fragment_404.1500	: 0.01
fragment_347.1500	: 0.01

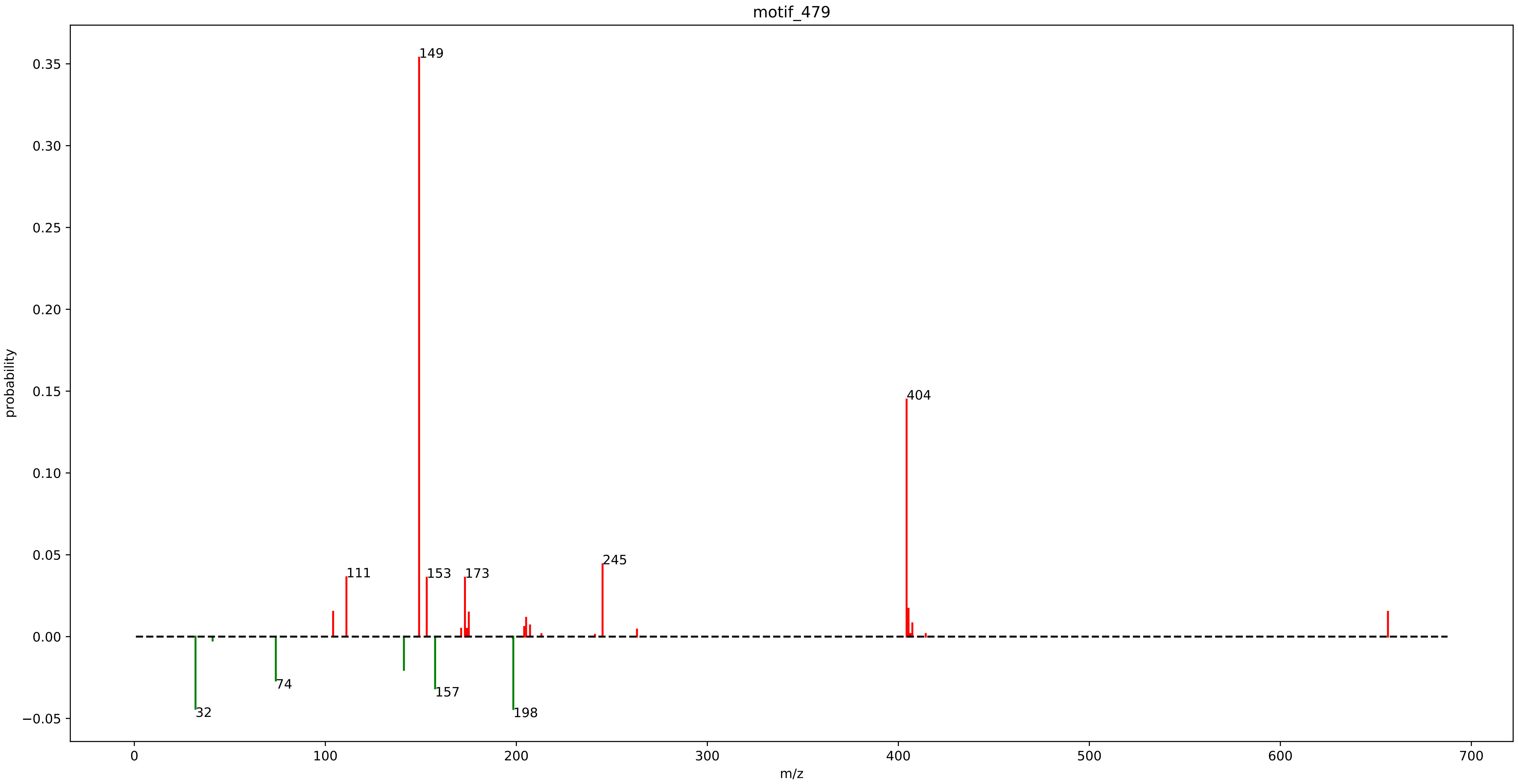

motif_479
probability
m/z
0.35
0.30
0.25
0.20
0.15
0.10
0.05
0.00
−0.05
0
100
200
300
400
500
600
700
149
404
111
153
173
245
32
74
157
198

motif_479

fragment_149.0975	: 0.35
fragment_404.3125	: 0.14
loss_198.4175	: 0.04
fragment_245.1375	: 0.04
loss_32.0275	: 0.04
fragment_111.0425	: 0.04
fragment_153.0875	: 0.04
fragment_173.0975	: 0.04
loss_157.4775	: 0.03
loss_74.0725	: 0.03
loss_141.1425	: 0.02
fragment_405.3175	: 0.02
fragment_656.3225	: 0.02
fragment_104.0725	: 0.02
fragment_175.1125	: 0.01
fragment_205.1225	: 0.01
fragment_407.3275	: 0.01
fragment_207.1725	: 0.01
fragment_204.1075	: 0.01

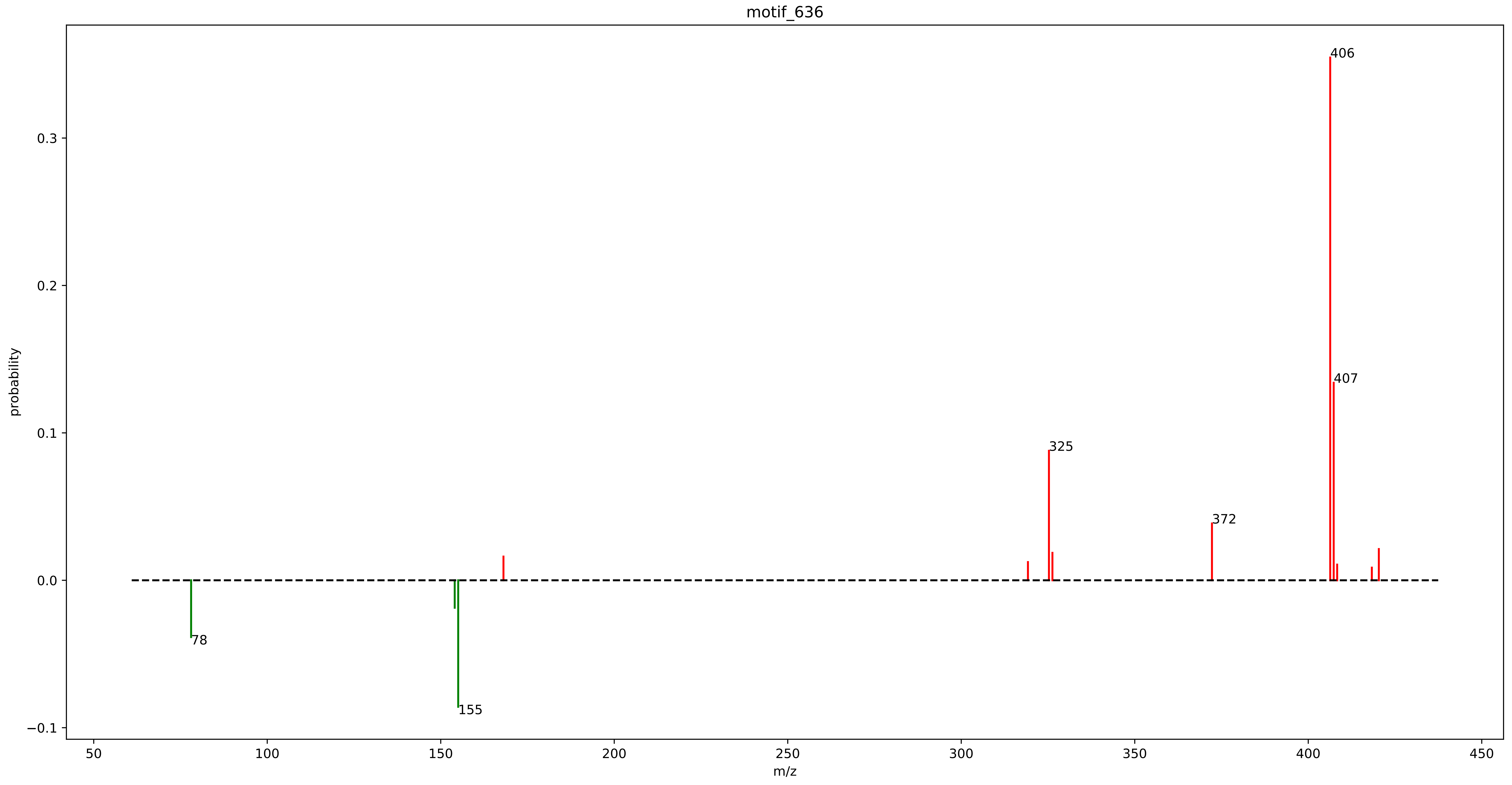

motif_636
probability
m/z
406
407
325
372
78
155
0.3
0.2
0.1
0.0
−0.1
50
100
150
200
250
300
350
400
450

motif_636

fragment_406.3275	: 0.35
fragment_407.3325	: 0.13
fragment_325.2725	: 0.09
loss_155.0325	: 0.09
fragment_372.2525	: 0.04
loss_78.0625	: 0.04
fragment_420.3425	: 0.02
fragment_326.2775	: 0.02
loss_154.0275	: 0.02
fragment_168.0775	: 0.02
fragment_319.2225	: 0.01
fragment_408.3325	: 0.01
fragment_418.3275	: 0.01

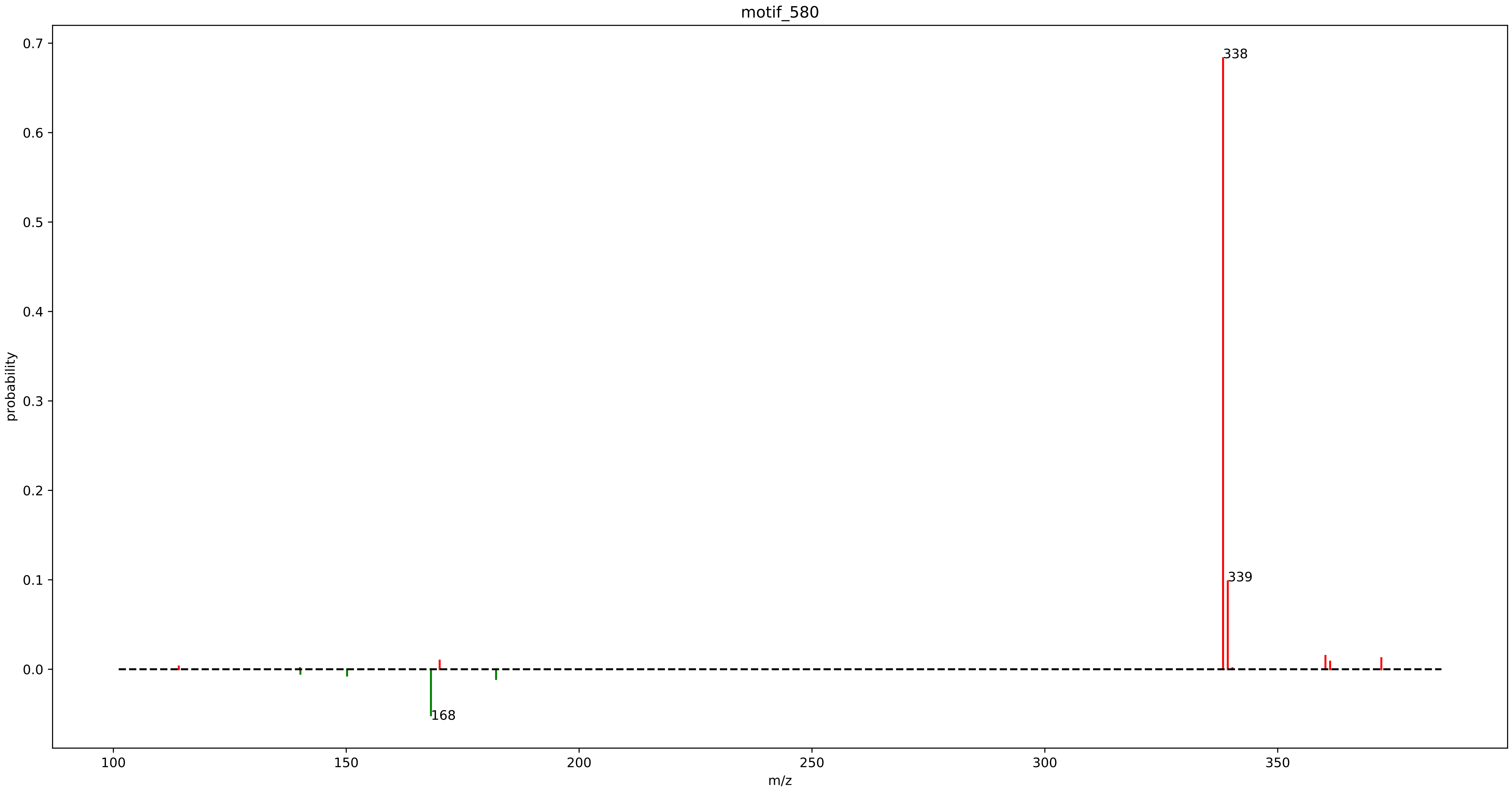
motif_580
probability
m/z
338
339
168
0.7
0.6
0.5
0.4
0.3
0.2
0.1
0.0
100
150
200
250
300
350

motif_580

fragment_338.2675	: 0.68
fragment_339.2725	: 0.10
loss_168.1875	: 0.05
fragment_360.2475	: 0.01
fragment_372.2475	: 0.01
loss_182.1725	: 0.01
fragment_170.0575	: 0.01
fragment_361.2525	: 0.01
loss_150.1775	: 0.01

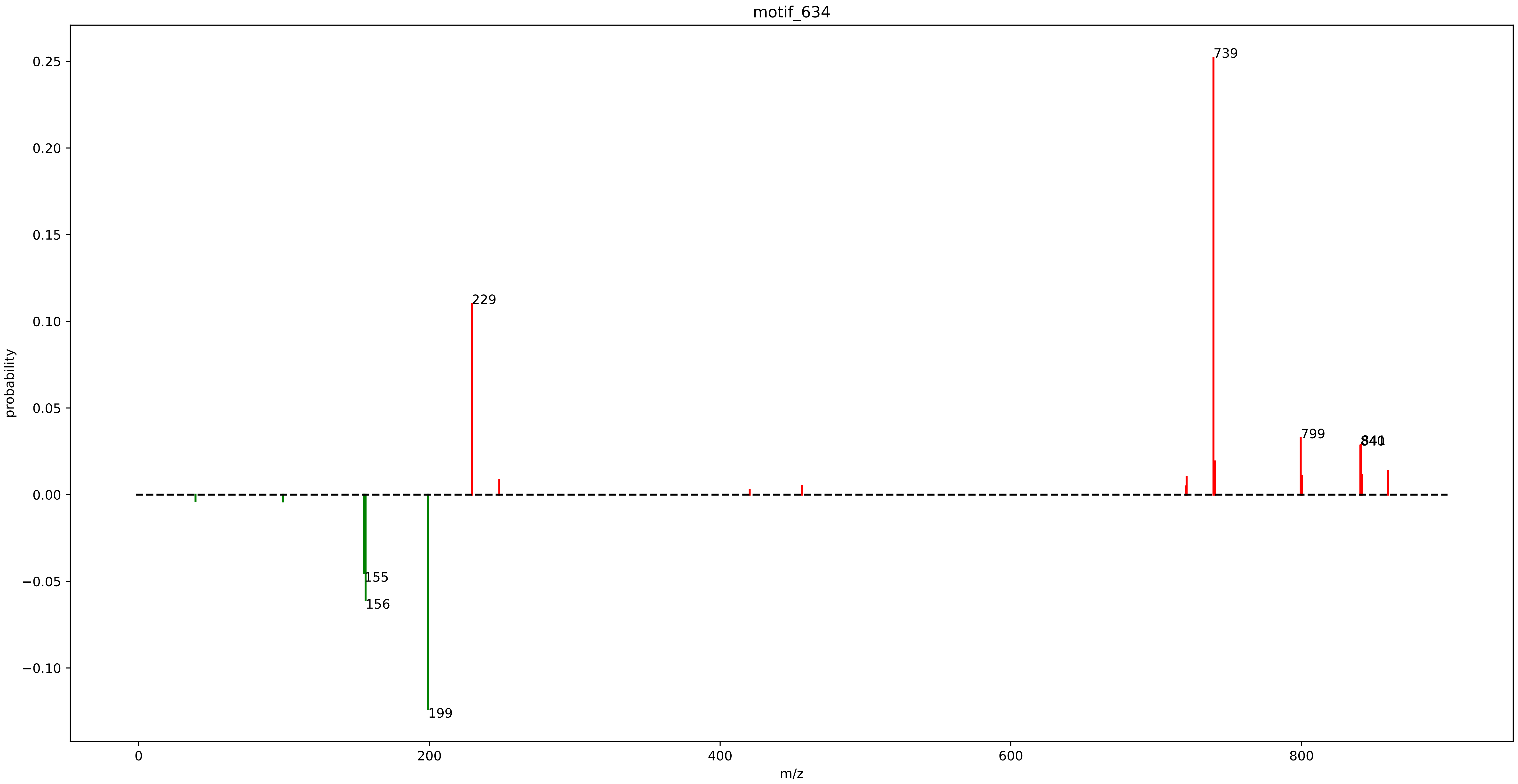
motif_634
probability
m/z
0.25
0.20
0.15
0.10
0.05
0.00
−0.05
−0.10
0
200
400
600
800
739
229
799
840
841
155
156
199

motif_634

fragment_739.3725	: 0.25
loss_199.1025	: 0.12
fragment_229.1325	: 0.11
loss_156.0975	: 0.06
loss_155.1375	: 0.05
fragment_799.3975	: 0.03
fragment_840.9475	: 0.03
fragment_840.4425	: 0.03
fragment_740.3775	: 0.02
fragment_859.4175	: 0.01
fragment_841.4475	: 0.01
fragment_800.3975	: 0.01
fragment_720.9025	: 0.01
fragment_248.0825	: 0.01
loss_155.0775	: 0.01
fragment_456.3575	: 0.01

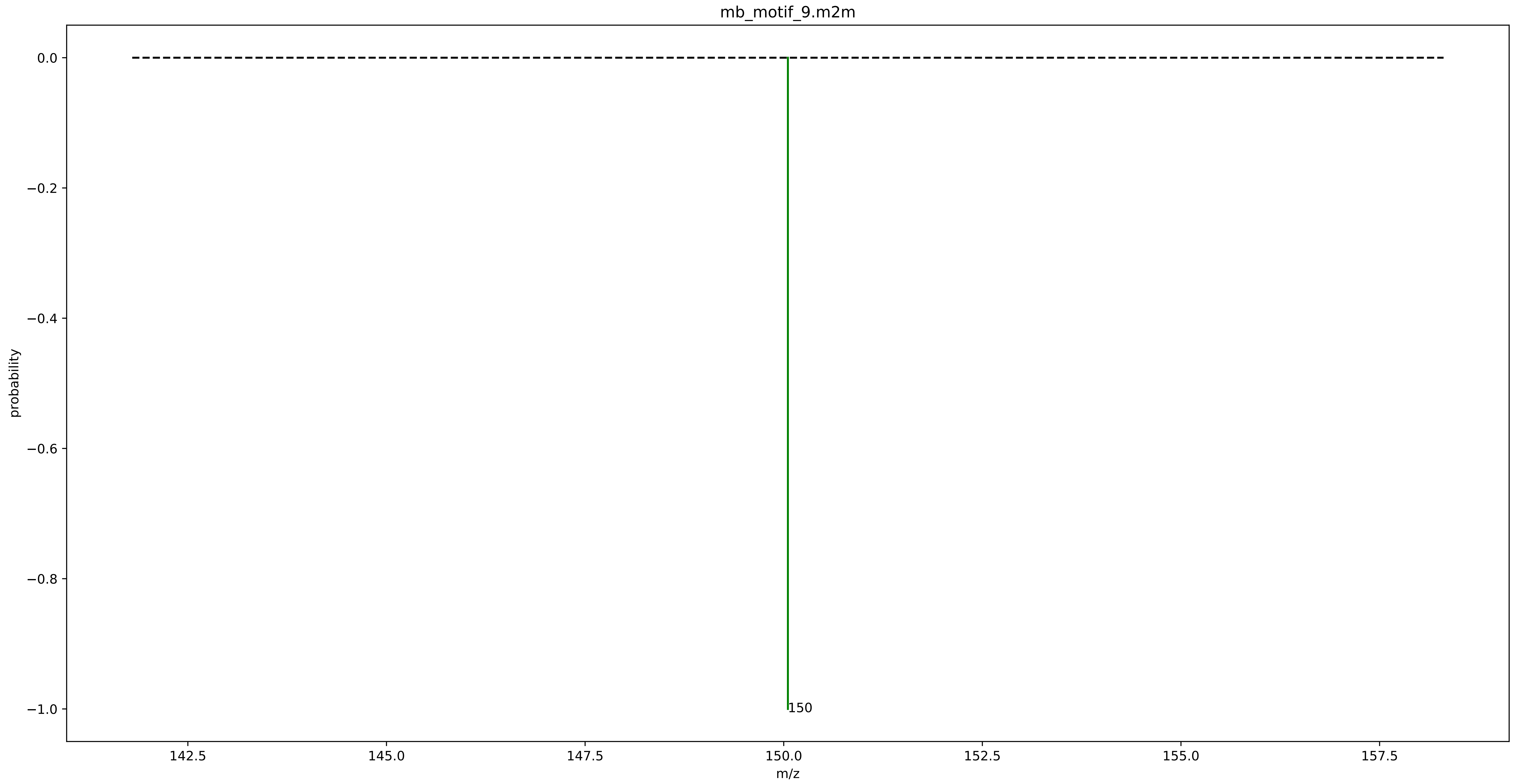

mb_motif_9.m2m
0.0
−0.2
−0.4
−0.6
−0.8
−1.0
probability
150
142.5
145.0
147.5
150.0
152.5
155.0
157.5
m/z

mb_motif_9.m2m

loss_150.0525 : 1.00

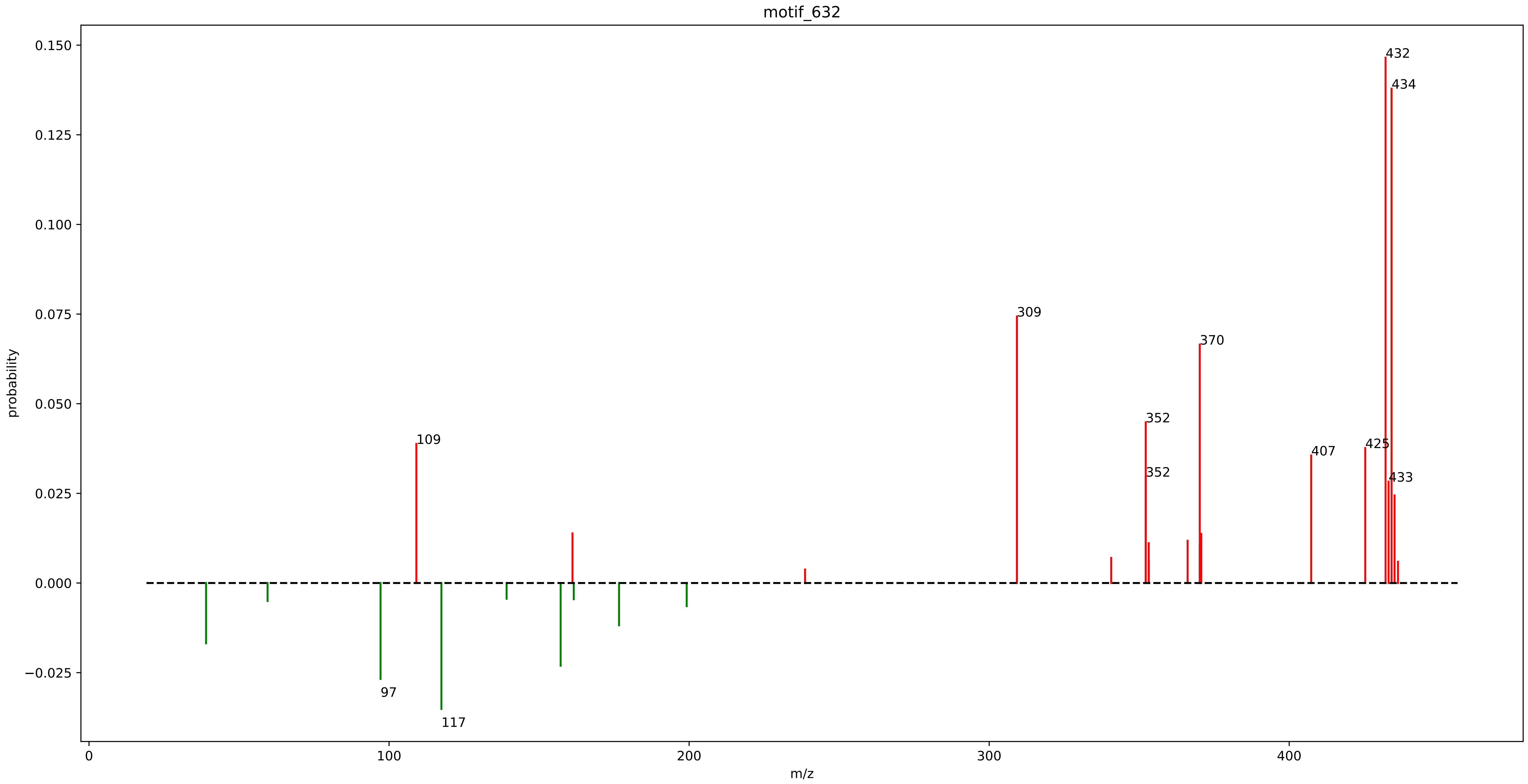

motif_632
probability
m/z
0.150
0.125
0.100
0.075
0.050
0.025
0.000
−0.025
0
100
200
300
400
432
434
309
370
352
352
109
407
425
433
97
117

motif_632

fragment_432.1125	: 0.15
fragment_434.1125	: 0.14
fragment_309.2575	: 0.07
fragment_370.1925	: 0.07
fragment_352.1825	: 0.04
fragment_109.0975	: 0.04
fragment_425.3425	: 0.04
fragment_407.3275	: 0.04
loss_117.4475	: 0.04
fragment_352.1875	: 0.03
fragment_433.1175	: 0.03
loss_97.1625	: 0.03
fragment_435.1125	: 0.02
loss_157.1825	: 0.02
loss_39.0175	: 0.02
fragment_161.1325	: 0.01
fragment_370.6925	: 0.01
loss_176.6475	: 0.01
fragment_366.1425	: 0.01
fragment_353.1825	: 0.01
fragment_370.1875	: 0.01
fragment_340.6825	: 0.01
loss_199.1925	: 0.01
fragment_353.1925	: 0.01
fragment_436.2575	: 0.01
loss_59.5175	: 0.01

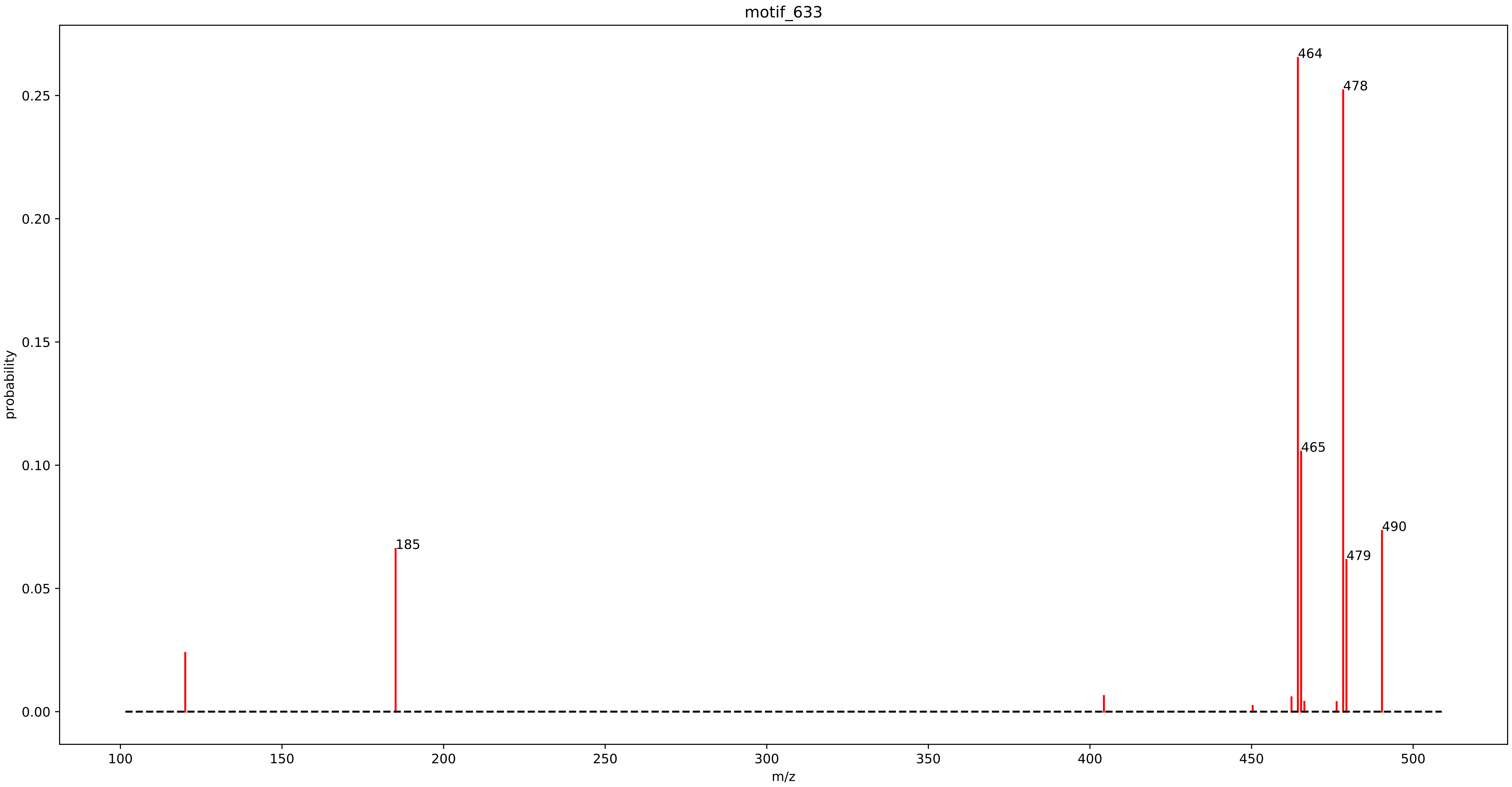

motif_633
probability
m/z
0.00
0.05
0.10
0.15
0.20
0.25
100
150
200
250
300
350
400
450
500
185
464
465
478
479
490

motif_633

fragment_464.3325	: 0.27
fragment_478.3525	: 0.25
fragment_465.3375	: 0.11
fragment_490.3525	: 0.07
fragment_185.1525	: 0.07
fragment_479.3525	: 0.06
fragment_120.0675	: 0.02
fragment_404.3125	: 0.01
fragment_462.3575	: 0.01

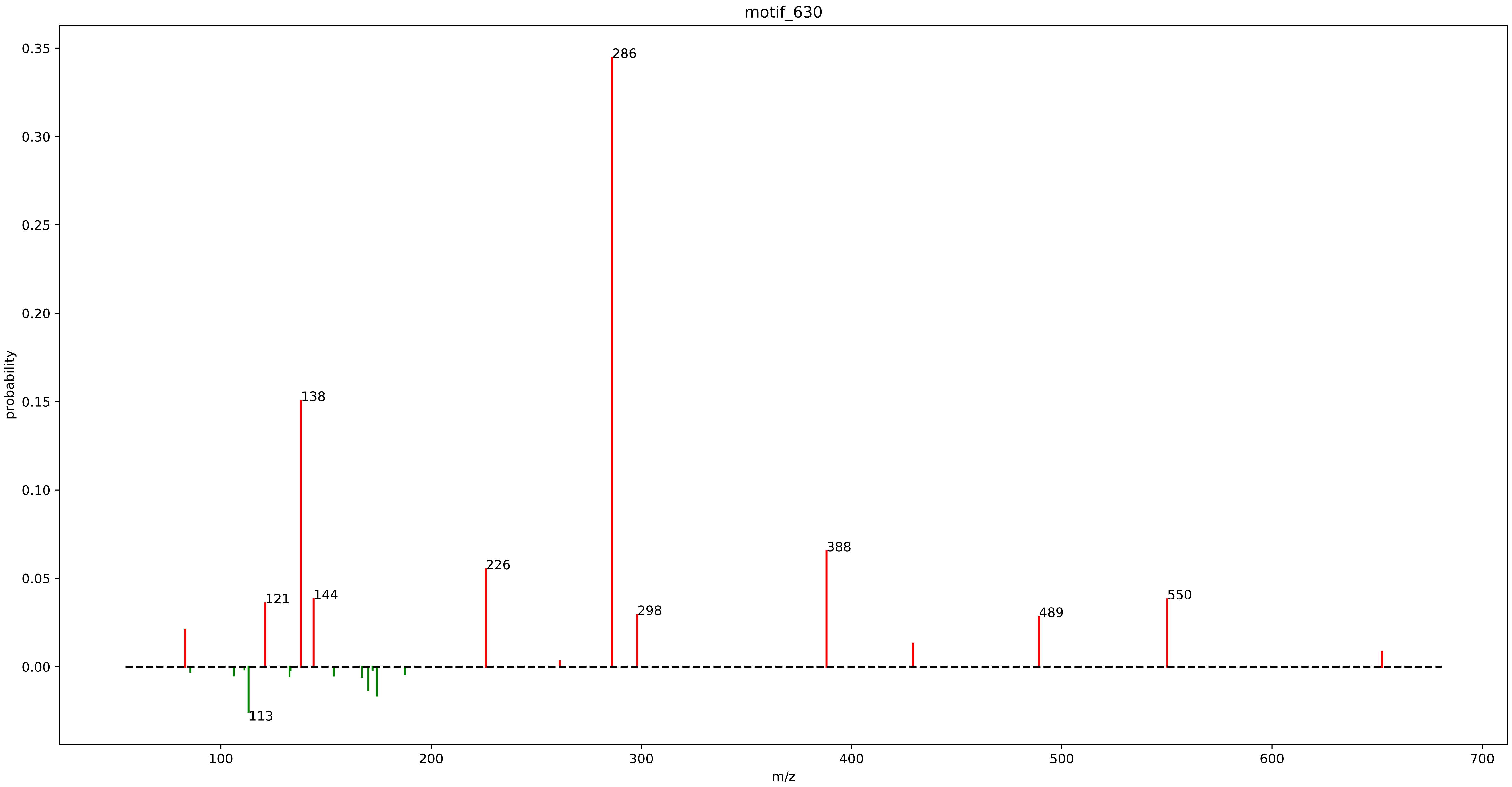

motif_630
probability
m/z
0.35
0.30
0.25
0.20
0.15
0.10
0.05
0.00
100
200
300
400
500
600
700
286
138
388
226
550
144
121
298
489
113

motif_630

fragment_286.0775	: 0.34
fragment_138.0525	: 0.15
fragment_388.1075	: 0.07
fragment_226.0525	: 0.06
fragment_144.0675	: 0.04
fragment_550.1625	: 0.04
fragment_121.1025	: 0.04
fragment_298.0775	: 0.03
fragment_489.1575	: 0.03
loss_113.1625	: 0.03
fragment_83.0475	: 0.02
loss_174.1675	: 0.02
loss_170.1375	: 0.01
fragment_429.1375	: 0.01
fragment_652.2975	: 0.01
loss_167.1575	: 0.01
loss_132.6425	: 0.01

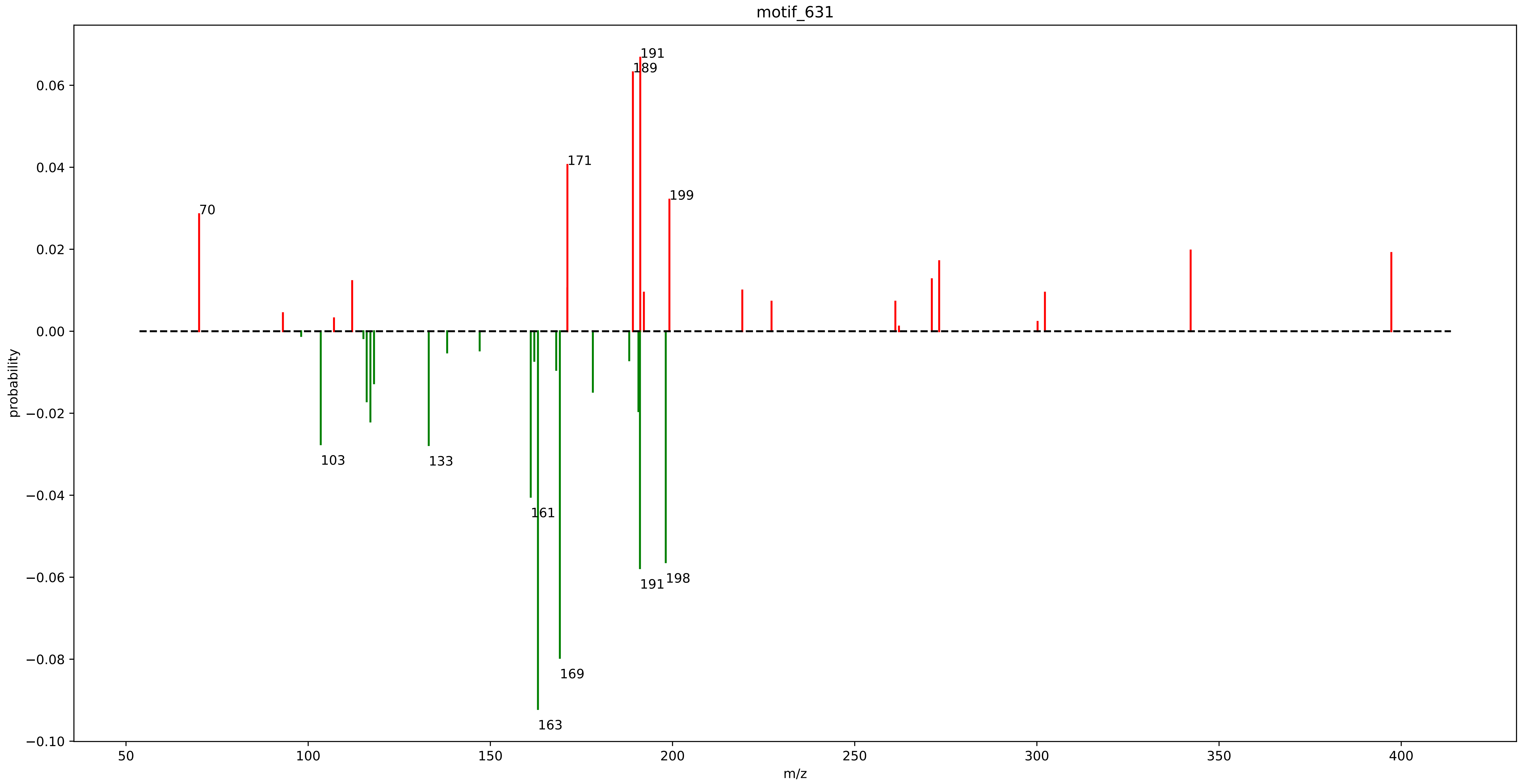

motif_631
probability
m/z
0.06
0.04
0.02
0.00
−0.02
−0.04
−0.06
−0.08
−0.10
50
100
150
200
250
300
350
400
191
189
171
199
70
103
133
161
163
169
191
198

motif_631

loss_163.0625	: 0.09
loss_169.0725	: 0.08
fragment_191.1425	: 0.07
fragment_189.1225	: 0.06
loss_191.0575	: 0.06
loss_198.1375	: 0.06
fragment_171.1475	: 0.04
loss_161.0825	: 0.04
fragment_199.1425	: 0.03
fragment_70.0625	: 0.03
loss_133.0875	: 0.03
loss_103.4475	: 0.03
loss_117.0775	: 0.02
fragment_342.2025	: 0.02
loss_190.6425	: 0.02
fragment_397.2825	: 0.02
loss_116.0575	: 0.02
fragment_273.1825	: 0.02
loss_178.1325	: 0.01
loss_118.0725	: 0.01
fragment_271.1675	: 0.01
fragment_112.0725	: 0.01
fragment_171.1275	: 0.01
fragment_219.1475	: 0.01
fragment_192.1375	: 0.01
loss_168.0775	: 0.01
fragment_302.2075	: 0.01
loss_162.0625	: 0.01
fragment_227.1725	: 0.01
fragment_261.1575	: 0.01
loss_188.1175	: 0.01
loss_138.1425	: 0.01

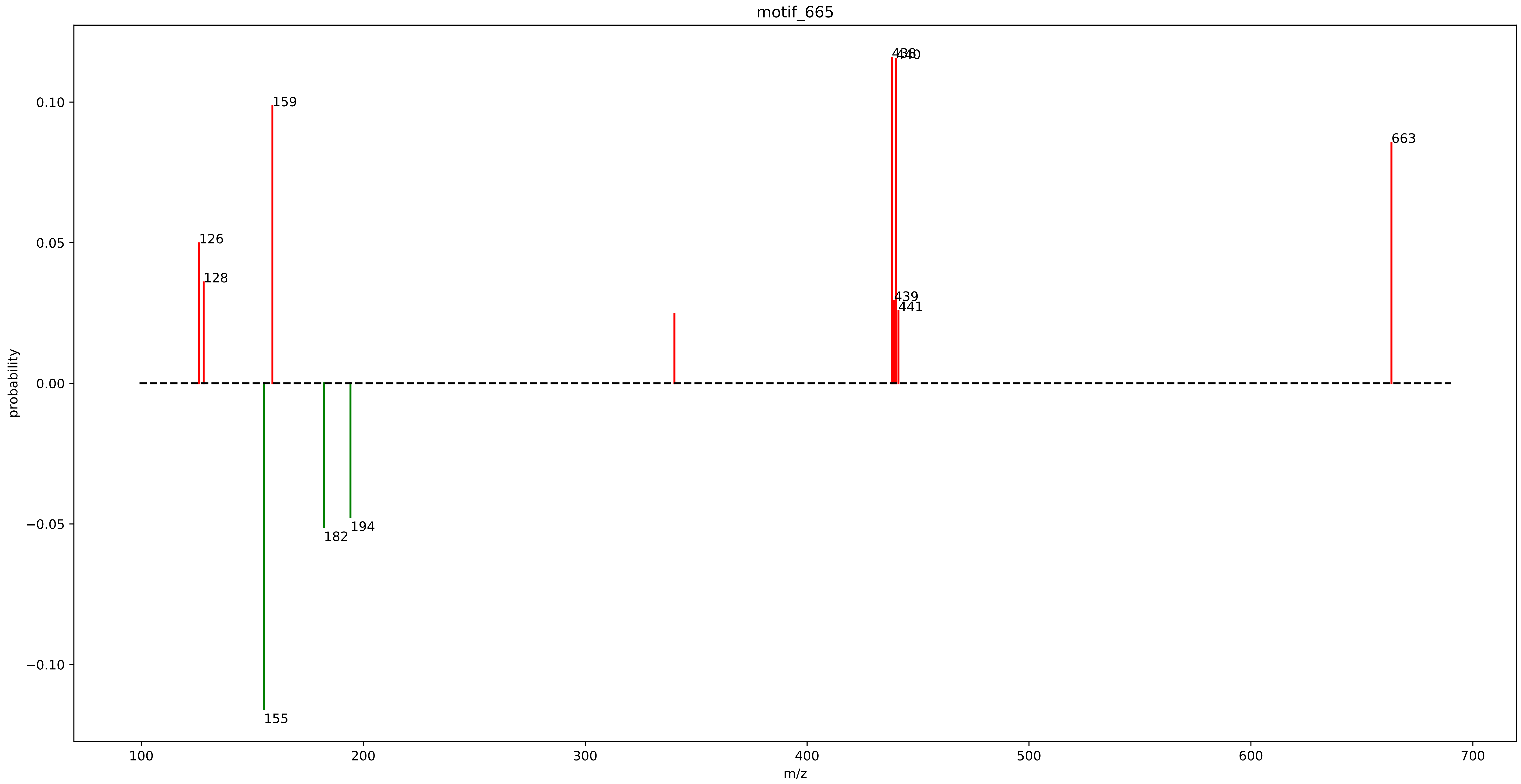

motif_665
126
128
159
438
440
439
441
663
155
182
194
0.10
0.05
0.00
−0.05
−0.10
probability
100
200
300
400
500
600
700
m/z

motif_665

fragment_438.1625	: 0.12
loss_155.2325	: 0.12
fragment_440.1625	: 0.12
fragment_159.0925	: 0.10
fragment_663.3125	: 0.09
loss_182.2475	: 0.05
fragment_126.0575	: 0.05
loss_194.2475	: 0.05
fragment_128.0725	: 0.04
fragment_439.1675	: 0.03
fragment_441.1675	: 0.03
fragment_340.2275	: 0.02

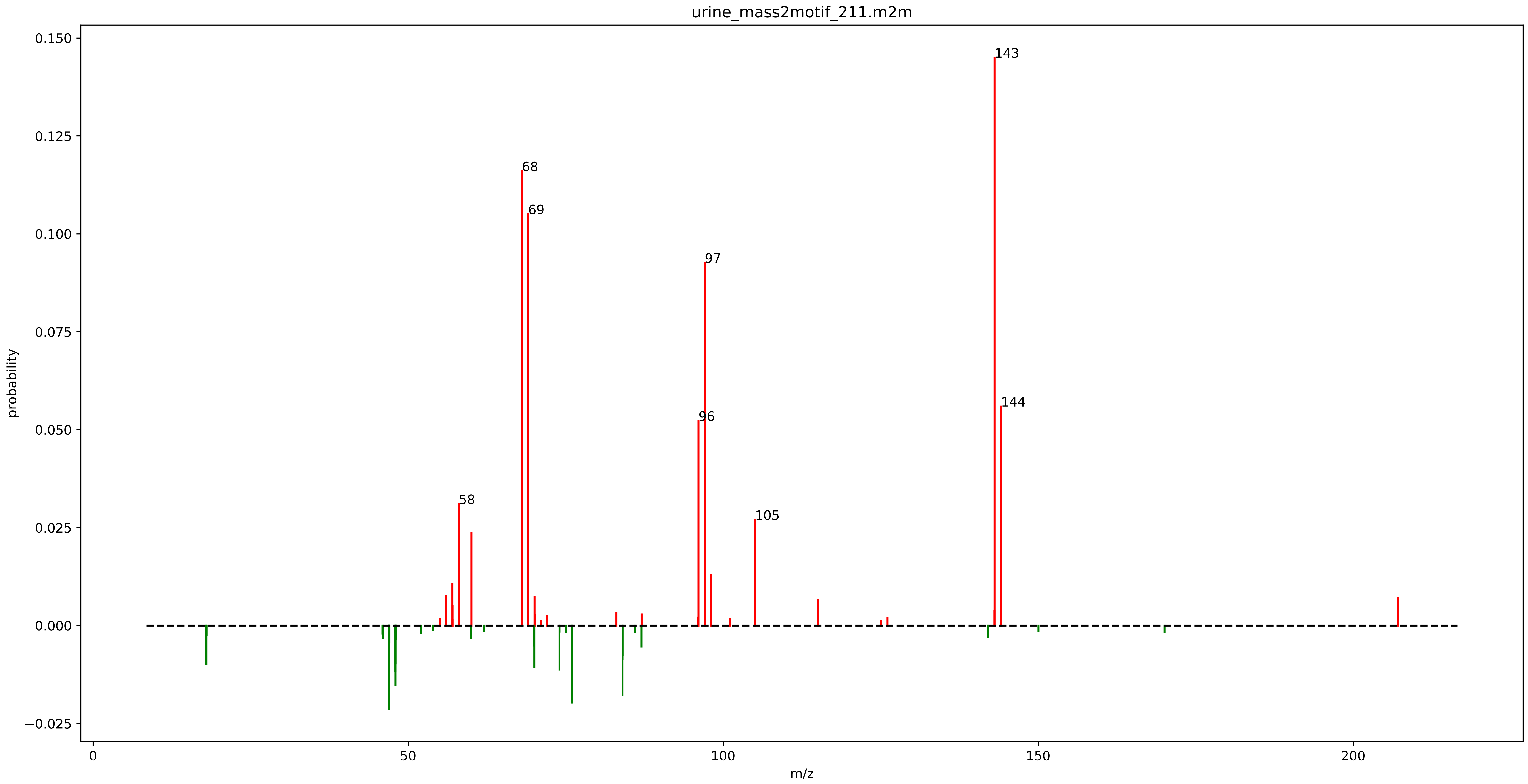

urine_mass2motif_211.m2m
probability
m/z
0.150
0.125
0.100
0.075
0.050
0.025
0.000
−0.025
0
50
100
150
200
143
68
69
97
96
144
58
105

urine_mass2motif_211.m2m

fragment_143.0875	: 0.14
fragment_68.0475	: 0.12
fragment_69.0525	: 0.11
fragment_97.0825	: 0.09
fragment_144.0925	: 0.06
fragment_96.0825	: 0.05
fragment_58.0375	: 0.03
fragment_105.0725	: 0.03
fragment_60.0425	: 0.02
loss_47.0025	: 0.02
loss_76.0325	: 0.02
loss_84.0325	: 0.02
loss_76.0375	: 0.02
loss_48.0075	: 0.02
fragment_98.0875	: 0.01
loss_76.0425	: 0.01
loss_47.9975	: 0.01
loss_74.0225	: 0.01
fragment_57.0325	: 0.01
loss_70.0325	: 0.01
loss_17.9375	: 0.01
loss_18.0025	: 0.01
loss_48.0125	: 0.01
loss_84.0425	: 0.01
fragment_56.0475	: 0.01
loss_84.0525	: 0.01
fragment_70.0575	: 0.01
fragment_207.1075	: 0.01
fragment_115.0575	: 0.01
fragment_69.0475	: 0.01
loss_46.9875	: 0.01
loss_87.0425	: 0.01
loss_70.0175	: 0.01

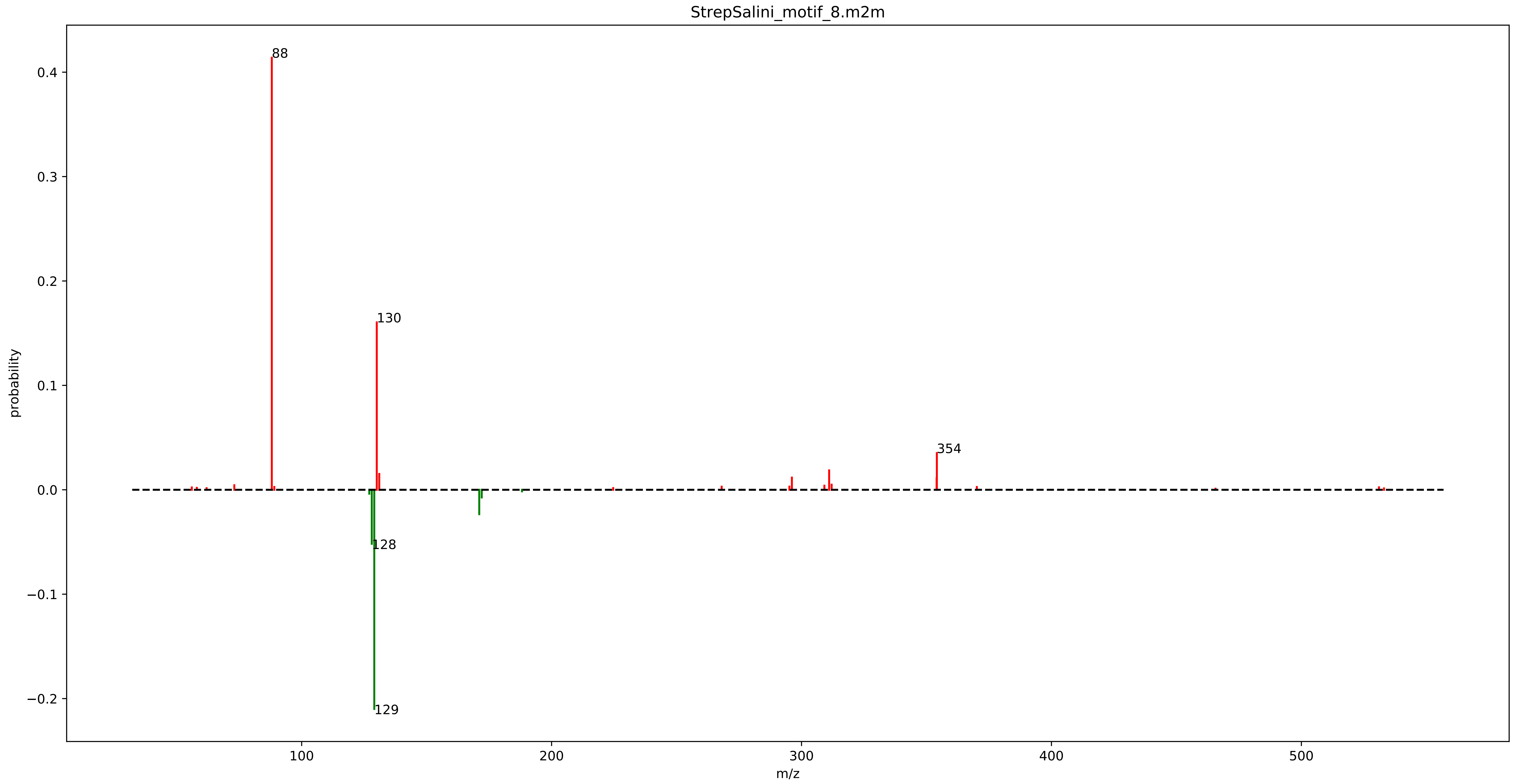

StrepSalini_motif_8.m2m
probability
m/z
0.4
0.3
0.2
0.1
0.0
−0.1
−0.2
100
200
300
400
500
88
130
354
128
129

StrepSalini_motif_8.m2m

fragment_88.0500	: 0.41
loss_129.0525	: 0.21
fragment_130.0500	: 0.16
loss_128.0500	: 0.05
fragment_354.1500	: 0.04
loss_171.0525	: 0.02
fragment_311.0500	: 0.02
fragment_131.0500	: 0.02
fragment_296.1500	: 0.01
fragment_354.0500	: 0.01
loss_172.0500	: 0.01

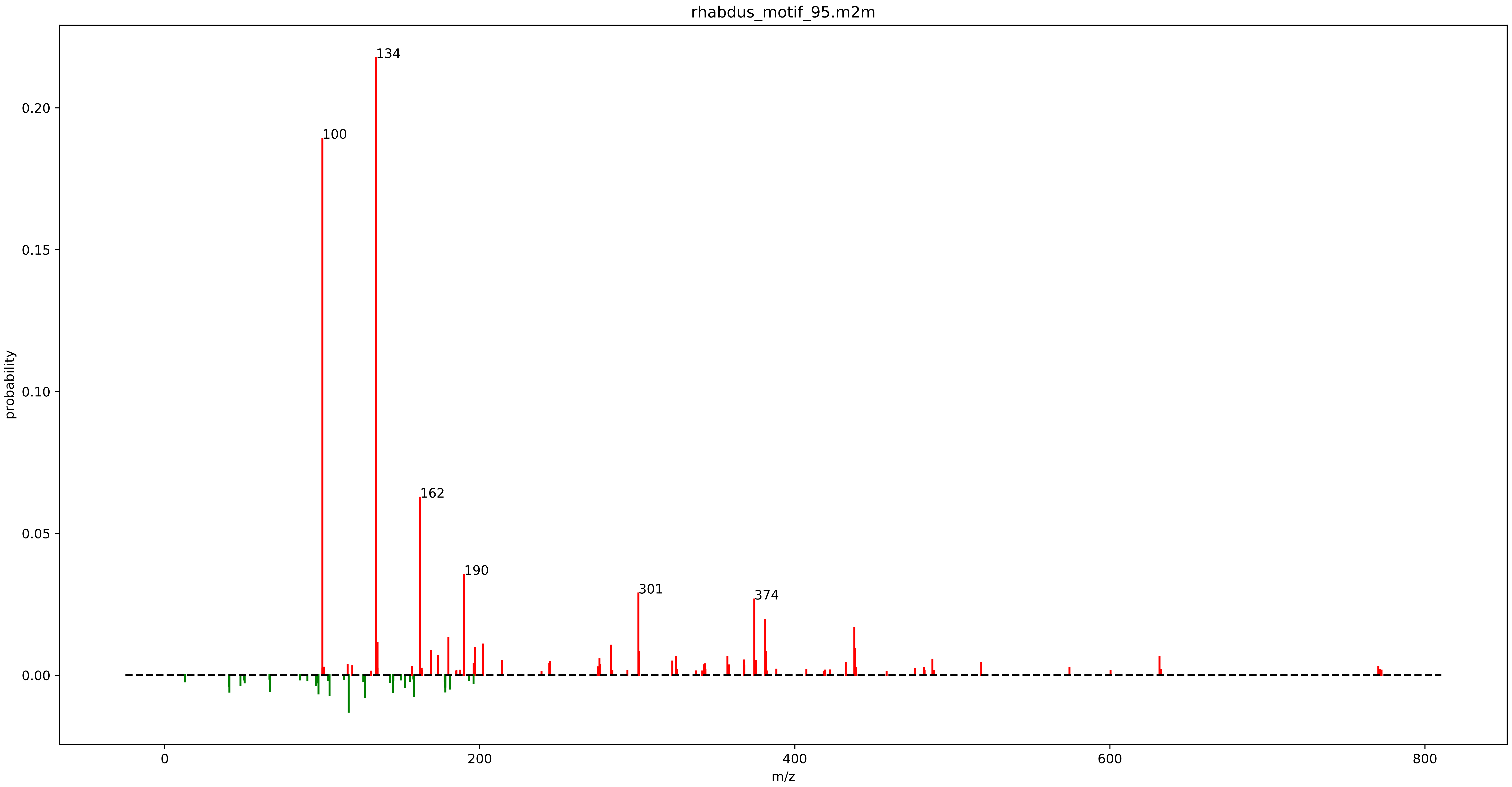

rhabdus_motif_95.m2m
probability
m/z
0.20
0.15
0.10
0.05
0.00
0
200
400
600
800
134
100
162
190
301
374

rhabdus_motif_95.m2m

fragment_134.0975	: 0.22
fragment_100.0725	: 0.19
fragment_162.0925	: 0.06
fragment_190.0875	: 0.04
fragment_300.7075	: 0.03
fragment_374.2425	: 0.03
fragment_381.2525	: 0.02
fragment_437.7925	: 0.02
fragment_180.0675	: 0.01
loss_116.7725	: 0.01
fragment_135.0975	: 0.01
fragment_202.1925	: 0.01
fragment_283.1775	: 0.01
fragment_197.0925	: 0.01
fragment_438.2975	: 0.01
fragment_169.0975	: 0.01
fragment_381.7525	: 0.01
fragment_301.2125	: 0.01
loss_127.0975	: 0.01
loss_158.1075	: 0.01
loss_104.6125	: 0.01
fragment_173.6275	: 0.01
fragment_631.4675	: 0.01
fragment_357.2125	: 0.01
fragment_324.7075	: 0.01
loss_97.6025	: 0.01
loss_144.7725	: 0.01
loss_41.0625	: 0.01
loss_178.1425	: 0.01
loss_66.9575	: 0.01
fragment_275.9575	: 0.01
fragment_487.3275	: 0.01
fragment_367.6075	: 0.01
fragment_375.2475	: 0.01

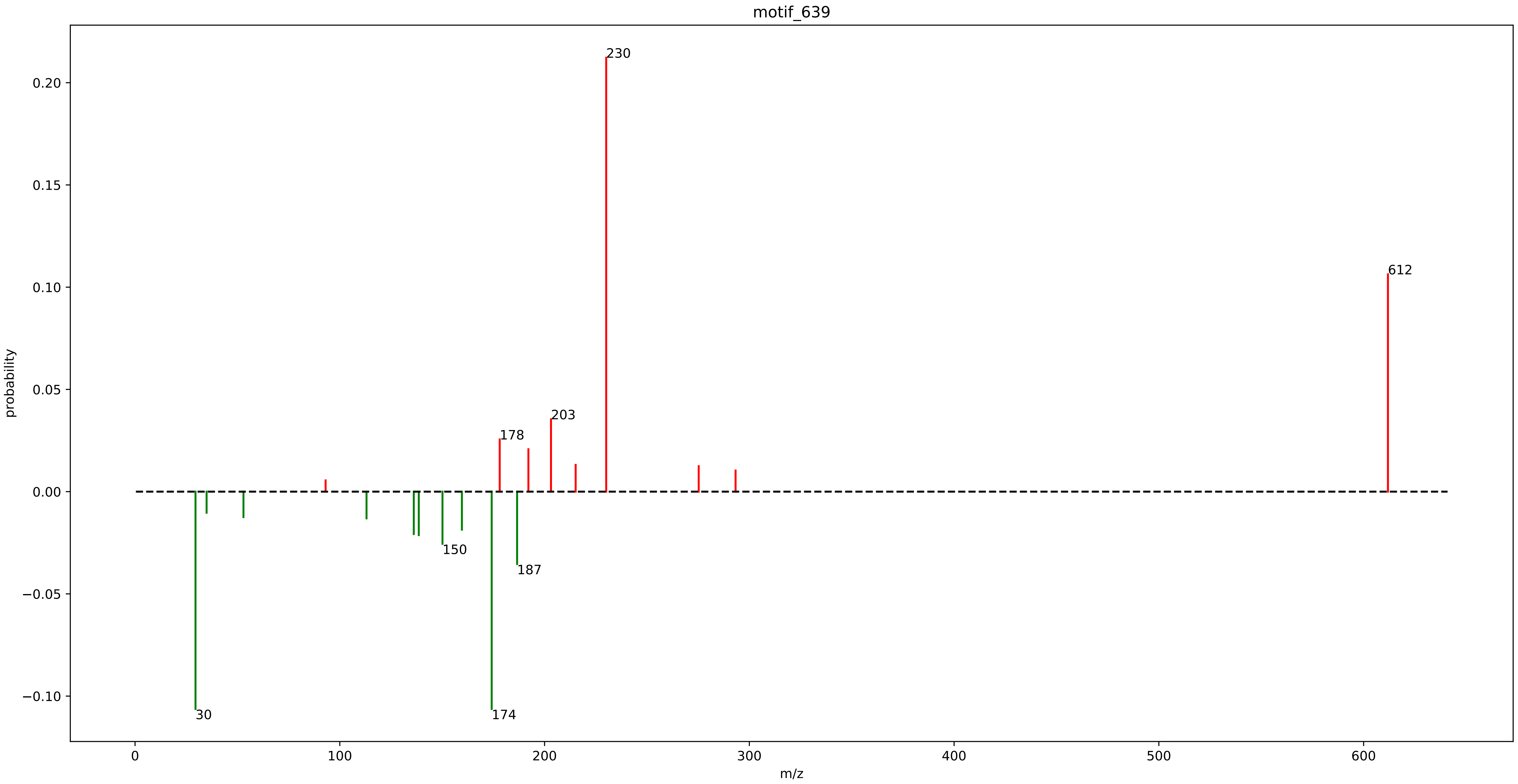

motif_639
probability
m/z
230
612
203
178
150
187
30
174
0.20
0.15
0.10
0.05
0.00
−0.05
−0.10
0
100
200
300
400
500
600

motif_639

fragment_230.0975	: 0.21
loss_174.1775	: 0.11
loss_29.5275	: 0.11
fragment_611.8775	: 0.11
loss_186.5875	: 0.04
fragment_203.1375	: 0.04
loss_150.1425	: 0.03
fragment_178.0875	: 0.03
loss_138.5725	: 0.02
loss_136.1225	: 0.02
fragment_192.1025	: 0.02
loss_159.6275	: 0.02
fragment_215.1775	: 0.01
loss_113.0475	: 0.01
fragment_275.2725	: 0.01
loss_52.9525	: 0.01
loss_34.9425	: 0.01
fragment_293.2825	: 0.01
fragment_93.0675	: 0.01

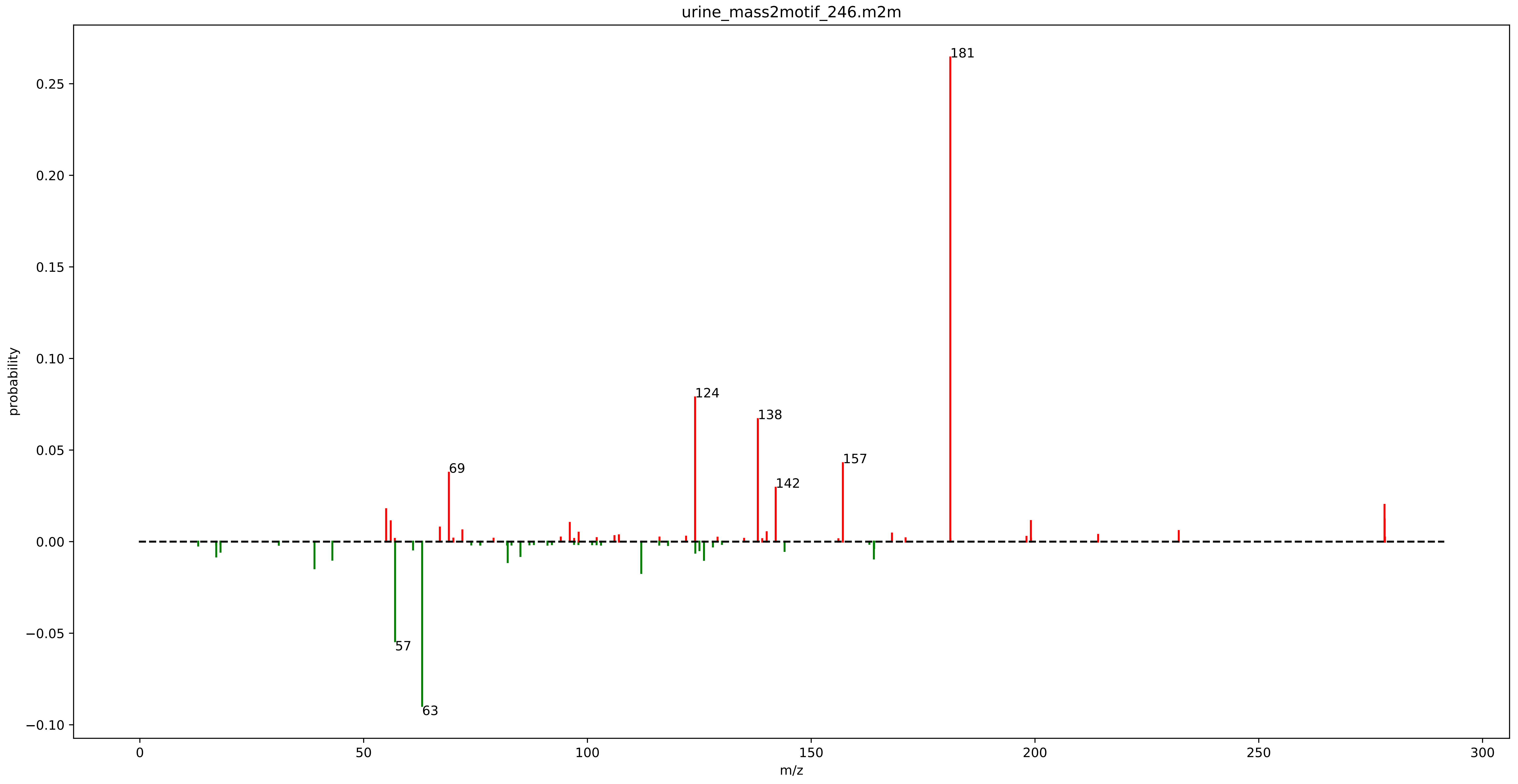

urine_mass2motif_246.m2m
probability
m/z
0.25
0.20
0.15
0.10
0.05
0.00
−0.05
−0.10
0
50
100
150
200
250
300
181
124
138
157
142
69
57
63

urine_mass2motif_246.m2m

fragment_181.0725	: 0.26
loss_63.0675	: 0.09
fragment_124.0525	: 0.08
fragment_138.0675	: 0.07
loss_57.0225	: 0.05
fragment_157.0725	: 0.04
fragment_69.0425	: 0.04
fragment_142.0625	: 0.03
fragment_278.0925	: 0.02
fragment_55.0275	: 0.02
loss_112.0375	: 0.02
loss_112.0275	: 0.02
loss_39.0125	: 0.01
fragment_278.1225	: 0.01
fragment_199.0775	: 0.01
loss_82.1875	: 0.01
fragment_56.0475	: 0.01
fragment_96.0575	: 0.01
loss_126.0425	: 0.01
loss_43.0075	: 0.01
loss_164.0025	: 0.01
loss_17.0675	: 0.01
loss_85.0275	: 0.01
fragment_67.0275	: 0.01
fragment_72.0575	: 0.01
loss_124.1075	: 0.01
fragment_232.1175	: 0.01
loss_85.0175	: 0.01
loss_18.0225	: 0.01
fragment_140.0525	: 0.01
loss_144.0475	: 0.01

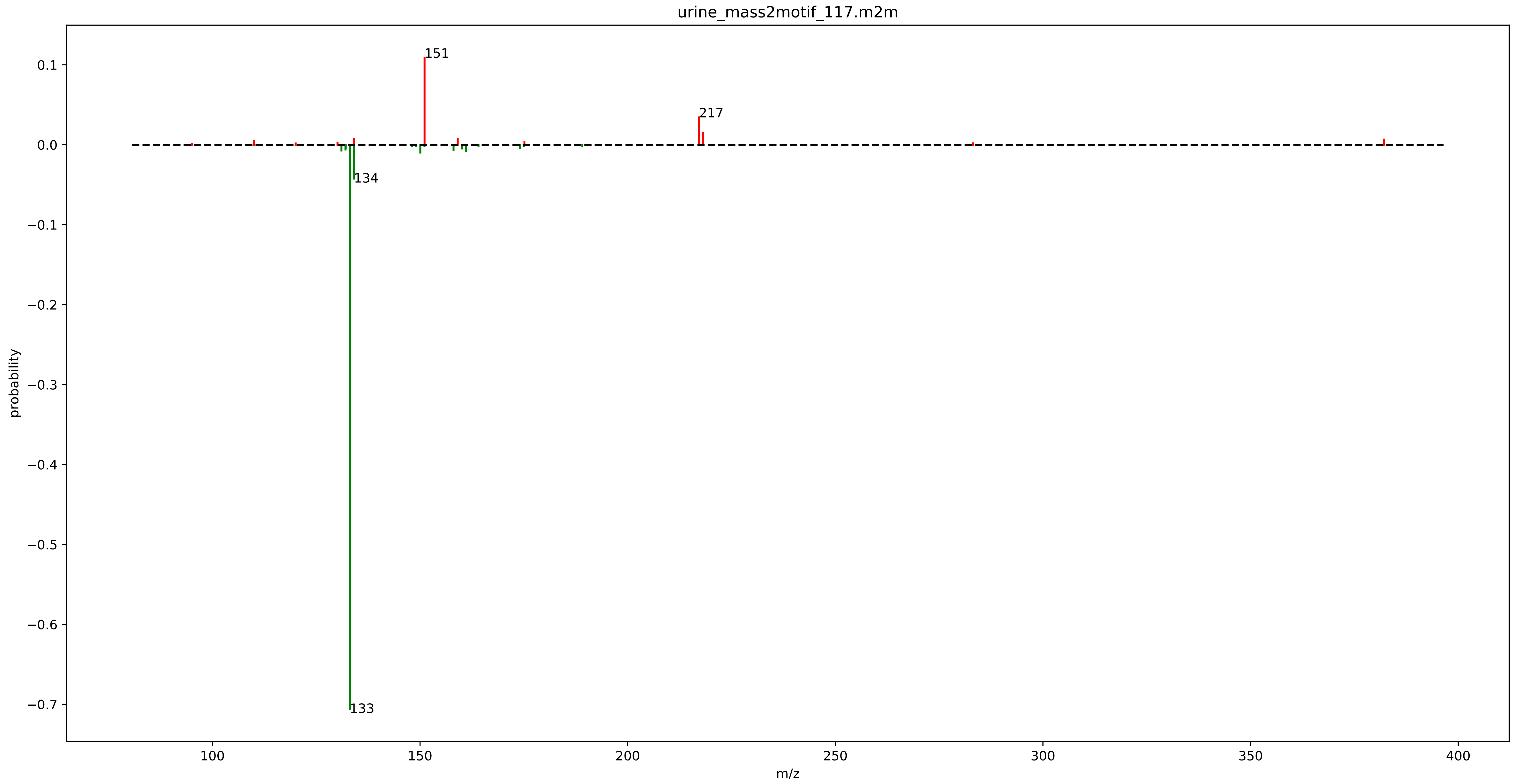

urine_mass2motif_117.m2m
probability
m/z
151
217
134
133
0.1
0.0
−0.1
−0.2
−0.3
−0.4
−0.5
−0.6
−0.7
100
150
200
250
300
350
400

urine_mass2motif_117.m2m

loss_133.0675	: 0.71
fragment_151.0825	: 0.11
loss_134.0625	: 0.04
fragment_217.1575	: 0.03
fragment_218.1375	: 0.01
loss_150.0725	: 0.01
loss_161.0825	: 0.01
fragment_159.0675	: 0.01
fragment_134.0525	: 0.01
loss_131.0675	: 0.01
fragment_382.1175	: 0.01
loss_158.0675	: 0.01
loss_132.0425	: 0.01

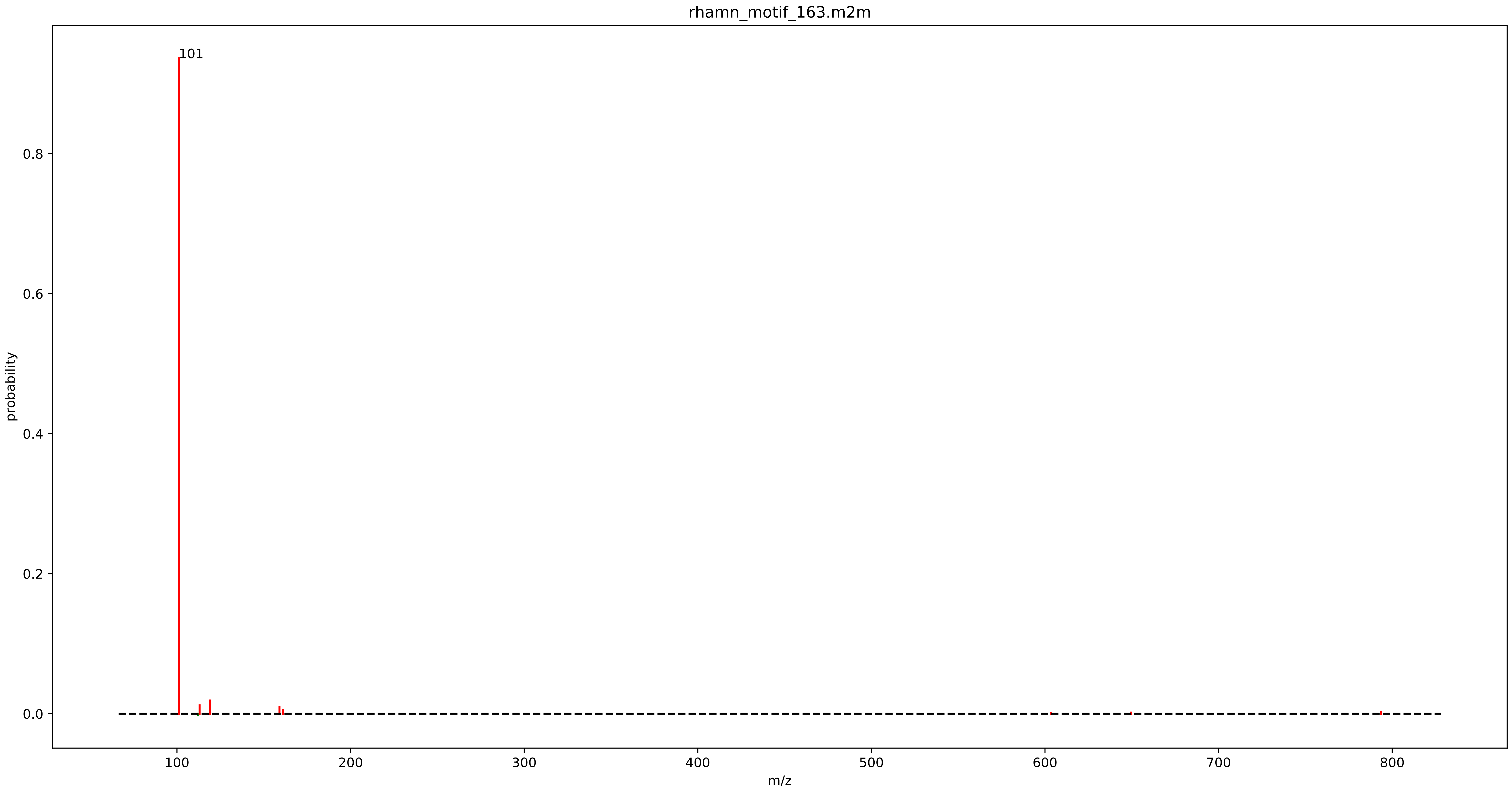
rhamn_motif_163.m2m
101
probability
0.0
0.2
0.4
0.6
0.8
m/z
100
200
300
400
500
600
700
800

rhamn_motif_163.m2m

fragment_101.0225	: 0.94
fragment_119.0325	: 0.02
fragment_113.0175	: 0.01
fragment_159.0275	: 0.01
fragment_119.0375	: 0.01
fragment_161.0375	: 0.01

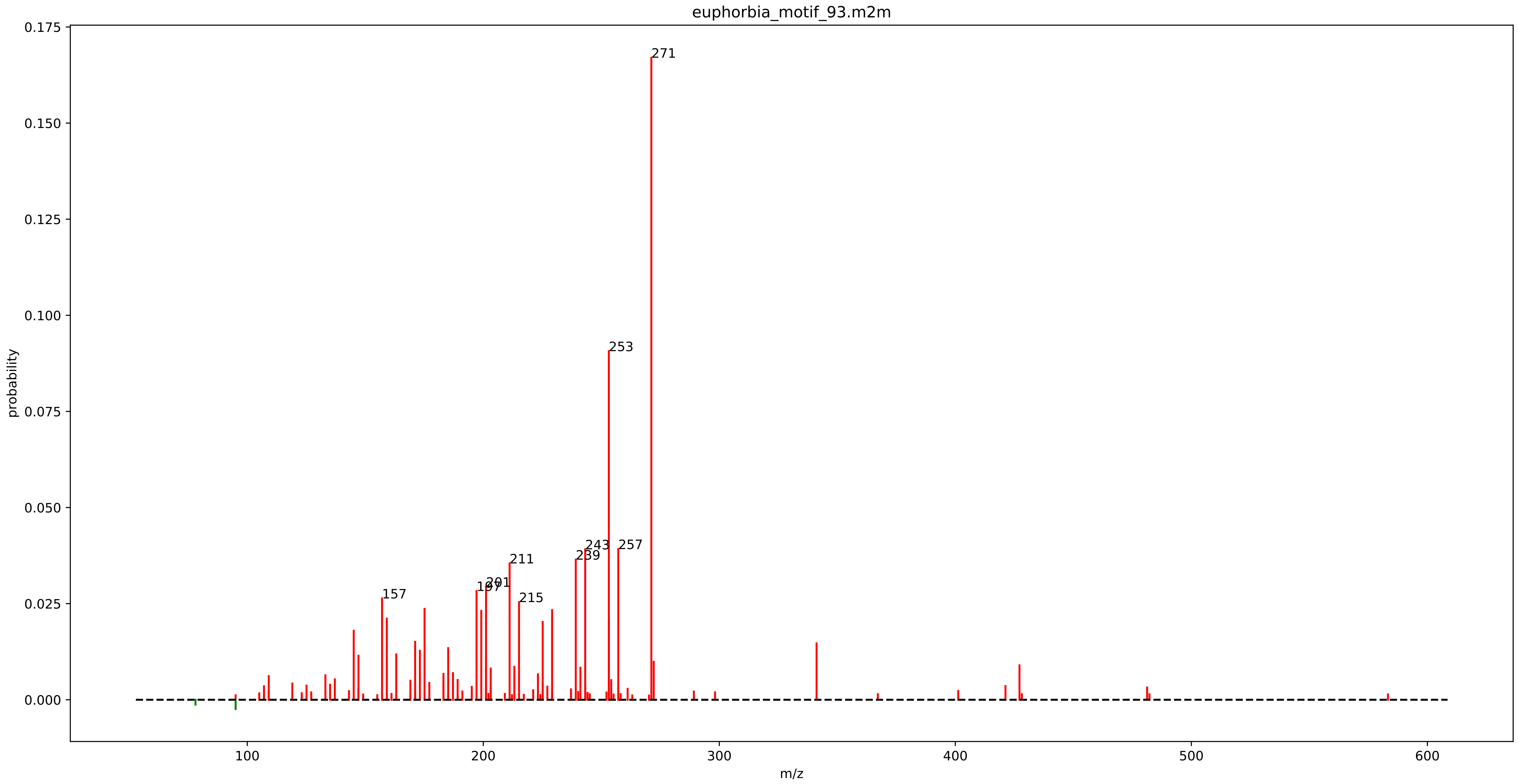

euphorbia_motif_93.m2m
probability
m/z
0.175
0.150
0.125
0.100
0.075
0.050
0.025
0.000
100
200
300
400
500
600
271
253
243
257
239
211
201
197
215
157

euphorbia_motif_93.m2m

fragment_271.2025	: 0.17
fragment_253.1925	: 0.09
fragment_257.1875	: 0.04
fragment_243.1725	: 0.04
fragment_239.1775	: 0.04
fragment_211.1475	: 0.04
fragment_201.1625	: 0.03
fragment_197.1325	: 0.03
fragment_157.1025	: 0.03
fragment_215.1425	: 0.03
fragment_175.1125	: 0.02
fragment_229.1575	: 0.02
fragment_199.1475	: 0.02
fragment_159.1175	: 0.02
fragment_225.1625	: 0.02
fragment_253.1975	: 0.02
fragment_145.0975	: 0.02
fragment_171.1175	: 0.02
fragment_341.2075	: 0.01
fragment_185.1325	: 0.01
fragment_173.1325	: 0.01
fragment_163.1125	: 0.01
fragment_147.1175	: 0.01
fragment_272.2075	: 0.01
fragment_427.1725	: 0.01
fragment_201.1275	: 0.01
fragment_213.1625	: 0.01
fragment_241.1575	: 0.01
fragment_203.1425	: 0.01
fragment_157.0975	: 0.01
fragment_187.1475	: 0.01
fragment_183.1125	: 0.01
fragment_223.1475	: 0.01
fragment_133.1025	: 0.01
fragment_109.1025	: 0.01
fragment_213.1675	: 0.01
fragment_137.0925	: 0.01
fragment_189.1275	: 0.01
fragment_254.1975	: 0.01

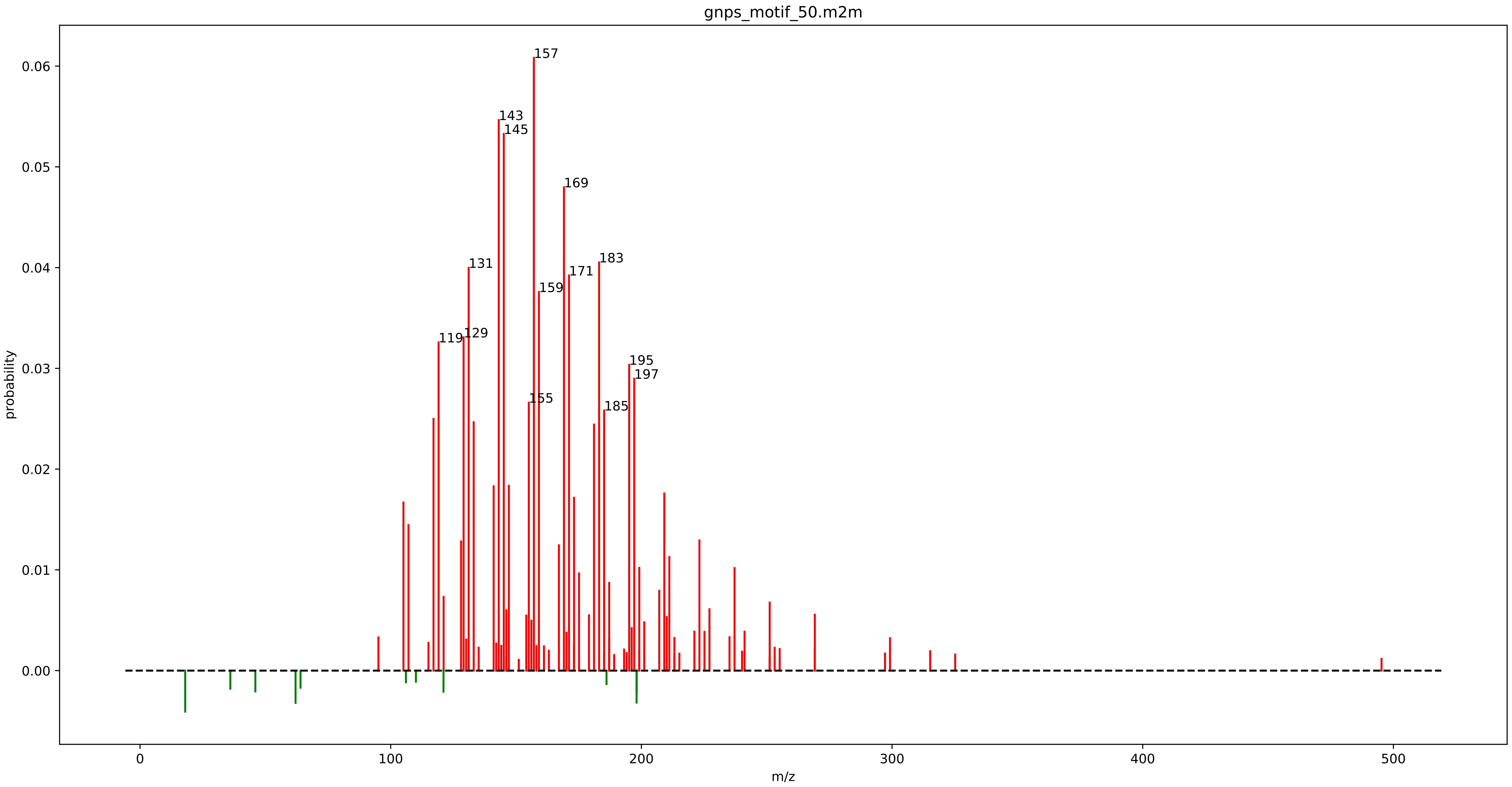

gnps_motif_50.m2m
probability
m/z
0.06
0.05
0.04
0.03
0.02
0.01
0.00
0
100
200
300
400
500
157
143
145
169
183
131
171
159
119
129
195
197
155
185

gnps_motif_50.m2m

fragment_157.1025	: 0.06
fragment_143.0875	: 0.05
fragment_145.1025	: 0.05
fragment_169.1025	: 0.05
fragment_183.1175	: 0.04
fragment_131.0875	: 0.04
fragment_171.1175	: 0.04
fragment_159.1175	: 0.04
fragment_129.0675	: 0.03
fragment_119.0875	: 0.03
fragment_195.1175	: 0.03
fragment_197.1325	: 0.03
fragment_155.0875	: 0.03
fragment_185.1325	: 0.03
fragment_117.0675	: 0.02
fragment_133.1025	: 0.02
fragment_181.1025	: 0.02
fragment_147.1175	: 0.02
fragment_141.0675	: 0.02
fragment_209.1325	: 0.02
fragment_173.1325	: 0.02
fragment_105.0675	: 0.02
fragment_107.0875	: 0.01
fragment_223.1475	: 0.01
fragment_128.0625	: 0.01
fragment_167.0875	: 0.01
fragment_211.1475	: 0.01
fragment_199.1475	: 0.01
fragment_237.1625	: 0.01
fragment_175.1125	: 0.01
fragment_187.1475	: 0.01
fragment_207.1175	: 0.01
fragment_121.1025	: 0.01
fragment_251.1775	: 0.01
fragment_227.1425	: 0.01
fragment_146.1025	: 0.01
fragment_269.1875	: 0.01
fragment_179.0875	: 0.01
fragment_154.0775	: 0.01
fragment_210.1375	: 0.01

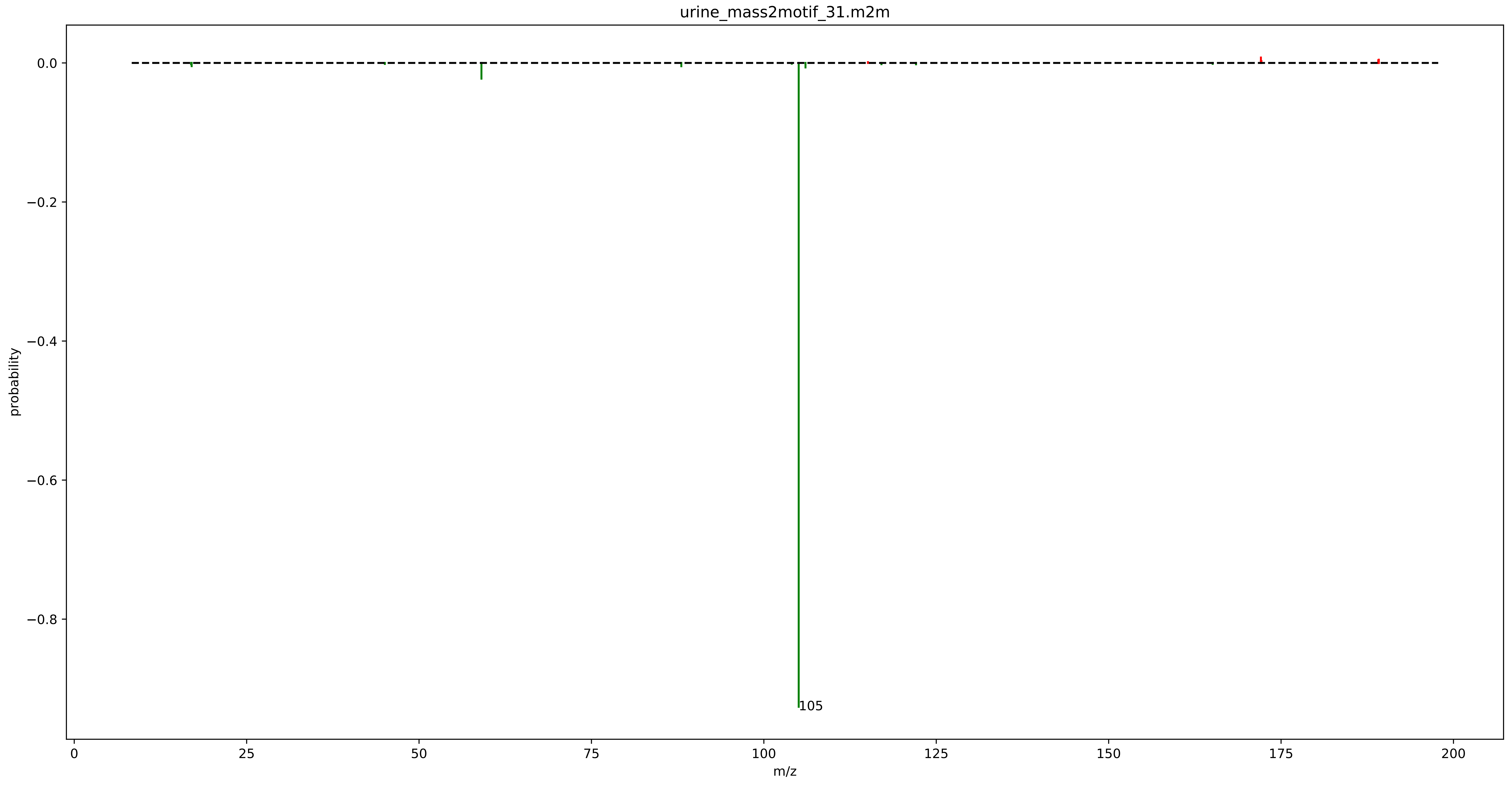
urine_mass2motif_31.m2m
0.0
−0.2
−0.4
−0.6
−0.8
probability
105
0
25
50
75
100
125
150
175
200
m/z

urine_mass2motif_31.m2m

loss_105.0525	: 0.93
loss_59.0475	: 0.02
fragment_172.0775	: 0.01
loss_106.0375	: 0.01

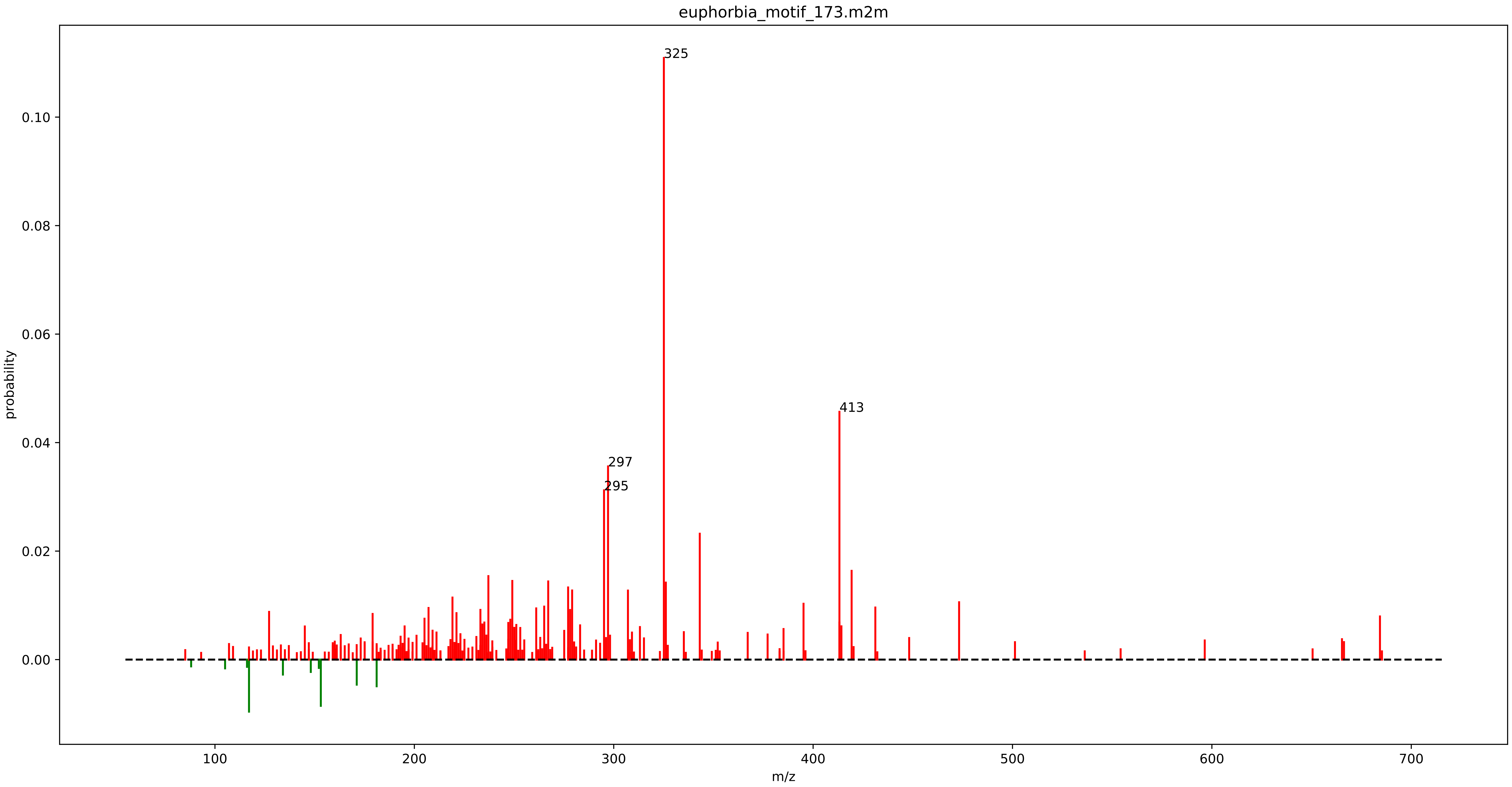

euphorbia_motif_173.m2m
probability
m/z
325
413
297
295
0.00
0.02
0.04
0.06
0.08
0.10
100
200
300
400
500
600
700

euphorbia_motif_173.m2m

fragment_325.1425	: 0.11
fragment_413.1975	: 0.05
fragment_297.1475	: 0.04
fragment_295.1325	: 0.03
fragment_343.1525	: 0.02
fragment_419.3125	: 0.02
fragment_237.1275	: 0.02
fragment_249.1275	: 0.01
fragment_267.1375	: 0.01
fragment_326.1475	: 0.01
fragment_277.1225	: 0.01
fragment_279.1375	: 0.01
fragment_307.1325	: 0.01
fragment_219.1175	: 0.01
fragment_473.2175	: 0.01
fragment_395.1875	: 0.01
fragment_265.1225	: 0.01
fragment_431.2075	: 0.01
loss_117.0775	: 0.01
fragment_207.1175	: 0.01
fragment_261.1275	: 0.01
fragment_233.1325	: 0.01
fragment_278.1275	: 0.01
fragment_127.1475	: 0.01
fragment_221.1325	: 0.01
loss_153.0975	: 0.01
fragment_179.0725	: 0.01
fragment_684.2975	: 0.01
fragment_205.0875	: 0.01
fragment_248.1175	: 0.01
fragment_235.1125	: 0.01
fragment_413.1925	: 0.01
fragment_247.1125	: 0.01
fragment_234.1025	: 0.01
fragment_251.1425	: 0.01
fragment_283.1325	: 0.01
fragment_414.1975	: 0.01
fragment_195.1175	: 0.01
fragment_145.0625	: 0.01
fragment_313.1425	: 0.01
fragment_250.1325	: 0.01
fragment_253.1225	: 0.01
fragment_385.1625	: 0.01
fragment_209.1325	: 0.01
fragment_275.1625	: 0.01
fragment_335.1625	: 0.01

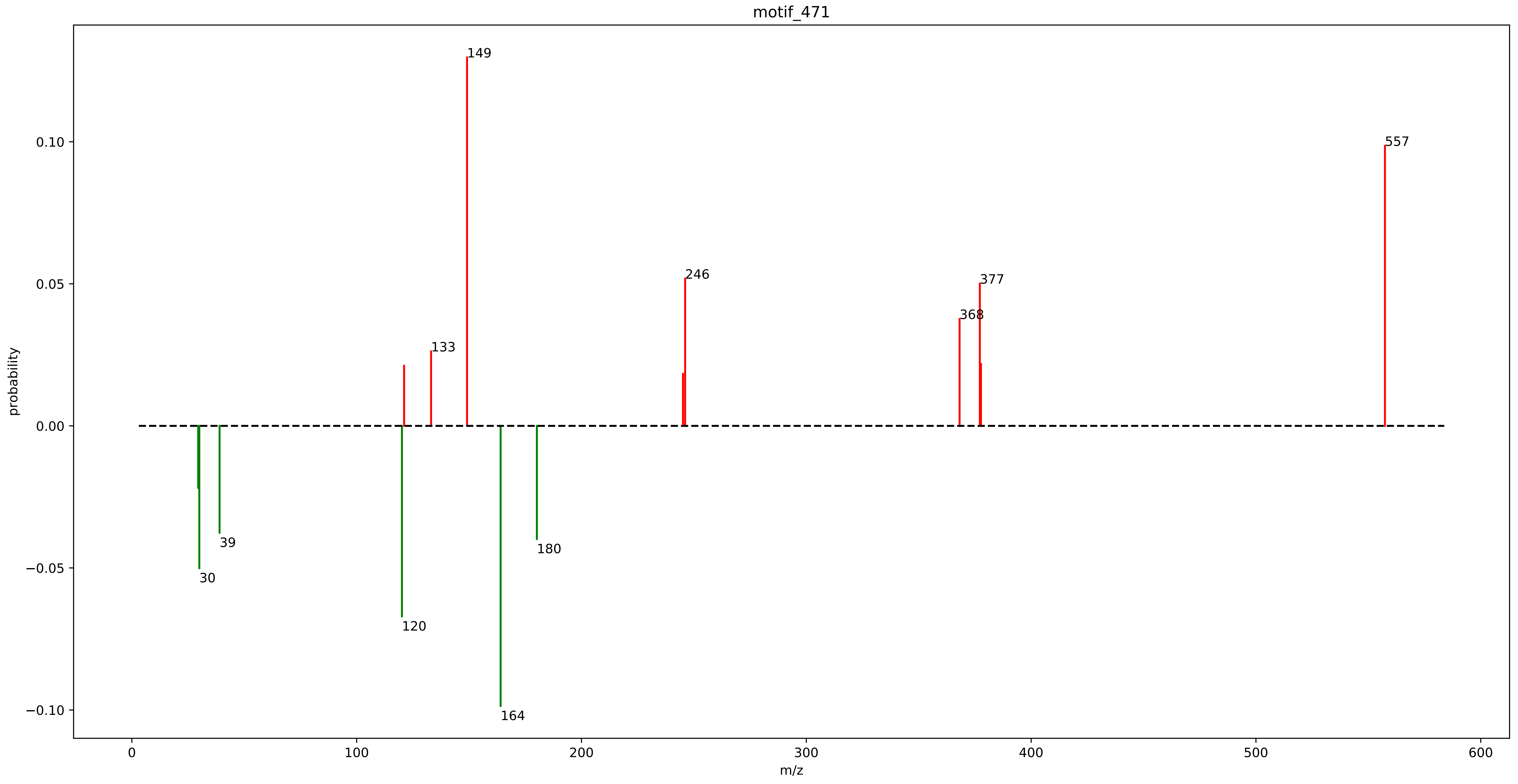

motif_471
probability
m/z
0.10
0.05
0.00
−0.05
−0.10
0
100
200
300
400
500
600
149
557
246
377
368
133
39
30
120
164
180

motif_471

fragment_149.0975	: 0.13
loss_164.0325	: 0.10
fragment_557.3825	: 0.10
loss_120.1375	: 0.07
fragment_246.0975	: 0.05
fragment_377.1875	: 0.05
loss_30.0075	: 0.05
loss_180.1575	: 0.04
fragment_368.1775	: 0.04
loss_39.0175	: 0.04
fragment_133.1025	: 0.03
fragment_377.6875	: 0.02
loss_29.5075	: 0.02
fragment_121.0975	: 0.02
fragment_245.1475	: 0.02

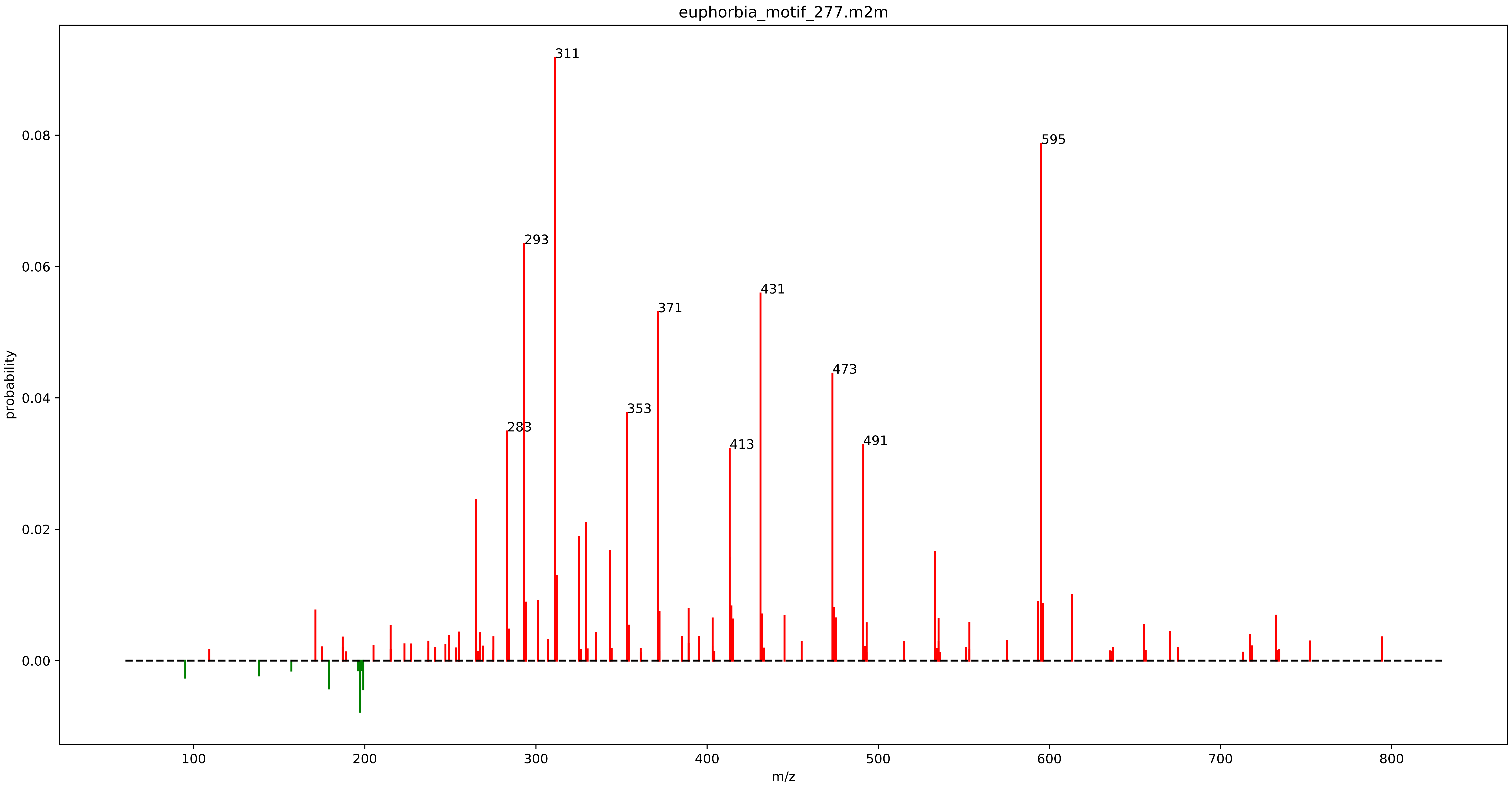

euphorbia_motif_277.m2m
probability
m/z
0.00
0.02
0.04
0.06
0.08
100
200
300
400
500
600
700
800
311
293
283
353
371
413
431
473
491
595

euphorbia_motif_277.m2m

fragment_311.1625	: 0.09
fragment_595.2525	: 0.08
fragment_293.1525	: 0.06
fragment_431.2075	: 0.06
fragment_371.1825	: 0.05
fragment_473.2175	: 0.04
fragment_353.1725	: 0.04
fragment_283.1675	: 0.03
fragment_491.2275	: 0.03
fragment_413.1925	: 0.03
fragment_265.1575	: 0.02
fragment_329.1725	: 0.02
fragment_325.1775	: 0.02
fragment_343.1875	: 0.02
fragment_533.2375	: 0.02
fragment_413.1975	: 0.02
fragment_312.1675	: 0.01
fragment_613.2625	: 0.01
fragment_301.1775	: 0.01
fragment_593.2575	: 0.01
fragment_294.1575	: 0.01
fragment_596.2575	: 0.01
fragment_414.1975	: 0.01
fragment_474.2175	: 0.01
fragment_389.1925	: 0.01
loss_197.0925	: 0.01
fragment_171.1175	: 0.01
fragment_372.1875	: 0.01
fragment_432.2075	: 0.01
fragment_732.3225	: 0.01
fragment_445.2225	: 0.01
fragment_475.2075	: 0.01
fragment_403.2125	: 0.01
fragment_535.2325	: 0.01
fragment_415.1875	: 0.01
fragment_553.2425	: 0.01
fragment_493.2175	: 0.01
fragment_343.1925	: 0.01
fragment_655.2725	: 0.01
fragment_354.1775	: 0.01
fragment_215.0675	: 0.01

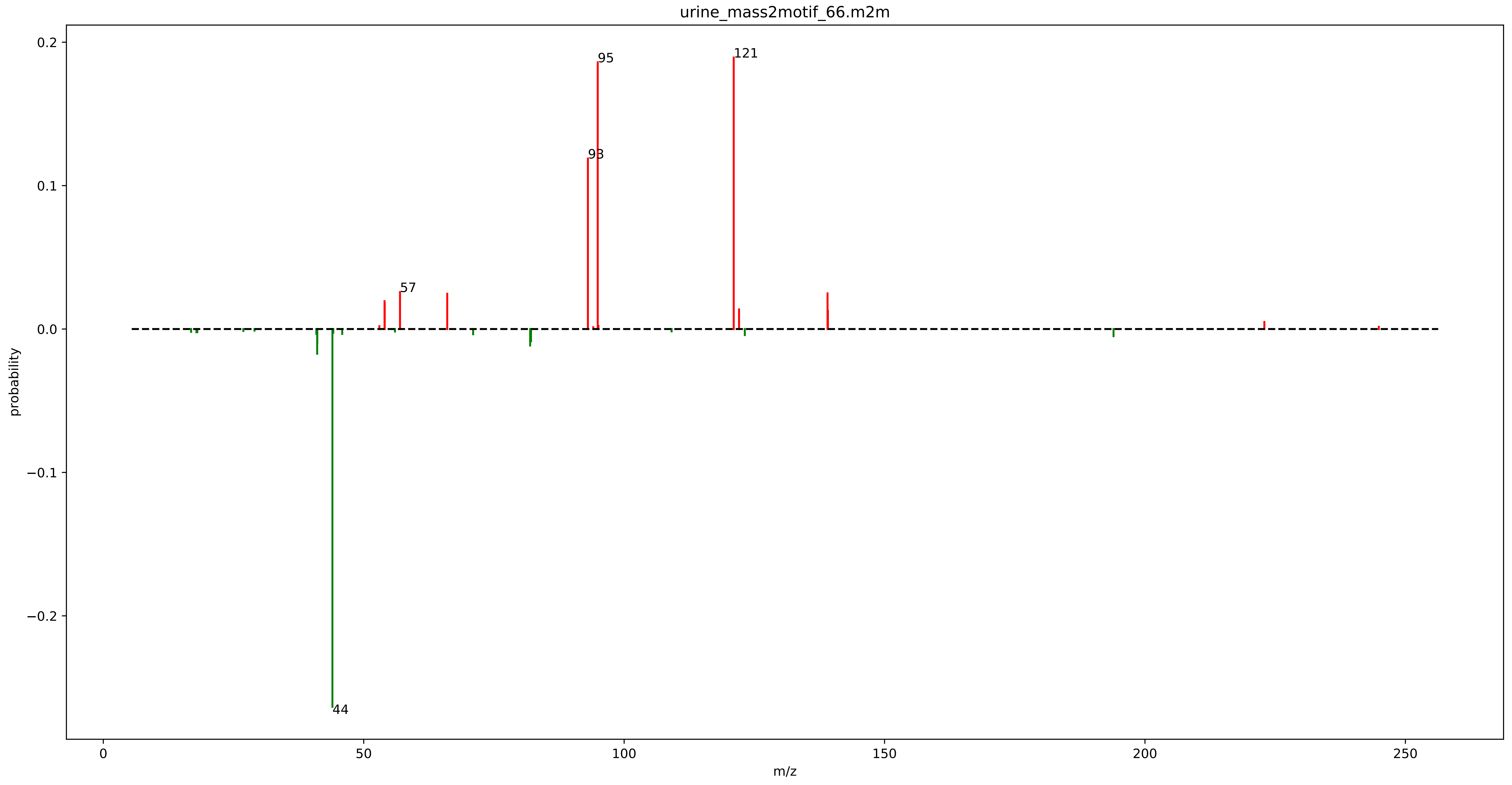

urine_mass2motif_66.m2m
probability
m/z
0.2
0.1
0.0
−0.1
−0.2
0
50
100
150
200
250
95
121
93
57
44

urine_mass2motif_66.m2m

loss_43.9875	: 0.26
fragment_121.0375	: 0.19
fragment_94.9275	: 0.19
fragment_93.0425	: 0.12
fragment_56.9675	: 0.03
fragment_139.0525	: 0.02
fragment_66.0325	: 0.02
fragment_53.9975	: 0.02
fragment_54.0325	: 0.02
loss_41.0625	: 0.02
fragment_122.0625	: 0.01
fragment_139.1225	: 0.01
loss_81.9525	: 0.01
loss_41.0875	: 0.01
loss_82.1225	: 0.01
loss_193.9625	: 0.01

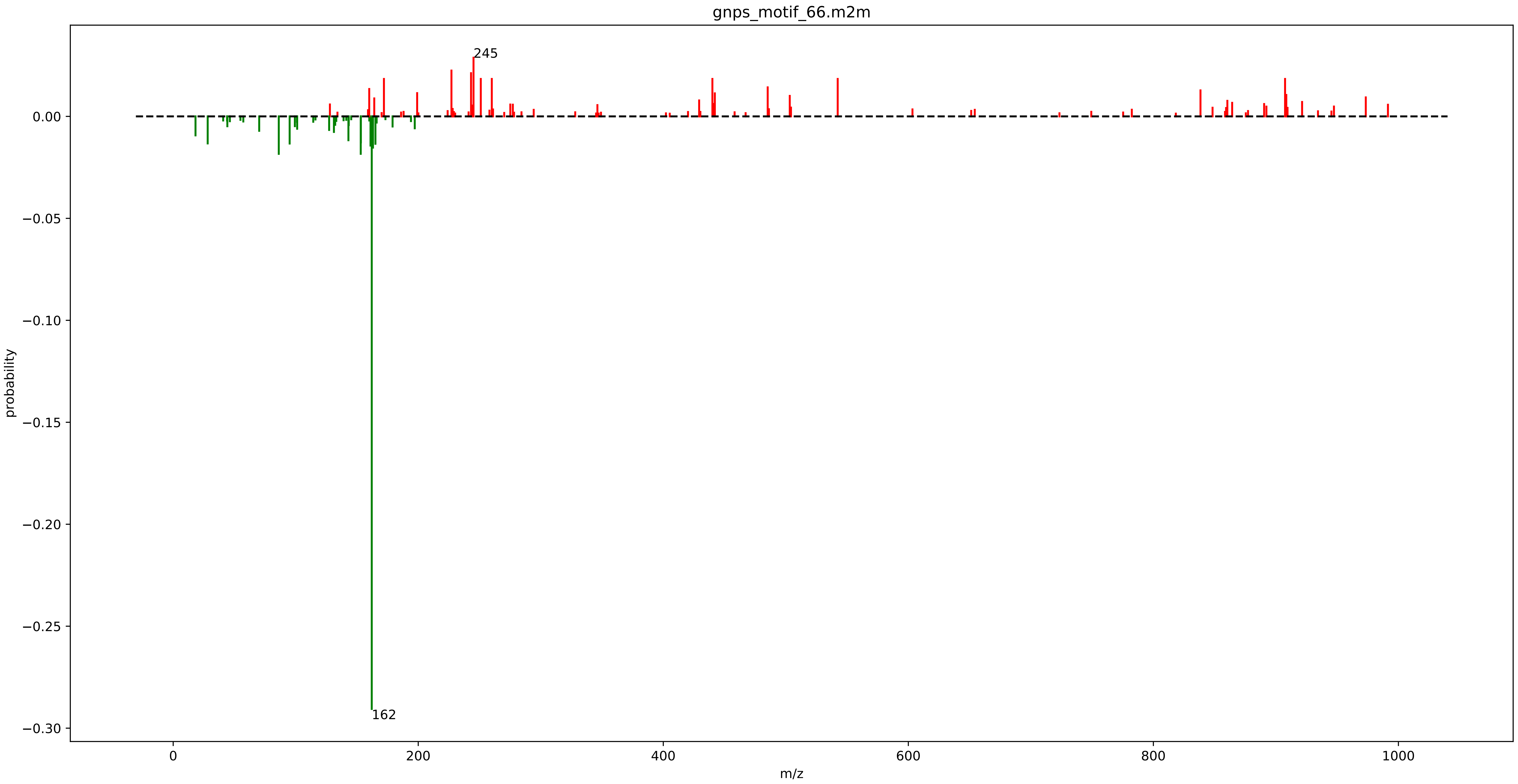
gnps_motif_66.m2m
245
162
probability
m/z
0.00
−0.05
−0.10
−0.15
−0.20
−0.25
−0.30
0
200
400
600
800
1000

gnps_motif_66.m2m

loss_162.0525	: 0.29
fragment_245.1175	: 0.03
fragment_227.1075	: 0.02
fragment_243.0975	: 0.02
fragment_542.3575	: 0.02
fragment_260.0375	: 0.02
fragment_440.0875	: 0.02
loss_86.1675	: 0.02
fragment_907.4875	: 0.02
fragment_172.0025	: 0.02
fragment_251.0575	: 0.02
loss_153.0675	: 0.02
loss_163.0625	: 0.02
loss_161.0475	: 0.01
fragment_485.2175	: 0.01
fragment_160.0025	: 0.01
loss_165.0625	: 0.01
loss_95.0475	: 0.01
loss_28.1525	: 0.01
loss_153.1725	: 0.01
fragment_838.4525	: 0.01
loss_143.0175	: 0.01
fragment_199.1125	: 0.01
fragment_442.0825	: 0.01
fragment_908.5025	: 0.01
fragment_503.2275	: 0.01
fragment_973.3675	: 0.01
loss_18.2125	: 0.01
fragment_164.1075	: 0.01
fragment_429.2725	: 0.01
loss_131.1875	: 0.01
fragment_860.3925	: 0.01
fragment_921.3875	: 0.01
loss_70.1975	: 0.01
loss_127.2775	: 0.01
fragment_864.3075	: 0.01
fragment_441.0925	: 0.01
fragment_890.4025	: 0.01
loss_101.1875	: 0.01
loss_197.1525	: 0.01
fragment_127.9075	: 0.01
fragment_275.1425	: 0.01
fragment_277.2175	: 0.01
fragment_991.4575	: 0.01
fragment_346.2375	: 0.01
fragment_244.1075	: 0.01

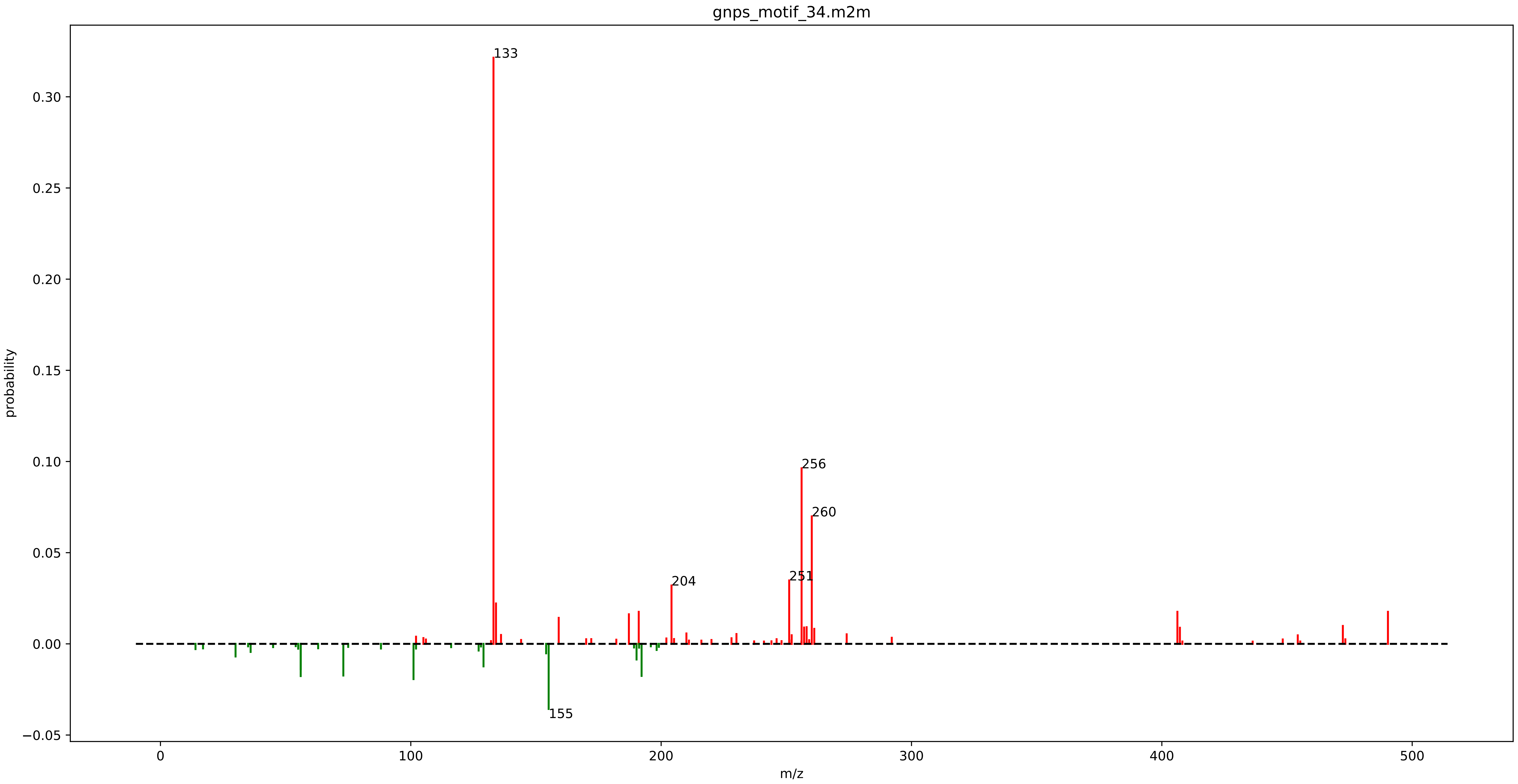
gnps_motif_34.m2m
probability
m/z
0.30
0.25
0.20
0.15
0.10
0.05
0.00
−0.05
0
100
200
300
400
500
133
256
260
204
251
155

gnps_motif_34.m2m

fragment_133.0275	: 0.32
fragment_256.0975	: 0.10
fragment_260.1625	: 0.07
loss_155.0675	: 0.04
fragment_251.1425	: 0.03
fragment_204.1375	: 0.03
fragment_134.0325	: 0.02
loss_101.0825	: 0.02
loss_56.0225	: 0.02
loss_192.1875	: 0.02
fragment_406.2125	: 0.02
fragment_490.3125	: 0.02
fragment_191.0475	: 0.02
loss_73.0525	: 0.02
fragment_187.1125	: 0.02
fragment_159.0825	: 0.01
loss_56.0275	: 0.01
loss_129.0425	: 0.01
fragment_472.3025	: 0.01
fragment_258.1125	: 0.01
fragment_257.1025	: 0.01
fragment_407.2125	: 0.01
loss_190.1725	: 0.01
fragment_261.1675	: 0.01
loss_29.9975	: 0.01
fragment_210.0925	: 0.01
fragment_230.0825	: 0.01
fragment_274.1075	: 0.01
loss_154.0625	: 0.01

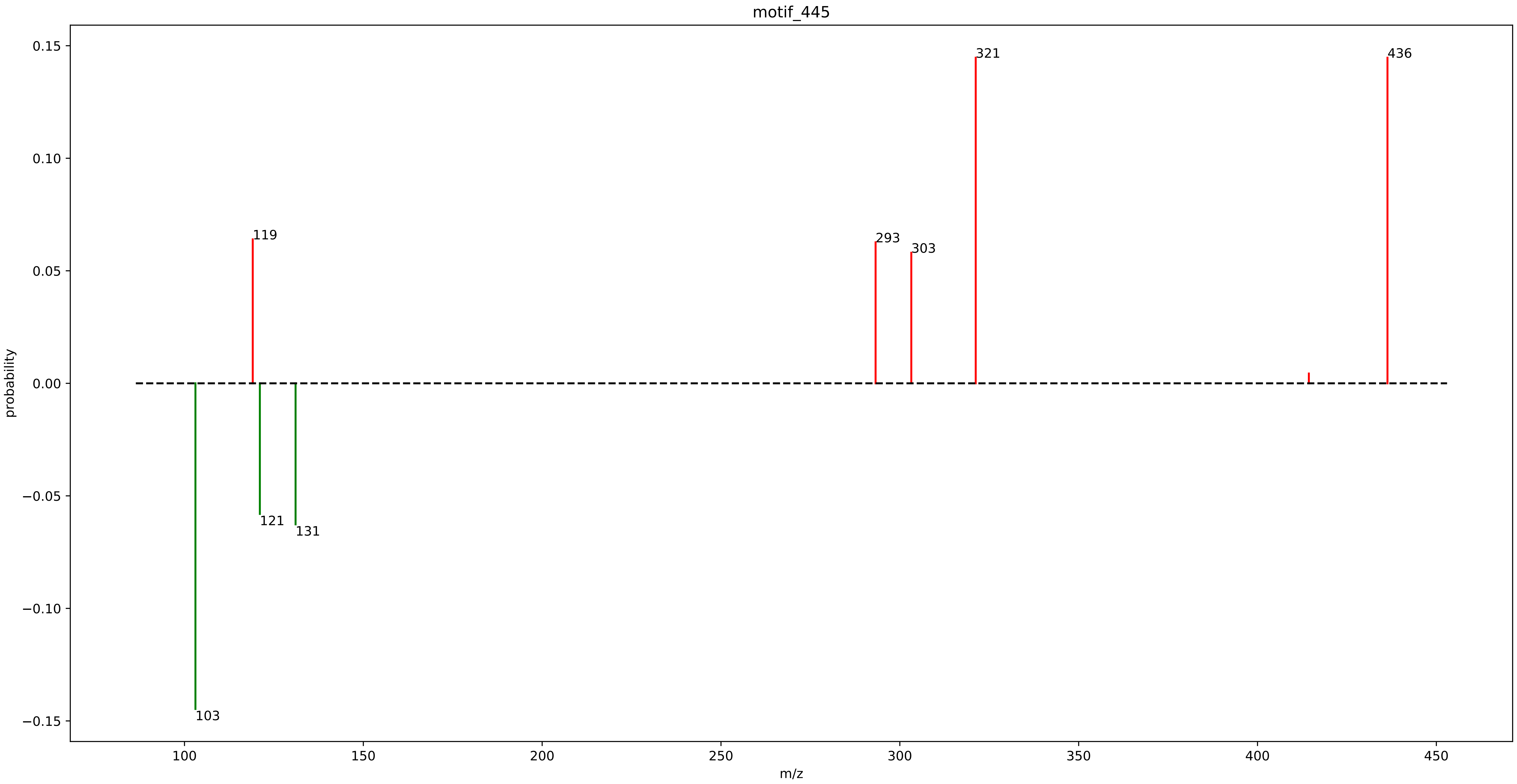

motif_445
probability
m/z
0.15
0.10
0.05
0.00
−0.05
−0.10
−0.15
100
150
200
250
300
350
400
450
119
293
303
321
436
103
121
131

motif_445

loss_103.0625	: 0.14
fragment_321.2175	: 0.14
fragment_436.3375	: 0.14
fragment_119.0825	: 0.06
loss_131.0575	: 0.06
fragment_293.2225	: 0.06
loss_121.0725	: 0.06
fragment_303.2075	: 0.06

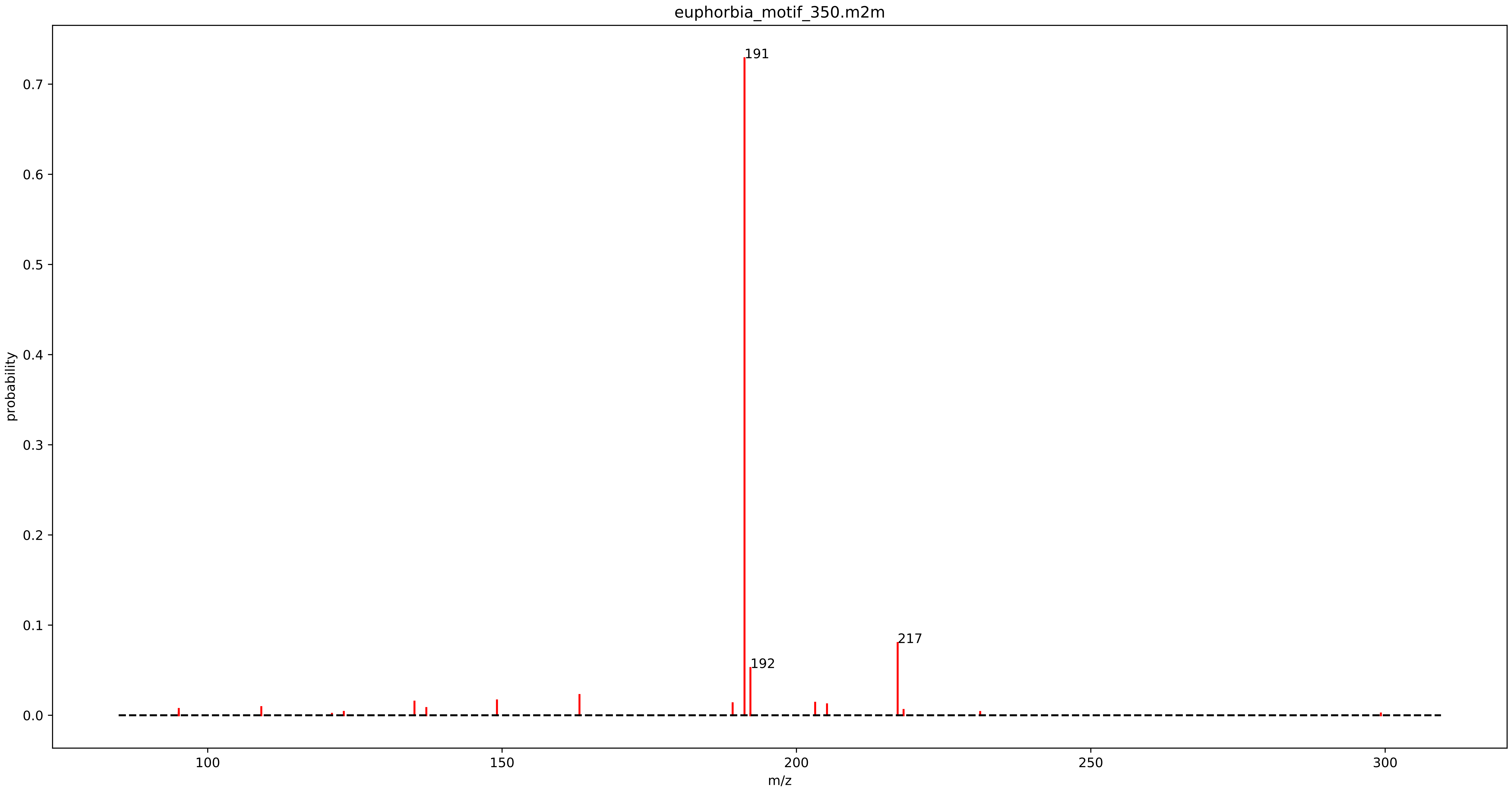

euphorbia_motif_350.m2m
191
192
217
probability
m/z
0.0
0.1
0.2
0.3
0.4
0.5
0.6
0.7
100
150
200
250
300

euphorbia_motif_350.m2m

fragment_191.1775	: 0.73
fragment_217.1925	: 0.08
fragment_192.1825	: 0.05
fragment_163.1475	: 0.02
fragment_149.1325	: 0.02
fragment_135.1175	: 0.02
fragment_203.1775	: 0.01
fragment_189.1625	: 0.01
fragment_205.1925	: 0.01
fragment_109.1025	: 0.01
fragment_137.1325	: 0.01
fragment_95.0875	: 0.01
fragment_218.1975	: 0.01

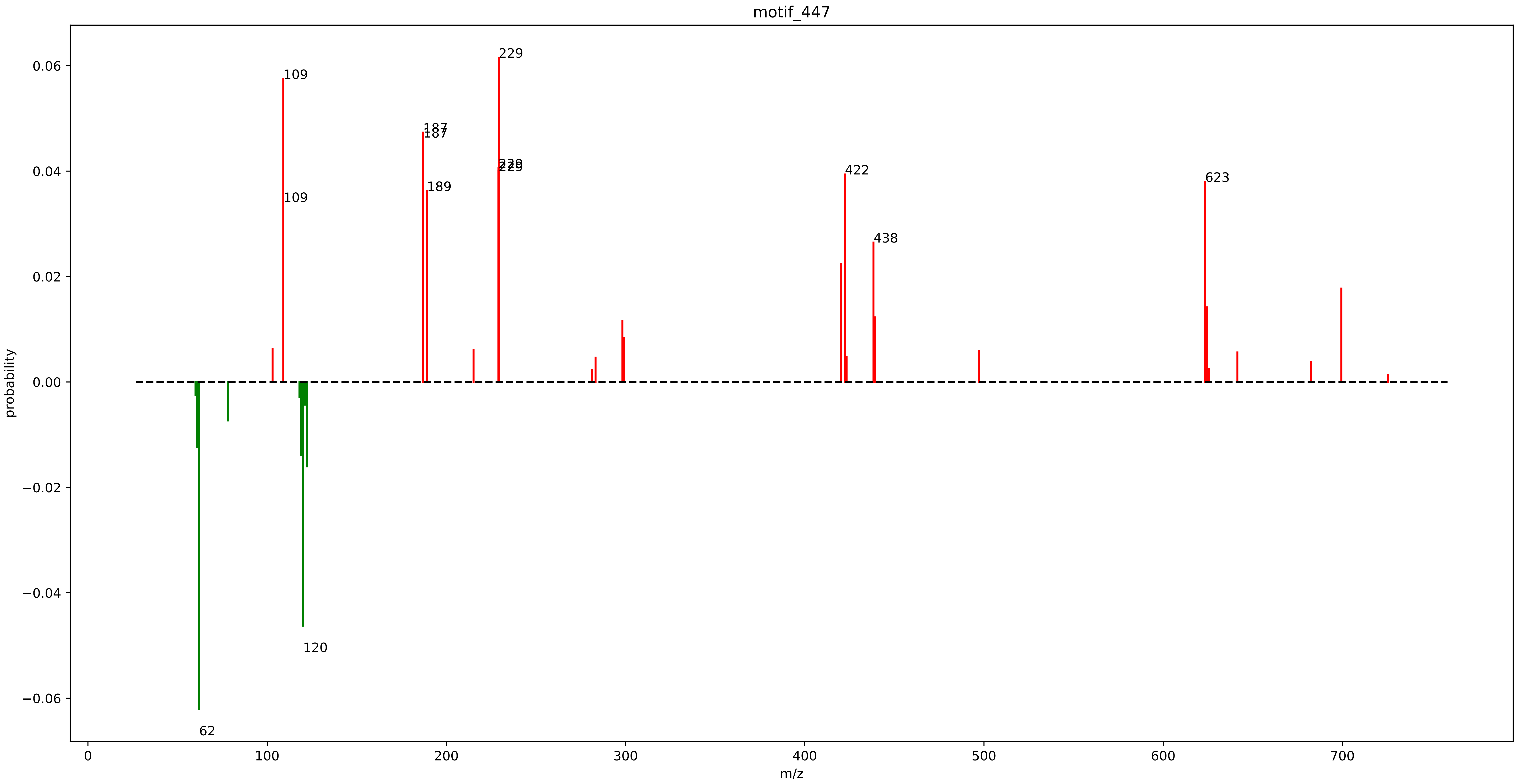

motif_447
109
229
187
187
229
229
189
109
422
623
438
120
62
probability
m/z
0.06
0.04
0.02
0.00
−0.02
−0.04
−0.06
0
100
200
300
400
500
600
700

motif_447

loss_62.0325	: 0.06
fragment_229.1775	: 0.06
fragment_109.0325	: 0.06
fragment_187.0675	: 0.05
fragment_187.0625	: 0.05
loss_120.0425	: 0.05
fragment_229.0825	: 0.04
fragment_229.1075	: 0.04
fragment_422.3475	: 0.04
fragment_623.3925	: 0.04
fragment_189.1625	: 0.04
fragment_109.0575	: 0.03
fragment_438.3425	: 0.03
fragment_420.3325	: 0.02
fragment_699.4075	: 0.02
loss_122.0525	: 0.02
fragment_624.3975	: 0.01
loss_119.0375	: 0.01
loss_61.0325	: 0.01
fragment_439.3575	: 0.01
fragment_298.2225	: 0.01
fragment_299.2375	: 0.01
loss_78.0375	: 0.01
fragment_103.0375	: 0.01
fragment_215.1775	: 0.01
fragment_497.3625	: 0.01
fragment_641.3975	: 0.01

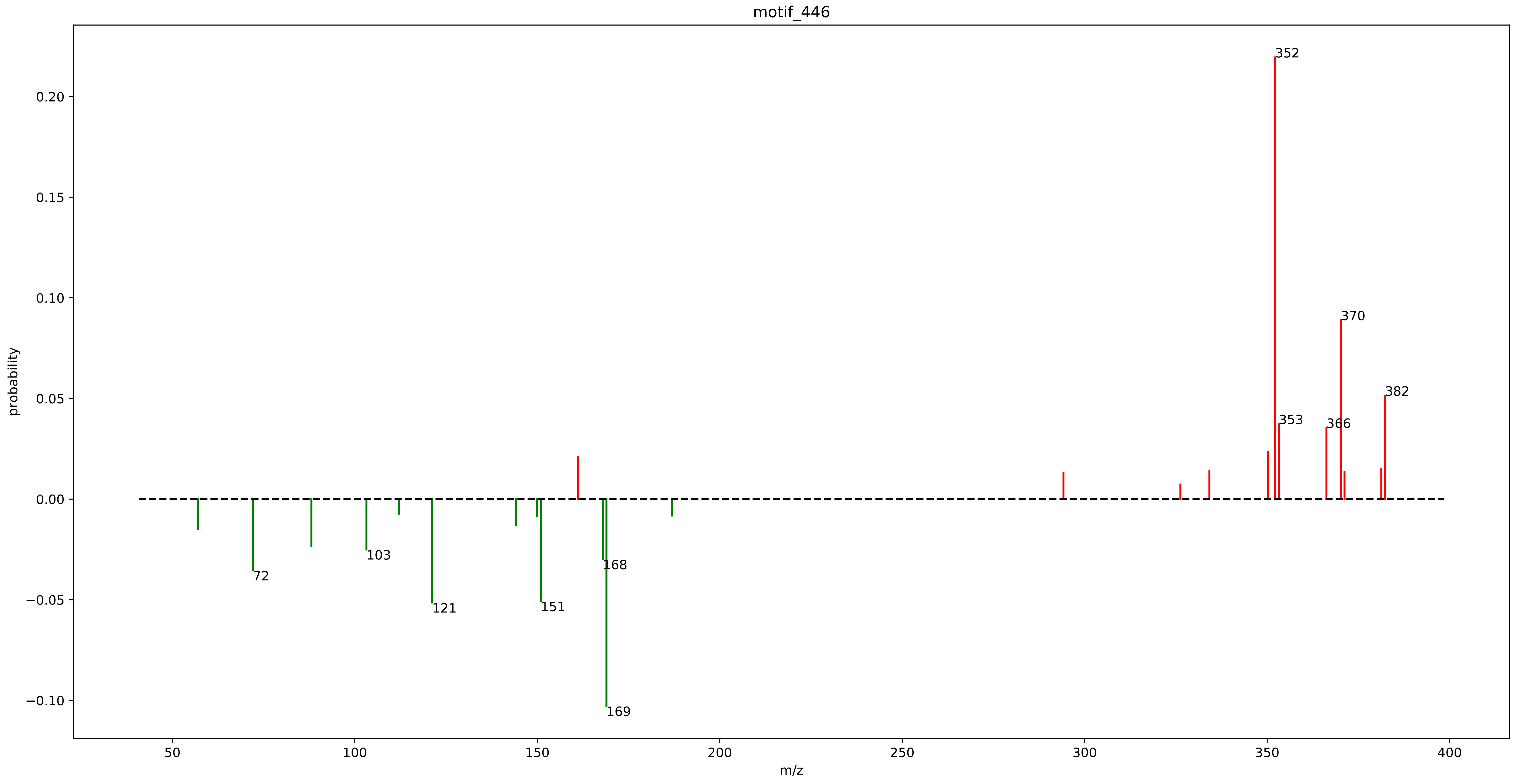

motif_446
probability
m/z
0.20
0.15
0.10
0.05
0.00
−0.05
−0.10
50
100
150
200
250
300
350
400
352
370
382
353
366
72
103
121
151
168
169

motif_446

fragment_352.1725	: 0.22
loss_168.9375	: 0.10
fragment_370.1875	: 0.09
loss_121.1875	: 0.05
fragment_382.2725	: 0.05
loss_150.9275	: 0.05
fragment_353.1775	: 0.04
fragment_366.2425	: 0.04
loss_72.0925	: 0.04
loss_167.9325	: 0.03
loss_103.1725	: 0.03
loss_88.0875	: 0.02
fragment_350.2475	: 0.02
fragment_161.1325	: 0.02
loss_57.0675	: 0.02
fragment_381.2675	: 0.02
fragment_334.1675	: 0.01
fragment_371.1875	: 0.01
loss_144.1475	: 0.01
fragment_294.1825	: 0.01
loss_149.9225	: 0.01
loss_186.9475	: 0.01
loss_112.1225	: 0.01
fragment_326.2125	: 0.01

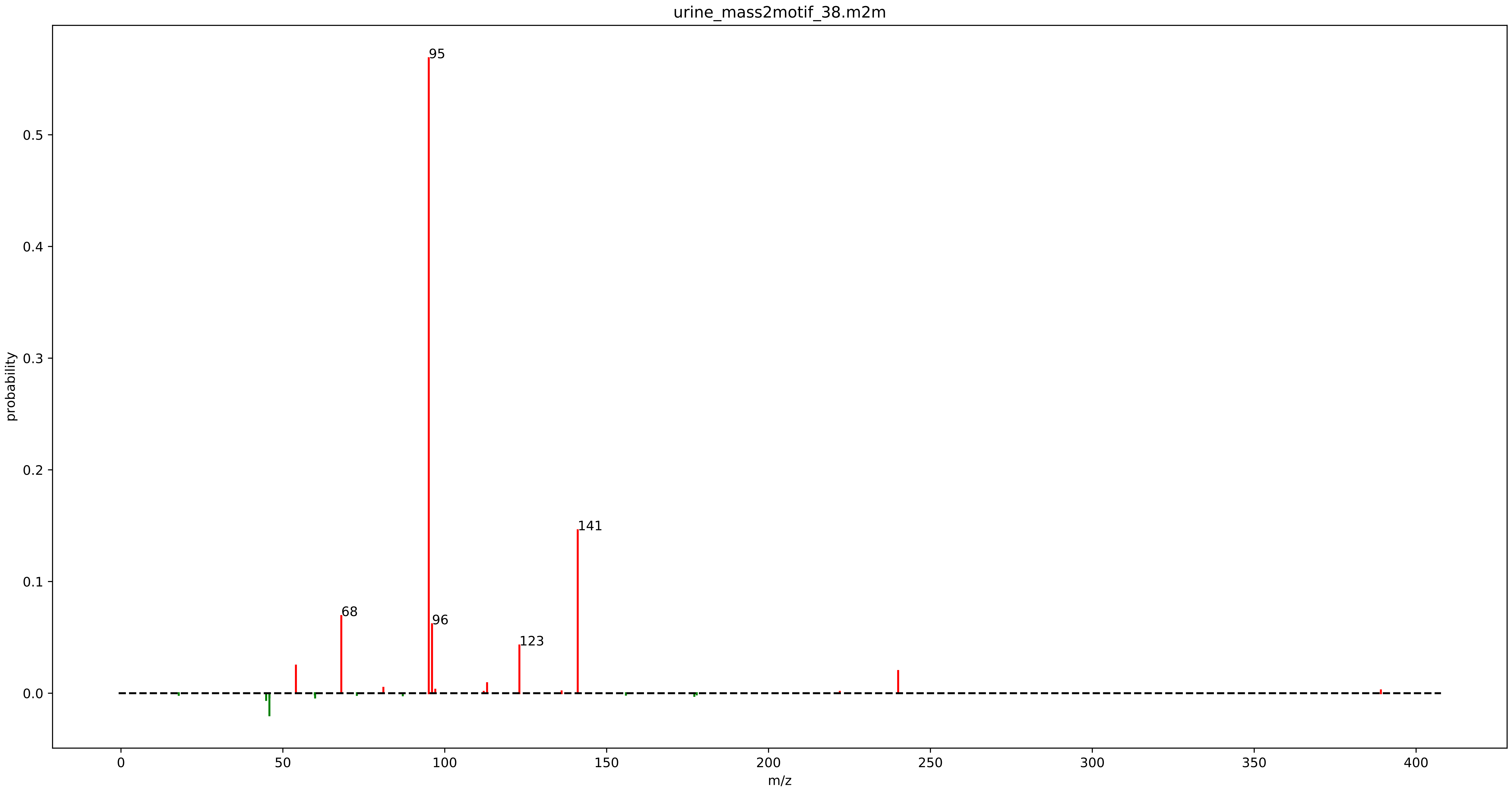
urine_mass2motif_38.m2m
probability
m/z
95
141
68
96
123
0.0
0.1
0.2
0.3
0.4
0.5
0
50
100
150
200
250
300
350
400

urine_mass2motif_38.m2m

fragment_95.0625	: 0.57
fragment_141.0675	: 0.15
fragment_68.0475	: 0.07
fragment_96.0675	: 0.06
fragment_123.0525	: 0.04
fragment_54.0325	: 0.02
fragment_240.0325	: 0.02
loss_45.8425	: 0.02
fragment_113.0725	: 0.01
loss_44.8375	: 0.01

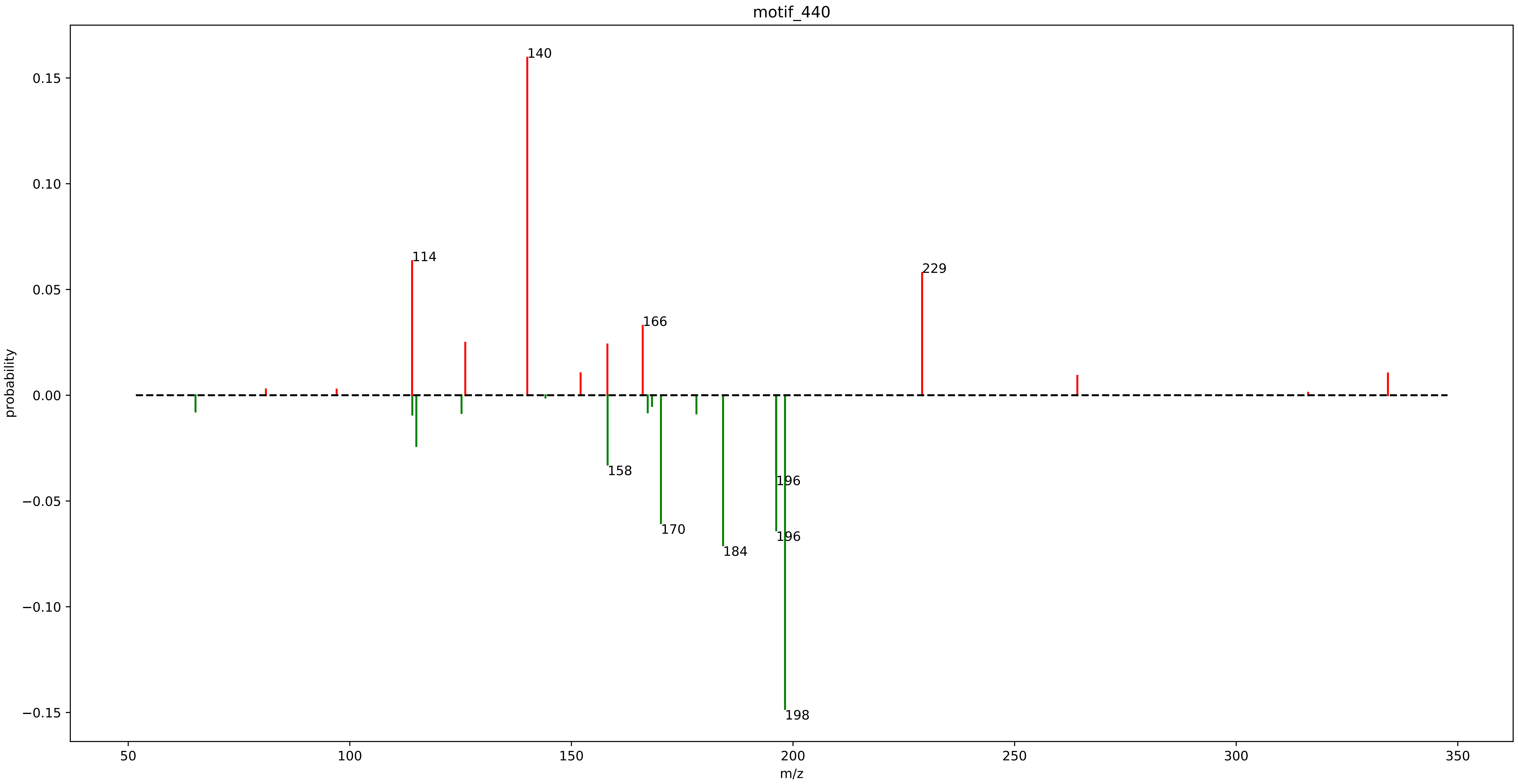
motif_440
140
114
229
166
158
196
170
196
184
198
probability
0.15
0.10
0.05
0.00
−0.05
−0.10
−0.15
50
100
150
200
250
300
350
m/z

motif_440

fragment_140.0425	: 0.16
loss_198.1975	: 0.15
loss_184.2175	: 0.07
loss_196.2175	: 0.06
fragment_114.0525	: 0.06
loss_170.2025	: 0.06
fragment_229.1325	: 0.06
loss_196.1825	: 0.04
loss_158.1675	: 0.03
fragment_166.0875	: 0.03
fragment_126.0525	: 0.02
fragment_158.1175	: 0.02
loss_115.0075	: 0.02
fragment_152.0725	: 0.01
fragment_334.2375	: 0.01
fragment_264.1575	: 0.01
loss_114.1025	: 0.01
loss_178.2075	: 0.01
loss_125.2075	: 0.01
loss_167.2175	: 0.01
loss_65.1825	: 0.01
loss_168.1875	: 0.01

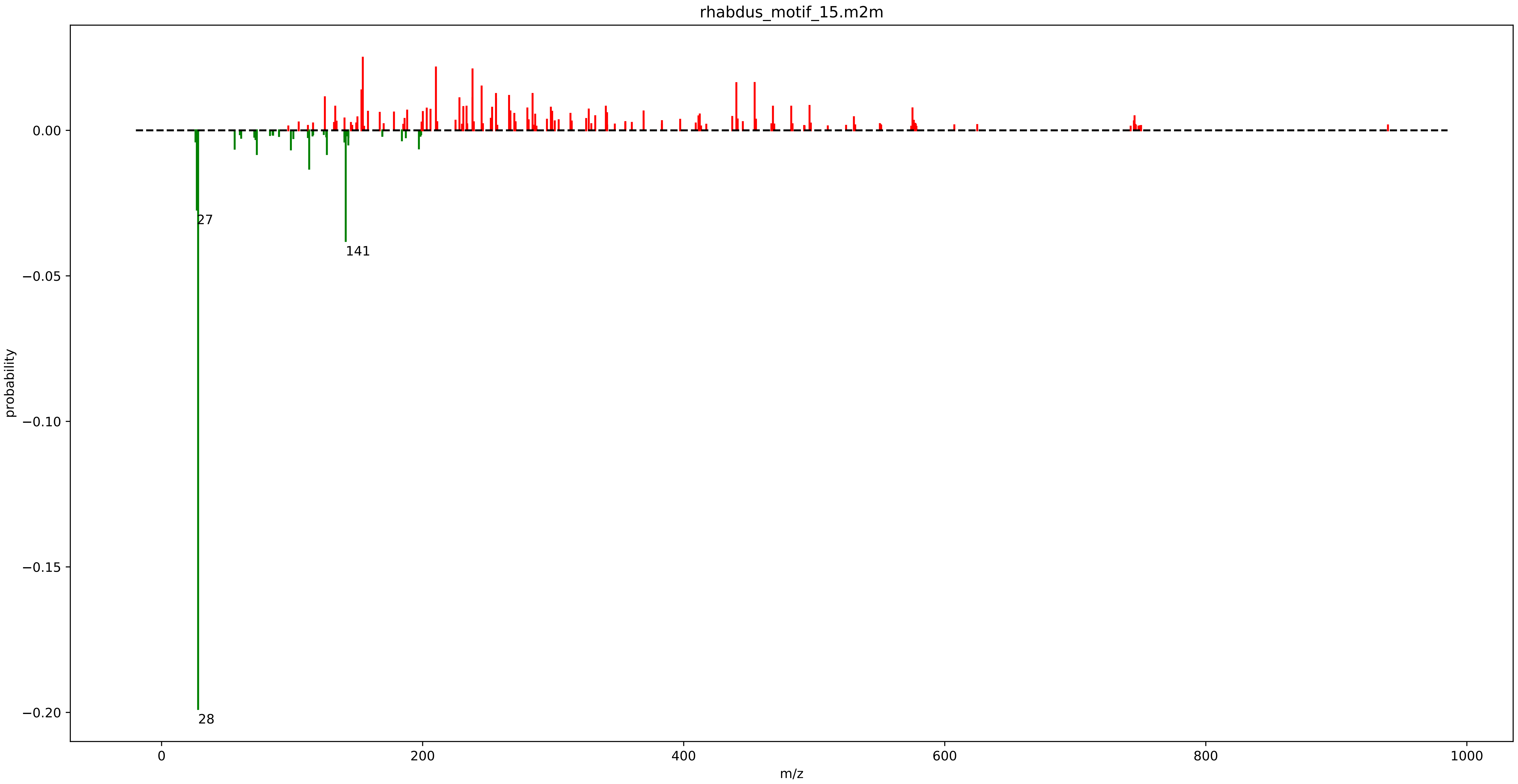
rhabdus_motif_15.m2m
probability
m/z
0.00
−0.05
−0.10
−0.15
−0.20
0
200
400
600
800
1000
27
28
141

rhabdus_motif_15.m2m

loss_27.9925	: 0.20
loss_141.0775	: 0.04
loss_26.9925	: 0.03
fragment_154.1575	: 0.02
fragment_210.1825	: 0.02
fragment_238.2175	: 0.02
fragment_454.3625	: 0.02
fragment_440.3475	: 0.02
fragment_245.1875	: 0.02
fragment_153.1275	: 0.01
loss_113.0825	: 0.01
fragment_284.2225	: 0.01
fragment_256.1925	: 0.01
fragment_266.2125	: 0.01
fragment_125.0975	: 0.01
fragment_238.1775	: 0.01
fragment_228.1975	: 0.01
fragment_256.2275	: 0.01
fragment_496.4125	: 0.01
fragment_468.3775	: 0.01
fragment_482.3925	: 0.01
fragment_133.0525	: 0.01
loss_72.9775	: 0.01
fragment_340.3575	: 0.01
loss_126.6375	: 0.01
fragment_233.6375	: 0.01
fragment_231.1675	: 0.01
fragment_298.2375	: 0.01
fragment_253.2275	: 0.01
fragment_575.2825	: 0.01
fragment_280.2275	: 0.01
fragment_203.1375	: 0.01
fragment_327.2625	: 0.01
fragment_206.0275	: 0.01
fragment_188.1425	: 0.01
loss_99.0675	: 0.01
fragment_267.2425	: 0.01
fragment_369.2725	: 0.01
fragment_158.1175	: 0.01
fragment_299.2325	: 0.01
loss_55.9925	: 0.01
fragment_200.1625	: 0.01
loss_197.1425	: 0.01
fragment_178.0325	: 0.01
fragment_167.1425	: 0.01
fragment_341.2775	: 0.01
fragment_313.2475	: 0.01
fragment_270.2425	: 0.01
fragment_412.3175	: 0.01
fragment_286.2175	: 0.01

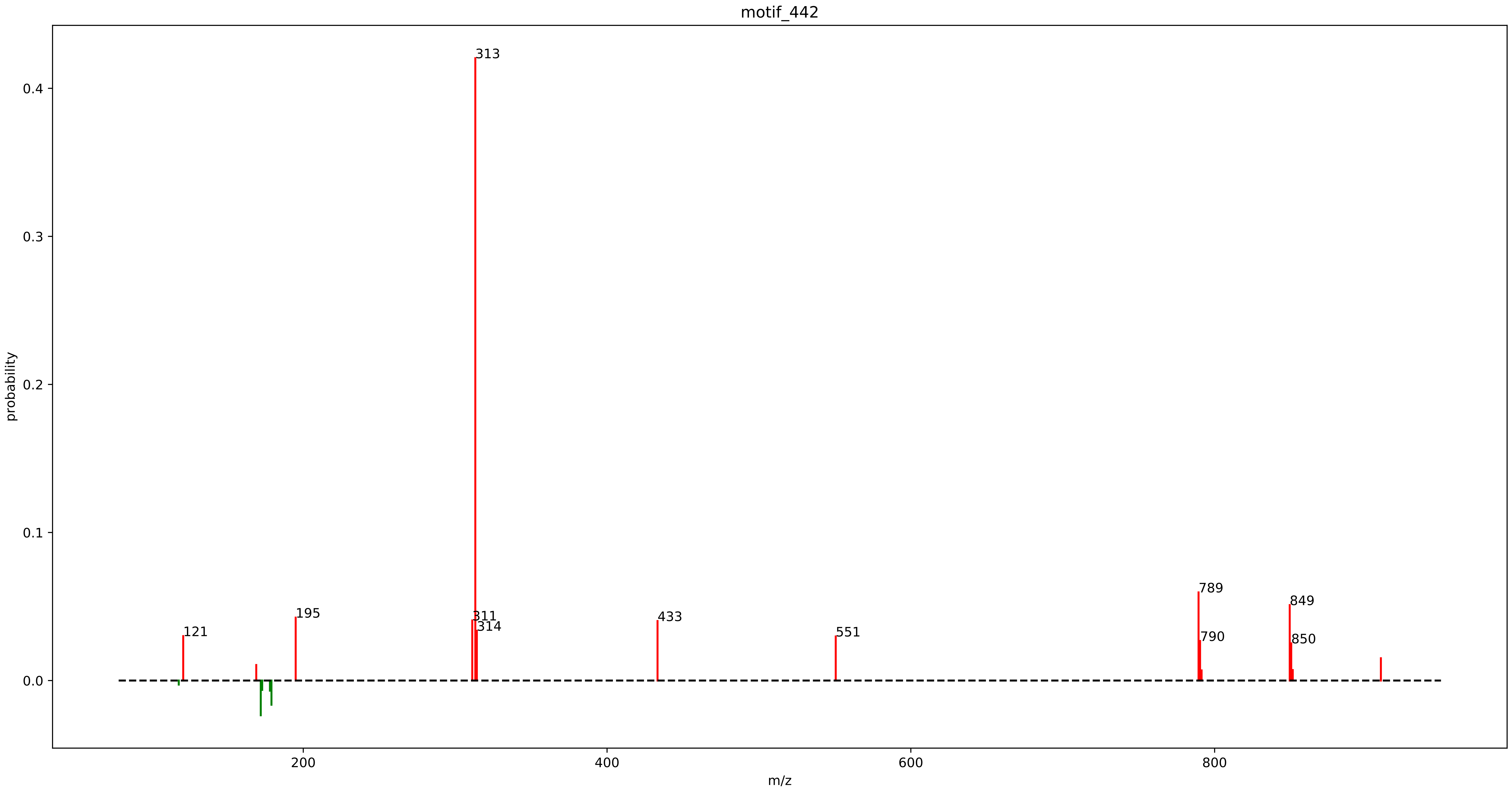

motif_442
probability
m/z
0.4
0.3
0.2
0.1
0.0
200
400
600
800
313
121
195
311
314
433
551
789
790
849
850

motif_442

fragment_313.2725	: 0.42
fragment_789.4175	: 0.06
fragment_849.4425	: 0.05
fragment_195.0025	: 0.04
fragment_311.2575	: 0.04
fragment_433.2325	: 0.04
fragment_314.2775	: 0.03
fragment_120.9625	: 0.03
fragment_550.5525	: 0.03
fragment_790.4225	: 0.03
fragment_850.4425	: 0.03
loss_172.0125	: 0.02
loss_179.0575	: 0.02
fragment_909.4625	: 0.02
fragment_169.0475	: 0.01
fragment_851.4475	: 0.01
fragment_791.4275	: 0.01
loss_178.0525	: 0.01
loss_172.9575	: 0.01

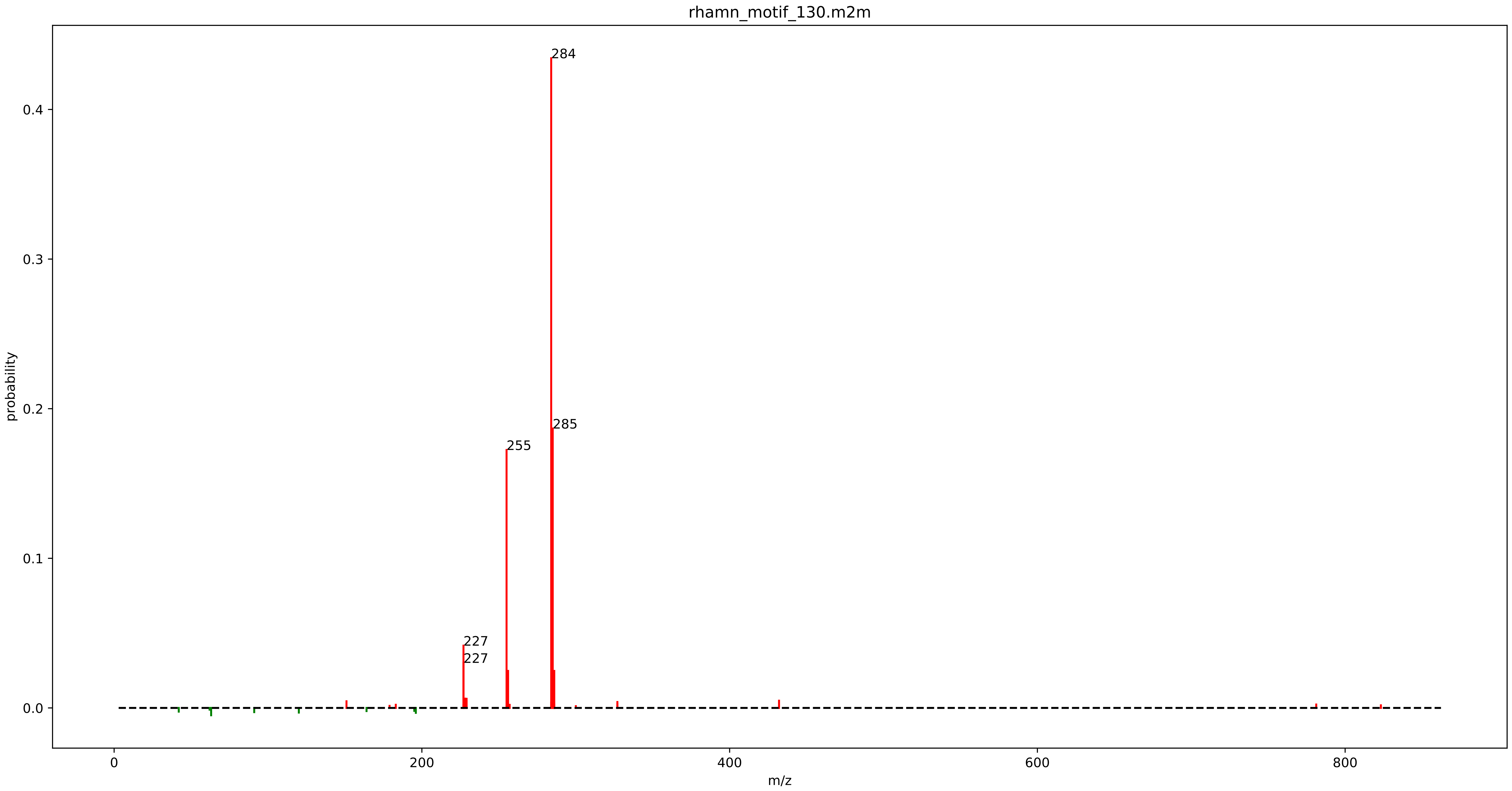

rhamn_motif_130.m2m
284
285
255
227
227
probability
m/z
0.0
0.1
0.2
0.3
0.4
0
200
400
600
800

rhamn_motif_130.m2m

fragment_284.0325	: 0.43
fragment_285.0375	: 0.19
fragment_255.0275	: 0.17
fragment_227.0325	: 0.04
fragment_227.0375	: 0.03
fragment_256.0325	: 0.02
fragment_286.0425	: 0.02
fragment_228.0375	: 0.01
fragment_229.0475	: 0.01

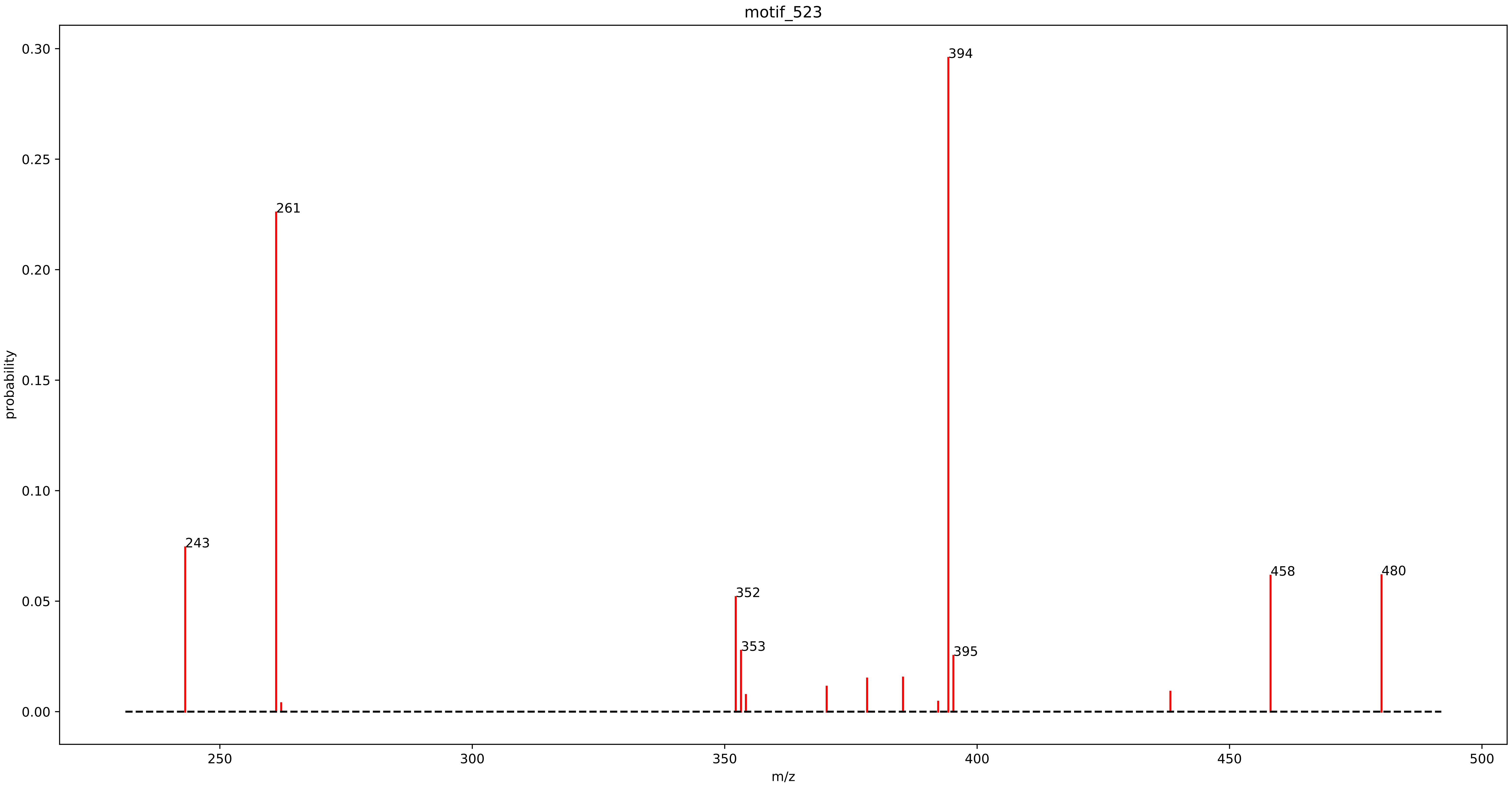

motif_523
394
261
243
352
353
395
458
480
probability
m/z
0.30
0.25
0.20
0.15
0.10
0.05
0.00
250
300
350
400
450
500

motif_523

fragment_394.2925	: 0.30
fragment_261.1425	: 0.23
fragment_243.1325	: 0.07
fragment_480.1025	: 0.06
fragment_458.1225	: 0.06
fragment_352.1775	: 0.05
fragment_353.2225	: 0.03
fragment_395.2975	: 0.03
fragment_385.3125	: 0.02
fragment_378.2025	: 0.01
fragment_370.1875	: 0.01
fragment_438.2825	: 0.01
fragment_354.1925	: 0.01

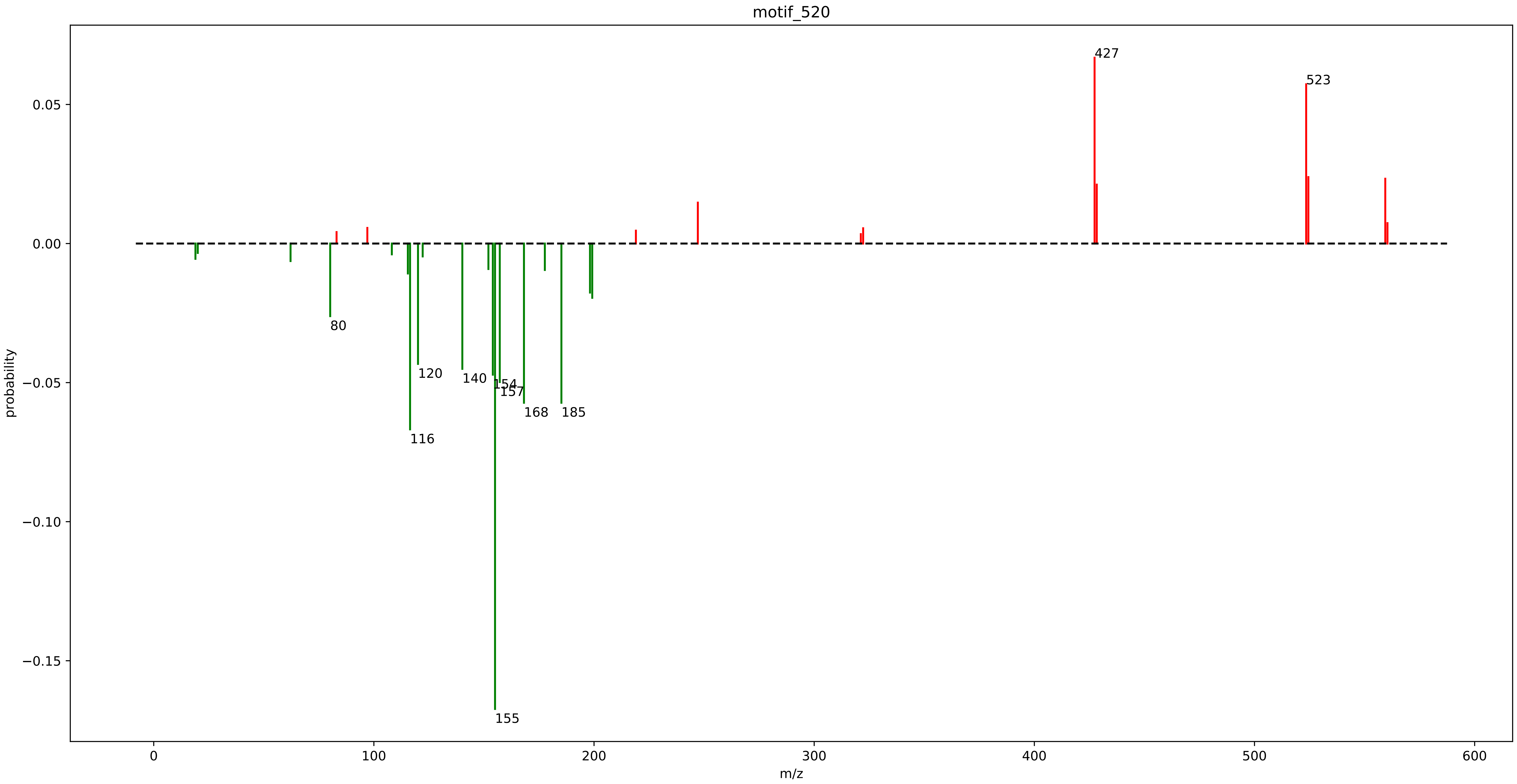

motif_520
427
523
80
120
140
154
157
168
185
116
155
probability
0.05
0.00
−0.05
−0.10
−0.15
0
100
200
300
400
500
600
m/z

motif_520

loss_155.0325	: 0.17
loss_116.4375	: 0.07
fragment_427.3575	: 0.07
fragment_523.4725	: 0.06
loss_168.1725	: 0.06
loss_185.1775	: 0.06
loss_157.1825	: 0.05
loss_154.0275	: 0.05
loss_140.1775	: 0.05
loss_120.0425	: 0.04
loss_80.1575	: 0.03
fragment_524.4775	: 0.02
fragment_559.4025	: 0.02
fragment_428.3575	: 0.02
loss_199.1925	: 0.02
loss_198.1675	: 0.02
fragment_247.1525	: 0.01
loss_115.4375	: 0.01
loss_115.4325	: 0.01
loss_177.6575	: 0.01
loss_152.0225	: 0.01
fragment_560.4025	: 0.01
loss_62.1475	: 0.01
fragment_97.0275	: 0.01
loss_18.9475	: 0.01
fragment_322.2375	: 0.01

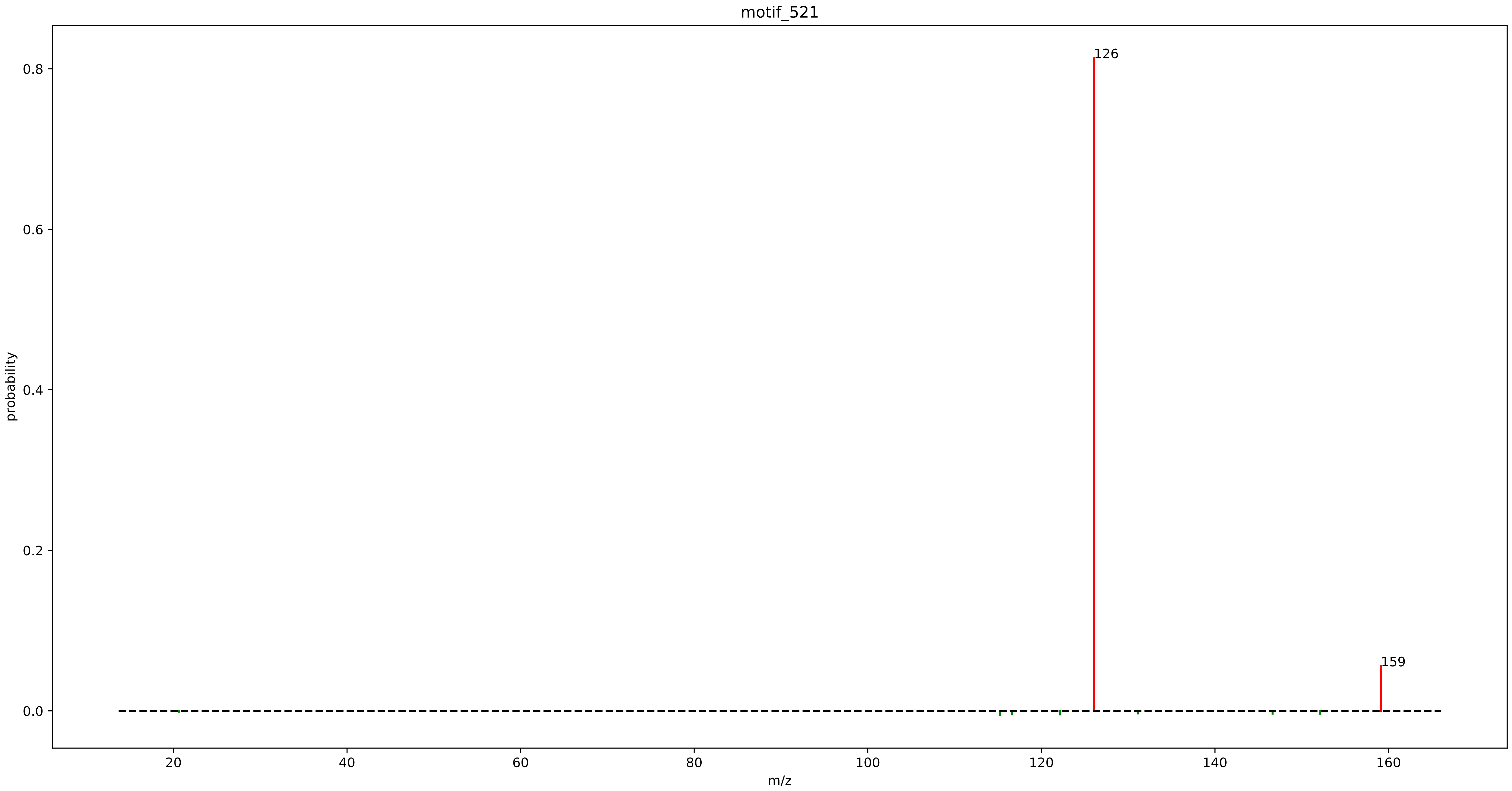
motif_521
probability
m/z
126
159
0.0
0.2
0.4
0.6
0.8
20
40
60
80
100
120
140
160

motif_521

fragment_126.0525	: 0.81
fragment_159.1175	: 0.06
loss_115.2275	: 0.01

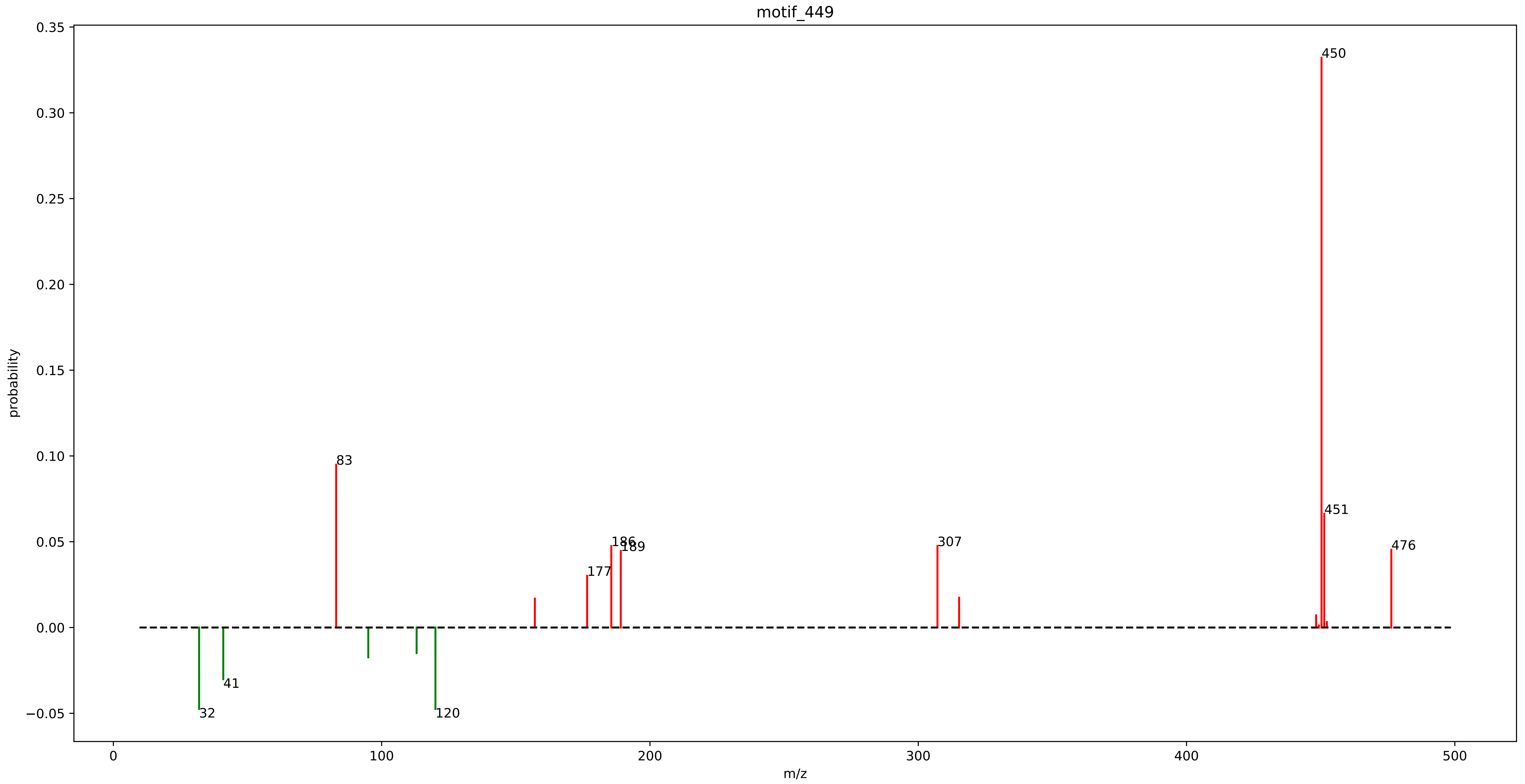

motif_449
probability
m/z
0.35
0.30
0.25
0.20
0.15
0.10
0.05
0.00
−0.05
0
100
200
300
400
500
450
83
451
186
189
307
476
177
41
32
120

motif_449

fragment_450.3175	: 0.33
fragment_83.0475	: 0.09
fragment_451.3225	: 0.07
fragment_307.1625	: 0.05
fragment_185.5975	: 0.05
loss_120.0425	: 0.05
loss_31.9575	: 0.05
fragment_476.3325	: 0.05
fragment_189.1275	: 0.04
loss_40.9625	: 0.03
fragment_176.5875	: 0.03
loss_95.0175	: 0.02
fragment_315.2325	: 0.02
fragment_157.1025	: 0.02
loss_113.0275	: 0.01
fragment_448.3025	: 0.01

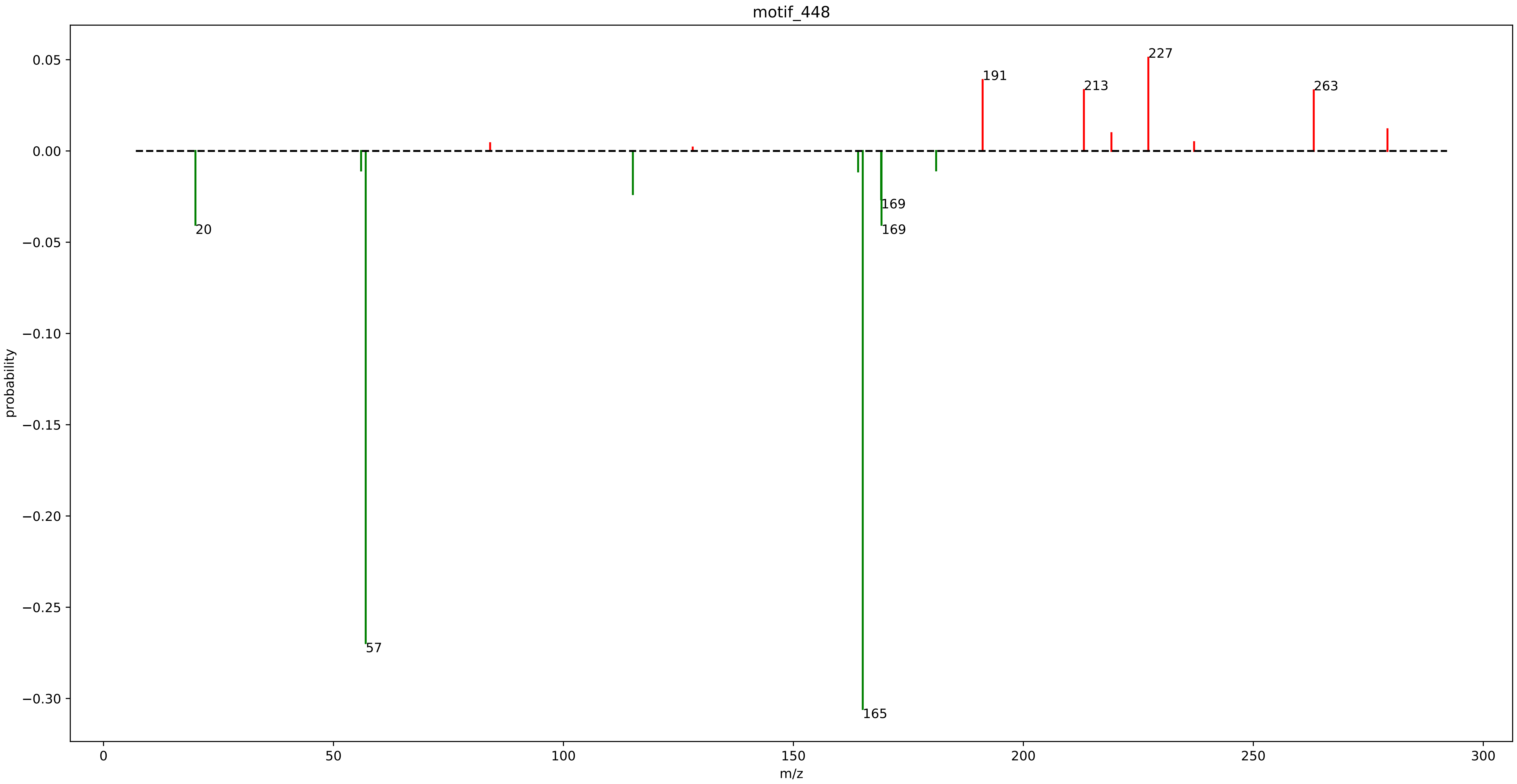

motif_448
probability
m/z
0.05
0.00
−0.05
−0.10
−0.15
−0.20
−0.25
−0.30
0
50
100
150
200
250
300
20
57
165
169
169
191
213
227
263

motif_448

loss_165.0775	: 0.31
loss_57.0075	: 0.27
fragment_227.1725	: 0.05
loss_169.1575	: 0.04
loss_19.9875	: 0.04
fragment_191.1425	: 0.04
fragment_213.1625	: 0.03
fragment_263.1375	: 0.03
loss_169.0725	: 0.03
loss_115.0975	: 0.02
fragment_279.1675	: 0.01
loss_164.0725	: 0.01
loss_56.0075	: 0.01
loss_181.0375	: 0.01
fragment_219.1475	: 0.01

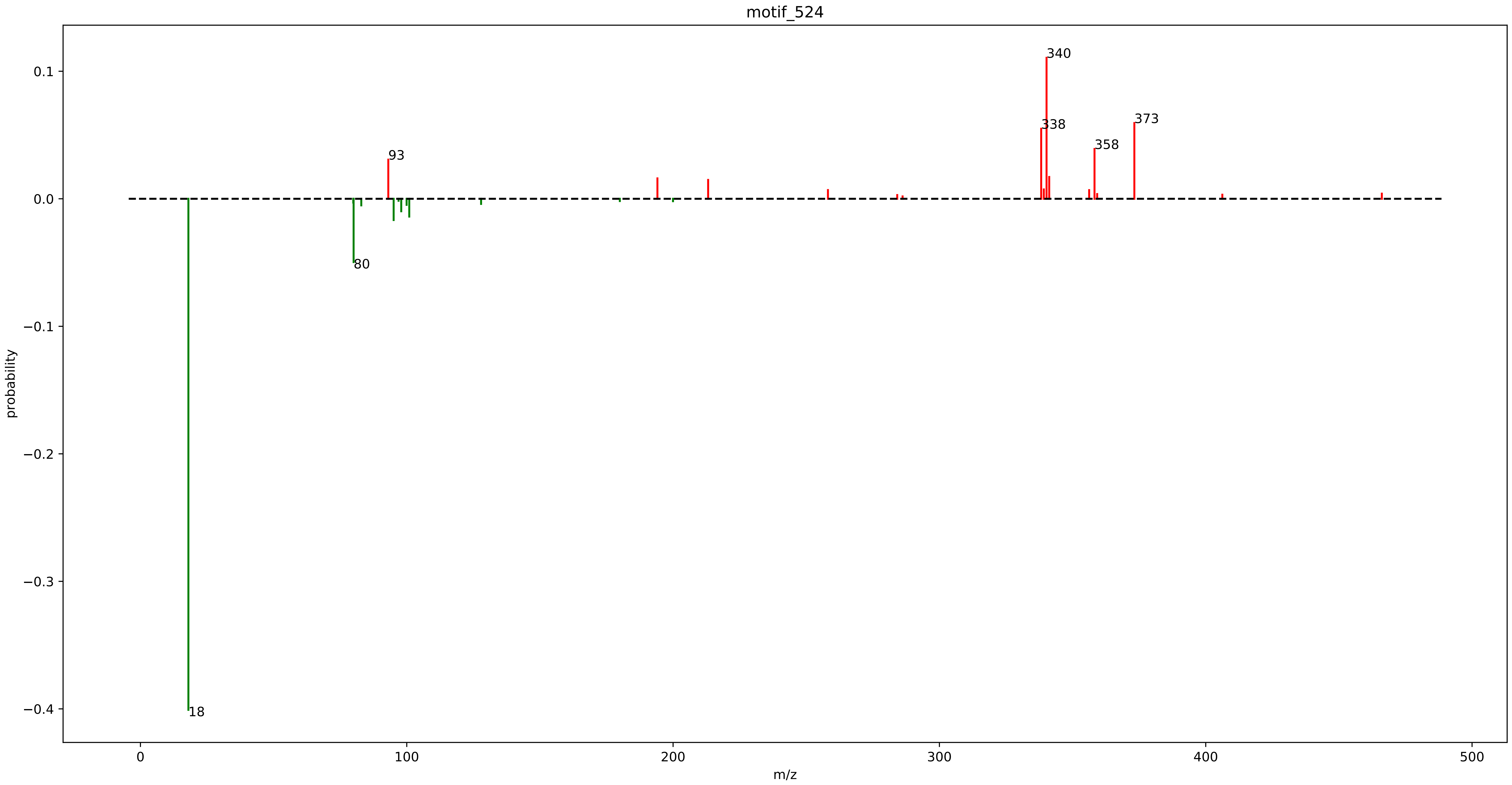
motif_524
340
338
373
358
93
80
18
0.1
0.0
−0.1
−0.2
−0.3
−0.4
probability
0
100
200
300
400
500
m/z

motif_524

loss_18.0075	: 0.40
fragment_340.2275	: 0.11
fragment_373.1975	: 0.06
fragment_338.2125	: 0.05
loss_80.0425	: 0.05
fragment_358.2375	: 0.04
fragment_93.0675	: 0.03
fragment_341.2325	: 0.02
loss_95.0775	: 0.02
fragment_194.1175	: 0.02
fragment_213.1625	: 0.01
loss_100.9375	: 0.01
loss_97.9275	: 0.01
fragment_339.2125	: 0.01
fragment_258.1475	: 0.01
fragment_356.2225	: 0.01
loss_82.9275	: 0.01

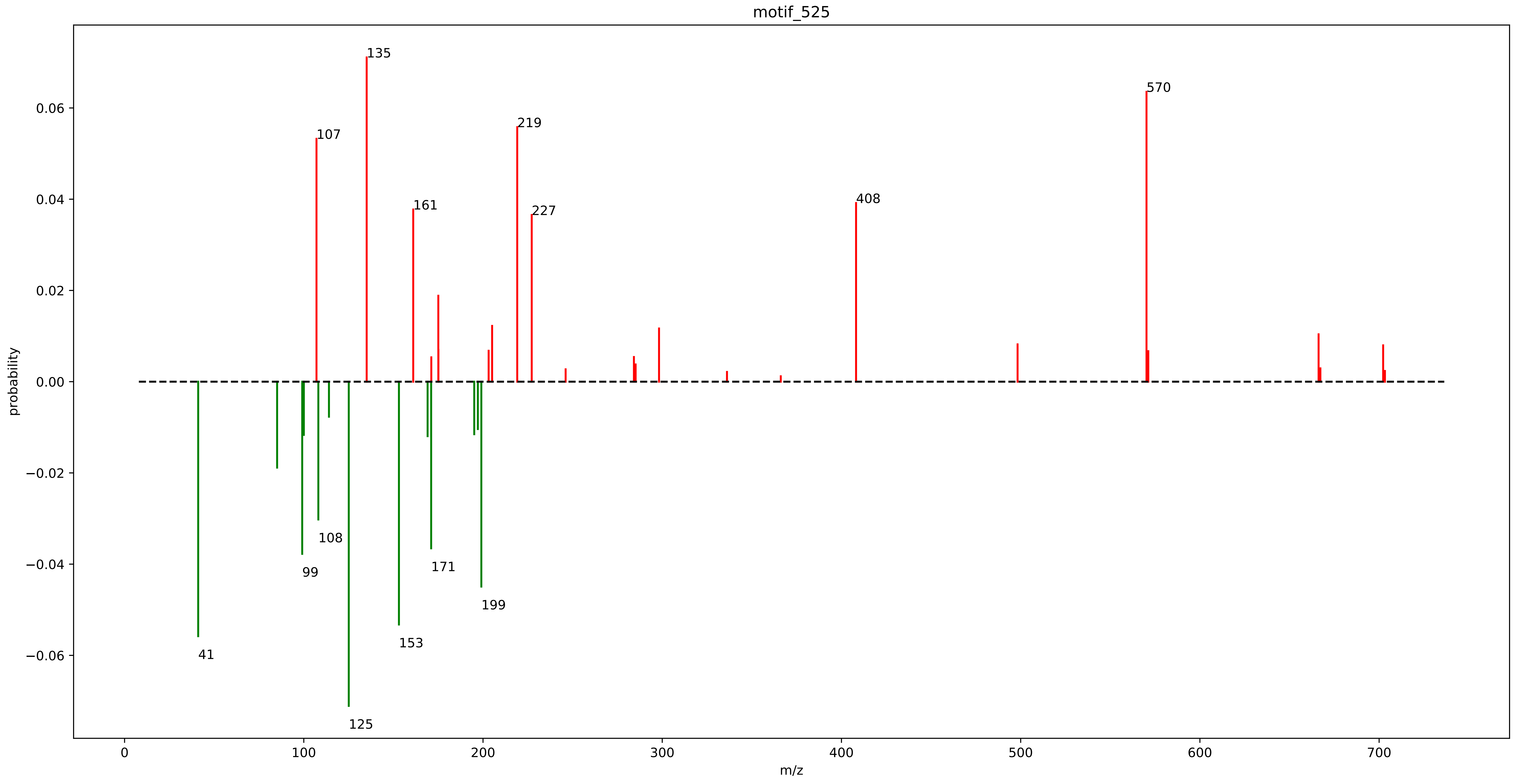

motif_525
probability
m/z
0.06
0.04
0.02
0.00
−0.02
−0.04
−0.06
0
100
200
300
400
500
600
700
135
570
107
219
161
227
408
41
99
108
125
153
171
199

motif_525

fragment_135.0825	: 0.07
loss_125.0925	: 0.07
fragment_570.2025	: 0.06
loss_41.0775	: 0.06
fragment_219.0925	: 0.06
fragment_107.0825	: 0.05
loss_153.0875	: 0.05
loss_199.0625	: 0.04
fragment_408.1475	: 0.04
fragment_161.0625	: 0.04
loss_99.1125	: 0.04
loss_171.0675	: 0.04
fragment_227.1725	: 0.04
loss_108.1125	: 0.03
fragment_175.0575	: 0.02
loss_85.1125	: 0.02
fragment_205.0775	: 0.01
loss_169.0825	: 0.01
loss_100.0275	: 0.01
fragment_298.2125	: 0.01
loss_195.1025	: 0.01
loss_197.1175	: 0.01
fragment_666.2225	: 0.01
fragment_498.2675	: 0.01
fragment_702.2475	: 0.01
loss_114.0425	: 0.01
fragment_175.1175	: 0.01
fragment_203.1375	: 0.01
fragment_571.2075	: 0.01
fragment_284.1975	: 0.01
fragment_171.1475	: 0.01

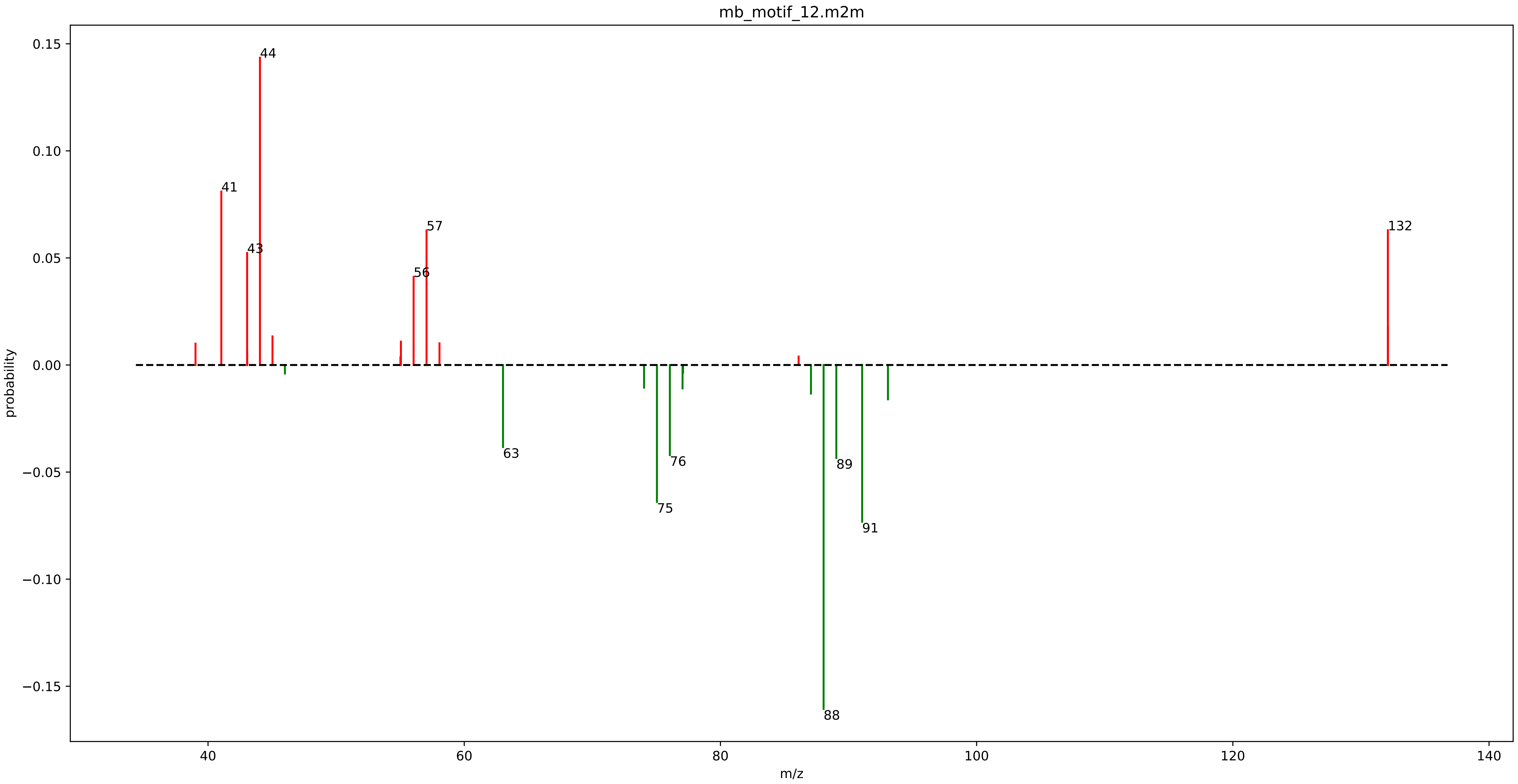

mb_motif_12.m2m
probability
m/z
0.15
0.10
0.05
0.00
−0.05
−0.10
−0.15
40
60
80
100
120
140
44
41
43
57
56
132
63
75
76
88
89
91

mb_motif_12.m2m

loss_88.0525	: 0.16
fragment_44.0525	: 0.14
fragment_41.0375	: 0.08
loss_91.0625	: 0.07
loss_75.0425	: 0.06
fragment_132.1025	: 0.06
fragment_57.0575	: 0.06
fragment_43.0525	: 0.05
loss_89.0475	: 0.04
loss_76.0525	: 0.04
fragment_56.0475	: 0.04
loss_63.0275	: 0.04
fragment_132.1075	: 0.02
loss_93.0775	: 0.02
fragment_45.0325	: 0.01
loss_87.0675	: 0.01
loss_77.0425	: 0.01
fragment_55.0575	: 0.01
loss_74.0375	: 0.01
fragment_58.0675	: 0.01
fragment_39.0225	: 0.01
fragment_43.0575	: 0.01

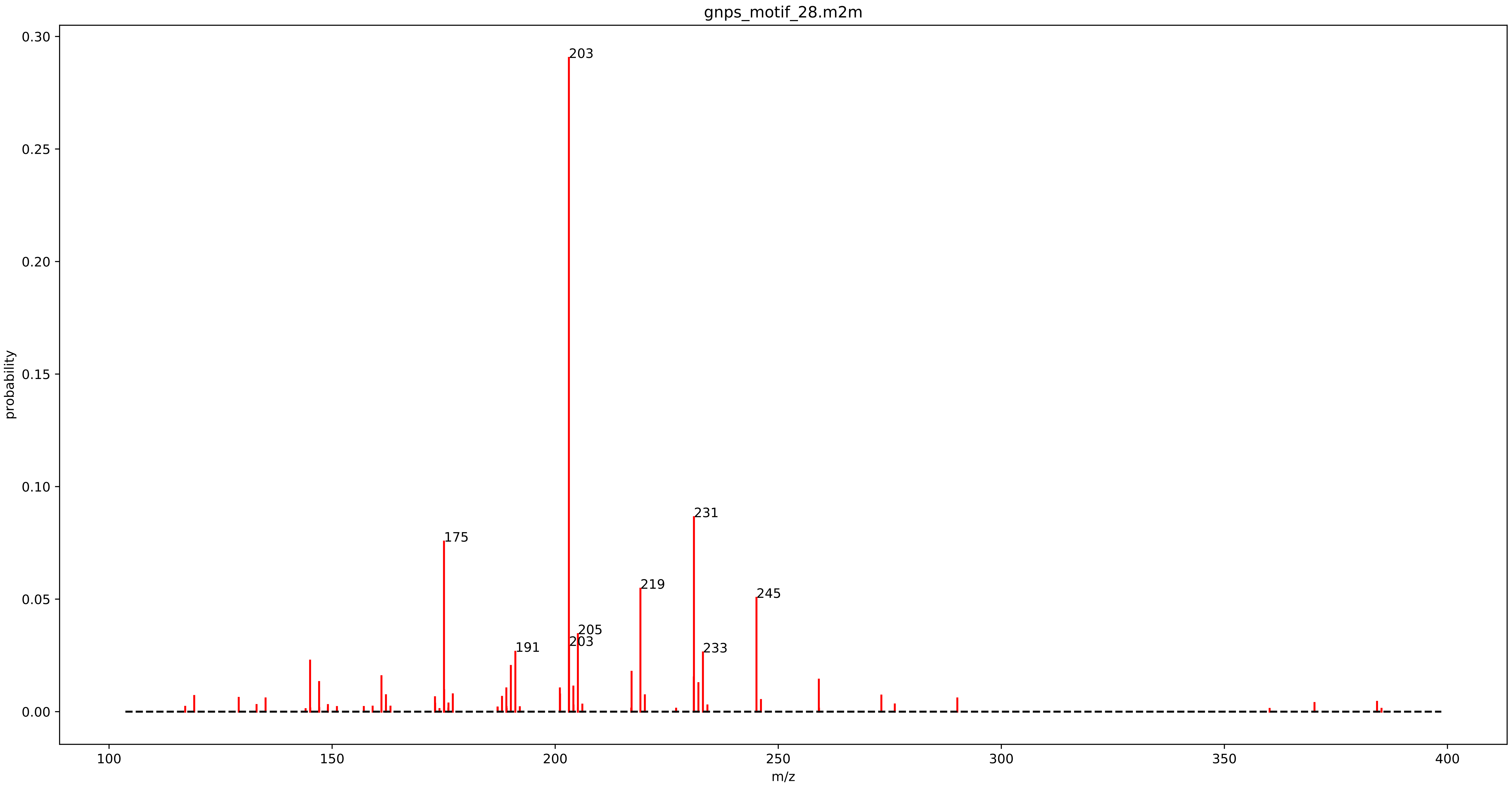
gnps_motif_28.m2m
probability
m/z
0.00
0.05
0.10
0.15
0.20
0.25
0.30
100
150
200
250
300
350
400
203
175
231
219
245
205
203
191
233

gnps_motif_28.m2m

fragment_203.0725	: 0.29
fragment_231.1025	: 0.09
fragment_175.0775	: 0.08
fragment_219.1025	: 0.05
fragment_245.1175	: 0.05
fragment_205.0875	: 0.03
fragment_203.1075	: 0.03
fragment_191.0725	: 0.03
fragment_233.1175	: 0.03
fragment_145.0625	: 0.02
fragment_190.0625	: 0.02
fragment_217.1225	: 0.02
fragment_161.0575	: 0.02
fragment_231.0675	: 0.02
fragment_259.0975	: 0.01
fragment_147.0825	: 0.01
fragment_232.1025	: 0.01
fragment_204.0725	: 0.01
fragment_189.0525	: 0.01
fragment_201.0525	: 0.01
fragment_175.1125	: 0.01
fragment_177.0525	: 0.01
fragment_201.0925	: 0.01
fragment_162.0675	: 0.01
fragment_220.1025	: 0.01
fragment_273.1125	: 0.01
fragment_119.0875	: 0.01
fragment_188.0825	: 0.01
fragment_173.0575	: 0.01
fragment_129.0675	: 0.01
fragment_135.0825	: 0.01
fragment_290.1375	: 0.01
fragment_246.1225	: 0.01

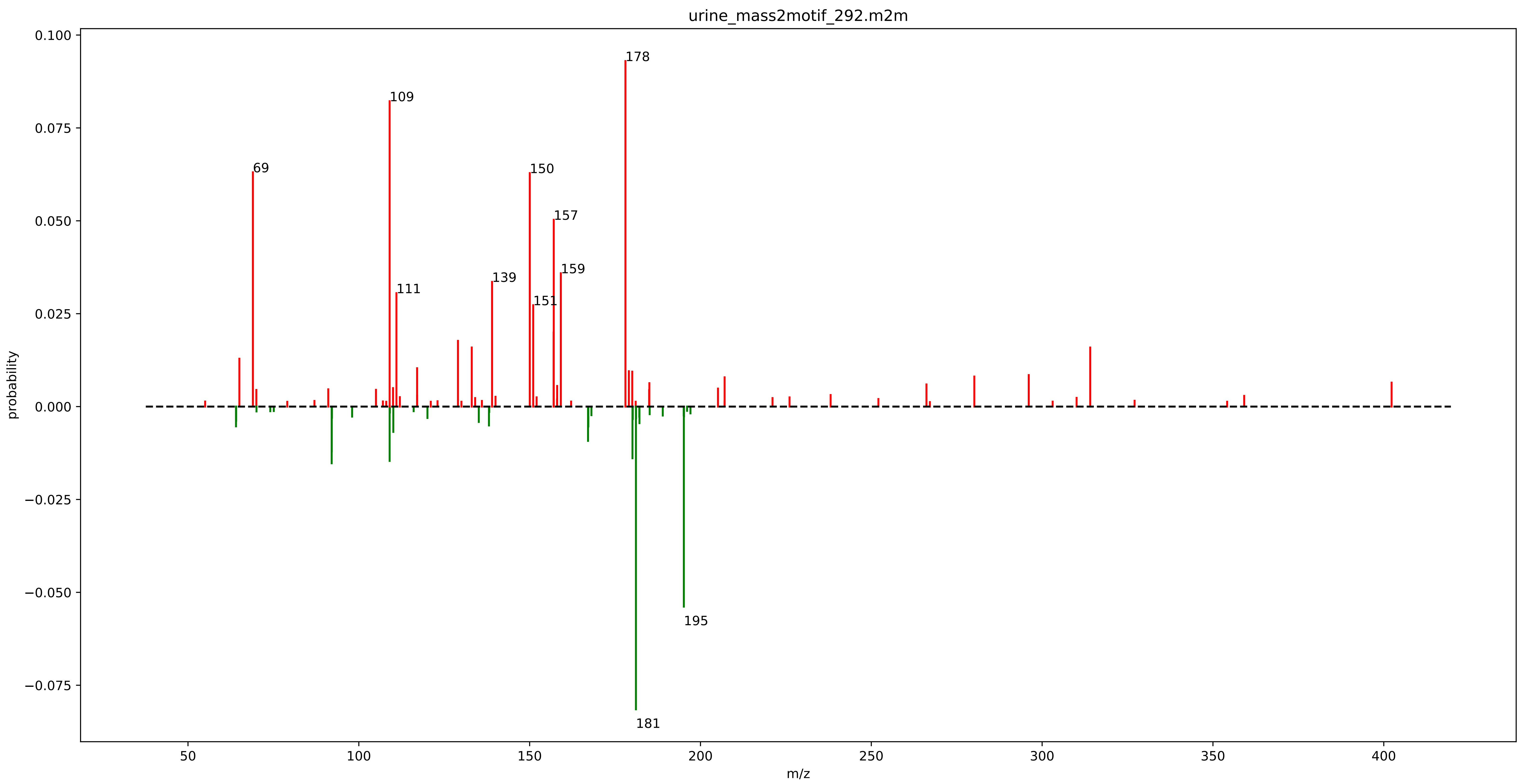
urine_mass2motif_292.m2m
probability
m/z
0.100
0.075
0.050
0.025
0.000
−0.025
−0.050
−0.075
50
100
150
200
250
300
350
400
178
109
69
150
157
159
139
111
151
195
181

urine_mass2motif_292.m2m

fragment_178.0325	: 0.09
fragment_109.0125	: 0.08
loss_181.1075	: 0.08
fragment_68.9975	: 0.06
fragment_150.0375	: 0.06
loss_195.1275	: 0.05
fragment_157.0525	: 0.05
fragment_159.1175	: 0.04
fragment_139.0025	: 0.03
fragment_111.0075	: 0.03
fragment_151.0375	: 0.03
fragment_157.0125	: 0.02
fragment_129.0175	: 0.02
fragment_133.0525	: 0.02
fragment_314.0825	: 0.02
loss_92.0475	: 0.02
loss_109.0175	: 0.01
loss_180.0975	: 0.01
fragment_65.0375	: 0.01
loss_92.0825	: 0.01
fragment_117.0575	: 0.01
fragment_179.0325	: 0.01
fragment_180.0275	: 0.01
loss_167.0925	: 0.01
fragment_296.0775	: 0.01
fragment_280.1525	: 0.01
fragment_207.0675	: 0.01
loss_110.0925	: 0.01
fragment_402.2875	: 0.01
fragment_185.0425	: 0.01
fragment_266.1375	: 0.01
fragment_158.0575	: 0.01
loss_167.1775	: 0.01
loss_64.0525	: 0.01
loss_138.0875	: 0.01

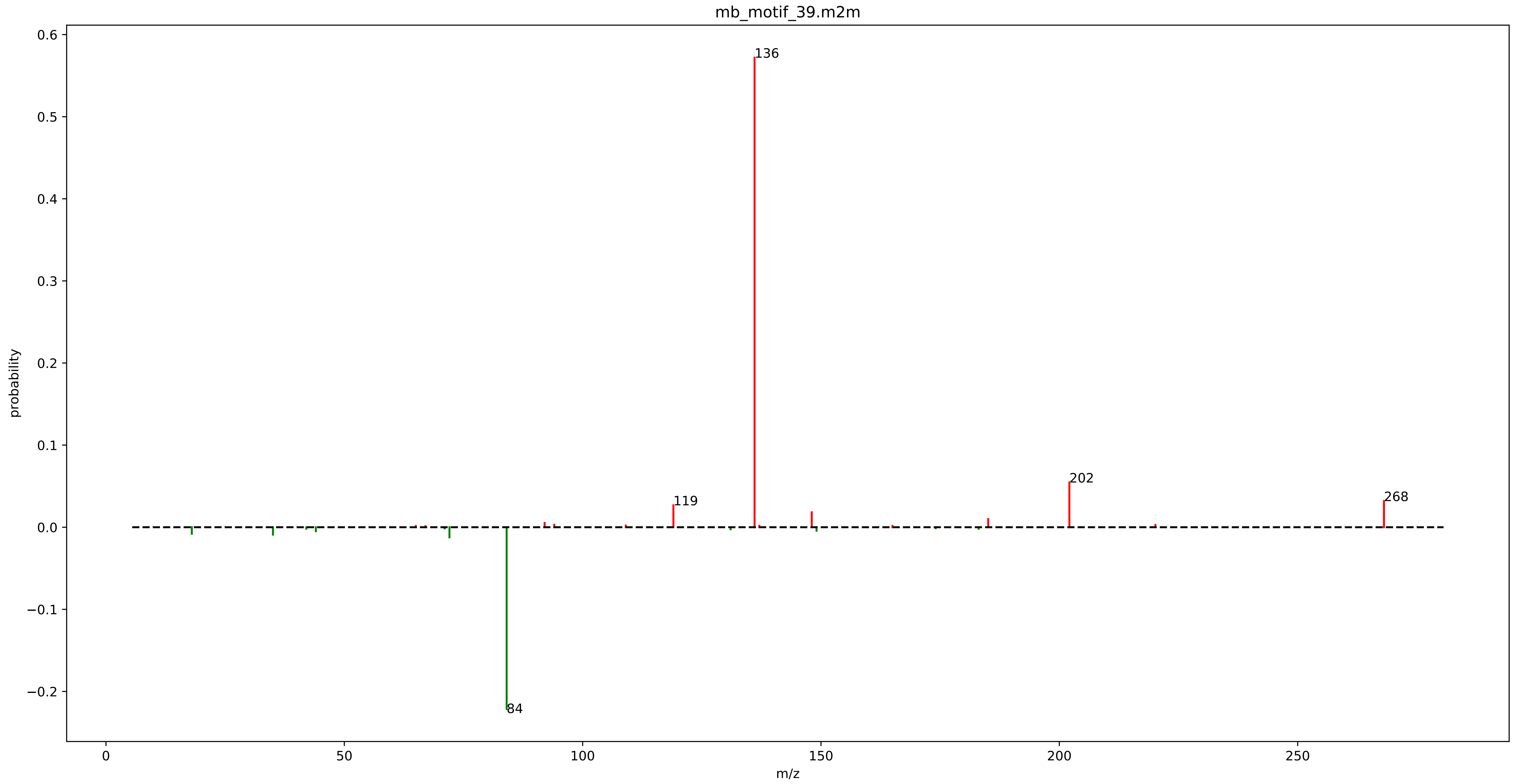

mb_motif_39.m2m
probability
m/z
136
119
202
268
84
0.6
0.5
0.4
0.3
0.2
0.1
0.0
−0.1
−0.2
0
50
100
150
200
250

mb_motif_39.m2m

fragment_136.0625	: 0.57
loss_84.0575	: 0.22
fragment_202.1075	: 0.05
fragment_268.1025	: 0.03
fragment_119.0375	: 0.03
fragment_148.0625	: 0.02
loss_72.0575	: 0.01
fragment_185.0825	: 0.01
loss_35.0375	: 0.01
loss_18.0125	: 0.01
fragment_92.0225	: 0.01

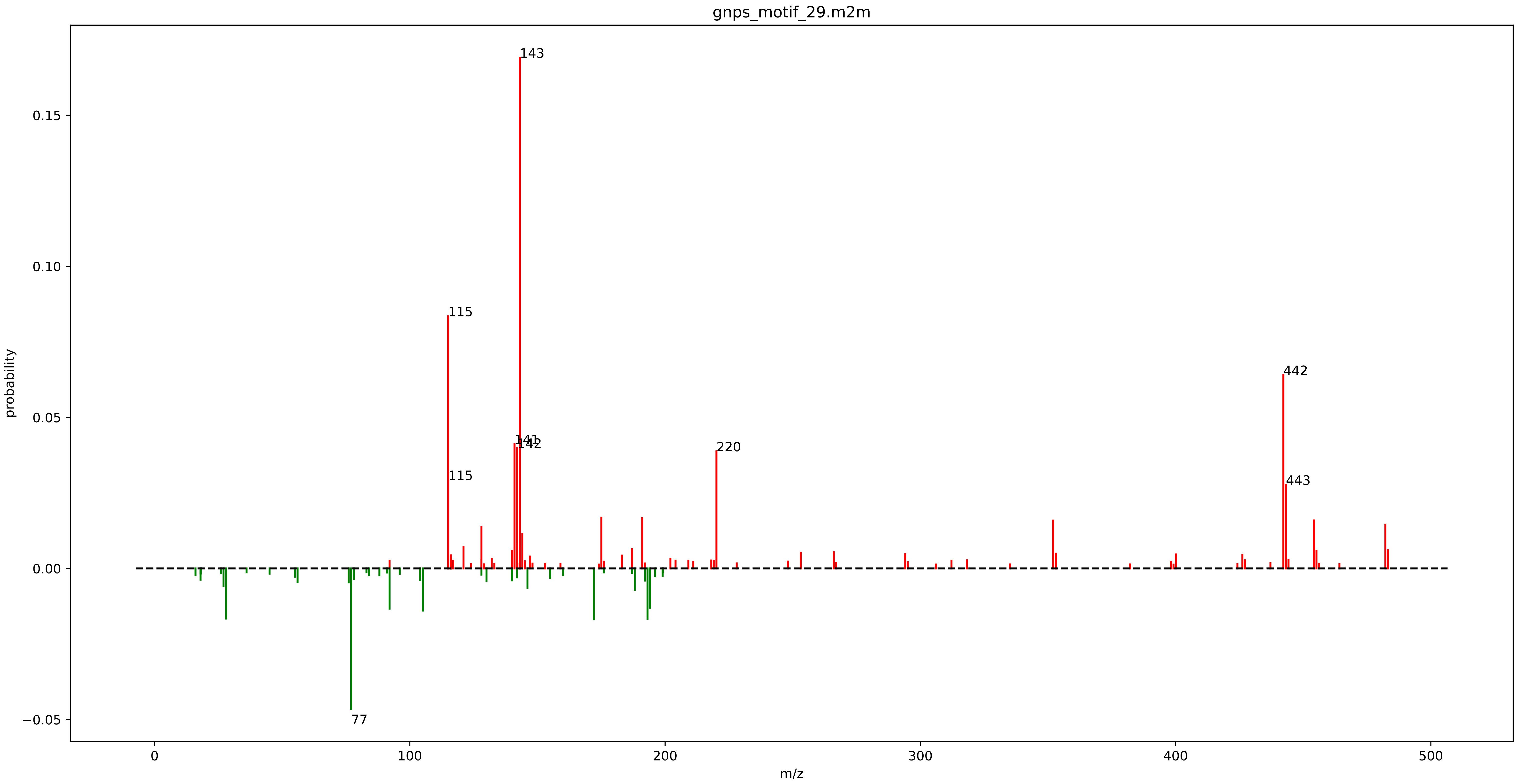

gnps_motif_29.m2m
probability
m/z
0.15
0.10
0.05
0.00
−0.05
0
100
200
300
400
500
143
115
141
142
220
115
442
443
77

gnps_motif_29.m2m

fragment_143.0725	: 0.17
fragment_115.0325	: 0.08
fragment_442.2225	: 0.06
loss_77.0375	: 0.05
fragment_141.0125	: 0.04
fragment_142.0675	: 0.04
fragment_220.1125	: 0.04
fragment_115.0525	: 0.03
fragment_443.2275	: 0.03
loss_172.0625	: 0.02
fragment_175.0375	: 0.02
loss_193.1225	: 0.02
fragment_191.0325	: 0.02
loss_28.0075	: 0.02
fragment_454.1775	: 0.02
fragment_352.0425	: 0.02
fragment_482.1875	: 0.01
loss_105.0575	: 0.01
fragment_128.0625	: 0.01
loss_92.0475	: 0.01
loss_194.1325	: 0.01
fragment_144.0825	: 0.01
fragment_142.0475	: 0.01
fragment_121.0275	: 0.01
fragment_143.0625	: 0.01
loss_188.0675	: 0.01
loss_146.0825	: 0.01
fragment_187.0525	: 0.01
fragment_483.1825	: 0.01
loss_27.0025	: 0.01
fragment_455.1775	: 0.01
fragment_140.0475	: 0.01
fragment_266.0775	: 0.01
fragment_253.1225	: 0.01

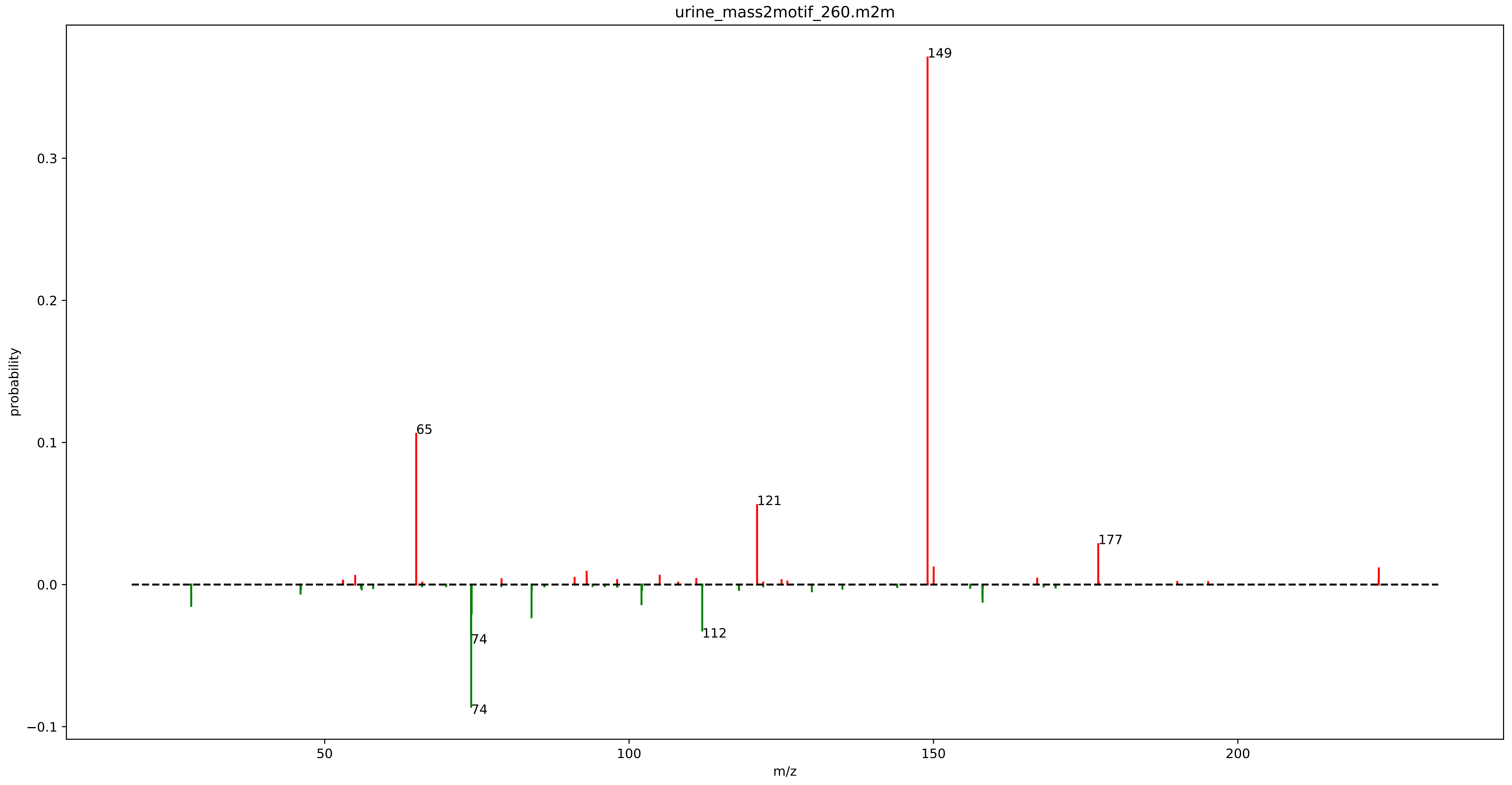

urine_mass2motif_260.m2m
probability
m/z
0.3
0.2
0.1
0.0
−0.1
50
100
150
200
149
65
121
177
74
74
112

urine_mass2motif_260.m2m

fragment_149.0225	: 0.37
fragment_65.0375	: 0.11
loss_74.0725	: 0.09
fragment_121.0275	: 0.06
loss_74.0475	: 0.04
loss_112.0175	: 0.03
fragment_177.0575	: 0.03
loss_83.9825	: 0.02
loss_74.1475	: 0.02
loss_28.0875	: 0.01
loss_102.0425	: 0.01
loss_158.0675	: 0.01
fragment_150.0275	: 0.01
fragment_223.1475	: 0.01
loss_74.1225	: 0.01
fragment_93.0325	: 0.01
loss_158.0375	: 0.01
loss_46.0375	: 0.01
fragment_105.0325	: 0.01
fragment_55.0175	: 0.01
loss_74.1075	: 0.01

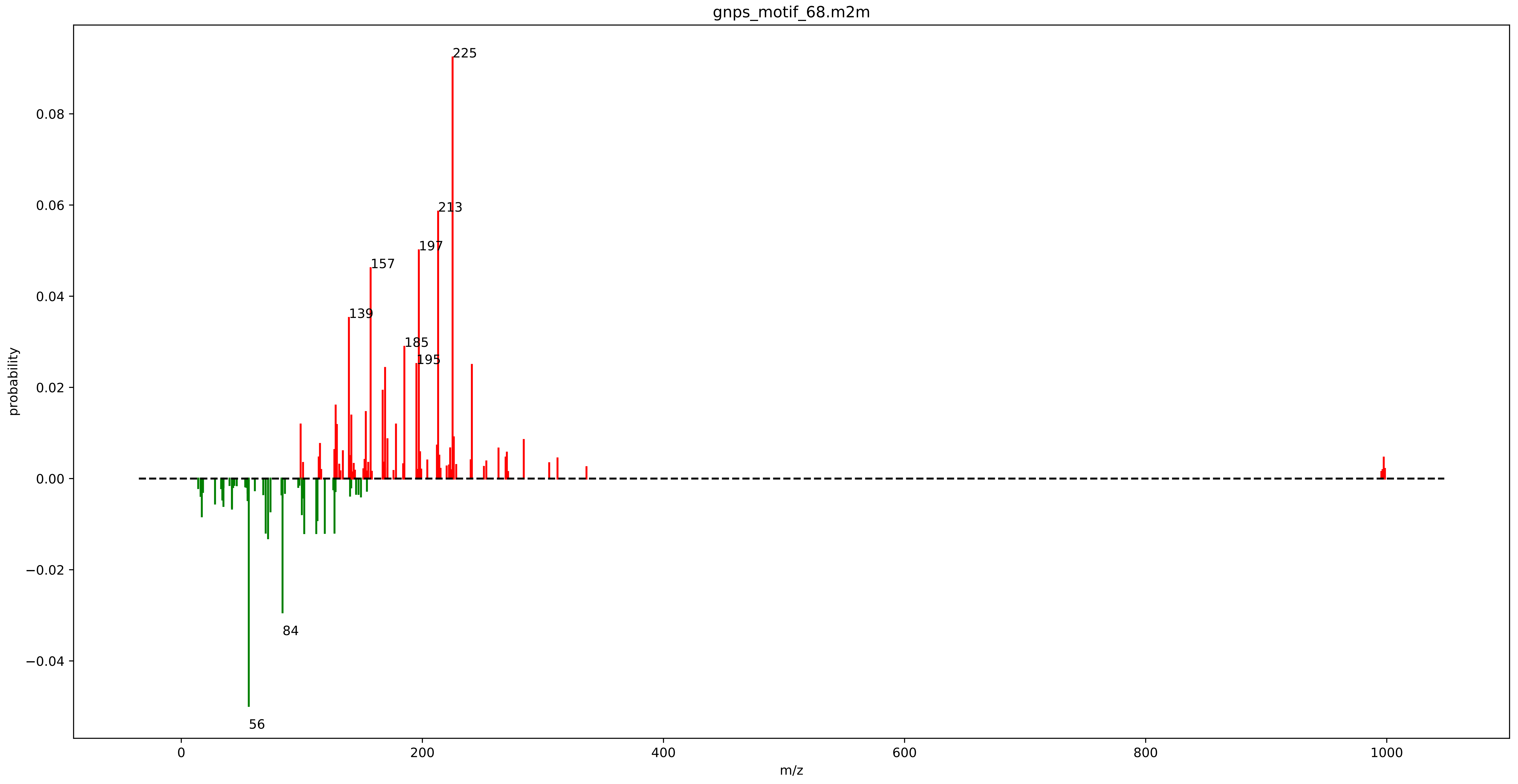

gnps_motif_68.m2m
probability
m/z
225
213
197
157
139
185
195
84
56
0.08
0.06
0.04
0.02
0.00
−0.02
−0.04
0
200
400
600
800
1000

gnps_motif_68.m2m

fragment_225.0525 : 0.09
fragment_213.0525 : 0.06
fragment_197.0575 : 0.05
loss_55.9875 : 0.05
fragment_157.0625 : 0.05
fragment_139.0525 : 0.04
loss_83.9875 : 0.03
fragment_185.0575 : 0.03
fragment_195.0425 : 0.03
fragment_241.0475 : 0.02
fragment_169.0625 : 0.02
fragment_167.0475 : 0.02
fragment_128.0625 : 0.02
fragment_153.0675 : 0.01
fragment_141.0675 : 0.01
loss_71.9825 : 0.01
loss_101.9975 : 0.01
loss_111.9775 : 0.01
loss_119.0375 : 0.01
loss_127.0975 : 0.01
loss_70.0175 : 0.01
fragment_99.0025 : 0.01
fragment_178.0975 : 0.01
fragment_129.0675 : 0.01
loss_112.9875 : 0.01
fragment_226.0575 : 0.01
fragment_169.0175 : 0.01
fragment_171.0425 : 0.01
fragment_284.0925 : 0.01
loss_17.0225 : 0.01
loss_100.0175 : 0.01
fragment_115.0525 : 0.01
fragment_212.0475 : 0.01
loss_74.0025 : 0.01
fragment_223.0525 : 0.01
fragment_263.1525 : 0.01
loss_42.0475 : 0.01
loss_42.0225 : 0.01
fragment_126.9975 : 0.01
fragment_223.0475 : 0.01
loss_34.9675 : 0.01
fragment_134.0475 : 0.01
fragment_198.0675 : 0.01
fragment_270.0775 : 0.01
loss_27.9925 : 0.01
fragment_214.0625 : 0.01

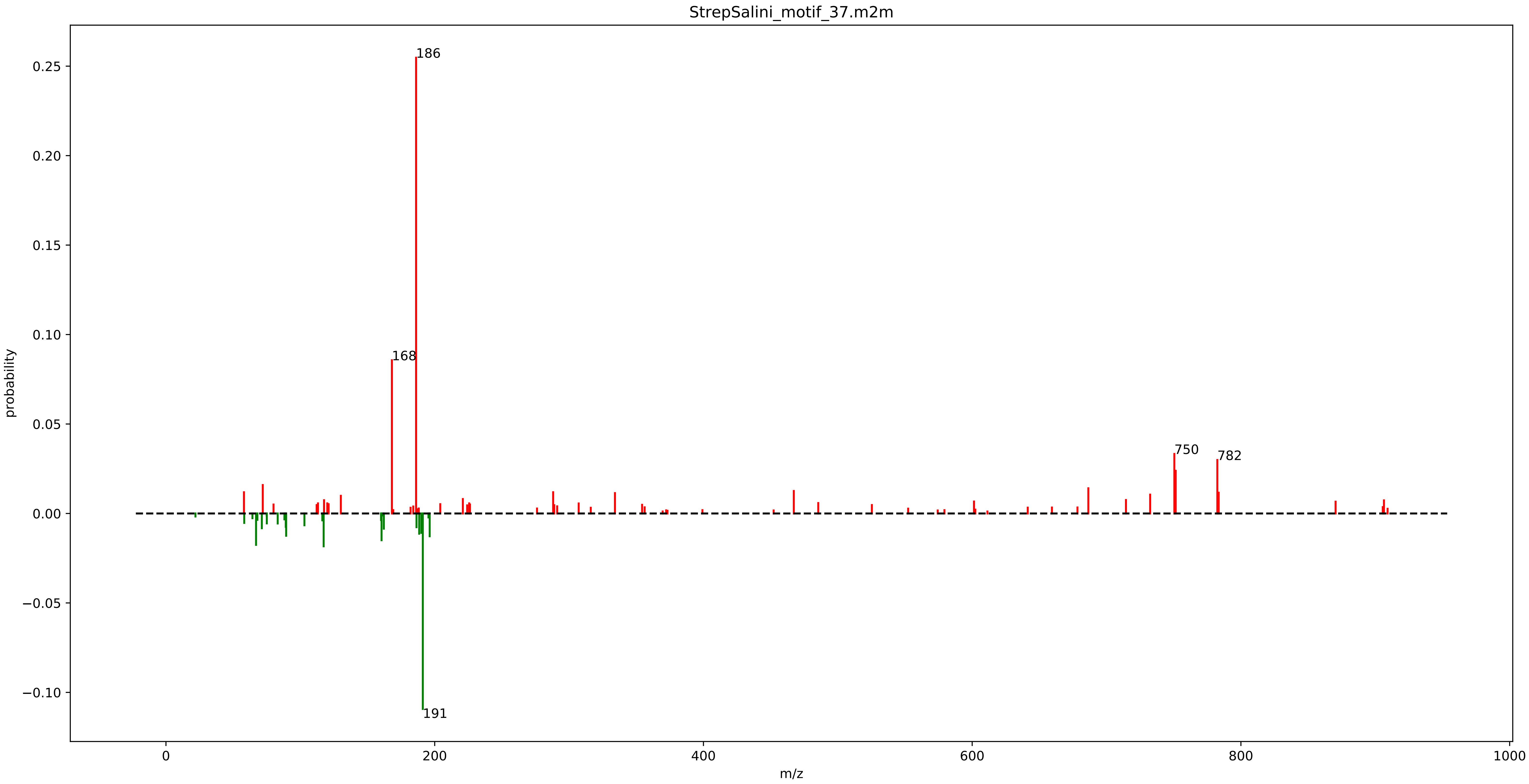

StrepSalini_motif_37.m2m
probability
m/z
186
168
750
782
191
0.25
0.20
0.15
0.10
0.05
0.00
−0.05
−0.10
0
200
400
600
800
1000

StrepSalini_motif_37.m2m

fragment_186.1500	: 0.25
loss_191.1525	: 0.11
fragment_168.1500	: 0.09
fragment_750.4500	: 0.03
fragment_782.4500	: 0.03
fragment_751.4500	: 0.02
loss_117.3500	: 0.02
loss_67.0500	: 0.02
fragment_72.0500	: 0.02
loss_160.4500	: 0.01
fragment_686.4500	: 0.01
loss_196.2525	: 0.01
fragment_467.2500	: 0.01
loss_89.4500	: 0.01
fragment_288.1500	: 0.01
fragment_58.0500	: 0.01
fragment_783.4500	: 0.01
fragment_334.1500	: 0.01
loss_188.4500	: 0.01
loss_190.0500	: 0.01
fragment_732.4500	: 0.01
fragment_130.1500	: 0.01
loss_162.1500	: 0.01
loss_71.3500	: 0.01
fragment_220.9500	: 0.01
loss_186.4500	: 0.01
fragment_714.4500	: 0.01
loss_89.1500	: 0.01
fragment_117.6500	: 0.01
fragment_906.4500	: 0.01
fragment_601.3500	: 0.01
loss_103.0500	: 0.01
fragment_870.4500	: 0.01
fragment_485.4500	: 0.01
fragment_120.0500	: 0.01
fragment_113.1500	: 0.01
fragment_225.6500	: 0.01
loss_83.1500	: 0.01
fragment_307.1500	: 0.01
loss_75.0475	: 0.01
loss_58.2500	: 0.01
fragment_204.1500	: 0.01
fragment_121.0525	: 0.01
fragment_226.2500	: 0.01

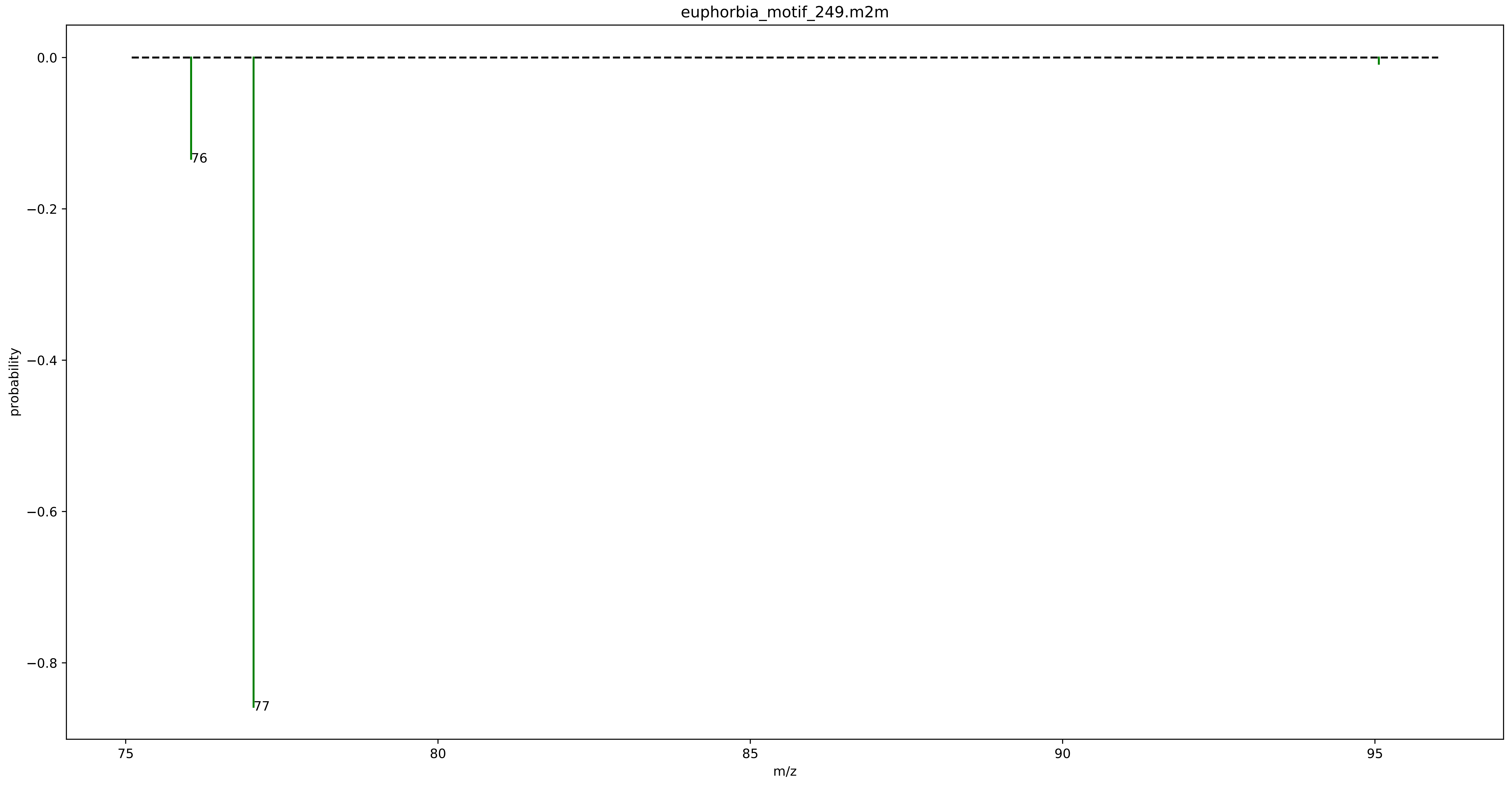

euphorbia_motif_249.m2m
0.0
−0.2
−0.4
−0.6
−0.8
probability
76
77
75
80
85
90
95
m/z

euphorbia_motif_249.m2m

loss_77.0475	: 0.86
loss_76.0475	: 0.13
loss_95.0625	: 0.01

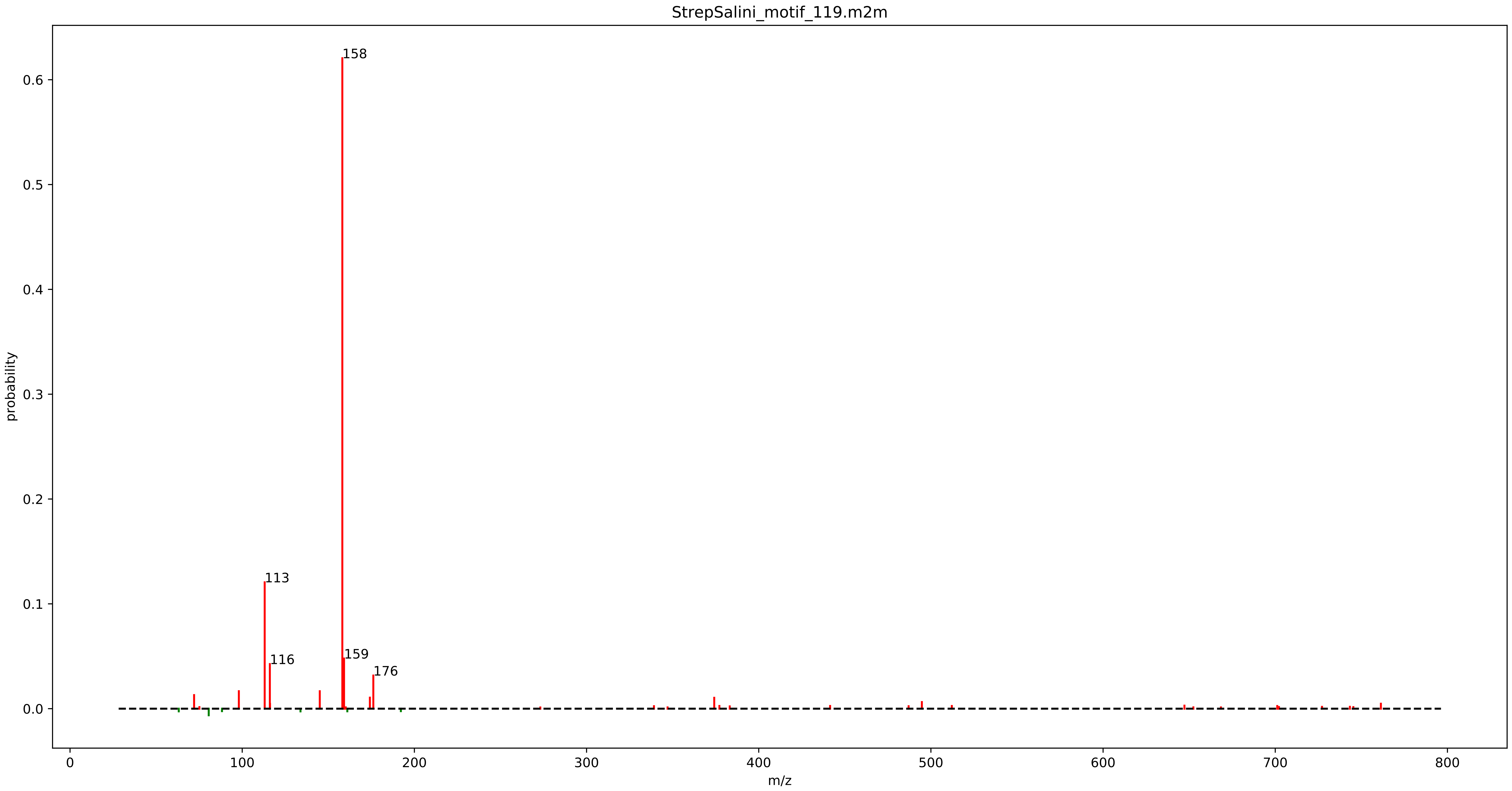

StrepSalini_motif_119.m2m
probability
m/z
158
113
116
159
176
0.0
0.1
0.2
0.3
0.4
0.5
0.6
0
100
200
300
400
500
600
700
800

StrepSalini_motif_119.m2m

fragment_158.1500	: 0.62
fragment_113.0500	: 0.12
fragment_159.1500	: 0.05
fragment_116.0525	: 0.04
fragment_176.1500	: 0.03
fragment_98.0500	: 0.02
fragment_145.0500	: 0.02
fragment_72.0500	: 0.01
fragment_174.1500	: 0.01
fragment_374.1500	: 0.01
fragment_494.7500	: 0.01
loss_80.5500	: 0.01

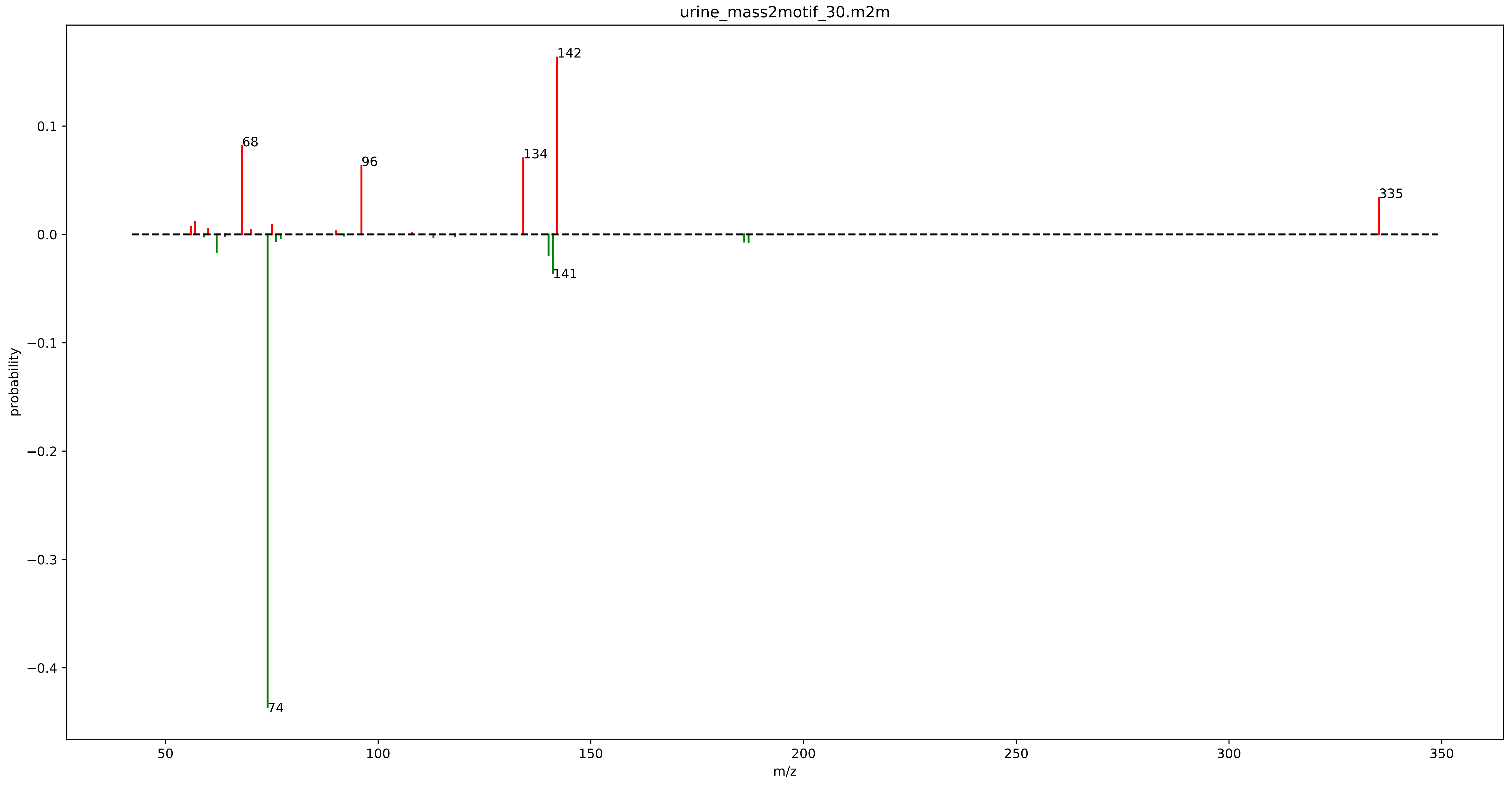

urine_mass2motif_30.m2m
probability
m/z
142
68
96
134
335
141
74
0.1
0.0
−0.1
−0.2
−0.3
−0.4
50
100
150
200
250
300
350

urine_mass2motif_30.m2m

loss_74.0225	: 0.44
fragment_142.0875	: 0.16
fragment_68.0475	: 0.08
fragment_134.1175	: 0.07
fragment_96.0825	: 0.06
loss_141.0775	: 0.04
fragment_335.2175	: 0.03
loss_140.0575	: 0.02
loss_62.0375	: 0.02
fragment_57.0325	: 0.01
fragment_75.0425	: 0.01
loss_187.0725	: 0.01
fragment_56.0475	: 0.01
loss_186.0625	: 0.01
loss_76.0525	: 0.01
fragment_60.0825	: 0.01

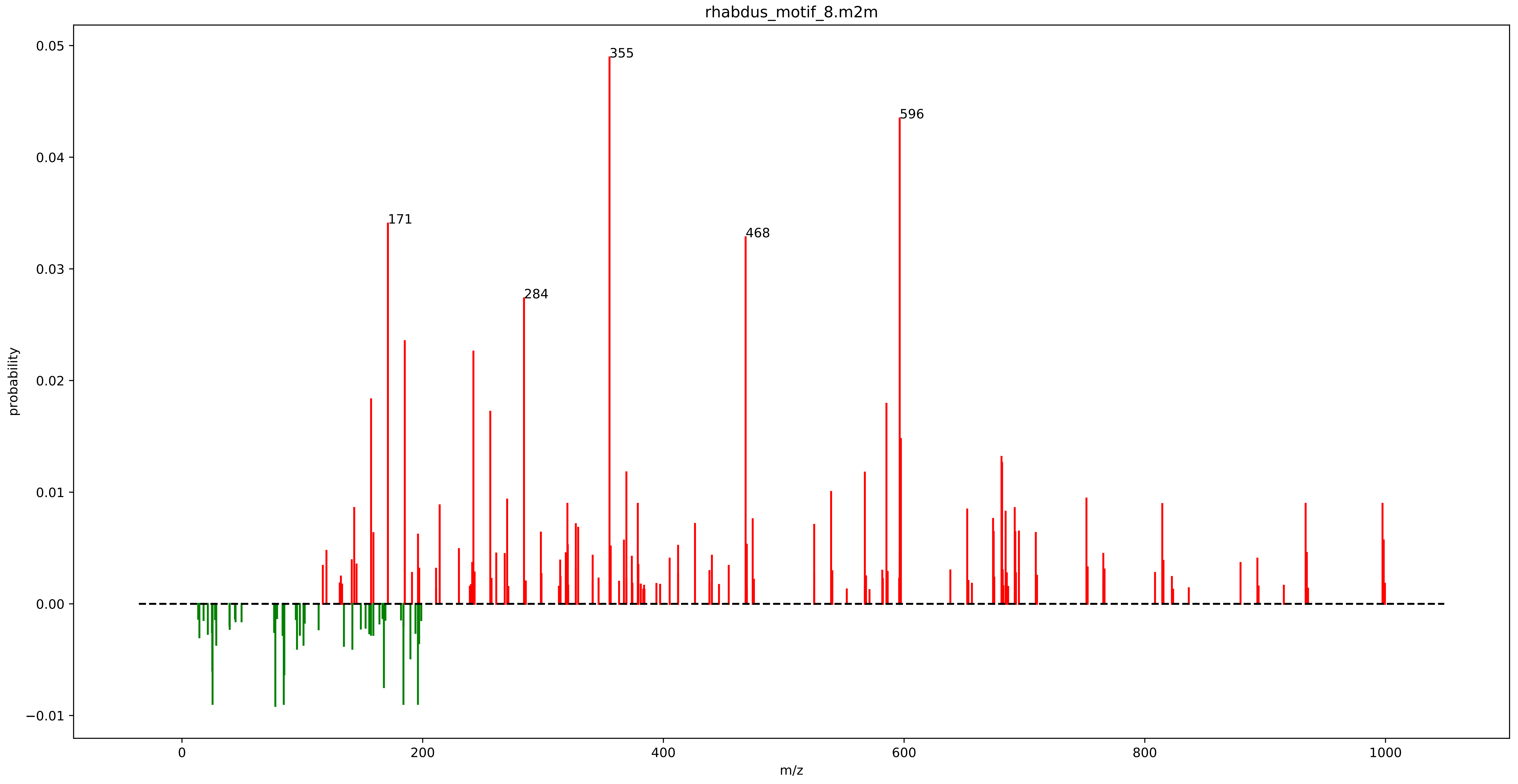

rhabdus_motif_8.m2m
probability
m/z
355
596
171
468
284
0.05
0.04
0.03
0.02
0.01
0.00
−0.01
0
200
400
600
800
1000

rhabdus_motif_8.m2m

fragment_355.2325	: 0.05
fragment_596.3425	: 0.04
fragment_171.1125	: 0.03
fragment_468.3175	: 0.03
fragment_284.1975	: 0.03
fragment_185.1325	: 0.02
fragment_242.1475	: 0.02
fragment_157.1325	: 0.02
fragment_585.3275	: 0.02
fragment_256.1675	: 0.02
fragment_597.3425	: 0.01
fragment_680.9325	: 0.01
fragment_681.4325	: 0.01
fragment_369.2525	: 0.01
fragment_567.3875	: 0.01
fragment_539.3525	: 0.01
fragment_751.5075	: 0.01
fragment_270.1825	: 0.01
loss_77.5825	: 0.01
loss_84.5875	: 0.01
fragment_933.6125	: 0.01
fragment_320.2375	: 0.01
fragment_997.5075	: 0.01
loss_184.0175	: 0.01
loss_196.0525	: 0.01
fragment_378.7275	: 0.01
loss_25.4275	: 0.01
fragment_814.5075	: 0.01
fragment_214.1175	: 0.01
fragment_143.1175	: 0.01
fragment_691.9225	: 0.01
fragment_652.4375	: 0.01
fragment_684.3475	: 0.01
fragment_673.9225	: 0.01
fragment_474.2075	: 0.01
loss_167.7825	: 0.01
fragment_185.1275	: 0.01
fragment_426.2625	: 0.01
fragment_327.2375	: 0.01
fragment_525.3025	: 0.01
fragment_329.1825	: 0.01
fragment_695.4075	: 0.01
fragment_674.4275	: 0.01
fragment_692.4275	: 0.01
fragment_298.2125	: 0.01
fragment_709.4225	: 0.01
fragment_159.0775	: 0.01
loss_85.0475	: 0.01
fragment_196.1075	: 0.01
loss_25.1775	: 0.01
fragment_998.5125	: 0.01
fragment_367.2325	: 0.01
fragment_469.3225	: 0.01
fragment_320.5775	: 0.01
fragment_412.2225	: 0.01
fragment_356.2375	: 0.01

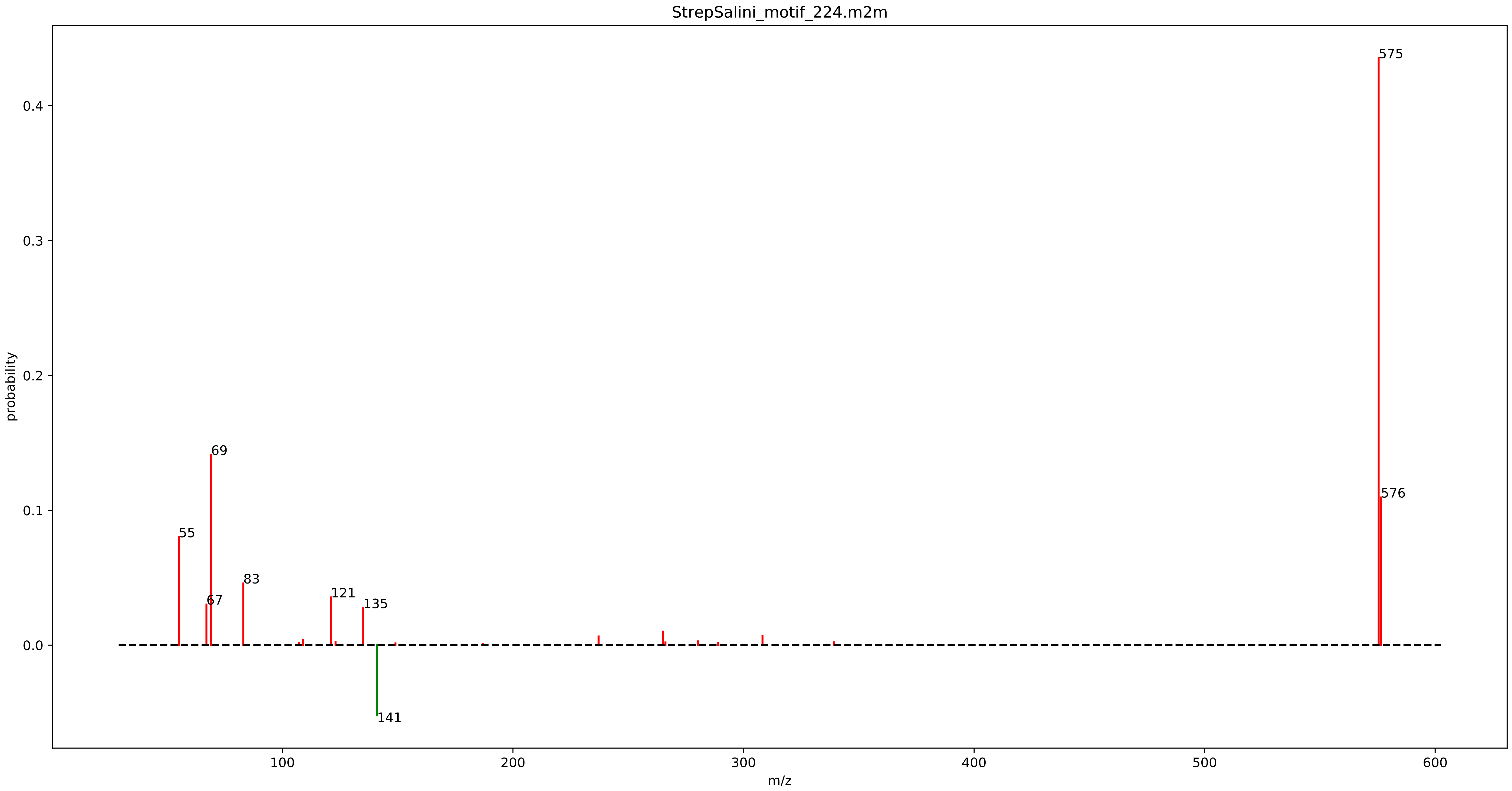

StrepSalini_motif_224.m2m
probability
m/z
575
576
69
55
83
67
121
135
141
0.4
0.3
0.2
0.1
0.0
100
200
300
400
500
600

StrepSalini_motif_224.m2m

fragment_575.4500	: 0.44
fragment_69.0500	: 0.14
fragment_576.4500	: 0.11
fragment_55.0500	: 0.08
loss_141.0525	: 0.05
fragment_83.0500	: 0.05
fragment_121.0525	: 0.04
fragment_67.0500	: 0.03
fragment_135.0500	: 0.03
fragment_265.1525	: 0.01
fragment_308.2500	: 0.01
fragment_237.1500	: 0.01

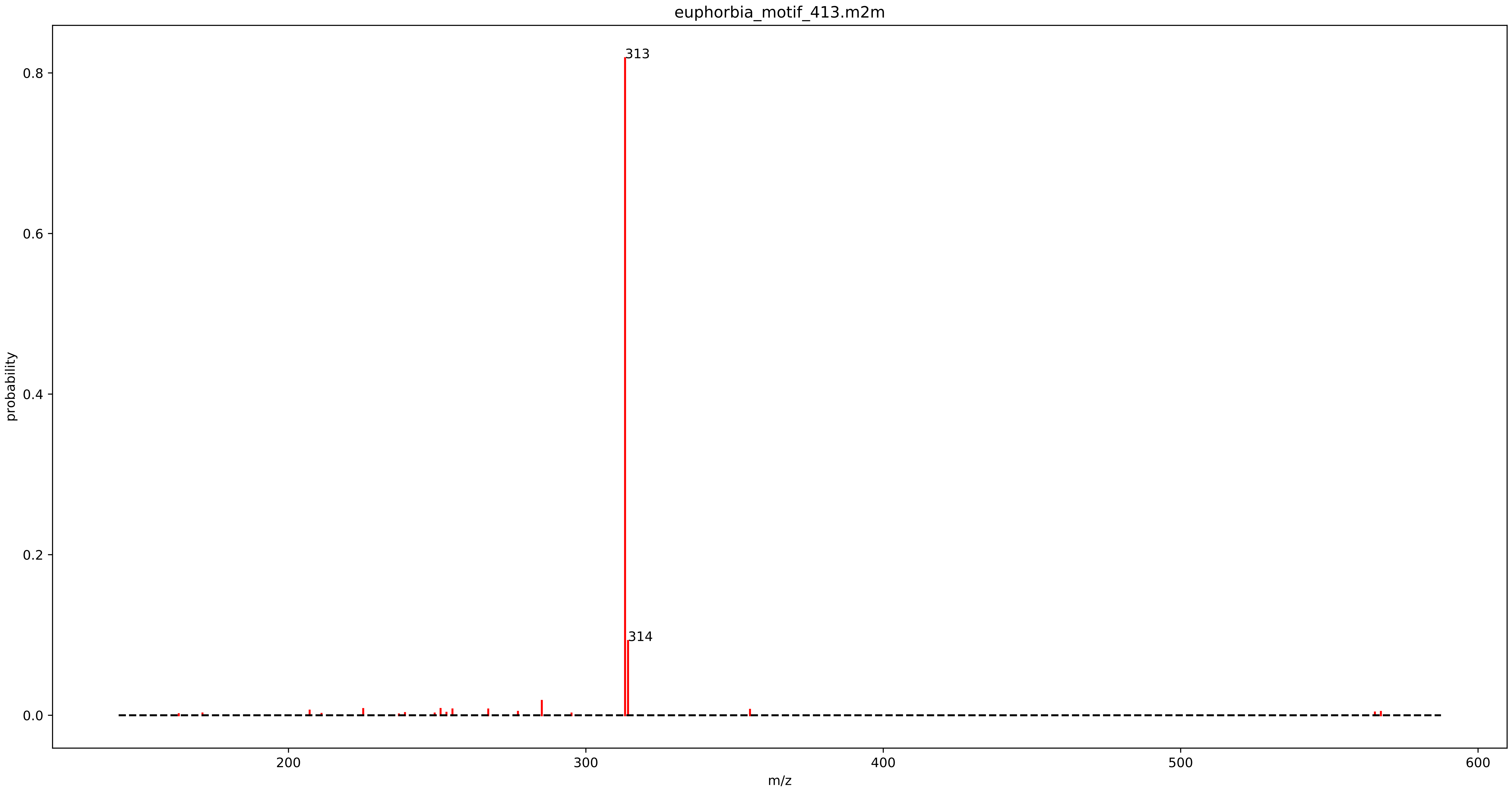
euphorbia_motif_413.m2m
313
314
probability
0.0
0.2
0.4
0.6
0.8
200
300
400
500
600
m/z

euphorbia_motif_413.m2m

fragment_313.1775	: 0.82
fragment_314.1825	: 0.09
fragment_285.1825	: 0.02
fragment_251.1425	: 0.01
fragment_225.1275	: 0.01
fragment_255.1375	: 0.01
fragment_267.1725	: 0.01
fragment_355.1875	: 0.01
fragment_207.1175	: 0.01

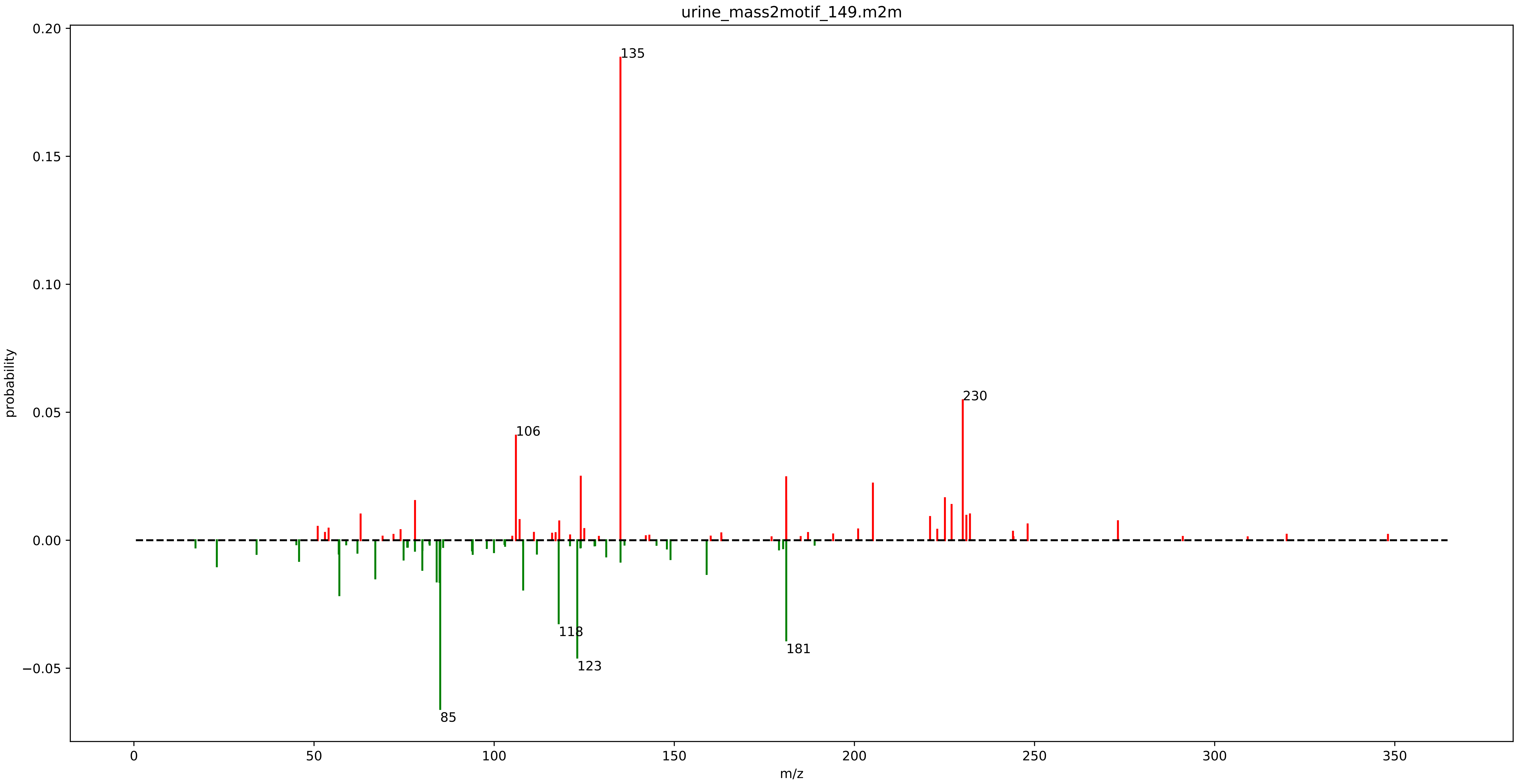

urine_mass2motif_149.m2m
probability
m/z
0.20
0.15
0.10
0.05
0.00
−0.05
0
50
100
150
200
250
300
350
135
230
106
118
123
181
85

urine_mass2motif_149.m2m

fragment_135.0525	: 0.19	loss_80.0575	: 0.01
loss_85.0275	: 0.07	loss_23.0175	: 0.01
fragment_230.0725	: 0.05	fragment_232.0725	: 0.01
loss_123.0675	: 0.05	fragment_62.9275	: 0.01
fragment_106.0375	: 0.04	fragment_231.0775	: 0.01
loss_181.0825	: 0.04	fragment_220.9975	: 0.01
loss_117.9125	: 0.03	loss_135.0775	: 0.01
fragment_124.0375	: 0.02	loss_45.8425	: 0.01
fragment_181.0625	: 0.02	fragment_107.0625	: 0.01
fragment_205.1325	: 0.02	loss_74.8575	: 0.01
loss_57.0275	: 0.02	fragment_273.1475	: 0.01
loss_108.0525	: 0.02	loss_148.9525	: 0.01
fragment_225.1225	: 0.02	fragment_118.0675	: 0.01
loss_84.8525	: 0.02	loss_131.1075	: 0.01
loss_84.0425	: 0.02	fragment_248.0825	: 0.01
fragment_181.0925	: 0.02	loss_34.0525	: 0.01
fragment_78.0325	: 0.02	loss_94.0425	: 0.01
loss_67.0075	: 0.01	fragment_51.0225	: 0.01
fragment_226.9775	: 0.01	loss_111.8625	: 0.01
loss_158.9825	: 0.01	loss_56.8575	: 0.01

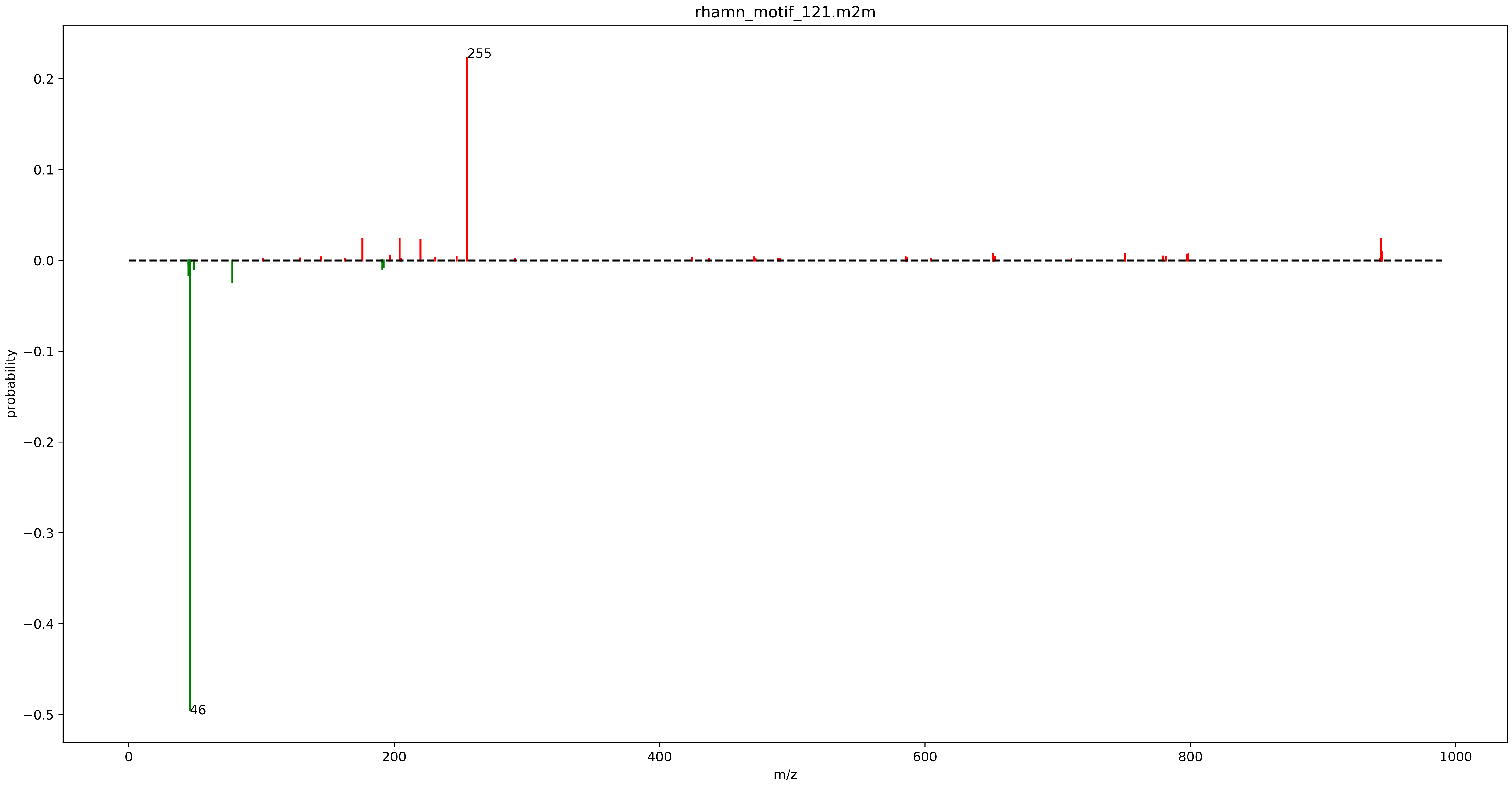

rhamn_motif_121.m2m
255
46
probability
m/z
0.2
0.1
0.0
−0.1
−0.2
−0.3
−0.4
−0.5
0
200
400
600
800
1000

rhamn_motif_121.m2m

loss_46.0025	: 0.49
fragment_255.0325	: 0.22
fragment_176.0525	: 0.02
loss_78.0325	: 0.02
fragment_943.5275	: 0.02
fragment_204.1125	: 0.02
fragment_219.7975	: 0.02
loss_44.9875	: 0.02
loss_49.0275	: 0.01
fragment_944.5375	: 0.01
loss_191.0575	: 0.01
loss_192.0675	: 0.01
fragment_651.4175	: 0.01
fragment_798.4725	: 0.01
fragment_750.4625	: 0.01
fragment_797.4625	: 0.01
fragment_197.0125	: 0.01
loss_45.0125	: 0.01

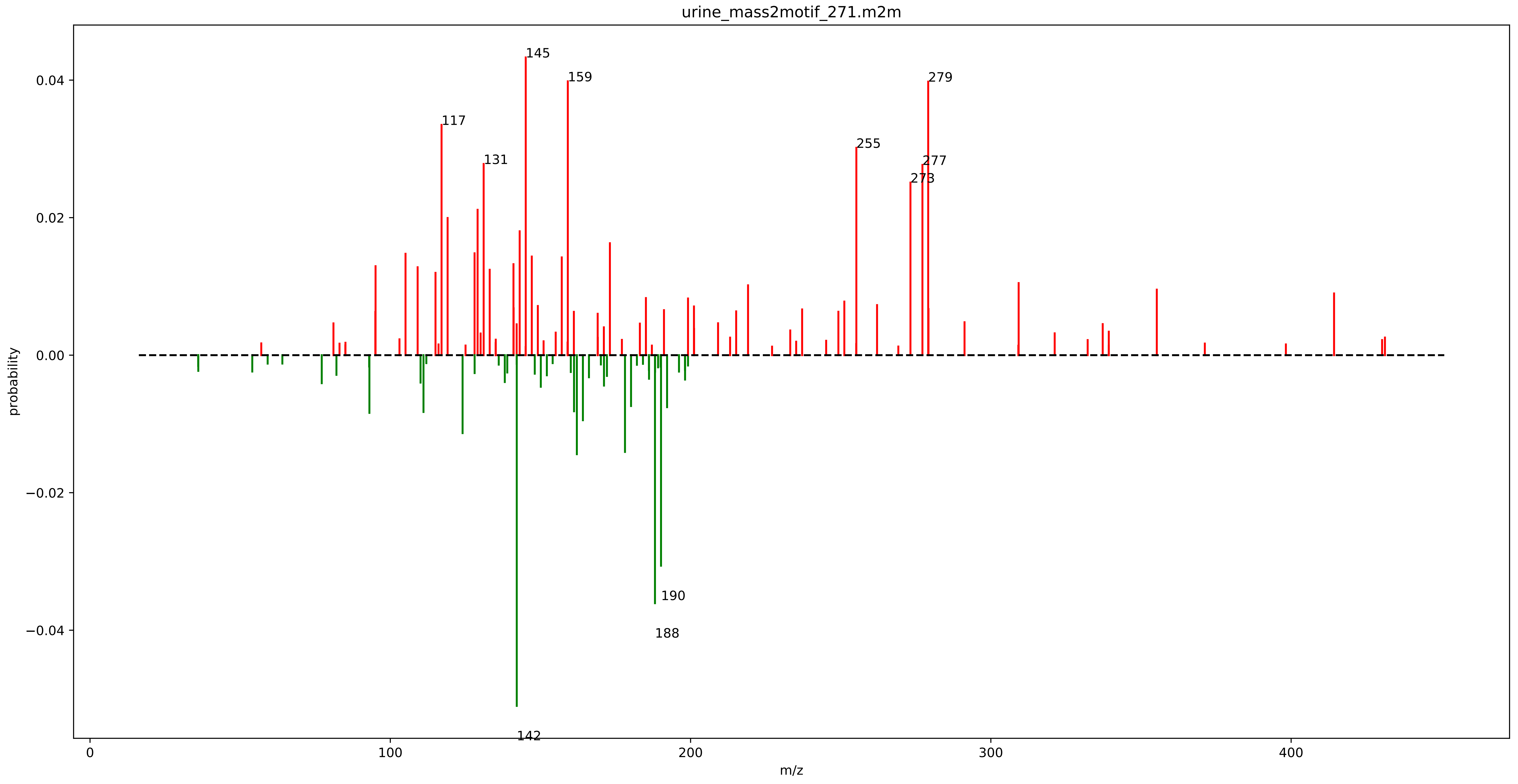
urine_mass2motif_271.m2m
probability
m/z
0.04
0.02
0.00
−0.02
−0.04
0
100
200
300
400
145
159
279
117
131
255
277
273
190
188
142

urine_mass2motif_271.m2m

loss_142.1125	: 0.05
fragment_145.1025	: 0.04
fragment_159.1175	: 0.04
fragment_279.1325	: 0.04
loss_188.1425	: 0.04
fragment_117.0675	: 0.03
loss_190.1575	: 0.03
fragment_255.2075	: 0.03
fragment_131.0875	: 0.03
fragment_277.2125	: 0.03
fragment_273.2225	: 0.03
fragment_129.0725	: 0.02
fragment_119.0875	: 0.02
fragment_143.0825	: 0.02
fragment_173.1325	: 0.02
fragment_128.0625	: 0.01
fragment_105.0675	: 0.01
loss_162.1275	: 0.01
fragment_147.1175	: 0.01
fragment_157.0975	: 0.01
loss_178.1575	: 0.01
fragment_141.0175	: 0.01
fragment_95.0875	: 0.01
fragment_109.1025	: 0.01
fragment_133.1025	: 0.01
fragment_115.0525	: 0.01
loss_124.0725	: 0.01
fragment_309.2575	: 0.01
fragment_219.1375	: 0.01
fragment_355.2625	: 0.01
loss_164.1425	: 0.01
fragment_414.2975	: 0.01
loss_93.0425	: 0.01
fragment_185.1275	: 0.01
loss_111.0525	: 0.01
fragment_199.1475	: 0.01
loss_161.1875	: 0.01
fragment_251.2025	: 0.01
loss_192.1725	: 0.01
loss_180.1725	: 0.01
fragment_262.1025	: 0.01
fragment_149.1325	: 0.01
fragment_201.1275	: 0.01
fragment_141.0675	: 0.01
fragment_279.2325	: 0.01
fragment_237.1475	: 0.01
fragment_191.1425	: 0.01
fragment_215.1825	: 0.01
fragment_249.2225	: 0.01
fragment_161.1325	: 0.01
fragment_95.0125	: 0.01
fragment_169.0625	: 0.01

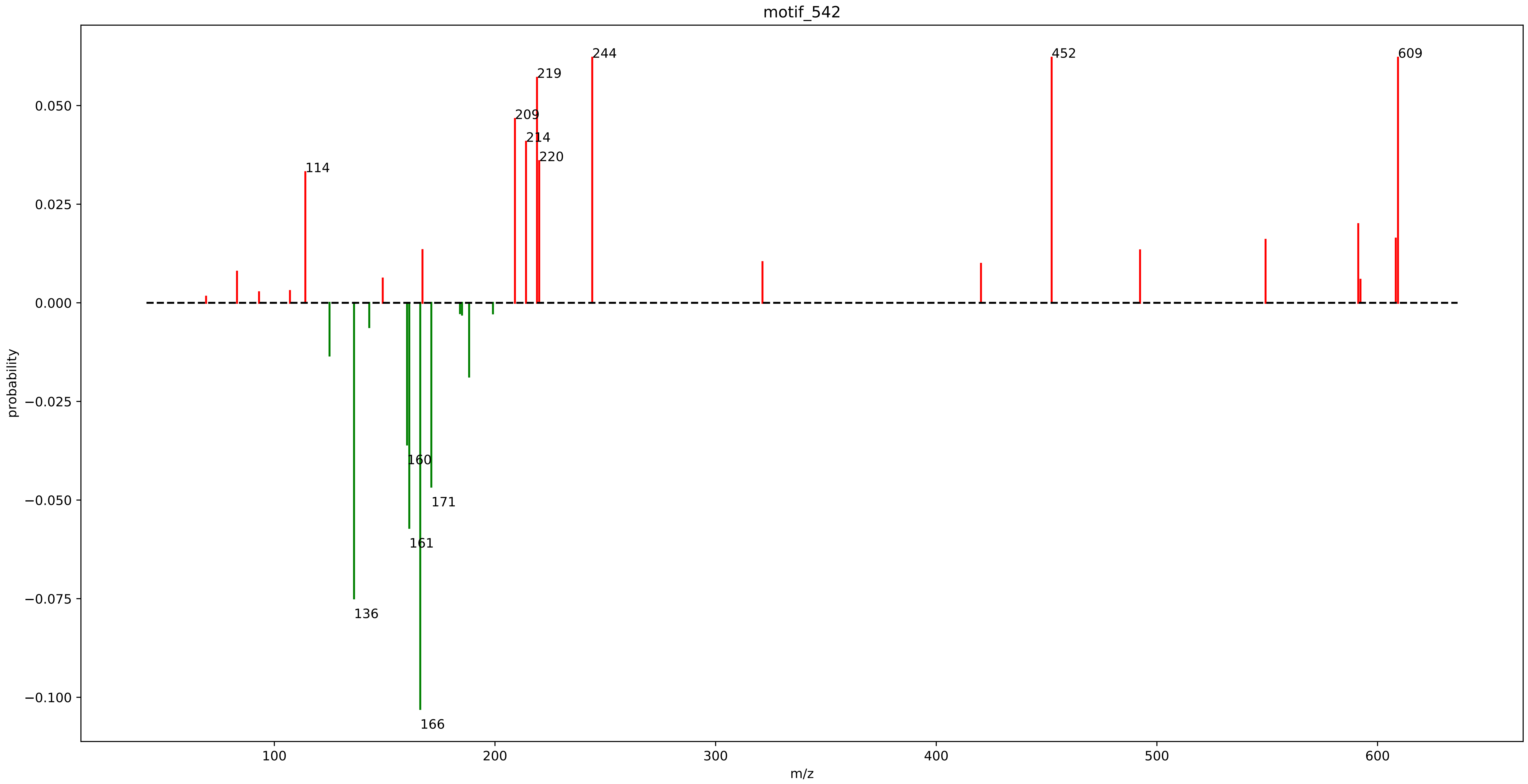
motif_542
probability
m/z
0.050
0.025
0.000
−0.025
−0.050
−0.075
−0.100
100
200
300
400
500
600
114
209
214
219
220
244
452
609
136
160
161
166
171

motif_542

loss_166.1375	: 0.10
loss_136.1275	: 0.07
fragment_609.2725	: 0.06
fragment_244.0825	: 0.06
fragment_452.2975	: 0.06
fragment_219.0475	: 0.06
loss_161.1625	: 0.06
fragment_209.0525	: 0.05
loss_171.1575	: 0.05
fragment_214.0725	: 0.04
loss_160.1575	: 0.04
fragment_220.0525	: 0.04
fragment_114.0525	: 0.03
fragment_591.2575	: 0.02
loss_188.2875	: 0.02
fragment_608.2625	: 0.02
fragment_549.2475	: 0.02
fragment_167.1425	: 0.01
loss_125.0125	: 0.01
fragment_492.3675	: 0.01
fragment_321.2375	: 0.01
fragment_420.2725	: 0.01
fragment_83.0825	: 0.01
fragment_149.1325	: 0.01
loss_143.0225	: 0.01
fragment_592.2625	: 0.01

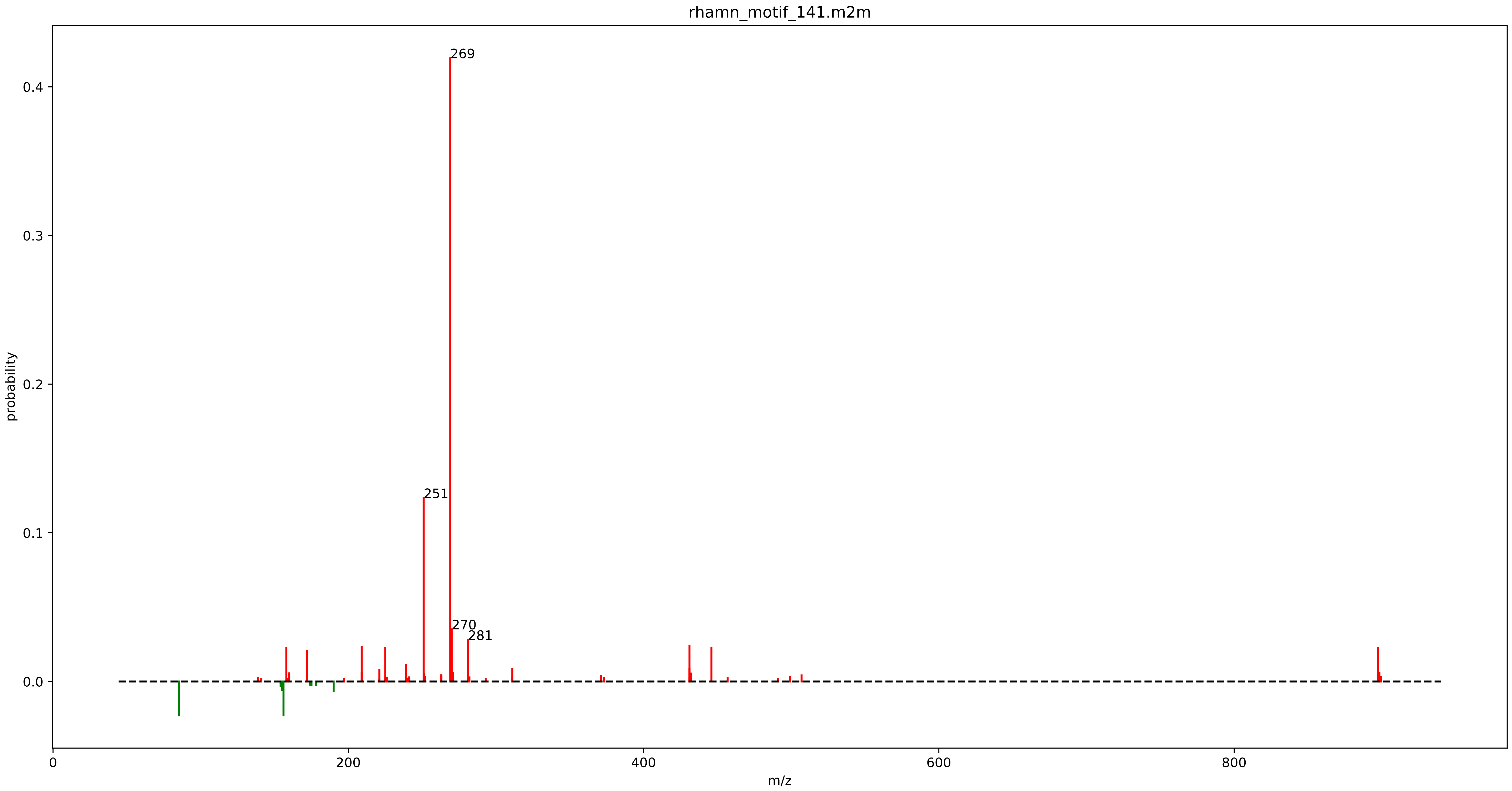
rhamn_motif_141.m2m
probability
m/z
0.4
0.3
0.2
0.1
0.0
0
200
400
600
800
269
251
270
281

rhamn_motif_141.m2m

fragment_269.0475	: 0.42
fragment_251.0525	: 0.12
fragment_270.0475	: 0.04
fragment_281.0625	: 0.03
fragment_431.1025	: 0.02
fragment_209.0475	: 0.02
loss_85.1725	: 0.02
fragment_445.9825	: 0.02
fragment_158.0825	: 0.02
fragment_897.3775	: 0.02
loss_156.1175	: 0.02
fragment_225.0575	: 0.02
fragment_171.9525	: 0.02
fragment_270.0525	: 0.01
fragment_239.0575	: 0.01
fragment_239.0525	: 0.01
fragment_311.0725	: 0.01
fragment_431.0875	: 0.01
fragment_221.0475	: 0.01
loss_190.0475	: 0.01
fragment_898.3925	: 0.01
loss_155.0975	: 0.01
fragment_271.0525	: 0.01
fragment_209.0325	: 0.01
fragment_160.0775	: 0.01
fragment_432.1025	: 0.01
fragment_251.1575	: 0.01

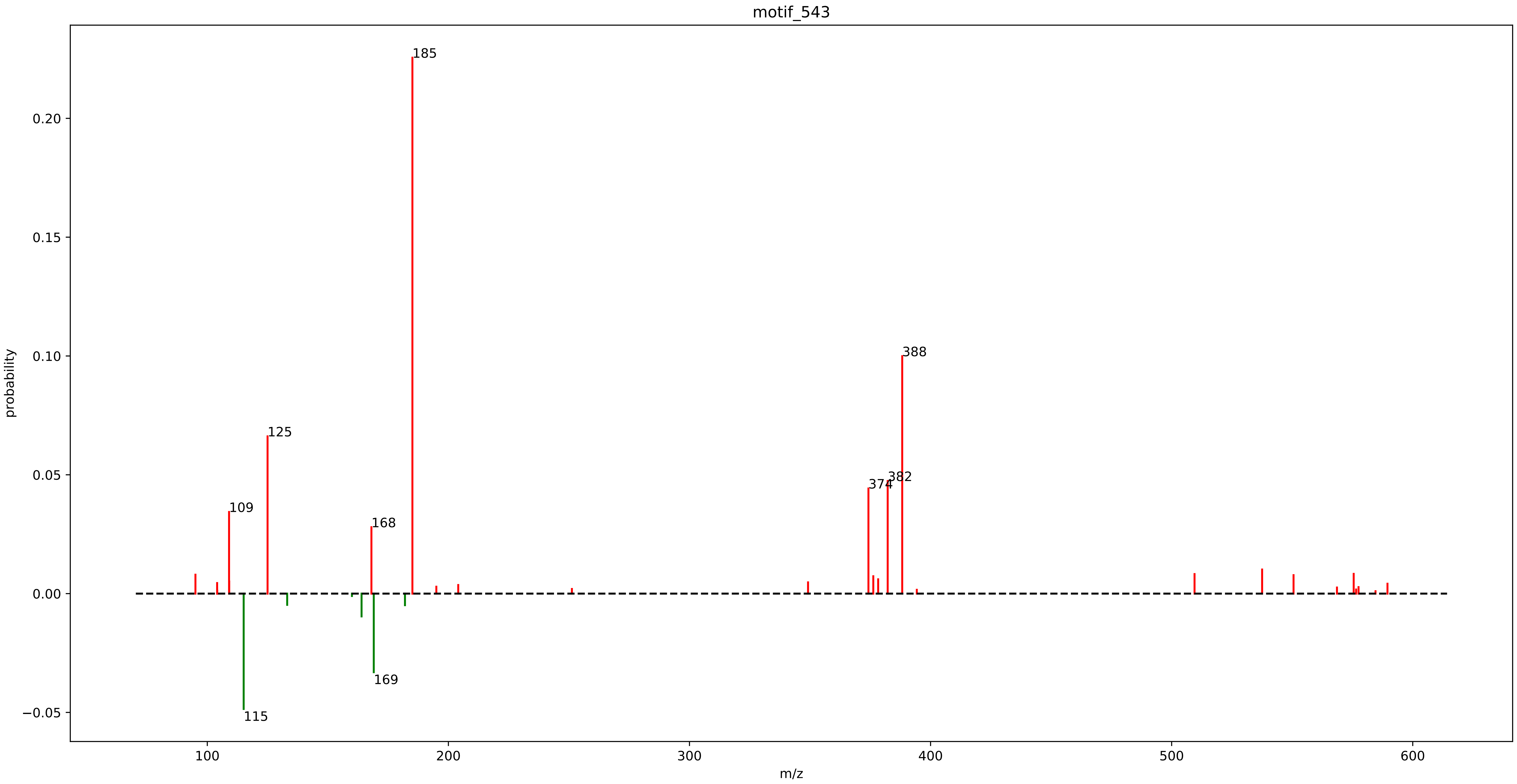

motif_543
probability
m/z
0.20
0.15
0.10
0.05
0.00
−0.05
100
200
300
400
500
600
185
125
109
168
115
169
374
382
388

motif_543

fragment_185.0775	: 0.23
fragment_388.2425	: 0.10
fragment_124.9975	: 0.07
loss_115.0875	: 0.05
fragment_382.2125	: 0.05
fragment_374.2275	: 0.04
fragment_109.0275	: 0.03
loss_169.0475	: 0.03
fragment_168.0775	: 0.03
fragment_537.5225	: 0.01
loss_163.9875	: 0.01
fragment_575.5025	: 0.01
fragment_509.4925	: 0.01
fragment_95.0825	: 0.01
fragment_550.5575	: 0.01
fragment_376.2425	: 0.01
fragment_378.2625	: 0.01
fragment_109.0975	: 0.01

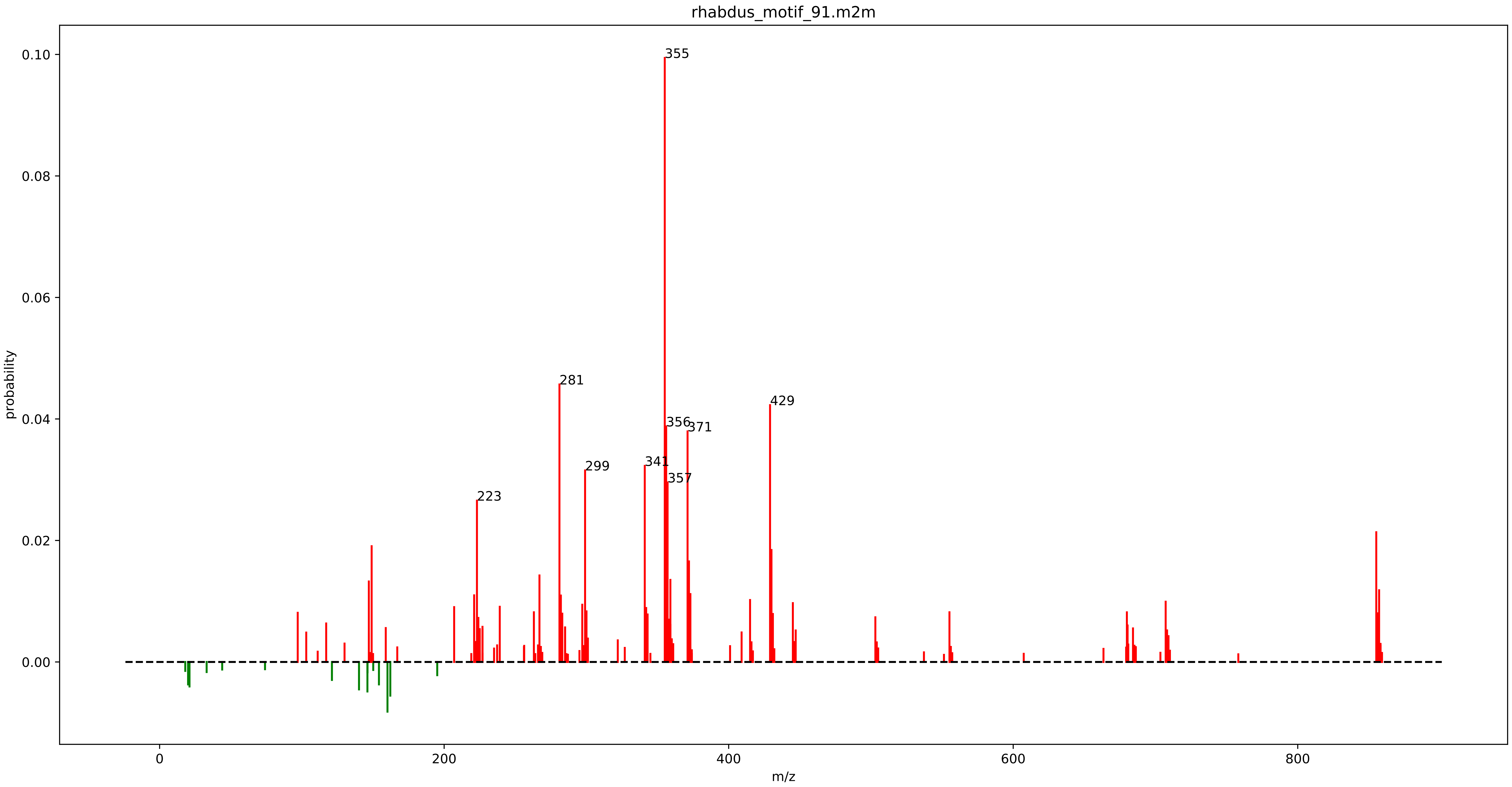
rhabdus_motif_91.m2m
probability
m/z
0.10
0.08
0.06
0.04
0.02
0.00
0
200
400
600
800
355
281
429
356
371
341
299
357
223

rhabdus_motif_91.m2m

fragment_355.0725	: 0.10
fragment_281.0525	: 0.05
fragment_429.0875	: 0.04
fragment_356.0725	: 0.04
fragment_371.1025	: 0.04
fragment_341.0175	: 0.03
fragment_299.0625	: 0.03
fragment_357.0675	: 0.03
fragment_223.0625	: 0.03
fragment_855.2075	: 0.02
fragment_149.0425	: 0.02
fragment_430.0875	: 0.02
fragment_372.1025	: 0.02
fragment_266.9975	: 0.01
fragment_359.0275	: 0.01
fragment_147.0625	: 0.01
fragment_857.2075	: 0.01
fragment_373.0975	: 0.01
fragment_221.0825	: 0.01
fragment_282.0525	: 0.01
fragment_415.0375	: 0.01
fragment_707.1675	: 0.01
fragment_445.1175	: 0.01
fragment_297.0825	: 0.01
fragment_239.0975	: 0.01
fragment_207.0325	: 0.01
fragment_342.0175	: 0.01
fragment_300.0625	: 0.01
fragment_263.0825	: 0.01
loss_160.1675	: 0.01
fragment_555.1875	: 0.01
fragment_679.9025	: 0.01
fragment_97.0775	: 0.01
fragment_856.2075	: 0.01
fragment_283.0475	: 0.01
fragment_431.0875	: 0.01
fragment_221.0875	: 0.01
fragment_343.0175	: 0.01
fragment_503.1075	: 0.01
fragment_856.2125	: 0.01
fragment_224.0625	: 0.01
fragment_358.0675	: 0.01
fragment_117.0875	: 0.01
fragment_359.0325	: 0.01
fragment_680.4025	: 0.01
fragment_226.9525	: 0.01
fragment_285.0075	: 0.01
fragment_285.0125	: 0.01
fragment_158.9625	: 0.01
loss_162.1625	: 0.01
fragment_684.2025	: 0.01
fragment_225.0425	: 0.01
fragment_226.9575	: 0.01
fragment_708.1675	: 0.01
fragment_447.1175	: 0.01

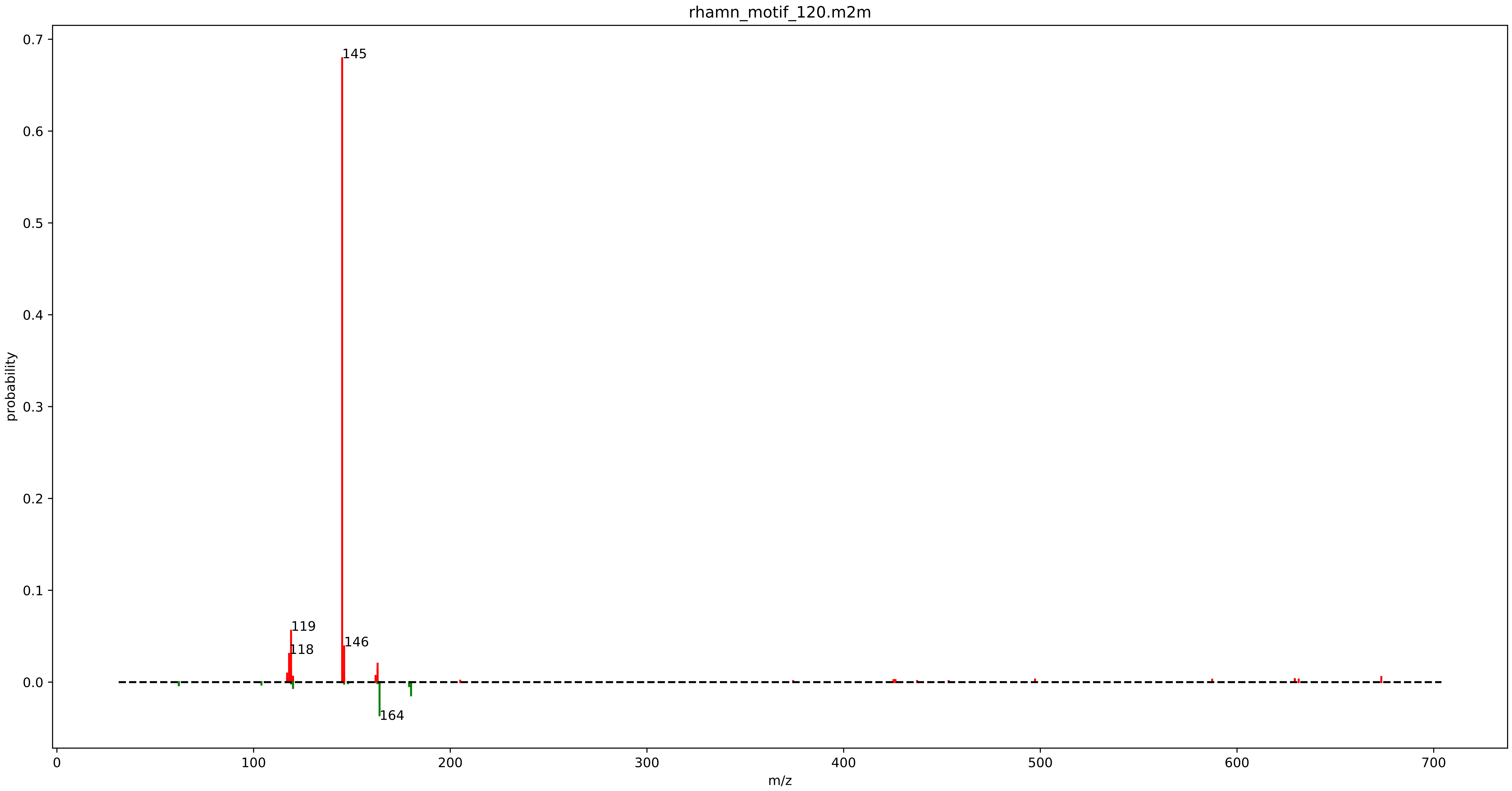
rhamn_motif_120.m2m
probability
m/z
145
119
118
146
164
0.7
0.6
0.5
0.4
0.3
0.2
0.1
0.0
0
100
200
300
400
500
600
700

rhamn_motif_120.m2m

fragment_145.0275	: 0.68
fragment_119.0525	: 0.06
fragment_146.0325	: 0.04
loss_164.0475	: 0.04
fragment_118.0425	: 0.03
fragment_163.0425	: 0.02
loss_180.0625	: 0.01
fragment_163.0375	: 0.01
fragment_119.0475	: 0.01
fragment_146.0275	: 0.01
fragment_117.0375	: 0.01
fragment_162.0325	: 0.01
loss_120.0575	: 0.01
fragment_120.0475	: 0.01
fragment_673.3725	: 0.01

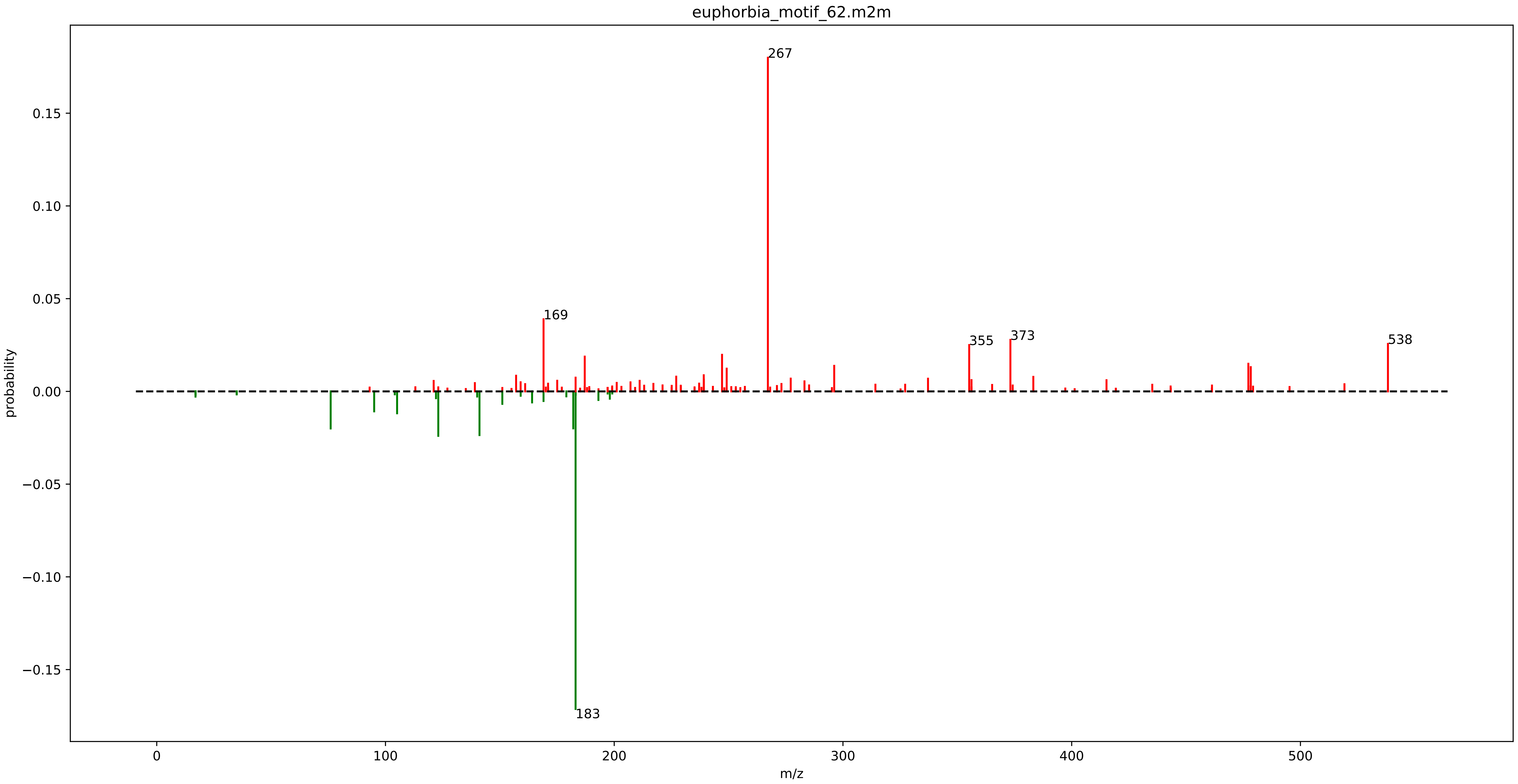

euphorbia_motif_62.m2m
probability
m/z
0.15
0.10
0.05
0.00
−0.05
−0.10
−0.15
0
100
200
300
400
500
267
169
355
373
538
183

euphorbia_motif_62.m2m

fragment_267.1775	: 0.18
loss_183.1075	: 0.17
fragment_169.1025	: 0.04
fragment_373.2025	: 0.03
fragment_538.2575	: 0.03
fragment_355.1925	: 0.03
loss_123.0875	: 0.02
loss_141.0975	: 0.02
loss_76.0375	: 0.02
loss_182.1025	: 0.02
fragment_247.1375	: 0.02
fragment_187.1125	: 0.02
fragment_477.2325	: 0.01
fragment_296.1775	: 0.01
fragment_478.2825	: 0.01
fragment_249.1675	: 0.01
loss_105.0775	: 0.01
loss_95.0575	: 0.01
fragment_239.1425	: 0.01
fragment_157.1025	: 0.01
fragment_227.1075	: 0.01
fragment_383.2225	: 0.01
fragment_183.1175	: 0.01
fragment_277.1625	: 0.01
fragment_337.1825	: 0.01
loss_151.0825	: 0.01
fragment_239.1825	: 0.01
fragment_356.1975	: 0.01
fragment_415.2175	: 0.01
loss_164.0925	: 0.01
fragment_175.0775	: 0.01
fragment_211.1125	: 0.01
fragment_121.0675	: 0.01
fragment_283.1725	: 0.01
loss_169.0975	: 0.01

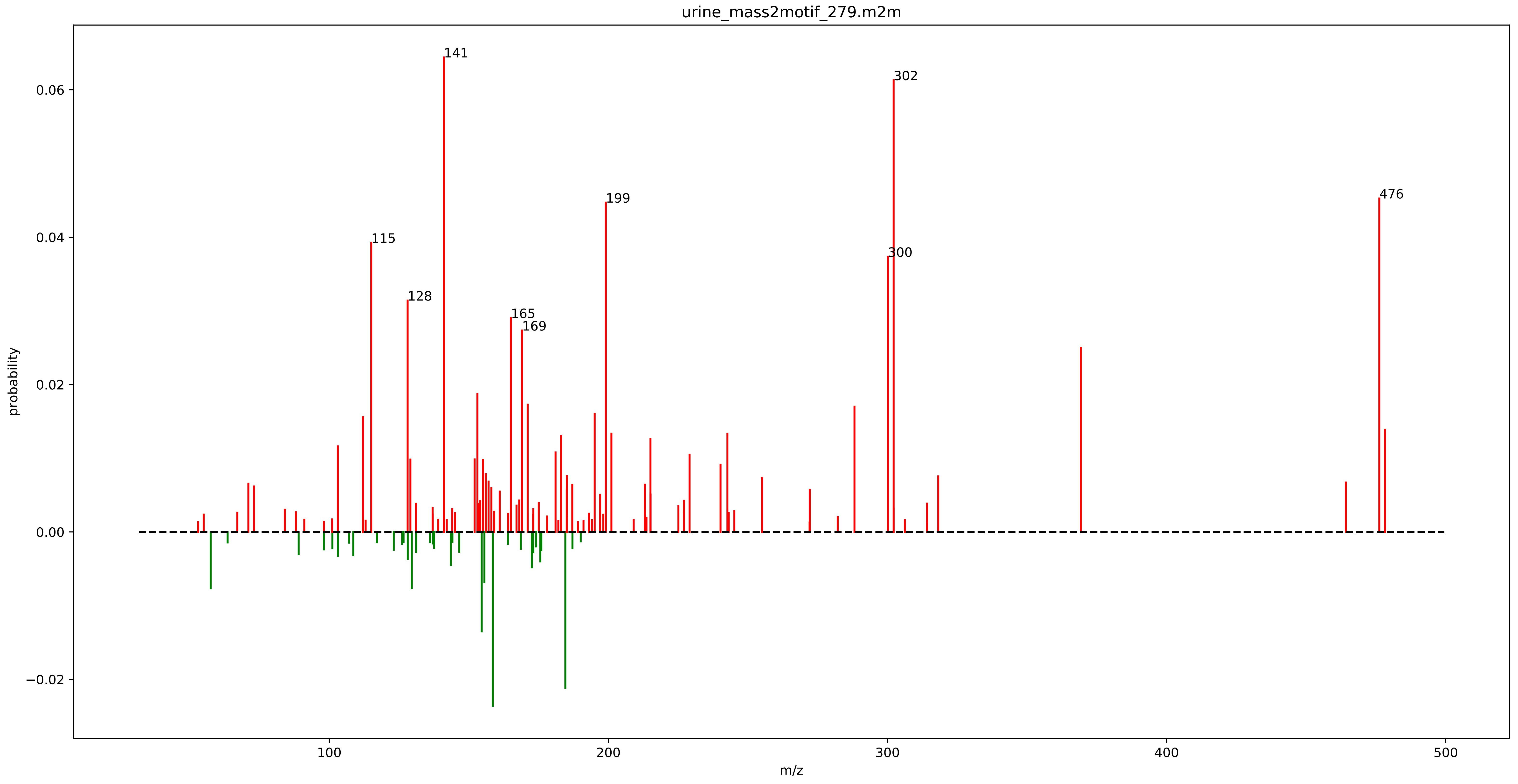
urine_mass2motif_279.m2m
probability
m/z
0.06
0.04
0.02
0.00
−0.02
100
200
300
400
500
141
302
199
476
115
300
128
165
169

urine_mass2motif_279.m2m

fragment_141.0675	: 0.06
fragment_302.1725	: 0.06
fragment_476.1925	: 0.05
fragment_199.0775	: 0.04
fragment_115.0525	: 0.04
fragment_300.1625	: 0.04
fragment_128.0625	: 0.03
fragment_165.0675	: 0.03
fragment_169.0625	: 0.03
fragment_369.2475	: 0.02
loss_158.5625	: 0.02
loss_184.5775	: 0.02
fragment_153.0675	: 0.02
fragment_171.0825	: 0.02
fragment_288.1575	: 0.02
fragment_195.0775	: 0.02
fragment_112.0775	: 0.02
fragment_478.2075	: 0.01
loss_154.6025	: 0.01
fragment_201.0875	: 0.01
fragment_242.6425	: 0.01
fragment_183.0775	: 0.01
fragment_215.0725	: 0.01
fragment_103.0525	: 0.01
fragment_181.0675	: 0.01
fragment_229.0825	: 0.01
fragment_152.0675	: 0.01
fragment_129.0725	: 0.01
fragment_155.0825	: 0.01
fragment_240.1725	: 0.01
fragment_156.0575	: 0.01
loss_57.5125	: 0.01
loss_129.5575	: 0.01
fragment_185.1275	: 0.01
fragment_318.1725	: 0.01
fragment_255.0625	: 0.01
fragment_157.0625	: 0.01
loss_155.5725	: 0.01
fragment_464.1925	: 0.01
fragment_71.0125	: 0.01
fragment_213.0875	: 0.01
fragment_187.0725	: 0.01
fragment_73.0275	: 0.01
fragment_158.0725	: 0.01
fragment_272.1325	: 0.01
fragment_185.0625	: 0.01
fragment_161.0625	: 0.01
fragment_215.1025	: 0.01
fragment_197.0575	: 0.01

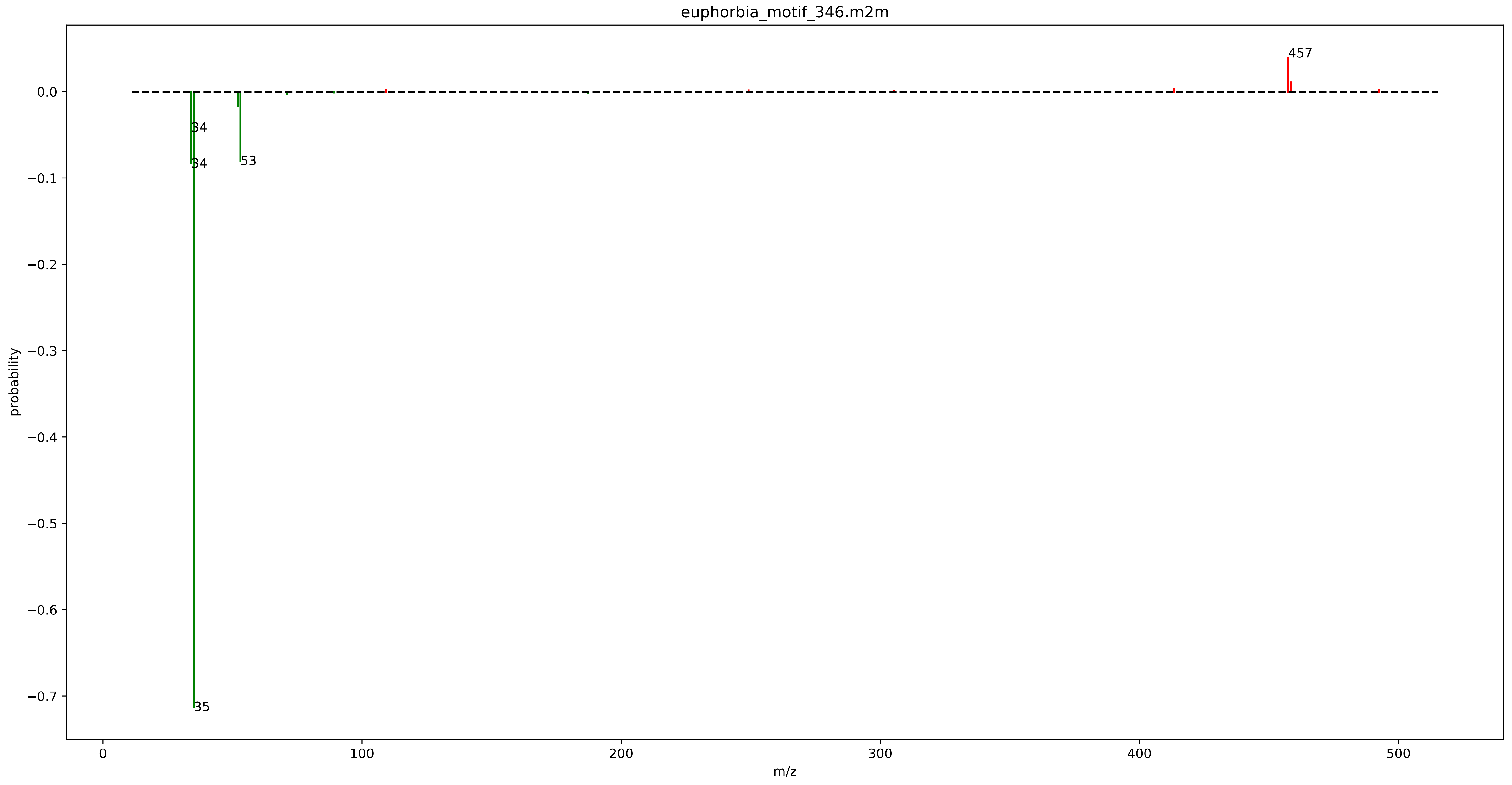

euphorbia_motif_346.m2m
457
0.0
−0.1
−0.2
−0.3
−0.4
−0.5
−0.6
−0.7
probability
34
34
53
35
0
100
200
300
400
500
m/z

euphorbia_motif_346.m2m

loss_35.0325	: 0.71
loss_34.0325	: 0.08
loss_53.0425	: 0.08
loss_34.0275	: 0.04
fragment_457.3725	: 0.04
loss_52.0425	: 0.02
fragment_458.3725	: 0.01

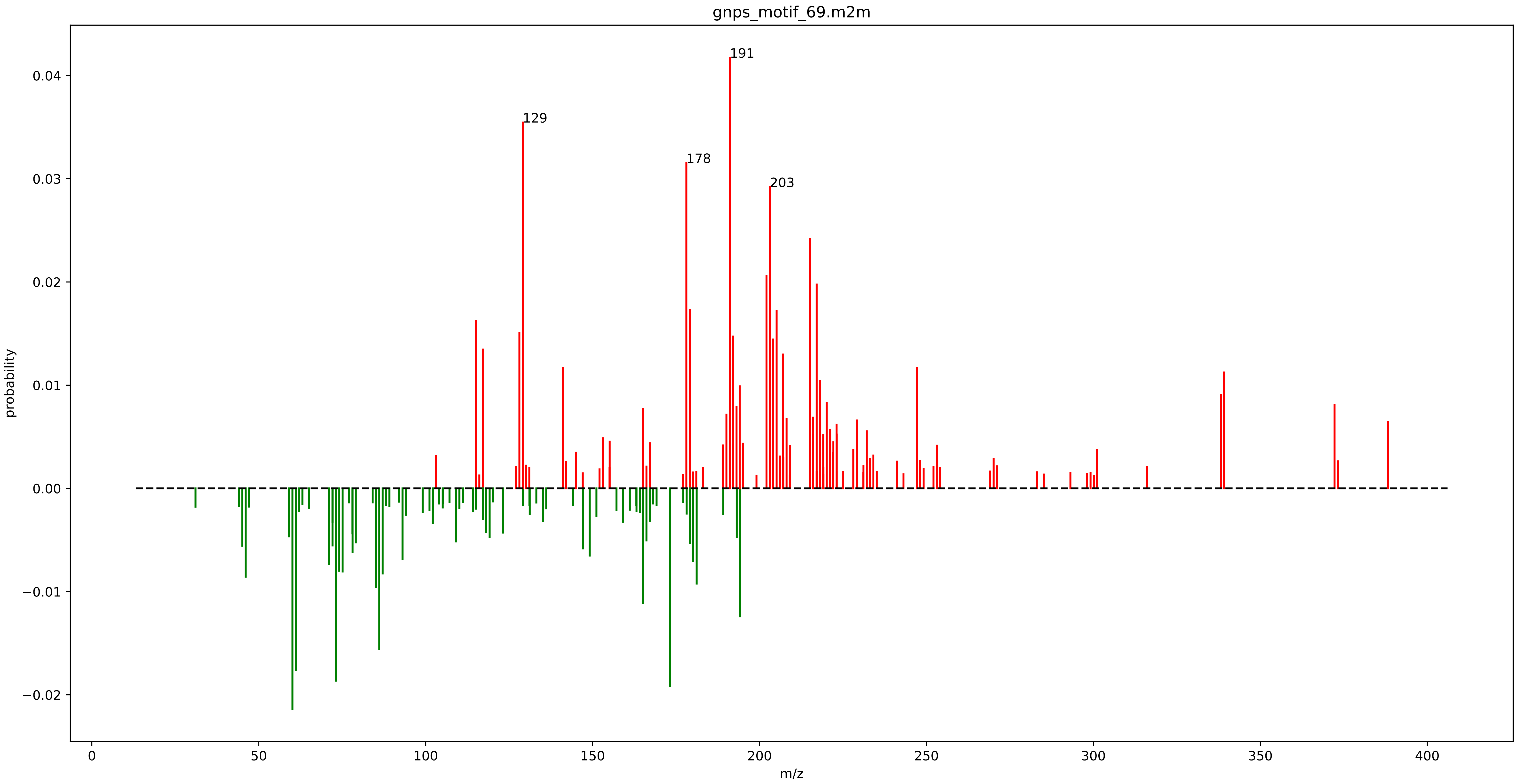

gnps_motif_69.m2m
probability
m/z
0.04
0.03
0.02
0.01
0.00
−0.01
−0.02
0
50
100
150
200
250
300
350
400
129
178
191
203

gnps_motif_69.m2m

fragment_191.0875	: 0.04
fragment_129.0675	: 0.04
fragment_178.0775	: 0.03
fragment_203.0875	: 0.03
fragment_215.0875	: 0.02
loss_60.0825	: 0.02
fragment_202.0775	: 0.02
fragment_217.1025	: 0.02
loss_173.1225	: 0.02
loss_73.0875	: 0.02
loss_61.0875	: 0.02
fragment_179.0875	: 0.02
fragment_205.1025	: 0.02
fragment_115.0525	: 0.02
loss_86.0975	: 0.02
fragment_128.0625	: 0.02
fragment_192.0925	: 0.01
fragment_204.0925	: 0.01
fragment_117.0675	: 0.01
fragment_207.0775	: 0.01
loss_194.1525	: 0.01
fragment_247.1125	: 0.01
fragment_141.0675	: 0.01
fragment_339.1775	: 0.01
loss_165.1175	: 0.01
fragment_218.1075	: 0.01
fragment_194.0725	: 0.01
loss_85.0875	: 0.01
loss_181.1475	: 0.01
fragment_338.1775	: 0.01
loss_46.0675	: 0.01
fragment_220.0875	: 0.01
loss_87.1025	: 0.01
fragment_372.2325	: 0.01
loss_75.1025	: 0.01
loss_74.0975	: 0.01
fragment_193.1025	: 0.01
fragment_165.0675	: 0.01
loss_71.0725	: 0.01
fragment_190.0775	: 0.01
loss_180.1375	: 0.01
loss_93.0575	: 0.01
fragment_216.0925	: 0.01
fragment_208.0875	: 0.01
fragment_229.0975	: 0.01
loss_149.1225	: 0.01
fragment_388.2275	: 0.01
fragment_223.0575	: 0.01
loss_78.0925	: 0.01
loss_147.1025	: 0.01
fragment_221.0975	: 0.01
loss_165.1525	: 0.01
loss_45.0575	: 0.01
fragment_232.0875	: 0.01
loss_72.0825	: 0.01
loss_179.1325	: 0.01
loss_79.0475	: 0.01
fragment_223.1125	: 0.01
fragment_219.0825	: 0.01
loss_109.0875	: 0.01
loss_166.1225	: 0.01

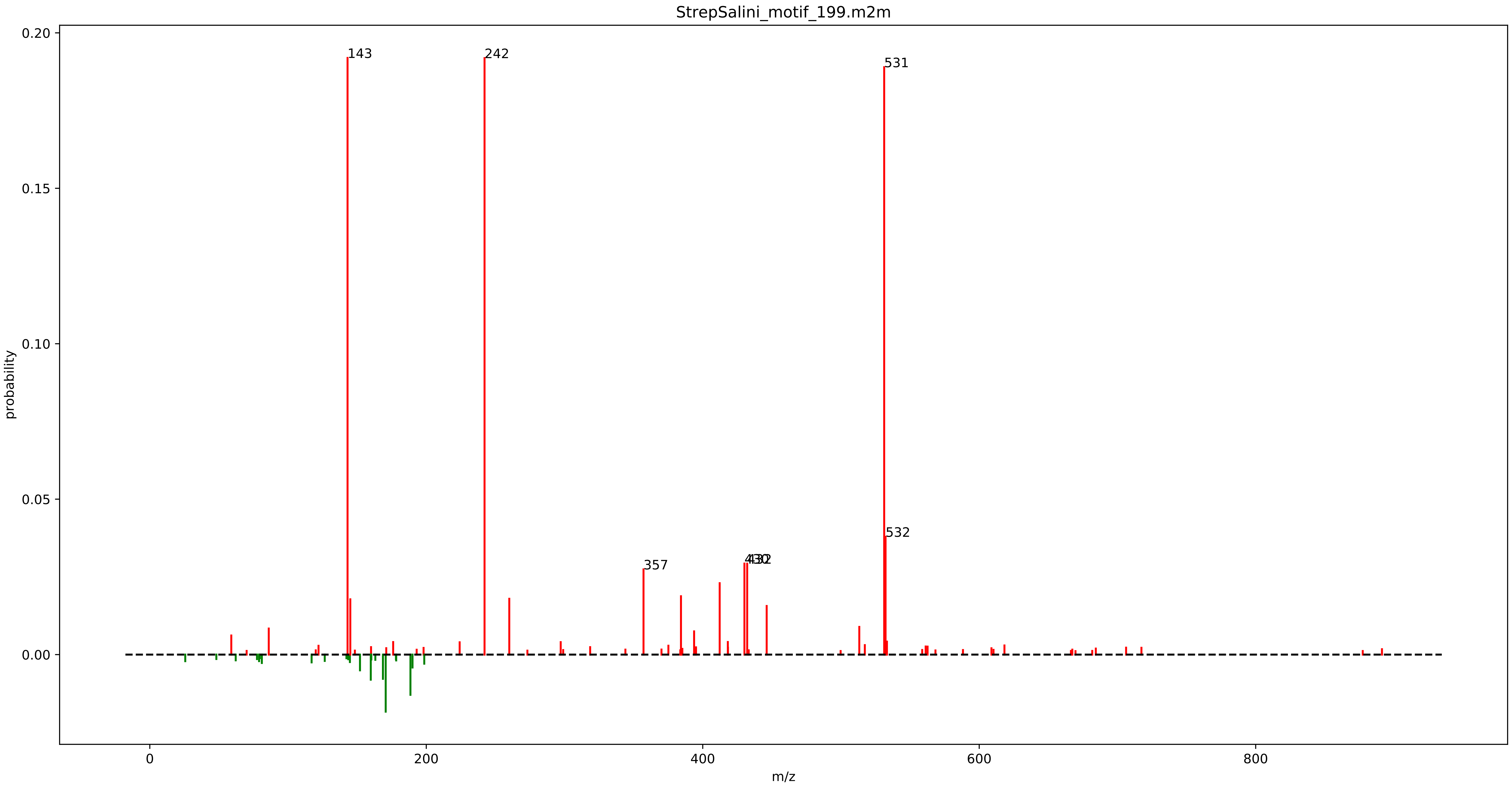

StrepSalini_motif_199.m2m
probability
m/z
0.20
0.15
0.10
0.05
0.00
0
200
400
600
800
143
242
531
532
357
430
432

StrepSalini_motif_199.m2m

fragment_143.0500	: 0.19
fragment_242.1500	: 0.19
fragment_531.2500	: 0.19
fragment_532.2500	: 0.04
fragment_430.1500	: 0.03
fragment_432.1525	: 0.03
fragment_357.1500	: 0.03
fragment_412.2500	: 0.02
fragment_384.2525	: 0.02
loss_170.6500	: 0.02
fragment_260.0500	: 0.02
fragment_145.0500	: 0.02
fragment_446.2500	: 0.02
loss_188.5500	: 0.01
fragment_513.2500	: 0.01
fragment_86.0500	: 0.01
loss_159.8500	: 0.01
loss_168.6525	: 0.01
fragment_393.7500	: 0.01
fragment_58.9500	: 0.01
loss_152.0500	: 0.01

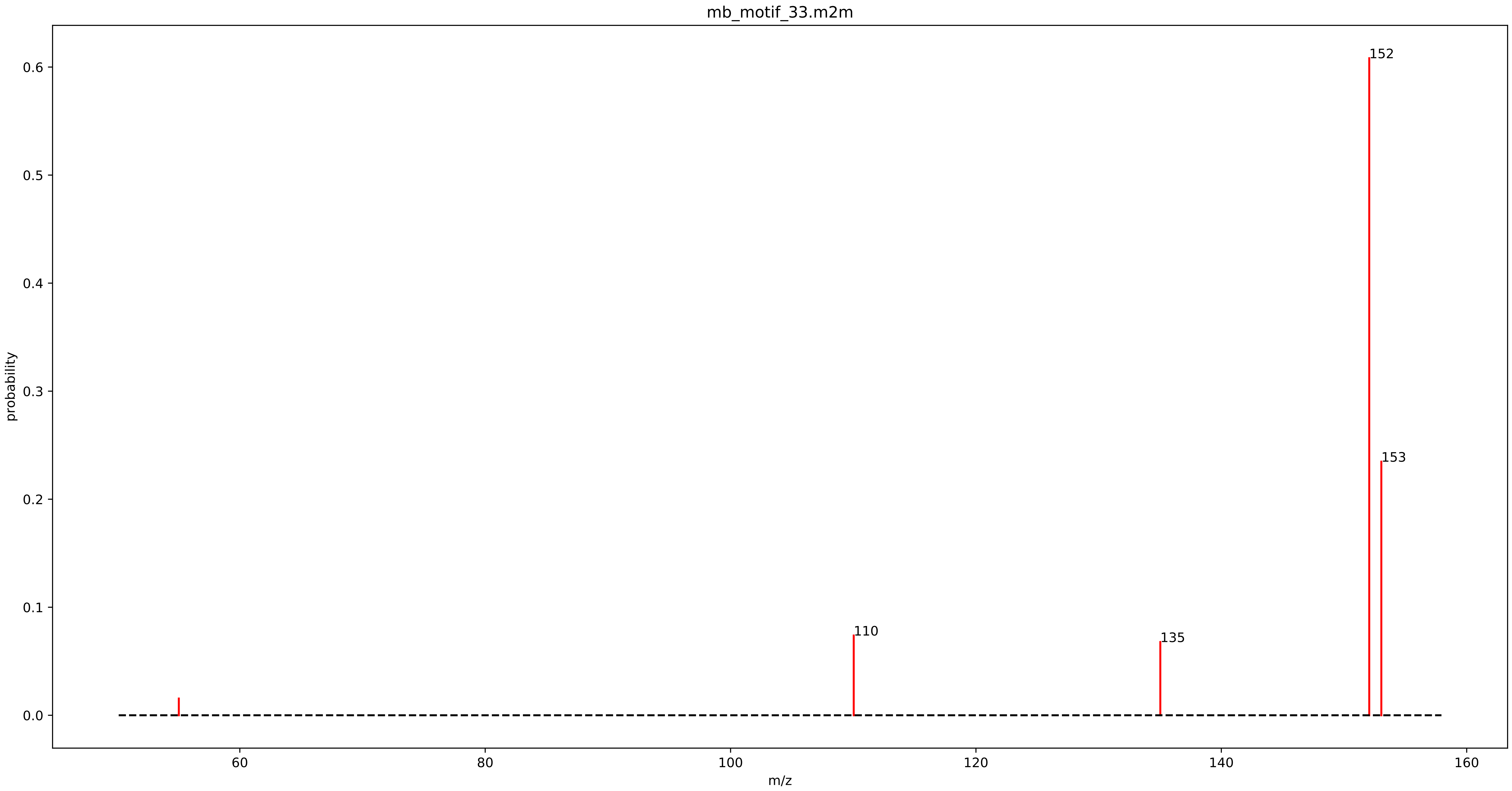

mb_motif_33.m2m
probability
m/z
0.0
0.1
0.2
0.3
0.4
0.5
0.6
60
80
100
120
140
160
110
135
152
153

mb_motif_33.m2m

fragment_152.0575	: 0.61
fragment_153.0425	: 0.23
fragment_110.0375	: 0.07
fragment_135.0275	: 0.07
fragment_55.0275	: 0.02

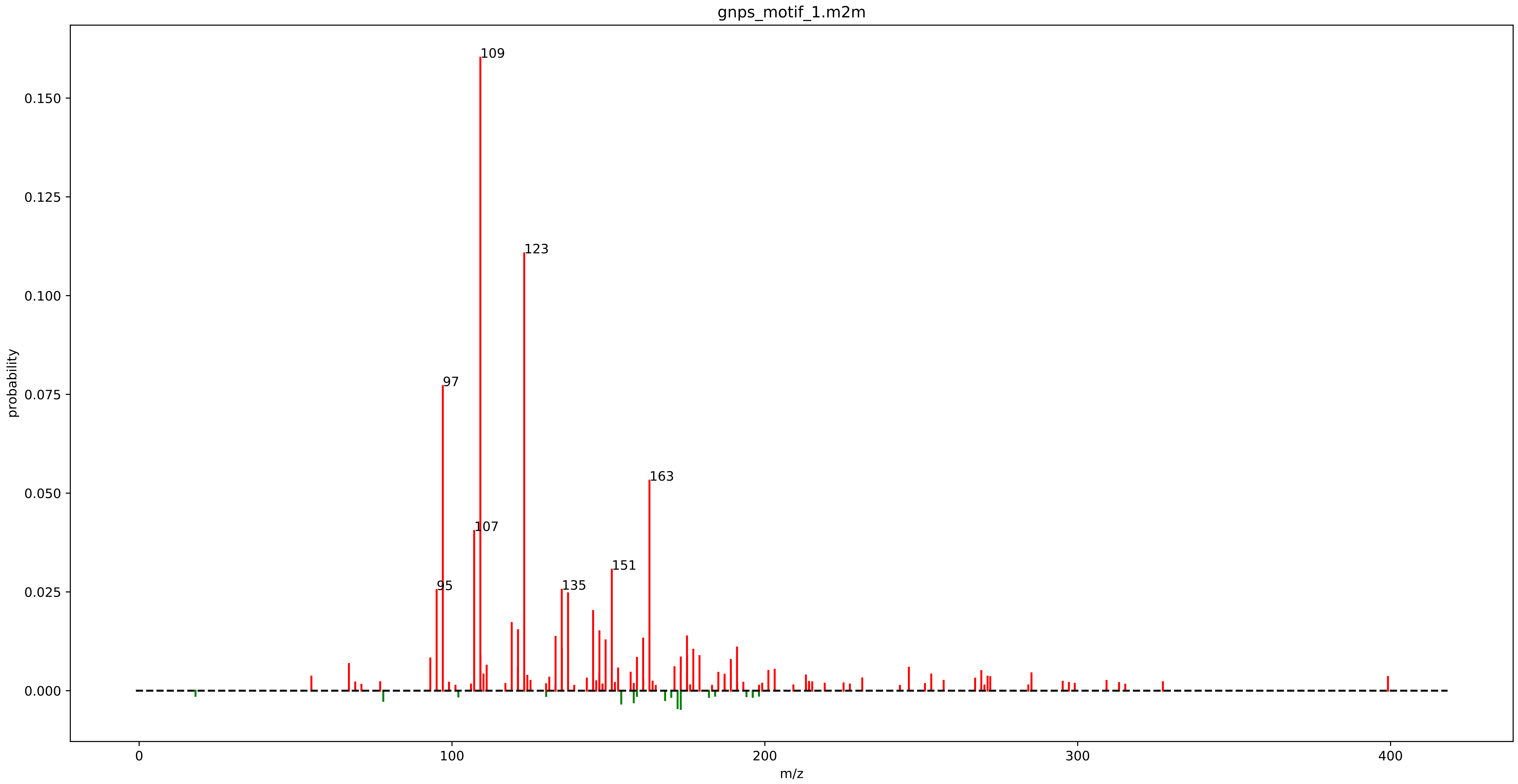
gnps_motif_1.m2m
probability
m/z
0.150
0.125
0.100
0.075
0.050
0.025
0.000
0
100
200
300
400
109
123
97
163
107
151
95
135

gnps_motif_1.m2m

fragment_109.0625	: 0.16
fragment_123.0825	: 0.11
fragment_97.0625	: 0.08
fragment_163.1125	: 0.05
fragment_107.0875	: 0.04
fragment_151.0775	: 0.03
fragment_135.0825	: 0.03
fragment_95.0875	: 0.03
fragment_137.0975	: 0.02
fragment_145.1025	: 0.02
fragment_119.0875	: 0.02
fragment_121.1025	: 0.02
fragment_147.1175	: 0.02
fragment_175.1125	: 0.01
fragment_133.1025	: 0.01
fragment_161.0975	: 0.01
fragment_149.0975	: 0.01
fragment_191.1075	: 0.01
fragment_135.1175	: 0.01
fragment_177.1275	: 0.01
fragment_179.1075	: 0.01
fragment_109.1025	: 0.01
fragment_175.1475	: 0.01
fragment_173.1325	: 0.01
fragment_159.1175	: 0.01
fragment_93.0675	: 0.01
fragment_189.1275	: 0.01
fragment_67.0525	: 0.01
fragment_111.0825	: 0.01
fragment_171.1175	: 0.01
fragment_246.0375	: 0.01
fragment_121.0625	: 0.01
fragment_153.0925	: 0.01
fragment_203.1425	: 0.01

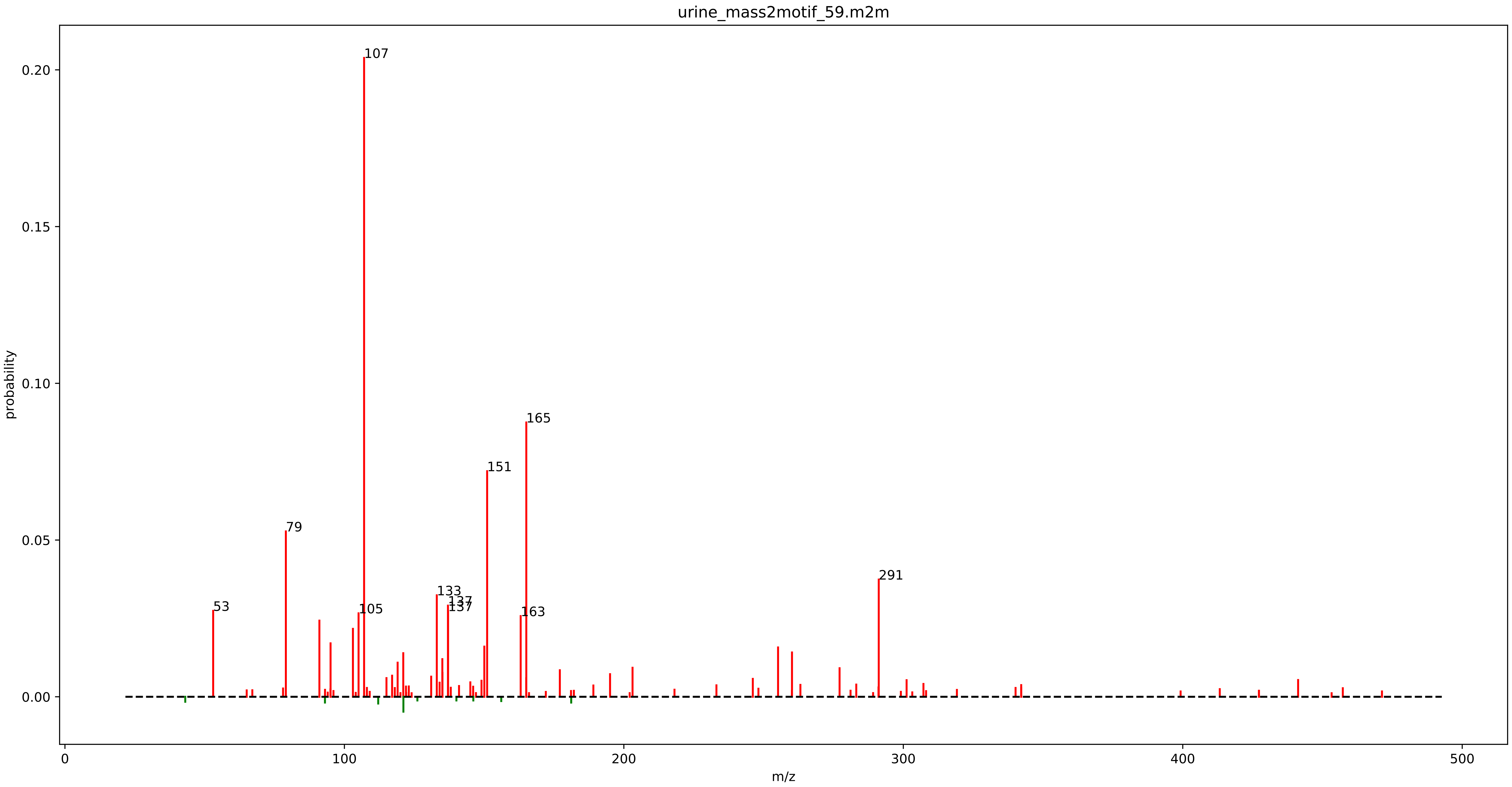

urine_mass2motif_59.m2m
probability
m/z
0.20
0.15
0.10
0.05
0.00
0
100
200
300
400
500
107
165
151
79
291
53
105
133
137
137
163

urine_mass2motif_59.m2m

fragment_107.0475	: 0.20
fragment_165.0925	: 0.09
fragment_151.0775	: 0.07
fragment_79.0525	: 0.05
fragment_291.2075	: 0.04
fragment_133.0625	: 0.03
fragment_137.0575	: 0.03
fragment_53.0375	: 0.03
fragment_137.0975	: 0.03
fragment_105.0675	: 0.03
fragment_163.0775	: 0.03
fragment_91.0525	: 0.02
fragment_103.0525	: 0.02
fragment_95.0475	: 0.02
fragment_150.0675	: 0.02
fragment_255.1825	: 0.02
fragment_260.1675	: 0.01
fragment_121.0625	: 0.01
fragment_135.0475	: 0.01
fragment_119.0475	: 0.01
fragment_203.1075	: 0.01
fragment_277.1925	: 0.01
fragment_177.0925	: 0.01
fragment_135.0825	: 0.01
fragment_195.0625	: 0.01
fragment_117.0675	: 0.01
fragment_131.0525	: 0.01
fragment_115.0525	: 0.01
fragment_165.0575	: 0.01
fragment_246.1475	: 0.01
fragment_441.2775	: 0.01
fragment_301.1775	: 0.01
fragment_149.0625	: 0.01

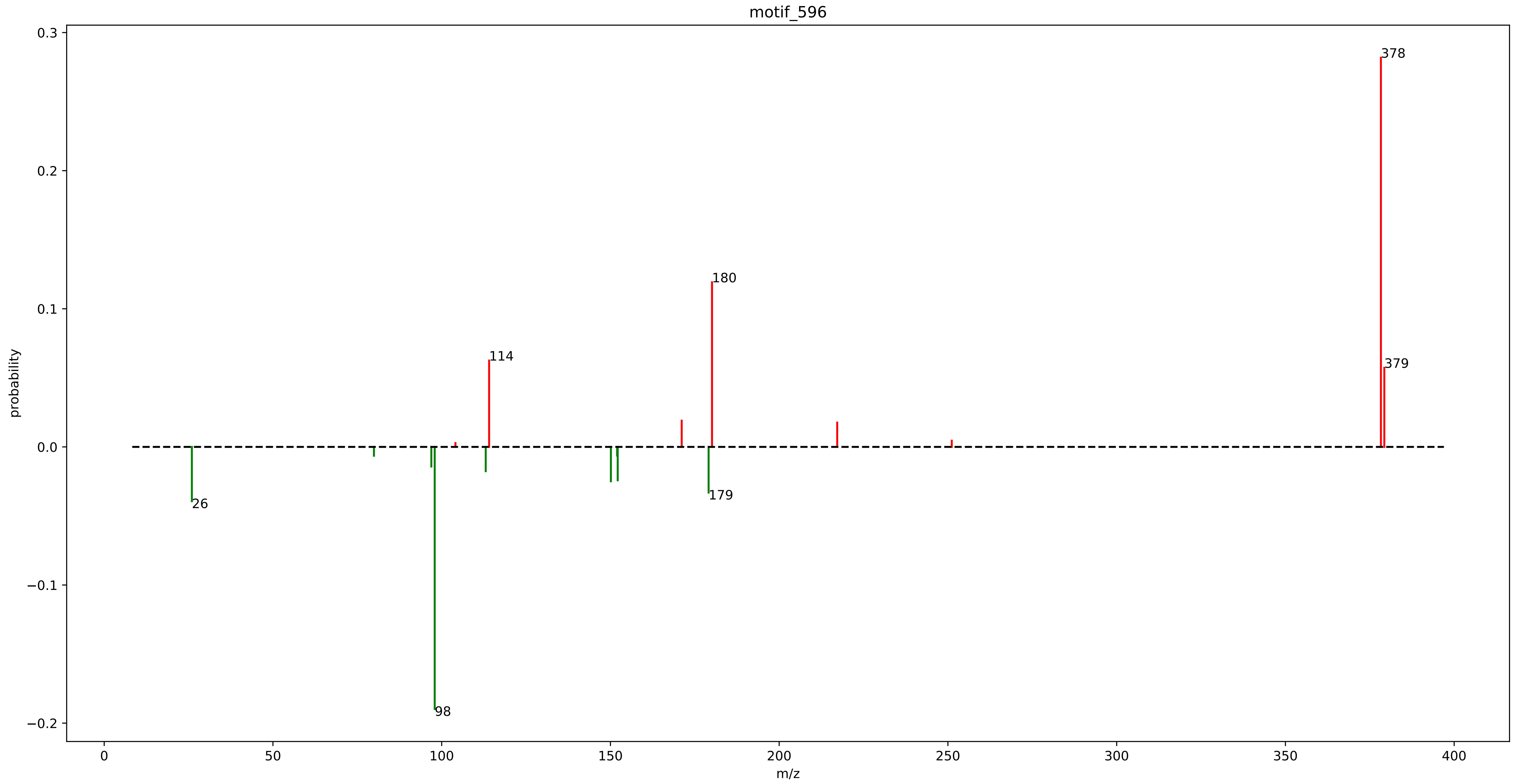

motif_596
probability
m/z
0.3
0.2
0.1
0.0
−0.1
−0.2
0
50
100
150
200
250
300
350
400
378
379
180
114
26
98
179

motif_596

fragment_378.2975	: 0.28
loss_97.9325	: 0.19
fragment_180.1025	: 0.12
fragment_114.0525	: 0.06
fragment_379.3025	: 0.06
loss_25.9775	: 0.04
loss_179.0925	: 0.03
loss_150.1375	: 0.02
loss_152.1575	: 0.02
fragment_171.1175	: 0.02
loss_113.0475	: 0.02
fragment_217.1925	: 0.02
loss_96.9325	: 0.01
loss_79.9275	: 0.01
loss_151.9825	: 0.01

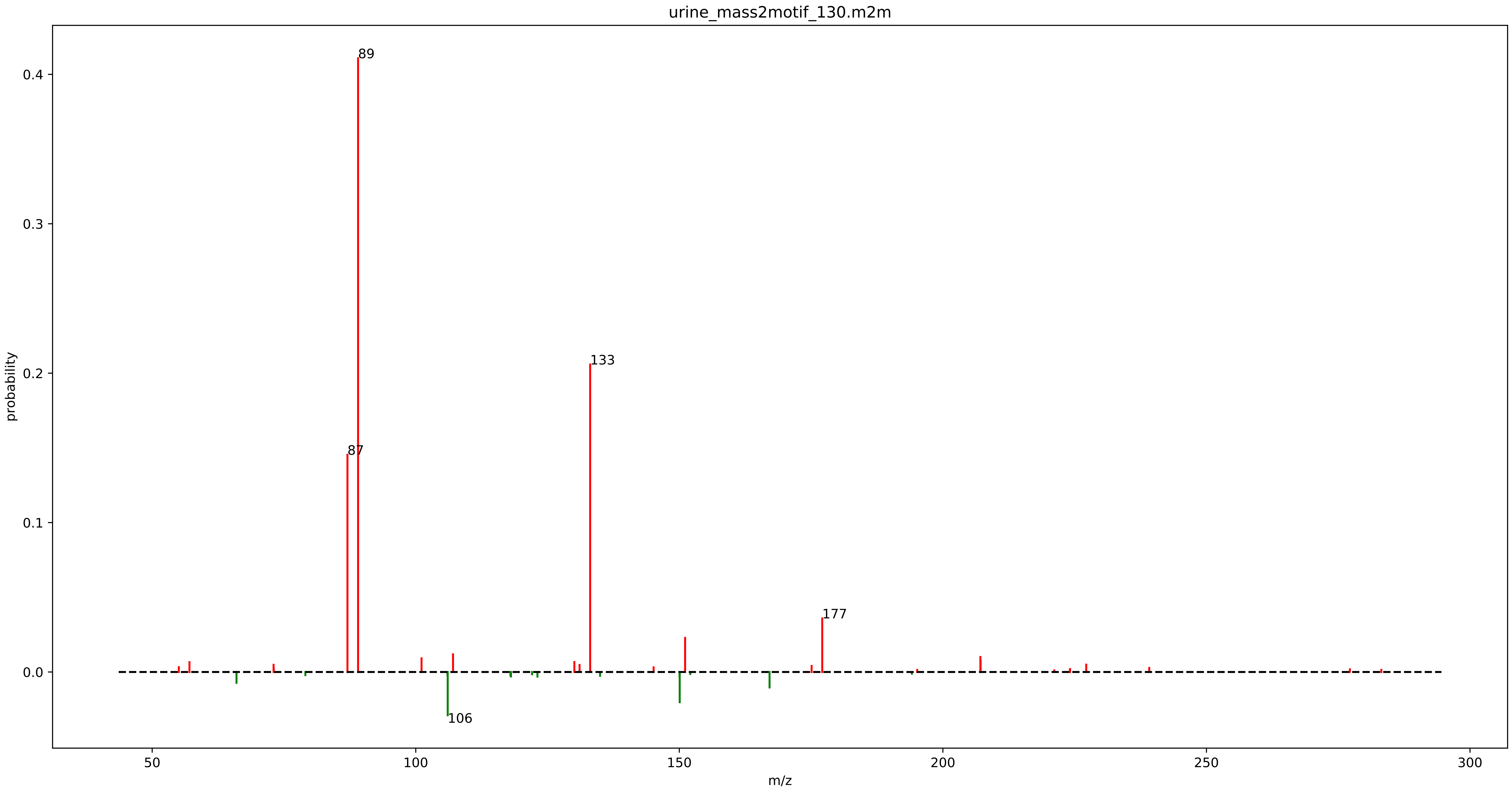
urine_mass2motif_130.m2m
probability
m/z
0.4
0.3
0.2
0.1
0.0
50
100
150
200
250
300
89
87
133
177
106

urine_mass2motif_130.m2m

fragment_89.0575	: 0.41
fragment_133.0875	: 0.21
fragment_87.0425	: 0.15
fragment_177.1125	: 0.04
loss_106.0625	: 0.03
fragment_151.0975	: 0.02
loss_150.0725	: 0.02
fragment_107.0725	: 0.01
loss_167.1175	: 0.01
fragment_207.1125	: 0.01
fragment_101.0975	: 0.01
fragment_207.1575	: 0.01
loss_65.9925	: 0.01
fragment_130.0875	: 0.01
fragment_57.0725	: 0.01

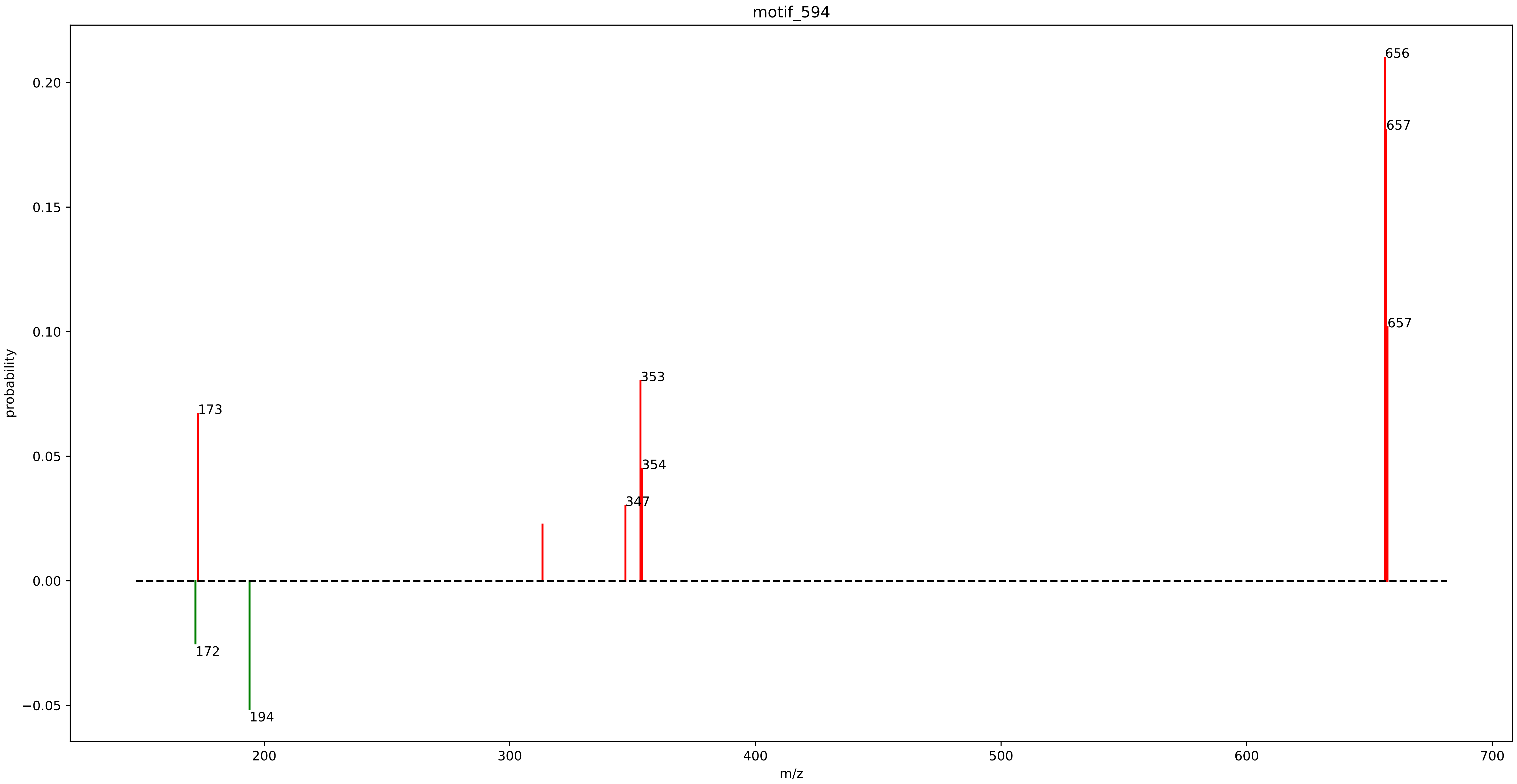

motif_594
probability
m/z
0.20
0.15
0.10
0.05
0.00
−0.05
200
300
400
500
600
700
656
657
657
353
173
354
347
172
194

motif_594

fragment_656.3225	: 0.21
fragment_656.8225	: 0.18
fragment_657.3225	: 0.10
fragment_353.1975	: 0.08
fragment_173.0225	: 0.07
loss_194.0325	: 0.05
fragment_353.6975	: 0.04
fragment_347.0825	: 0.03
loss_172.0125	: 0.03
fragment_313.3075	: 0.02

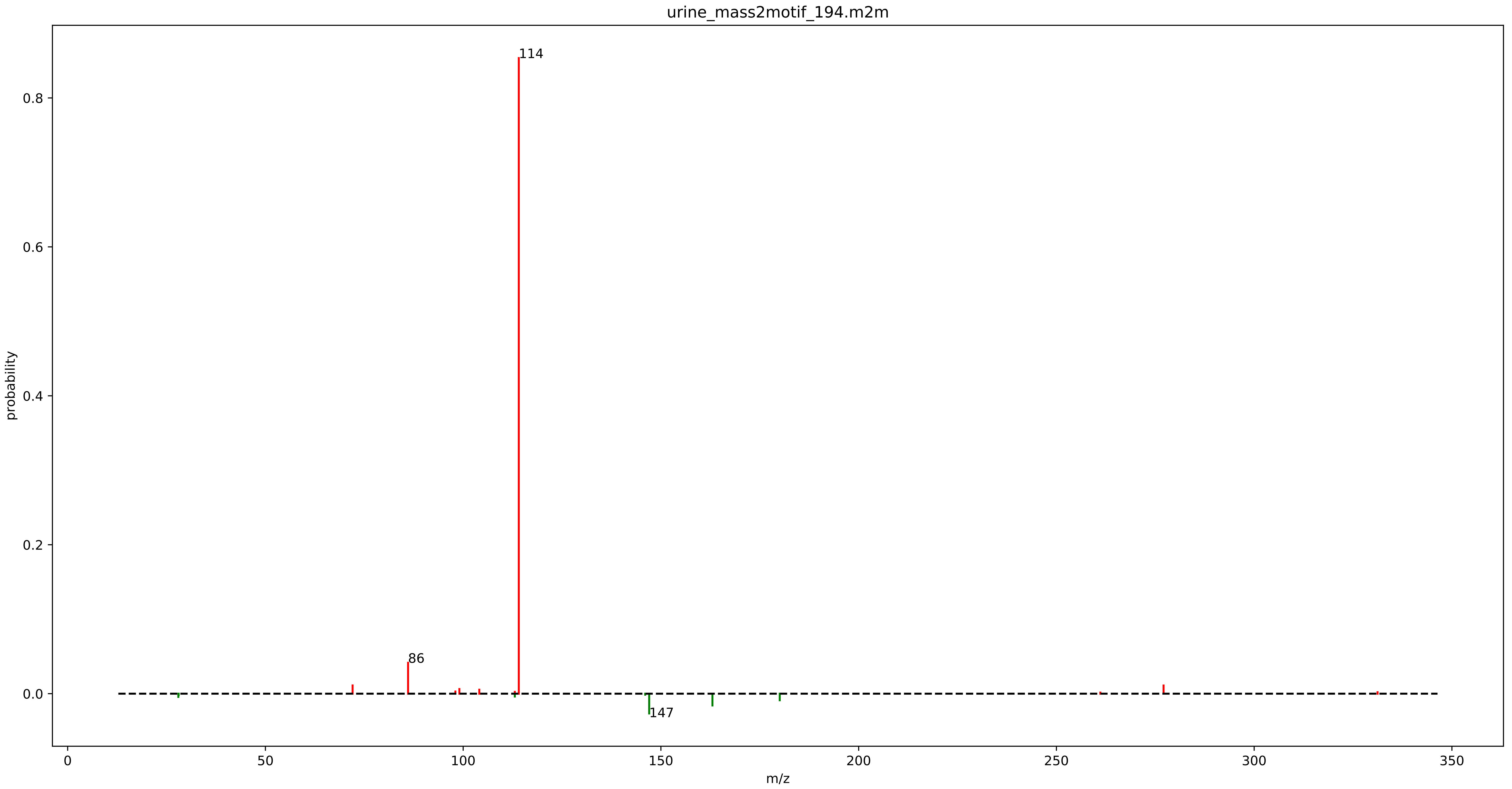

urine_mass2motif_194.m2m
probability
m/z
114
86
147
0.8
0.6
0.4
0.2
0.0
0
50
100
150
200
250
300
350

urine_mass2motif_194.m2m

fragment_114.0675	: 0.85
fragment_86.0725	: 0.04
loss_147.0275	: 0.03
loss_163.0275	: 0.02
fragment_72.0425	: 0.01
fragment_277.0875	: 0.01
loss_180.0425	: 0.01
fragment_99.0425	: 0.01
fragment_104.0825	: 0.01

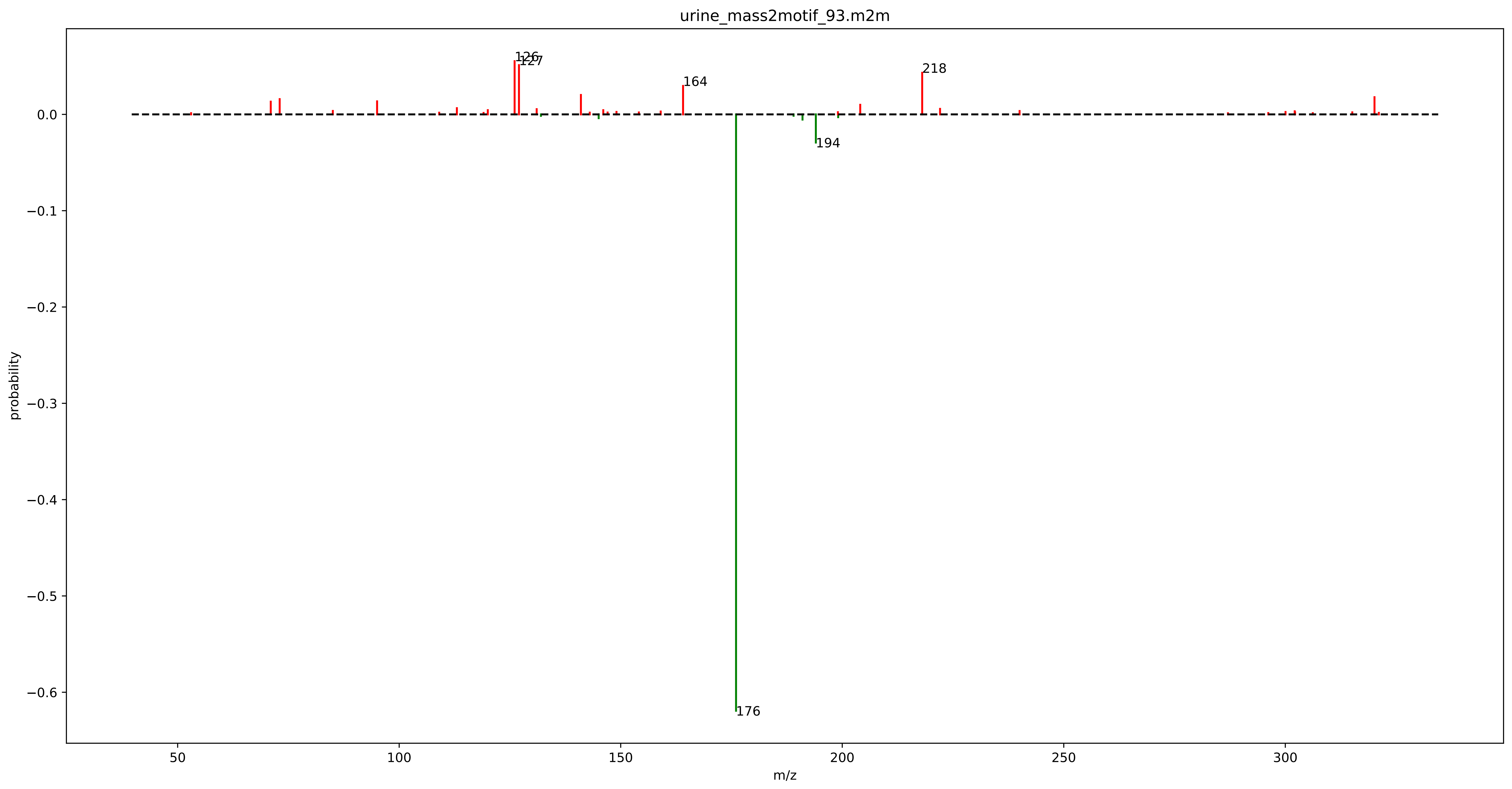

urine_mass2motif_93.m2m
126
127
164
218
194
176
probability
0.0
−0.1
−0.2
−0.3
−0.4
−0.5
−0.6
50
100
150
200
250
300
m/z

urine_mass2motif_93.m2m

loss_176.0325	: 0.62
fragment_126.0525	: 0.06
fragment_127.0375	: 0.05
fragment_218.0425	: 0.04
fragment_164.0725	: 0.03
loss_194.0425	: 0.03
fragment_141.0175	: 0.02
fragment_320.1325	: 0.02
fragment_73.0275	: 0.02
fragment_95.0125	: 0.01
fragment_71.0125	: 0.01
fragment_204.0625	: 0.01
fragment_113.0225	: 0.01
fragment_222.0775	: 0.01
fragment_131.0325	: 0.01
loss_191.0325	: 0.01

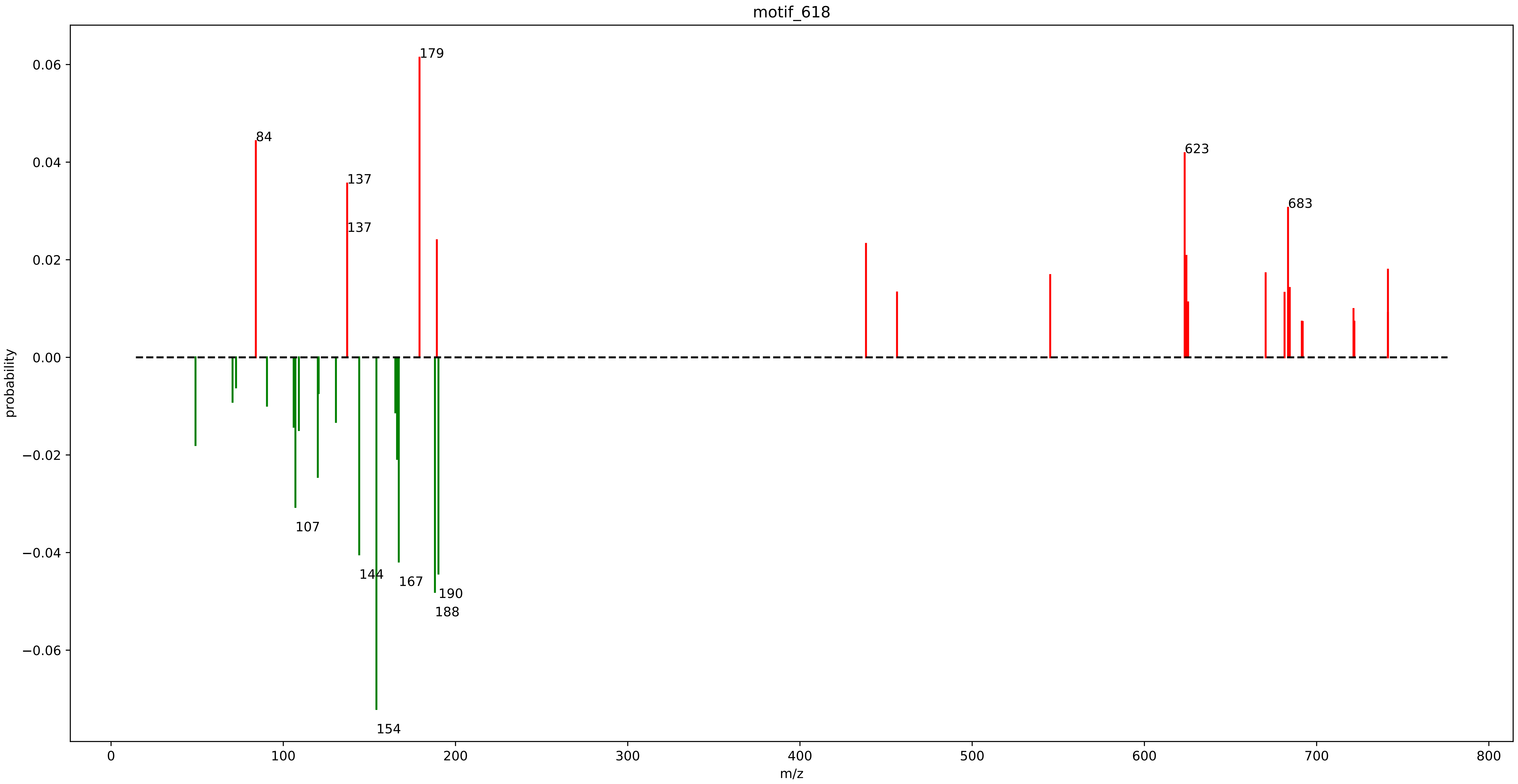

motif_618
probability
m/z
0.06
0.04
0.02
0.00
−0.02
−0.04
−0.06
0
100
200
300
400
500
600
700
800
179
84
137
137
623
683
107
144
167
190
188
154

motif_618

loss_154.0725	: 0.07
fragment_179.1025	: 0.06
loss_188.0575	: 0.05
fragment_84.0625	: 0.04
loss_190.0925	: 0.04
fragment_623.3775	: 0.04
loss_167.0575	: 0.04
loss_144.0875	: 0.04
fragment_137.0925	: 0.04
fragment_683.3975	: 0.03
loss_107.0375	: 0.03
fragment_137.0975	: 0.03
loss_120.0425	: 0.02
fragment_189.1625	: 0.02
fragment_438.3425	: 0.02
loss_166.0575	: 0.02
fragment_624.3825	: 0.02
loss_49.0325	: 0.02
fragment_741.4075	: 0.02
fragment_670.3975	: 0.02
fragment_545.2975	: 0.02
loss_109.0525	: 0.01
loss_106.0375	: 0.01
fragment_684.4025	: 0.01
fragment_456.3575	: 0.01
fragment_681.3725	: 0.01
loss_130.5725	: 0.01
fragment_681.3875	: 0.01
loss_165.0575	: 0.01
fragment_625.3825	: 0.01
loss_90.5325	: 0.01
fragment_721.4075	: 0.01
loss_70.5475	: 0.01
fragment_741.3975	: 0.01
loss_120.5425	: 0.01
fragment_691.3975	: 0.01
fragment_721.9125	: 0.01
fragment_691.9025	: 0.01
loss_72.5725	: 0.01

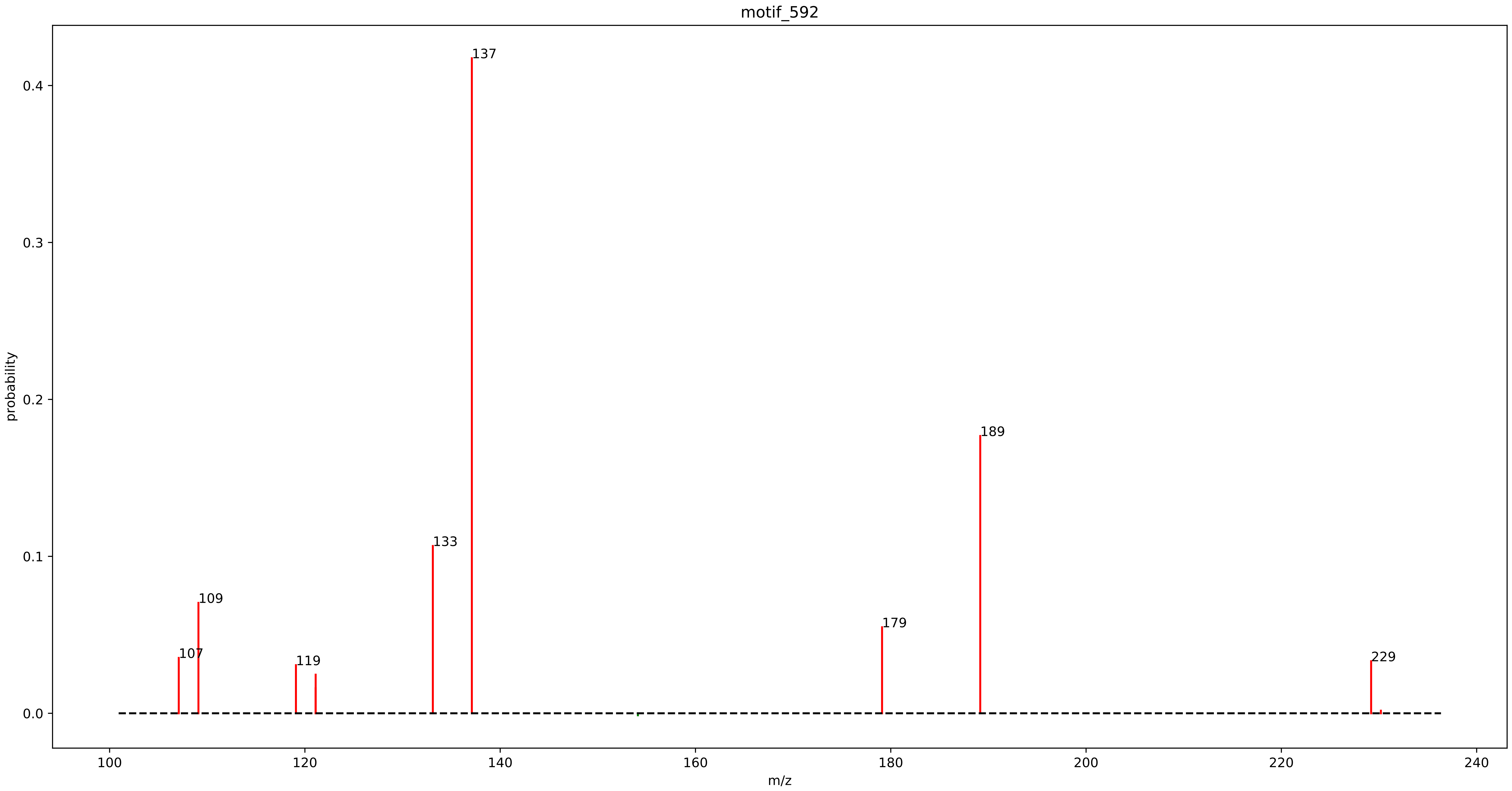

motif_592
probability
m/z
0.0
0.1
0.2
0.3
0.4
100
120
140
160
180
200
220
240
137
189
133
109
179
107
119
229

motif_592

fragment_137.0975	: 0.42
fragment_189.1625	: 0.18
fragment_133.1025	: 0.11
fragment_109.0975	: 0.07
fragment_179.1075	: 0.05
fragment_107.0825	: 0.04
fragment_229.1925	: 0.03
fragment_119.0825	: 0.03
fragment_121.1025	: 0.02
fragment_121.0975	: 0.02

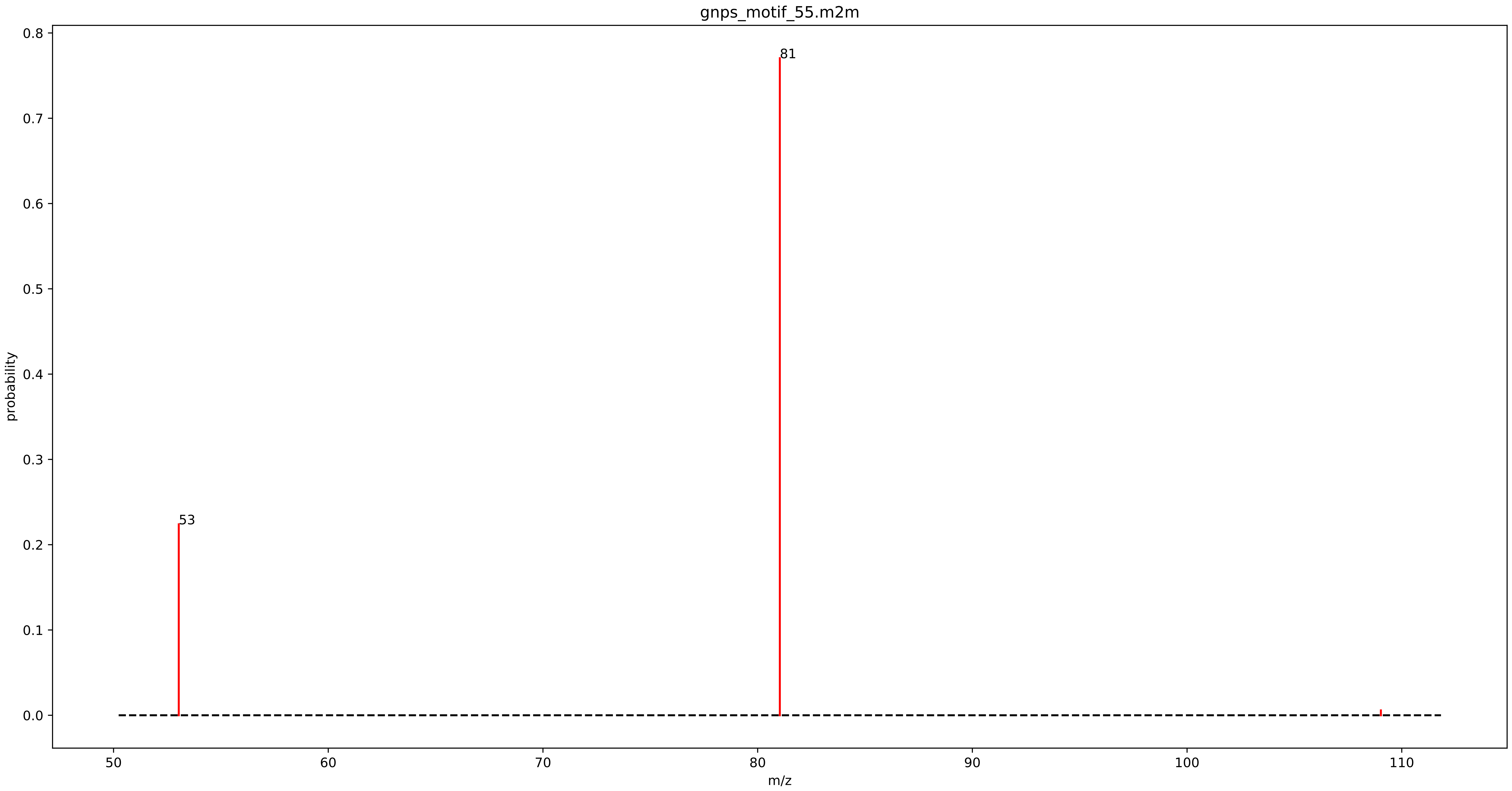

gnps_motif_55.m2m
probability
m/z
81
53
0.0
0.1
0.2
0.3
0.4
0.5
0.6
0.7
0.8
50
60
70
80
90
100
110

gnps_motif_55.m2m

fragment_81.0325	: 0.77
fragment_53.0375	: 0.22
fragment_109.0275	: 0.01

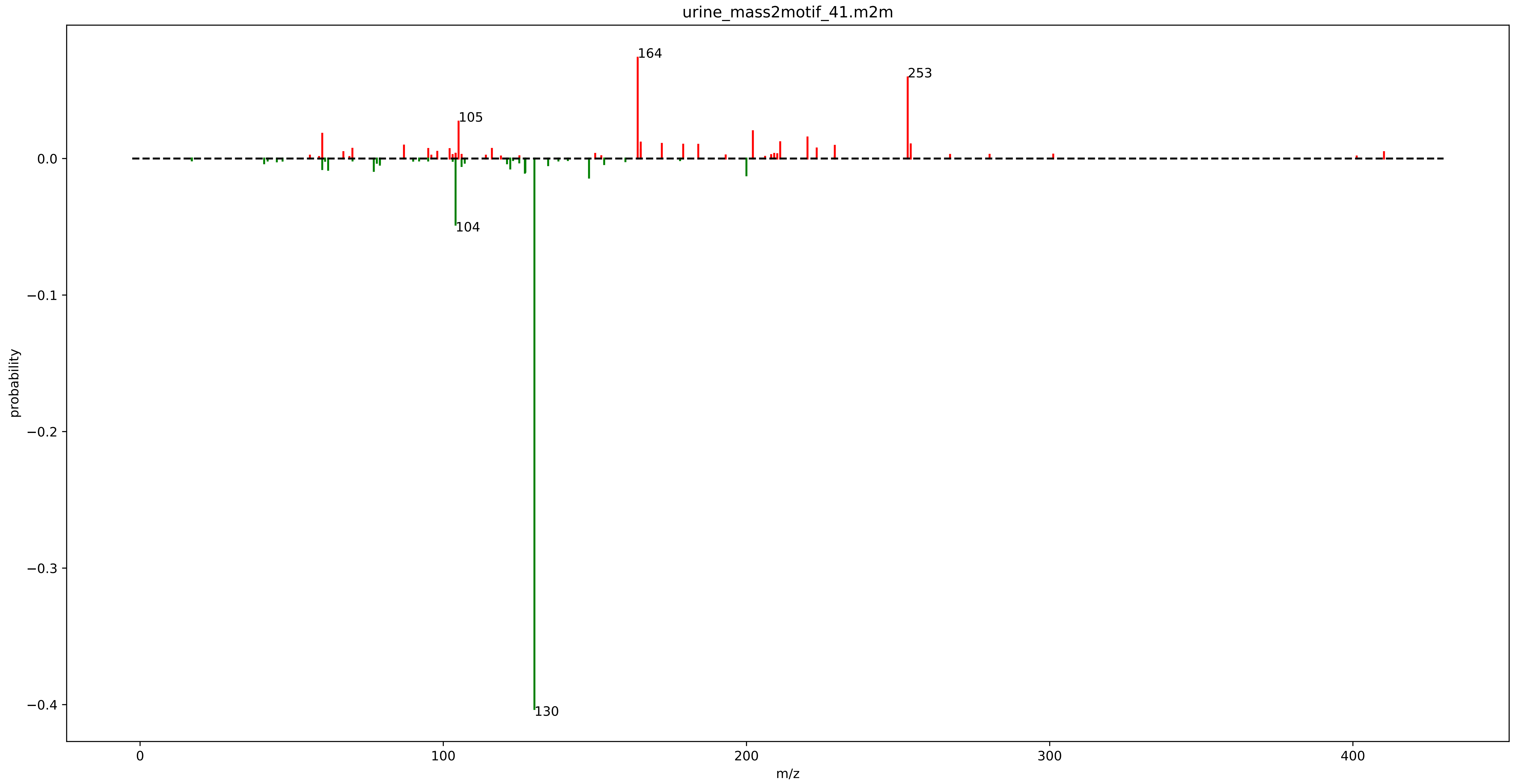
urine_mass2motif_41.m2m
probability
m/z
0.0
−0.1
−0.2
−0.3
−0.4
0
100
200
300
400
164
253
105
104
130

urine_mass2motif_41.m2m

loss_130.0575	: 0.40
fragment_164.1175	: 0.07
fragment_253.1675	: 0.06
loss_104.0525	: 0.05
fragment_105.0425	: 0.03
fragment_202.1075	: 0.02
fragment_60.0825	: 0.02
fragment_220.1175	: 0.02
loss_148.0675	: 0.01
loss_199.9775	: 0.01
fragment_211.1175	: 0.01
fragment_165.1175	: 0.01
fragment_172.0775	: 0.01
fragment_254.1675	: 0.01
loss_126.9075	: 0.01
fragment_179.1275	: 0.01
fragment_184.0975	: 0.01
loss_127.0725	: 0.01
fragment_87.0325	: 0.01
fragment_229.1225	: 0.01
loss_77.0825	: 0.01
loss_62.0275	: 0.01
loss_60.0775	: 0.01
fragment_223.1575	: 0.01
loss_122.0925	: 0.01
fragment_70.0275	: 0.01
fragment_116.0325	: 0.01
fragment_95.0475	: 0.01
fragment_102.0925	: 0.01
fragment_165.1125	: 0.01
loss_106.0375	: 0.01

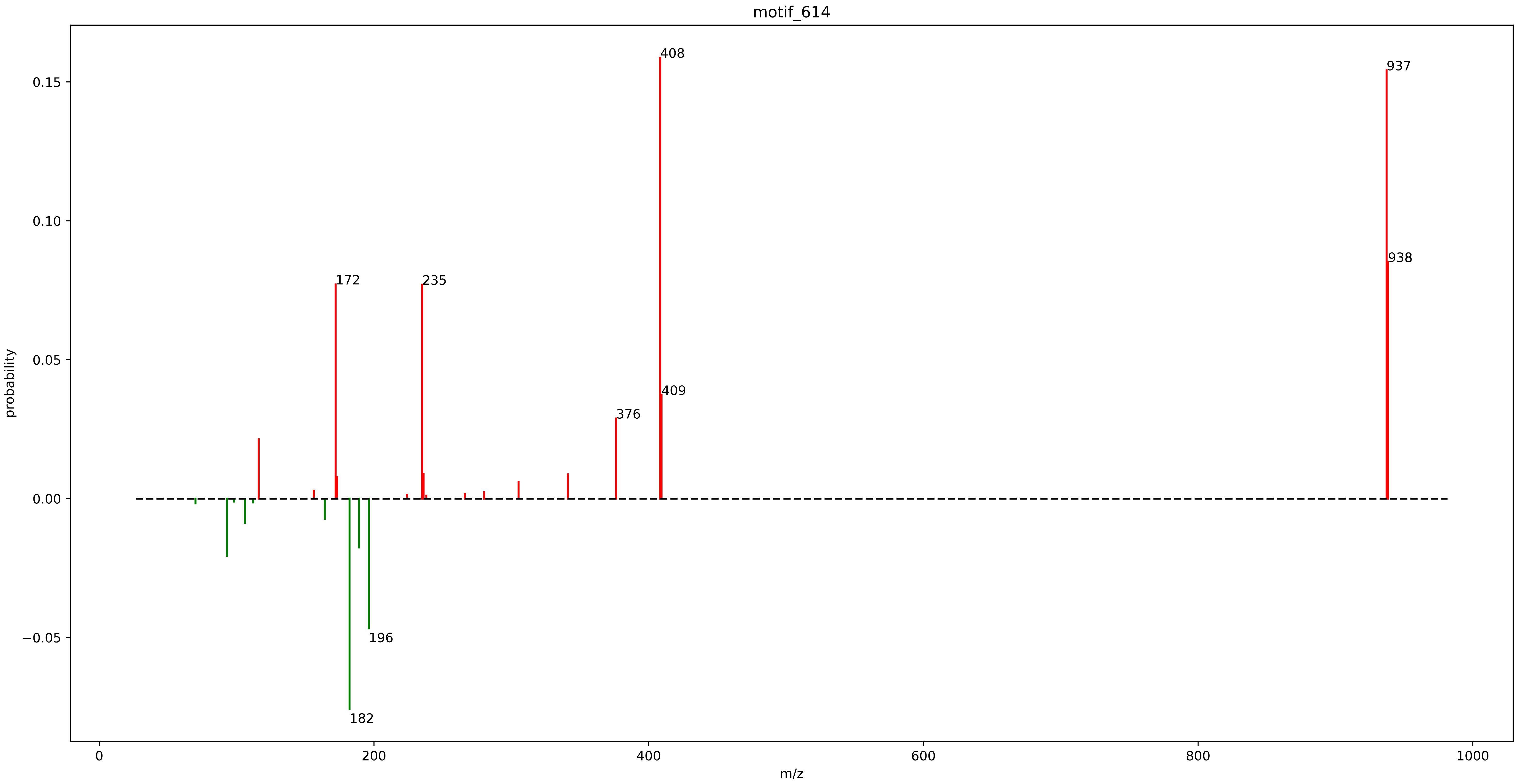

motif_614
probability
m/z
0.15
0.10
0.05
0.00
−0.05
0
200
400
600
800
1000
408
937
938
172
235
409
376
196
182

motif_614

fragment_408.3475	: 0.16
fragment_937.2025	: 0.15
fragment_938.2025	: 0.09
fragment_172.1125	: 0.08
fragment_235.1325	: 0.08
loss_182.2025	: 0.08
loss_196.2125	: 0.05
fragment_409.3525	: 0.04
fragment_376.3225	: 0.03
fragment_116.0525	: 0.02
loss_93.0475	: 0.02
loss_189.1175	: 0.02
fragment_236.1325	: 0.01
fragment_341.1975	: 0.01
loss_106.0725	: 0.01
fragment_173.1175	: 0.01
loss_164.1925	: 0.01
fragment_305.2825	: 0.01

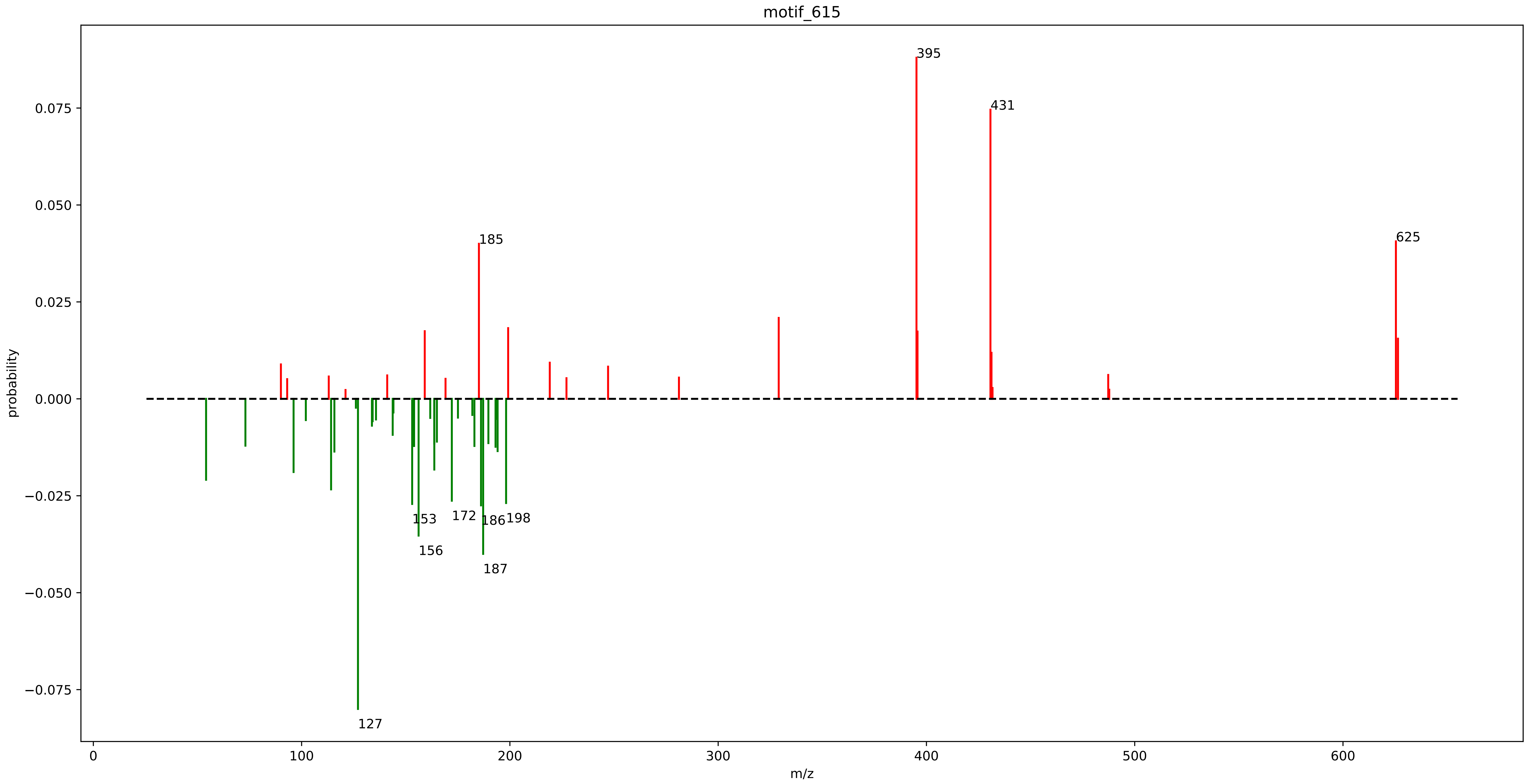

motif_615
probability
m/z
0.075
0.050
0.025
0.000
−0.025
−0.050
−0.075
0
100
200
300
400
500
600
395
431
185
625
153
172
186
198
156
187
127

motif_615

fragment_395.2175	: 0.09
loss_127.0625	: 0.08
fragment_430.7325	: 0.07
fragment_625.4075	: 0.04
fragment_185.1325	: 0.04
loss_187.1675	: 0.04
loss_156.1675	: 0.04
loss_186.1375	: 0.03
loss_153.0775	: 0.03
loss_198.1775	: 0.03
loss_172.1225	: 0.03
loss_114.1525	: 0.02
loss_54.1325	: 0.02
fragment_329.0725	: 0.02
loss_96.1425	: 0.02
fragment_199.1675	: 0.02
loss_163.7125	: 0.02
fragment_159.1175	: 0.02
fragment_395.7175	: 0.02
fragment_626.4125	: 0.02
loss_115.7375	: 0.01
loss_194.1075	: 0.01
loss_193.1075	: 0.01
loss_154.0125	: 0.01
loss_182.9675	: 0.01
loss_73.0175	: 0.01
fragment_431.2375	: 0.01
loss_163.7025	: 0.01
loss_189.6775	: 0.01
loss_164.9575	: 0.01
fragment_219.1475	: 0.01
loss_143.7325	: 0.01
fragment_90.0575	: 0.01
fragment_247.1425	: 0.01
loss_133.7625	: 0.01
fragment_487.2775	: 0.01
fragment_141.1025	: 0.01
loss_134.1075	: 0.01
fragment_113.0325	: 0.01
loss_102.0275	: 0.01
fragment_281.1725	: 0.01
loss_135.7125	: 0.01
fragment_227.1675	: 0.01
fragment_169.0975	: 0.01
loss_193.7825	: 0.01
fragment_93.0675	: 0.01

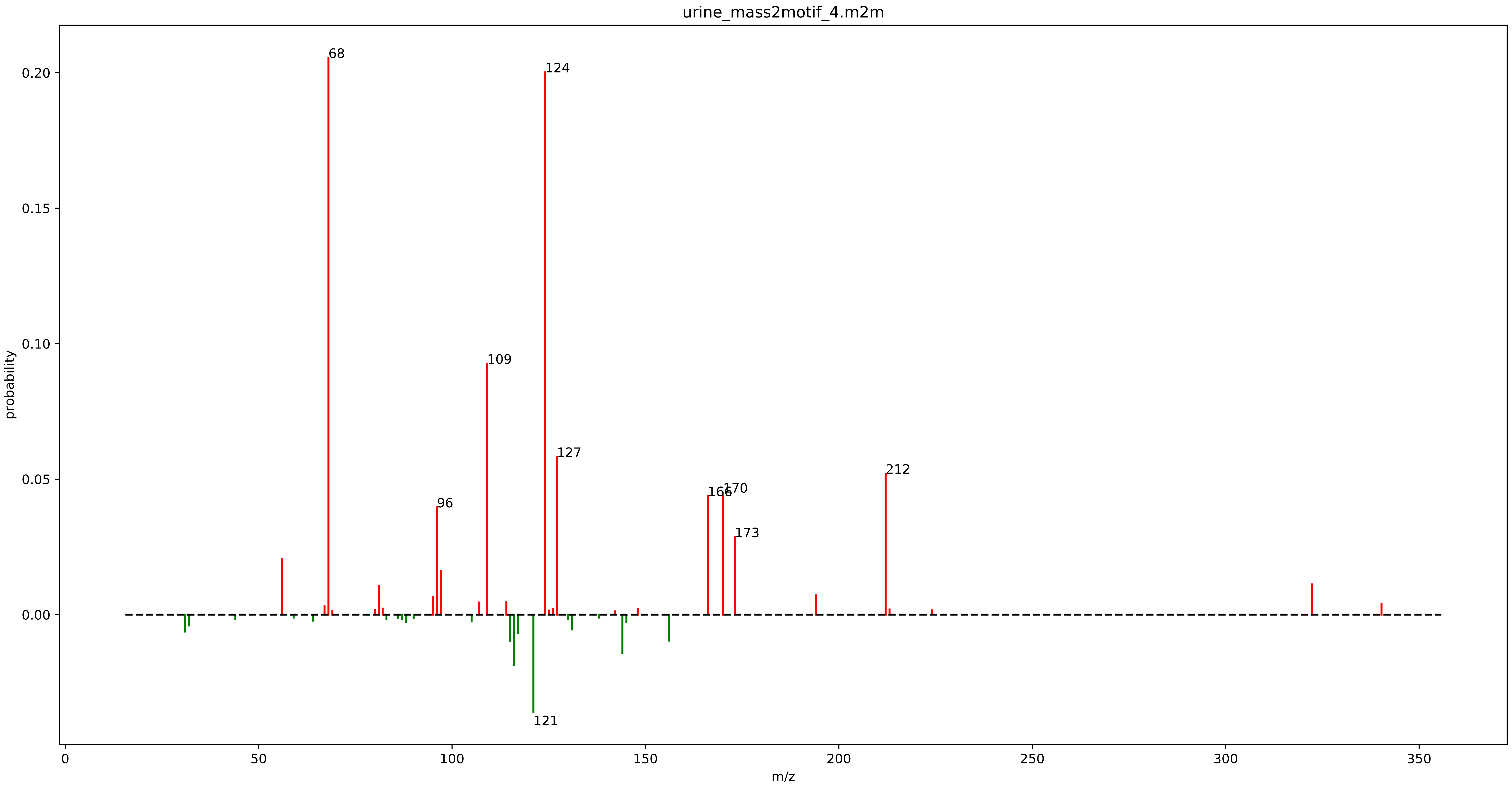
urine_mass2motif_4.m2m
probability
m/z
0.20
0.15
0.10
0.05
0.00
0
50
100
150
200
250
300
350
68
124
109
127
96
166
170
173
212
121

urine_mass2motif_4.m2m

fragment_68.0475	: 0.21
fragment_124.0875	: 0.20
fragment_109.0775	: 0.09
fragment_127.0875	: 0.06
fragment_212.1025	: 0.05
fragment_170.0925	: 0.05
fragment_166.0975	: 0.04
fragment_96.0675	: 0.04
loss_121.0375	: 0.04
fragment_173.0925	: 0.03
fragment_56.0475	: 0.02
loss_116.0425	: 0.02
fragment_97.0775	: 0.02
loss_144.0475	: 0.01
fragment_322.2725	: 0.01
fragment_81.0425	: 0.01
loss_115.0425	: 0.01
loss_156.0775	: 0.01
fragment_194.0925	: 0.01
loss_117.0525	: 0.01
fragment_95.0625	: 0.01
loss_31.0175	: 0.01
loss_131.0675	: 0.01

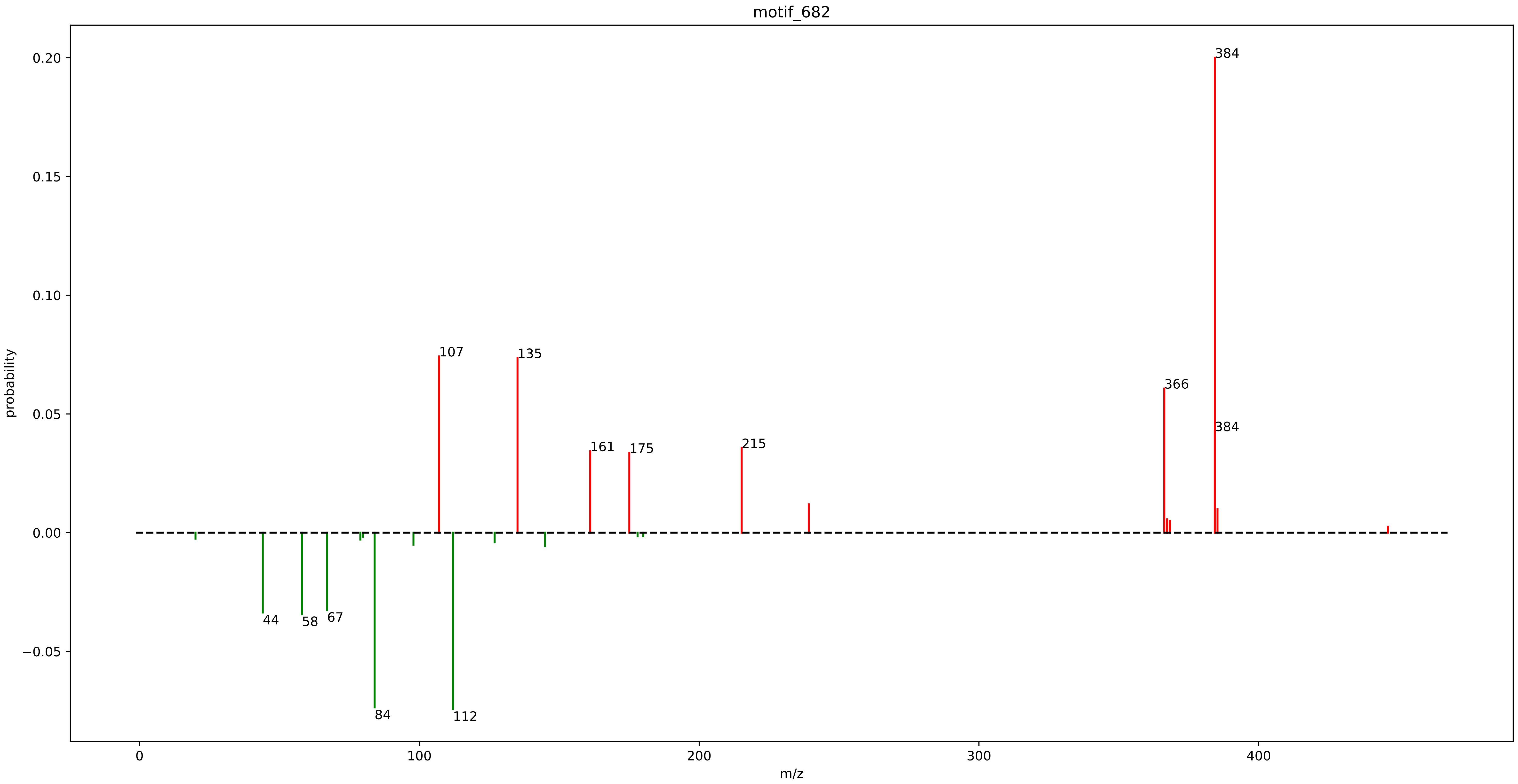
motif_682
probability
m/z
0.20
0.15
0.10
0.05
0.00
−0.05
0
100
200
300
400
384
107
135
366
384
161
175
215
44
58
67
84
112

motif_682

fragment_384.3075	: 0.20
loss_112.0075	: 0.07
fragment_107.0825	: 0.07
fragment_135.0825	: 0.07
loss_84.0125	: 0.07
fragment_366.2425	: 0.06
fragment_384.2525	: 0.04
fragment_215.1775	: 0.04
fragment_161.0625	: 0.03
loss_58.0325	: 0.03
fragment_175.0575	: 0.03
loss_44.0325	: 0.03
loss_67.0325	: 0.03
fragment_239.1775	: 0.01
fragment_385.2575	: 0.01
loss_144.9275	: 0.01
fragment_367.2375	: 0.01
fragment_368.2725	: 0.01
loss_97.8975	: 0.01

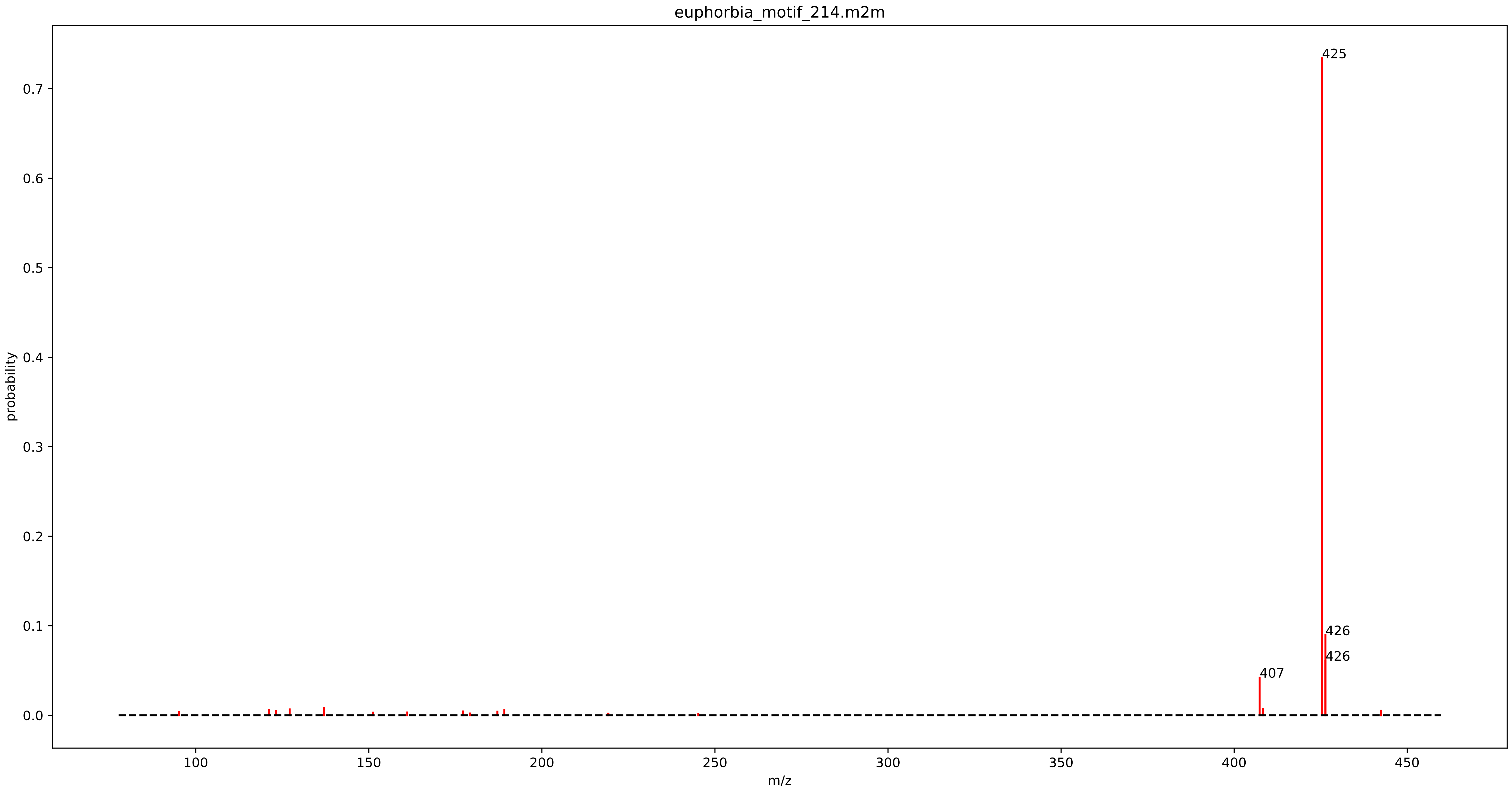
euphorbia_motif_214.m2m
probability
m/z
425
426
426
407
0.0
0.1
0.2
0.3
0.4
0.5
0.6
0.7
100
150
200
250
300
350
400
450

euphorbia_motif_214.m2m

fragment_425.3775	: 0.73
fragment_426.3825	: 0.09
fragment_426.3775	: 0.06
fragment_407.3675	: 0.04
fragment_137.1325	: 0.01
fragment_408.3675	: 0.01
fragment_127.1125	: 0.01
fragment_121.1025	: 0.01
fragment_189.1625	: 0.01
fragment_408.3725	: 0.01
fragment_442.4025	: 0.01

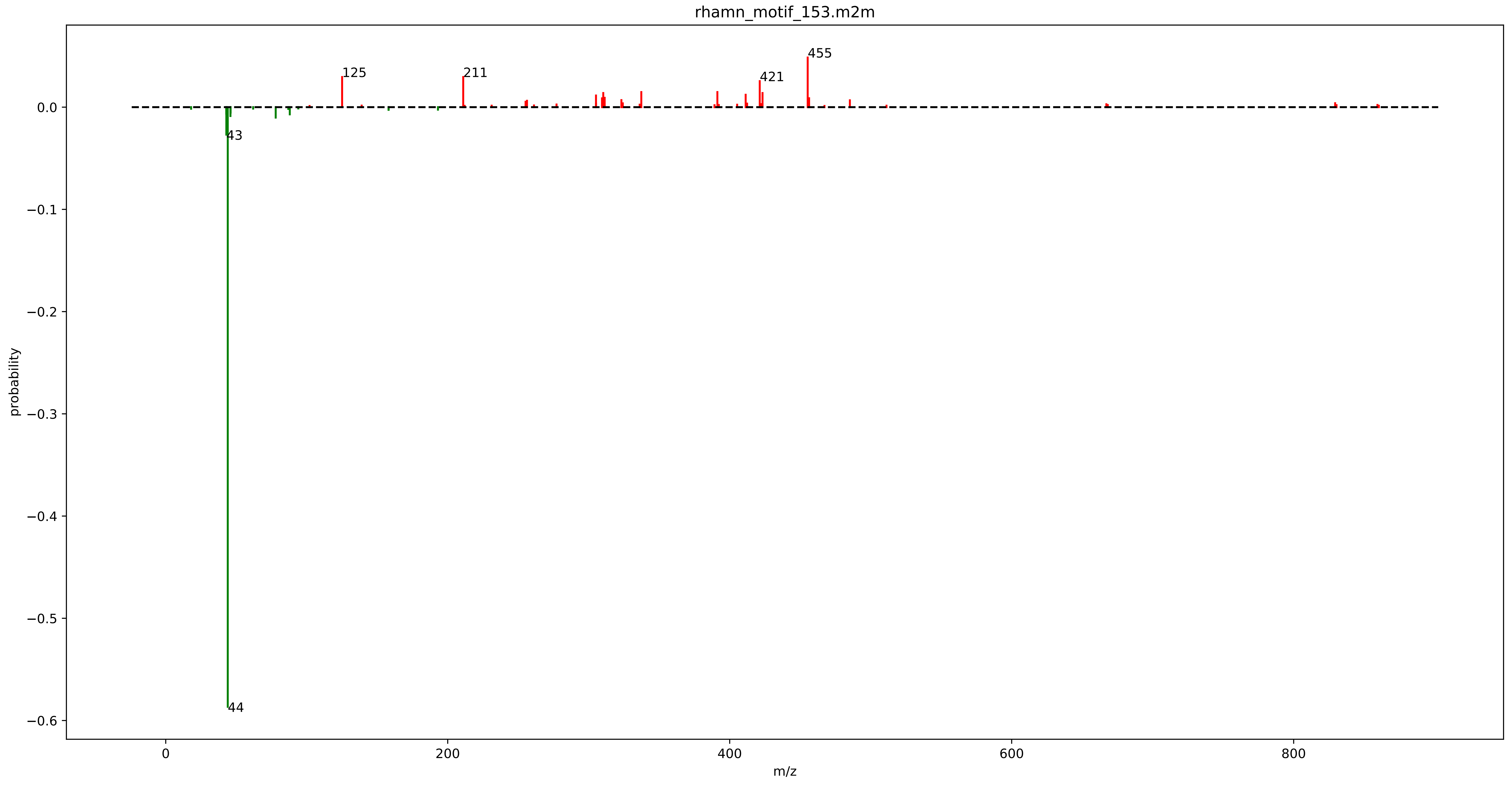

rhamn_motif_153.m2m
probability
m/z
0.0
−0.1
−0.2
−0.3
−0.4
−0.5
−0.6
0
200
400
600
800
125
211
421
455
43
44

rhamn_motif_153.m2m

loss_43.9925	: 0.59
fragment_455.3125	: 0.05
fragment_125.0975	: 0.03
fragment_210.9925	: 0.03
loss_42.9825	: 0.03
fragment_421.3075	: 0.03
fragment_391.2625	: 0.01
fragment_337.3125	: 0.01
fragment_310.3225	: 0.01
fragment_423.2875	: 0.01
fragment_411.3225	: 0.01
fragment_305.1725	: 0.01
loss_77.9975	: 0.01
fragment_311.3275	: 0.01
fragment_456.3225	: 0.01
loss_45.9025	: 0.01
fragment_309.4225	: 0.01
loss_87.9825	: 0.01
fragment_323.1875	: 0.01
fragment_485.2325	: 0.01
loss_42.9775	: 0.01
fragment_256.2325	: 0.01
loss_42.9975	: 0.01
loss_46.0125	: 0.01
fragment_255.2225	: 0.01

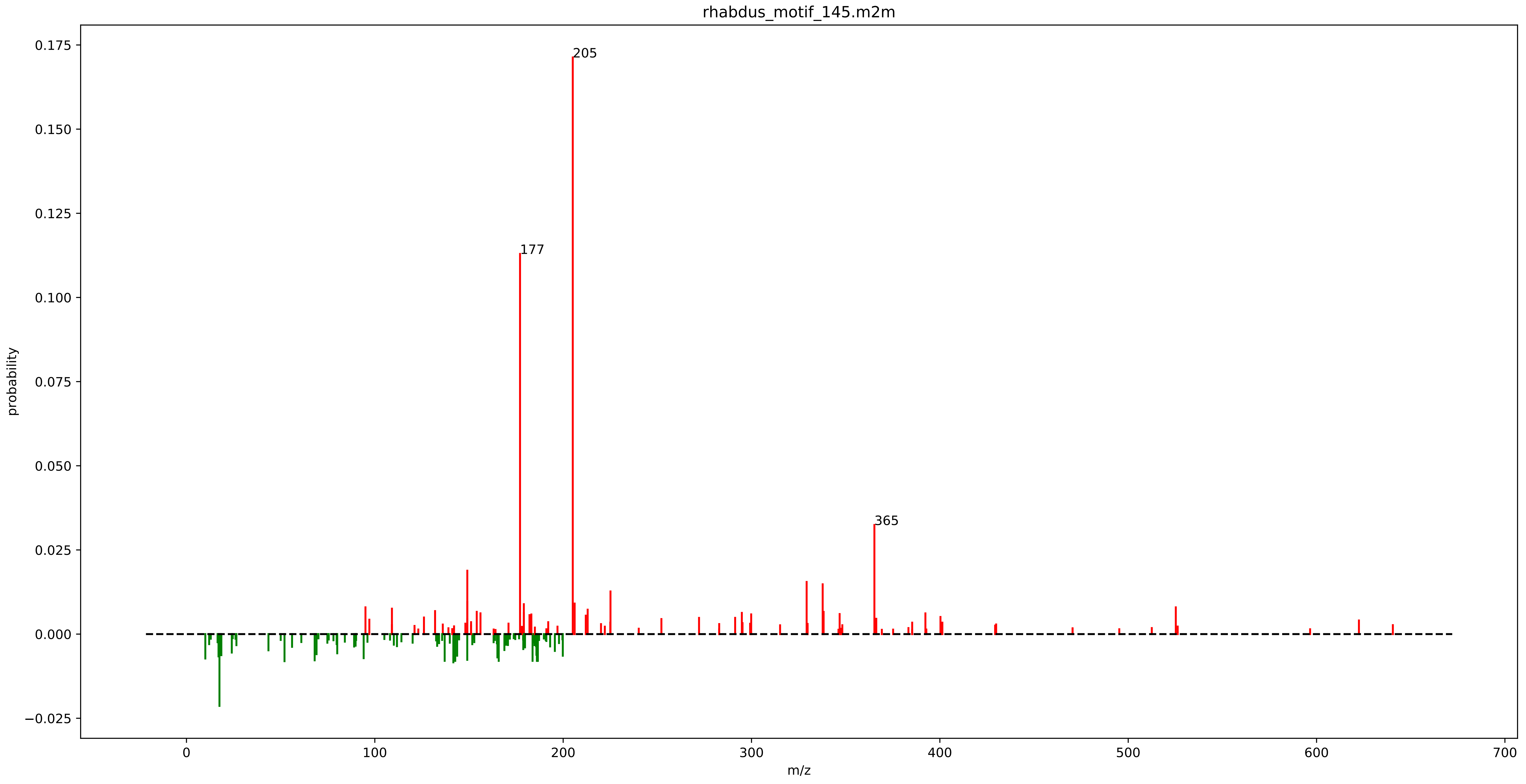

rhabdus_motif_145.m2m
205
177
365
probability
m/z
0.175
0.150
0.125
0.100
0.075
0.050
0.025
0.000
−0.025
0
100
200
300
400
500
600
700

rhabdus_motif_145.m2m

fragment_205.0975	: 0.17
fragment_177.1025	: 0.11
fragment_365.2425	: 0.03
loss_17.5175	: 0.02
fragment_149.1075	: 0.02
fragment_329.2675	: 0.02
fragment_337.7825	: 0.01
fragment_225.1225	: 0.01
fragment_149.1025	: 0.01
fragment_206.1025	: 0.01
fragment_179.1175	: 0.01
loss_141.6925	: 0.01
loss_52.0525	: 0.01
loss_142.6975	: 0.01
fragment_525.2675	: 0.01
fragment_95.0325	: 0.01
loss_186.0475	: 0.01
loss_165.7875	: 0.01
loss_186.5625	: 0.01
loss_137.0875	: 0.01
loss_183.7525	: 0.01
loss_68.0625	: 0.01
loss_149.0825	: 0.01
fragment_109.0875	: 0.01
fragment_213.0125	: 0.01
loss_10.0125	: 0.01
loss_94.0775	: 0.01
loss_165.0825	: 0.01
fragment_131.9925	: 0.01
loss_17.0175	: 0.01
fragment_154.1175	: 0.01
fragment_338.2825	: 0.01
loss_199.8075	: 0.01
loss_143.7175	: 0.01
fragment_294.8625	: 0.01
loss_18.5275	: 0.01
fragment_179.1125	: 0.01
fragment_156.1025	: 0.01
fragment_392.3225	: 0.01
loss_185.8025	: 0.01
loss_69.0675	: 0.01
fragment_346.7875	: 0.01
fragment_299.8225	: 0.01
fragment_183.1125	: 0.01
loss_80.0525	: 0.01
fragment_182.1175	: 0.01
loss_24.0575	: 0.01
fragment_212.0075	: 0.01
fragment_400.3175	: 0.01

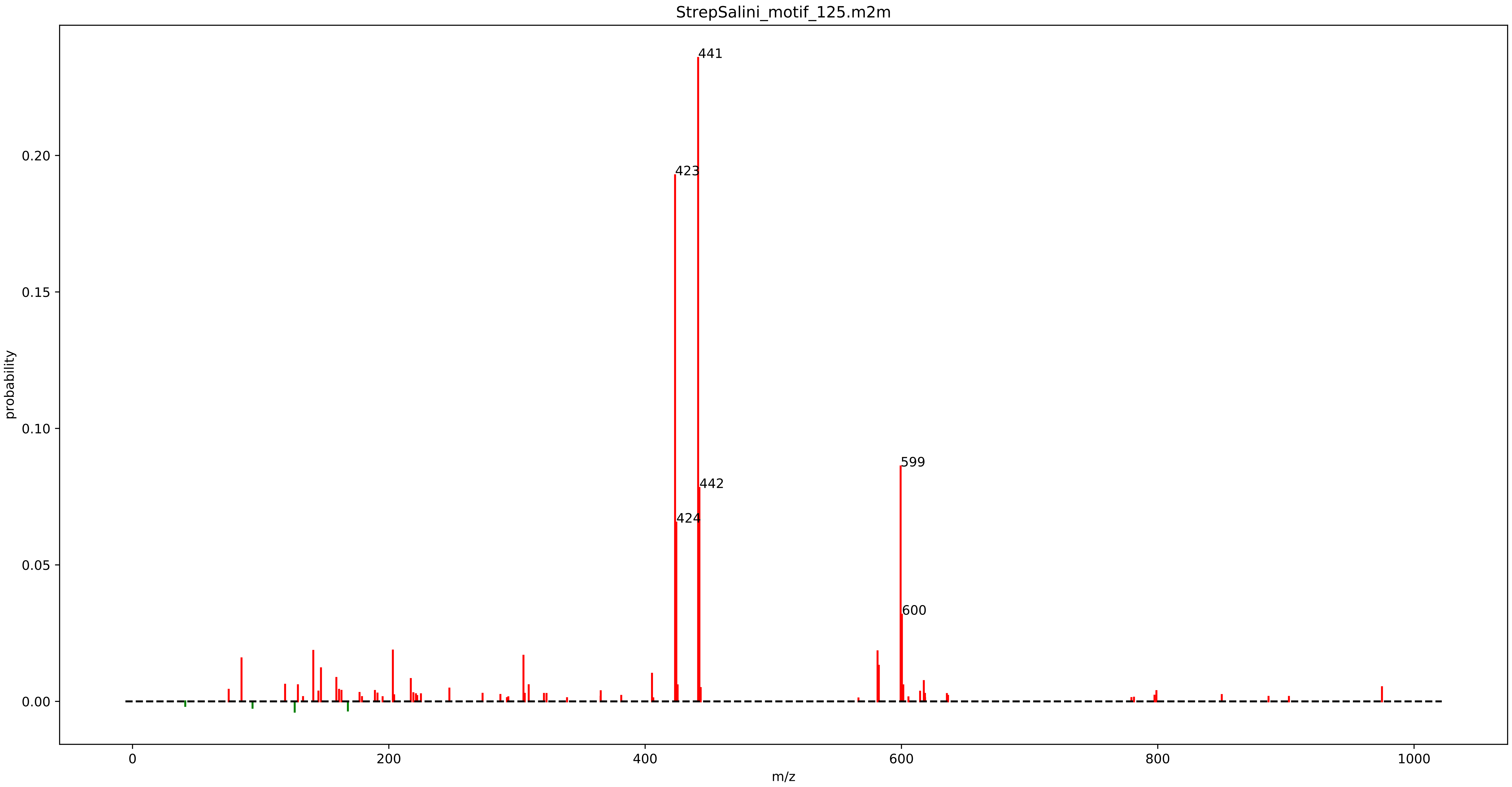

StrepSalini_motif_125.m2m
probability
m/z
441
423
599
442
424
600
0.20
0.15
0.10
0.05
0.00
0
200
400
600
800
1000

StrepSalini_motif_125.m2m

fragment_441.3500	: 0.24
fragment_423.3525	: 0.19
fragment_599.3500	: 0.09
fragment_442.3525	: 0.08
fragment_424.3500	: 0.07
fragment_600.3500	: 0.03
fragment_203.1500	: 0.02
fragment_141.0500	: 0.02
fragment_581.3500	: 0.02
fragment_305.0500	: 0.02
fragment_85.0500	: 0.02
fragment_582.3500	: 0.01
fragment_147.0500	: 0.01
fragment_405.3525	: 0.01
fragment_159.0500	: 0.01
fragment_217.1525	: 0.01
fragment_617.4500	: 0.01
fragment_119.0500	: 0.01
fragment_309.1500	: 0.01
fragment_129.0525	: 0.01
fragment_425.3500	: 0.01
fragment_601.4500	: 0.01
fragment_974.9500	: 0.01
fragment_599.4500	: 0.01

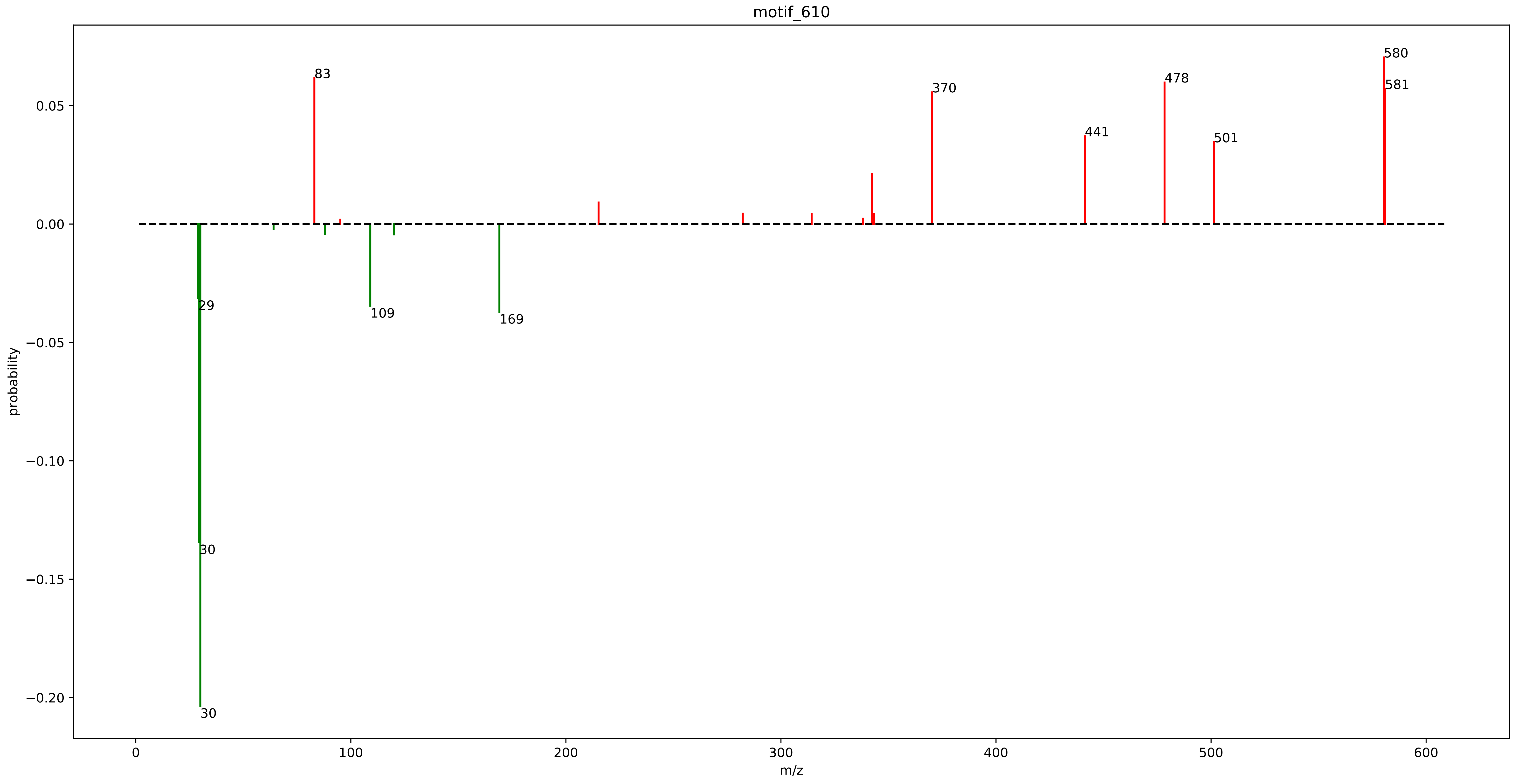

motif_610
probability
m/z
0.05
0.00
−0.05
−0.10
−0.15
−0.20
0
100
200
300
400
500
600
83
370
441
478
501
580
581
29
30
30
109
169

motif_610

loss_30.0075	: 0.20
loss_29.5075	: 0.13
fragment_580.3625	: 0.07
fragment_83.0475	: 0.06
fragment_478.3675	: 0.06
fragment_580.8625	: 0.06
fragment_370.2575	: 0.06
loss_169.0825	: 0.04
fragment_441.2875	: 0.04
loss_109.0625	: 0.03
fragment_501.3075	: 0.03
loss_29.0075	: 0.03
fragment_342.2625	: 0.02
fragment_215.1775	: 0.01

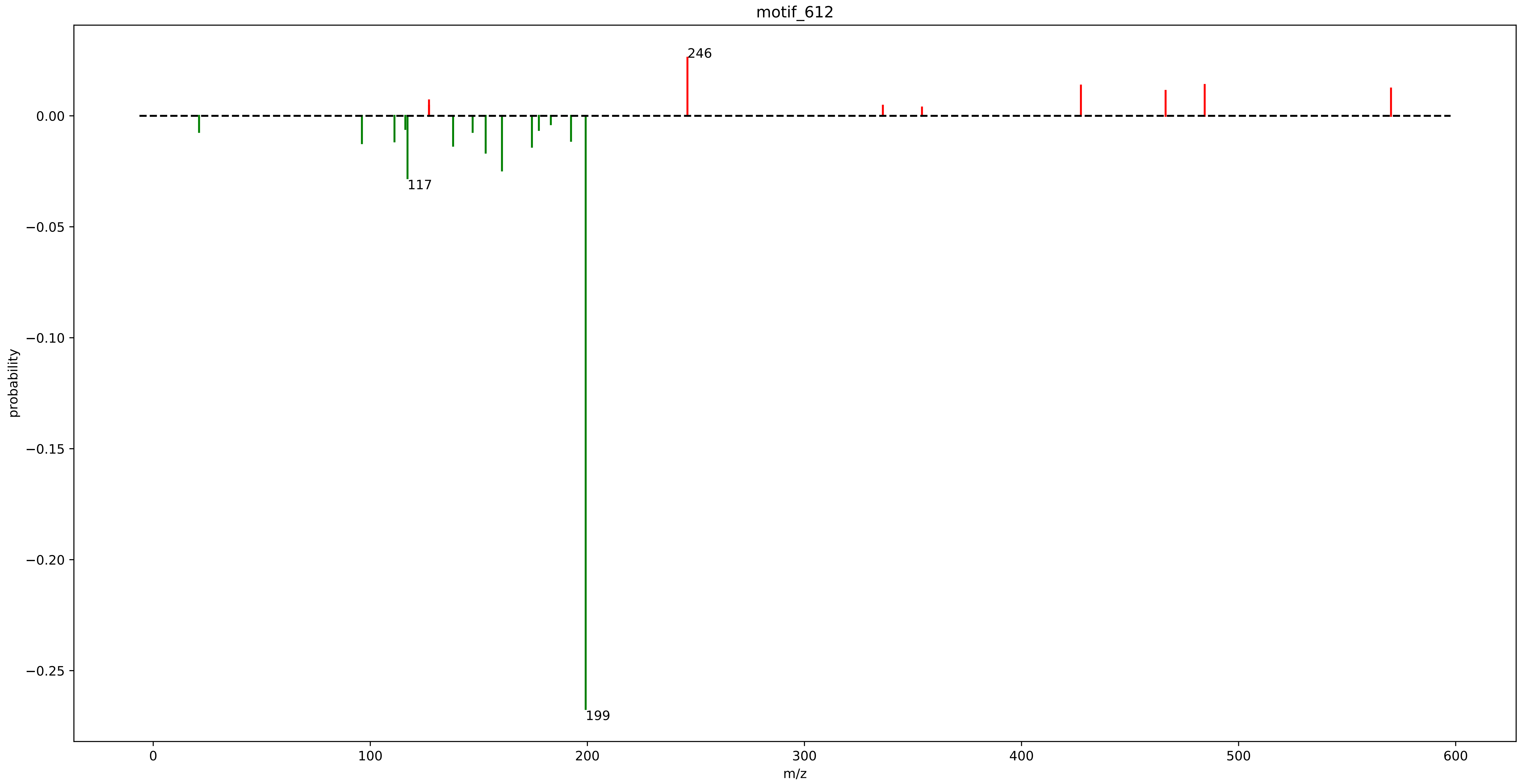

motif_612
246
117
199
0.00
−0.05
−0.10
−0.15
−0.20
−0.25
probability
0
100
200
300
400
500
600
m/z

motif_612

loss_199.2175	: 0.27
loss_117.1425	: 0.03
fragment_246.0975	: 0.03
loss_160.6575	: 0.02
loss_153.1525	: 0.02
loss_174.4575	: 0.01
fragment_484.3775	: 0.01
fragment_427.3575	: 0.01
loss_138.1475	: 0.01
loss_96.1375	: 0.01
fragment_570.2025	: 0.01
loss_111.1375	: 0.01
loss_192.4675	: 0.01
fragment_466.3675	: 0.01
loss_21.1175	: 0.01
loss_147.1525	: 0.01
fragment_127.0425	: 0.01
loss_177.6575	: 0.01
loss_116.1375	: 0.01

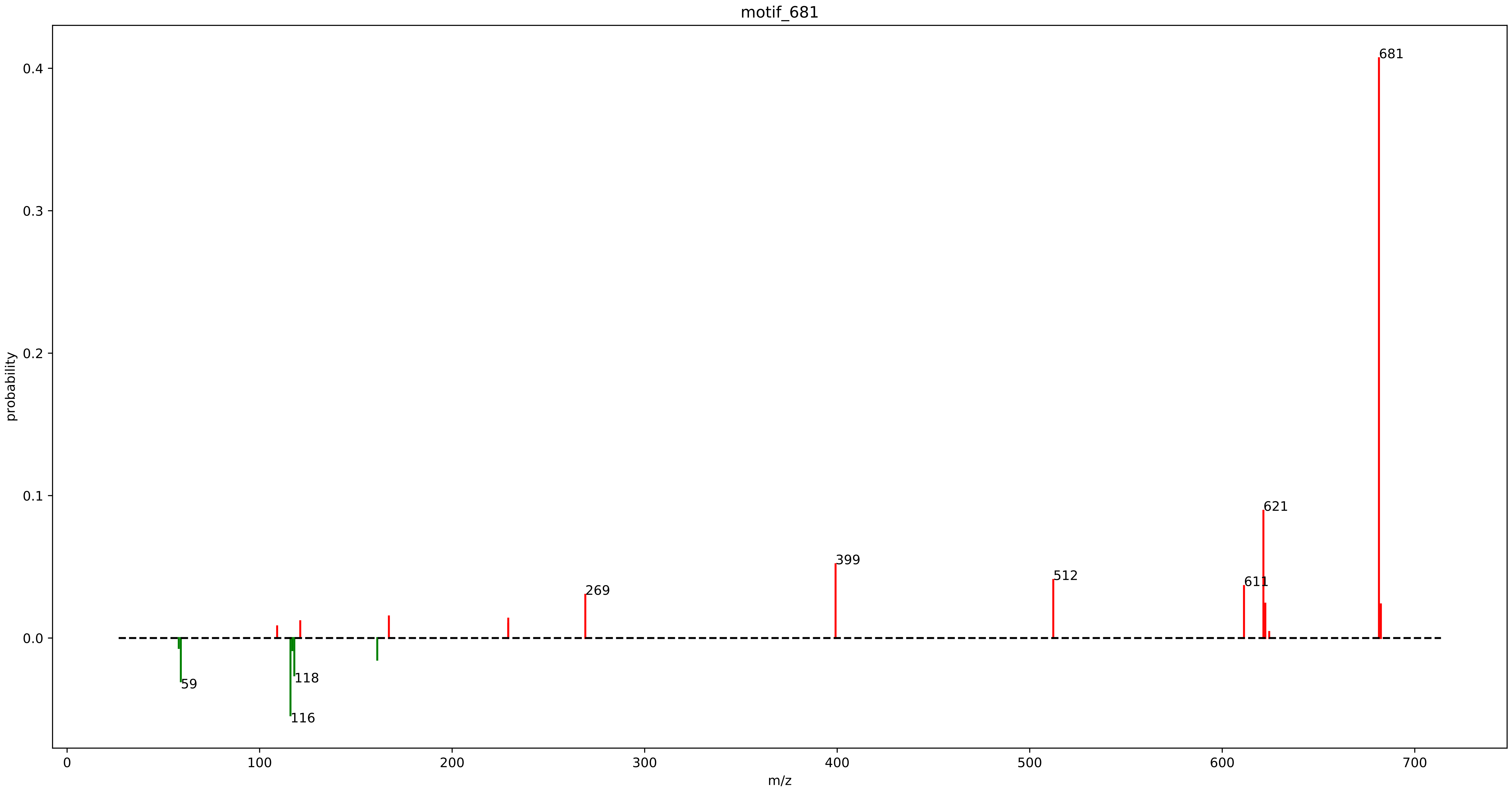
motif_681
probability
m/z
0.4
0.3
0.2
0.1
0.0
0
100
200
300
400
500
600
700
681
621
399
512
611
269
59
118
116

motif_681

fragment_681.3975	: 0.41
fragment_621.3775	: 0.09
loss_116.0275	: 0.05
fragment_399.1525	: 0.05
fragment_512.2375	: 0.04
fragment_611.3025	: 0.04
loss_59.0725	: 0.03
fragment_269.1725	: 0.03
loss_118.0225	: 0.03
fragment_622.3825	: 0.02
fragment_682.4025	: 0.02
loss_161.1025	: 0.02
fragment_167.1425	: 0.02
fragment_229.1475	: 0.01
fragment_121.1025	: 0.01
fragment_229.1225	: 0.01
loss_117.0175	: 0.01
fragment_109.0975	: 0.01
loss_58.0025	: 0.01

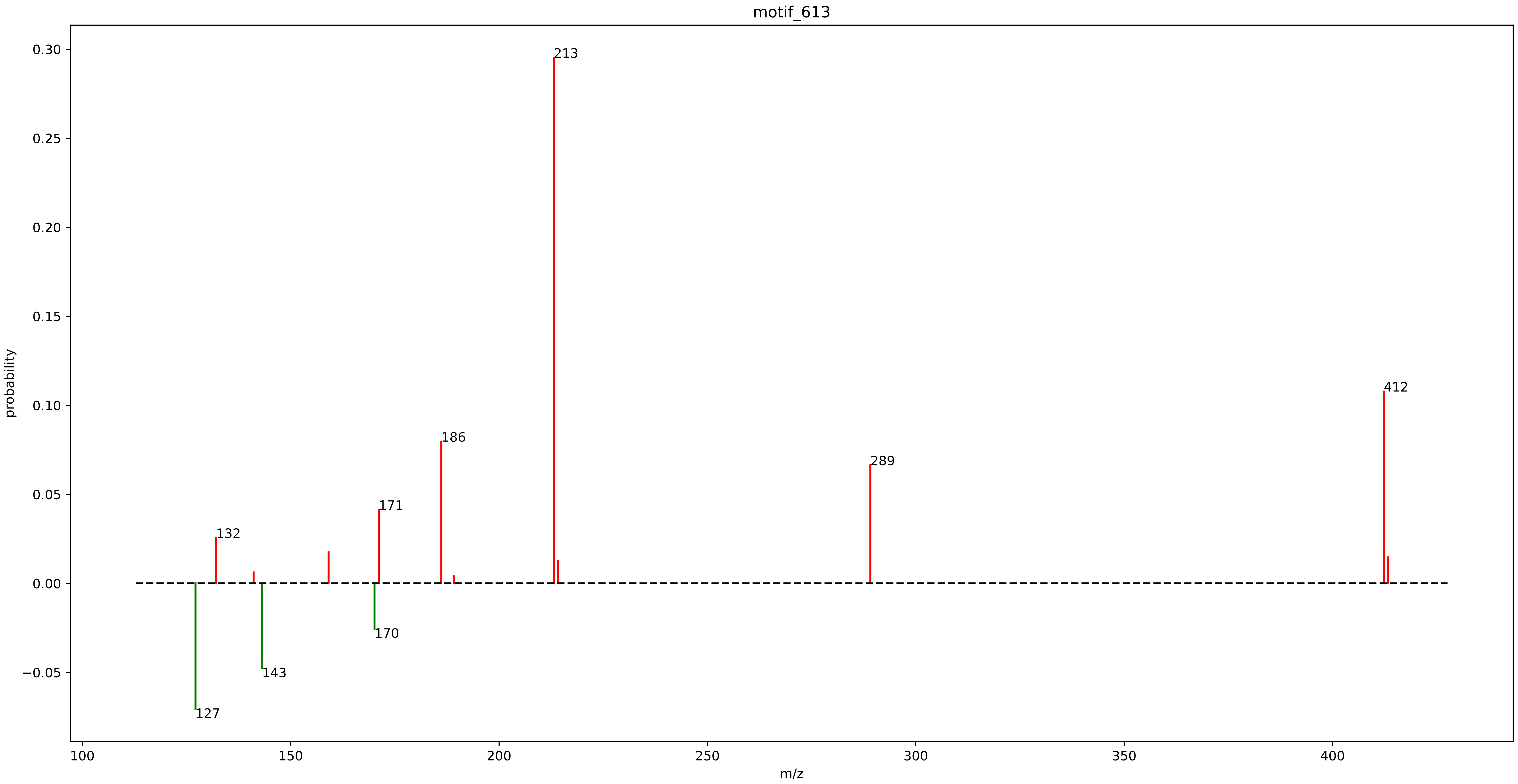

motif_613
probability
m/z
0.30
0.25
0.20
0.15
0.10
0.05
0.00
−0.05
100
150
200
250
300
350
400
213
412
186
289
171
132
170
143
127

motif_613

fragment_213.1225	: 0.30
fragment_412.2825	: 0.11
fragment_186.1275	: 0.08
loss_127.1575	: 0.07
fragment_289.0875	: 0.07
loss_143.1125	: 0.05
fragment_171.1125	: 0.04
loss_170.1075	: 0.03
fragment_132.0975	: 0.03
fragment_159.0925	: 0.02
fragment_413.2875	: 0.01
fragment_214.1275	: 0.01
fragment_141.1025	: 0.01

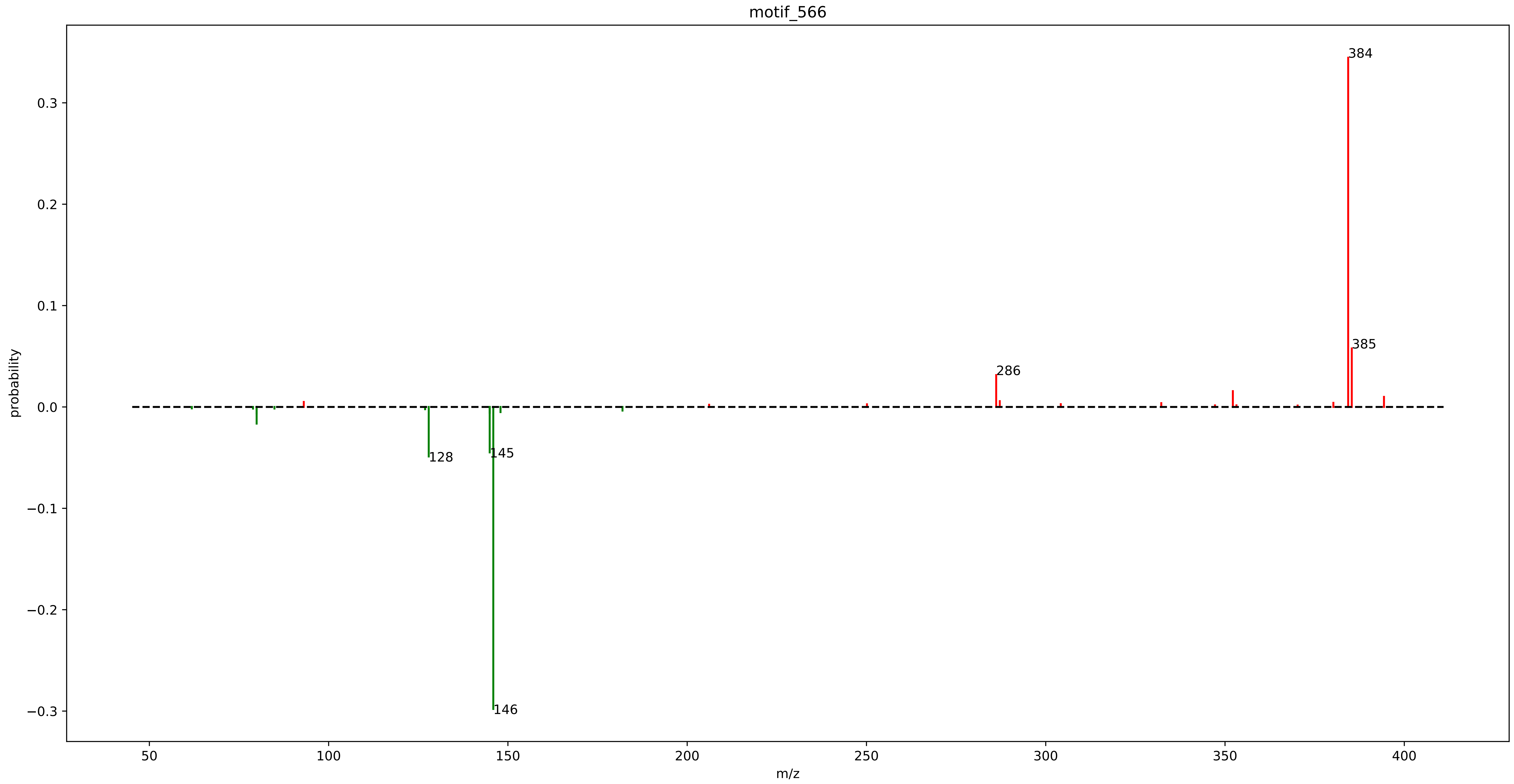

motif_566
probability
m/z
384
385
286
128
145
146
0.3
0.2
0.1
0.0
−0.1
−0.2
−0.3
50
100
150
200
250
300
350
400

motif_566

fragment_384.3475	: 0.34
loss_145.9225	: 0.30
fragment_385.3525	: 0.06
loss_127.9125	: 0.05
loss_144.9175	: 0.04
fragment_286.1775	: 0.03
loss_79.9175	: 0.02
fragment_352.1875	: 0.02
fragment_394.3325	: 0.01
loss_79.9125	: 0.01
fragment_287.1775	: 0.01
loss_144.9225	: 0.01
loss_147.9375	: 0.01

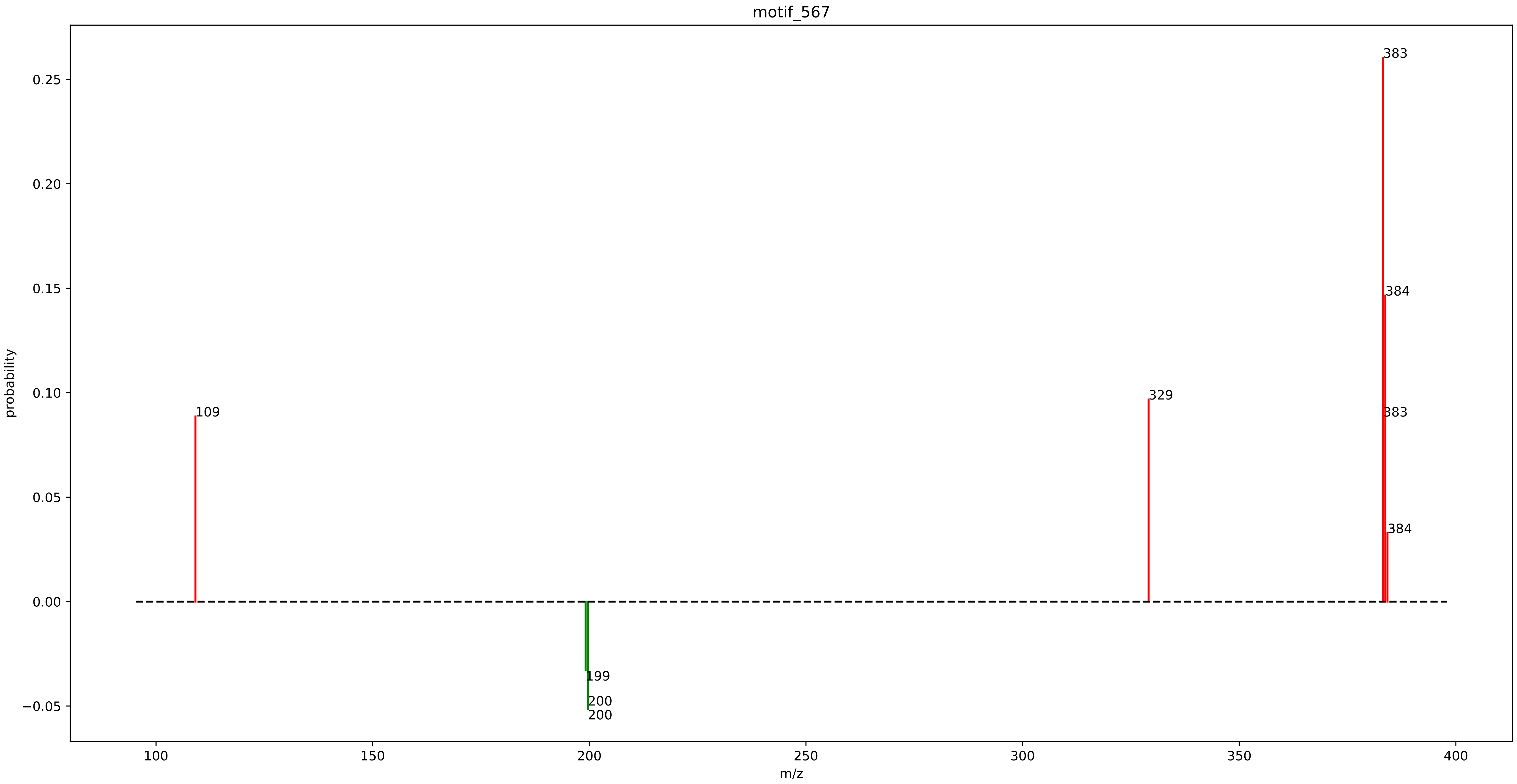

motif_567
probability
m/z
383
384
383
384
329
109
199
200
200
0.25
0.20
0.15
0.10
0.05
0.00
−0.05
100
150
200
250
300
350
400

motif_567

fragment_383.2075	: 0.26
fragment_383.7075	: 0.15
fragment_329.0725	: 0.10
fragment_109.0975	: 0.09
fragment_383.2025	: 0.09
loss_199.6475	: 0.05
loss_199.6425	: 0.04
loss_199.1475	: 0.03
fragment_384.2075	: 0.03

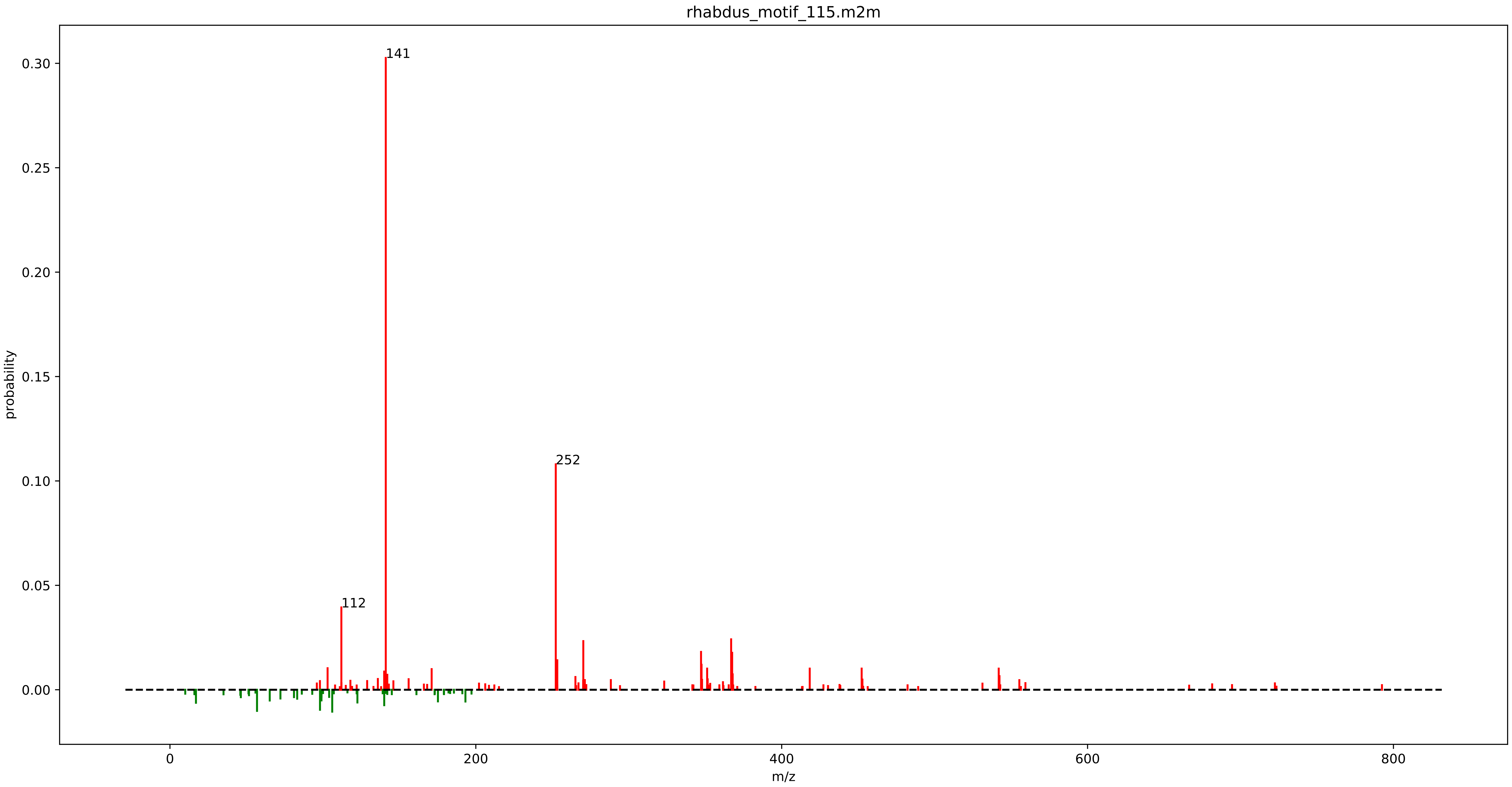
rhabdus_motif_115.m2m
probability
m/z
141
252
112
0.30
0.25
0.20
0.15
0.10
0.05
0.00
0
200
400
600
800

rhabdus_motif_115.m2m

fragment_141.1125	: 0.30
fragment_252.2675	: 0.11
fragment_112.0825	: 0.04
fragment_366.9375	: 0.02
fragment_270.2775	: 0.02
fragment_347.2575	: 0.02
fragment_367.6075	: 0.02
fragment_367.2725	: 0.02
fragment_253.2725	: 0.01
fragment_347.5925	: 0.01
loss_106.1025	: 0.01
fragment_103.0725	: 0.01
loss_56.9325	: 0.01
fragment_351.2475	: 0.01
fragment_452.2825	: 0.01
fragment_418.3325	: 0.01
fragment_541.9025	: 0.01
fragment_171.1175	: 0.01
loss_98.1125	: 0.01
fragment_140.0675	: 0.01
loss_140.1025	: 0.01
fragment_367.9425	: 0.01
fragment_142.1175	: 0.01
fragment_542.4075	: 0.01
loss_17.0025	: 0.01
fragment_265.1225	: 0.01
loss_122.5725	: 0.01
loss_193.2075	: 0.01
loss_175.2275	: 0.01
loss_65.2425	: 0.01
fragment_135.9425	: 0.01
loss_99.0775	: 0.01
fragment_156.0675	: 0.01

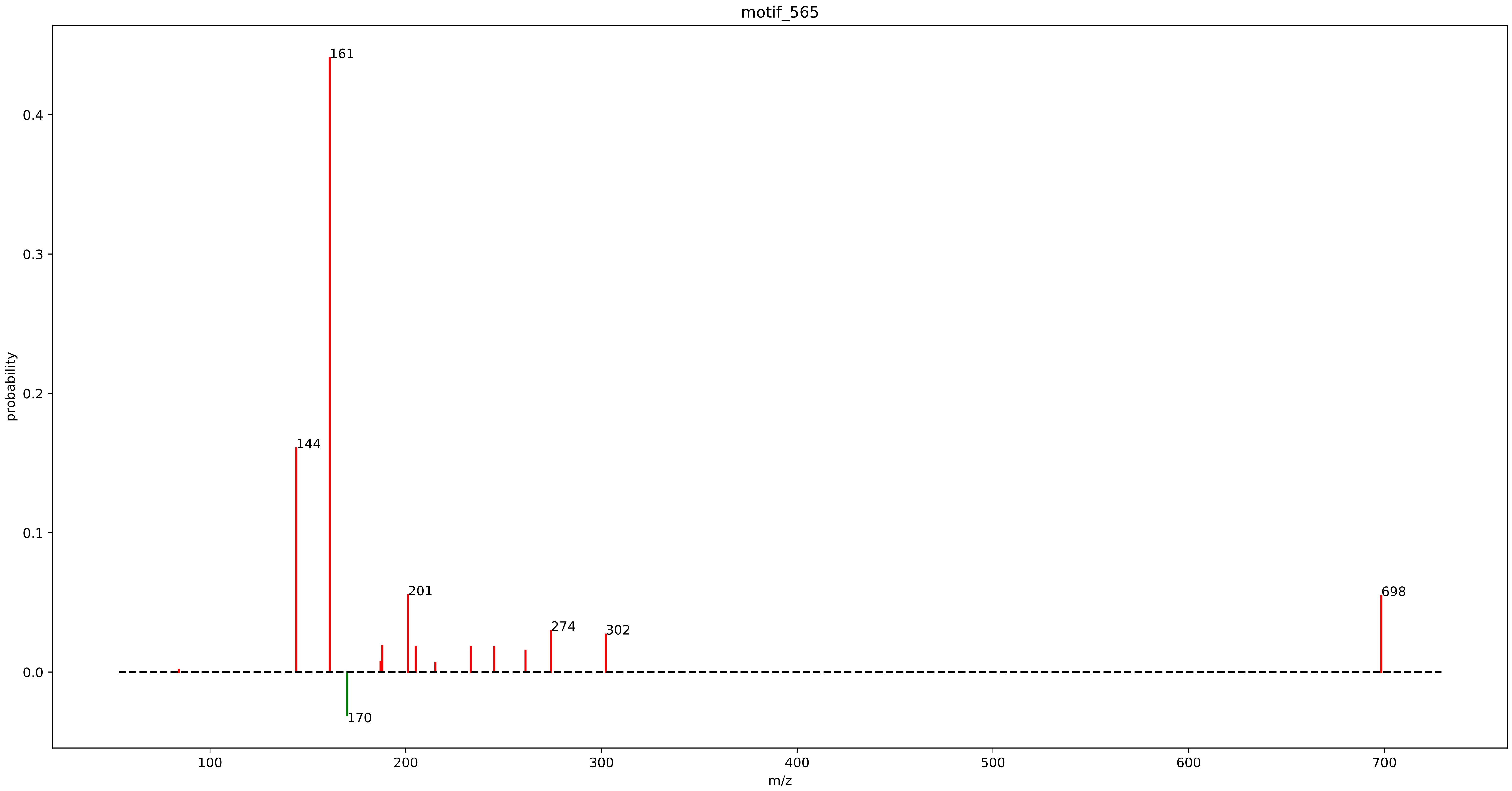

motif_565
161
144
201
274
302
698
170
probability
m/z
0.0
0.1
0.2
0.3
0.4
100
200
300
400
500
600
700

motif_565

fragment_161.0925	: 0.44
fragment_144.0675	: 0.16
fragment_201.1225	: 0.06
fragment_698.4275	: 0.05
loss_170.0675	: 0.03
fragment_274.1775	: 0.03
fragment_302.1325	: 0.03
fragment_188.0375	: 0.02
fragment_205.0625	: 0.02
fragment_233.1625	: 0.02
fragment_245.1125	: 0.02
fragment_261.1575	: 0.02
fragment_187.1125	: 0.01
fragment_215.1325	: 0.01

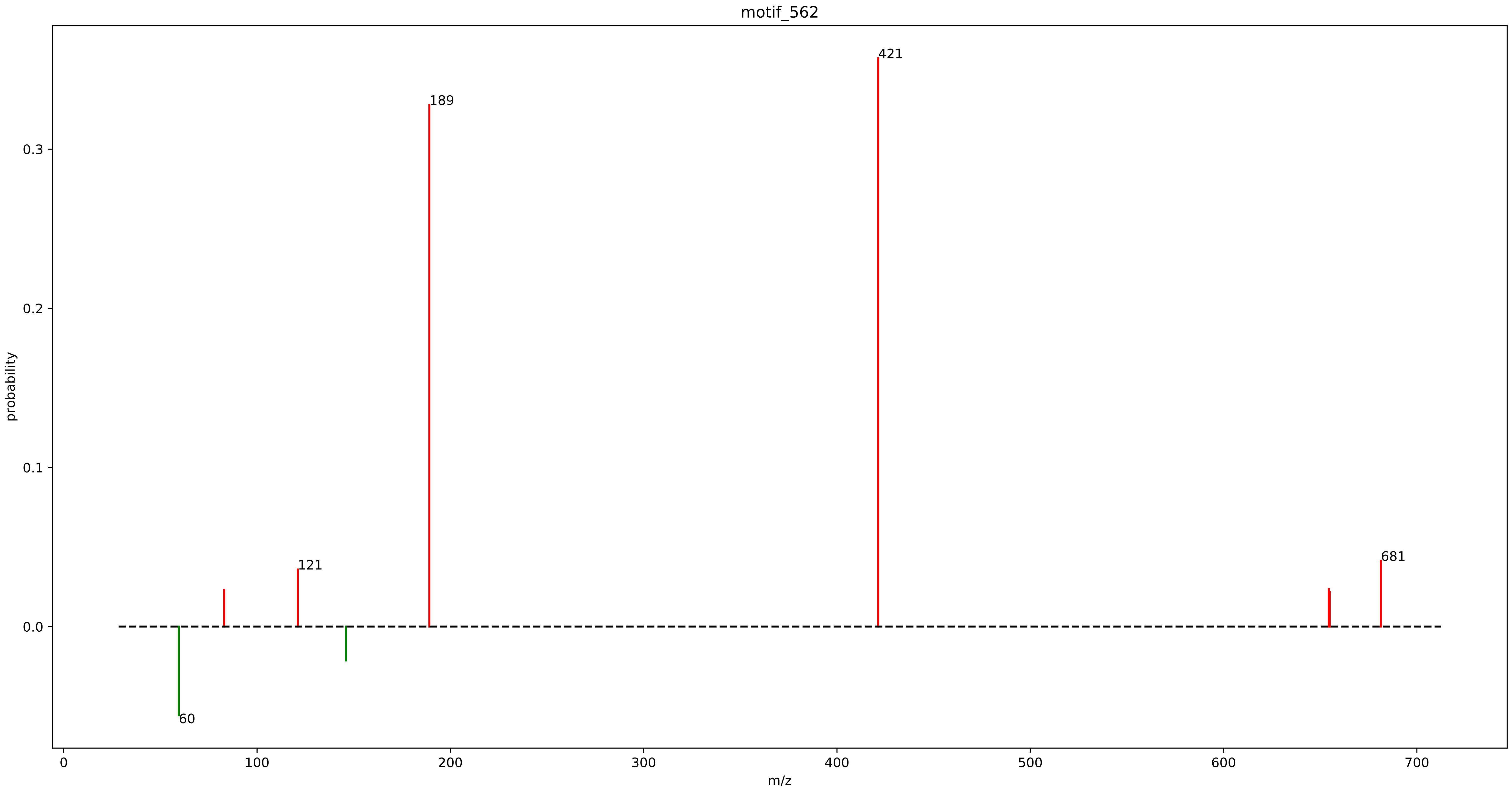
motif_562
probability
m/z
0.3
0.2
0.1
0.0
0
100
200
300
400
500
600
700
189
421
121
681
60

motif_562

fragment_421.3475	: 0.36
fragment_189.1625	: 0.33
loss_59.5125	: 0.06
fragment_681.3675	: 0.04
fragment_121.1025	: 0.04
fragment_654.4075	: 0.02
fragment_83.0475	: 0.02
fragment_654.9075	: 0.02
loss_146.0525	: 0.02

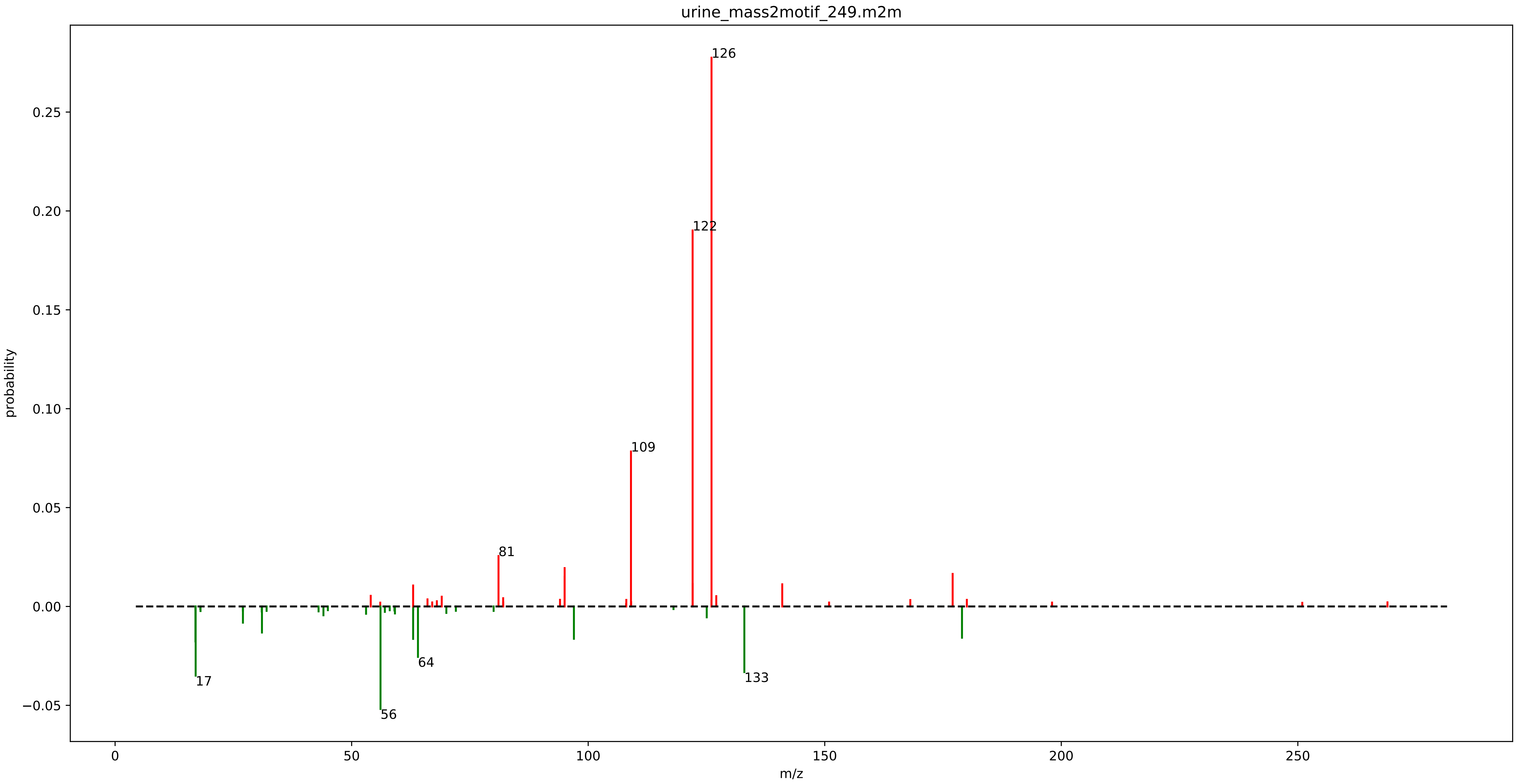

urine_mass2motif_249.m2m
probability
m/z
0.25
0.20
0.15
0.10
0.05
0.00
−0.05
0
50
100
150
200
250
126
122
109
81
17
56
64
133

urine_mass2motif_249.m2m

fragment_126.0675	: 0.28
fragment_122.0725	: 0.19
fragment_109.0425	: 0.08
loss_56.1025	: 0.05
loss_17.0275	: 0.03
loss_133.0075	: 0.03
loss_64.0175	: 0.03
fragment_81.0425	: 0.03
fragment_95.0225	: 0.02
loss_16.9825	: 0.02
fragment_177.0375	: 0.02
loss_62.9975	: 0.02
loss_96.9825	: 0.02
loss_179.0125	: 0.02
fragment_95.0125	: 0.01
loss_31.0425	: 0.01
fragment_122.0825	: 0.01
fragment_141.0175	: 0.01
fragment_62.9975	: 0.01
loss_27.0225	: 0.01
loss_125.0575	: 0.01
fragment_54.0325	: 0.01
fragment_127.0725	: 0.01

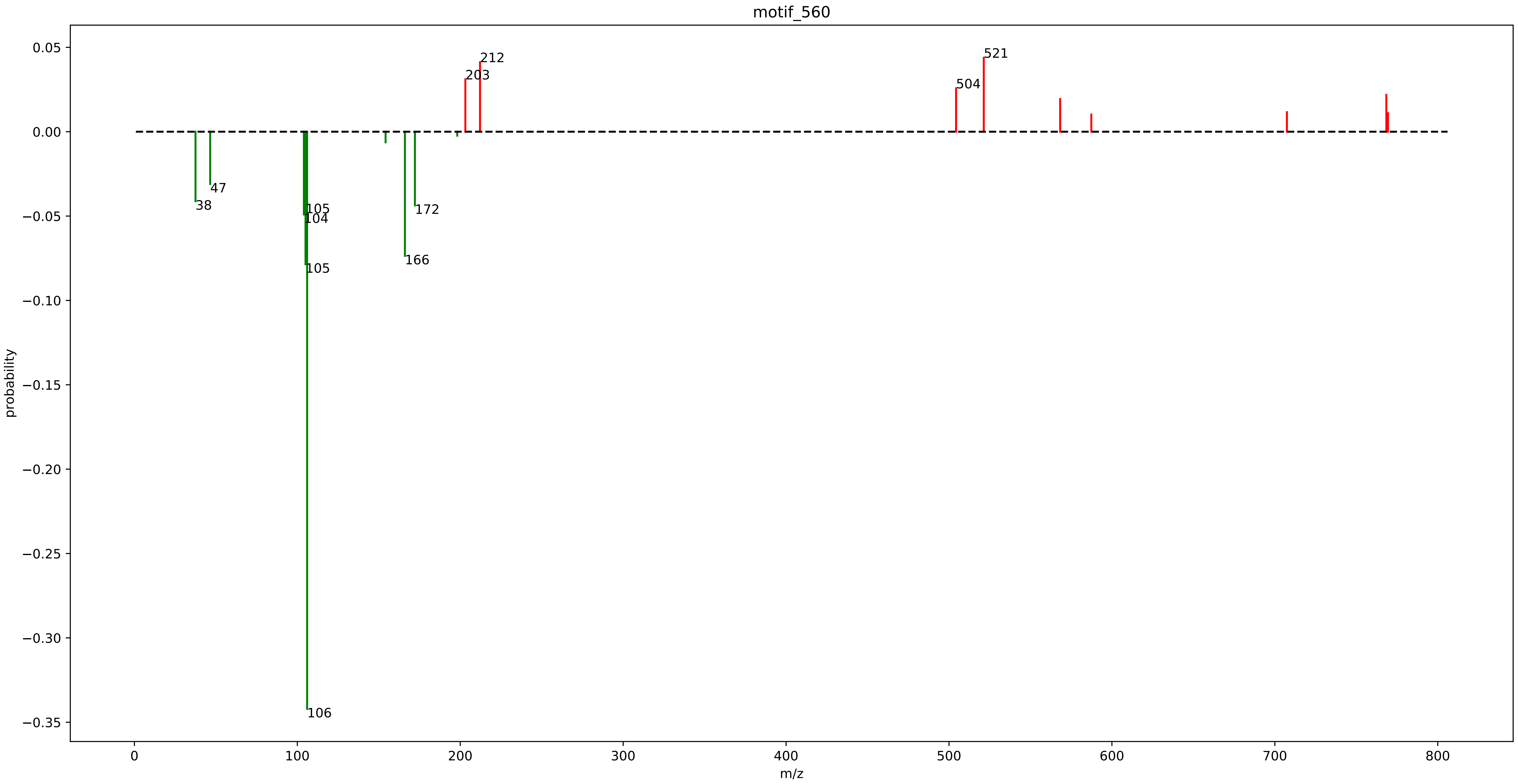

motif_560
probability
m/z
0.05
0.00
−0.05
−0.10
−0.15
−0.20
−0.25
−0.30
−0.35
0
100
200
300
400
500
600
700
800
212
203
521
504
38
47
105
104
105
166
172
106

motif_560

loss_106.0225	: 0.34
loss_105.0225	: 0.08
loss_166.0425	: 0.07
loss_104.0225	: 0.05
fragment_521.3475	: 0.04
loss_172.1825	: 0.04
loss_105.0175	: 0.04
loss_37.5075	: 0.04
fragment_212.1375	: 0.04
loss_46.5125	: 0.03
fragment_203.1325	: 0.03
fragment_504.3375	: 0.03
fragment_768.4275	: 0.02
fragment_568.2275	: 0.02
fragment_707.4025	: 0.01
fragment_769.4225	: 0.01
fragment_587.3575	: 0.01
loss_154.1725	: 0.01

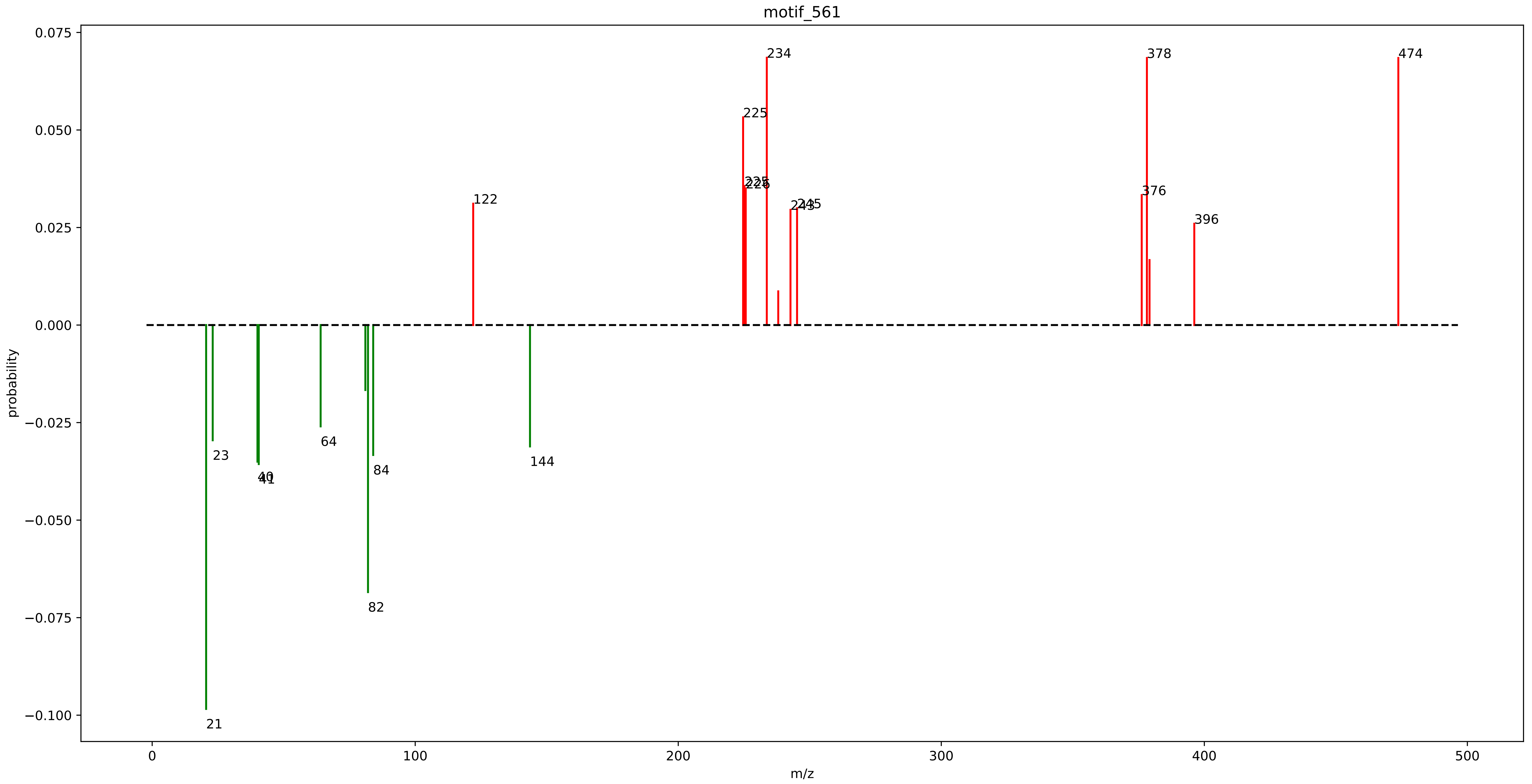
motif_561
probability
m/z
0.075
0.050
0.025
0.000
−0.025
−0.050
−0.075
−0.100
0
100
200
300
400
500
234
378
474
225
225
226
122
243
245
376
396
23
40
41
64
84
144
82
21

motif_561

loss_20.5125	: 0.10
fragment_233.6625	: 0.07
loss_82.0525	: 0.07
fragment_378.1725	: 0.07
fragment_473.7575	: 0.07
fragment_224.6525	: 0.05
fragment_225.1575	: 0.04
loss_40.5225	: 0.04
loss_40.0225	: 0.04
fragment_225.6575	: 0.04
fragment_376.1975	: 0.03
loss_84.0275	: 0.03
fragment_122.0425	: 0.03
loss_143.6375	: 0.03
fragment_245.1675	: 0.03
fragment_242.6675	: 0.03
loss_23.0125	: 0.03
fragment_396.1825	: 0.03
loss_64.0425	: 0.03
loss_81.0475	: 0.02
fragment_379.1725	: 0.02
fragment_238.0175	: 0.01

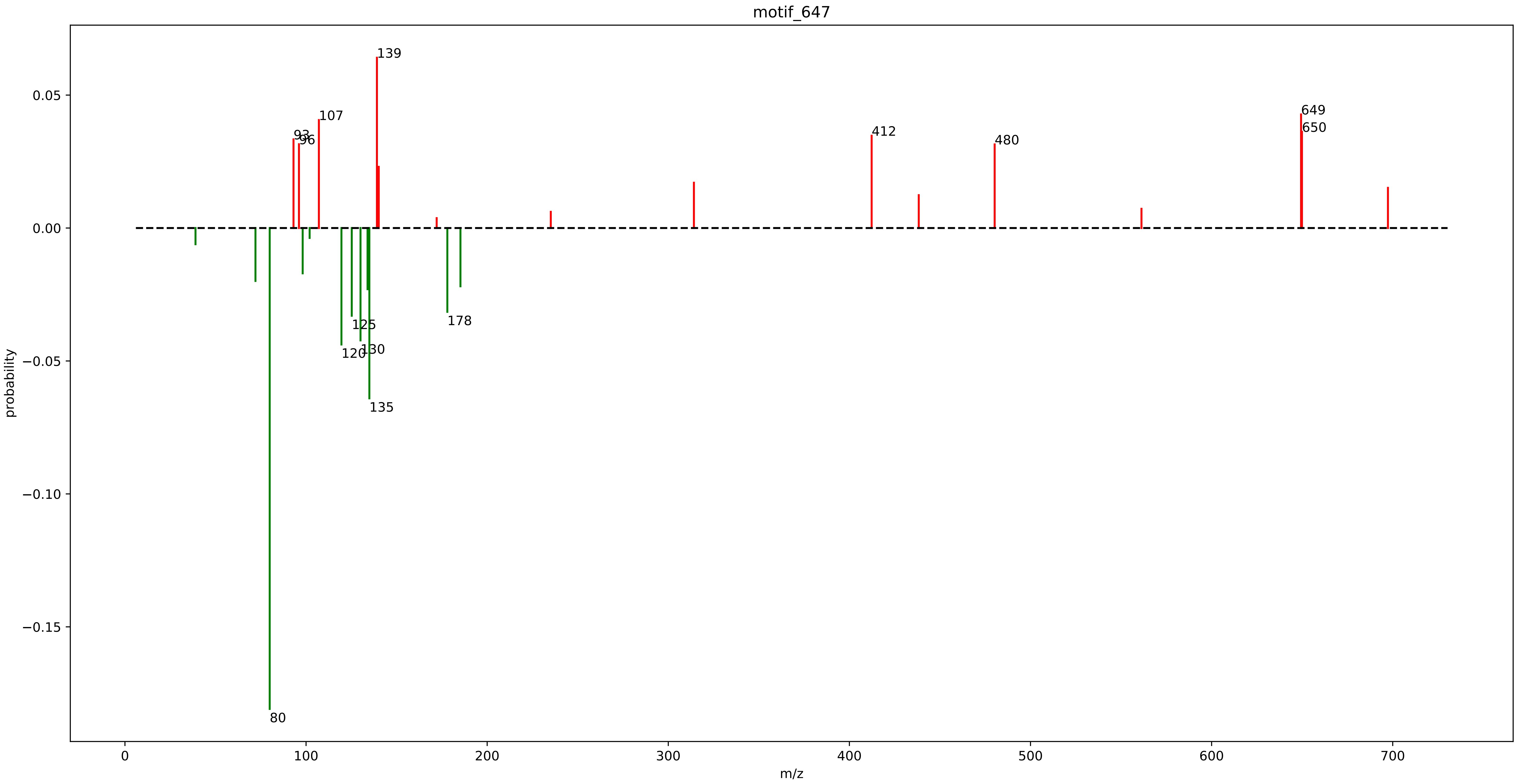

motif_647
probability
m/z
0.05
0.00
−0.05
−0.10
−0.15
0
100
200
300
400
500
600
700
139
107
93
96
412
480
649
650
125
120
130
135
178
80

motif_647

loss_79.9225	: 0.18
fragment_139.1325	: 0.06
loss_134.9525	: 0.06
loss_119.5375	: 0.04
fragment_649.3875	: 0.04
loss_130.0675	: 0.04
fragment_107.0825	: 0.04
fragment_649.8875	: 0.04
fragment_412.2875	: 0.03
fragment_93.0675	: 0.03
loss_125.2275	: 0.03
loss_178.0075	: 0.03
fragment_96.0825	: 0.03
fragment_480.2075	: 0.03
loss_133.9475	: 0.02
fragment_140.1425	: 0.02
loss_185.2475	: 0.02
loss_72.0625	: 0.02
loss_98.1325	: 0.02
fragment_314.1525	: 0.02
fragment_697.3625	: 0.02
fragment_438.2975	: 0.01
fragment_561.2725	: 0.01
loss_38.9575	: 0.01
fragment_235.1275	: 0.01

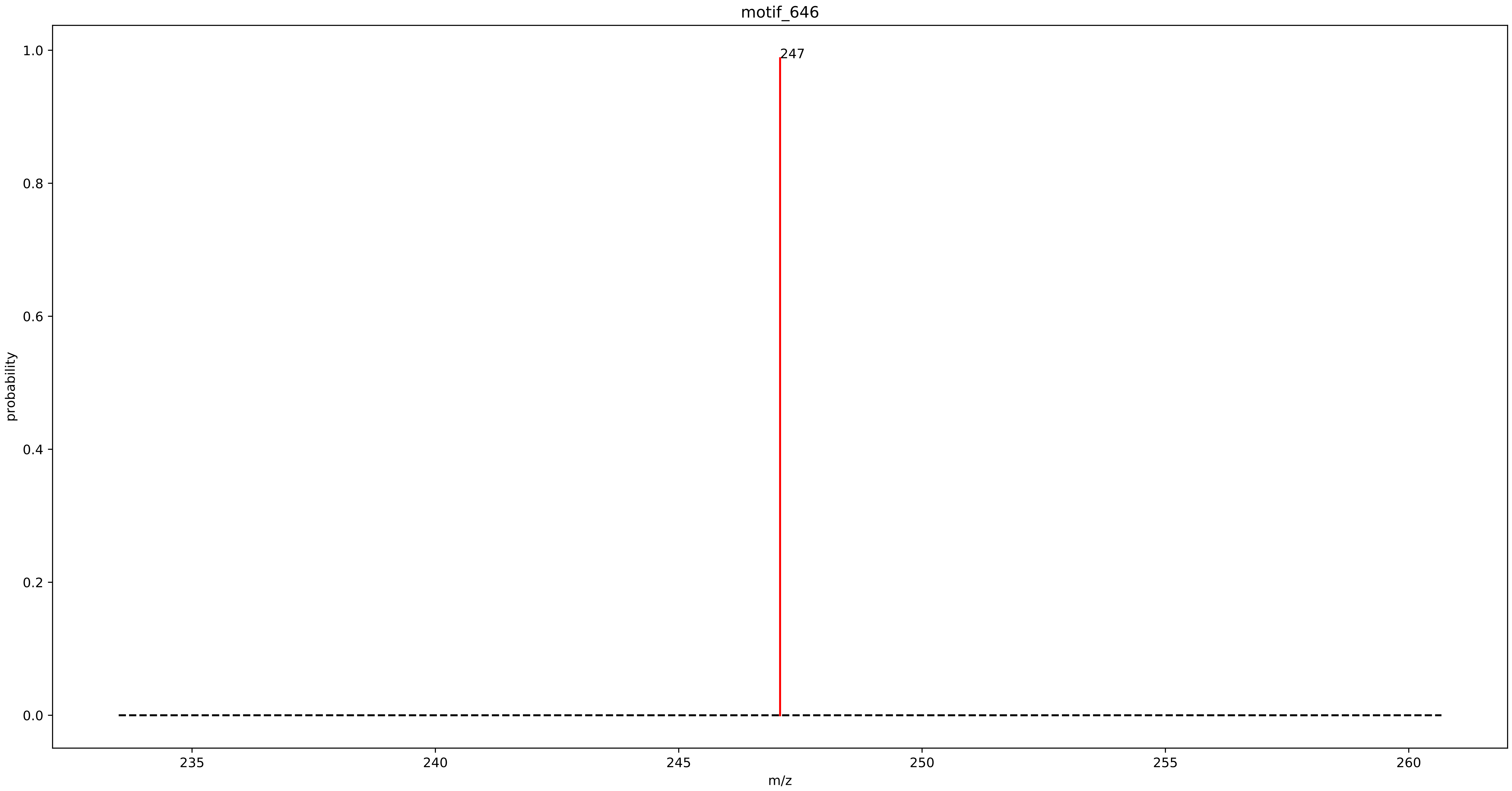

motif_646
247
probability
m/z
1.0
0.8
0.6
0.4
0.2
0.0
235
240
245
250
255
260

motif_646

fragment_247.0825 : 0.99

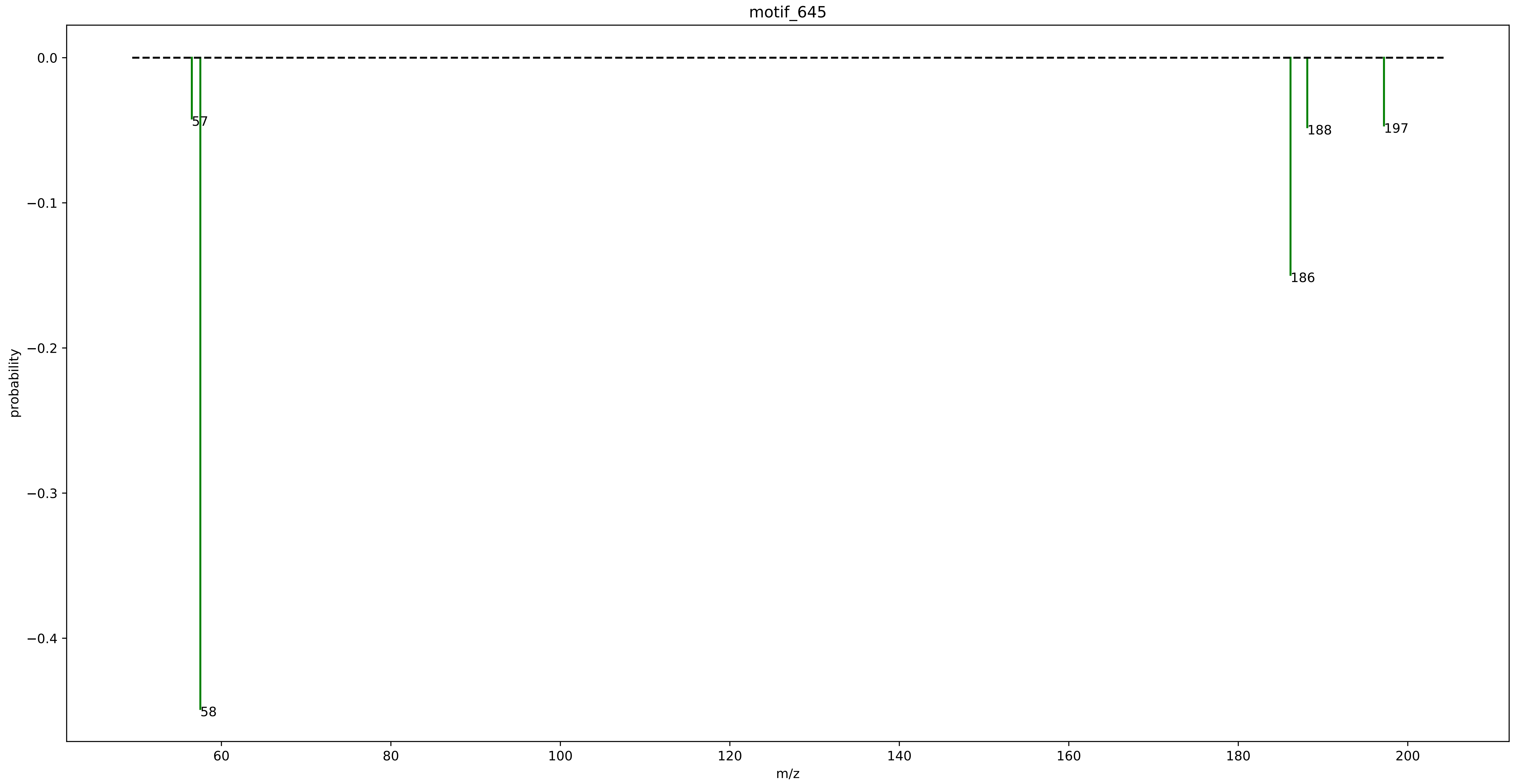

motif_645
probability
m/z
0.0
−0.1
−0.2
−0.3
−0.4
60
80
100
120
140
160
180
200
57
58
186
188
197

motif_645

loss_57.5125	: 0.45
loss_186.1625	: 0.15
loss_188.1425	: 0.05
loss_197.1925	: 0.05
loss_56.5075	: 0.04

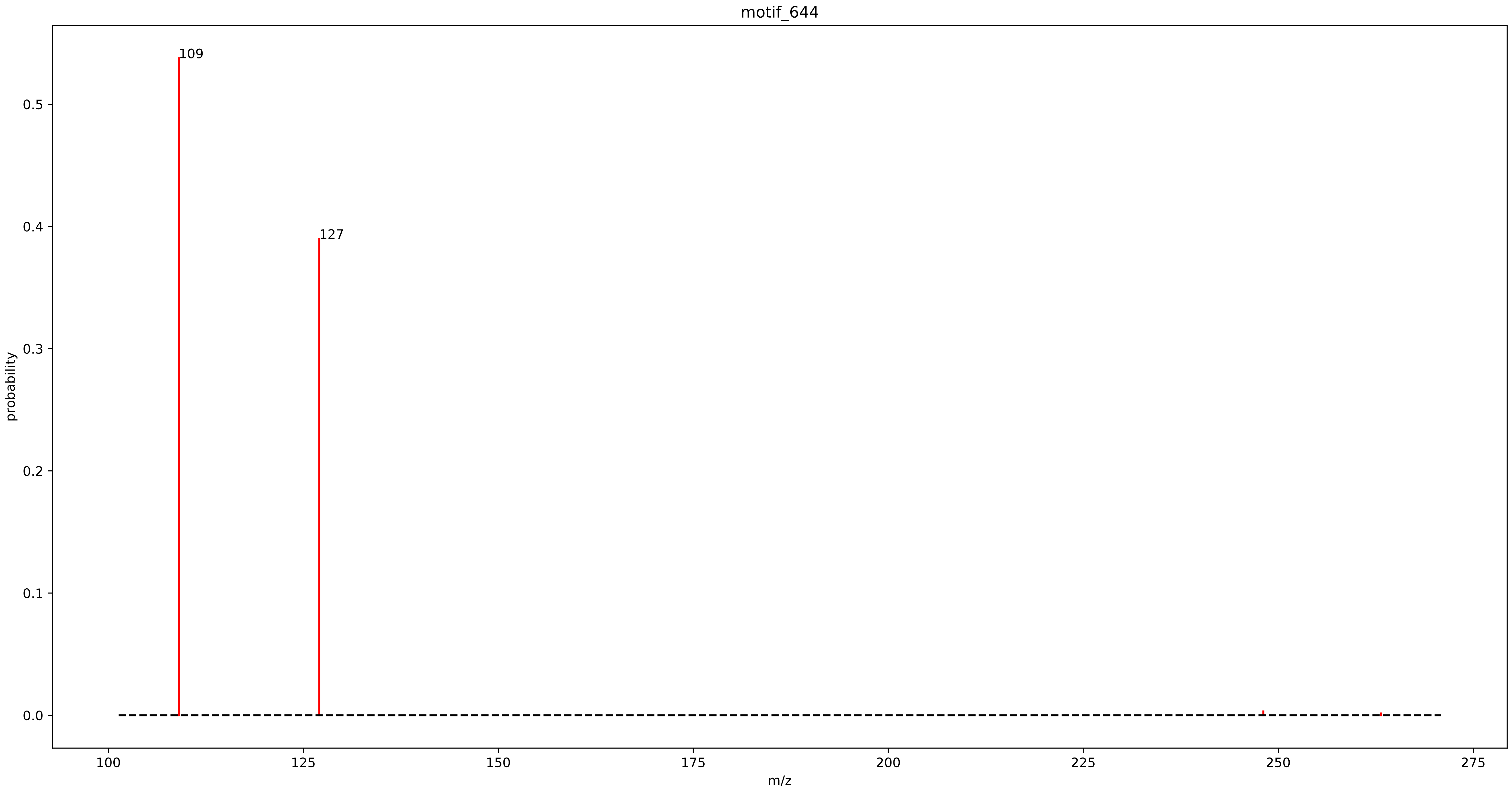
motif_644
probability
m/z
109
127
0.0
0.1
0.2
0.3
0.4
0.5
100
125
150
175
200
225
250
275

motif_644

fragment_109.0275	: 0.54
fragment_127.0375	: 0.39

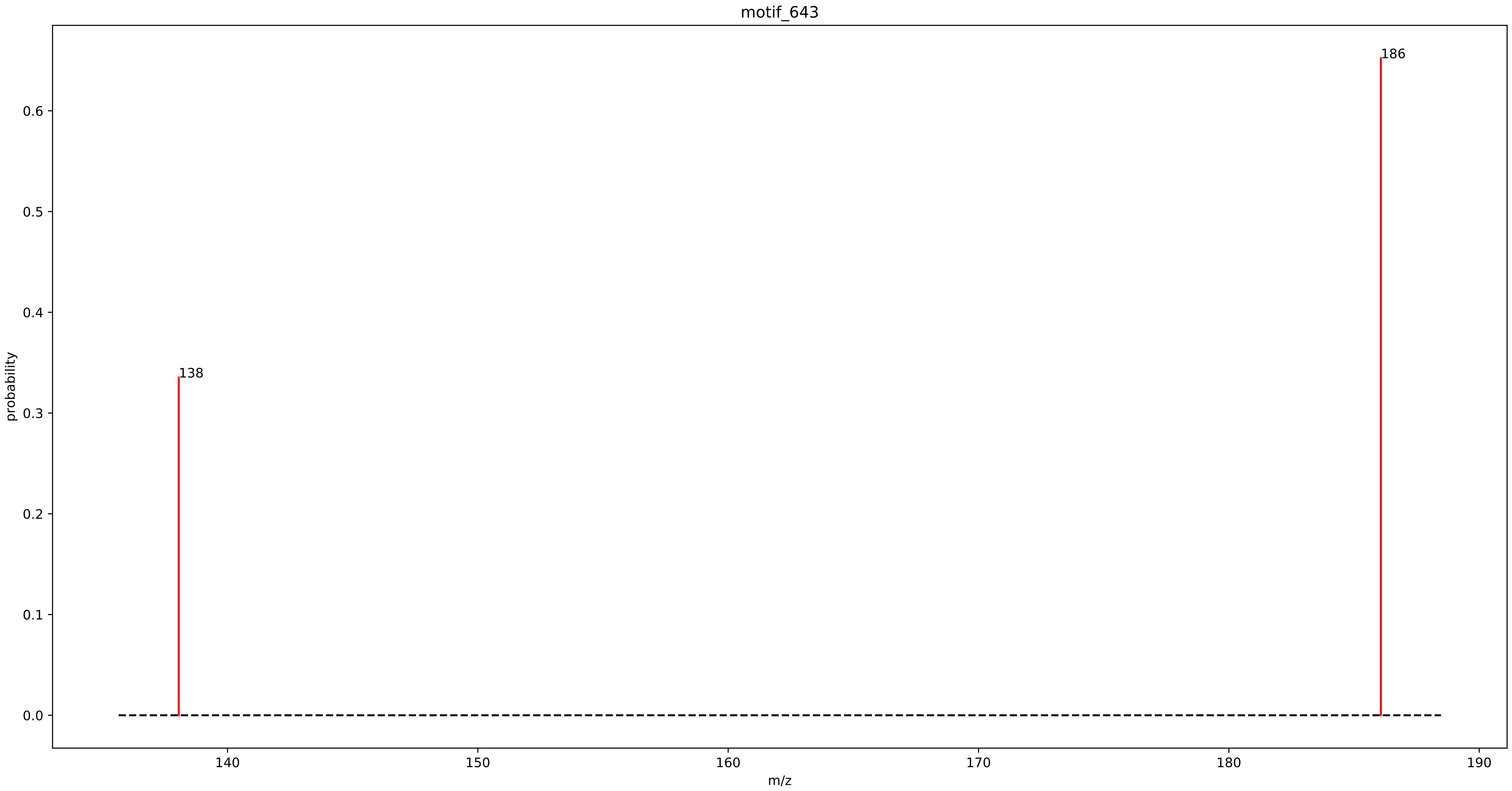

motif_643
186
138
0.6
0.5
0.4
0.3
0.2
0.1
0.0
probability
140
150
160
170
180
190
m/z

motif_643

fragment_186.0725	: 0.65
fragment_138.0525	: 0.34

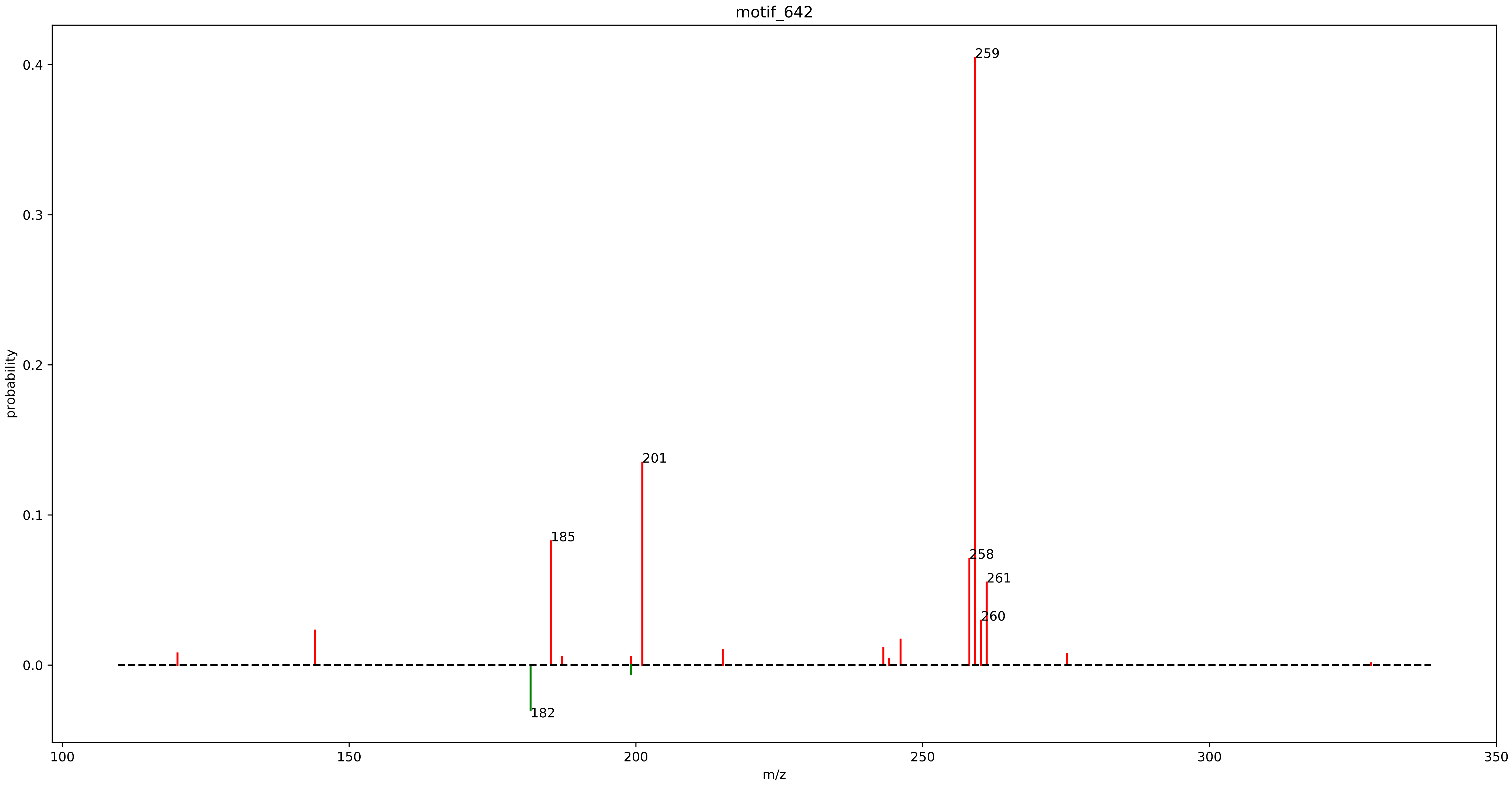
motif_642
probability
m/z
0.4
0.3
0.2
0.1
0.0
100
150
200
250
300
350
259
201
185
258
261
260
182

motif_642

fragment_259.1275	: 0.40
fragment_201.1225	: 0.13
fragment_185.1625	: 0.08
fragment_258.1425	: 0.07
fragment_261.1425	: 0.06
loss_181.6375	: 0.03
fragment_260.1575	: 0.03
fragment_144.0675	: 0.02
fragment_246.1425	: 0.02
fragment_243.1375	: 0.01
fragment_215.1375	: 0.01
fragment_120.0775	: 0.01
fragment_275.1575	: 0.01
loss_199.1675	: 0.01
fragment_199.1625	: 0.01
fragment_187.1425	: 0.01
fragment_275.1625	: 0.01
fragment_260.1425	: 0.01

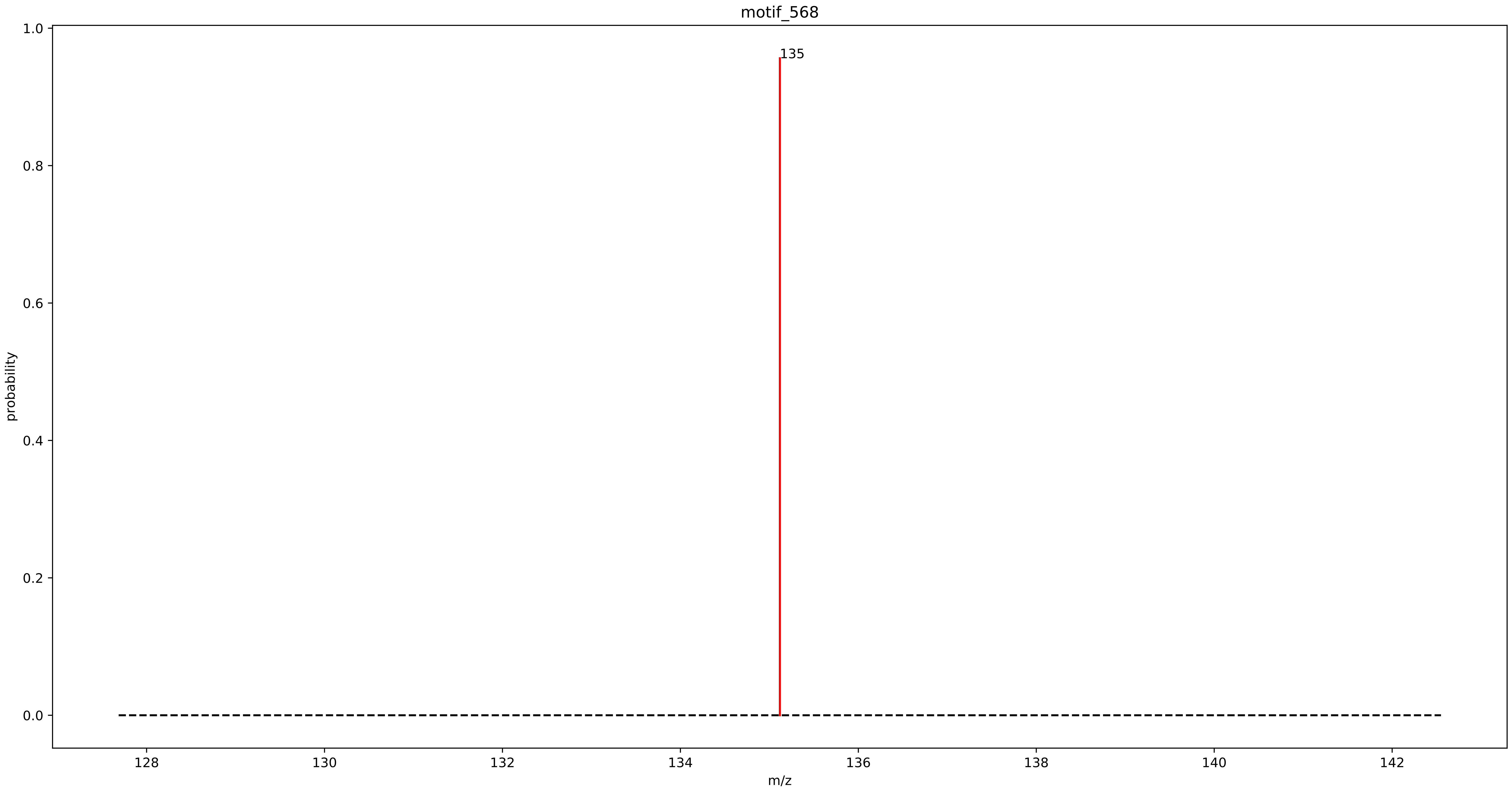
motif_568
135
probability
m/z
1.0
0.8
0.6
0.4
0.2
0.0
128
130
132
134
136
138
140
142

motif_568

fragment_135.1175 : 0.96

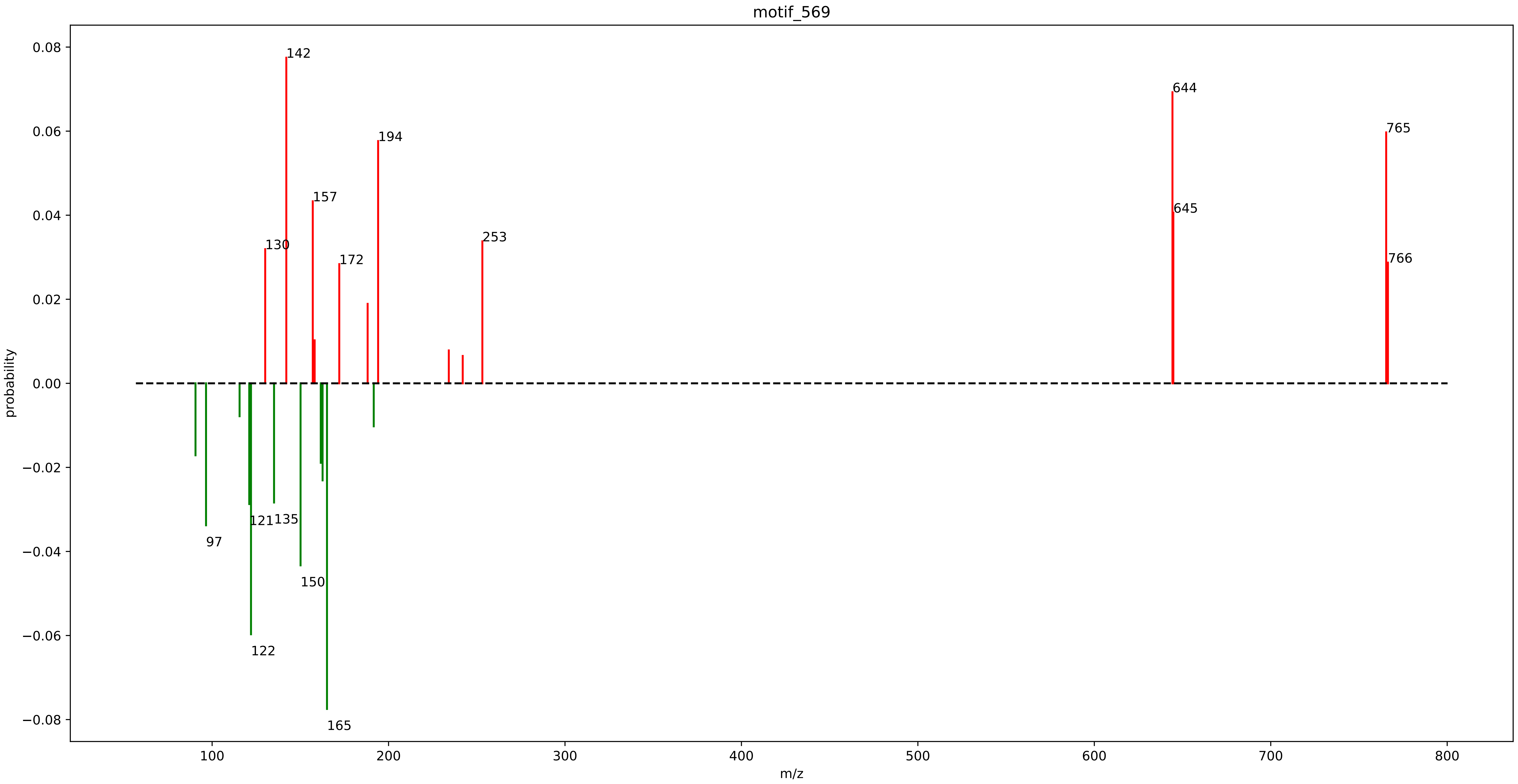

motif_569
probability
m/z
0.08
0.06
0.04
0.02
0.00
−0.02
−0.04
−0.06
−0.08
100
200
300
400
500
600
700
800
142
644
765
194
157
645
253
130
172
766
97
121
135
150
122
165

motif_569

loss_165.1075	: 0.08
fragment_142.0525	: 0.08
fragment_644.2925	: 0.07
loss_122.0375	: 0.06
fragment_765.4225	: 0.06
fragment_194.0825	: 0.06
loss_150.1225	: 0.04
fragment_157.0425	: 0.04
fragment_644.7975	: 0.04
fragment_253.1625	: 0.03
loss_96.5475	: 0.03
fragment_130.0875	: 0.03
loss_121.0375	: 0.03
fragment_766.4225	: 0.03
loss_135.0975	: 0.03
fragment_172.0675	: 0.03
loss_162.6075	: 0.02
loss_161.5725	: 0.02
fragment_188.1425	: 0.02
loss_90.5825	: 0.02
loss_191.5975	: 0.01
fragment_158.1175	: 0.01
fragment_234.1475	: 0.01
loss_115.5625	: 0.01
fragment_242.0325	: 0.01

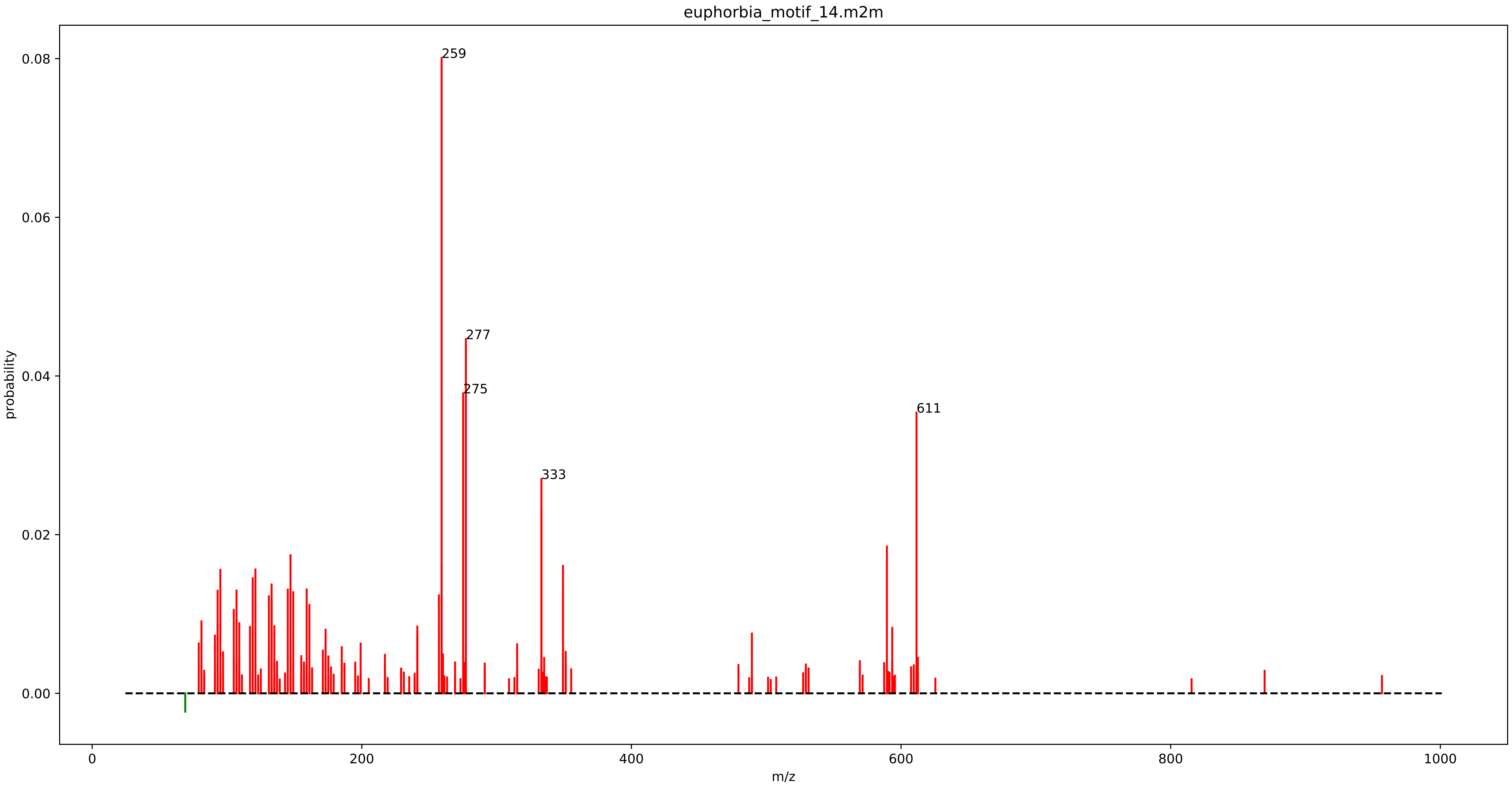

euphorbia_motif_14.m2m
probability
m/z
0.08
0.06
0.04
0.02
0.00
0
200
400
600
800
1000
259
277
275
333
611

euphorbia_motif_14.m2m

fragment_259.2025	: 0.08
fragment_277.2125	: 0.04
fragment_275.1975	: 0.04
fragment_611.4625	: 0.04
fragment_333.2375	: 0.03
fragment_333.2425	: 0.02
fragment_589.4775	: 0.02
fragment_147.1175	: 0.02
fragment_259.2075	: 0.02
fragment_349.2325	: 0.02
fragment_121.0975	: 0.02
fragment_95.0825	: 0.02
fragment_119.0825	: 0.01
fragment_133.0975	: 0.01
fragment_159.1175	: 0.01
fragment_145.0975	: 0.01
fragment_349.2375	: 0.01
fragment_107.0825	: 0.01
fragment_93.0675	: 0.01
fragment_149.1325	: 0.01
fragment_257.1875	: 0.01
fragment_131.0825	: 0.01
fragment_121.1025	: 0.01
fragment_133.1025	: 0.01
fragment_161.1325	: 0.01
fragment_105.0675	: 0.01
fragment_81.0675	: 0.01
fragment_109.0975	: 0.01
fragment_93.0725	: 0.01
fragment_135.1175	: 0.01
fragment_241.1925	: 0.01
fragment_117.0675	: 0.01
fragment_593.4525	: 0.01
fragment_173.1325	: 0.01
fragment_257.1925	: 0.01
fragment_489.3525	: 0.01
fragment_241.1975	: 0.01
fragment_91.0525	: 0.01
fragment_147.1125	: 0.01
fragment_79.0525	: 0.01
fragment_199.1475	: 0.01
fragment_315.2275	: 0.01
fragment_277.1775	: 0.01
fragment_185.1325	: 0.01
fragment_171.1125	: 0.01
fragment_351.2525	: 0.01
fragment_97.0625	: 0.01

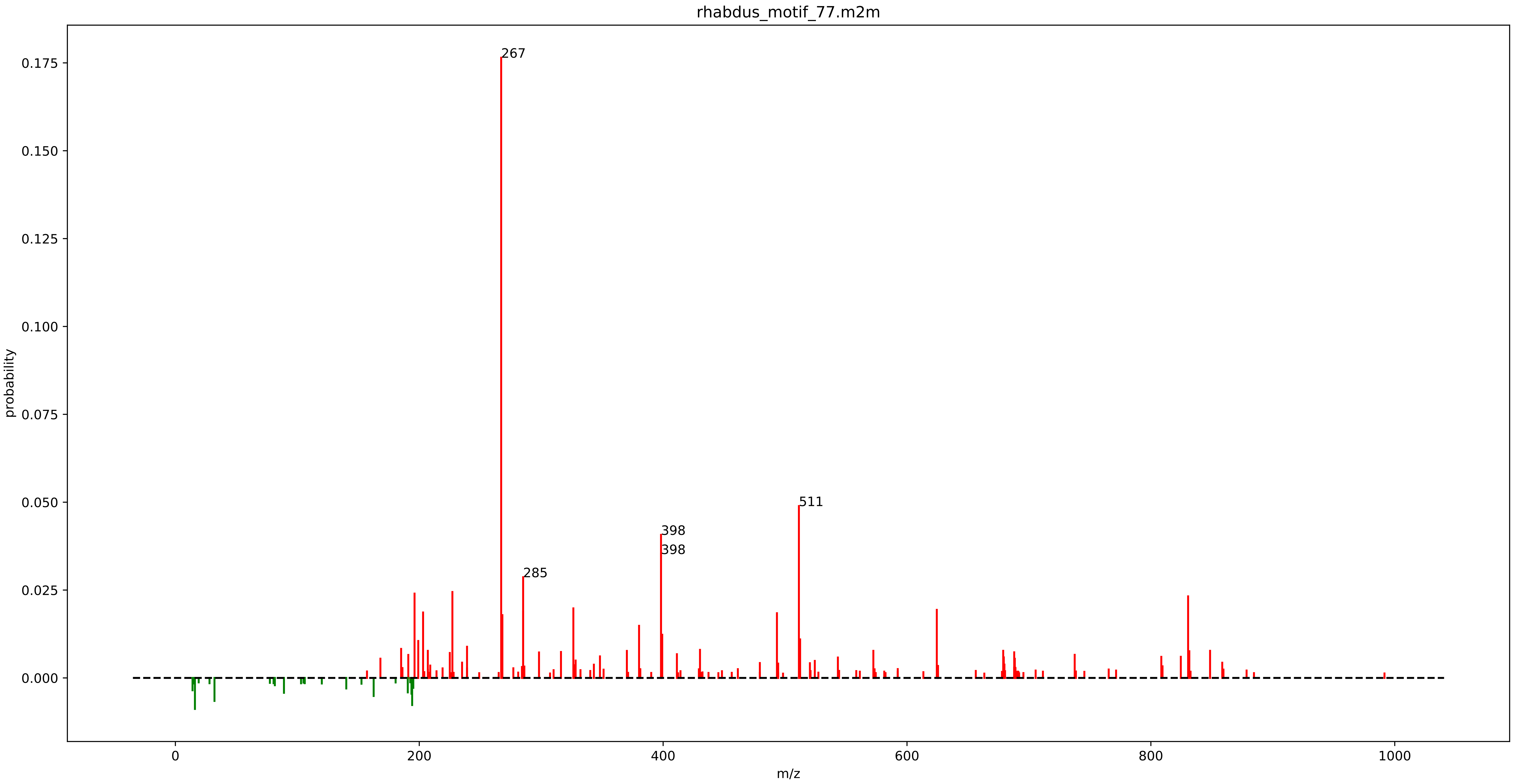
rhabdus_motif_77.m2m
probability
m/z
0.175
0.150
0.125
0.100
0.075
0.050
0.025
0.000
0
200
400
600
800
1000
267
285
398
398
511

rhabdus_motif_77.m2m

fragment_267.1725	: 0.18
fragment_511.3475	: 0.05
fragment_398.2625	: 0.04
fragment_398.2675	: 0.04
fragment_285.1825	: 0.03
fragment_227.1775	: 0.02
fragment_196.1325	: 0.02
fragment_830.5325	: 0.02
fragment_326.3775	: 0.02
fragment_624.4325	: 0.02
fragment_285.1775	: 0.02
fragment_203.1375	: 0.02
fragment_493.3375	: 0.02
fragment_268.1725	: 0.02
fragment_380.2575	: 0.01
fragment_399.2675	: 0.01
fragment_512.3525	: 0.01
fragment_199.1775	: 0.01
fragment_239.1775	: 0.01
loss_16.0325	: 0.01
fragment_185.1275	: 0.01
fragment_430.2325	: 0.01
fragment_848.5425	: 0.01
fragment_678.8925	: 0.01
loss_194.1775	: 0.01
fragment_207.0325	: 0.01
fragment_572.4025	: 0.01
fragment_370.2675	: 0.01
fragment_831.5375	: 0.01
fragment_316.2225	: 0.01
fragment_687.9125	: 0.01
fragment_298.2125	: 0.01
fragment_225.0475	: 0.01
fragment_411.2975	: 0.01
fragment_737.5175	: 0.01
loss_32.0625	: 0.01
fragment_191.0025	: 0.01
fragment_348.1925	: 0.01
fragment_824.5475	: 0.01
fragment_808.5525	: 0.01
fragment_679.3975	: 0.01
fragment_543.3175	: 0.01
fragment_168.1375	: 0.01
fragment_688.4175	: 0.01
loss_162.6025	: 0.01

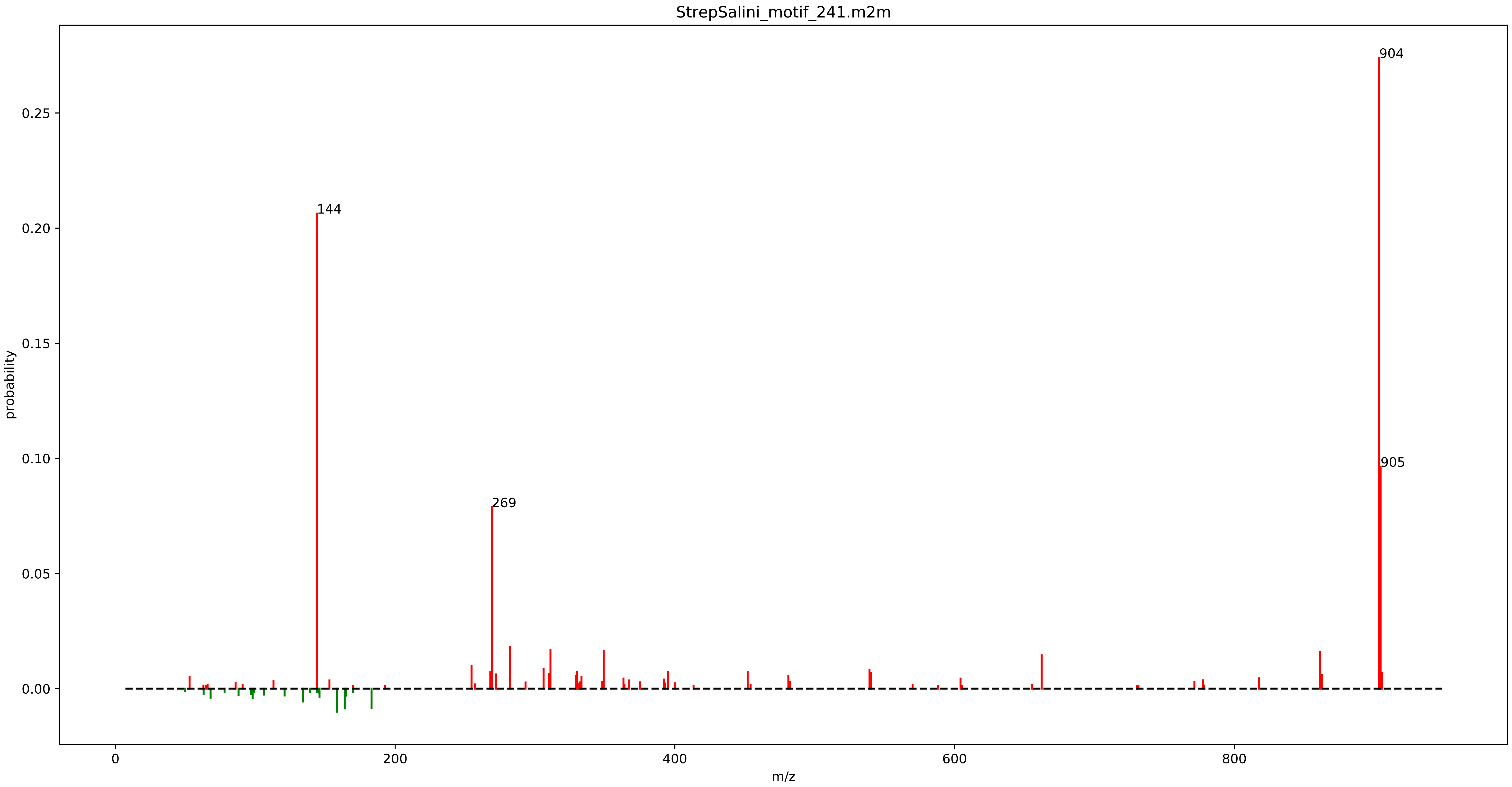

StrepSalini_motif_241.m2m
probability
m/z
0.25
0.20
0.15
0.10
0.05
0.00
0
200
400
600
800
904
144
269
905

StrepSalini_motif_241.m2m

fragment_903.5500	: 0.27
fragment_144.0500	: 0.21
fragment_904.5500	: 0.10
fragment_269.0500	: 0.08
fragment_282.0500	: 0.02
fragment_311.0500	: 0.02
fragment_349.1500	: 0.02
fragment_861.4525	: 0.02
fragment_662.2500	: 0.01
loss_158.5500	: 0.01
fragment_254.6500	: 0.01
fragment_306.1500	: 0.01
loss_163.9500	: 0.01
loss_183.1525	: 0.01
fragment_539.1500	: 0.01
fragment_452.0500	: 0.01
fragment_330.0500	: 0.01
fragment_268.0500	: 0.01
fragment_395.2500	: 0.01
fragment_540.1500	: 0.01
fragment_905.5500	: 0.01
fragment_310.0500	: 0.01
fragment_272.0500	: 0.01
fragment_862.5500	: 0.01
loss_134.0500	: 0.01
fragment_481.1500	: 0.01
fragment_329.2500	: 0.01
fragment_333.2500	: 0.01
fragment_53.0500	: 0.01

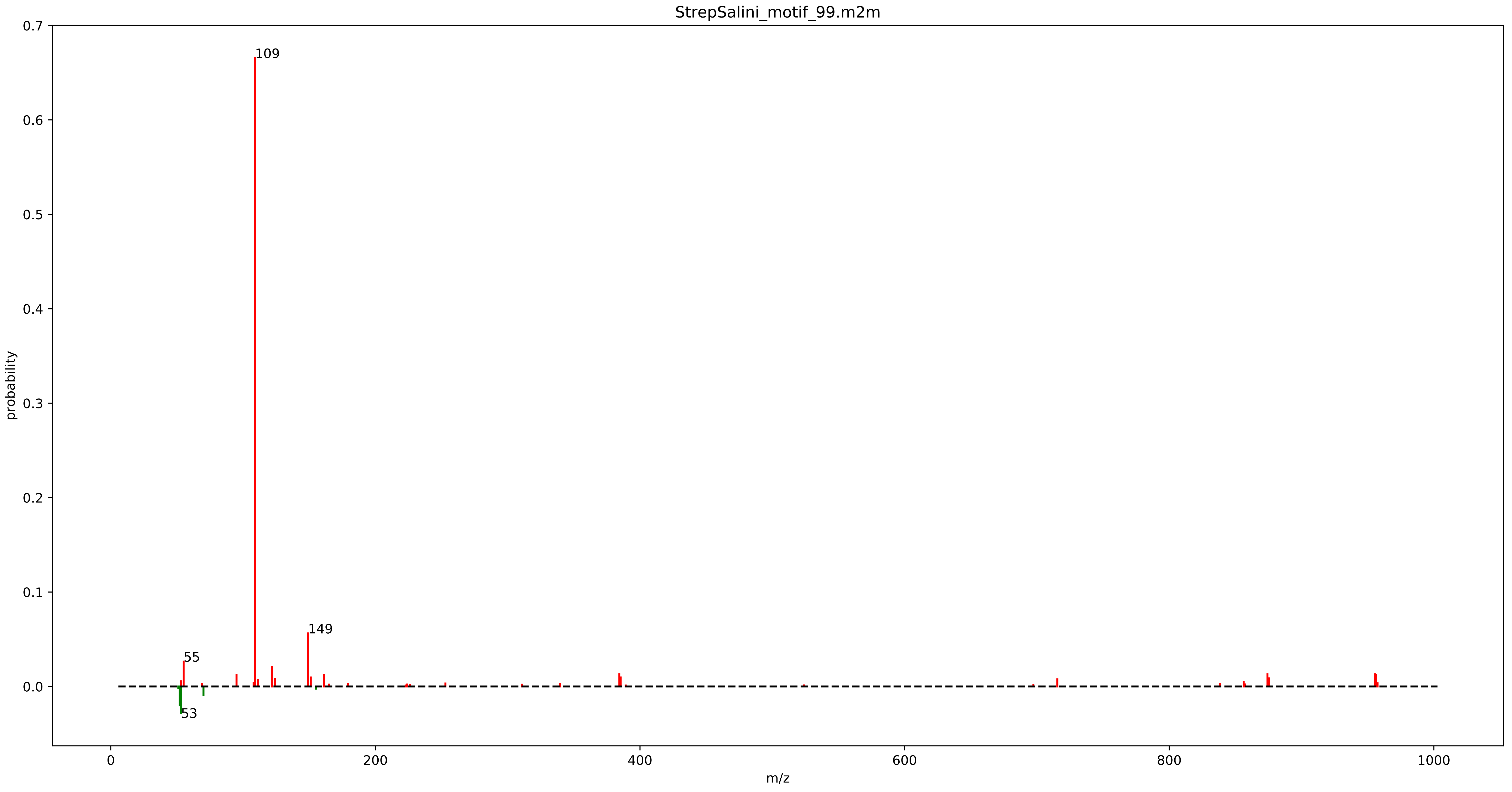

StrepSalini_motif_99.m2m
probability
m/z
109
149
55
53
0.7
0.6
0.5
0.4
0.3
0.2
0.1
0.0
0
200
400
600
800
1000

StrepSalini_motif_99.m2m

fragment_109.0525	: 0.67
fragment_149.1500	: 0.06
loss_53.0500	: 0.03
fragment_55.0500	: 0.03
fragment_122.0500	: 0.02
loss_52.0500	: 0.02
fragment_955.3500	: 0.01
fragment_384.3500	: 0.01
fragment_874.2500	: 0.01
fragment_95.0500	: 0.01
fragment_161.1500	: 0.01
fragment_956.3500	: 0.01
fragment_385.3525	: 0.01
fragment_151.1500	: 0.01
loss_70.0475	: 0.01
fragment_875.2500	: 0.01
fragment_124.1500	: 0.01
fragment_715.4500	: 0.01
fragment_111.1500	: 0.01
fragment_53.0500	: 0.01

gnps_motif_43.m2m

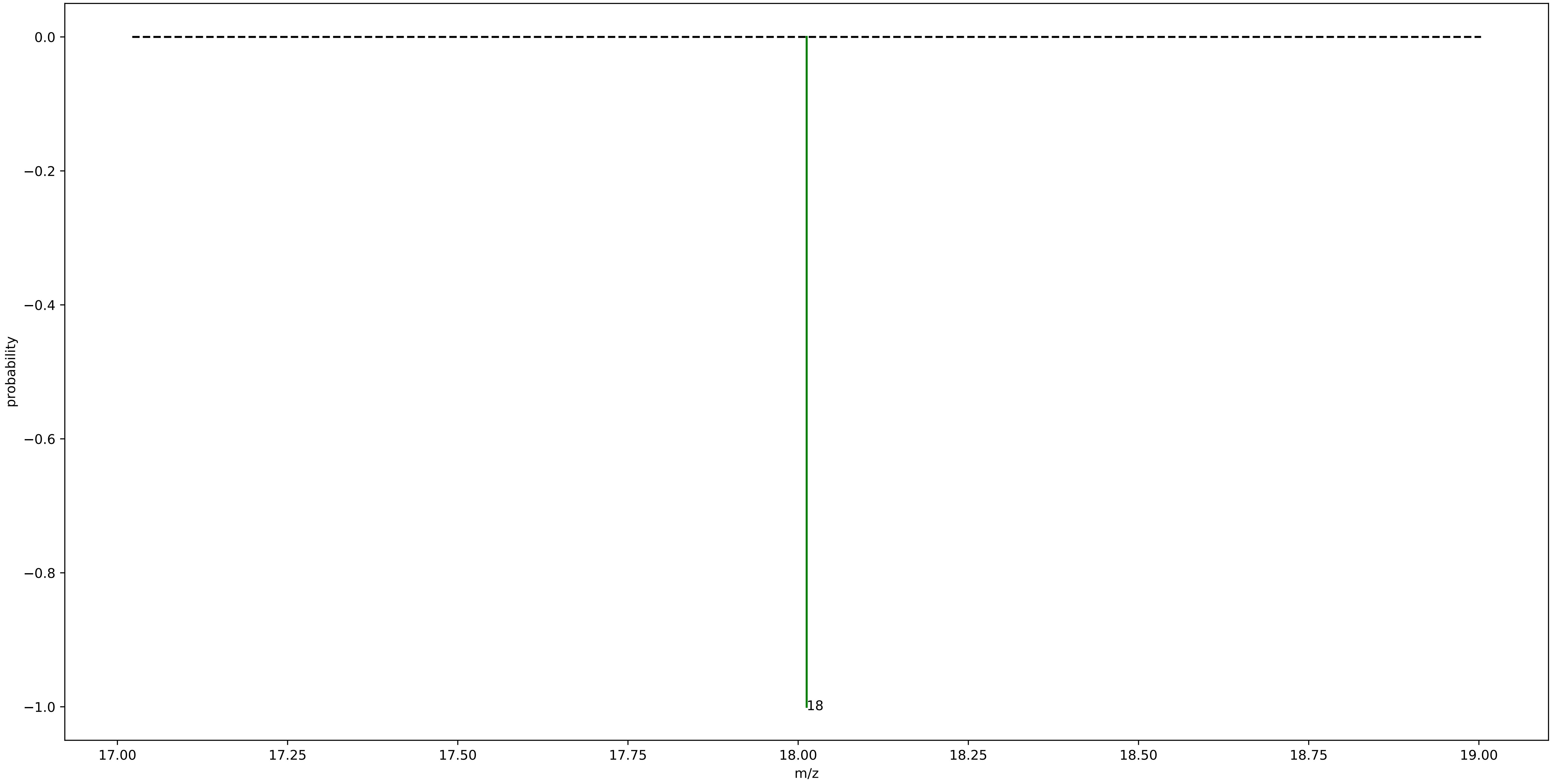

gnps_motif_43.m2m

loss_18.0125 : 1.00

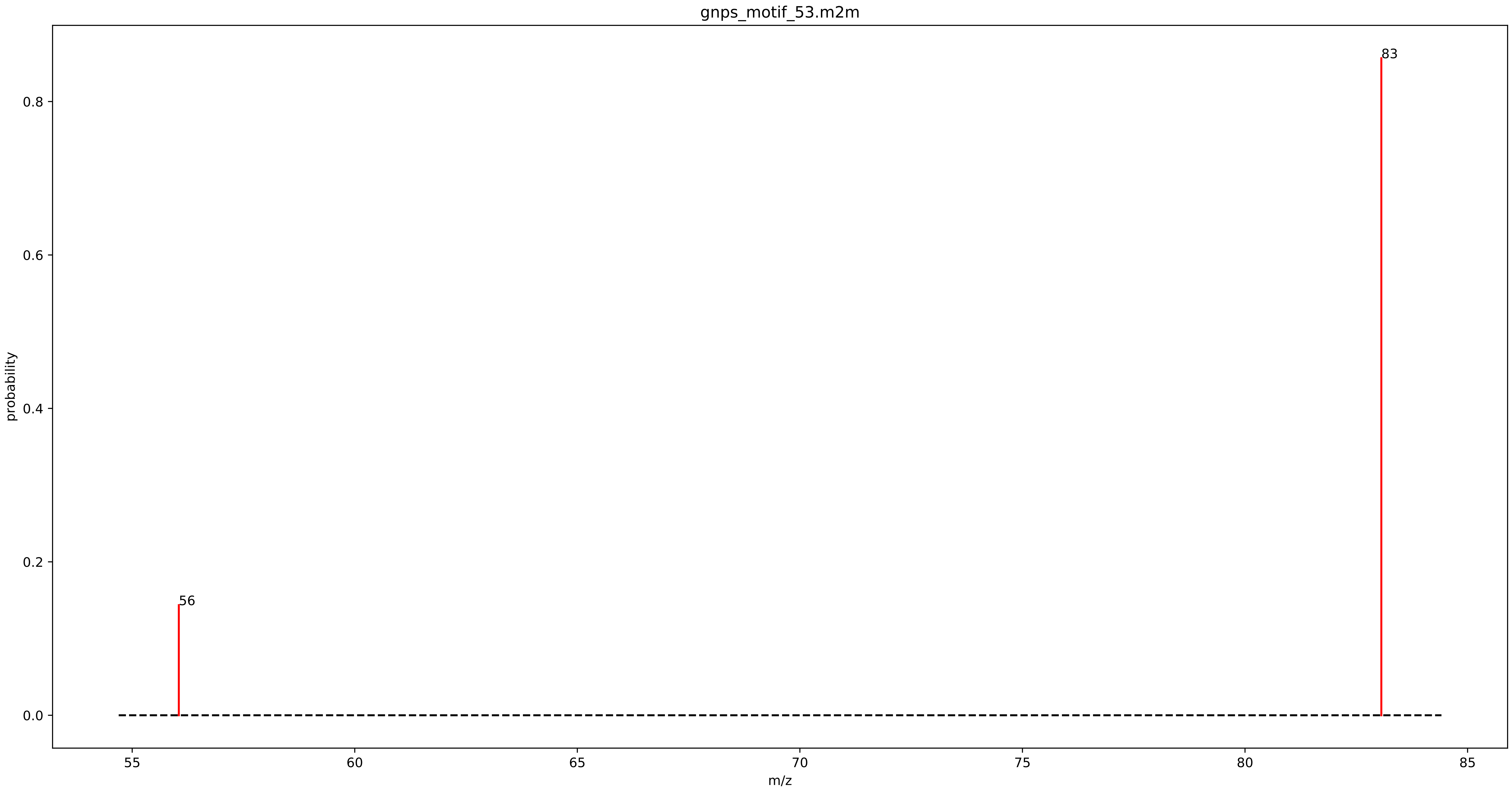

gnps_motif_53.m2m
probability
m/z
83
56
0.0
0.2
0.4
0.6
0.8
55
60
65
70
75
80
85

gnps_motif_53.m2m

fragment_83.0625	: 0.86
fragment_56.0475	: 0.14

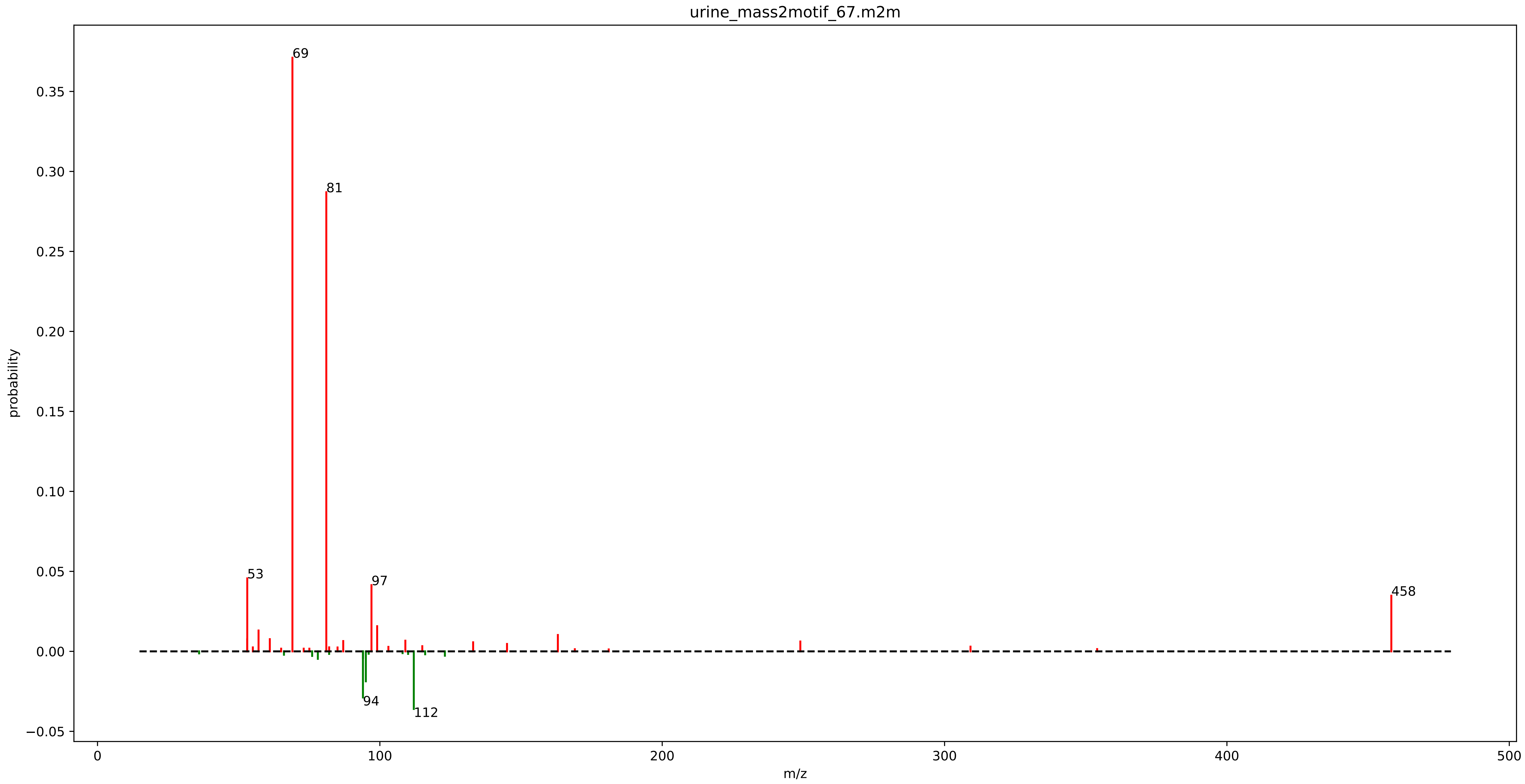
urine_mass2motif_67.m2m
probability
m/z
69
81
53
97
458
94
112
0.35
0.30
0.25
0.20
0.15
0.10
0.05
0.00
−0.05
0
100
200
300
400
500

urine_mass2motif_67.m2m

fragment_69.0325	: 0.37
fragment_81.0325	: 0.29
fragment_53.0375	: 0.05
fragment_97.0275	: 0.04
loss_112.0175	: 0.04
fragment_458.2275	: 0.03
loss_94.0075	: 0.03
loss_95.0375	: 0.02
fragment_99.0425	: 0.02
fragment_57.0325	: 0.01
fragment_163.0425	: 0.01
fragment_61.0275	: 0.01
fragment_53.0025	: 0.01
fragment_109.0275	: 0.01
fragment_87.0275	: 0.01
fragment_248.9175	: 0.01
fragment_133.0325	: 0.01

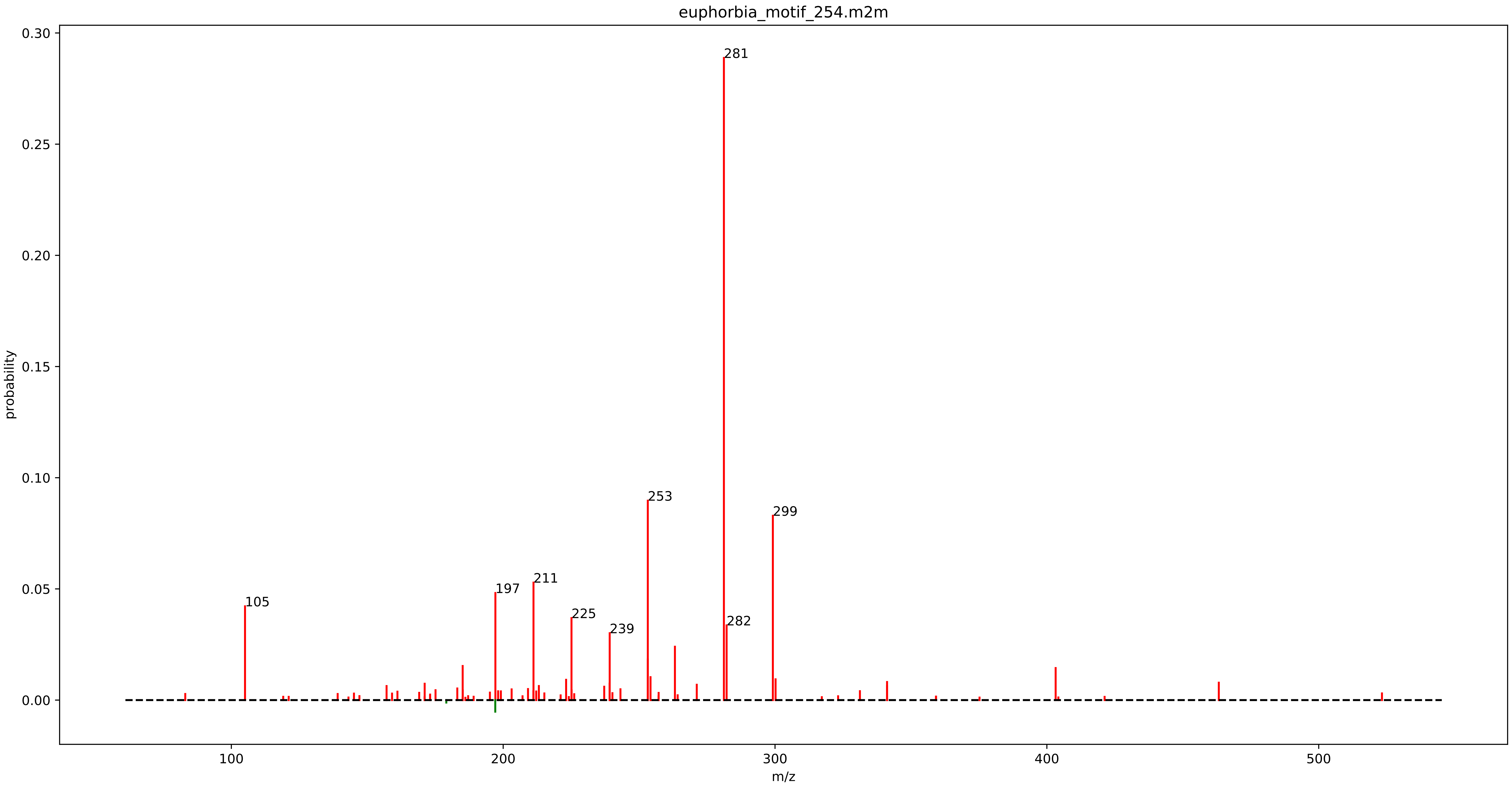

euphorbia_motif_254.m2m
probability
m/z
0.30
0.25
0.20
0.15
0.10
0.05
0.00
100
200
300
400
500
281
253
299
211
197
105
225
282
239

euphorbia_motif_254.m2m

fragment_281.1925	: 0.29
fragment_253.1975	: 0.09
fragment_299.2025	: 0.08
fragment_211.1475	: 0.05
fragment_197.1325	: 0.05
fragment_105.0325	: 0.04
fragment_225.1275	: 0.04
fragment_282.1925	: 0.03
fragment_239.1775	: 0.03
fragment_263.1775	: 0.02
fragment_253.1925	: 0.02
fragment_185.0975	: 0.02
fragment_403.2275	: 0.01
fragment_254.1975	: 0.01
fragment_300.2025	: 0.01
fragment_223.1475	: 0.01
fragment_341.2125	: 0.01
fragment_463.2475	: 0.01
fragment_239.1425	: 0.01
fragment_171.1175	: 0.01
fragment_271.2075	: 0.01
fragment_213.1275	: 0.01
fragment_157.1025	: 0.01
fragment_341.2075	: 0.01
fragment_237.1625	: 0.01
fragment_183.1175	: 0.01
loss_197.0875	: 0.01

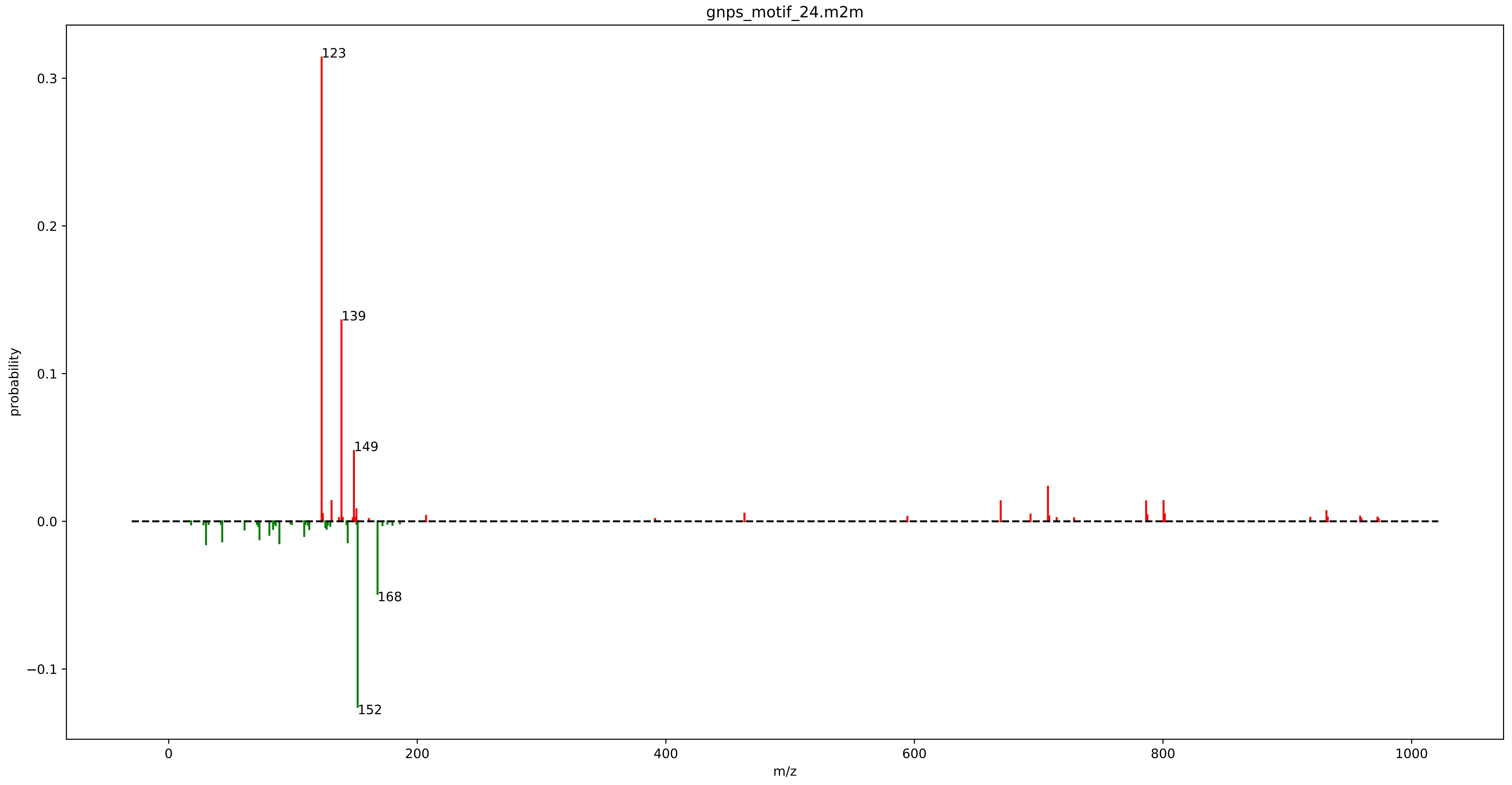

gnps_motif_24.m2m
probability
m/z
0.3
0.2
0.1
0.0
−0.1
0
200
400
600
800
1000
123
139
149
168
152

gnps_motif_24.m2m

fragment_123.0425	: 0.31
fragment_139.0375	: 0.14
loss_152.0475	: 0.13
loss_168.0425	: 0.05
fragment_149.0575	: 0.05
fragment_707.4275	: 0.02
loss_30.0475	: 0.02
loss_89.0475	: 0.01
loss_144.0425	: 0.01
fragment_131.0475	: 0.01
fragment_800.4525	: 0.01
fragment_786.4275	: 0.01
fragment_669.4175	: 0.01
loss_43.0725	: 0.01
loss_73.0525	: 0.01
loss_109.0375	: 0.01
loss_81.0375	: 0.01
fragment_151.0625	: 0.01
fragment_931.5025	: 0.01
loss_61.0275	: 0.01
loss_113.1325	: 0.01
fragment_463.2475	: 0.01
loss_84.0225	: 0.01
loss_127.1675	: 0.01

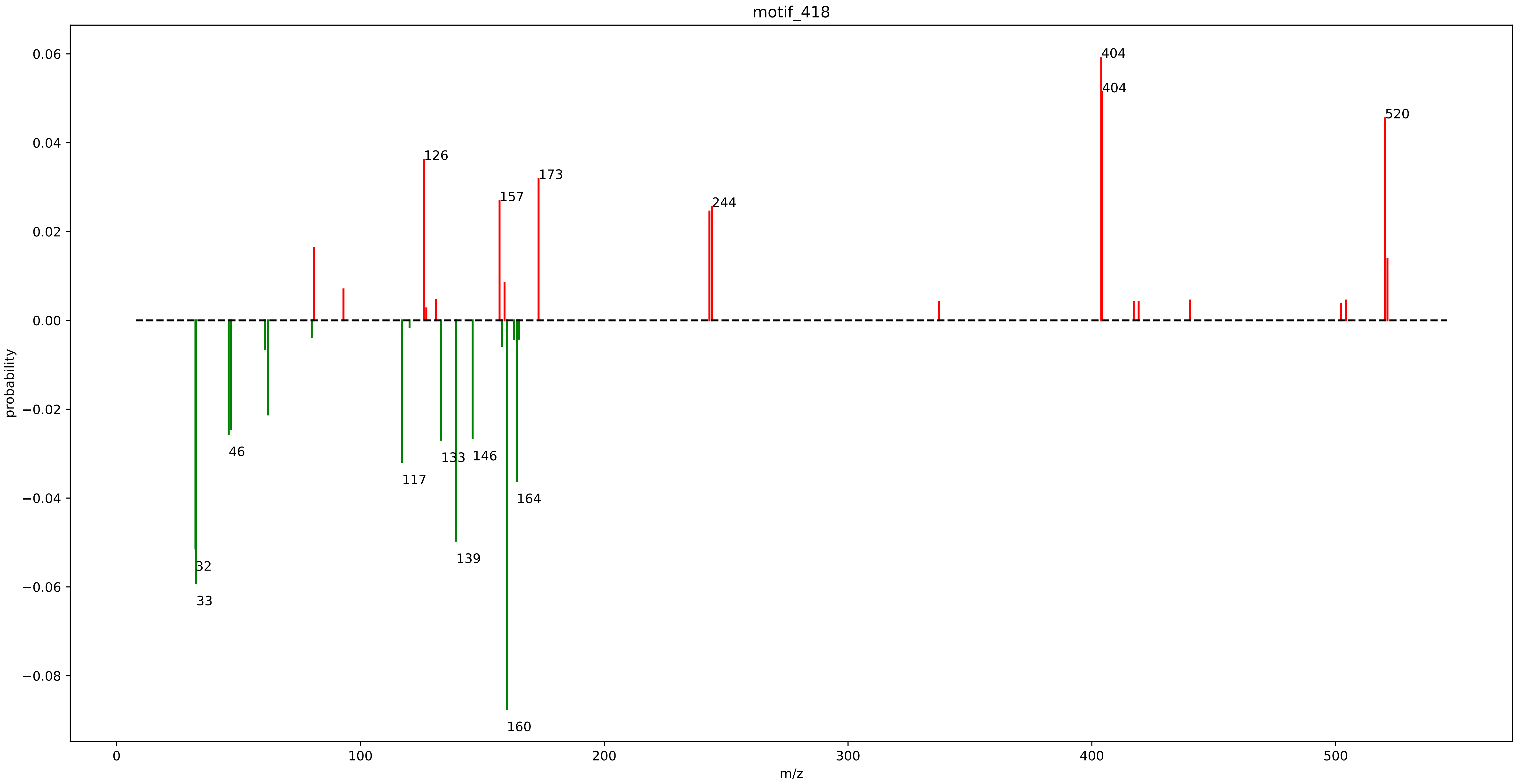

motif_418
0.06
0.04
0.02
0.00
−0.02
−0.04
−0.06
−0.08
probability
0
100
200
300
400
500
m/z
404
404
520
126
173
157
244
46
117
133
146
164
139
32
33
160

motif_418

loss_160.0775	: 0.09
loss_32.6925	: 0.06
fragment_403.8475	: 0.06
loss_32.3525	: 0.05
fragment_404.1825	: 0.05
loss_139.3175	: 0.05
fragment_520.2425	: 0.05
fragment_126.0225	: 0.04
loss_164.1175	: 0.04
fragment_173.0725	: 0.03
loss_117.0725	: 0.03
fragment_157.0775	: 0.03
loss_133.0625	: 0.03
loss_146.0375	: 0.03
fragment_244.1425	: 0.03
loss_45.9975	: 0.03
loss_46.9925	: 0.02
fragment_243.1475	: 0.02
loss_62.0175	: 0.02
fragment_81.0675	: 0.02
fragment_521.2425	: 0.01
fragment_159.1175	: 0.01
fragment_93.0675	: 0.01
loss_61.0125	: 0.01
loss_158.1075	: 0.01

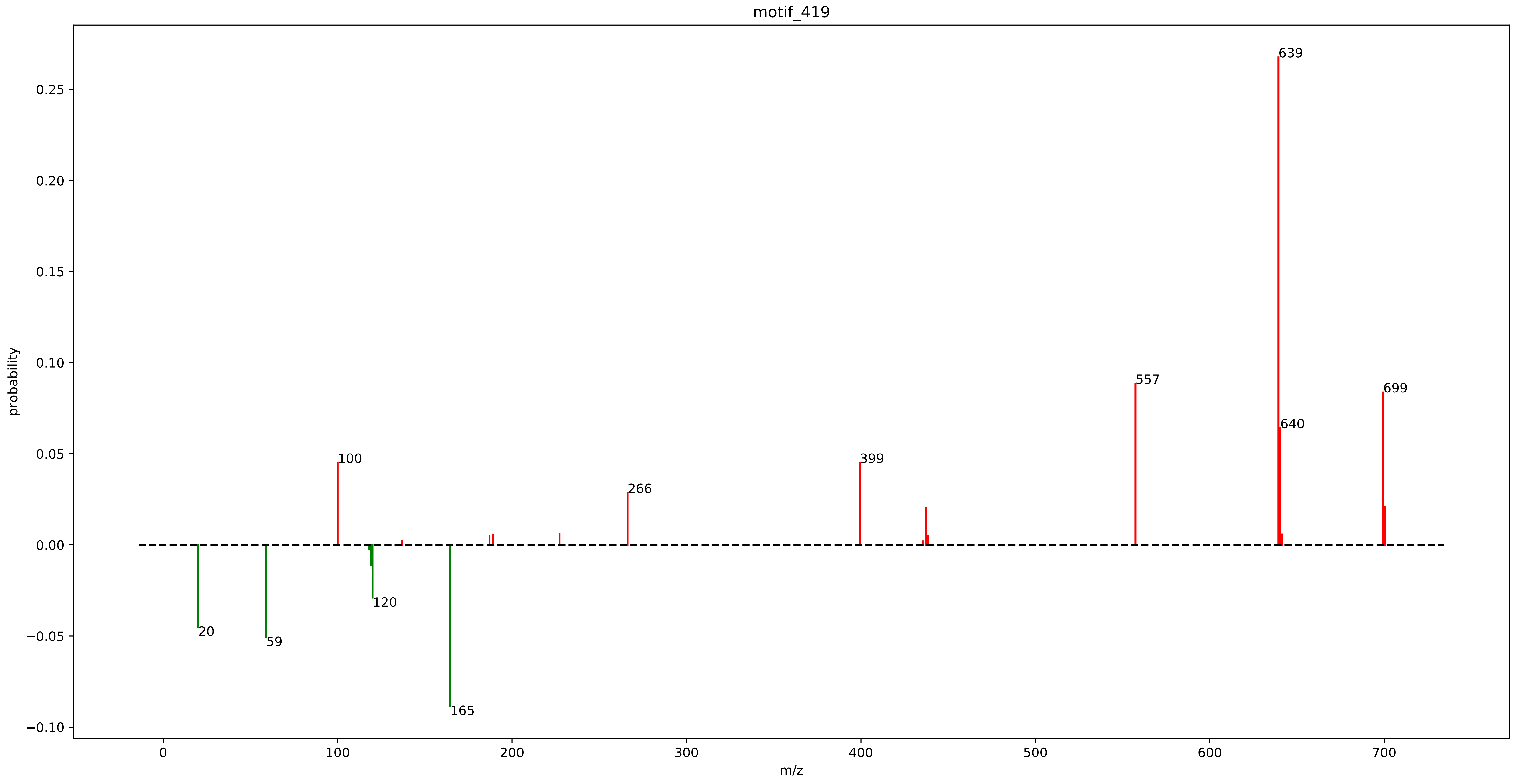

motif_419
probability
m/z
0.25
0.20
0.15
0.10
0.05
0.00
−0.05
−0.10
0
100
200
300
400
500
600
700
639
557
699
640
100
399
266
20
59
120
165

motif_419

fragment_639.3875	: 0.27
loss_164.5325	: 0.09
fragment_557.3825	: 0.09
fragment_699.4075	: 0.08
fragment_640.3925	: 0.06
loss_59.0125	: 0.05
fragment_399.2875	: 0.04
loss_20.0425	: 0.04
fragment_100.0375	: 0.04
loss_120.0425	: 0.03
fragment_266.2475	: 0.03
fragment_700.4125	: 0.02
fragment_437.3325	: 0.02
loss_119.0375	: 0.01
fragment_227.1975	: 0.01
fragment_641.3925	: 0.01
fragment_189.1475	: 0.01
fragment_438.3425	: 0.01

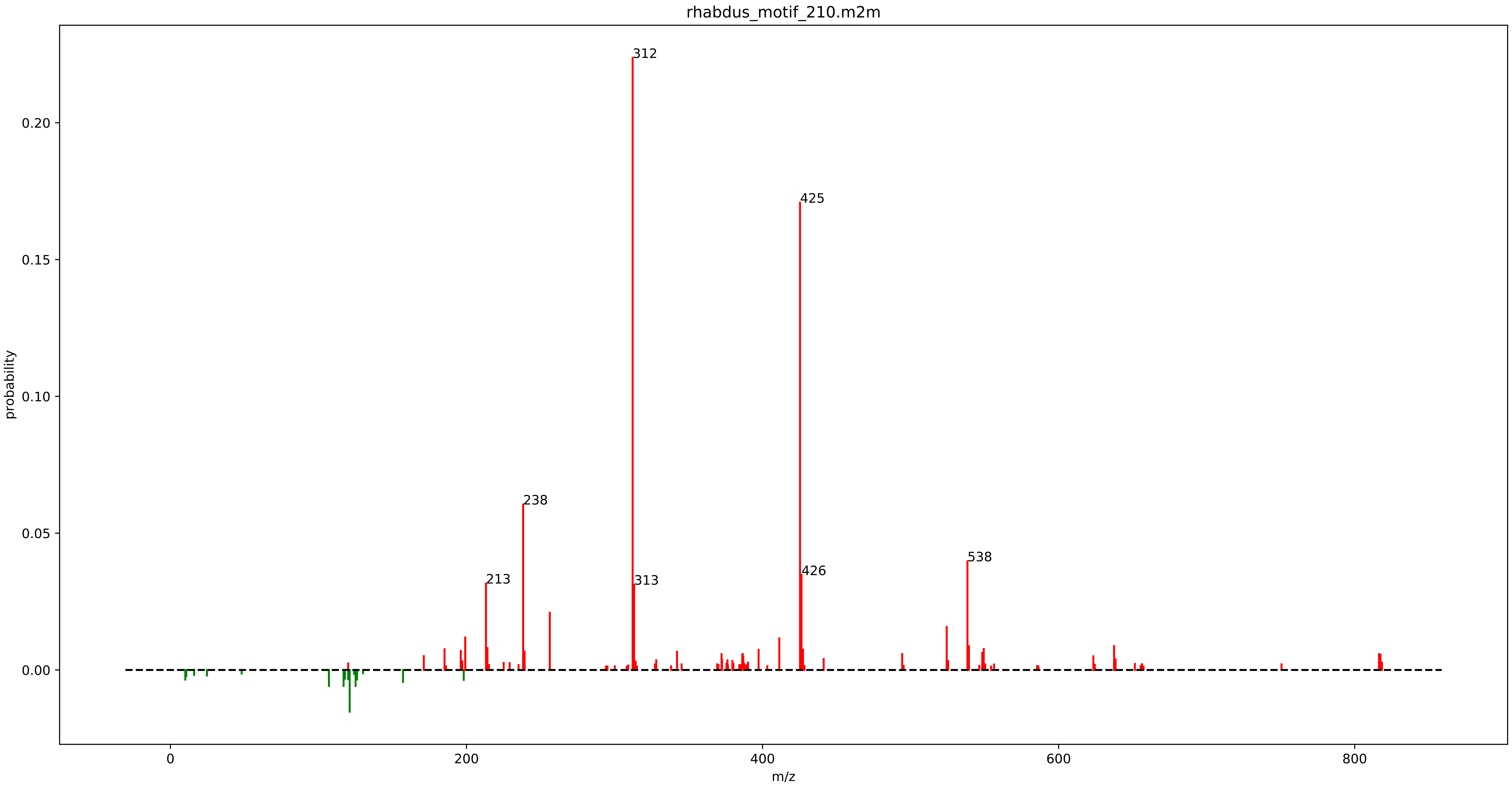

rhabdus_motif_210.m2m
probability
m/z
0.20
0.15
0.10
0.05
0.00
0
200
400
600
800
312
425
238
538
426
213
313

rhabdus_motif_210.m2m

fragment_312.2275	: 0.22
fragment_425.3125	: 0.17
fragment_238.2525	: 0.06
fragment_538.3975	: 0.04
fragment_426.3175	: 0.03
fragment_213.1575	: 0.03
fragment_313.2325	: 0.03
fragment_256.2625	: 0.02
fragment_524.3825	: 0.02
loss_121.0875	: 0.02
fragment_199.1525	: 0.01
fragment_411.2975	: 0.01
fragment_199.1475	: 0.01
fragment_637.4625	: 0.01
fragment_539.3975	: 0.01
fragment_214.1625	: 0.01
fragment_549.4075	: 0.01
fragment_185.1625	: 0.01
fragment_427.3175	: 0.01
fragment_426.3125	: 0.01
fragment_397.3175	: 0.01
fragment_196.1325	: 0.01
fragment_239.2575	: 0.01
fragment_342.2025	: 0.01
fragment_548.4025	: 0.01
loss_107.0575	: 0.01
fragment_494.3075	: 0.01
loss_116.9675	: 0.01
fragment_386.6125	: 0.01
loss_125.0925	: 0.01
fragment_372.2825	: 0.01
fragment_816.4025	: 0.01
fragment_817.4125	: 0.01
fragment_386.2725	: 0.01
fragment_171.1475	: 0.01

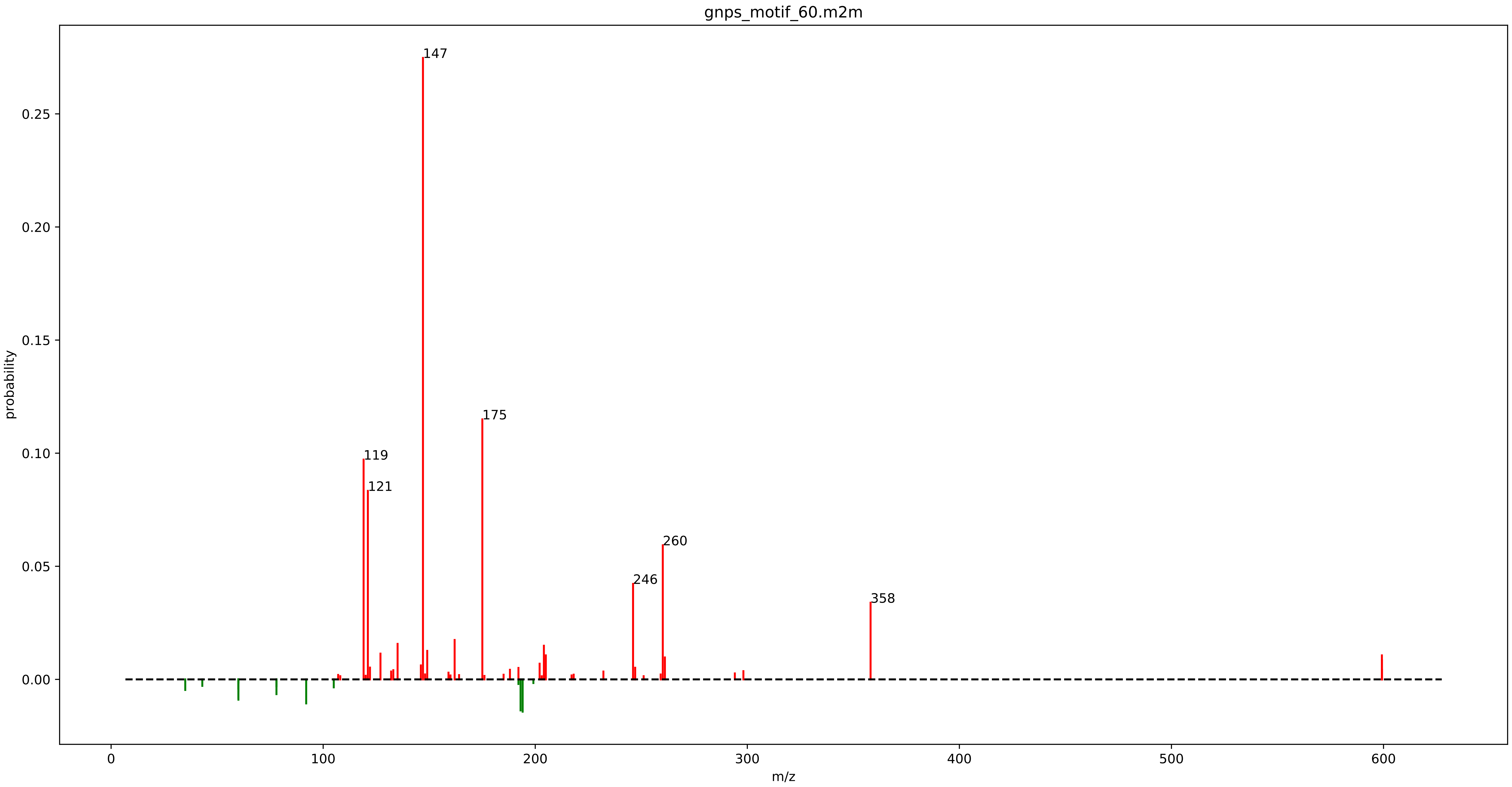

gnps_motif_60.m2m
probability
m/z
0.25
0.20
0.15
0.10
0.05
0.00
0
100
200
300
400
500
600
147
175
119
121
260
246
358

gnps_motif_60.m2m

fragment_147.0575	: 0.27
fragment_175.0525	: 0.11
fragment_119.0625	: 0.10
fragment_121.0775	: 0.08
fragment_260.1425	: 0.06
fragment_246.1225	: 0.04
fragment_358.1225	: 0.03
fragment_161.9875	: 0.02
fragment_135.0925	: 0.02
fragment_204.0775	: 0.01
loss_194.0675	: 0.01
loss_193.0725	: 0.01
fragment_149.0725	: 0.01
fragment_127.0175	: 0.01
loss_91.9925	: 0.01
fragment_599.2325	: 0.01
fragment_204.9925	: 0.01
fragment_261.1425	: 0.01
loss_60.0325	: 0.01
fragment_599.2575	: 0.01
loss_60.0125	: 0.01
fragment_202.0625	: 0.01
loss_77.9725	: 0.01
fragment_146.0625	: 0.01
fragment_122.0775	: 0.01
fragment_247.1275	: 0.01
fragment_192.0825	: 0.01

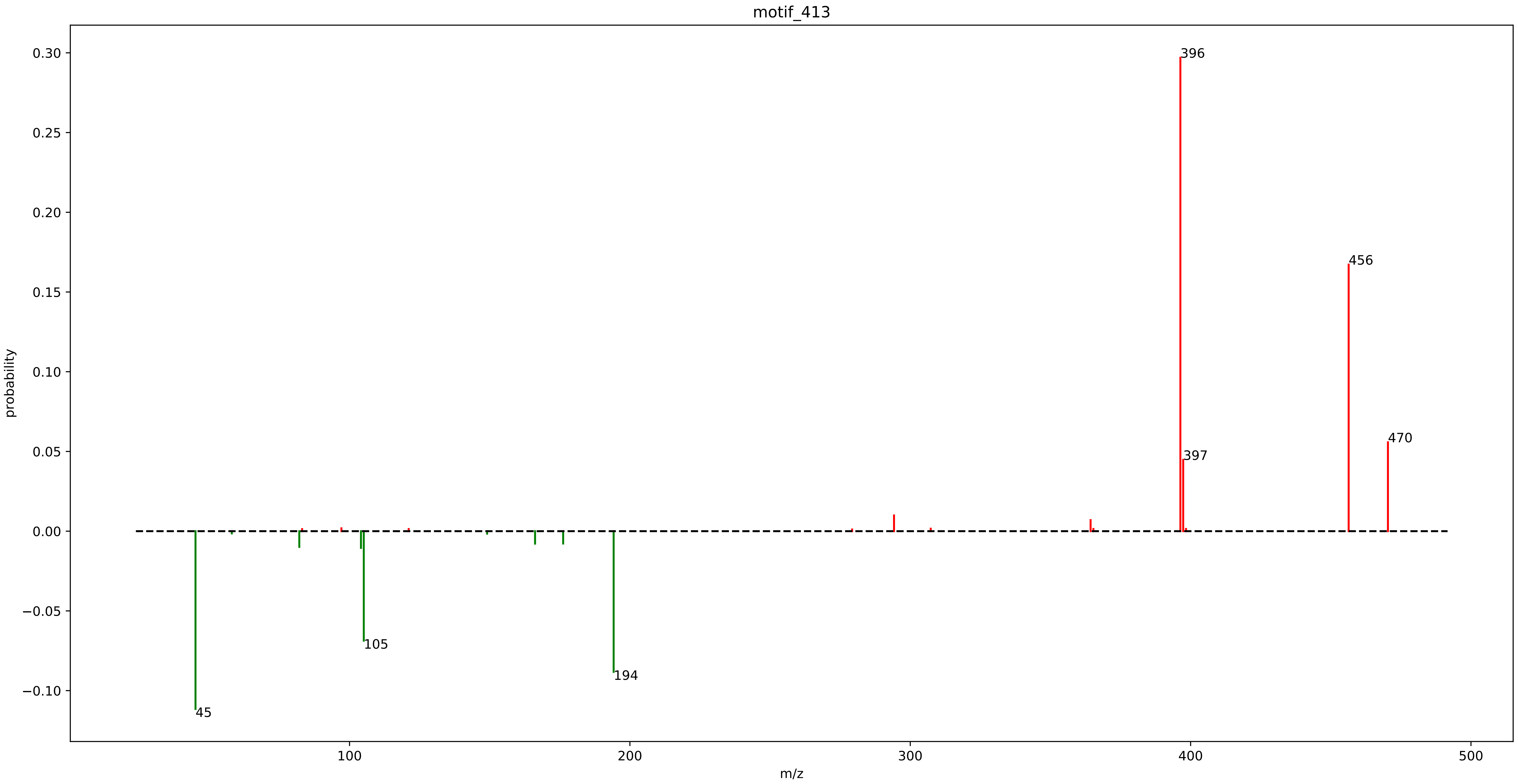
motif_413
probability
m/z
0.30
0.25
0.20
0.15
0.10
0.05
0.00
−0.05
−0.10
100
200
300
400
500
396
456
470
397
45
105
194

motif_413

fragment_396.3475	: 0.30
fragment_456.3675	: 0.17
loss_45.0575	: 0.11
loss_194.2025	: 0.09
loss_105.0775	: 0.07
fragment_470.3825	: 0.06
fragment_397.3525	: 0.04
loss_104.0725	: 0.01
fragment_294.2075	: 0.01
loss_82.0775	: 0.01
loss_166.1725	: 0.01
loss_176.1925	: 0.01
fragment_364.3225	: 0.01

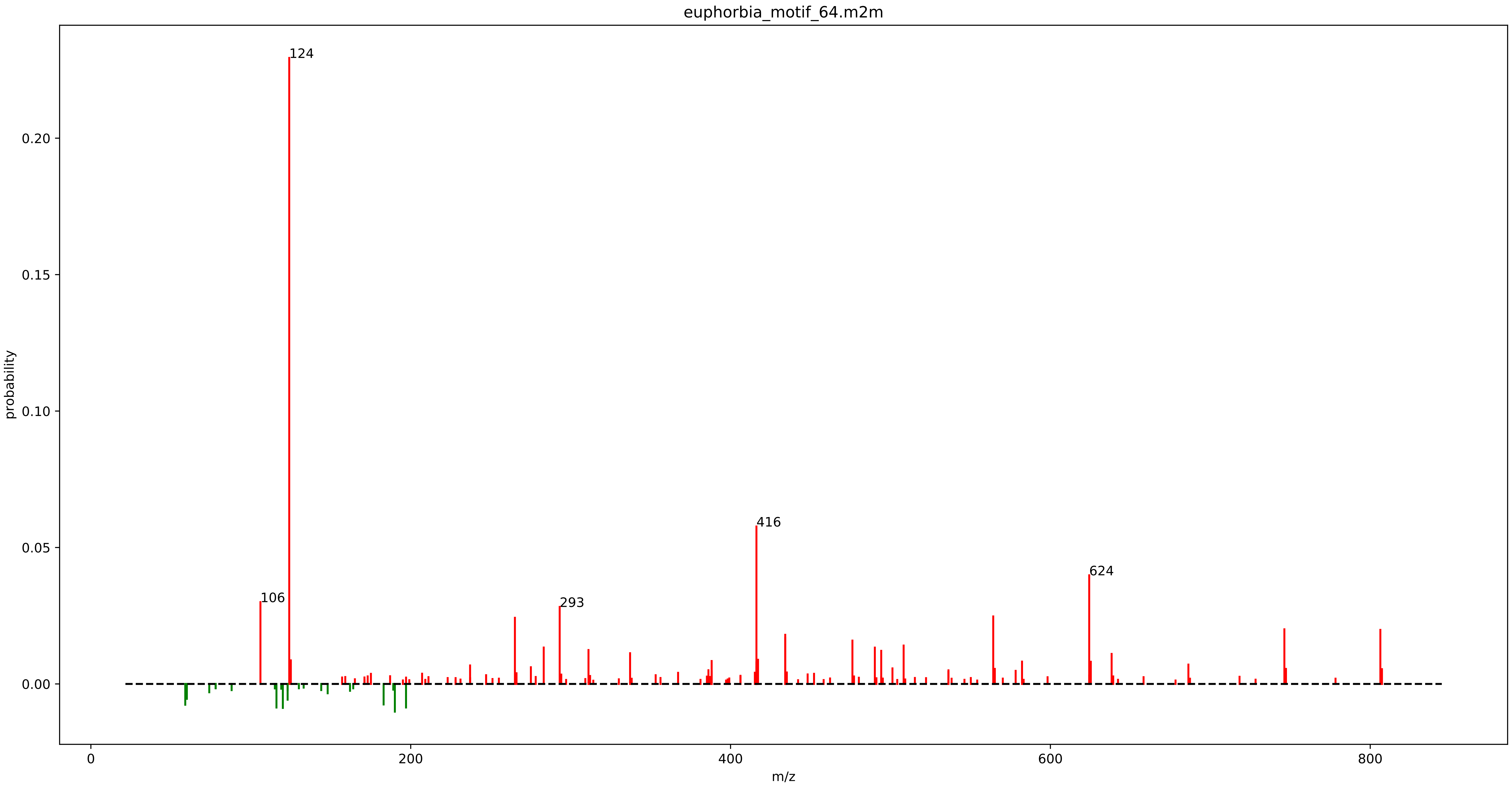

euphorbia_motif_64.m2m
probability
m/z
124
106
293
416
624
0.20
0.15
0.10
0.05
0.00
0
200
400
600
800

euphorbia_motif_64.m2m

fragment_124.0375 : 0.23
fragment_416.1875 : 0.06
fragment_624.2775 : 0.04
fragment_106.0275 : 0.03
fragment_293.1525 : 0.03
fragment_564.2575 : 0.02
fragment_265.1575 : 0.02
fragment_746.3175 : 0.02
fragment_806.3375 : 0.02
fragment_434.1925 : 0.02
fragment_434.1975 : 0.02
fragment_476.2075 : 0.02
fragment_508.2325 : 0.01
fragment_283.1675 : 0.01
fragment_490.2225 : 0.01
fragment_311.1625 : 0.01
fragment_494.2175 : 0.01
fragment_337.1775 : 0.01
fragment_638.2975 : 0.01
loss_190.0875 : 0.01
fragment_417.1875 : 0.01
loss_120.0425 : 0.01
loss_116.0475 : 0.01
fragment_125.0425 : 0.01
loss_197.0675 : 0.01
fragment_388.1875 : 0.01
fragment_582.2675 : 0.01
fragment_625.2825 : 0.01
fragment_416.1825 : 0.01
loss_59.0175 : 0.01
loss_183.0525 : 0.01
fragment_686.2975 : 0.01
fragment_237.1275 : 0.01
fragment_275.1425 : 0.01
fragment_416.1925 : 0.01
loss_123.0325 : 0.01
fragment_501.2425 : 0.01
fragment_747.3225 : 0.01
fragment_565.2625 : 0.01
loss_60.0225 : 0.01
fragment_807.3425 : 0.01
fragment_386.1725 : 0.01

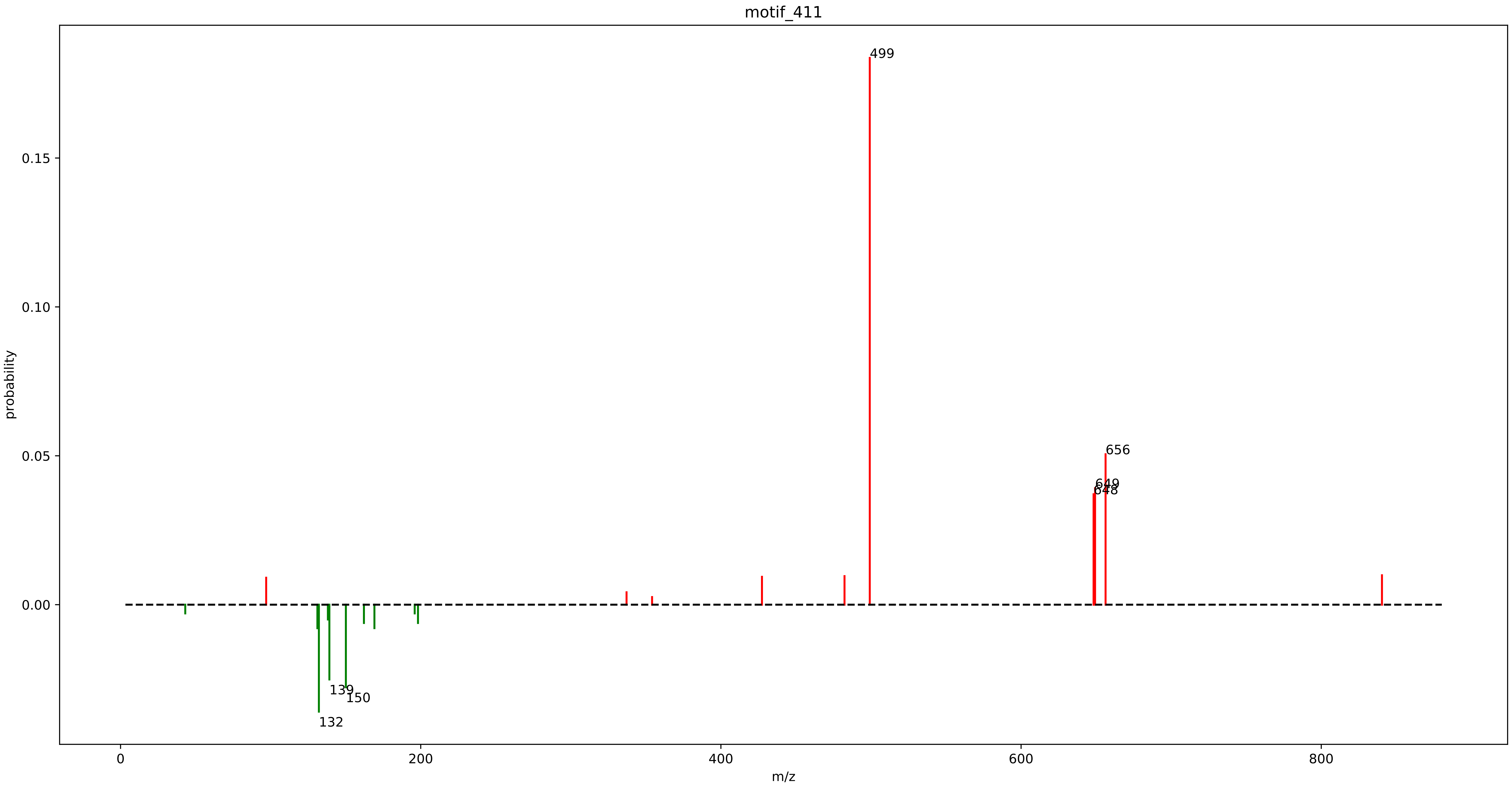

motif_411
probability
m/z
0.15
0.10
0.05
0.00
0
200
400
600
800
499
656
649
648
139
150
132

motif_411

fragment_499.1825	: 0.18
fragment_656.2925	: 0.05
fragment_649.3025	: 0.04
fragment_648.2975	: 0.04
loss_132.1475	: 0.04
loss_150.1275	: 0.03
loss_139.1375	: 0.03
fragment_840.4475	: 0.01
fragment_482.3575	: 0.01
fragment_427.3575	: 0.01
fragment_97.0275	: 0.01
loss_131.1425	: 0.01
loss_169.1475	: 0.01
loss_198.1775	: 0.01
loss_162.1575	: 0.01

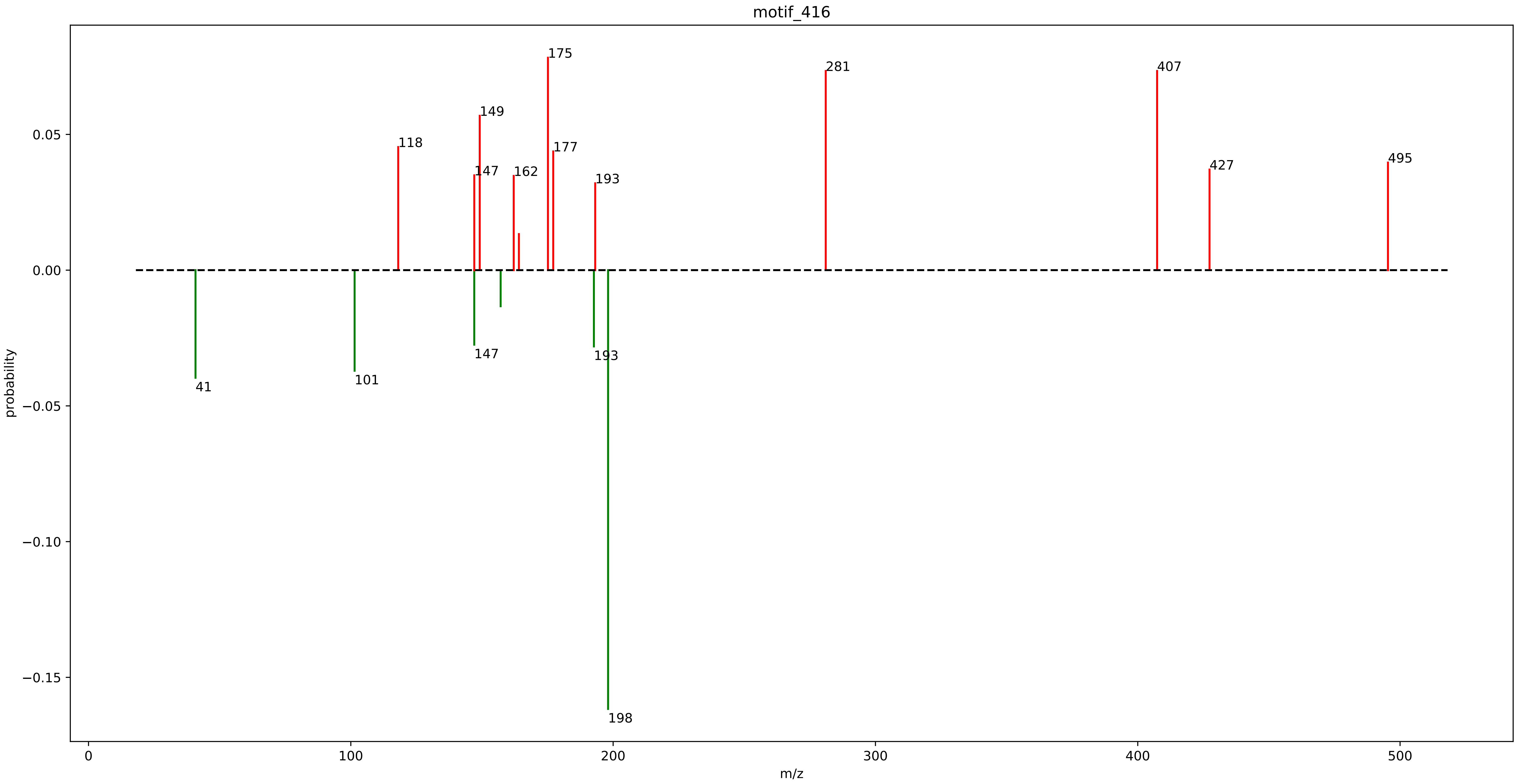

motif_416
probability
m/z
0.05
0.00
−0.05
−0.10
−0.15
0
100
200
300
400
500
175
281
407
149
118
177
147
162
193
427
495
41
101
147
193
198

motif_416

loss_198.0725	: 0.16
fragment_175.1475	: 0.08
fragment_281.0475	: 0.07
fragment_407.3675	: 0.07
fragment_149.1325	: 0.06
fragment_118.0625	: 0.05
fragment_177.1625	: 0.04
fragment_495.3775	: 0.04
loss_40.7875	: 0.04
loss_101.4325	: 0.04
fragment_427.3575	: 0.04
fragment_147.0625	: 0.03
fragment_162.0875	: 0.03
fragment_193.1575	: 0.03
loss_192.6625	: 0.03
loss_147.0525	: 0.03
loss_157.1075	: 0.01
fragment_164.0725	: 0.01

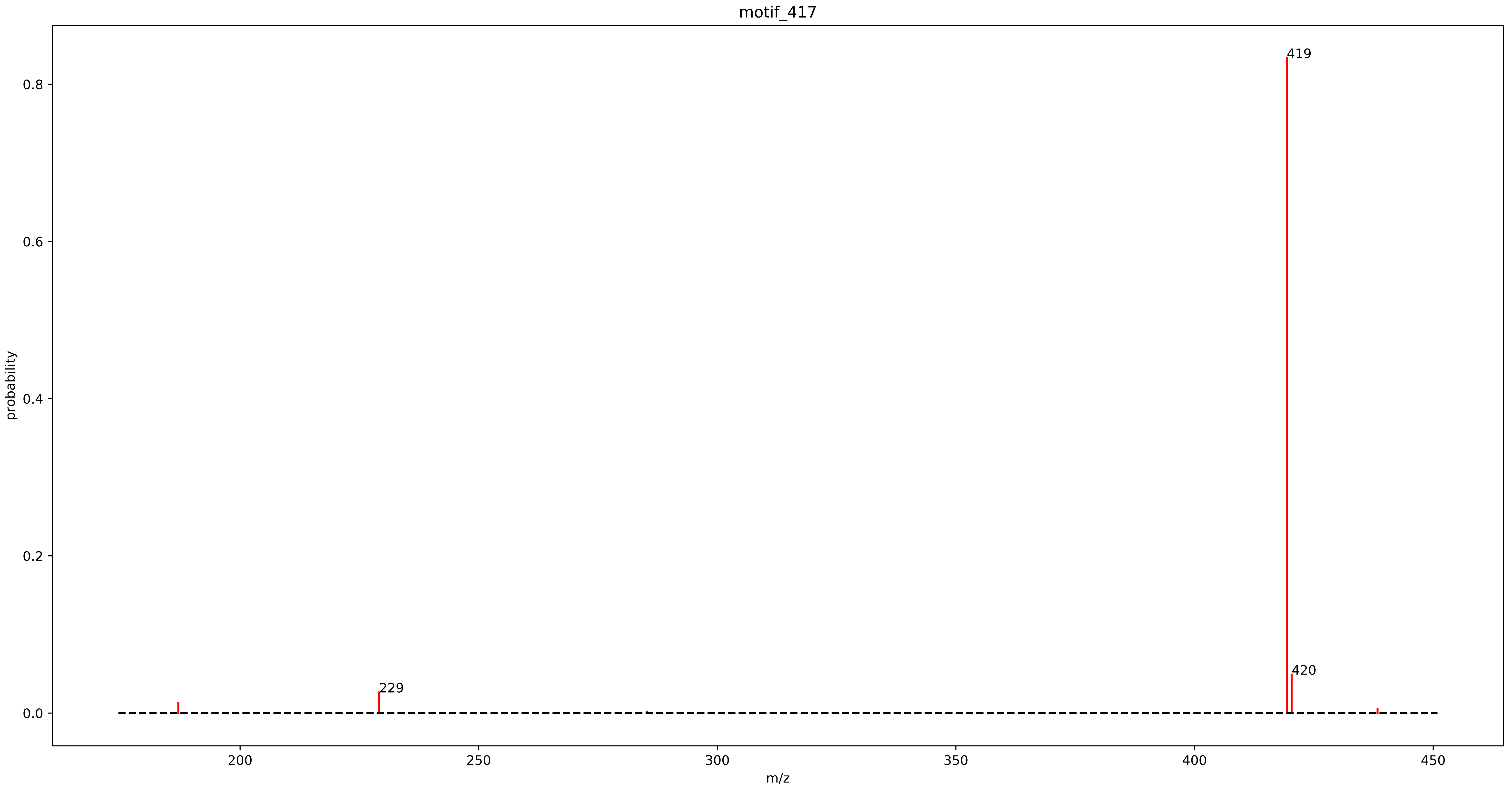

motif_417
419
420
229
0.8
0.6
0.4
0.2
0.0
probability
200
250
300
350
400
450
m/z

motif_417

fragment_419.3275	: 0.83
fragment_420.3325	: 0.05
fragment_229.1425	: 0.03
fragment_187.0625	: 0.01
fragment_438.3425	: 0.01

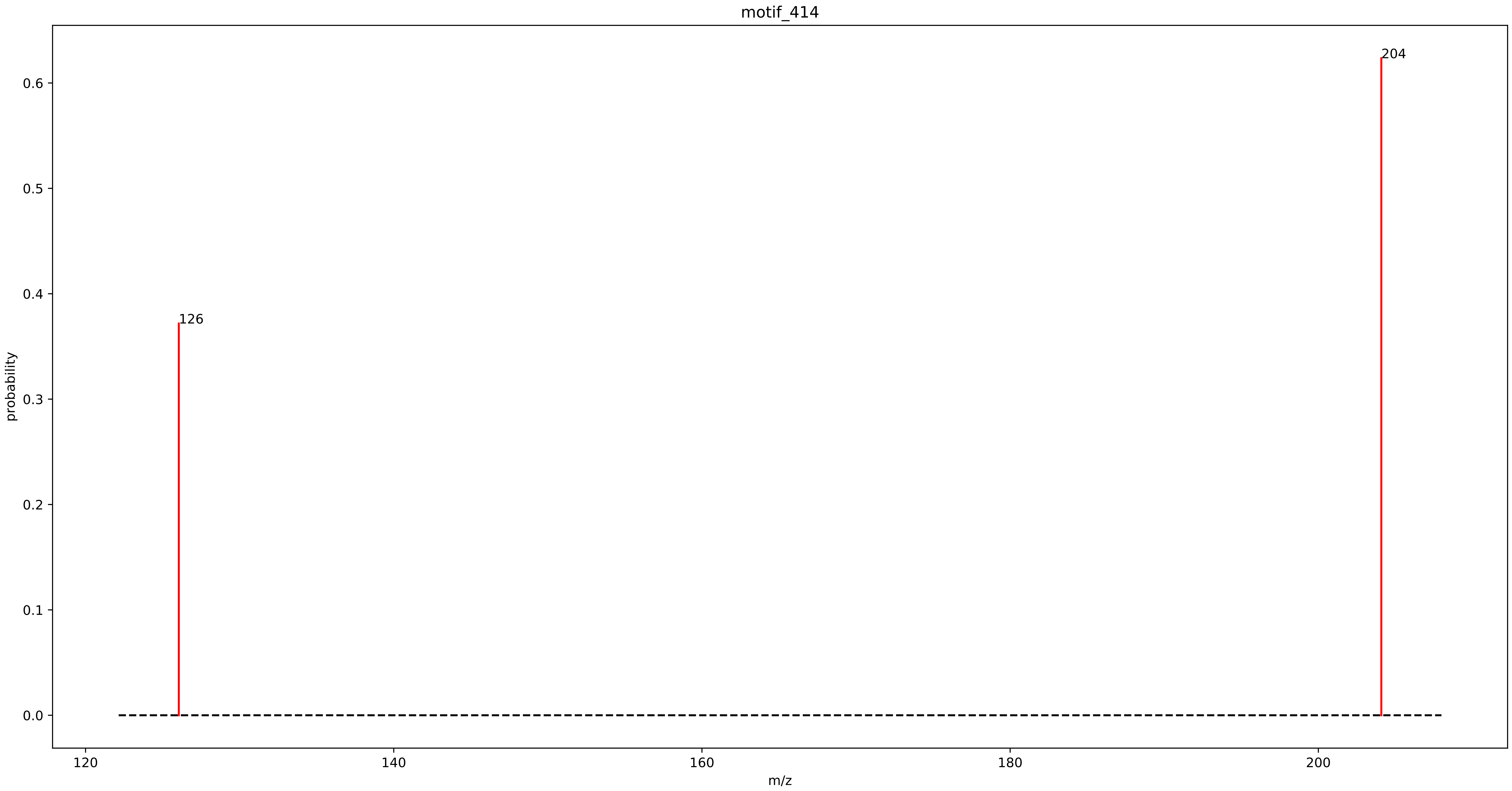
motif_414
probability
m/z
126
204
0.0
0.1
0.2
0.3
0.4
0.5
0.6
120
140
160
180
200

motif_414

fragment_204.0875	: 0.62
fragment_126.0525	: 0.37

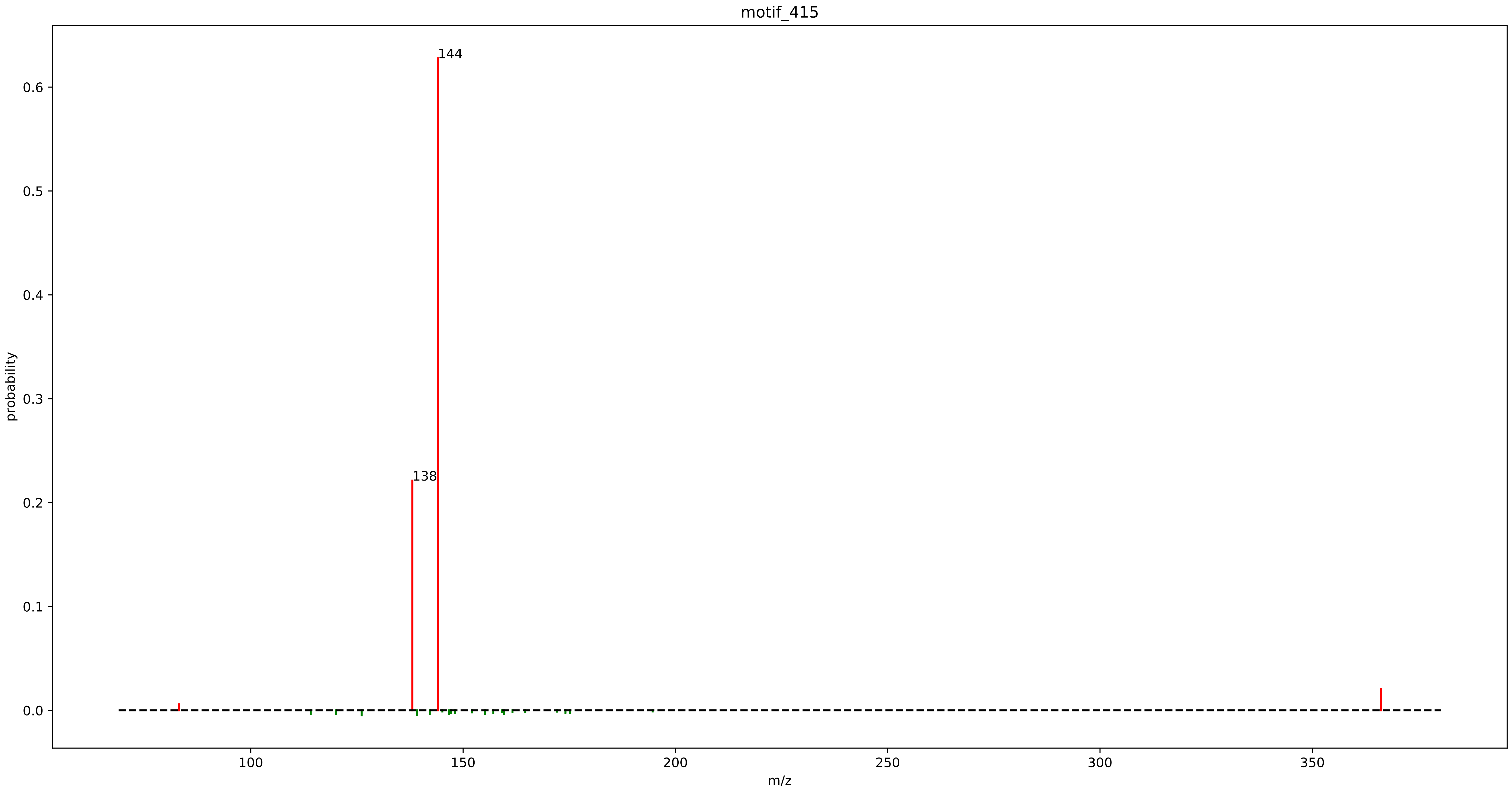

motif_415
144
138
probability
0.6
0.5
0.4
0.3
0.2
0.1
0.0
100
150
200
250
300
350
m/z

motif_415

fragment_144.0675	: 0.63
fragment_138.0525	: 0.22
fragment_366.1425	: 0.02
fragment_83.0475	: 0.01

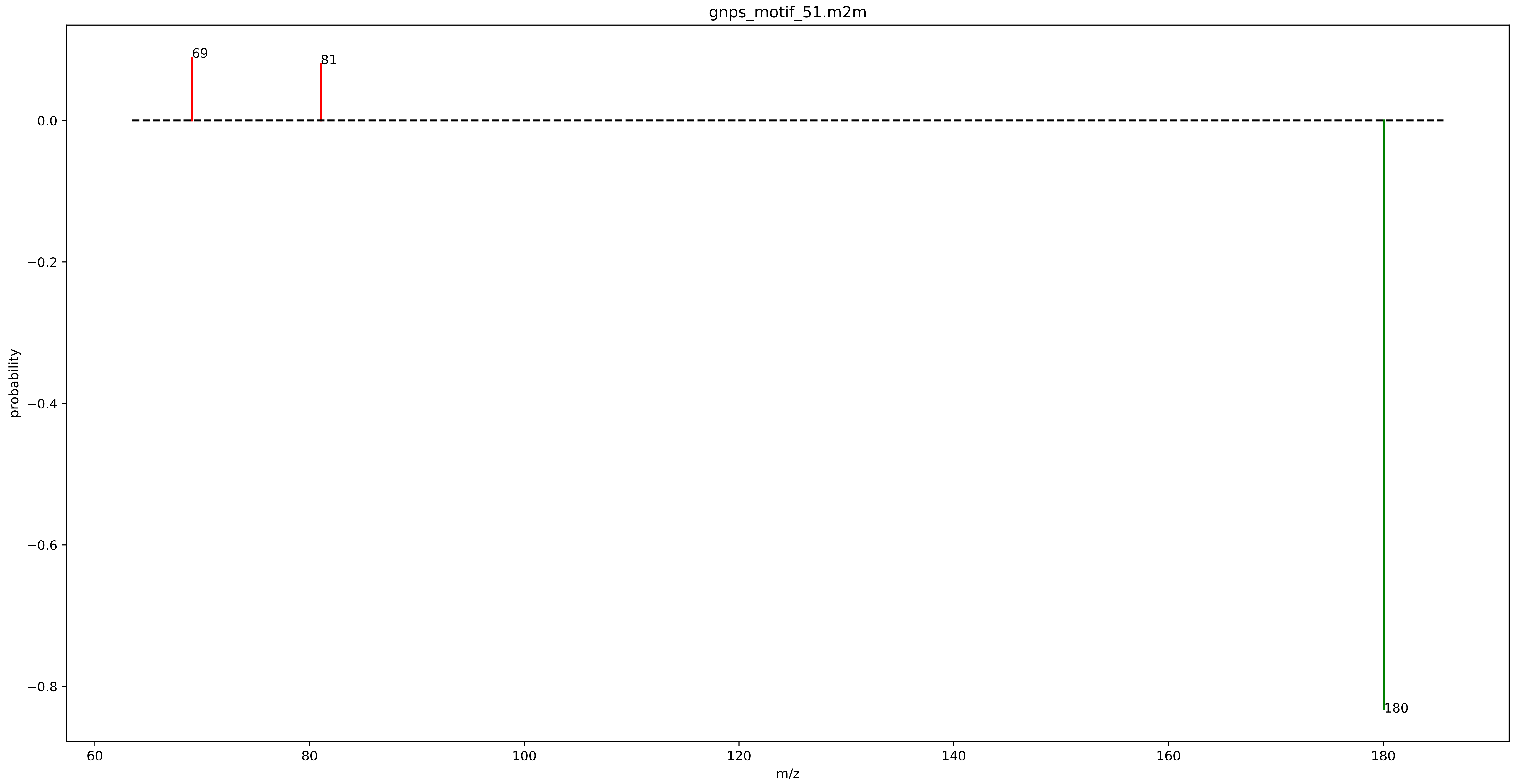

gnps_motif_51.m2m
69
81
180
0.0
−0.2
−0.4
−0.6
−0.8
probability
60
80
100
120
140
160
180
m/z

gnps_motif_51.m2m

loss_180.0625	: 0.83
fragment_69.0325	: 0.09
fragment_81.0325	: 0.08

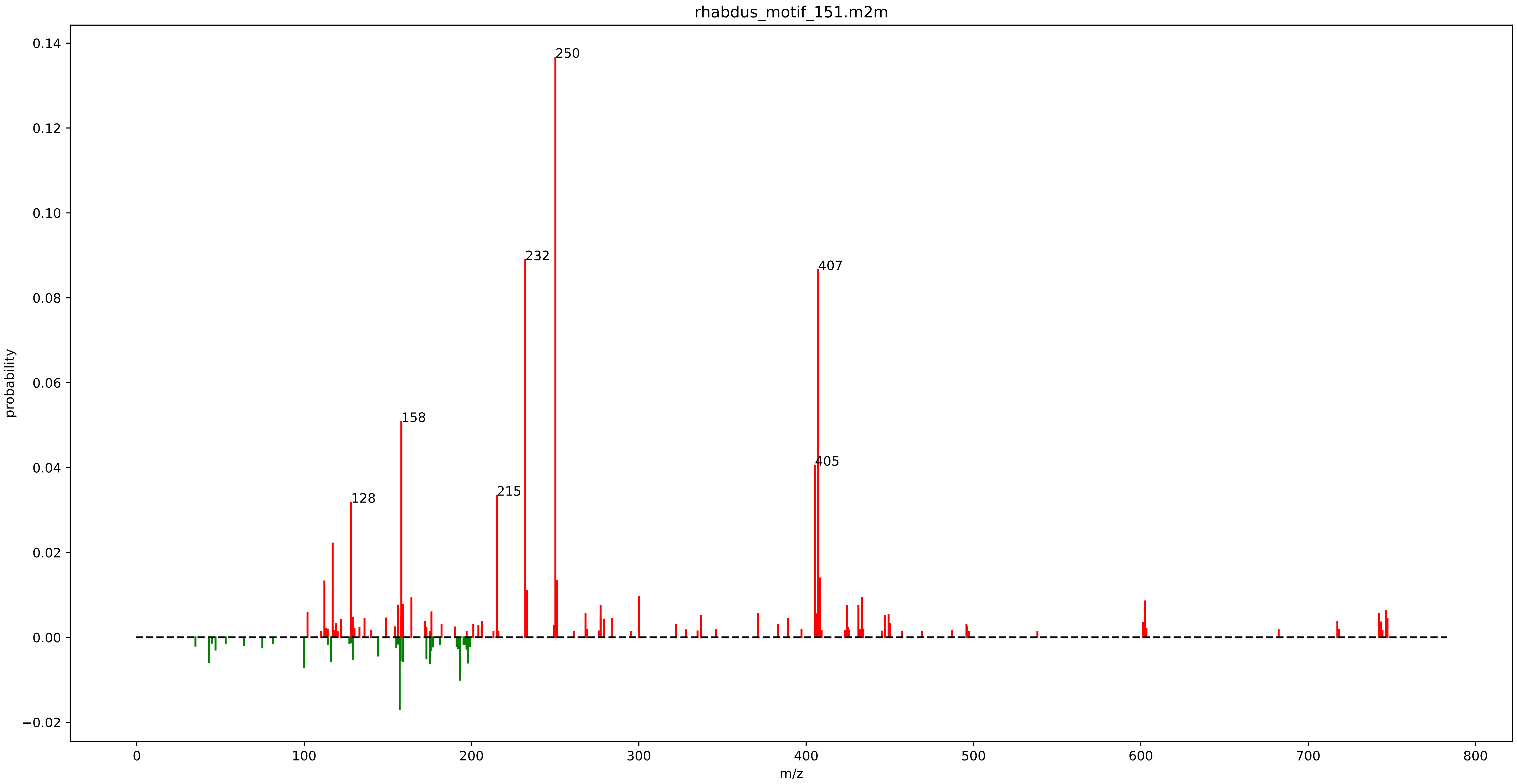

rhabdus_motif_151.m2m
probability
m/z
0.14
0.12
0.10
0.08
0.06
0.04
0.02
0.00
−0.02
0
100
200
300
400
500
600
700
800
250
232
407
158
405
215
128

rhabdus_motif_151.m2m

fragment_250.1425	: 0.14
fragment_232.1325	: 0.09
fragment_407.2175	: 0.09
fragment_158.0825	: 0.05
fragment_405.2025	: 0.04
fragment_215.1075	: 0.03
fragment_128.0675	: 0.03
fragment_117.0375	: 0.02
loss_157.0725	: 0.02
fragment_408.2225	: 0.01
fragment_251.1475	: 0.01
fragment_112.0725	: 0.01
fragment_233.1225	: 0.01
loss_193.0775	: 0.01
fragment_300.1925	: 0.01
fragment_433.1975	: 0.01
fragment_164.0825	: 0.01
fragment_602.3625	: 0.01
fragment_159.0475	: 0.01
fragment_156.0675	: 0.01
fragment_431.2175	: 0.01
fragment_424.3775	: 0.01
fragment_277.1625	: 0.01
loss_100.0525	: 0.01
fragment_128.0725	: 0.01
fragment_746.3575	: 0.01
loss_175.0875	: 0.01
loss_198.0325	: 0.01
fragment_176.0725	: 0.01
fragment_102.0125	: 0.01
loss_43.0025	: 0.01
fragment_233.1275	: 0.01
loss_116.0275	: 0.01
fragment_371.1975	: 0.01
fragment_742.4025	: 0.01
loss_159.0175	: 0.01
fragment_268.1525	: 0.01
loss_157.6375	: 0.01
fragment_406.2025	: 0.01
fragment_449.2275	: 0.01
fragment_233.1325	: 0.01
fragment_215.1175	: 0.01
fragment_447.2125	: 0.01
loss_129.0575	: 0.01

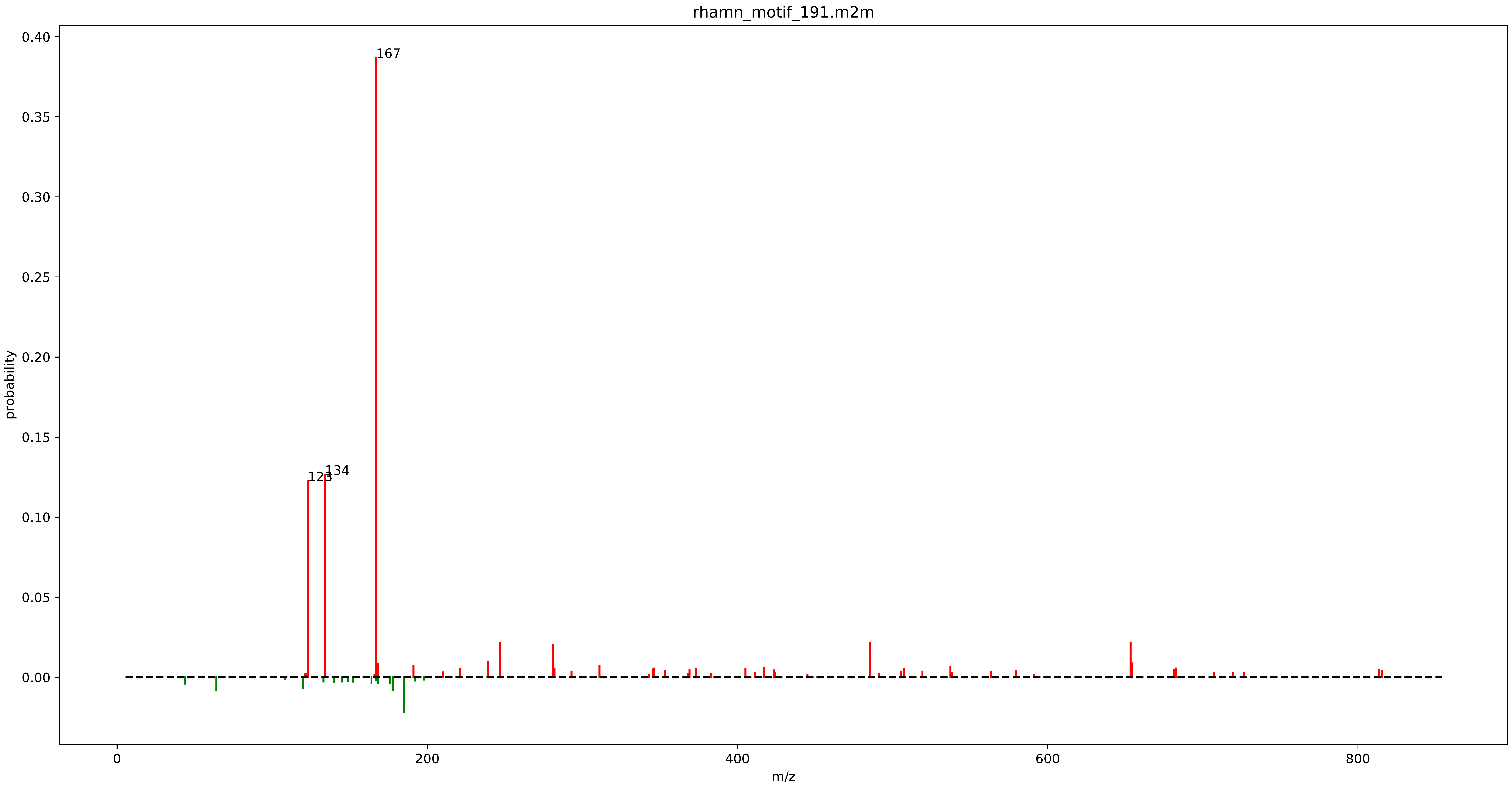

rhamn_motif_191.m2m
probability
m/z
0.40
0.35
0.30
0.25
0.20
0.15
0.10
0.05
0.00
0
200
400
600
800
167
123
134

rhamn_motif_191.m2m

fragment_167.0325	: 0.39
fragment_134.0375	: 0.13
fragment_123.0425	: 0.12
loss_184.9625	: 0.02
fragment_485.3325	: 0.02
fragment_653.3525	: 0.02
fragment_247.1675	: 0.02
fragment_281.0675	: 0.02
fragment_239.0525	: 0.01
fragment_654.3525	: 0.01
fragment_168.0375	: 0.01
loss_64.0275	: 0.01
fragment_247.1625	: 0.01
loss_178.0625	: 0.01
fragment_311.0775	: 0.01
loss_120.0925	: 0.01
fragment_191.0925	: 0.01
fragment_537.2125	: 0.01
fragment_417.2975	: 0.01
fragment_682.3925	: 0.01
fragment_346.2425	: 0.01
fragment_405.1575	: 0.01
fragment_221.0425	: 0.01
fragment_507.2925	: 0.01
fragment_282.0725	: 0.01
fragment_373.2675	: 0.01

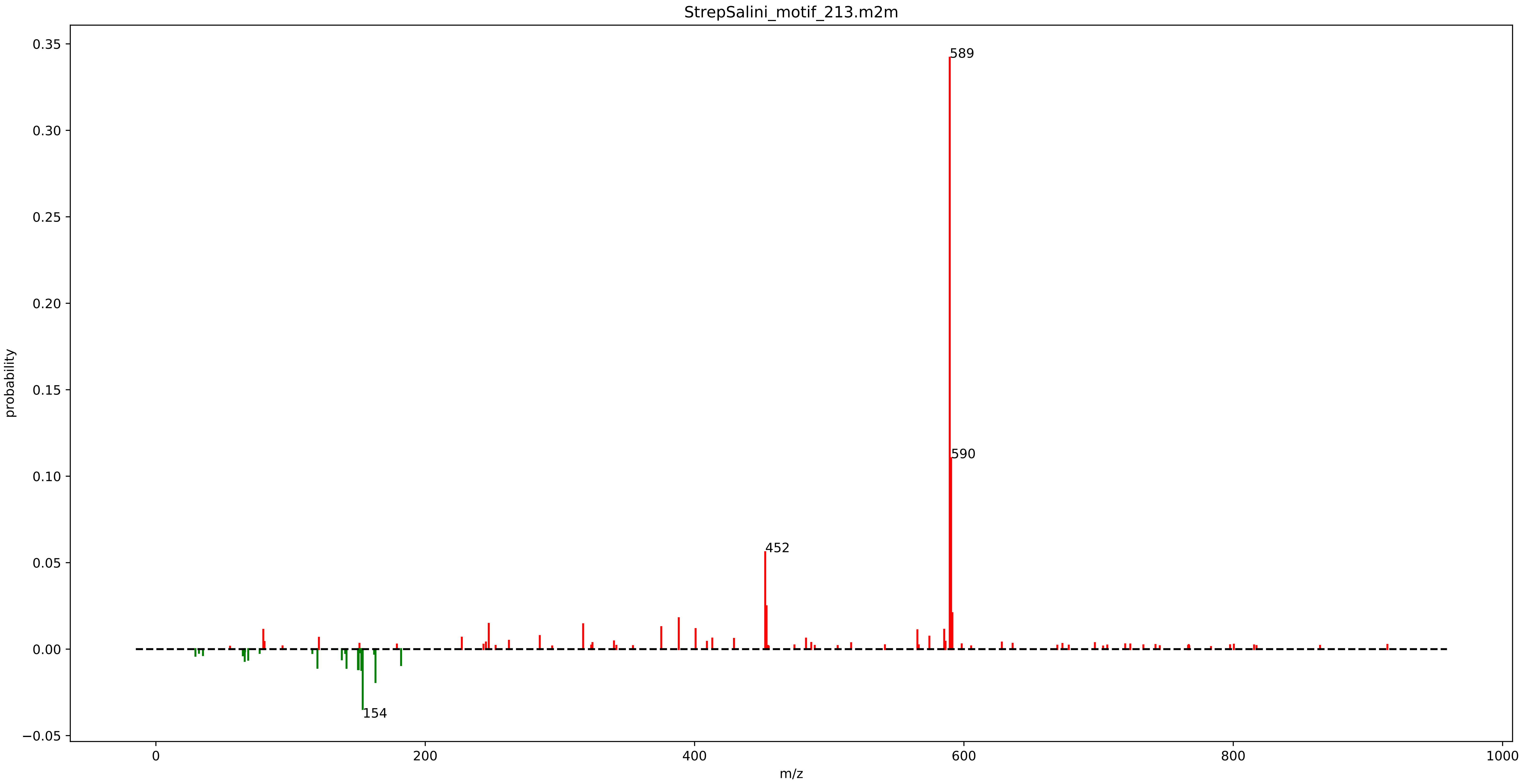

StrepSalini_motif_213.m2m
probability
m/z
589
590
452
154
0.35
0.30
0.25
0.20
0.15
0.10
0.05
0.00
−0.05
0
200
400
600
800
1000

StrepSalini_motif_213.m2m

fragment_589.4500	: 0.34
fragment_590.4500	: 0.11
fragment_452.4500	: 0.06
loss_153.5500	: 0.03
fragment_453.4500	: 0.02
fragment_591.4500	: 0.02
loss_163.0500	: 0.02
fragment_388.2500	: 0.02
fragment_247.1525	: 0.01
fragment_317.2500	: 0.01
fragment_375.2500	: 0.01
loss_152.5500	: 0.01
fragment_400.7500	: 0.01
loss_150.5500	: 0.01
loss_150.0525	: 0.01
fragment_585.3500	: 0.01
fragment_79.7500	: 0.01
fragment_565.4500	: 0.01
loss_141.5500	: 0.01
loss_119.9500	: 0.01
loss_182.0500	: 0.01
fragment_285.0500	: 0.01
fragment_574.3500	: 0.01
loss_65.9500	: 0.01
fragment_227.1500	: 0.01
fragment_121.0525	: 0.01
fragment_413.1500	: 0.01
loss_68.5500	: 0.01
fragment_482.7500	: 0.01
fragment_429.2500	: 0.01
loss_138.0525	: 0.01

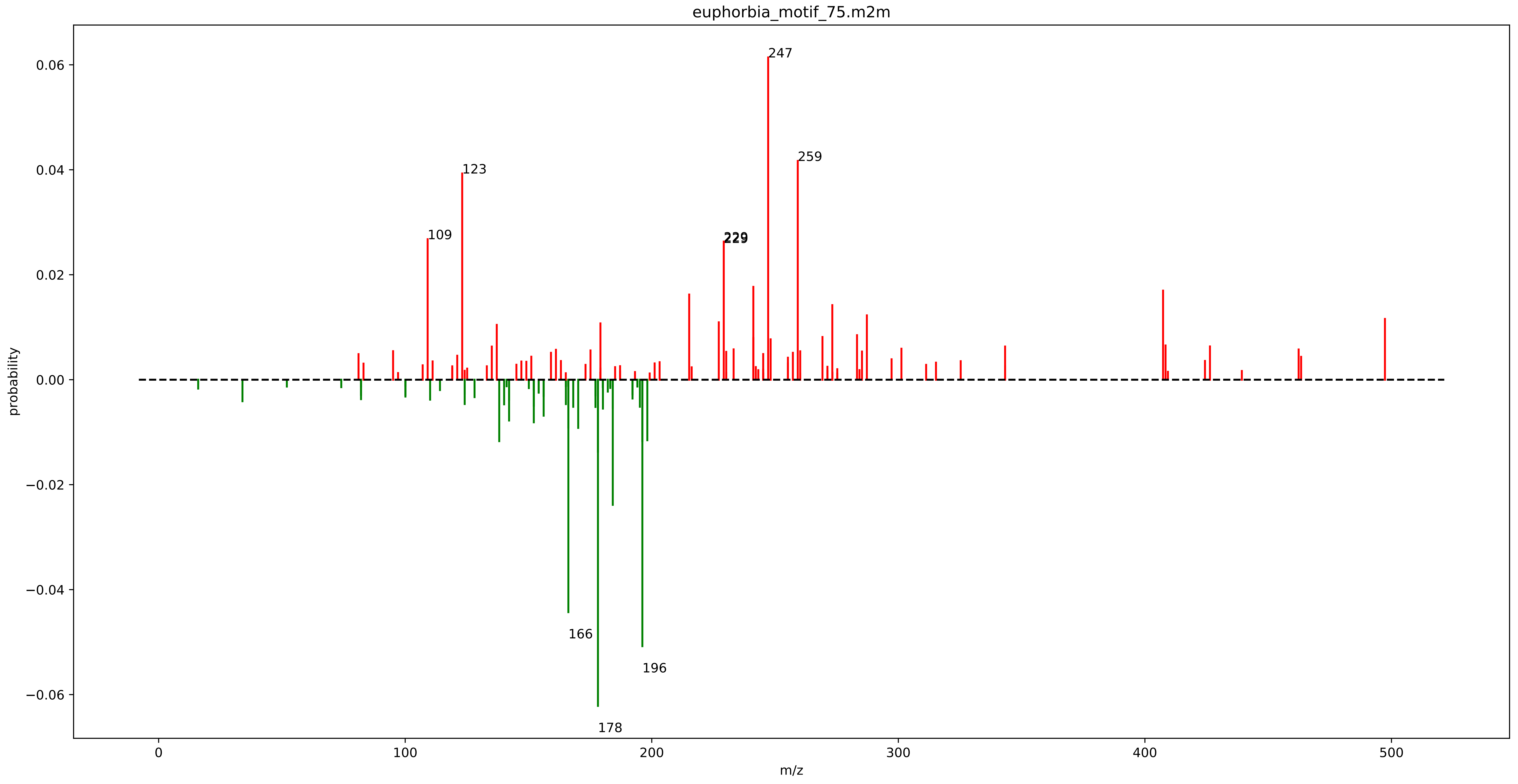

euphorbia_motif_75.m2m
probability
m/z
247
259
123
109
229
166
196
178

euphorbia_motif_75.m2m

loss_178.1725	: 0.06
fragment_247.2075	: 0.06
loss_196.1825	: 0.05
loss_166.1725	: 0.04
fragment_259.2075	: 0.04
fragment_123.1175	: 0.04
fragment_109.1025	: 0.03
fragment_229.1925	: 0.03
fragment_229.1975	: 0.03
loss_184.1825	: 0.02
fragment_241.1925	: 0.02
fragment_407.3675	: 0.02
fragment_215.1775	: 0.02
fragment_273.2225	: 0.01
loss_178.1675	: 0.01
fragment_287.2375	: 0.01
loss_138.1375	: 0.01
loss_196.1775	: 0.01
fragment_497.3525	: 0.01
loss_198.1975	: 0.01
fragment_227.1775	: 0.01
fragment_179.1775	: 0.01
fragment_137.1325	: 0.01
loss_170.1675	: 0.01
loss_166.1675	: 0.01
fragment_283.2425	: 0.01
fragment_269.2275	: 0.01
loss_152.1525	: 0.01
fragment_241.1975	: 0.01
loss_142.1325	: 0.01
fragment_248.2075	: 0.01
loss_156.1525	: 0.01
fragment_408.3725	: 0.01
fragment_426.3825	: 0.01
fragment_343.2975	: 0.01
fragment_135.1175	: 0.01
fragment_301.2525	: 0.01
fragment_233.1925	: 0.01
fragment_462.3475	: 0.01
fragment_161.1325	: 0.01
fragment_175.1475	: 0.01
loss_180.1875	: 0.01
fragment_95.0875	: 0.01
fragment_260.2075	: 0.01
fragment_285.2575	: 0.01
loss_184.1775	: 0.01
fragment_230.1975	: 0.01
loss_177.1675	: 0.01
loss_168.1525	: 0.01
loss_195.1775	: 0.01
fragment_257.2275	: 0.01
fragment_159.1175	: 0.01

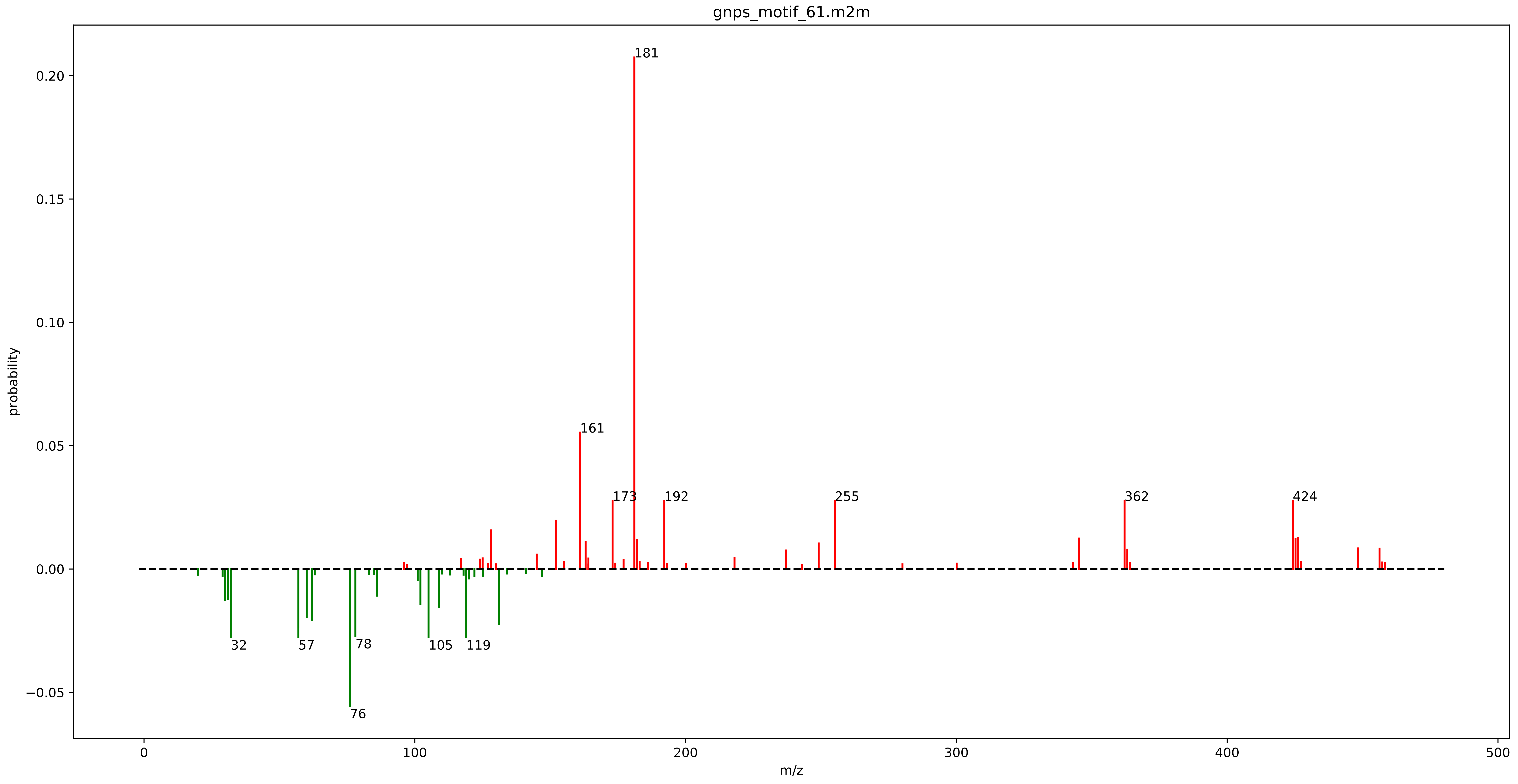

gnps_motif_61.m2m
probability
m/z
0.20
0.15
0.10
0.05
0.00
−0.05
0
100
200
300
400
500
181
161
173
192
255
362
424
32
57
78
105
119
76

gnps_motif_61.m2m

fragment_181.0725	: 0.21
loss_76.0275	: 0.06
fragment_161.0425	: 0.06
loss_119.0125	: 0.03
loss_32.0225	: 0.03
fragment_424.2225	: 0.03
fragment_362.1075	: 0.03
loss_105.0775	: 0.03
fragment_173.0225	: 0.03
fragment_255.1075	: 0.03
fragment_192.1125	: 0.03
loss_56.9975	: 0.03
loss_78.0675	: 0.03
loss_57.0225	: 0.02
loss_131.0575	: 0.02
loss_61.9925	: 0.02
loss_60.0525	: 0.02
fragment_152.0725	: 0.02
fragment_128.0625	: 0.02
loss_109.0075	: 0.02
loss_102.0675	: 0.01
fragment_426.2225	: 0.01
loss_30.0275	: 0.01
fragment_345.2025	: 0.01
fragment_425.2275	: 0.01
loss_31.0225	: 0.01
fragment_182.0725	: 0.01
fragment_163.0875	: 0.01
loss_86.0475	: 0.01
fragment_249.1375	: 0.01
fragment_448.2775	: 0.01
fragment_456.2525	: 0.01
fragment_363.1075	: 0.01
fragment_237.0675	: 0.01
fragment_145.0275	: 0.01
fragment_363.1325	: 0.01

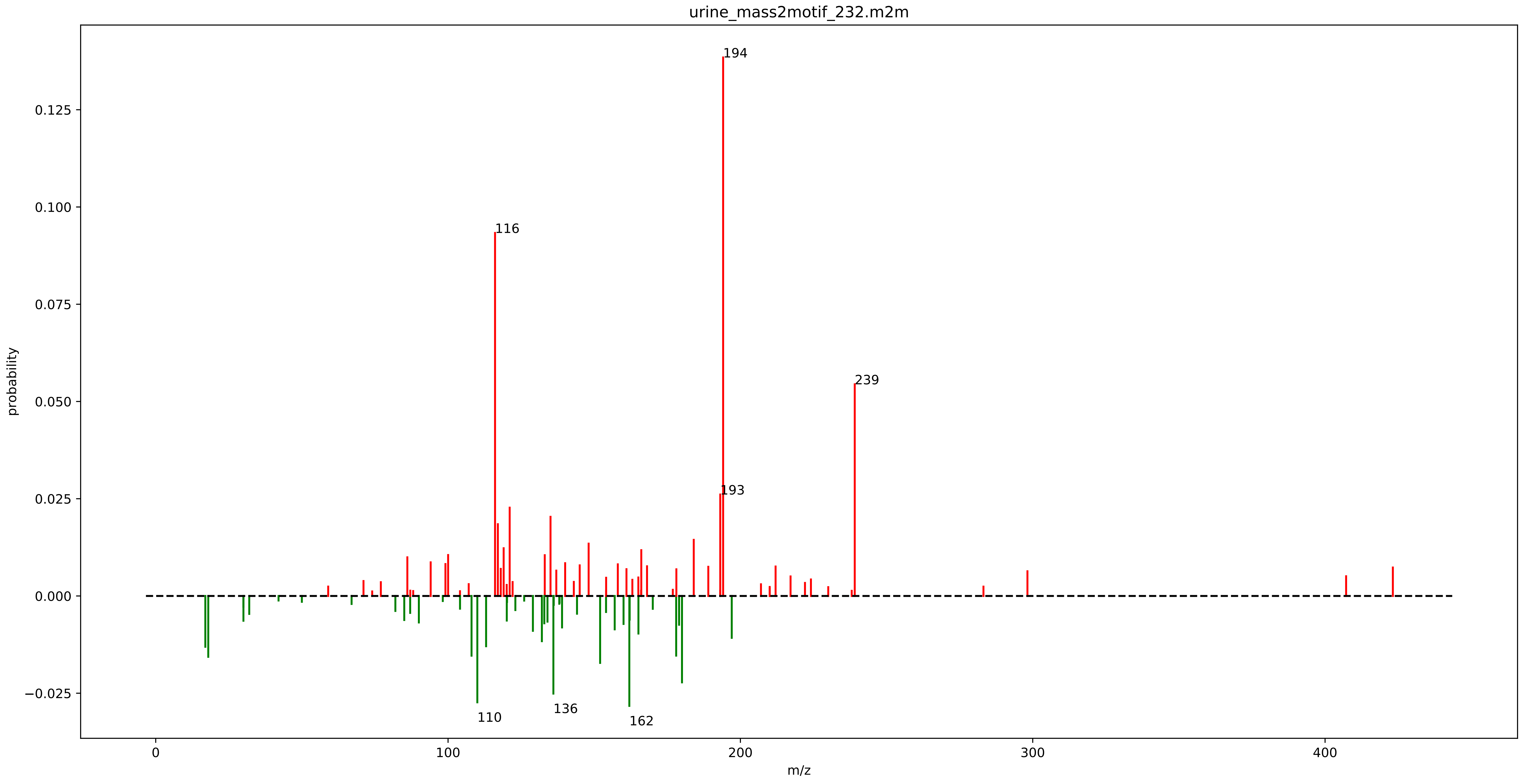

urine_mass2motif_232.m2m
probability
m/z
194
116
239
193
110
136
162
0.125
0.100
0.075
0.050
0.025
0.000
−0.025
0
100
200
300
400

urine_mass2motif_232.m2m

fragment_194.0925	: 0.14
fragment_116.0725	: 0.09
fragment_239.1125	: 0.05
loss_162.0075	: 0.03
loss_110.0125	: 0.03
fragment_193.1075	: 0.03
loss_136.0275	: 0.03
fragment_121.0775	: 0.02
loss_180.0225	: 0.02
fragment_135.0475	: 0.02
fragment_117.0375	: 0.02
loss_152.0175	: 0.02
loss_17.9725	: 0.02
loss_108.0325	: 0.02
loss_178.0325	: 0.02
fragment_184.0425	: 0.01
fragment_148.0875	: 0.01
loss_16.9675	: 0.01
loss_113.0125	: 0.01
fragment_119.0175	: 0.01
fragment_166.0975	: 0.01
loss_132.0925	: 0.01
loss_197.0375	: 0.01
fragment_100.0225	: 0.01
fragment_133.0775	: 0.01
fragment_86.0725	: 0.01
loss_165.1175	: 0.01
loss_129.0225	: 0.01
fragment_94.0525	: 0.01
loss_157.0025	: 0.01
fragment_140.0525	: 0.01
fragment_99.1025	: 0.01
fragment_158.0675	: 0.01
loss_138.9925	: 0.01
fragment_145.0325	: 0.01
fragment_168.0625	: 0.01
fragment_212.0375	: 0.01
fragment_189.0225	: 0.01
loss_179.0275	: 0.01
fragment_423.1875	: 0.01
loss_160.0275	: 0.01
loss_132.9275	: 0.01
fragment_118.0325	: 0.01
fragment_161.0275	: 0.01
fragment_178.0975	: 0.01
loss_90.0125	: 0.01
loss_134.0075	: 0.01
fragment_137.0275	: 0.01
loss_30.0025	: 0.01
fragment_298.1875	: 0.01
loss_120.0875	: 0.01
loss_85.0175	: 0.01
loss_162.1275	: 0.01
fragment_407.1975	: 0.01
fragment_217.1575	: 0.01

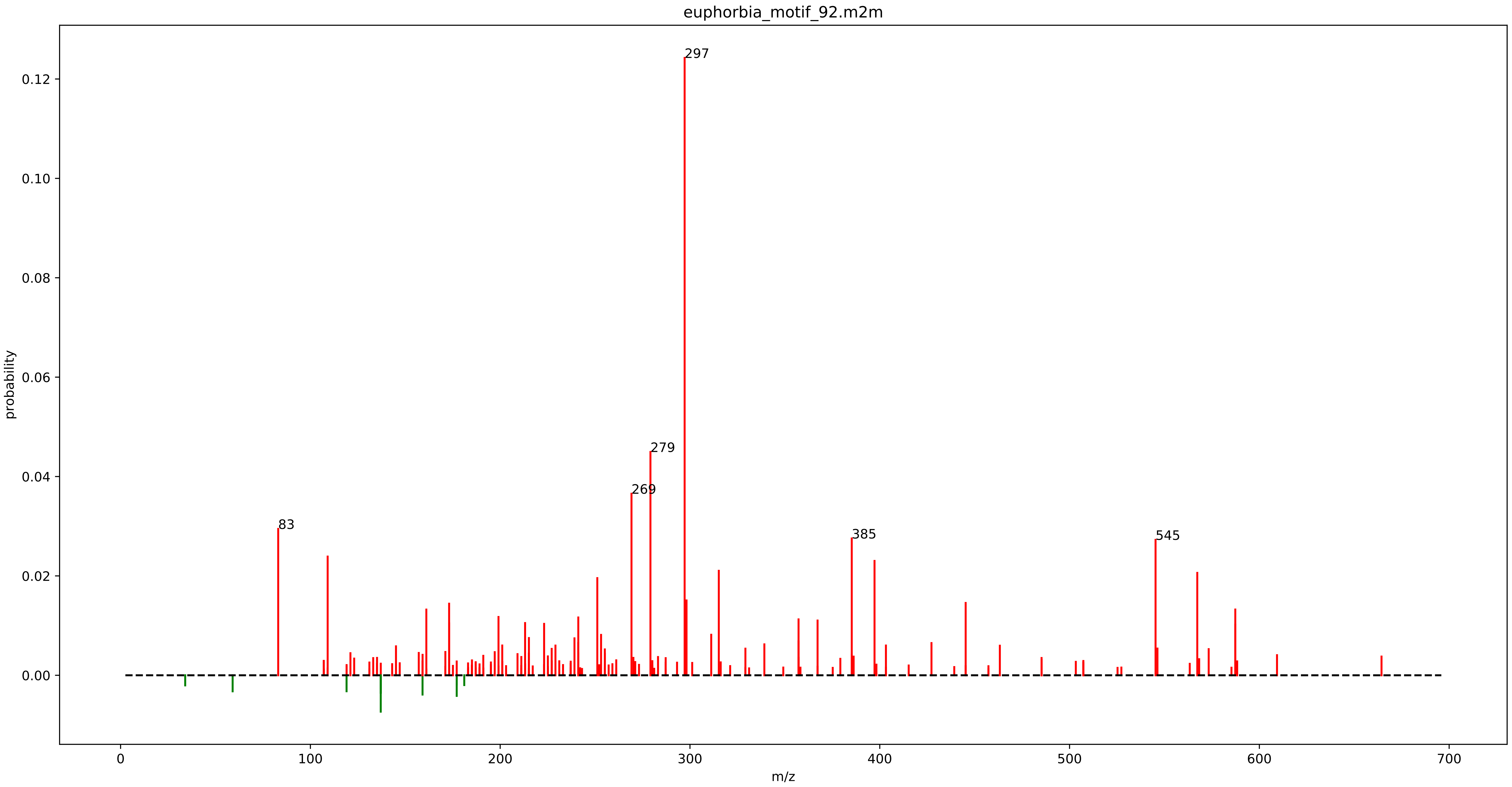

euphorbia_motif_92.m2m
probability
m/z
0.12
0.10
0.08
0.06
0.04
0.02
0.00
0
100
200
300
400
500
600
700
297
279
269
83
385
545

euphorbia_motif_92.m2m

fragment_297.1825	: 0.12
fragment_279.1725	: 0.04
fragment_269.1875	: 0.04
fragment_83.0475	: 0.03
fragment_385.2375	: 0.03
fragment_545.3075	: 0.03
fragment_109.1025	: 0.02
fragment_397.2375	: 0.02
fragment_315.1925	: 0.02
fragment_567.2925	: 0.02
fragment_251.1775	: 0.02
fragment_298.1875	: 0.02
fragment_445.2575	: 0.01
fragment_173.0975	: 0.01
fragment_587.3225	: 0.01
fragment_161.0975	: 0.01
fragment_199.1125	: 0.01
fragment_241.1575	: 0.01
fragment_357.2075	: 0.01
fragment_367.2275	: 0.01
fragment_213.1275	: 0.01
fragment_173.1325	: 0.01
fragment_223.1475	: 0.01
fragment_587.3175	: 0.01
fragment_311.1975	: 0.01
fragment_253.1575	: 0.01
fragment_215.1425	: 0.01
fragment_239.1425	: 0.01
loss_137.0725	: 0.01
fragment_357.2025	: 0.01
fragment_241.1225	: 0.01
fragment_427.2475	: 0.01
fragment_339.1925	: 0.01
fragment_201.0925	: 0.01
fragment_403.2475	: 0.01
fragment_229.1225	: 0.01
fragment_463.2675	: 0.01
fragment_145.1025	: 0.01
fragment_546.3125	: 0.01
fragment_329.2075	: 0.01
fragment_227.1425	: 0.01
fragment_573.3025	: 0.01
fragment_255.1375	: 0.01

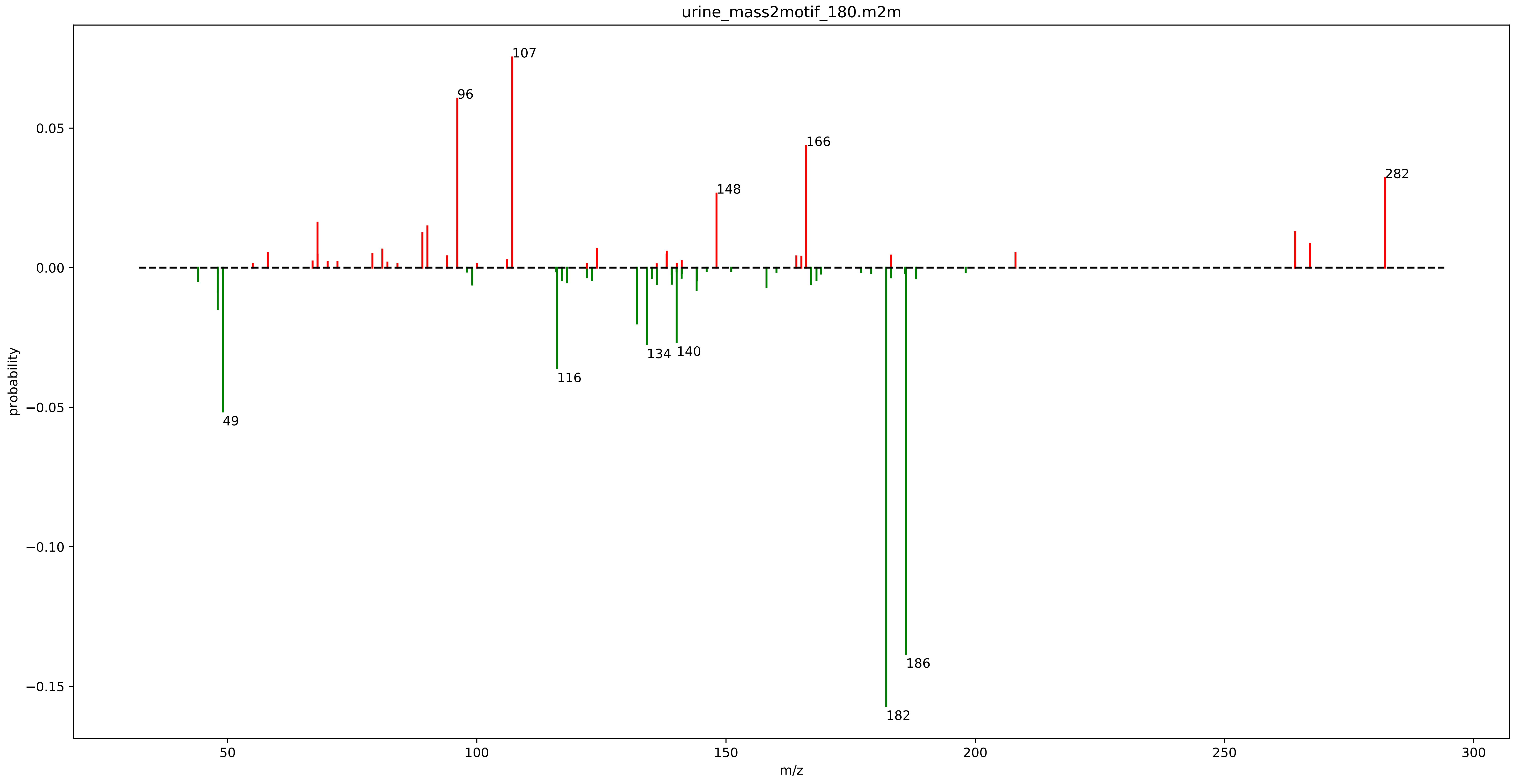

urine_mass2motif_180.m2m
probability
m/z
0.05
0.00
−0.05
−0.10
−0.15
50
100
150
200
250
300
107
96
166
282
148
49
116
134
140
186
182

urine_mass2motif_180.m2m

loss_182.1175	: 0.16
loss_186.1025	: 0.14
fragment_107.0875	: 0.08
fragment_96.0825	: 0.06
loss_49.0225	: 0.05
fragment_166.0975	: 0.04
loss_116.0975	: 0.04
fragment_282.1925	: 0.03
loss_134.1075	: 0.03
loss_140.0975	: 0.03
fragment_148.0875	: 0.03
loss_132.0925	: 0.02
fragment_68.0475	: 0.02
loss_48.0275	: 0.01
fragment_90.0875	: 0.01
fragment_96.0875	: 0.01
fragment_264.1825	: 0.01
fragment_89.0775	: 0.01
loss_48.0175	: 0.01
fragment_267.1375	: 0.01
loss_144.0975	: 0.01
loss_158.1175	: 0.01
fragment_124.0825	: 0.01
fragment_81.0575	: 0.01
loss_99.0675	: 0.01
loss_167.0675	: 0.01
loss_136.1175	: 0.01
fragment_138.1025	: 0.01
loss_139.0975	: 0.01
loss_118.0925	: 0.01
fragment_208.0725	: 0.01
fragment_58.0675	: 0.01

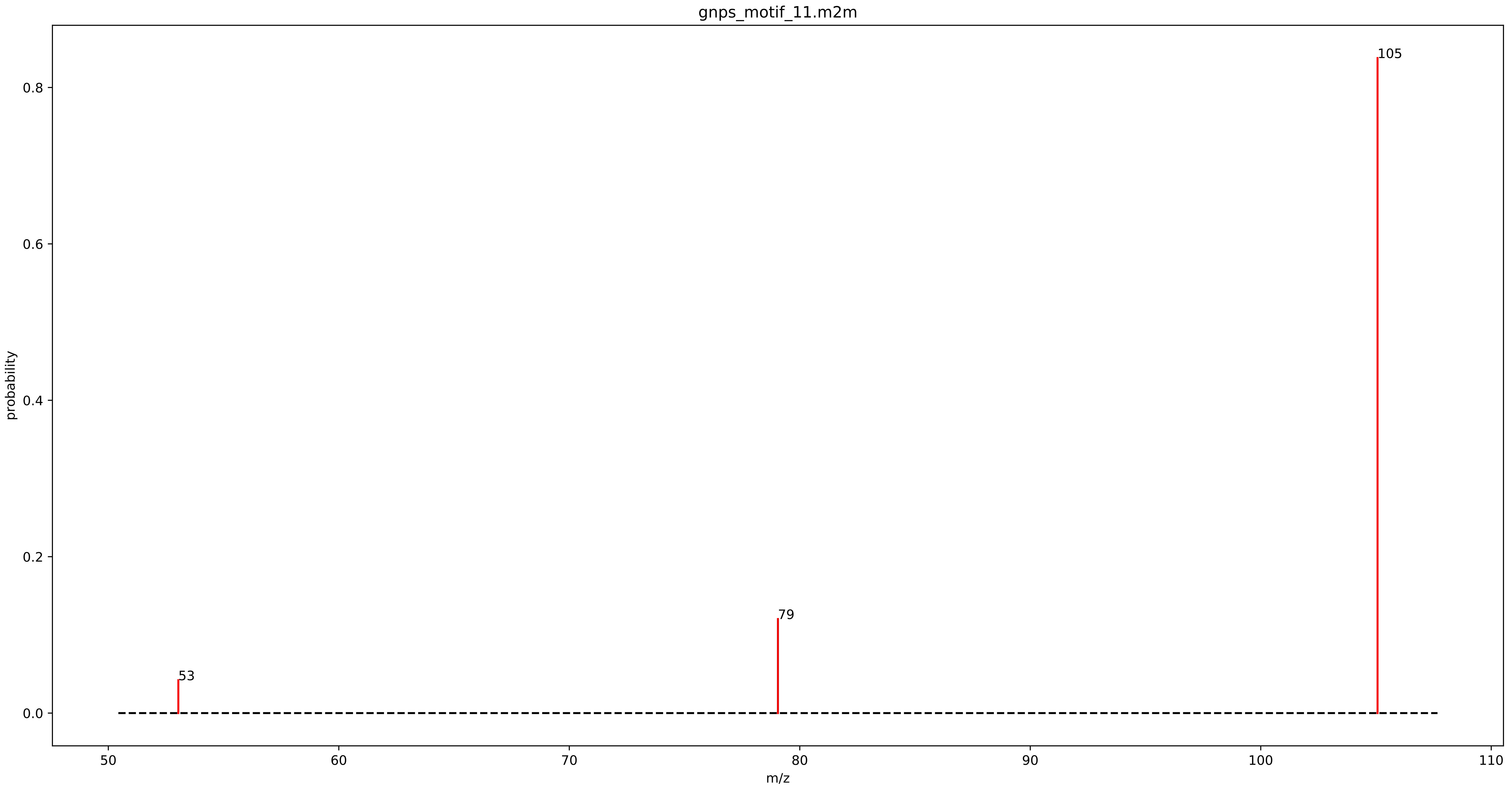
gnps_motif_11.m2m
105
79
53
probability
m/z
0.0
0.2
0.4
0.6
0.8
50
60
70
80
90
100
110

gnps_motif_11.m2m

fragment_105.0675	: 0.84
fragment_79.0525	: 0.12
fragment_53.0375	: 0.04

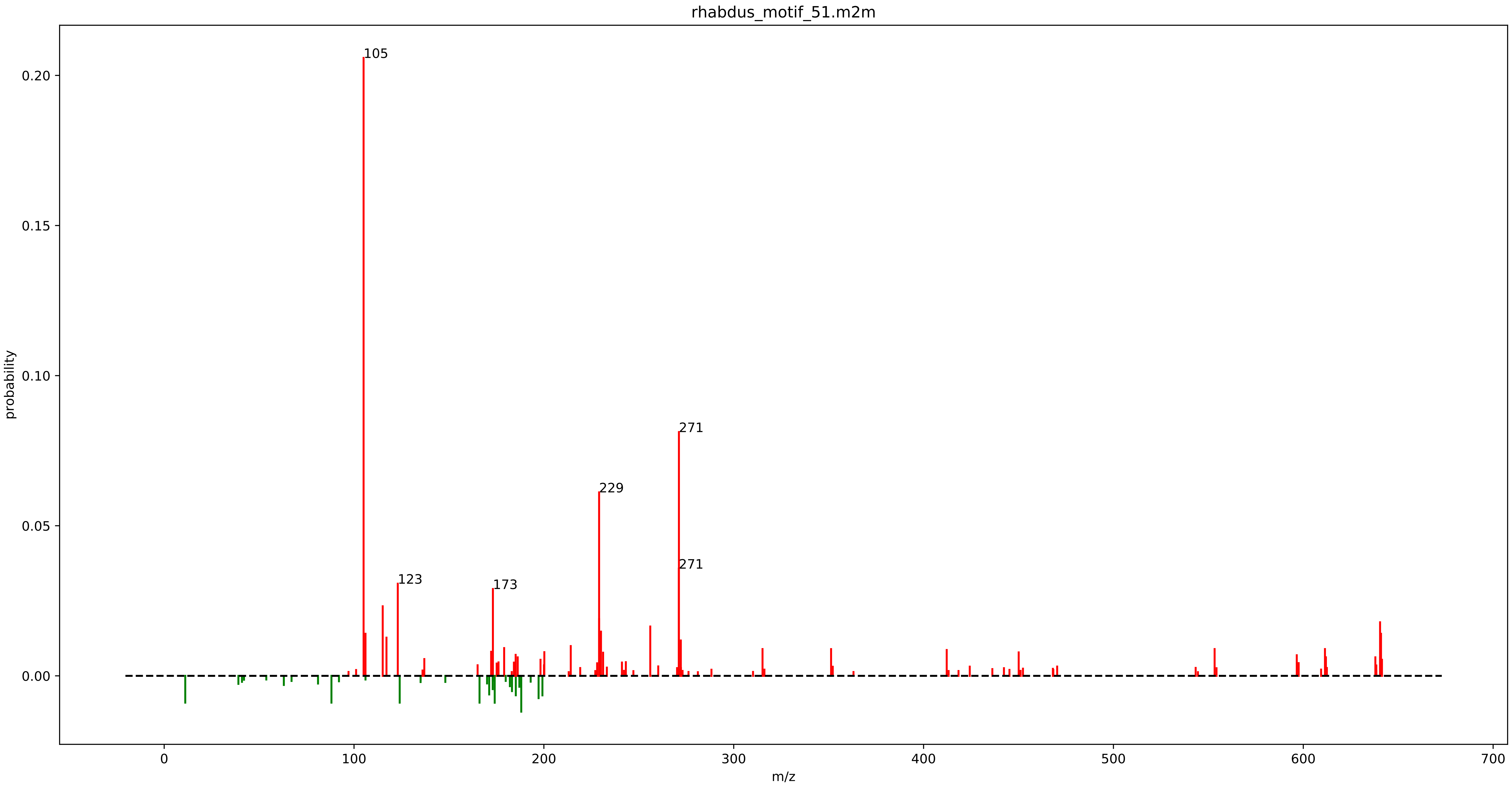
rhabdus_motif_51.m2m
probability
m/z
105
123
173
229
271
271
0.20
0.15
0.10
0.05
0.00
0
100
200
300
400
500
600
700

rhabdus_motif_51.m2m

fragment_105.0325	: 0.21
fragment_271.1325	: 0.08
fragment_229.0875	: 0.06
fragment_271.0575	: 0.04
fragment_123.0425	: 0.03
fragment_173.1525	: 0.03
fragment_115.1075	: 0.02
fragment_229.0975	: 0.02
fragment_640.4475	: 0.02
fragment_256.0375	: 0.02
fragment_230.0875	: 0.01
fragment_106.0375	: 0.01
fragment_640.9475	: 0.01
fragment_117.0875	: 0.01
loss_188.0825	: 0.01
fragment_272.1375	: 0.01
fragment_214.1525	: 0.01
fragment_179.0725	: 0.01
loss_174.0975	: 0.01
fragment_229.1625	: 0.01
loss_166.1025	: 0.01
loss_11.1025	: 0.01
loss_124.0525	: 0.01
fragment_553.2975	: 0.01
fragment_611.4225	: 0.01
fragment_315.1675	: 0.01
loss_88.1025	: 0.01
fragment_351.2775	: 0.01
fragment_412.1875	: 0.01
fragment_172.2075	: 0.01
fragment_200.2375	: 0.01
fragment_450.0975	: 0.01
fragment_231.1975	: 0.01
loss_197.2125	: 0.01
fragment_185.1325	: 0.01
fragment_596.5725	: 0.01
loss_199.2325	: 0.01
loss_185.2125	: 0.01
loss_171.1975	: 0.01
fragment_611.9275	: 0.01
fragment_637.9675	: 0.01
fragment_186.2225	: 0.01
fragment_640.4525	: 0.01
fragment_137.0225	: 0.01
fragment_641.4475	: 0.01
fragment_198.2225	: 0.01
loss_183.1975	: 0.01

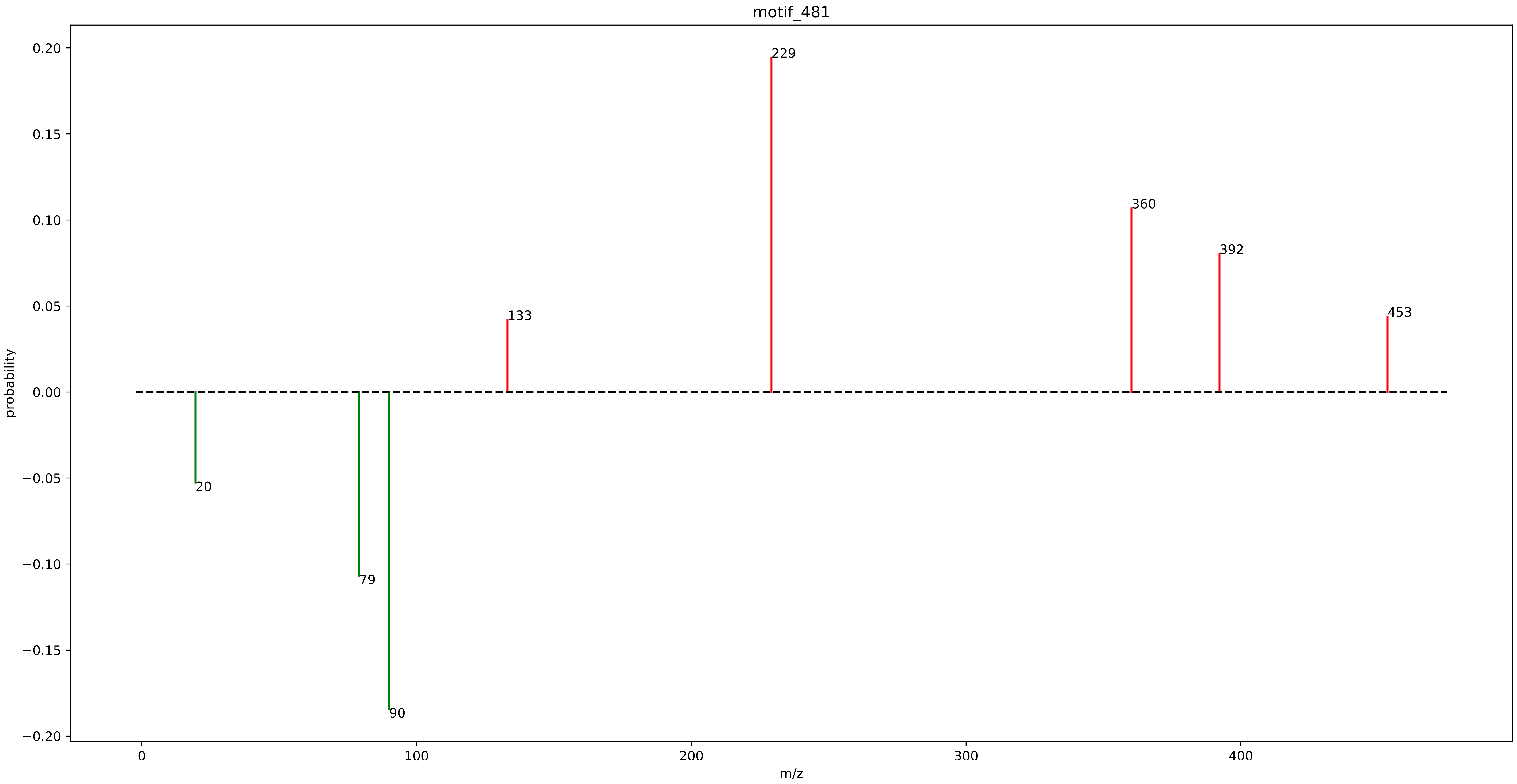
motif_481
probability
m/z
0.20
0.15
0.10
0.05
0.00
−0.05
−0.10
−0.15
−0.20
0
100
200
300
400
229
360
392
453
133
20
79
90

motif_481

fragment_229.1375	: 0.19
loss_90.0275	: 0.18
fragment_360.2025	: 0.11
loss_79.1525	: 0.11
fragment_392.2125	: 0.08
loss_19.5225	: 0.05
fragment_453.3325	: 0.04
fragment_133.1025	: 0.04

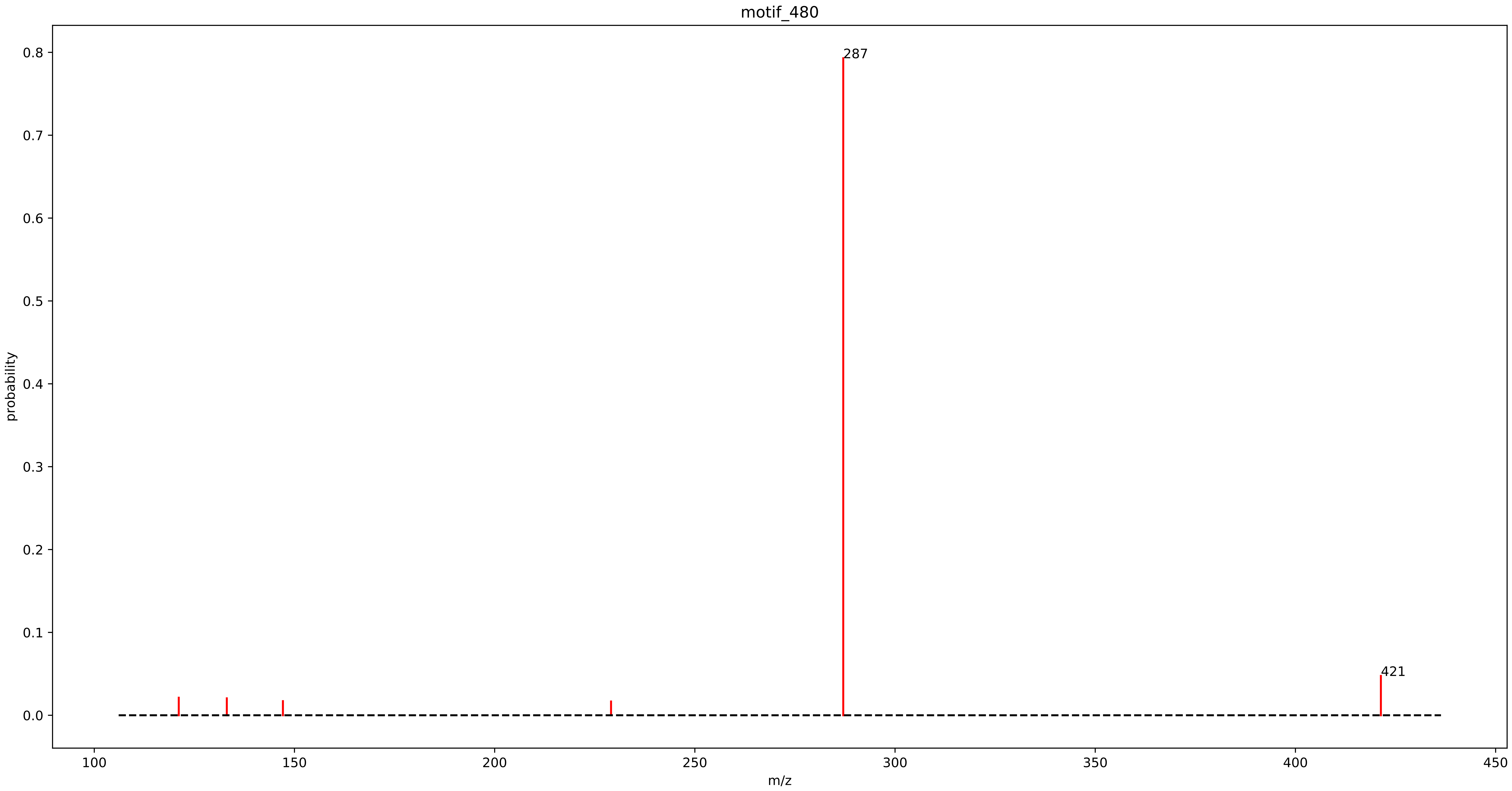

motif_480
287
421
probability
m/z
0.0
0.1
0.2
0.3
0.4
0.5
0.6
0.7
0.8
100
150
200
250
300
350
400
450

motif_480

fragment_287.0625	: 0.79
fragment_421.3425	: 0.05
fragment_121.1025	: 0.02
fragment_133.1025	: 0.02
fragment_147.1175	: 0.02
fragment_229.0675	: 0.02

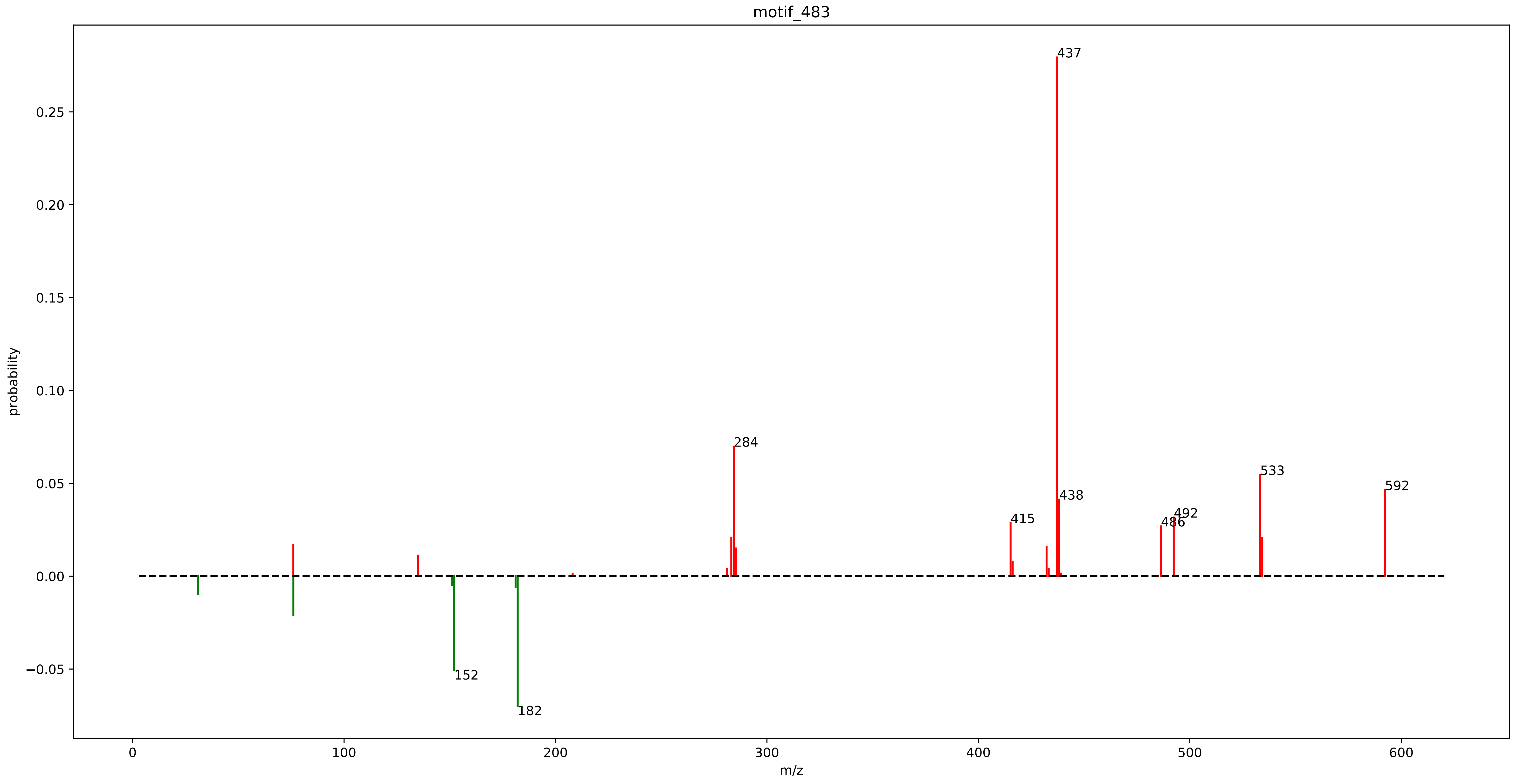
motif_483
probability
m/z
0.25
0.20
0.15
0.10
0.05
0.00
−0.05
0
100
200
300
400
500
600
437
284
533
438
592
415
492
486
152
182

motif_483

fragment_437.1925	: 0.28
loss_182.1125	: 0.07
fragment_284.2925	: 0.07
fragment_533.2525	: 0.05
loss_152.1025	: 0.05
fragment_592.2675	: 0.05
fragment_438.1975	: 0.04
fragment_492.3625	: 0.03
fragment_415.2125	: 0.03
fragment_486.3175	: 0.03
fragment_283.1475	: 0.02
loss_76.0525	: 0.02
fragment_534.2575	: 0.02
fragment_438.2025	: 0.02
fragment_76.0375	: 0.02
fragment_432.2375	: 0.02
fragment_285.2975	: 0.01
fragment_135.0825	: 0.01
loss_31.0225	: 0.01
fragment_416.2125	: 0.01
loss_181.1125	: 0.01

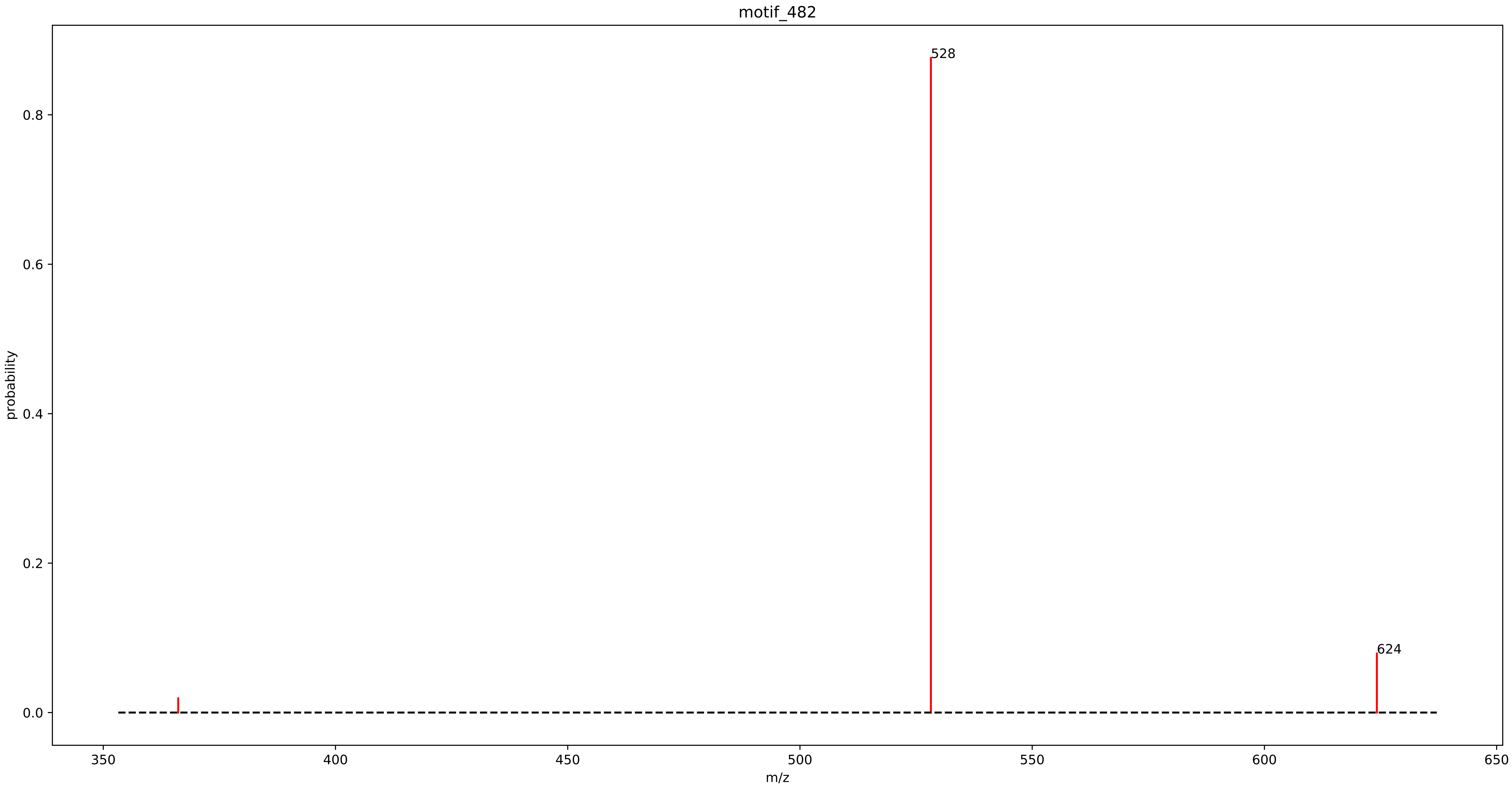

motif_482
528
624
0.0
0.2
0.4
0.6
0.8
probability
350
400
450
500
550
600
650
m/z

motif_482

fragment_528.1925	: 0.88
fragment_624.2125	: 0.08
fragment_366.1425	: 0.02

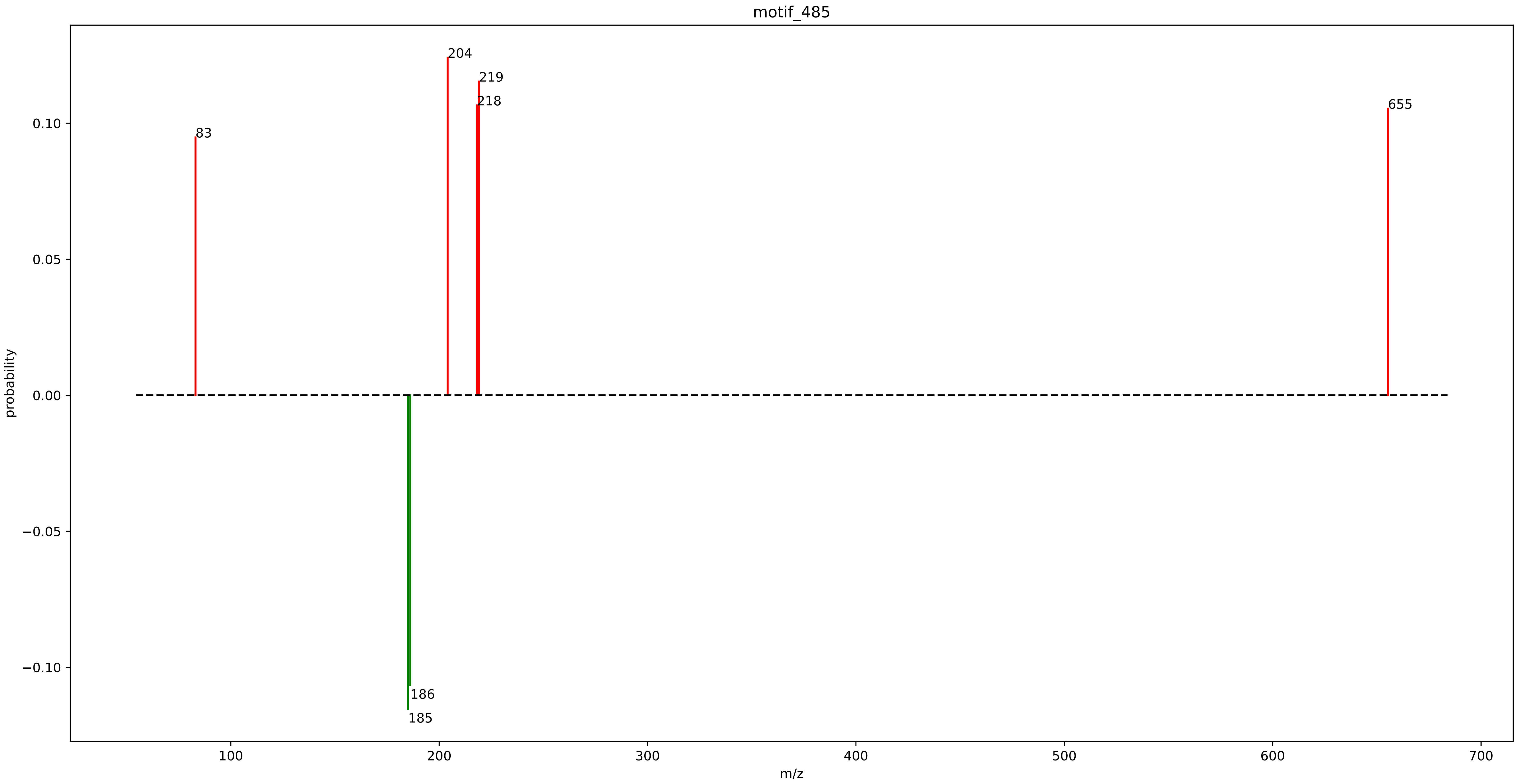

motif_485
probability
m/z
0.10
0.05
0.00
−0.05
−0.10
100
200
300
400
500
600
700
83
204
219
218
655
186
185

motif_485

fragment_204.0625	: 0.12
fragment_219.0875	: 0.12
loss_185.1175	: 0.12
fragment_218.0825	: 0.11
loss_186.1225	: 0.11
fragment_655.3125	: 0.11
fragment_83.0475	: 0.09

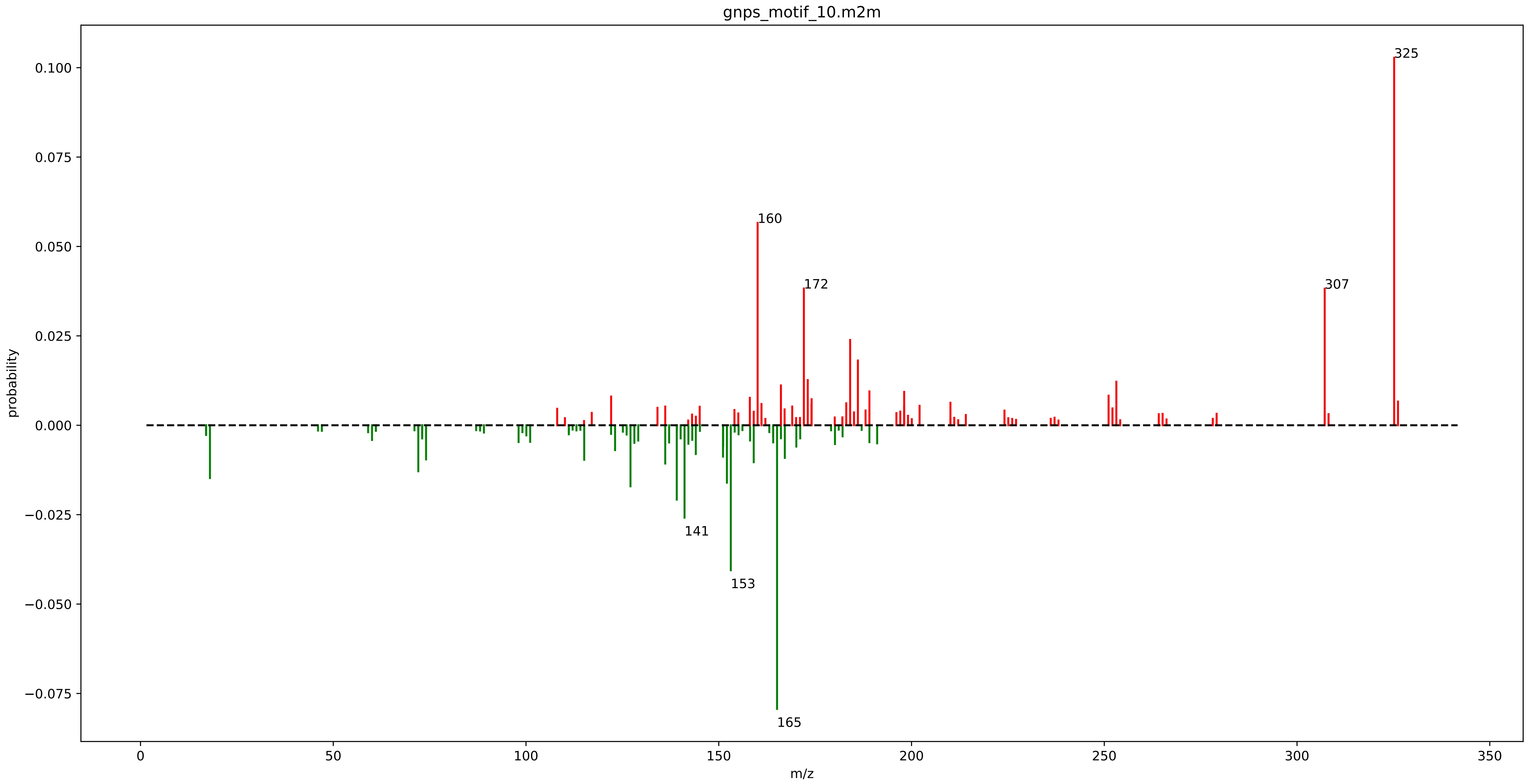

gnps_motif_10.m2m
probability
m/z
0.100
0.075
0.050
0.025
0.000
−0.025
−0.050
−0.075
0
50
100
150
200
250
300
350
325
160
172
307
141
153
165

gnps_motif_10.m2m

fragment_325.1925	: 0.10
loss_165.1175	: 0.08
fragment_160.0775	: 0.06
loss_153.1175	: 0.04
fragment_172.0775	: 0.04
fragment_307.1775	: 0.04
loss_141.1175	: 0.03
fragment_184.0775	: 0.02
loss_139.1025	: 0.02
fragment_186.0925	: 0.02
loss_127.0975	: 0.02
loss_152.1075	: 0.02
loss_18.0125	: 0.01
loss_72.0575	: 0.01
fragment_173.0825	: 0.01
fragment_253.1325	: 0.01
fragment_166.1225	: 0.01
loss_136.1125	: 0.01
loss_159.0675	: 0.01
loss_115.0975	: 0.01
loss_74.0725	: 0.01
fragment_189.0775	: 0.01
fragment_198.0925	: 0.01
loss_167.1325	: 0.01
loss_151.0975	: 0.01
fragment_251.1175	: 0.01
fragment_122.0775	: 0.01
loss_144.0425	: 0.01
fragment_158.0625	: 0.01
fragment_174.0925	: 0.01
loss_123.1025	: 0.01
fragment_326.1925	: 0.01
fragment_210.0925	: 0.01
fragment_183.0675	: 0.01
loss_170.1175	: 0.01
fragment_161.0825	: 0.01
fragment_174.0525	: 0.01
loss_151.1375	: 0.01
fragment_202.0875	: 0.01
loss_180.1375	: 0.01
fragment_169.0525	: 0.01
fragment_136.1125	: 0.01
fragment_145.0525	: 0.01
loss_142.1225	: 0.01
fragment_198.0875	: 0.01
loss_191.0925	: 0.01

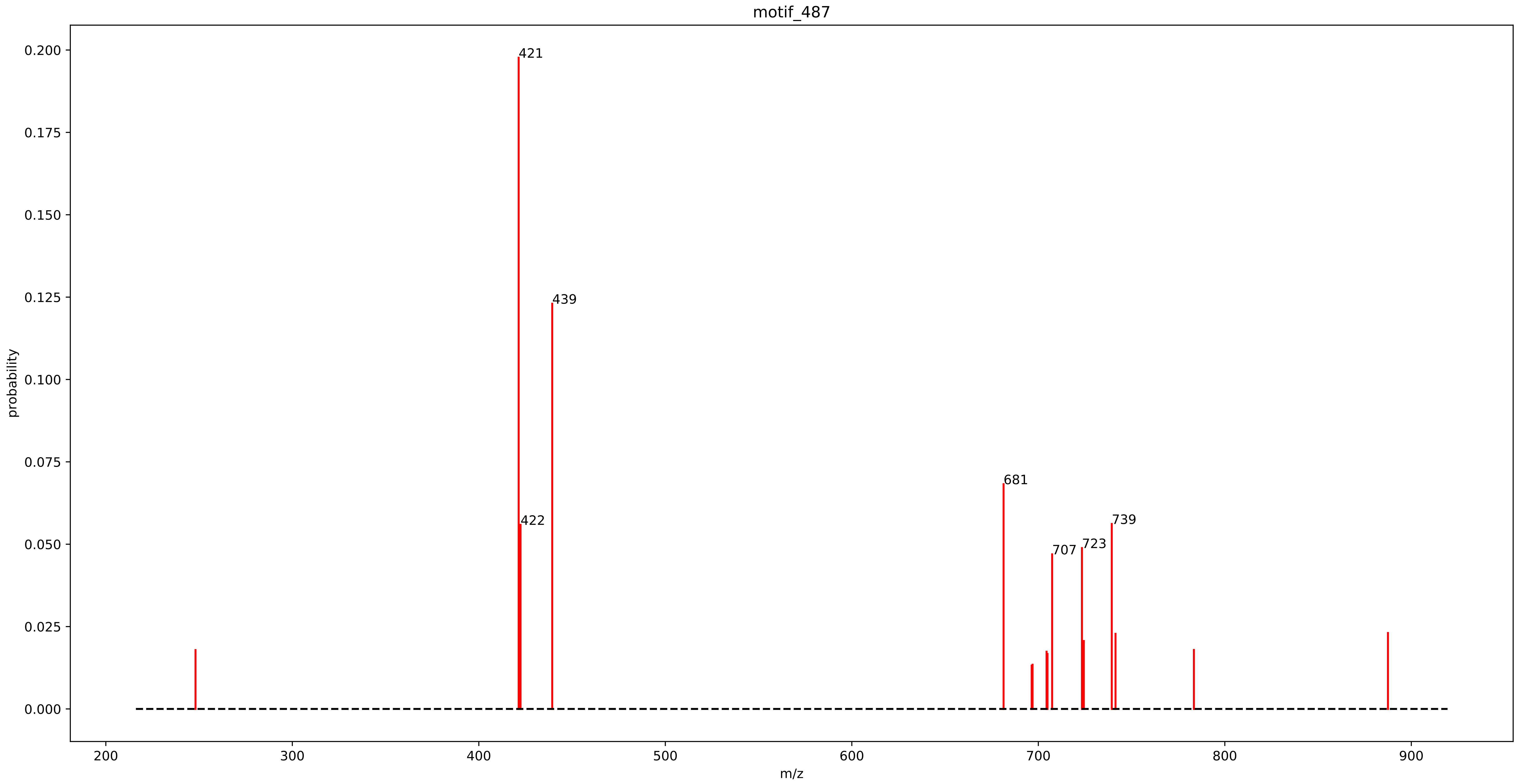

motif_487
421
439
422
681
739
723
707
0.200
0.175
0.150
0.125
0.100
0.075
0.050
0.025
0.000
probability
200
300
400
500
600
700
800
900
m/z

motif_487

fragment_421.3475	: 0.20
fragment_439.3575	: 0.12
fragment_681.3675	: 0.07
fragment_739.3775	: 0.06
fragment_422.3475	: 0.06
fragment_723.3775	: 0.05
fragment_707.4025	: 0.05
fragment_887.4675	: 0.02
fragment_741.3875	: 0.02
fragment_724.3825	: 0.02
fragment_783.4025	: 0.02
fragment_248.0825	: 0.02
fragment_704.4075	: 0.02
fragment_704.9075	: 0.02
fragment_696.9175	: 0.01
fragment_696.4175	: 0.01

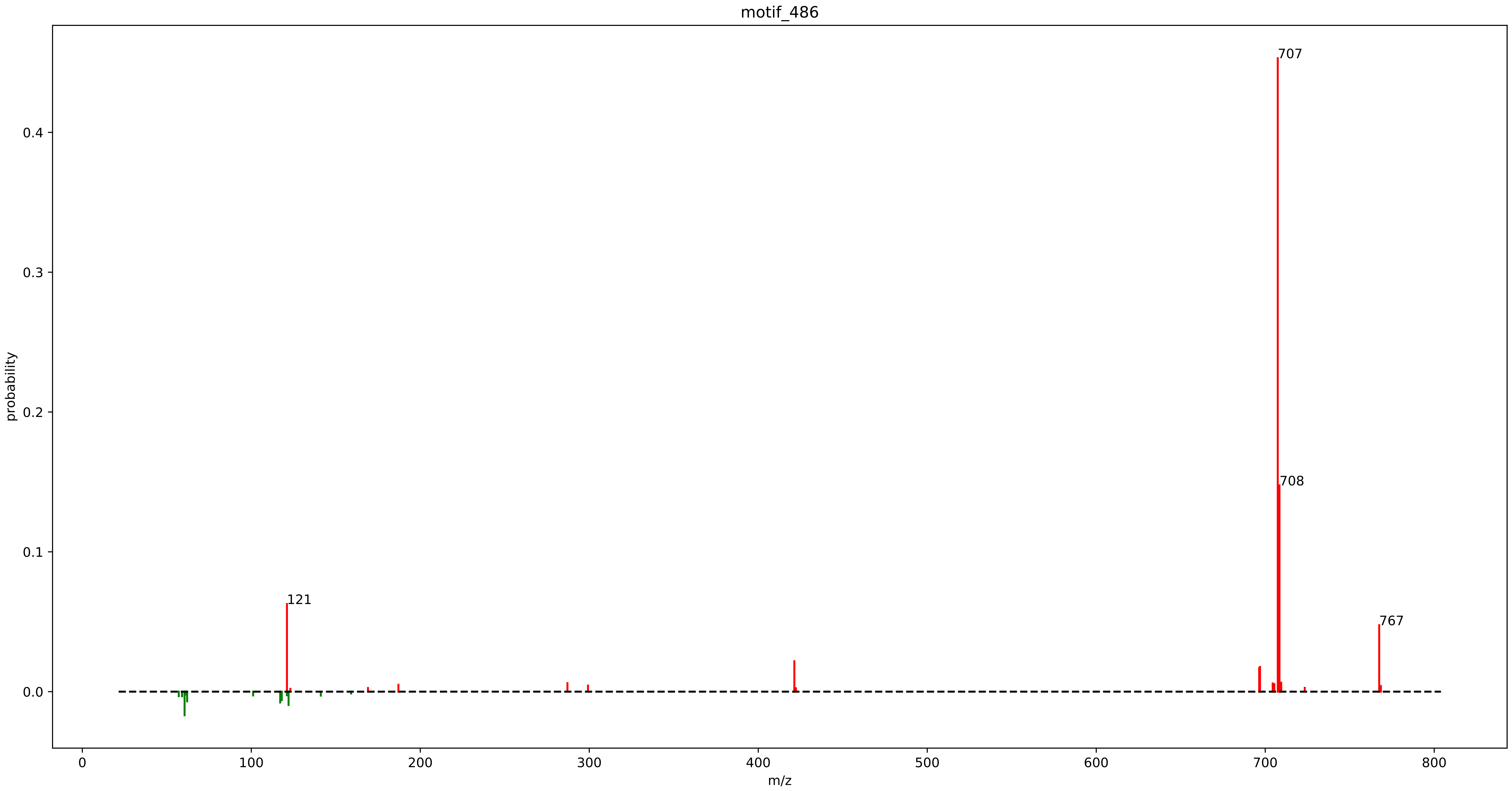

motif_486
707
708
121
767
0.4
0.3
0.2
0.1
0.0
probability
0
100
200
300
400
500
600
700
800
m/z

motif_486

fragment_707.4125	: 0.45
fragment_708.4175	: 0.15
fragment_121.1025	: 0.06
fragment_767.4325	: 0.05
fragment_421.3475	: 0.02
fragment_696.9175	: 0.02
loss_60.5175	: 0.02
fragment_696.4175	: 0.02
loss_122.0525	: 0.01
loss_117.0975	: 0.01
loss_62.0325	: 0.01
fragment_709.4225	: 0.01
fragment_287.0725	: 0.01
loss_118.0275	: 0.01
fragment_709.4175	: 0.01
fragment_704.4075	: 0.01
fragment_705.3975	: 0.01

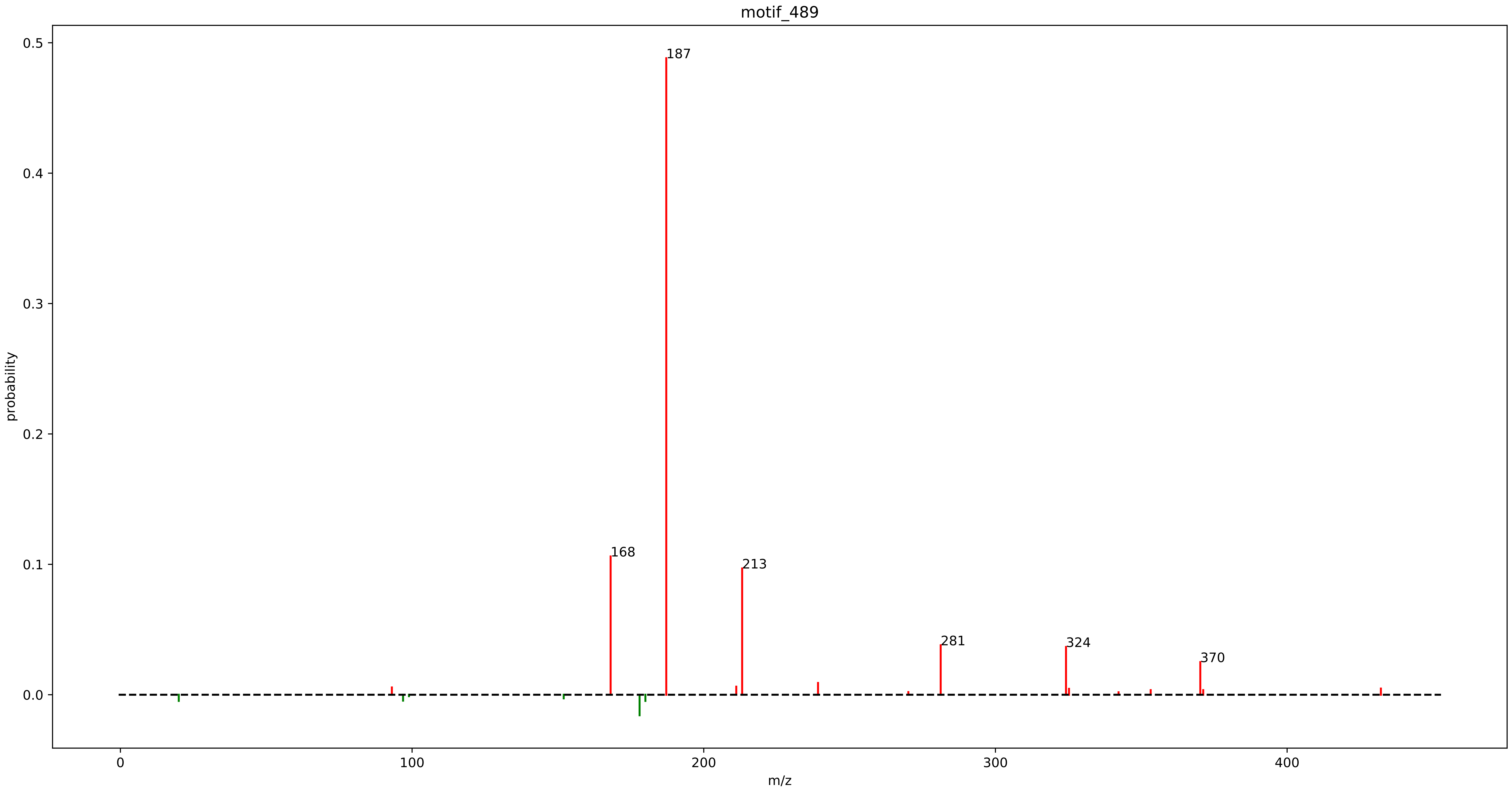

motif_489
probability
m/z
0.5
0.4
0.3
0.2
0.1
0.0
0
100
200
300
400
187
168
213
281
324
370

motif_489

fragment_187.1475	: 0.49
fragment_168.0675	: 0.11
fragment_213.1625	: 0.10
fragment_281.2275	: 0.04
fragment_324.1975	: 0.04
fragment_370.2375	: 0.03
loss_177.9975	: 0.02
fragment_239.1775	: 0.01
fragment_211.1475	: 0.01
fragment_93.0675	: 0.01

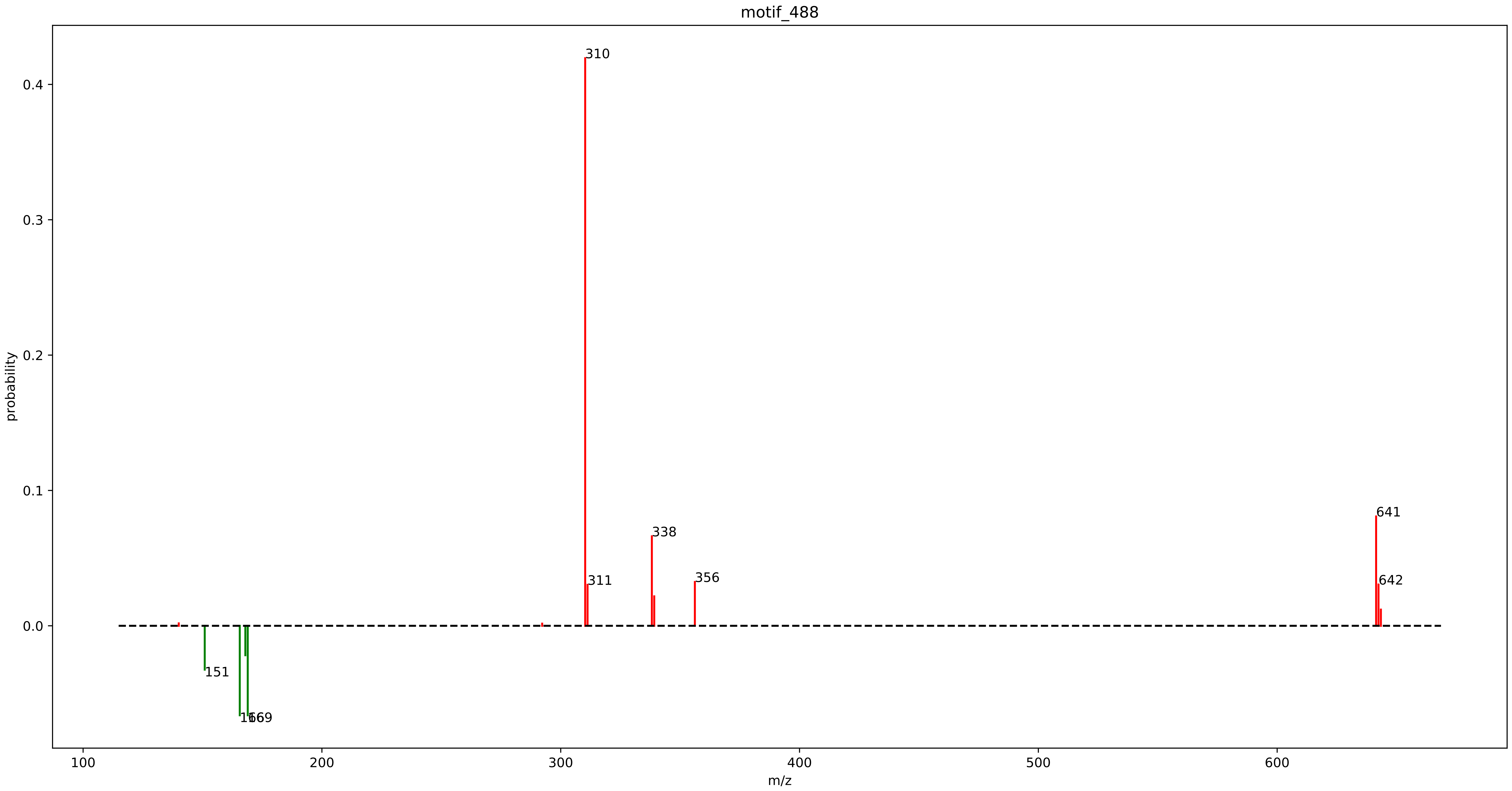

motif_488
310
311
338
356
641
642
151
169
probability
m/z

motif_488

fragment_310.2375	: 0.42
fragment_641.4375	: 0.08
fragment_338.1625	: 0.07
loss_165.5975	: 0.07
loss_168.9375	: 0.07
loss_150.9275	: 0.03
fragment_356.1725	: 0.03
fragment_642.4375	: 0.03
fragment_311.2375	: 0.03
fragment_339.1625	: 0.02
loss_167.9375	: 0.02
fragment_643.4375	: 0.01

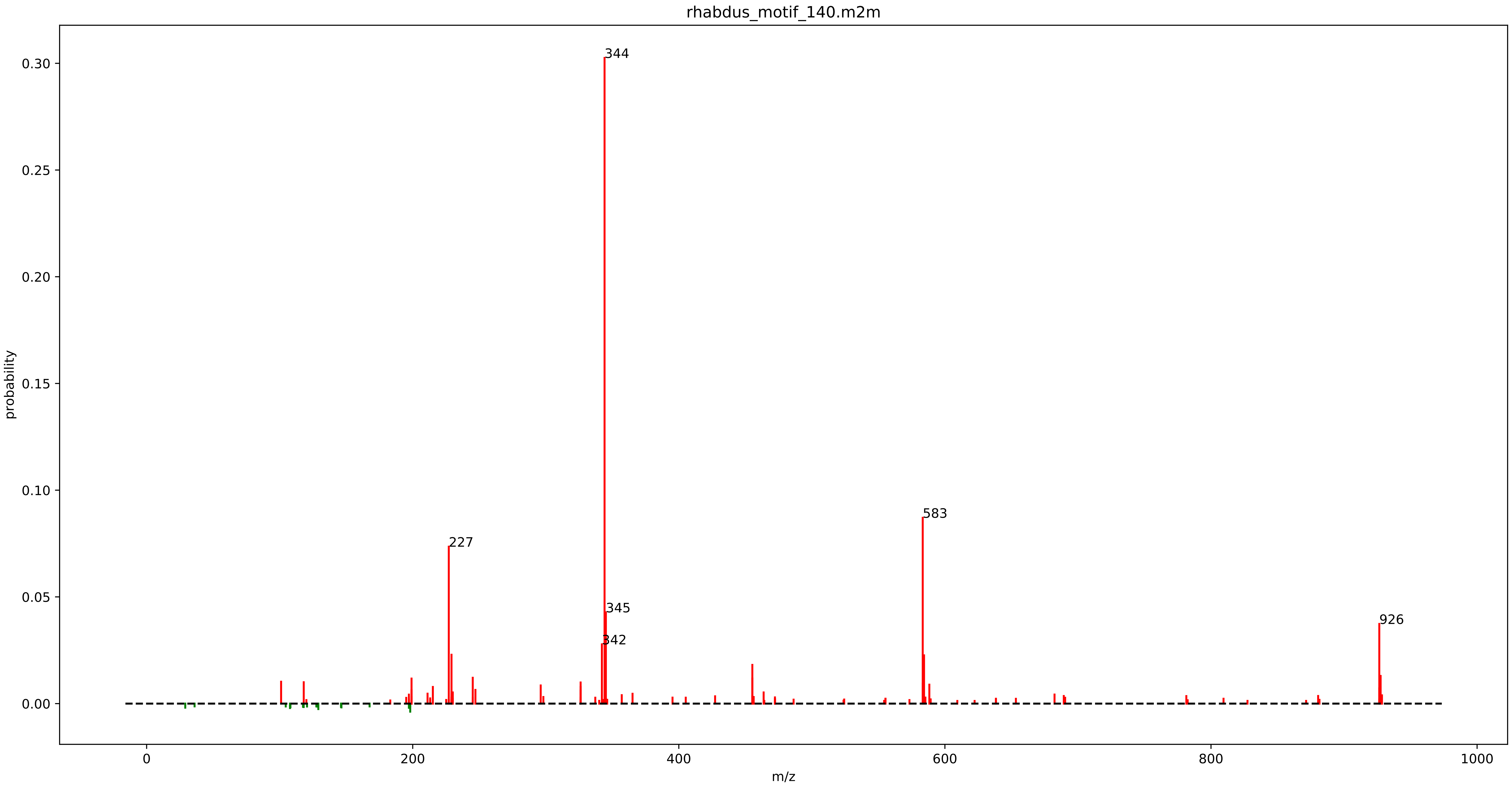
rhabdus_motif_140.m2m
probability
m/z
344
583
227
345
342
926
0.30
0.25
0.20
0.15
0.10
0.05
0.00
0
200
400
600
800
1000

rhabdus_motif_140.m2m

fragment_344.1825	: 0.30
fragment_583.3225	: 0.09
fragment_227.1025	: 0.07
fragment_345.1825	: 0.04
fragment_926.4975	: 0.04
fragment_342.1825	: 0.03
fragment_229.1475	: 0.02
fragment_584.3275	: 0.02
fragment_455.2625	: 0.02
fragment_927.4975	: 0.01
fragment_245.1175	: 0.01
fragment_455.2675	: 0.01
fragment_199.1075	: 0.01
fragment_101.0675	: 0.01
fragment_118.0825	: 0.01
fragment_326.1975	: 0.01
fragment_583.3275	: 0.01
fragment_588.3025	: 0.01
fragment_296.1975	: 0.01
fragment_215.1375	: 0.01
fragment_927.5025	: 0.01
fragment_326.2025	: 0.01
fragment_926.4925	: 0.01
fragment_247.1275	: 0.01
fragment_199.1125	: 0.01
fragment_326.1925	: 0.01
fragment_229.1375	: 0.01
fragment_230.1175	: 0.01
fragment_463.7525	: 0.01

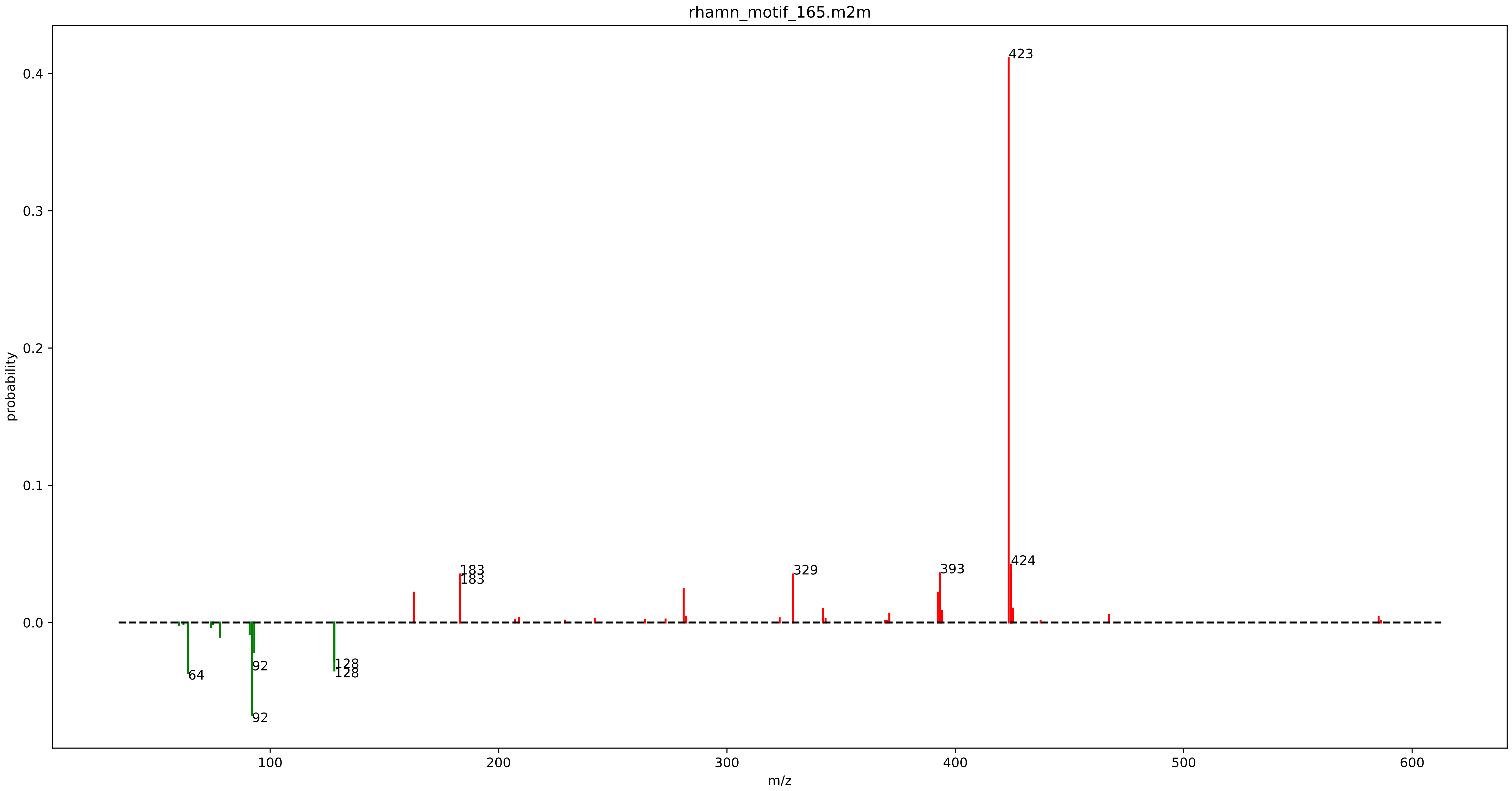

rhamn_motif_165.m2m
probability
m/z
0.4
0.3
0.2
0.1
0.0
100
200
300
400
500
600
423
424
393
329
183
183
64
92
92
128
128

rhamn_motif_165.m2m

fragment_423.3275	: 0.41
loss_92.0475	: 0.07
fragment_424.3275	: 0.04
loss_64.0475	: 0.04
fragment_393.2775	: 0.04
fragment_329.0375	: 0.03
fragment_183.0975	: 0.03
loss_128.1225	: 0.03
loss_92.0425	: 0.03
fragment_183.1075	: 0.03
loss_128.1175	: 0.03
fragment_281.0675	: 0.02
fragment_392.2675	: 0.02
loss_93.0575	: 0.02
fragment_163.0075	: 0.02
fragment_424.3375	: 0.02
loss_78.0325	: 0.01
fragment_425.3325	: 0.01
fragment_342.1825	: 0.01
loss_91.0475	: 0.01
fragment_394.2825	: 0.01
fragment_371.1025	: 0.01
fragment_467.3125	: 0.01

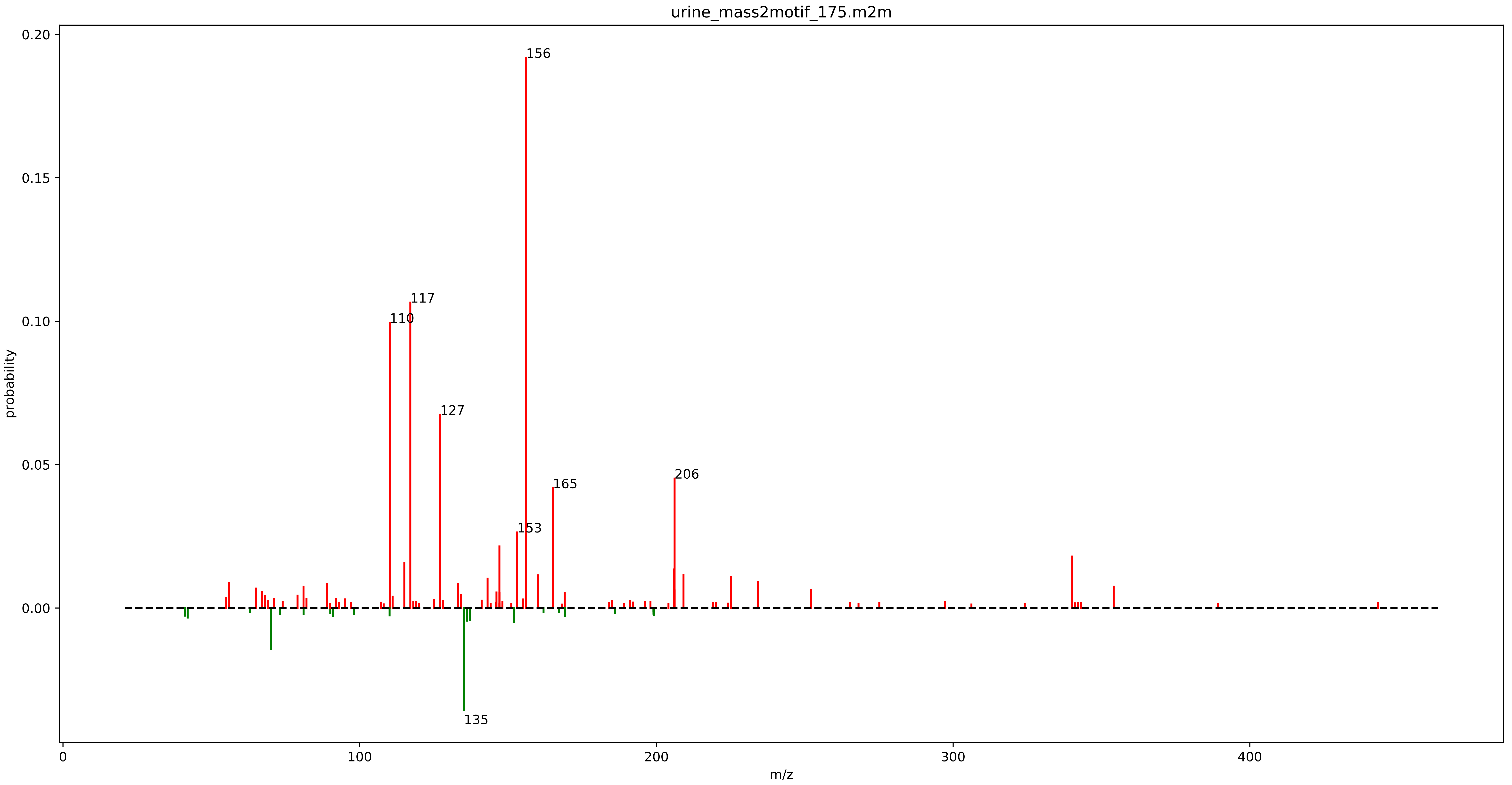
urine_mass2motif_175.m2m
probability
m/z
0.20
0.15
0.10
0.05
0.00
0
100
200
300
400
156
117
110
127
206
165
153
135

urine_mass2motif_175.m2m

fragment_156.1025 : 0.19
fragment_117.0675 : 0.11
fragment_110.0975 : 0.10
fragment_127.1225 : 0.07
fragment_206.1175 : 0.05
fragment_165.1025 : 0.04
loss_135.1175 : 0.04
fragment_153.1025 : 0.03
fragment_147.0925 : 0.02
fragment_340.1325 : 0.02
fragment_115.0525 : 0.02
loss_70.0475 : 0.01
fragment_206.0025 : 0.01
fragment_209.1275 : 0.01
fragment_160.1125 : 0.01
fragment_225.1225 : 0.01
fragment_143.0925 : 0.01
fragment_234.1475 : 0.01
fragment_56.0475 : 0.01
fragment_89.0375 : 0.01
fragment_133.0775 : 0.01
fragment_354.1125 : 0.01
fragment_81.0675 : 0.01
fragment_65.0375 : 0.01
fragment_252.1375 : 0.01
fragment_206.1125 : 0.01
fragment_67.0525 : 0.01
fragment_146.0975 : 0.01
fragment_169.0975 : 0.01
fragment_147.1125 : 0.01

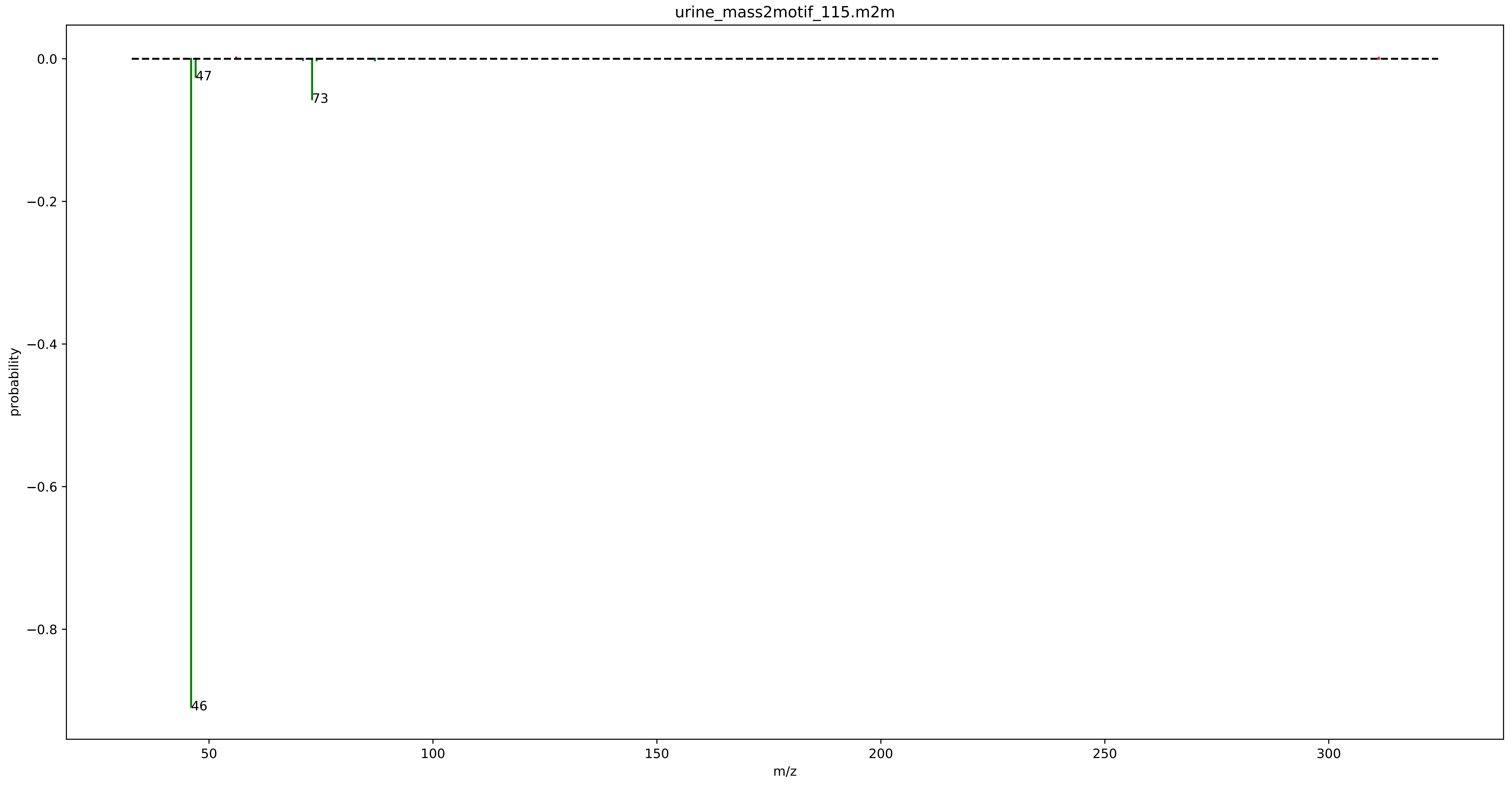
urine_mass2motif_115.m2m
probability
m/z
0.0
−0.2
−0.4
−0.6
−0.8
50
100
150
200
250
300
47
73
46

urine_mass2motif_115.m2m

loss_46.0025	: 0.91
loss_73.0175	: 0.06
loss_47.0075	: 0.03

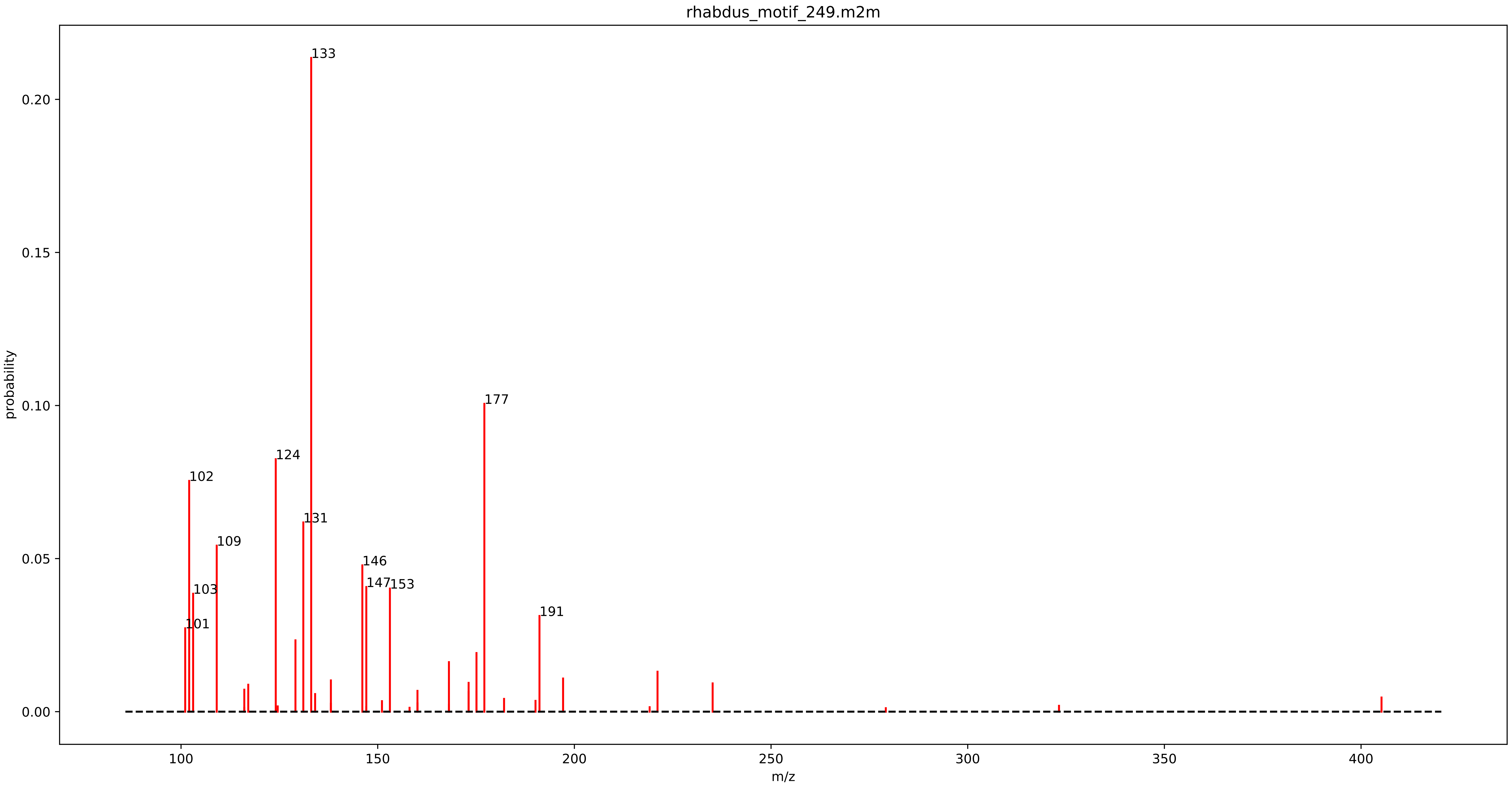
rhabdus_motif_249.m2m
probability
m/z
0.00
0.05
0.10
0.15
0.20
100
150
200
250
300
350
400
133
177
124
102
131
109
146
147
153
103
101
191

rhabdus_motif_249.m2m

fragment_133.0875	: 0.21
fragment_177.1125	: 0.10
fragment_124.0775	: 0.08
fragment_102.0675	: 0.08
fragment_131.0875	: 0.06
fragment_109.0725	: 0.05
fragment_146.0925	: 0.05
fragment_147.1025	: 0.04
fragment_153.1025	: 0.04
fragment_103.0725	: 0.04
fragment_191.1275	: 0.03
fragment_101.0575	: 0.03
fragment_129.0875	: 0.02
fragment_175.1125	: 0.02
fragment_168.1075	: 0.02
fragment_221.1375	: 0.01
fragment_197.1275	: 0.01
fragment_138.0975	: 0.01
fragment_173.1125	: 0.01
fragment_235.1525	: 0.01
fragment_117.0875	: 0.01
fragment_116.0825	: 0.01
fragment_160.1075	: 0.01
fragment_175.1075	: 0.01
fragment_173.1075	: 0.01
fragment_134.0875	: 0.01

urine_mass2motif_64.m2m

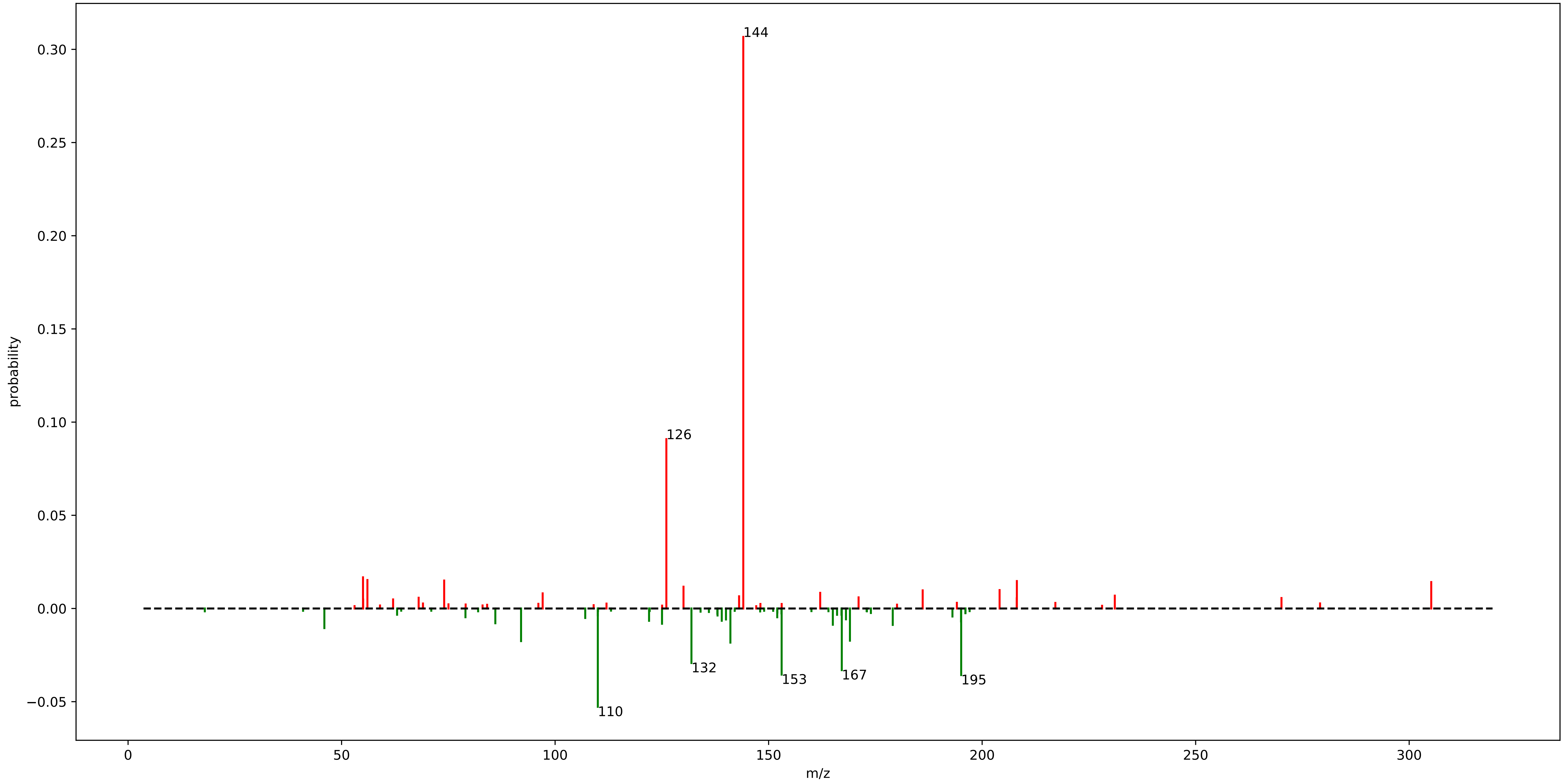

urine_mass2motif_64.m2m

fragment_144.0675	: 0.31
fragment_126.0525	: 0.09
loss_110.0025	: 0.05
loss_195.0925	: 0.04
loss_153.0475	: 0.04
loss_167.1325	: 0.03
loss_131.9275	: 0.03
loss_141.0475	: 0.02
loss_92.0275	: 0.02
loss_169.0425	: 0.02
fragment_55.0175	: 0.02
fragment_56.0475	: 0.02
fragment_74.0225	: 0.01
fragment_208.1325	: 0.01
fragment_305.1675	: 0.01
fragment_130.0675	: 0.01
loss_45.9675	: 0.01
fragment_204.0875	: 0.01
fragment_186.0775	: 0.01
loss_179.0675	: 0.01
loss_165.0375	: 0.01
fragment_162.0775	: 0.01
loss_125.0375	: 0.01
fragment_97.0875	: 0.01
loss_86.0025	: 0.01
loss_195.0575	: 0.01
fragment_231.0675	: 0.01
loss_122.0025	: 0.01
loss_139.0275	: 0.01
fragment_143.0825	: 0.01
fragment_171.0675	: 0.01
loss_140.0125	: 0.01
loss_168.1125	: 0.01
fragment_68.0475	: 0.01
fragment_270.0975	: 0.01
fragment_208.0975	: 0.01
loss_107.0575	: 0.01

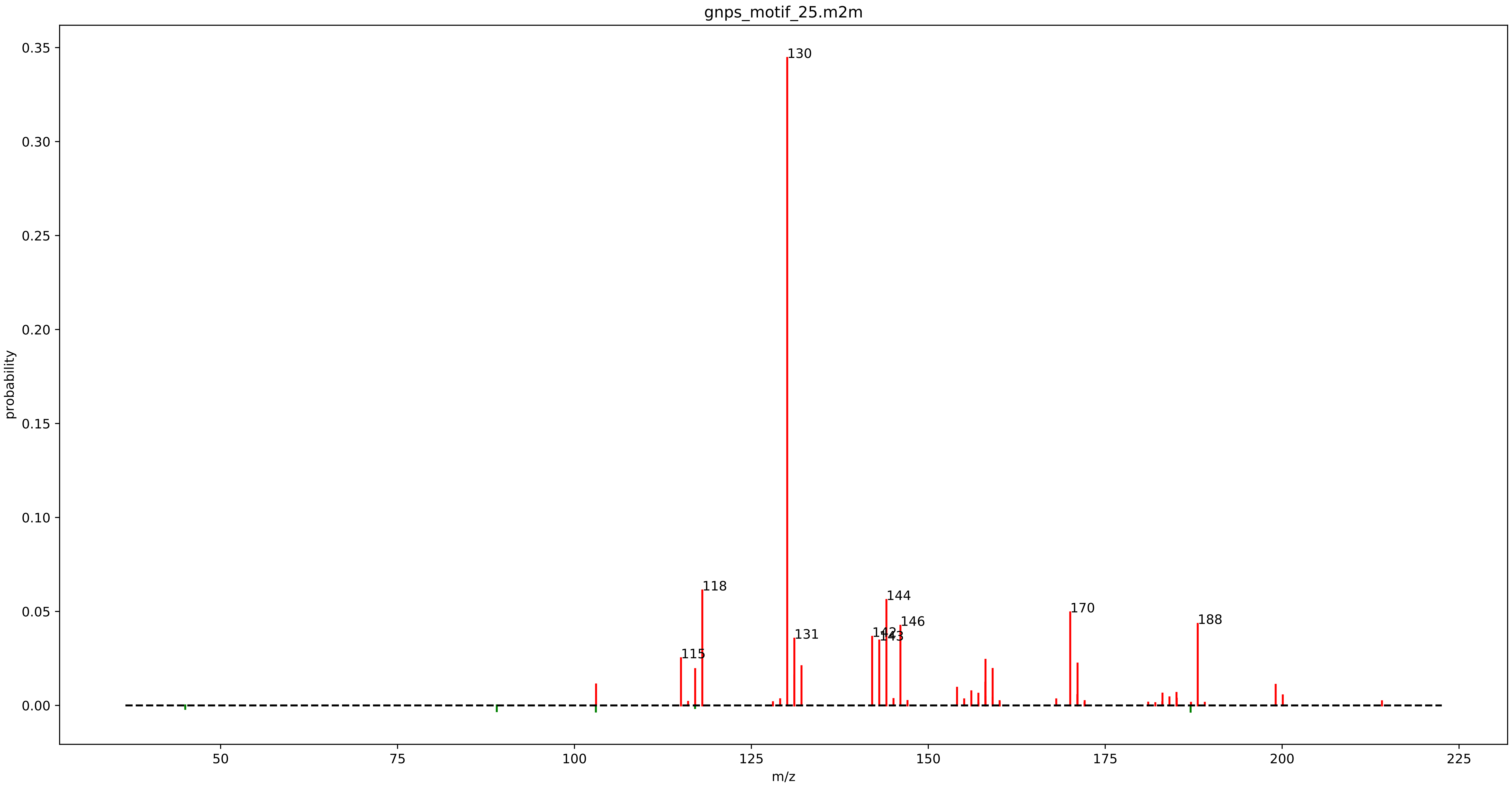

gnps_motif_25.m2m
probability
m/z
0.35
0.30
0.25
0.20
0.15
0.10
0.05
0.00
50
75
100
125
150
175
200
225
130
118
144
146
170
188
131
142
143
115

gnps_motif_25.m2m

fragment_130.0675	: 0.34
fragment_118.0675	: 0.06
fragment_144.0825	: 0.06
fragment_170.0575	: 0.05
fragment_188.0725	: 0.04
fragment_146.0625	: 0.04
fragment_142.0675	: 0.04
fragment_131.0725	: 0.04
fragment_143.0725	: 0.03
fragment_115.0525	: 0.03
fragment_158.0825	: 0.02
fragment_171.0925	: 0.02
fragment_132.0825	: 0.02
fragment_159.0925	: 0.02
fragment_117.0575	: 0.02
fragment_131.0675	: 0.02
fragment_158.0625	: 0.01
fragment_103.0525	: 0.01
fragment_199.0875	: 0.01
fragment_154.0625	: 0.01
fragment_156.0825	: 0.01
fragment_185.0725	: 0.01
fragment_183.0925	: 0.01
fragment_157.0775	: 0.01
fragment_171.0625	: 0.01
fragment_200.0925	: 0.01

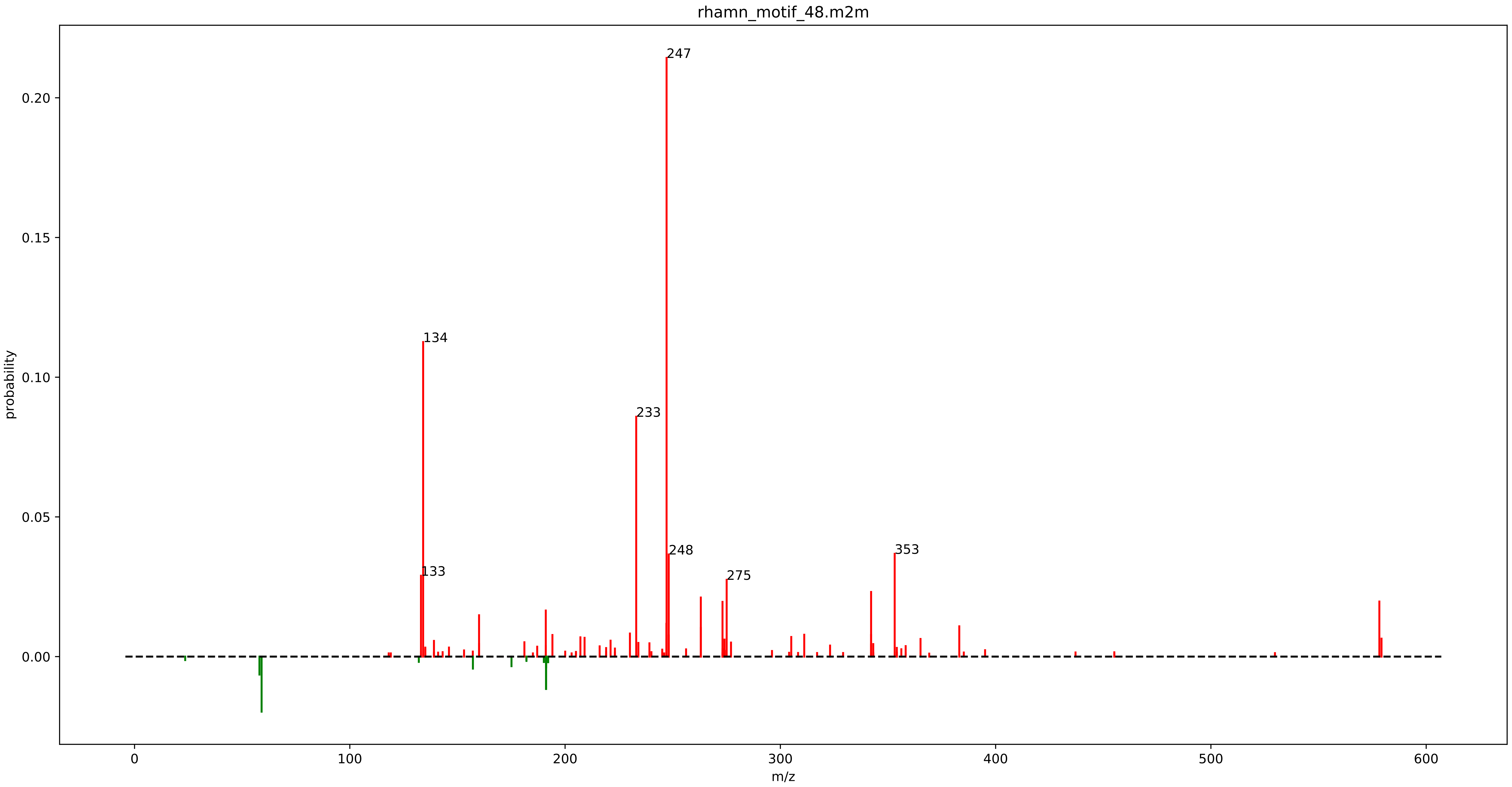

rhamn_motif_48.m2m
probability
m/z
0.20
0.15
0.10
0.05
0.00
0
100
200
300
400
500
600
247
134
233
133
248
275
353

rhamn_motif_48.m2m

fragment_247.1425	: 0.21
fragment_134.0625	: 0.11
fragment_233.0425	: 0.09
fragment_353.1025	: 0.04
fragment_248.1475	: 0.04
fragment_133.0525	: 0.03
fragment_275.0525	: 0.03
fragment_342.1475	: 0.02
fragment_263.0525	: 0.02
loss_59.0225	: 0.02
fragment_578.2475	: 0.02
fragment_273.1225	: 0.02
fragment_191.0325	: 0.02
fragment_160.0375	: 0.01
fragment_247.0625	: 0.01
loss_191.1675	: 0.01
loss_191.1625	: 0.01
fragment_383.1125	: 0.01
fragment_263.0575	: 0.01
fragment_230.1175	: 0.01
fragment_311.0925	: 0.01
fragment_342.1375	: 0.01
fragment_194.1175	: 0.01
fragment_248.1525	: 0.01
fragment_305.0625	: 0.01
fragment_207.1125	: 0.01
fragment_209.0475	: 0.01
loss_58.0175	: 0.01
fragment_579.2525	: 0.01
fragment_365.1075	: 0.01
fragment_274.0475	: 0.01
fragment_247.0575	: 0.01
fragment_221.1275	: 0.01
fragment_139.0875	: 0.01
fragment_181.0975	: 0.01
fragment_277.0725	: 0.01

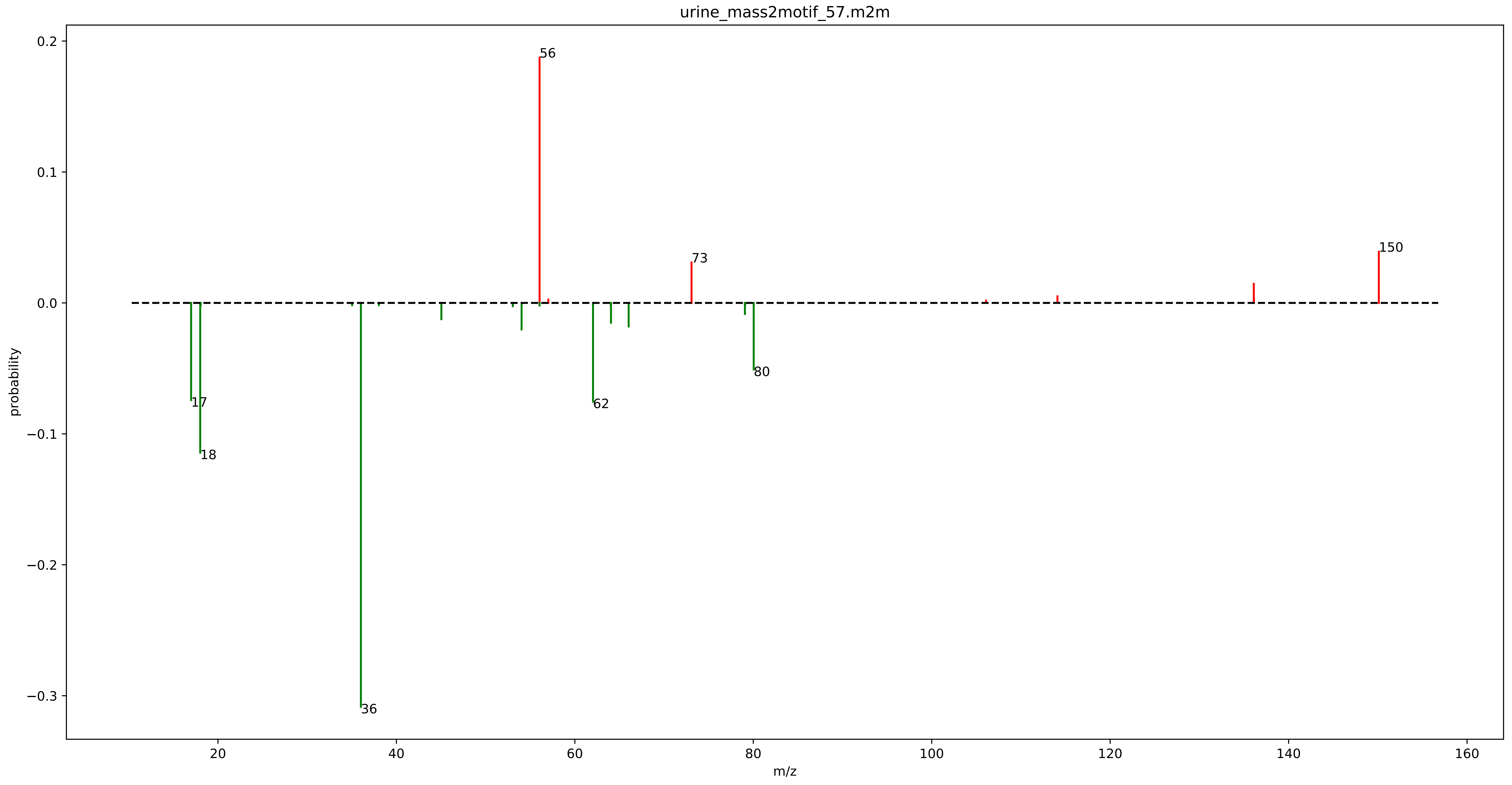

urine_mass2motif_57.m2m
0.2
0.1
0.0
−0.1
−0.2
−0.3
probability
56
73
150
17
18
36
62
80
20
40
60
80
100
120
140
160
m/z

urine_mass2motif_57.m2m

loss_36.0225	: 0.31
fragment_56.0475	: 0.19
loss_18.0125	: 0.11
loss_62.0375	: 0.08
loss_16.9925	: 0.07
loss_80.0475	: 0.05
fragment_150.1125	: 0.04
fragment_73.0775	: 0.03
loss_54.0325	: 0.02
loss_66.0325	: 0.02
loss_64.0525	: 0.02
fragment_136.0975	: 0.01
loss_45.0425	: 0.01
loss_79.0625	: 0.01
fragment_73.0825	: 0.01
fragment_114.0925	: 0.01

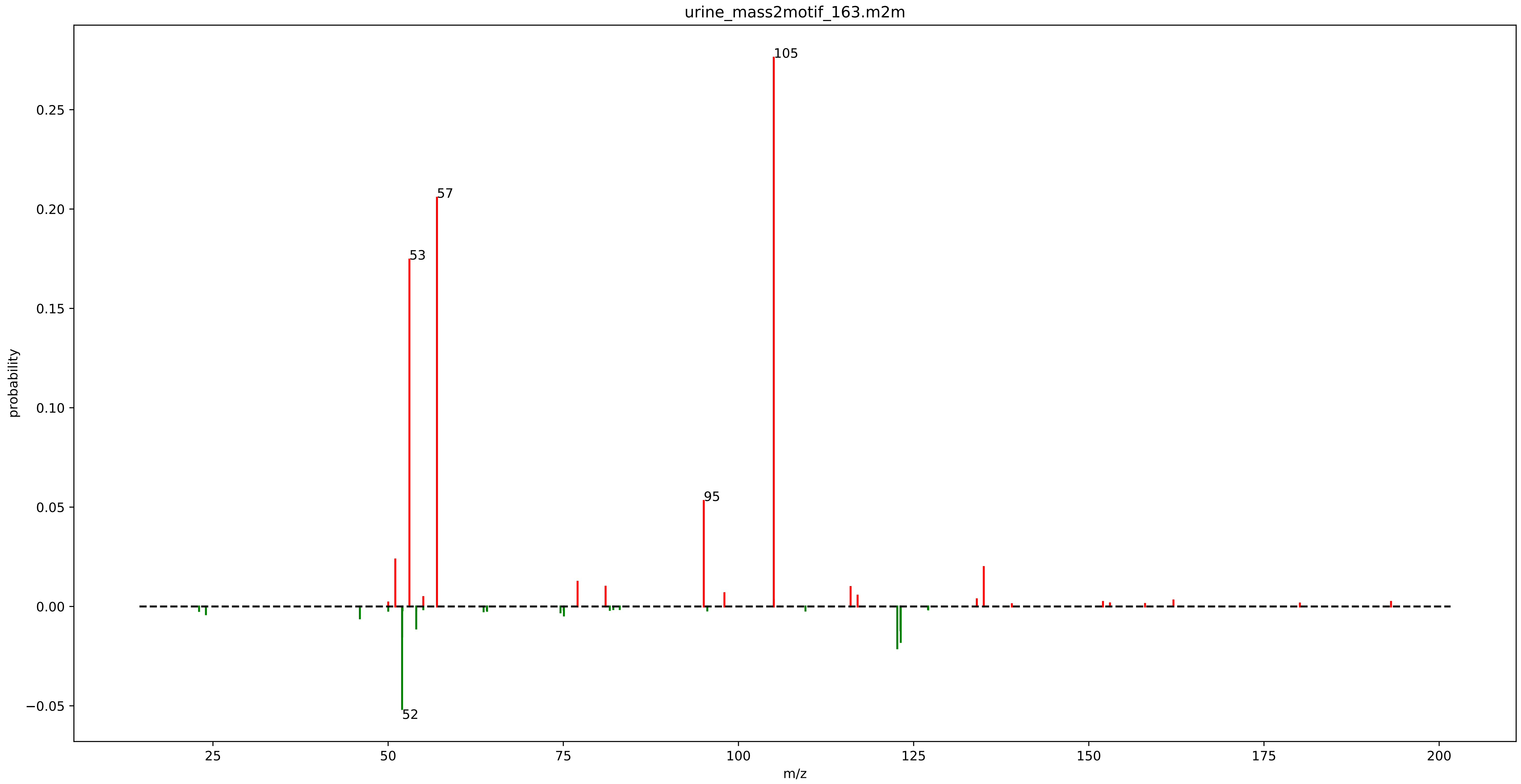

urine_mass2motif_163.m2m
probability
m/z
105
57
53
95
52
0.25
0.20
0.15
0.10
0.05
0.00
−0.05
25
50
75
100
125
150
175
200

urine_mass2motif_163.m2m

fragment_105.0325	: 0.28
fragment_56.9675	: 0.21
fragment_53.0375	: 0.17
fragment_95.0475	: 0.05
loss_51.9925	: 0.05
fragment_51.0225	: 0.02
loss_122.6625	: 0.02
fragment_135.0075	: 0.02
loss_123.1575	: 0.02
loss_52.0025	: 0.02
fragment_77.0375	: 0.01
loss_123.1225	: 0.01
loss_54.0125	: 0.01
fragment_81.0325	: 0.01
fragment_116.0025	: 0.01
fragment_97.9925	: 0.01
loss_45.9675	: 0.01
fragment_116.9975	: 0.01

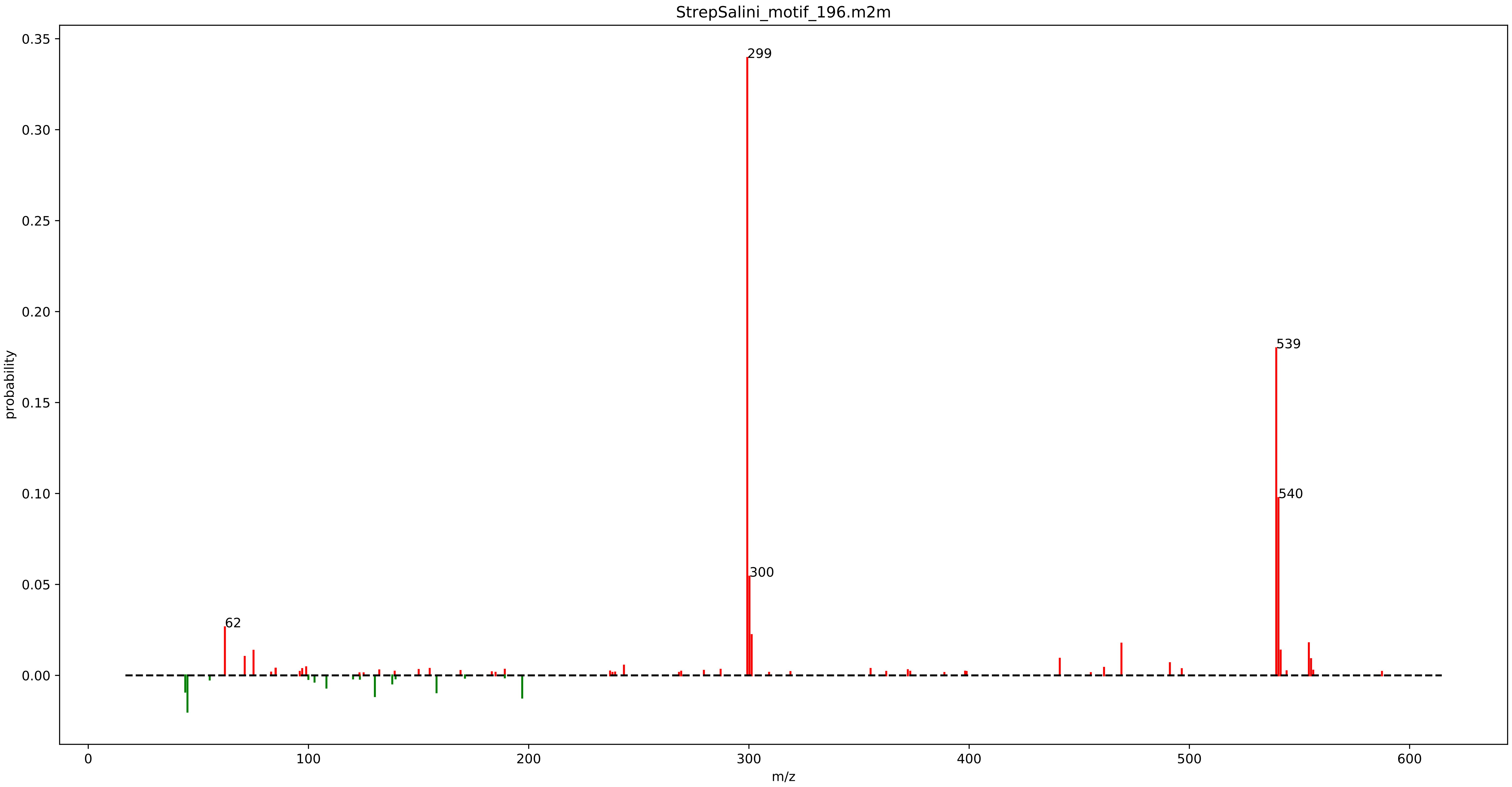

StrepSalini_motif_196.m2m
probability
m/z
299
300
539
540
62
0.35
0.30
0.25
0.20
0.15
0.10
0.05
0.00
0
100
200
300
400
500
600

StrepSalini_motif_196.m2m

fragment_299.2500	: 0.34
fragment_539.4500	: 0.18
fragment_540.4500	: 0.10
fragment_300.2500	: 0.05
fragment_62.0500	: 0.03
fragment_301.2525	: 0.02
loss_45.0500	: 0.02
fragment_554.2500	: 0.02
fragment_469.1500	: 0.02
fragment_541.4500	: 0.01
fragment_75.0500	: 0.01
loss_197.0500	: 0.01
loss_130.1500	: 0.01
fragment_71.0500	: 0.01
loss_158.1500	: 0.01
fragment_441.1500	: 0.01
fragment_555.2500	: 0.01
loss_44.0500	: 0.01
loss_108.1525	: 0.01
fragment_491.1500	: 0.01
fragment_243.2500	: 0.01

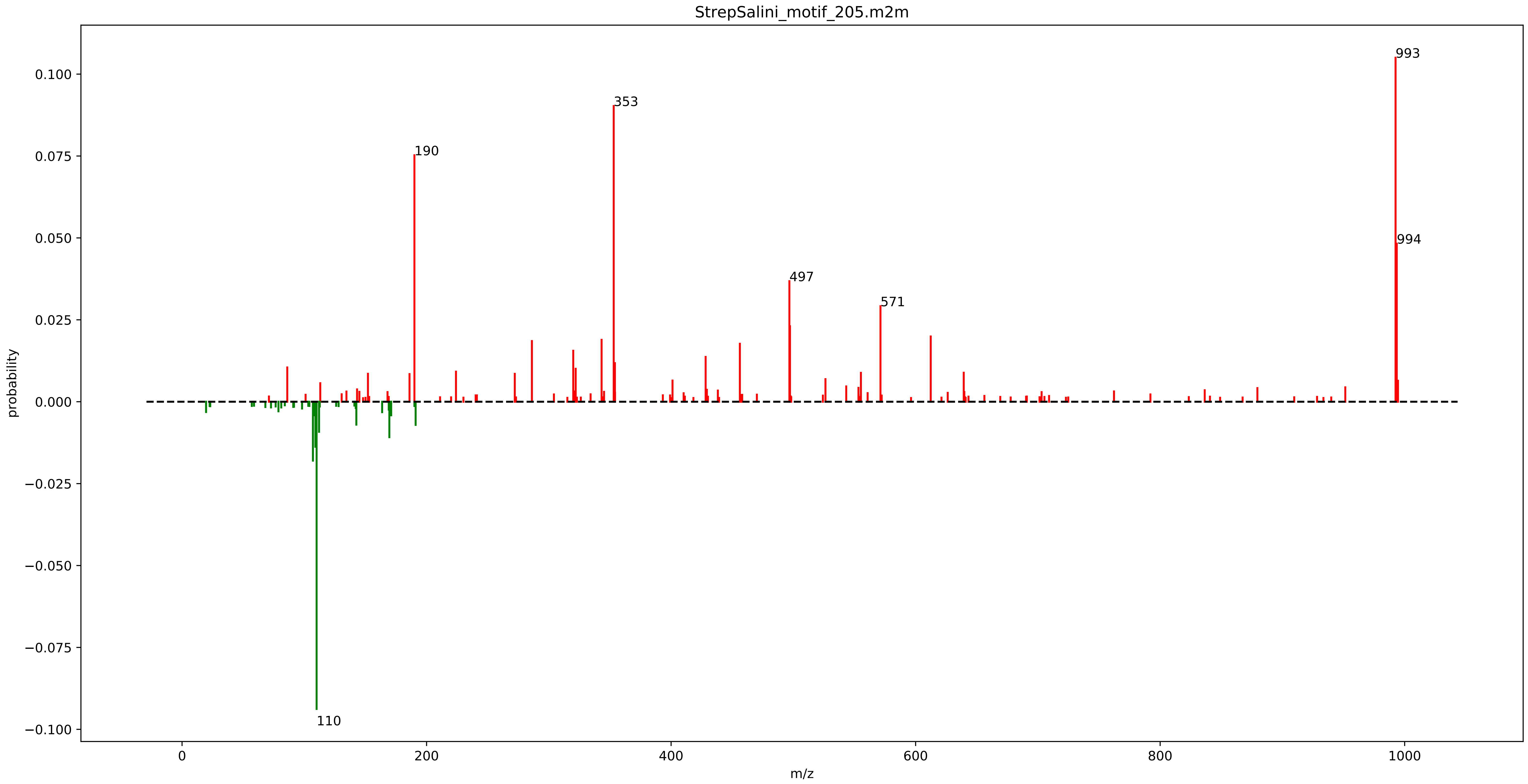

StrepSalini_motif_205.m2m
probability
m/z
0.100
0.075
0.050
0.025
0.000
−0.025
−0.050
−0.075
−0.100
0
200
400
600
800
1000
993
353
190
994
497
571
110

StrepSalini_motif_205.m2m

fragment_992.5500	: 0.11
loss_110.0500	: 0.09
fragment_353.0500	: 0.09
fragment_190.0500	: 0.08
fragment_993.5500	: 0.05
fragment_496.7500	: 0.04
fragment_571.2500	: 0.03
fragment_497.2500	: 0.02
fragment_612.3500	: 0.02
fragment_343.1500	: 0.02
fragment_286.1500	: 0.02
loss_107.0500	: 0.02
fragment_456.2500	: 0.02
fragment_319.9500	: 0.02
loss_109.0525	: 0.01
fragment_428.2500	: 0.01
fragment_354.0500	: 0.01
loss_169.5500	: 0.01
fragment_86.0500	: 0.01
fragment_321.9500	: 0.01
loss_112.0500	: 0.01
fragment_224.0500	: 0.01
fragment_639.3500	: 0.01
fragment_555.2500	: 0.01
fragment_152.0500	: 0.01
fragment_272.1500	: 0.01
fragment_186.0500	: 0.01
fragment_353.2500	: 0.01
loss_191.0500	: 0.01
loss_142.5500	: 0.01
fragment_526.2500	: 0.01
fragment_401.1500	: 0.01
fragment_994.5500	: 0.01
fragment_113.0500	: 0.01

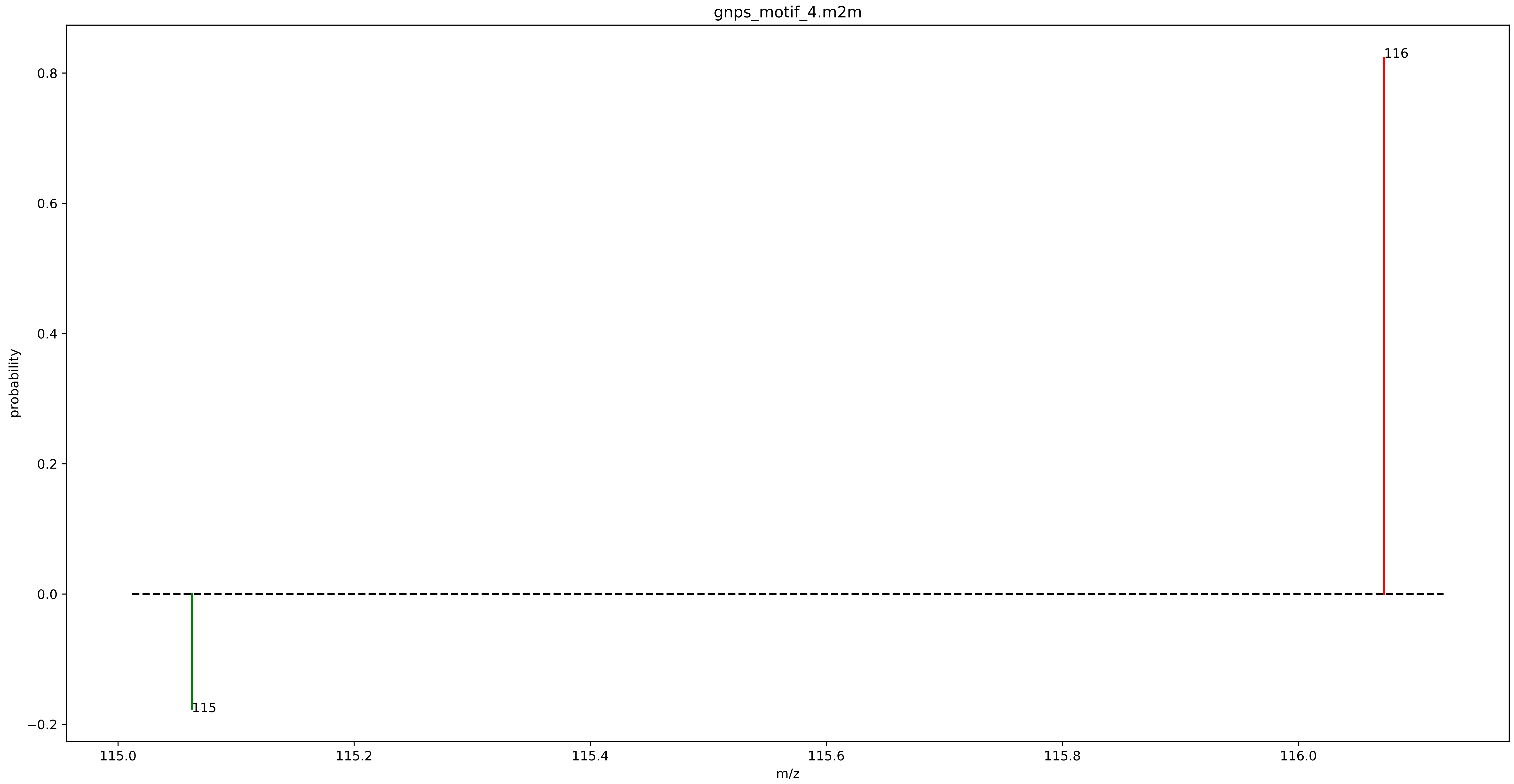

gnps_motif_4.m2m
116
115
0.8
0.6
0.4
0.2
0.0
−0.2
probability
115.0
115.2
115.4
115.6
115.8
116.0
m/z

gnps_motif_4.m2m

fragment_116.0725	: 0.82
loss_115.0625	: 0.18

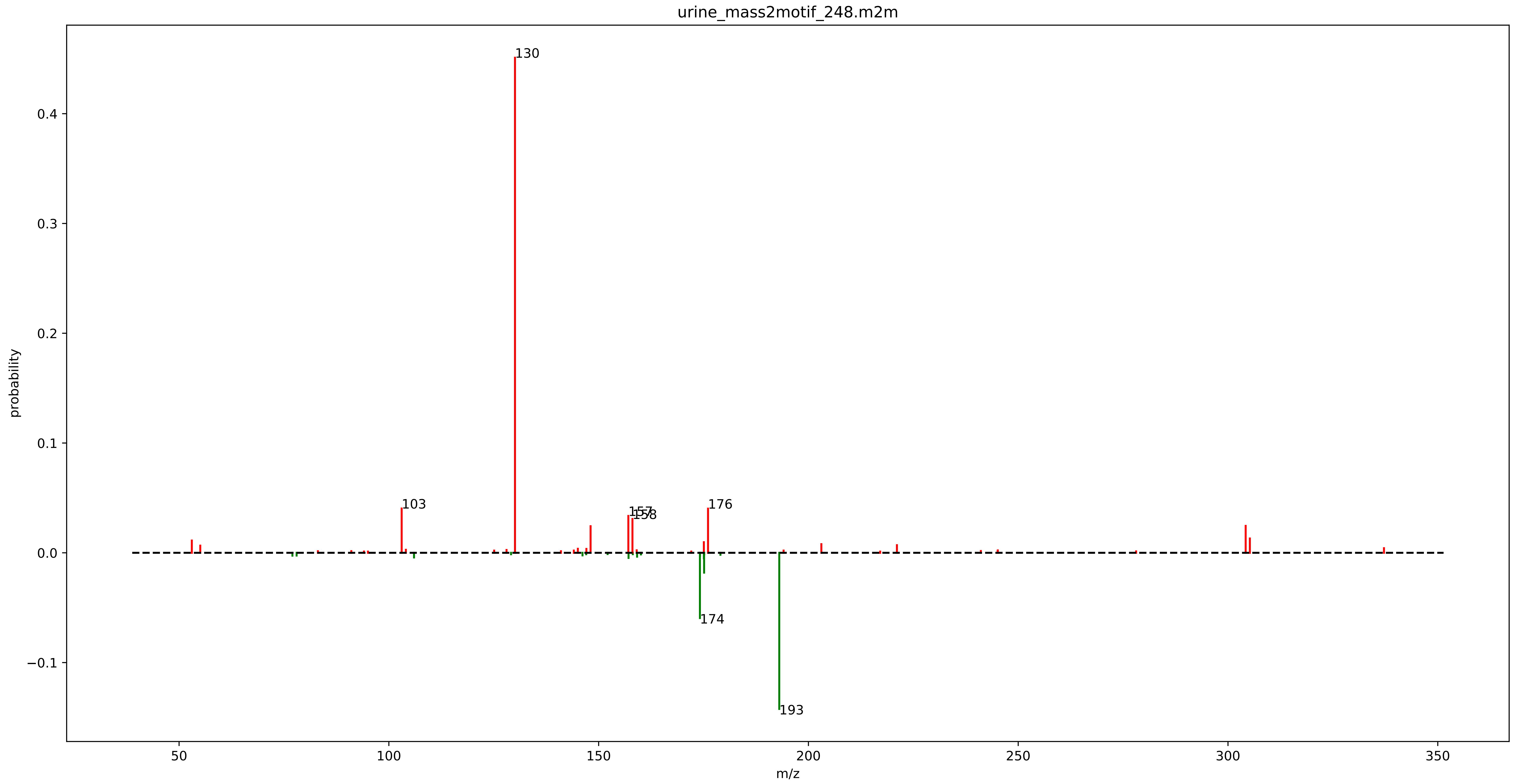

urine_mass2motif_248.m2m
probability
m/z
0.4
0.3
0.2
0.1
0.0
−0.1
50
100
150
200
250
300
350
130
103
157
158
176
174
193

urine_mass2motif_248.m2m

fragment_130.0625	: 0.45
loss_193.0475	: 0.14
loss_174.1375	: 0.06
fragment_103.0525	: 0.04
fragment_176.0725	: 0.04
fragment_157.0775	: 0.03
fragment_158.0575	: 0.03
fragment_304.2125	: 0.02
fragment_148.0775	: 0.02
loss_175.1375	: 0.02
fragment_305.2125	: 0.01
fragment_53.0375	: 0.01
fragment_175.0775	: 0.01
fragment_203.0825	: 0.01
fragment_221.0925	: 0.01
fragment_55.0525	: 0.01
loss_175.0775	: 0.01

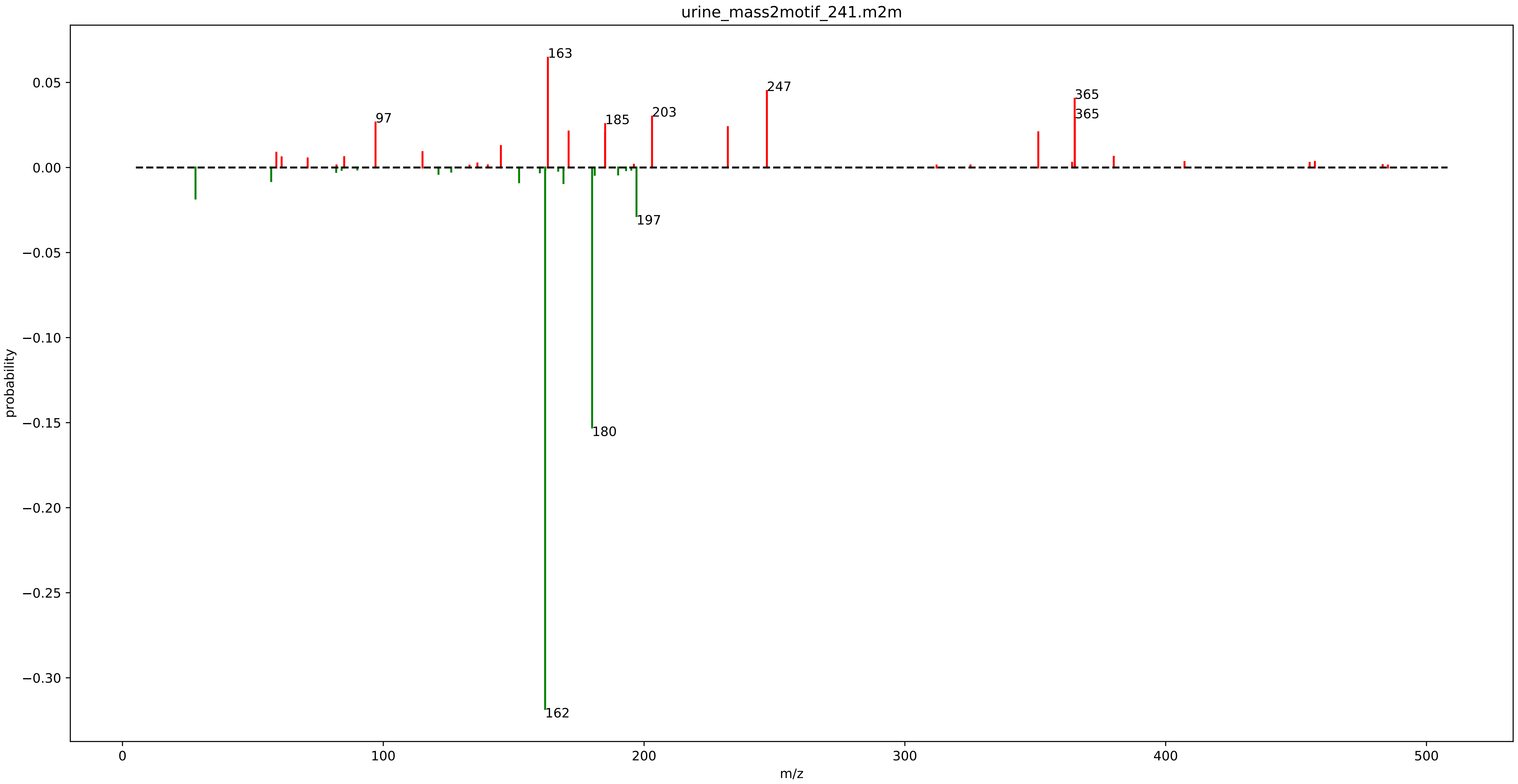

urine_mass2motif_241.m2m
probability
m/z
0.05
0.00
−0.05
−0.10
−0.15
−0.20
−0.25
−0.30
0
100
200
300
400
500
163
247
365
365
97
185
203
197
180
162

urine_mass2motif_241.m2m

loss_162.0575	: 0.32
loss_180.0775	: 0.15
fragment_163.1125	: 0.06
fragment_247.0925	: 0.04
fragment_365.1075	: 0.04
fragment_203.0525	: 0.03
fragment_365.1725	: 0.03
loss_197.0925	: 0.03
fragment_97.0275	: 0.03
fragment_185.0825	: 0.03
fragment_232.1175	: 0.02
fragment_171.0675	: 0.02
fragment_351.1575	: 0.02
fragment_185.0425	: 0.02
loss_28.0025	: 0.02
fragment_145.0975	: 0.01
loss_169.0925	: 0.01
fragment_115.0375	: 0.01
fragment_58.9975	: 0.01
loss_152.0775	: 0.01
loss_57.0225	: 0.01
fragment_380.0925	: 0.01
fragment_85.0125	: 0.01
fragment_61.0275	: 0.01
fragment_71.0125	: 0.01

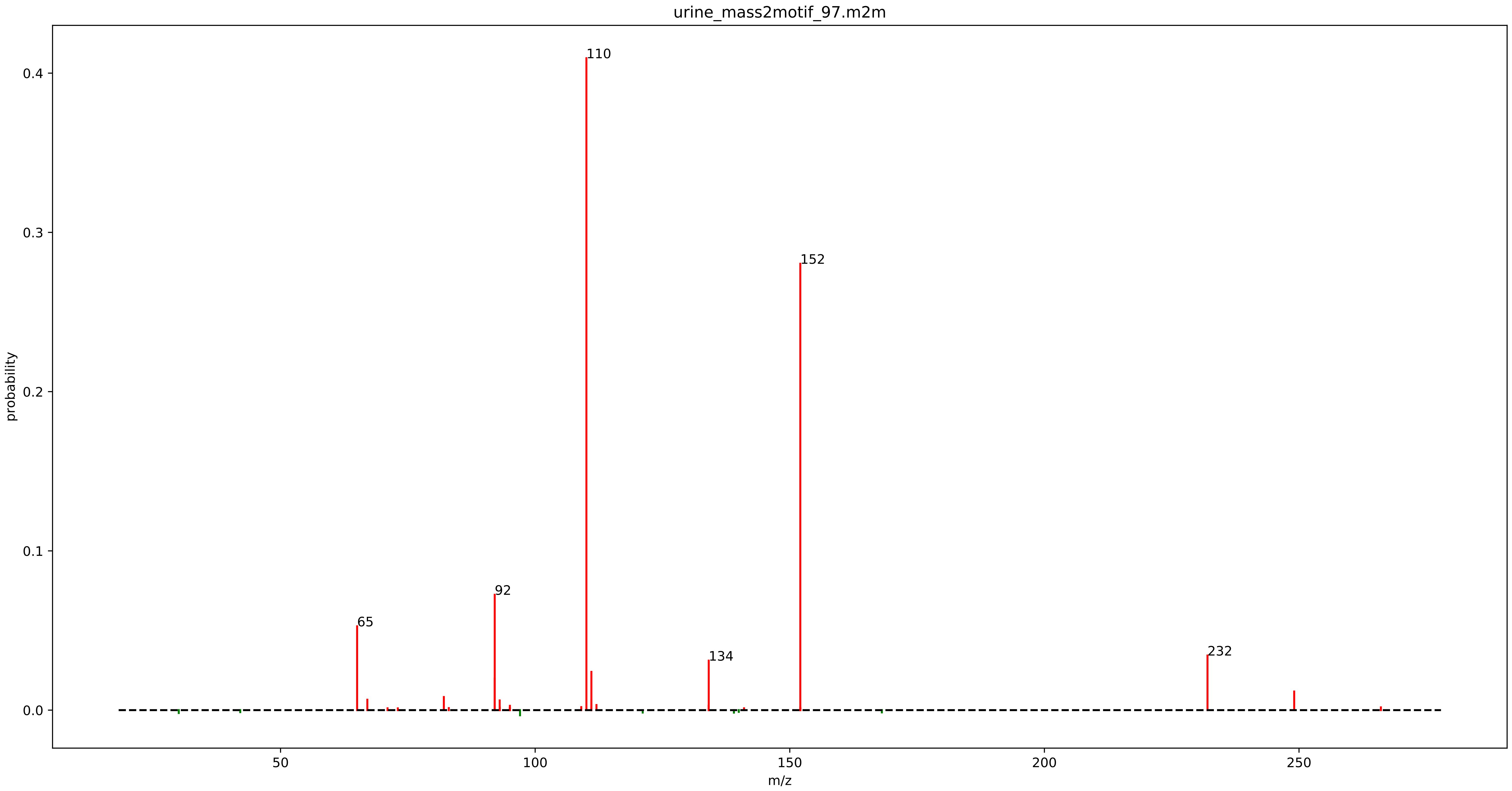
urine_mass2motif_97.m2m
probability
m/z
110
152
92
65
134
232
0.4
0.3
0.2
0.1
0.0
50
100
150
200
250

urine_mass2motif_97.m2m

fragment_110.0625	: 0.41
fragment_152.0675	: 0.28
fragment_92.0575	: 0.07
fragment_65.0375	: 0.05
fragment_232.0275	: 0.03
fragment_134.0825	: 0.03
fragment_111.0425	: 0.02
fragment_92.0475	: 0.02
fragment_249.0525	: 0.01
fragment_82.0675	: 0.01
fragment_67.0425	: 0.01
fragment_111.0375	: 0.01
fragment_93.0325	: 0.01

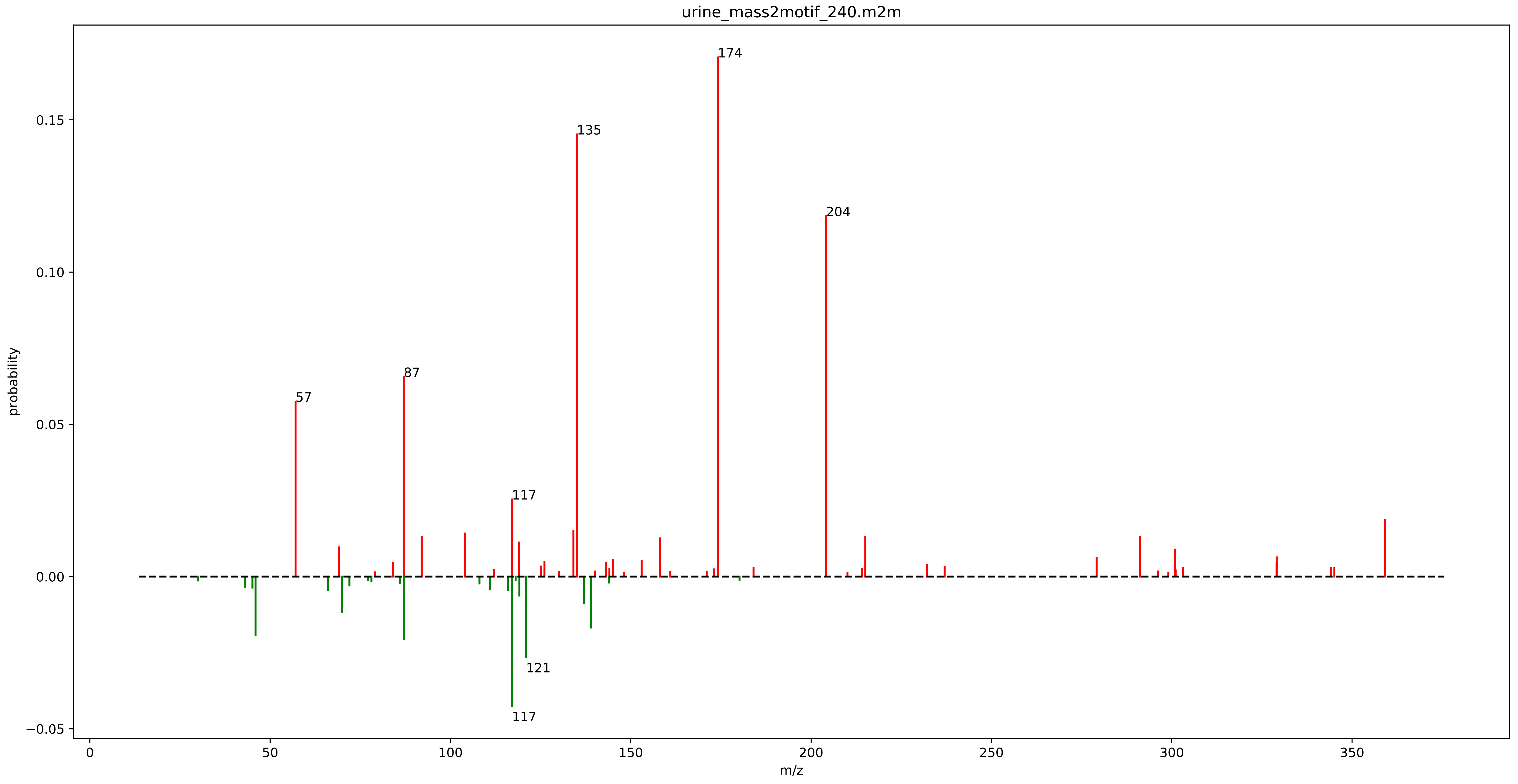
urine_mass2motif_240.m2m
probability
m/z
0.15
0.10
0.05
0.00
−0.05
0
50
100
150
200
250
300
350
174
135
204
87
57
117
121
117

urine_mass2motif_240.m2m

fragment_174.1225	: 0.17
fragment_135.0475	: 0.15
fragment_204.1375	: 0.12
fragment_87.0425	: 0.07
fragment_57.0475	: 0.06
loss_117.0525	: 0.04
loss_120.9775	: 0.03
fragment_117.0375	: 0.03
loss_87.0425	: 0.02
loss_45.9575	: 0.02
fragment_359.1125	: 0.02
loss_138.9925	: 0.02
fragment_134.0825	: 0.02
fragment_104.0725	: 0.01
fragment_291.1625	: 0.01
fragment_215.0125	: 0.01
fragment_92.0225	: 0.01
fragment_158.1275	: 0.01
loss_70.0075	: 0.01
fragment_119.0175	: 0.01
fragment_69.0325	: 0.01
fragment_300.8675	: 0.01
loss_137.0125	: 0.01
fragment_329.1025	: 0.01
loss_119.1075	: 0.01
fragment_279.1775	: 0.01
fragment_145.0325	: 0.01
fragment_153.0175	: 0.01

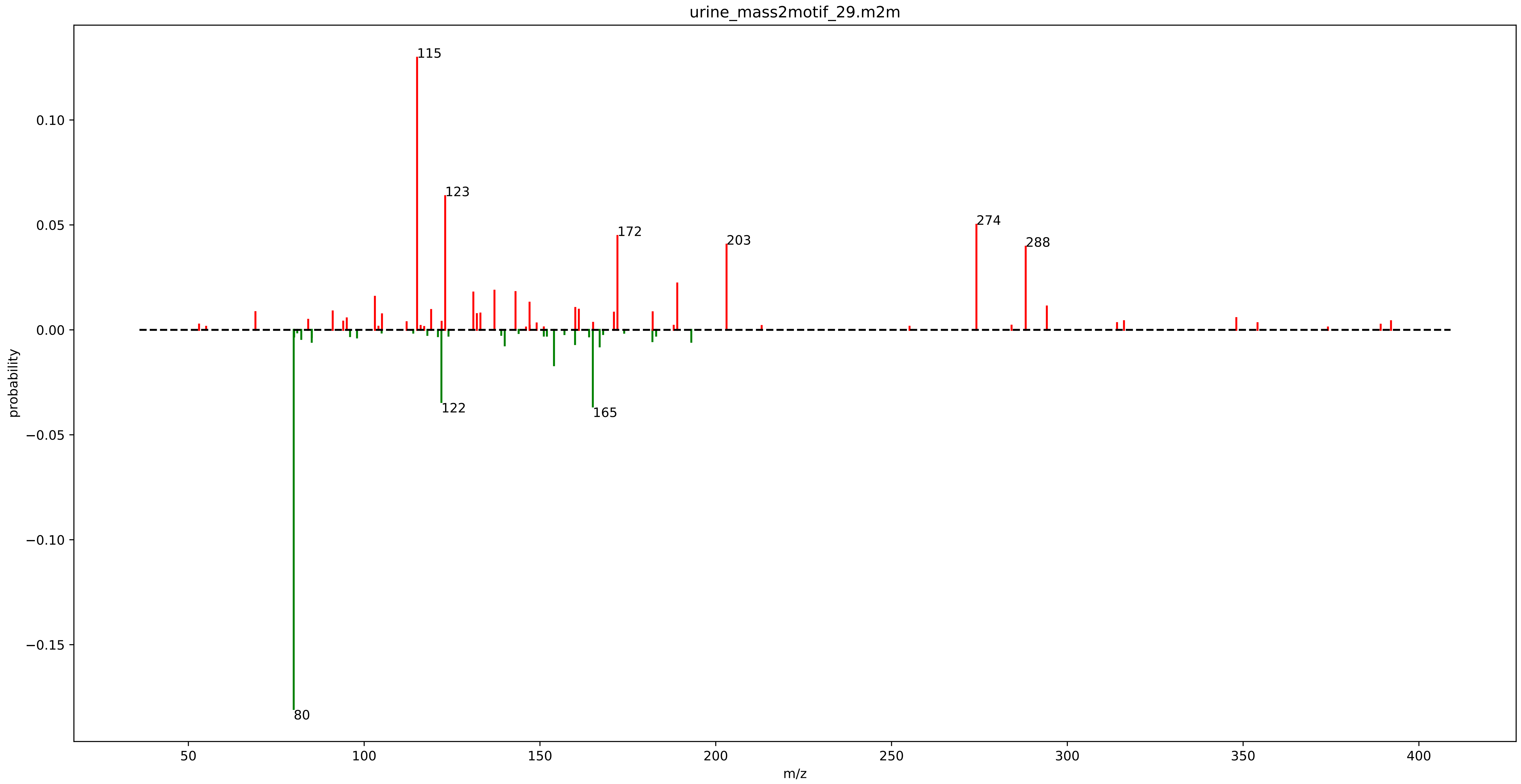

urine_mass2motif_29.m2m
probability
m/z
0.10
0.05
0.00
−0.05
−0.10
−0.15
50
100
150
200
250
300
350
400
115
123
172
203
274
288
122
165
80

urine_mass2motif_29.m2m

loss_79.9575	: 0.18
fragment_115.0525	: 0.13
fragment_123.0425	: 0.06
fragment_274.1425	: 0.05
fragment_172.0375	: 0.04
fragment_203.0725	: 0.04
fragment_288.1575	: 0.04
loss_165.0375	: 0.04
loss_121.9675	: 0.03
fragment_189.0525	: 0.02
fragment_137.0575	: 0.02
fragment_143.0475	: 0.02
fragment_131.0525	: 0.02
loss_153.9925	: 0.02
fragment_103.0525	: 0.02
fragment_147.0425	: 0.01
fragment_294.1725	: 0.01
fragment_160.0525	: 0.01
fragment_161.0625	: 0.01
fragment_119.0475	: 0.01
fragment_91.0525	: 0.01
fragment_69.0725	: 0.01
fragment_182.0625	: 0.01
fragment_171.0425	: 0.01
loss_166.9875	: 0.01
fragment_133.0625	: 0.01
fragment_132.0575	: 0.01
fragment_105.0675	: 0.01
loss_139.9775	: 0.01
loss_159.9825	: 0.01
loss_85.0875	: 0.01
loss_193.0475	: 0.01
fragment_348.0675	: 0.01
fragment_95.0475	: 0.01
loss_181.9875	: 0.01

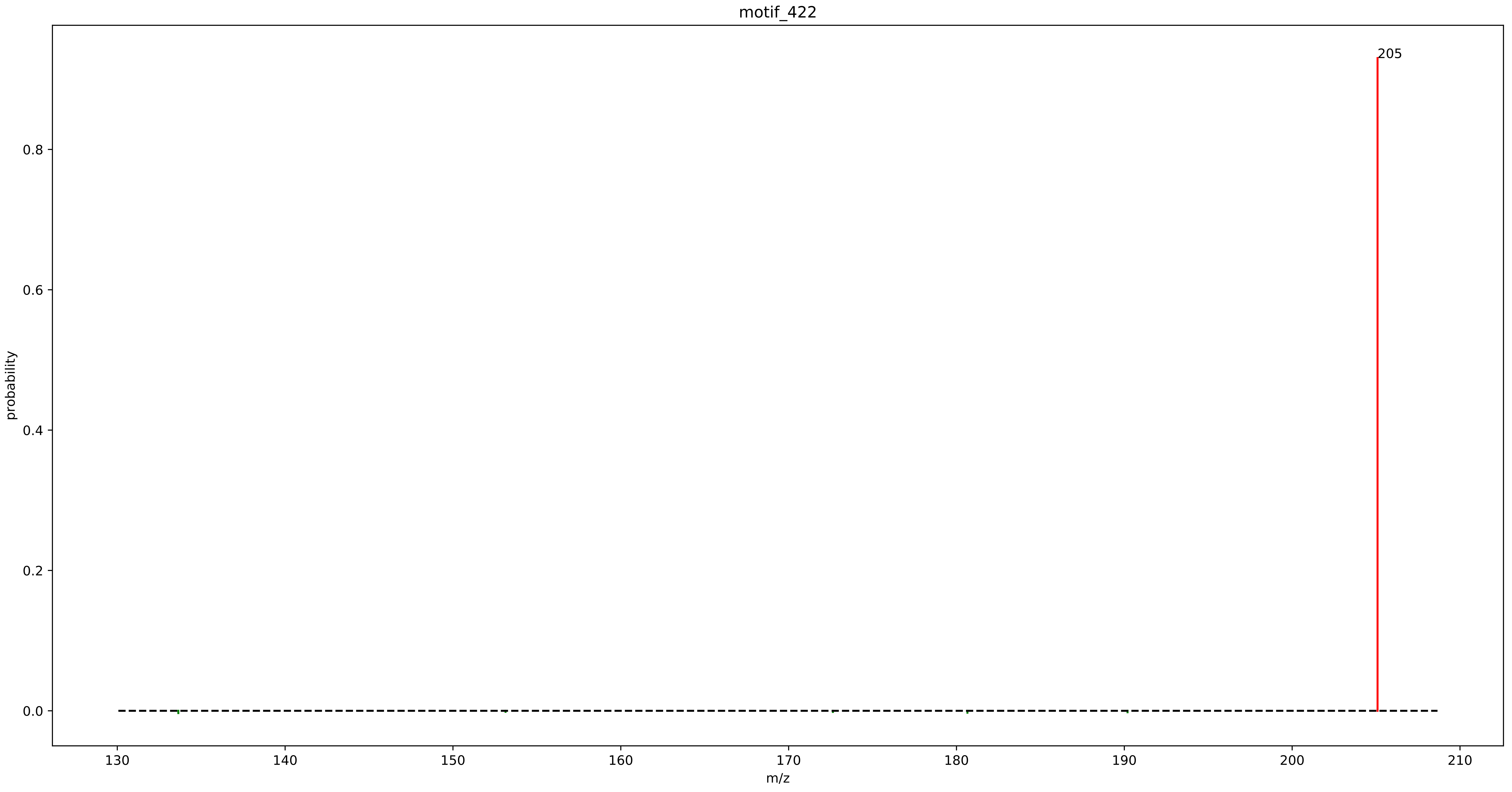
motif_422
205
probability
0.0
0.2
0.4
0.6
0.8
130
140
150
160
170
180
190
200
210
m/z

motif_422

fragment_205.0875 : 0.93

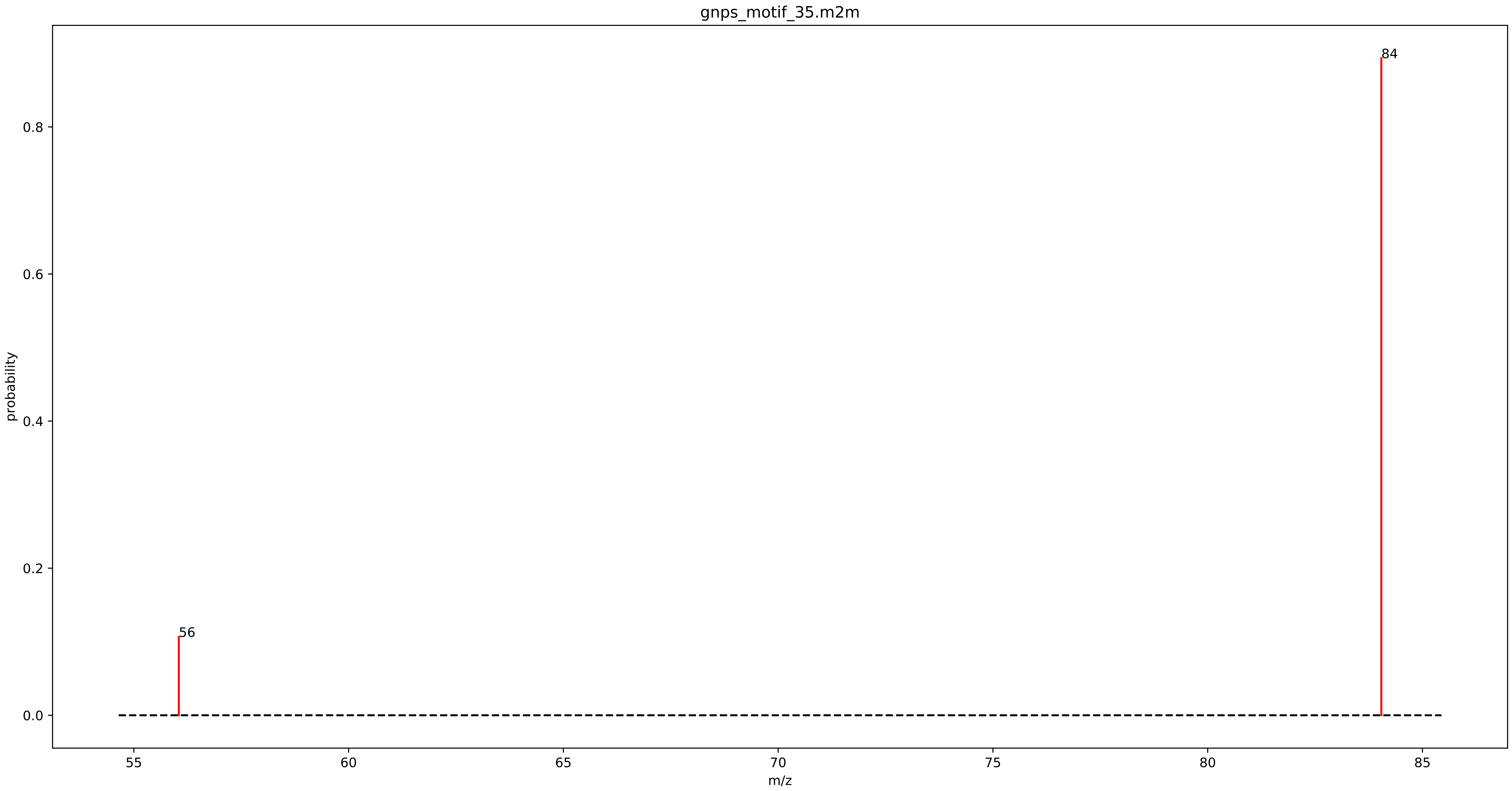

gnps_motif_35.m2m
probability
m/z
84
56
0.0
0.2
0.4
0.6
0.8
55
60
65
70
75
80
85

gnps_motif_35.m2m

fragment_84.0425	: 0.89
fragment_56.0475	: 0.11

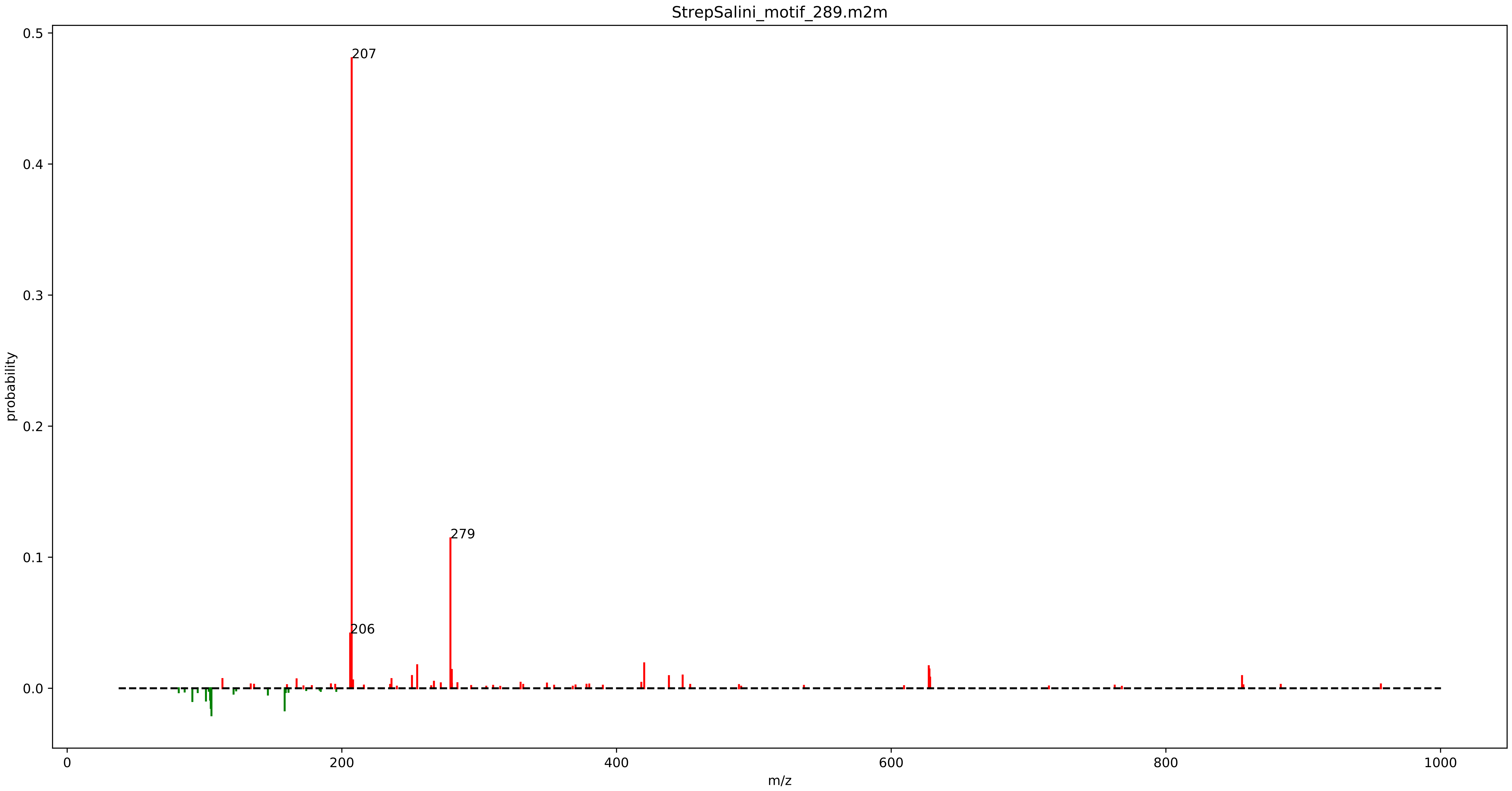

StrepSalini_motif_289.m2m
probability
m/z
207
279
206
0.5
0.4
0.3
0.2
0.1
0.0
0
200
400
600
800
1000

StrepSalini_motif_289.m2m

fragment_207.1500	: 0.48
fragment_279.0500	: 0.11
fragment_206.0500	: 0.04
loss_105.0500	: 0.02
fragment_420.1500	: 0.02
fragment_254.8500	: 0.02
loss_158.3500	: 0.02
fragment_627.3500	: 0.02
loss_104.5500	: 0.01
fragment_627.8500	: 0.01
fragment_280.0500	: 0.01
loss_91.1500	: 0.01
fragment_448.1500	: 0.01
loss_101.0500	: 0.01
fragment_251.0500	: 0.01
fragment_855.4500	: 0.01
fragment_438.1500	: 0.01
loss_104.0500	: 0.01
fragment_628.3500	: 0.01
fragment_113.0500	: 0.01
fragment_236.1500	: 0.01
fragment_167.0500	: 0.01
fragment_208.1500	: 0.01

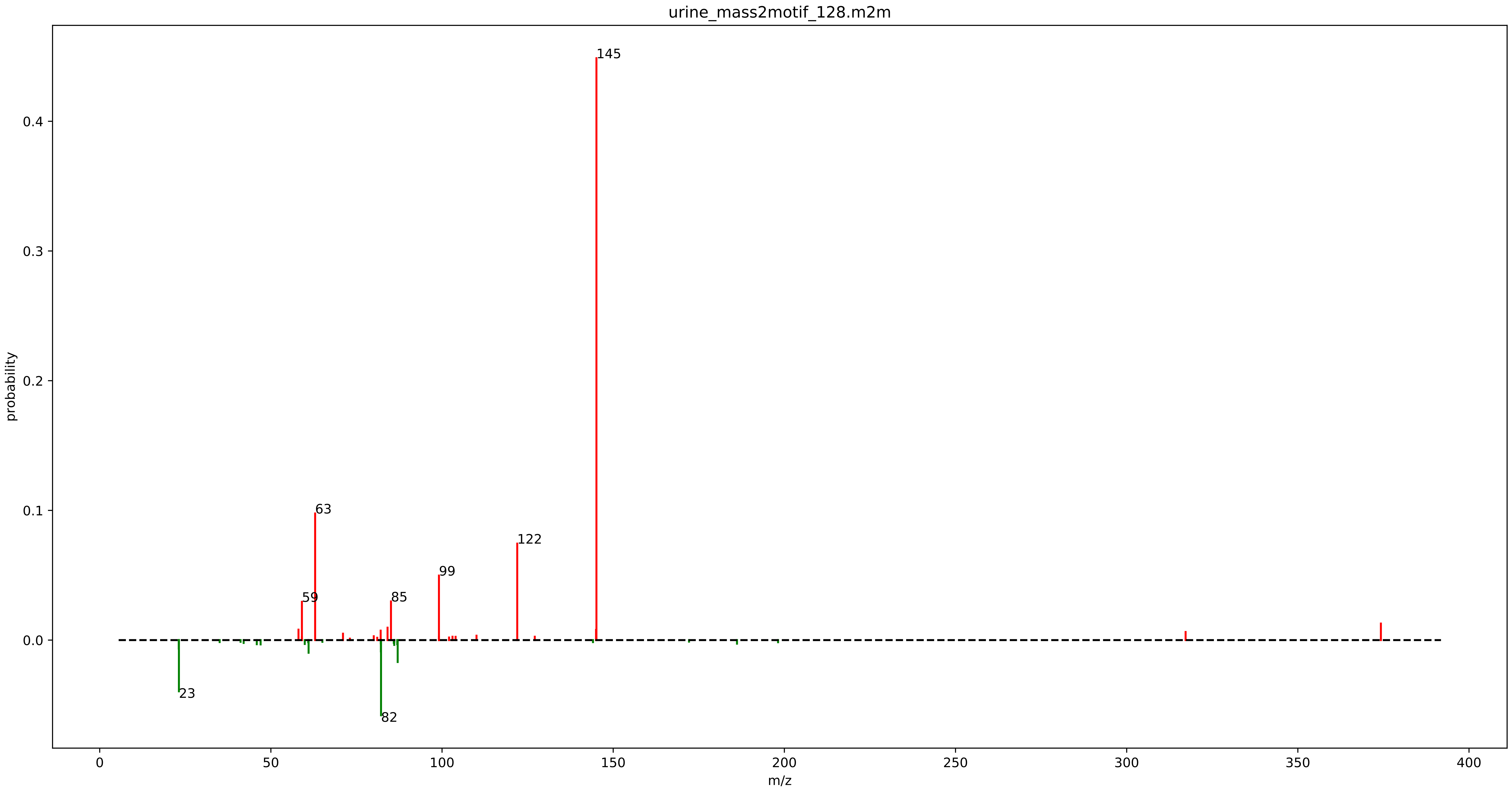

urine_mass2motif_128.m2m
probability
m/z
145
63
122
99
59
85
23
82
0.4
0.3
0.2
0.1
0.0
0
50
100
150
200
250
300
350
400

urine_mass2motif_128.m2m

fragment_145.1025	: 0.45
fragment_62.9275	: 0.10
fragment_121.9675	: 0.07
loss_82.1775	: 0.06
fragment_99.0975	: 0.05
loss_23.1375	: 0.04
fragment_85.0825	: 0.03
fragment_59.0675	: 0.03
loss_87.0425	: 0.02
fragment_374.2575	: 0.01
loss_61.0175	: 0.01
fragment_84.0825	: 0.01
loss_82.1325	: 0.01
fragment_58.0675	: 0.01
fragment_144.9825	: 0.01
fragment_82.0725	: 0.01
loss_82.1675	: 0.01
loss_23.0925	: 0.01
fragment_317.2125	: 0.01

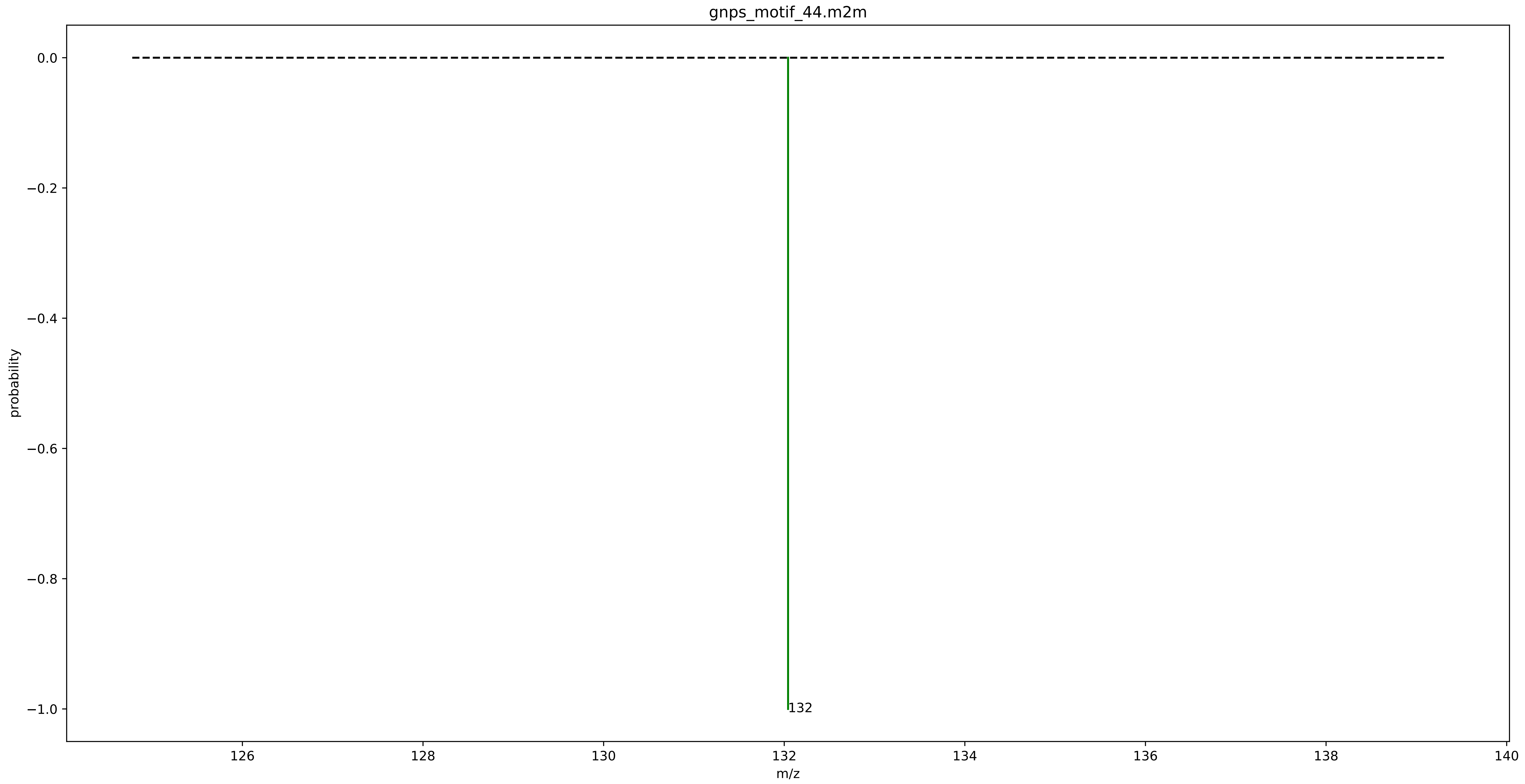

gnps_motif_44.m2m
0.0
−0.2
−0.4
−0.6
−0.8
−1.0
probability
132
126
128
130
132
134
136
138
140
m/z

gnps_motif_44.m2m

loss_132.0425 : 1.00

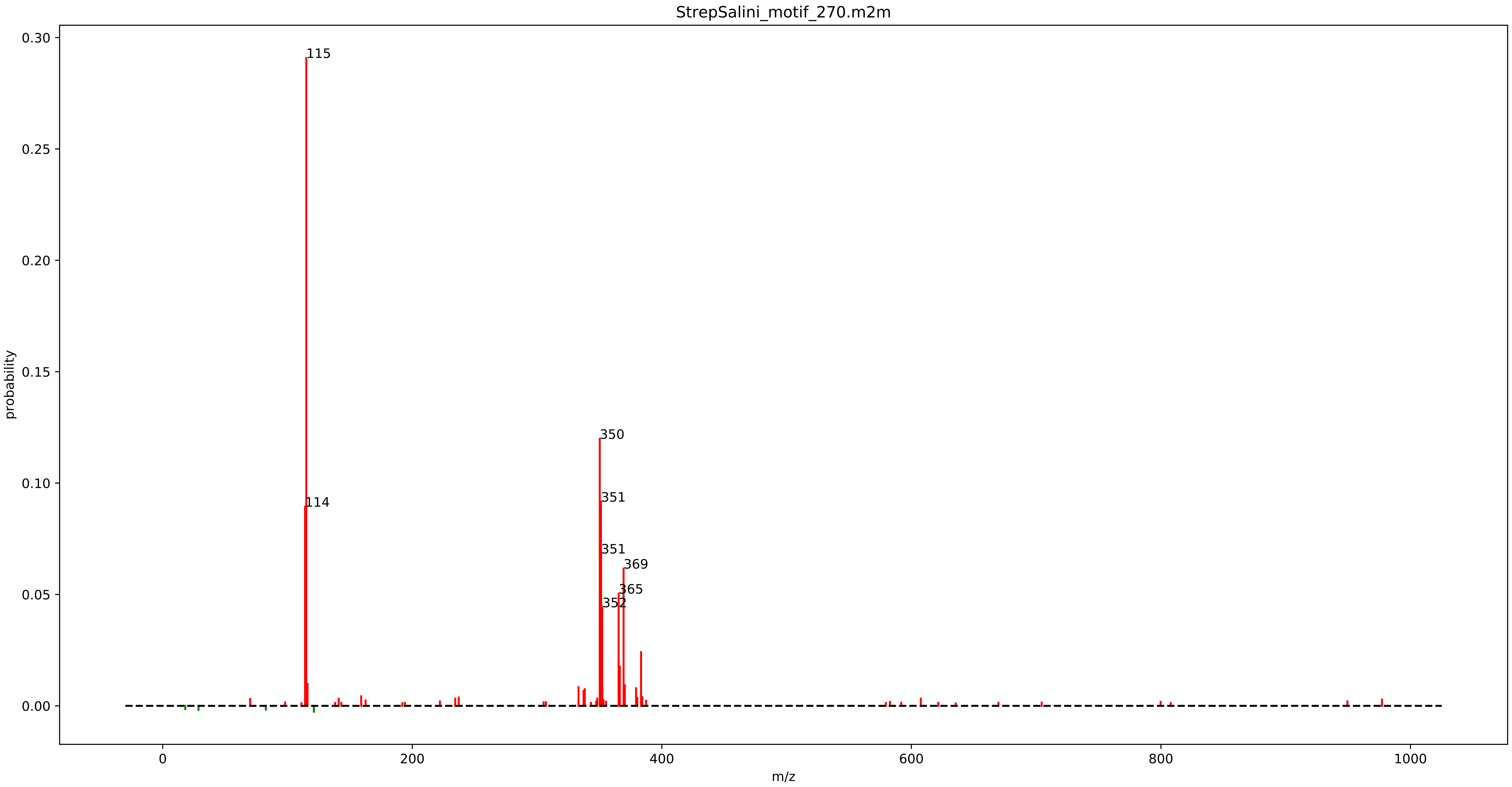

StrepSalini_motif_270.m2m
0.30
0.25
0.20
0.15
0.10
0.05
0.00
probability
115
114
350
351
351
369
365
352
0
200
400
600
800
1000
m/z

StrepSalini_motif_270.m2m

fragment_115.0500	: 0.29
fragment_350.2525	: 0.12
fragment_351.2500	: 0.09
fragment_114.0525	: 0.09
fragment_351.3500	: 0.07
fragment_369.3500	: 0.06
fragment_365.3500	: 0.05
fragment_352.2500	: 0.04
fragment_383.3500	: 0.02
fragment_366.3500	: 0.02
fragment_116.0525	: 0.01
fragment_370.3500	: 0.01
fragment_352.3500	: 0.01
fragment_333.2500	: 0.01
fragment_379.3500	: 0.01
fragment_338.2500	: 0.01
fragment_337.2525	: 0.01

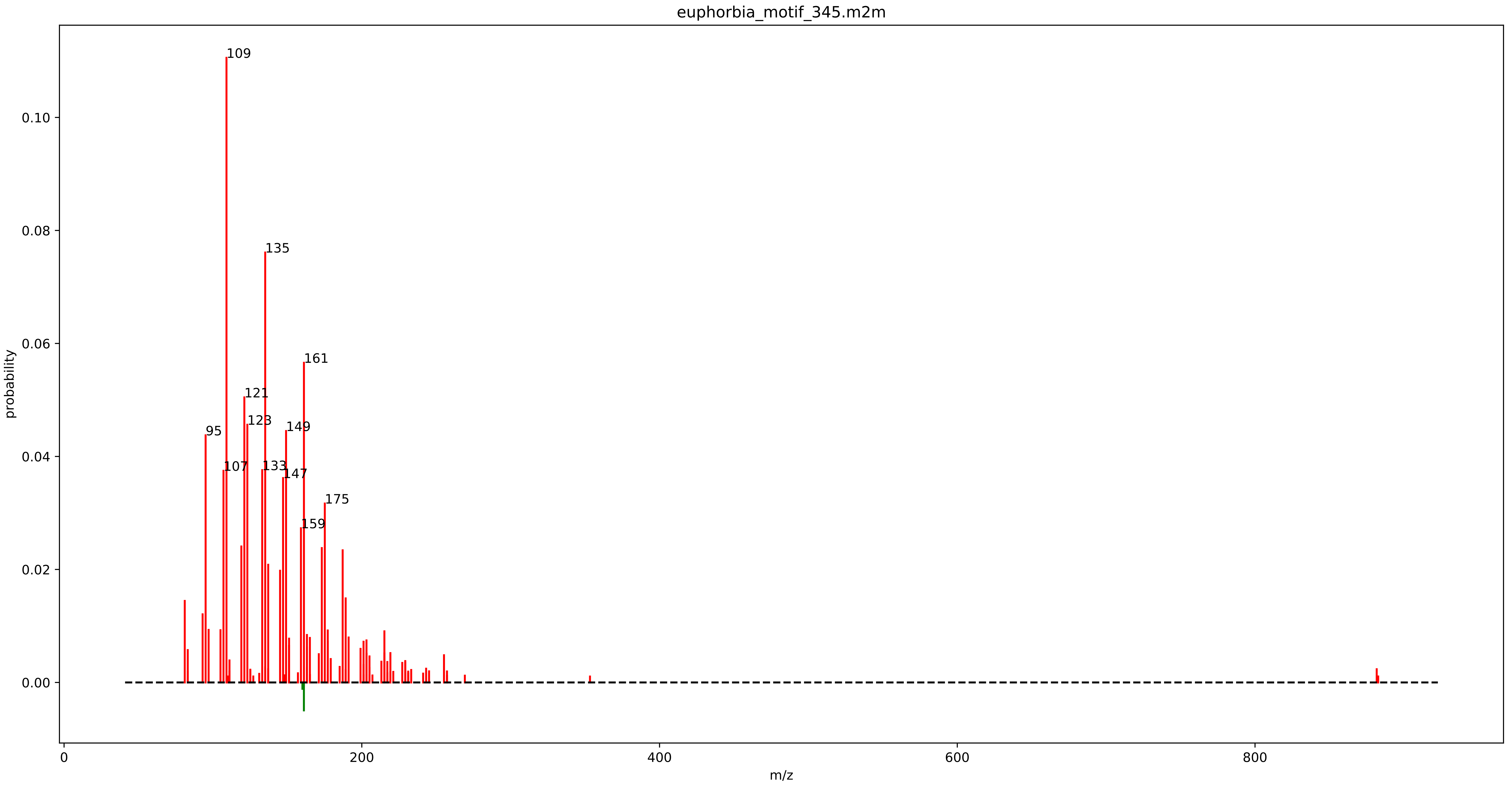

euphorbia_motif_345.m2m
probability
m/z
0.10
0.08
0.06
0.04
0.02
0.00
0
200
400
600
800
109
135
161
121
123
95
149
107
133
147
175
159

euphorbia_motif_345.m2m

fragment_109.0975	: 0.11
fragment_135.1125	: 0.08
fragment_161.1275	: 0.06
fragment_121.0975	: 0.05
fragment_123.1125	: 0.05
fragment_149.1275	: 0.04
fragment_95.0825	: 0.04
fragment_133.0975	: 0.04
fragment_107.0825	: 0.04
fragment_147.1125	: 0.04
fragment_175.1425	: 0.03
fragment_159.1125	: 0.03
fragment_119.0825	: 0.02
fragment_173.1275	: 0.02
fragment_187.1425	: 0.02
fragment_137.1275	: 0.02
fragment_145.0975	: 0.02
fragment_189.1575	: 0.01
fragment_81.0675	: 0.01
fragment_93.0675	: 0.01
fragment_97.0975	: 0.01
fragment_105.0675	: 0.01
fragment_177.1225	: 0.01
fragment_215.1725	: 0.01
fragment_163.1425	: 0.01
fragment_191.1375	: 0.01
fragment_165.1225	: 0.01
fragment_151.1425	: 0.01
fragment_203.1375	: 0.01
fragment_177.1575	: 0.01
fragment_201.1625	: 0.01
fragment_151.1075	: 0.01
fragment_163.1475	: 0.01
fragment_199.1425	: 0.01
fragment_83.0825	: 0.01
fragment_219.2075	: 0.01
fragment_215.1775	: 0.01
fragment_203.1725	: 0.01

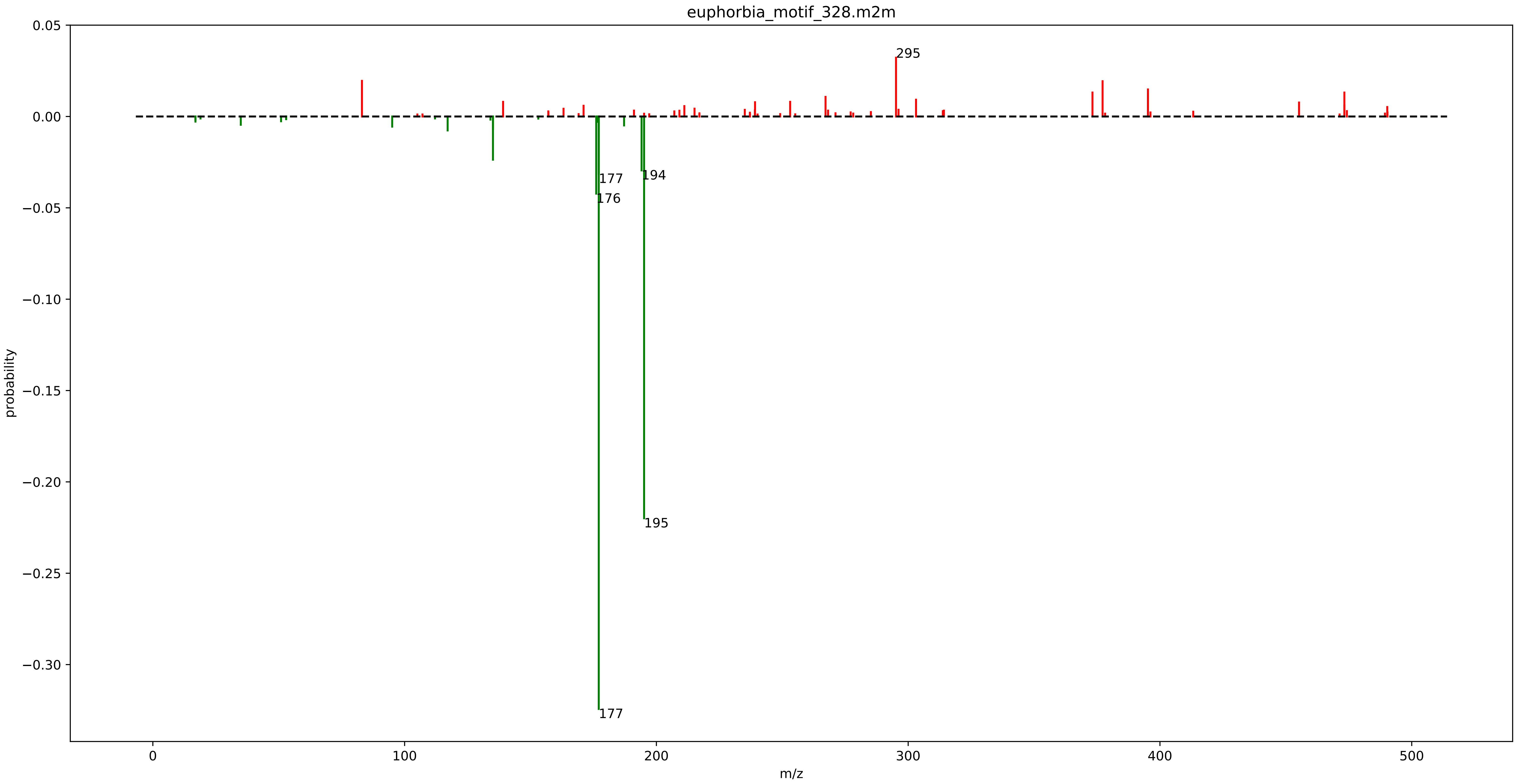

euphorbia_motif_328.m2m
0.05
0.00
−0.05
−0.10
−0.15
−0.20
−0.25
−0.30
probability
0
100
200
300
400
500
m/z
295
177
176
194
195
177

euphorbia_motif_328.m2m

loss_177.0975	: 0.32
loss_195.1125	: 0.22
loss_176.0975	: 0.04
fragment_295.1675	: 0.03
loss_177.1025	: 0.03
loss_194.1075	: 0.03
loss_135.0875	: 0.02
fragment_83.0475	: 0.02
fragment_377.2125	: 0.02
fragment_395.2225	: 0.01
fragment_373.2025	: 0.01
fragment_473.2475	: 0.01
fragment_395.2175	: 0.01
fragment_267.1725	: 0.01
fragment_303.1225	: 0.01
fragment_253.1175	: 0.01
fragment_139.1125	: 0.01
fragment_239.1775	: 0.01
loss_117.0775	: 0.01
fragment_455.2425	: 0.01
loss_135.0925	: 0.01
fragment_473.2525	: 0.01
fragment_171.0775	: 0.01
fragment_211.1075	: 0.01
loss_95.0625	: 0.01
fragment_490.2775	: 0.01

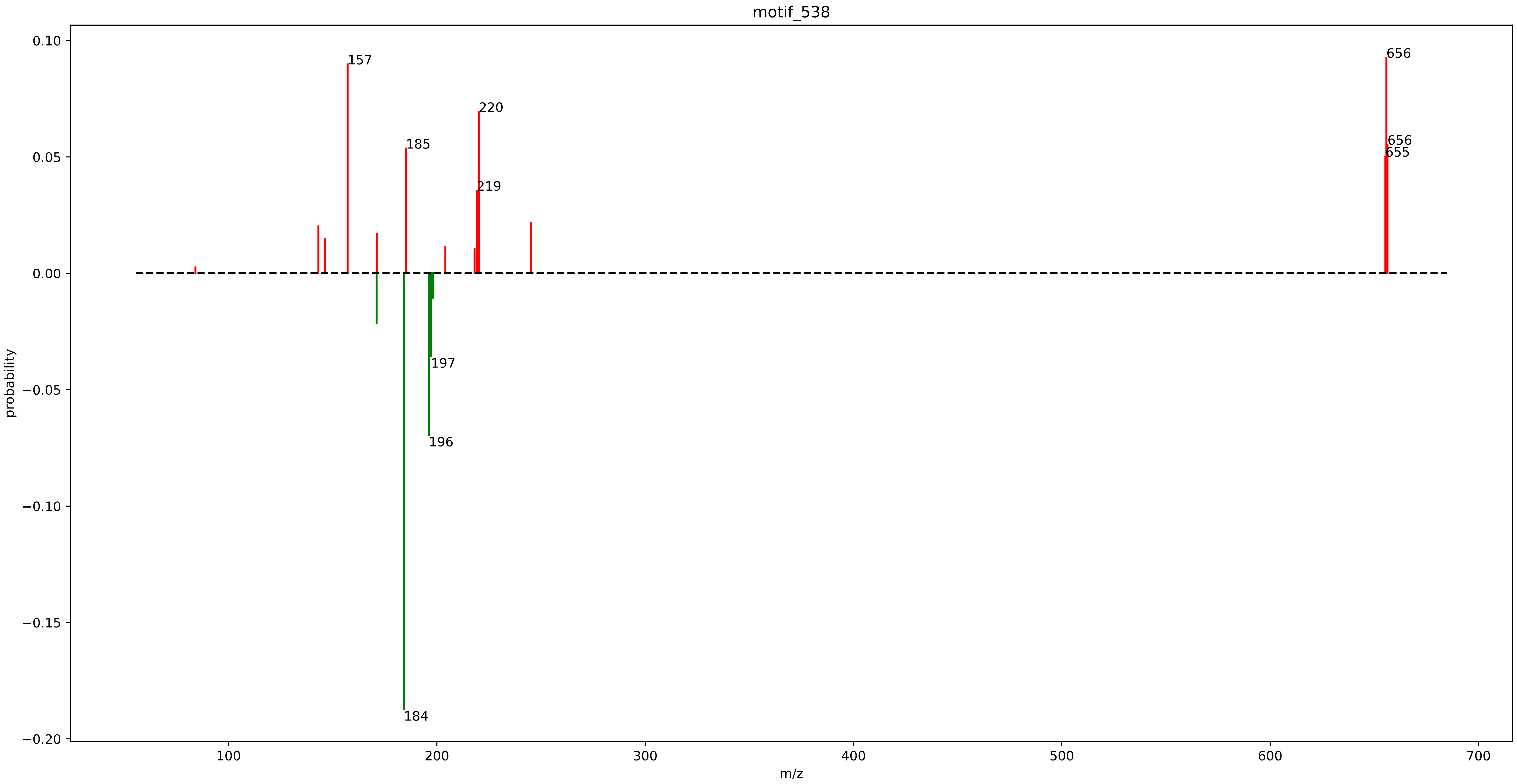

motif_538
0.10
0.05
0.00
−0.05
−0.10
−0.15
−0.20
probability
157
185
220
219
656
656
655
197
196
184
100
200
300
400
500
600
700
m/z

motif_538

loss_184.1175	: 0.19
fragment_655.8175	: 0.09
fragment_157.1325	: 0.09
loss_196.1075	: 0.07
fragment_220.0975	: 0.07
fragment_656.3175	: 0.06
fragment_185.1275	: 0.05
fragment_655.3175	: 0.05
fragment_219.0875	: 0.04
loss_197.1175	: 0.04
fragment_245.1825	: 0.02
loss_171.0175	: 0.02
fragment_143.1175	: 0.02
fragment_171.1125	: 0.02
fragment_146.1175	: 0.01
fragment_204.0675	: 0.01
loss_198.1225	: 0.01
fragment_218.0825	: 0.01
loss_198.1375	: 0.01

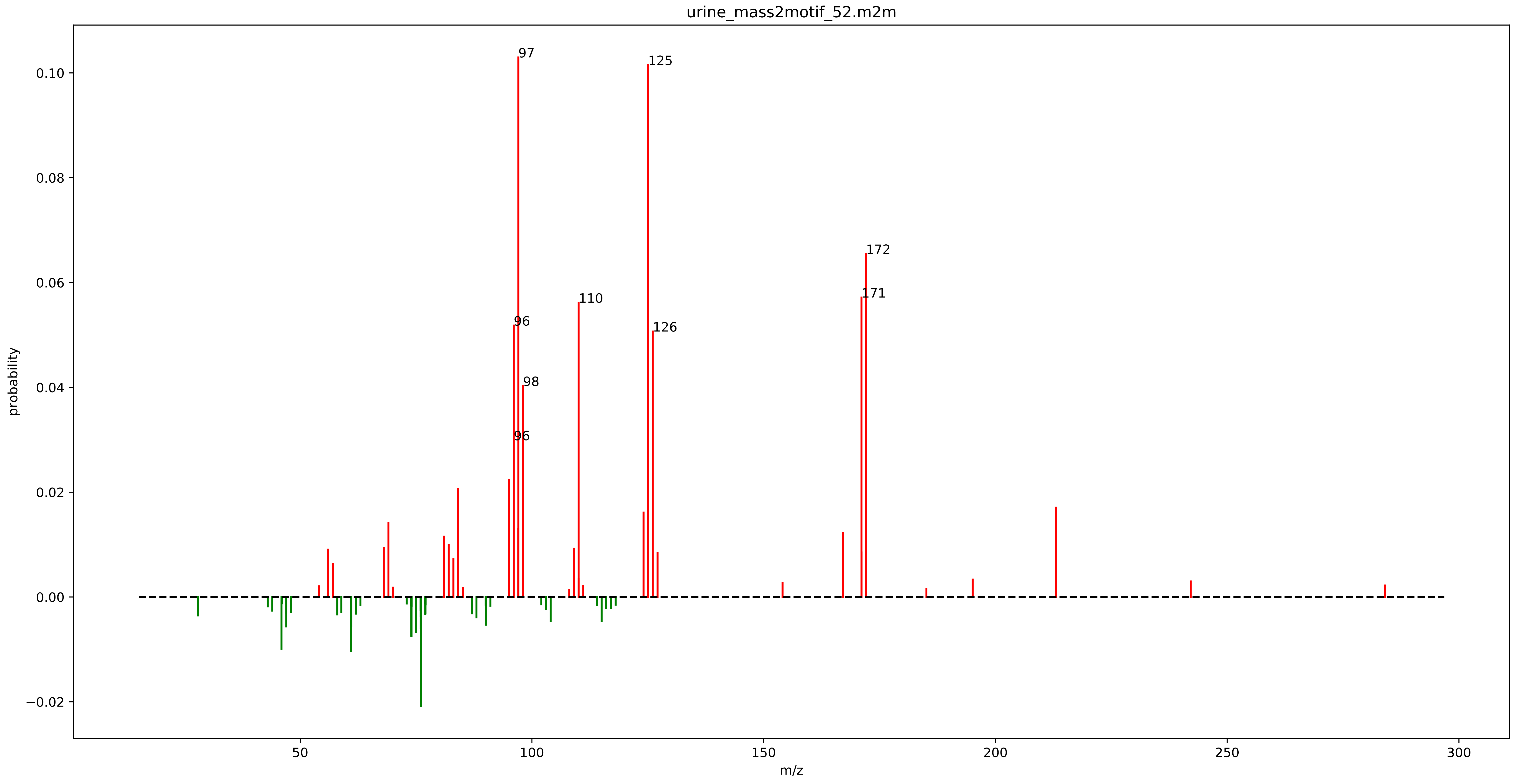

urine_mass2motif_52.m2m
probability
m/z
0.10
0.08
0.06
0.04
0.02
0.00
−0.02
50
100
150
200
250
300
97
125
172
171
110
126
96
98
96

urine_mass2motif_52.m2m

fragment_97.0725	: 0.10
fragment_125.0925	: 0.10
fragment_172.0825	: 0.07
fragment_171.0975	: 0.06
fragment_110.0775	: 0.06
fragment_96.0675	: 0.05
fragment_126.0725	: 0.05
fragment_98.0775	: 0.04
fragment_96.0625	: 0.03
fragment_95.0625	: 0.02
loss_76.0325	: 0.02
fragment_84.0625	: 0.02
fragment_213.1075	: 0.02
fragment_124.0875	: 0.02
fragment_69.0525	: 0.01
fragment_97.0625	: 0.01
fragment_167.1025	: 0.01
fragment_172.0975	: 0.01
fragment_81.0425	: 0.01
loss_61.0075	: 0.01
fragment_82.0475	: 0.01
loss_45.9675	: 0.01
fragment_68.0475	: 0.01
fragment_109.0775	: 0.01
fragment_56.0475	: 0.01
fragment_171.1125	: 0.01
fragment_127.1075	: 0.01
loss_74.0225	: 0.01
loss_73.9925	: 0.01
fragment_83.0625	: 0.01
fragment_125.0725	: 0.01
loss_74.9725	: 0.01
fragment_57.0525	: 0.01
loss_76.0075	: 0.01
loss_47.0025	: 0.01
loss_61.0325	: 0.01
loss_90.0475	: 0.01

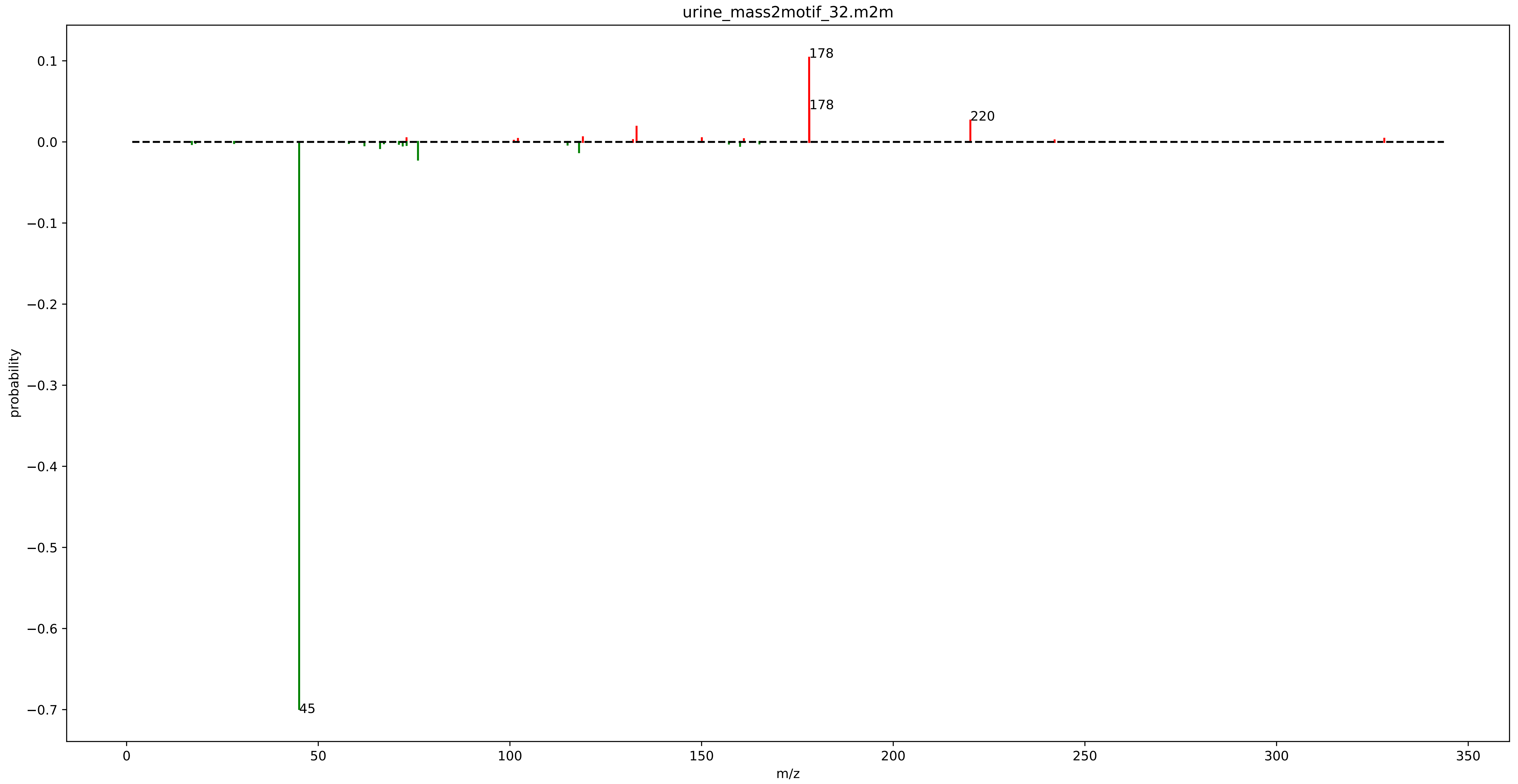

urine_mass2motif_32.m2m
probability
m/z
0.1
0.0
−0.1
−0.2
−0.3
−0.4
−0.5
−0.6
−0.7
0
50
100
150
200
250
300
350
178
178
220
45

urine_mass2motif_32.m2m

loss_45.0125	: 0.70
fragment_178.0575	: 0.10
fragment_178.1075	: 0.04
fragment_220.1175	: 0.03
loss_76.0175	: 0.02
fragment_133.0375	: 0.02
loss_118.0325	: 0.01
loss_66.1225	: 0.01
fragment_119.0375	: 0.01

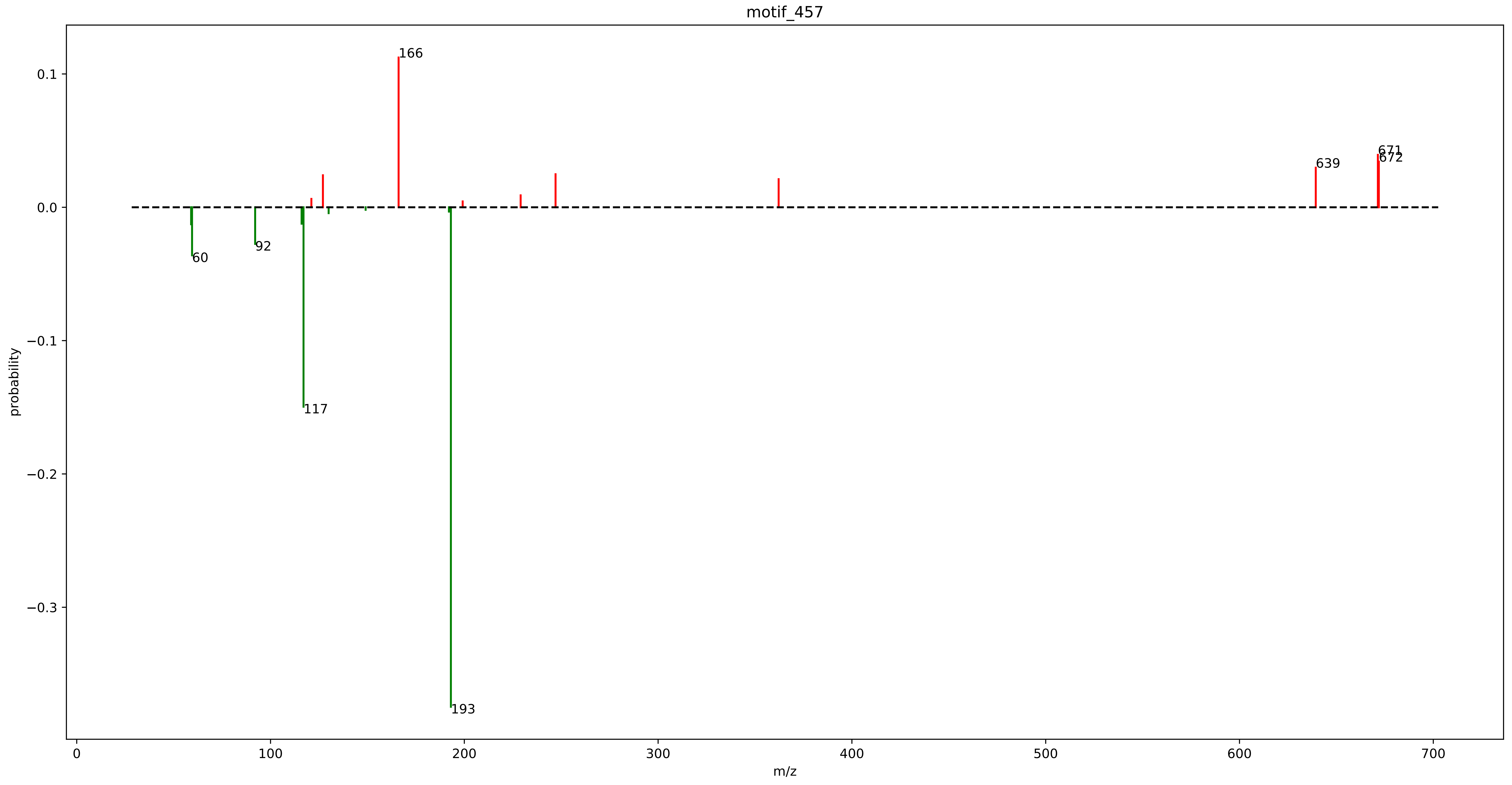

motif_457
166
639
671
672
60
92
117
193
probability
m/z
0.1
0.0
−0.1
−0.2
−0.3
0
100
200
300
400
500
600
700

motif_457

loss_193.0725	: 0.37
loss_117.0275	: 0.15
fragment_166.0875	: 0.11
fragment_671.4025	: 0.04
loss_59.5125	: 0.04
fragment_671.9025	: 0.03
fragment_639.3575	: 0.03
loss_92.0625	: 0.03
fragment_247.0775	: 0.02
fragment_127.0375	: 0.02
fragment_362.1925	: 0.02
loss_59.0225	: 0.01
loss_116.0275	: 0.01
fragment_229.0675	: 0.01
fragment_121.0825	: 0.01

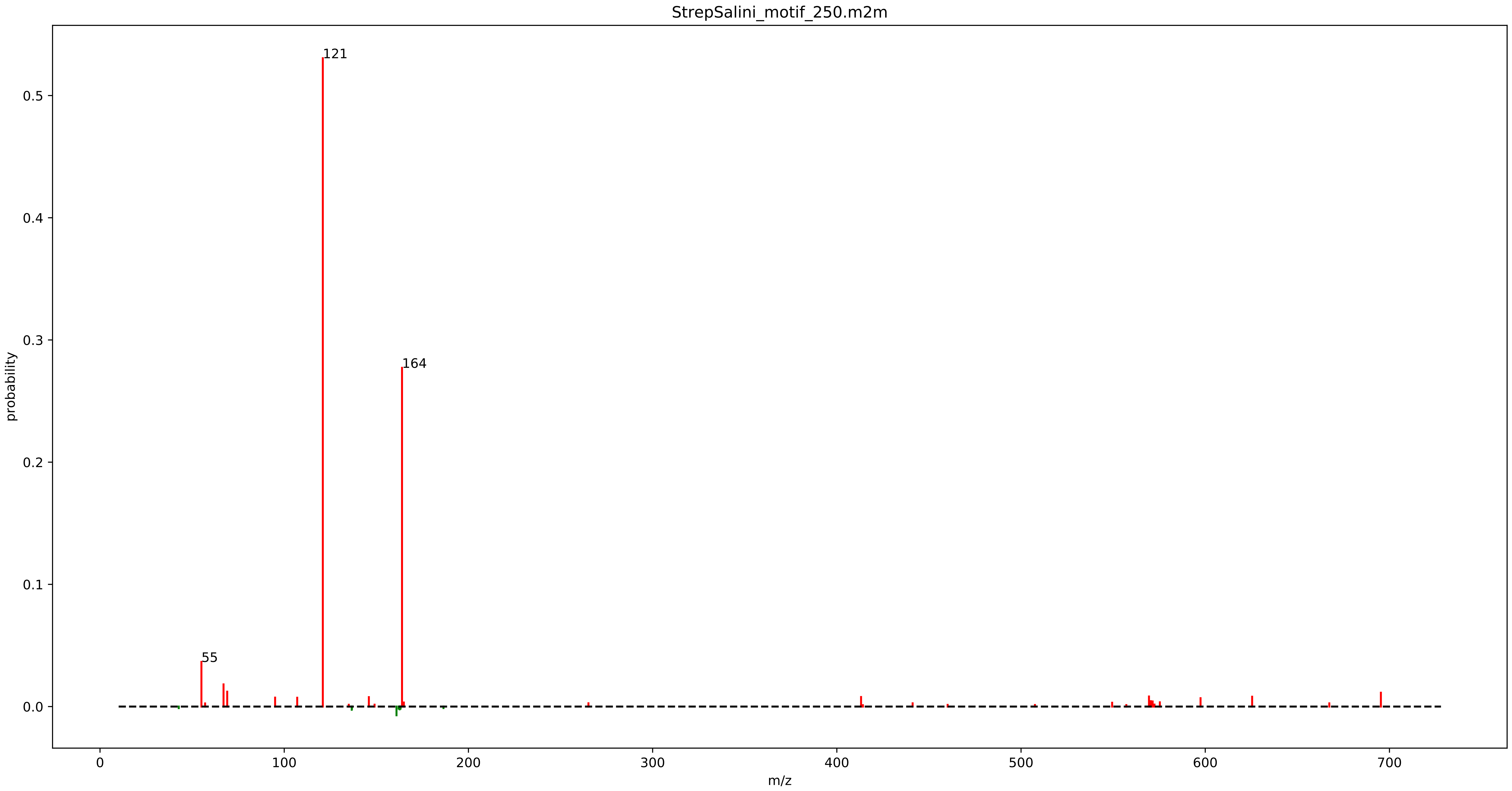

StrepSalini_motif_250.m2m
121
164
55
probability
0.5
0.4
0.3
0.2
0.1
0.0
0
100
200
300
400
500
600
700
m/z

StrepSalini_motif_250.m2m

fragment_120.9500	: 0.53
fragment_163.9500	: 0.28
fragment_55.0500	: 0.04
fragment_67.0500	: 0.02
fragment_69.0500	: 0.01
fragment_695.3500	: 0.01
fragment_569.4500	: 0.01
fragment_625.4500	: 0.01
fragment_413.1500	: 0.01
fragment_145.9500	: 0.01
fragment_95.0500	: 0.01
fragment_107.0500	: 0.01
loss_160.9500	: 0.01
fragment_597.4500	: 0.01

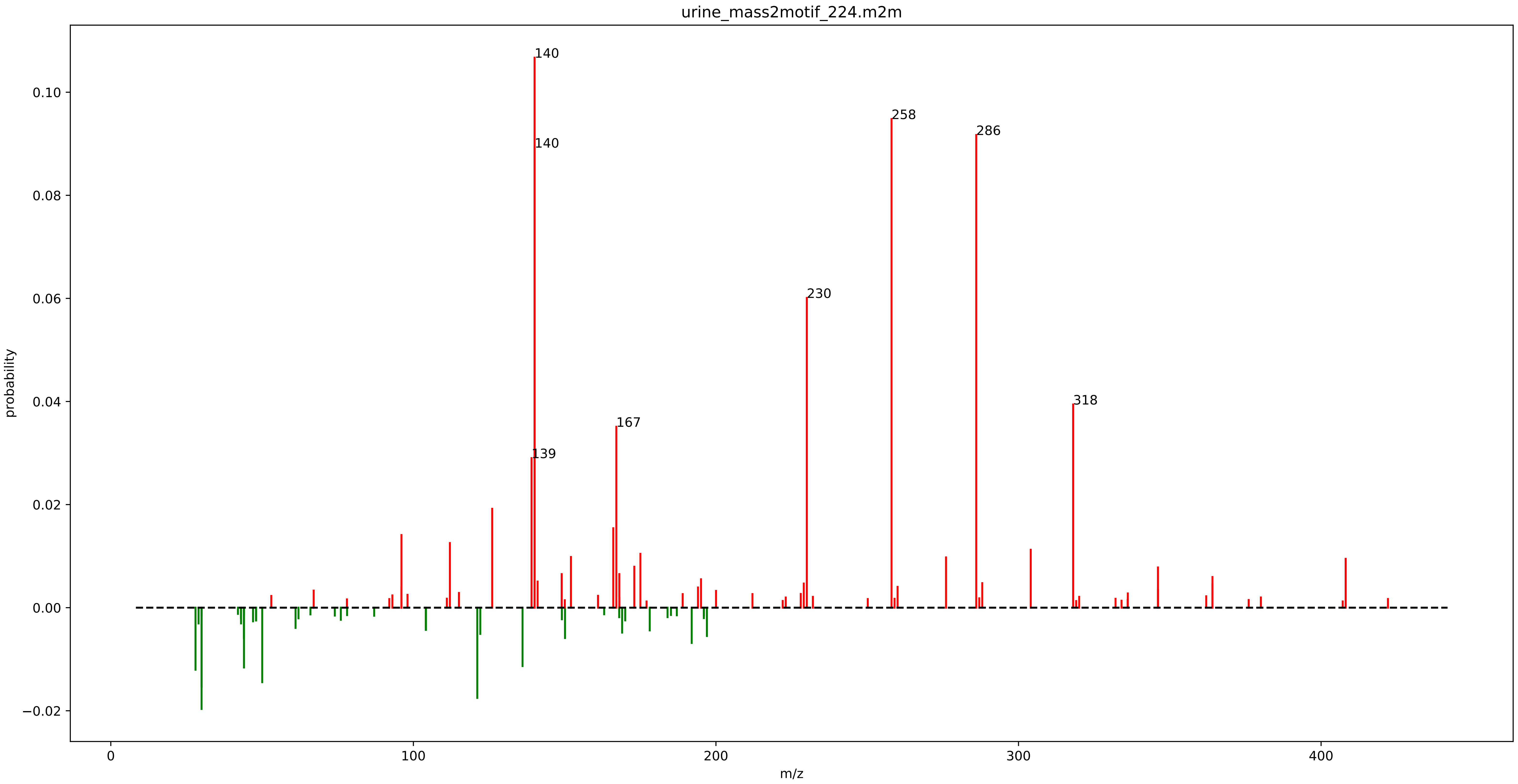

urine_mass2motif_224.m2m
probability
m/z
0.10
0.08
0.06
0.04
0.02
0.00
−0.02
0
100
200
300
400
140
140
139
167
230
258
286
318

urine_mass2motif_224.m2m

fragment_140.0525	: 0.11
fragment_258.0325	: 0.09
fragment_286.0275	: 0.09
fragment_140.0625	: 0.09
fragment_230.0375	: 0.06
fragment_318.0525	: 0.04
fragment_167.0775	: 0.04
fragment_139.0525	: 0.03
loss_29.9975	: 0.02
fragment_126.0475	: 0.02
loss_121.1125	: 0.02
fragment_166.0675	: 0.02
loss_30.0025	: 0.02
loss_50.0375	: 0.01
fragment_96.0675	: 0.01
fragment_112.0675	: 0.01
loss_27.9925	: 0.01
loss_44.0125	: 0.01
loss_136.0775	: 0.01
fragment_304.0375	: 0.01
fragment_175.0325	: 0.01
fragment_152.0675	: 0.01
fragment_276.0425	: 0.01
fragment_408.1175	: 0.01
fragment_173.0175	: 0.01
fragment_346.0825	: 0.01
loss_191.9825	: 0.01
fragment_168.0625	: 0.01
fragment_149.0125	: 0.01
fragment_364.0975	: 0.01
loss_150.1125	: 0.01
loss_43.9875	: 0.01
fragment_195.0675	: 0.01
loss_197.0125	: 0.01
loss_122.1125	: 0.01
fragment_141.0625	: 0.01

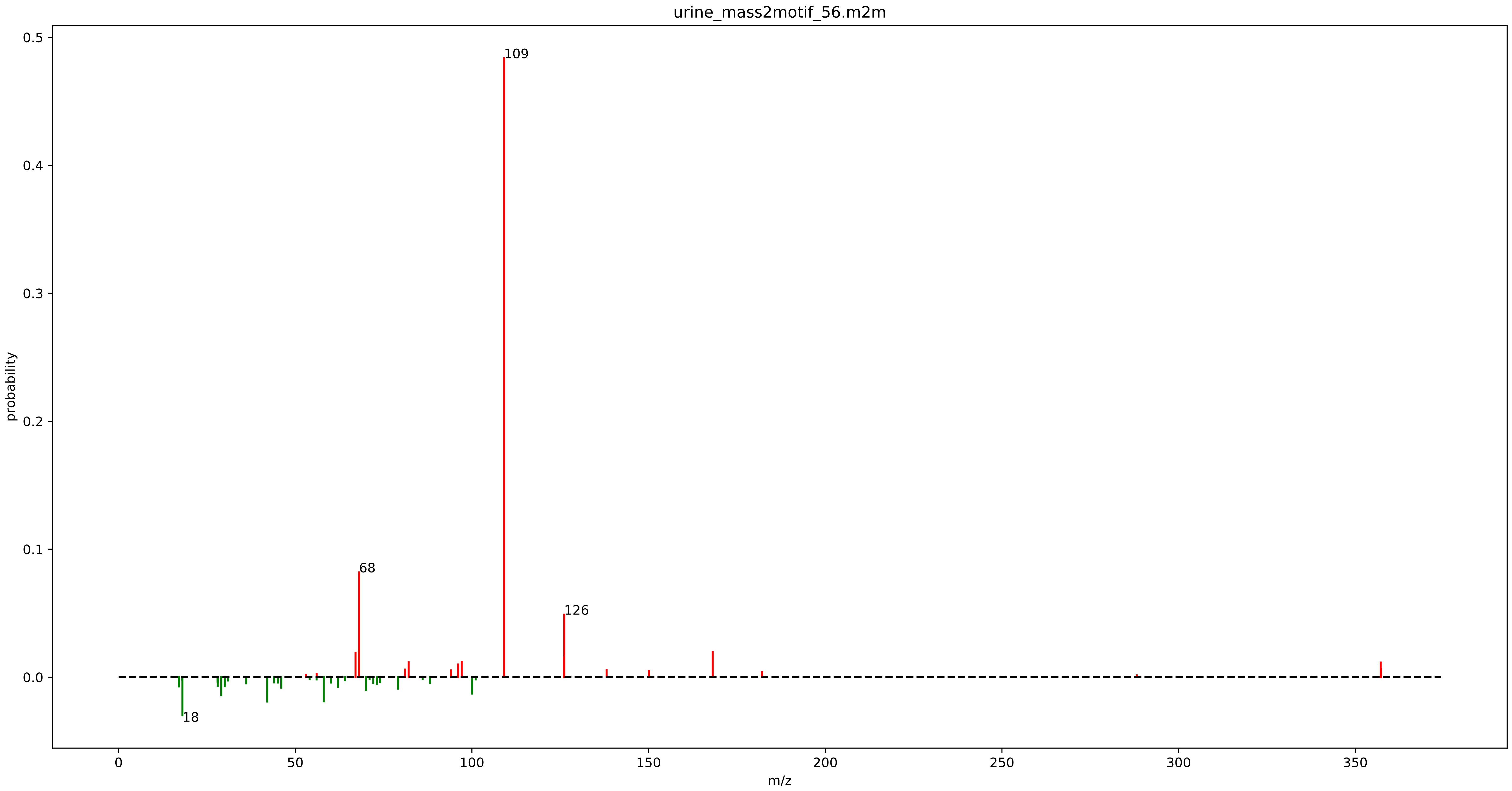
urine_mass2motif_56.m2m
probability
m/z
0.5
0.4
0.3
0.2
0.1
0.0
0
50
100
150
200
250
300
350
109
68
126
18

urine_mass2motif_56.m2m

fragment_109.0775	: 0.48
fragment_68.0475	: 0.08
fragment_126.1025	: 0.05
loss_18.0575	: 0.03
fragment_168.1125	: 0.02
fragment_67.0425	: 0.02
loss_42.0575	: 0.02
loss_58.0525	: 0.02
fragment_126.0525	: 0.02
loss_29.0275	: 0.01
loss_100.0625	: 0.01
fragment_97.0775	: 0.01
fragment_82.0675	: 0.01
fragment_357.1825	: 0.01
loss_42.0325	: 0.01
loss_70.0475	: 0.01
fragment_96.0675	: 0.01
loss_79.0525	: 0.01
loss_46.0375	: 0.01
loss_62.0475	: 0.01
loss_17.0275	: 0.01
loss_30.0225	: 0.01
loss_28.0425	: 0.01
fragment_357.2425	: 0.01
fragment_81.0425	: 0.01
fragment_138.1025	: 0.01
loss_30.0325	: 0.01
fragment_94.0525	: 0.01
loss_73.0525	: 0.01

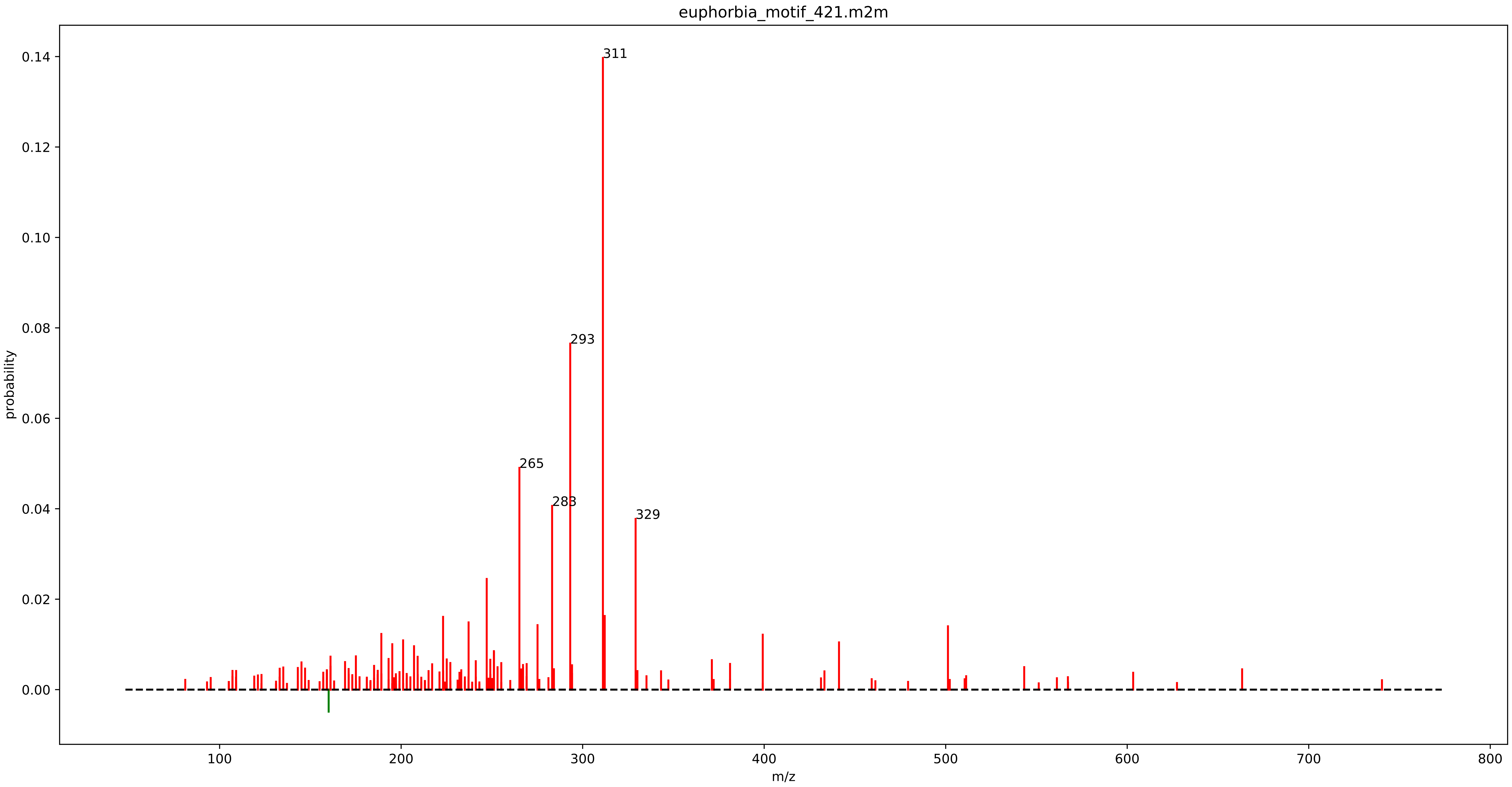

euphorbia_motif_421.m2m
0.14
0.12
0.10
0.08
0.06
0.04
0.02
0.00
probability
311
293
265
283
329
100
200
300
400
500
600
700
800
m/z

euphorbia_motif_421.m2m

fragment_311.1625	: 0.14
fragment_293.1525	: 0.08
fragment_265.1575	: 0.05
fragment_283.1675	: 0.04
fragment_329.1725	: 0.04
fragment_247.1475	: 0.02
fragment_312.1675	: 0.02
fragment_223.1075	: 0.02
fragment_237.1625	: 0.01
fragment_275.1425	: 0.01
fragment_501.2475	: 0.01
fragment_189.0925	: 0.01
fragment_399.2175	: 0.01
fragment_201.0875	: 0.01
fragment_441.2275	: 0.01
fragment_195.1175	: 0.01
fragment_207.1075	: 0.01
fragment_251.1075	: 0.01
fragment_251.1425	: 0.01
fragment_175.0725	: 0.01
fragment_161.0925	: 0.01
fragment_209.0975	: 0.01
fragment_193.0875	: 0.01
fragment_225.1225	: 0.01
fragment_249.1275	: 0.01
fragment_371.1825	: 0.01
fragment_241.1225	: 0.01
fragment_169.1025	: 0.01
fragment_145.1025	: 0.01
fragment_227.1075	: 0.01
fragment_255.1725	: 0.01
fragment_381.2025	: 0.01
fragment_269.1525	: 0.01
fragment_217.0875	: 0.01
fragment_267.1325	: 0.01
fragment_294.1575	: 0.01
fragment_185.0975	: 0.01
fragment_161.0975	: 0.01

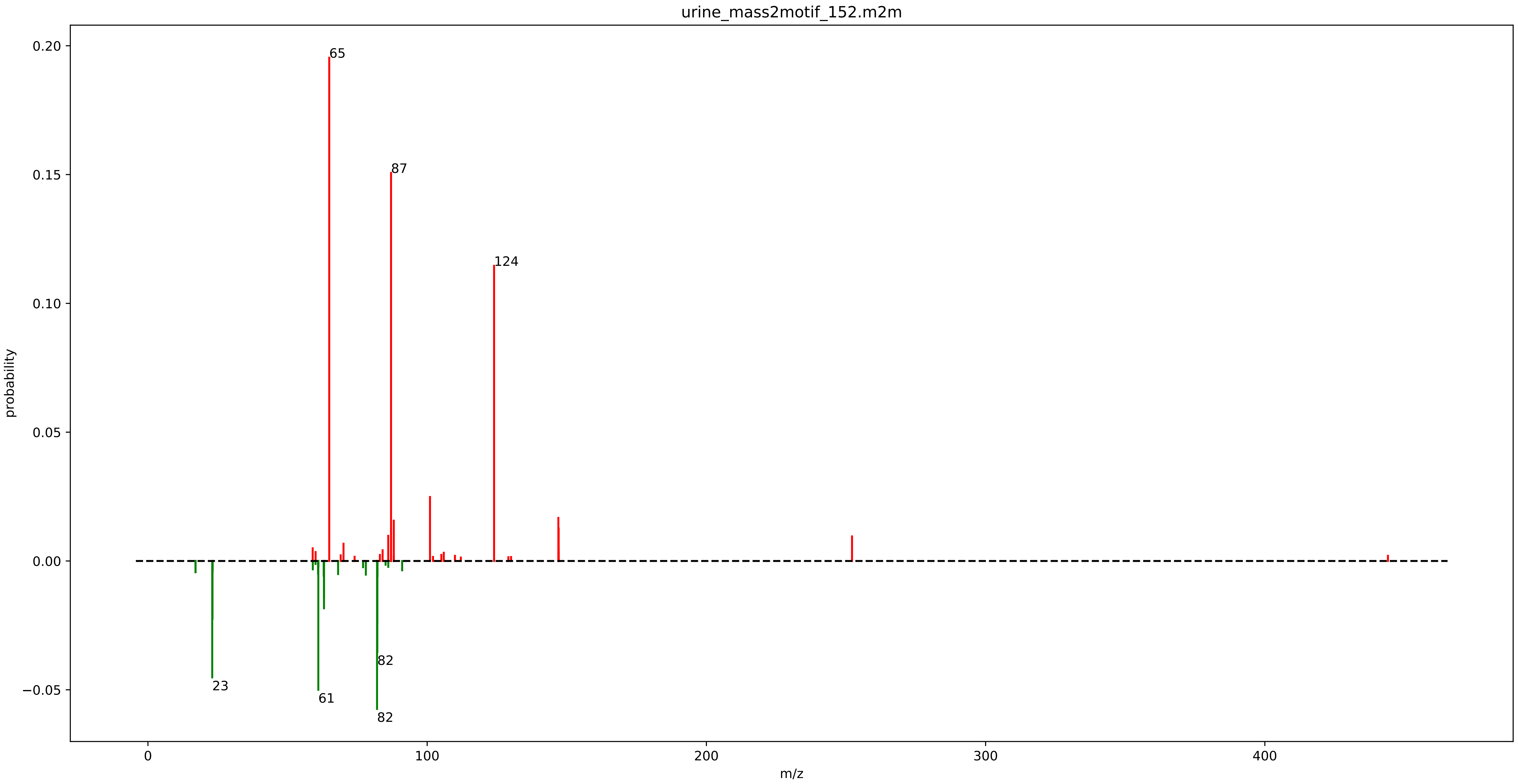
urine_mass2motif_152.m2m
probability
m/z
0.20
0.15
0.10
0.05
0.00
−0.05
0
100
200
300
400
65
87
124
23
61
82
82

urine_mass2motif_152.m2m

fragment_64.9275	: 0.20
fragment_87.0625	: 0.15
fragment_123.9625	: 0.11
loss_82.0525	: 0.06
loss_61.0175	: 0.05
loss_23.0175	: 0.05
loss_82.1575	: 0.04
fragment_101.0175	: 0.02
loss_82.1475	: 0.02
loss_23.1225	: 0.02
loss_63.0475	: 0.02
fragment_146.9825	: 0.02
fragment_88.0475	: 0.02
loss_63.0525	: 0.01
fragment_147.0975	: 0.01
fragment_87.0575	: 0.01
fragment_86.0575	: 0.01
fragment_252.1575	: 0.01
loss_82.0975	: 0.01
fragment_70.0375	: 0.01
loss_82.2225	: 0.01
loss_82.1375	: 0.01
loss_62.9375	: 0.01
loss_78.0375	: 0.01
fragment_87.0425	: 0.01
loss_68.1025	: 0.01

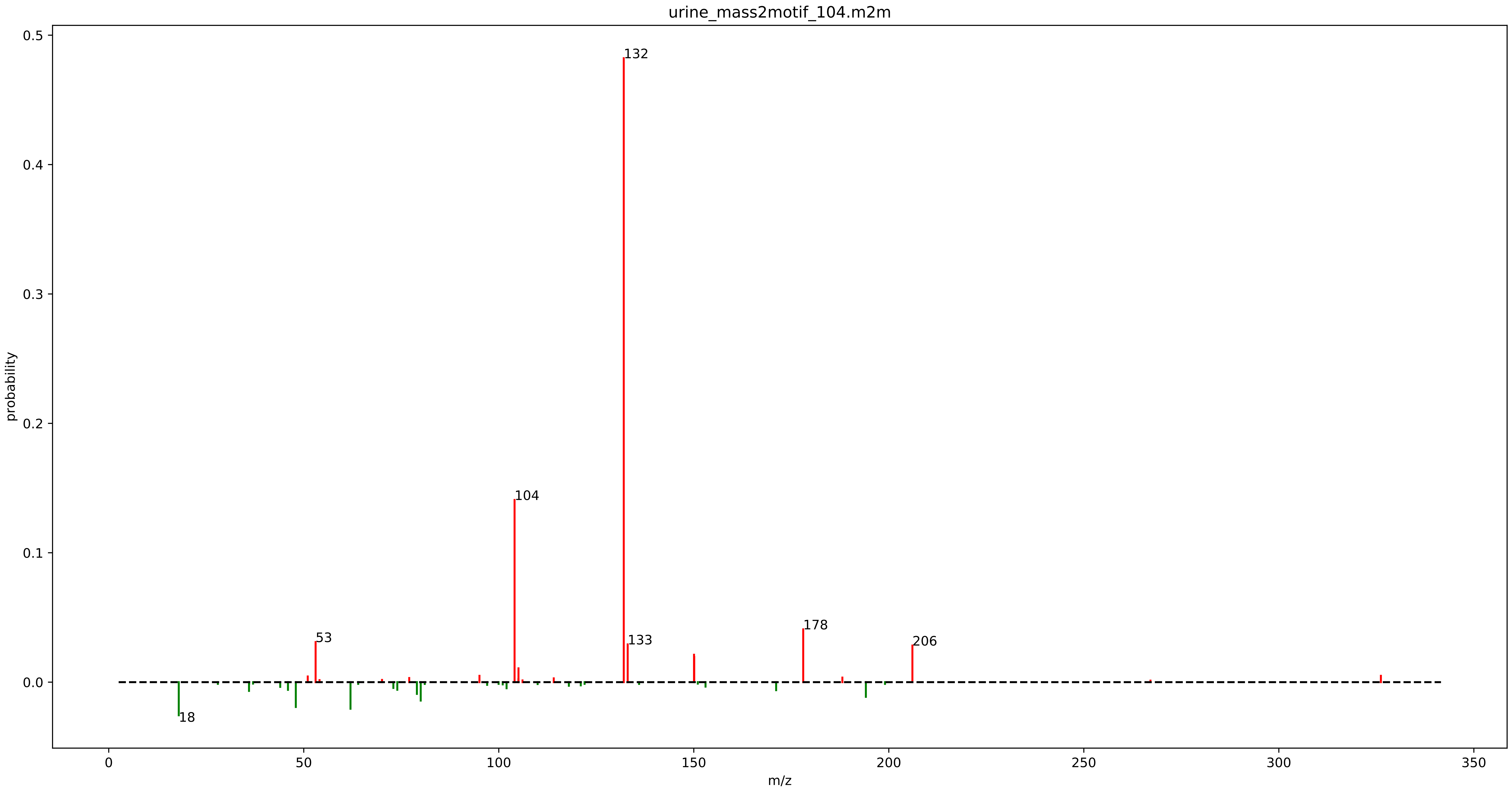
urine_mass2motif_104.m2m
probability
m/z
132
104
53
133
178
206
18
0.5
0.4
0.3
0.2
0.1
0.0
0
50
100
150
200
250
300
350

urine_mass2motif_104.m2m

fragment_132.0425	: 0.48
fragment_104.0475	: 0.14
fragment_178.0525	: 0.04
fragment_53.0375	: 0.03
fragment_133.0525	: 0.03
fragment_206.0475	: 0.03
loss_17.9525	: 0.03
fragment_150.0575	: 0.02
loss_61.9825	: 0.02
fragment_150.1125	: 0.02
loss_47.9625	: 0.02
loss_79.9875	: 0.01
loss_194.1175	: 0.01
fragment_105.0525	: 0.01
loss_79.0075	: 0.01
loss_35.9625	: 0.01
loss_171.1125	: 0.01
loss_45.9525	: 0.01
loss_73.9825	: 0.01

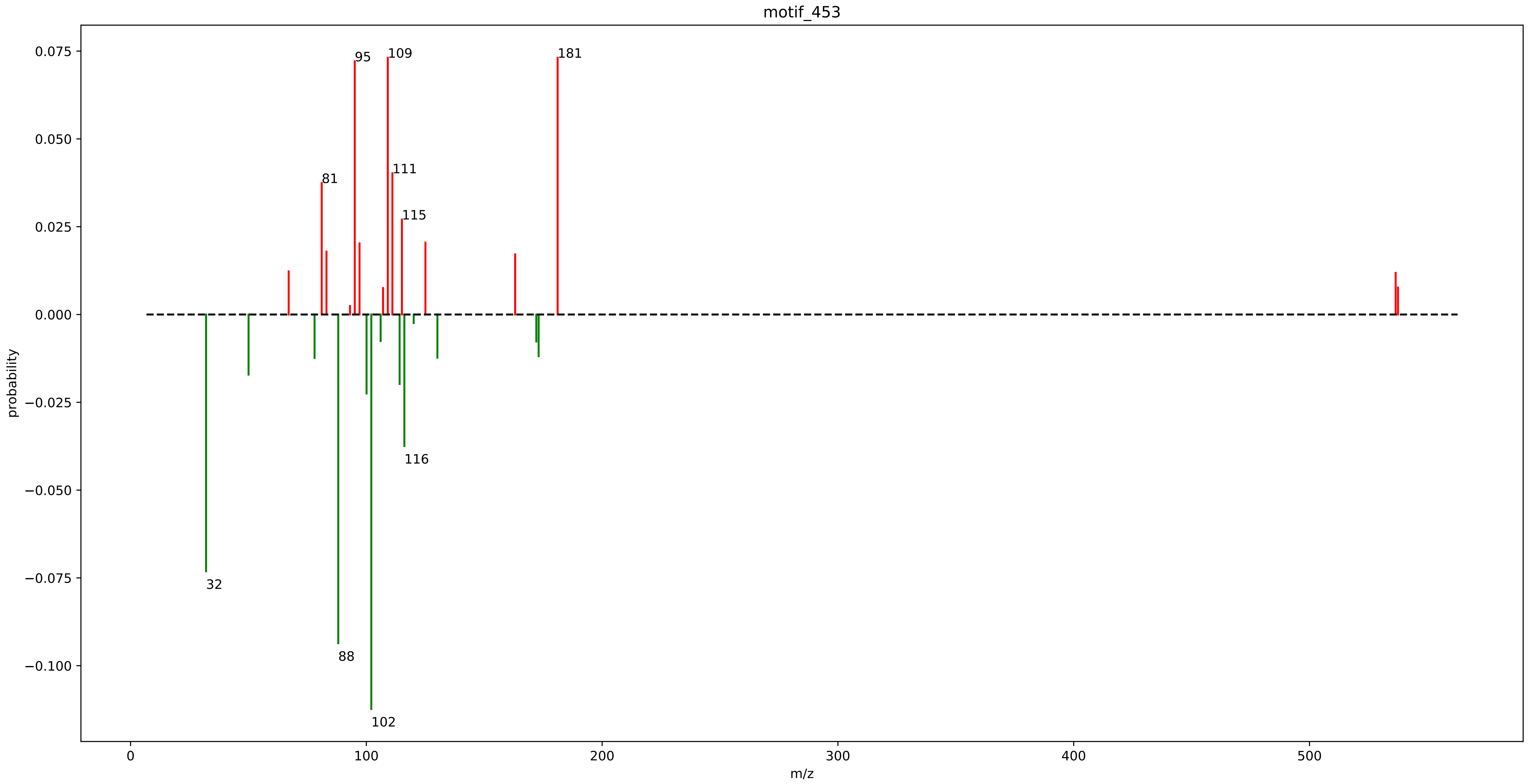

motif_453
95
109
181
81
111
115
116
32
88
102
0.075
0.050
0.025
0.000
−0.025
−0.050
−0.075
−0.100
probability
0
100
200
300
400
500
m/z

motif_453

loss_102.1025	: 0.11
loss_88.0875	: 0.09
fragment_109.0975	: 0.07
loss_32.0275	: 0.07
fragment_181.1225	: 0.07
fragment_95.0825	: 0.07
fragment_111.0425	: 0.04
loss_116.1175	: 0.04
fragment_81.0675	: 0.04
fragment_115.0875	: 0.03
loss_100.0875	: 0.02
fragment_125.0575	: 0.02
fragment_97.1025	: 0.02
loss_114.1025	: 0.02
fragment_83.0825	: 0.02
fragment_163.1125	: 0.02
loss_50.0375	: 0.02
loss_78.0325	: 0.01
fragment_67.0525	: 0.01
loss_130.1325	: 0.01
loss_173.0675	: 0.01
fragment_536.5375	: 0.01
loss_172.0775	: 0.01
fragment_537.5325	: 0.01
loss_106.0625	: 0.01
fragment_107.0825	: 0.01

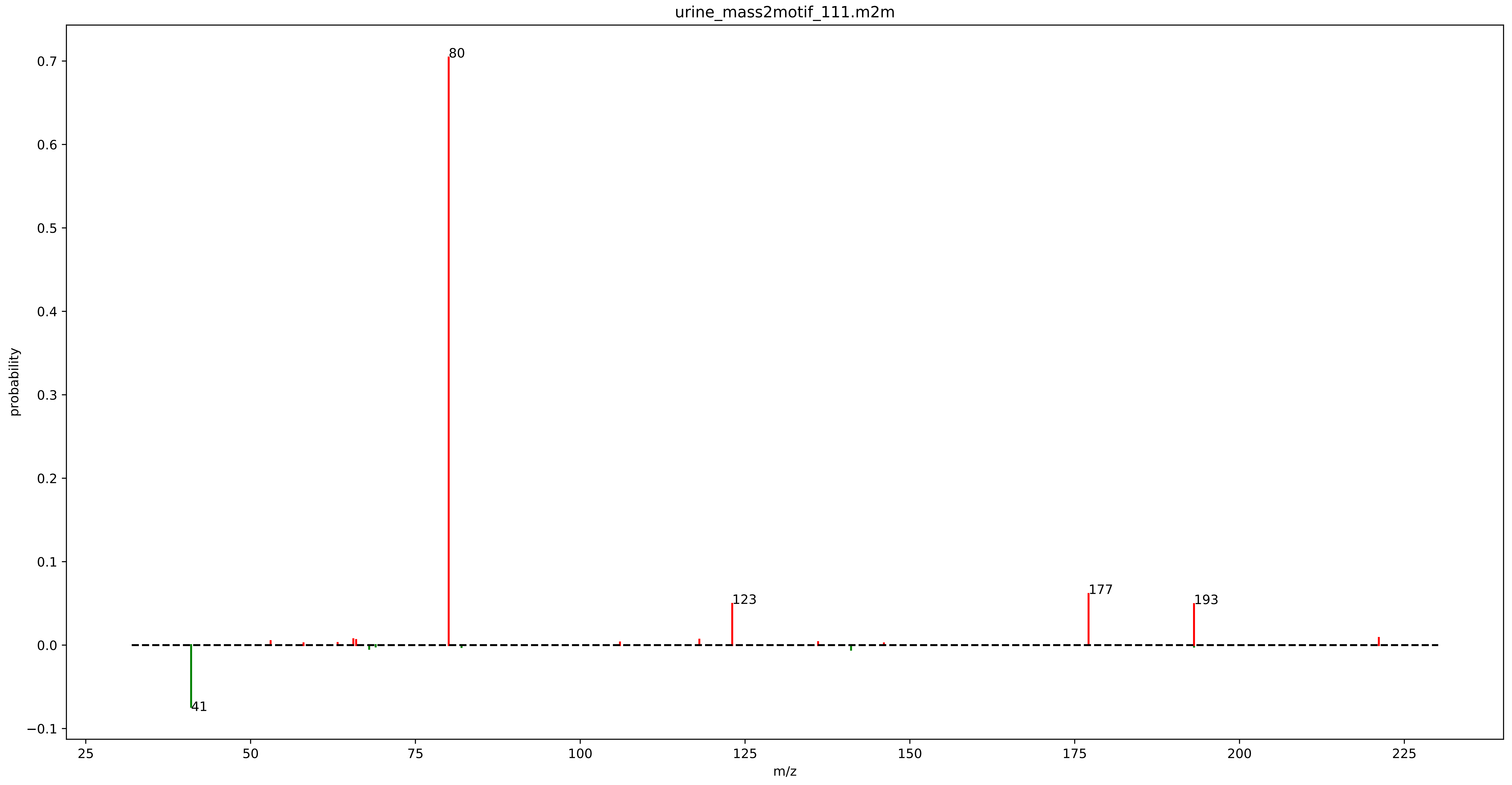

urine_mass2motif_111.m2m
probability
m/z
80
123
177
193
41
0.7
0.6
0.5
0.4
0.3
0.2
0.1
0.0
−0.1
25
50
75
100
125
150
175
200
225

urine_mass2motif_111.m2m

fragment_80.0475	: 0.70
loss_40.9775	: 0.07
fragment_177.1025	: 0.06
fragment_123.0525	: 0.05
fragment_193.0975	: 0.05
fragment_221.1325	: 0.01
fragment_65.5825	: 0.01
fragment_118.0675	: 0.01
fragment_66.0075	: 0.01
loss_141.0775	: 0.01

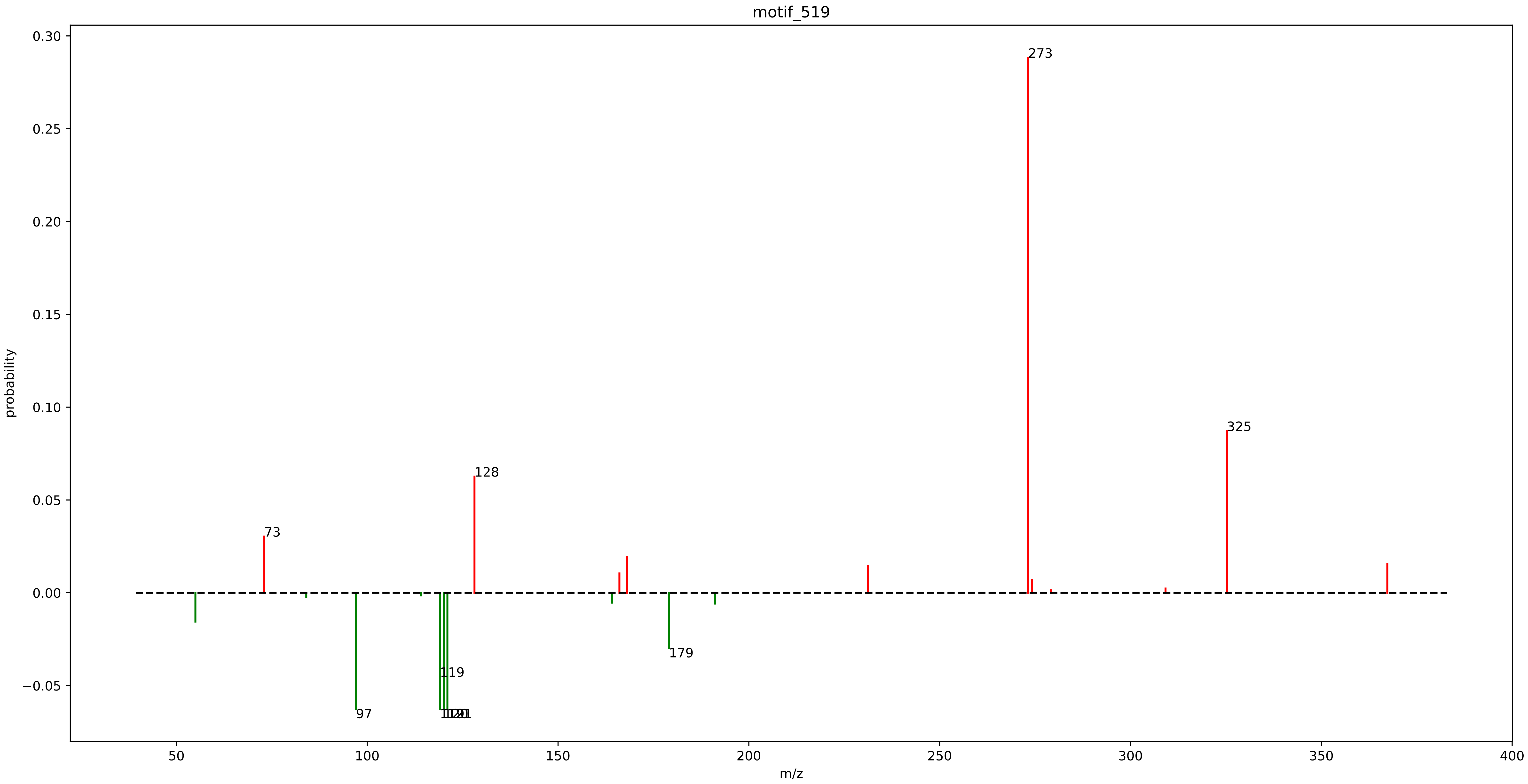
motif_519
probability
m/z
273
325
128
73
179
119
97
0.30
0.25
0.20
0.15
0.10
0.05
0.00
−0.05
50
100
150
200
250
300
350
400

motif_519

fragment_273.1775	: 0.29
fragment_325.2525	: 0.09
fragment_128.1075	: 0.06
loss_121.0175	: 0.06
loss_119.0425	: 0.06
loss_97.0325	: 0.06
loss_120.0425	: 0.06
loss_119.0375	: 0.04
fragment_73.0275	: 0.03
loss_179.0575	: 0.03
fragment_168.0625	: 0.02
loss_54.9875	: 0.02
fragment_367.2975	: 0.02
fragment_231.1675	: 0.01
fragment_166.0875	: 0.01
fragment_274.1825	: 0.01
loss_191.0925	: 0.01
loss_164.1025	: 0.01

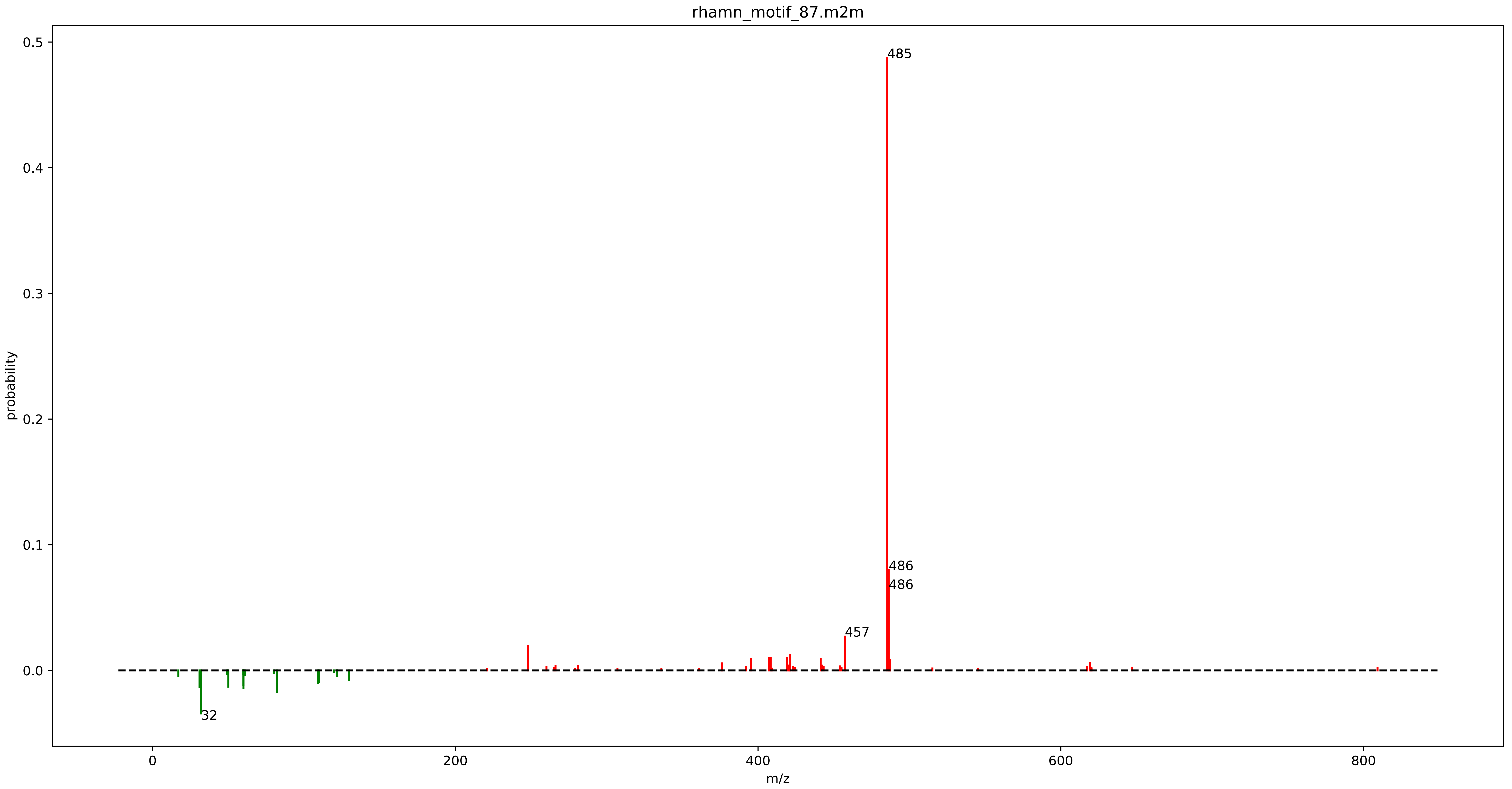

rhamn_motif_87.m2m
probability
m/z
0.5
0.4
0.3
0.2
0.1
0.0
0
200
400
600
800
485
486
486
457
32

rhamn_motif_87.m2m

fragment_485.3275	: 0.49
fragment_486.3325	: 0.08
fragment_486.3275	: 0.06
loss_32.0225	: 0.03
fragment_457.3275	: 0.03
fragment_248.1425	: 0.02
loss_82.0275	: 0.02
loss_60.0275	: 0.01
loss_31.0175	: 0.01
loss_50.0375	: 0.01
fragment_421.3075	: 0.01
loss_60.0075	: 0.01
fragment_457.3425	: 0.01
fragment_407.3025	: 0.01
fragment_419.1825	: 0.01
loss_109.0525	: 0.01
fragment_408.3025	: 0.01
loss_110.0525	: 0.01
fragment_441.3325	: 0.01
fragment_395.3325	: 0.01
fragment_487.3325	: 0.01
loss_129.9875	: 0.01
fragment_619.3025	: 0.01
fragment_376.1375	: 0.01

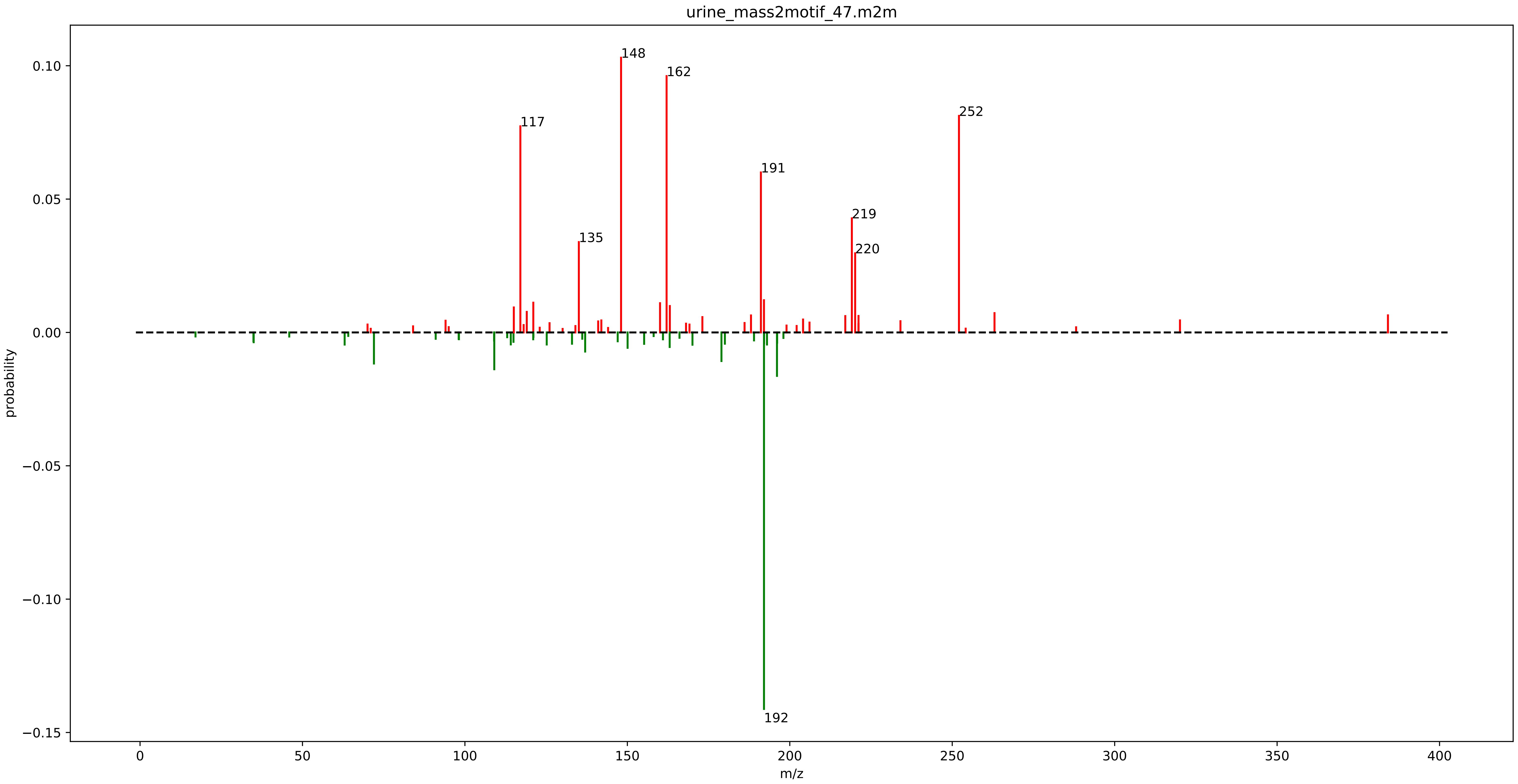

urine_mass2motif_47.m2m
probability
m/z
0.10
0.05
0.00
−0.05
−0.10
−0.15
0
50
100
150
200
250
300
350
400
148
162
117
252
191
135
219
220
192

urine_mass2motif_47.m2m

loss_192.0575	: 0.14
fragment_148.0625	: 0.10
fragment_162.0775	: 0.10
fragment_252.0725	: 0.08
fragment_117.0675	: 0.08
fragment_191.1075	: 0.06
fragment_219.1025	: 0.04
fragment_135.0675	: 0.03
fragment_220.1175	: 0.03
loss_196.0575	: 0.02
loss_109.0375	: 0.01
fragment_192.0525	: 0.01
loss_71.9975	: 0.01
fragment_121.0525	: 0.01
fragment_160.0625	: 0.01
loss_178.9675	: 0.01
fragment_163.0775	: 0.01
fragment_115.0525	: 0.01
fragment_119.0375	: 0.01
fragment_263.0125	: 0.01
loss_137.0125	: 0.01
fragment_384.1175	: 0.01
fragment_188.0575	: 0.01
fragment_221.1525	: 0.01
fragment_217.0825	: 0.01
loss_150.0725	: 0.01
fragment_173.0925	: 0.01
loss_163.0275	: 0.01

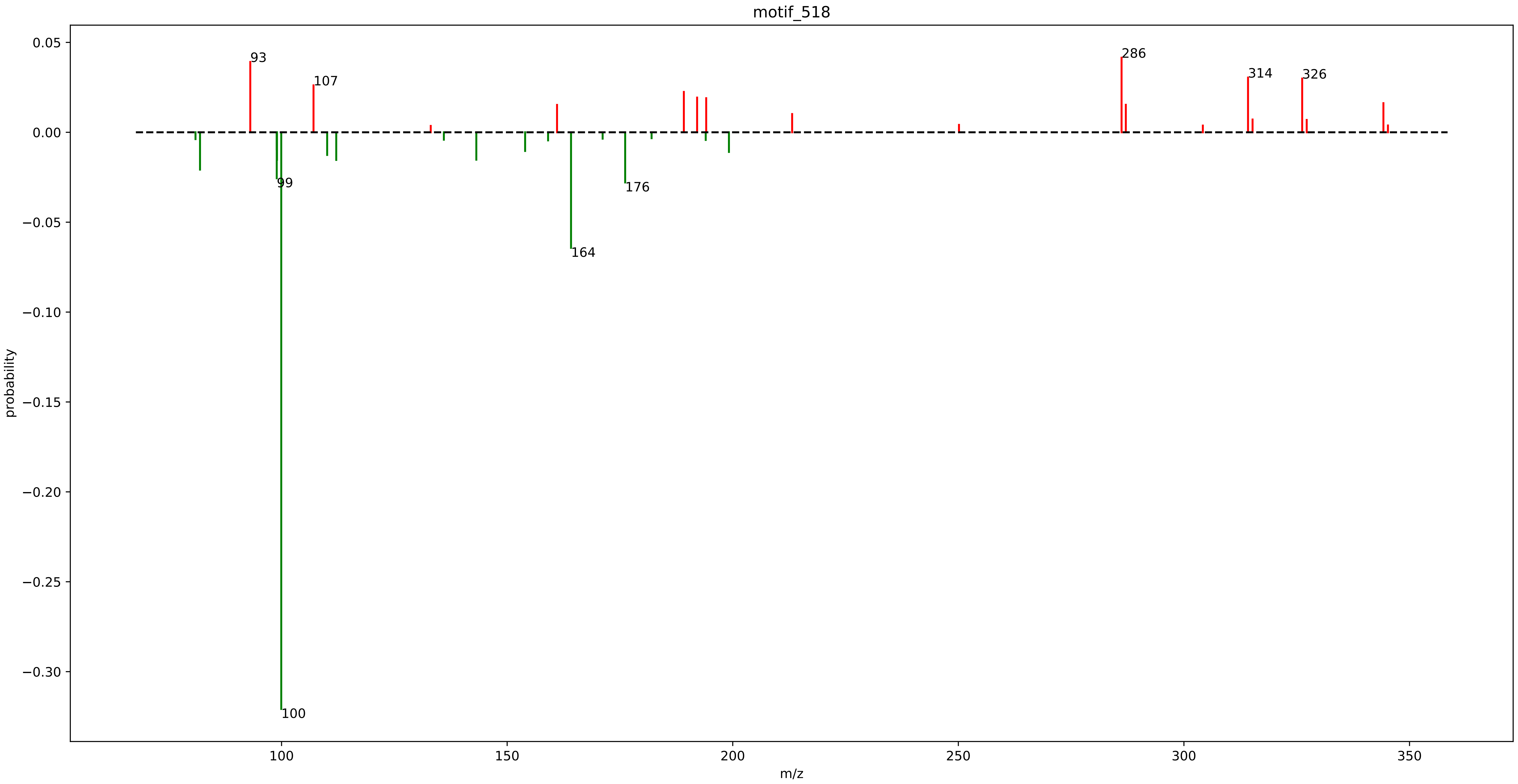

motif_518
probability
m/z
0.05
0.00
−0.05
−0.10
−0.15
−0.20
−0.25
−0.30
100
150
200
250
300
350
93
107
286
314
326
99
176
164
100

motif_518

loss_99.9325	: 0.32
loss_164.1575	: 0.06
fragment_286.1775	: 0.04
fragment_93.0675	: 0.04
fragment_314.2075	: 0.03
fragment_326.2075	: 0.03
loss_176.1575	: 0.03
fragment_107.0825	: 0.03
loss_98.9325	: 0.03
fragment_189.1625	: 0.02
loss_81.9275	: 0.02
fragment_192.1025	: 0.02
fragment_194.1175	: 0.02
fragment_344.2125	: 0.02
loss_112.1225	: 0.02
loss_98.9875	: 0.02
fragment_287.1225	: 0.02
fragment_161.0575	: 0.02
loss_143.1675	: 0.02
loss_110.1075	: 0.01
loss_199.1575	: 0.01
loss_153.9825	: 0.01
fragment_213.1625	: 0.01
loss_98.9275	: 0.01
fragment_315.2125	: 0.01
fragment_327.2125	: 0.01

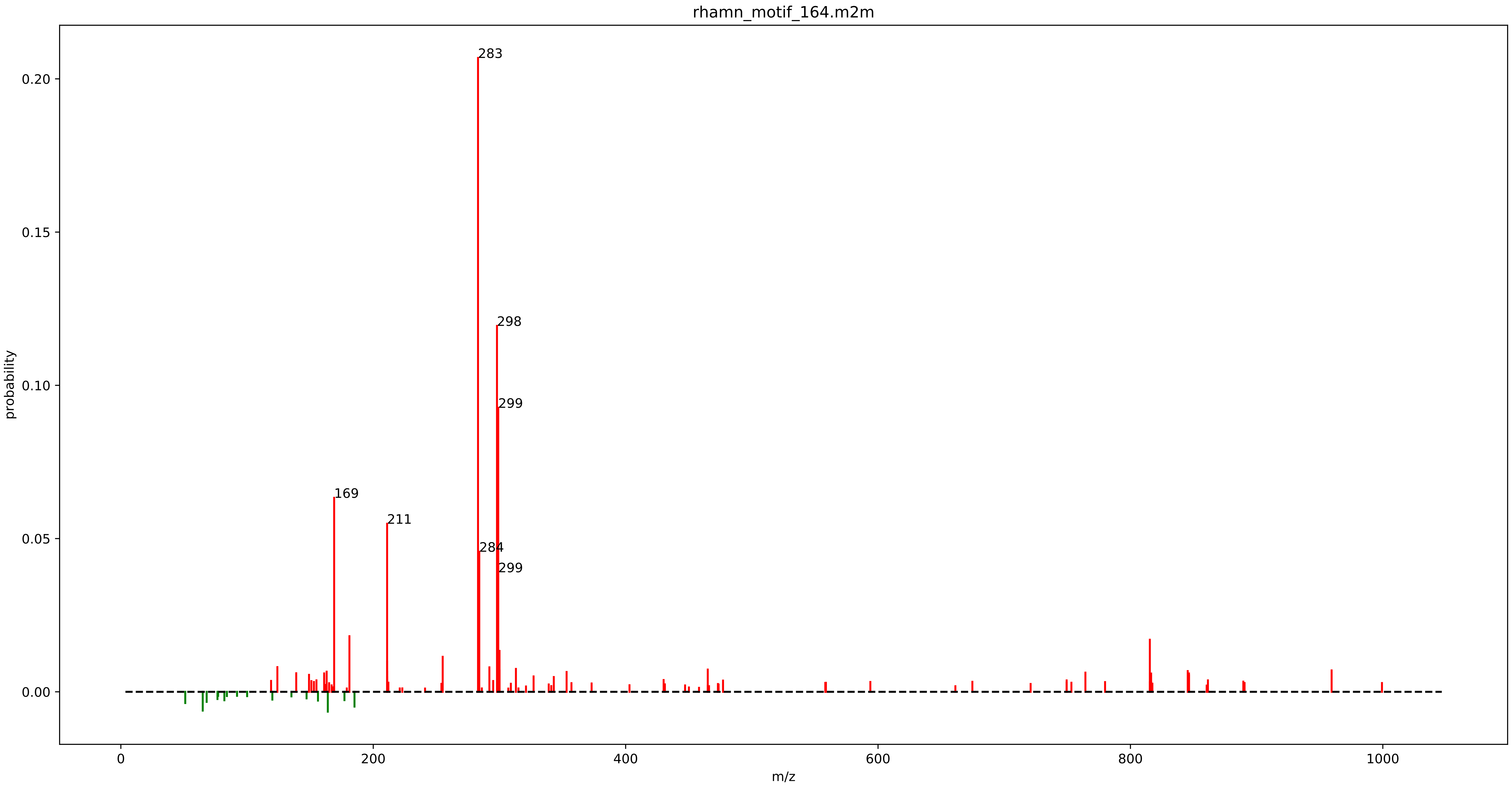
rhamn_motif_164.m2m
probability
m/z
283
298
299
169
211
284
299
0.20
0.15
0.10
0.05
0.00
0
200
400
600
800
1000

rhamn_motif_164.m2m

fragment_283.0225	: 0.21
fragment_298.0475	: 0.12
fragment_299.0575	: 0.09
fragment_169.0175	: 0.06
fragment_210.9875	: 0.05
fragment_284.0275	: 0.05
fragment_299.0525	: 0.04
fragment_181.1175	: 0.02
fragment_815.3775	: 0.02
fragment_300.0575	: 0.01
fragment_255.0275	: 0.01
fragment_210.9975	: 0.01
fragment_124.0225	: 0.01
fragment_292.0175	: 0.01
fragment_313.0575	: 0.01
fragment_465.0725	: 0.01
fragment_959.4225	: 0.01
fragment_300.0625	: 0.01
fragment_845.4075	: 0.01
fragment_211.0225	: 0.01
fragment_163.0775	: 0.01
fragment_353.2125	: 0.01
loss_163.9925	: 0.01
fragment_764.3175	: 0.01
loss_64.8825	: 0.01
fragment_138.9675	: 0.01
fragment_161.0975	: 0.01
fragment_846.3975	: 0.01
fragment_816.4075	: 0.01
fragment_149.1025	: 0.01
fragment_846.4275	: 0.01
fragment_327.0525	: 0.01

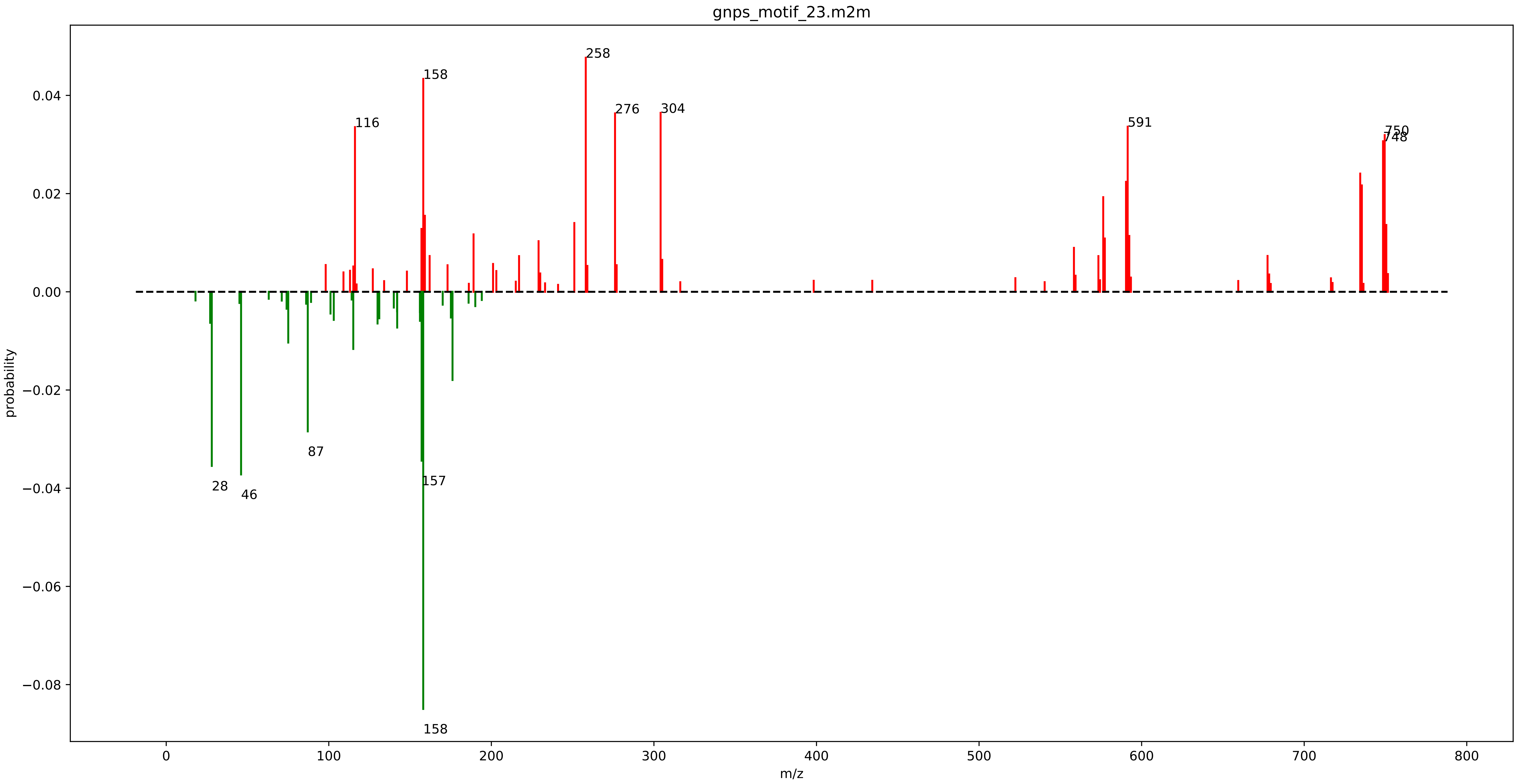
gnps_motif_23.m2m
probability
m/z
0.04
0.02
0.00
−0.02
−0.04
−0.06
−0.08
0
100
200
300
400
500
600
700
800
116
158
258
276
304
591
750
748
28
46
87
157
158

gnps_motif_23.m2m

loss_158.0925	: 0.08
fragment_258.0675	: 0.05
fragment_158.1175	: 0.04
loss_46.0425	: 0.04
fragment_304.1075	: 0.04
fragment_276.0775	: 0.04
loss_28.0275	: 0.04
loss_157.0925	: 0.03
fragment_591.4175	: 0.03
fragment_116.1075	: 0.03
fragment_749.5175	: 0.03
fragment_748.4875	: 0.03
loss_87.0675	: 0.03
fragment_734.4675	: 0.02
fragment_590.3875	: 0.02
fragment_735.4725	: 0.02
fragment_576.3775	: 0.02
loss_176.1075	: 0.02
fragment_159.1175	: 0.02
fragment_251.0375	: 0.01
fragment_750.5225	: 0.01
fragment_156.9975	: 0.01
fragment_749.4875	: 0.01
fragment_189.0475	: 0.01
loss_115.0625	: 0.01
fragment_592.4225	: 0.01
fragment_577.3775	: 0.01
loss_75.0675	: 0.01
fragment_229.0425	: 0.01
fragment_558.3625	: 0.01
fragment_591.3925	: 0.01
fragment_677.4575	: 0.01
fragment_162.0375	: 0.01
loss_142.0725	: 0.01
fragment_217.0425	: 0.01
fragment_573.4125	: 0.01
fragment_305.1125	: 0.01
loss_129.9925	: 0.01
loss_27.0275	: 0.01
loss_156.0875	: 0.01
loss_103.0625	: 0.01
fragment_201.0475	: 0.01
fragment_98.0975	: 0.01
fragment_277.0825	: 0.01
fragment_173.0525	: 0.01
loss_131.0575	: 0.01
fragment_259.0725	: 0.01
loss_175.0975	: 0.01
fragment_115.0775	: 0.01

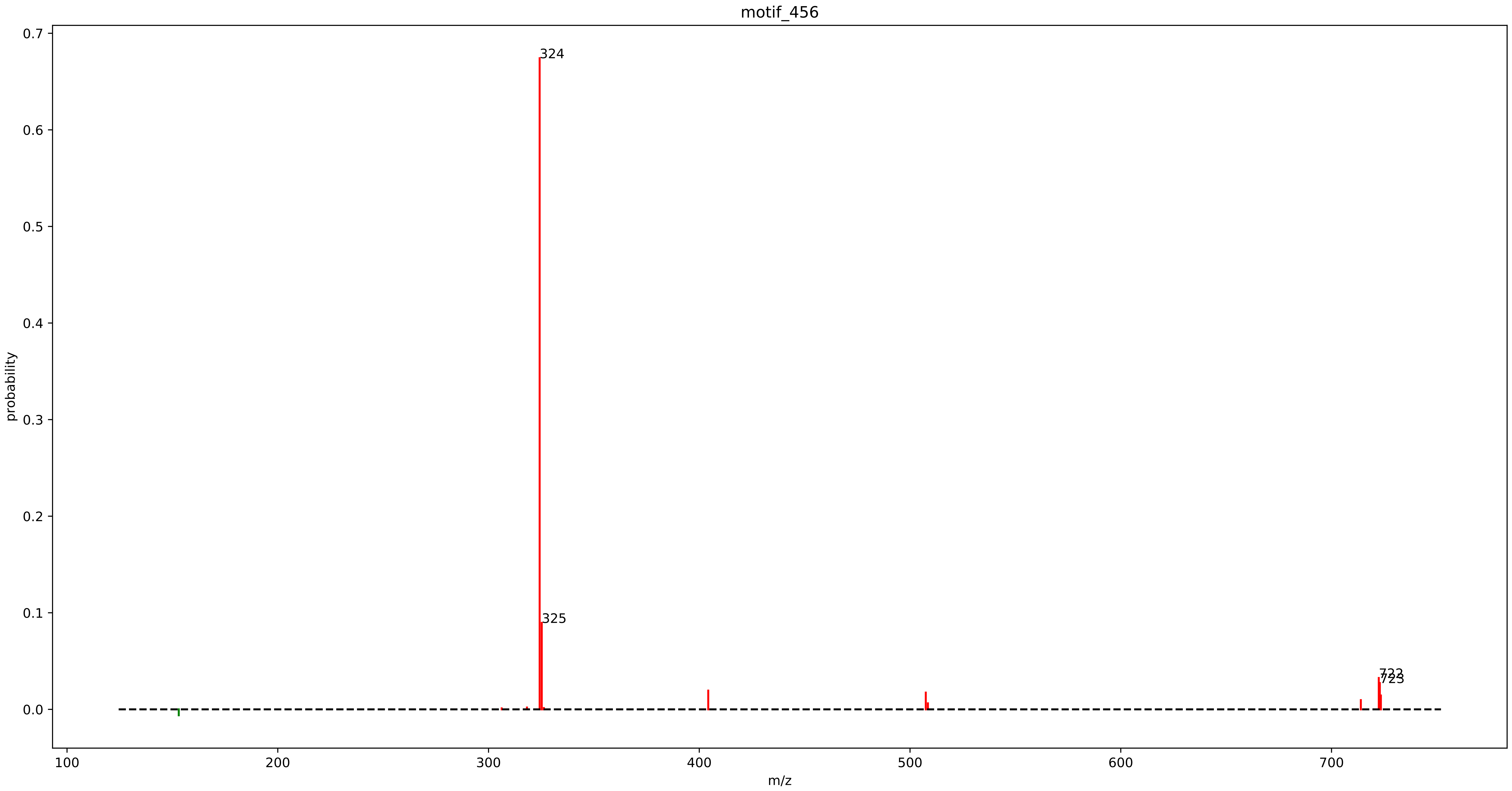
motif_456
probability
m/z
324
325
722
723
0.0
0.1
0.2
0.3
0.4
0.5
0.6
0.7
100
200
300
400
500
600
700

motif_456

fragment_324.2525	: 0.67
fragment_325.2575	: 0.09
fragment_722.4175	: 0.03
fragment_722.9175	: 0.03
fragment_404.2575	: 0.02
fragment_507.4775	: 0.02
fragment_723.4225	: 0.01
fragment_713.9125	: 0.01
fragment_508.4825	: 0.01
loss_153.0175	: 0.01

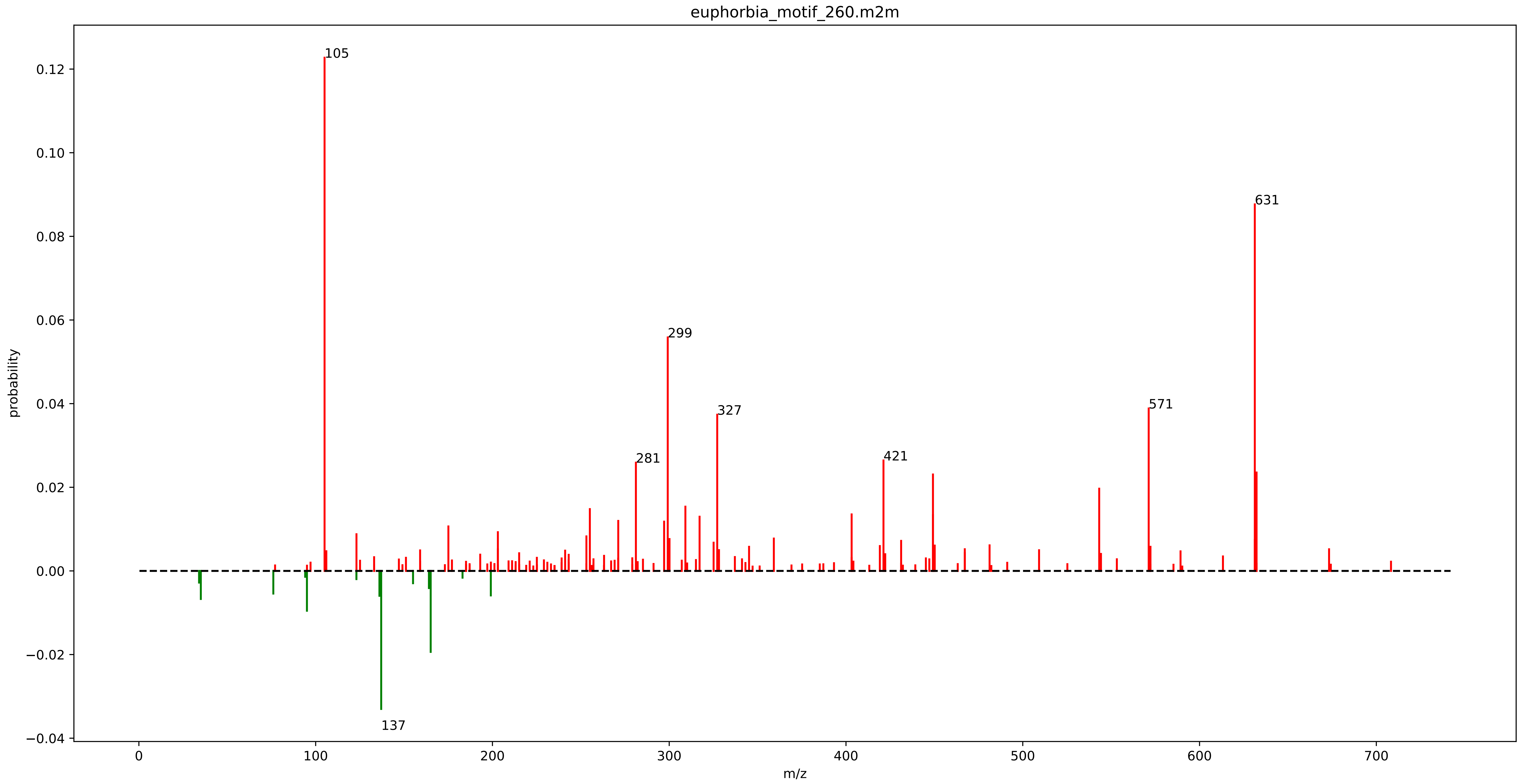

euphorbia_motif_260.m2m
probability
m/z
105
631
299
327
571
281
421
137
0.12
0.10
0.08
0.06
0.04
0.02
0.00
−0.02
−0.04
0
100
200
300
400
500
600
700

euphorbia_motif_260.m2m

fragment_105.0325	: 0.12
fragment_631.2575	: 0.09
fragment_299.1625	: 0.06
fragment_571.2325	: 0.04
fragment_327.1575	: 0.04
loss_137.0725	: 0.03
fragment_421.2025	: 0.03
fragment_281.1525	: 0.03
fragment_632.2575	: 0.02
fragment_449.1975	: 0.02
fragment_543.2375	: 0.02
loss_165.0675	: 0.02
fragment_309.1475	: 0.02
fragment_255.1025	: 0.01
fragment_403.1925	: 0.01
fragment_449.1925	: 0.01
fragment_317.1725	: 0.01
fragment_271.1675	: 0.01
fragment_297.1125	: 0.01
fragment_175.0775	: 0.01
loss_95.0625	: 0.01
fragment_203.0725	: 0.01
fragment_123.0825	: 0.01
fragment_253.1575	: 0.01
fragment_359.1825	: 0.01
fragment_300.1675	: 0.01
fragment_631.2525	: 0.01
fragment_431.1875	: 0.01
fragment_325.1075	: 0.01
loss_35.0375	: 0.01
fragment_481.2225	: 0.01
fragment_450.1975	: 0.01
loss_136.0675	: 0.01
fragment_419.1475	: 0.01
loss_199.0875	: 0.01
fragment_572.2375	: 0.01
fragment_345.1675	: 0.01
loss_76.0475	: 0.01
fragment_467.2025	: 0.01
fragment_673.2675	: 0.01
fragment_203.0675	: 0.01

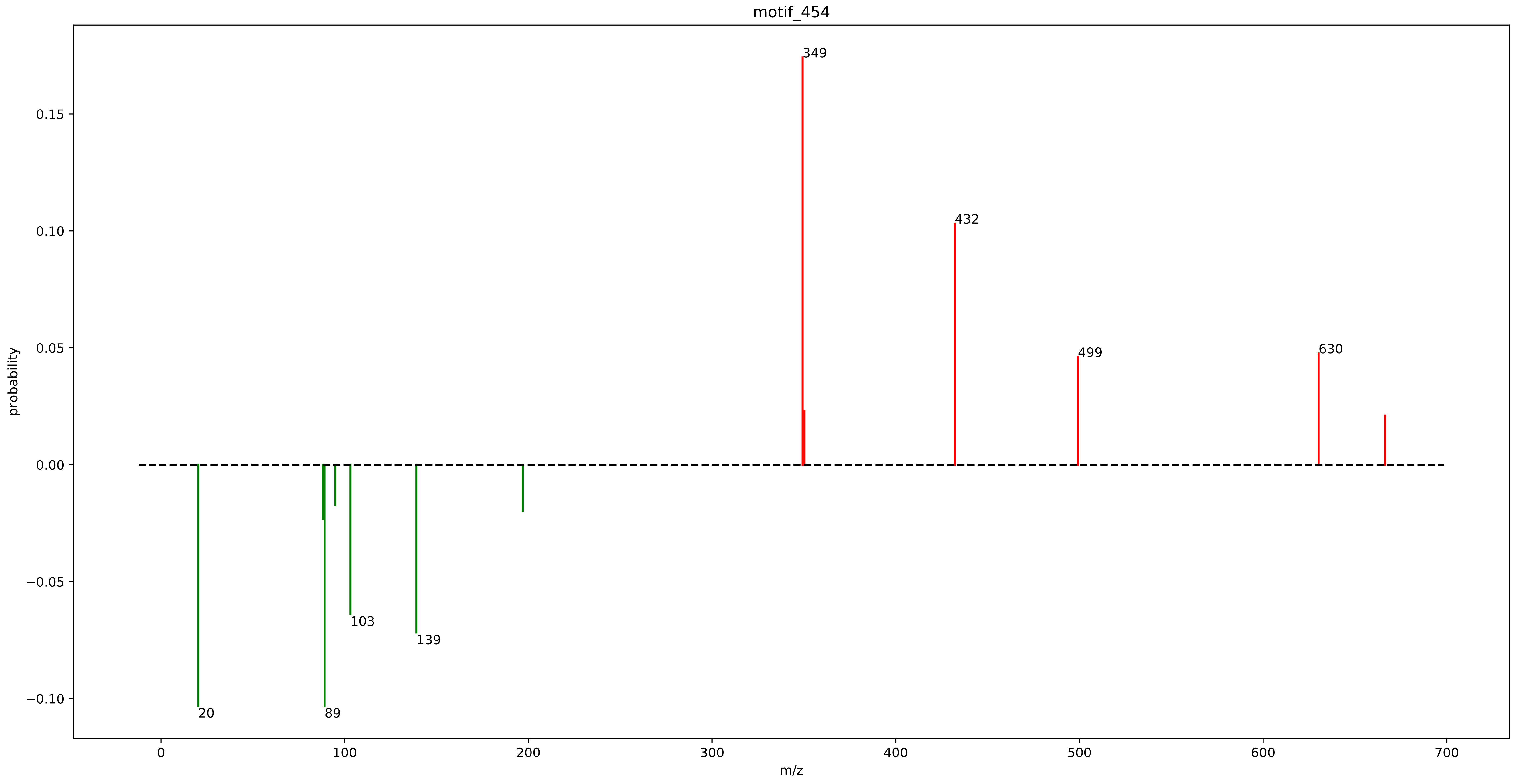

motif_454
349
432
499
630
20
89
103
139
0.15
0.10
0.05
0.00
−0.05
−0.10
probability
0
100
200
300
400
500
600
700
m/z

motif_454

fragment_349.2475	: 0.17
loss_89.0475	: 0.10
fragment_432.0975	: 0.10
loss_20.2125	: 0.10
loss_139.0375	: 0.07
loss_103.0625	: 0.06
fragment_630.2225	: 0.05
fragment_499.1825	: 0.05
fragment_350.2525	: 0.02
loss_88.0425	: 0.02
fragment_666.3175	: 0.02
loss_196.8225	: 0.02
loss_94.7875	: 0.02

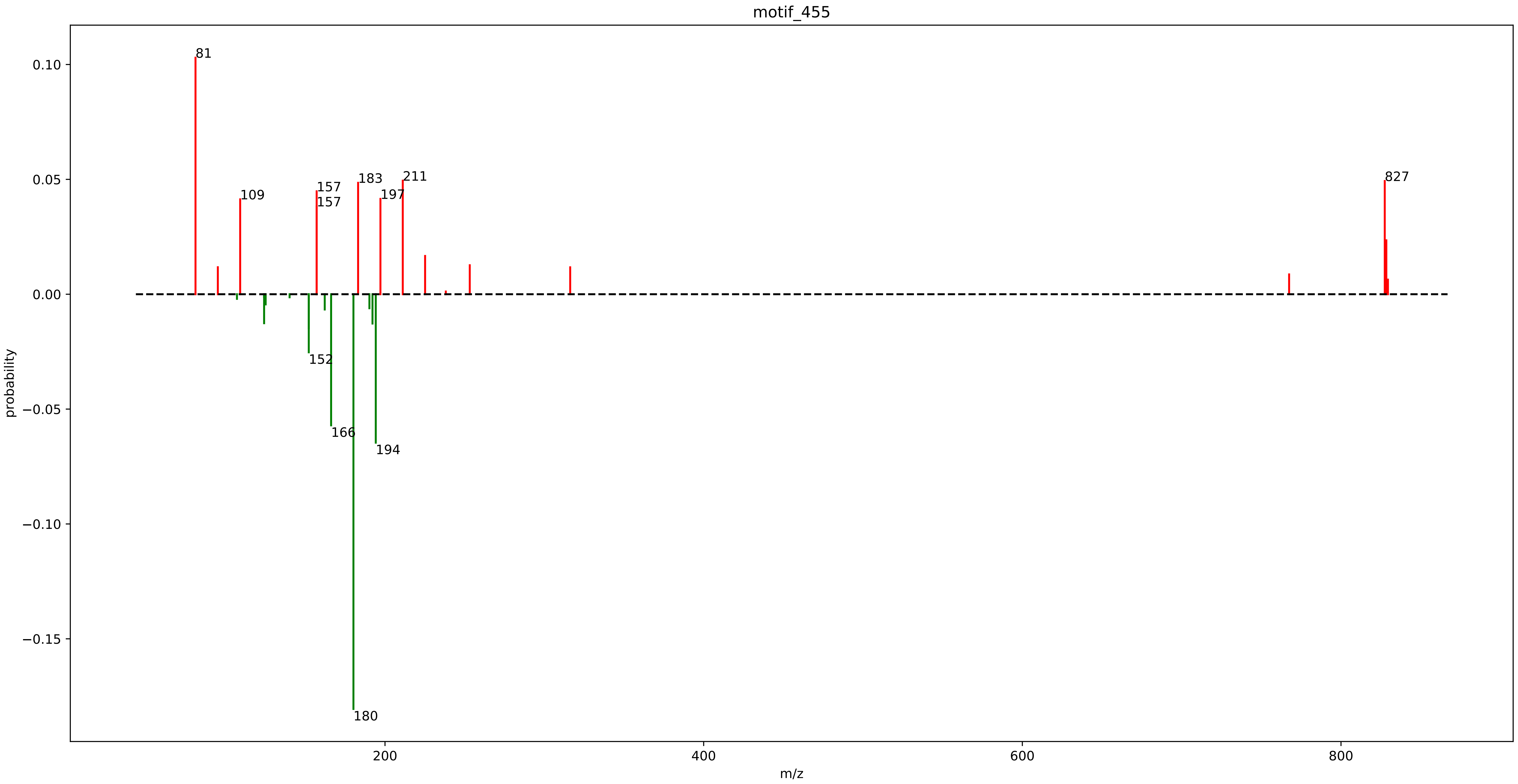

motif_455
81
109
157
157
183
197
211
827
152
166
194
180
0.10
0.05
0.00
−0.05
−0.10
−0.15
probability
200
400
600
800
m/z

motif_455

loss_180.1875	: 0.18
fragment_81.0675	: 0.10
loss_194.2025	: 0.06
loss_166.1725	: 0.06
fragment_211.1475	: 0.05
fragment_827.4575	: 0.05
fragment_183.1175	: 0.05
fragment_157.1025	: 0.04
fragment_197.1325	: 0.04
fragment_109.0975	: 0.04
fragment_157.0975	: 0.04
loss_152.1525	: 0.03
fragment_828.4575	: 0.02
fragment_225.1625	: 0.02
loss_152.1575	: 0.01
loss_192.1475	: 0.01
fragment_253.1925	: 0.01
loss_124.1225	: 0.01
loss_194.1875	: 0.01
fragment_95.0825	: 0.01
fragment_316.2275	: 0.01
fragment_767.4325	: 0.01
loss_162.1725	: 0.01
fragment_829.4625	: 0.01
loss_190.2075	: 0.01

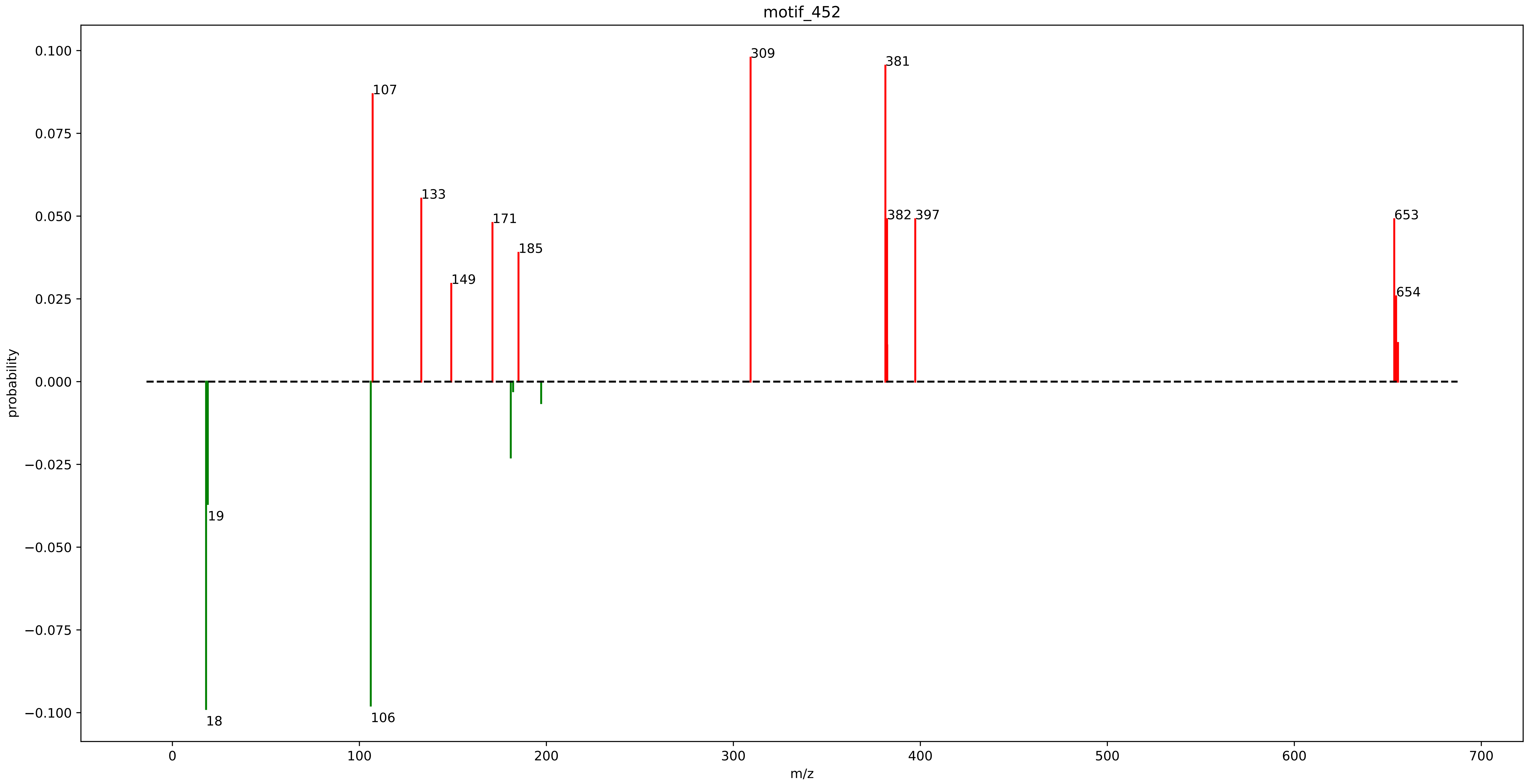
motif_452
probability
m/z
107
133
149
171
185
309
381
382
397
653
654
19
18
106
0.100
0.075
0.050
0.025
0.000
−0.025
−0.050
−0.075
−0.100
0
100
200
300
400
500
600
700

motif_452

loss_18.0075	: 0.10
loss_106.0775	: 0.10
fragment_309.2025	: 0.10
fragment_381.2975	: 0.10
fragment_107.0825	: 0.09
fragment_133.1025	: 0.06
fragment_397.2725	: 0.05
fragment_653.4375	: 0.05
fragment_382.1975	: 0.05
fragment_171.1625	: 0.05
fragment_185.0825	: 0.04
loss_18.9275	: 0.04
fragment_149.1325	: 0.03
fragment_654.4425	: 0.03
loss_180.9525	: 0.02
fragment_655.4425	: 0.01
fragment_382.3025	: 0.01
loss_197.2075	: 0.01

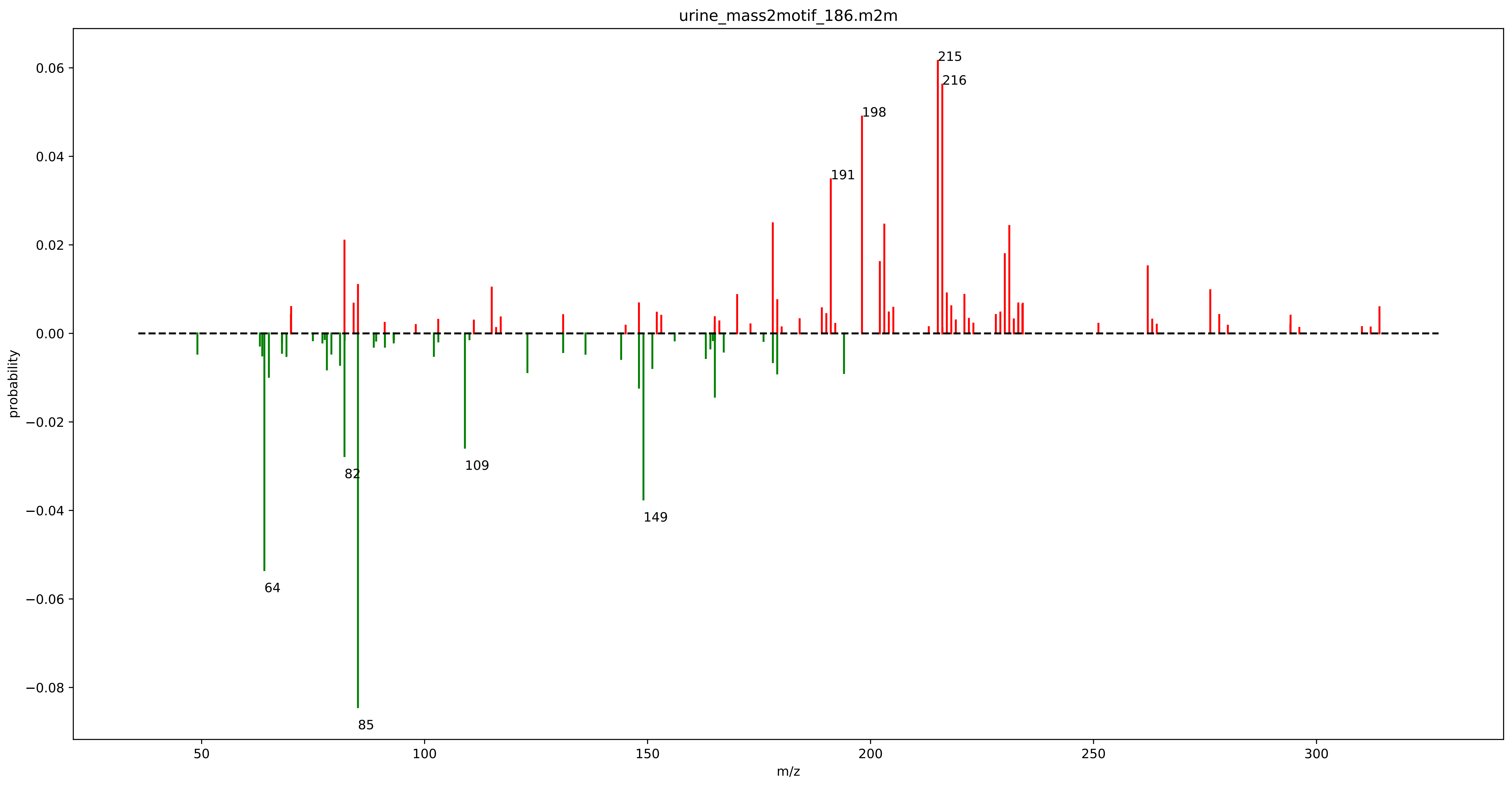
urine_mass2motif_186.m2m
probability
m/z
0.06
0.04
0.02
0.00
−0.02
−0.04
−0.06
−0.08
50
100
150
200
250
300
215
216
198
191
82
109
149
64
85

urine_mass2motif_186.m2m

loss_85.0525	: 0.08
fragment_215.0875	: 0.06
fragment_216.0875	: 0.06
loss_64.0675	: 0.05
fragment_198.0775	: 0.05
loss_149.0725	: 0.04
fragment_191.0825	: 0.03
loss_82.0325	: 0.03
loss_109.0375	: 0.03
fragment_178.0775	: 0.02
fragment_203.0825	: 0.02
fragment_231.1125	: 0.02
fragment_82.0275	: 0.02
fragment_230.1025	: 0.02
fragment_202.0825	: 0.02
fragment_262.1575	: 0.02
loss_165.0875	: 0.01
loss_148.0675	: 0.01
fragment_85.0575	: 0.01
fragment_115.0525	: 0.01
loss_65.0825	: 0.01
fragment_276.1775	: 0.01
loss_179.0675	: 0.01
fragment_217.1025	: 0.01
loss_194.0425	: 0.01
loss_123.0525	: 0.01
fragment_221.0425	: 0.01
fragment_170.0825	: 0.01
loss_78.0875	: 0.01
loss_151.0675	: 0.01
fragment_179.0825	: 0.01
loss_81.0425	: 0.01
fragment_148.0625	: 0.01
fragment_233.1275	: 0.01
fragment_84.0825	: 0.01
fragment_234.1325	: 0.01
loss_178.0975	: 0.01
fragment_234.0475	: 0.01
fragment_218.1025	: 0.01
fragment_233.1225	: 0.01
fragment_70.0675	: 0.01
fragment_314.1175	: 0.01
fragment_205.1025	: 0.01
loss_144.0725	: 0.01
fragment_189.0725	: 0.01
loss_163.0575	: 0.01
loss_69.0225	: 0.01
loss_102.0775	: 0.01

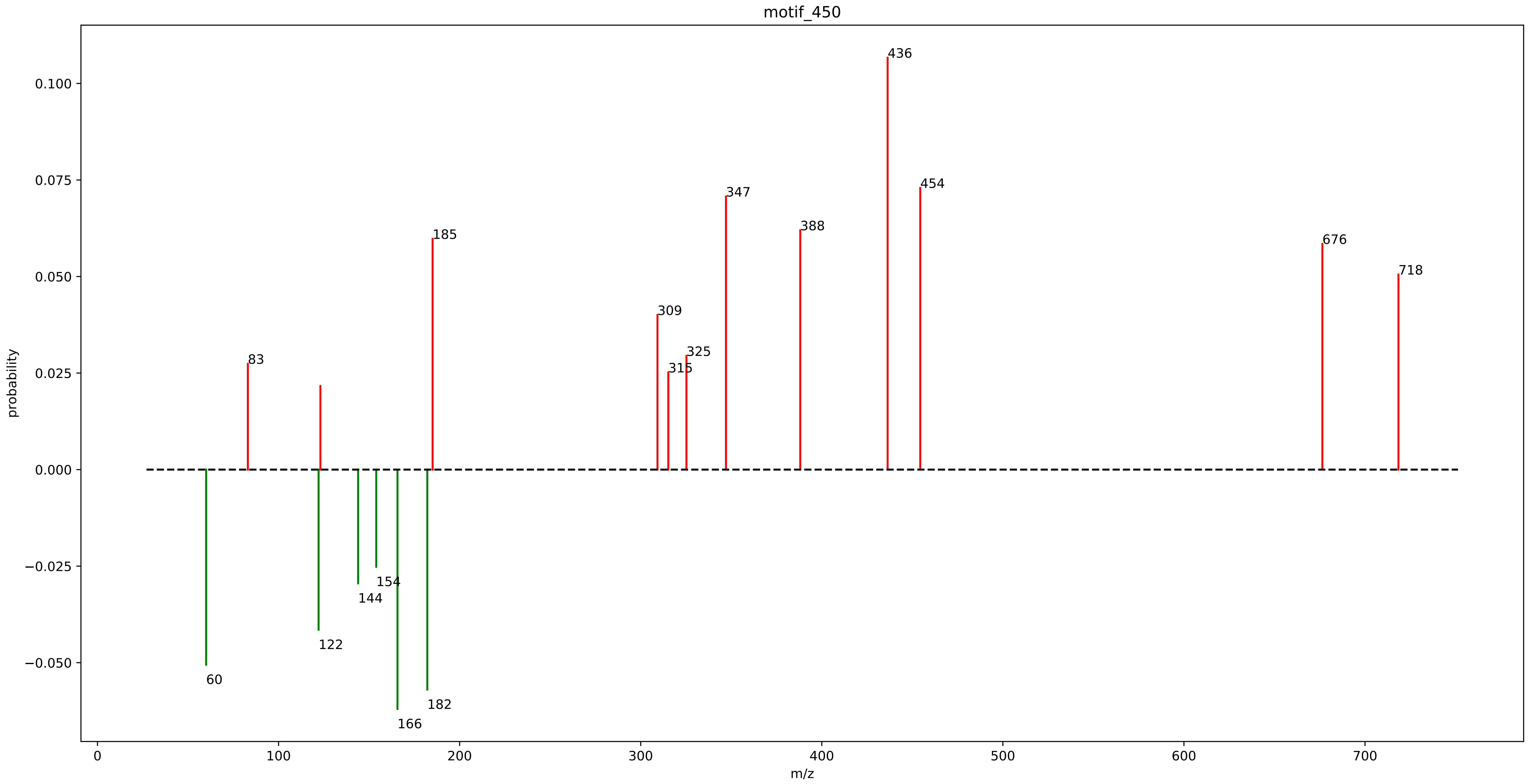

motif_450
probability
m/z
0.100
0.075
0.050
0.025
0.000
−0.025
−0.050
0
100
200
300
400
500
600
700
436
347
454
388
185
676
718
309
325
315
83
154
144
122
60
182
166

motif_450

fragment_436.3575	: 0.11
fragment_454.3675	: 0.07
fragment_347.0825	: 0.07
loss_165.6625	: 0.06
fragment_388.1075	: 0.06
fragment_185.0675	: 0.06
fragment_676.4425	: 0.06
loss_182.1275	: 0.06
fragment_718.4575	: 0.05
loss_60.0125	: 0.05
loss_122.1075	: 0.04
fragment_309.2575	: 0.04
loss_143.9375	: 0.03
fragment_325.2525	: 0.03
fragment_83.0475	: 0.03
loss_153.9575	: 0.03
fragment_315.2325	: 0.03
fragment_123.1175	: 0.02

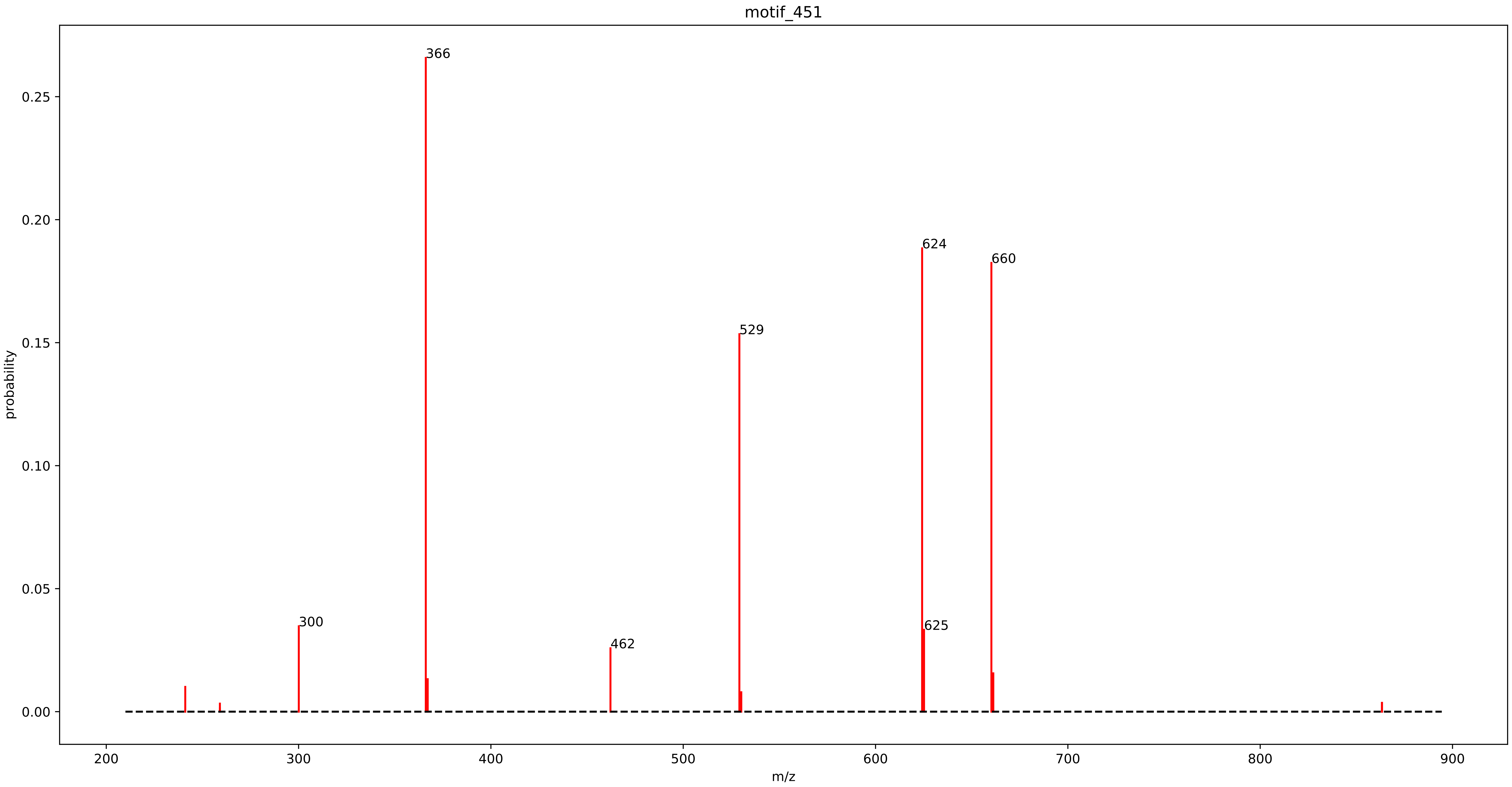
motif_451
366
624
660
529
300
462
625
probability
0.25
0.20
0.15
0.10
0.05
0.00
200
300
400
500
600
700
800
900
m/z

motif_451

fragment_366.1375	: 0.27
fragment_624.2125	: 0.19
fragment_660.2325	: 0.18
fragment_529.1975	: 0.15
fragment_300.1075	: 0.03
fragment_625.2175	: 0.03
fragment_462.1625	: 0.03
fragment_661.2375	: 0.02
fragment_367.1425	: 0.01
fragment_241.0725	: 0.01
fragment_530.1975	: 0.01

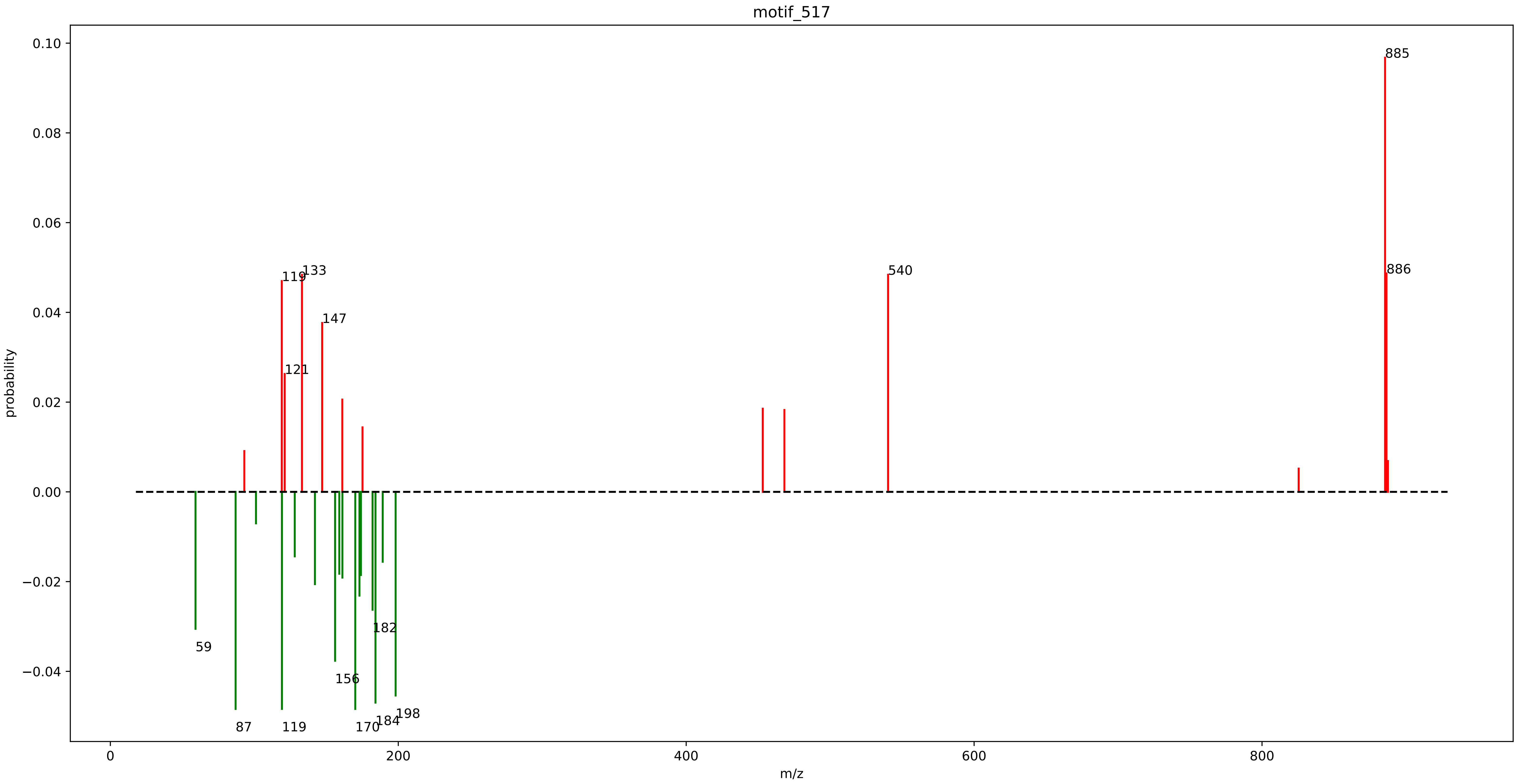

motif_517
probability
m/z
0.10
0.08
0.06
0.04
0.02
0.00
−0.02
−0.04
0
200
400
600
800
885
886
540
119
133
147
121
59
87
119
156
170
182
184
198

motif_517

fragment_885.4625	: 0.10
fragment_886.4675	: 0.05
fragment_133.1025	: 0.05
loss_170.1325	: 0.05
loss_87.0025	: 0.05
fragment_540.2375	: 0.05
loss_119.1725	: 0.05
fragment_119.0825	: 0.05
loss_184.1475	: 0.05
loss_198.1625	: 0.05
fragment_147.1175	: 0.04
loss_156.1125	: 0.04
loss_59.1525	: 0.03
fragment_121.1025	: 0.03
loss_182.1275	: 0.03
loss_173.0275	: 0.02
loss_142.0975	: 0.02
fragment_161.1325	: 0.02
loss_161.1875	: 0.02
fragment_453.1925	: 0.02
loss_174.0475	: 0.02
fragment_468.2175	: 0.02
loss_159.0275	: 0.02
loss_189.1825	: 0.02
loss_128.0825	: 0.01
fragment_175.1475	: 0.01
fragment_93.0675	: 0.01
loss_101.1625	: 0.01
fragment_887.4675	: 0.01
fragment_825.4425	: 0.01

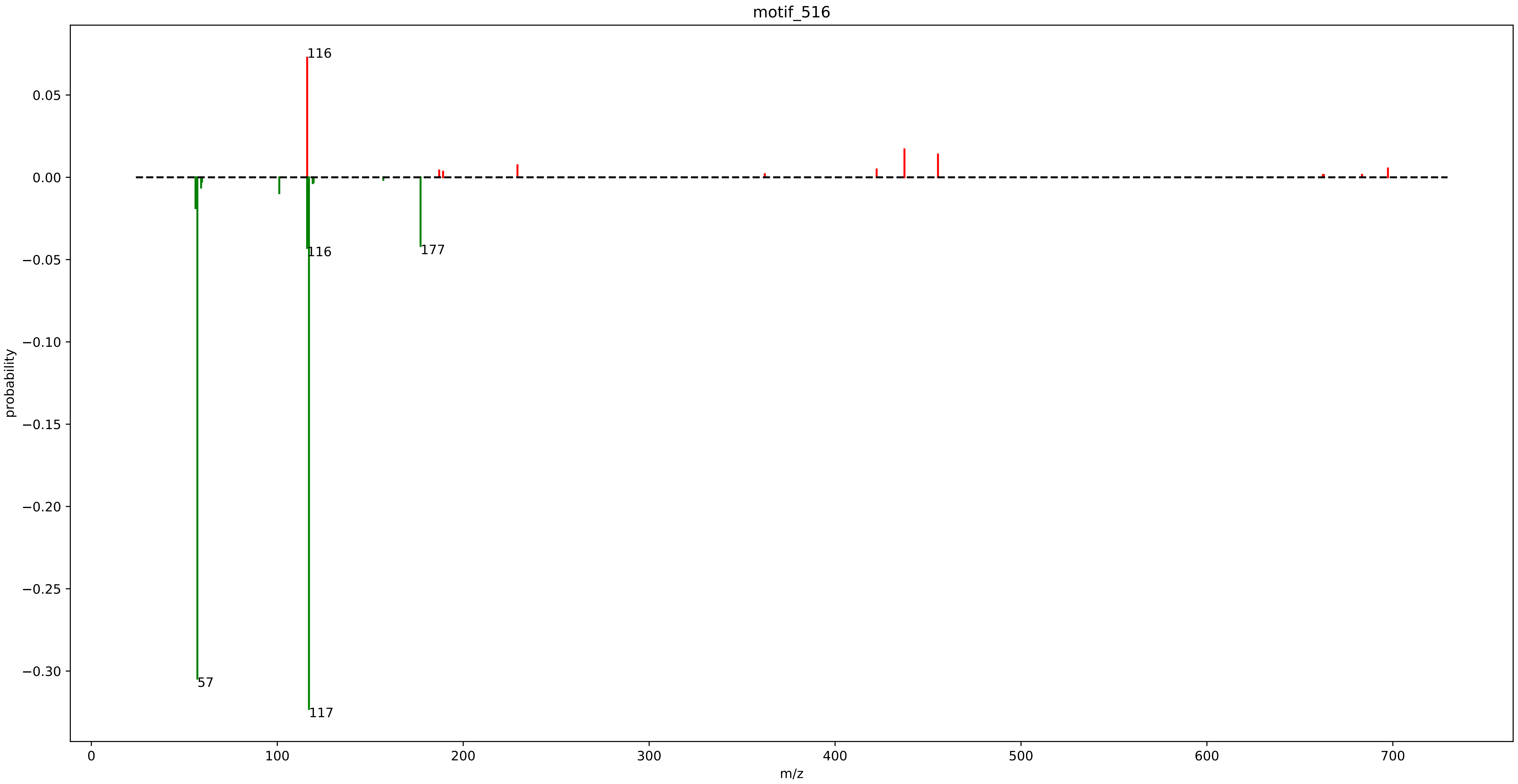

motif_516
116
116
177
57
117
0.05
0.00
−0.05
−0.10
−0.15
−0.20
−0.25
−0.30
probability
0
100
200
300
400
500
600
700
m/z

motif_516

loss_117.0325	: 0.32
loss_57.0125	: 0.30
fragment_116.0675	: 0.07
loss_116.0275	: 0.04
loss_177.0525	: 0.04
loss_56.0075	: 0.02
fragment_437.3225	: 0.02
fragment_455.3525	: 0.01
loss_101.0675	: 0.01
fragment_229.1775	: 0.01
loss_59.0225	: 0.01
fragment_697.3625	: 0.01

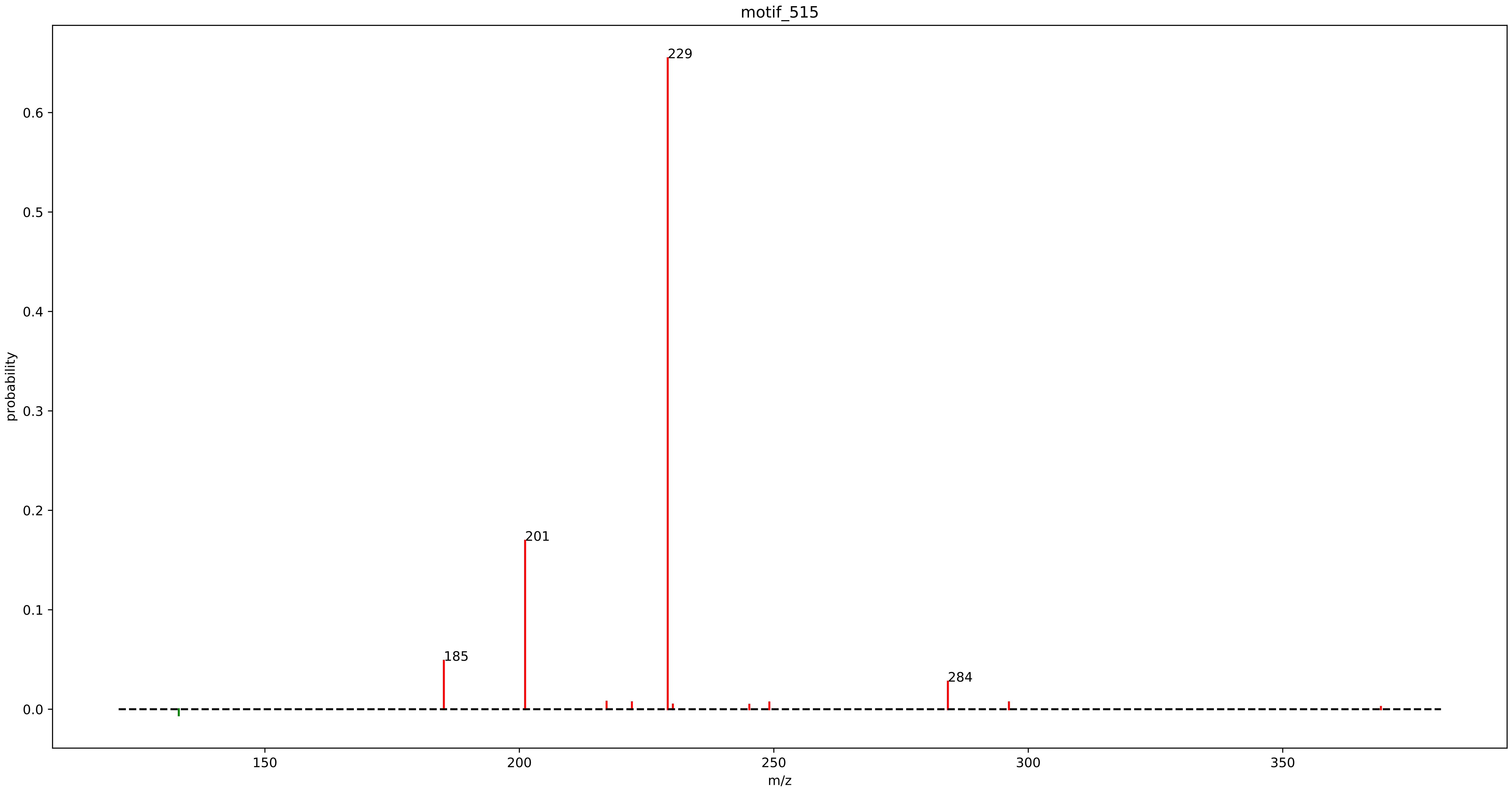

motif_515
229
201
185
284
probability
m/z
0.0
0.1
0.2
0.3
0.4
0.5
0.6
150
200
250
300
350

motif_515

fragment_229.1525	: 0.65
fragment_201.1225	: 0.17
fragment_185.1625	: 0.05
fragment_284.1975	: 0.03
fragment_217.1375	: 0.01
fragment_222.1125	: 0.01
fragment_296.1975	: 0.01
fragment_249.1225	: 0.01
loss_133.0725	: 0.01

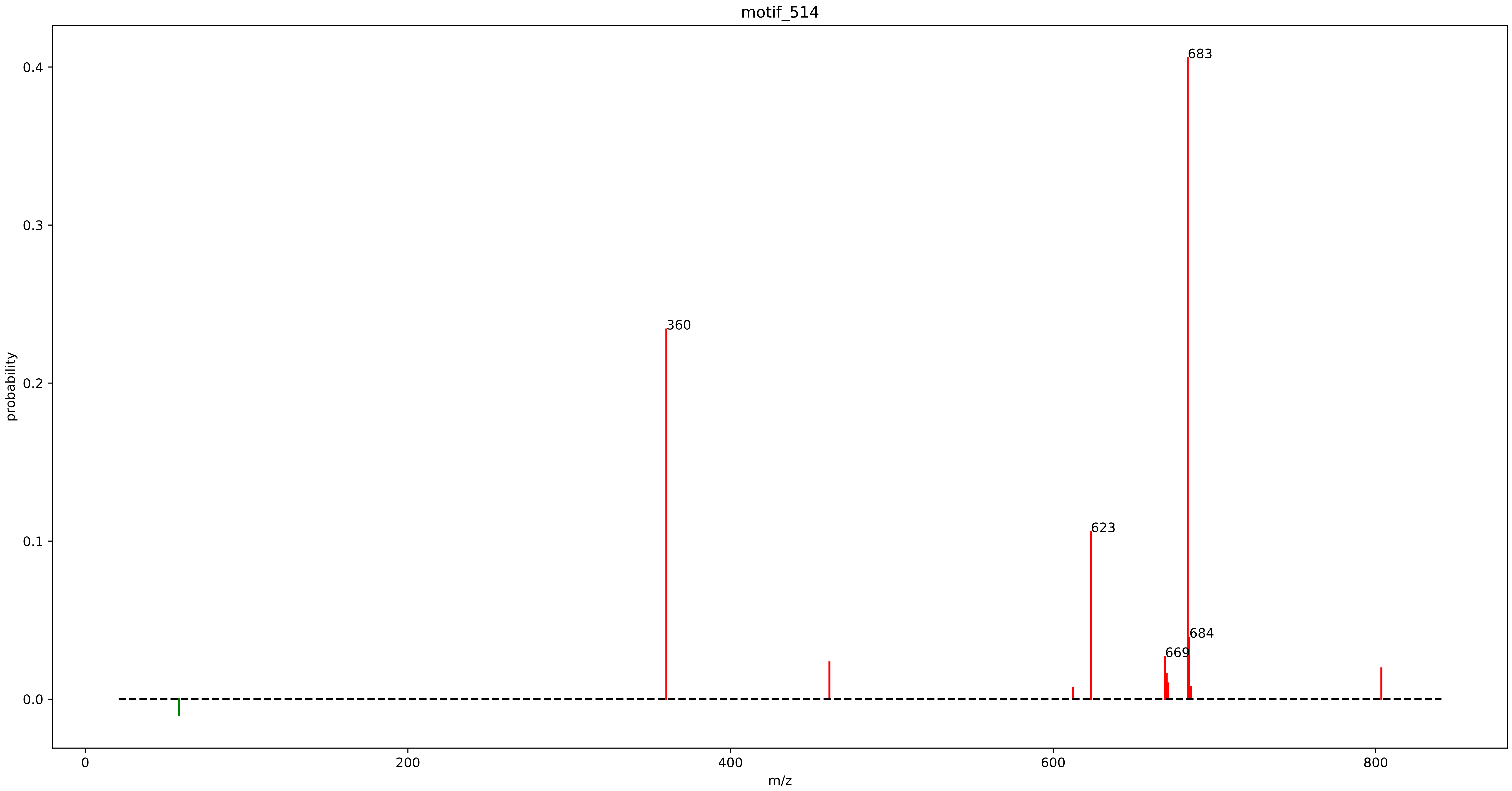

motif_514
probability
m/z
0.4
0.3
0.2
0.1
0.0
0
200
400
600
800
683
360
623
684
669

motif_514

fragment_683.4025	: 0.41
fragment_360.2525	: 0.23
fragment_623.3775	: 0.11
fragment_684.4025	: 0.04
fragment_669.3875	: 0.03
fragment_461.3275	: 0.02
fragment_803.4425	: 0.02
fragment_670.3875	: 0.02
loss_58.0075	: 0.01
fragment_671.3975	: 0.01
fragment_685.4025	: 0.01
fragment_612.3925	: 0.01

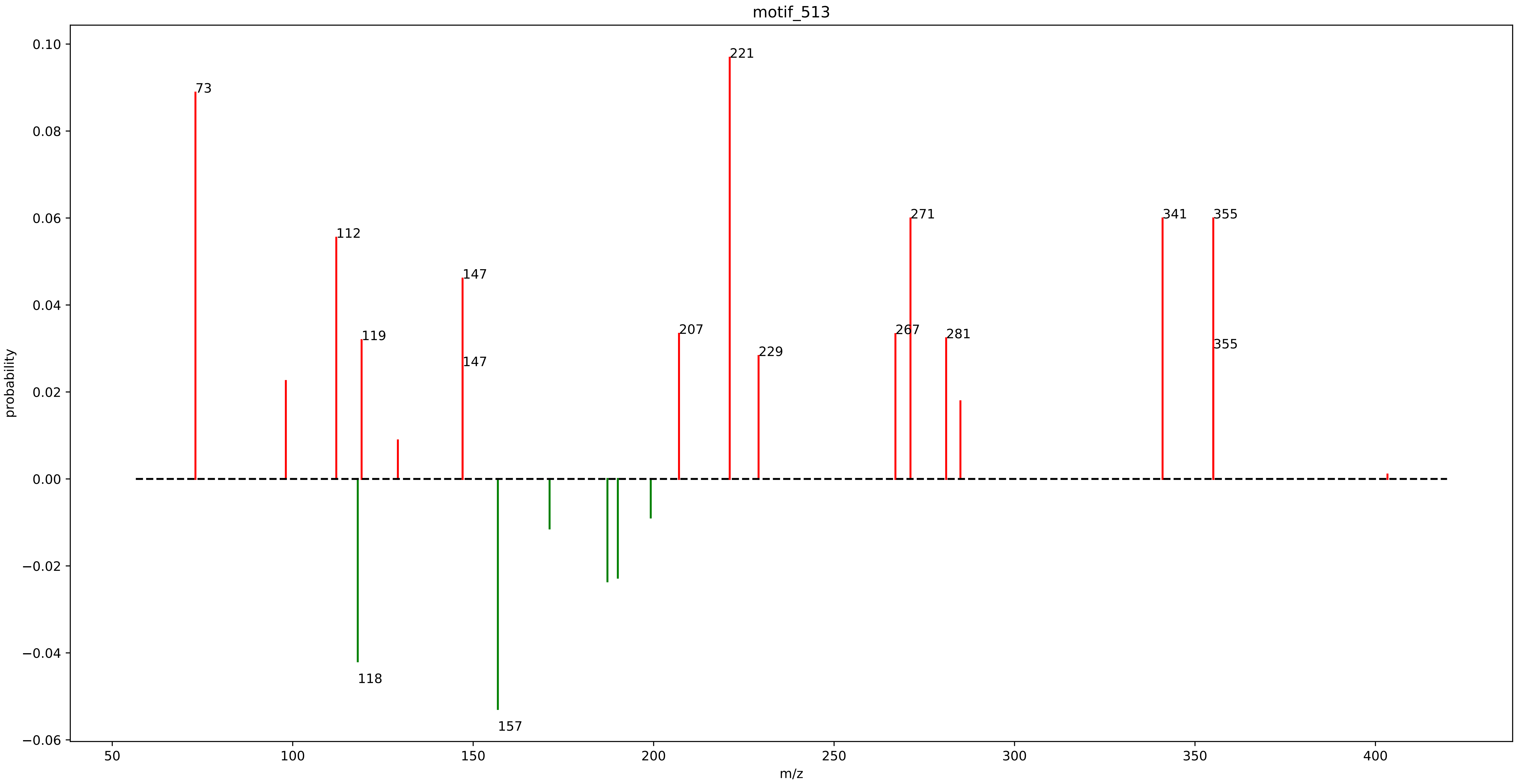

motif_513
probability
m/z
0.10
0.08
0.06
0.04
0.02
0.00
−0.02
−0.04
−0.06
50
100
150
200
250
300
350
400
73
112
119
147
147
207
221
229
267
271
281
341
355
355
118
157

motif_513

fragment_221.0825	: 0.10
fragment_73.0475	: 0.09
fragment_355.0675	: 0.06
fragment_341.0175	: 0.06
fragment_271.1675	: 0.06
fragment_112.0725	: 0.06
loss_156.8425	: 0.05
fragment_147.0625	: 0.05
loss_118.0225	: 0.04
fragment_207.0325	: 0.03
fragment_266.9975	: 0.03
fragment_281.0525	: 0.03
fragment_119.0875	: 0.03
fragment_355.0725	: 0.03
fragment_229.0775	: 0.03
fragment_147.0675	: 0.03
loss_187.2025	: 0.02
loss_190.0825	: 0.02
fragment_98.0975	: 0.02
fragment_285.0075	: 0.02
loss_171.1725	: 0.01
loss_199.2025	: 0.01
fragment_129.1375	: 0.01

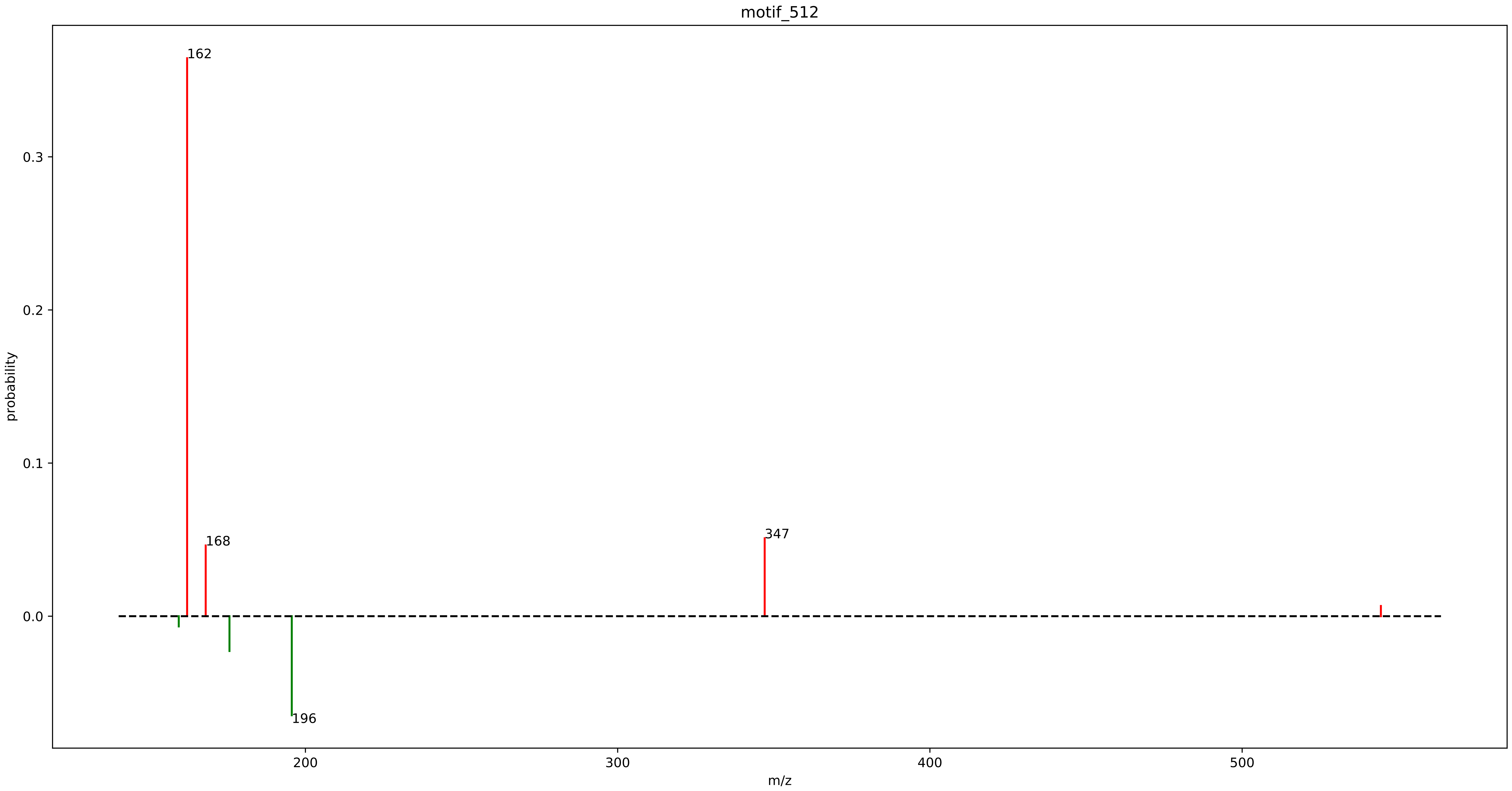
motif_512
162
168
347
196
0.3
0.2
0.1
0.0
probability
200
300
400
500
m/z

motif_512

fragment_162.1125	: 0.36
loss_195.6275	: 0.06
fragment_347.0825	: 0.05
fragment_168.0625	: 0.05
loss_175.6775	: 0.02
fragment_544.4325	: 0.01
loss_159.4375	: 0.01

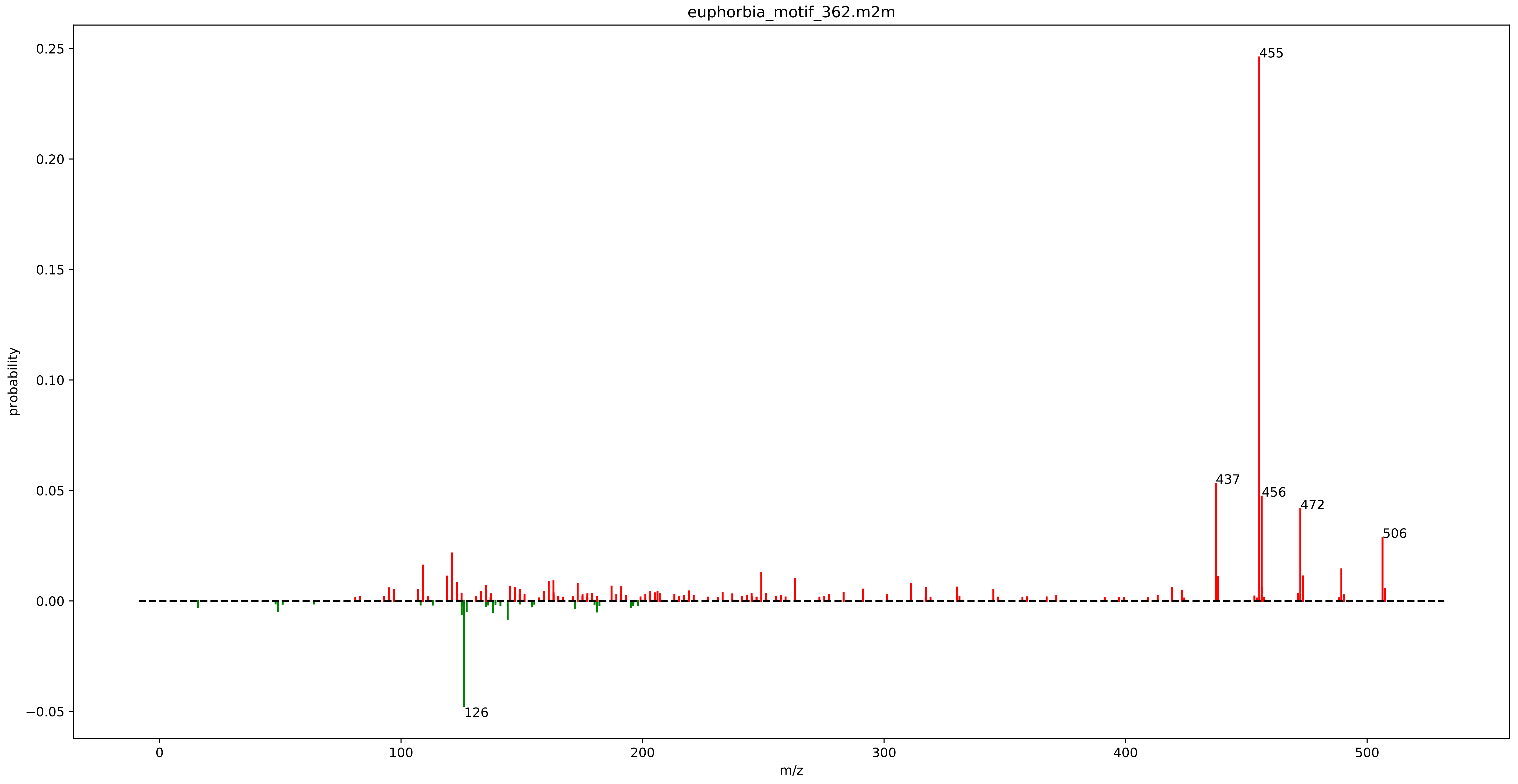

euphorbia_motif_362.m2m
probability
m/z
0.25
0.20
0.15
0.10
0.05
0.00
−0.05
0
100
200
300
400
500
455
437
456
472
506
126

euphorbia_motif_362.m2m

fragment_455.3425	: 0.25
fragment_437.3325	: 0.05
loss_126.1075	: 0.05
fragment_456.3475	: 0.05
fragment_472.3675	: 0.04
fragment_506.3775	: 0.03
fragment_121.0975	: 0.02
fragment_109.0975	: 0.02
fragment_489.3475	: 0.01
fragment_249.1425	: 0.01
fragment_473.3725	: 0.01
fragment_119.0825	: 0.01
fragment_438.3375	: 0.01
fragment_263.1575	: 0.01
fragment_163.1075	: 0.01
fragment_161.1275	: 0.01
loss_144.1175	: 0.01
fragment_123.1125	: 0.01
fragment_173.1275	: 0.01
fragment_311.2325	: 0.01
fragment_135.1125	: 0.01
fragment_187.1475	: 0.01
fragment_145.0975	: 0.01
fragment_191.1725	: 0.01
fragment_330.2425	: 0.01
fragment_456.3525	: 0.01
loss_125.1075	: 0.01
fragment_317.2425	: 0.01
fragment_147.1125	: 0.01
fragment_419.3225	: 0.01
fragment_95.0825	: 0.01
fragment_507.3775	: 0.01
fragment_291.1875	: 0.01
loss_138.1075	: 0.01
fragment_149.1275	: 0.01

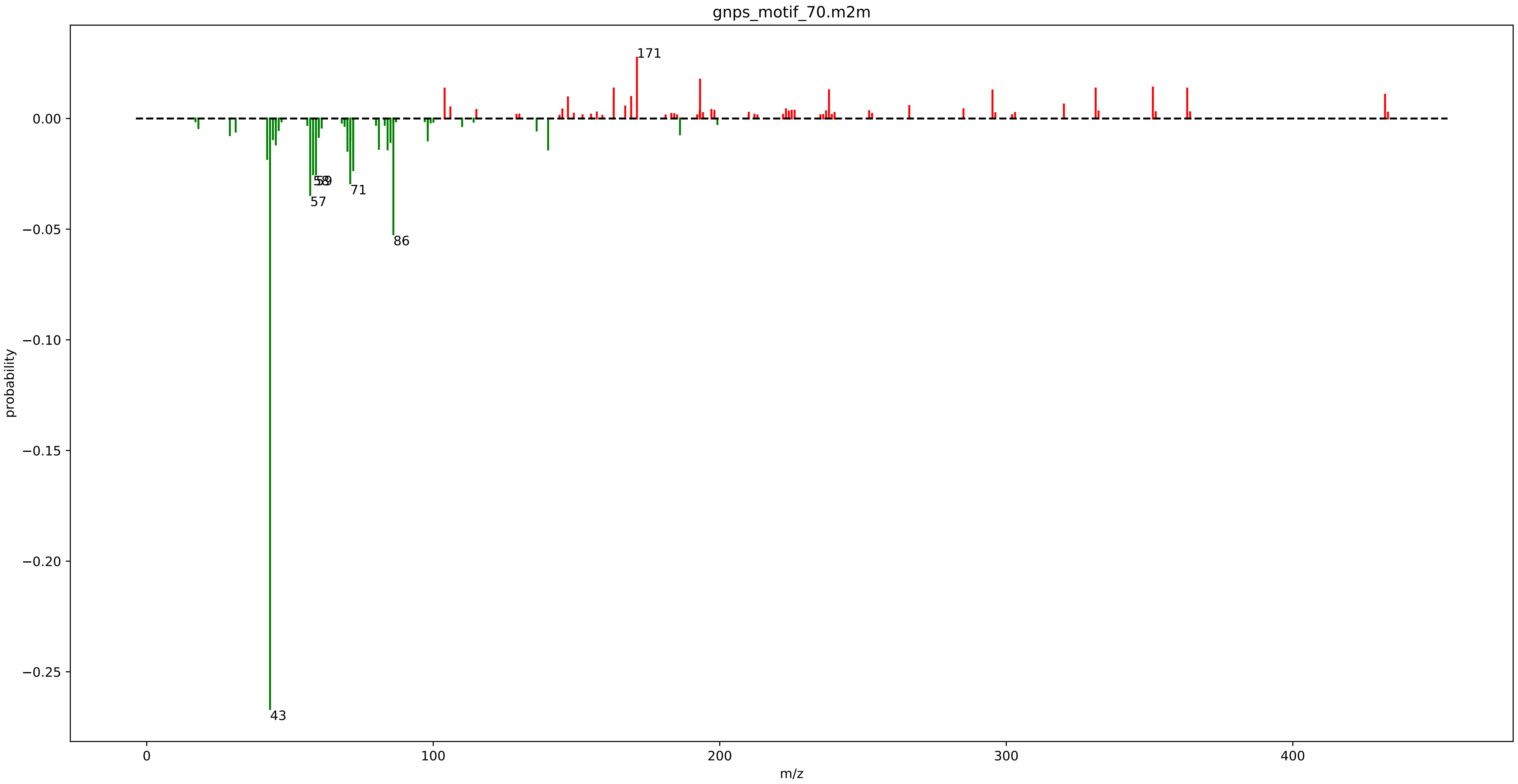

gnps_motif_70.m2m
171
0.00
−0.05
−0.10
−0.15
−0.20
−0.25
58
59
57
71
86
43
probability
0
100
200
300
400
m/z

gnps_motif_70.m2m

loss_43.0425	: 0.27	fragment_238.1225	: 0.01
loss_86.0825	: 0.05	fragment_295.1825	: 0.01
loss_57.0575	: 0.03	loss_45.0575	: 0.01
loss_71.0375	: 0.03	fragment_432.1975	: 0.01
fragment_171.0925	: 0.03	loss_85.0775	: 0.01
loss_59.0725	: 0.03	loss_98.0825	: 0.01
loss_58.0675	: 0.03	fragment_169.0875	: 0.01
loss_72.0825	: 0.02	fragment_147.0125	: 0.01
loss_42.0375	: 0.02	loss_71.0725	: 0.01
fragment_193.1325	: 0.02	loss_44.0525	: 0.01
loss_72.0675	: 0.01	loss_60.0675	: 0.01
loss_70.0675	: 0.01	loss_29.0275	: 0.01
fragment_351.1625	: 0.01	loss_186.1175	: 0.01
loss_140.0975	: 0.01	fragment_320.1025	: 0.01
loss_84.0675	: 0.01	loss_31.0425	: 0.01
loss_81.0325	: 0.01	fragment_266.1525	: 0.01
fragment_331.1975	: 0.01	fragment_166.9975	: 0.01
fragment_103.9675	: 0.01	loss_136.0875	: 0.01
fragment_162.9925	: 0.01	loss_46.0675	: 0.01
fragment_363.1475	: 0.01	fragment_105.9825	: 0.01

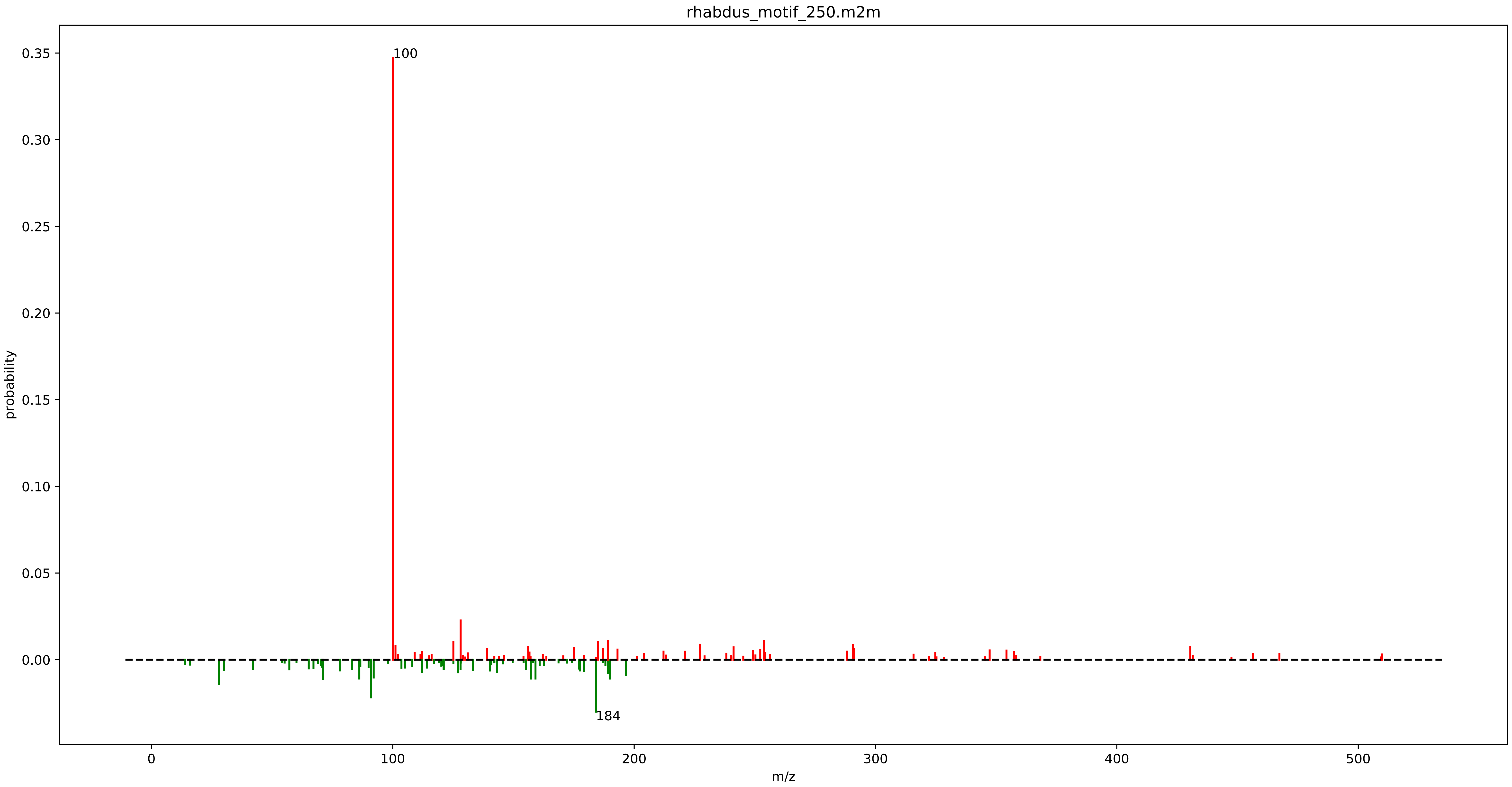

rhabdus_motif_250.m2m
probability
m/z
100
184
0.35
0.30
0.25
0.20
0.15
0.10
0.05
0.00
0
100
200
300
400
500

rhabdus_motif_250.m2m

fragment_100.1125	: 0.35
loss_184.1475	: 0.03
fragment_128.1075	: 0.02
loss_90.9825	: 0.02
loss_28.0325	: 0.01
loss_71.0675	: 0.01
fragment_253.6925	: 0.01
fragment_189.1375	: 0.01
loss_189.8525	: 0.01
loss_157.1825	: 0.01
loss_159.1225	: 0.01
loss_86.1375	: 0.01
fragment_185.0925	: 0.01
fragment_125.1075	: 0.01
loss_92.0625	: 0.01
loss_196.6575	: 0.01
fragment_227.1725	: 0.01
fragment_290.7275	: 0.01
fragment_101.1125	: 0.01
loss_189.1725	: 0.01
fragment_430.4375	: 0.01
fragment_156.1025	: 0.01
loss_127.0975	: 0.01
fragment_241.1925	: 0.01
loss_143.1675	: 0.01
loss_112.1025	: 0.01
fragment_175.1225	: 0.01
loss_179.1875	: 0.01
fragment_187.1225	: 0.01
loss_140.1775	: 0.01
loss_177.6125	: 0.01
fragment_291.2275	: 0.01
fragment_139.1225	: 0.01
loss_78.0475	: 0.01
loss_30.0475	: 0.01
loss_133.1725	: 0.01
fragment_193.1025	: 0.01
fragment_252.2575	: 0.01
loss_57.1375	: 0.01
loss_121.0675	: 0.01
loss_83.1725	: 0.01
fragment_347.2675	: 0.01
loss_42.0475	: 0.01
loss_155.1675	: 0.01
fragment_354.2725	: 0.01
loss_128.0775	: 0.01
loss_121.0875	: 0.01
loss_177.1125	: 0.01
fragment_249.1975	: 0.01
loss_65.1525	: 0.01

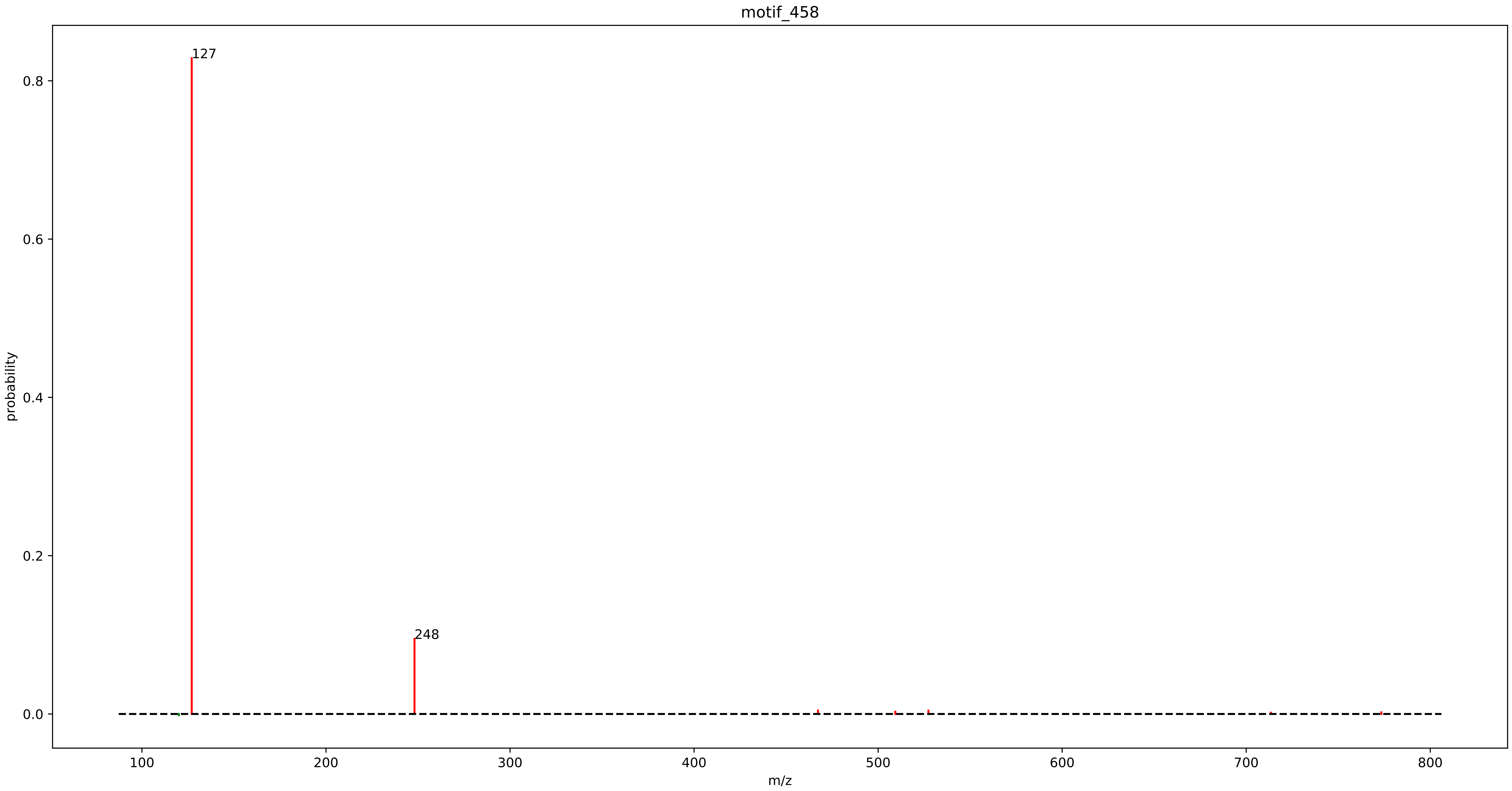

motif_458
127
248
probability
0.8
0.6
0.4
0.2
0.0
100
200
300
400
500
600
700
800
m/z

motif_458

fragment_127.0375	: 0.83
fragment_248.0825	: 0.09

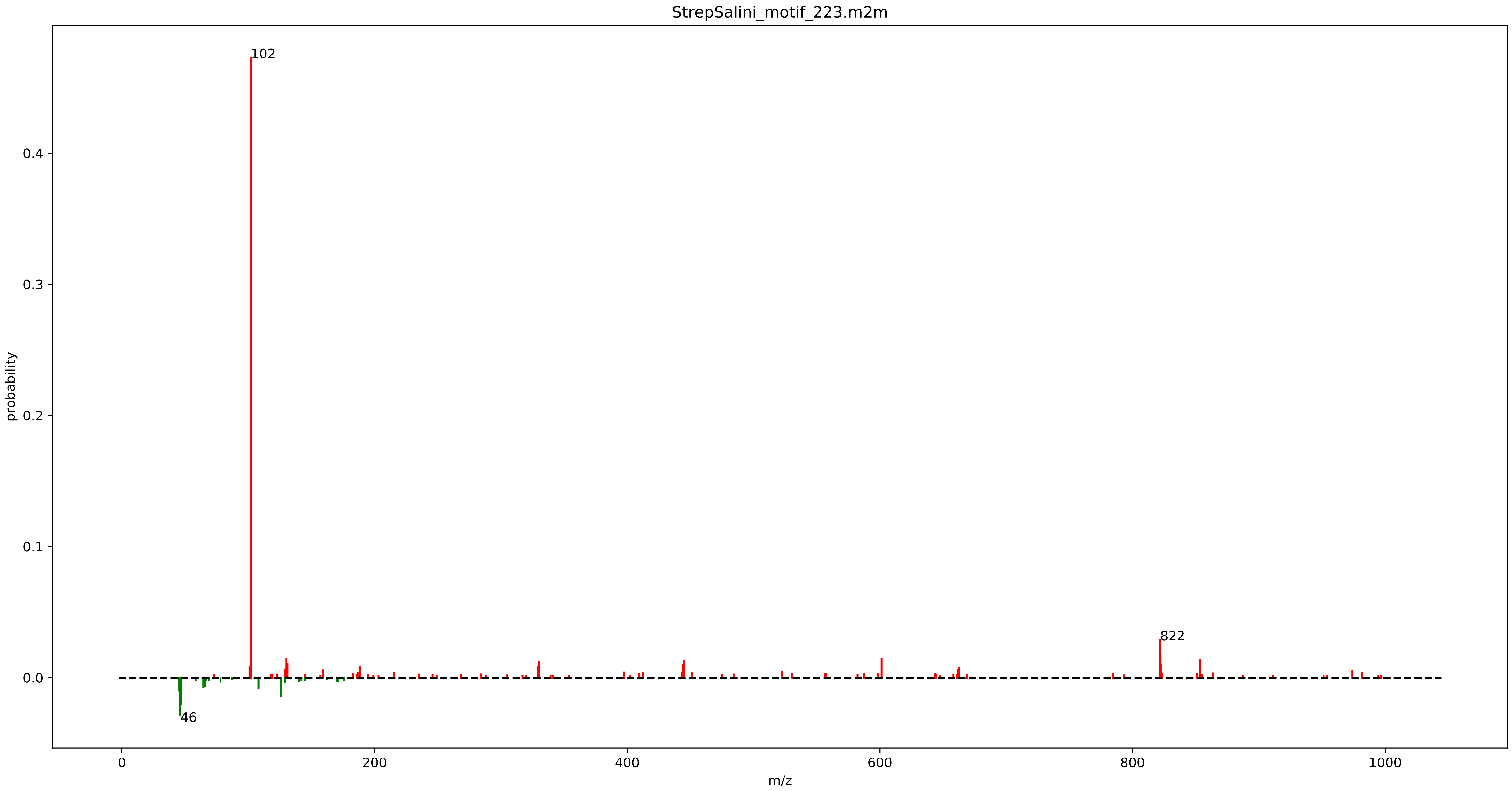

StrepSalini_motif_223.m2m
probability
m/z
0.4
0.3
0.2
0.1
0.0
0
200
400
600
800
1000
102
822
46

StrepSalini_motif_223.m2m

fragment_102.0525	: 0.47
loss_46.2500	: 0.03
fragment_821.8500	: 0.03
loss_46.5500	: 0.02
fragment_821.6500	: 0.02
loss_46.0500	: 0.02
fragment_822.1500	: 0.02
fragment_130.1500	: 0.01
fragment_601.2500	: 0.01
loss_126.0500	: 0.01
fragment_853.4500	: 0.01
fragment_445.1500	: 0.01
fragment_330.1500	: 0.01
fragment_131.1500	: 0.01
fragment_822.6500	: 0.01
fragment_822.3500	: 0.01
loss_45.5500	: 0.01
loss_45.7500	: 0.01
fragment_444.2500	: 0.01
fragment_101.0500	: 0.01
fragment_821.3500	: 0.01
loss_46.8500	: 0.01
fragment_188.1500	: 0.01
loss_108.1525	: 0.01
fragment_329.1500	: 0.01
fragment_662.8500	: 0.01
loss_64.5500	: 0.01
loss_65.5500	: 0.01
fragment_129.1500	: 0.01
fragment_661.8500	: 0.01
fragment_159.0500	: 0.01
fragment_974.0500	: 0.01

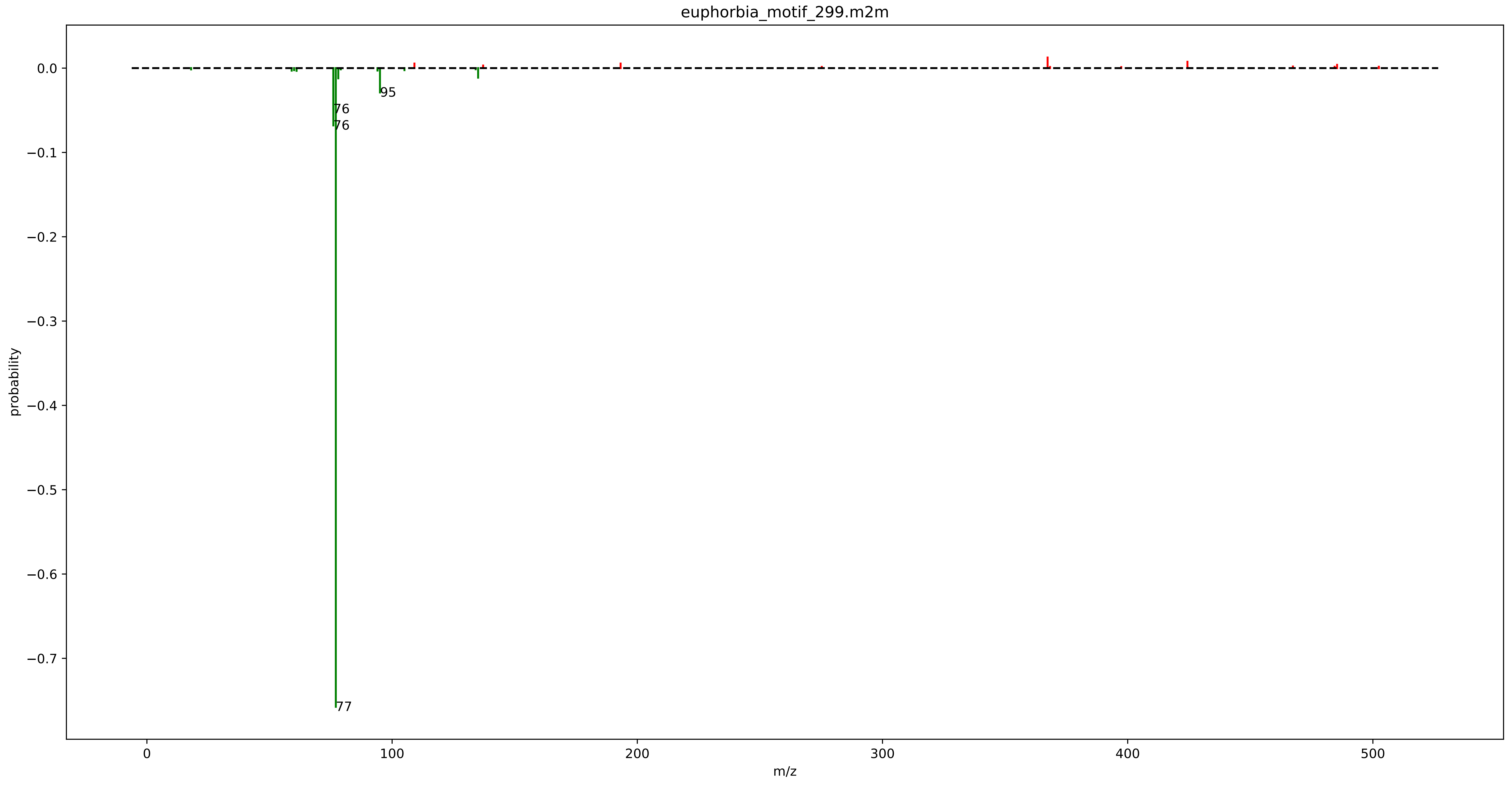
euphorbia_motif_299.m2m
probability
m/z
0.0
−0.1
−0.2
−0.3
−0.4
−0.5
−0.6
−0.7
0
100
200
300
400
500
95
76
76
77

euphorbia_motif_299.m2m

loss_77.0425	: 0.76
loss_76.0375	: 0.07
loss_76.0425	: 0.05
loss_95.0525	: 0.03
fragment_367.3325	: 0.01
loss_78.0525	: 0.01
loss_135.0825	: 0.01
fragment_424.3625	: 0.01
fragment_109.1025	: 0.01
fragment_193.1925	: 0.01

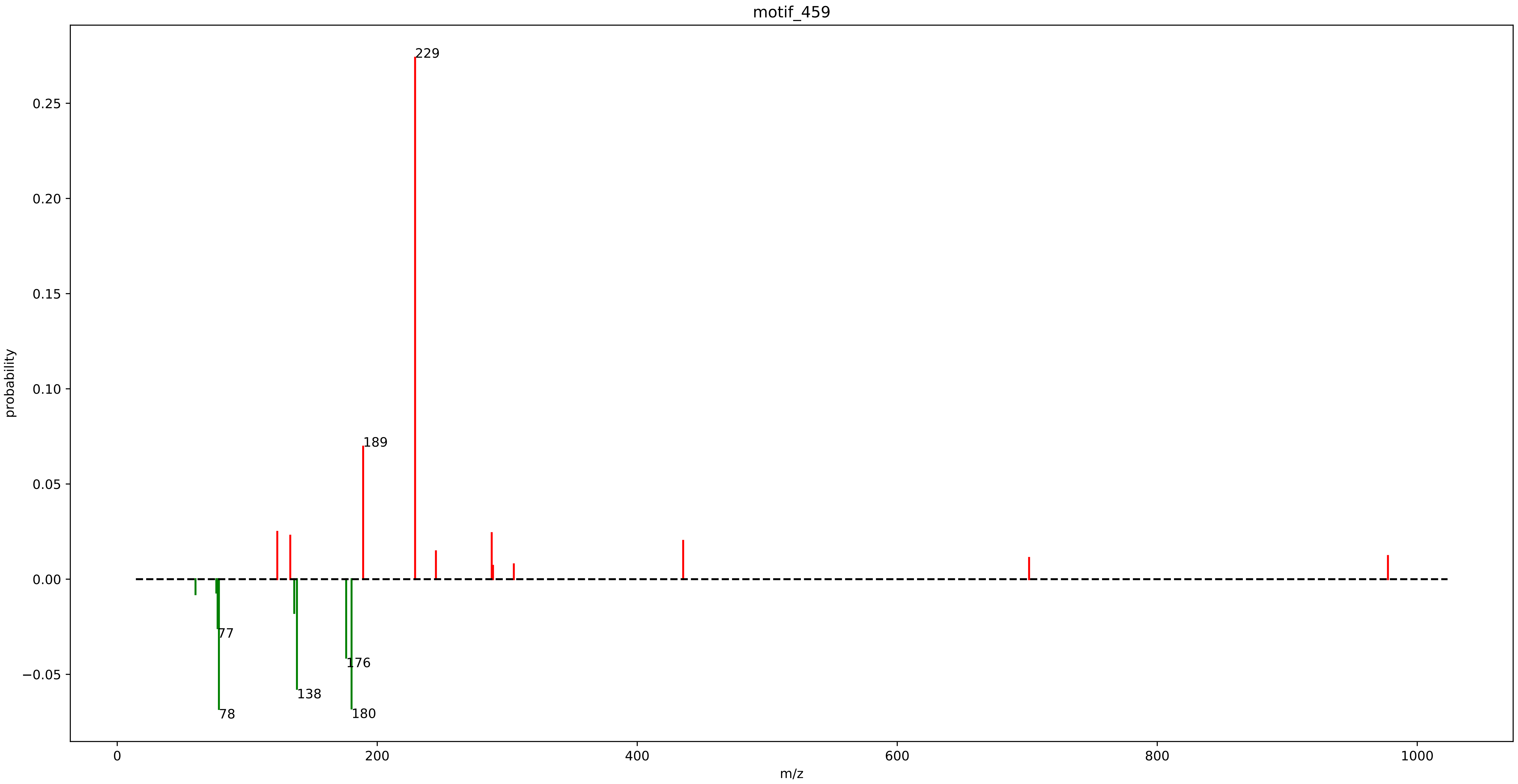

motif_459
229
189
77
176
138
78
180
probability
m/z
0.25
0.20
0.15
0.10
0.05
0.00
−0.05
0
200
400
600
800
1000

motif_459

fragment_229.1275	: 0.27
fragment_189.1625	: 0.07
loss_78.2225	: 0.07
loss_180.2525	: 0.07
loss_138.2425	: 0.06
loss_176.1175	: 0.04
loss_77.2175	: 0.03
fragment_123.1175	: 0.02
fragment_288.0625	: 0.02
fragment_133.1025	: 0.02
fragment_435.3275	: 0.02
loss_136.1575	: 0.02
fragment_245.1525	: 0.01
fragment_977.4625	: 0.01
fragment_701.4275	: 0.01
loss_60.2125	: 0.01
fragment_305.0725	: 0.01
loss_76.2225	: 0.01
fragment_289.0575	: 0.01

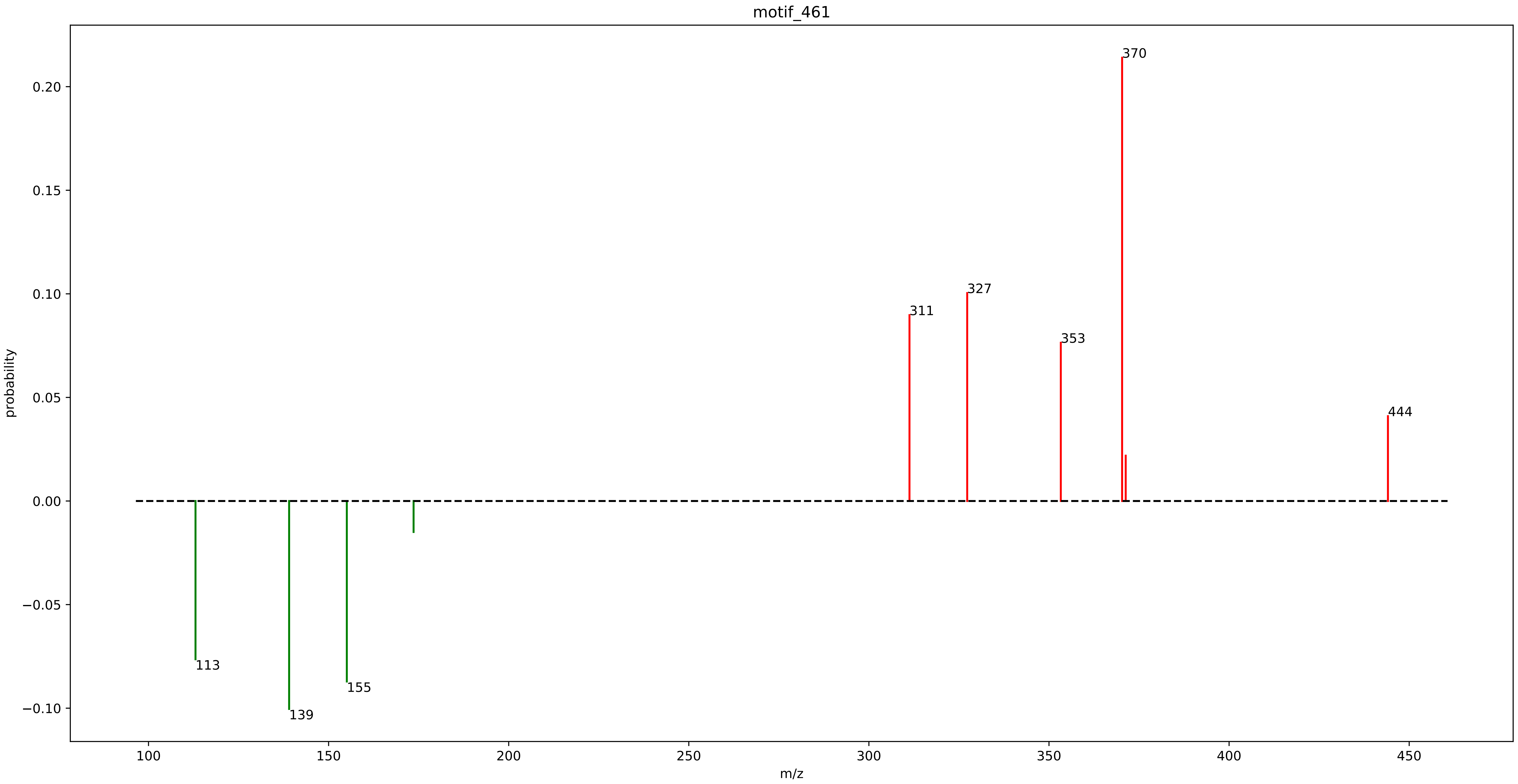

motif_461
probability
m/z
0.20
0.15
0.10
0.05
0.00
−0.05
−0.10
100
150
200
250
300
350
400
450
370
327
311
353
444
113
139
155

motif_461

fragment_370.2975	: 0.21
fragment_327.2925	: 0.10
loss_139.0375	: 0.10
fragment_311.2675	: 0.09
loss_155.0575	: 0.09
fragment_353.2775	: 0.08
loss_113.0475	: 0.08
fragment_444.1025	: 0.04
fragment_371.2975	: 0.02
loss_173.5925	: 0.01

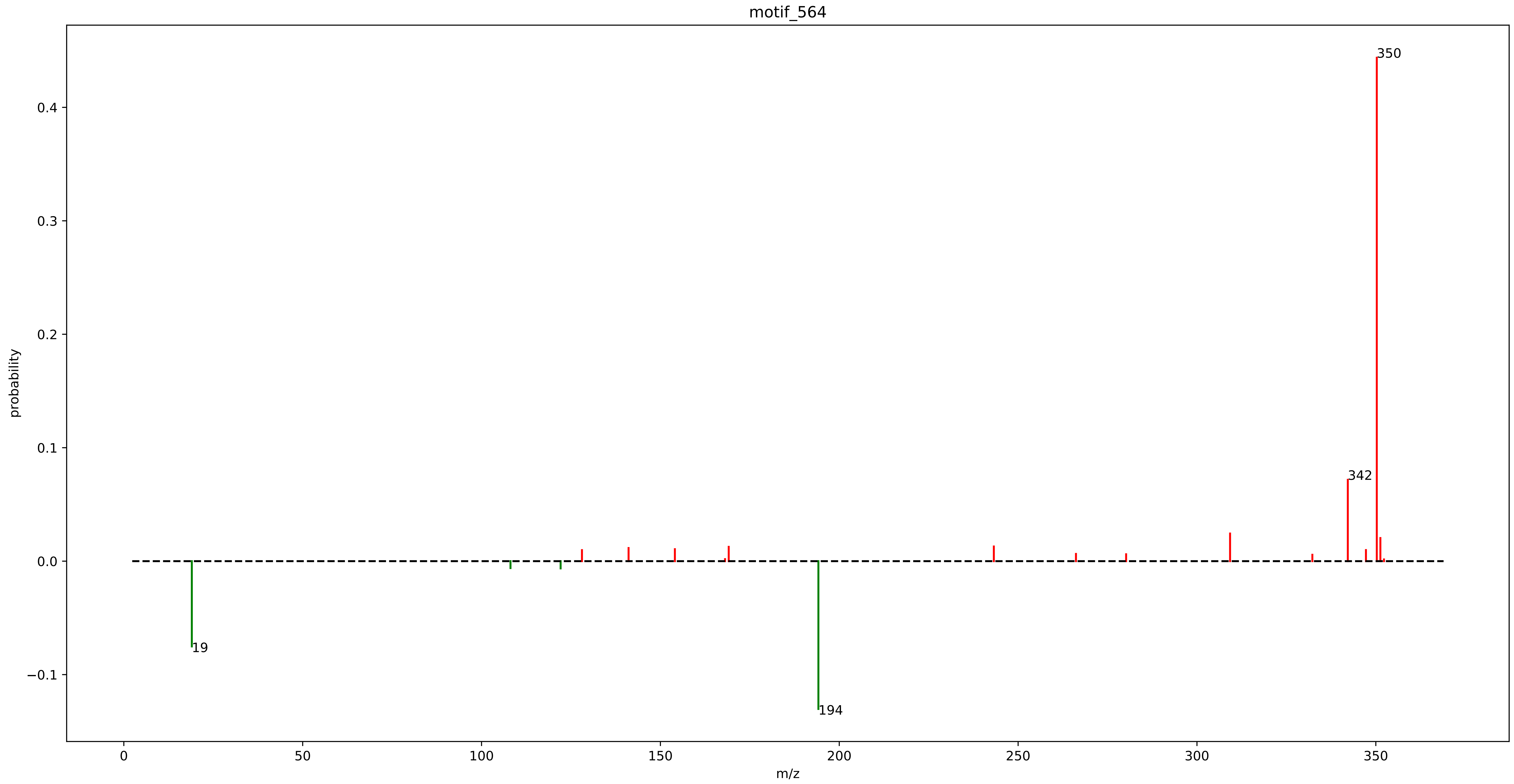
motif_564
probability
m/z
0.4
0.3
0.2
0.1
0.0
−0.1
0
50
100
150
200
250
300
350
350
342
19
194

motif_564

fragment_350.2675	: 0.44
loss_194.1675	: 0.13
loss_19.0075	: 0.08
fragment_342.1675	: 0.07
fragment_309.2525	: 0.02
fragment_351.2725	: 0.02
fragment_243.2125	: 0.01
fragment_169.0975	: 0.01
fragment_141.1025	: 0.01
fragment_154.0475	: 0.01
fragment_347.2375	: 0.01
fragment_128.0725	: 0.01
loss_122.1075	: 0.01
fragment_266.1725	: 0.01
loss_108.0925	: 0.01
fragment_280.1925	: 0.01
fragment_332.2575	: 0.01

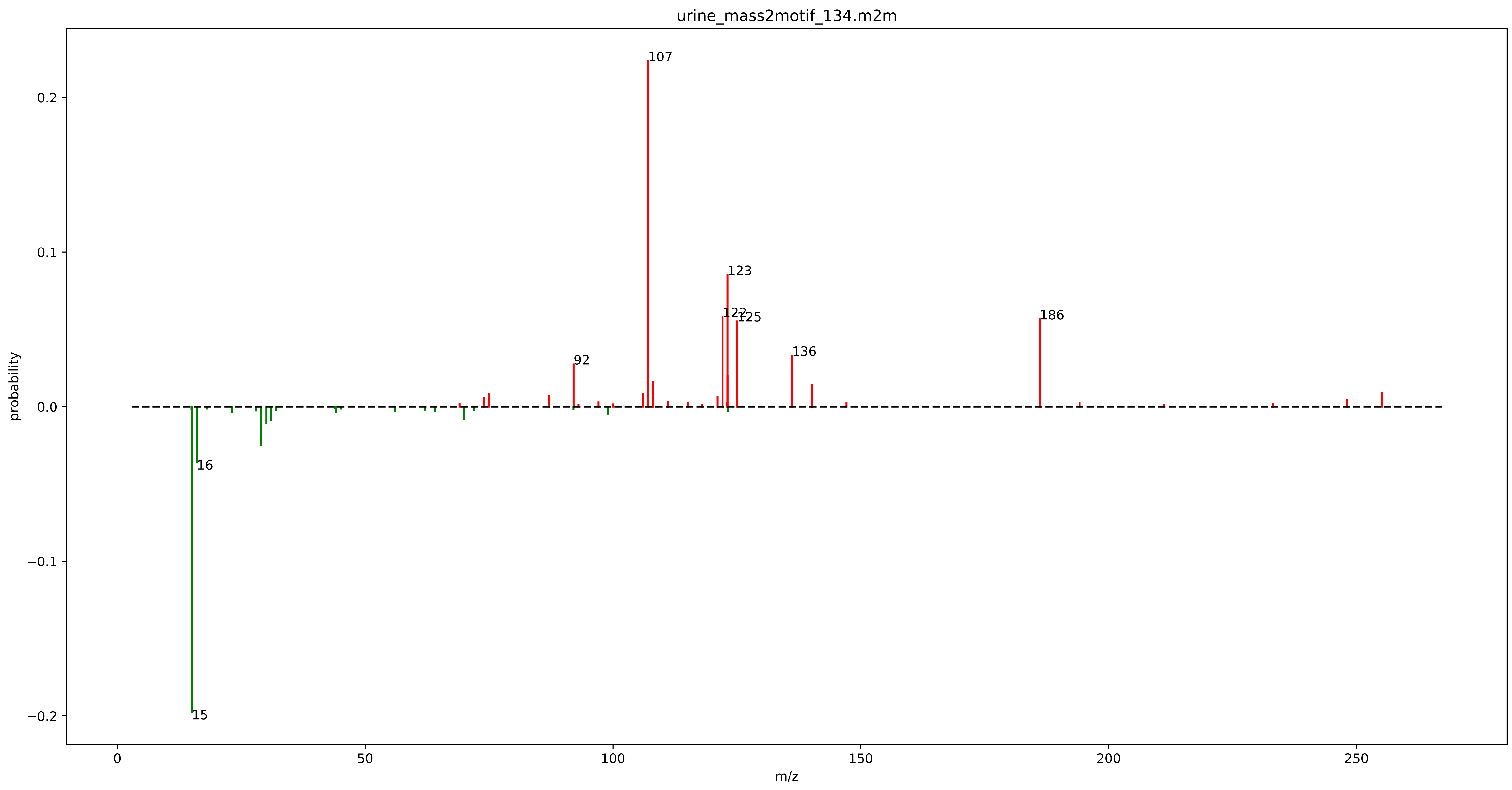
urine_mass2motif_134.m2m
probability
m/z
0.2
0.1
0.0
−0.1
−0.2
0
50
100
150
200
250
107
123
122
125
186
92
136
16
15

urine_mass2motif_134.m2m

fragment_107.0725	: 0.22
loss_15.0225	: 0.20
fragment_123.0925	: 0.09
fragment_122.0975	: 0.06
fragment_186.0875	: 0.06
fragment_125.0475	: 0.06
loss_16.0325	: 0.04
fragment_136.1125	: 0.03
fragment_92.0475	: 0.03
loss_29.0375	: 0.02
fragment_123.0975	: 0.02
fragment_108.0775	: 0.02
fragment_140.0825	: 0.01
loss_30.0475	: 0.01
fragment_255.1775	: 0.01
loss_31.0175	: 0.01
fragment_108.0675	: 0.01
loss_70.0075	: 0.01
fragment_75.0125	: 0.01
fragment_106.0675	: 0.01
fragment_87.0575	: 0.01
fragment_121.0875	: 0.01
fragment_74.0075	: 0.01
fragment_140.0725	: 0.01

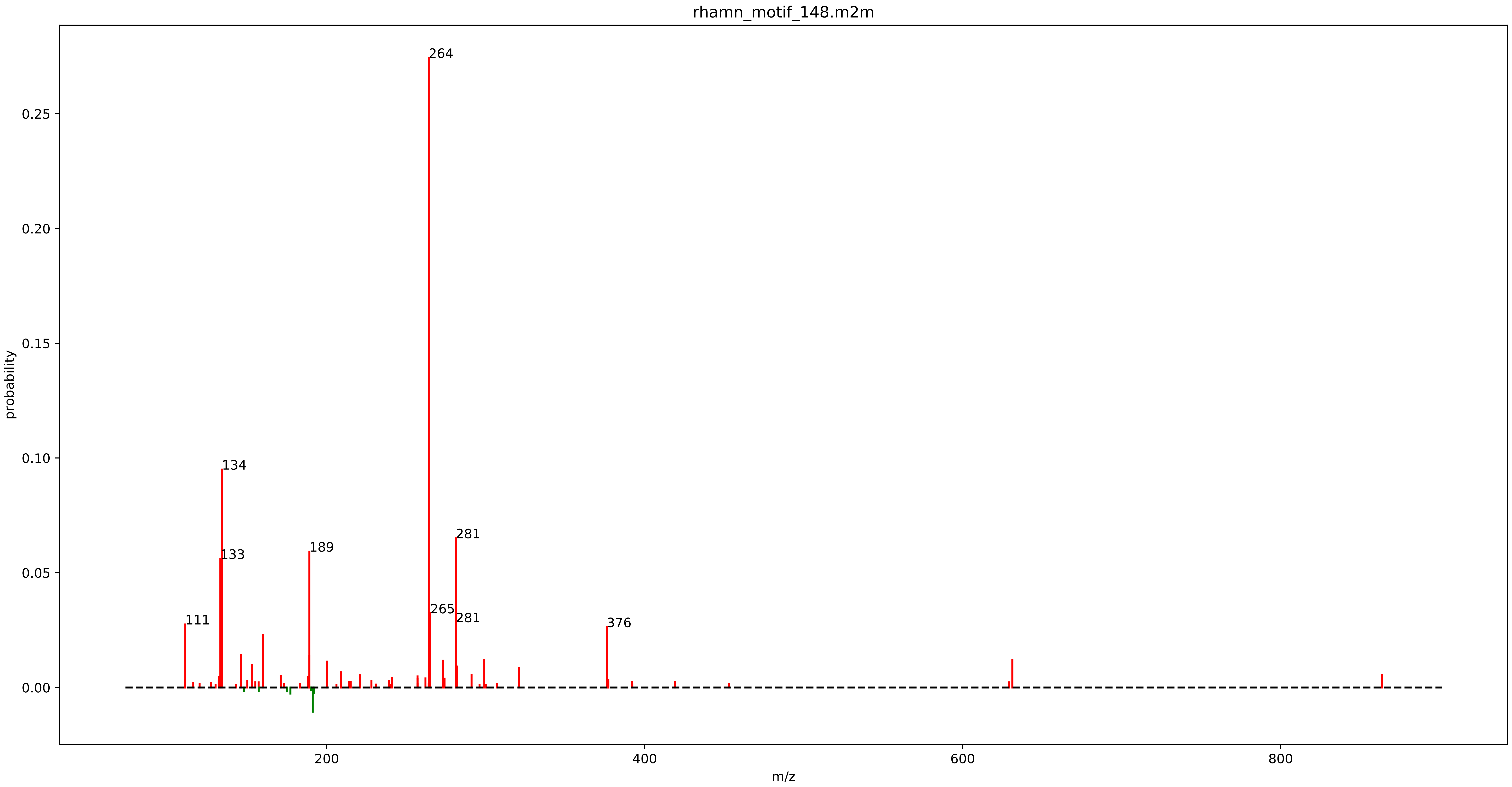
rhamn_motif_148.m2m
probability
m/z
0.25
0.20
0.15
0.10
0.05
0.00
200
400
600
800
264
134
281
189
133
265
281
111
376

rhamn_motif_148.m2m

fragment_264.1025	: 0.27
fragment_134.0625	: 0.09
fragment_281.1275	: 0.07
fragment_189.0675	: 0.06
fragment_133.0525	: 0.06
fragment_265.1075	: 0.03
fragment_281.1325	: 0.03
fragment_111.0175	: 0.03
fragment_376.1325	: 0.03
fragment_160.0375	: 0.02
fragment_376.1275	: 0.02
fragment_146.0625	: 0.01
fragment_189.0625	: 0.01
fragment_265.1025	: 0.01
fragment_299.0525	: 0.01
fragment_631.2425	: 0.01
fragment_273.1025	: 0.01
fragment_200.0575	: 0.01
loss_191.1675	: 0.01
fragment_153.0675	: 0.01
fragment_282.1325	: 0.01
fragment_321.0325	: 0.01
fragment_209.0975	: 0.01
fragment_863.7075	: 0.01
fragment_291.1125	: 0.01
fragment_221.0975	: 0.01

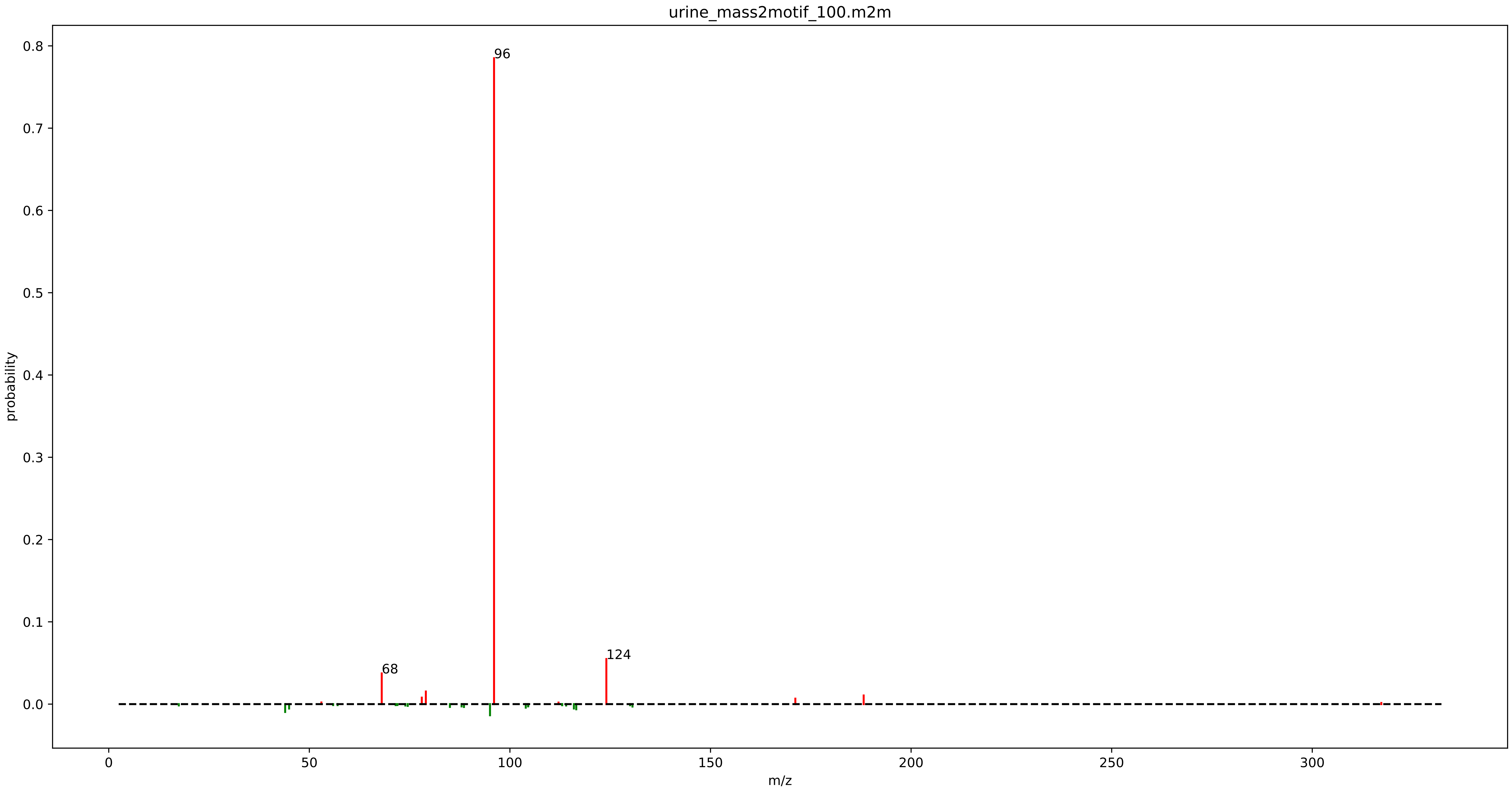

urine_mass2motif_100.m2m
probability
m/z
96
68
124
0.8
0.7
0.6
0.5
0.4
0.3
0.2
0.1
0.0
0
50
100
150
200
250
300

urine_mass2motif_100.m2m

fragment_96.0425	: 0.78
fragment_124.0375	: 0.05
fragment_68.0475	: 0.04
fragment_79.0425	: 0.02
loss_95.0375	: 0.01
fragment_188.1775	: 0.01
loss_43.9725	: 0.01
fragment_78.0325	: 0.01
fragment_171.1475	: 0.01
loss_116.5375	: 0.01
loss_44.9575	: 0.01
loss_115.9475	: 0.01

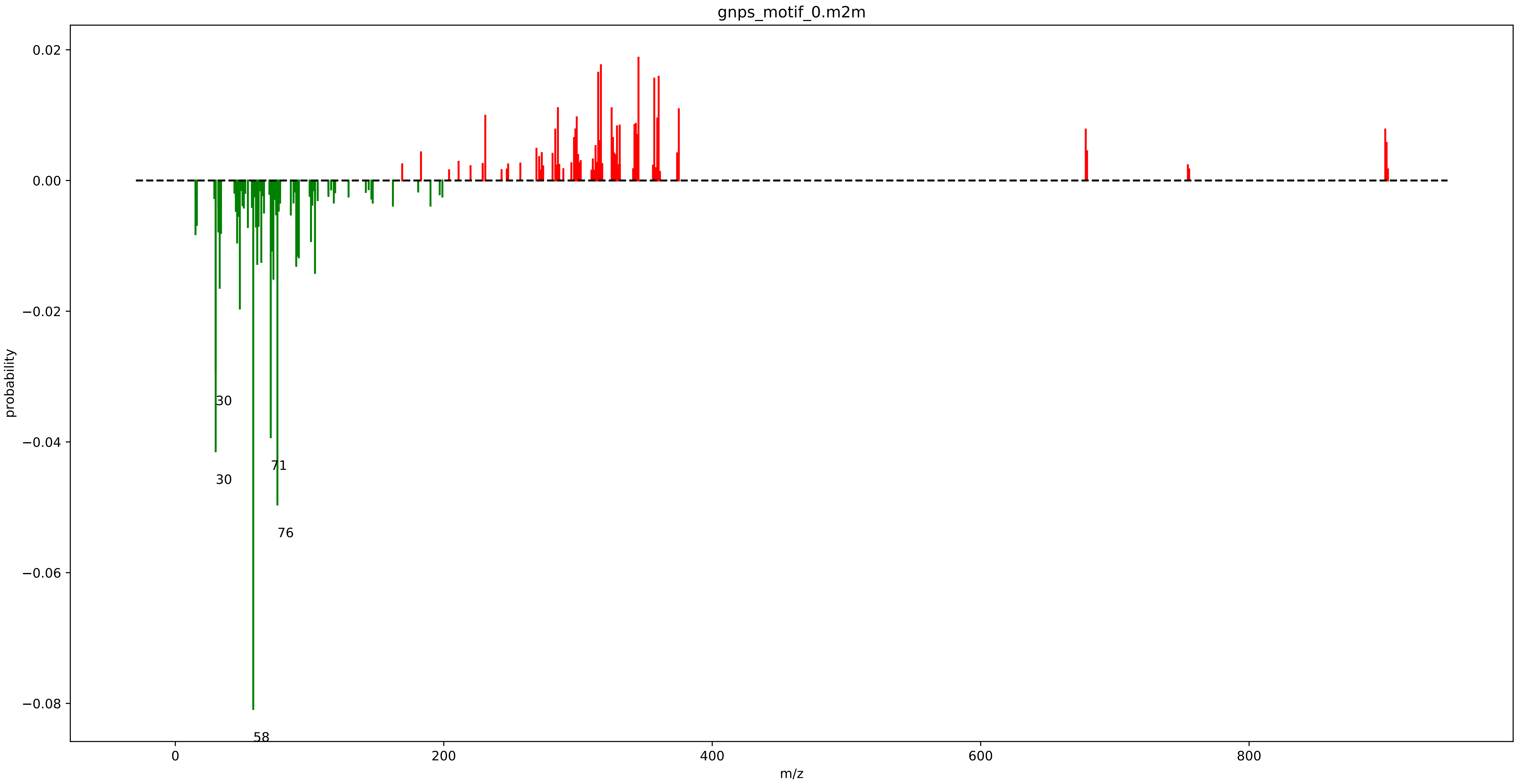
gnps_motif_0.m2m
probability
0.02
0.00
−0.02
−0.04
−0.06
−0.08
m/z
0
200
400
600
800
30
30
71
76
58

gnps_motif_0.m2m

loss_58.0425 : 0.08
loss_76.0525 : 0.05
loss_30.0475 : 0.04
loss_71.0725 : 0.04
loss_30.0425 : 0.03
loss_48.0575 : 0.02
fragment_345.0625 : 0.02
fragment_317.0675 : 0.02
fragment_315.0525 : 0.02
loss_33.0325 : 0.02
fragment_360.0875 : 0.02
fragment_356.8275 : 0.02
loss_73.0625 : 0.02
loss_104.0475 : 0.01
loss_90.0675 : 0.01
loss_61.0275 : 0.01
loss_64.0525 : 0.01
loss_92.0475 : 0.01
loss_91.0775 : 0.01
fragment_285.0375 : 0.01
fragment_325.0675 : 0.01
fragment_375.1075 : 0.01
loss_72.0575 : 0.01
fragment_230.9325 : 0.01
fragment_299.0575 : 0.01
fragment_359.0775 : 0.01
loss_46.0425 : 0.01
loss_101.0625 : 0.01
fragment_343.0825 : 0.01
fragment_342.0725 : 0.01
fragment_343.0775 : 0.01
fragment_331.0825 : 0.01
fragment_329.0675 : 0.01
loss_15.0225 : 0.01
loss_34.0425 : 0.01
fragment_298.0475 : 0.01
fragment_678.2875 : 0.01
loss_32.0375 : 0.01
fragment_901.4425 : 0.01
fragment_283.0625 : 0.01
loss_54.0475 : 0.01
loss_60.0575 : 0.01
fragment_344.0875 : 0.01
loss_62.0375 : 0.01
loss_16.0325 : 0.01
fragment_326.0775 : 0.01
fragment_297.0425 : 0.01
fragment_316.0575 : 0.01
fragment_902.4475 : 0.01
loss_47.0525 : 0.01
fragment_313.0675 : 0.01
loss_86.0375 : 0.01
fragment_297.0775 : 0.01
loss_75.0475 : 0.01

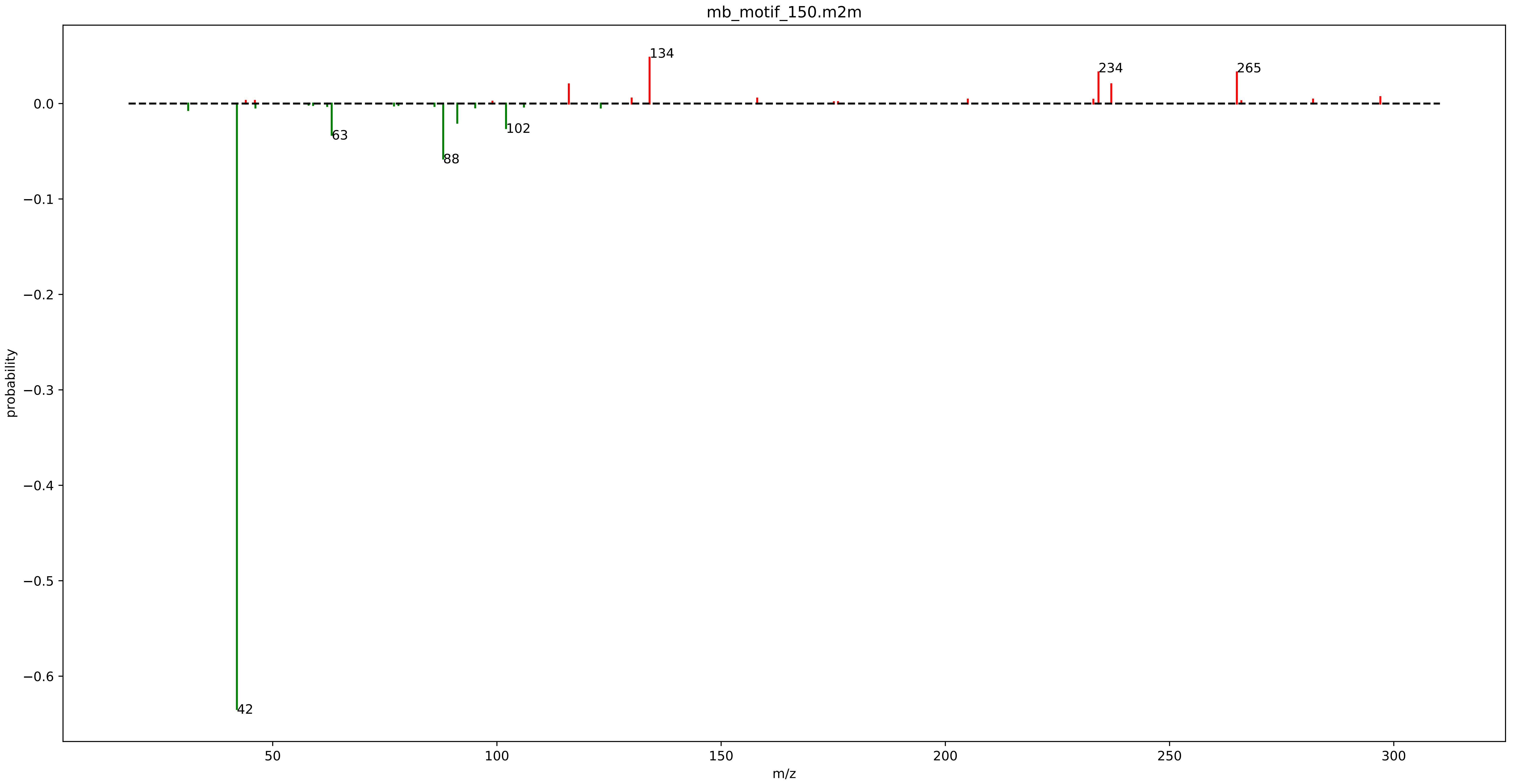

mb_motif_150.m2m
probability
m/z
0.0
−0.1
−0.2
−0.3
−0.4
−0.5
−0.6
50
100
150
200
250
300
134
234
265
63
88
102
42

mb_motif_150.m2m

loss_42.0125	: 0.63
loss_88.0175	: 0.06
fragment_134.0425	: 0.05
fragment_234.1475	: 0.03
loss_63.1525	: 0.03
fragment_265.0025	: 0.03
loss_102.0325	: 0.03
fragment_237.0025	: 0.02
fragment_116.0325	: 0.02
loss_91.1525	: 0.02
loss_31.1525	: 0.01
fragment_297.0025	: 0.01
fragment_130.0475	: 0.01
fragment_158.0425	: 0.01

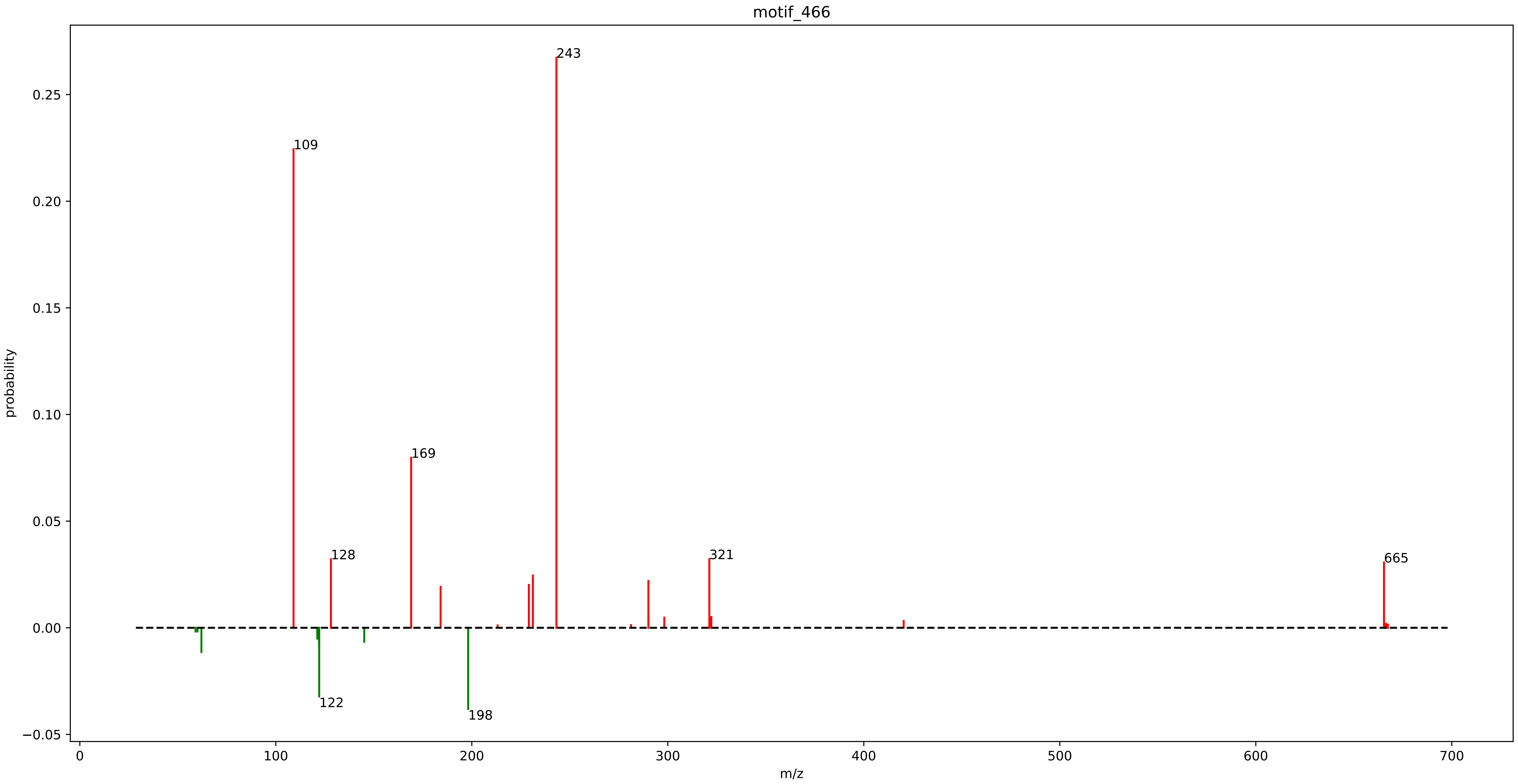

motif_466
probability
m/z
0.25
0.20
0.15
0.10
0.05
0.00
−0.05
0
100
200
300
400
500
600
700
109
243
169
128
321
665
122
198

motif_466

fragment_243.1675	: 0.27
fragment_109.0325	: 0.22
fragment_169.0475	: 0.08
loss_198.1375	: 0.04
fragment_321.1825	: 0.03
loss_122.1375	: 0.03
fragment_128.1075	: 0.03
fragment_665.4025	: 0.03
fragment_231.1675	: 0.02
fragment_290.0975	: 0.02
fragment_229.1075	: 0.02
fragment_184.0725	: 0.02
loss_62.0325	: 0.01
loss_122.0575	: 0.01
loss_145.1075	: 0.01
fragment_290.0925	: 0.01
fragment_322.1825	: 0.01
loss_121.1375	: 0.01

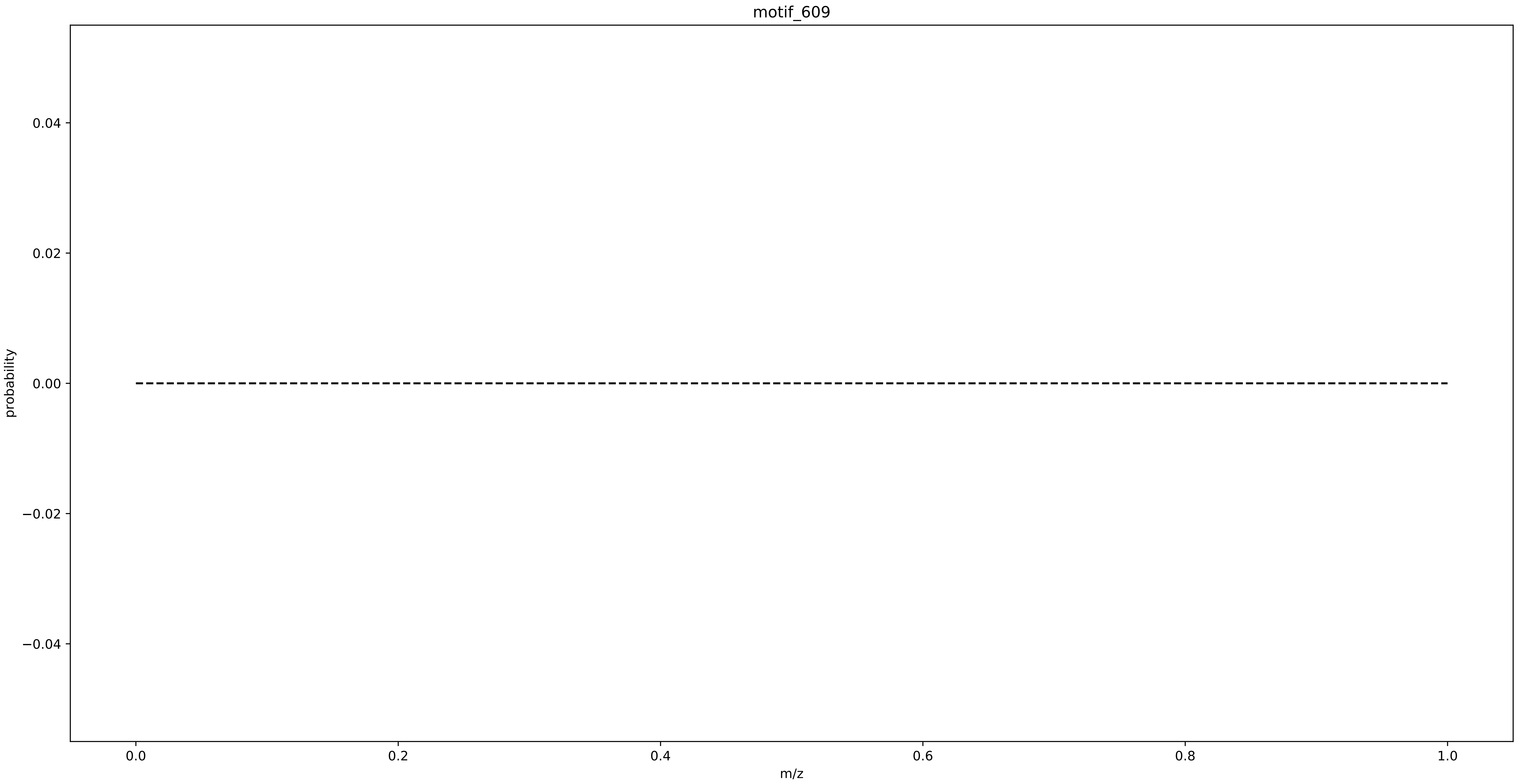
motif_609
probability
m/z
0.04
0.02
0.00
−0.02
−0.04
0.0
0.2
0.4
0.6
0.8
1.0

motif_609

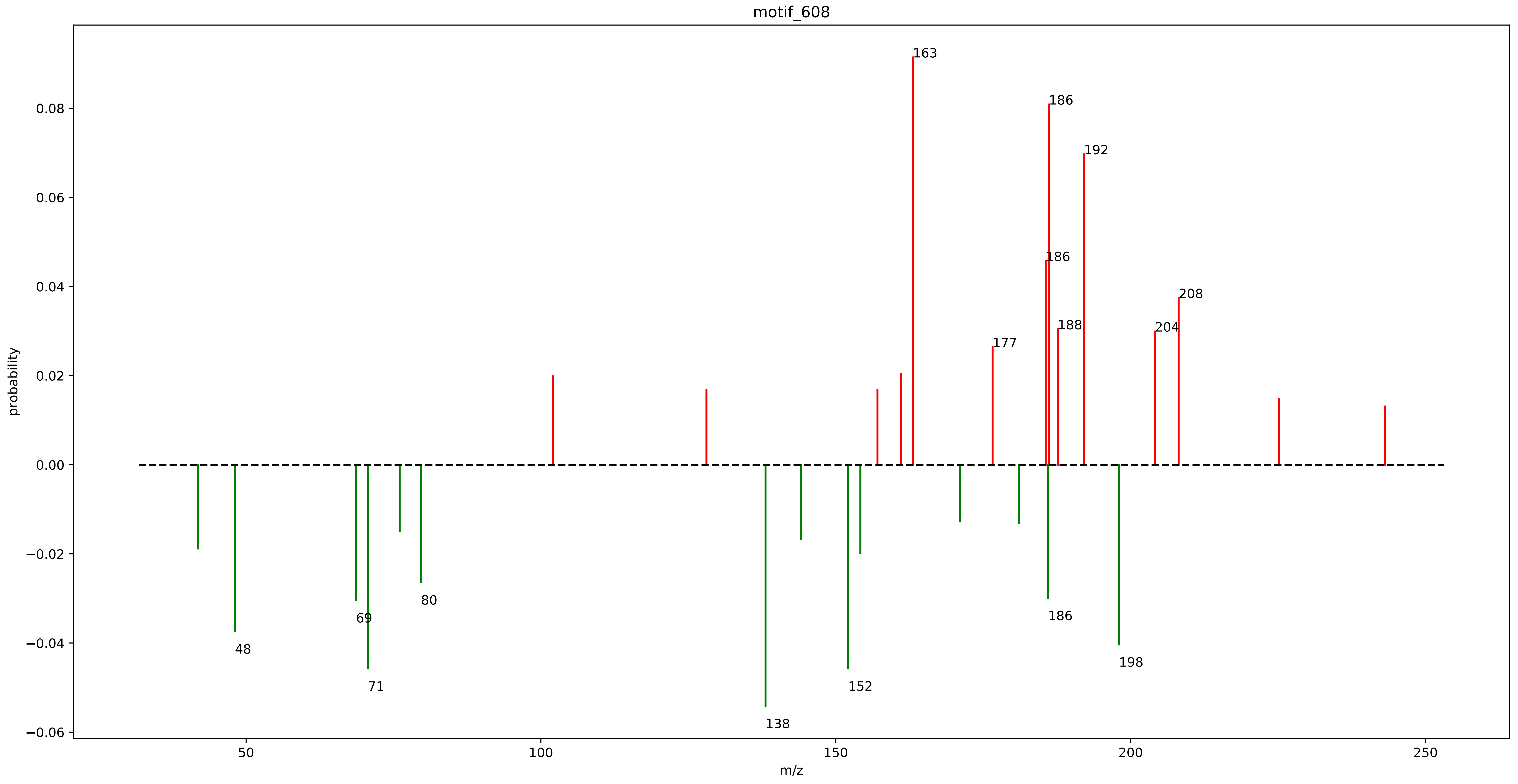

motif_608
probability
m/z
0.08
0.06
0.04
0.02
0.00
−0.02
−0.04
−0.06
50
100
150
200
250
163
186
192
186
208
188
204
177
48
69
71
80
138
152
186
198

motif_608

fragment_163.0725	: 0.09
fragment_186.1275	: 0.08
fragment_192.1025	: 0.07
loss_138.0875	: 0.05
fragment_185.5975	: 0.05
loss_70.6675	: 0.05
loss_152.1025	: 0.05
loss_198.0025	: 0.04
fragment_208.1425	: 0.04
loss_48.1175	: 0.04
loss_68.6325	: 0.03
fragment_187.6325	: 0.03
loss_186.0025	: 0.03
fragment_204.1025	: 0.03
loss_79.6675	: 0.03
fragment_176.5925	: 0.03
fragment_161.0575	: 0.02
loss_154.1725	: 0.02
fragment_102.0875	: 0.02
loss_41.8825	: 0.02
fragment_128.0725	: 0.02
fragment_157.0725	: 0.02
loss_144.0875	: 0.02
fragment_225.1075	: 0.01
loss_76.0525	: 0.01
loss_181.0775	: 0.01
fragment_243.1225	: 0.01
loss_171.0925	: 0.01

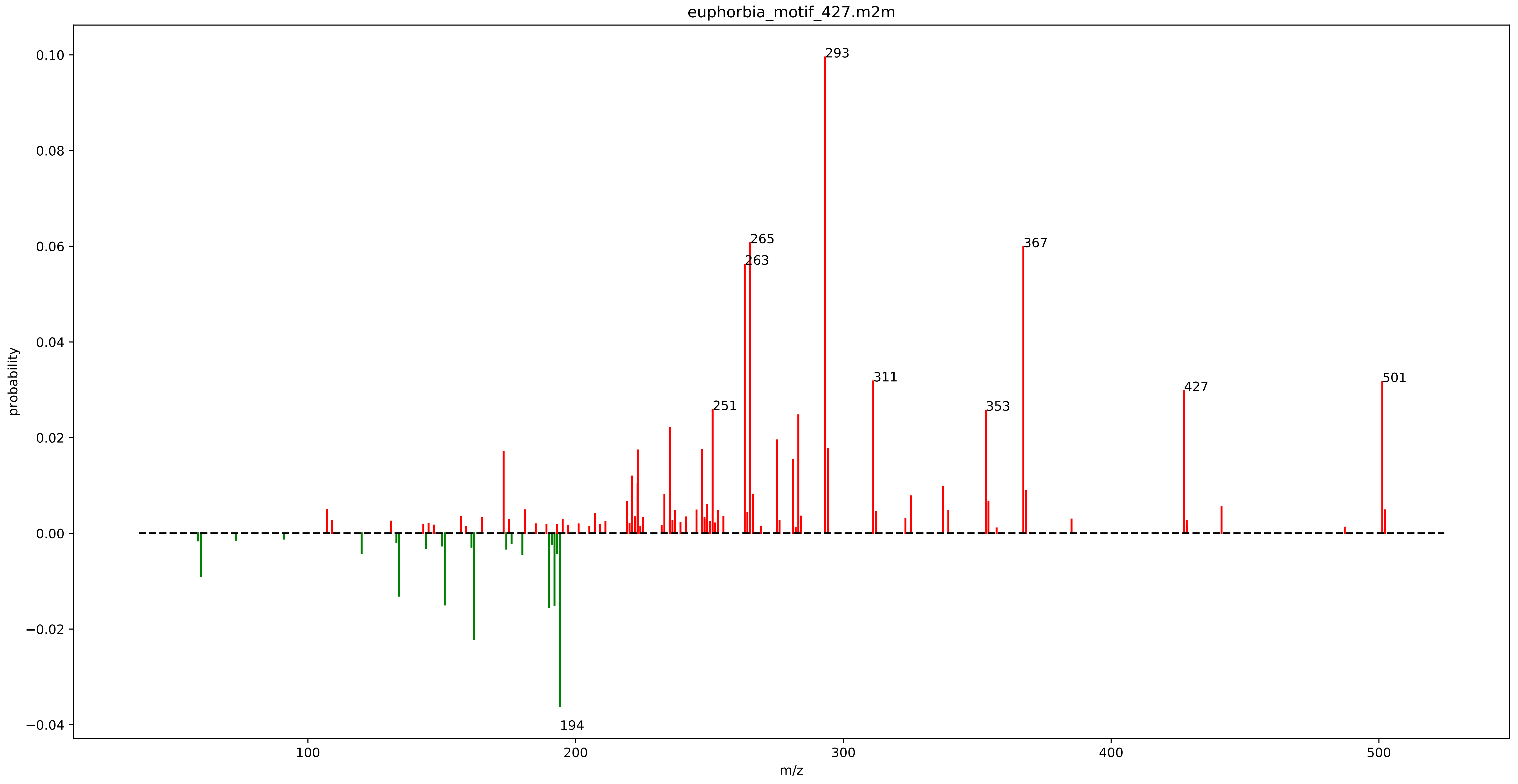

euphorbia_motif_427.m2m
probability
m/z
0.10
0.08
0.06
0.04
0.02
0.00
−0.02
−0.04
100
200
300
400
500
293
265
263
367
311
501
427
353
251
194

euphorbia_motif_427.m2m

fragment_293.1575	: 0.10
fragment_265.1575	: 0.06
fragment_367.1925	: 0.06
fragment_263.1425	: 0.06
loss_194.0775	: 0.04
fragment_311.1625	: 0.03
fragment_501.2475	: 0.03
fragment_427.2125	: 0.03
fragment_251.1425	: 0.03
fragment_353.1775	: 0.03
fragment_283.1675	: 0.02
loss_162.0875	: 0.02
fragment_235.1475	: 0.02
fragment_353.1725	: 0.02
fragment_275.1425	: 0.02
fragment_294.1575	: 0.02
fragment_247.1475	: 0.02
fragment_223.1475	: 0.02
fragment_173.0975	: 0.02
fragment_281.1525	: 0.02
loss_190.0825	: 0.02
loss_192.0975	: 0.01
loss_151.0825	: 0.01
loss_134.0575	: 0.01
fragment_221.1325	: 0.01
fragment_223.1125	: 0.01
fragment_337.1775	: 0.01
loss_60.0175	: 0.01
fragment_368.1925	: 0.01
fragment_233.1325	: 0.01
fragment_266.1625	: 0.01
fragment_325.1825	: 0.01
fragment_354.1775	: 0.01
fragment_219.1175	: 0.01
fragment_249.1275	: 0.01
fragment_441.2275	: 0.01

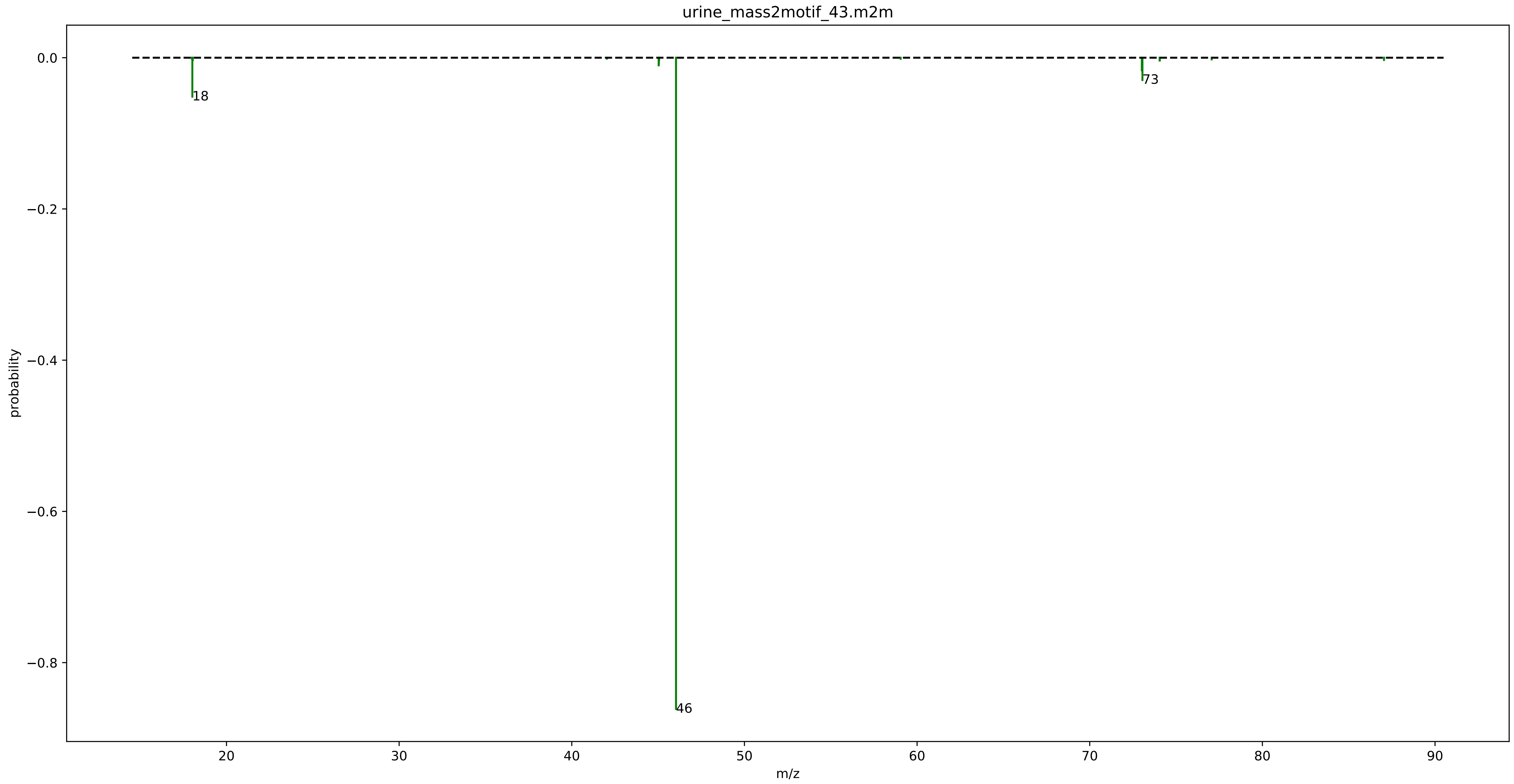

urine_mass2motif_43.m2m
0.0
−0.2
−0.4
−0.6
−0.8
probability
18
46
73
20
30
40
50
60
70
80
90
m/z

urine_mass2motif_43.m2m

loss_46.0375	: 0.86
loss_18.0225	: 0.05
loss_73.0525	: 0.03
loss_73.0175	: 0.02
loss_45.0325	: 0.01
loss_45.0425	: 0.01

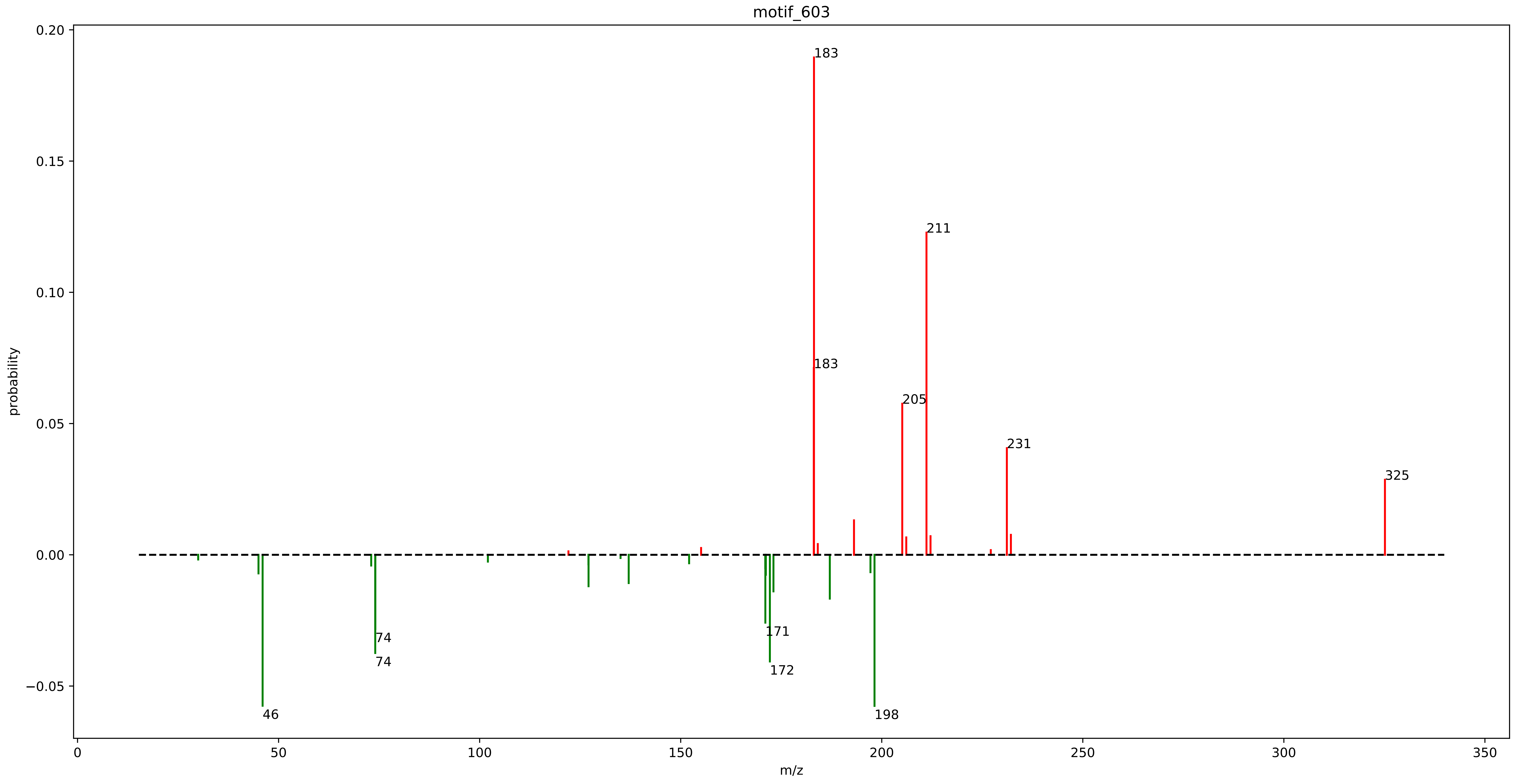

motif_603
probability
m/z
0.20
0.15
0.10
0.05
0.00
−0.05
0
50
100
150
200
250
300
350
183
183
211
205
231
325
46
74
74
171
172
198

motif_603

fragment_183.1475	: 0.19
fragment_211.1225	: 0.12
fragment_183.0925	: 0.07
loss_198.1975	: 0.06
fragment_205.0975	: 0.06
loss_46.0275	: 0.06
loss_172.1775	: 0.04
fragment_231.1125	: 0.04
loss_74.0375	: 0.04
fragment_325.1375	: 0.03
loss_74.0575	: 0.03
loss_171.0525	: 0.03
loss_187.0825	: 0.02
loss_173.0675	: 0.01
fragment_193.0975	: 0.01
loss_127.0825	: 0.01
loss_137.0675	: 0.01
loss_171.1775	: 0.01
fragment_232.1175	: 0.01
fragment_212.1275	: 0.01
loss_45.0025	: 0.01
loss_197.1925	: 0.01
fragment_206.0975	: 0.01
loss_171.0325	: 0.01

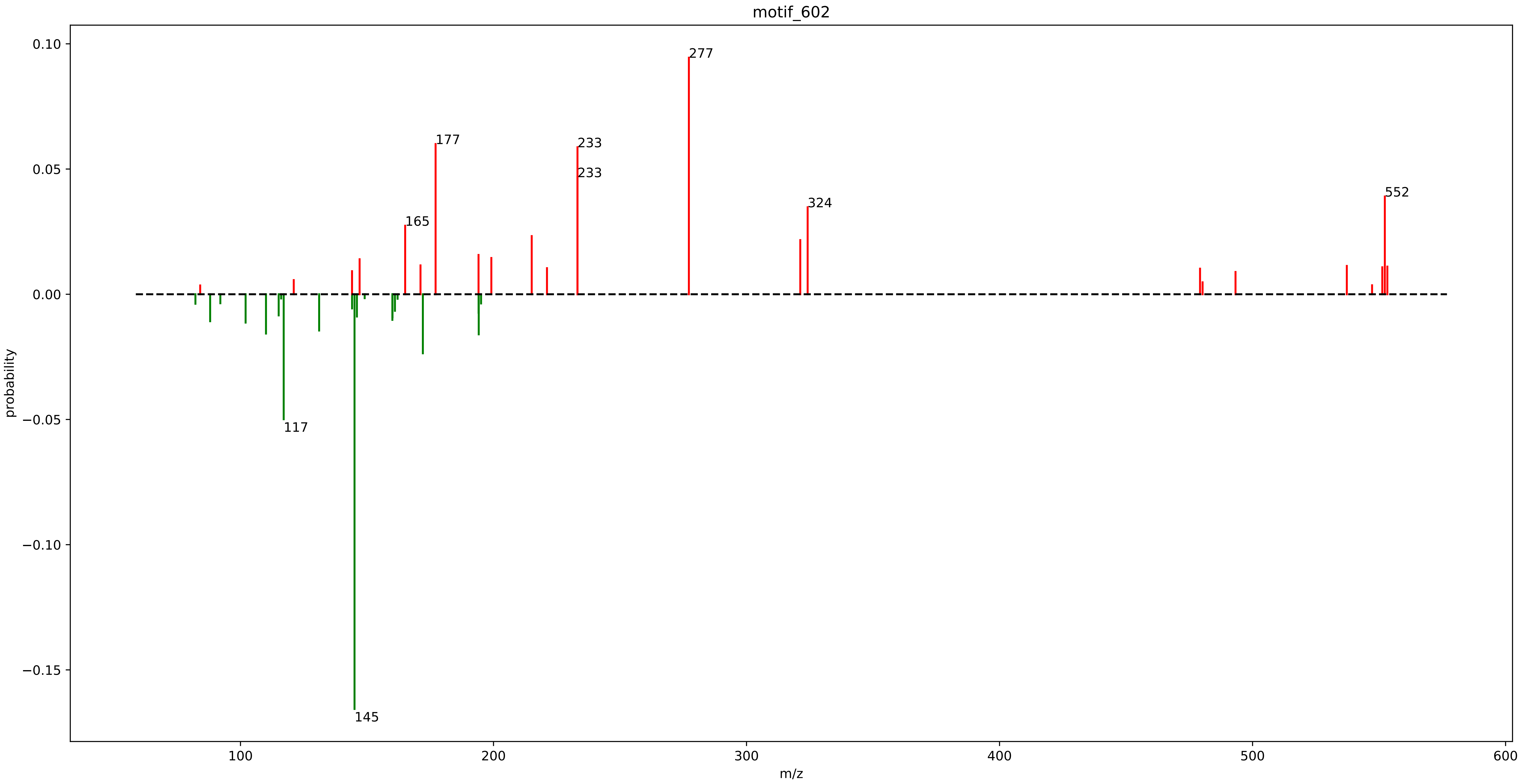

motif_602
0.10
0.05
0.00
−0.05
−0.10
−0.15
probability
100
200
300
400
500
600
m/z
277
177
233
233
324
552
165
117
145

motif_602

loss_145.0725	: 0.17
fragment_277.2175	: 0.09
fragment_177.1125	: 0.06
fragment_233.1925	: 0.06
loss_117.0775	: 0.05
fragment_233.1875	: 0.05
fragment_552.2725	: 0.04
fragment_324.1575	: 0.03
fragment_165.0875	: 0.03
loss_172.0825	: 0.02
fragment_215.1025	: 0.02
fragment_321.2425	: 0.02
loss_194.1625	: 0.02
fragment_194.0825	: 0.02
loss_110.0825	: 0.02
loss_131.0875	: 0.01
fragment_199.1475	: 0.01
fragment_147.0825	: 0.01
fragment_171.1475	: 0.01
fragment_537.2525	: 0.01
loss_102.0275	: 0.01
fragment_553.2775	: 0.01
fragment_551.2675	: 0.01
loss_88.0125	: 0.01
fragment_221.1375	: 0.01
fragment_479.2425	: 0.01
loss_160.0325	: 0.01
fragment_144.0875	: 0.01
loss_160.0775	: 0.01
fragment_493.2625	: 0.01
loss_146.0175	: 0.01
loss_115.0975	: 0.01
loss_194.1125	: 0.01
loss_161.0475	: 0.01
loss_144.1025	: 0.01
fragment_121.0525	: 0.01

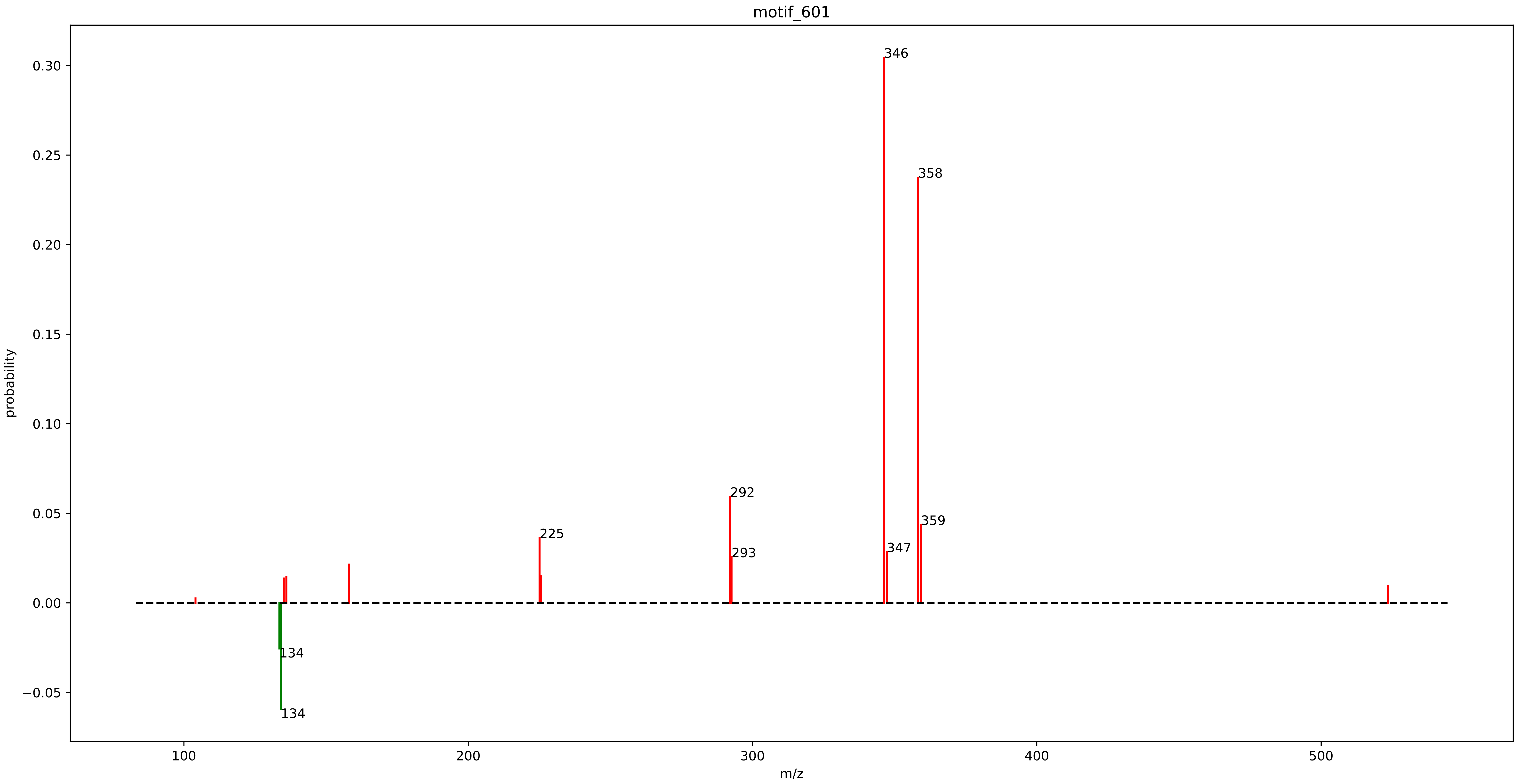

motif_601
probability
m/z
0.30
0.25
0.20
0.15
0.10
0.05
0.00
−0.05
100
200
300
400
500
346
358
292
225
293
347
359
134
134

motif_601

fragment_346.2325	: 0.30
fragment_358.2325	: 0.24
fragment_292.1225	: 0.06
loss_134.0725	: 0.06
fragment_359.2375	: 0.04
fragment_225.0875	: 0.04
fragment_347.2375	: 0.03
fragment_292.6225	: 0.03
loss_133.5725	: 0.03
fragment_158.0475	: 0.02
fragment_225.5875	: 0.01
fragment_136.0375	: 0.01
fragment_135.1025	: 0.01
fragment_523.5075	: 0.01

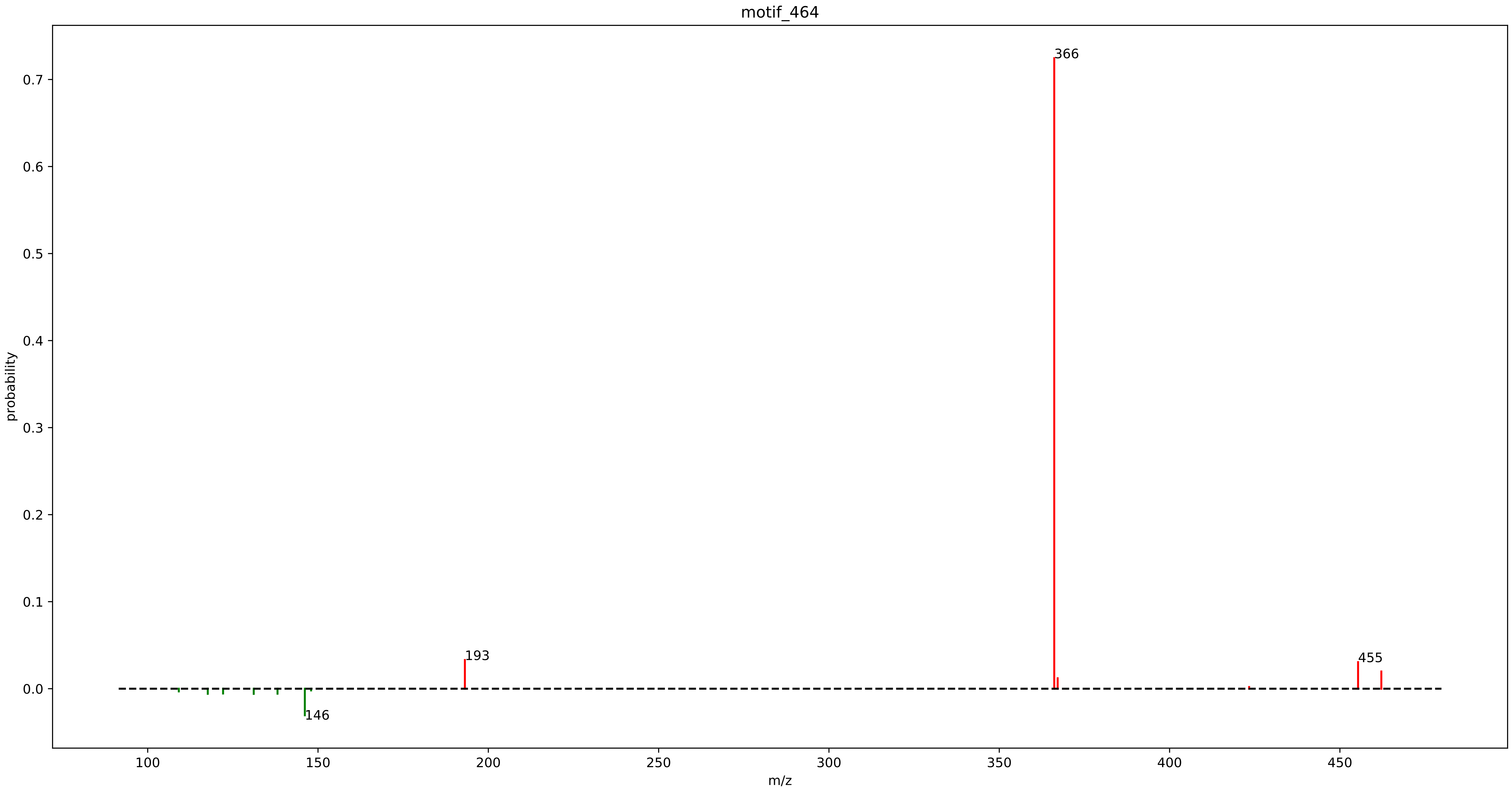

motif_464
366
193
455
146
probability
0.7
0.6
0.5
0.4
0.3
0.2
0.1
0.0
100
150
200
250
300
350
400
450
m/z

motif_464

fragment_366.1375	: 0.72
fragment_193.1175	: 0.03
loss_146.1275	: 0.03
fragment_455.3325	: 0.03
fragment_462.1625	: 0.02
fragment_367.1425	: 0.01
loss_131.1275	: 0.01
loss_117.6375	: 0.01
loss_138.1125	: 0.01
loss_122.1275	: 0.01

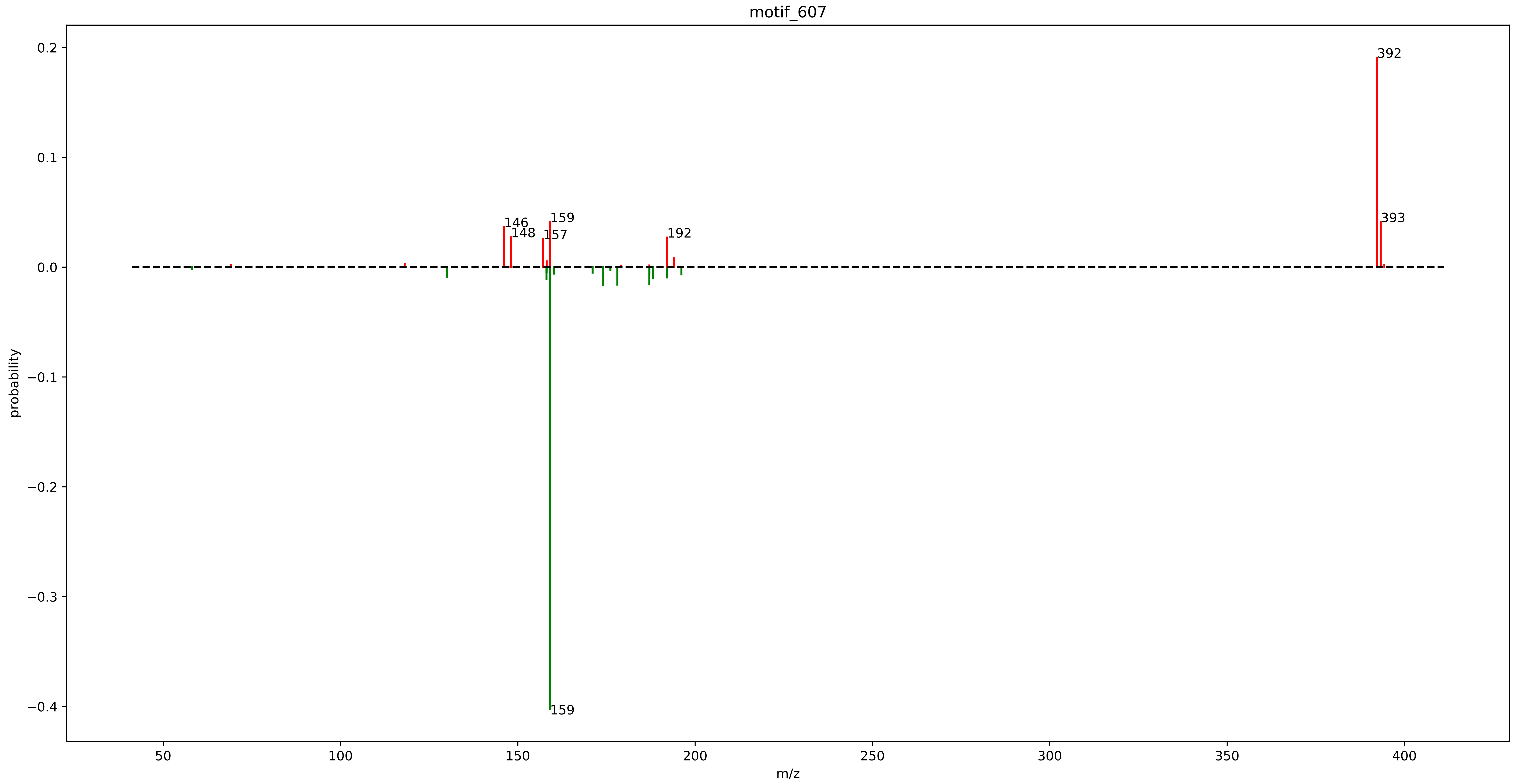
motif_607
392
393
146
148
157
159
192
159
probability
m/z
0.2
0.1
0.0
−0.1
−0.2
−0.3
−0.4
50
100
150
200
250
300
350
400

motif_607

loss_159.0875	: 0.40
fragment_392.3125	: 0.19
fragment_159.0925	: 0.04
fragment_393.3175	: 0.04
fragment_146.0975	: 0.04
fragment_148.0725	: 0.03
fragment_192.1025	: 0.03
fragment_157.1325	: 0.03
loss_174.0975	: 0.02
loss_178.0625	: 0.02
loss_187.0825	: 0.02
loss_158.0875	: 0.01
loss_188.1125	: 0.01
loss_192.0975	: 0.01
loss_130.1075	: 0.01
fragment_194.0825	: 0.01
loss_196.1375	: 0.01
loss_160.1575	: 0.01
fragment_158.1175	: 0.01
loss_187.1175	: 0.01

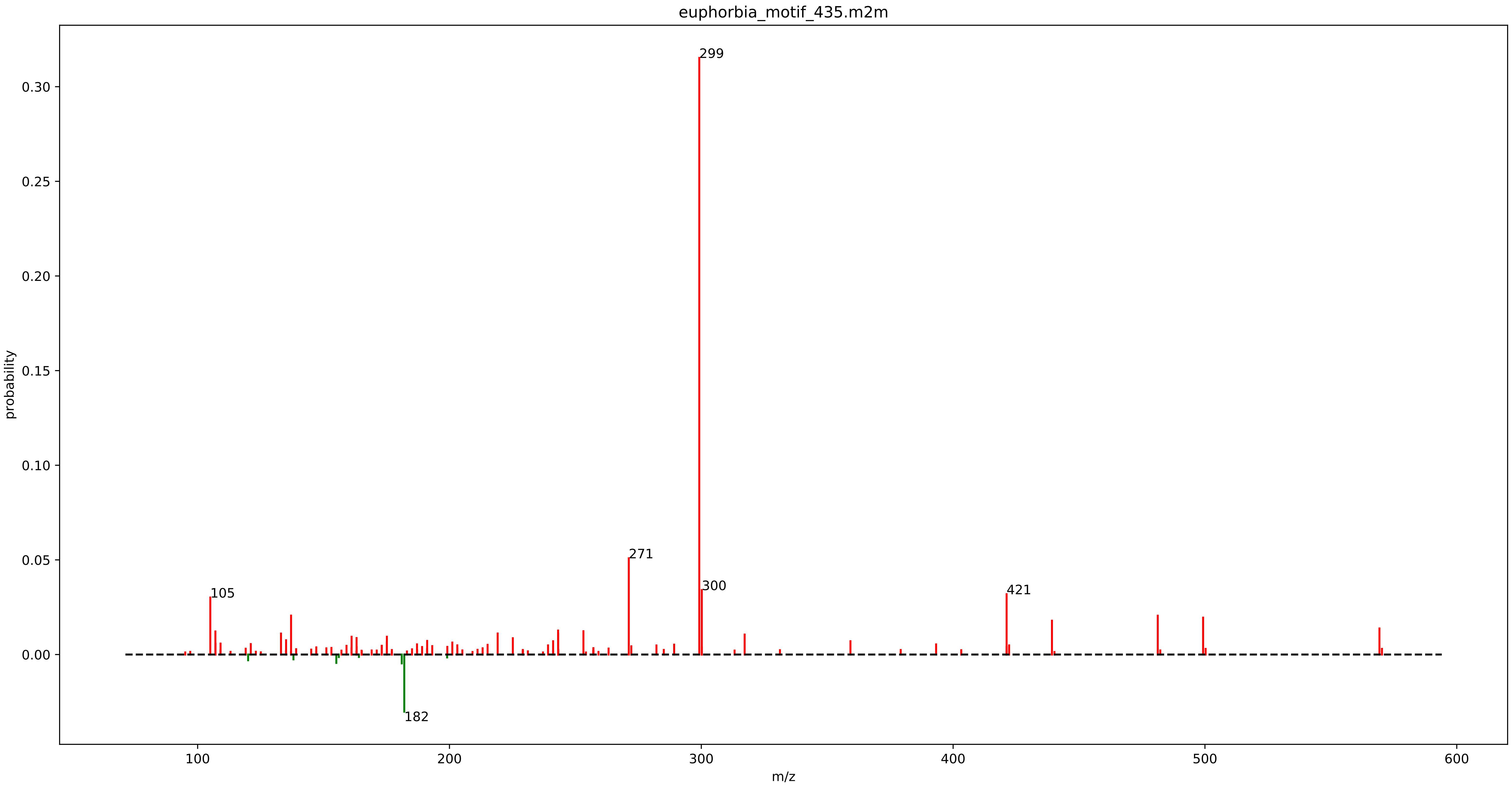
euphorbia_motif_435.m2m
probability
m/z
0.30
0.25
0.20
0.15
0.10
0.05
0.00
100
200
300
400
500
600
299
271
300
105
421
182

euphorbia_motif_435.m2m

fragment_299.2025	: 0.32
fragment_271.2075	: 0.05
fragment_300.2025	: 0.03
fragment_421.2375	: 0.03
loss_182.0525	: 0.03
fragment_105.0325	: 0.03
fragment_137.0975	: 0.02
fragment_481.2575	: 0.02
fragment_499.2725	: 0.02
fragment_439.2475	: 0.02
fragment_569.2925	: 0.01
fragment_243.1375	: 0.01
fragment_253.1925	: 0.01
fragment_107.0475	: 0.01
fragment_219.1375	: 0.01
fragment_133.1025	: 0.01
fragment_243.1725	: 0.01
fragment_317.2125	: 0.01
fragment_175.1475	: 0.01
fragment_161.1325	: 0.01
fragment_163.1125	: 0.01
fragment_225.1625	: 0.01
fragment_135.1175	: 0.01
fragment_191.1425	: 0.01
fragment_359.2225	: 0.01
fragment_241.1575	: 0.01
fragment_175.1125	: 0.01
fragment_201.1275	: 0.01
fragment_109.1025	: 0.01
fragment_107.0875	: 0.01
fragment_121.1025	: 0.01
fragment_187.1125	: 0.01
fragment_393.2425	: 0.01
fragment_289.2175	: 0.01
fragment_215.1425	: 0.01

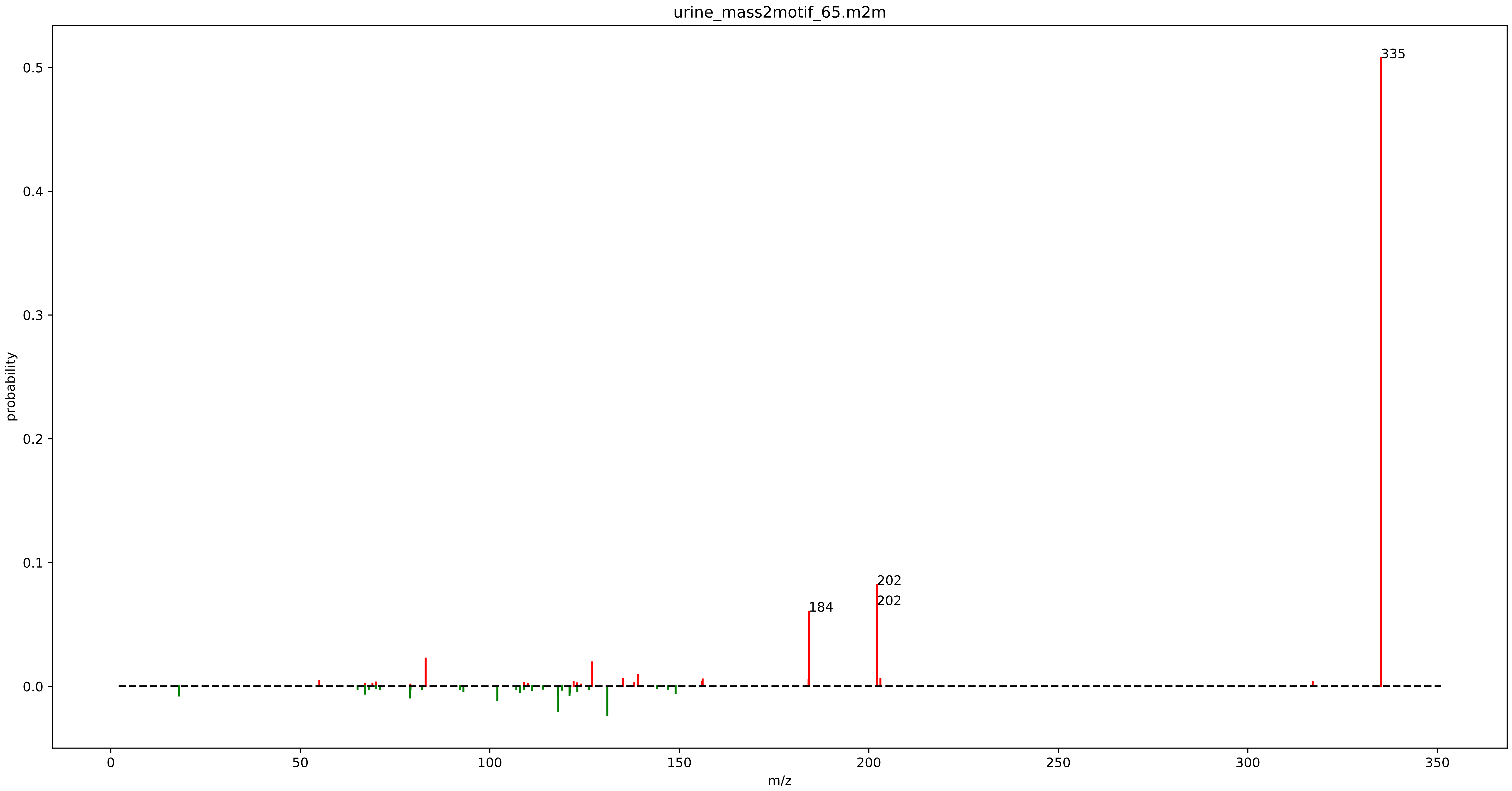

urine_mass2motif_65.m2m
probability
m/z
0.5
0.4
0.3
0.2
0.1
0.0
0
50
100
150
200
250
300
350
335
202
202
184

urine_mass2motif_65.m2m

fragment_335.0975	: 0.51
fragment_202.1425	: 0.08
fragment_202.1075	: 0.07
fragment_184.1325	: 0.06
loss_131.0125	: 0.02
fragment_83.0725	: 0.02
loss_118.0625	: 0.02
fragment_127.0375	: 0.02
loss_101.9975	: 0.01
fragment_139.0375	: 0.01
loss_79.0225	: 0.01
loss_17.9375	: 0.01
loss_117.9875	: 0.01
loss_121.0375	: 0.01
fragment_202.0825	: 0.01
fragment_203.0525	: 0.01
fragment_135.1025	: 0.01
loss_67.0375	: 0.01
loss_118.0325	: 0.01
fragment_156.1375	: 0.01
fragment_184.0975	: 0.01
loss_149.0275	: 0.01

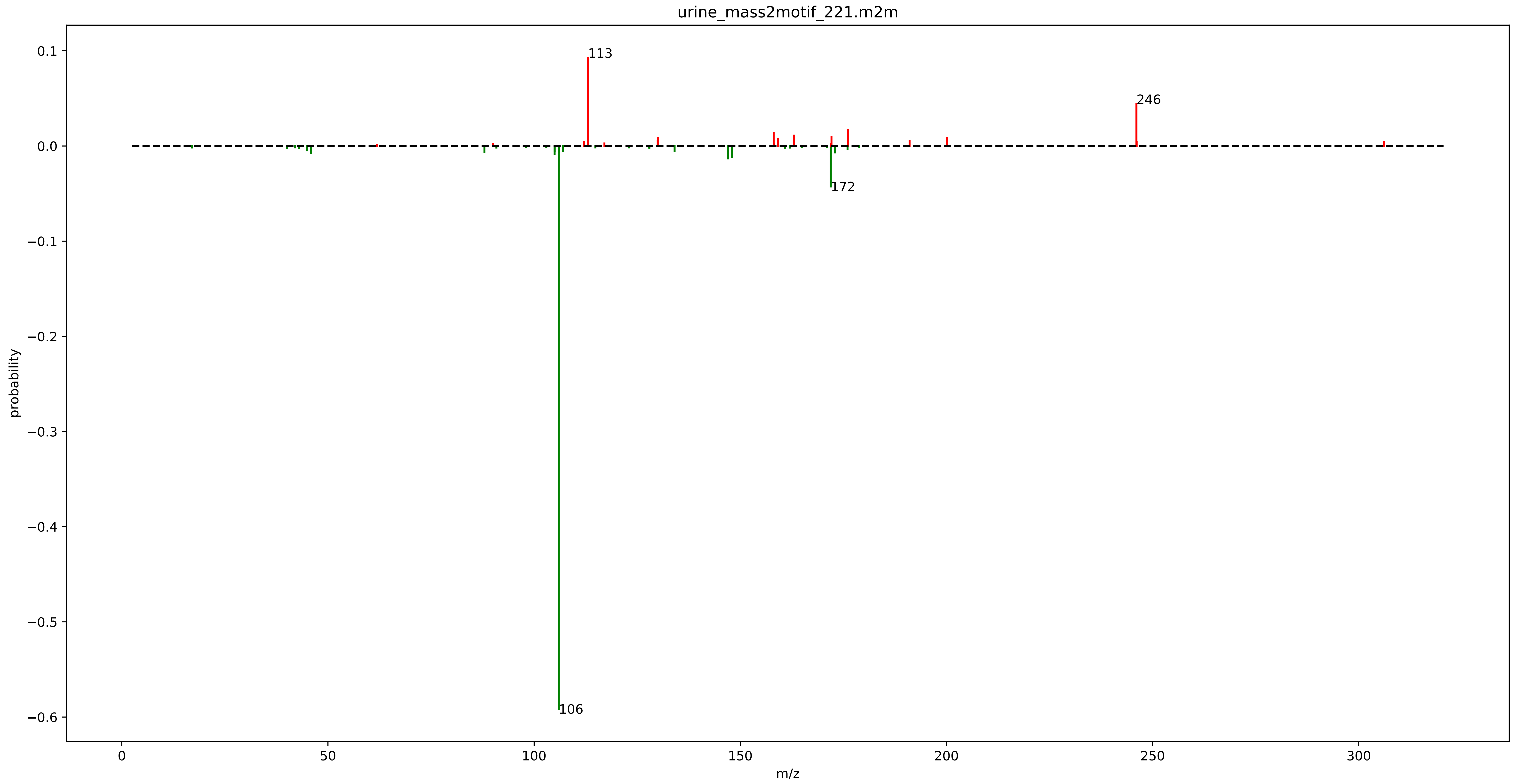

urine_mass2motif_221.m2m
probability
m/z
0.1
0.0
−0.1
−0.2
−0.3
−0.4
−0.5
−0.6
0
50
100
150
200
250
300
113
246
172
106

urine_mass2motif_221.m2m

loss_105.9675	: 0.59
fragment_113.0775	: 0.09
fragment_246.0775	: 0.04
loss_171.9375	: 0.04
fragment_176.1125	: 0.02
fragment_158.1025	: 0.01
loss_146.9725	: 0.01
loss_147.9775	: 0.01
fragment_163.0575	: 0.01
fragment_172.1175	: 0.01
loss_104.9625	: 0.01
fragment_200.1125	: 0.01
fragment_130.1075	: 0.01
fragment_159.0825	: 0.01
loss_45.9225	: 0.01
loss_172.9425	: 0.01
loss_106.0175	: 0.01
loss_87.9525	: 0.01
fragment_191.0525	: 0.01
loss_104.9725	: 0.01
loss_106.9675	: 0.01
loss_134.0625	: 0.01

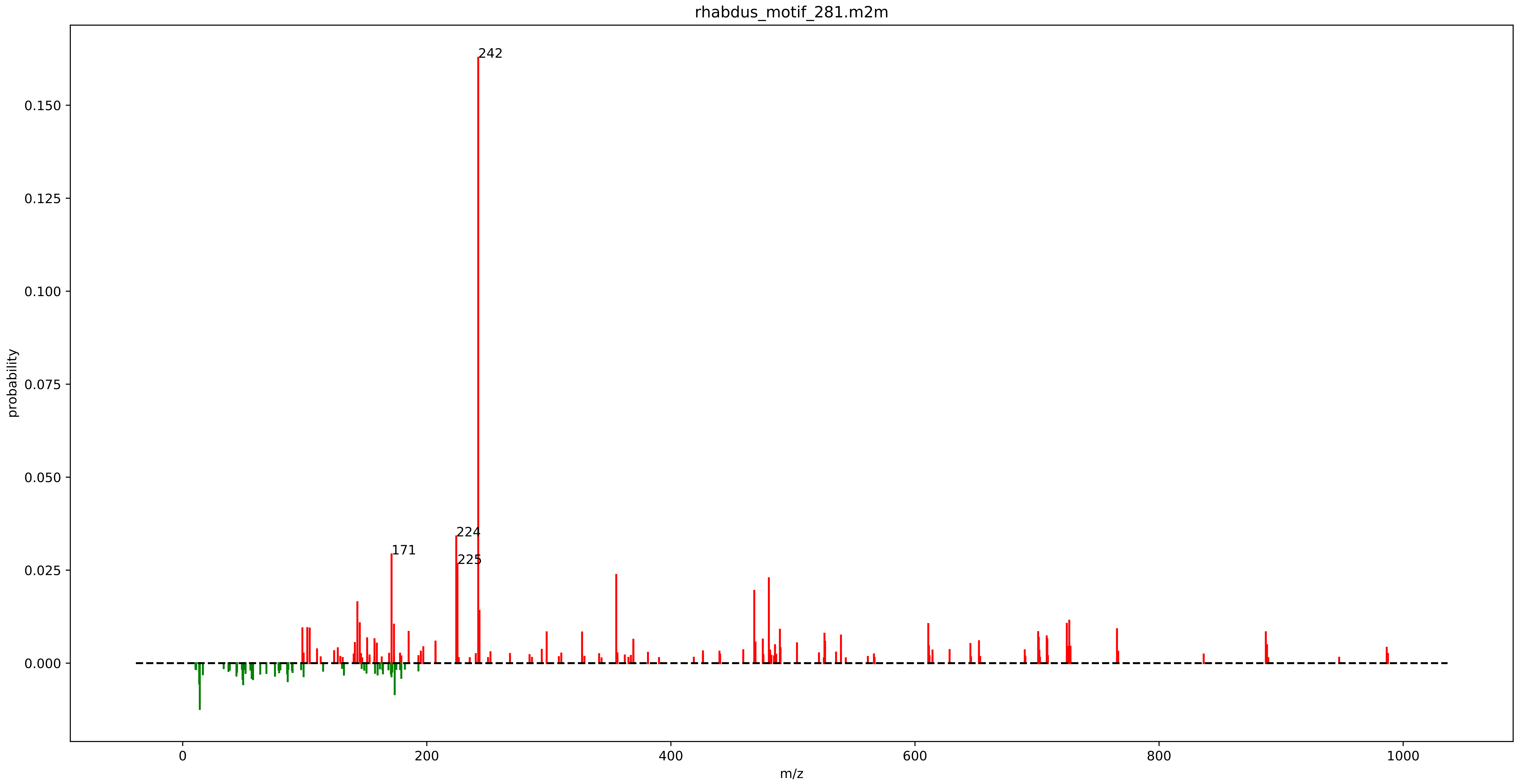

rhabdus_motif_281.m2m
probability
m/z
242
224
225
171
0.150
0.125
0.100
0.075
0.050
0.025
0.000
0
200
400
600
800
1000

rhabdus_motif_281.m2m

fragment_242.1475	: 0.16
fragment_224.1375	: 0.03
fragment_171.1125	: 0.03
fragment_225.1225	: 0.03
fragment_355.2325	: 0.02
fragment_480.2825	: 0.02
fragment_468.3175	: 0.02
fragment_143.1175	: 0.02
fragment_243.1525	: 0.01
loss_13.9975	: 0.01
fragment_726.4275	: 0.01
fragment_145.0975	: 0.01
fragment_724.4025	: 0.01
fragment_610.9025	: 0.01
fragment_173.0975	: 0.01
fragment_102.0675	: 0.01
fragment_98.0575	: 0.01
fragment_104.0675	: 0.01
fragment_765.5225	: 0.01
fragment_489.3225	: 0.01
fragment_185.1275	: 0.01
fragment_700.9275	: 0.01
loss_173.6975	: 0.01
fragment_887.4675	: 0.01
fragment_298.2125	: 0.01
fragment_327.2325	: 0.01
fragment_525.8475	: 0.01
fragment_539.3525	: 0.01
fragment_707.9375	: 0.01
fragment_701.4325	: 0.01
fragment_151.1125	: 0.01
fragment_157.1325	: 0.01
fragment_708.4425	: 0.01
fragment_475.3275	: 0.01
fragment_369.2525	: 0.01
fragment_652.4375	: 0.01
fragment_207.1125	: 0.01
fragment_526.3475	: 0.01
loss_49.5325	: 0.01
fragment_469.3225	: 0.01
loss_13.4975	: 0.01
fragment_141.0675	: 0.01
fragment_503.3225	: 0.01
fragment_159.0775	: 0.01
fragment_645.4125	: 0.01
fragment_369.2475	: 0.01

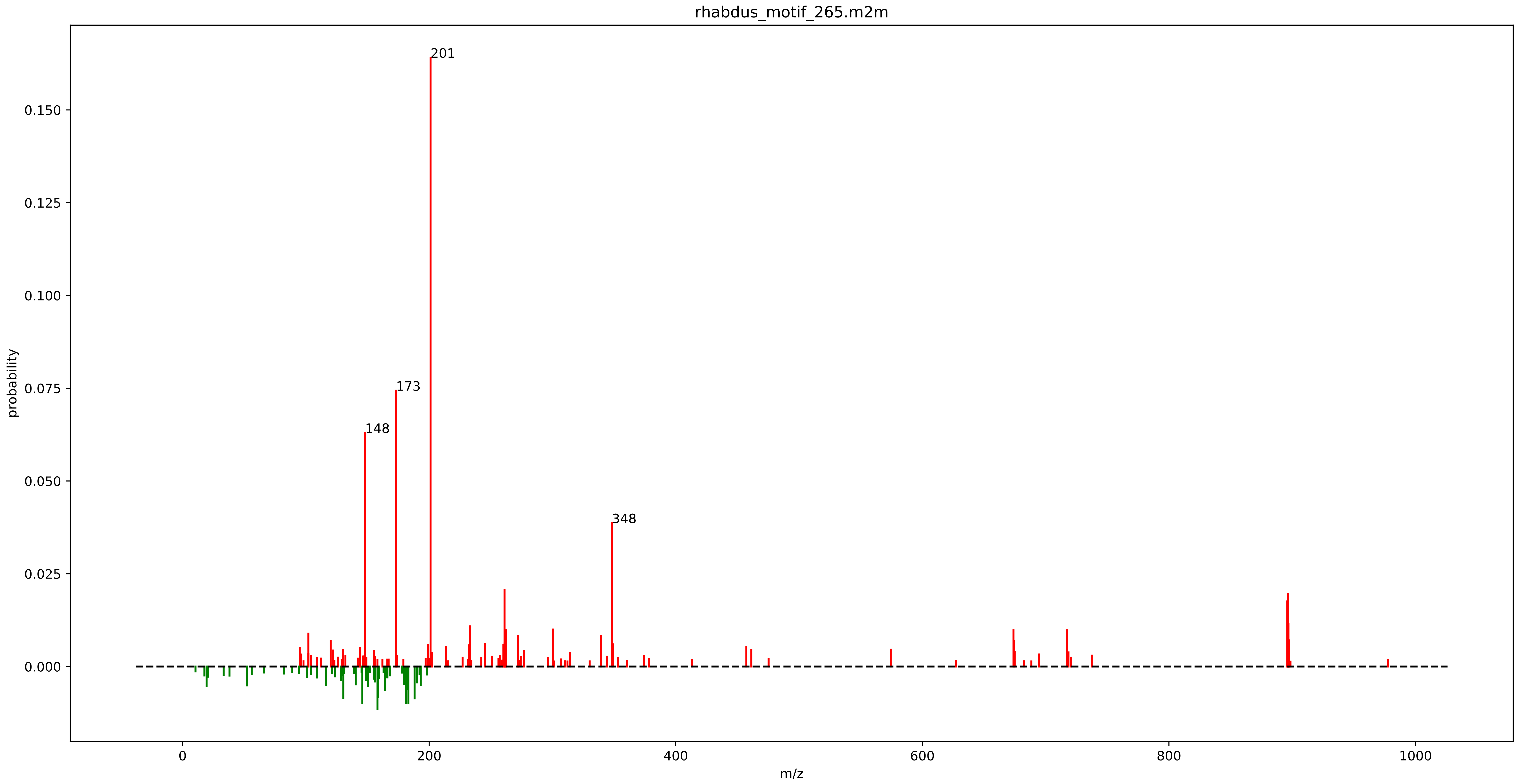

rhabdus_motif_265.m2m
201
173
148
348
probability
0.150
0.125
0.100
0.075
0.050
0.025
0.000
0
200
400
600
800
1000
m/z

rhabdus_motif_265.m2m

fragment_201.1225	: 0.16
fragment_173.1275	: 0.07
fragment_148.0575	: 0.06
fragment_348.1775	: 0.04
fragment_261.1425	: 0.02
fragment_896.5425	: 0.02
fragment_895.9475	: 0.02
fragment_896.9525	: 0.01
loss_158.0975	: 0.01
fragment_233.1625	: 0.01
fragment_300.1725	: 0.01
fragment_896.4525	: 0.01
fragment_896.4475	: 0.01
fragment_673.8825	: 0.01
fragment_262.0975	: 0.01
loss_181.0675	: 0.01
loss_145.8125	: 0.01
loss_183.1525	: 0.01
fragment_717.4875	: 0.01
fragment_102.0575	: 0.01
loss_188.1775	: 0.01
loss_130.3775	: 0.01
fragment_272.1775	: 0.01
fragment_339.2275	: 0.01
loss_158.6025	: 0.01
fragment_897.5475	: 0.01
fragment_120.0775	: 0.01
fragment_674.3875	: 0.01
loss_164.3625	: 0.01
loss_164.0725	: 0.01
fragment_261.1575	: 0.01
fragment_245.1775	: 0.01
loss_182.1725	: 0.01
fragment_349.1775	: 0.01
fragment_260.1675	: 0.01
fragment_199.1525	: 0.01
fragment_232.1375	: 0.01
fragment_457.2825	: 0.01
loss_150.3725	: 0.01
fragment_213.6675	: 0.01
loss_19.4875	: 0.01
loss_52.0575	: 0.01
fragment_261.1475	: 0.01
loss_193.1575	: 0.01
fragment_95.0025	: 0.01

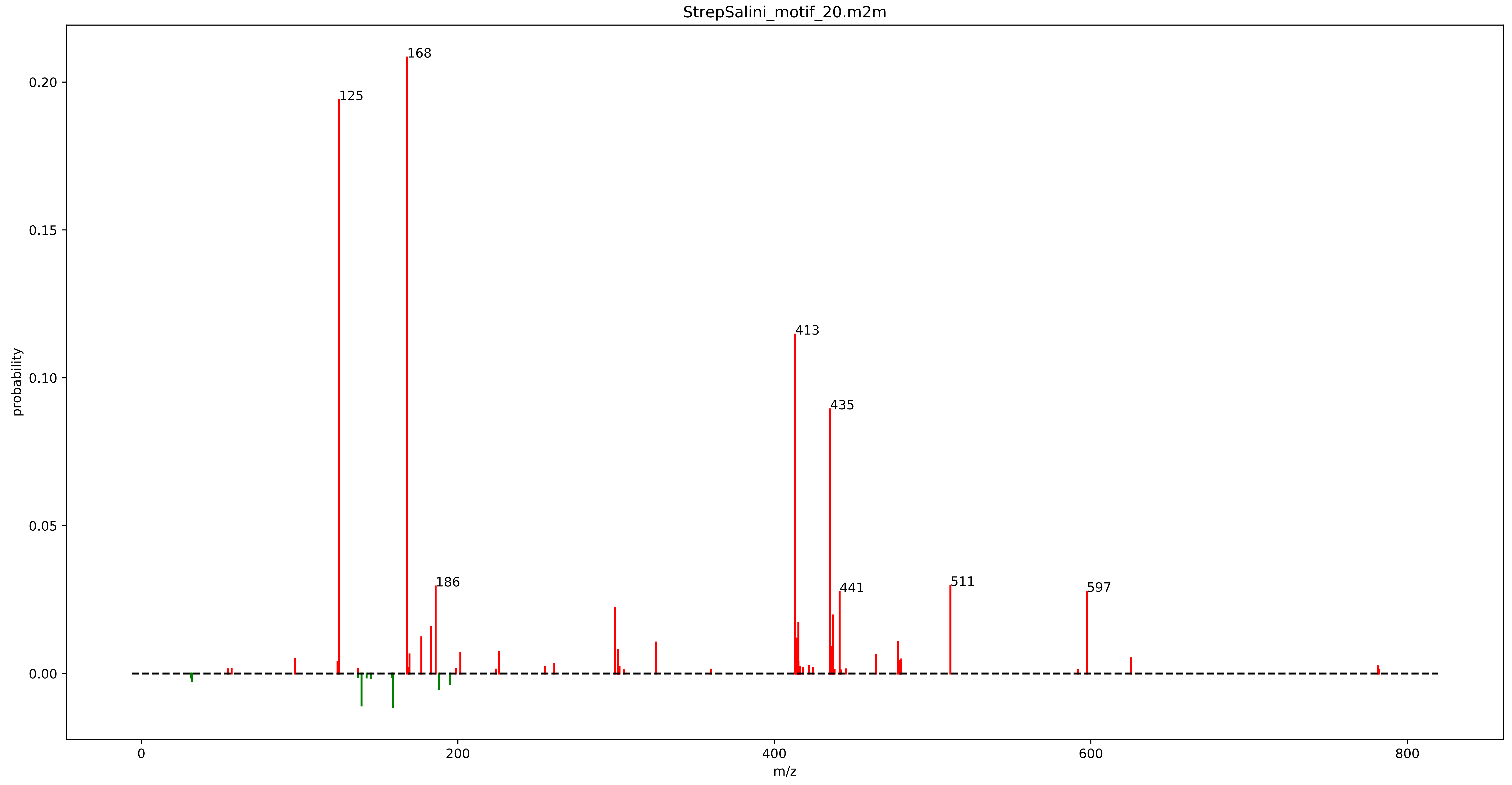

StrepSalini_motif_20.m2m
125
168
186
413
435
441
511
597
probability
m/z
0.00
0.05
0.10
0.15
0.20
0
200
400
600
800

StrepSalini_motif_20.m2m

fragment_167.9500	: 0.21
fragment_124.9500	: 0.19
fragment_413.1500	: 0.11
fragment_435.1500	: 0.09
fragment_511.2500	: 0.03
fragment_185.9500	: 0.03
fragment_597.4500	: 0.03
fragment_441.2500	: 0.03
fragment_299.1500	: 0.02
fragment_437.1500	: 0.02
fragment_415.1500	: 0.02
fragment_182.9500	: 0.02
fragment_176.9500	: 0.01
fragment_414.1500	: 0.01
loss_158.9500	: 0.01
loss_139.1525	: 0.01
fragment_478.2500	: 0.01
fragment_325.2525	: 0.01
fragment_436.1500	: 0.01
fragment_301.1500	: 0.01
fragment_225.9500	: 0.01
fragment_201.5500	: 0.01
fragment_169.4500	: 0.01
fragment_464.1500	: 0.01
loss_188.1500	: 0.01
fragment_625.3500	: 0.01
fragment_97.0500	: 0.01

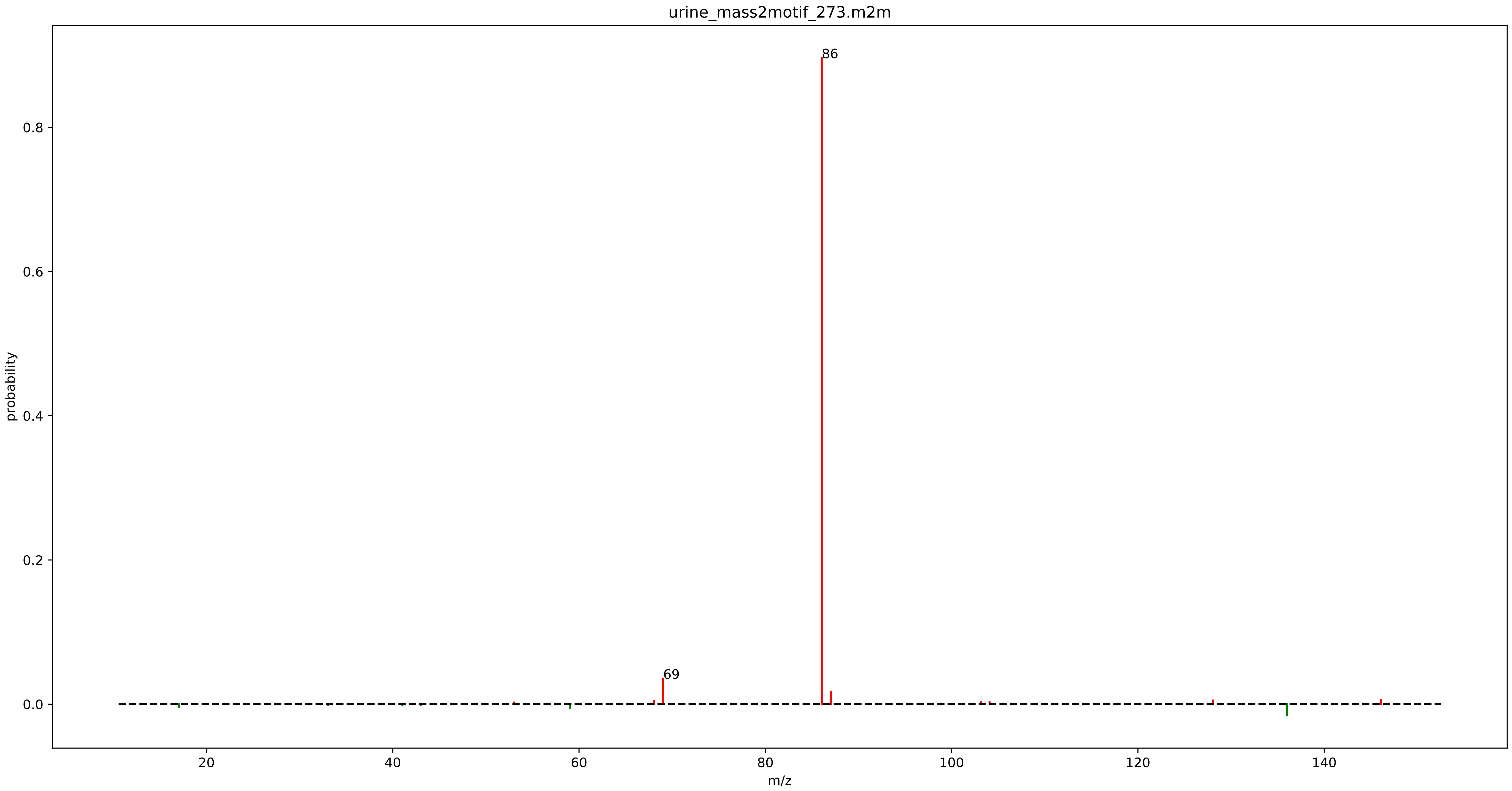

urine_mass2motif_273.m2m
probability
m/z
86
69
0.0
0.2
0.4
0.6
0.8
20
40
60
80
100
120
140

urine_mass2motif_273.m2m

fragment_86.0575	: 0.90
fragment_69.0325	: 0.04
fragment_87.0425	: 0.02
loss_136.0175	: 0.02
loss_59.0475	: 0.01
fragment_146.0825	: 0.01
fragment_128.0725	: 0.01

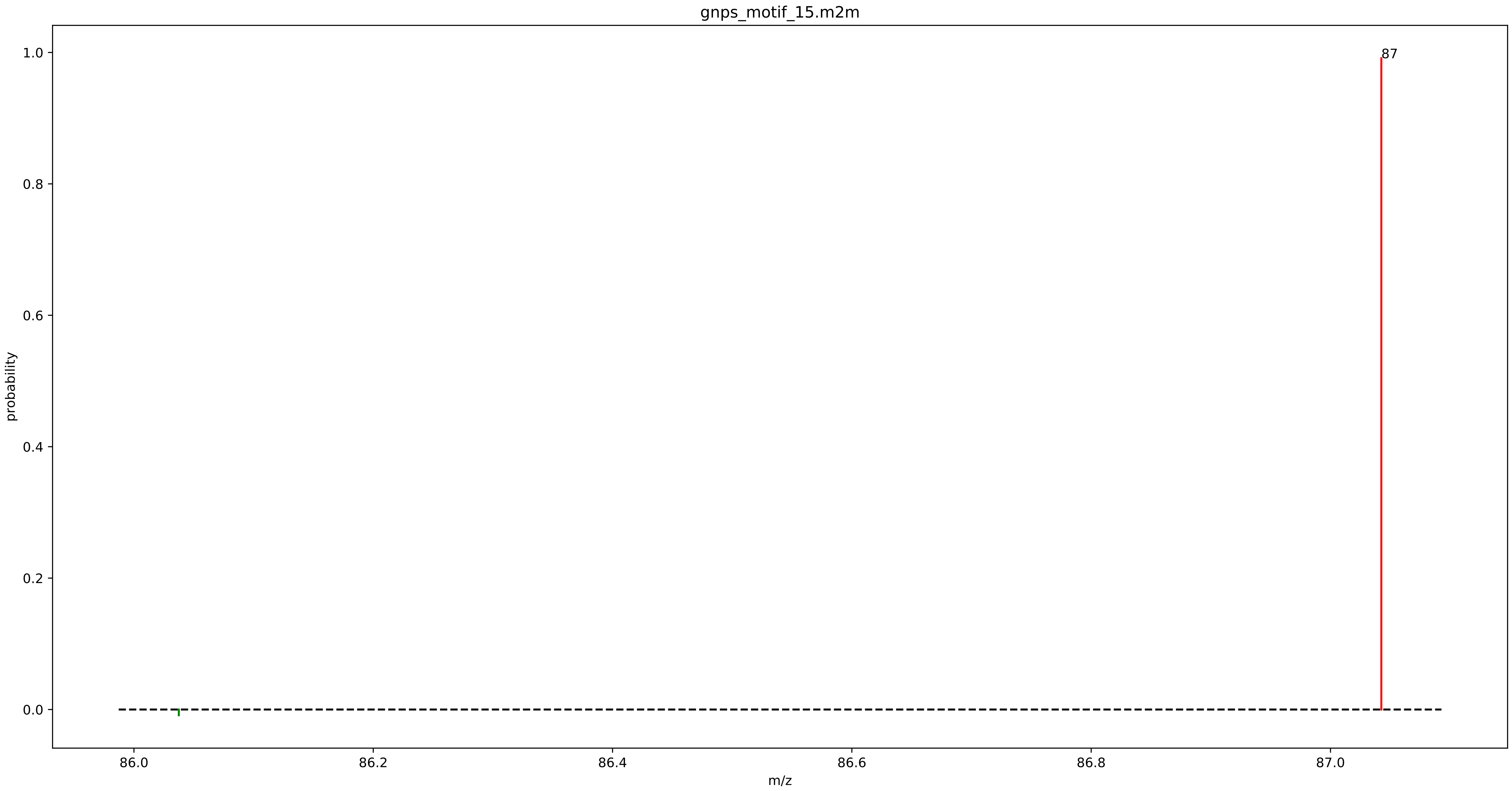

gnps_motif_15.m2m
probability
m/z
87
1.0
0.8
0.6
0.4
0.2
0.0
86.0
86.2
86.4
86.6
86.8
87.0

gnps_motif_15.m2m

fragment_87.0425	: 0.99
loss_86.0375	: 0.01

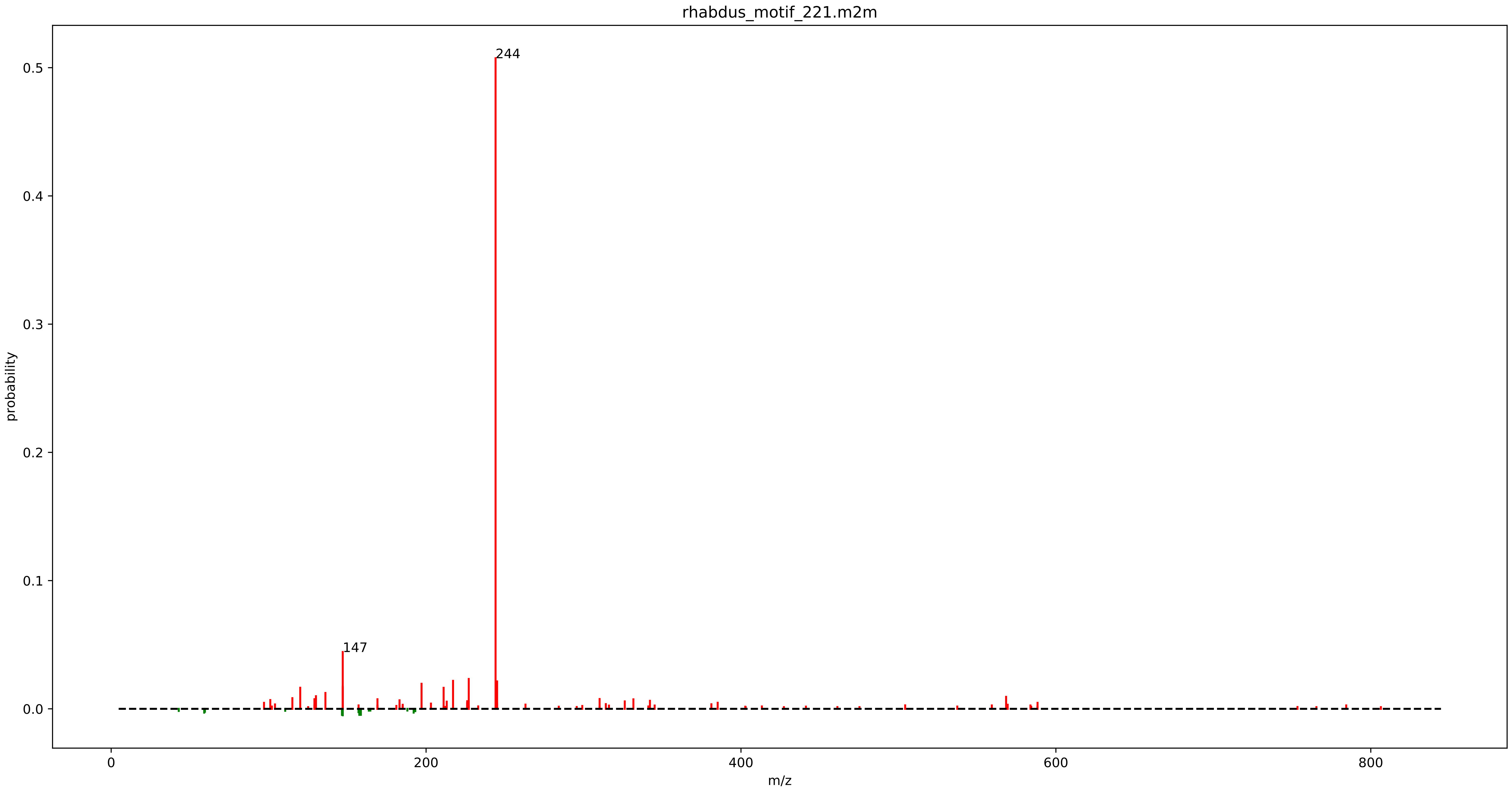

rhabdus_motif_221.m2m
probability
m/z
244
147
0.5
0.4
0.3
0.2
0.1
0.0
0
200
400
600
800

rhabdus_motif_221.m2m

fragment_244.1325	: 0.51
fragment_147.0775	: 0.04
fragment_227.1025	: 0.02
fragment_217.1275	: 0.02
fragment_245.1325	: 0.02
fragment_197.1275	: 0.02
fragment_147.0725	: 0.02
fragment_120.0775	: 0.02
fragment_211.1425	: 0.02
fragment_147.0825	: 0.02
fragment_136.0725	: 0.01
fragment_130.0575	: 0.01
fragment_568.3825	: 0.01
fragment_115.0825	: 0.01
fragment_130.0525	: 0.01
fragment_310.2125	: 0.01
fragment_129.1025	: 0.01
fragment_169.1275	: 0.01
fragment_331.6725	: 0.01
fragment_101.0675	: 0.01
fragment_183.1425	: 0.01
fragment_342.1825	: 0.01
fragment_226.1275	: 0.01
fragment_326.2025	: 0.01
fragment_213.1175	: 0.01
loss_147.0975	: 0.01

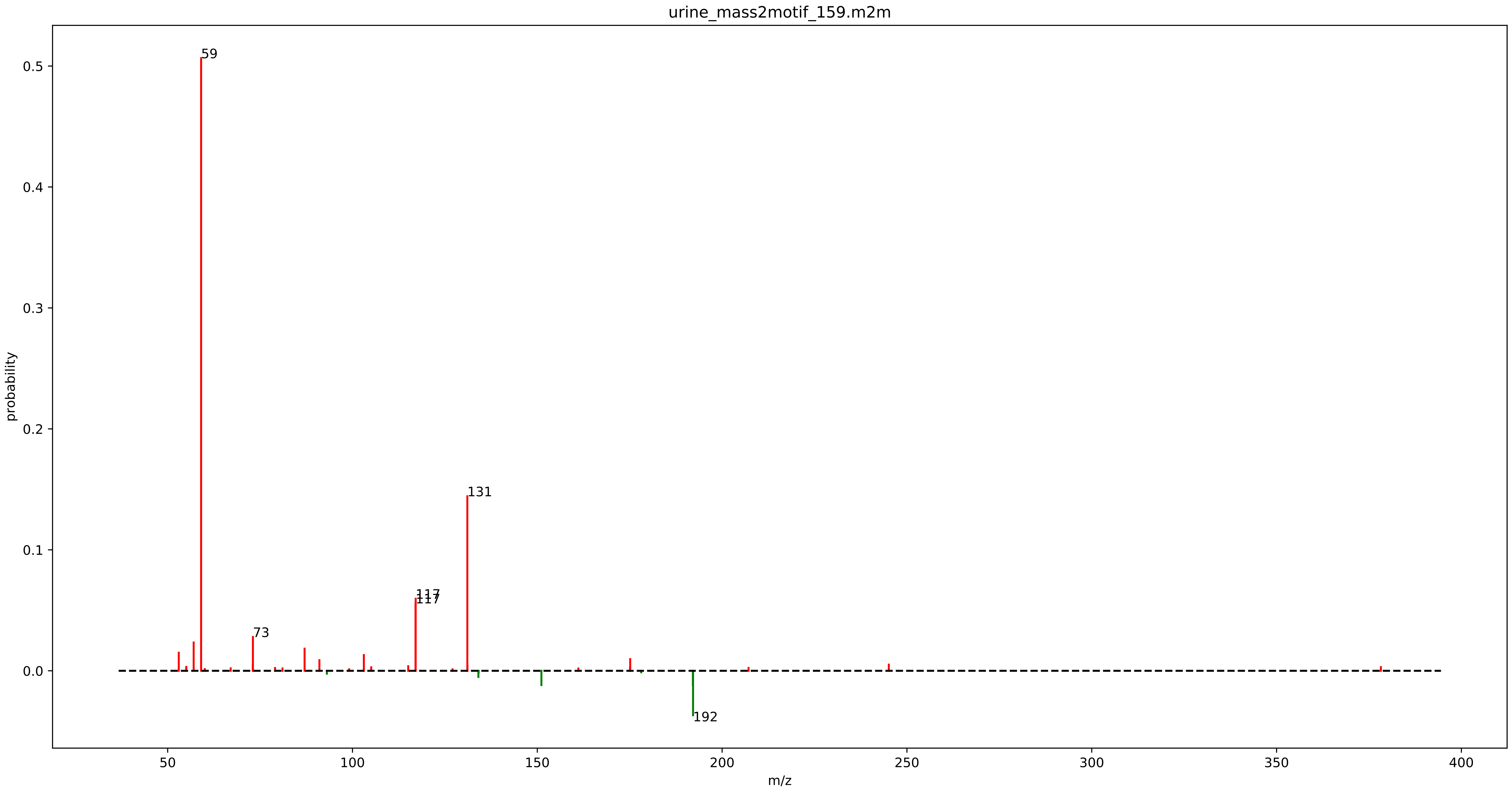
urine_mass2motif_159.m2m
probability
m/z
0.5
0.4
0.3
0.2
0.1
0.0
50
100
150
200
250
300
350
400
59
73
117
117
131
192

urine_mass2motif_159.m2m

fragment_59.0475	: 0.51
fragment_131.0725	: 0.14
fragment_117.0925	: 0.06
fragment_117.0575	: 0.06
loss_192.1375	: 0.04
fragment_73.0625	: 0.03
fragment_57.0325	: 0.02
fragment_87.0425	: 0.02
fragment_53.0025	: 0.01
fragment_103.0775	: 0.01
loss_192.0925	: 0.01
loss_151.1125	: 0.01
fragment_175.1325	: 0.01
fragment_91.0525	: 0.01
fragment_245.0975	: 0.01
loss_134.0625	: 0.01

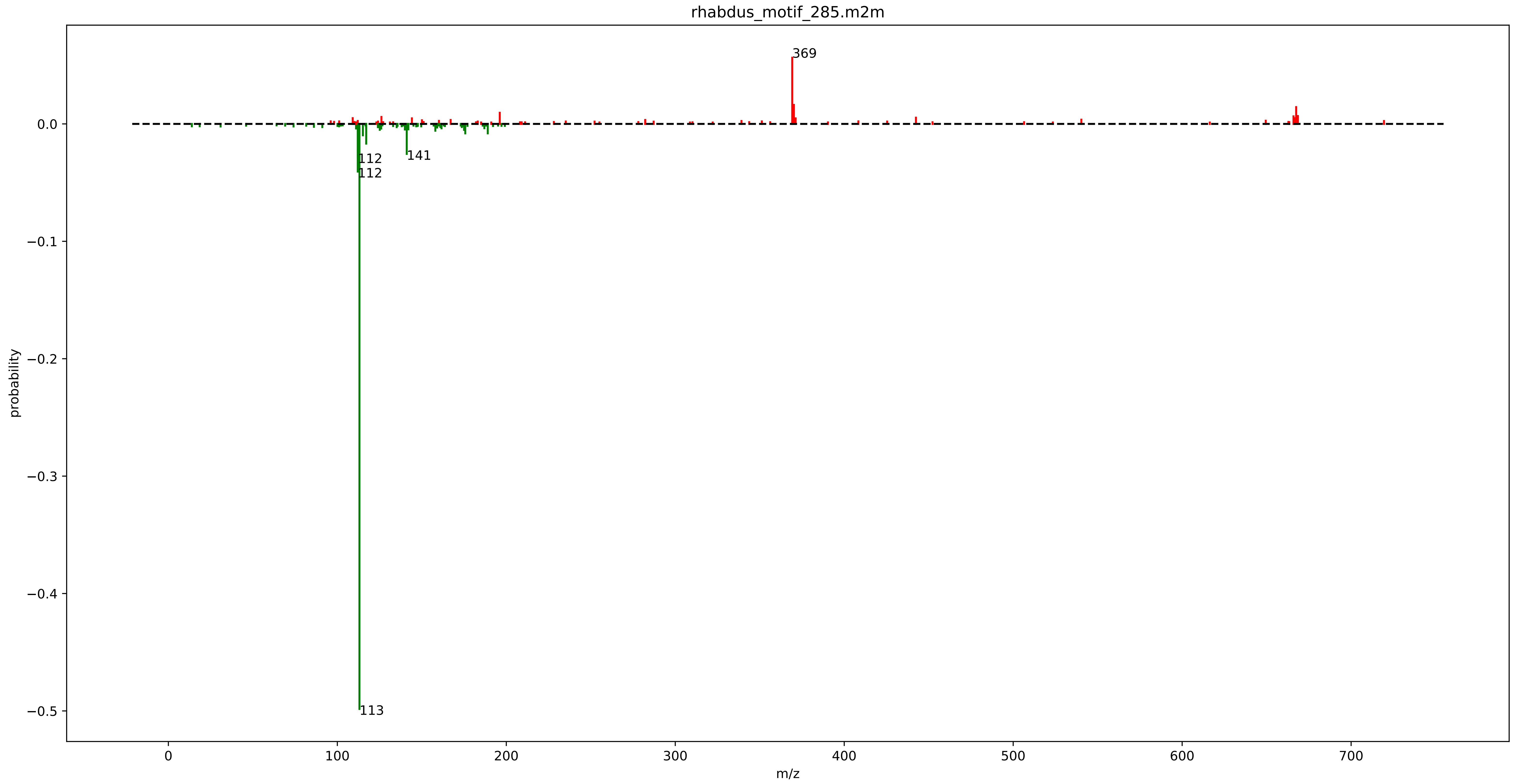
rhabdus_motif_285.m2m
369
112
112
141
113
probability
m/z
0.0
−0.1
−0.2
−0.3
−0.4
−0.5
0
100
200
300
400
500
600
700

rhabdus_motif_285.m2m

loss_113.0825	: 0.50
fragment_369.2125	: 0.06
loss_112.0775	: 0.04
loss_112.0825	: 0.03
loss_141.0775	: 0.03
loss_117.0775	: 0.02
fragment_370.2175	: 0.02
fragment_667.4775	: 0.01
loss_115.1175	: 0.01
fragment_196.1325	: 0.01
loss_189.0225	: 0.01
loss_175.7125	: 0.01
fragment_668.4825	: 0.01
fragment_665.8925	: 0.01
fragment_126.0975	: 0.01
loss_158.0025	: 0.01
fragment_442.4375	: 0.01
loss_175.0075	: 0.01
loss_125.1025	: 0.01

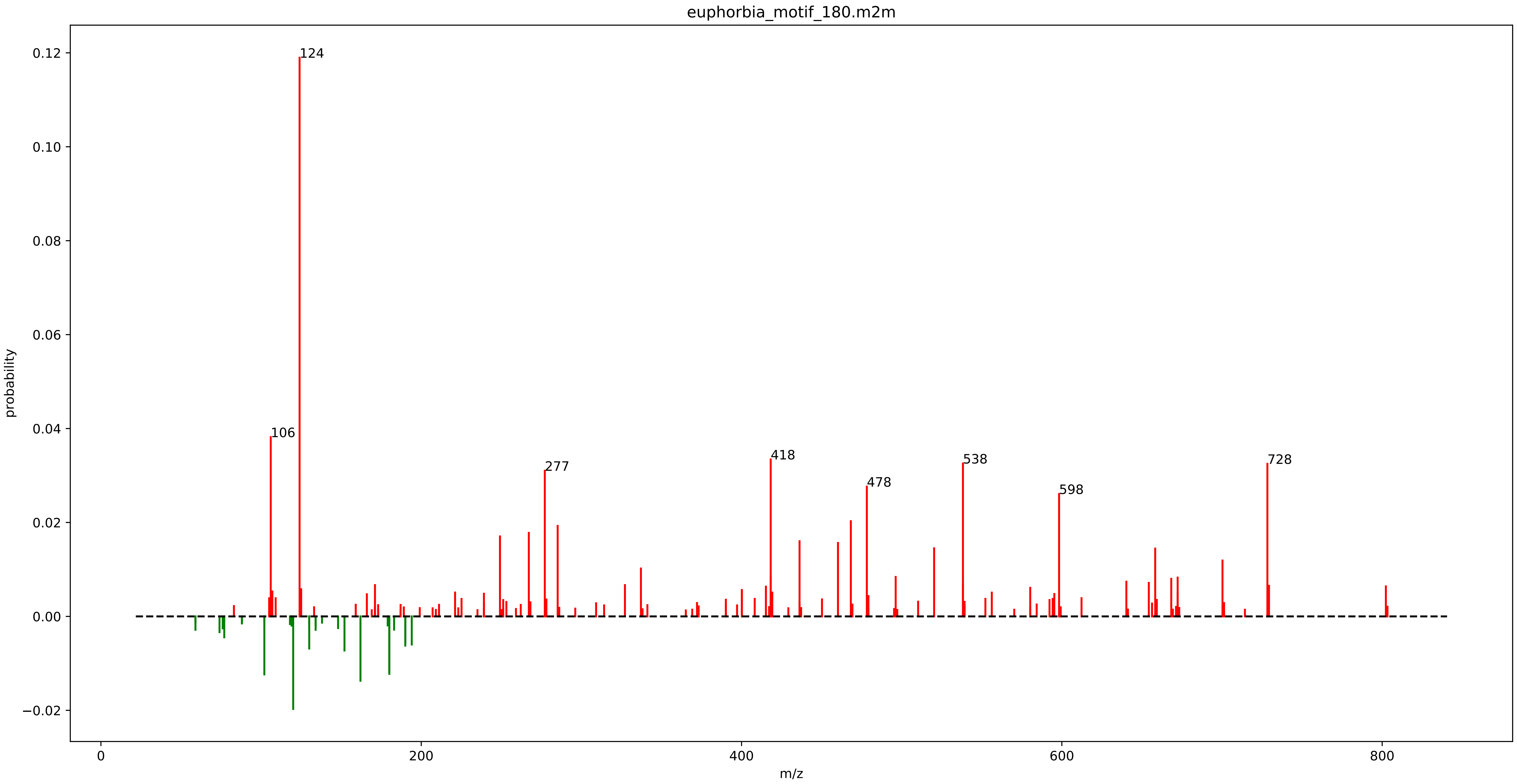

euphorbia_motif_180.m2m
probability
m/z
0.12
0.10
0.08
0.06
0.04
0.02
0.00
−0.02
0
200
400
600
800
124
106
277
418
478
538
598
728

euphorbia_motif_180.m2m

fragment_124.0375	: 0.12
fragment_106.0275	: 0.04
fragment_418.1975	: 0.03
fragment_538.2425	: 0.03
fragment_728.3325	: 0.03
fragment_277.1575	: 0.03
fragment_478.2225	: 0.03
fragment_598.2625	: 0.03
fragment_468.2375	: 0.02
loss_120.0425	: 0.02
fragment_285.1825	: 0.02
fragment_267.1725	: 0.02
fragment_249.1625	: 0.02
fragment_436.2075	: 0.02
fragment_460.2125	: 0.02
fragment_520.2325	: 0.01
fragment_658.2825	: 0.01
loss_162.0525	: 0.01
loss_102.0325	: 0.01
loss_180.0675	: 0.01
fragment_700.2925	: 0.01
loss_180.0625	: 0.01
fragment_478.2175	: 0.01
fragment_337.1775	: 0.01
fragment_468.2325	: 0.01
fragment_418.2025	: 0.01
fragment_496.2325	: 0.01
fragment_672.2975	: 0.01
fragment_668.3075	: 0.01
fragment_640.2725	: 0.01
loss_152.0675	: 0.01
fragment_654.2875	: 0.01
loss_130.0675	: 0.01
fragment_538.2375	: 0.01
fragment_327.1925	: 0.01
fragment_171.1175	: 0.01
fragment_729.3325	: 0.01
fragment_802.3075	: 0.01
fragment_415.2075	: 0.01
loss_190.0475	: 0.01
fragment_580.2525	: 0.01
loss_194.0775	: 0.01
fragment_125.0425	: 0.01
fragment_400.1925	: 0.01
loss_190.0825	: 0.01
fragment_400.1875	: 0.01
fragment_107.0475	: 0.01
fragment_221.1325	: 0.01
fragment_419.2025	: 0.01
fragment_556.2525	: 0.01

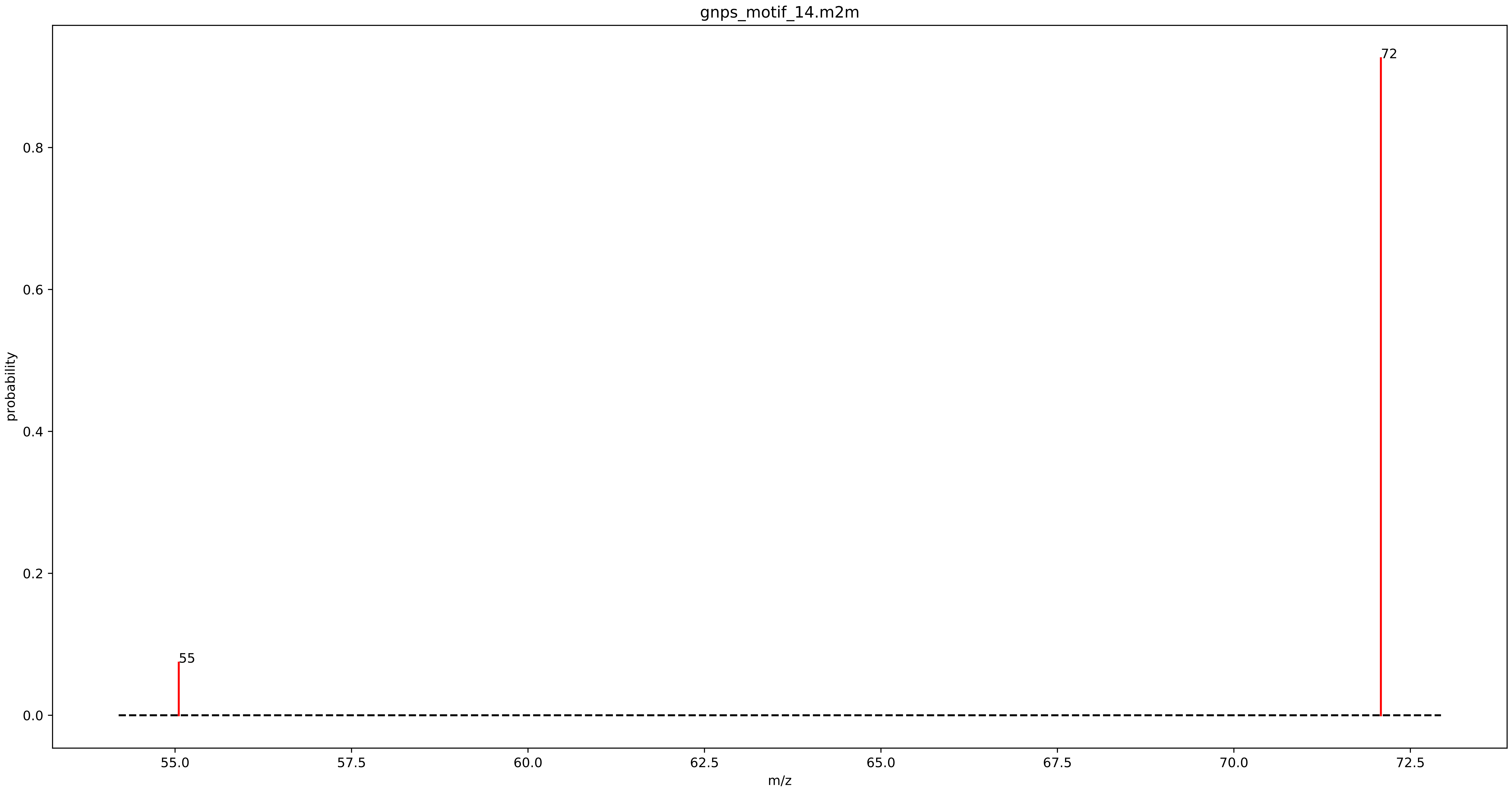
gnps_motif_14.m2m
probability
m/z
72
55
0.0
0.2
0.4
0.6
0.8
55.0
57.5
60.0
62.5
65.0
67.5
70.0
72.5

gnps_motif_14.m2m

fragment_72.0825	: 0.93
fragment_55.0525	: 0.07

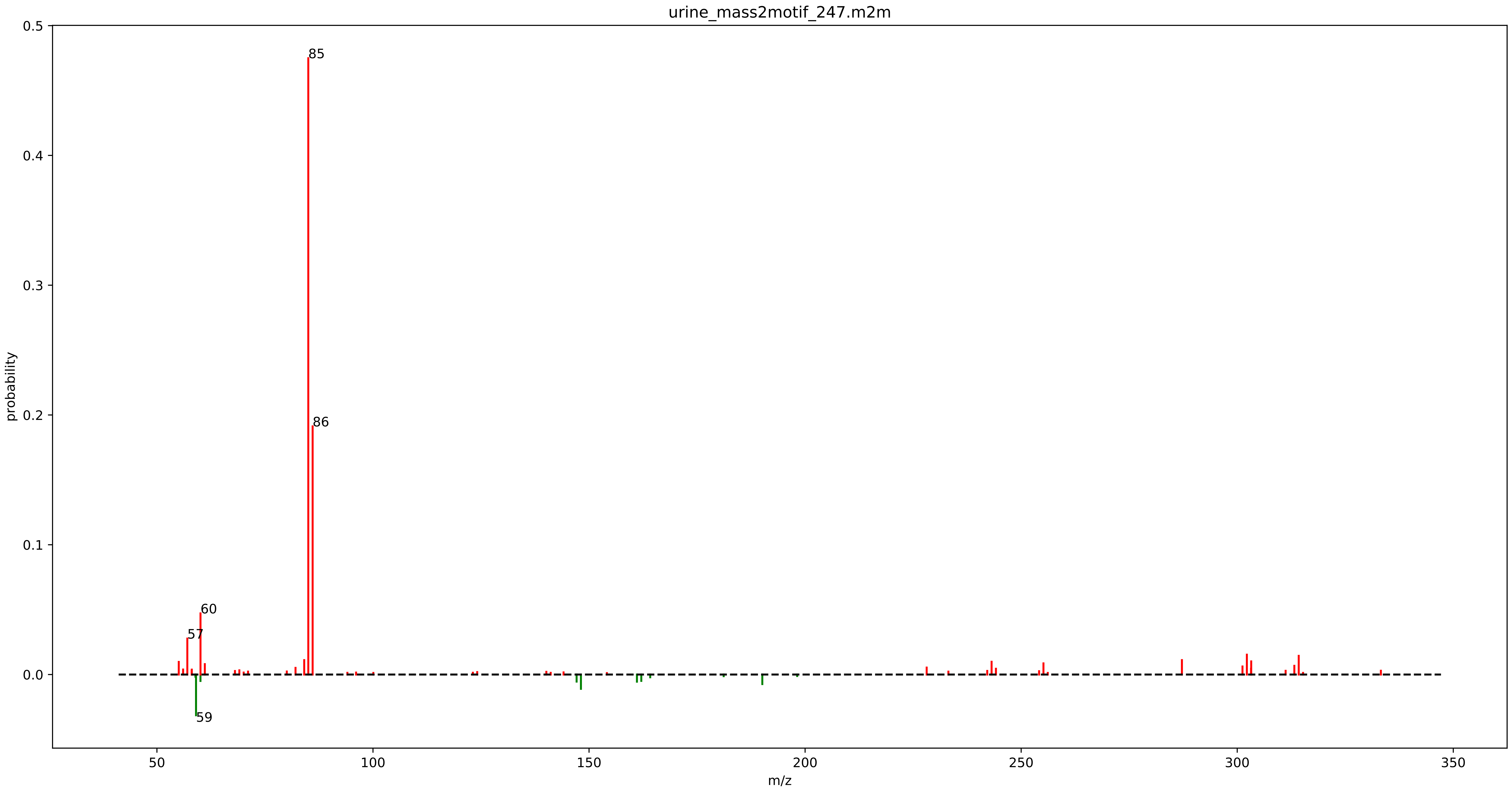

urine_mass2motif_247.m2m
probability
m/z
0.5
0.4
0.3
0.2
0.1
0.0
50
100
150
200
250
300
350
85
86
60
57
59

urine_mass2motif_247.m2m

fragment_85.0275	: 0.47
fragment_86.0325	: 0.19
fragment_60.0825	: 0.05
loss_59.0475	: 0.03
fragment_57.0325	: 0.03
fragment_302.2325	: 0.02
fragment_314.2325	: 0.01
fragment_287.2025	: 0.01
fragment_84.0825	: 0.01
loss_148.1225	: 0.01
fragment_303.2375	: 0.01
fragment_243.1575	: 0.01
fragment_55.0525	: 0.01
fragment_255.1575	: 0.01
fragment_61.0825	: 0.01
loss_190.0975	: 0.01
fragment_313.2175	: 0.01
fragment_301.2175	: 0.01
loss_161.0825	: 0.01
loss_147.1225	: 0.01
fragment_228.1325	: 0.01
fragment_82.0725	: 0.01

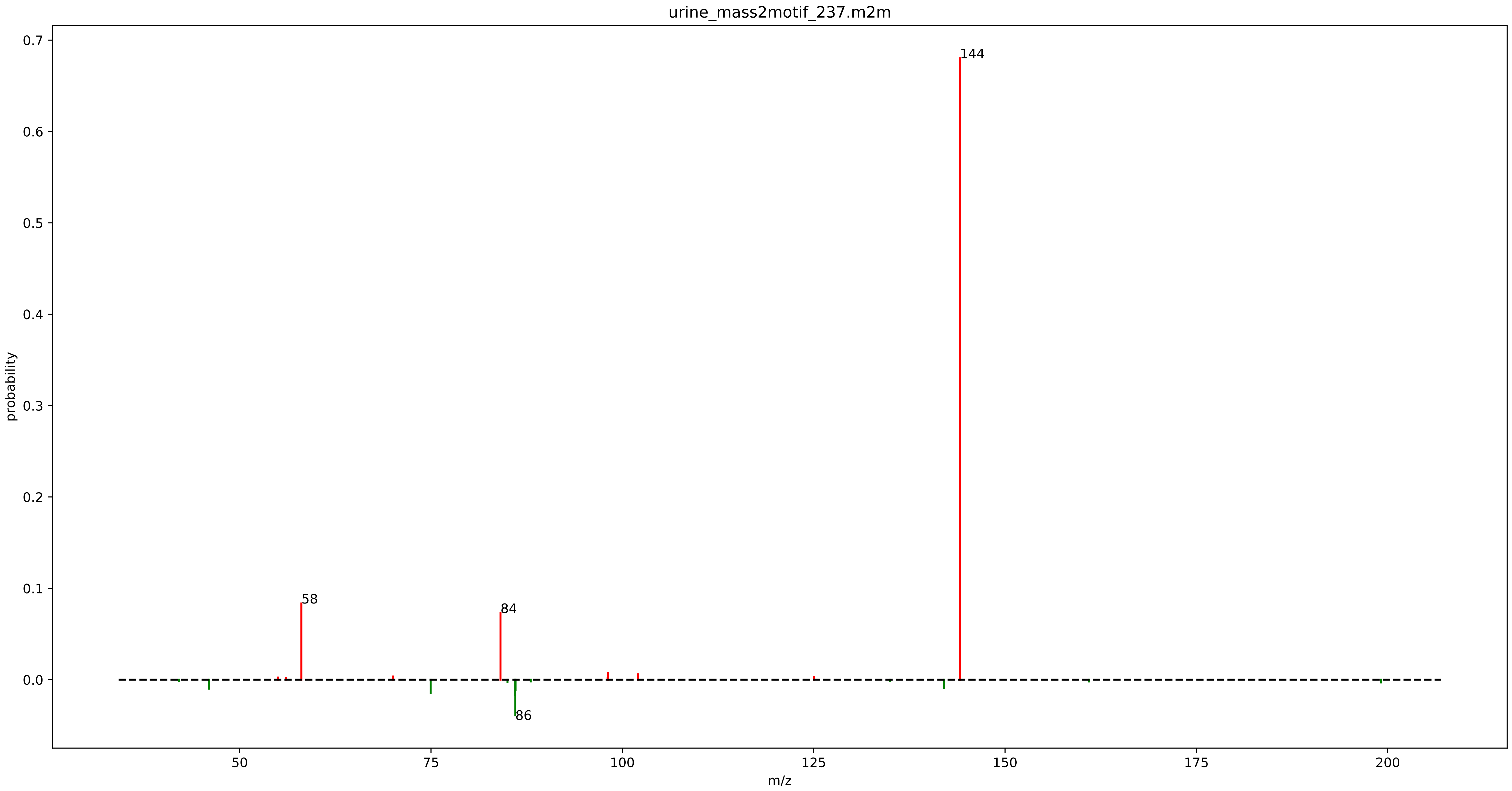

urine_mass2motif_237.m2m
probability
m/z
0.7
0.6
0.5
0.4
0.3
0.2
0.1
0.0
50
75
100
125
150
175
200
144
58
84
86

urine_mass2motif_237.m2m

fragment_144.1025	: 0.68
fragment_58.0675	: 0.08
fragment_84.0825	: 0.07
loss_86.0175	: 0.04
fragment_144.0825	: 0.02
loss_86.0025	: 0.02
loss_74.9475	: 0.01
loss_86.0475	: 0.01
loss_45.9675	: 0.01
loss_142.0275	: 0.01
fragment_98.0975	: 0.01
fragment_102.0525	: 0.01
fragment_144.1225	: 0.01

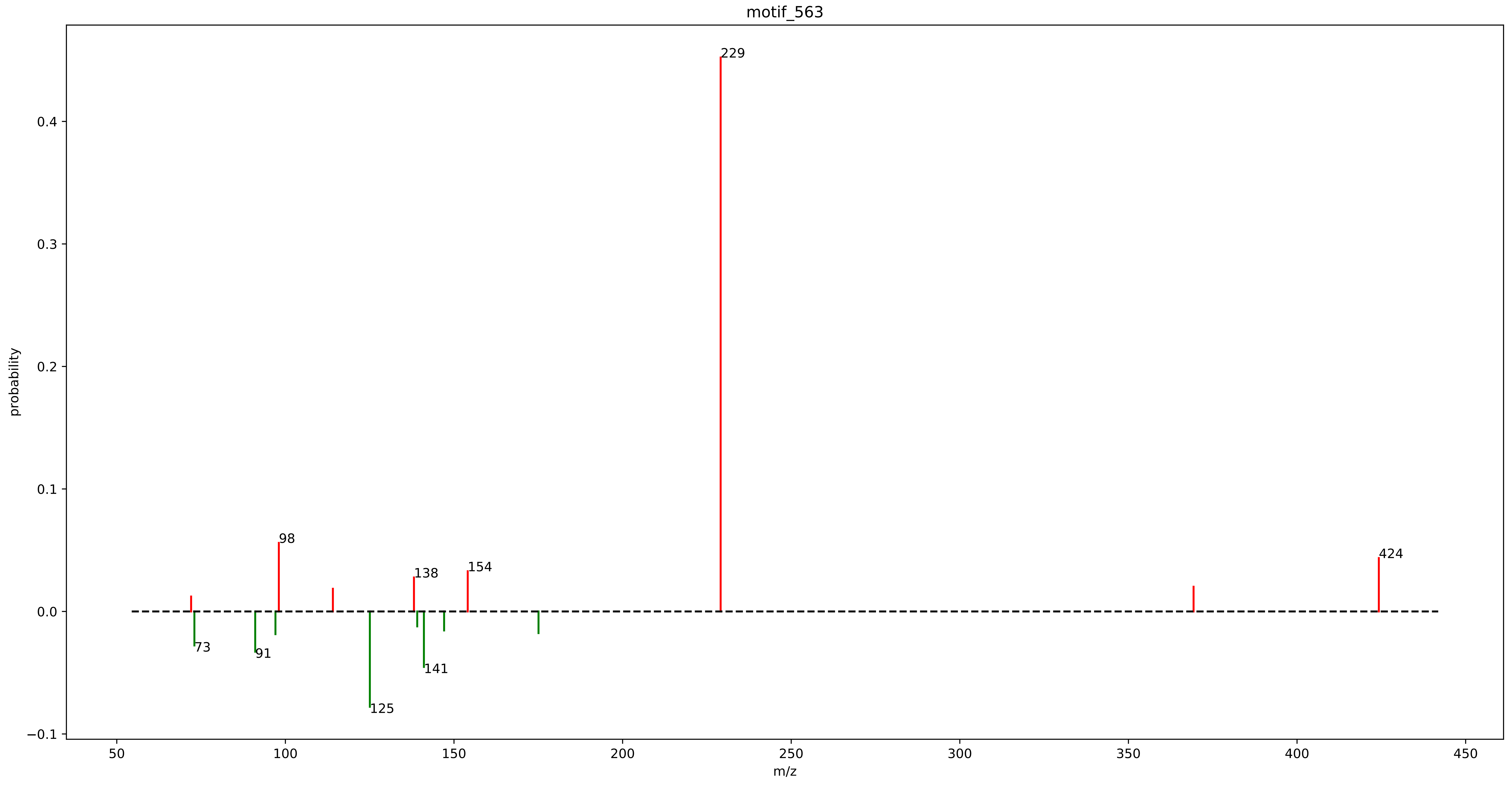

motif_563
229
98
138
154
424
73
91
141
125
0.4
0.3
0.2
0.1
0.0
−0.1
probability
50
100
150
200
250
300
350
400
450
m/z

motif_563

fragment_229.0725	: 0.45
loss_125.0475	: 0.08
fragment_98.0575	: 0.06
loss_141.0775	: 0.05
fragment_424.2575	: 0.04
fragment_154.0725	: 0.03
loss_91.0525	: 0.03
fragment_138.1275	: 0.03
loss_73.0175	: 0.03
fragment_369.3175	: 0.02
fragment_114.0875	: 0.02
loss_97.0525	: 0.02
loss_175.0625	: 0.02
loss_147.0675	: 0.02
loss_139.0975	: 0.01
fragment_72.0425	: 0.01

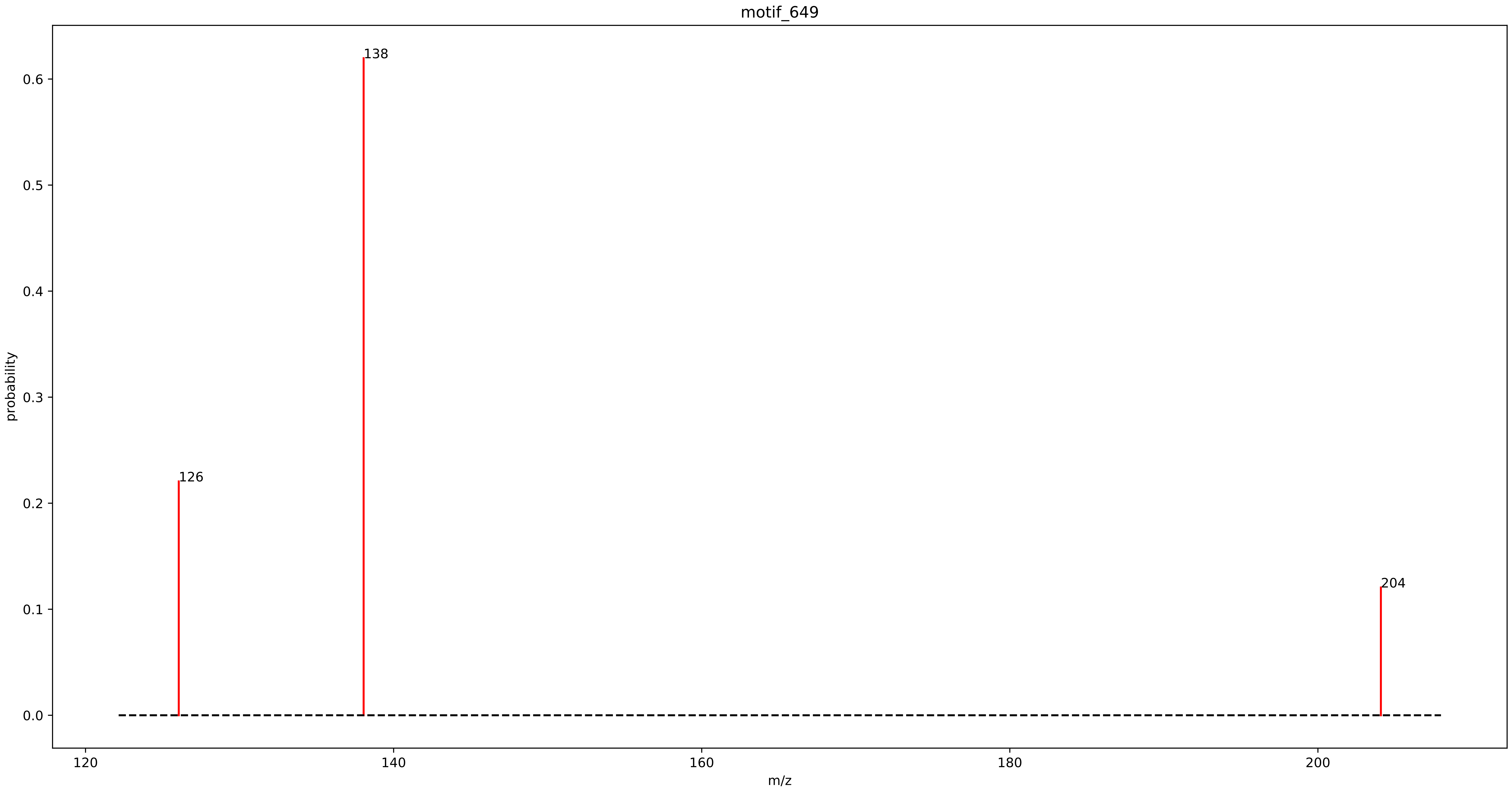

motif_649
138
126
204
0.6
0.5
0.4
0.3
0.2
0.1
0.0
probability
120
140
160
180
200
m/z

motif_649

fragment_138.0525	: 0.62
fragment_126.0525	: 0.22
fragment_204.0875	: 0.12

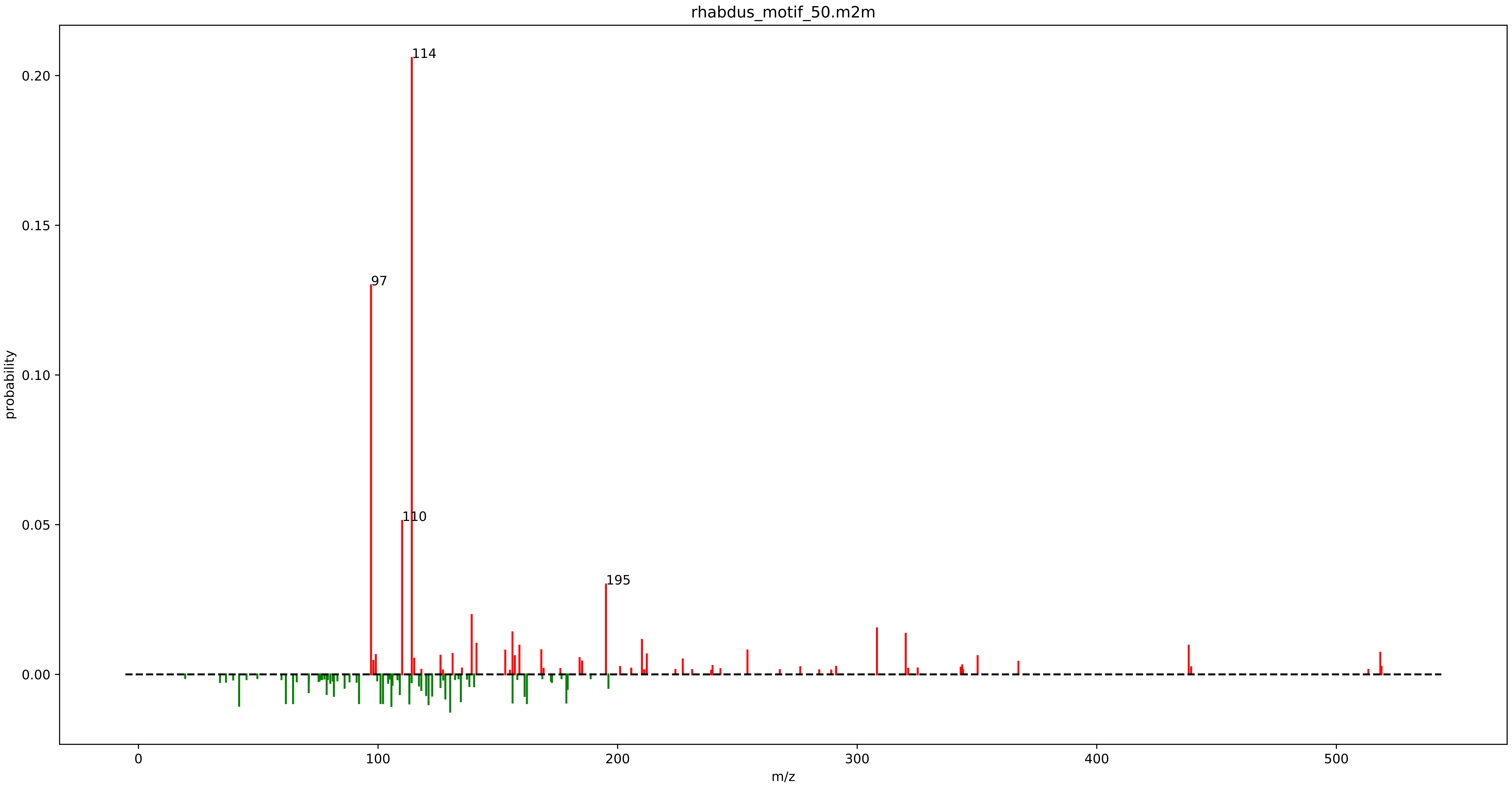

rhabdus_motif_50.m2m
114
97
110
195
0.20
0.15
0.10
0.05
0.00
probability
0
100
200
300
400
500
m/z

rhabdus_motif_50.m2m

fragment_114.1025	: 0.21
fragment_97.0725	: 0.13
fragment_110.0725	: 0.05
fragment_195.1475	: 0.03
fragment_139.0975	: 0.02
fragment_308.2425	: 0.02
fragment_156.1225	: 0.01
fragment_320.2575	: 0.01
loss_130.1225	: 0.01
fragment_210.1725	: 0.01
loss_105.5875	: 0.01
loss_42.0225	: 0.01
fragment_141.1025	: 0.01
loss_121.0875	: 0.01
loss_113.0725	: 0.01
loss_61.5375	: 0.01
loss_64.5425	: 0.01
loss_92.0675	: 0.01
fragment_438.3675	: 0.01
loss_162.1325	: 0.01
loss_102.0725	: 0.01
loss_101.0375	: 0.01
fragment_159.0125	: 0.01
loss_178.6075	: 0.01
loss_156.1575	: 0.01
loss_134.5675	: 0.01
loss_128.0975	: 0.01
fragment_168.1475	: 0.01
fragment_254.1975	: 0.01
fragment_153.1025	: 0.01
fragment_518.3425	: 0.01
loss_161.1775	: 0.01
loss_81.5725	: 0.01
loss_122.6125	: 0.01
loss_120.0825	: 0.01
fragment_131.1275	: 0.01
fragment_212.1775	: 0.01
loss_109.0975	: 0.01
loss_78.5625	: 0.01
fragment_99.0875	: 0.01
fragment_126.0875	: 0.01
fragment_350.2675	: 0.01
loss_113.0925	: 0.01
fragment_157.0975	: 0.01
loss_71.0725	: 0.01
fragment_184.1325	: 0.01
fragment_115.1025	: 0.01
loss_118.0675	: 0.01

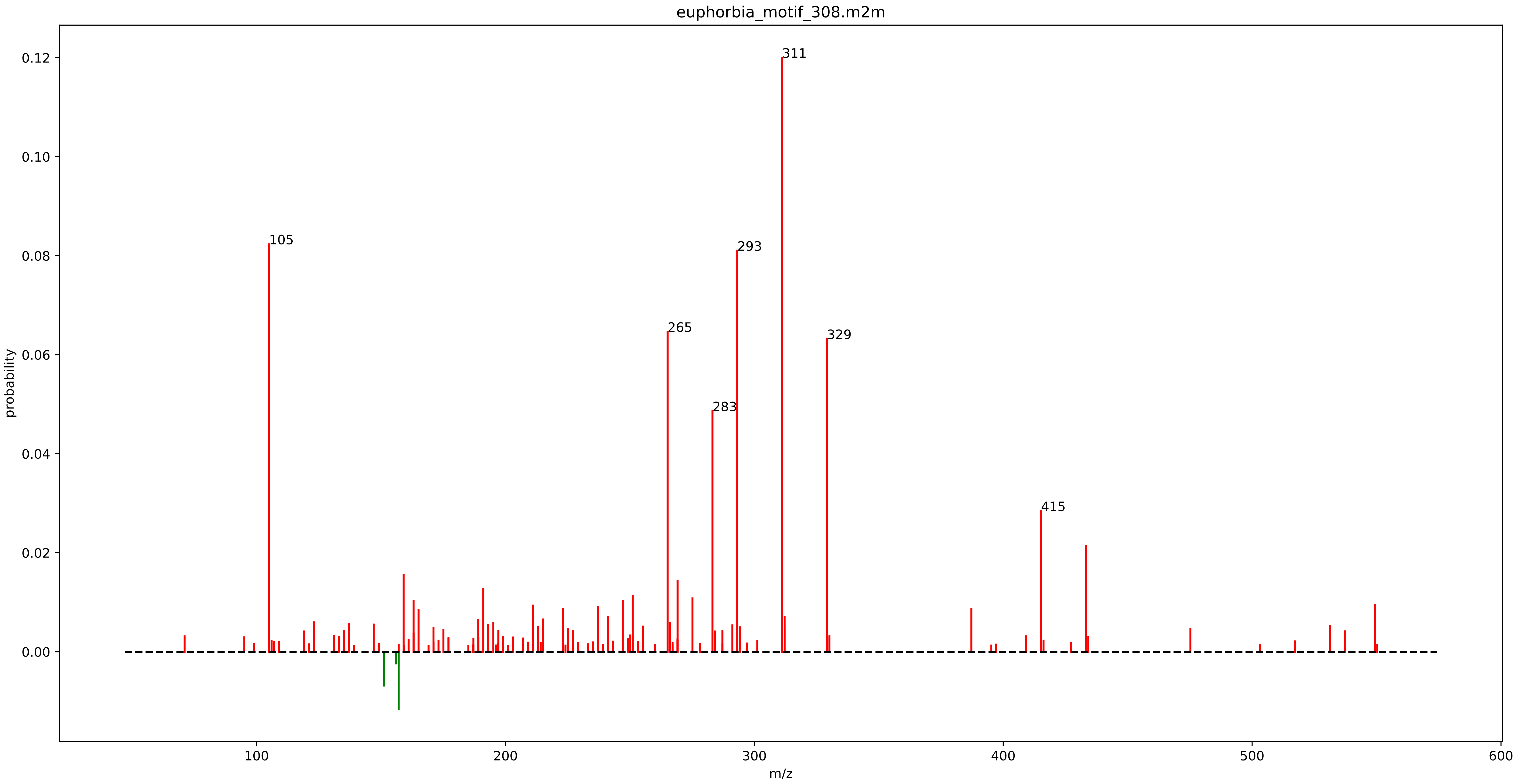

euphorbia_motif_308.m2m
probability
m/z
0.12
0.10
0.08
0.06
0.04
0.02
0.00
100
200
300
400
500
600
311
105
293
265
329
283
415

euphorbia_motif_308.m2m

fragment_311.1625	: 0.12
fragment_105.0325	: 0.08
fragment_293.1525	: 0.08
fragment_265.1575	: 0.06
fragment_329.1725	: 0.06
fragment_283.1675	: 0.05
fragment_415.1875	: 0.03
fragment_433.1975	: 0.02
fragment_159.0775	: 0.02
fragment_269.1525	: 0.01
fragment_191.0675	: 0.01
loss_157.0725	: 0.01
fragment_251.1425	: 0.01
fragment_275.1425	: 0.01
fragment_163.0725	: 0.01
fragment_247.1475	: 0.01
fragment_549.2825	: 0.01
fragment_211.1125	: 0.01
fragment_237.1625	: 0.01
fragment_223.1125	: 0.01
fragment_387.1925	: 0.01
fragment_165.0875	: 0.01
fragment_241.1225	: 0.01
fragment_312.1625	: 0.01
loss_151.1175	: 0.01
fragment_215.1075	: 0.01
fragment_189.0875	: 0.01
fragment_223.1475	: 0.01
fragment_123.0775	: 0.01
fragment_266.1625	: 0.01
fragment_195.1175	: 0.01
fragment_137.0975	: 0.01
fragment_433.2025	: 0.01
fragment_147.0775	: 0.01
fragment_193.0825	: 0.01
fragment_291.1575	: 0.01
fragment_531.2725	: 0.01
fragment_255.1725	: 0.01
fragment_213.1275	: 0.01

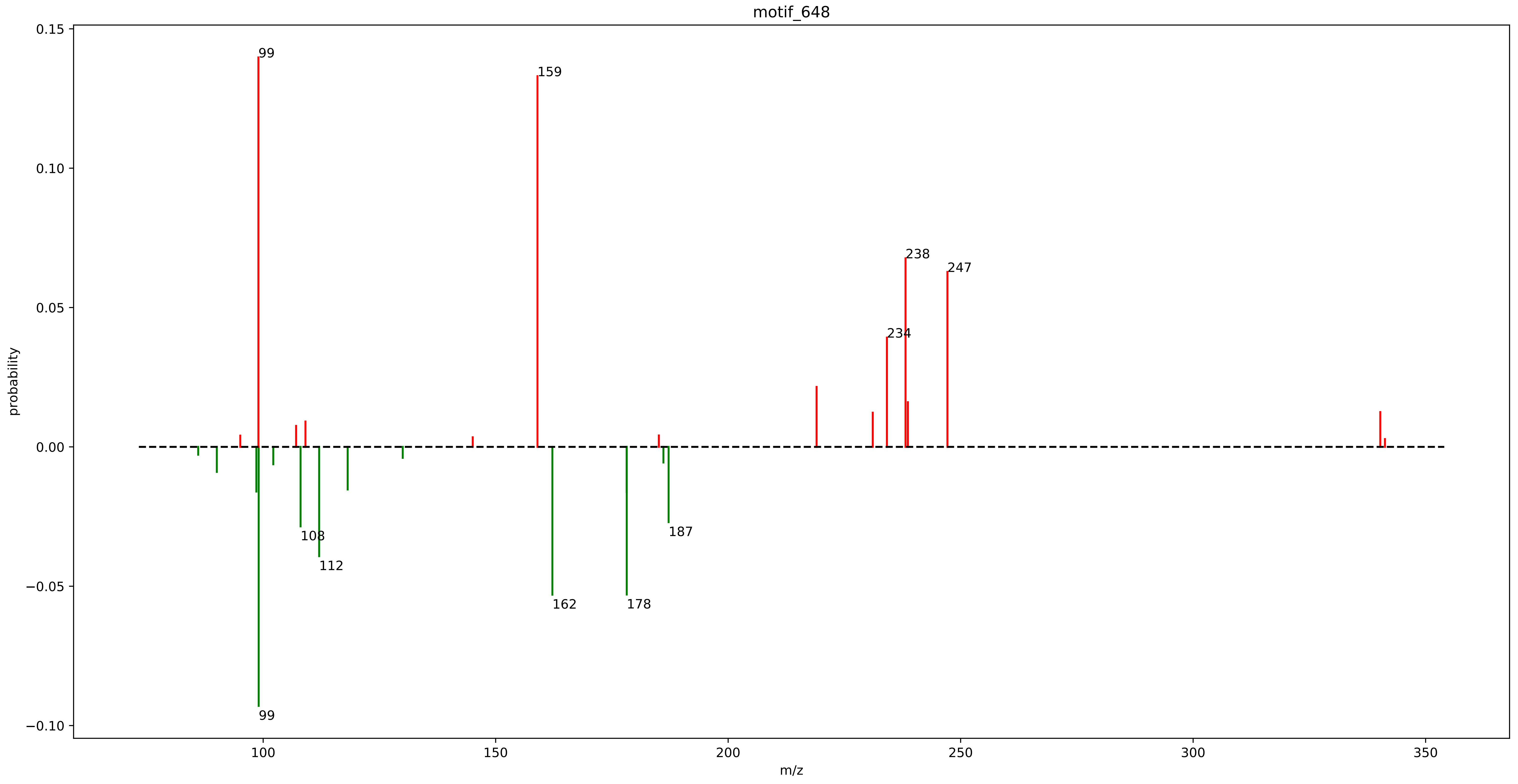

motif_648
probability
m/z
0.15
0.10
0.05
0.00
−0.05
−0.10
100
150
200
250
300
350
99
159
238
247
234
108
112
162
178
187
99

motif_648

fragment_98.9725	: 0.14
fragment_158.9925	: 0.13
loss_99.0375	: 0.09
fragment_238.1475	: 0.07
fragment_247.1525	: 0.06
loss_162.1975	: 0.05
loss_178.1875	: 0.05
fragment_234.1475	: 0.04
loss_112.0375	: 0.04
loss_108.0425	: 0.03
loss_187.1925	: 0.03
fragment_219.0175	: 0.02
loss_178.1725	: 0.02
loss_98.5325	: 0.02
fragment_238.6475	: 0.02
loss_118.1675	: 0.02
fragment_340.2475	: 0.01
fragment_231.0975	: 0.01
fragment_109.0975	: 0.01
loss_90.0325	: 0.01
fragment_107.0825	: 0.01
loss_102.1725	: 0.01
loss_186.0775	: 0.01

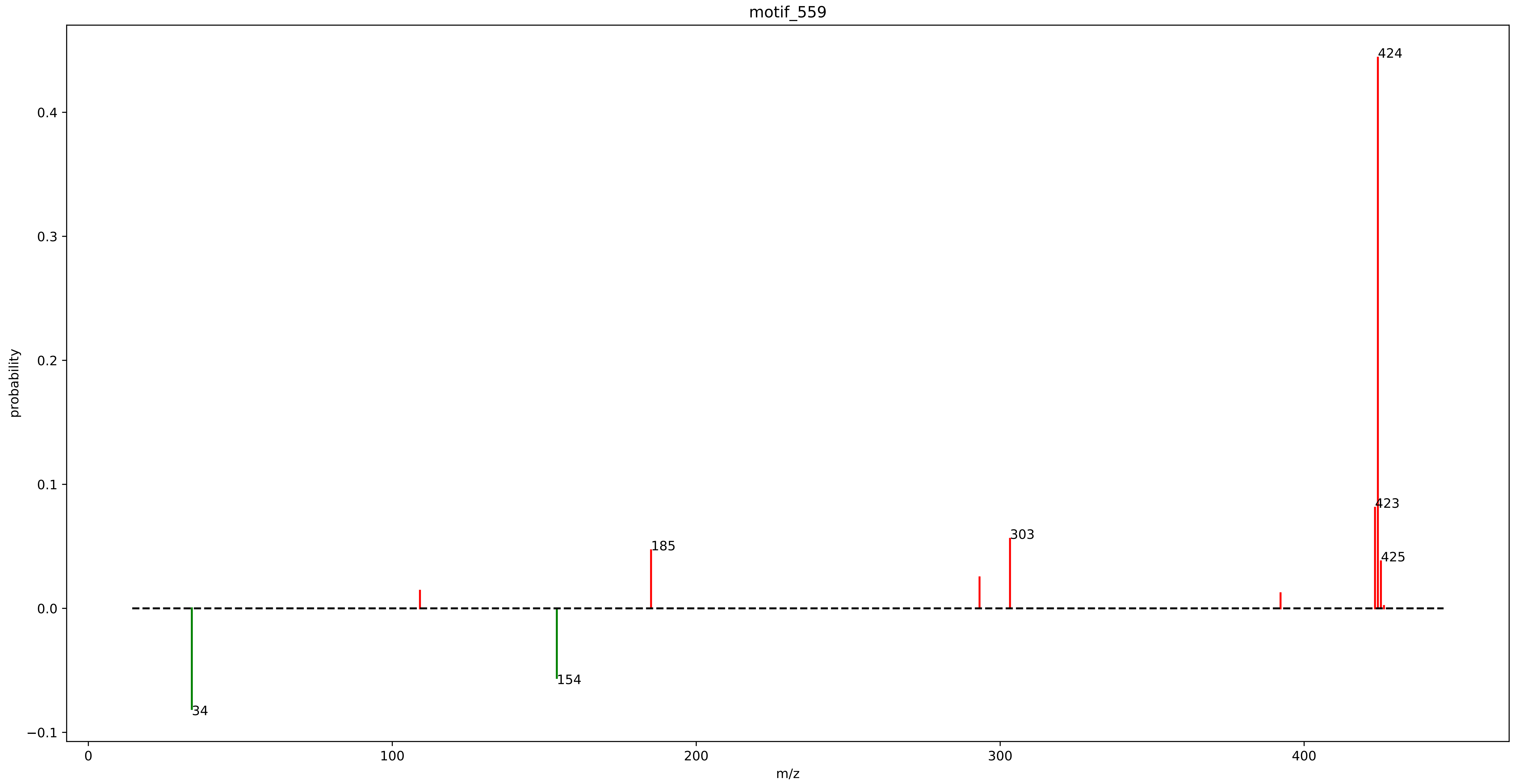
motif_559
probability
m/z
0.4
0.3
0.2
0.1
0.0
−0.1
0
100
200
300
400
424
423
425
303
185
154
34

motif_559

fragment_424.2675	: 0.44
loss_34.0425	: 0.08
fragment_423.3225	: 0.08
fragment_303.2325	: 0.06
loss_154.1325	: 0.06
fragment_185.1325	: 0.05
fragment_425.2675	: 0.04
fragment_293.2075	: 0.02
fragment_109.0975	: 0.01
fragment_392.2425	: 0.01

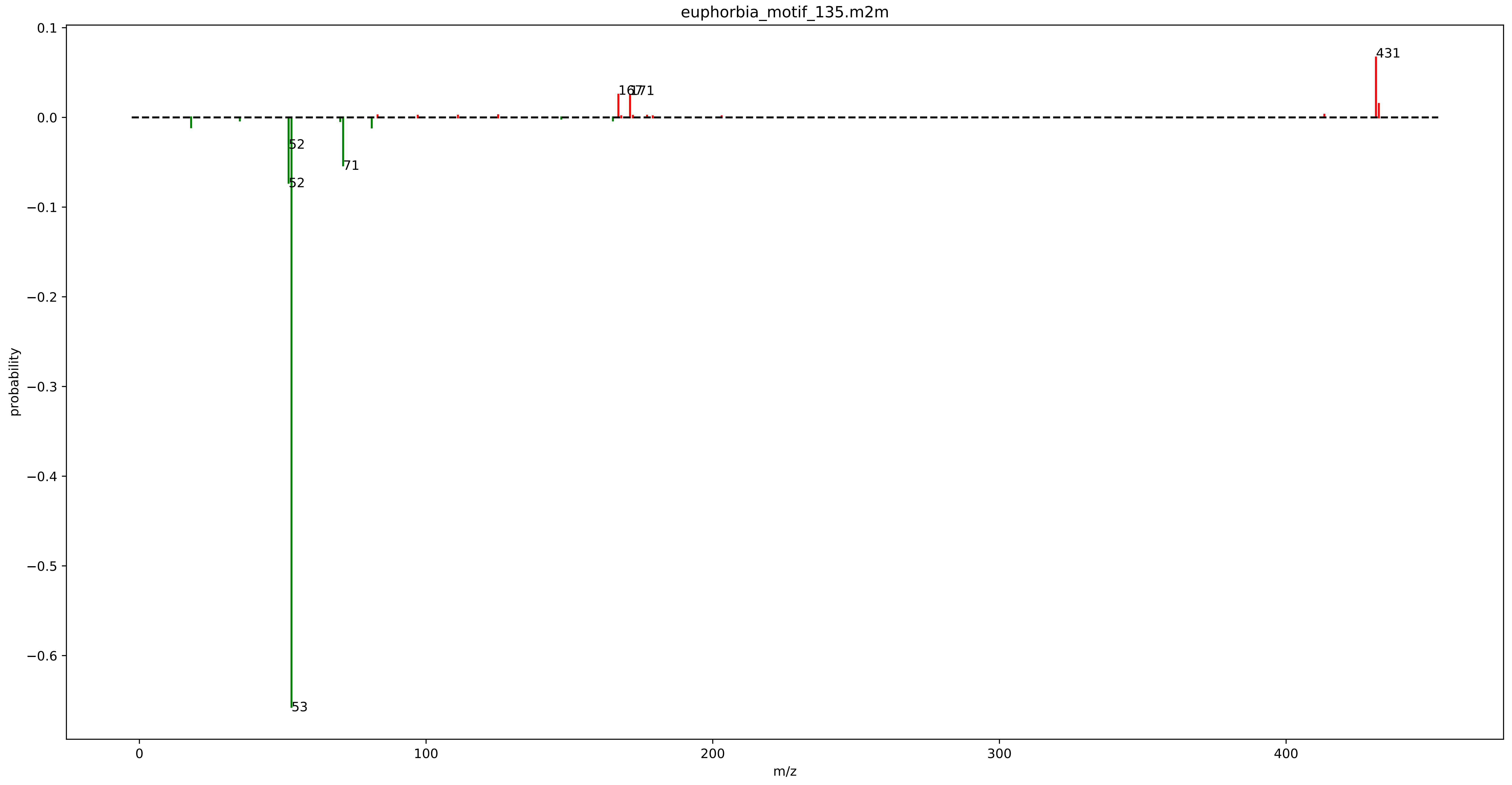

euphorbia_motif_135.m2m
probability
m/z
431
167
171
52
52
71
53

euphorbia_motif_135.m2m

loss_53.0475	: 0.66
loss_52.0425	: 0.07
fragment_431.3475	: 0.07
loss_71.0575	: 0.05
loss_52.0475	: 0.03
fragment_167.0675	: 0.03
fragment_171.1375	: 0.03
fragment_432.3475	: 0.02
loss_81.0425	: 0.01
loss_18.0375	: 0.01

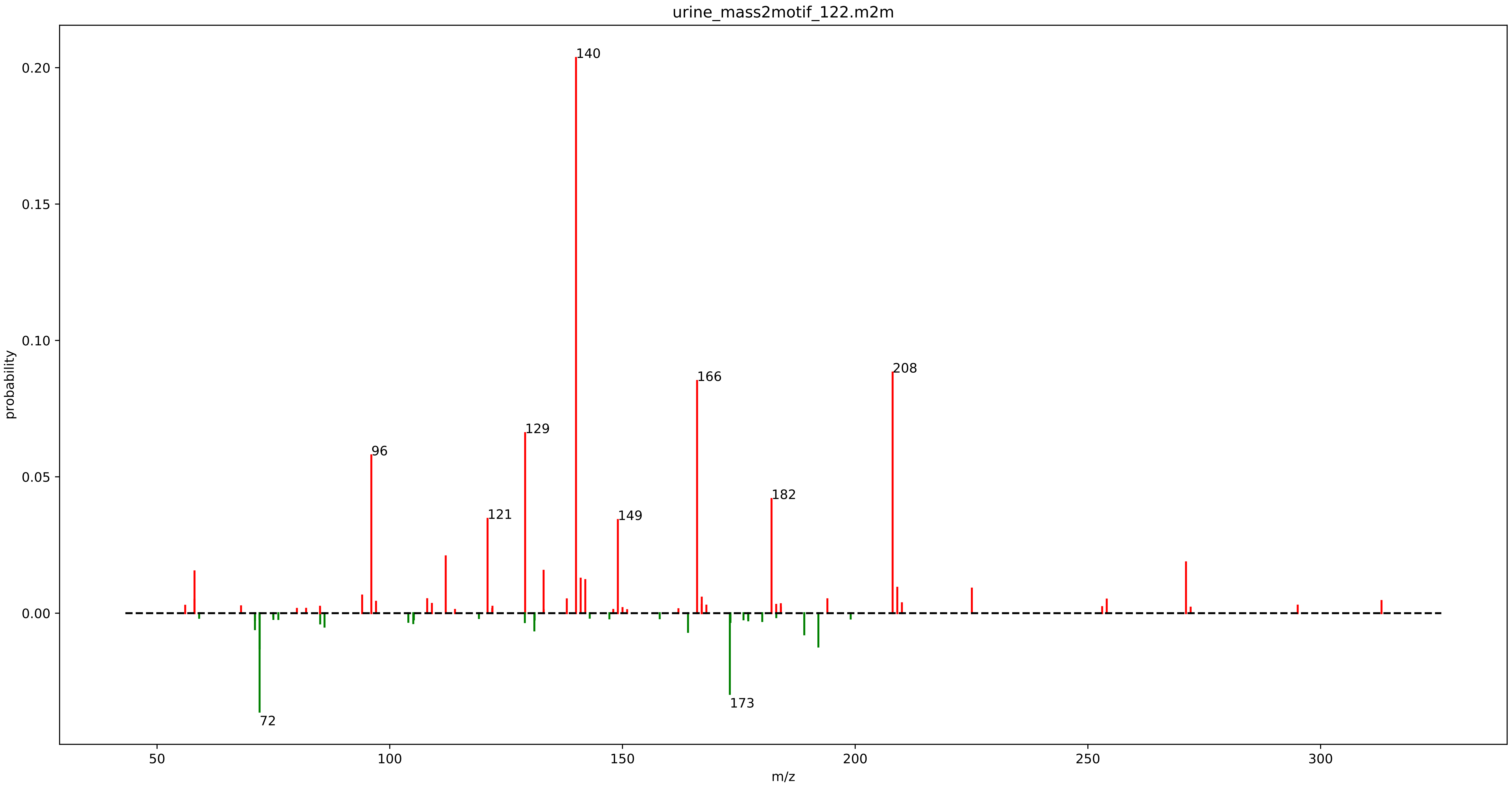

urine_mass2motif_122.m2m
probability
m/z
0.20
0.15
0.10
0.05
0.00
50
100
150
200
250
300
140
166
208
129
96
182
121
149
72
173

urine_mass2motif_122.m2m

fragment_140.0175	: 0.20
fragment_208.0425	: 0.09
fragment_166.0325	: 0.09
fragment_129.0875	: 0.07
fragment_96.0425	: 0.06
fragment_182.0275	: 0.04
loss_72.0325	: 0.04
fragment_121.0125	: 0.03
fragment_149.0075	: 0.03
loss_173.0675	: 0.03
fragment_112.0225	: 0.02
fragment_271.0775	: 0.02
fragment_133.0525	: 0.02
fragment_58.0475	: 0.02
loss_72.0425	: 0.01
fragment_141.0175	: 0.01
loss_192.0925	: 0.01
fragment_142.0125	: 0.01
fragment_209.0475	: 0.01
fragment_225.0675	: 0.01
loss_189.0625	: 0.01
loss_164.0825	: 0.01
fragment_94.0675	: 0.01
loss_131.0675	: 0.01
loss_71.0325	: 0.01
fragment_167.0325	: 0.01
fragment_108.0425	: 0.01
fragment_194.0275	: 0.01
fragment_138.0375	: 0.01
fragment_254.0475	: 0.01

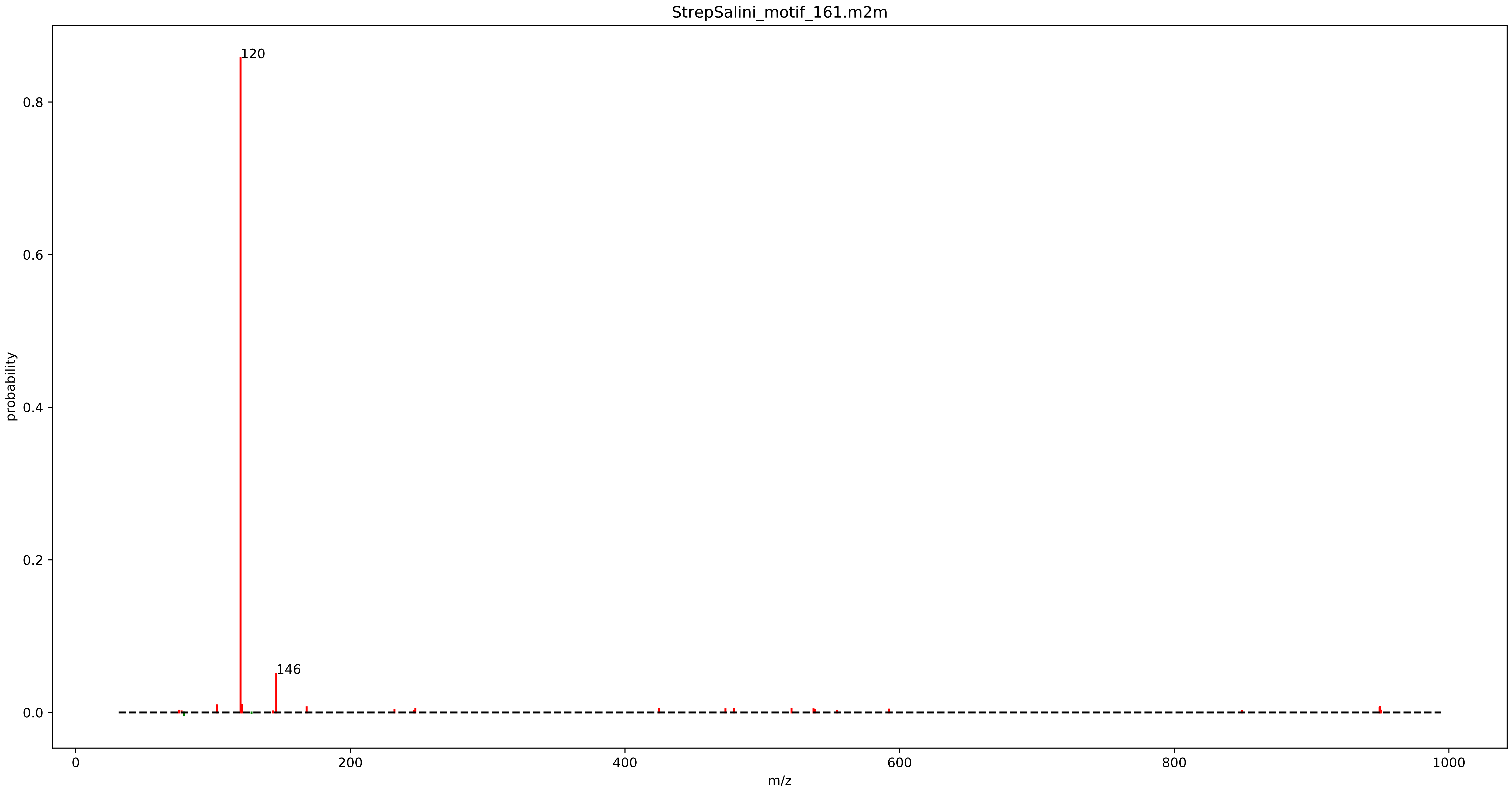

StrepSalini_motif_161.m2m
probability
m/z
120
146
0.8
0.6
0.4
0.2
0.0
0
200
400
600
800
1000

StrepSalini_motif_161.m2m

fragment_120.0500	: 0.86
fragment_146.0500	: 0.05
fragment_121.0525	: 0.01
fragment_103.0525	: 0.01
fragment_949.9500	: 0.01
fragment_168.1500	: 0.01
fragment_949.4500	: 0.01

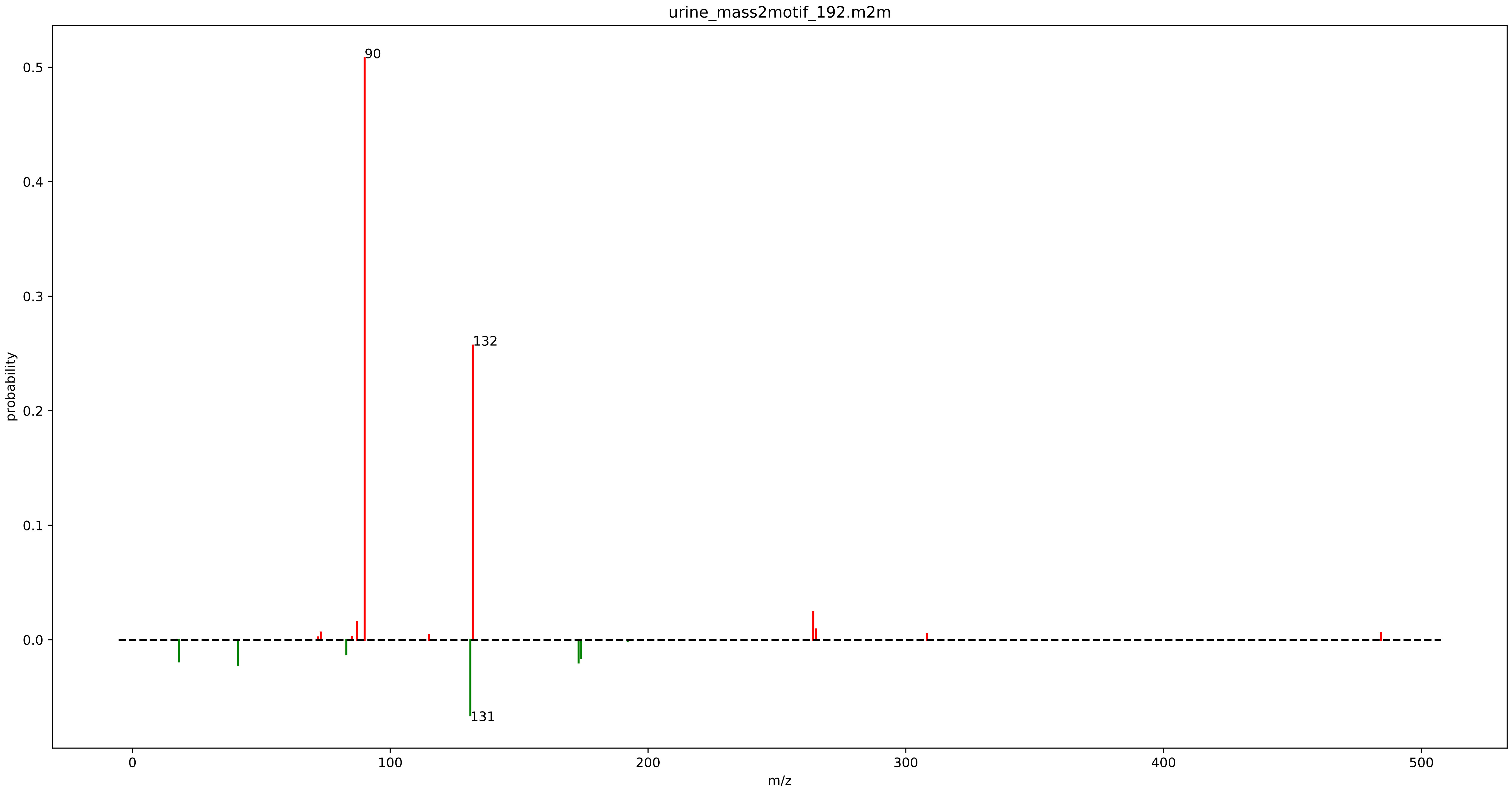

urine_mass2motif_192.m2m
probability
m/z
0.5
0.4
0.3
0.2
0.1
0.0
0
100
200
300
400
500
90
132
131

urine_mass2motif_192.m2m

fragment_90.0525	: 0.51
fragment_132.0775	: 0.26
loss_131.0675	: 0.07
fragment_264.1175	: 0.02
loss_40.9525	: 0.02
loss_173.0675	: 0.02
loss_17.9725	: 0.02
loss_174.0825	: 0.02
fragment_87.0575	: 0.02
loss_82.9725	: 0.01
fragment_265.1175	: 0.01
fragment_73.0275	: 0.01
fragment_484.2775	: 0.01

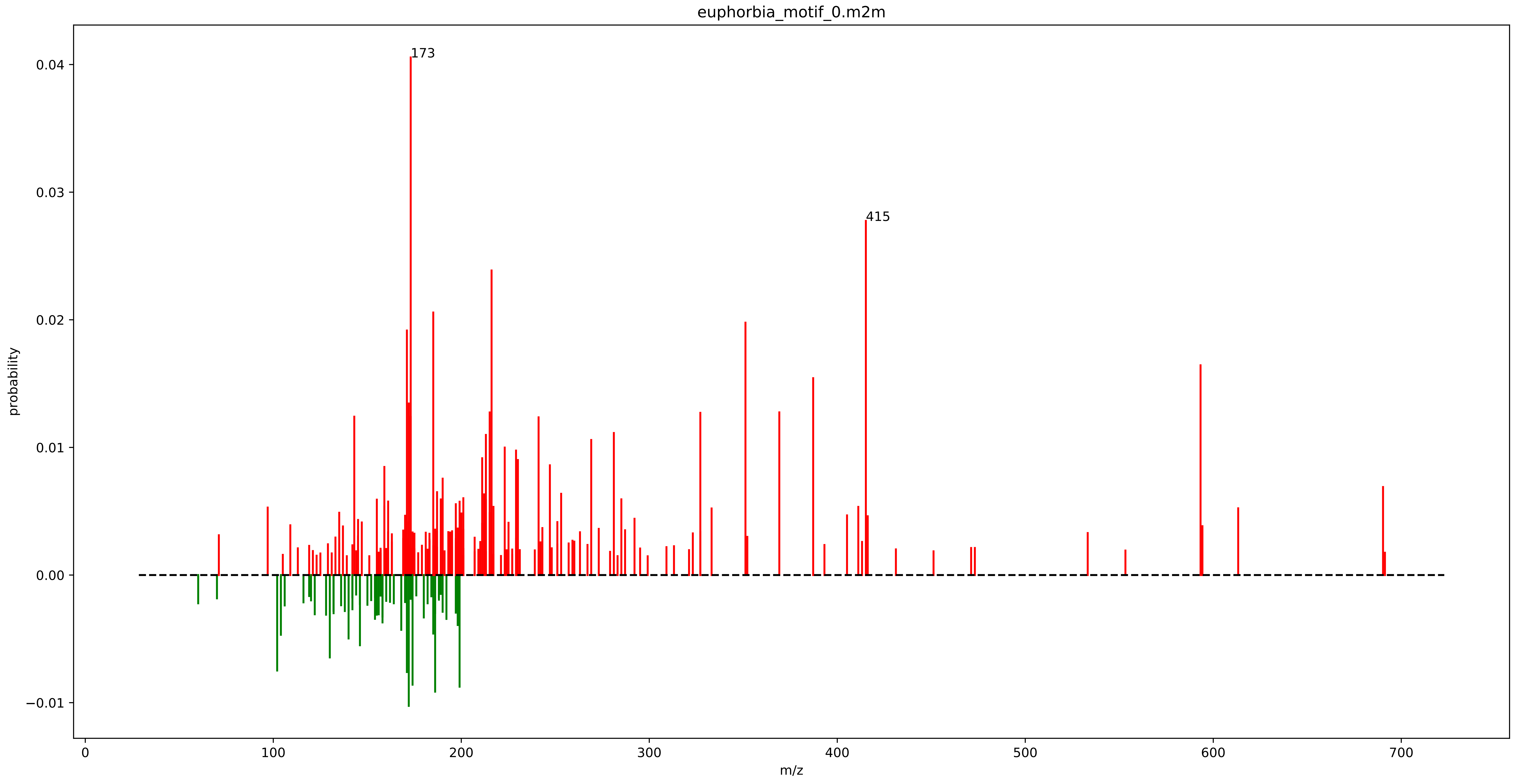

euphorbia_motif_0.m2m
173
415
probability
m/z
0.04
0.03
0.02
0.01
0.00
−0.01
0
100
200
300
400
500
600
700

euphorbia_motif_0.m2m

fragment_173.0975	: 0.04
fragment_415.2075	: 0.03
fragment_216.1125	: 0.02
fragment_185.0975	: 0.02
fragment_351.1625	: 0.02
fragment_171.0775	: 0.02
fragment_593.2375	: 0.02
fragment_387.1775	: 0.02
fragment_172.0875	: 0.01
fragment_369.1725	: 0.01
fragment_215.1075	: 0.01
fragment_327.1575	: 0.01
fragment_143.0825	: 0.01
fragment_173.0925	: 0.01
fragment_241.1225	: 0.01
fragment_281.1575	: 0.01
fragment_213.0925	: 0.01
fragment_269.1175	: 0.01
loss_172.0725	: 0.01
fragment_223.1075	: 0.01
fragment_229.1225	: 0.01
fragment_211.1125	: 0.01
loss_186.0925	: 0.01
fragment_230.1275	: 0.01
loss_199.0975	: 0.01
fragment_247.1325	: 0.01
loss_174.0875	: 0.01
fragment_159.0825	: 0.01
loss_171.0625	: 0.01
fragment_190.0975	: 0.01
loss_102.0675	: 0.01
fragment_690.3075	: 0.01
fragment_187.1125	: 0.01
loss_130.0625	: 0.01
fragment_253.1225	: 0.01
fragment_212.1175	: 0.01
fragment_201.0925	: 0.01
fragment_285.1475	: 0.01
fragment_189.0875	: 0.01
fragment_155.0825	: 0.01
fragment_213.1275	: 0.01
fragment_161.0975	: 0.01
fragment_199.1125	: 0.01
fragment_197.0975	: 0.01
loss_146.0925	: 0.01
fragment_159.0775	: 0.01
fragment_217.1175	: 0.01
fragment_411.1825	: 0.01
fragment_97.0275	: 0.01
fragment_613.2625	: 0.01
fragment_333.1525	: 0.01

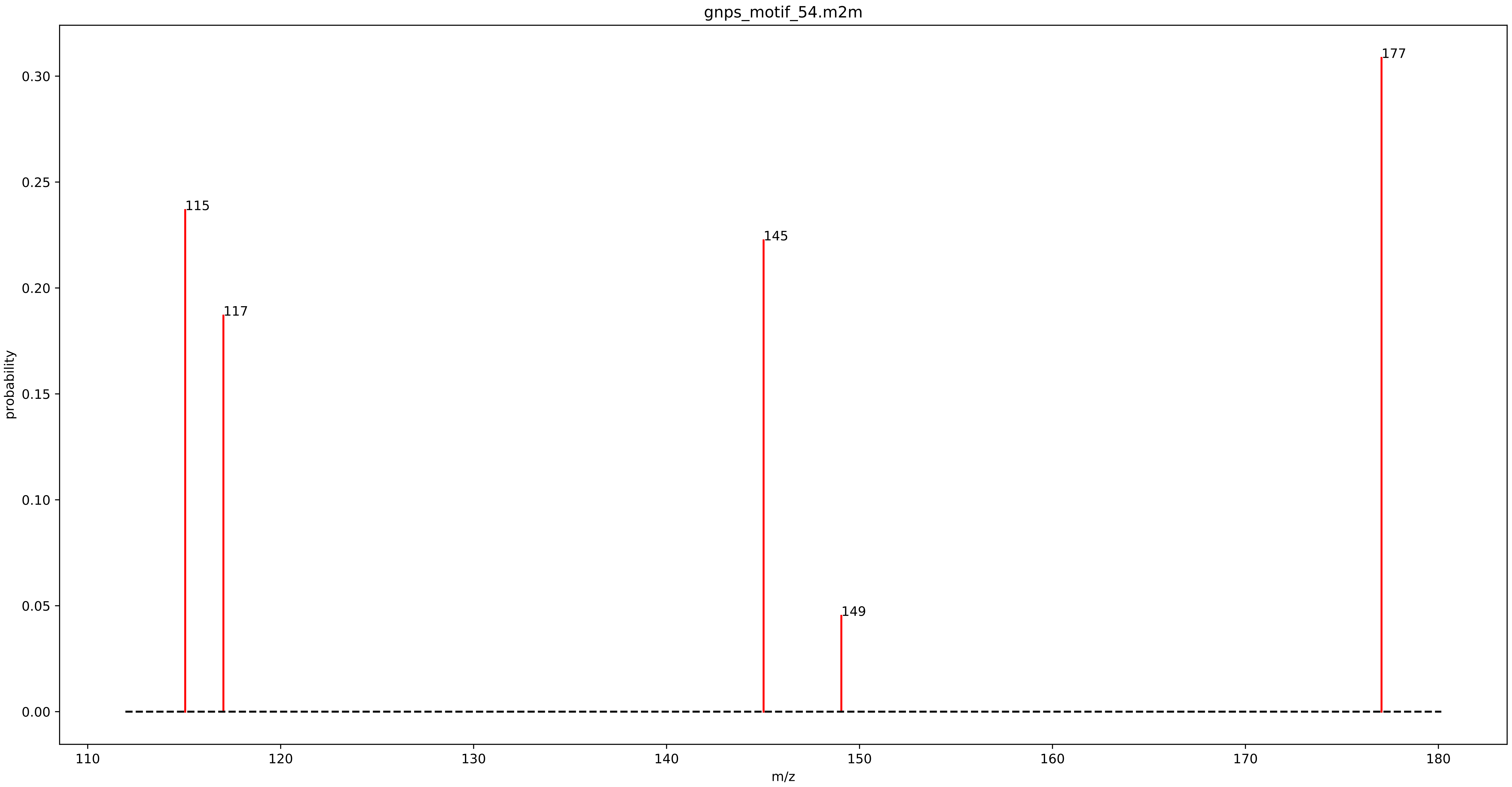
gnps_motif_54.m2m
probability
m/z
0.00
0.05
0.10
0.15
0.20
0.25
0.30
110
120
130
140
150
160
170
180
115
117
145
149
177

gnps_motif_54.m2m

fragment_177.0525	: 0.31
fragment_115.0525	: 0.24
fragment_145.0275	: 0.22
fragment_117.0325	: 0.19
fragment_149.0575	: 0.05

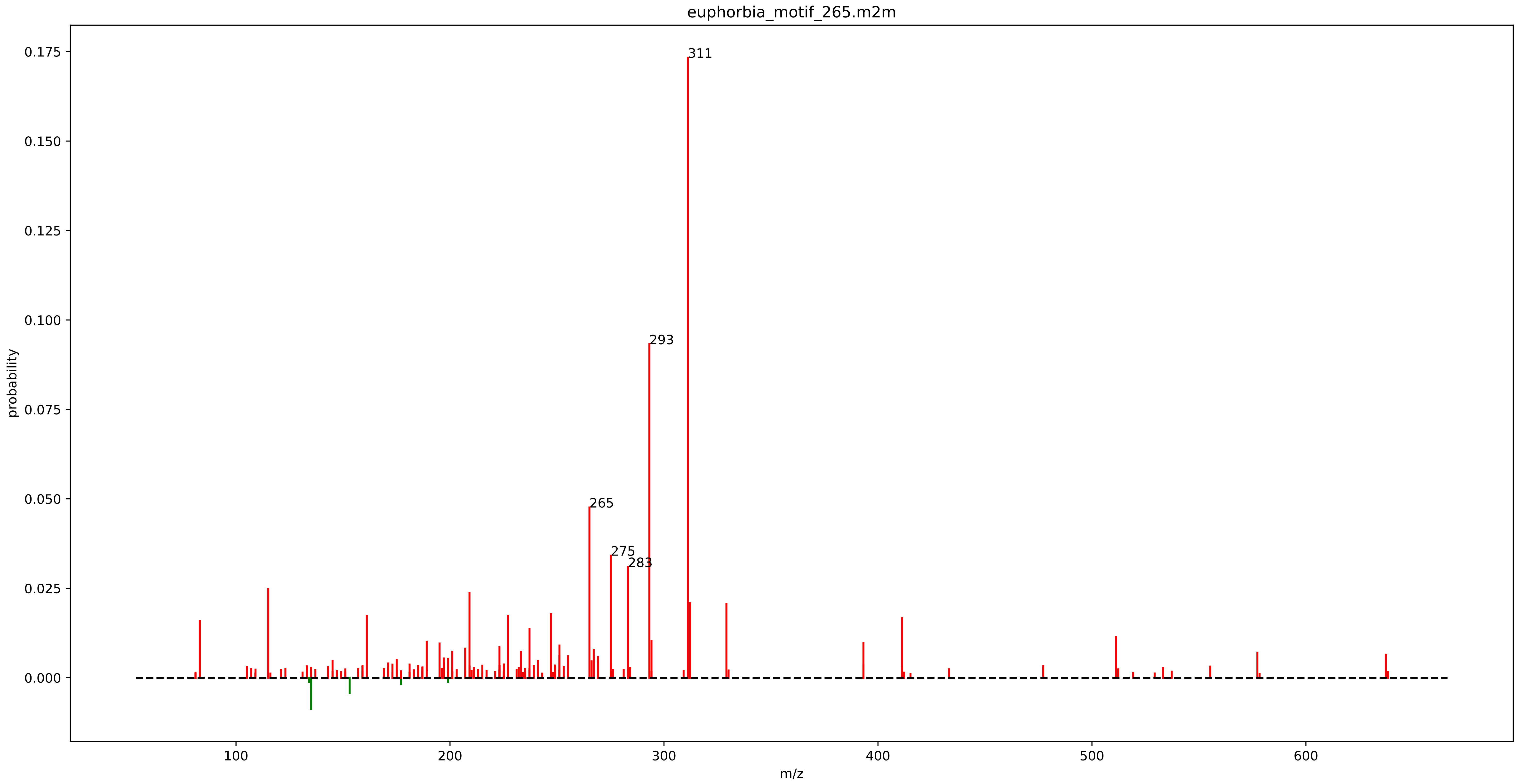

euphorbia_motif_265.m2m
probability
m/z
0.175
0.150
0.125
0.100
0.075
0.050
0.025
0.000
100
200
300
400
500
600
311
293
265
275
283

euphorbia_motif_265.m2m

fragment_311.1625	: 0.17
fragment_293.1525	: 0.09
fragment_265.1575	: 0.05
fragment_275.1375	: 0.03
fragment_283.1675	: 0.03
fragment_115.0525	: 0.02
fragment_209.0975	: 0.02
fragment_312.1675	: 0.02
fragment_329.1725	: 0.02
fragment_247.1475	: 0.02
fragment_227.1075	: 0.02
fragment_161.0975	: 0.02
fragment_411.2175	: 0.02
fragment_83.0475	: 0.02
fragment_237.1625	: 0.01
fragment_511.2675	: 0.01
fragment_294.1575	: 0.01
fragment_189.0925	: 0.01
fragment_393.2025	: 0.01
fragment_195.1175	: 0.01
fragment_251.1425	: 0.01
loss_135.0925	: 0.01
fragment_223.1125	: 0.01
fragment_207.1025	: 0.01
fragment_267.1375	: 0.01
fragment_201.0925	: 0.01
fragment_233.1325	: 0.01
fragment_577.3125	: 0.01
fragment_637.3175	: 0.01
fragment_251.1075	: 0.01
fragment_255.1725	: 0.01
fragment_269.1525	: 0.01
fragment_197.0975	: 0.01
fragment_199.1125	: 0.01
fragment_267.1725	: 0.01

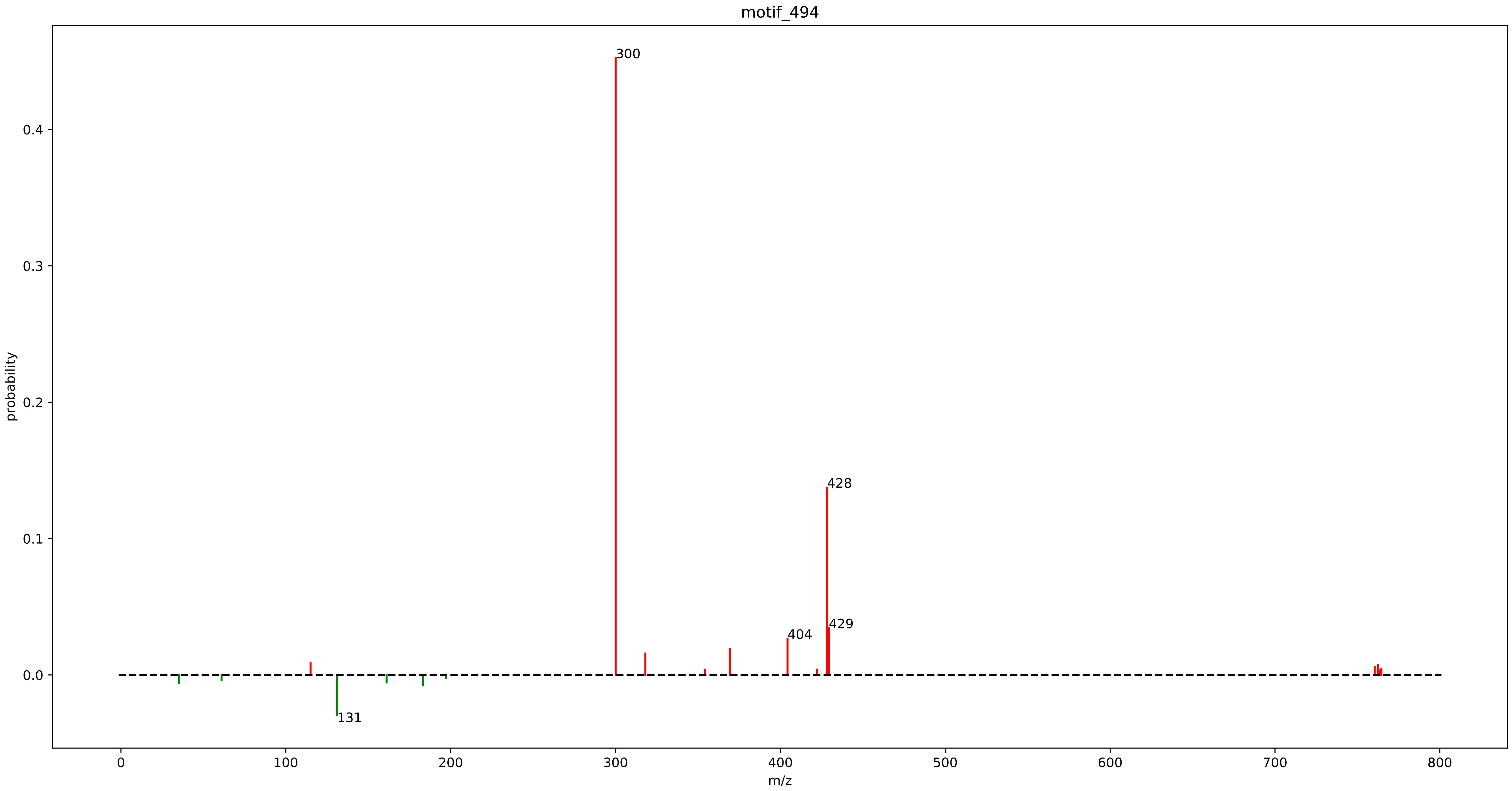

motif_494
300
428
429
404
131
probability
0.4
0.3
0.2
0.1
0.0
0
100
200
300
400
500
600
700
800
m/z

motif_494

fragment_300.1075	: 0.45
fragment_428.3375	: 0.14
fragment_429.3425	: 0.03
loss_131.1375	: 0.03
fragment_404.3125	: 0.03
fragment_369.3225	: 0.02
fragment_318.1175	: 0.02
fragment_115.0375	: 0.01
loss_183.1725	: 0.01
fragment_762.4775	: 0.01
loss_35.1175	: 0.01
fragment_760.4625	: 0.01
loss_161.1475	: 0.01

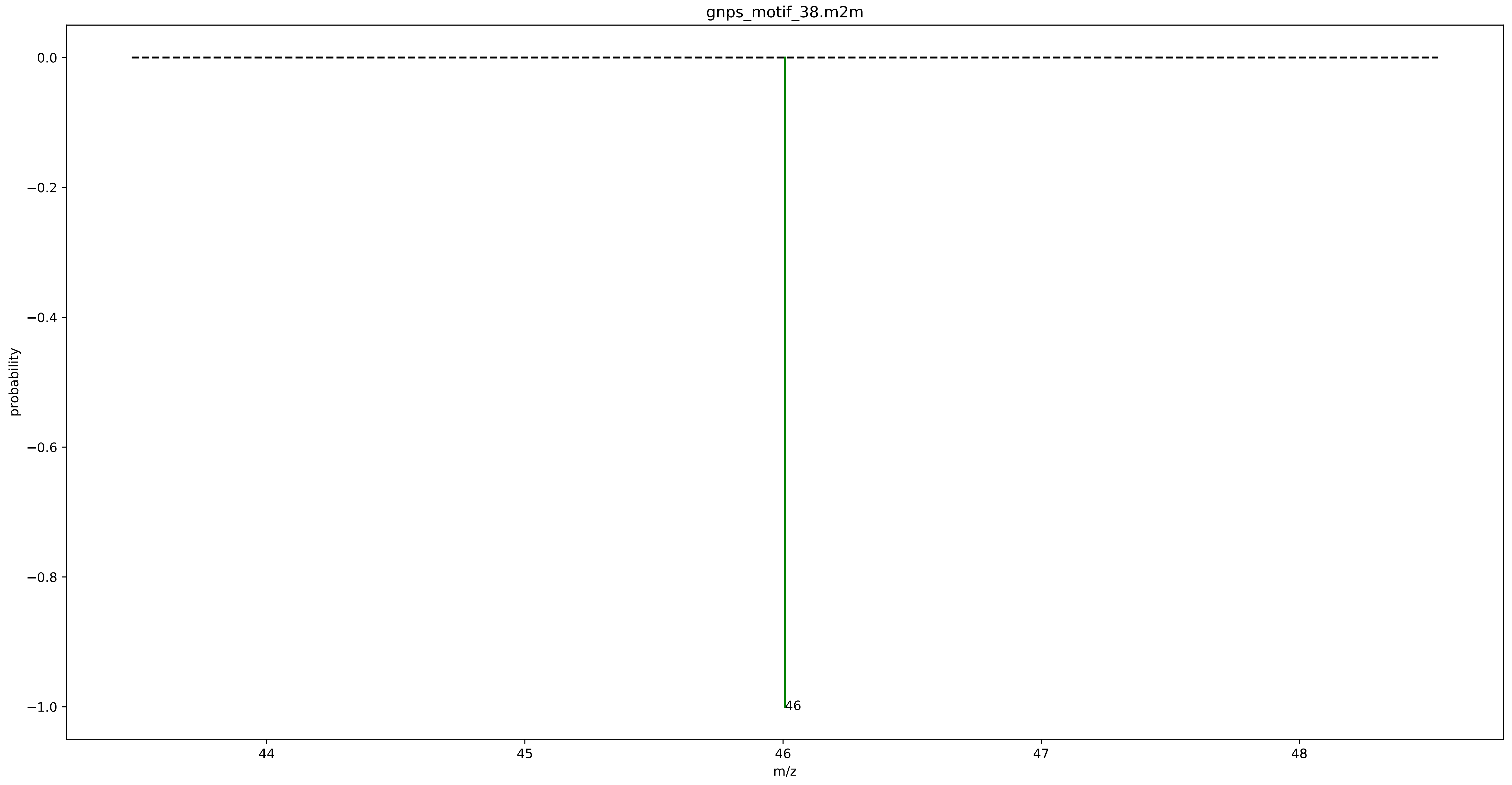

gnps_motif_38.m2m
0.0
−0.2
−0.4
−0.6
−0.8
−1.0
probability
46
44
45
46
47
48
m/z

gnps_motif_38.m2m

loss_46.0075 : 1.00

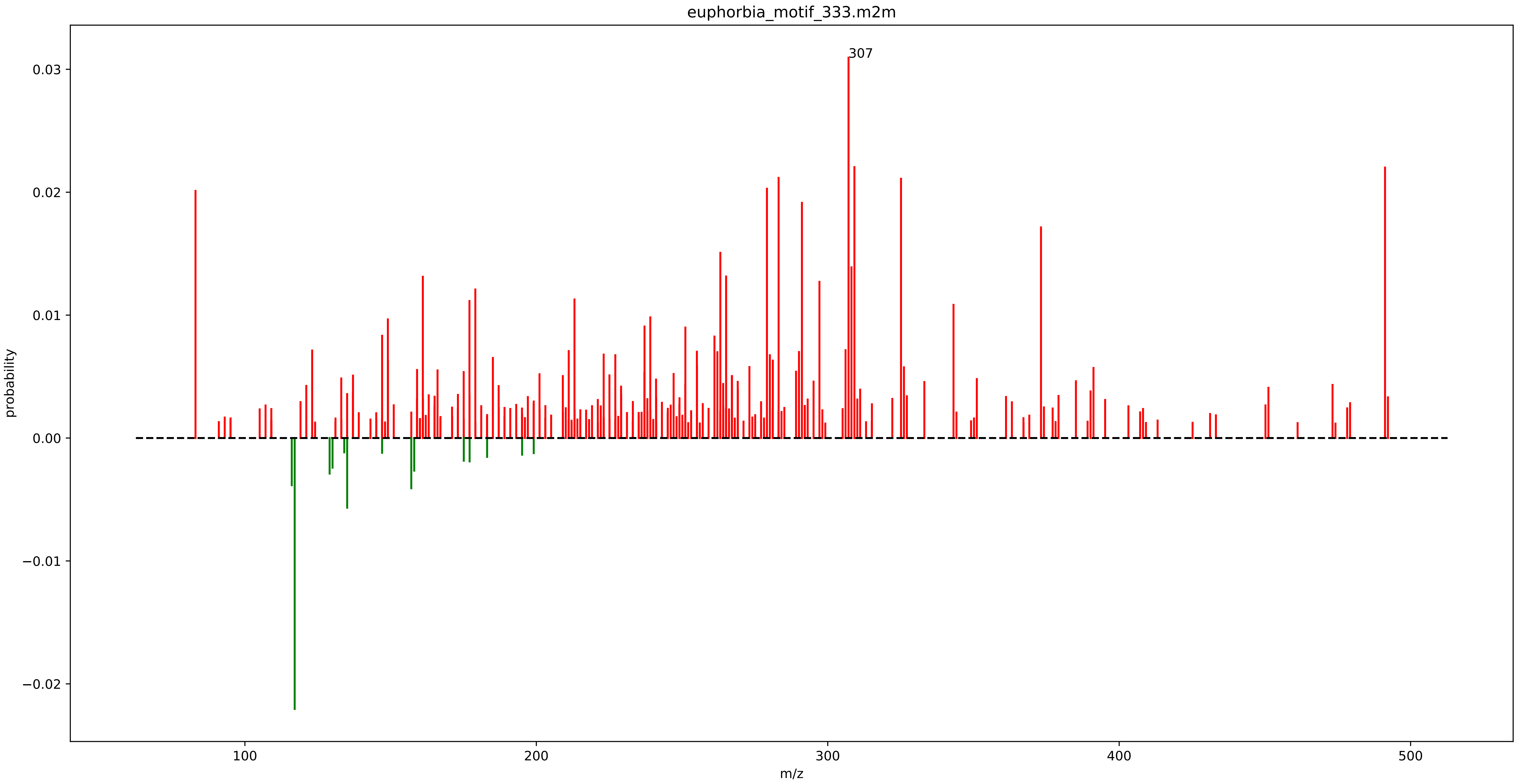

euphorbia_motif_333.m2m
307
0.03
0.02
0.01
0.00
−0.01
−0.02
probability
100
200
300
400
500
m/z

euphorbia_motif_333.m2m

fragment_307.1275	: 0.03
fragment_309.1475	: 0.02
loss_117.0775	: 0.02
fragment_491.2275	: 0.02
fragment_283.1325	: 0.02
fragment_325.1425	: 0.02
fragment_279.1375	: 0.02
fragment_83.0475	: 0.02
fragment_291.1375	: 0.02
fragment_373.1775	: 0.02
fragment_263.1425	: 0.02
fragment_308.1375	: 0.01
fragment_265.1225	: 0.01
fragment_161.0575	: 0.01
fragment_297.1475	: 0.01
fragment_179.0675	: 0.01
fragment_213.0875	: 0.01
fragment_177.0525	: 0.01
fragment_343.1525	: 0.01
fragment_239.1075	: 0.01
fragment_149.0575	: 0.01
fragment_237.1225	: 0.01
fragment_251.1425	: 0.01
fragment_147.0775	: 0.01
fragment_261.1225	: 0.01
fragment_306.1075	: 0.01
fragment_123.0775	: 0.01
fragment_211.1125	: 0.01
fragment_255.0975	: 0.01
fragment_290.1275	: 0.01
fragment_262.1325	: 0.01
fragment_223.1075	: 0.01
fragment_227.1025	: 0.01
fragment_280.1425	: 0.01
fragment_185.0925	: 0.01
fragment_281.1525	: 0.01
fragment_149.0925	: 0.01
fragment_255.1325	: 0.01
fragment_273.1175	: 0.01
fragment_326.1475	: 0.01
fragment_391.1875	: 0.01
loss_135.0925	: 0.01
fragment_159.0775	: 0.01
fragment_166.0975	: 0.01
fragment_289.1175	: 0.01
fragment_175.0725	: 0.01
fragment_237.0875	: 0.01
fragment_247.1075	: 0.01
fragment_201.0875	: 0.01
fragment_225.0875	: 0.01
fragment_137.0925	: 0.01
fragment_209.0925	: 0.01
fragment_267.1025	: 0.01
fragment_267.1325	: 0.01

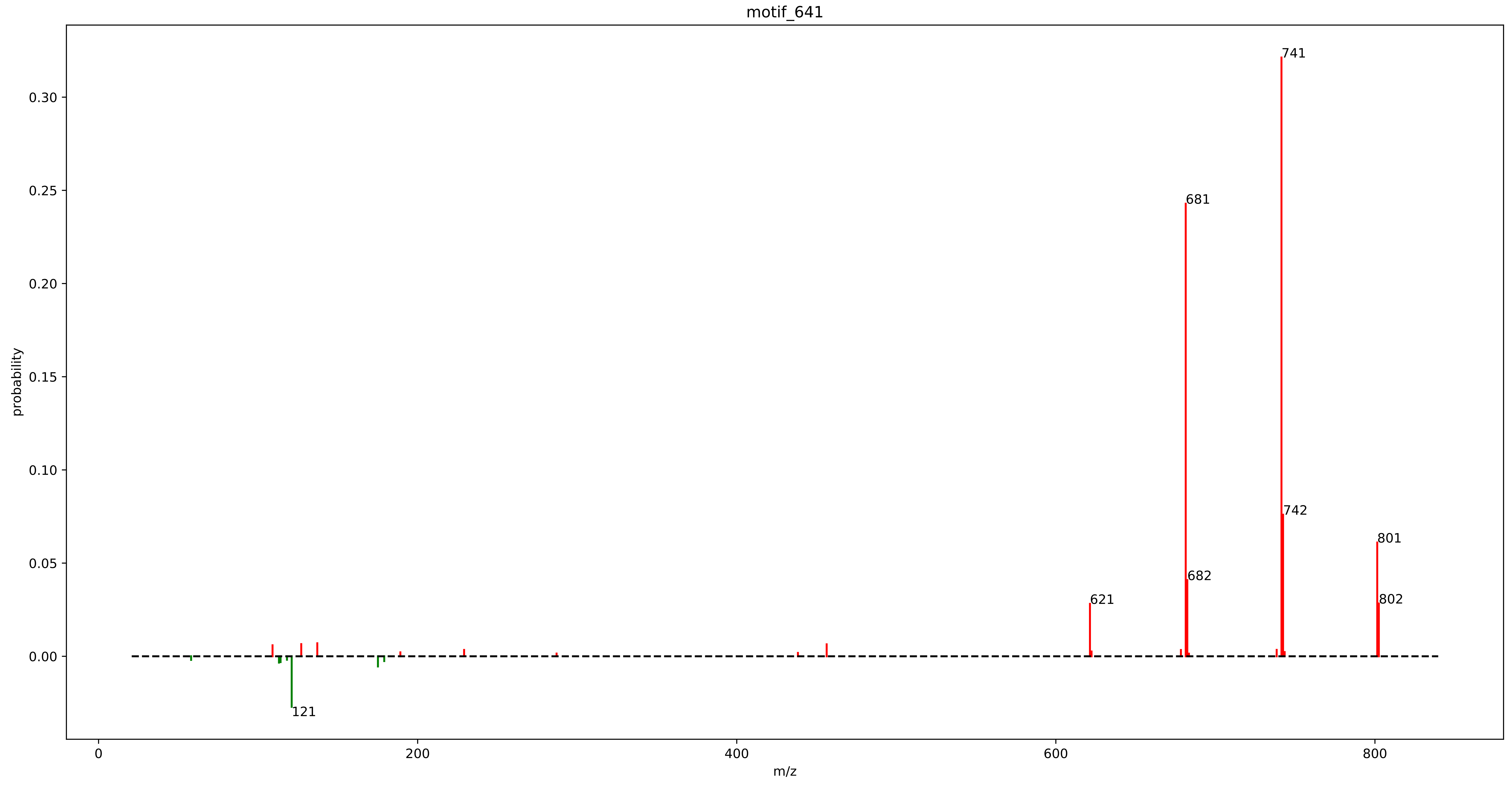

motif_641
probability
m/z
0.30
0.25
0.20
0.15
0.10
0.05
0.00
0
200
400
600
800
741
681
742
801
682
621
802
121

motif_641

fragment_741.4175	: 0.32
fragment_681.3975	: 0.24
fragment_742.4225	: 0.08
fragment_801.4425	: 0.06
fragment_682.4025	: 0.04
fragment_802.4425	: 0.03
fragment_621.3775	: 0.03
loss_121.0425	: 0.03
fragment_137.0975	: 0.01
fragment_127.0375	: 0.01
fragment_456.3575	: 0.01
fragment_109.0425	: 0.01
loss_175.1075	: 0.01

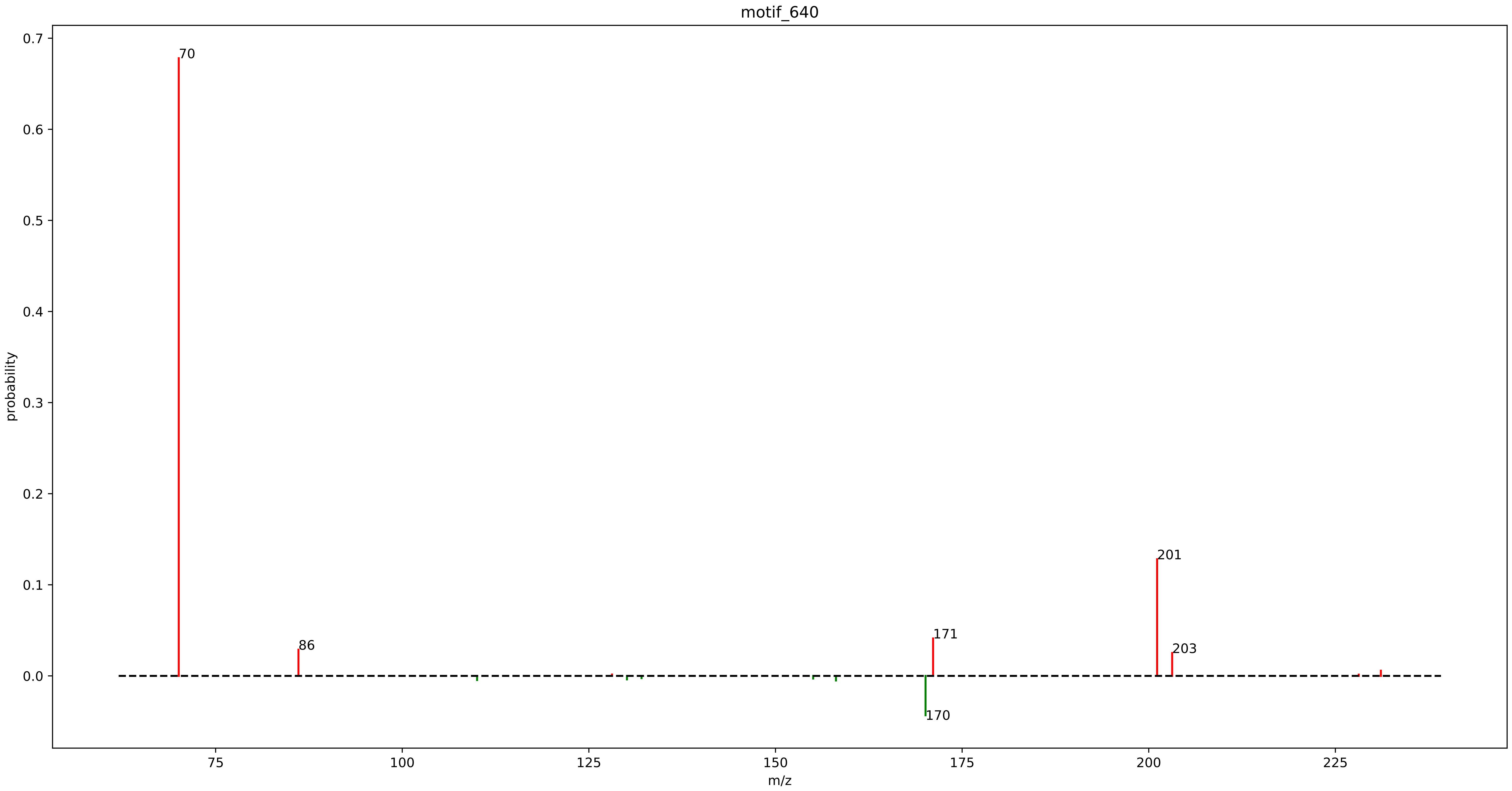
motif_640
probability
m/z
70
86
171
170
201
203
0.7
0.6
0.5
0.4
0.3
0.2
0.1
0.0
75
100
125
150
175
200
225

motif_640

fragment_70.0625	: 0.68
fragment_201.1225	: 0.13
loss_170.1025	: 0.04
fragment_171.1125	: 0.04
fragment_86.0975	: 0.03
fragment_203.1375	: 0.03
fragment_231.0975	: 0.01
loss_158.1025	: 0.01

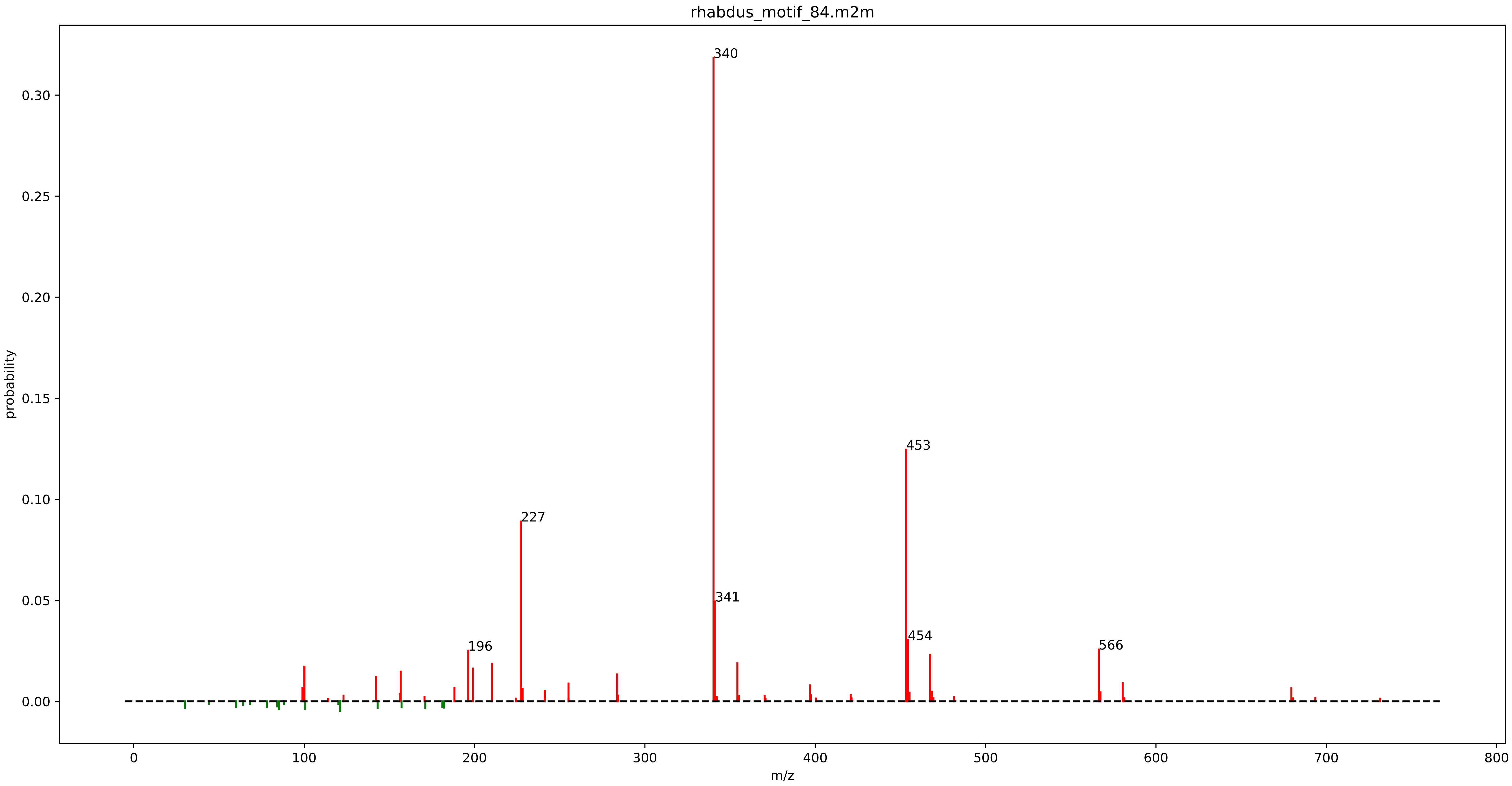

rhabdus_motif_84.m2m
probability
m/z
340
341
453
454
227
196
566
0.00
0.05
0.10
0.15
0.20
0.25
0.30
0
100
200
300
400
500
600
700
800

rhabdus_motif_84.m2m

fragment_340.2575	: 0.32
fragment_453.3425	: 0.12
fragment_227.1775	: 0.09
fragment_341.2625	: 0.05
fragment_454.3475	: 0.03
fragment_566.4275	: 0.03
fragment_196.1325	: 0.03
fragment_467.3575	: 0.02
fragment_354.2725	: 0.02
fragment_210.1475	: 0.02
fragment_100.1125	: 0.02
fragment_199.1775	: 0.02
fragment_156.6325	: 0.01
fragment_227.1725	: 0.01
fragment_283.7175	: 0.01
fragment_142.0875	: 0.01
fragment_580.4425	: 0.01
fragment_255.1675	: 0.01
fragment_396.8025	: 0.01
fragment_188.1775	: 0.01
fragment_679.5125	: 0.01
fragment_98.9825	: 0.01
fragment_228.1775	: 0.01
fragment_241.1925	: 0.01

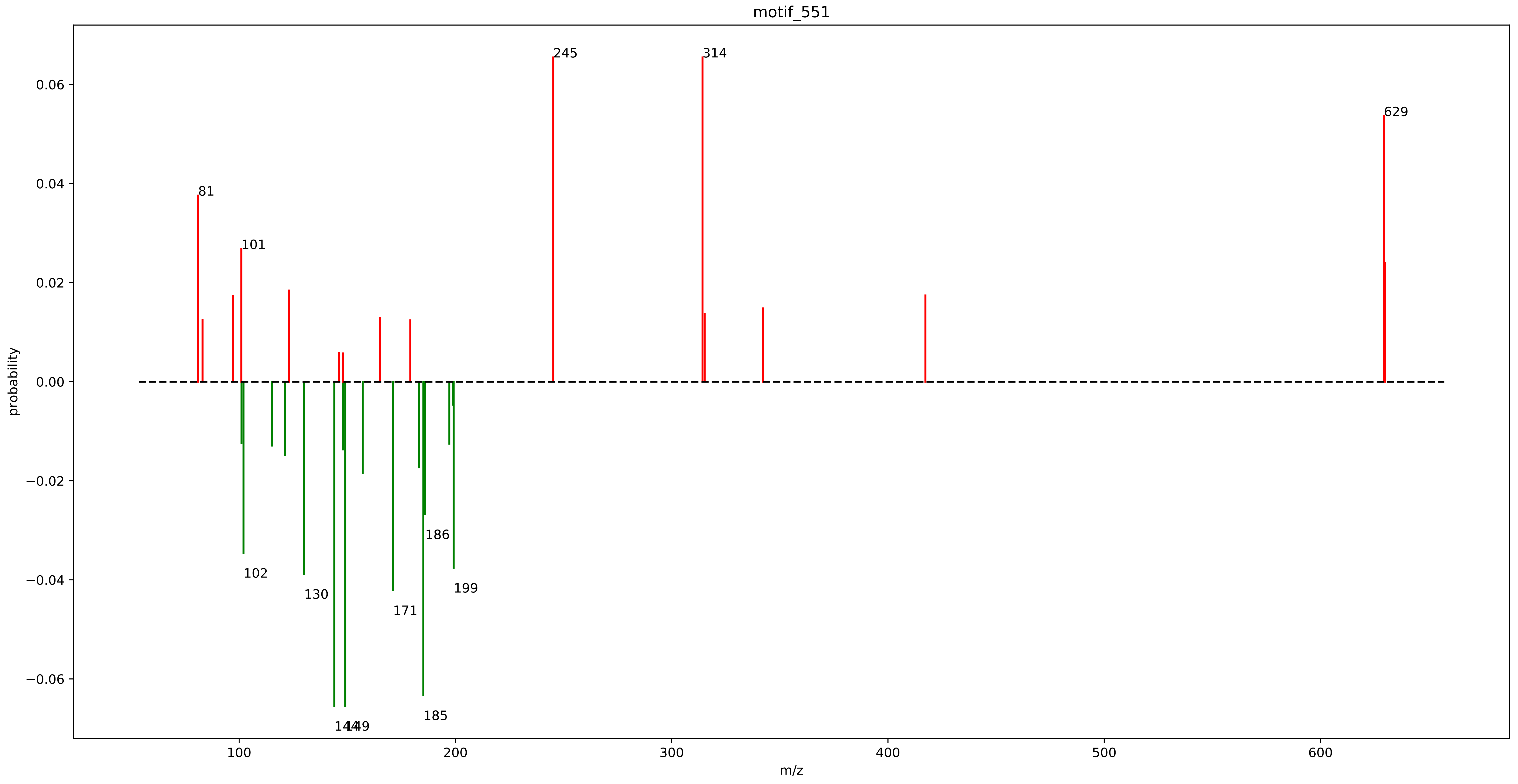

motif_551
probability
m/z
0.06
0.04
0.02
0.00
−0.02
−0.04
−0.06
100
200
300
400
500
600
81
101
245
314
629
102
130
171
186
199
185
144
149

motif_551

loss_149.0675	: 0.07
fragment_314.2475	: 0.07
fragment_245.2275	: 0.07
loss_144.0425	: 0.07
loss_185.1775	: 0.06
fragment_629.2875	: 0.05
loss_171.1575	: 0.04
loss_130.0275	: 0.04
loss_199.1925	: 0.04
fragment_81.0675	: 0.04
loss_102.0275	: 0.03
loss_186.0525	: 0.03
fragment_101.0075	: 0.03
fragment_629.7925	: 0.02
loss_157.1425	: 0.02
fragment_123.1175	: 0.02
fragment_417.3125	: 0.02
loss_183.1575	: 0.02
fragment_97.1025	: 0.02
loss_121.0725	: 0.01
fragment_342.2425	: 0.01
loss_148.0625	: 0.01
fragment_315.2525	: 0.01
loss_115.0975	: 0.01
fragment_165.1625	: 0.01
loss_197.1775	: 0.01
fragment_83.0825	: 0.01
loss_101.0825	: 0.01
fragment_179.1775	: 0.01
fragment_146.0575	: 0.01
fragment_148.0725	: 0.01

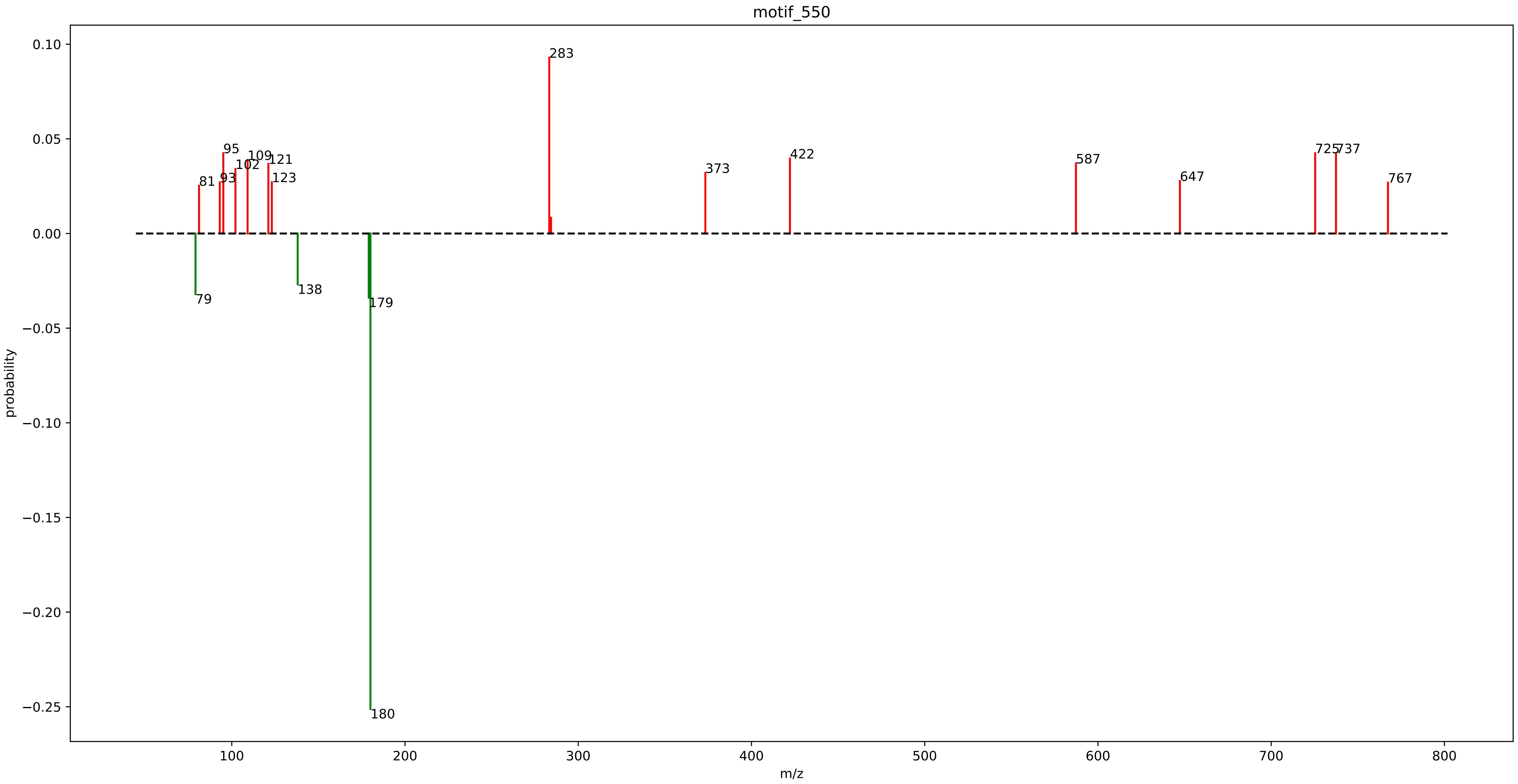

motif_550
probability
m/z
0.10
0.05
0.00
−0.05
−0.10
−0.15
−0.20
−0.25
100
200
300
400
500
600
700
800
283
95
109
121
102
81
93
123
373
422
587
647
725
737
767
79
138
179
180

motif_550

loss_180.0575	: 0.25
fragment_283.2625	: 0.09
fragment_95.0825	: 0.04
fragment_725.3875	: 0.04
fragment_737.3875	: 0.04
fragment_422.1925	: 0.04
fragment_109.0975	: 0.04
fragment_587.2825	: 0.04
fragment_121.1025	: 0.04
fragment_102.0925	: 0.03
loss_179.0575	: 0.03
loss_79.0625	: 0.03
fragment_373.3475	: 0.03
fragment_647.3025	: 0.03
fragment_123.1175	: 0.03
fragment_93.0725	: 0.03
fragment_767.3975	: 0.03
loss_138.0475	: 0.03
fragment_81.0725	: 0.03
fragment_284.2675	: 0.01

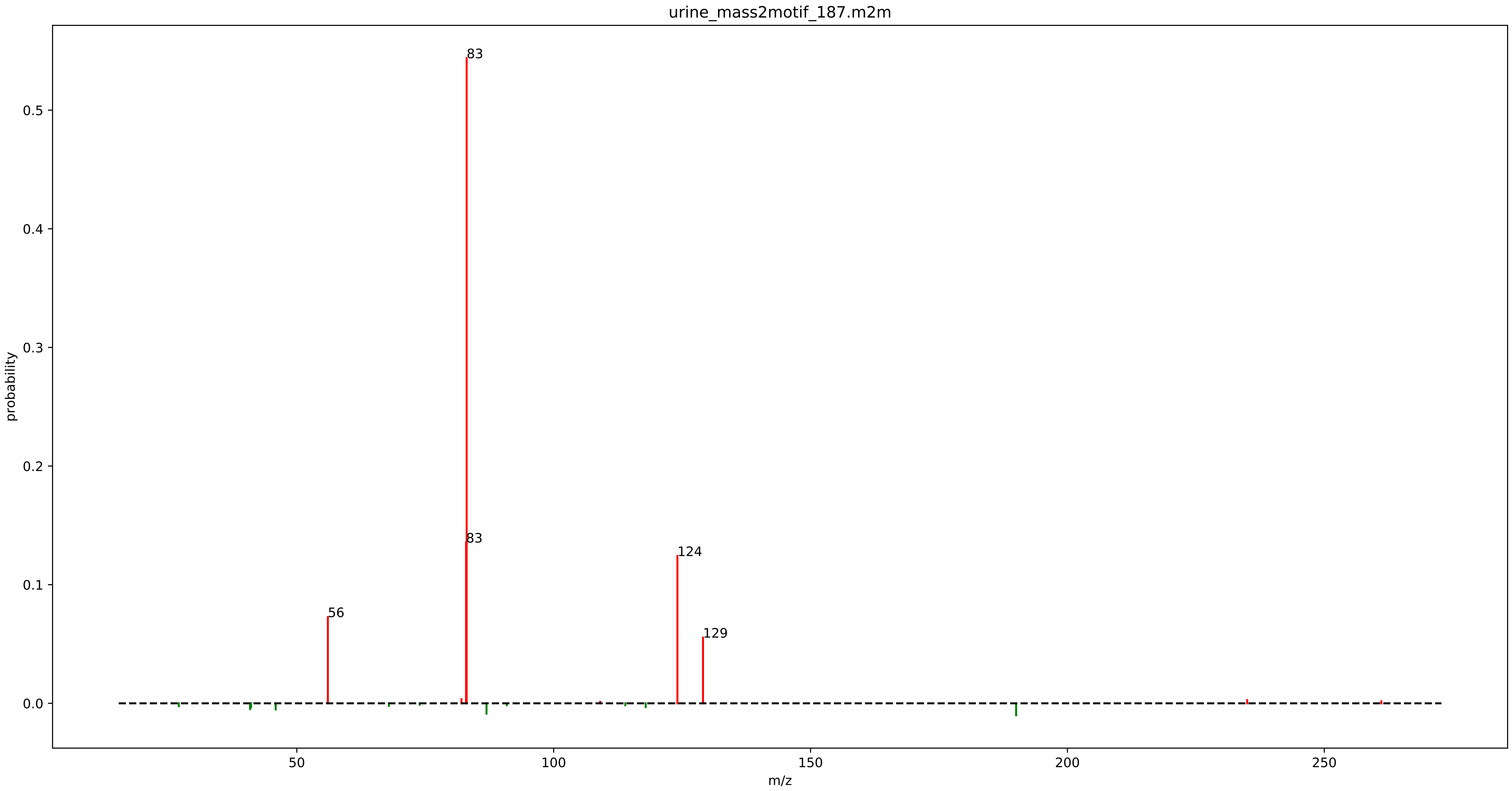
urine_mass2motif_187.m2m
probability
m/z
0.0
0.1
0.2
0.3
0.4
0.5
50
100
150
200
250
83
83
56
124
129

urine_mass2motif_187.m2m

fragment_83.0625	: 0.54
fragment_82.9425	: 0.14
fragment_124.0875	: 0.12
fragment_56.0475	: 0.07
fragment_129.0675	: 0.06
loss_190.0175	: 0.01
loss_86.9275	: 0.01
fragment_83.0225	: 0.01
loss_45.8975	: 0.01

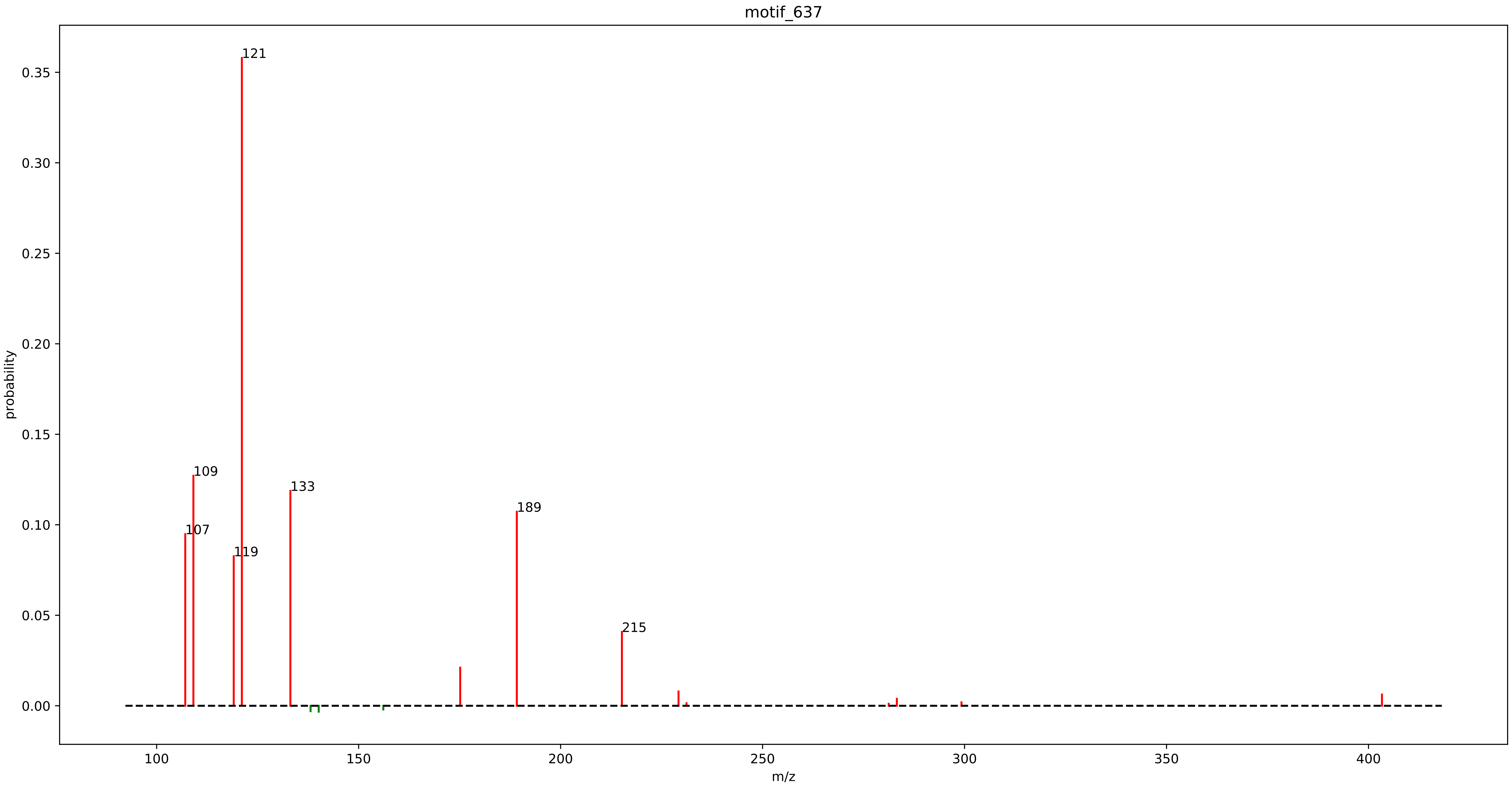

motif_637
probability
m/z
121
109
133
189
107
119
215
0.35
0.30
0.25
0.20
0.15
0.10
0.05
0.00
100
150
200
250
300
350
400

motif_637

fragment_121.0975	: 0.36
fragment_109.0975	: 0.13
fragment_133.1025	: 0.12
fragment_189.1625	: 0.11
fragment_107.0825	: 0.09
fragment_119.0825	: 0.08
fragment_215.1775	: 0.04
fragment_175.1475	: 0.02
fragment_229.1925	: 0.01
fragment_403.3325	: 0.01

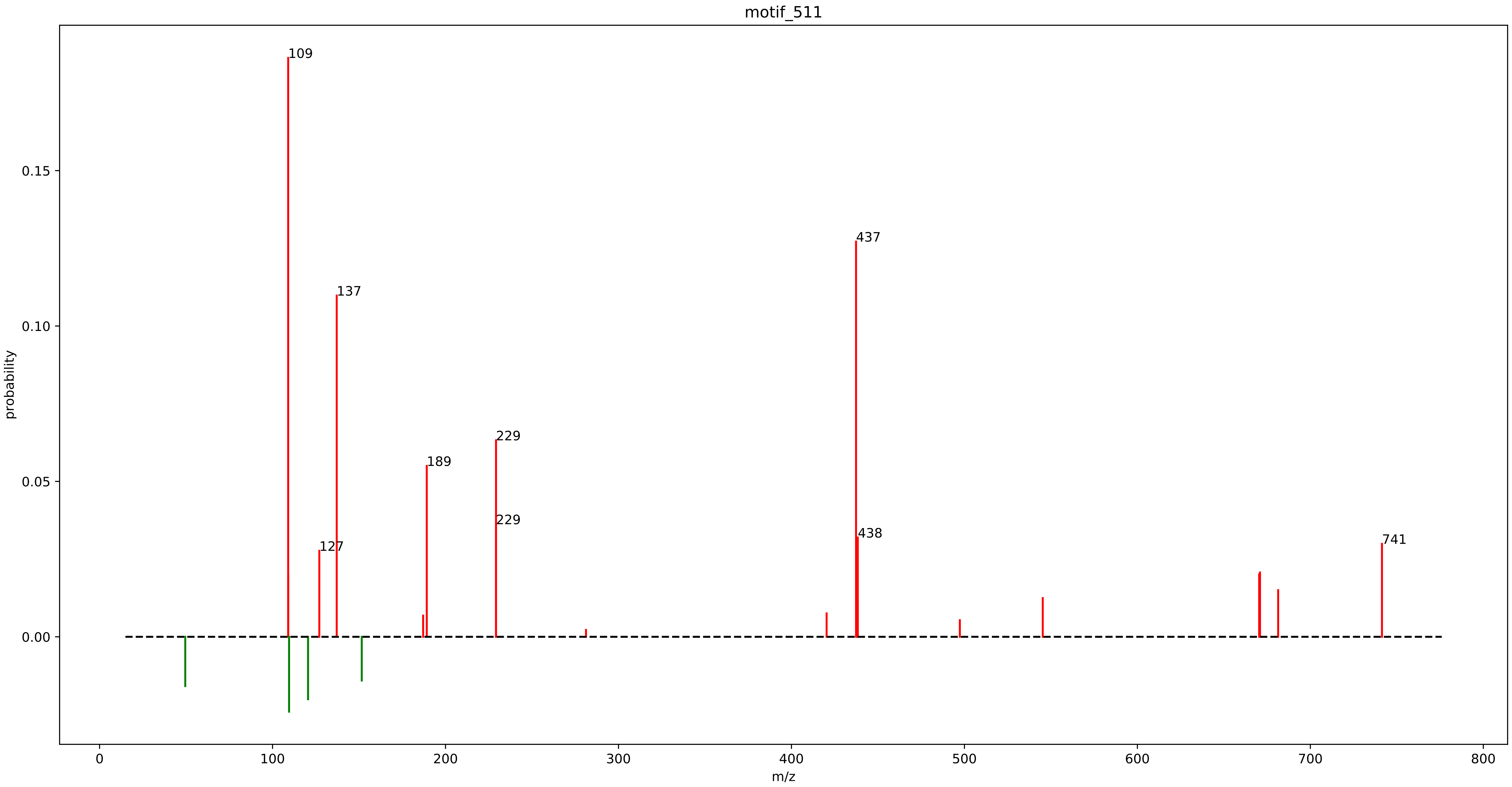

motif_511
probability
m/z
109
137
127
189
229
229
437
438
741
0.15
0.10
0.05
0.00
0
100
200
300
400
500
600
700
800

motif_511

fragment_109.0375	: 0.19
fragment_437.3425	: 0.13
fragment_137.0975	: 0.11
fragment_229.1975	: 0.06
fragment_189.1625	: 0.05
fragment_229.1875	: 0.04
fragment_438.3475	: 0.03
fragment_741.4075	: 0.03
fragment_127.0375	: 0.03
loss_109.5525	: 0.02
fragment_670.9025	: 0.02
fragment_670.4025	: 0.02
loss_120.5375	: 0.02
fragment_109.0725	: 0.02
loss_49.5325	: 0.02
fragment_681.3875	: 0.02
loss_151.5675	: 0.01
fragment_545.2975	: 0.01
fragment_438.3425	: 0.01
fragment_420.3325	: 0.01
fragment_187.0625	: 0.01
fragment_497.3625	: 0.01

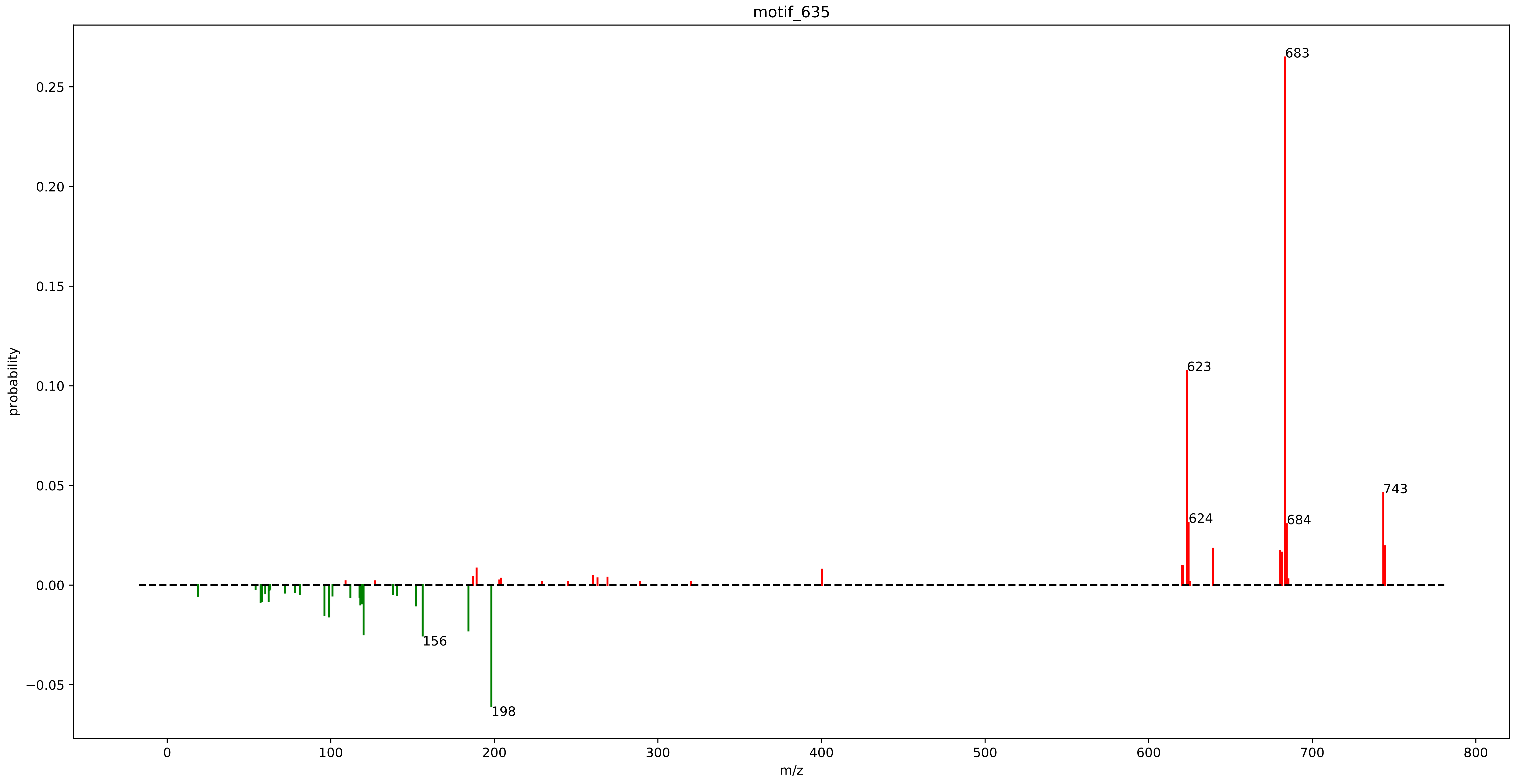

motif_635
probability
m/z
683
623
624
684
743
156
198
0.25
0.20
0.15
0.10
0.05
0.00
−0.05
0
100
200
300
400
500
600
700
800

motif_635

fragment_683.4125	: 0.26
fragment_623.3925	: 0.11
loss_198.1775	: 0.06
fragment_743.4325	: 0.05
fragment_624.3975	: 0.03
fragment_684.4175	: 0.03
loss_156.1625	: 0.03
loss_120.0425	: 0.02
loss_184.1625	: 0.02
fragment_744.4375	: 0.02
fragment_639.3575	: 0.02
fragment_680.4075	: 0.02
fragment_681.4025	: 0.02
loss_99.1175	: 0.02
loss_96.1425	: 0.01
fragment_680.9075	: 0.01
loss_152.0325	: 0.01
fragment_620.3875	: 0.01
loss_118.0875	: 0.01
fragment_620.8875	: 0.01
loss_119.0375	: 0.01
loss_57.0725	: 0.01
fragment_189.1625	: 0.01
loss_62.0325	: 0.01
loss_58.0675	: 0.01
fragment_400.2075	: 0.01
loss_112.0025	: 0.01
loss_117.5875	: 0.01
loss_18.9575	: 0.01
loss_101.0675	: 0.01

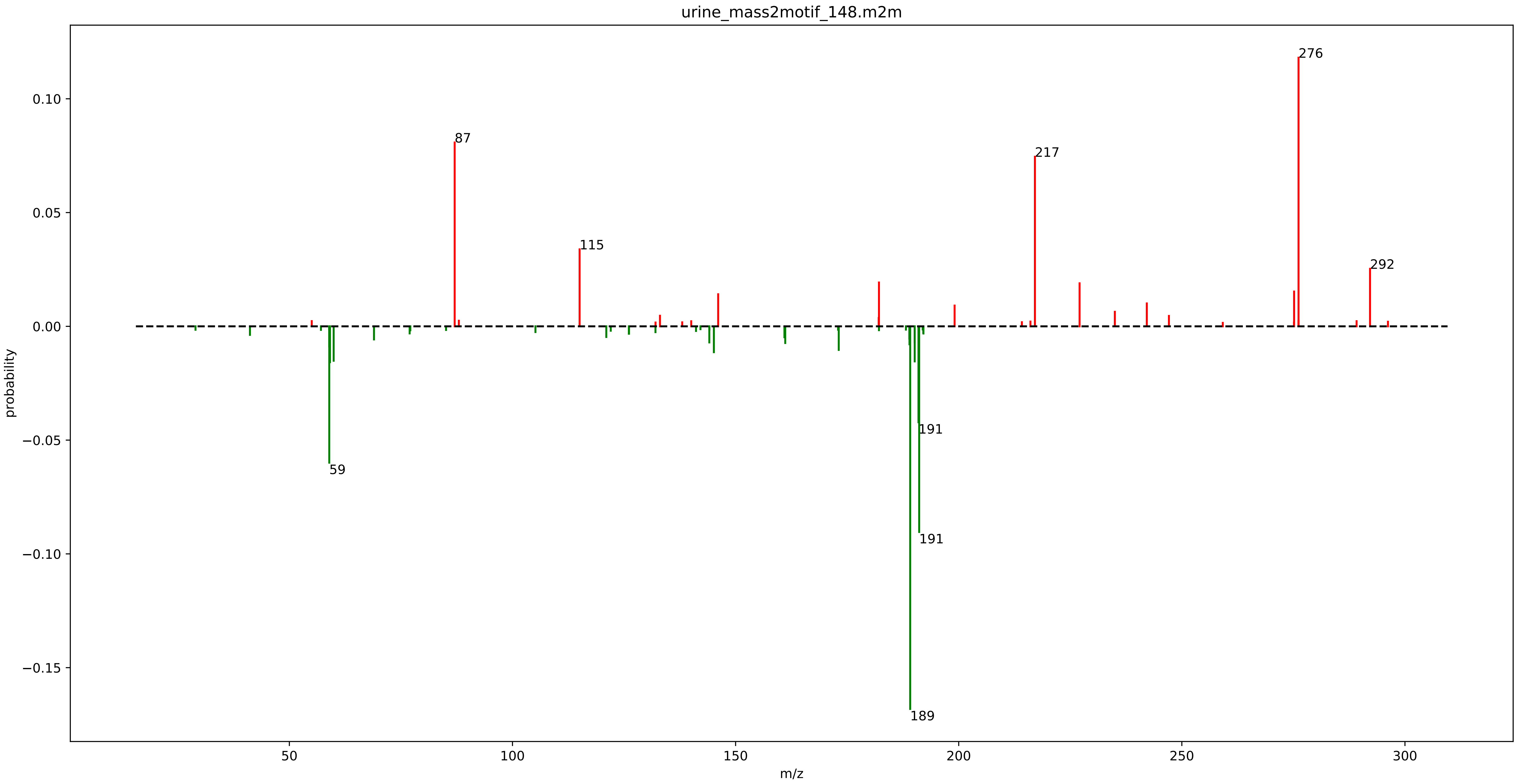

urine_mass2motif_148.m2m
probability
m/z
0.10
0.05
0.00
−0.05
−0.10
−0.15
50
100
150
200
250
300
87
115
217
276
292
59
191
191
189

urine_mass2motif_148.m2m

loss_189.1225	: 0.17
fragment_276.1475	: 0.12
loss_191.1375	: 0.09
fragment_87.0425	: 0.08
fragment_217.0725	: 0.07
loss_58.9425	: 0.06
loss_190.9775	: 0.04
fragment_115.0375	: 0.03
fragment_292.1725	: 0.03
fragment_182.1275	: 0.02
fragment_227.0825	: 0.02
loss_59.0975	: 0.02
loss_190.1325	: 0.02
fragment_275.1575	: 0.02
loss_59.9275	: 0.02
fragment_146.0925	: 0.01
loss_145.1275	: 0.01
loss_173.1125	: 0.01
fragment_242.1475	: 0.01
fragment_199.0775	: 0.01
loss_58.9375	: 0.01
loss_188.9625	: 0.01
loss_161.1225	: 0.01
loss_144.1125	: 0.01
loss_190.1225	: 0.01
fragment_234.9825	: 0.01
loss_68.9625	: 0.01
loss_188.9375	: 0.01

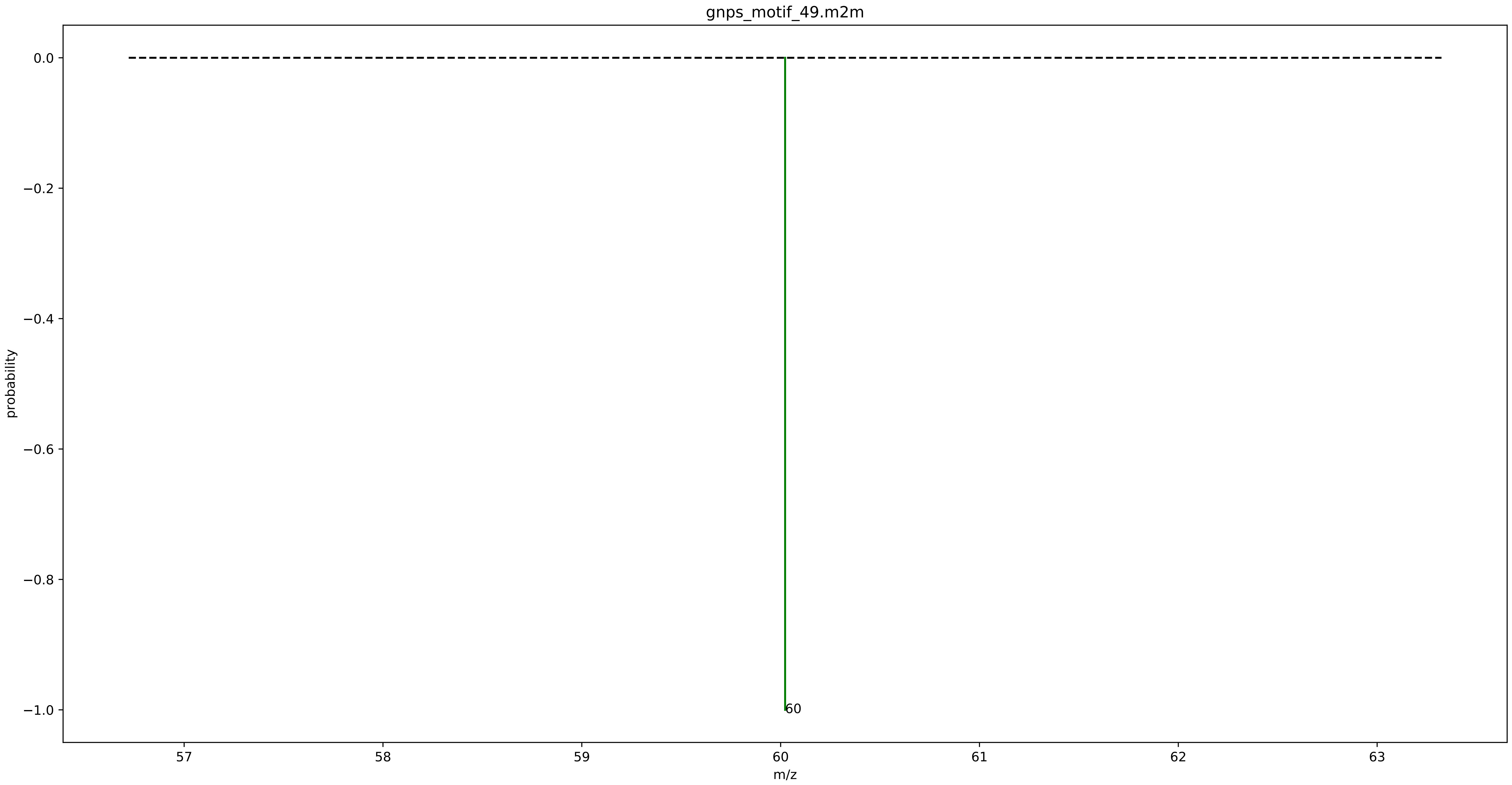
gnps_motif_49.m2m
0.0
−0.2
−0.4
−0.6
−0.8
−1.0
probability
60
57
58
59
60
61
62
63
m/z

gnps_motif_49.m2m

loss_60.0225 : 1.00

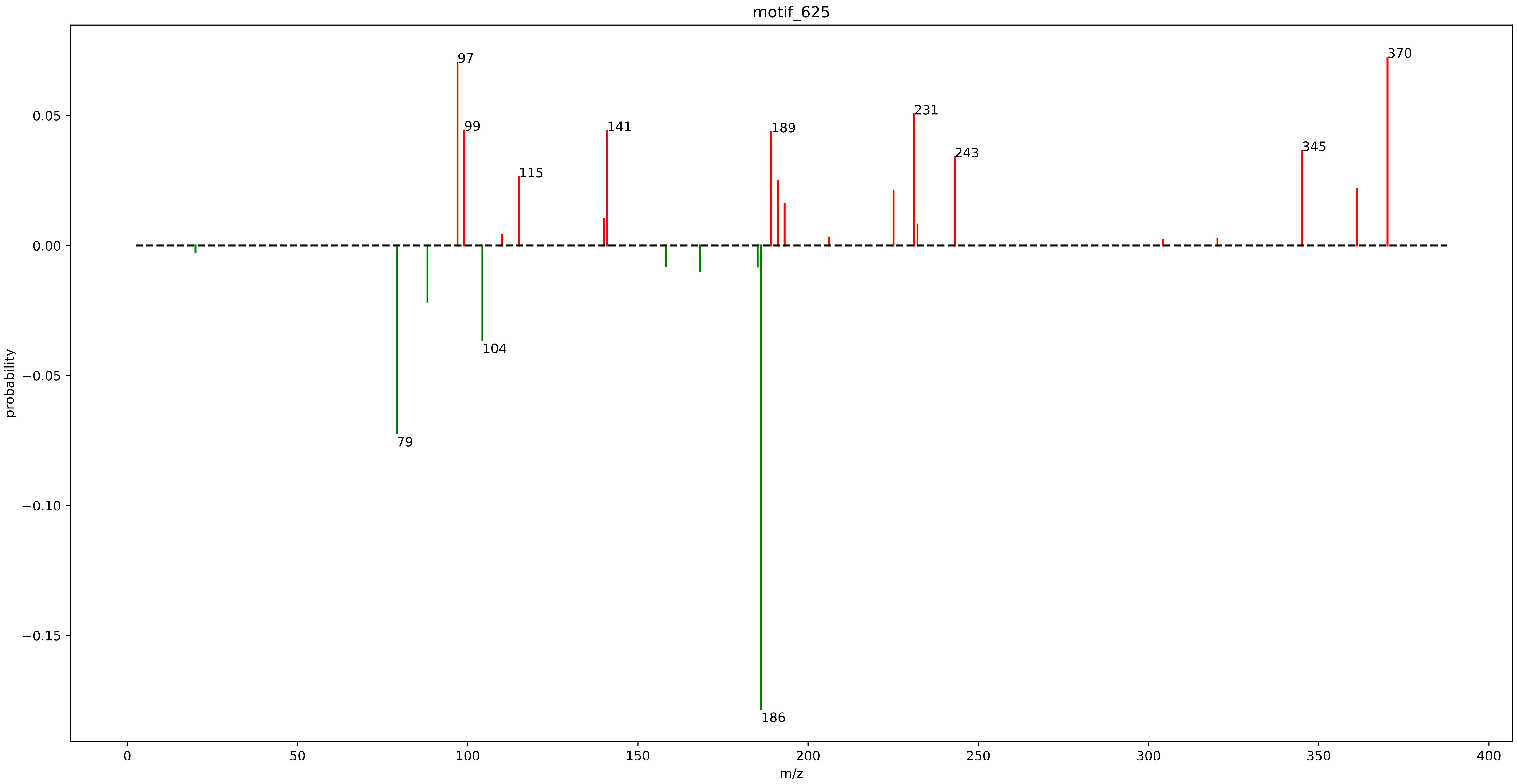

motif_625
0.05
0.00
−0.05
−0.10
−0.15
probability
0
50
100
150
200
250
300
350
400
m/z
97
99
115
141
189
231
243
345
370
79
104
186

motif_625

loss_186.1975	: 0.18
loss_79.1575	: 0.07
fragment_370.1875	: 0.07
fragment_97.0275	: 0.07
fragment_231.1125	: 0.05
fragment_98.9725	: 0.04
fragment_140.9875	: 0.04
fragment_189.1625	: 0.04
fragment_345.0475	: 0.04
loss_104.2975	: 0.04
fragment_243.0175	: 0.03
fragment_115.0375	: 0.03
fragment_191.0875	: 0.02
fragment_361.1825	: 0.02
loss_88.1625	: 0.02
fragment_225.0975	: 0.02
fragment_193.0975	: 0.02
fragment_140.0625	: 0.01
loss_168.1875	: 0.01
loss_185.1925	: 0.01
fragment_232.1175	: 0.01
loss_158.1675	: 0.01

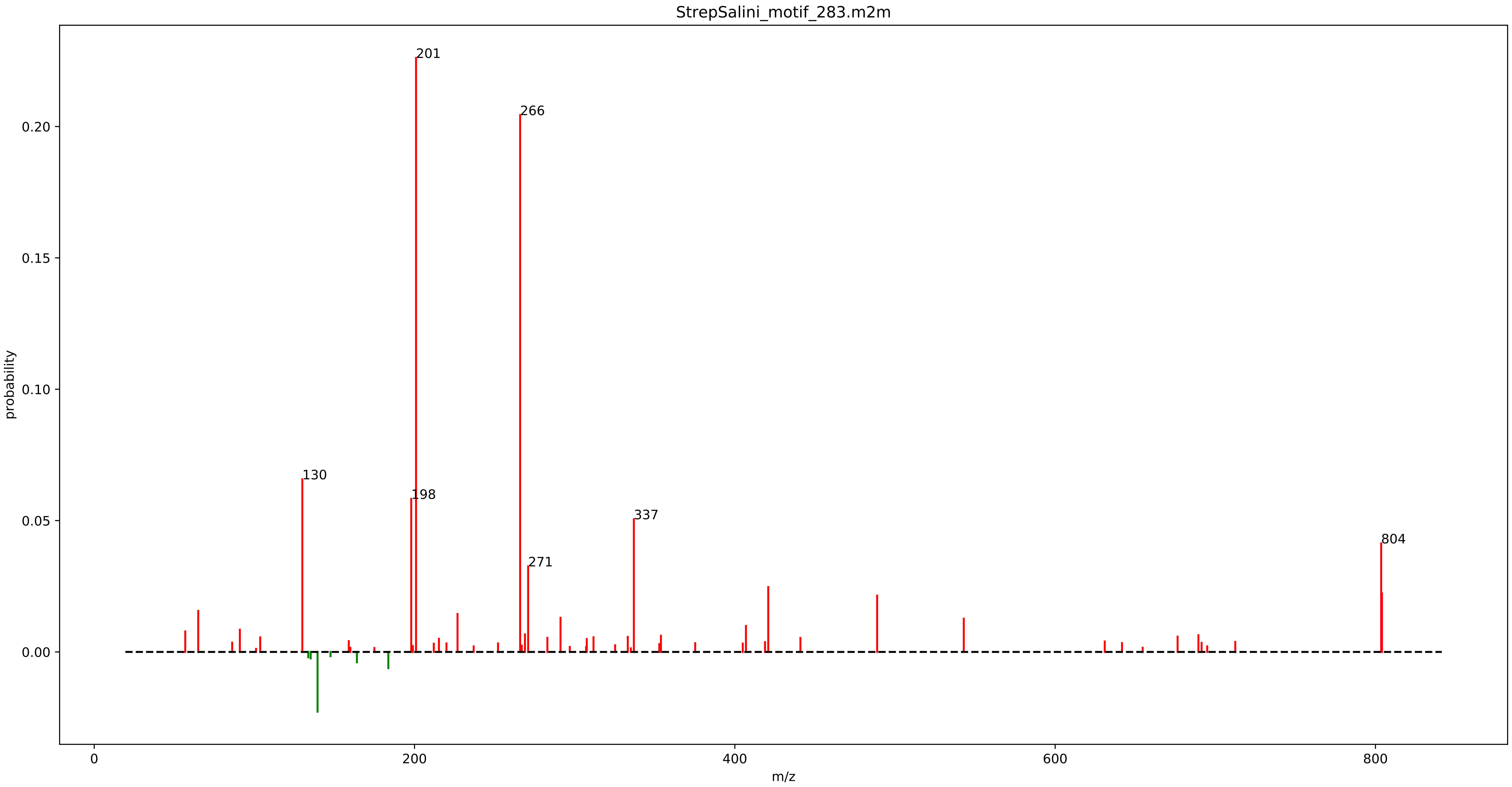

StrepSalini_motif_283.m2m
probability
m/z
0.20
0.15
0.10
0.05
0.00
0
200
400
600
800
201
266
130
198
337
271
804

StrepSalini_motif_283.m2m

fragment_200.9500	: 0.23
fragment_265.9500	: 0.20
fragment_129.9500	: 0.07
fragment_197.9500	: 0.06
fragment_336.9500	: 0.05
fragment_803.5500	: 0.04
fragment_270.9500	: 0.03
fragment_420.8500	: 0.02
loss_139.4500	: 0.02
fragment_804.0500	: 0.02
fragment_488.8500	: 0.02
fragment_64.9500	: 0.02
fragment_226.8500	: 0.01
fragment_291.1500	: 0.01
fragment_542.9500	: 0.01
fragment_406.9500	: 0.01
fragment_90.9500	: 0.01
fragment_56.8500	: 0.01
fragment_268.9500	: 0.01
fragment_689.4500	: 0.01
fragment_353.8500	: 0.01
loss_183.6500	: 0.01
fragment_676.4500	: 0.01
fragment_333.1500	: 0.01
fragment_311.7500	: 0.01
fragment_103.6500	: 0.01
fragment_282.9500	: 0.01
fragment_440.9500	: 0.01

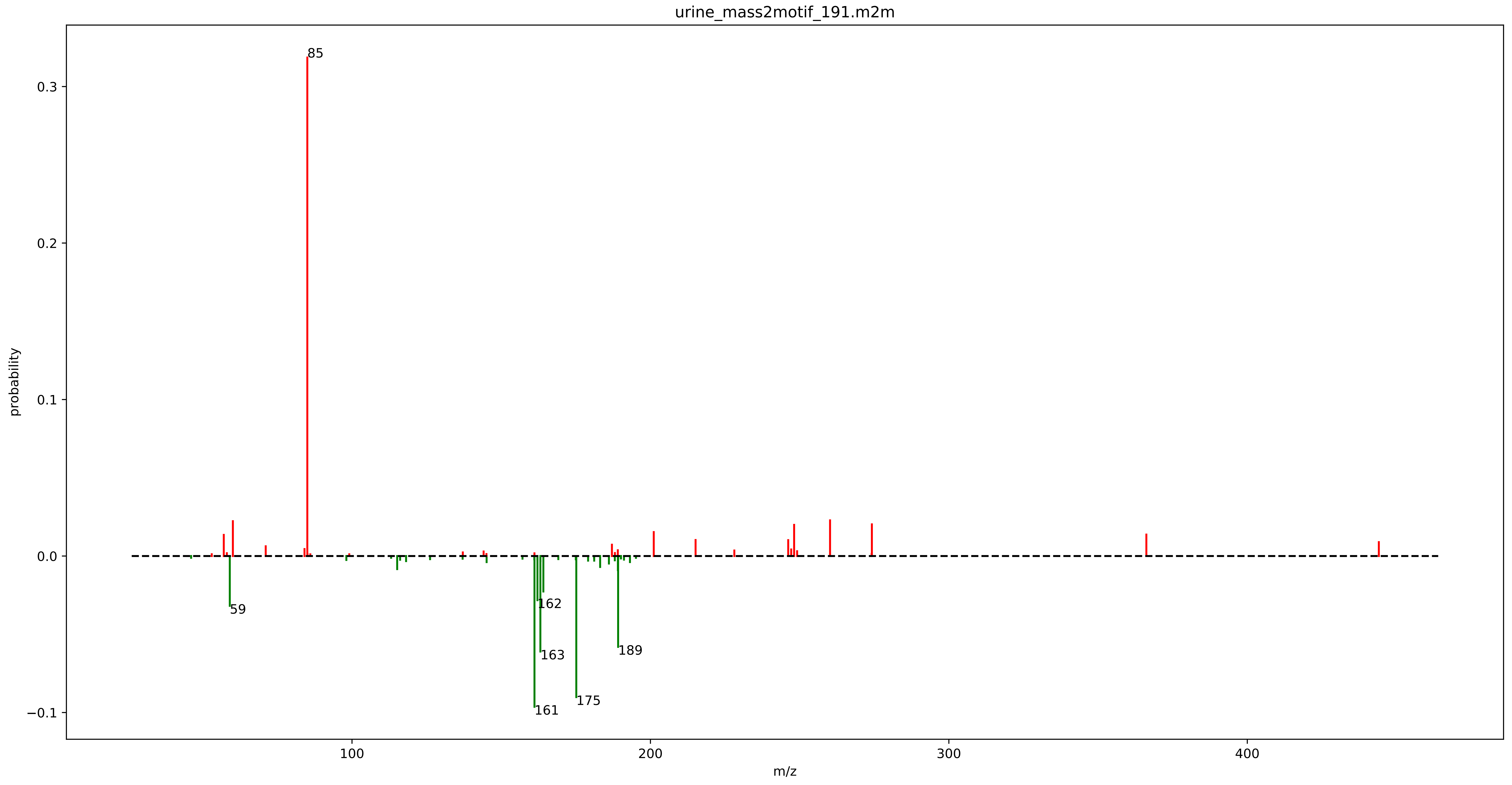

urine_mass2motif_191.m2m
probability
m/z
0.3
0.2
0.1
0.0
−0.1
100
200
300
400
85
59
162
163
161
175
189

urine_mass2motif_191.m2m

fragment_85.0275	: 0.32
loss_161.1425	: 0.10
loss_175.1575	: 0.09
loss_163.1225	: 0.06
loss_189.1725	: 0.06
loss_59.0475	: 0.03
loss_162.1425	: 0.03
fragment_260.1875	: 0.02
loss_164.1225	: 0.02
fragment_60.0825	: 0.02
fragment_274.2025	: 0.02
fragment_248.1475	: 0.02
fragment_201.1125	: 0.02
fragment_366.1775	: 0.01
fragment_57.0325	: 0.01
fragment_215.1275	: 0.01
fragment_246.1675	: 0.01
loss_189.0925	: 0.01
fragment_444.0775	: 0.01
loss_115.1375	: 0.01
fragment_187.0975	: 0.01
loss_183.1475	: 0.01
fragment_71.0875	: 0.01
loss_189.1225	: 0.01

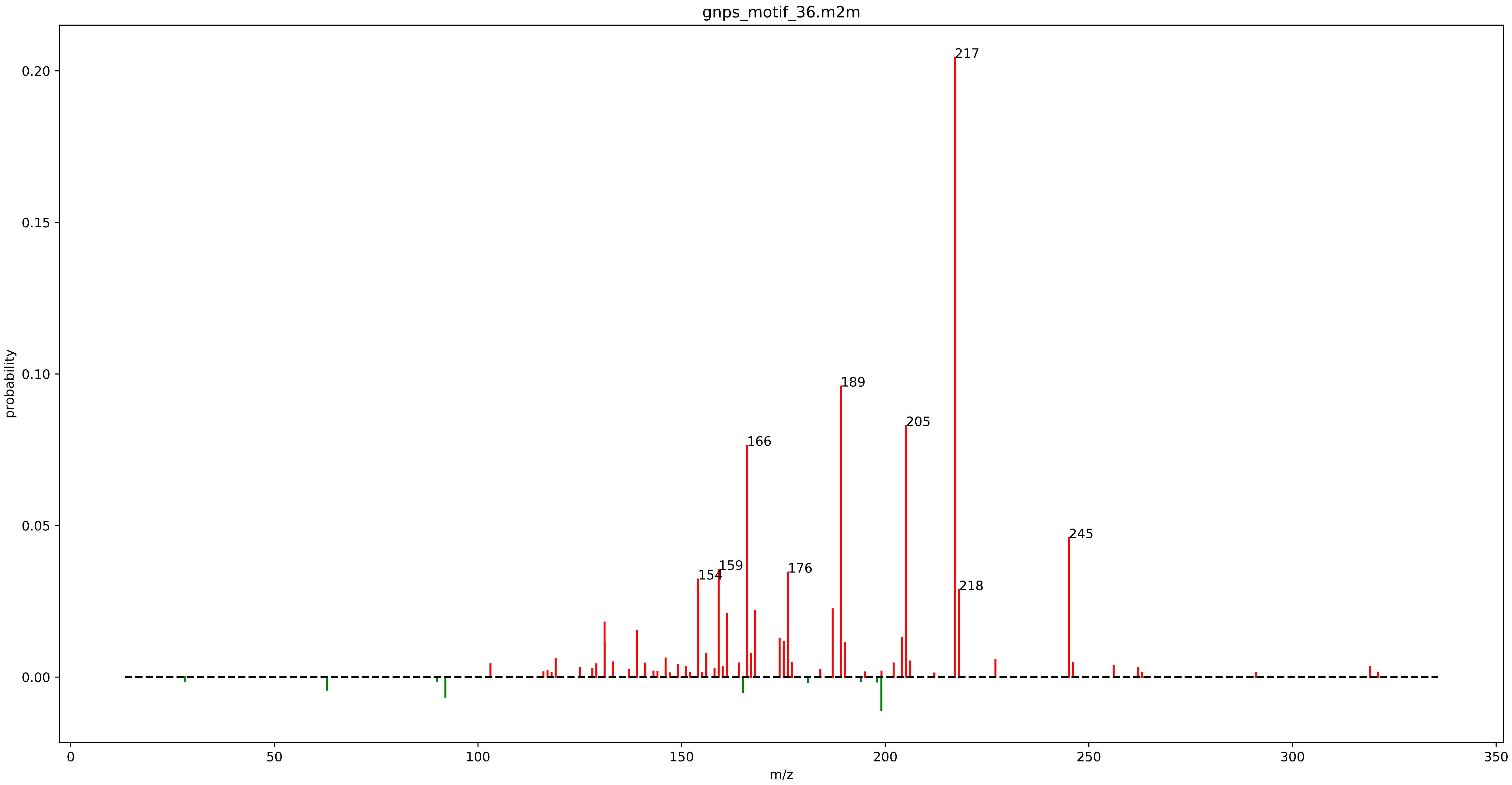

gnps_motif_36.m2m
probability
m/z
0.20
0.15
0.10
0.05
0.00
0
50
100
150
200
250
300
350
217
189
205
166
245
154
159
176
218

gnps_motif_36.m2m

fragment_217.0875	: 0.20
fragment_189.0925	: 0.10
fragment_205.0875	: 0.08
fragment_166.0425	: 0.08
fragment_245.0825	: 0.05
fragment_159.0825	: 0.04
fragment_176.0825	: 0.03
fragment_154.0425	: 0.03
fragment_218.0875	: 0.03
fragment_187.0775	: 0.02
fragment_168.0375	: 0.02
fragment_161.0975	: 0.02
fragment_131.0725	: 0.02
fragment_161.0575	: 0.02
fragment_139.0325	: 0.02
fragment_204.0775	: 0.01
fragment_174.0675	: 0.01
fragment_204.0825	: 0.01
fragment_175.0775	: 0.01
fragment_190.0925	: 0.01
fragment_131.0875	: 0.01
loss_199.0425	: 0.01
fragment_167.0475	: 0.01
fragment_156.0375	: 0.01
loss_91.9925	: 0.01
fragment_146.0725	: 0.01
fragment_119.0725	: 0.01
fragment_227.0475	: 0.01
fragment_206.0875	: 0.01

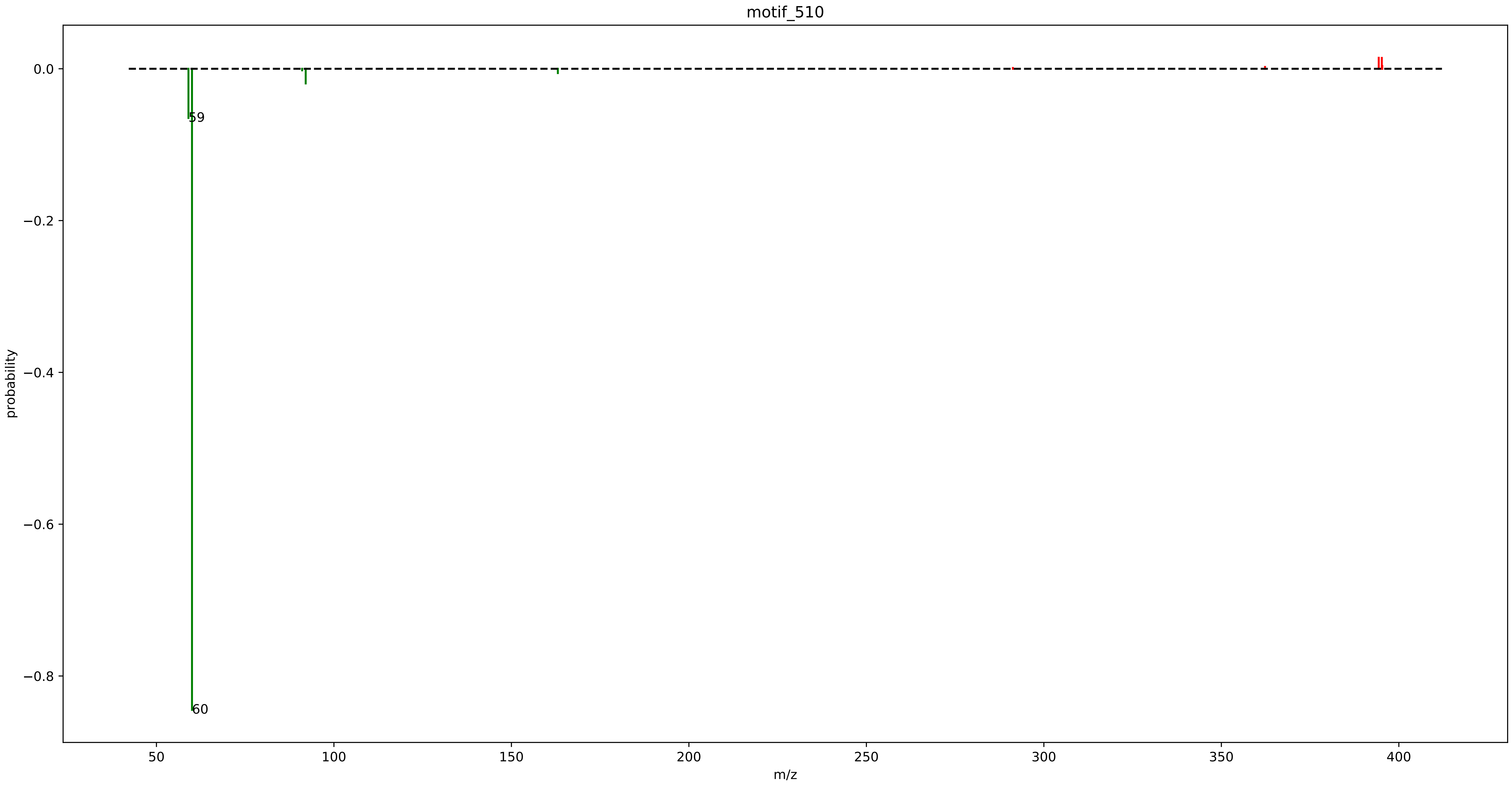

motif_510
0.0
−0.2
−0.4
−0.6
−0.8
probability
59
60
50
100
150
200
250
300
350
400
m/z

motif_510

loss_60.0175	: 0.84
loss_59.0175	: 0.06
loss_92.0475	: 0.02
fragment_394.3325	: 0.01
fragment_395.2025	: 0.01
loss_163.0825	: 0.01

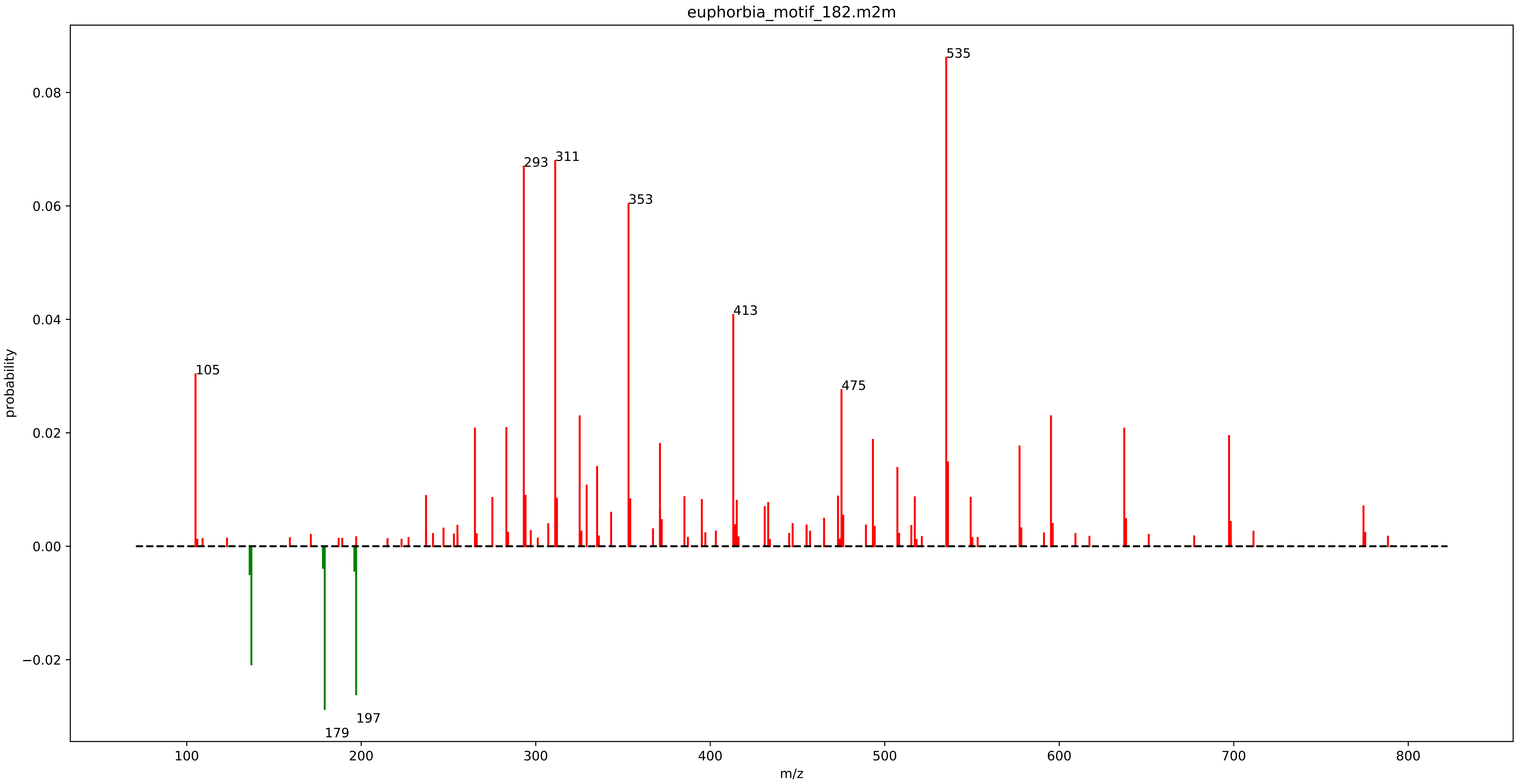

euphorbia_motif_182.m2m
probability
m/z
0.08
0.06
0.04
0.02
0.00
−0.02
100
200
300
400
500
600
700
800
105
293
311
353
413
475
535
179
197

euphorbia_motif_182.m2m

fragment_535.2325	: 0.09	fragment_335.1625	: 0.01
fragment_311.1625	: 0.07	fragment_507.2375	: 0.01
fragment_293.1525	: 0.07	fragment_329.1725	: 0.01
fragment_353.1725	: 0.06	fragment_294.1575	: 0.01
fragment_413.1975	: 0.04	fragment_237.1275	: 0.01
fragment_105.0325	: 0.03	fragment_473.2175	: 0.01
loss_179.0775	: 0.03	fragment_385.1975	: 0.01
fragment_475.2125	: 0.03	fragment_517.2225	: 0.01
loss_197.0875	: 0.03	fragment_549.2475	: 0.01
fragment_595.2525	: 0.02	fragment_275.1425	: 0.01
fragment_325.1775	: 0.02	fragment_312.1675	: 0.01
fragment_283.1675	: 0.02	fragment_354.1775	: 0.01
loss_137.0675	: 0.02	fragment_395.1875	: 0.01
fragment_265.1575	: 0.02	fragment_415.1925	: 0.01
fragment_637.2625	: 0.02	fragment_433.2025	: 0.01
fragment_697.2875	: 0.02	fragment_774.3325	: 0.01
fragment_493.2225	: 0.02	fragment_431.2075	: 0.01
fragment_371.1825	: 0.02	fragment_371.1875	: 0.01
fragment_577.2425	: 0.02	fragment_343.1925	: 0.01
fragment_536.2375	: 0.01	fragment_476.2175	: 0.01

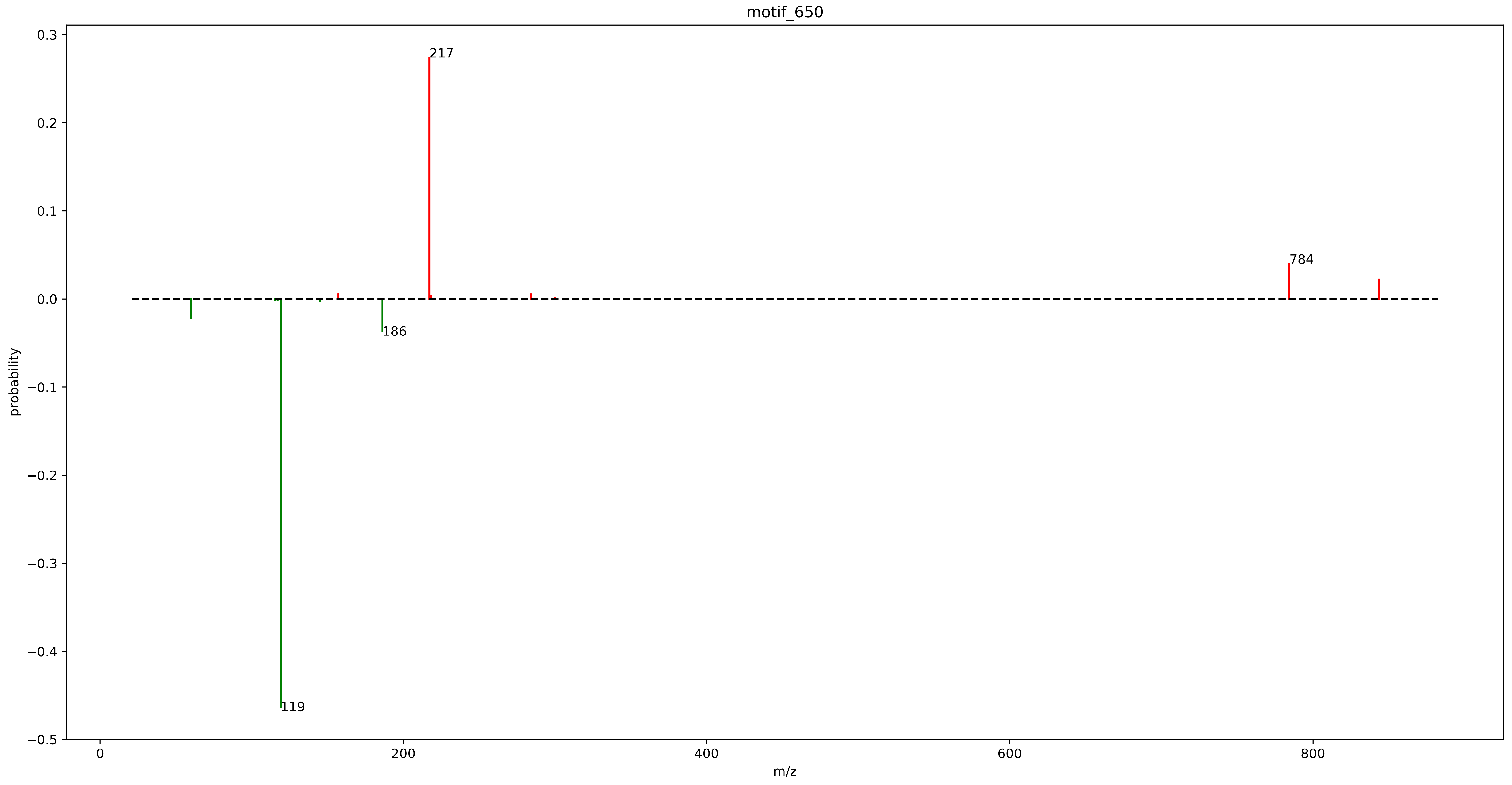

motif_650
probability
m/z
0.3
0.2
0.1
0.0
−0.1
−0.2
−0.3
−0.4
−0.5
0
200
400
600
800
217
784
186
119

motif_650

loss_119.0325	: 0.46
fragment_217.1525	: 0.27
fragment_784.4225	: 0.04
loss_186.1325	: 0.04
loss_60.0125	: 0.02
fragment_843.4375	: 0.02
fragment_157.1125	: 0.01
fragment_284.1975	: 0.01

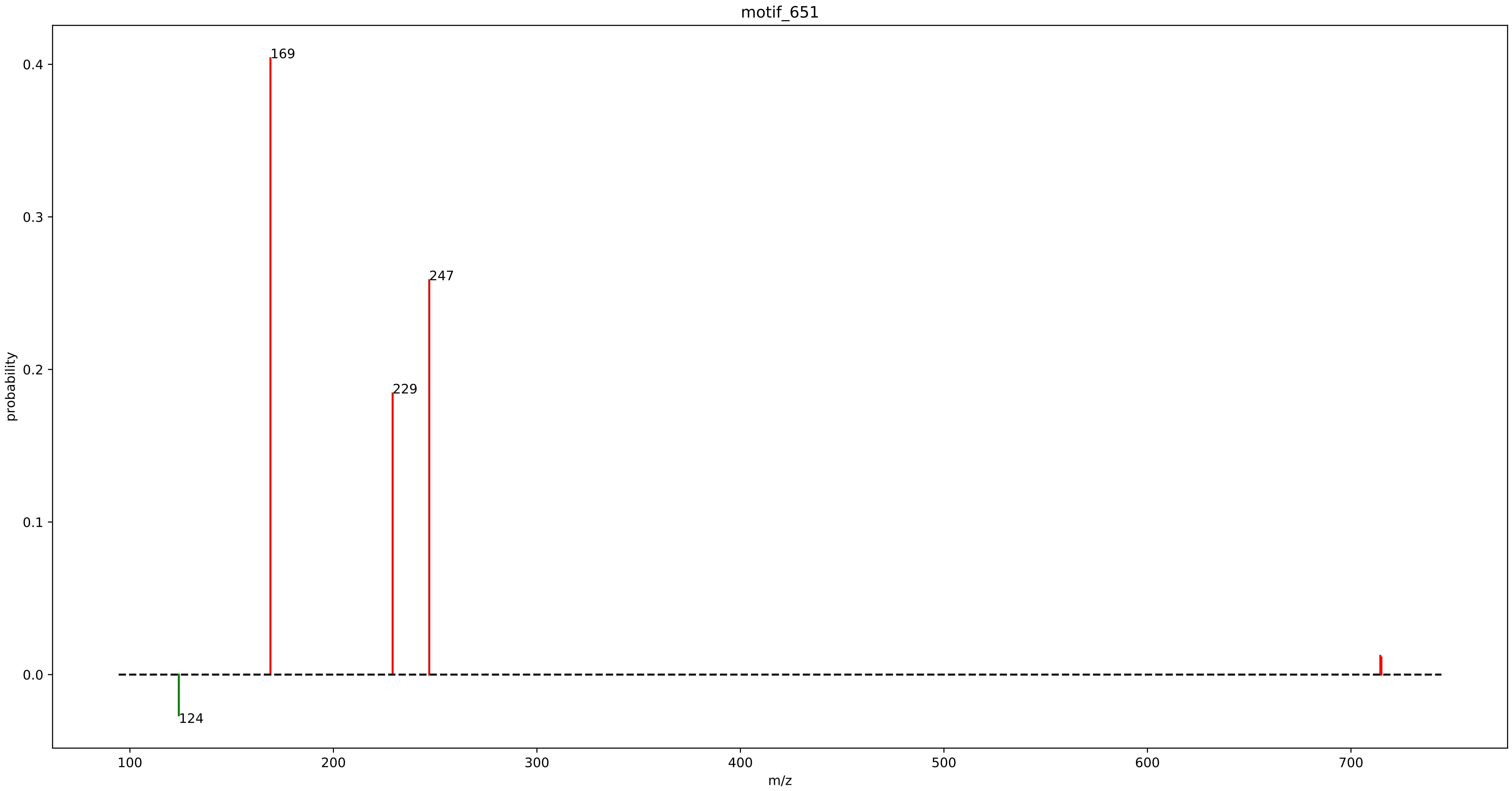
motif_651
169
247
229
124
probability
0.4
0.3
0.2
0.1
0.0
100
200
300
400
500
600
700
m/z

motif_651

fragment_169.0475	: 0.40
fragment_247.0825	: 0.26
fragment_229.0975	: 0.18
loss_124.0325	: 0.03
fragment_714.4275	: 0.01
fragment_714.9275	: 0.01

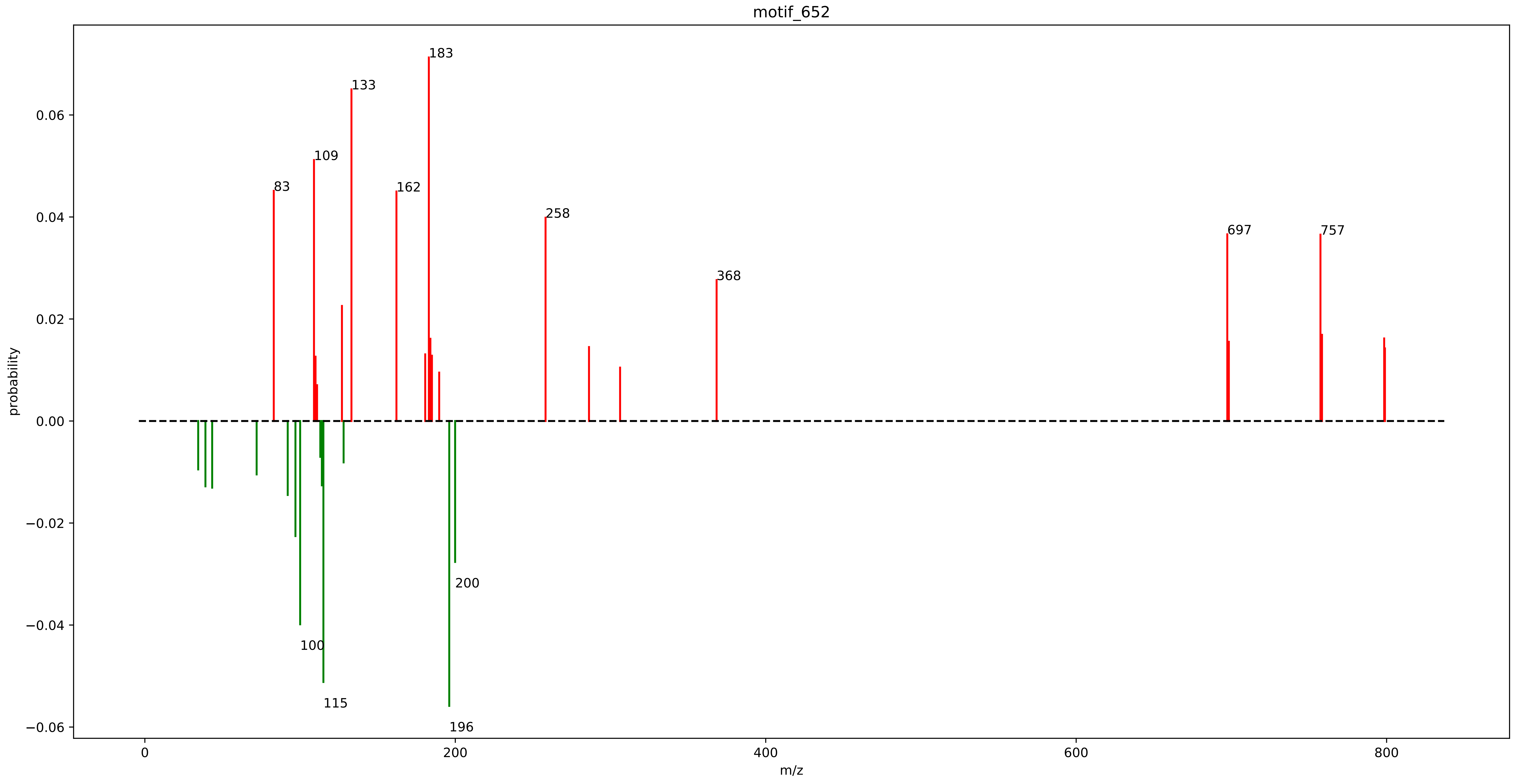

motif_652
probability
m/z
0.06
0.04
0.02
0.00
−0.02
−0.04
−0.06
0
200
400
600
800
83
109
133
162
183
258
368
697
757
100
115
196
200

motif_652

fragment_182.9625	: 0.07
fragment_133.1025	: 0.07
loss_196.1075	: 0.06
fragment_108.9625	: 0.05
loss_115.0275	: 0.05
fragment_83.0475	: 0.05
fragment_162.0875	: 0.04
fragment_258.1475	: 0.04
loss_100.0525	: 0.04
fragment_697.3625	: 0.04
fragment_757.3875	: 0.04
fragment_368.3525	: 0.03
loss_199.8925	: 0.03
fragment_126.9725	: 0.02
loss_97.0175	: 0.02
fragment_758.3875	: 0.02
fragment_798.4325	: 0.02
fragment_183.9625	: 0.02
fragment_698.3675	: 0.02
loss_92.0425	: 0.01
fragment_286.1425	: 0.01
fragment_798.9325	: 0.01
loss_43.3675	: 0.01
fragment_180.6225	: 0.01
loss_39.0275	: 0.01
fragment_184.9625	: 0.01
fragment_109.9625	: 0.01
loss_114.0275	: 0.01
loss_72.0175	: 0.01
fragment_306.1725	: 0.01
loss_34.3625	: 0.01
fragment_189.6275	: 0.01
loss_128.0475	: 0.01
loss_113.0275	: 0.01
fragment_110.9575	: 0.01

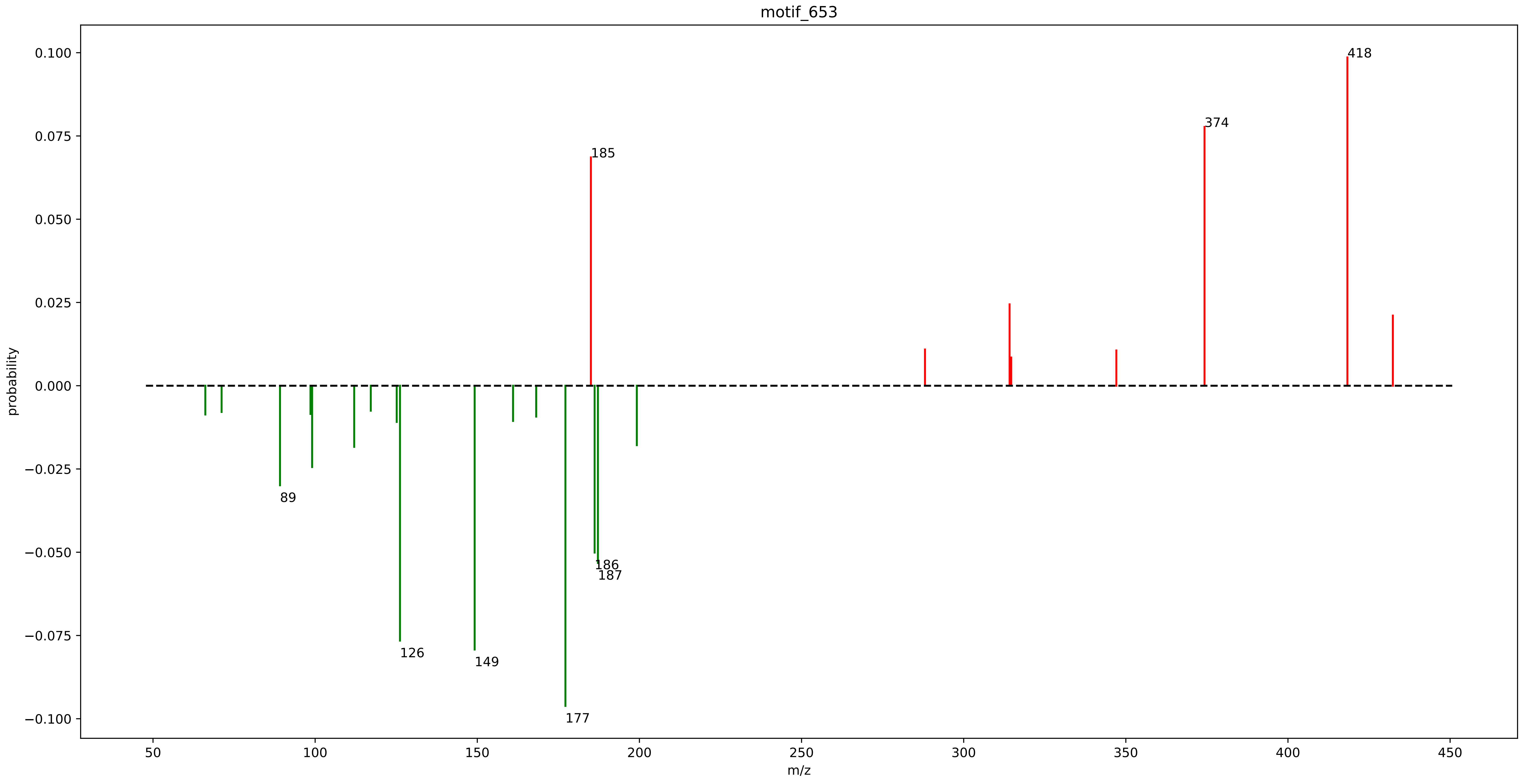

motif_653
0.100
0.075
0.050
0.025
0.000
−0.025
−0.050
−0.075
−0.100
probability
50
100
150
200
250
300
350
400
450
m/z
418
374
185
89
126
149
177
186
187

motif_653

fragment_418.3275	: 0.10
loss_177.1825	: 0.10
loss_149.1875	: 0.08
fragment_374.2675	: 0.08
loss_126.1575	: 0.08
fragment_185.0375	: 0.07
loss_187.1975	: 0.05
loss_186.1775	: 0.05
loss_89.1625	: 0.03
loss_99.0625	: 0.02
fragment_314.1525	: 0.02
fragment_432.3475	: 0.02
loss_112.0425	: 0.02
loss_199.1975	: 0.02
loss_125.1525	: 0.01
fragment_288.0625	: 0.01
fragment_347.0825	: 0.01
loss_161.0275	: 0.01
loss_168.1625	: 0.01
loss_66.1325	: 0.01
loss_98.5625	: 0.01
fragment_314.6525	: 0.01
loss_71.1525	: 0.01
loss_117.1575	: 0.01

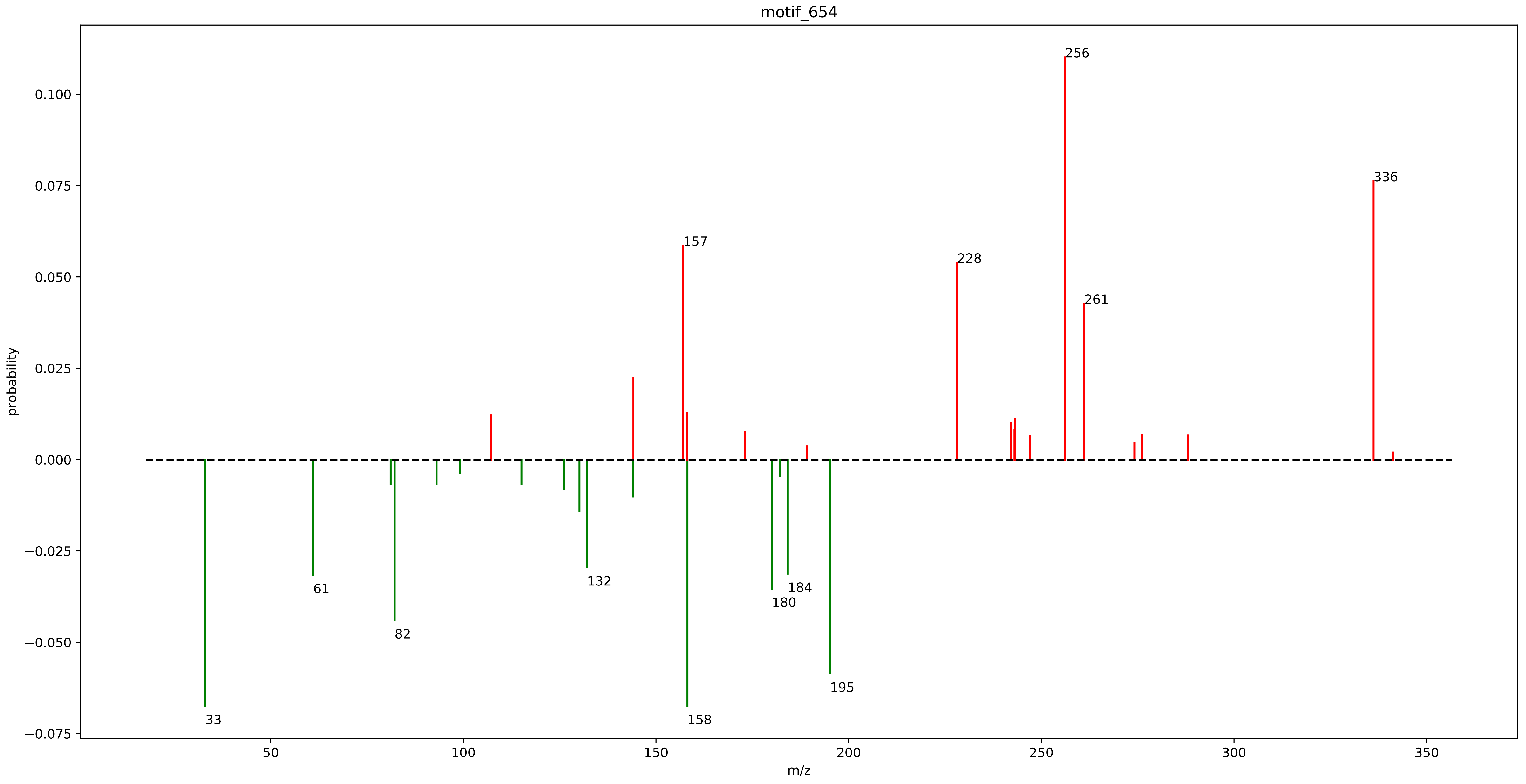

motif_654
probability
m/z
0.100
0.075
0.050
0.025
0.000
−0.025
−0.050
−0.075
50
100
150
200
250
300
350
256
336
157
228
261
61
132
184
180
82
195
33
158

motif_654

fragment_256.1425	: 0.11
fragment_336.1975	: 0.08
loss_33.0075	: 0.07
loss_158.1075	: 0.07
loss_195.1325	: 0.06
fragment_157.0725	: 0.06
fragment_228.1475	: 0.05
loss_82.1275	: 0.04
fragment_261.1425	: 0.04
loss_180.0275	: 0.04
loss_61.0025	: 0.03
loss_184.1575	: 0.03
loss_132.0775	: 0.03
fragment_144.0725	: 0.02
loss_130.1125	: 0.01
fragment_158.0575	: 0.01
fragment_107.0825	: 0.01
fragment_243.1525	: 0.01
loss_144.0525	: 0.01
fragment_242.1625	: 0.01
loss_126.1725	: 0.01
fragment_243.0175	: 0.01
fragment_173.0725	: 0.01
loss_93.0225	: 0.01
fragment_276.1625	: 0.01
loss_115.0975	: 0.01
fragment_288.0975	: 0.01
loss_81.0925	: 0.01
fragment_247.1275	: 0.01

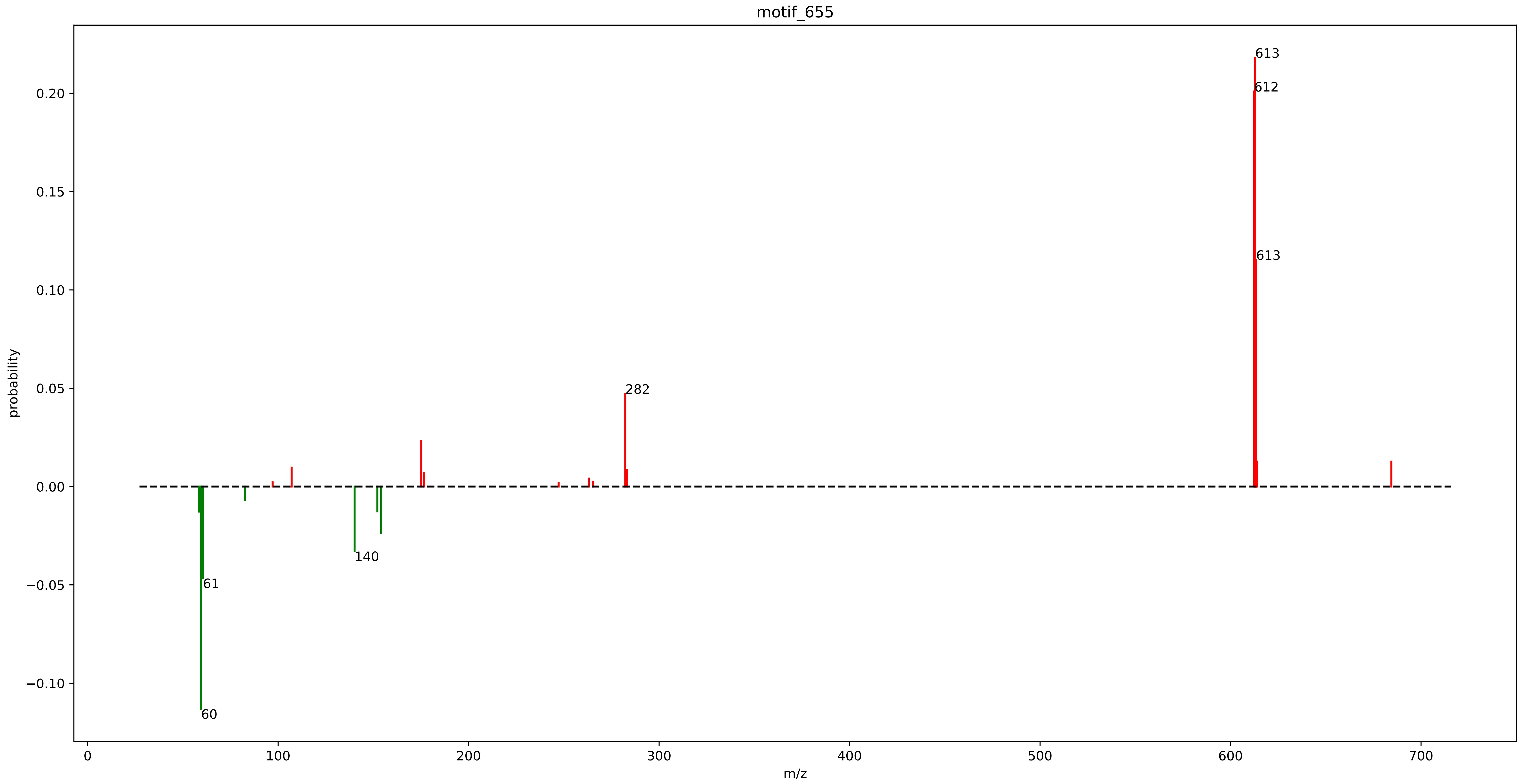

motif_655
probability
m/z
613
612
613
282
61
60
140
0.20
0.15
0.10
0.05
0.00
−0.05
−0.10
0
100
200
300
400
500
600
700

motif_655

fragment_612.8975	: 0.22
fragment_612.3975	: 0.20
fragment_613.3975	: 0.12
loss_59.5175	: 0.11
fragment_282.2775	: 0.05
loss_60.5175	: 0.05
loss_140.1175	: 0.03
loss_154.1375	: 0.02
fragment_175.1475	: 0.02
fragment_613.8975	: 0.01
loss_58.5125	: 0.01
fragment_684.4075	: 0.01
loss_152.1175	: 0.01
fragment_107.0825	: 0.01
fragment_283.2825	: 0.01
loss_82.6125	: 0.01
fragment_176.5925	: 0.01

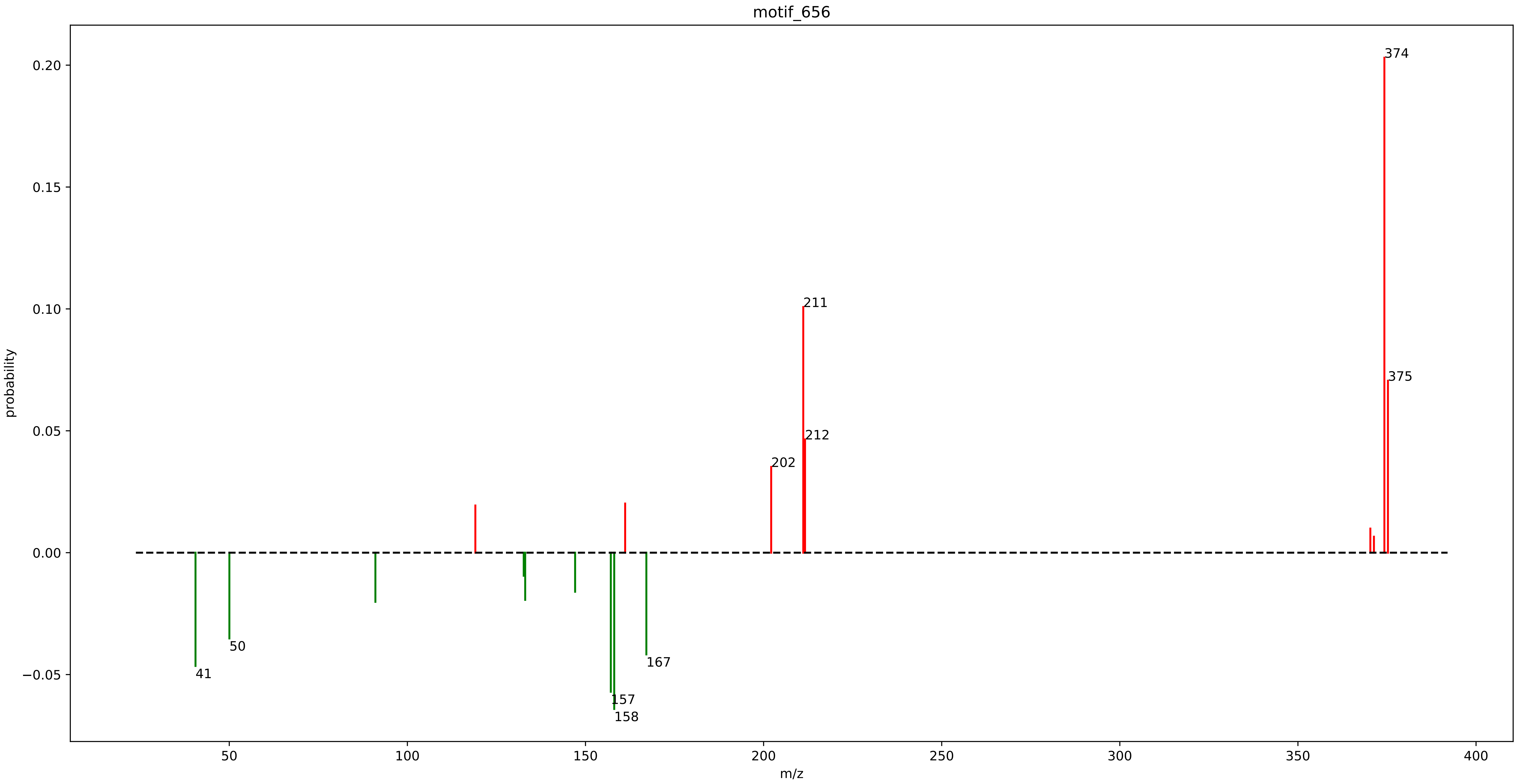

motif_656
probability
m/z
0.20
0.15
0.10
0.05
0.00
−0.05
50
100
150
200
250
300
350
400
374
211
375
212
202
41
50
167
157
158

motif_656

fragment_374.2675	: 0.20
fragment_211.1325	: 0.10
fragment_375.2675	: 0.07
loss_158.0675	: 0.06
loss_157.1075	: 0.06
fragment_211.6325	: 0.05
loss_40.5225	: 0.05
loss_167.0875	: 0.04
loss_50.0325	: 0.04
fragment_202.1275	: 0.04
loss_91.0225	: 0.02
fragment_161.1325	: 0.02
fragment_119.0825	: 0.02
loss_133.0725	: 0.02
loss_147.0875	: 0.02
fragment_370.3275	: 0.01
loss_132.6575	: 0.01
fragment_371.3325	: 0.01

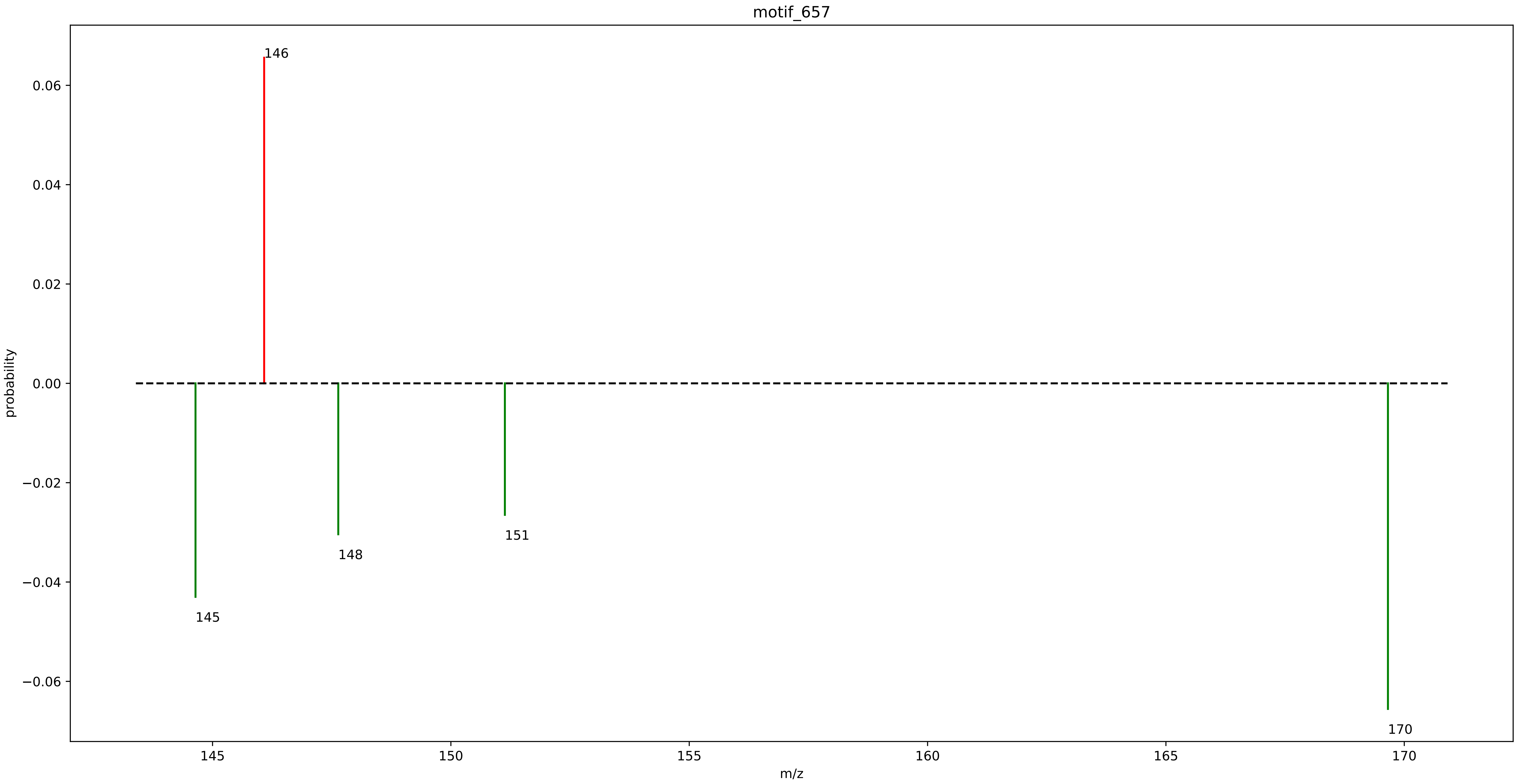

motif_657
probability
m/z
0.06
0.04
0.02
0.00
−0.02
−0.04
−0.06
145
150
155
160
165
170
146
145
148
151
170

motif_657

fragment_146.0825	: 0.07
loss_169.6575	: 0.07
loss_144.6425	: 0.04
loss_147.6375	: 0.03
loss_151.1325	: 0.03

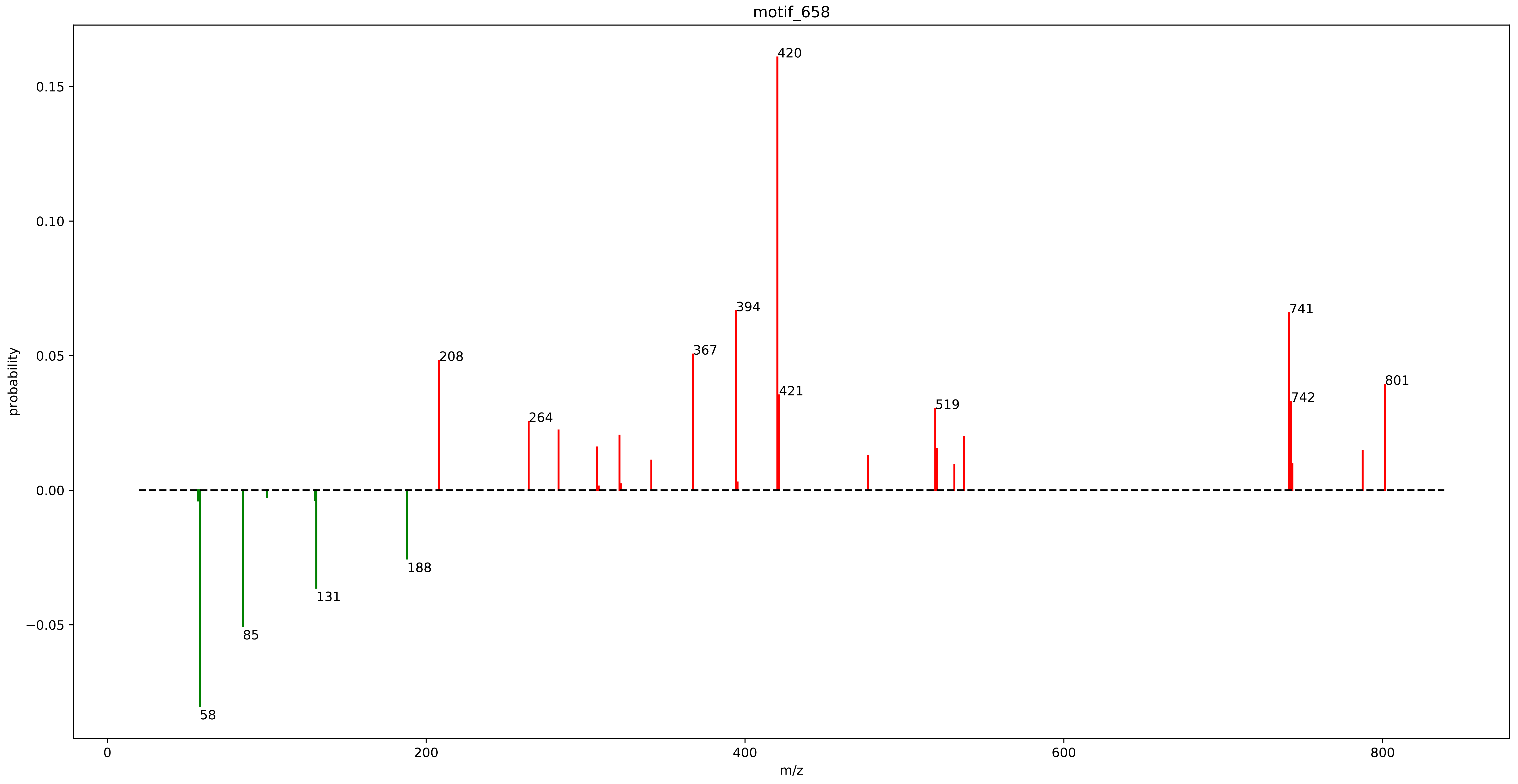

motif_658
0.15
0.10
0.05
0.00
−0.05
probability
0
200
400
600
800
m/z
420
394
741
208
367
421
801
264
742
519
58
85
131
188

motif_658

fragment_420.3075	: 0.16
loss_57.9625	: 0.08
fragment_394.3325	: 0.07
fragment_741.4075	: 0.07
loss_85.0025	: 0.05
fragment_367.2975	: 0.05
fragment_208.1125	: 0.05
fragment_801.4275	: 0.04
loss_131.0575	: 0.04
fragment_421.3125	: 0.04
fragment_742.4075	: 0.03
fragment_519.3325	: 0.03
fragment_264.2325	: 0.03
loss_188.0675	: 0.03
fragment_283.0175	: 0.02
fragment_321.2375	: 0.02
fragment_537.3425	: 0.02
fragment_307.2225	: 0.02
fragment_520.3375	: 0.02
fragment_787.4125	: 0.01
fragment_477.3225	: 0.01
fragment_341.2325	: 0.01
fragment_743.4075	: 0.01
fragment_531.3325	: 0.01

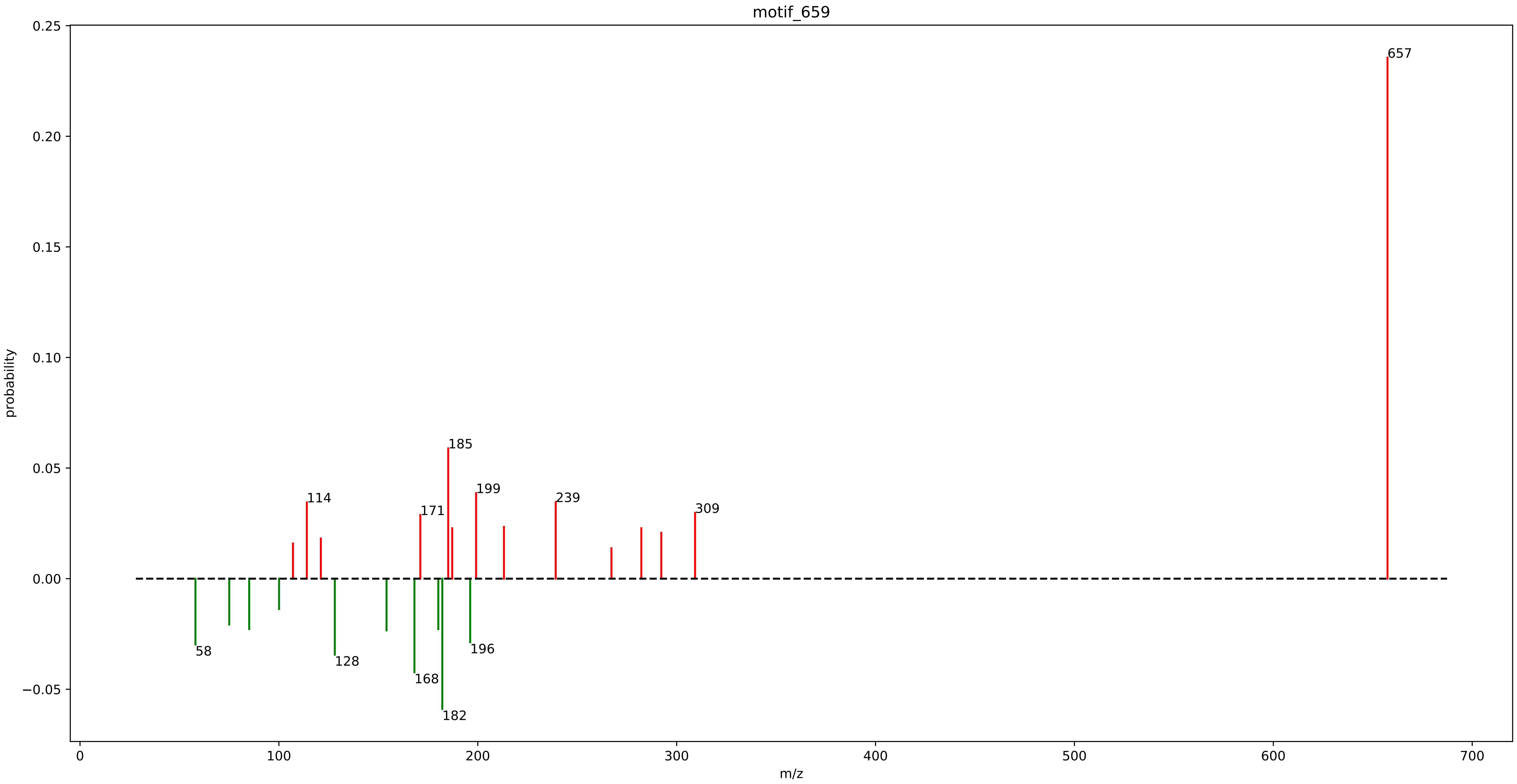
motif_659
probability
m/z
0.25
0.20
0.15
0.10
0.05
0.00
−0.05
0
100
200
300
400
500
600
700
657
185
199
239
114
171
309
58
128
168
182
196

motif_659

fragment_657.3975	: 0.24
fragment_185.1325	: 0.06
loss_182.1675	: 0.06
loss_168.1475	: 0.04
fragment_199.1475	: 0.04
fragment_239.1775	: 0.03
fragment_114.0525	: 0.03
loss_128.1175	: 0.03
loss_58.0425	: 0.03
fragment_309.2575	: 0.03
fragment_171.1175	: 0.03
loss_196.1825	: 0.03
loss_154.1325	: 0.02
fragment_213.1625	: 0.02
loss_180.1475	: 0.02
fragment_282.2325	: 0.02
loss_85.0625	: 0.02
fragment_187.1475	: 0.02
loss_75.0325	: 0.02
fragment_292.2625	: 0.02
fragment_121.0975	: 0.02
fragment_107.0825	: 0.02
loss_100.0875	: 0.01
fragment_267.2075	: 0.01

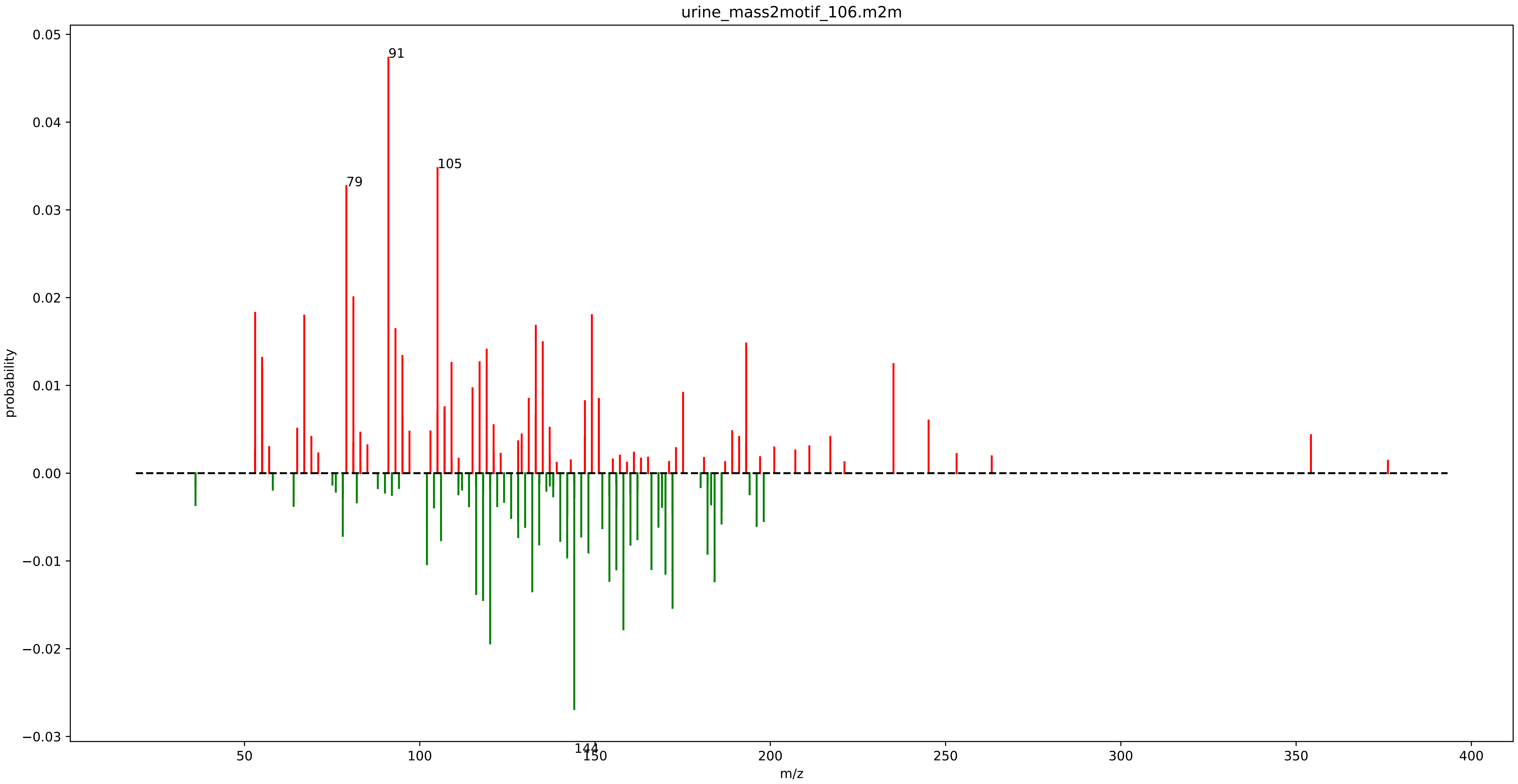

urine_mass2motif_106.m2m
probability
m/z
91
105
79
144
0.05
0.04
0.03
0.02
0.01
0.00
−0.01
−0.02
−0.03
50
100
150
200
250
300
350
400

urine_mass2motif_106.m2m

fragment_91.0525	: 0.05
fragment_105.0675	: 0.03
fragment_79.0525	: 0.03
loss_144.0725	: 0.03
fragment_81.0675	: 0.02
loss_120.0875	: 0.02
fragment_53.0375	: 0.02
fragment_149.0975	: 0.02
fragment_67.0525	: 0.02
loss_158.0925	: 0.02
fragment_133.1025	: 0.02
fragment_93.0675	: 0.02
loss_172.1075	: 0.02
fragment_135.0825	: 0.01
fragment_193.1225	: 0.01
loss_118.0625	: 0.01
fragment_119.0875	: 0.01
loss_116.0825	: 0.01
loss_132.0925	: 0.01
fragment_95.0475	: 0.01
fragment_55.0175	: 0.01
fragment_117.0675	: 0.01
fragment_109.0625	: 0.01
fragment_235.1325	: 0.01
loss_184.1075	: 0.01
loss_154.0975	: 0.01
fragment_55.0525	: 0.01
loss_170.0925	: 0.01
loss_156.0775	: 0.01
loss_166.0975	: 0.01
loss_102.0425	: 0.01
fragment_115.0525	: 0.01
loss_142.0625	: 0.01
loss_182.0925	: 0.01
fragment_175.1125	: 0.01
loss_148.1075	: 0.01
fragment_131.0875	: 0.01
fragment_151.1125	: 0.01
fragment_151.0775	: 0.01
fragment_147.1175	: 0.01
loss_160.1075	: 0.01
loss_134.0625	: 0.01
loss_140.0825	: 0.01
loss_106.0625	: 0.01
loss_162.0925	: 0.01
fragment_107.0875	: 0.01
fragment_107.0475	: 0.01
loss_128.0825	: 0.01
loss_146.0675	: 0.01
loss_78.0375	: 0.01
fragment_105.0325	: 0.01
fragment_133.0625	: 0.01
fragment_95.0875	: 0.01
loss_152.0775	: 0.01
loss_130.0575	: 0.01
loss_168.0825	: 0.01
loss_196.1025	: 0.01
fragment_245.1525	: 0.01
loss_186.0875	: 0.01
fragment_121.0625	: 0.01
loss_198.1275	: 0.01
loss_128.0475	: 0.01
fragment_137.0575	: 0.01
loss_126.0675	: 0.01
fragment_65.0375	: 0.01

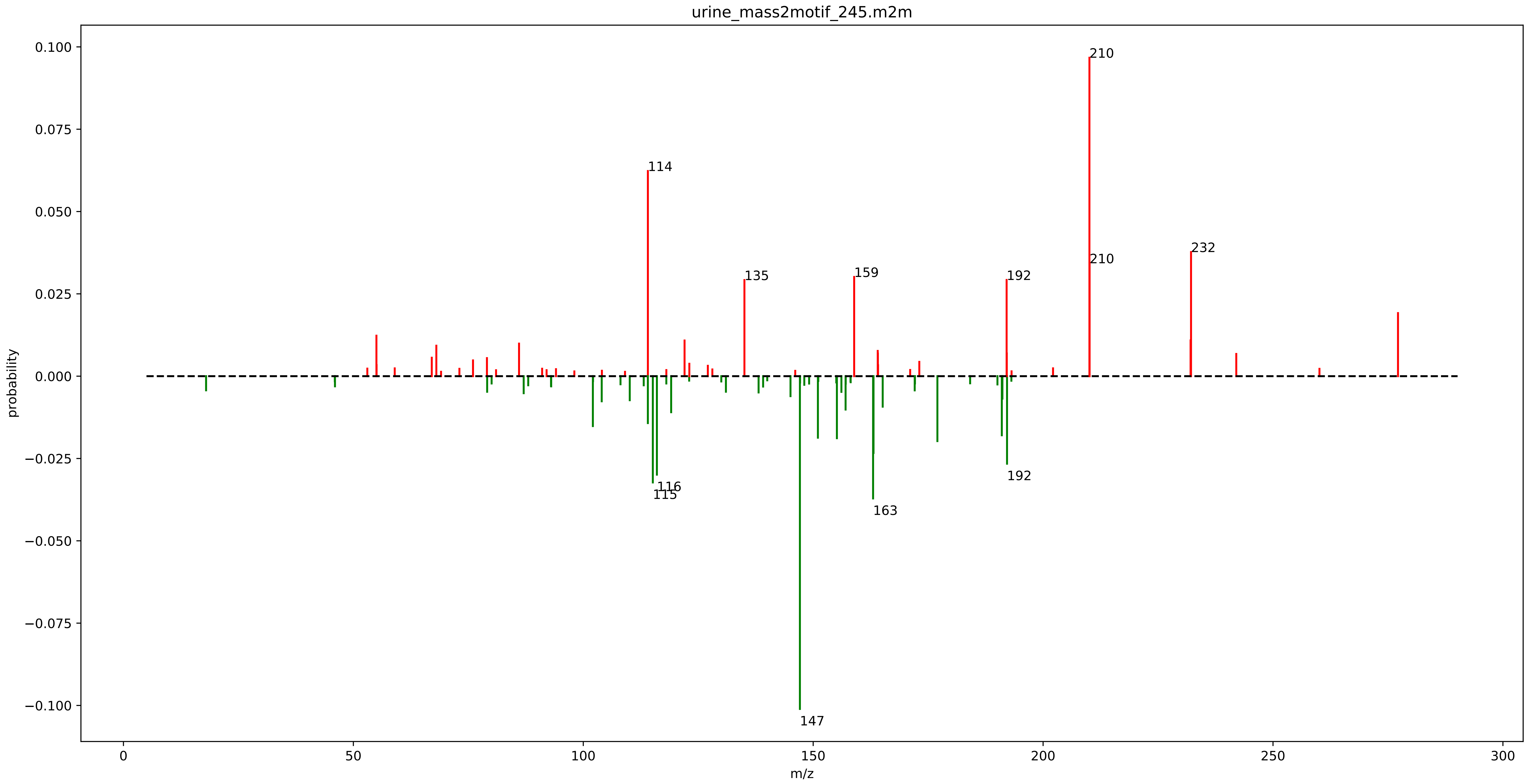
urine_mass2motif_245.m2m
probability
m/z
0.100
0.075
0.050
0.025
0.000
−0.025
−0.050
−0.075
−0.100
0
50
100
150
200
250
300
210
114
210
232
135
159
192
116
115
192
163
147

urine_mass2motif_245.m2m

loss_147.1125 : 0.10	fragment_55.0175 : 0.01
fragment_210.0775 : 0.10	loss_119.1125 : 0.01
fragment_114.0525 : 0.06	fragment_232.0725 : 0.01
fragment_232.1725 : 0.04	fragment_122.0275 : 0.01
loss_163.0275 : 0.04	loss_157.0475 : 0.01
fragment_210.1125 : 0.03	fragment_86.0325 : 0.01
loss_115.1275 : 0.03	loss_165.1175 : 0.01
fragment_158.9125 : 0.03	fragment_68.0475 : 0.01
loss_116.0125 : 0.03	fragment_164.0375 : 0.01
fragment_192.0675 : 0.03	loss_104.0125 : 0.01
fragment_135.0475 : 0.03	loss_110.1075 : 0.01
loss_192.1575 : 0.03	fragment_192.1025 : 0.01
loss_163.1225 : 0.02	fragment_164.0725 : 0.01
loss_177.0125 : 0.02	fragment_232.1525 : 0.01
fragment_277.1825 : 0.02	loss_191.1525 : 0.01
loss_155.1575 : 0.02	fragment_242.0125 : 0.01
loss_151.0275 : 0.02	loss_145.0675 : 0.01
loss_191.0225 : 0.02	fragment_67.0525 : 0.01
loss_102.0875 : 0.02	fragment_79.0525 : 0.01
loss_114.0475 : 0.01	loss_87.0425 : 0.01

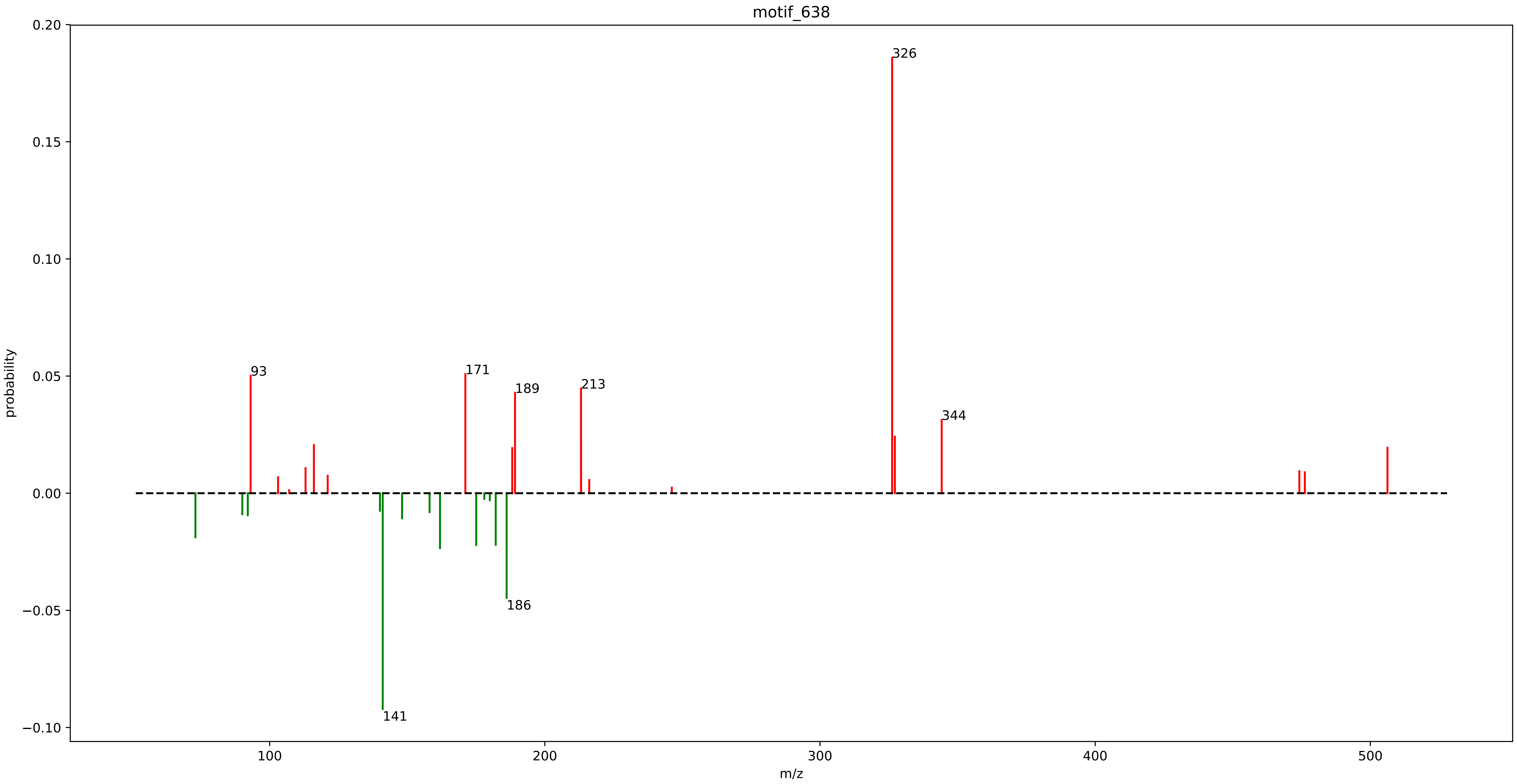

motif_638
326
93
171
189
213
344
141
186
probability
m/z
0.20
0.15
0.10
0.05
0.00
−0.05
−0.10
100
200
300
400
500

motif_638

fragment_326.2125	: 0.19
loss_141.0775	: 0.09
fragment_171.1125	: 0.05
fragment_93.0675	: 0.05
fragment_213.1425	: 0.04
loss_186.1125	: 0.04
fragment_189.1625	: 0.04
fragment_344.2225	: 0.03
fragment_327.2125	: 0.02
loss_161.8975	: 0.02
loss_175.0625	: 0.02
loss_182.1425	: 0.02
fragment_213.1625	: 0.02
fragment_116.0675	: 0.02
fragment_506.2275	: 0.02
fragment_188.1425	: 0.02
loss_73.0175	: 0.02
loss_148.1225	: 0.01
fragment_113.0325	: 0.01
fragment_474.2025	: 0.01
loss_92.0425	: 0.01
loss_90.0475	: 0.01
fragment_476.1975	: 0.01
loss_158.1025	: 0.01
loss_140.0725	: 0.01
fragment_121.0825	: 0.01
fragment_103.0525	: 0.01
fragment_216.1375	: 0.01

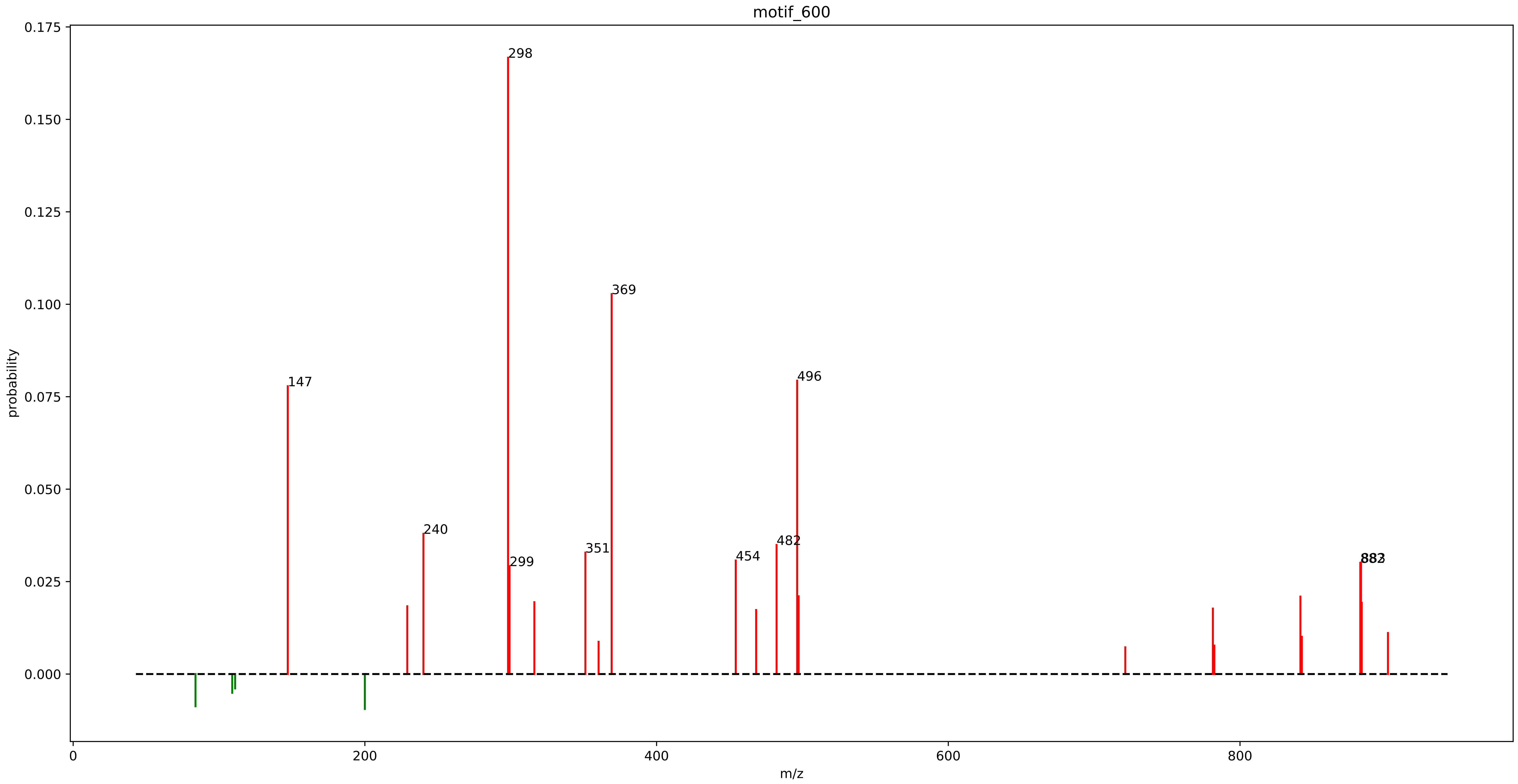
motif_600
probability
m/z
0.175
0.150
0.125
0.100
0.075
0.050
0.025
0.000
0
200
400
600
800
298
369
147
496
240
299
351
454
482
882
883

motif_600

fragment_298.1775	: 0.17
fragment_369.1775	: 0.10
fragment_496.3775	: 0.08
fragment_147.1175	: 0.08
fragment_240.1325	: 0.04
fragment_482.2625	: 0.03
fragment_351.1675	: 0.03
fragment_454.2675	: 0.03
fragment_882.9575	: 0.03
fragment_882.4575	: 0.03
fragment_299.1825	: 0.03
fragment_497.3775	: 0.02
fragment_841.4075	: 0.02
fragment_316.1925	: 0.02
fragment_883.4575	: 0.02
fragment_229.0775	: 0.02
fragment_781.3875	: 0.02
fragment_468.2425	: 0.02
fragment_901.4275	: 0.01
fragment_842.4075	: 0.01
loss_199.9675	: 0.01
fragment_360.2225	: 0.01
loss_83.8775	: 0.01
fragment_782.3875	: 0.01
fragment_721.3625	: 0.01
loss_109.0525	: 0.01

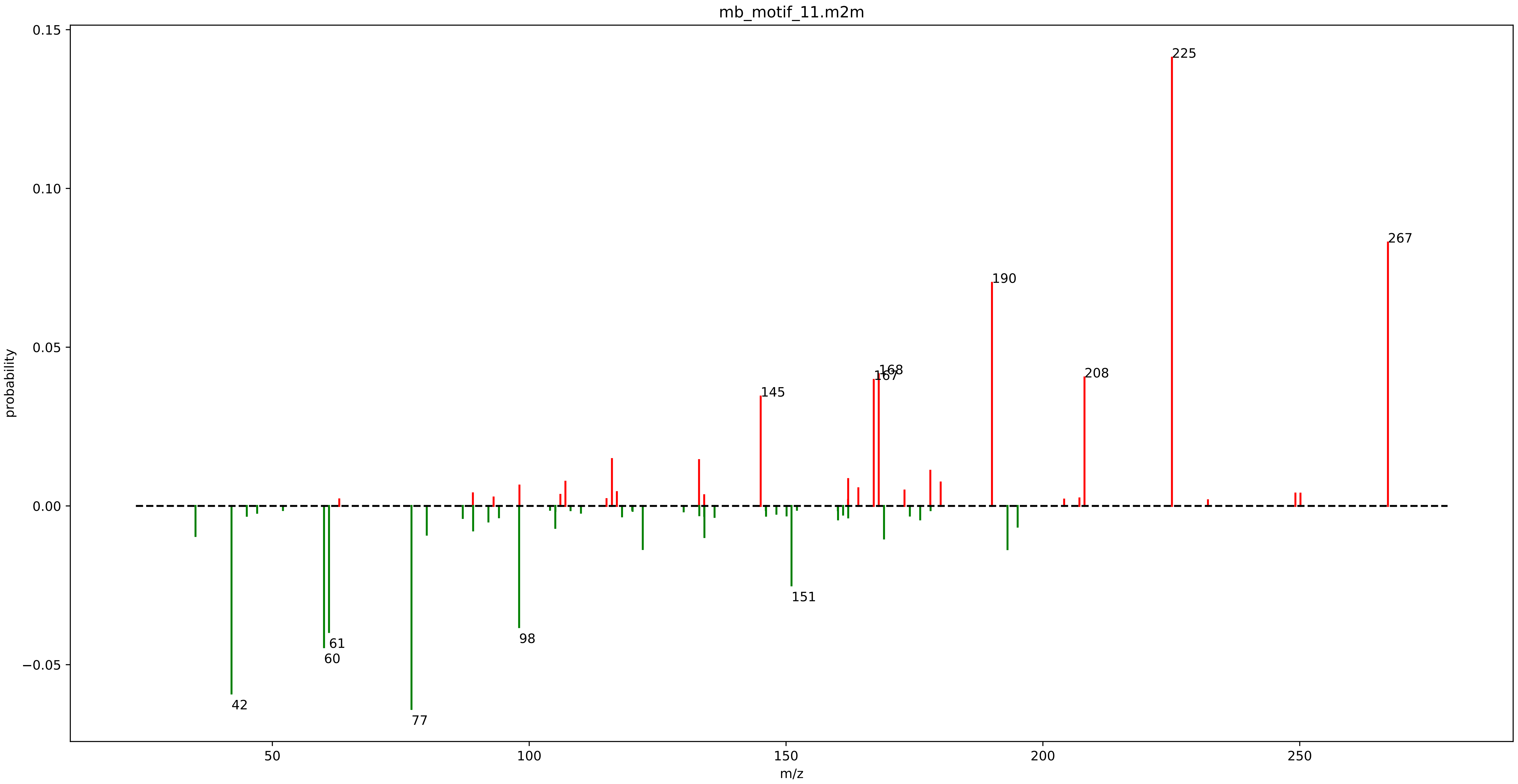

mb_motif_11.m2m
probability
m/z
0.15
0.10
0.05
0.00
−0.05
50
100
150
200
250
225
267
190
168
167
208
145
151
42
61
60
77
98

mb_motif_11.m2m

fragment_225.1225	: 0.14
fragment_267.1725	: 0.08
fragment_190.0875	: 0.07
loss_77.0825	: 0.06
loss_42.0475	: 0.06
loss_60.0575	: 0.04
fragment_168.0425	: 0.04
fragment_208.0975	: 0.04
loss_61.0275	: 0.04
fragment_167.0725	: 0.04
loss_98.0375	: 0.04
fragment_145.0625	: 0.03
loss_151.0625	: 0.03
fragment_116.1075	: 0.01
fragment_133.0625	: 0.01
loss_193.1125	: 0.01
loss_122.1075	: 0.01
fragment_178.0875	: 0.01
loss_169.0725	: 0.01
loss_134.1075	: 0.01
loss_35.0375	: 0.01
loss_80.0575	: 0.01
fragment_162.0925	: 0.01
loss_89.0825	: 0.01
fragment_107.0475	: 0.01
fragment_180.1025	: 0.01
loss_105.0775	: 0.01
loss_195.0875	: 0.01
fragment_98.0975	: 0.01
fragment_164.0725	: 0.01

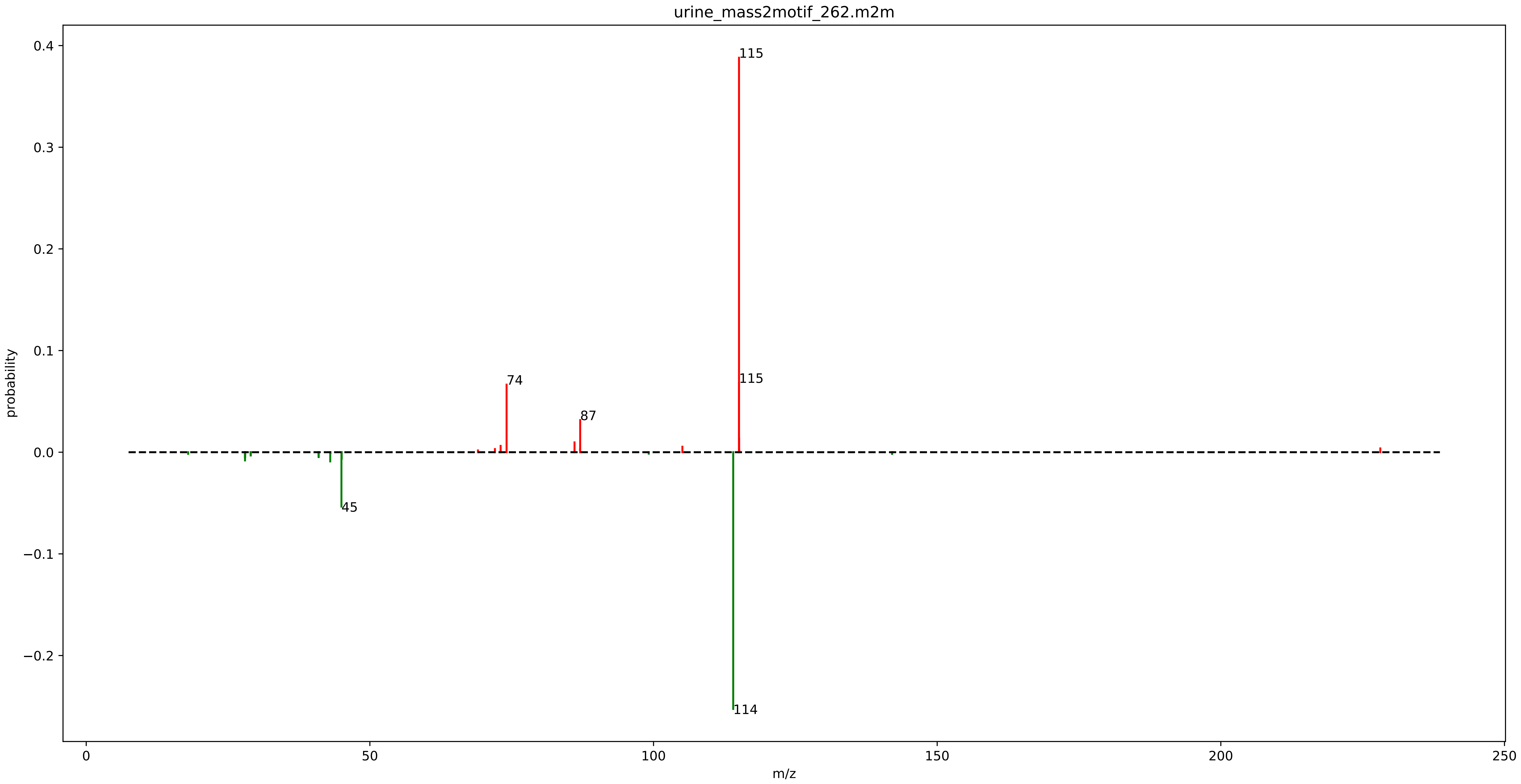

urine_mass2motif_262.m2m
probability
m/z
0.4
0.3
0.2
0.1
0.0
−0.1
−0.2
0
50
100
150
200
250
115
115
74
87
45
114

urine_mass2motif_262.m2m

fragment_115.0675	: 0.39
loss_114.0475	: 0.25
fragment_115.0625	: 0.07
fragment_74.0975	: 0.07
loss_44.9875	: 0.05
fragment_87.0725	: 0.03
fragment_115.0925	: 0.01
fragment_86.0725	: 0.01
fragment_87.0675	: 0.01
loss_43.0125	: 0.01
loss_27.9875	: 0.01
loss_27.9925	: 0.01
loss_45.0325	: 0.01
fragment_73.0475	: 0.01
fragment_105.0875	: 0.01
loss_28.0025	: 0.01
loss_27.9775	: 0.01

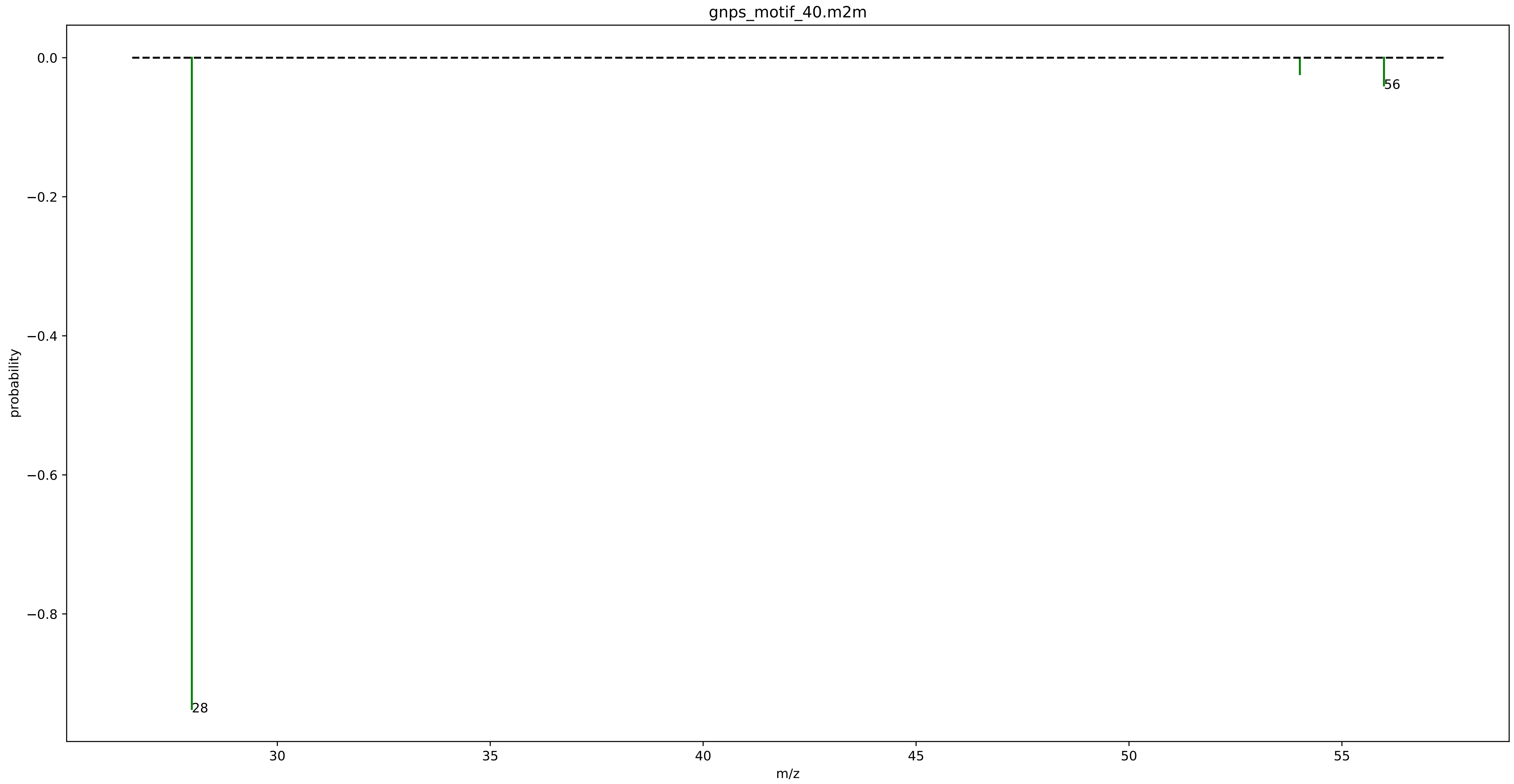
gnps_motif_40.m2m
0.0
−0.2
−0.4
−0.6
−0.8
probability
28
56
30
35
40
45
50
55
m/z

gnps_motif_40.m2m

loss_27.9925	: 0.94
loss_55.9875	: 0.04
loss_54.0125	: 0.02

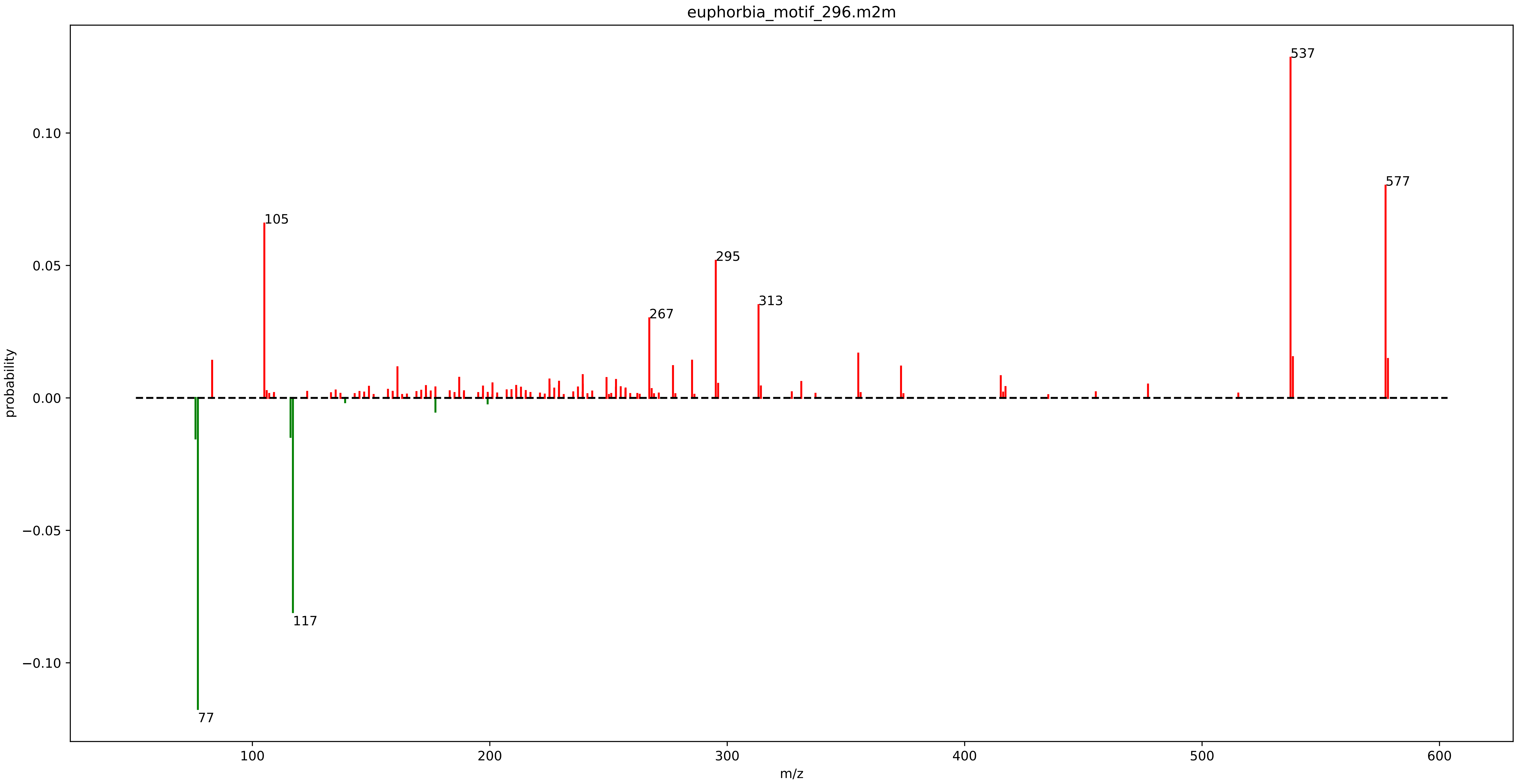

euphorbia_motif_296.m2m
537
577
105
295
313
267
117
77
0.10
0.05
0.00
−0.05
−0.10
probability
100
200
300
400
500
600
m/z

euphorbia_motif_296.m2m

fragment_537.2525	: 0.13
loss_77.0425	: 0.12
loss_117.0725	: 0.08
fragment_577.2825	: 0.08
fragment_105.0325	: 0.07
fragment_295.1725	: 0.05
fragment_313.1825	: 0.04
fragment_267.1725	: 0.03
fragment_355.1925	: 0.02
fragment_538.2525	: 0.02
loss_76.0425	: 0.02
fragment_578.2875	: 0.01
loss_116.0725	: 0.01
fragment_285.1875	: 0.01
fragment_83.0475	: 0.01
fragment_277.1575	: 0.01
fragment_373.2025	: 0.01
fragment_161.0975	: 0.01
fragment_239.1425	: 0.01
fragment_415.2125	: 0.01
fragment_187.1125	: 0.01
fragment_249.1625	: 0.01
fragment_225.1275	: 0.01
fragment_253.1575	: 0.01
fragment_229.1575	: 0.01
fragment_331.1925	: 0.01
fragment_201.1275	: 0.01
fragment_296.1725	: 0.01
loss_177.0975	: 0.01
fragment_477.2275	: 0.01

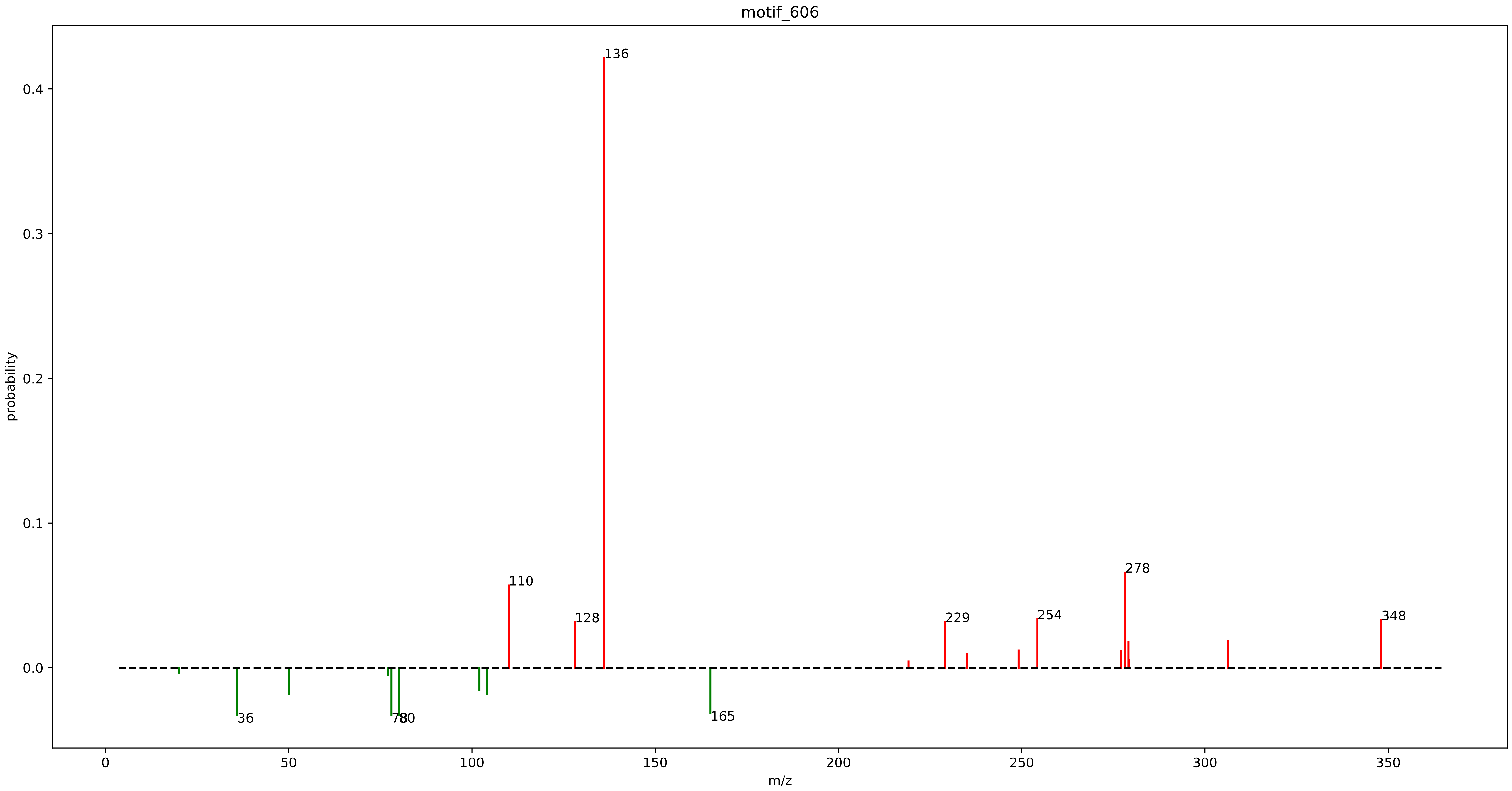

motif_606
probability
m/z
0.4
0.3
0.2
0.1
0.0
0
50
100
150
200
250
300
350
136
110
128
229
254
278
348
36
78
80
165

motif_606

fragment_136.0725	: 0.42
fragment_278.2475	: 0.07
fragment_110.0575	: 0.06
fragment_254.2475	: 0.03
loss_80.0475	: 0.03
loss_35.9775	: 0.03
fragment_348.1125	: 0.03
loss_78.0325	: 0.03
fragment_229.1225	: 0.03
loss_165.0725	: 0.03
fragment_128.1075	: 0.03
fragment_306.2425	: 0.02
loss_50.0375	: 0.02
loss_104.0475	: 0.02
fragment_279.1375	: 0.02
loss_102.0325	: 0.02
fragment_249.1575	: 0.01
fragment_277.1525	: 0.01
fragment_235.1425	: 0.01
fragment_279.2525	: 0.01
loss_77.0275	: 0.01

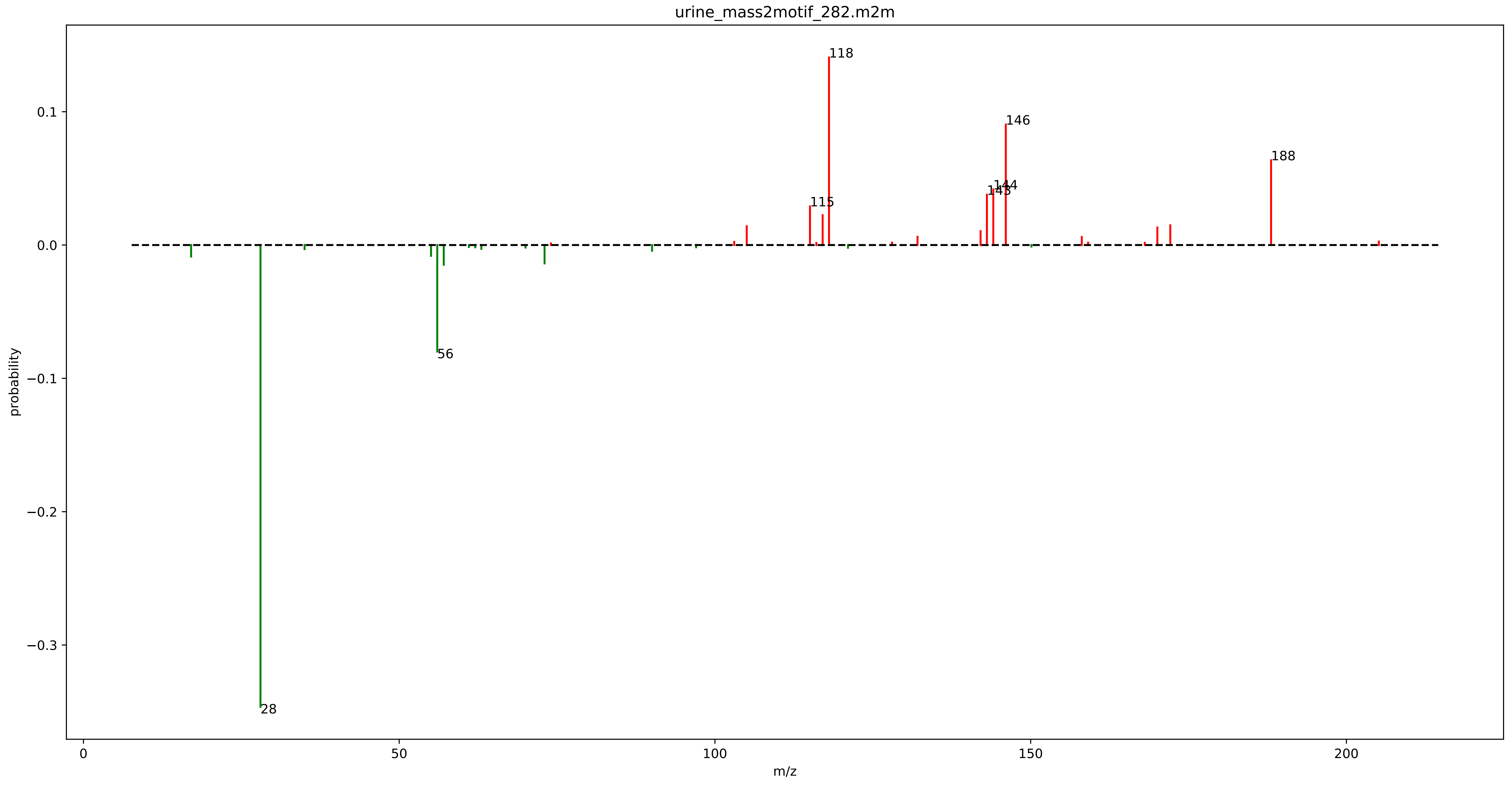
urine_mass2motif_282.m2m
probability
m/z
0.1
0.0
−0.1
−0.2
−0.3
0
50
100
150
200
118
146
188
144
143
115
56
28

urine_mass2motif_282.m2m

loss_28.0325	: 0.35
fragment_118.0675	: 0.14
fragment_146.0625	: 0.09
loss_56.0275	: 0.08
fragment_188.0725	: 0.06
fragment_144.0825	: 0.04
fragment_143.0725	: 0.04
fragment_115.0525	: 0.03
fragment_117.0575	: 0.02
loss_57.0575	: 0.01
fragment_172.1125	: 0.01
fragment_105.0375	: 0.01
loss_73.0175	: 0.01
fragment_170.0625	: 0.01
fragment_142.0675	: 0.01
loss_17.0475	: 0.01
loss_55.0425	: 0.01
fragment_132.0825	: 0.01
fragment_158.0975	: 0.01

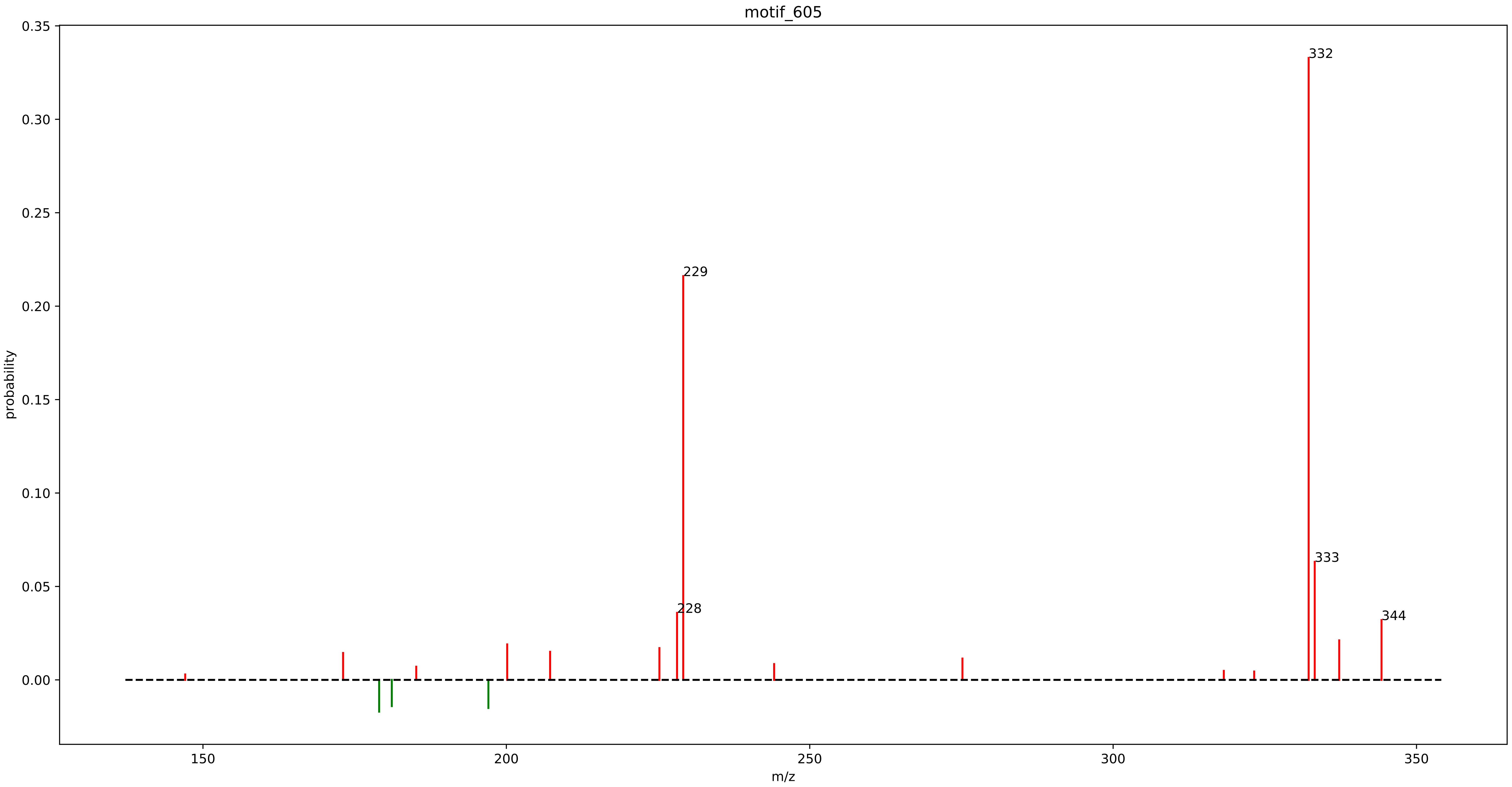

motif_605
332
229
333
228
344
probability
m/z
0.35
0.30
0.25
0.20
0.15
0.10
0.05
0.00
150
200
250
300
350

motif_605

fragment_332.2175	: 0.33
fragment_229.1475	: 0.22
fragment_333.2225	: 0.06
fragment_228.1325	: 0.04
fragment_344.2325	: 0.03
fragment_337.2575	: 0.02
fragment_200.1375	: 0.02
fragment_225.2225	: 0.02
loss_179.0325	: 0.02
loss_197.0425	: 0.02
fragment_207.2125	: 0.02
fragment_173.0925	: 0.01
loss_181.1175	: 0.01
fragment_275.1625	: 0.01
fragment_244.1275	: 0.01
fragment_185.1475	: 0.01

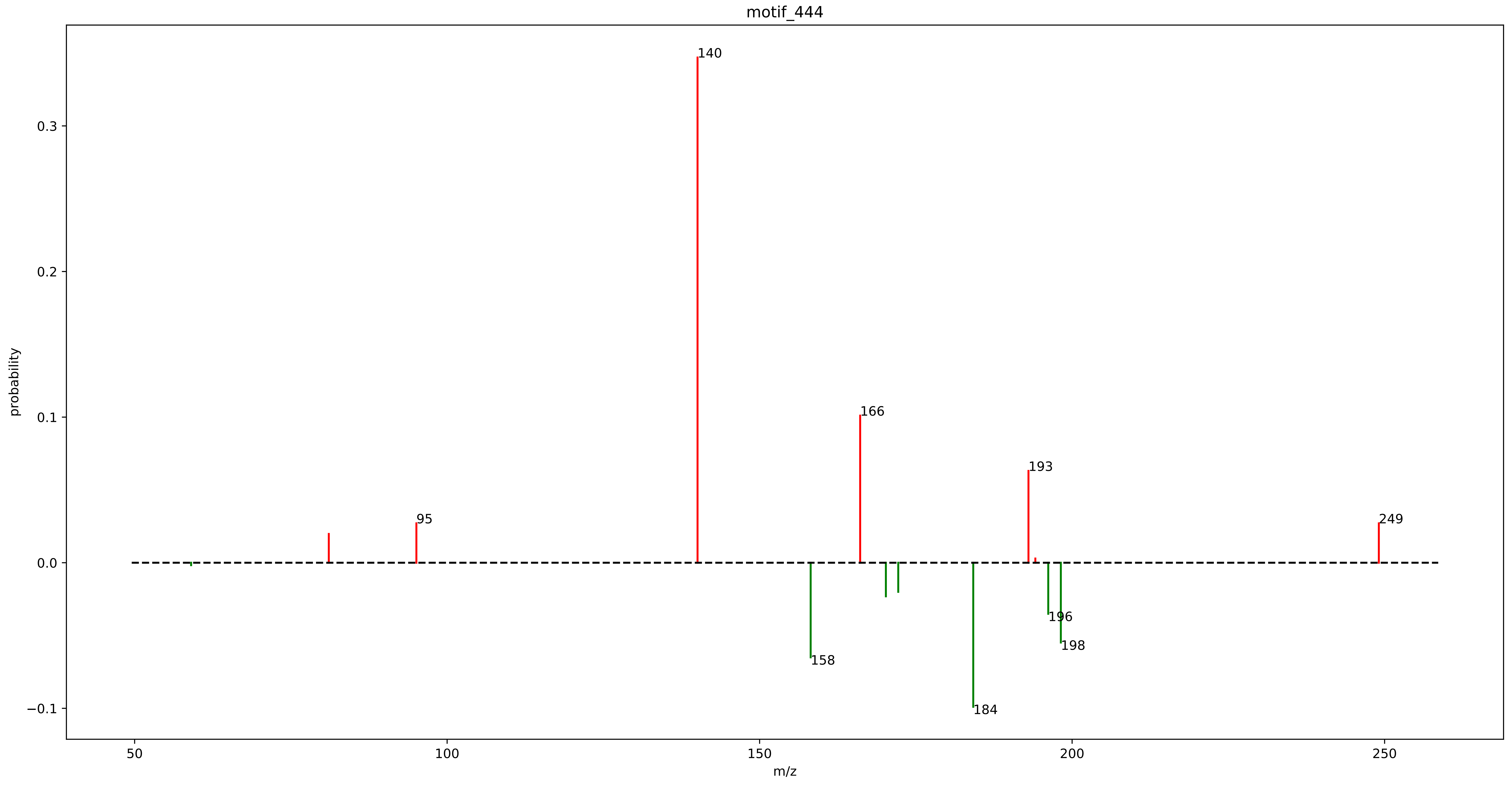
motif_444
140
166
193
95
249
158
184
196
198
0.3
0.2
0.1
0.0
−0.1
probability
50
100
150
200
250
m/z

motif_444

fragment_140.0675	: 0.35
fragment_166.0875	: 0.10
loss_184.1825	: 0.10
loss_158.1675	: 0.06
fragment_193.0175	: 0.06
loss_198.1975	: 0.05
loss_196.1825	: 0.04
fragment_249.0775	: 0.03
fragment_95.0825	: 0.03
loss_170.2025	: 0.02
loss_172.1825	: 0.02
fragment_81.0675	: 0.02

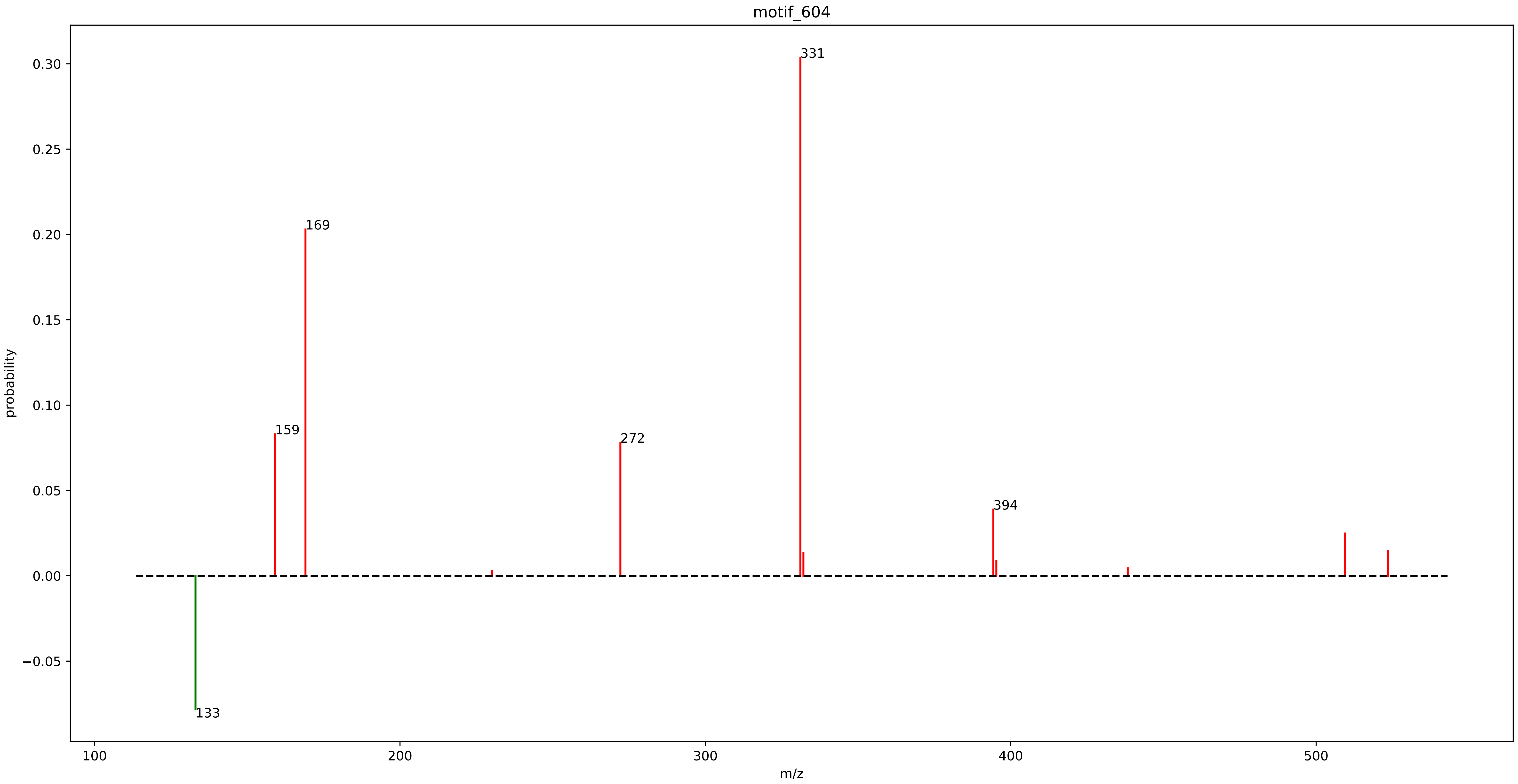
motif_604
331
169
159
272
394
133
probability
m/z
0.30
0.25
0.20
0.15
0.10
0.05
0.00
−0.05
100
200
300
400
500

motif_604

fragment_331.1025	: 0.30
fragment_169.0475	: 0.20
fragment_159.0925	: 0.08
fragment_272.1725	: 0.08
loss_133.0375	: 0.08
fragment_394.2825	: 0.04
fragment_509.4925	: 0.02
fragment_523.5075	: 0.01
fragment_332.1075	: 0.01
fragment_395.2875	: 0.01

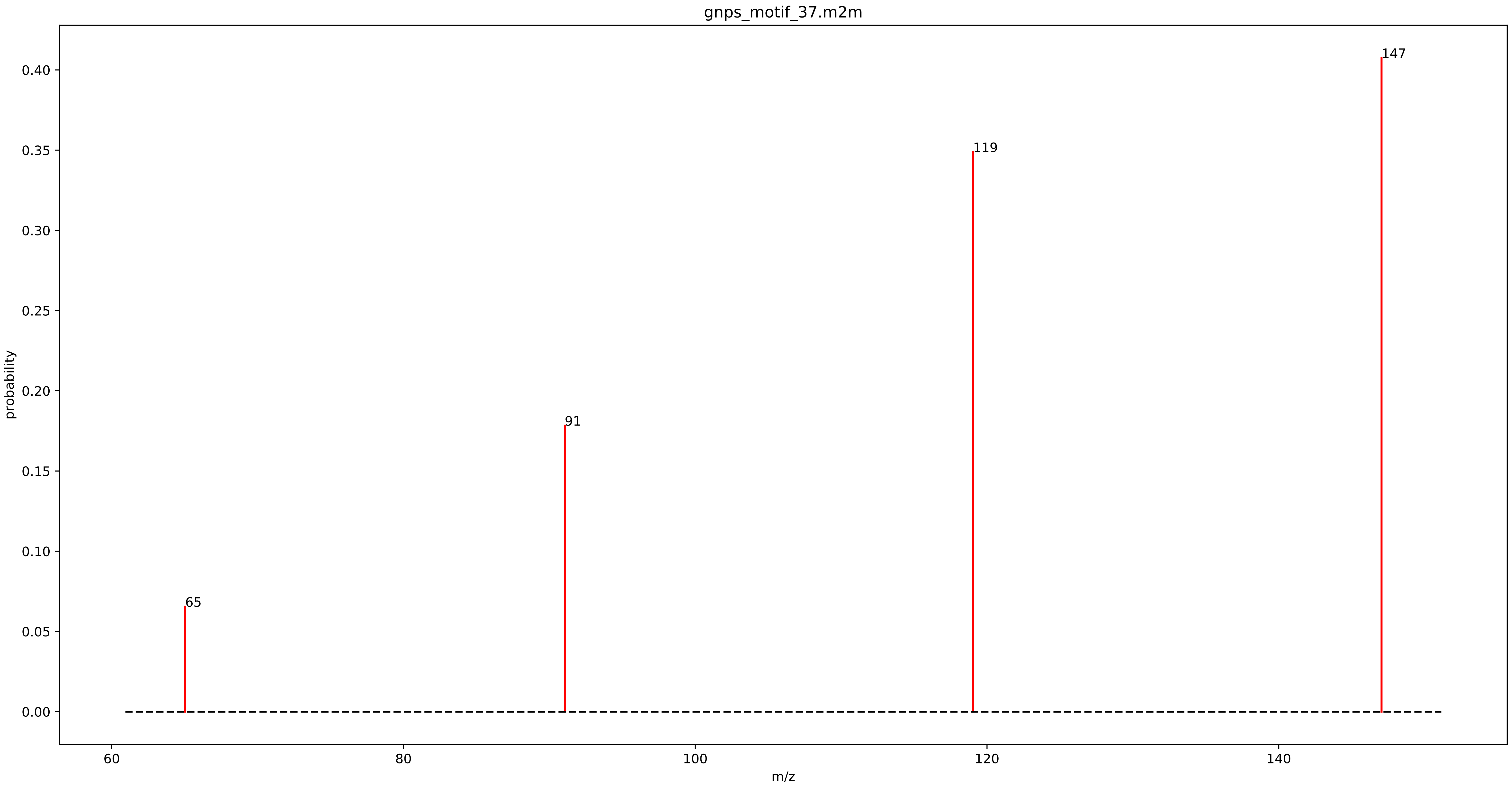

gnps_motif_37.m2m
probability
m/z
0.00
0.05
0.10
0.15
0.20
0.25
0.30
0.35
0.40
60
80
100
120
140
65
91
119
147

gnps_motif_37.m2m

fragment_147.0425	: 0.41
fragment_119.0475	: 0.35
fragment_91.0525	: 0.18
fragment_65.0375	: 0.07

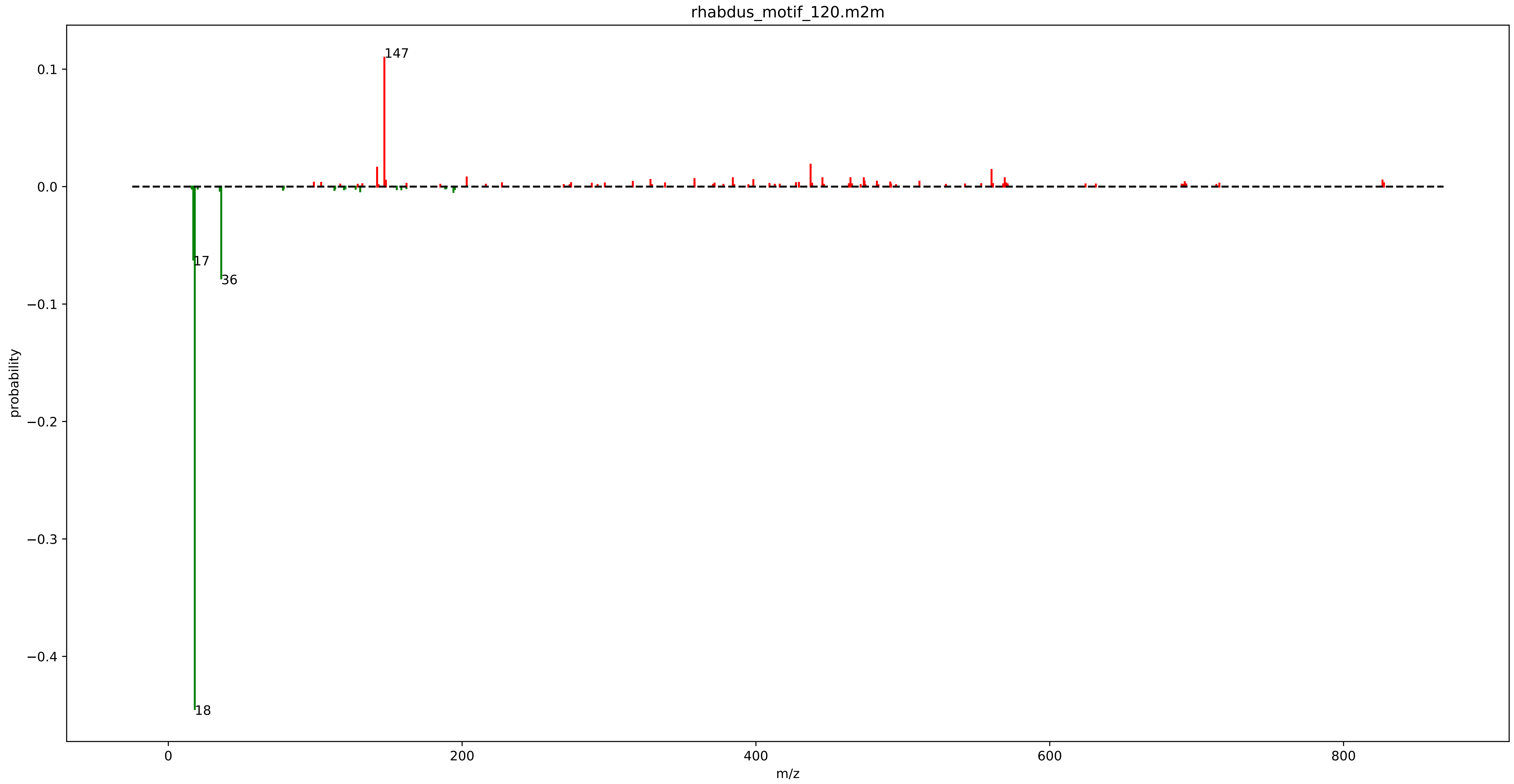

rhabdus_motif_120.m2m
147
17
36
18
0.1
0.0
−0.1
−0.2
−0.3
−0.4
probability
0
200
400
600
800
m/z

rhabdus_motif_120.m2m

loss_18.0125	: 0.44
fragment_147.0775	: 0.11
loss_36.0225	: 0.08
loss_17.0075	: 0.06
fragment_437.2025	: 0.02
fragment_142.1225	: 0.02
fragment_560.3775	: 0.01
fragment_203.1175	: 0.01
fragment_464.3525	: 0.01
fragment_473.4275	: 0.01
fragment_384.2875	: 0.01
fragment_445.2575	: 0.01
fragment_569.3625	: 0.01
fragment_358.1625	: 0.01
fragment_328.2225	: 0.01
fragment_398.2225	: 0.01
loss_17.5075	: 0.01
fragment_826.5125	: 0.01
fragment_148.0825	: 0.01

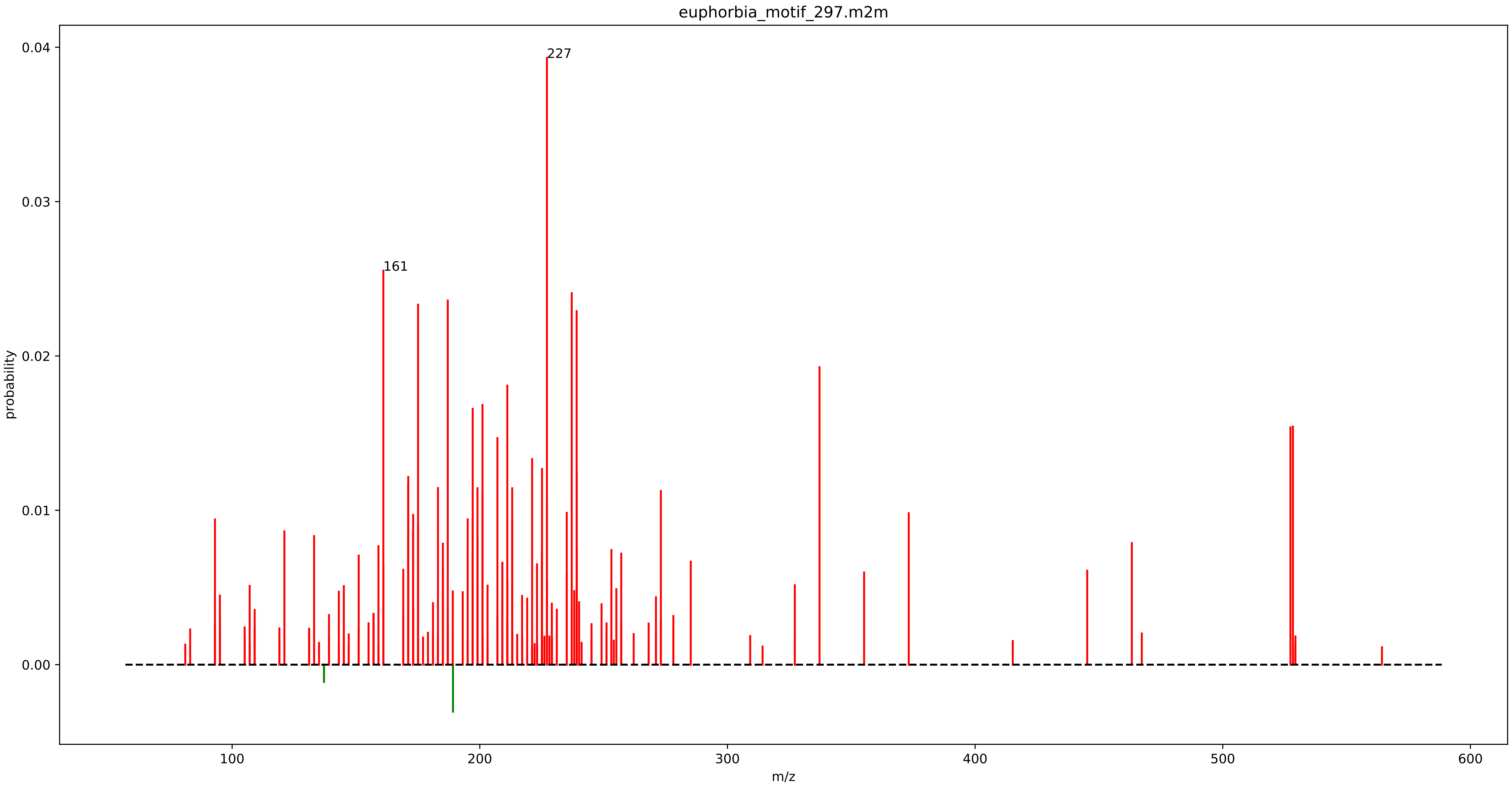
euphorbia_motif_297.m2m
227
161
0.04
0.03
0.02
0.01
0.00
probability
100
200
300
400
500
600
m/z

euphorbia_motif_297.m2m

fragment_227.1075	: 0.04
fragment_161.0575	: 0.03
fragment_237.1275	: 0.02
fragment_187.0725	: 0.02
fragment_175.0725	: 0.02
fragment_239.1075	: 0.02
fragment_337.1775	: 0.02
fragment_211.1125	: 0.02
fragment_201.0875	: 0.02
fragment_197.1325	: 0.02
fragment_528.3375	: 0.02
fragment_527.3325	: 0.02
fragment_207.1175	: 0.01
fragment_221.1325	: 0.01
fragment_225.1275	: 0.01
fragment_239.1425	: 0.01
fragment_171.1175	: 0.01
fragment_171.0775	: 0.01
fragment_183.1175	: 0.01
fragment_199.0725	: 0.01
fragment_213.0875	: 0.01
fragment_273.1125	: 0.01
fragment_201.0925	: 0.01
fragment_227.1025	: 0.01
fragment_235.1125	: 0.01
fragment_373.1975	: 0.01
fragment_173.0925	: 0.01
fragment_197.0925	: 0.01
fragment_195.1175	: 0.01
fragment_93.0675	: 0.01
fragment_213.0925	: 0.01
fragment_175.0775	: 0.01
fragment_121.1025	: 0.01
fragment_173.0575	: 0.01
fragment_133.1025	: 0.01
fragment_171.0825	: 0.01
fragment_199.1125	: 0.01
fragment_463.2825	: 0.01
fragment_185.0925	: 0.01
fragment_159.0775	: 0.01
fragment_239.1775	: 0.01
fragment_253.1525	: 0.01
fragment_225.0875	: 0.01
fragment_197.0975	: 0.01
fragment_257.1175	: 0.01
fragment_151.1125	: 0.01
fragment_225.0925	: 0.01
fragment_285.1875	: 0.01
fragment_209.1275	: 0.01
fragment_209.0925	: 0.01
fragment_161.0925	: 0.01
fragment_223.1125	: 0.01
fragment_169.1025	: 0.01
fragment_185.0975	: 0.01
fragment_445.2725	: 0.01
fragment_199.0775	: 0.01
fragment_355.1825	: 0.01
fragment_235.1075	: 0.01
fragment_171.1125	: 0.01
fragment_211.1475	: 0.01
fragment_173.0625	: 0.01
fragment_199.1075	: 0.01
fragment_227.1375	: 0.01
fragment_327.1975	: 0.01
fragment_203.1425	: 0.01
fragment_107.0825	: 0.01
fragment_145.1025	: 0.01

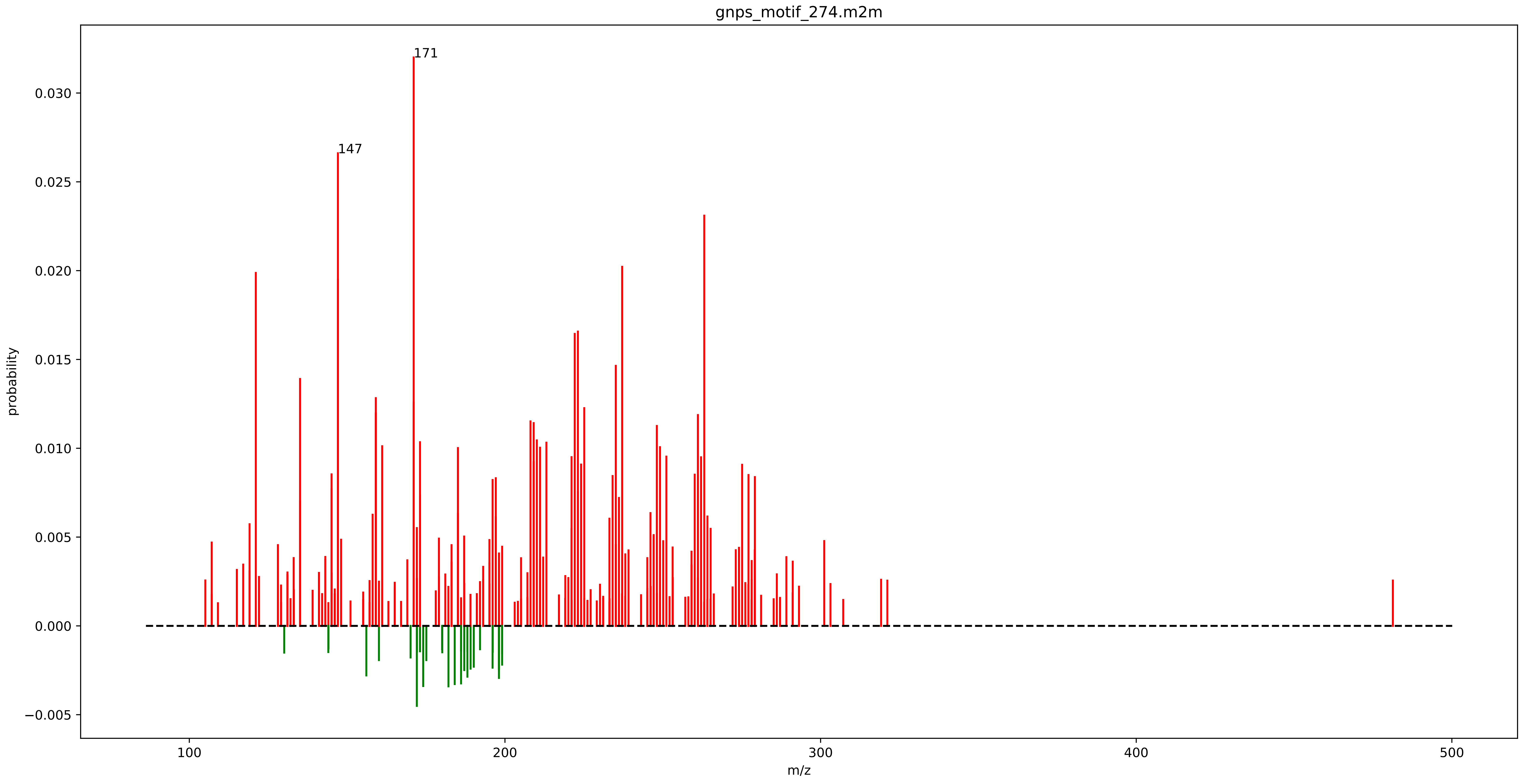

gnps_motif_274.m2m
171
147
probability
0.030
0.025
0.020
0.015
0.010
0.005
0.000
−0.005
100
200
300
400
500
m/z

gnps_motif_274.m2m

fragment_171.0775	: 0.03
fragment_147.0775	: 0.03
fragment_263.1425	: 0.02
fragment_237.1275	: 0.02
fragment_121.0625	: 0.02
fragment_147.0825	: 0.02
fragment_223.1125	: 0.02
fragment_222.1025	: 0.02
fragment_235.1125	: 0.01
fragment_135.0825	: 0.01
fragment_159.0825	: 0.01
fragment_171.0825	: 0.01
fragment_225.1275	: 0.01
fragment_159.0775	: 0.01
fragment_261.1275	: 0.01
fragment_208.0875	: 0.01
fragment_209.0975	: 0.01
fragment_248.1175	: 0.01
fragment_210.1025	: 0.01
fragment_173.0925	: 0.01
fragment_213.1275	: 0.01
fragment_161.0975	: 0.01
fragment_249.1275	: 0.01
fragment_211.1125	: 0.01
fragment_185.0975	: 0.01
fragment_251.1425	: 0.01
fragment_221.0975	: 0.01
fragment_262.1325	: 0.01
fragment_209.0925	: 0.01
fragment_224.1175	: 0.01
fragment_275.1425	: 0.01
fragment_145.0625	: 0.01
fragment_260.1175	: 0.01
fragment_277.1575	: 0.01
fragment_234.1025	: 0.01
fragment_279.1725	: 0.01
fragment_197.0975	: 0.01
fragment_196.0875	: 0.01
fragment_145.0675	: 0.01
fragment_223.1075	: 0.01
fragment_173.0975	: 0.01
fragment_236.1175	: 0.01
fragment_135.0775	: 0.01
fragment_246.1025	: 0.01
fragment_185.0925	: 0.01
fragment_158.0725	: 0.01
fragment_264.1475	: 0.01
fragment_197.0925	: 0.01
fragment_233.0975	: 0.01
fragment_119.0875	: 0.01
fragment_172.0825	: 0.01
fragment_265.1575	: 0.01
fragment_221.0925	: 0.01
fragment_247.1125	: 0.01
fragment_247.1075	: 0.01
fragment_187.0725	: 0.01

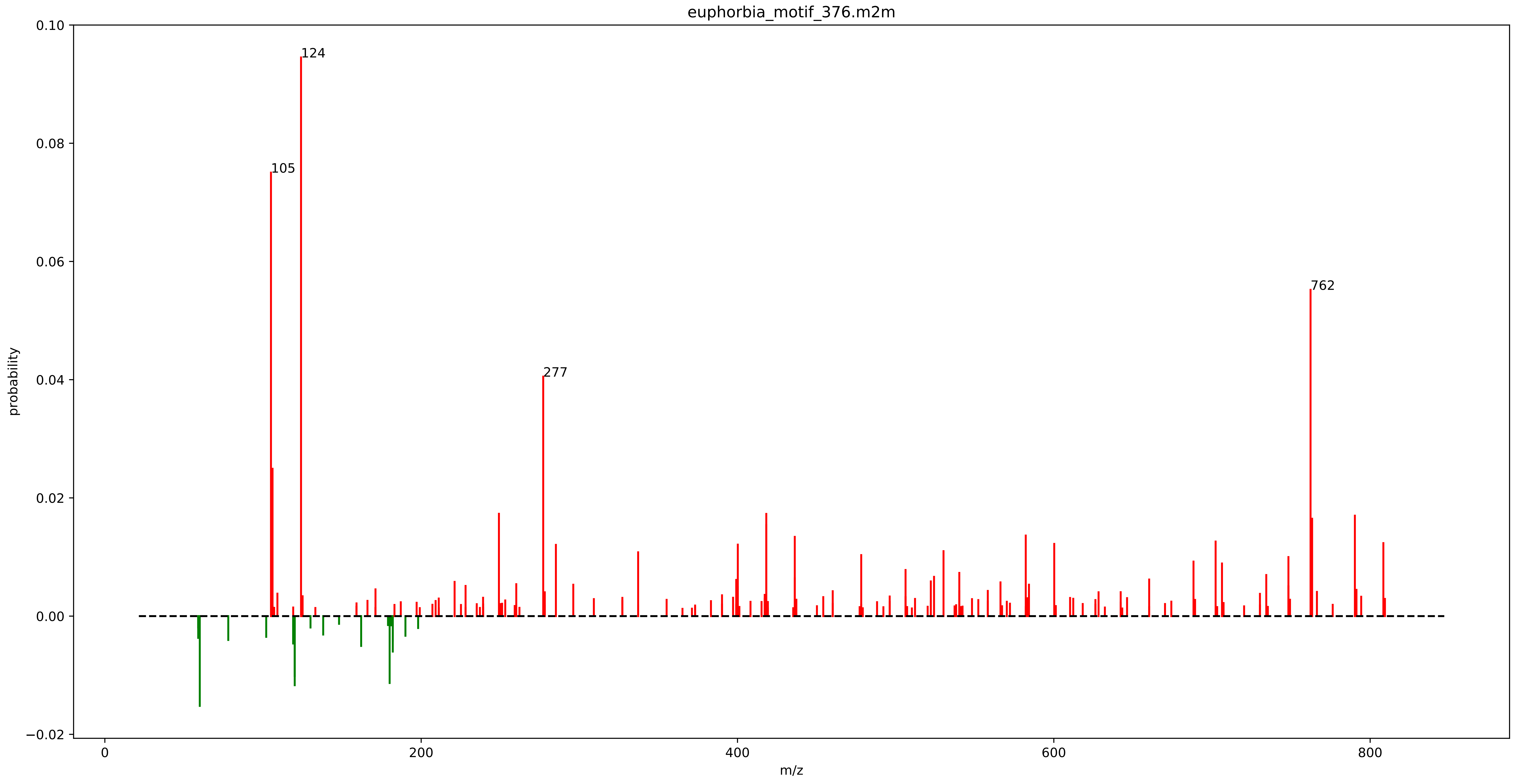

euphorbia_motif_376.m2m
probability
m/z
105
124
277
762
0.10
0.08
0.06
0.04
0.02
0.00
−0.02
0
200
400
600
800

euphorbia_motif_376.m2m

fragment_124.0375	: 0.09
fragment_105.0325	: 0.08
fragment_762.3125	: 0.06
fragment_277.1575	: 0.04
fragment_106.0275	: 0.02
fragment_249.1625	: 0.02
fragment_418.2025	: 0.02
fragment_790.3425	: 0.02
fragment_763.3175	: 0.02
fragment_418.1975	: 0.02
loss_60.0175	: 0.02
fragment_582.2475	: 0.01
fragment_436.2125	: 0.01
fragment_702.2925	: 0.01
fragment_808.3525	: 0.01
fragment_600.2575	: 0.01
fragment_400.1925	: 0.01
fragment_285.1825	: 0.01
loss_120.0475	: 0.01
loss_180.0625	: 0.01
fragment_530.2525	: 0.01
fragment_337.1775	: 0.01
fragment_478.2225	: 0.01
loss_120.0375	: 0.01
fragment_748.3275	: 0.01
fragment_688.3075	: 0.01
fragment_706.3225	: 0.01
fragment_506.2525	: 0.01
fragment_540.2375	: 0.01
fragment_734.3175	: 0.01
fragment_524.2625	: 0.01
fragment_436.2075	: 0.01
fragment_660.2825	: 0.01
fragment_399.1925	: 0.01
loss_182.0525	: 0.01
fragment_522.2275	: 0.01
fragment_221.1325	: 0.01
fragment_566.2725	: 0.01
fragment_260.1275	: 0.01
fragment_584.2825	: 0.01
fragment_296.1725	: 0.01
fragment_584.2875	: 0.01
fragment_228.0675	: 0.01
loss_162.0525	: 0.01
fragment_748.3325	: 0.01

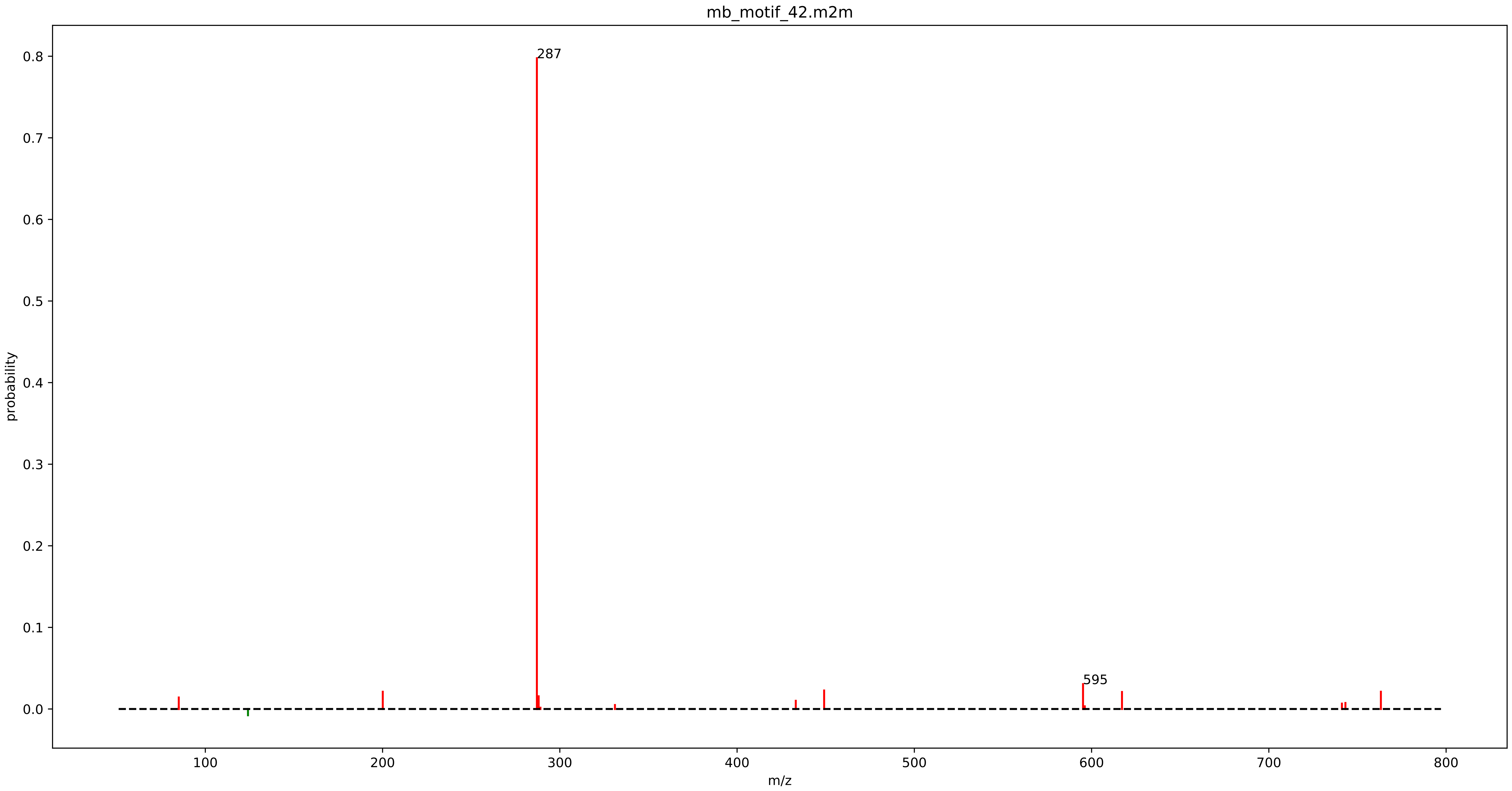

mb_motif_42.m2m
287
595
probability
m/z
0.0
0.1
0.2
0.3
0.4
0.5
0.6
0.7
0.8
100
200
300
400
500
600
700
800

mb_motif_42.m2m

fragment_287.0575	: 0.80
fragment_595.1675	: 0.03
fragment_449.1075	: 0.02
fragment_763.2075	: 0.02
fragment_200.1175	: 0.02
fragment_617.1475	: 0.02
fragment_288.0575	: 0.02
fragment_85.0275	: 0.01
fragment_287.1425	: 0.01
fragment_433.1125	: 0.01
loss_124.0725	: 0.01
fragment_743.2025	: 0.01
fragment_741.2225	: 0.01

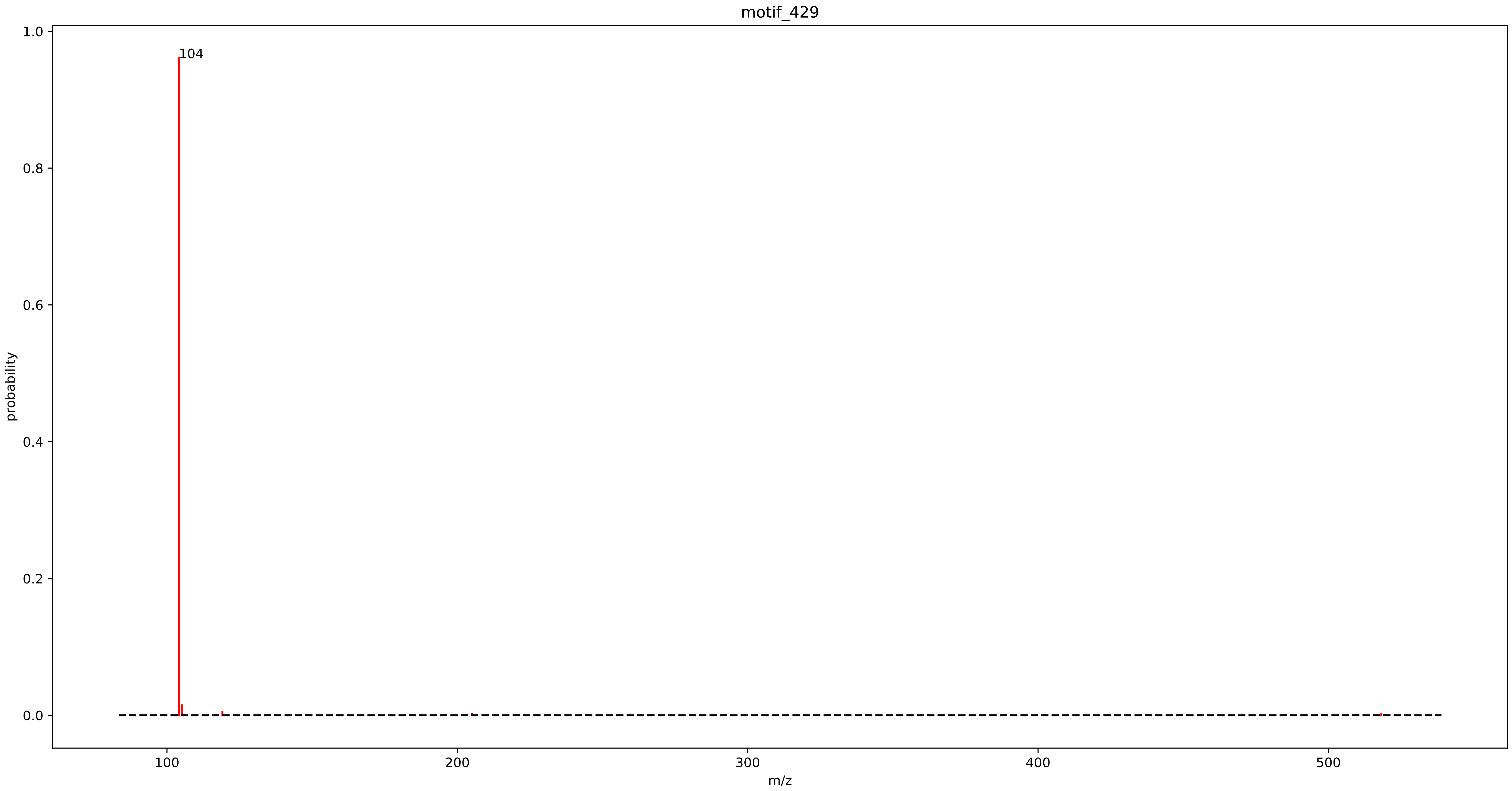

motif_429
104
probability
1.0
0.8
0.6
0.4
0.2
0.0
100
200
300
400
500
m/z

motif_429

fragment_104.0725	: 0.96
fragment_105.0725	: 0.01

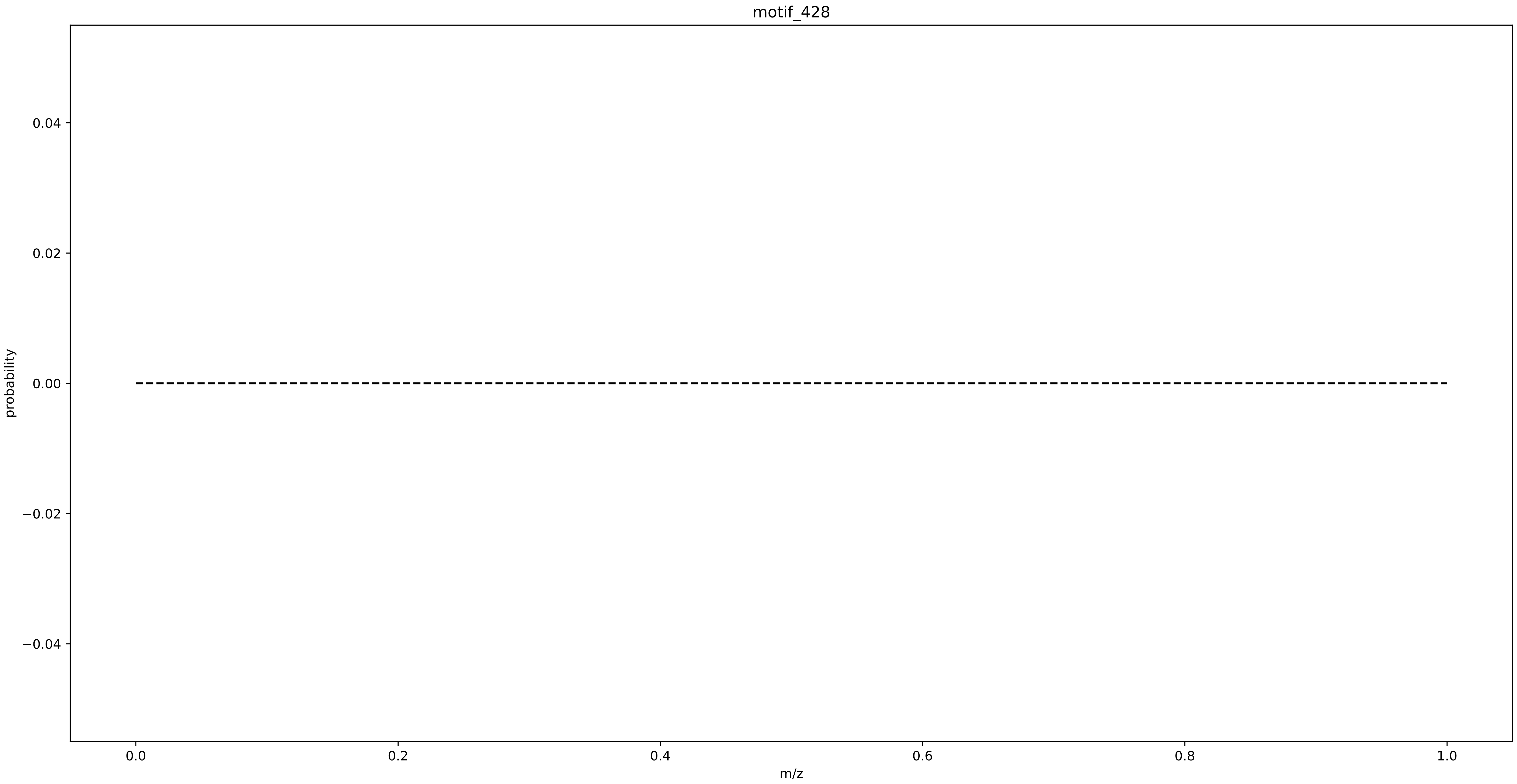

motif_428
probability
m/z
0.04
0.02
0.00
−0.02
−0.04
0.0
0.2
0.4
0.6
0.8
1.0

motif_428

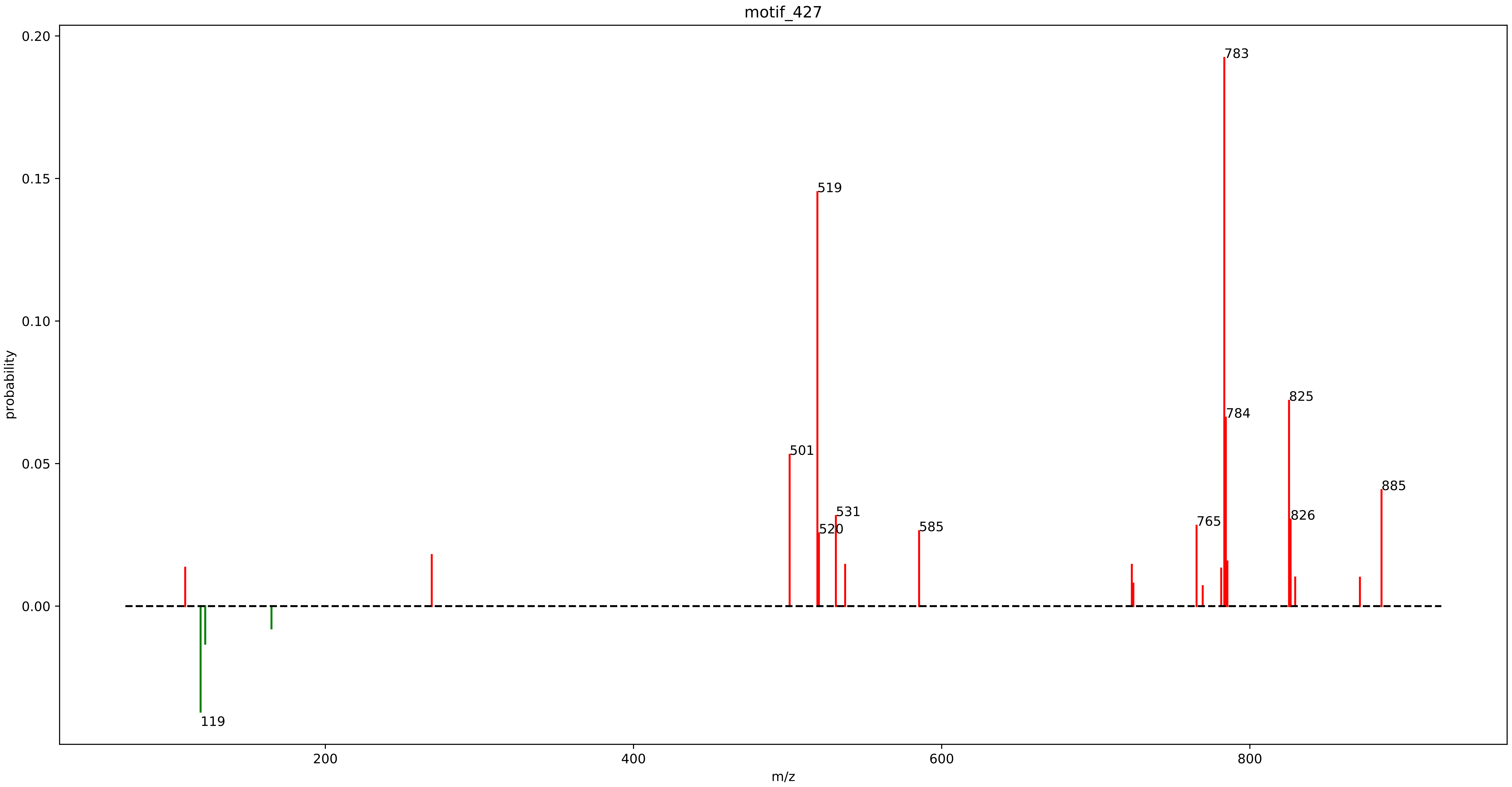

motif_427
probability
m/z
0.20
0.15
0.10
0.05
0.00
200
400
600
800
783
519
825
784
501
885
531
826
765
520
585
119

motif_427

fragment_783.4175	: 0.19
fragment_519.3325	: 0.15
fragment_825.4275	: 0.07
fragment_784.4175	: 0.07
fragment_501.3225	: 0.05
fragment_885.4475	: 0.04
loss_119.0375	: 0.04
fragment_531.3325	: 0.03
fragment_826.4275	: 0.03
fragment_765.4075	: 0.03
fragment_585.3425	: 0.03
fragment_520.3325	: 0.03
fragment_269.0475	: 0.02
fragment_785.4175	: 0.02
fragment_537.3425	: 0.01
fragment_723.3975	: 0.01
fragment_109.0425	: 0.01
fragment_781.4125	: 0.01
loss_122.0425	: 0.01
fragment_826.4325	: 0.01
fragment_829.4225	: 0.01
fragment_871.4325	: 0.01
fragment_724.3975	: 0.01
loss_165.0425	: 0.01
fragment_769.4025	: 0.01

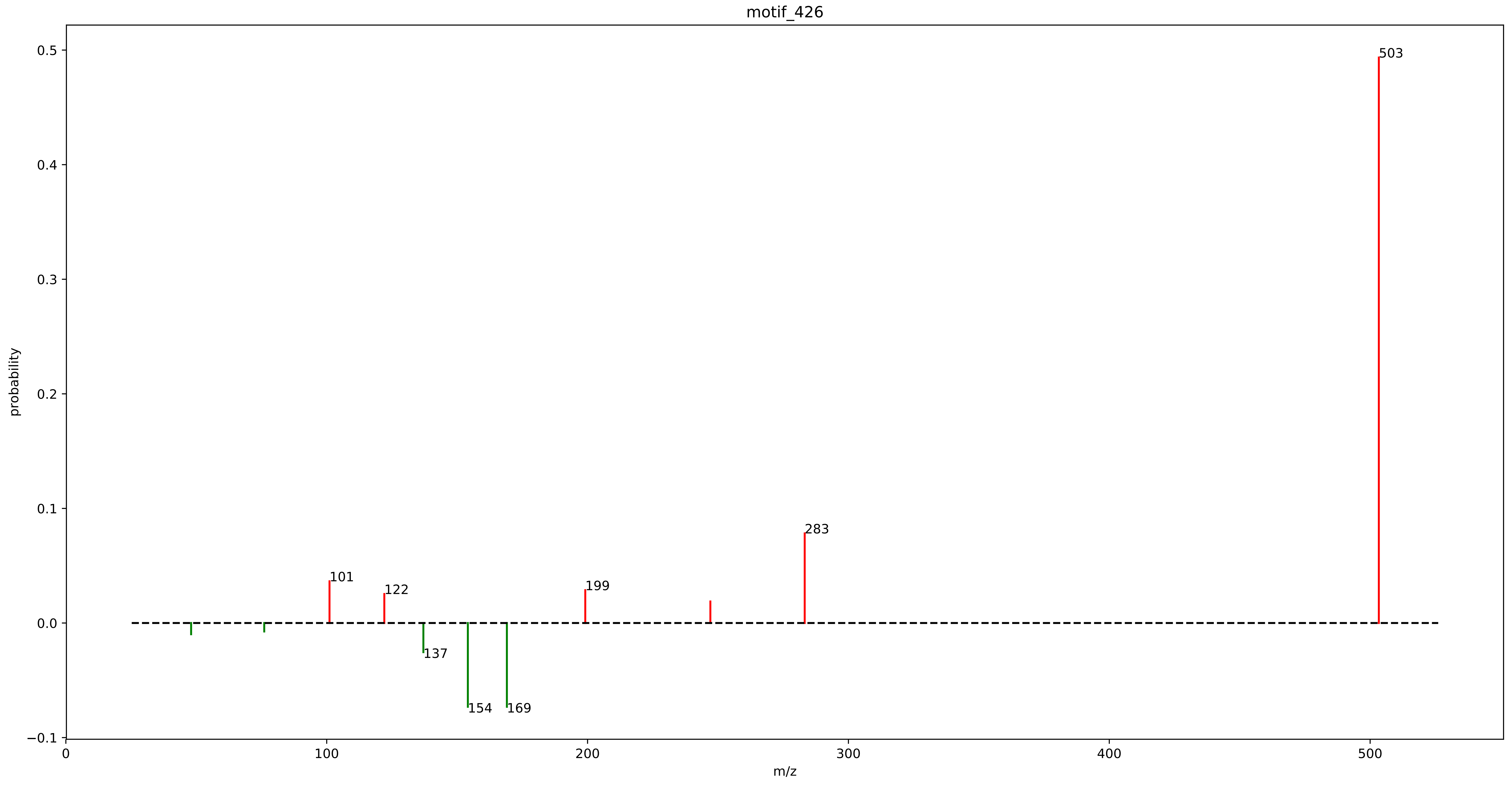
motif_426
probability
m/z
0.5
0.4
0.3
0.2
0.1
0.0
−0.1
0
100
200
300
400
500
503
283
101
122
199
137
154
169

motif_426

fragment_503.3375	: 0.49
fragment_283.2375	: 0.08
loss_154.1075	: 0.07
loss_169.0725	: 0.07
fragment_101.0725	: 0.04
fragment_199.1225	: 0.03
loss_137.0575	: 0.03
fragment_122.0825	: 0.03
fragment_247.0775	: 0.02
loss_48.0175	: 0.01
loss_76.0525	: 0.01

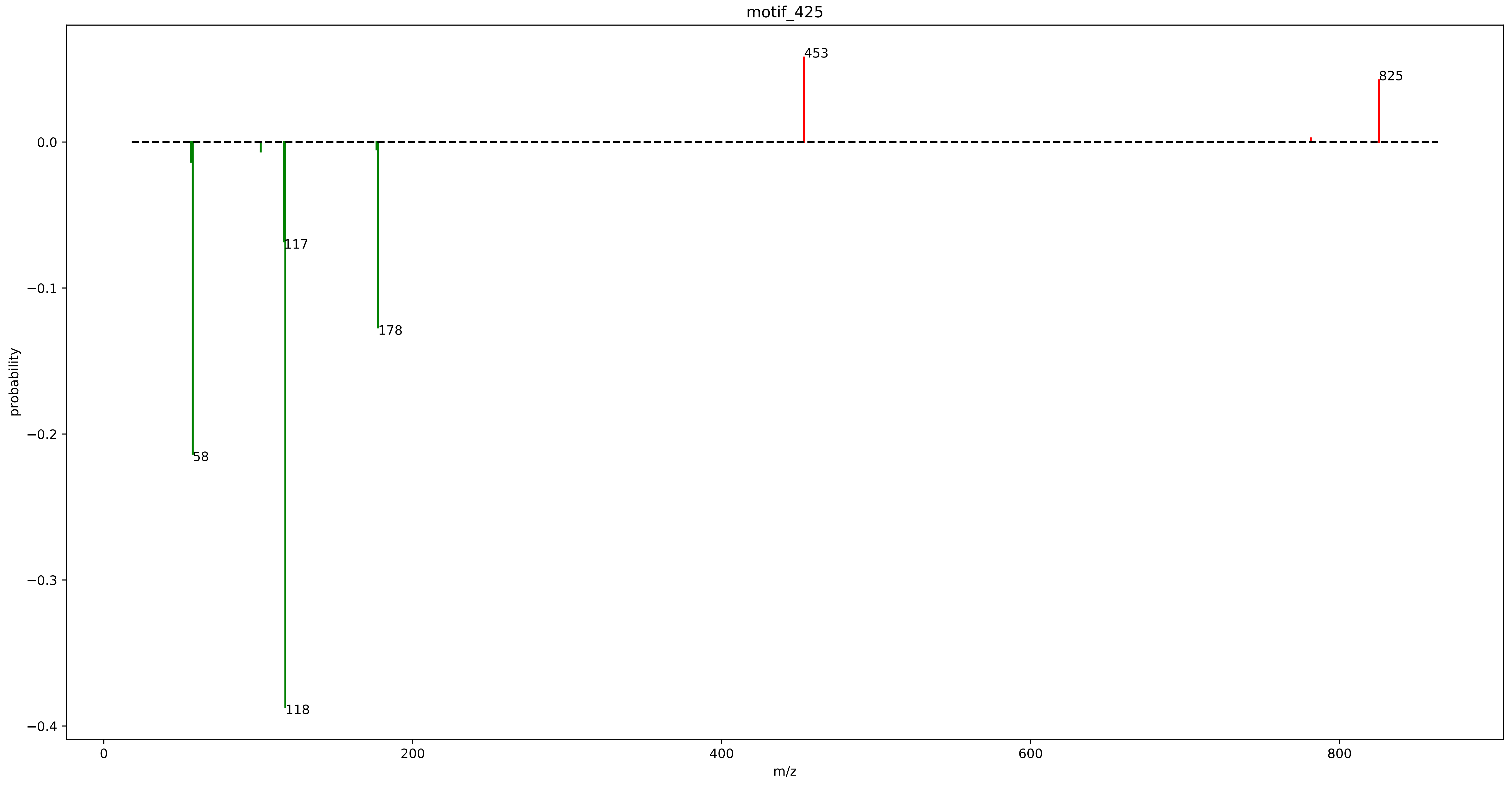

motif_425
453
825
0.0
117
−0.1
178
probability
−0.2
58
−0.3
118
−0.4
0
200
400
600
800
m/z

motif_425

loss_117.5325	: 0.39
loss_57.5125	: 0.21
loss_177.5525	: 0.13
loss_116.5275	: 0.07
fragment_453.3375	: 0.06
fragment_825.4375	: 0.04
loss_56.5075	: 0.01
loss_101.5675	: 0.01
loss_176.5475	: 0.01

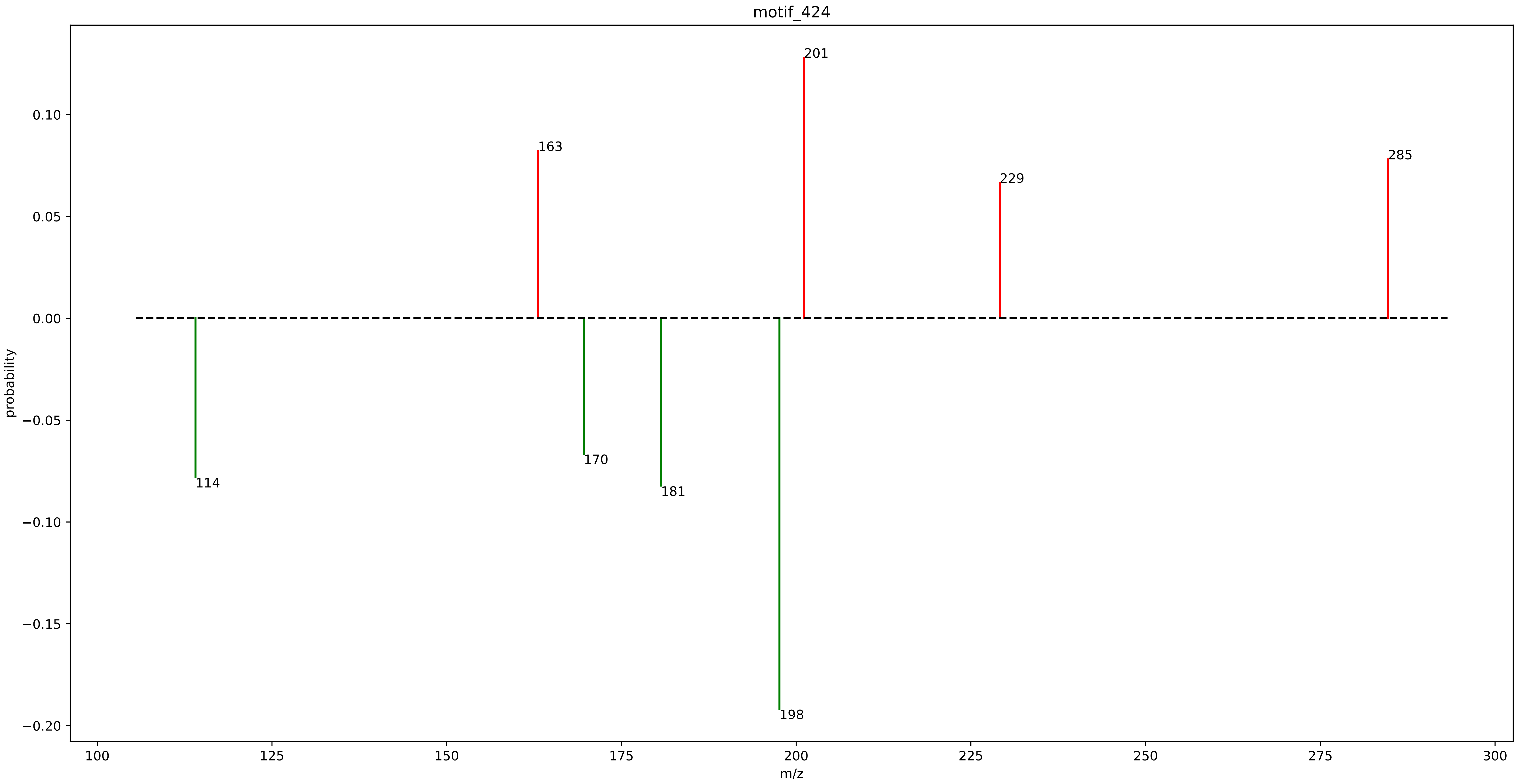

motif_424
201
163
285
229
114
170
181
198
0.10
0.05
0.00
−0.05
−0.10
−0.15
−0.20
probability
100
125
150
175
200
225
250
275
300
m/z

motif_424

loss_197.6075	: 0.19
fragment_201.1225	: 0.13
fragment_163.0725	: 0.08
loss_180.6525	: 0.08
fragment_284.6725	: 0.08
loss_114.0575	: 0.08
fragment_229.1225	: 0.07
loss_169.6075	: 0.07

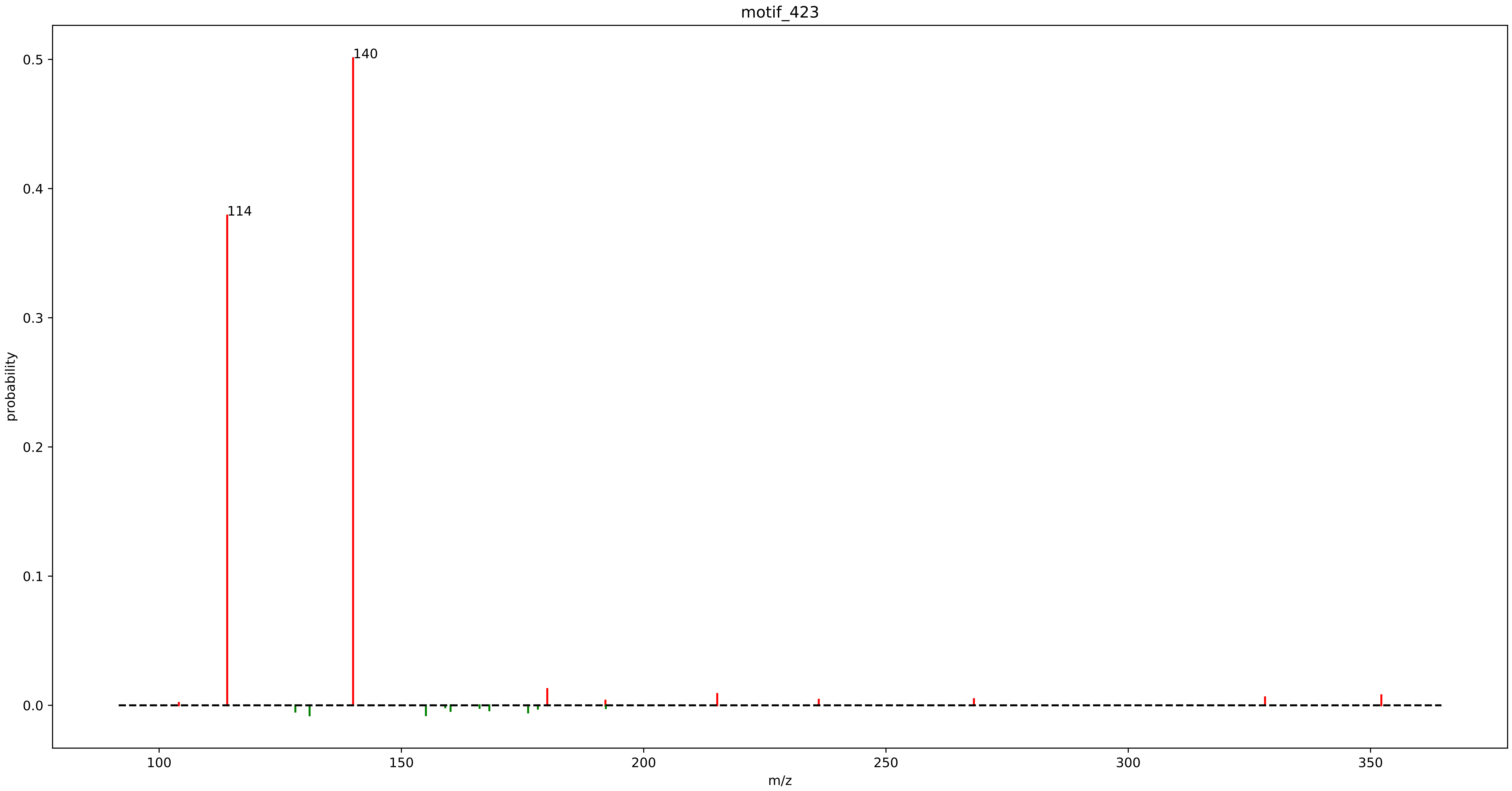

motif_423
0.5
0.4
0.3
0.2
0.1
0.0
probability
140
114
100
150
200
250
300
350
m/z

motif_423

fragment_140.0325	: 0.50
fragment_114.0525	: 0.38
fragment_180.1025	: 0.01
fragment_215.1775	: 0.01
fragment_352.2275	: 0.01
loss_131.0575	: 0.01
loss_155.0575	: 0.01
fragment_328.2275	: 0.01
loss_176.1525	: 0.01

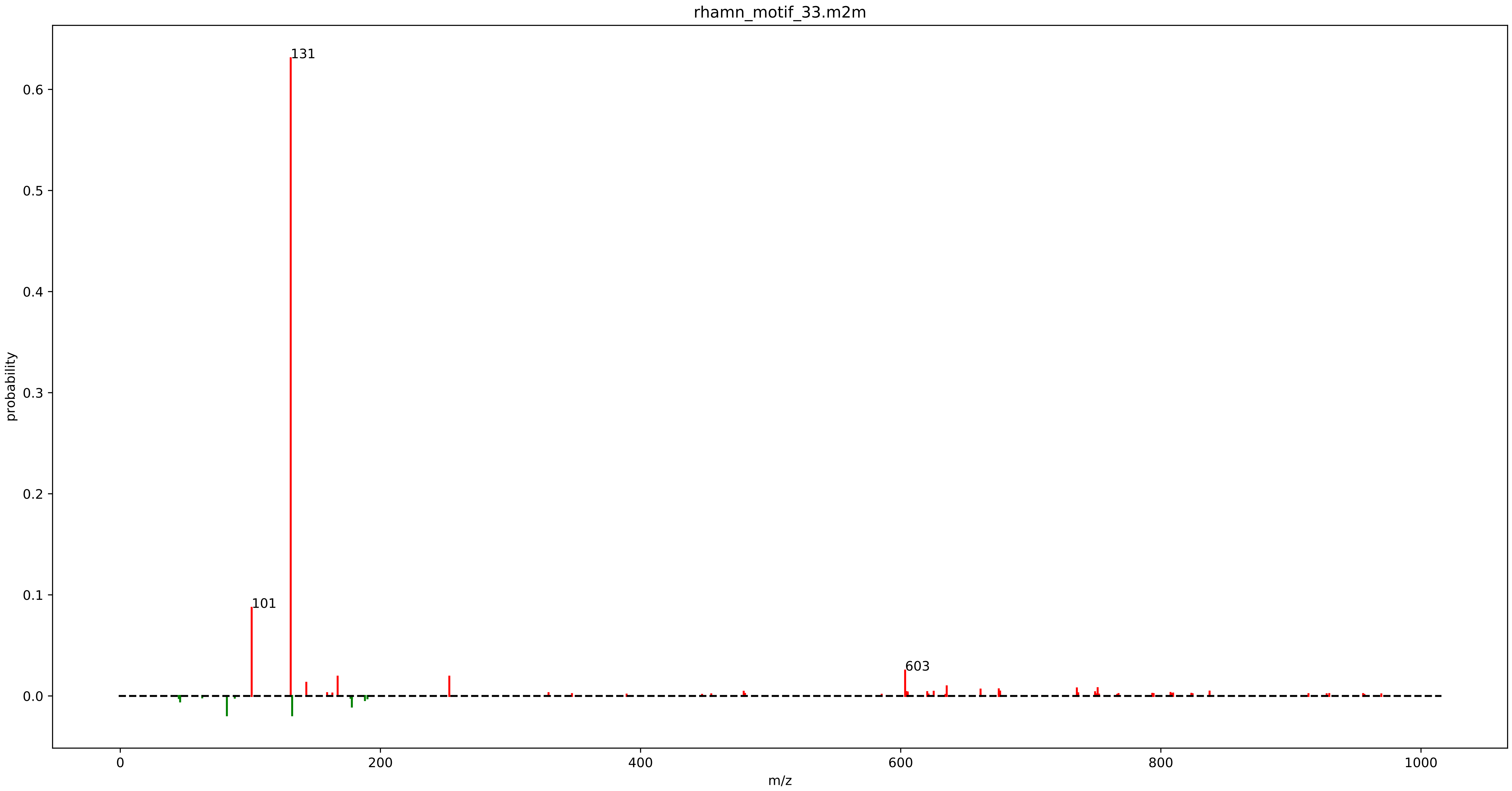

rhamn_motif_33.m2m
probability
m/z
131
101
603
0.6
0.5
0.4
0.3
0.2
0.1
0.0
0
200
400
600
800
1000

rhamn_motif_33.m2m

fragment_131.0325	: 0.63
fragment_101.0225	: 0.09
fragment_603.3925	: 0.03
loss_132.1375	: 0.02
fragment_167.1125	: 0.02
loss_81.9675	: 0.02
fragment_252.9725	: 0.02
fragment_143.0325	: 0.01
loss_178.0425	: 0.01
fragment_635.3725	: 0.01
fragment_751.4225	: 0.01
fragment_735.4275	: 0.01
fragment_675.3975	: 0.01
fragment_661.3975	: 0.01
loss_46.0125	: 0.01

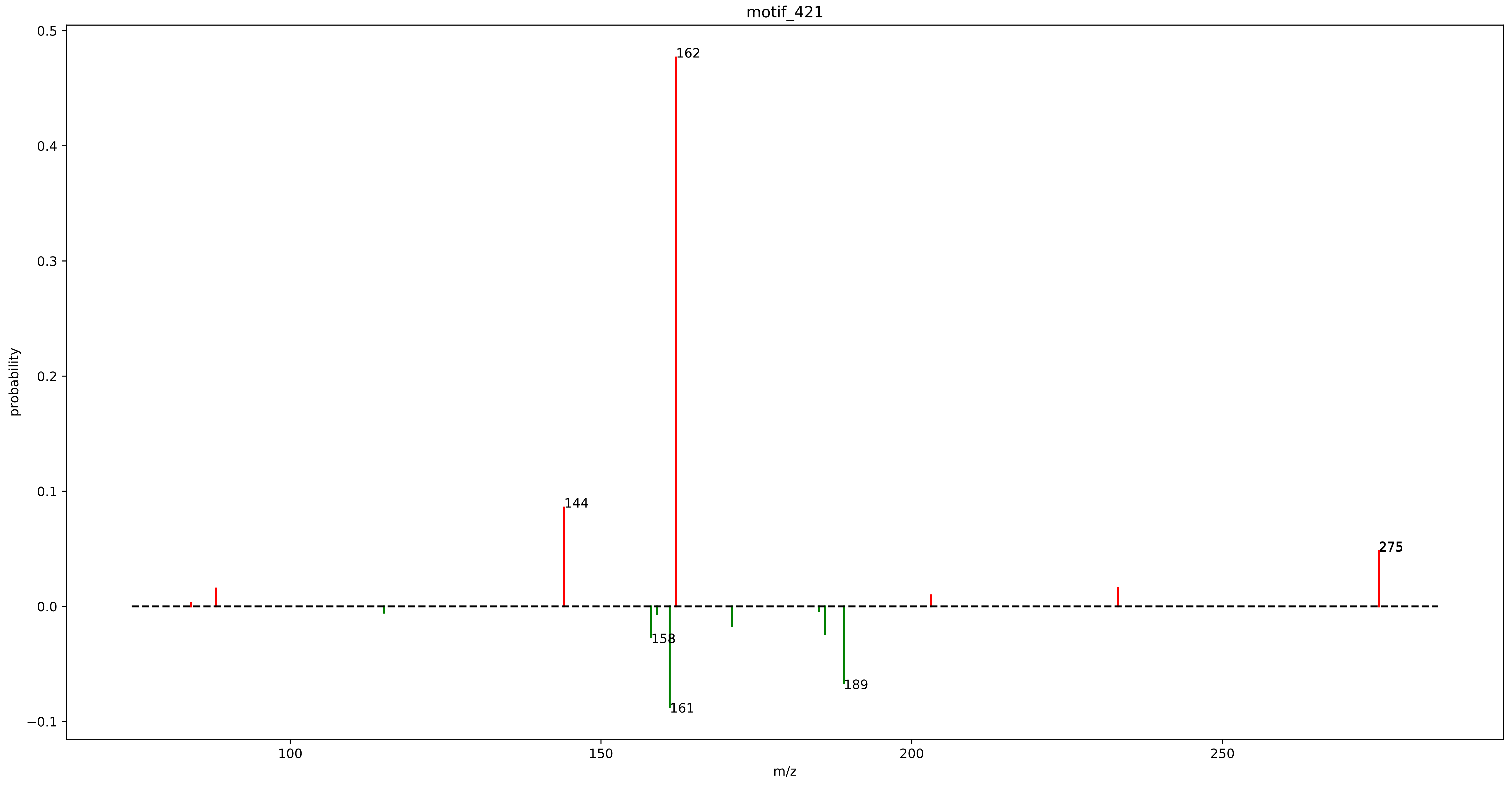

motif_421
probability
m/z
162
144
275
158
161
189
0.5
0.4
0.3
0.2
0.1
0.0
−0.1
100
150
200
250

motif_421

fragment_162.0725	: 0.48
loss_161.0675	: 0.09
fragment_144.0675	: 0.09
loss_189.0625	: 0.07
fragment_275.1625	: 0.05
fragment_275.1575	: 0.05
loss_158.0675	: 0.03
loss_186.0625	: 0.02
loss_171.0875	: 0.02
fragment_233.1625	: 0.02
fragment_88.0725	: 0.02
fragment_203.1375	: 0.01
loss_159.0525	: 0.01
loss_115.0975	: 0.01

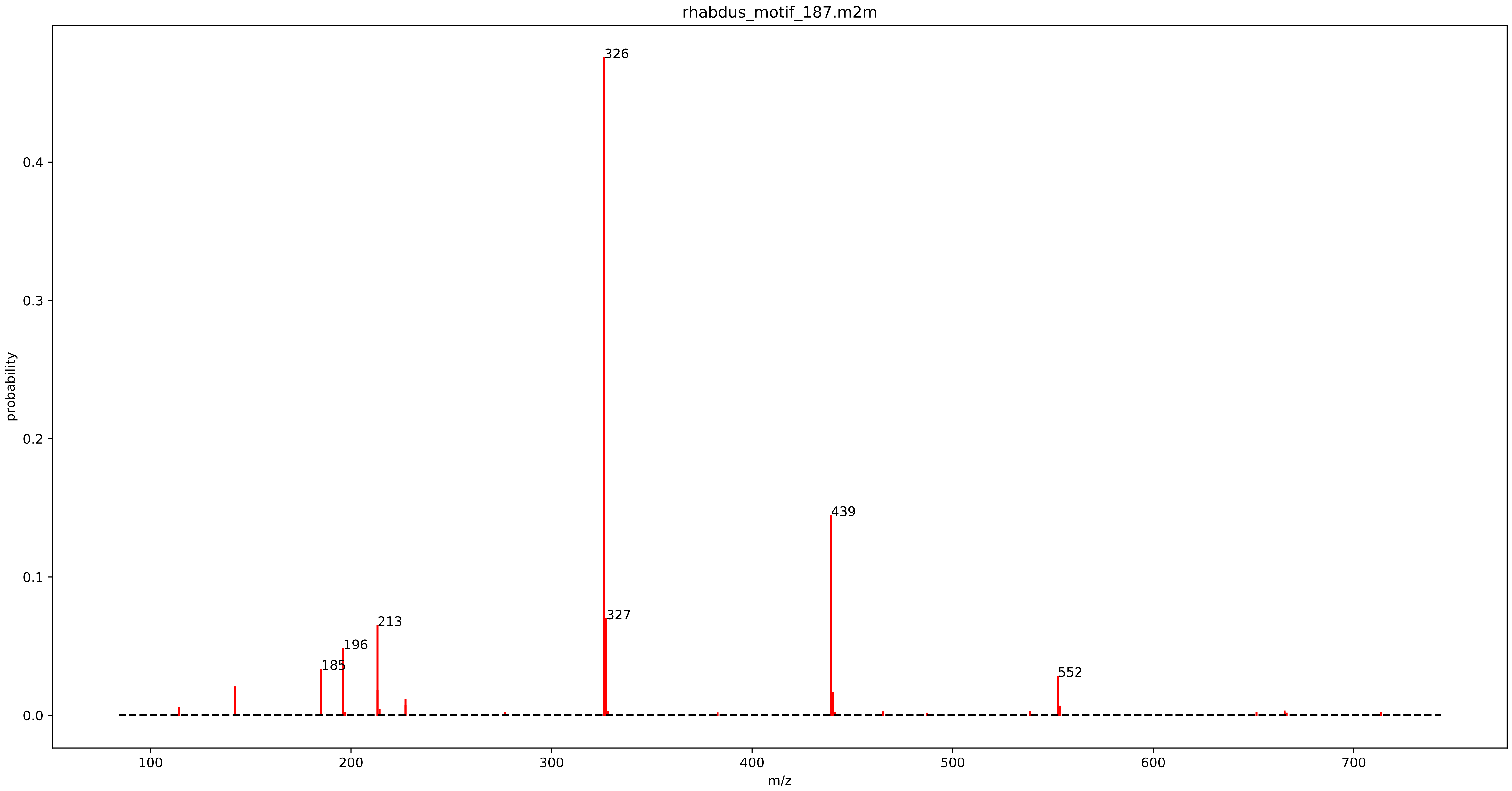

rhabdus_motif_187.m2m
probability
m/z
0.0
0.1
0.2
0.3
0.4
100
200
300
400
500
600
700
326
327
439
213
196
185
552

rhabdus_motif_187.m2m

fragment_326.2425	: 0.48
fragment_439.3275	: 0.14
fragment_327.2475	: 0.07
fragment_213.1575	: 0.06
fragment_196.1325	: 0.05
fragment_185.1625	: 0.03
fragment_552.4125	: 0.03
fragment_142.0875	: 0.02
fragment_213.1625	: 0.02
fragment_440.3275	: 0.02
fragment_440.3325	: 0.01
fragment_227.1775	: 0.01
fragment_227.1725	: 0.01
fragment_553.4125	: 0.01
fragment_327.2425	: 0.01
fragment_114.0875	: 0.01

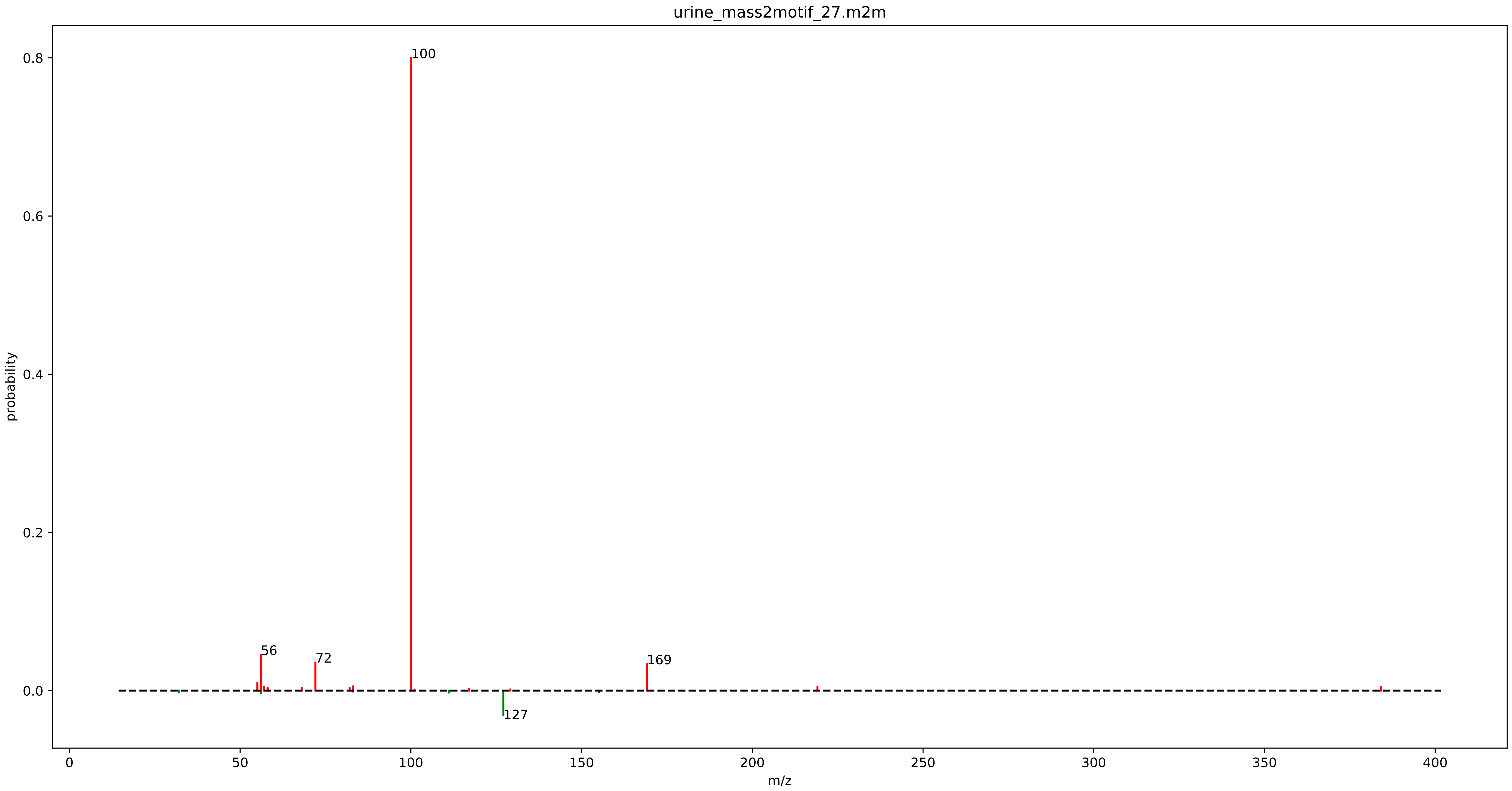
urine_mass2motif_27.m2m
probability
m/z
100
56
72
169
127
0.8
0.6
0.4
0.2
0.0
0
50
100
150
200
250
300
350
400

urine_mass2motif_27.m2m

fragment_100.0775	: 0.80
fragment_56.0475	: 0.05
fragment_72.0425	: 0.04
fragment_169.1325	: 0.03
loss_127.0975	: 0.03
fragment_55.0525	: 0.01
fragment_83.0725	: 0.01
fragment_57.0325	: 0.01

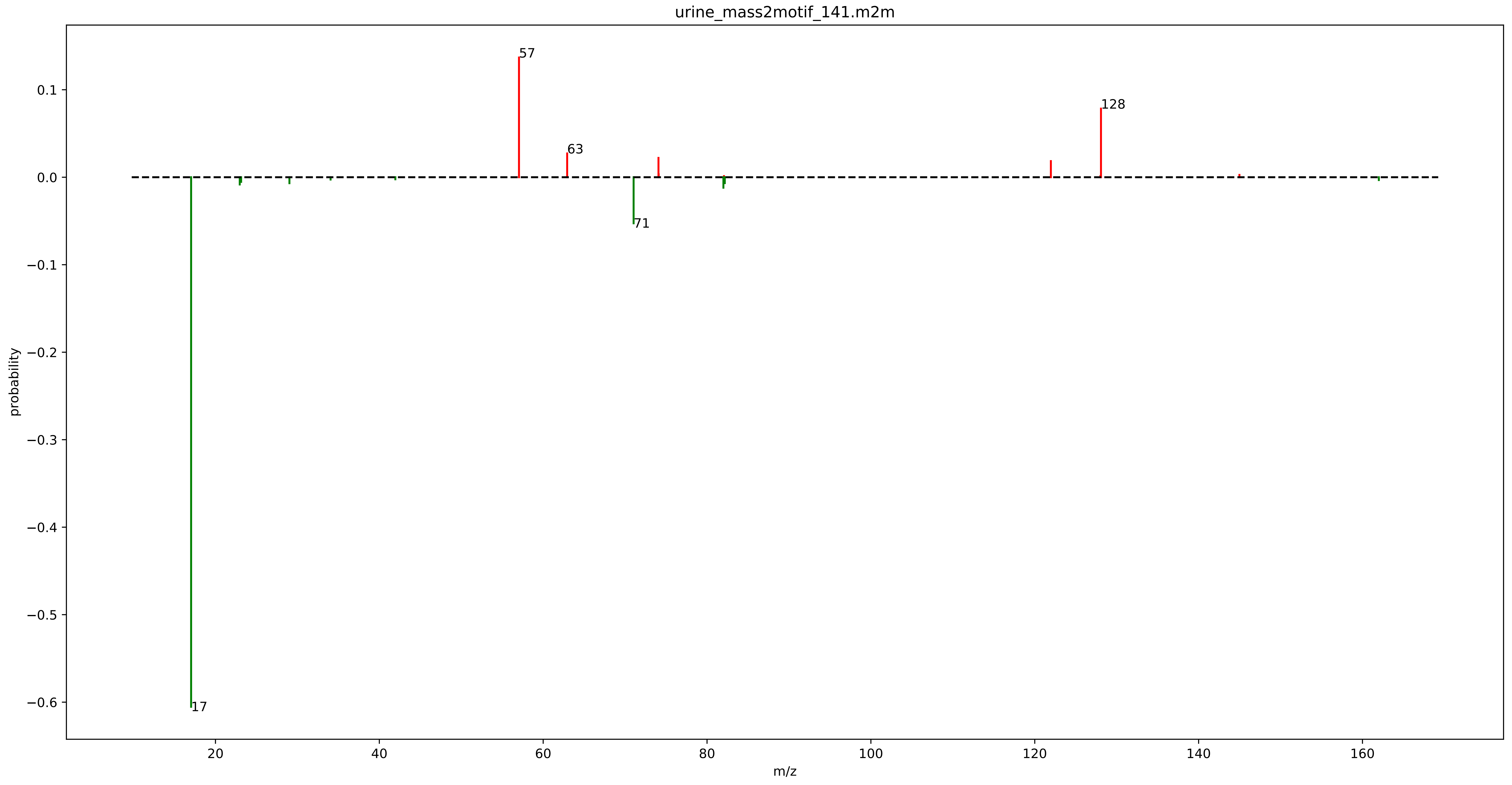
urine_mass2motif_141.m2m
probability
m/z
57
63
128
71
17
0.1
0.0
−0.1
−0.2
−0.3
−0.4
−0.5
−0.6
20
40
60
80
100
120
140
160

urine_mass2motif_141.m2m

loss_17.0275	: 0.61
fragment_57.0475	: 0.14
fragment_128.0825	: 0.08
loss_71.0375	: 0.05
fragment_62.9275	: 0.03
fragment_74.0725	: 0.02
fragment_121.9675	: 0.02
loss_81.9975	: 0.01
loss_22.9625	: 0.01
loss_29.0275	: 0.01
loss_82.1675	: 0.01
loss_23.1325	: 0.01

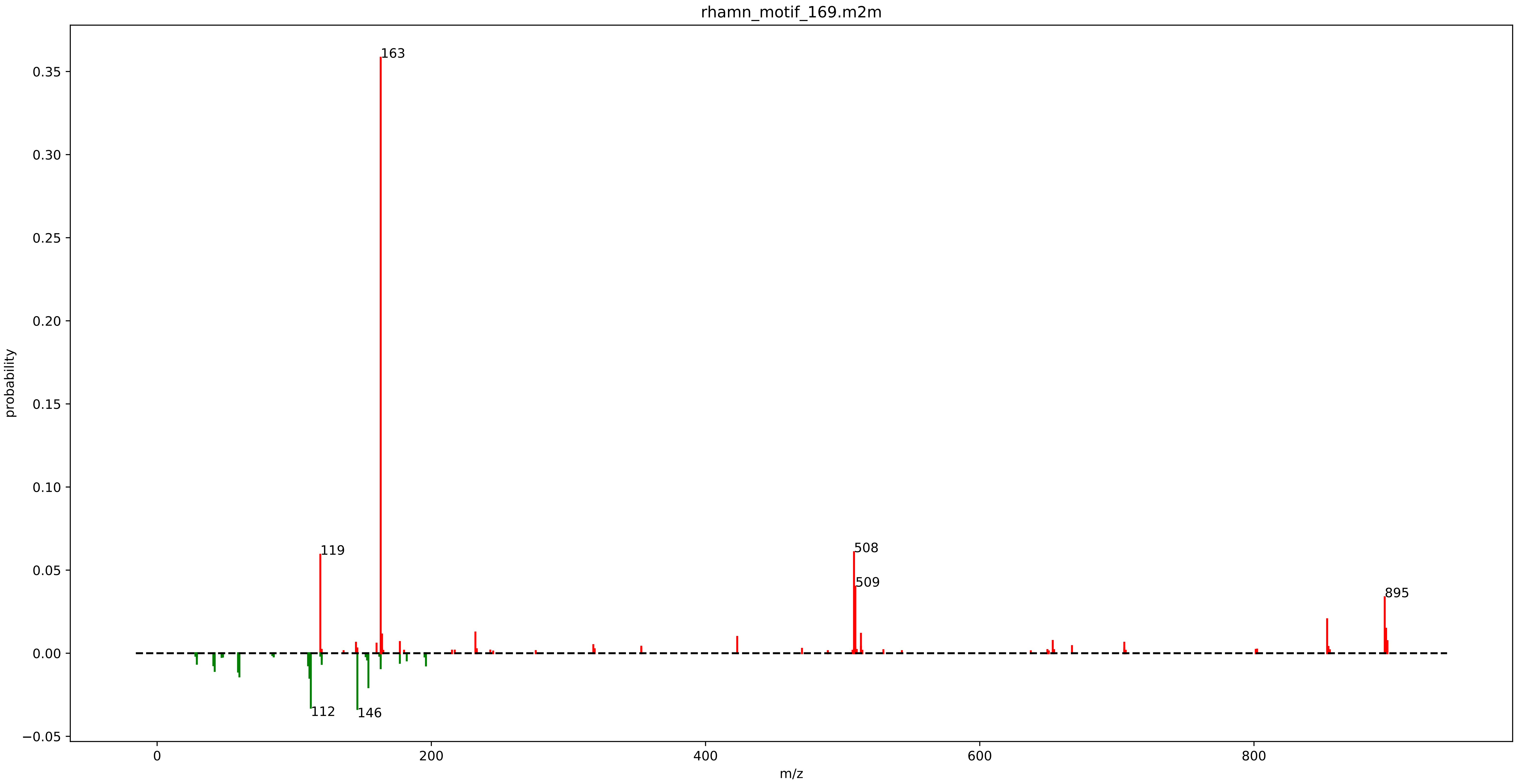

rhamn_motif_169.m2m
probability
m/z
0.35
0.30
0.25
0.20
0.15
0.10
0.05
0.00
−0.05
0
200
400
600
800
163
119
508
509
895
112
146

rhamn_motif_169.m2m

fragment_163.0425	: 0.36
fragment_508.2825	: 0.06
fragment_119.0475	: 0.06
fragment_509.2775	: 0.04
fragment_895.3625	: 0.03
loss_146.0425	: 0.03
loss_112.0925	: 0.03
fragment_853.3525	: 0.02
loss_154.1025	: 0.02
loss_111.0875	: 0.01
fragment_896.3625	: 0.01
loss_60.0275	: 0.01
fragment_232.1375	: 0.01
fragment_513.3225	: 0.01
fragment_164.0425	: 0.01
loss_59.0075	: 0.01
loss_41.9975	: 0.01
fragment_423.1025	: 0.01
loss_60.0025	: 0.01
loss_163.0425	: 0.01
fragment_653.2375	: 0.01
loss_196.0775	: 0.01
fragment_897.3625	: 0.01
loss_110.0925	: 0.01
loss_41.0075	: 0.01
fragment_177.0575	: 0.01
loss_120.0525	: 0.01
loss_28.9775	: 0.01
fragment_705.4125	: 0.01
fragment_145.0225	: 0.01
fragment_160.0525	: 0.01
loss_177.0425	: 0.01

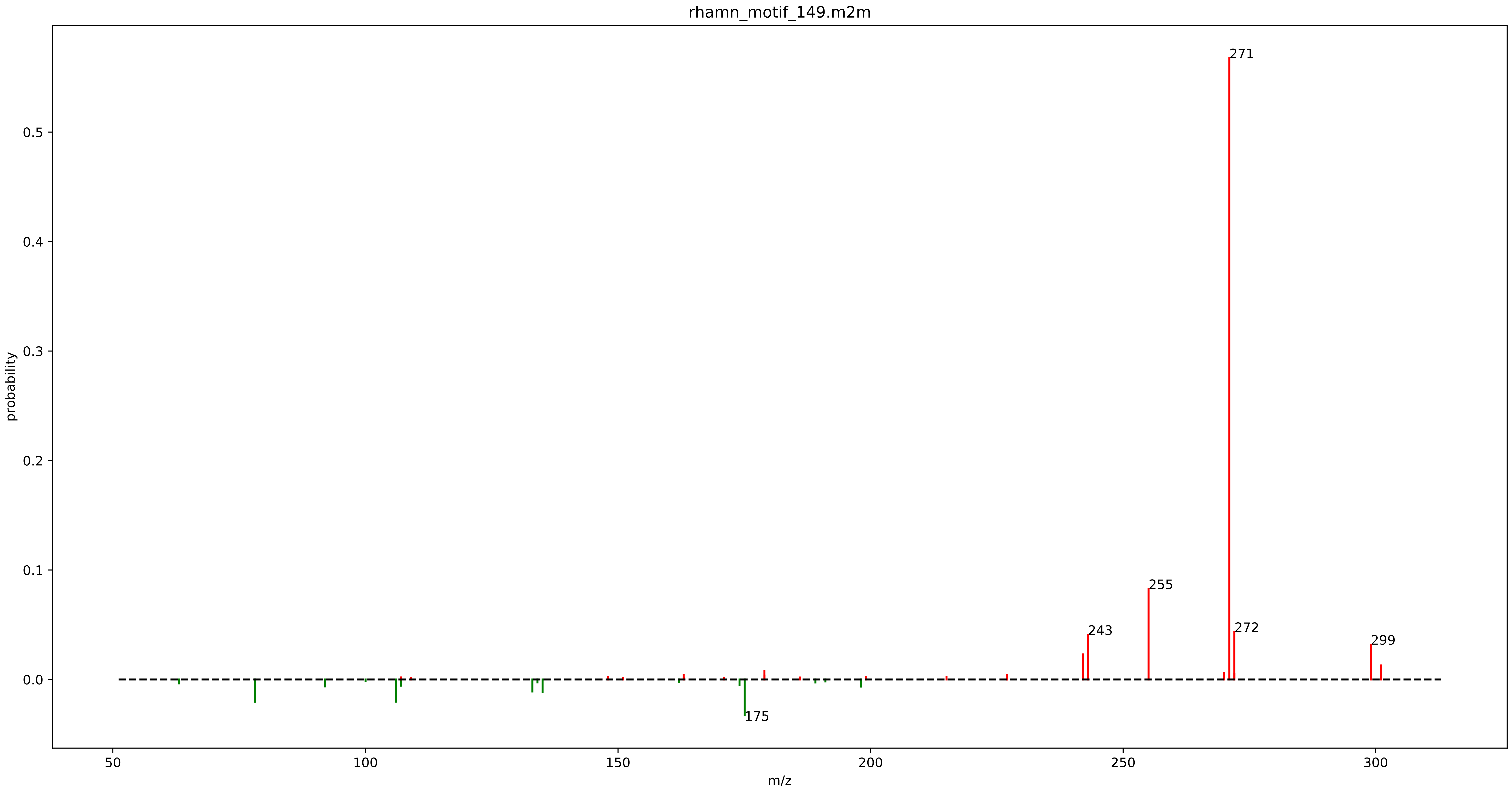

rhamn_motif_149.m2m
probability
m/z
0.5
0.4
0.3
0.2
0.1
0.0
50
100
150
200
250
300
271
255
243
272
299
175

rhamn_motif_149.m2m

fragment_271.0225	: 0.57
fragment_255.0275	: 0.08
fragment_272.0275	: 0.04
fragment_243.0275	: 0.04
loss_175.0625	: 0.03
fragment_299.0175	: 0.03
fragment_242.0225	: 0.02
loss_78.0675	: 0.02
loss_106.0625	: 0.02
fragment_243.0325	: 0.02
fragment_301.0275	: 0.01
loss_135.0675	: 0.01
loss_133.0475	: 0.01
fragment_178.9925	: 0.01
loss_198.0925	: 0.01
loss_92.0475	: 0.01
fragment_270.0125	: 0.01
loss_107.0725	: 0.01

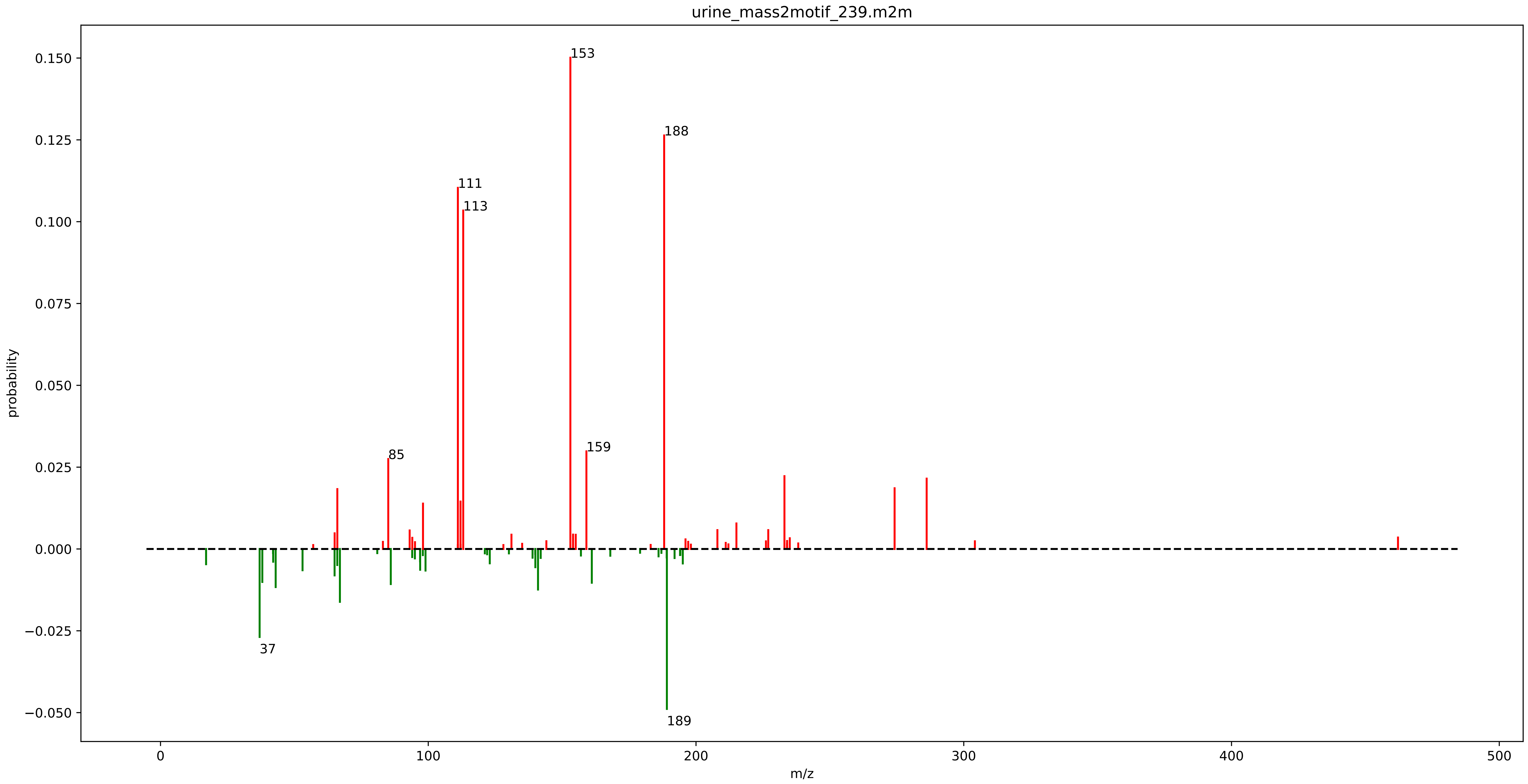

urine_mass2motif_239.m2m
0.150
0.125
0.100
0.075
0.050
0.025
0.000
−0.025
−0.050
probability
153
188
111
113
85
159
37
189
0
100
200
300
400
500
m/z

urine_mass2motif_239.m2m

fragment_153.0775	: 0.15
fragment_188.1025	: 0.13
fragment_111.0625	: 0.11
fragment_113.0575	: 0.10
loss_189.1225	: 0.05
fragment_159.0675	: 0.03
fragment_85.0625	: 0.03
loss_37.0175	: 0.03
fragment_233.0275	: 0.02
fragment_286.1425	: 0.02
fragment_274.1675	: 0.02
fragment_66.0425	: 0.02
loss_67.0025	: 0.02
fragment_112.0475	: 0.01
fragment_98.0375	: 0.01
loss_140.9925	: 0.01
loss_43.0125	: 0.01
fragment_274.1625	: 0.01
loss_86.0025	: 0.01
loss_161.0825	: 0.01
loss_38.0275	: 0.01
loss_65.0125	: 0.01
fragment_215.0925	: 0.01
loss_98.9775	: 0.01
loss_53.0475	: 0.01
loss_96.9775	: 0.01
fragment_207.9875	: 0.01
fragment_226.9825	: 0.01
fragment_93.0525	: 0.01
loss_139.9975	: 0.01

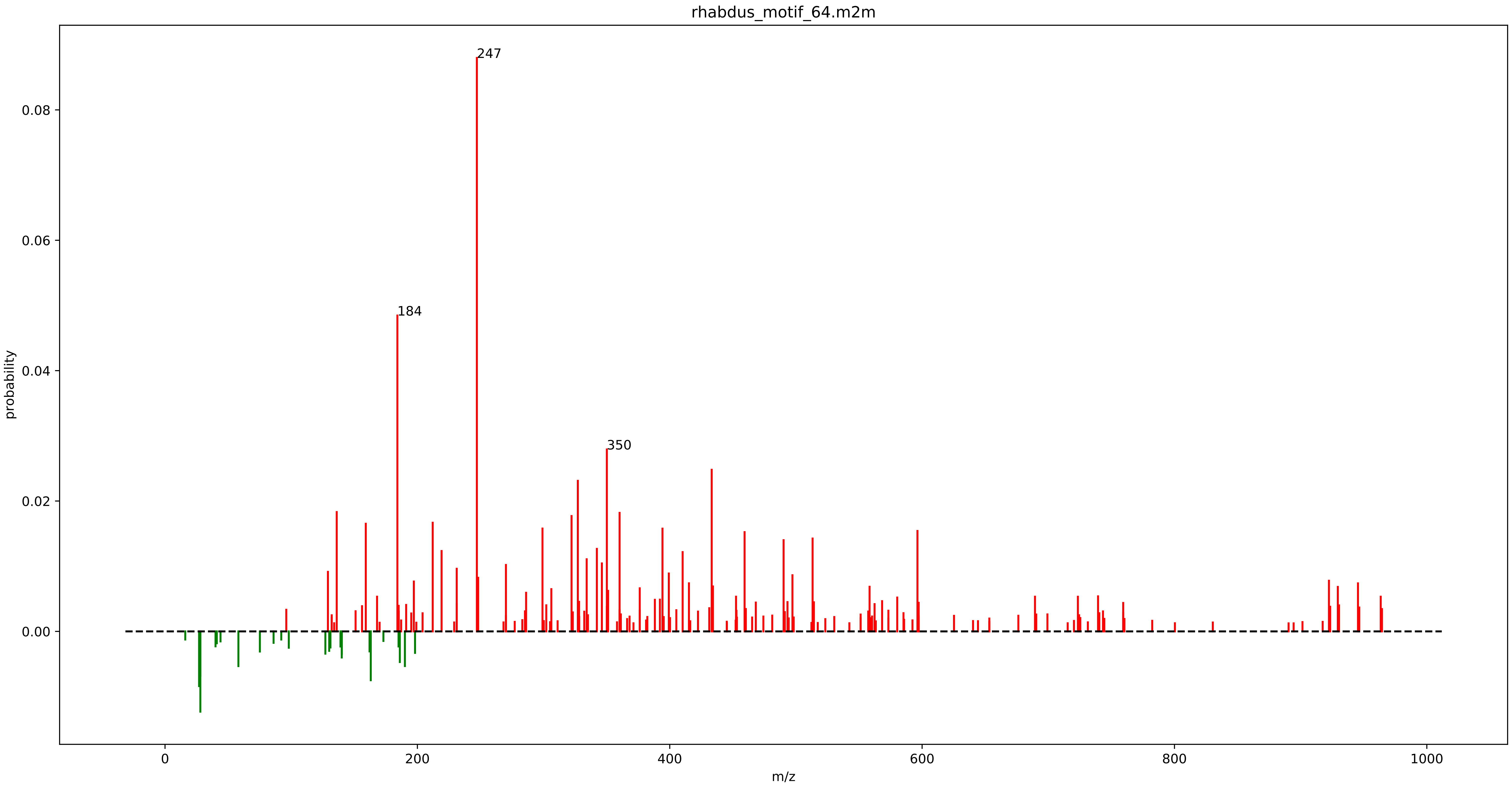

rhabdus_motif_64.m2m
247
184
350
0.08
0.06
0.04
0.02
0.00
probability
0
200
400
600
800
1000
m/z

rhabdus_motif_64.m2m

fragment_247.1425	: 0.09
fragment_184.1325	: 0.05
fragment_350.1475	: 0.03
fragment_433.2225	: 0.02
fragment_327.1325	: 0.02
fragment_136.0725	: 0.02
fragment_360.2275	: 0.02
fragment_322.1525	: 0.02
fragment_212.1275	: 0.02
fragment_159.0925	: 0.02
fragment_299.1375	: 0.02
fragment_394.2125	: 0.02
fragment_596.2875	: 0.02
fragment_459.2975	: 0.02
fragment_513.2125	: 0.01
fragment_490.1975	: 0.01
fragment_342.2175	: 0.01
fragment_219.1475	: 0.01
loss_27.9925	: 0.01
fragment_410.2075	: 0.01
fragment_334.1525	: 0.01
fragment_346.2125	: 0.01
fragment_270.1625	: 0.01
fragment_231.1475	: 0.01
fragment_129.1025	: 0.01
fragment_399.2375	: 0.01
fragment_497.2175	: 0.01
loss_26.9875	: 0.01
fragment_248.1475	: 0.01
fragment_922.4125	: 0.01
fragment_197.1625	: 0.01
loss_163.0575	: 0.01
fragment_415.2125	: 0.01
fragment_945.4275	: 0.01
fragment_434.2275	: 0.01
fragment_558.3625	: 0.01
fragment_929.4325	: 0.01
loss_27.9875	: 0.01
fragment_376.2025	: 0.01
fragment_306.1625	: 0.01
fragment_351.1525	: 0.01
fragment_286.1525	: 0.01
fragment_739.4175	: 0.01
fragment_452.5225	: 0.01
fragment_168.0325	: 0.01
loss_58.1475	: 0.01
fragment_689.4575	: 0.01
fragment_963.4375	: 0.01
fragment_723.4475	: 0.01
loss_190.1075	: 0.01
fragment_580.2925	: 0.01

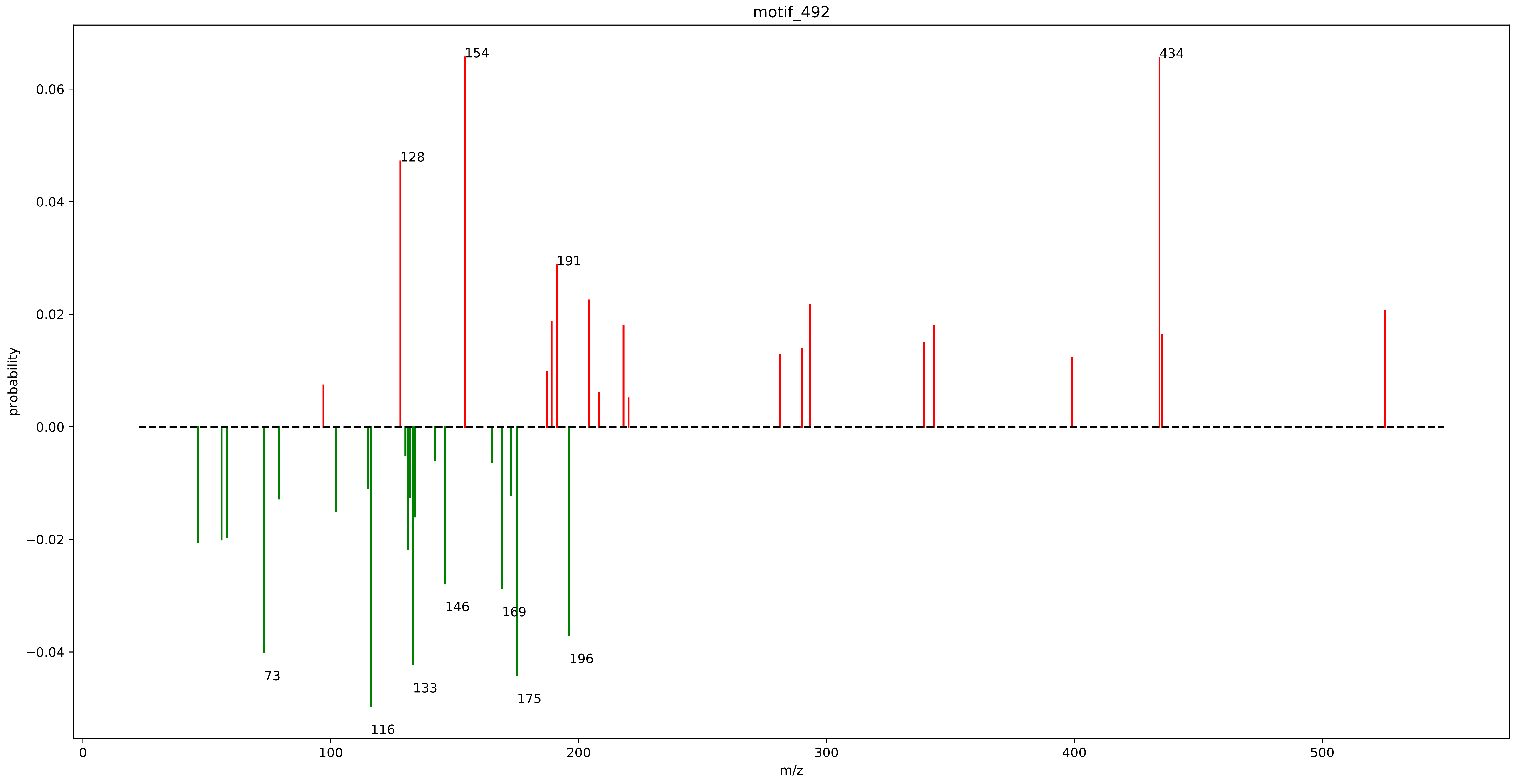

motif_492
154
434
128
191
73
116
133
146
169
175
196
0.06
0.04
0.02
0.00
−0.02
−0.04
probability
0
100
200
300
400
500
m/z

motif_492

fragment_154.0525	: 0.07	fragment_218.1175	: 0.02
fragment_434.3225	: 0.07	fragment_435.3275	: 0.02
loss_116.0825	: 0.05	loss_134.0925	: 0.02
fragment_128.0725	: 0.05	fragment_339.2125	: 0.01
loss_175.1825	: 0.04	loss_102.1175	: 0.01
loss_133.1725	: 0.04	fragment_290.1675	: 0.01
loss_73.1525	: 0.04	loss_79.0375	: 0.01
loss_196.1825	: 0.04	fragment_281.1725	: 0.01
fragment_191.1425	: 0.03	loss_132.1125	: 0.01
loss_169.0725	: 0.03	fragment_399.1525	: 0.01
loss_146.1275	: 0.03	loss_172.6475	: 0.01
fragment_204.1025	: 0.02	loss_131.0175	: 0.01
fragment_293.2075	: 0.02	loss_115.0775	: 0.01
loss_131.0575	: 0.02	fragment_187.1475	: 0.01
fragment_525.2825	: 0.02	fragment_97.0375	: 0.01
loss_46.5175	: 0.02	loss_165.2025	: 0.01
loss_55.9475	: 0.02	loss_142.1225	: 0.01
loss_57.9675	: 0.02	fragment_208.1125	: 0.01
fragment_189.1225	: 0.02	loss_130.0975	: 0.01
fragment_343.2625	: 0.02	fragment_220.1325	: 0.01

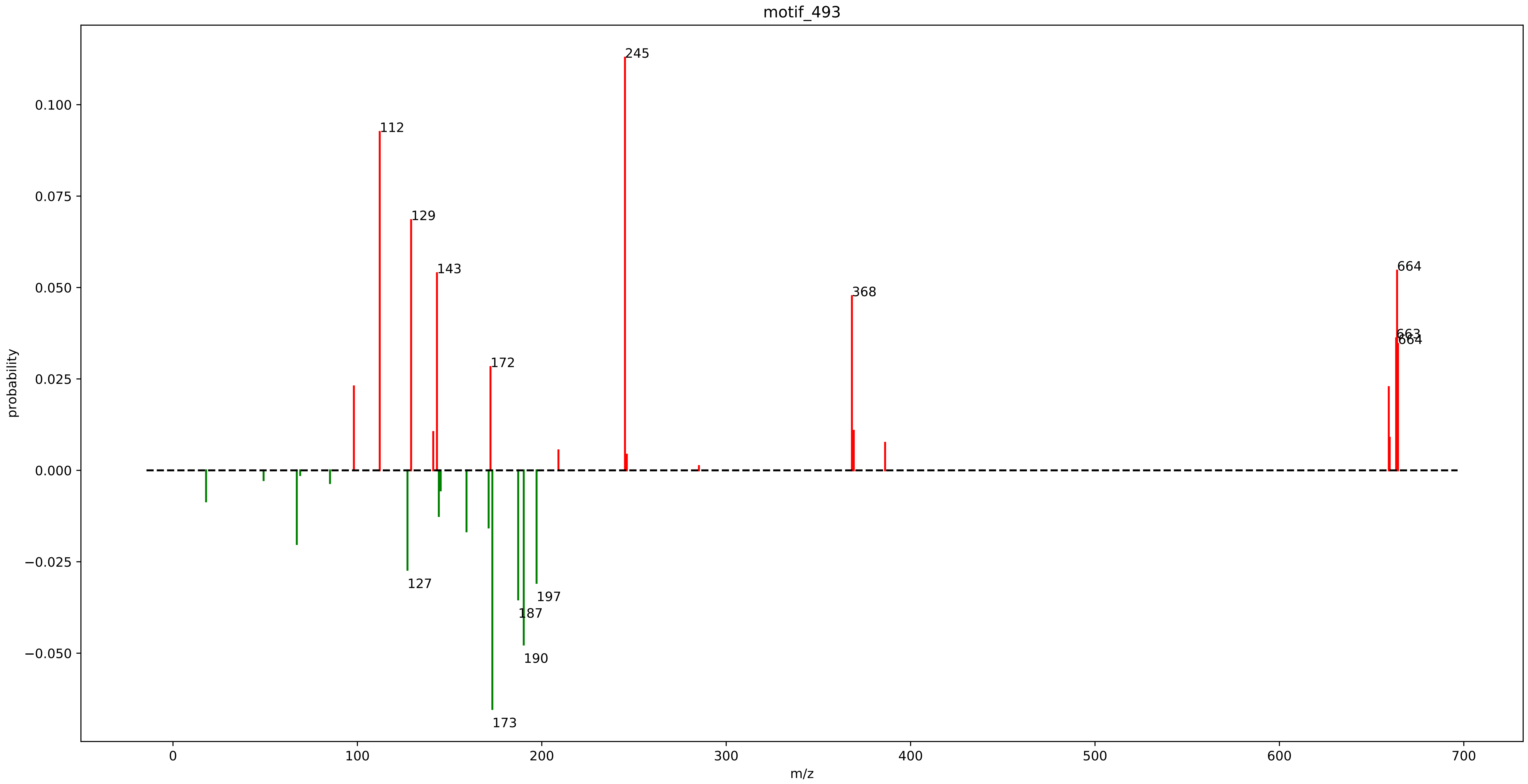

motif_493
probability
m/z
0.100
0.075
0.050
0.025
0.000
−0.025
−0.050
0
100
200
300
400
500
600
700
112
129
143
172
245
368
664
663
664
127
173
187
190
197

motif_493

fragment_245.1125	: 0.11
fragment_112.1125	: 0.09
fragment_129.1375	: 0.07
loss_173.1875	: 0.07
fragment_663.8125	: 0.05
fragment_143.1525	: 0.05
loss_190.2125	: 0.05
fragment_368.1775	: 0.05
fragment_663.3075	: 0.04
loss_187.2025	: 0.04
fragment_664.3125	: 0.03
loss_197.1675	: 0.03
fragment_172.1775	: 0.03
loss_127.1625	: 0.03
fragment_98.0975	: 0.02
fragment_659.3075	: 0.02
loss_67.1425	: 0.02
loss_159.1725	: 0.02
loss_171.1725	: 0.02
loss_144.1625	: 0.01
fragment_369.1825	: 0.01
fragment_141.1375	: 0.01
fragment_659.8075	: 0.01
loss_17.9475	: 0.01
fragment_386.1775	: 0.01
loss_145.1725	: 0.01
fragment_209.0275	: 0.01

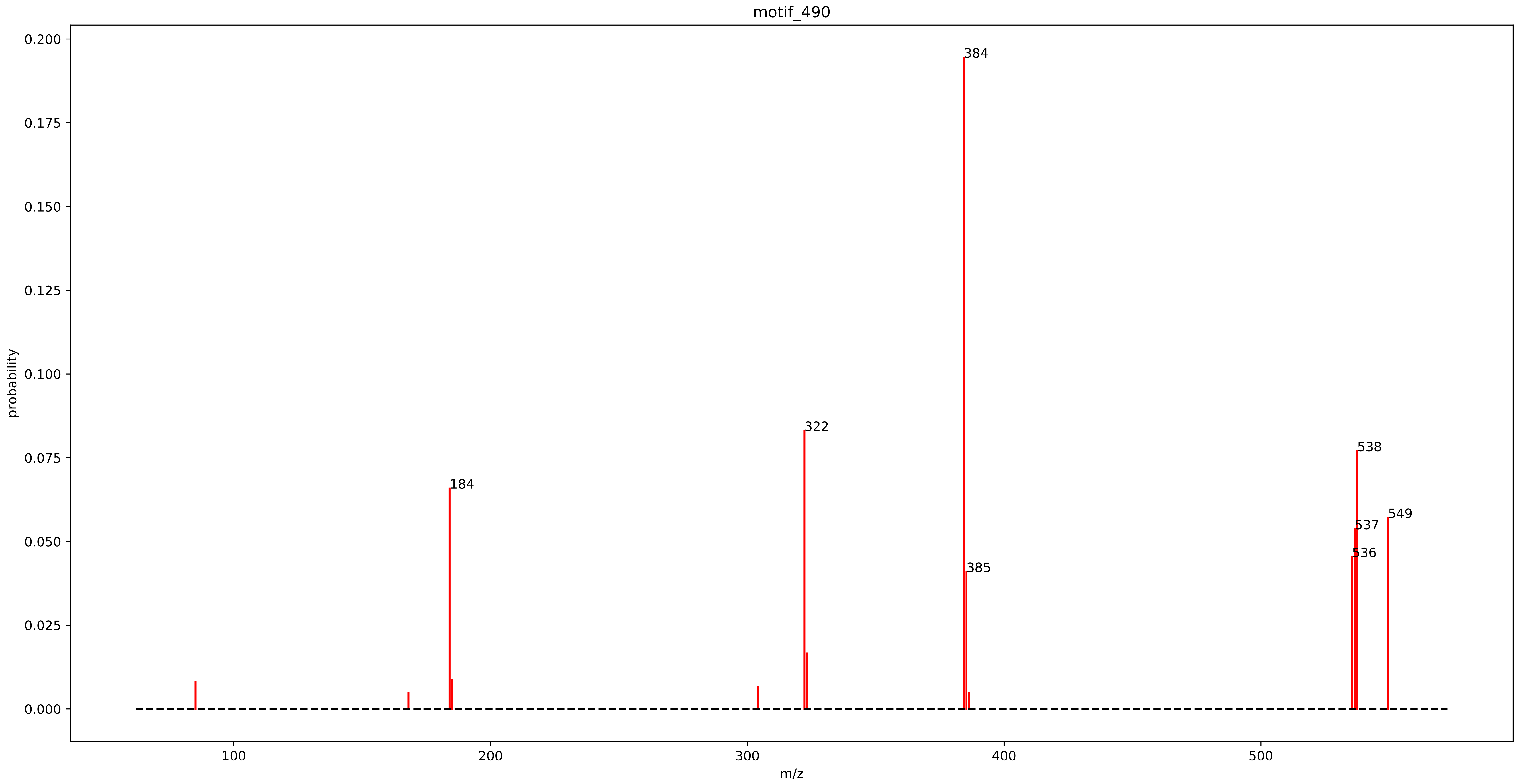

motif_490
probability
m/z
0.000
0.025
0.050
0.075
0.100
0.125
0.150
0.175
0.200
100
200
300
400
500
184
322
384
385
536
537
538
549

motif_490

fragment_384.3125	: 0.19
fragment_322.2375	: 0.08
fragment_537.5275	: 0.08
fragment_184.0725	: 0.07
fragment_549.4875	: 0.06
fragment_536.5375	: 0.05
fragment_535.5075	: 0.05
fragment_385.3125	: 0.04
fragment_535.4725	: 0.02
fragment_323.2425	: 0.02
fragment_185.0725	: 0.01
fragment_85.0975	: 0.01
fragment_304.2275	: 0.01

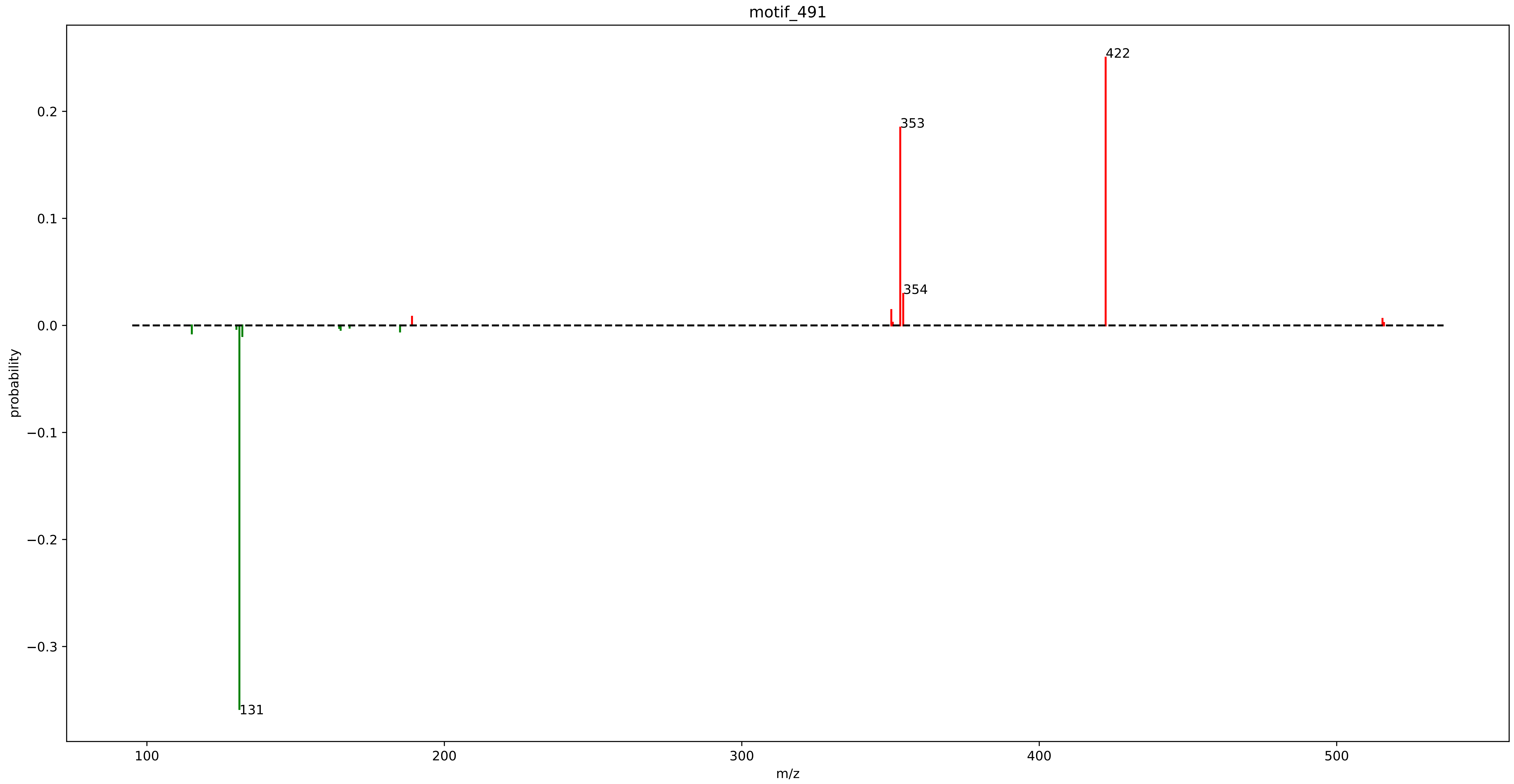

motif_491
422
353
354
131
0.2
0.1
0.0
−0.1
−0.2
−0.3
probability
100
200
300
400
500
m/z

motif_491

loss_131.0925	: 0.36
fragment_422.3225	: 0.25
fragment_353.2675	: 0.18
fragment_354.2675	: 0.03
fragment_350.2625	: 0.01
loss_132.0775	: 0.01
fragment_189.1225	: 0.01
loss_115.0975	: 0.01
fragment_515.3975	: 0.01
loss_185.1025	: 0.01

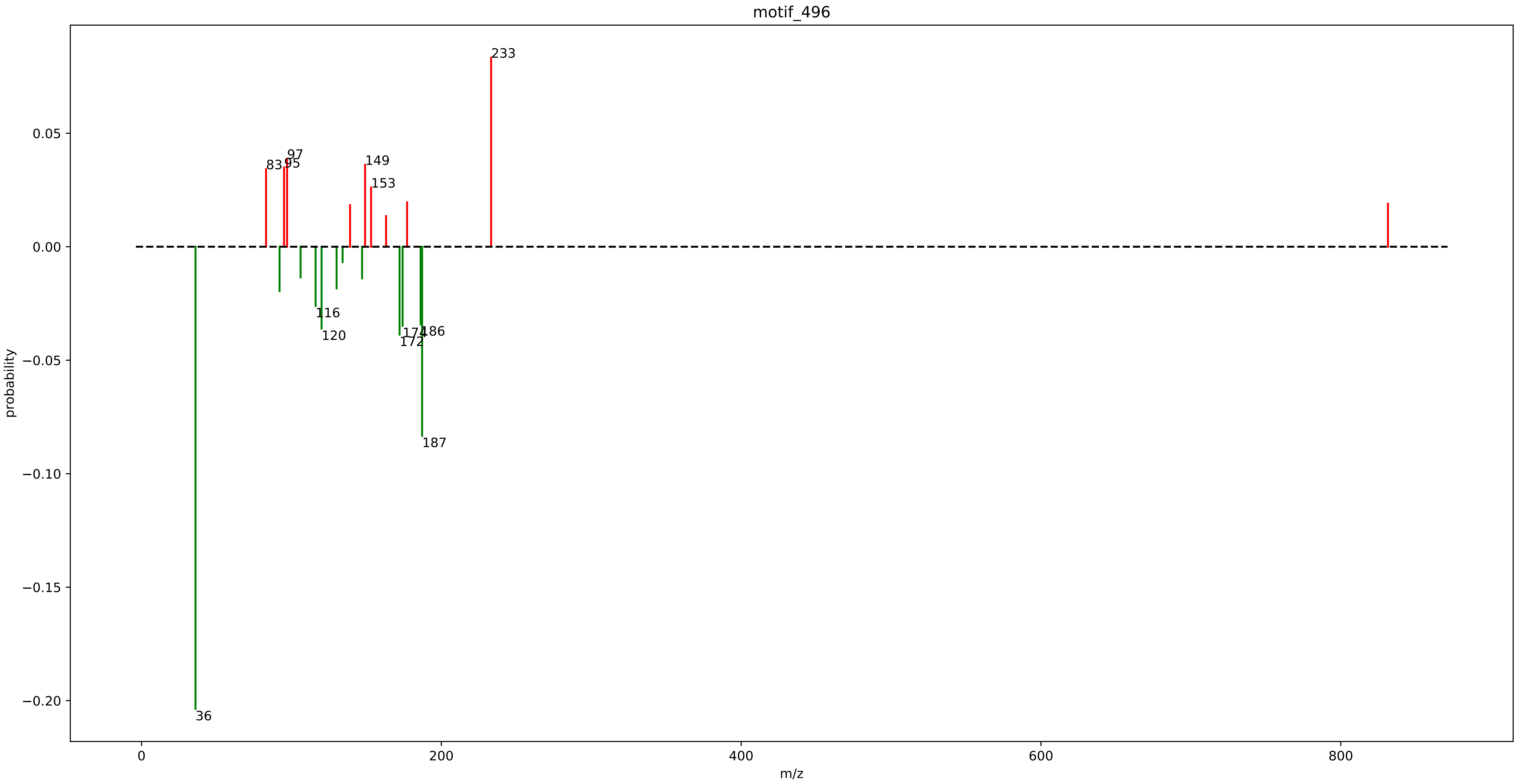

motif_496
233
97
83
95
149
153
116
120
174
186
172
187
36
0.05
0.00
−0.05
−0.10
−0.15
−0.20
probability
0
200
400
600
800
m/z

motif_496

loss_36.0175	: 0.20
fragment_233.2275	: 0.08
loss_187.1825	: 0.08
loss_172.1425	: 0.04
fragment_97.1025	: 0.04
fragment_149.1325	: 0.04
loss_120.1125	: 0.04
fragment_95.0825	: 0.03
loss_174.1625	: 0.03
loss_186.1625	: 0.03
fragment_83.0825	: 0.03
loss_116.1175	: 0.03
fragment_153.1275	: 0.03
fragment_177.1625	: 0.02
loss_92.0825	: 0.02
fragment_831.4425	: 0.02
fragment_139.1125	: 0.02
loss_130.1325	: 0.02
loss_147.1325	: 0.01
fragment_163.1475	: 0.01
loss_106.0975	: 0.01
loss_134.1375	: 0.01

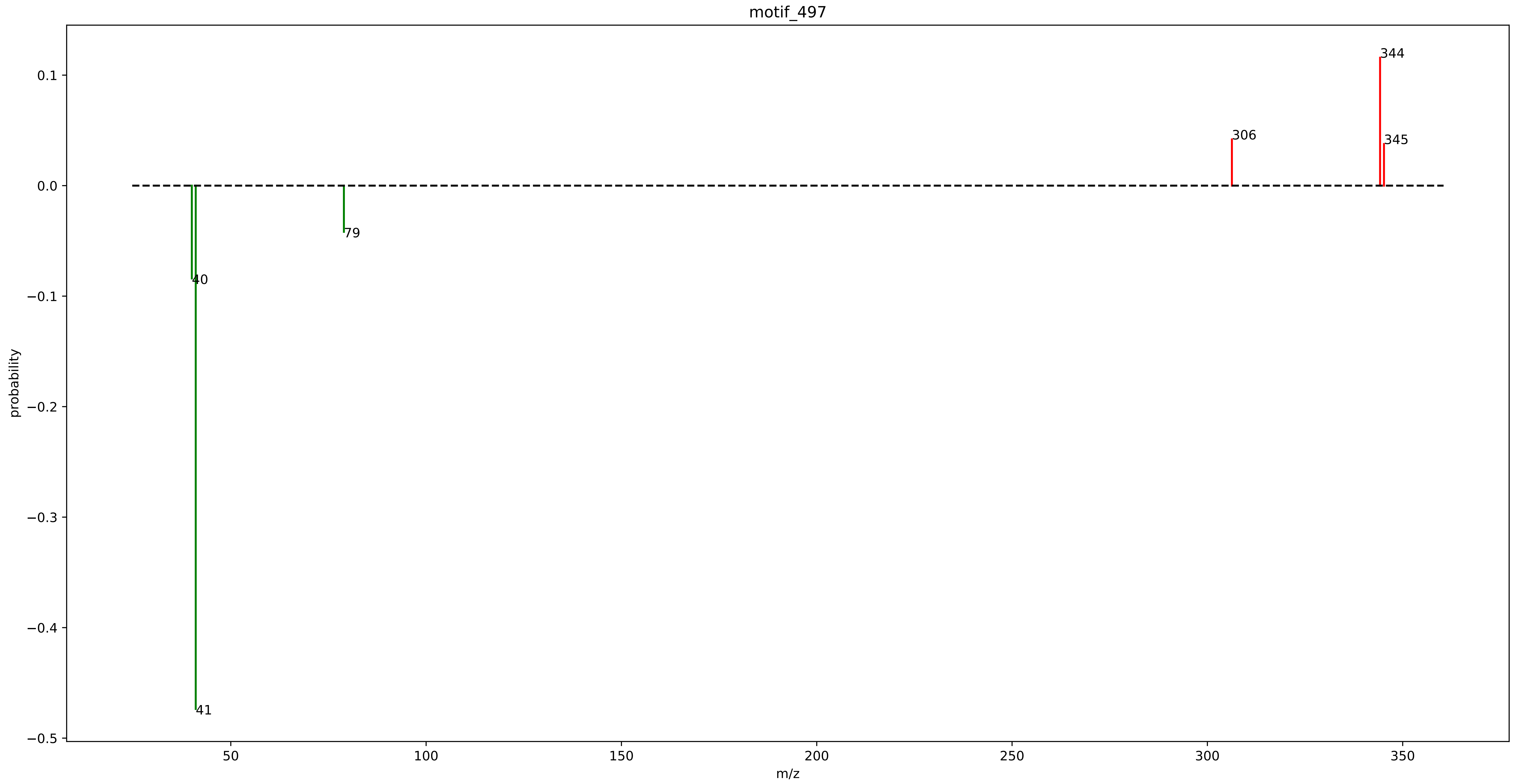
motif_497
probability
m/z
344
306
345
40
79
41
0.1
0.0
−0.1
−0.2
−0.3
−0.4
−0.5
50
100
150
200
250
300
350

motif_497

loss_41.0225	: 0.47
fragment_344.2075	: 0.12
loss_40.0225	: 0.08
loss_78.9525	: 0.04
fragment_306.2775	: 0.04
fragment_345.2075	: 0.04

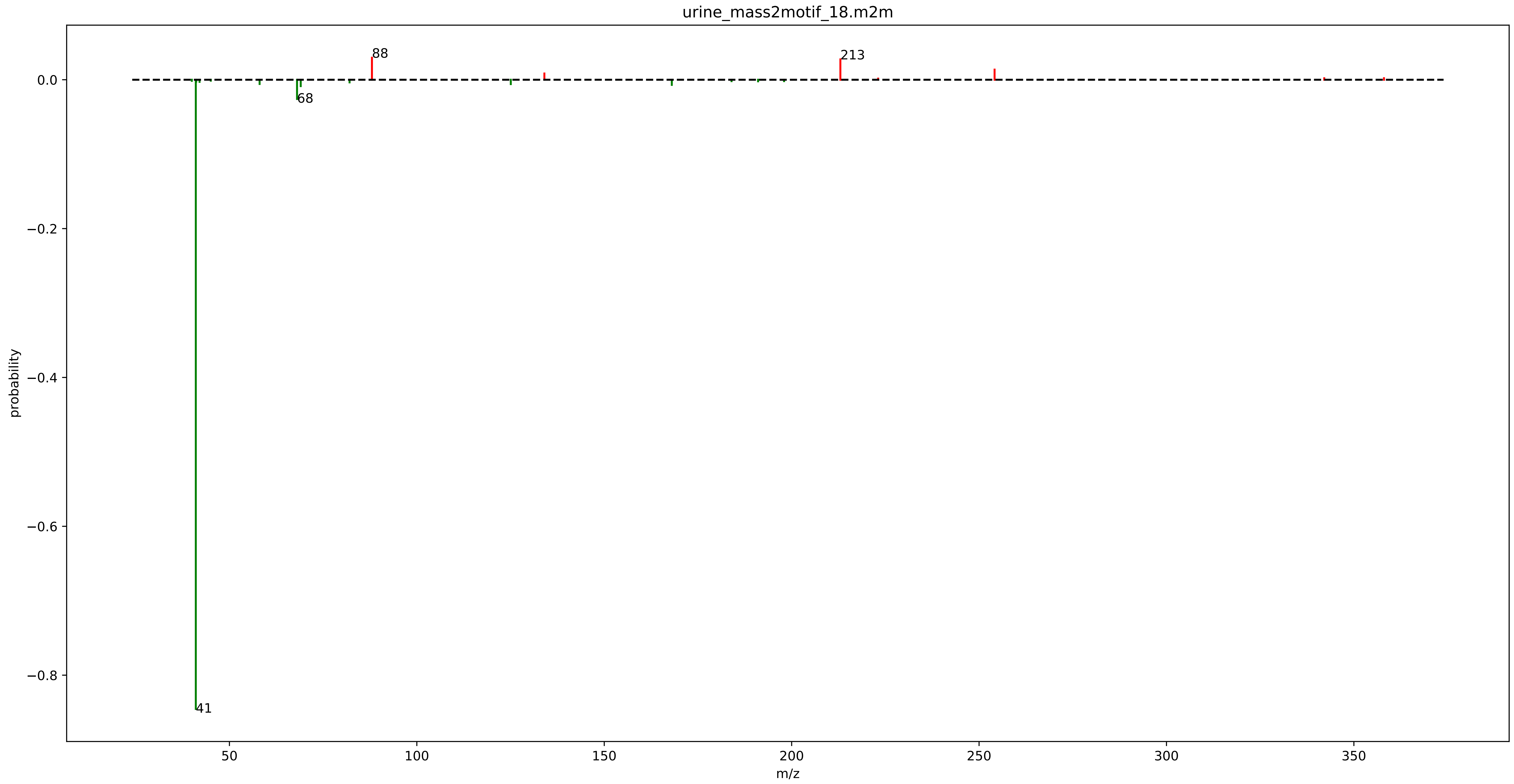

urine_mass2motif_18.m2m
88
213
68
41
0.0
−0.2
−0.4
−0.6
−0.8
probability
50
100
150
200
250
300
350
m/z

urine_mass2motif_18.m2m

loss_41.0275	: 0.85
fragment_88.0225	: 0.03
fragment_212.9975	: 0.03
loss_68.0375	: 0.03
fragment_254.1375	: 0.01
loss_69.0225	: 0.01
fragment_134.0275	: 0.01
loss_168.0225	: 0.01
loss_125.0675	: 0.01
loss_58.0525	: 0.01

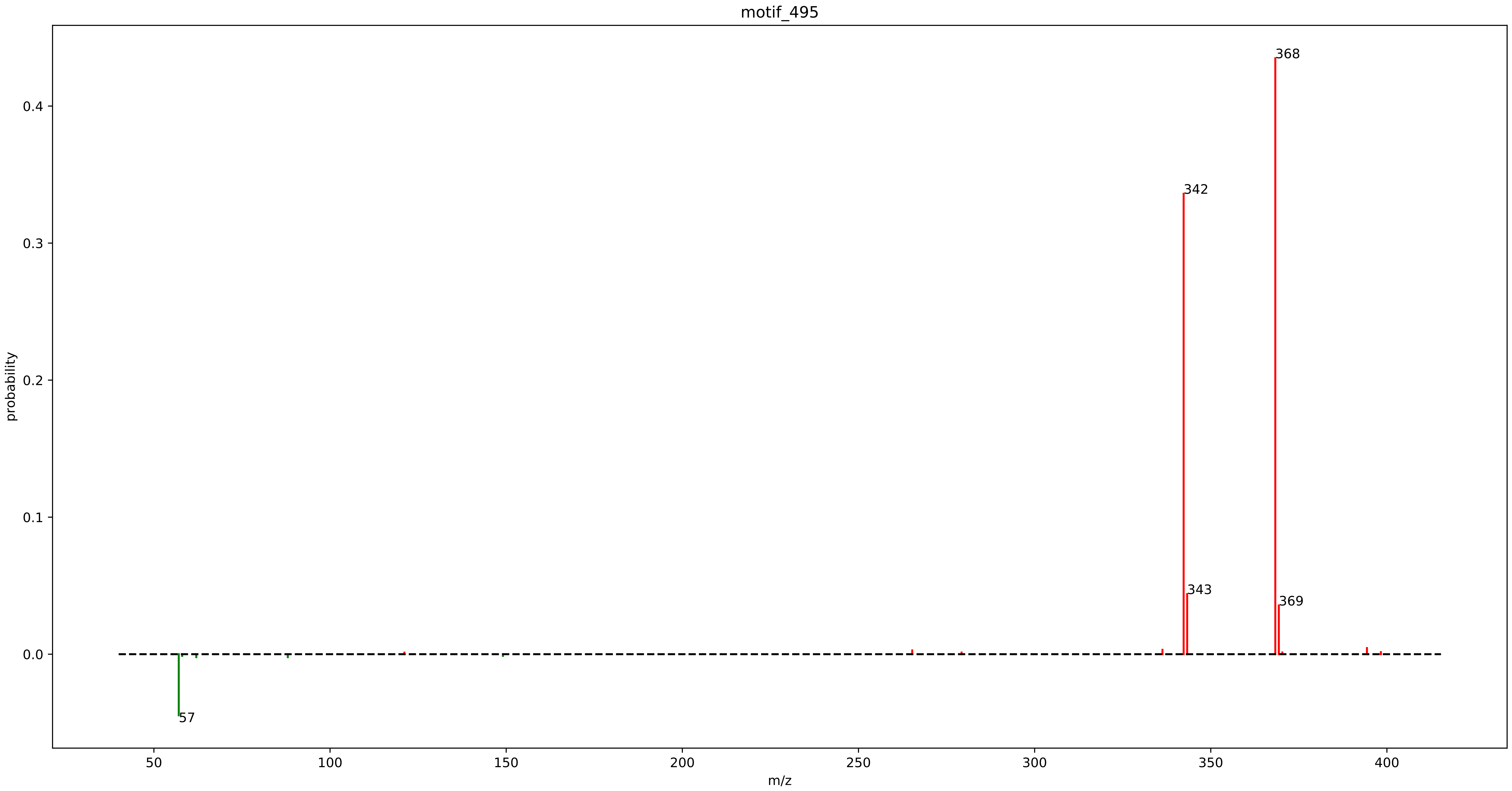
motif_495
probability
m/z
0.4
0.3
0.2
0.1
0.0
50
100
150
200
250
300
350
400
368
342
343
369
57

motif_495

fragment_368.3175	: 0.43
fragment_342.2975	: 0.34
loss_57.0575	: 0.04
fragment_343.3025	: 0.04
fragment_369.3175	: 0.04

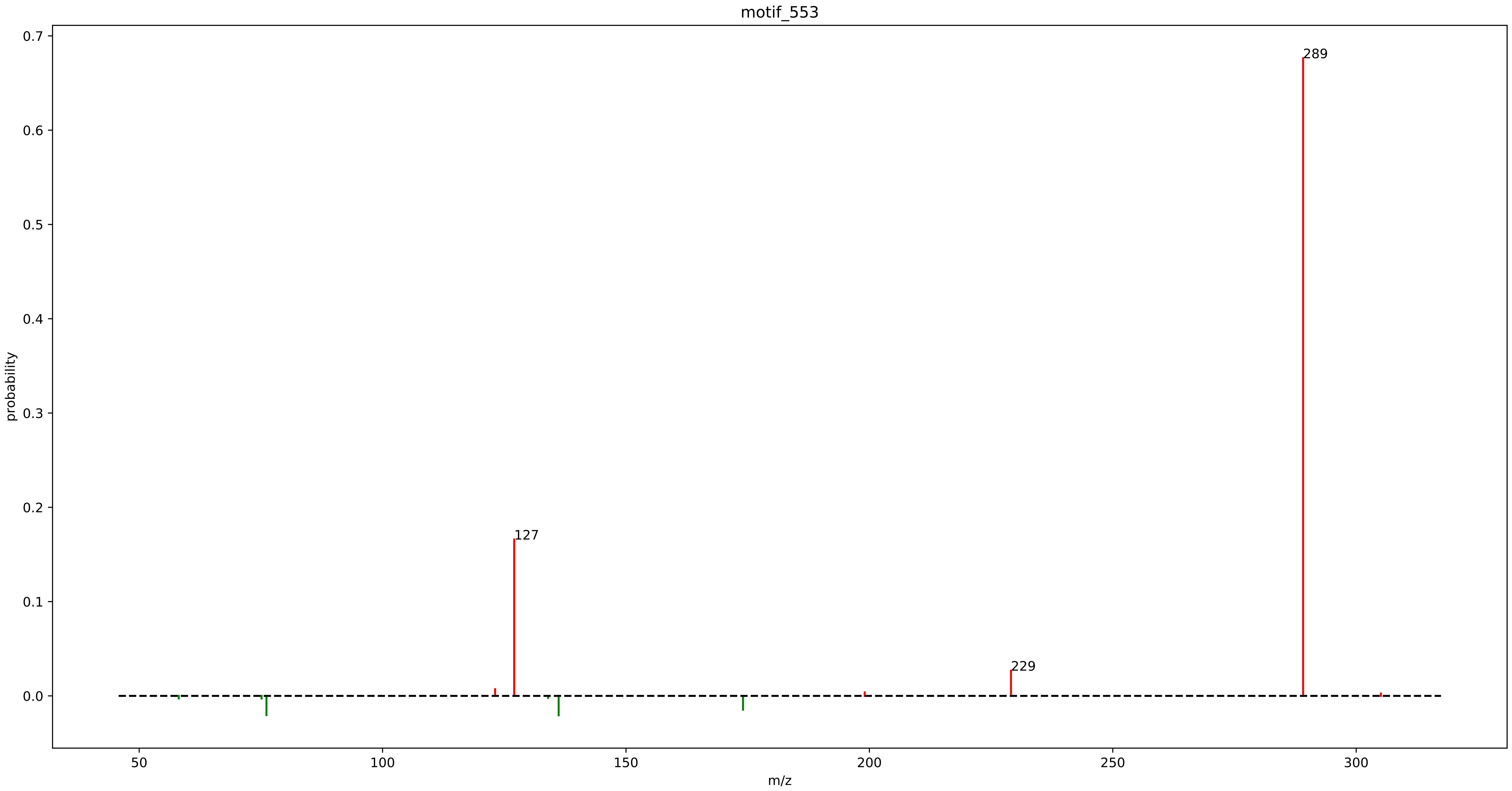
motif_553
289
127
229
probability
m/z
0.7
0.6
0.5
0.4
0.3
0.2
0.1
0.0
50
100
150
200
250
300

motif_553

fragment_289.0925	: 0.68
fragment_127.0375	: 0.17
fragment_229.0825	: 0.03
loss_136.1675	: 0.02
loss_76.1475	: 0.02
loss_174.0425	: 0.01
fragment_123.1175	: 0.01

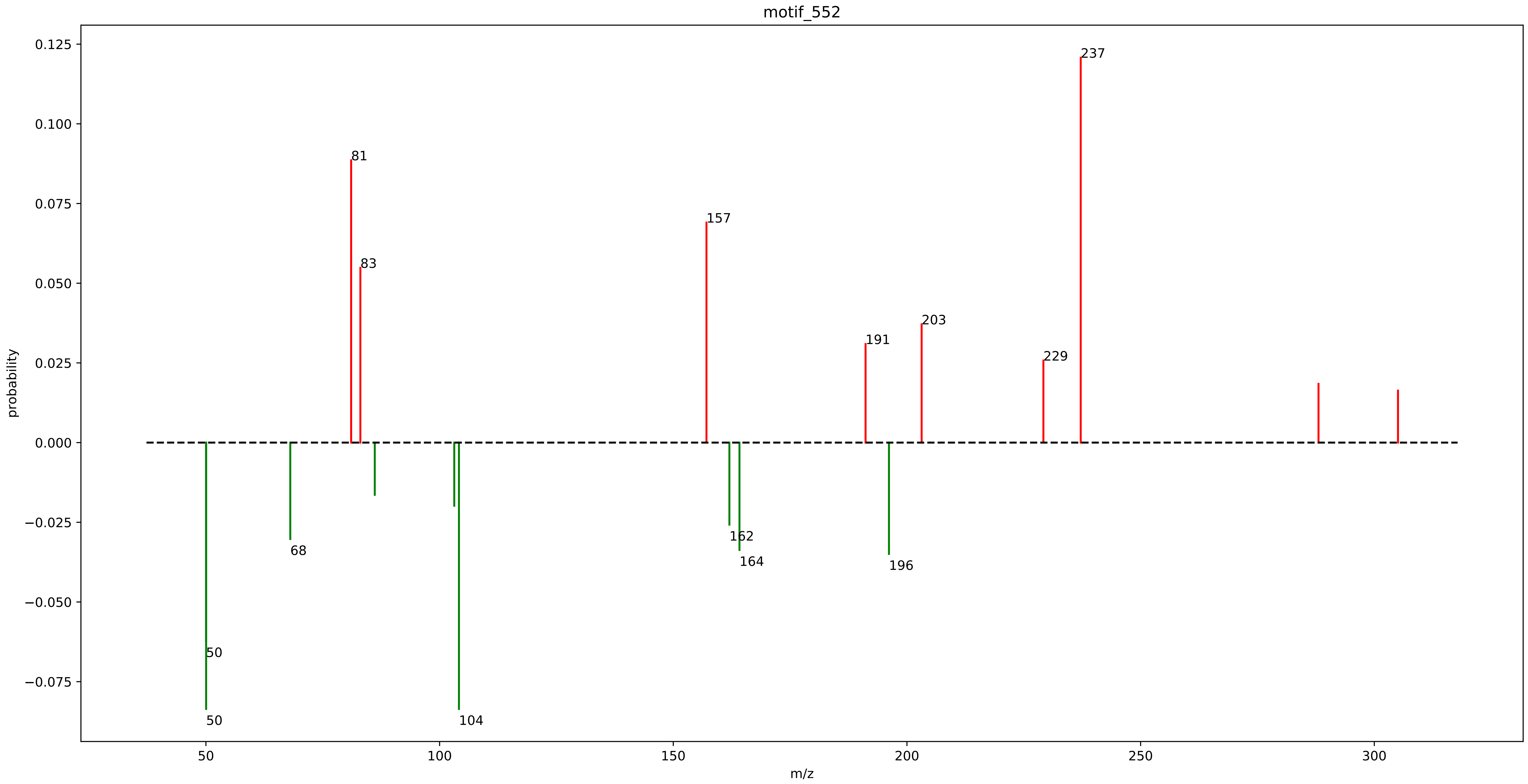
motif_552
probability
m/z
0.125
0.100
0.075
0.050
0.025
0.000
−0.025
−0.050
−0.075
50
100
150
200
250
300
81
83
157
191
203
229
237
68
50
50
104
162
164
196

motif_552

fragment_237.1825	: 0.12
fragment_81.0725	: 0.09
loss_104.1375	: 0.08
loss_50.0425	: 0.08
fragment_157.1025	: 0.07
loss_50.0325	: 0.06
fragment_83.0475	: 0.05
fragment_203.1425	: 0.04
loss_196.1525	: 0.03
loss_164.1625	: 0.03
fragment_191.1425	: 0.03
loss_68.0525	: 0.03
loss_162.0075	: 0.03
fragment_229.1975	: 0.03
loss_103.1375	: 0.02
fragment_288.0625	: 0.02
loss_86.1275	: 0.02
fragment_305.0725	: 0.02

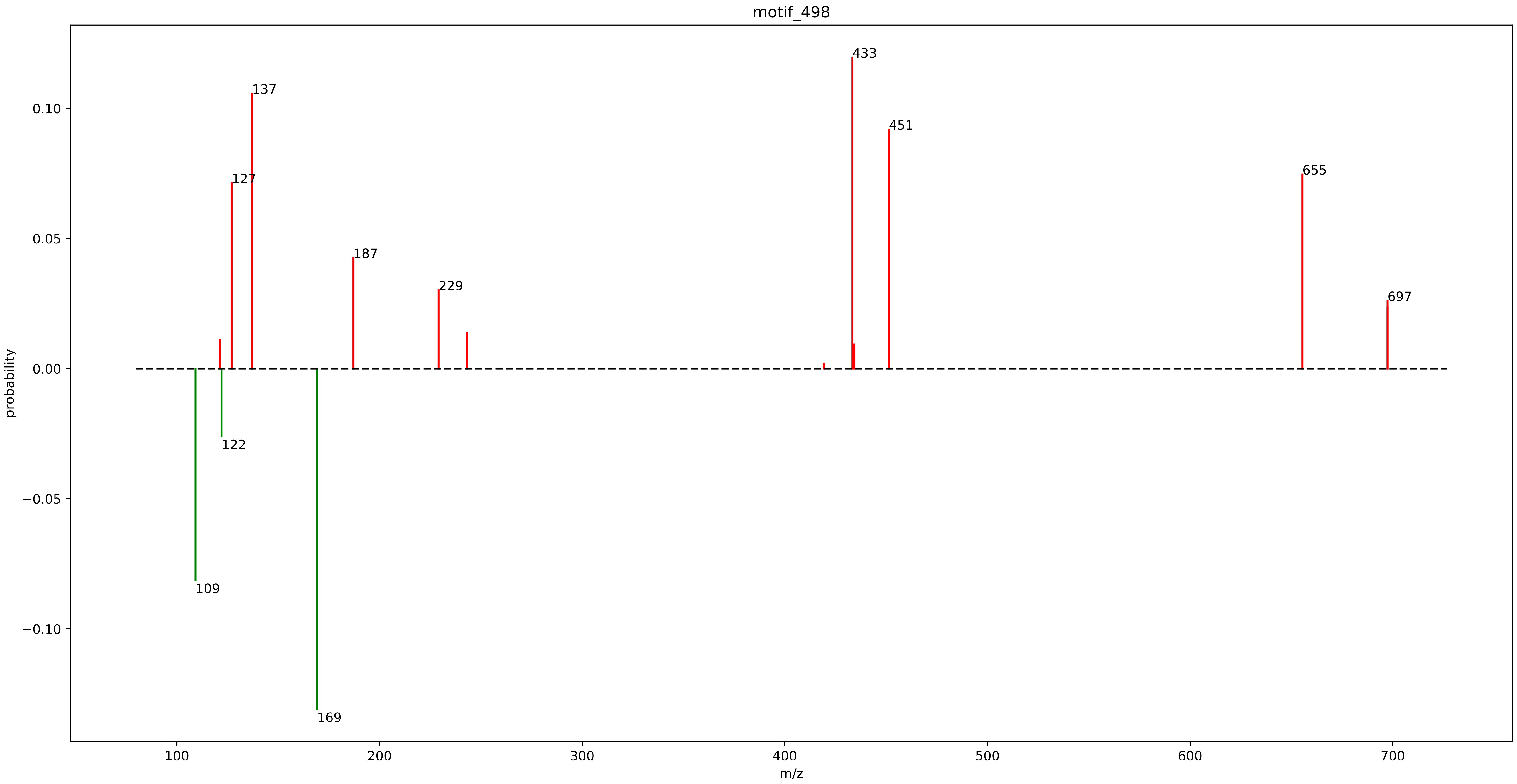

motif_498
probability
m/z
0.10
0.05
0.00
−0.05
−0.10
100
200
300
400
500
600
700
137
127
187
229
433
451
655
697
122
109
169

motif_498

loss_169.1725	: 0.13
fragment_433.3075	: 0.12
fragment_137.0975	: 0.11
fragment_451.3225	: 0.09
loss_109.1525	: 0.08
fragment_655.3825	: 0.07
fragment_127.0375	: 0.07
fragment_187.0725	: 0.04
fragment_229.1325	: 0.03
fragment_697.3875	: 0.03
loss_122.0475	: 0.03
fragment_697.3925	: 0.02
fragment_243.1375	: 0.01
fragment_121.1025	: 0.01
fragment_434.3125	: 0.01

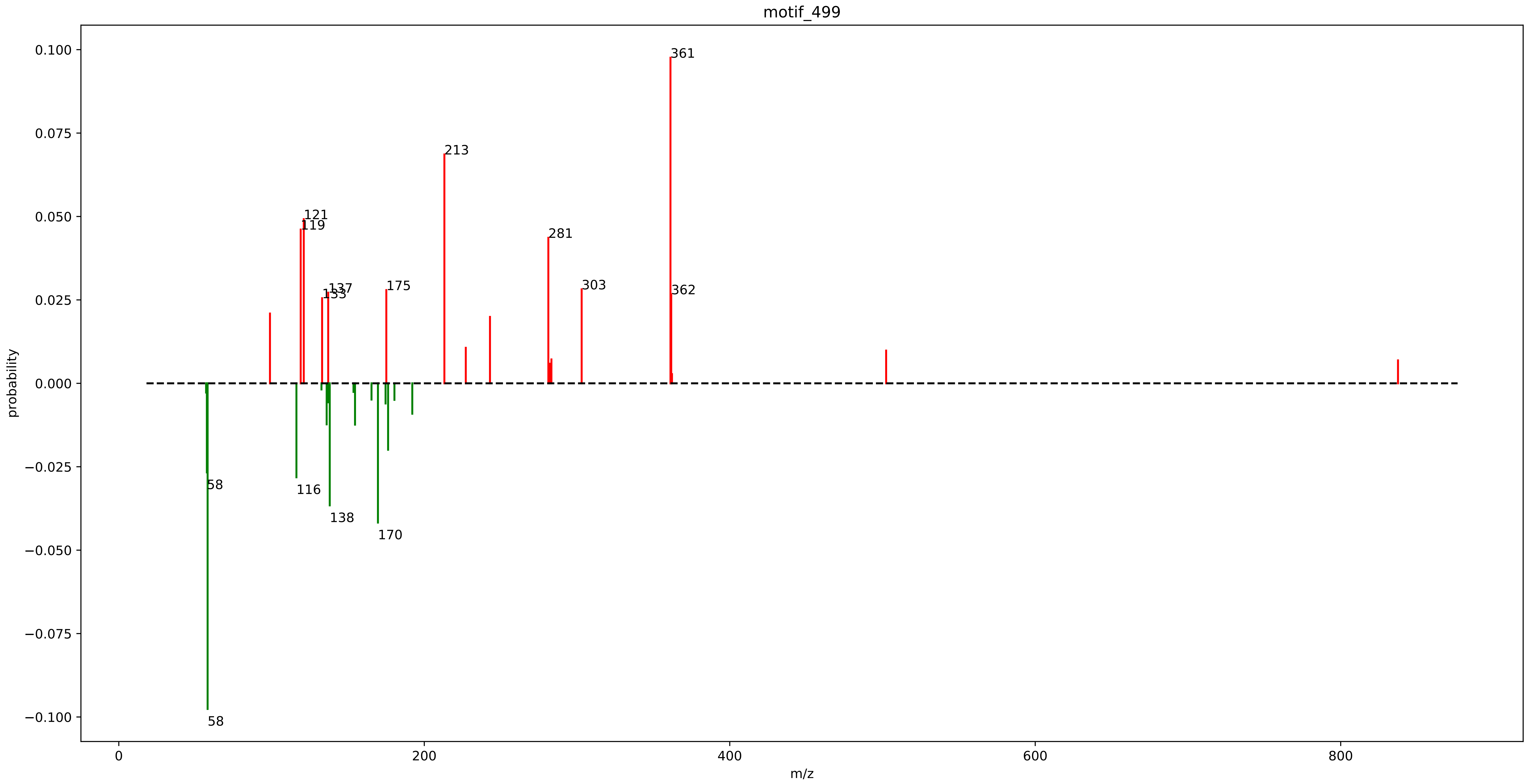

motif_499
361
213
121
119
281
137
133
175
303
362
58
116
138
170
58
probability
m/z
0.100
0.075
0.050
0.025
0.000
−0.025
−0.050
−0.075
−0.100
0
200
400
600
800

motif_499

loss_58.1425	: 0.10
fragment_361.1825	: 0.10
fragment_213.1625	: 0.07
fragment_121.0975	: 0.05
fragment_119.0825	: 0.05
fragment_281.2275	: 0.04
loss_169.6625	: 0.04
loss_138.1025	: 0.04
loss_116.2925	: 0.03
fragment_303.0375	: 0.03
fragment_175.1475	: 0.03
fragment_137.0975	: 0.03
loss_57.6425	: 0.03
fragment_361.6875	: 0.03
fragment_133.1025	: 0.03
fragment_98.9725	: 0.02
loss_176.3125	: 0.02
fragment_243.0175	: 0.02
loss_154.6575	: 0.01
loss_136.0875	: 0.01
fragment_227.1775	: 0.01
fragment_502.3875	: 0.01
loss_192.1475	: 0.01
fragment_283.2425	: 0.01
fragment_837.5125	: 0.01
loss_174.6525	: 0.01
fragment_282.2325	: 0.01
loss_137.0975	: 0.01

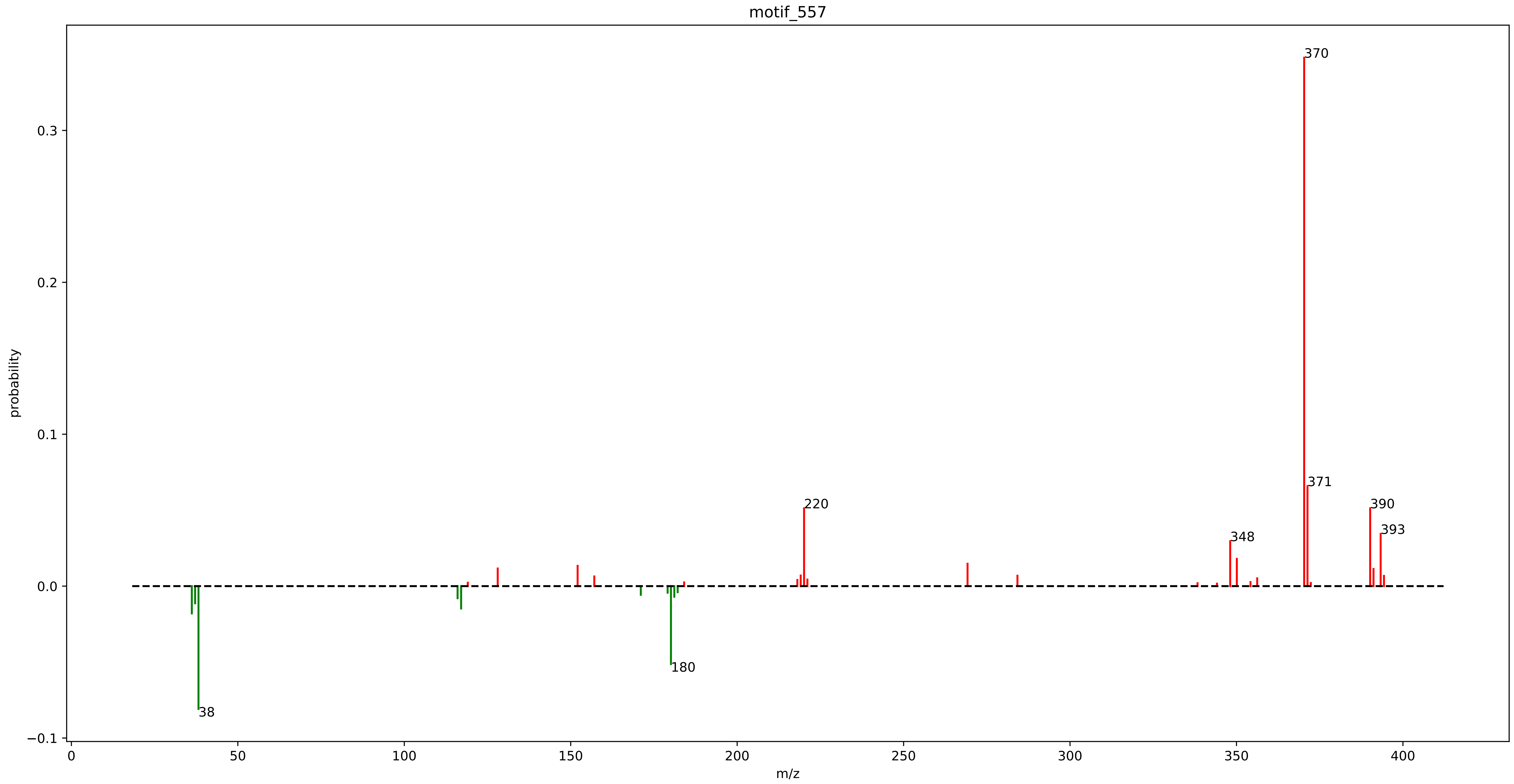

motif_557
probability
m/z
0.3
0.2
0.1
0.0
−0.1
0
50
100
150
200
250
300
350
400
370
371
390
393
348
220
180
38

motif_557

fragment_370.3325	: 0.35
loss_38.1775	: 0.08
fragment_371.3325	: 0.07
loss_180.1125	: 0.05
fragment_390.1575	: 0.05
fragment_220.0975	: 0.05
fragment_393.3075	: 0.03
fragment_348.1125	: 0.03
fragment_350.1075	: 0.02
loss_36.1775	: 0.02
loss_117.0775	: 0.01
fragment_269.2075	: 0.01
fragment_152.0725	: 0.01
fragment_128.0675	: 0.01
fragment_391.1625	: 0.01
loss_37.1725	: 0.01
loss_116.0125	: 0.01
fragment_219.0875	: 0.01
loss_181.1225	: 0.01
fragment_284.1975	: 0.01
fragment_394.3075	: 0.01
fragment_157.0825	: 0.01
loss_171.0575	: 0.01
fragment_356.2225	: 0.01

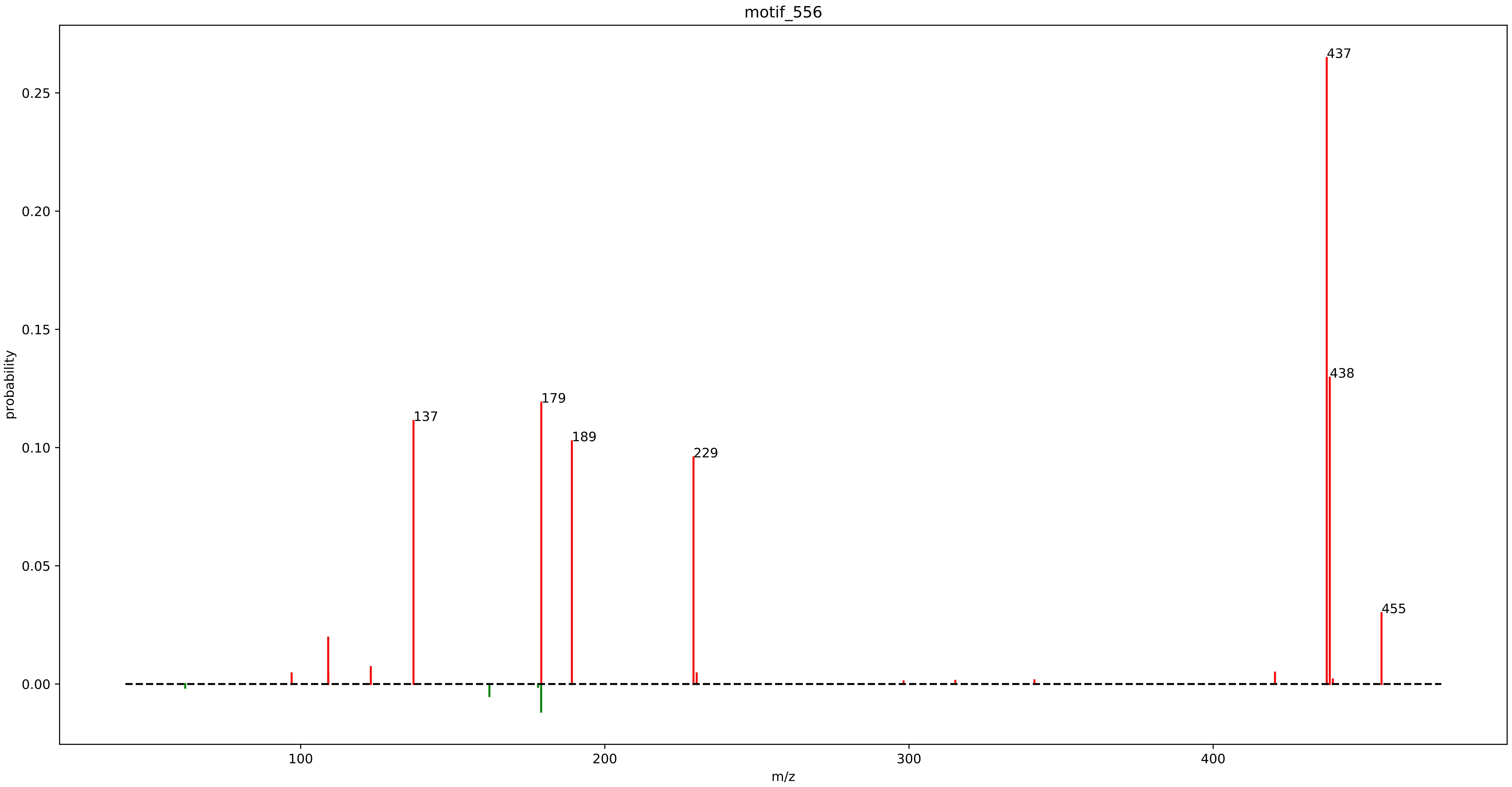
motif_556
probability
m/z
0.25
0.20
0.15
0.10
0.05
0.00
100
200
300
400
437
438
179
137
189
229
455

motif_556

fragment_437.3425	: 0.26
fragment_438.3425	: 0.13
fragment_179.1075	: 0.12
fragment_137.0975	: 0.11
fragment_189.1625	: 0.10
fragment_229.1475	: 0.10
fragment_455.3525	: 0.03
fragment_109.0425	: 0.02
loss_179.0575	: 0.01
fragment_123.0575	: 0.01
loss_162.0475	: 0.01

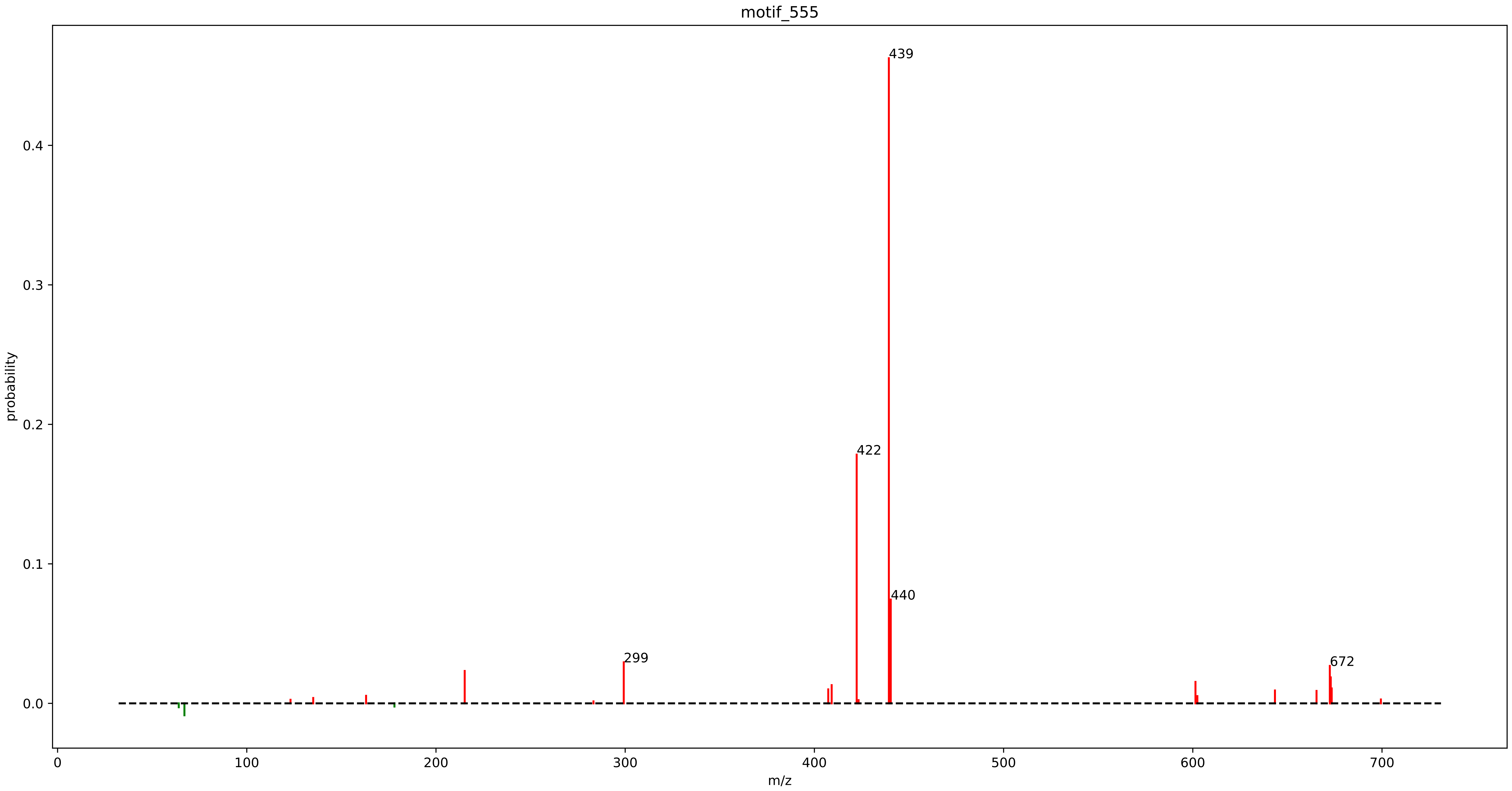

motif_555
439
422
440
299
672
probability
0.4
0.3
0.2
0.1
0.0
0
100
200
300
400
500
600
700
m/z

motif_555

fragment_439.3575	: 0.46
fragment_422.3475	: 0.18
fragment_440.3575	: 0.07
fragment_299.2375	: 0.03
fragment_672.4175	: 0.03
fragment_215.1775	: 0.02
fragment_672.9175	: 0.02
fragment_601.4075	: 0.02
fragment_409.1325	: 0.01
fragment_673.4175	: 0.01
fragment_407.3275	: 0.01
fragment_643.4175	: 0.01
fragment_665.3925	: 0.01
loss_67.0425	: 0.01
fragment_163.0575	: 0.01
fragment_602.4125	: 0.01

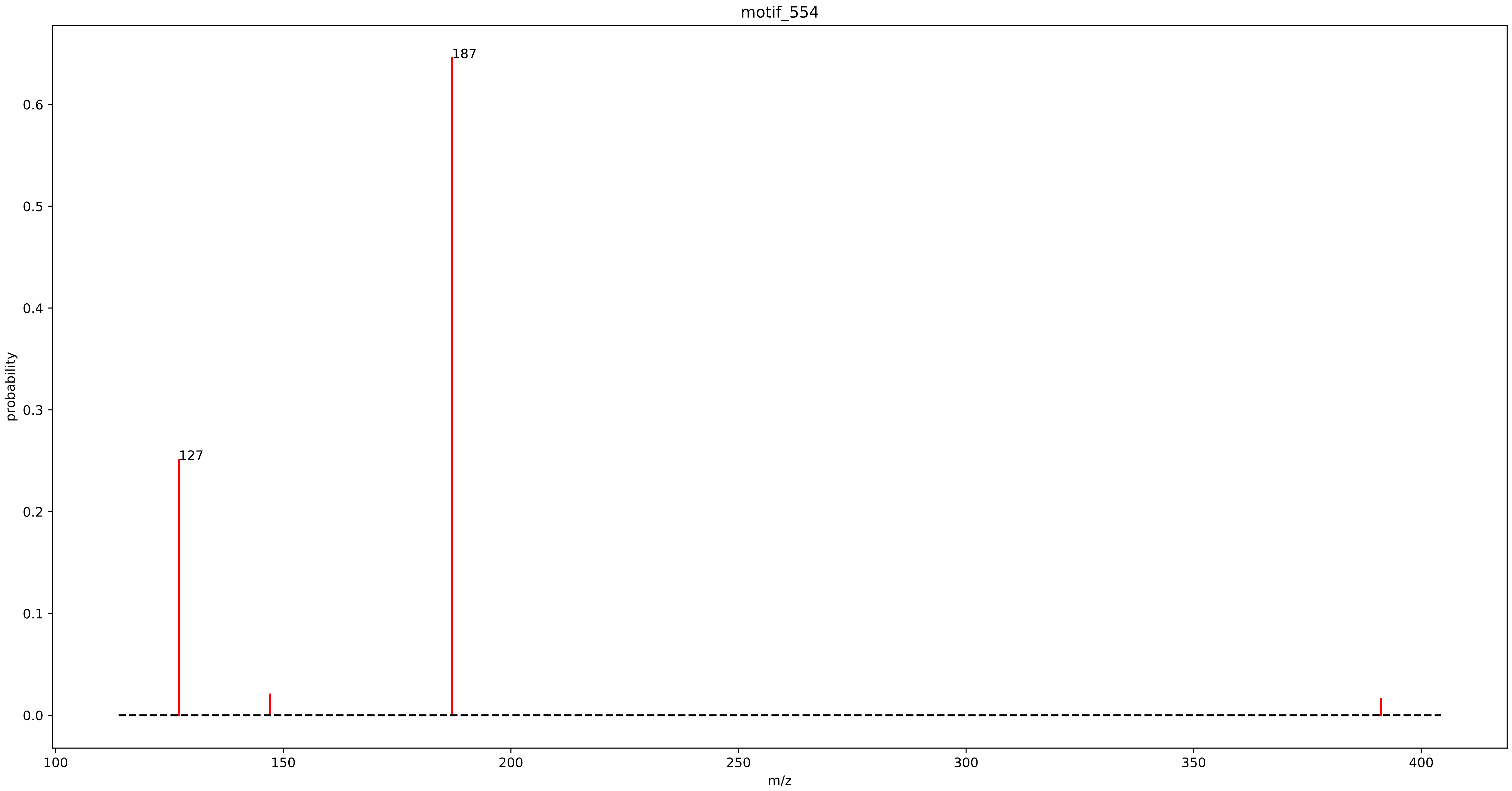

motif_554
187
127
probability
0.0
0.1
0.2
0.3
0.4
0.5
0.6
100
150
200
250
300
350
400
m/z

motif_554

fragment_187.0575	: 0.65
fragment_127.0375	: 0.25
fragment_147.1175	: 0.02
fragment_391.1225	: 0.02

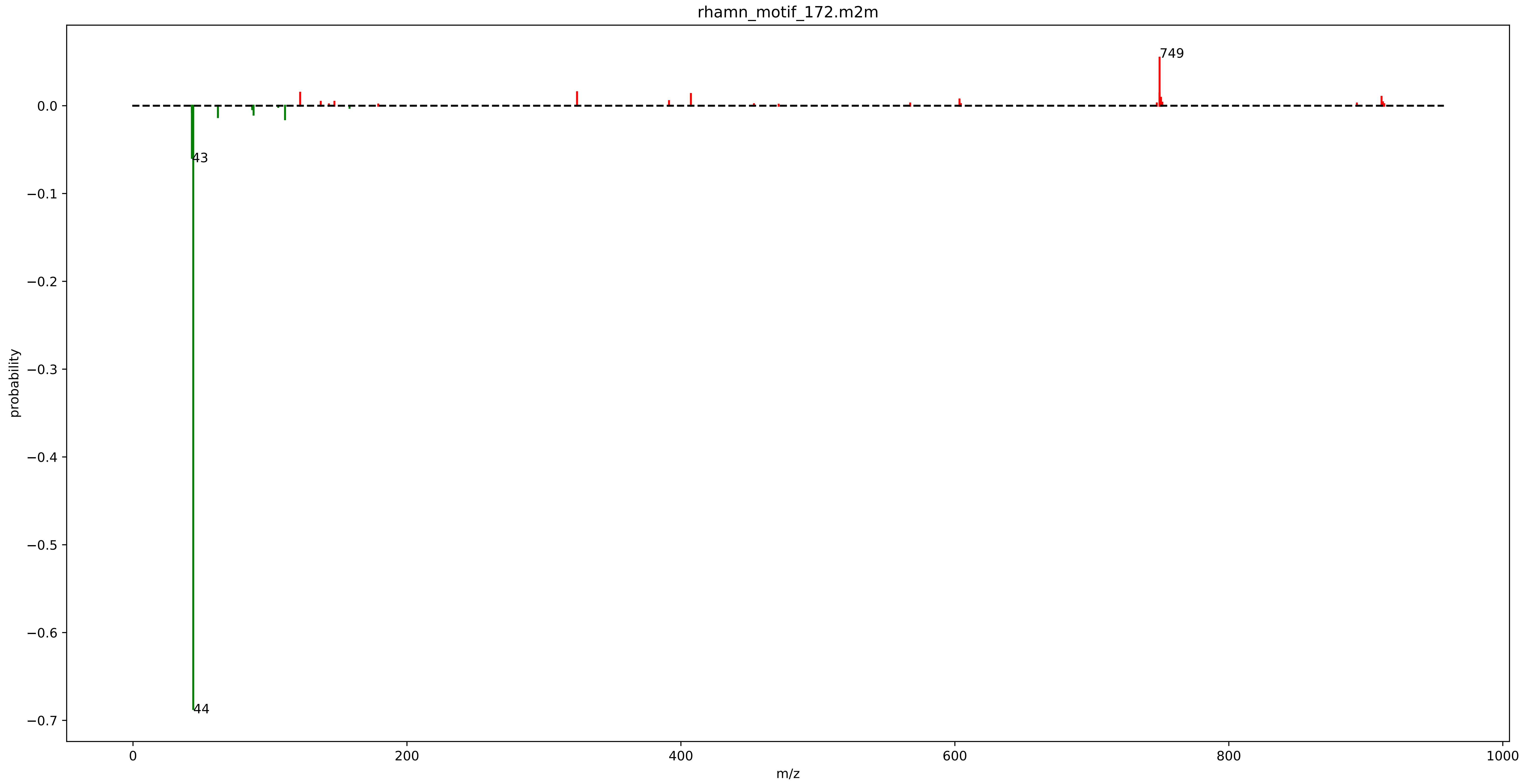

rhamn_motif_172.m2m
749
43
44
0.0
−0.1
−0.2
−0.3
−0.4
−0.5
−0.6
−0.7
probability
0
200
400
600
800
1000
m/z

rhamn_motif_172.m2m

loss_43.9875	: 0.69
loss_42.9825	: 0.06
fragment_749.4425	: 0.05
loss_110.9825	: 0.02
fragment_324.1775	: 0.02
fragment_122.0375	: 0.01
fragment_749.4225	: 0.01
fragment_407.2925	: 0.01
loss_61.9975	: 0.01
fragment_911.4875	: 0.01
loss_88.0275	: 0.01
fragment_750.4625	: 0.01
fragment_750.4375	: 0.01
fragment_603.3825	: 0.01
loss_42.9775	: 0.01
fragment_391.3025	: 0.01

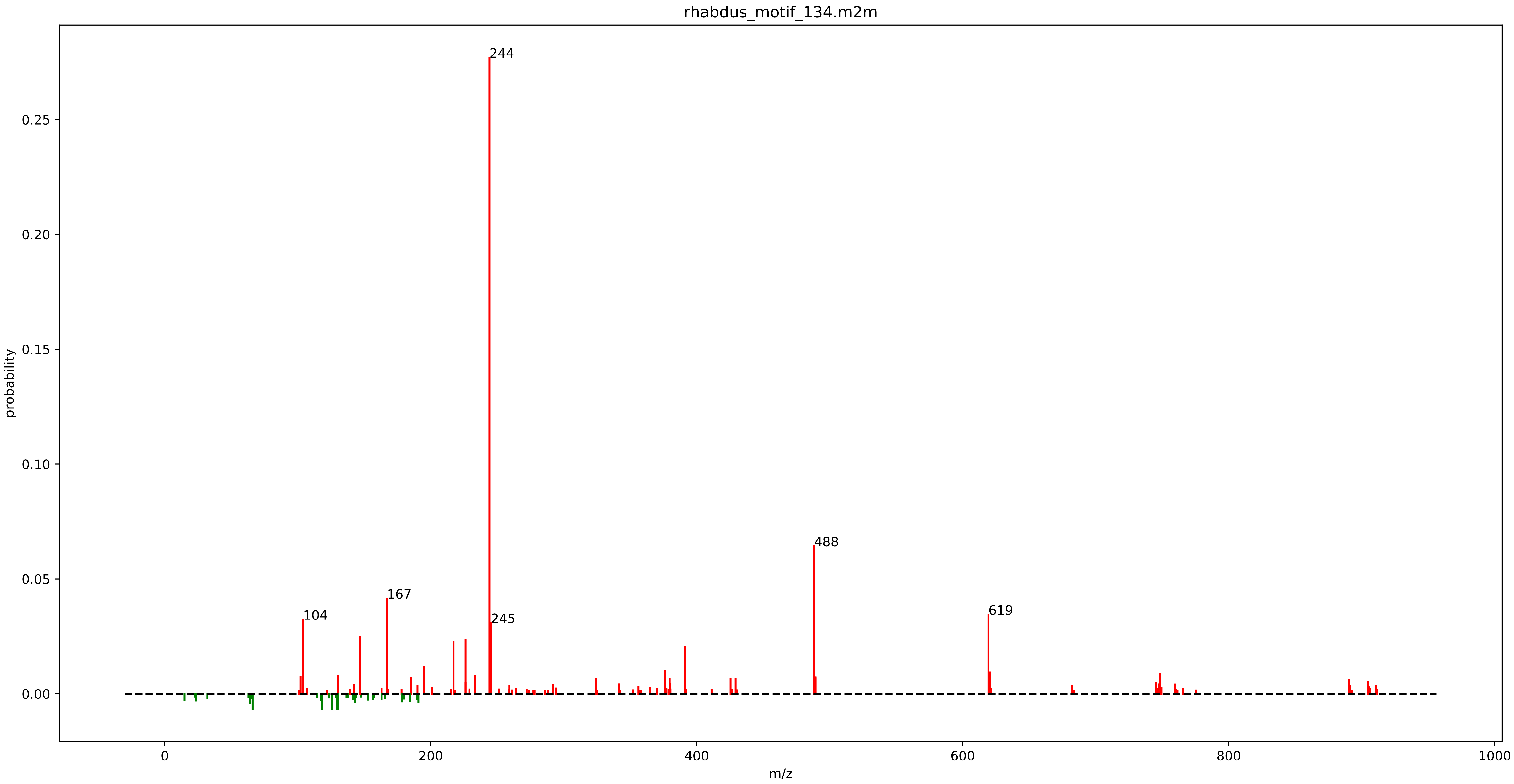
rhabdus_motif_134.m2m
probability
m/z
244
488
167
104
245
619
0.25
0.20
0.15
0.10
0.05
0.00
0
200
400
600
800
1000

rhabdus_motif_134.m2m

fragment_244.1625	: 0.28
fragment_488.2875	: 0.06
fragment_167.0825	: 0.04
fragment_619.3275	: 0.03
fragment_104.0525	: 0.03
fragment_245.1475	: 0.03
fragment_147.1125	: 0.02
fragment_226.1525	: 0.02
fragment_217.1325	: 0.02
fragment_391.2325	: 0.02
fragment_244.6475	: 0.02
fragment_245.1525	: 0.01
fragment_147.1075	: 0.01
fragment_195.0775	: 0.01
fragment_376.1675	: 0.01
fragment_620.3325	: 0.01
fragment_748.3675	: 0.01
fragment_233.0975	: 0.01
fragment_130.0825	: 0.01
fragment_102.0525	: 0.01
fragment_489.2875	: 0.01
fragment_185.0975	: 0.01
fragment_489.2925	: 0.01
fragment_324.1575	: 0.01
loss_130.5225	: 0.01
fragment_379.6275	: 0.01
loss_129.5175	: 0.01
fragment_425.3575	: 0.01
loss_118.3375	: 0.01
fragment_429.1975	: 0.01
loss_125.5625	: 0.01
loss_66.0025	: 0.01
fragment_890.4525	: 0.01
fragment_904.4675	: 0.01
fragment_130.0775	: 0.01

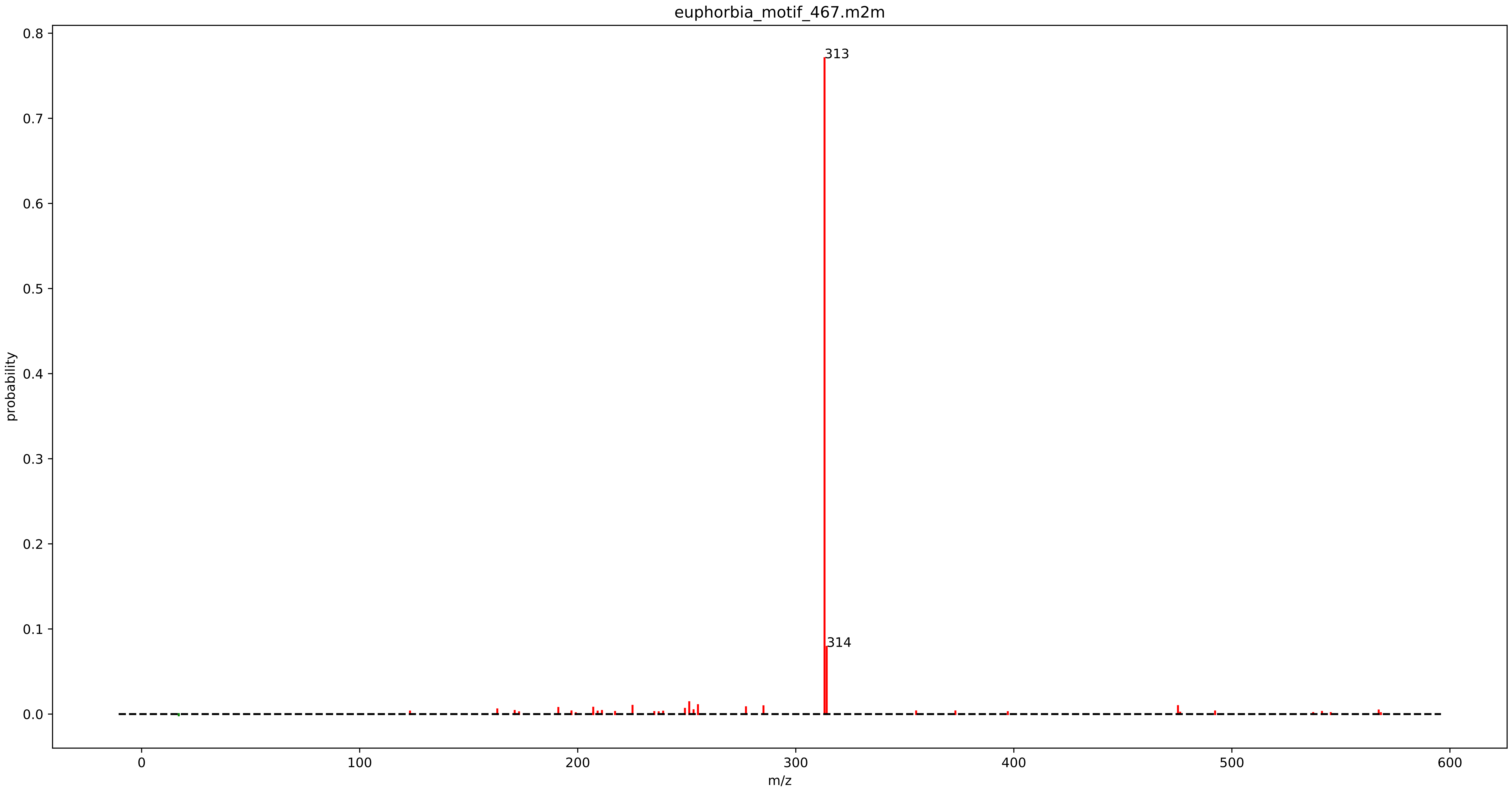

euphorbia_motif_467.m2m
313
314
probability
m/z
0.8
0.7
0.6
0.5
0.4
0.3
0.2
0.1
0.0
0
100
200
300
400
500
600

euphorbia_motif_467.m2m

fragment_313.1825	: 0.77
fragment_314.1825	: 0.08
fragment_251.1425	: 0.01
fragment_255.1375	: 0.01
fragment_225.1275	: 0.01
fragment_475.2725	: 0.01
fragment_285.1825	: 0.01
fragment_277.1575	: 0.01
fragment_207.1175	: 0.01
fragment_191.1075	: 0.01
fragment_249.1625	: 0.01
fragment_163.1125	: 0.01

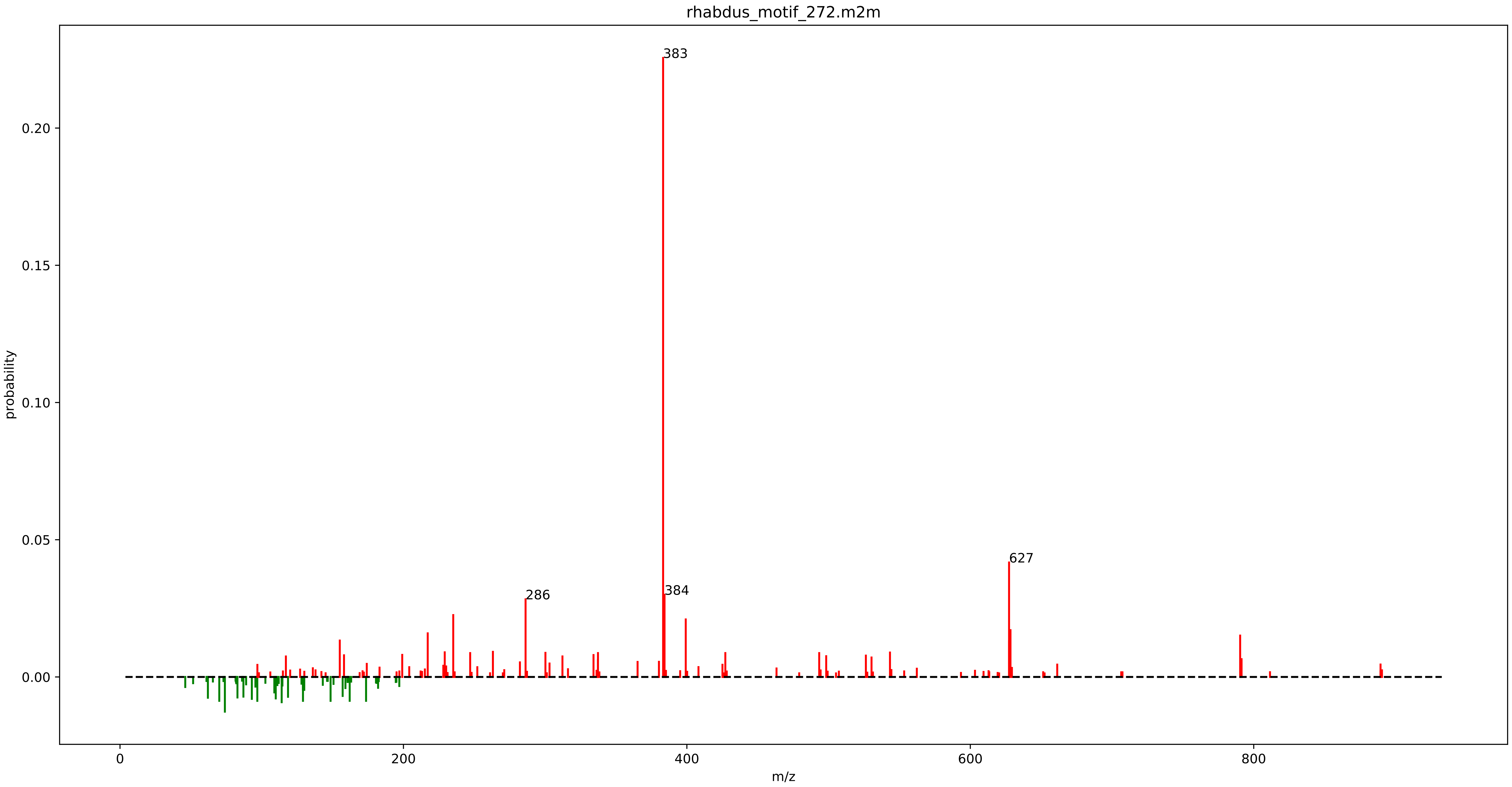

rhabdus_motif_272.m2m
probability
m/z
383
384
286
627
0.20
0.15
0.10
0.05
0.00
0
200
400
600
800

rhabdus_motif_272.m2m

fragment_383.2275 : 0.23
fragment_627.3475 : 0.04
fragment_384.2325 : 0.03
fragment_286.1775 : 0.03
fragment_235.1425 : 0.02
fragment_399.2025 : 0.02
fragment_628.3525 : 0.02
fragment_217.1325 : 0.02
fragment_790.4125 : 0.02
fragment_155.0825 : 0.01
fragment_627.3525 : 0.01
loss_74.0025 : 0.01
loss_114.0775 : 0.01
fragment_263.1375 : 0.01
fragment_229.1375 : 0.01
fragment_543.3175 : 0.01
fragment_300.1925 : 0.01
fragment_427.1375 : 0.01
loss_148.5825 : 0.01
fragment_337.2725 : 0.01
fragment_247.0875 : 0.01
loss_96.8875 : 0.01
loss_173.6275 : 0.01
loss_129.1125 : 0.01
fragment_493.3775 : 0.01
loss_70.0475 : 0.01
loss_162.0725 : 0.01
fragment_199.1425 : 0.01
fragment_334.1175 : 0.01
loss_93.0225 : 0.01
fragment_158.0625 : 0.01
loss_109.8975 : 0.01
fragment_526.3125 : 0.01
loss_62.0175 : 0.01
fragment_498.3175 : 0.01
fragment_312.1975 : 0.01
loss_82.8725 : 0.01
fragment_117.0525 : 0.01
loss_118.5575 : 0.01
loss_87.0775 : 0.01
fragment_530.2975 : 0.01
loss_157.1125 : 0.01
fragment_791.4175 : 0.01
fragment_337.2225 : 0.01
loss_108.8875 : 0.01
fragment_380.2925 : 0.01
fragment_365.2175 : 0.01
fragment_282.1925 : 0.01

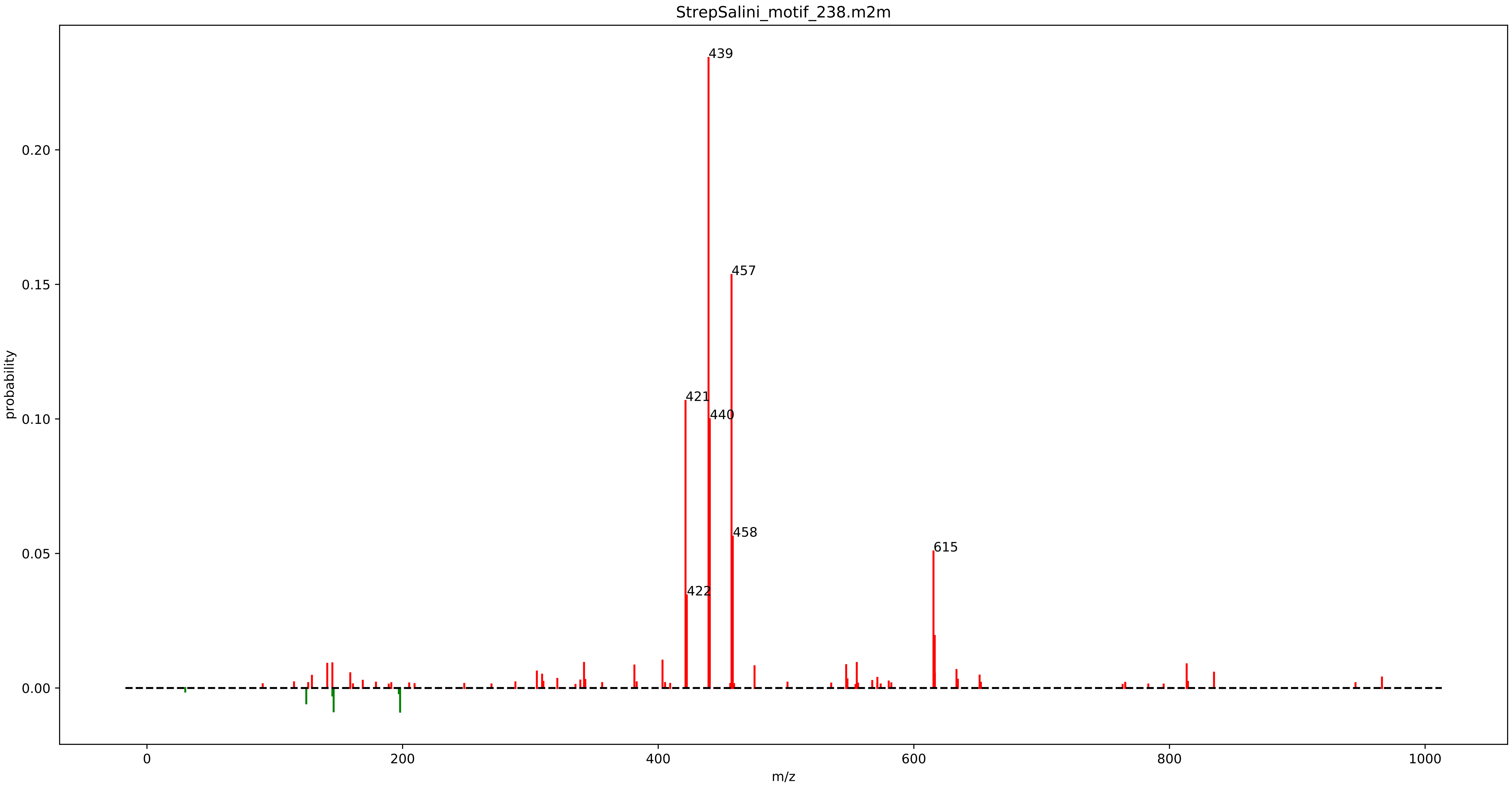

StrepSalini_motif_238.m2m
probability
m/z
0.20
0.15
0.10
0.05
0.00
0
200
400
600
800
1000
439
457
421
440
458
615
422

StrepSalini_motif_238.m2m

fragment_439.3525	: 0.23
fragment_457.3500	: 0.15
fragment_421.3500	: 0.11
fragment_440.3500	: 0.10
fragment_458.3500	: 0.06
fragment_615.3500	: 0.05
fragment_422.3525	: 0.03
fragment_616.3500	: 0.02
fragment_403.3500	: 0.01
fragment_341.9500	: 0.01
fragment_555.3500	: 0.01
fragment_145.0500	: 0.01
fragment_141.0500	: 0.01
fragment_813.4500	: 0.01
loss_198.0500	: 0.01
loss_146.0525	: 0.01
fragment_547.0500	: 0.01
fragment_381.3500	: 0.01
fragment_475.3500	: 0.01
fragment_633.3500	: 0.01
fragment_305.0500	: 0.01
fragment_834.8500	: 0.01
loss_124.6500	: 0.01
fragment_159.0500	: 0.01
fragment_616.4500	: 0.01

gnps_motif_42.m2m

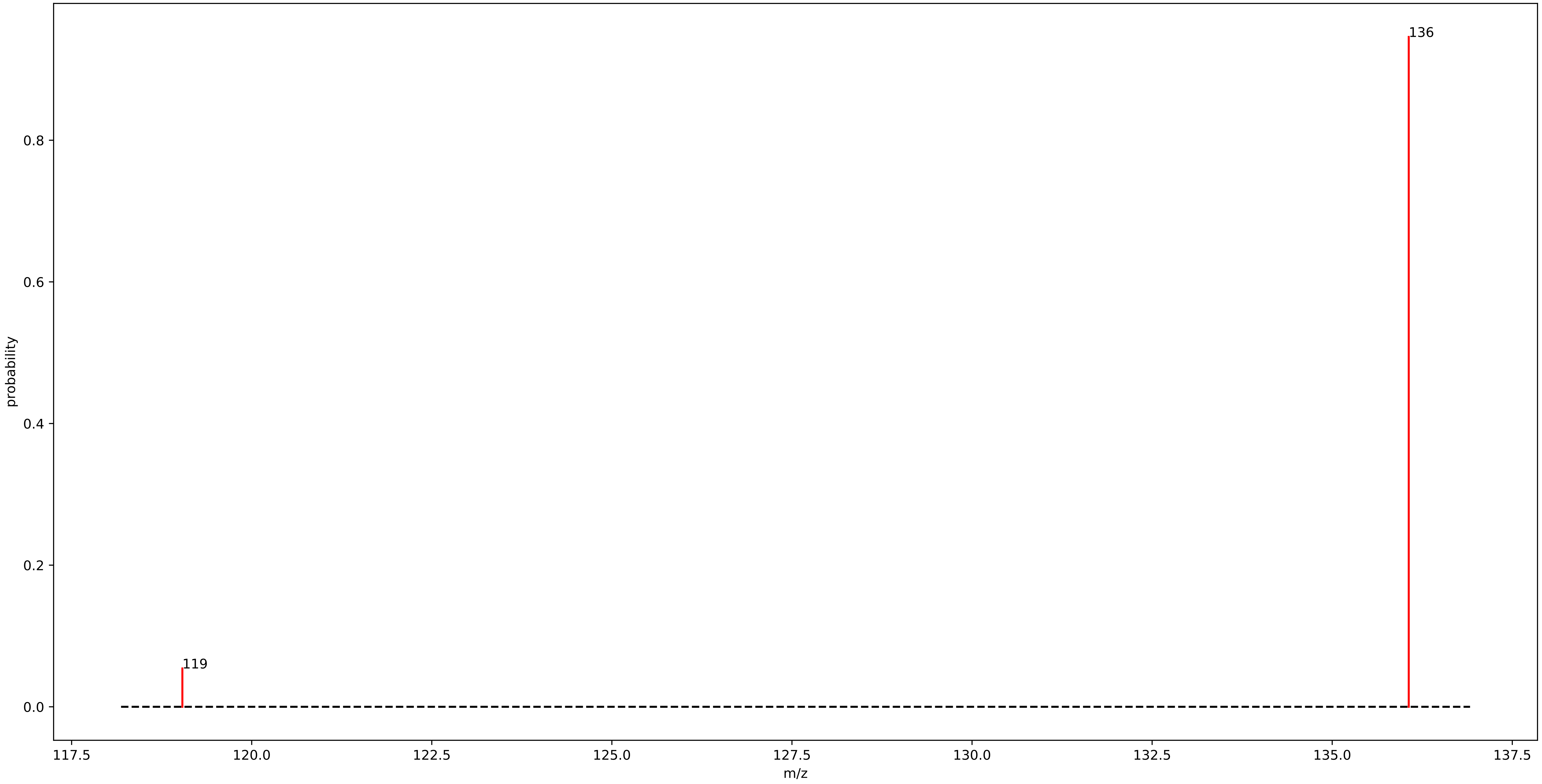

gnps_motif_42.m2m

fragment_136.0625	: 0.95
fragment_119.0375	: 0.05

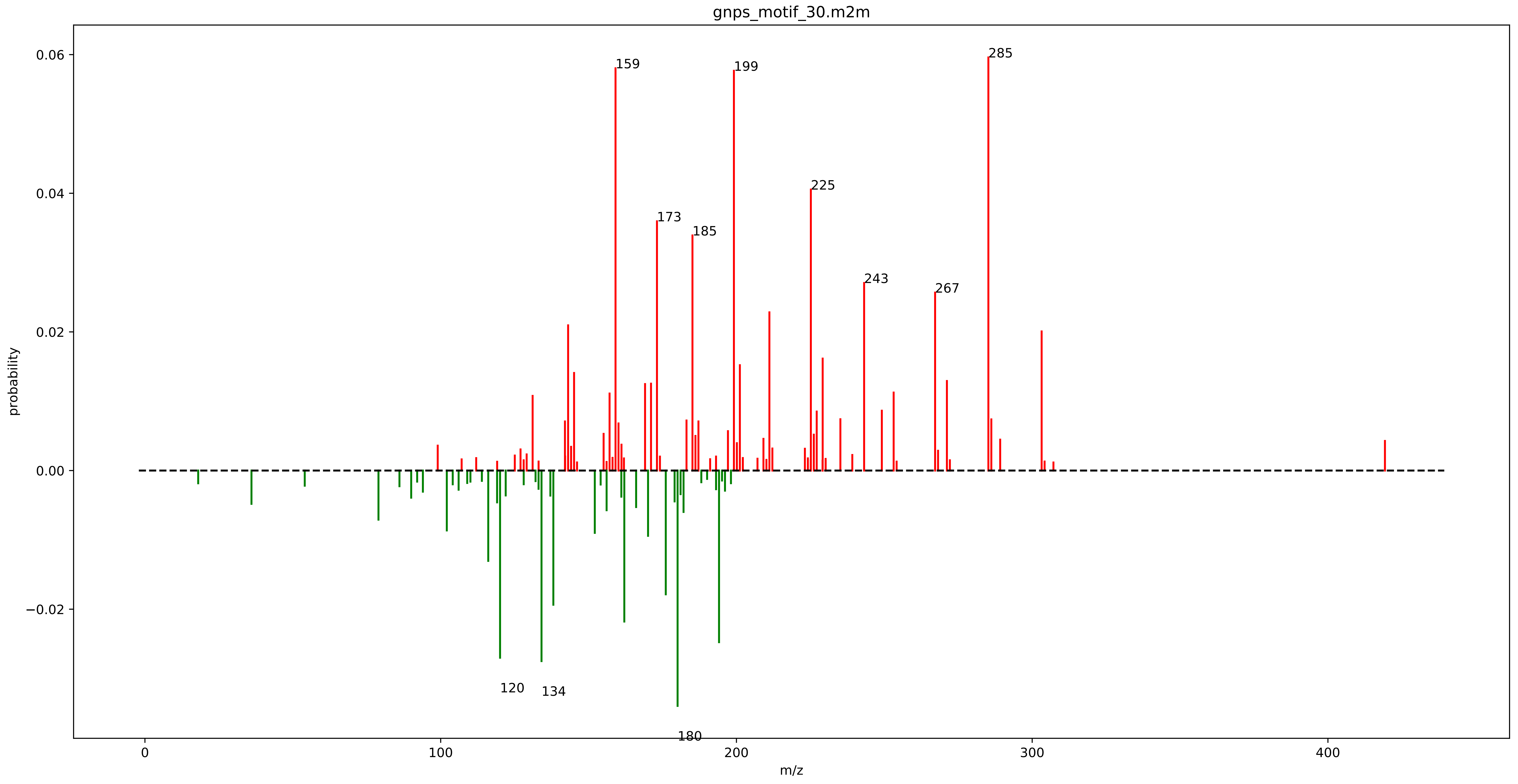

gnps_motif_30.m2m
probability
m/z
0.06
0.04
0.02
0.00
−0.02
0
100
200
300
400
159
199
285
225
173
185
243
267
120
134
180

gnps_motif_30.m2m

fragment_285.1875	: 0.06
fragment_159.1175	: 0.06
fragment_199.1475	: 0.06
fragment_225.1625	: 0.04
fragment_173.1325	: 0.04
loss_180.1025	: 0.03
fragment_185.1325	: 0.03
loss_134.0925	: 0.03
fragment_243.1725	: 0.03
loss_120.0775	: 0.03
fragment_267.1725	: 0.03
loss_194.1175	: 0.02
fragment_211.1475	: 0.02
loss_162.0875	: 0.02
fragment_143.0875	: 0.02
fragment_303.1975	: 0.02
loss_138.0925	: 0.02
loss_176.1075	: 0.02
fragment_229.1575	: 0.02
fragment_201.1625	: 0.02
fragment_145.1025	: 0.01
loss_116.0825	: 0.01
fragment_271.1675	: 0.01
fragment_171.1175	: 0.01
fragment_169.1025	: 0.01
fragment_253.1575	: 0.01
fragment_157.1025	: 0.01
fragment_131.0875	: 0.01
loss_170.1175	: 0.01
loss_152.1075	: 0.01
loss_102.0675	: 0.01
fragment_249.1625	: 0.01
fragment_227.1425	: 0.01
fragment_235.1475	: 0.01
fragment_286.1875	: 0.01
fragment_183.1175	: 0.01
loss_78.9625	: 0.01
fragment_142.0175	: 0.01
fragment_187.1525	: 0.01
fragment_160.1225	: 0.01
loss_182.1175	: 0.01
loss_156.1025	: 0.01
fragment_197.1325	: 0.01
fragment_155.0875	: 0.01
loss_166.0825	: 0.01
fragment_226.1675	: 0.01
fragment_187.1475	: 0.01

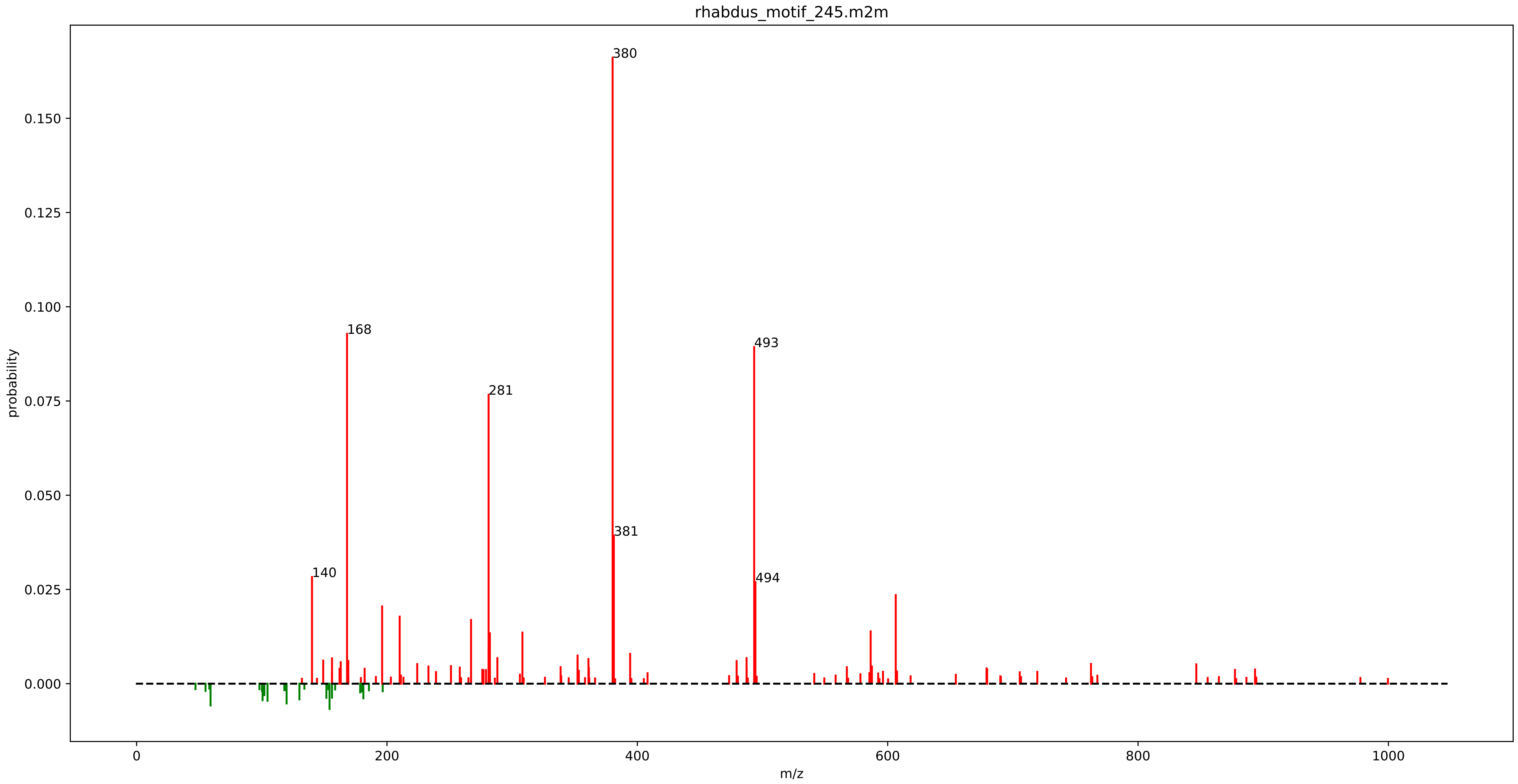

rhabdus_motif_245.m2m
probability
m/z
0.150
0.125
0.100
0.075
0.050
0.025
0.000
0
200
400
600
800
1000
380
168
493
281
381
494
140

rhabdus_motif_245.m2m

fragment_380.2525	: 0.17
fragment_168.1025	: 0.09
fragment_493.3375	: 0.09
fragment_281.1875	: 0.08
fragment_381.2575	: 0.04
fragment_140.1075	: 0.03
fragment_494.3425	: 0.03
fragment_606.4225	: 0.02
fragment_196.1325	: 0.02
fragment_210.1475	: 0.02
fragment_267.1725	: 0.02
fragment_586.3975	: 0.01
fragment_308.2125	: 0.01
fragment_282.1875	: 0.01
fragment_281.1825	: 0.01
fragment_394.2675	: 0.01
fragment_352.2625	: 0.01
fragment_487.2525	: 0.01
fragment_288.1925	: 0.01
fragment_156.0975	: 0.01
loss_154.1275	: 0.01
fragment_360.9475	: 0.01
fragment_352.2575	: 0.01
fragment_149.0725	: 0.01
fragment_169.1025	: 0.01
fragment_479.3225	: 0.01
loss_59.0725	: 0.01
fragment_163.0725	: 0.01
loss_119.8275	: 0.01
fragment_762.4025	: 0.01
fragment_224.1725	: 0.01
fragment_846.5325	: 0.01

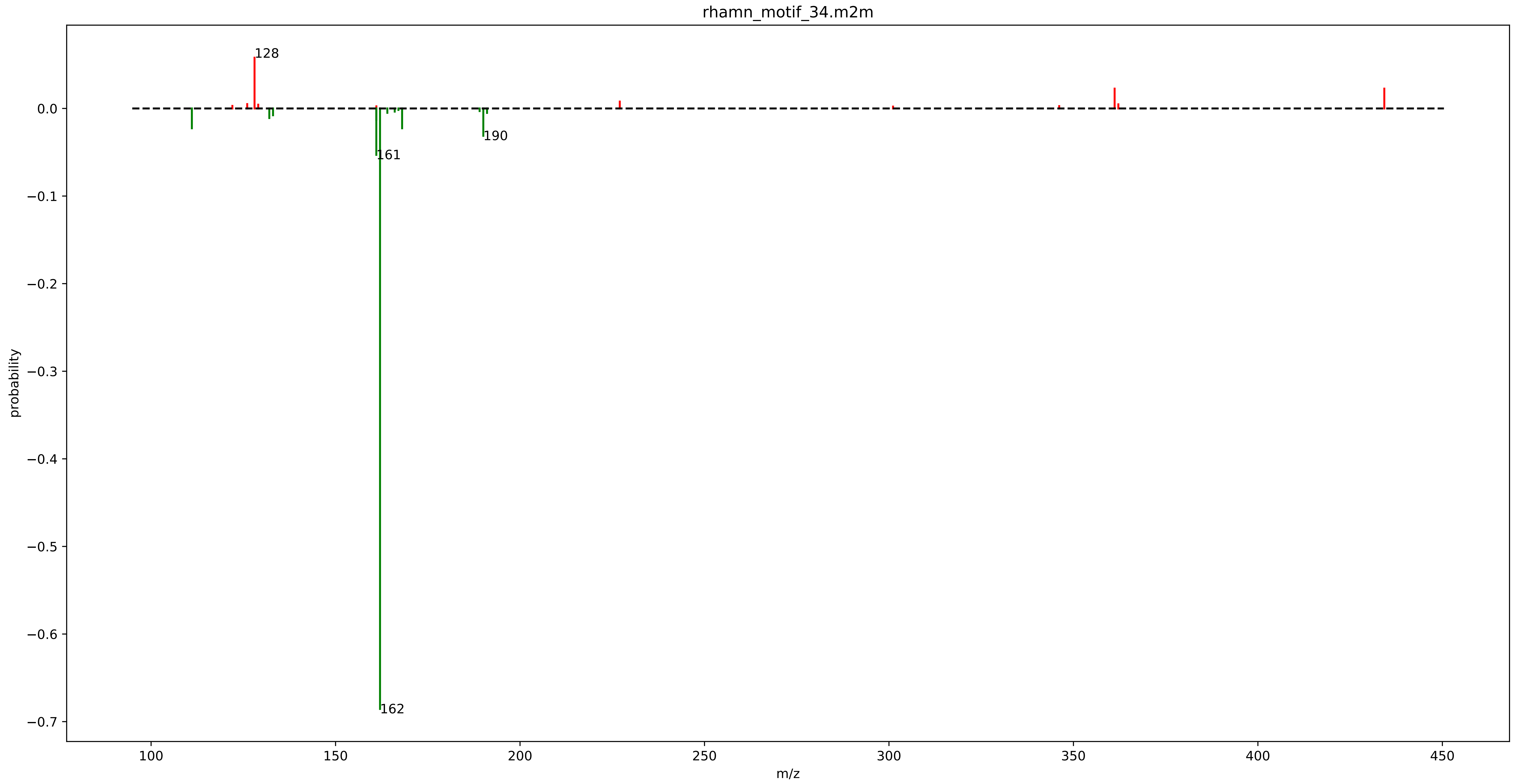

rhamn_motif_34.m2m
128
161
190
162
0.0
−0.1
−0.2
−0.3
−0.4
−0.5
−0.6
−0.7
probability
100
150
200
250
300
350
400
450
m/z

rhamn_motif_34.m2m

loss_162.0525	: 0.69
fragment_128.0375	: 0.06
loss_161.0475	: 0.05
loss_190.0475	: 0.03
loss_111.0525	: 0.02
fragment_434.2625	: 0.02
fragment_361.1625	: 0.02
loss_168.0375	: 0.02
loss_161.0525	: 0.01
loss_132.0425	: 0.01
fragment_227.0375	: 0.01
loss_133.0525	: 0.01
loss_191.0575	: 0.01

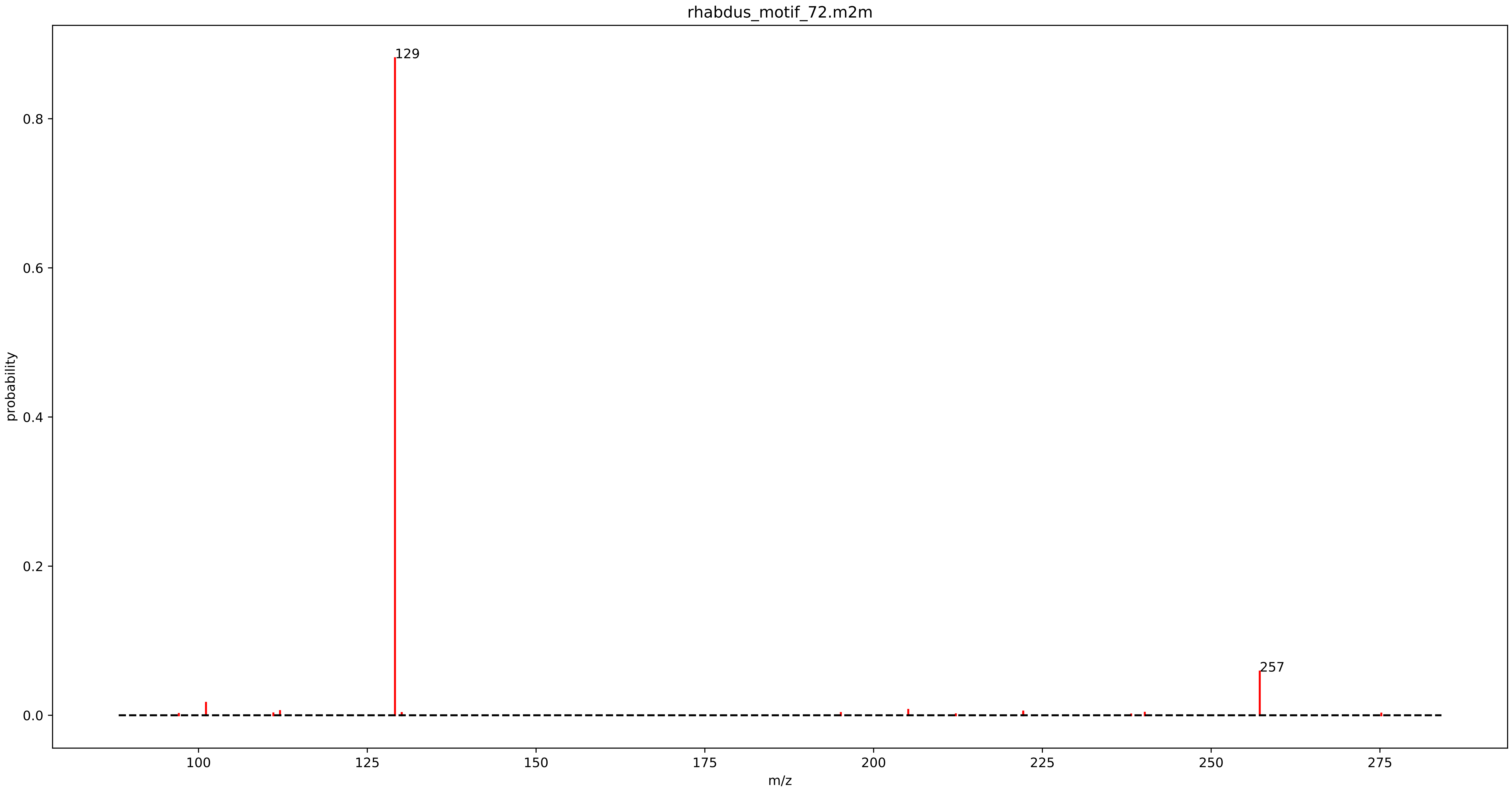

rhabdus_motif_72.m2m
129
257
probability
0.0
0.2
0.4
0.6
0.8
100
125
150
175
200
225
250
275
m/z

rhabdus_motif_72.m2m

fragment_129.1025	: 0.88
fragment_257.1975	: 0.06
fragment_101.1075	: 0.02
fragment_205.1325	: 0.01
fragment_112.0725	: 0.01
fragment_222.1625	: 0.01

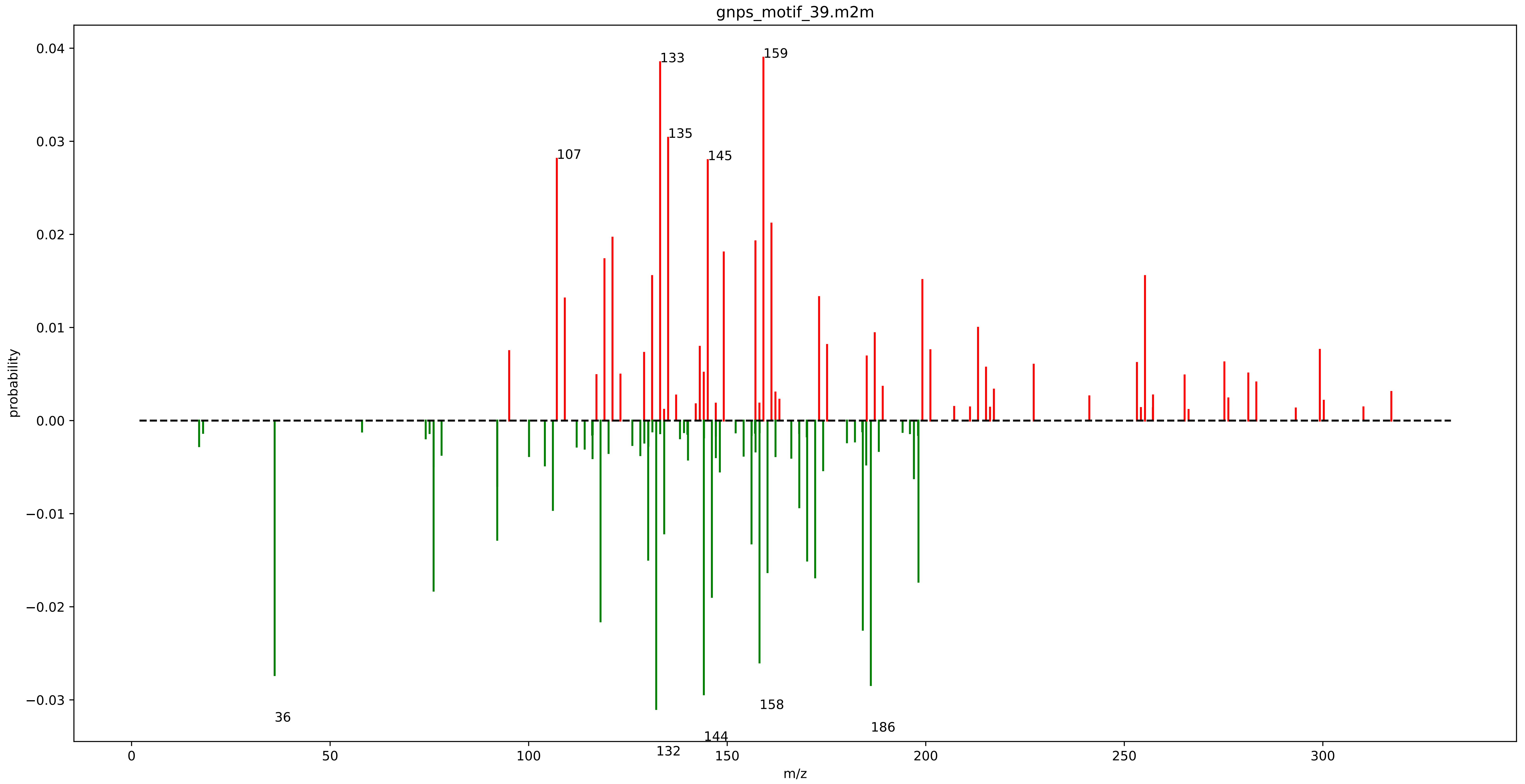

gnps_motif_39.m2m
0.04
0.03
0.02
0.01
0.00
−0.01
−0.02
−0.03
probability
133
159
135
107
145
36
158
186
144
132
0
50
100
150
200
250
300
m/z

gnps_motif_39.m2m

fragment_159.1175	: 0.04
fragment_133.1025	: 0.04
loss_132.1175	: 0.03
fragment_135.1175	: 0.03
loss_144.1125	: 0.03
loss_186.1625	: 0.03
fragment_107.0875	: 0.03
fragment_145.1025	: 0.03
loss_36.0225	: 0.03
loss_158.1325	: 0.03
loss_184.1475	: 0.02
loss_118.0975	: 0.02
fragment_161.1325	: 0.02
fragment_121.1025	: 0.02
fragment_157.1025	: 0.02
loss_146.1325	: 0.02
loss_76.0525	: 0.02
fragment_149.1325	: 0.02
fragment_119.0875	: 0.02
loss_198.1625	: 0.02
loss_172.1475	: 0.02
loss_160.1475	: 0.02
fragment_255.2125	: 0.02
fragment_131.0875	: 0.02
fragment_199.1475	: 0.02
loss_170.1325	: 0.02
loss_130.0975	: 0.01
fragment_173.1325	: 0.01
loss_156.1175	: 0.01
fragment_109.1025	: 0.01
loss_92.0825	: 0.01
loss_134.1325	: 0.01
fragment_213.1625	: 0.01
loss_106.0975	: 0.01
fragment_187.1475	: 0.01
loss_168.1525	: 0.01
fragment_175.1475	: 0.01
fragment_143.0875	: 0.01
fragment_299.2375	: 0.01
fragment_201.1625	: 0.01
fragment_95.0875	: 0.01
fragment_129.0675	: 0.01
loss_92.0875	: 0.01
fragment_185.1325	: 0.01
fragment_275.2025	: 0.01
loss_197.0175	: 0.01
fragment_253.1775	: 0.01
fragment_227.1775	: 0.01
fragment_215.1775	: 0.01
loss_148.1475	: 0.01
loss_174.1625	: 0.01
fragment_144.0925	: 0.01
fragment_281.2275	: 0.01

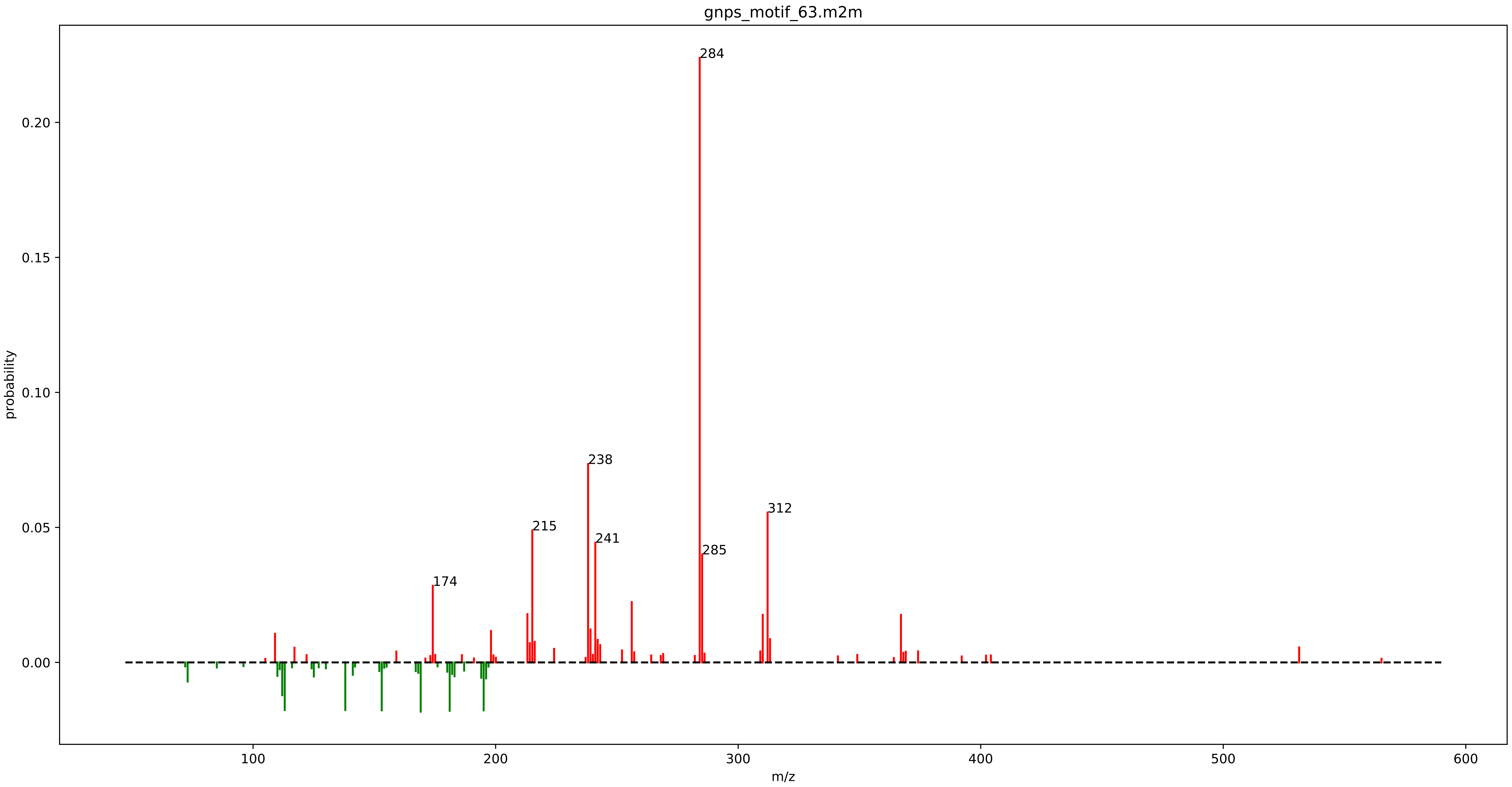

gnps_motif_63.m2m
probability
m/z
0.00
0.05
0.10
0.15
0.20
100
200
300
400
500
600
284
238
312
215
241
285
174

gnps_motif_63.m2m

fragment_284.1375	: 0.22
fragment_238.1225	: 0.07
fragment_312.1325	: 0.06
fragment_215.1175	: 0.05
fragment_241.0975	: 0.04
fragment_285.1425	: 0.04
fragment_174.0925	: 0.03
fragment_256.1325	: 0.02
loss_169.1125	: 0.02
loss_181.0575	: 0.02
fragment_213.1025	: 0.02
loss_195.0675	: 0.02
loss_153.0425	: 0.02
loss_113.0475	: 0.02
loss_138.0225	: 0.02
fragment_367.1125	: 0.02
fragment_310.1325	: 0.02
fragment_239.1275	: 0.01
loss_112.0075	: 0.01
fragment_198.0925	: 0.01
fragment_109.0425	: 0.01
fragment_313.1375	: 0.01
fragment_242.0975	: 0.01
fragment_241.1325	: 0.01
fragment_216.1225	: 0.01
fragment_214.1075	: 0.01
loss_73.0175	: 0.01
fragment_243.1125	: 0.01
fragment_241.1025	: 0.01
loss_196.0825	: 0.01
loss_194.0675	: 0.01
fragment_531.2875	: 0.01
fragment_117.0675	: 0.01
loss_125.0475	: 0.01
loss_183.0675	: 0.01
fragment_224.1075	: 0.01

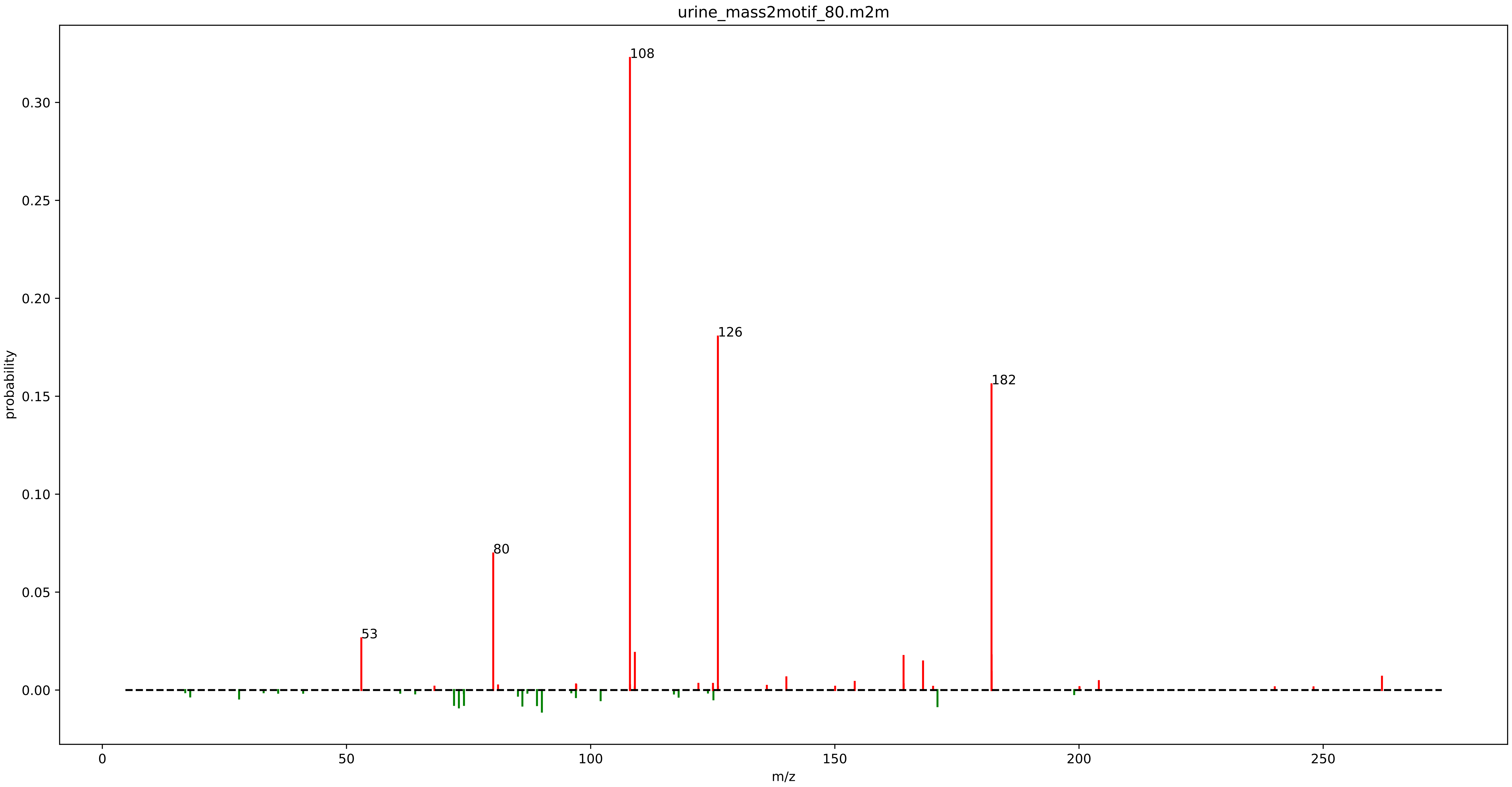

urine_mass2motif_80.m2m
probability
m/z
108
126
182
80
53
0.30
0.25
0.20
0.15
0.10
0.05
0.00
0
50
100
150
200
250

urine_mass2motif_80.m2m

fragment_108.0425	: 0.32
fragment_126.0525	: 0.18
fragment_182.0825	: 0.16
fragment_80.0475	: 0.07
fragment_53.0375	: 0.03
fragment_109.0525	: 0.02
fragment_182.1025	: 0.02
fragment_164.0725	: 0.02
fragment_168.0625	: 0.01
loss_90.0125	: 0.01
fragment_182.1175	: 0.01
loss_73.0175	: 0.01
loss_171.0175	: 0.01
loss_86.0175	: 0.01
loss_89.0075	: 0.01
loss_74.0575	: 0.01
loss_72.0225	: 0.01
fragment_262.0375	: 0.01
fragment_140.0725	: 0.01
loss_102.0425	: 0.01

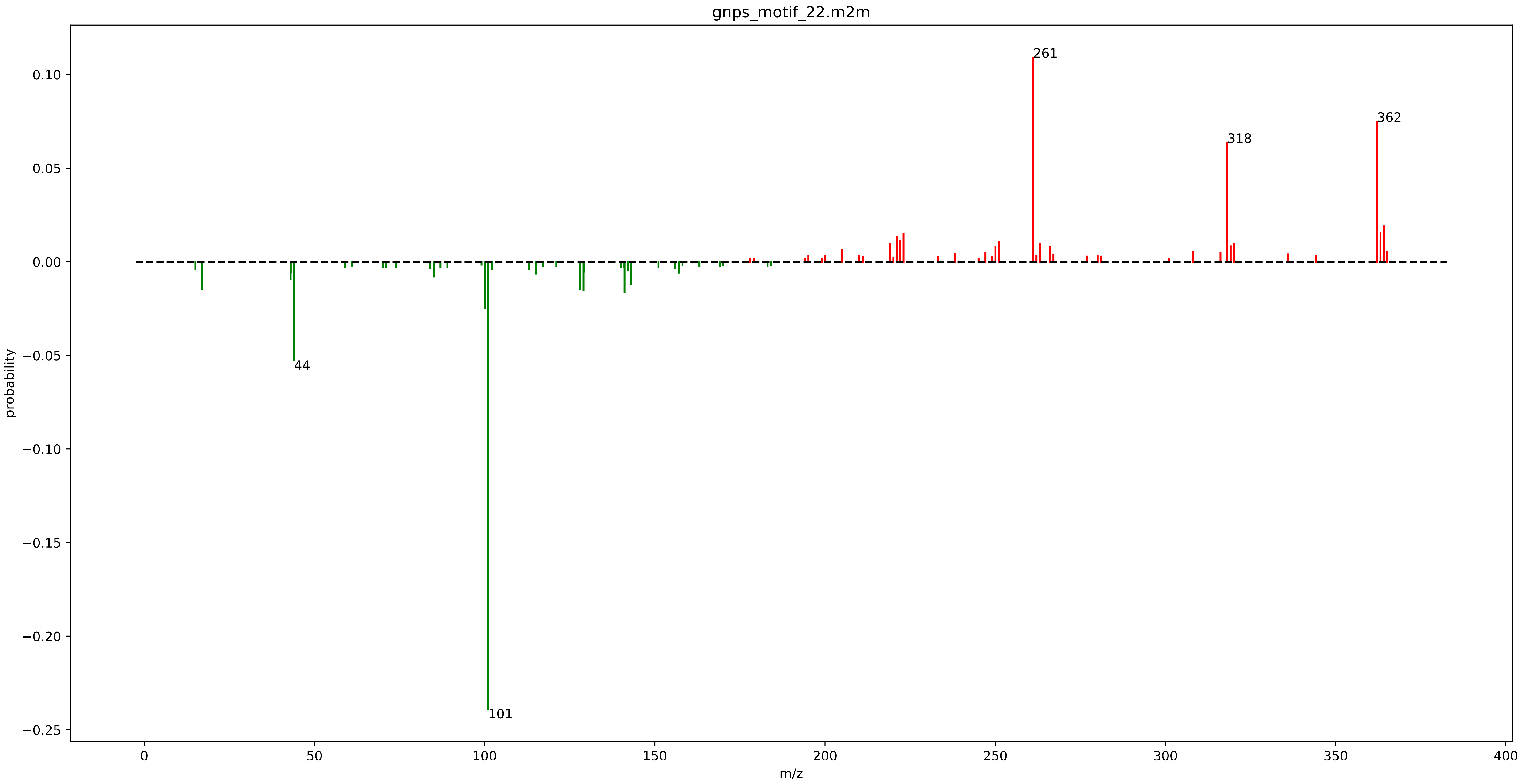
gnps_motif_22.m2m
261
318
362
44
101
0.10
0.05
0.00
−0.05
−0.10
−0.15
−0.20
−0.25
probability
0
50
100
150
200
250
300
350
400
m/z

gnps_motif_22.m2m

loss_101.0475	: 0.24
fragment_261.1025	: 0.11
fragment_362.1475	: 0.07
fragment_318.1625	: 0.06
loss_43.9875	: 0.05
loss_100.0425	: 0.02
fragment_364.1125	: 0.02
loss_141.0775	: 0.02
fragment_363.1575	: 0.02
loss_129.0425	: 0.02
fragment_223.0625	: 0.01
loss_128.0325	: 0.01
loss_17.0225	: 0.01
fragment_221.0725	: 0.01
loss_143.0975	: 0.01
fragment_222.0525	: 0.01
fragment_363.1525	: 0.01
loss_100.0375	: 0.01
fragment_251.0575	: 0.01
fragment_320.1225	: 0.01
fragment_219.0575	: 0.01
fragment_263.0675	: 0.01
loss_42.9875	: 0.01
fragment_319.1675	: 0.01
loss_85.0225	: 0.01
fragment_266.0775	: 0.01
fragment_250.0475	: 0.01
loss_115.0625	: 0.01
fragment_205.0775	: 0.01
loss_157.1125	: 0.01
loss_43.0225	: 0.01
fragment_308.0775	: 0.01
fragment_365.1175	: 0.01
loss_129.0775	: 0.01

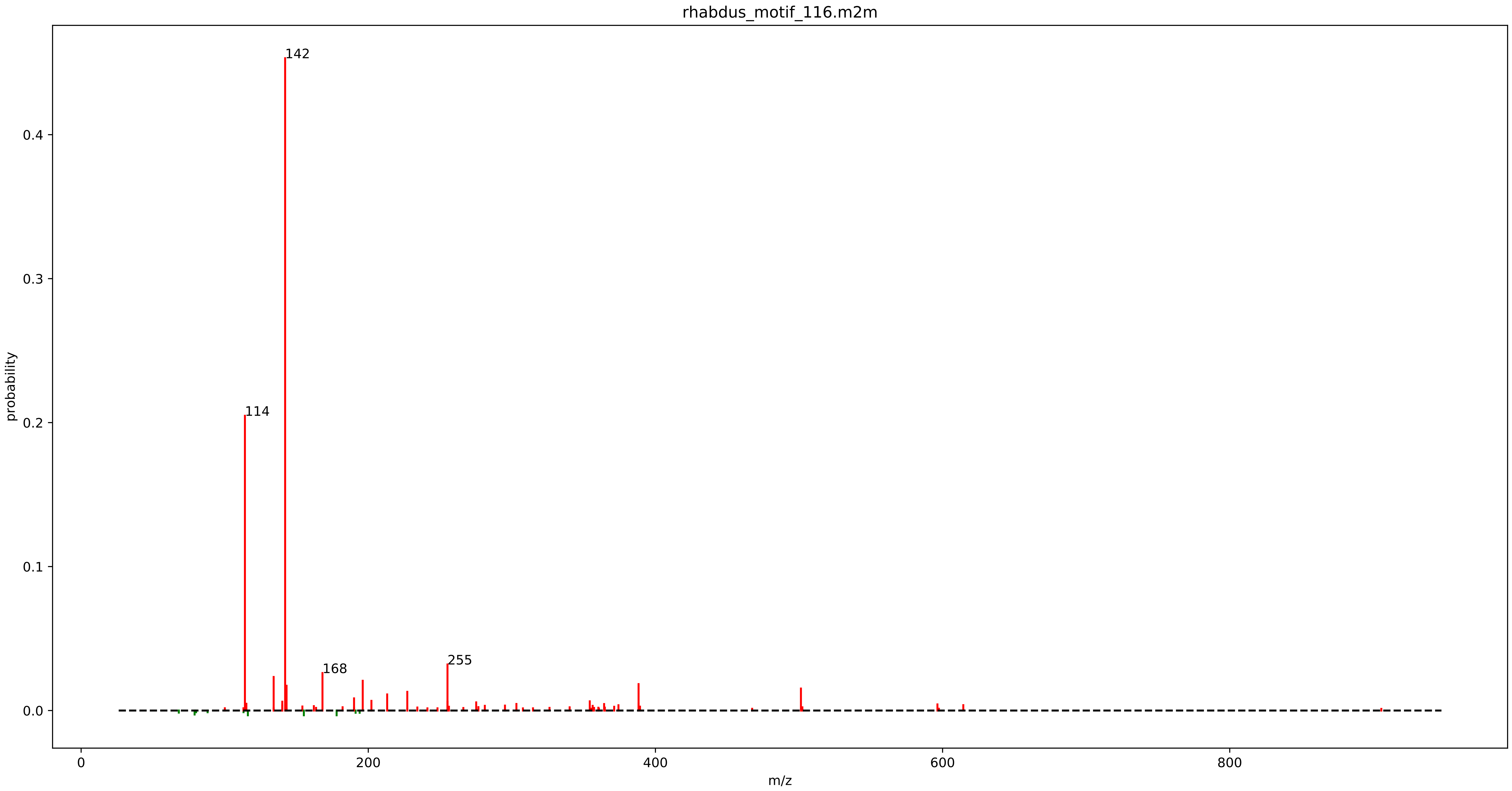

rhabdus_motif_116.m2m
142
114
168
255
probability
m/z
0.0
0.1
0.2
0.3
0.4
0
200
400
600
800

rhabdus_motif_116.m2m

fragment_142.0875	: 0.45
fragment_114.0875	: 0.20
fragment_255.1675	: 0.03
fragment_168.1025	: 0.03
fragment_134.0975	: 0.02
fragment_196.1325	: 0.02
fragment_388.2575	: 0.02
fragment_143.0875	: 0.02
fragment_501.3425	: 0.02
fragment_227.1775	: 0.01
fragment_213.1625	: 0.01
fragment_190.0875	: 0.01
fragment_202.1925	: 0.01
fragment_354.2725	: 0.01
fragment_140.1075	: 0.01
fragment_255.1725	: 0.01
fragment_275.1725	: 0.01

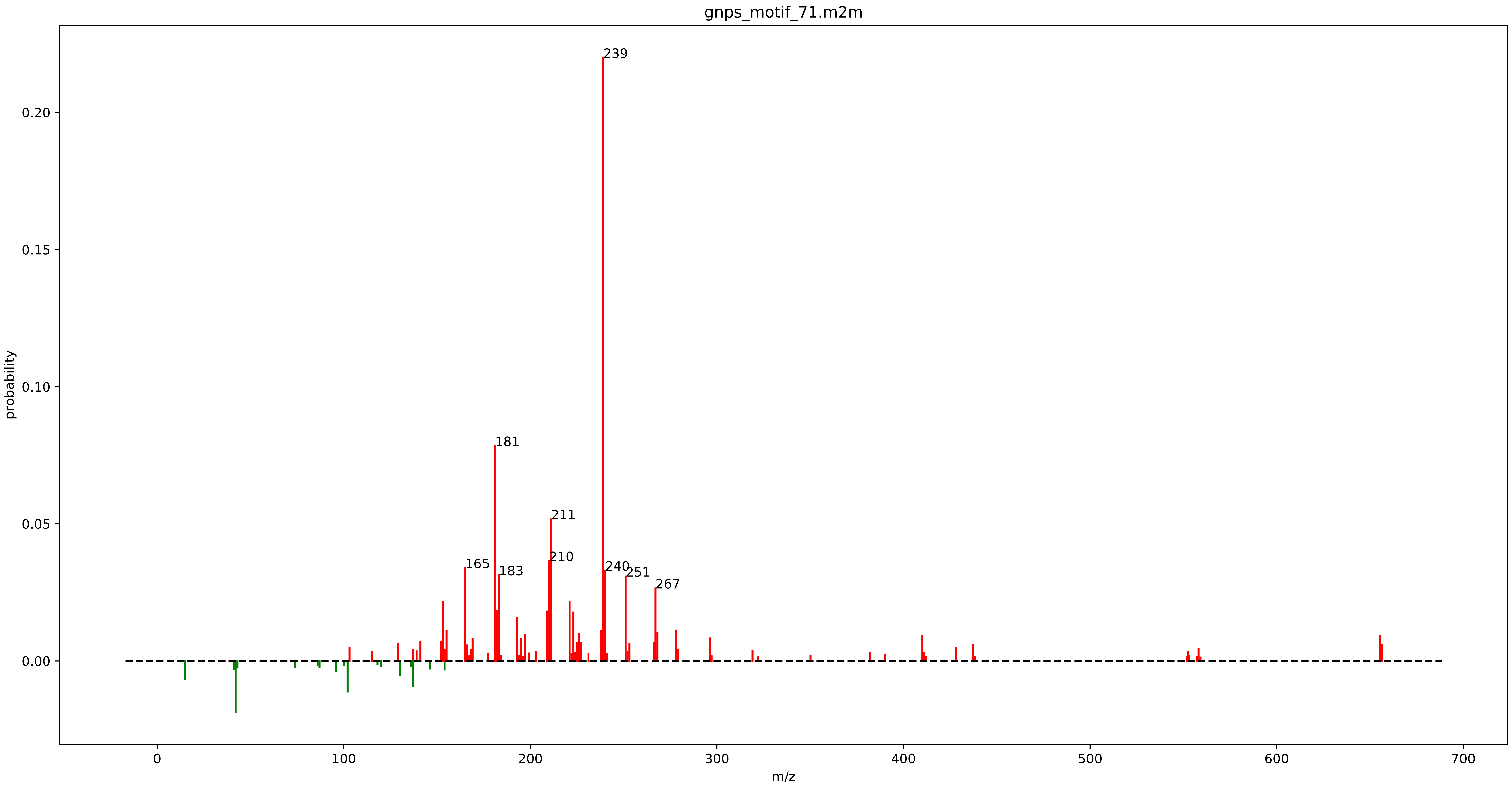

gnps_motif_71.m2m
probability
m/z
0.20
0.15
0.10
0.05
0.00
0
100
200
300
400
500
600
700
239
181
211
210
165
183
240
251
267

gnps_motif_71.m2m

fragment_239.0725	: 0.22
fragment_181.0625	: 0.08
fragment_211.0775	: 0.05
fragment_210.0675	: 0.04
fragment_165.0675	: 0.03
fragment_240.0725	: 0.03
fragment_183.0825	: 0.03
fragment_251.0725	: 0.03
fragment_267.0625	: 0.03
fragment_221.0575	: 0.02
fragment_153.0675	: 0.02
loss_42.0475	: 0.02
fragment_182.0725	: 0.02
fragment_209.0575	: 0.02
fragment_223.0775	: 0.02
fragment_193.0675	: 0.02
loss_102.0425	: 0.01
fragment_278.0825	: 0.01
fragment_238.0625	: 0.01
fragment_155.0875	: 0.01
fragment_268.0725	: 0.01
fragment_226.0625	: 0.01
fragment_197.0575	: 0.01
fragment_655.4225	: 0.01
loss_137.0525	: 0.01
fragment_410.0625	: 0.01
fragment_296.0925	: 0.01
fragment_195.0825	: 0.01
fragment_169.0625	: 0.01
fragment_152.0625	: 0.01
fragment_141.0675	: 0.01
loss_15.0225	: 0.01
fragment_266.1725	: 0.01
fragment_227.0725	: 0.01
fragment_225.0525	: 0.01
fragment_129.0325	: 0.01
fragment_253.0875	: 0.01
fragment_656.4325	: 0.01
fragment_437.1175	: 0.01
fragment_166.0775	: 0.01
loss_130.0625	: 0.01

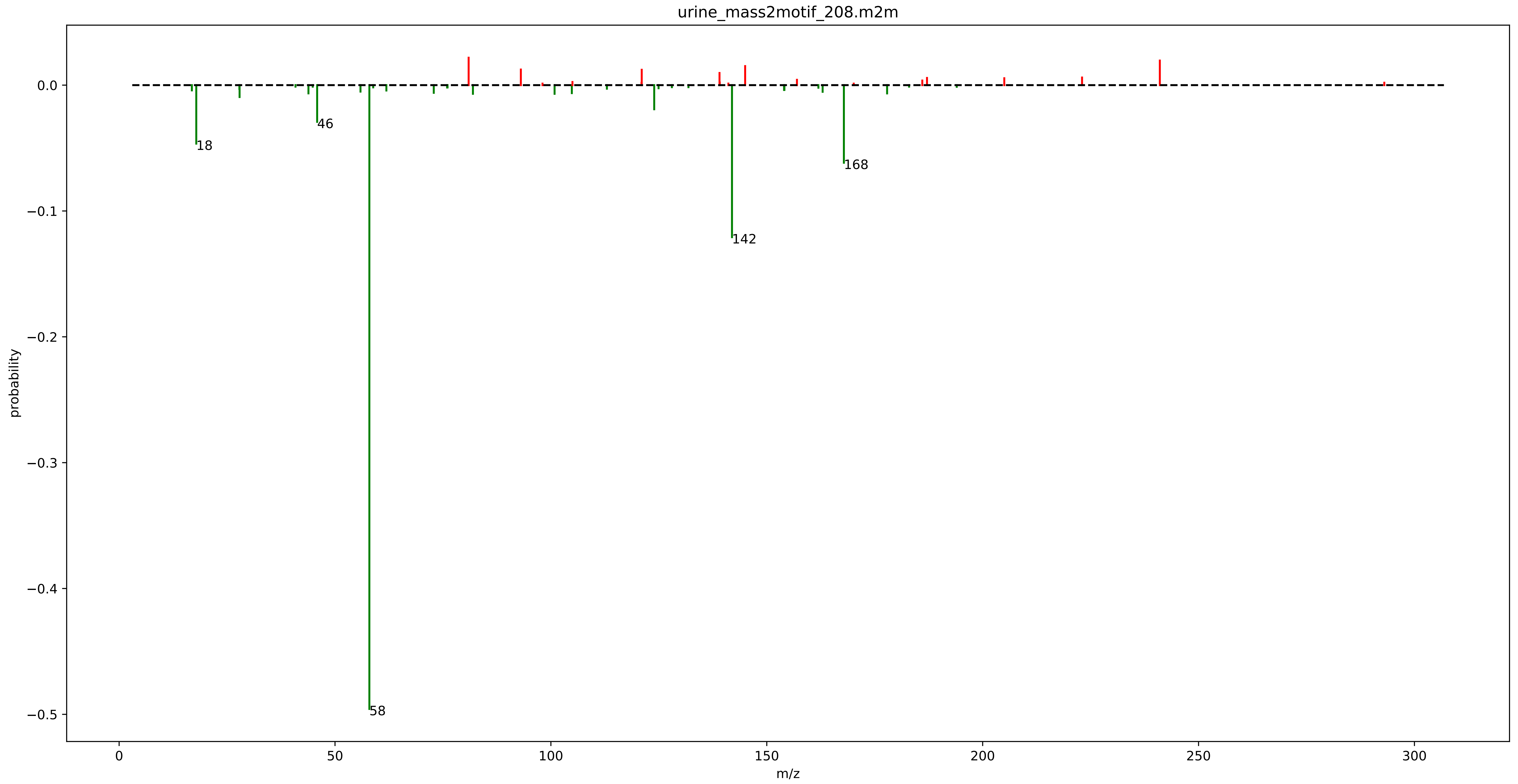

urine_mass2motif_208.m2m
probability
m/z
0.0
−0.1
−0.2
−0.3
−0.4
−0.5
0
50
100
150
200
250
300
18
46
58
142
168

urine_mass2motif_208.m2m

loss_57.9575	: 0.50
loss_141.9425	: 0.12
loss_167.8575	: 0.06
loss_17.8675	: 0.05
loss_45.8625	: 0.03
fragment_80.9475	: 0.02
fragment_241.0325	: 0.02
loss_123.9275	: 0.02
fragment_144.9875	: 0.02
fragment_93.0425	: 0.01
fragment_121.0375	: 0.01
fragment_139.0525	: 0.01
loss_27.8975	: 0.01
loss_81.9425	: 0.01
loss_100.8525	: 0.01
loss_177.8675	: 0.01
loss_43.8475	: 0.01
loss_104.8475	: 0.01
loss_72.8725	: 0.01
fragment_223.0175	: 0.01
fragment_187.1075	: 0.01
fragment_205.0075	: 0.01
loss_162.9375	: 0.01
loss_55.9025	: 0.01

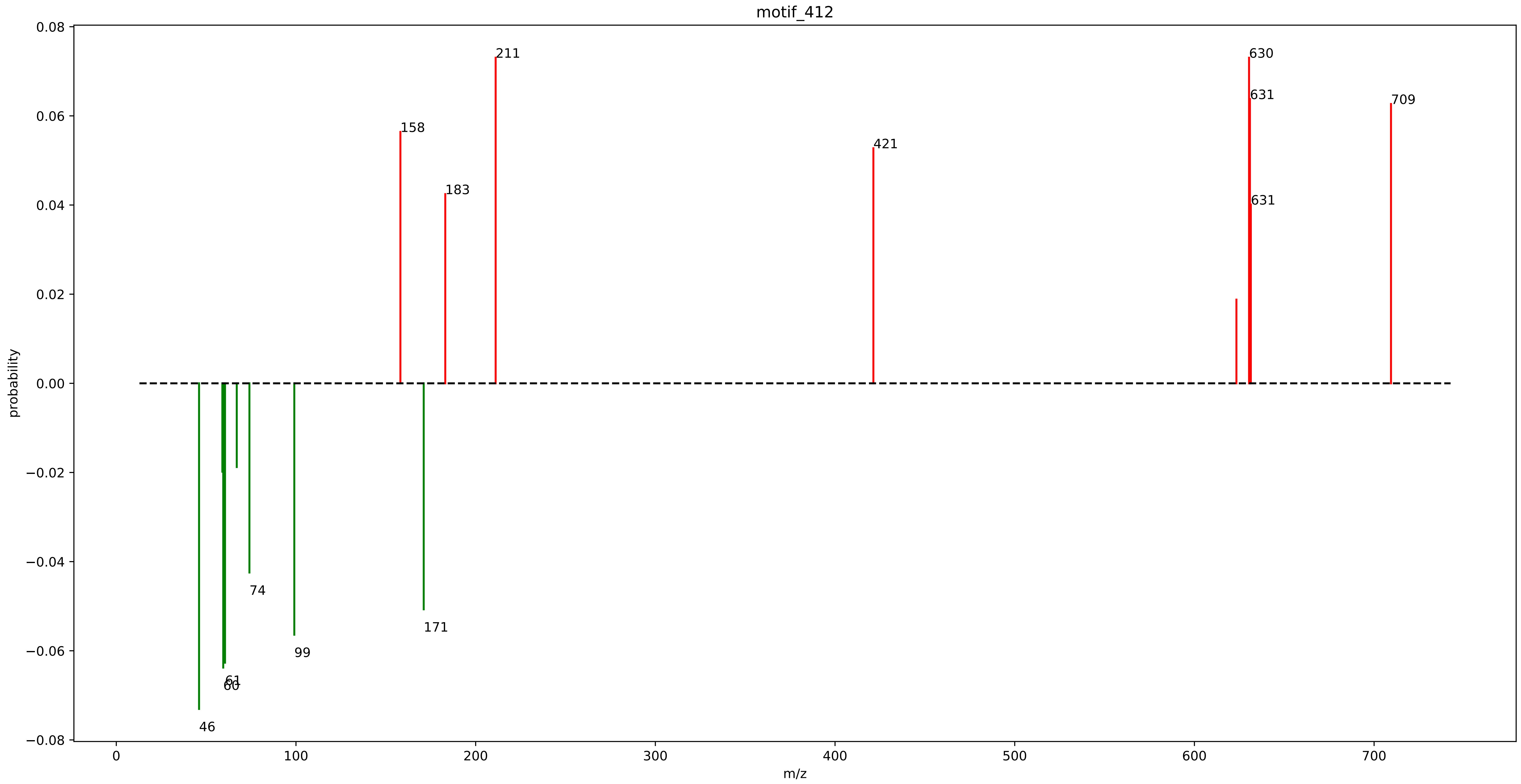
motif_412
probability
m/z
0.08
0.06
0.04
0.02
0.00
−0.02
−0.04
−0.06
−0.08
0
100
200
300
400
500
600
700
158
183
211
421
630
631
631
709
46
60
61
74
99
171

motif_412

fragment_211.1225	: 0.07
loss_46.0625	: 0.07
fragment_630.4075	: 0.07
fragment_630.9075	: 0.06
loss_59.5175	: 0.06
loss_60.5175	: 0.06
fragment_709.4075	: 0.06
fragment_158.1175	: 0.06
loss_99.0675	: 0.06
fragment_421.3425	: 0.05
loss_171.0875	: 0.05
loss_74.0925	: 0.04
fragment_183.0875	: 0.04
fragment_631.4075	: 0.04
loss_59.0175	: 0.02
fragment_623.3825	: 0.02
loss_67.0425	: 0.02

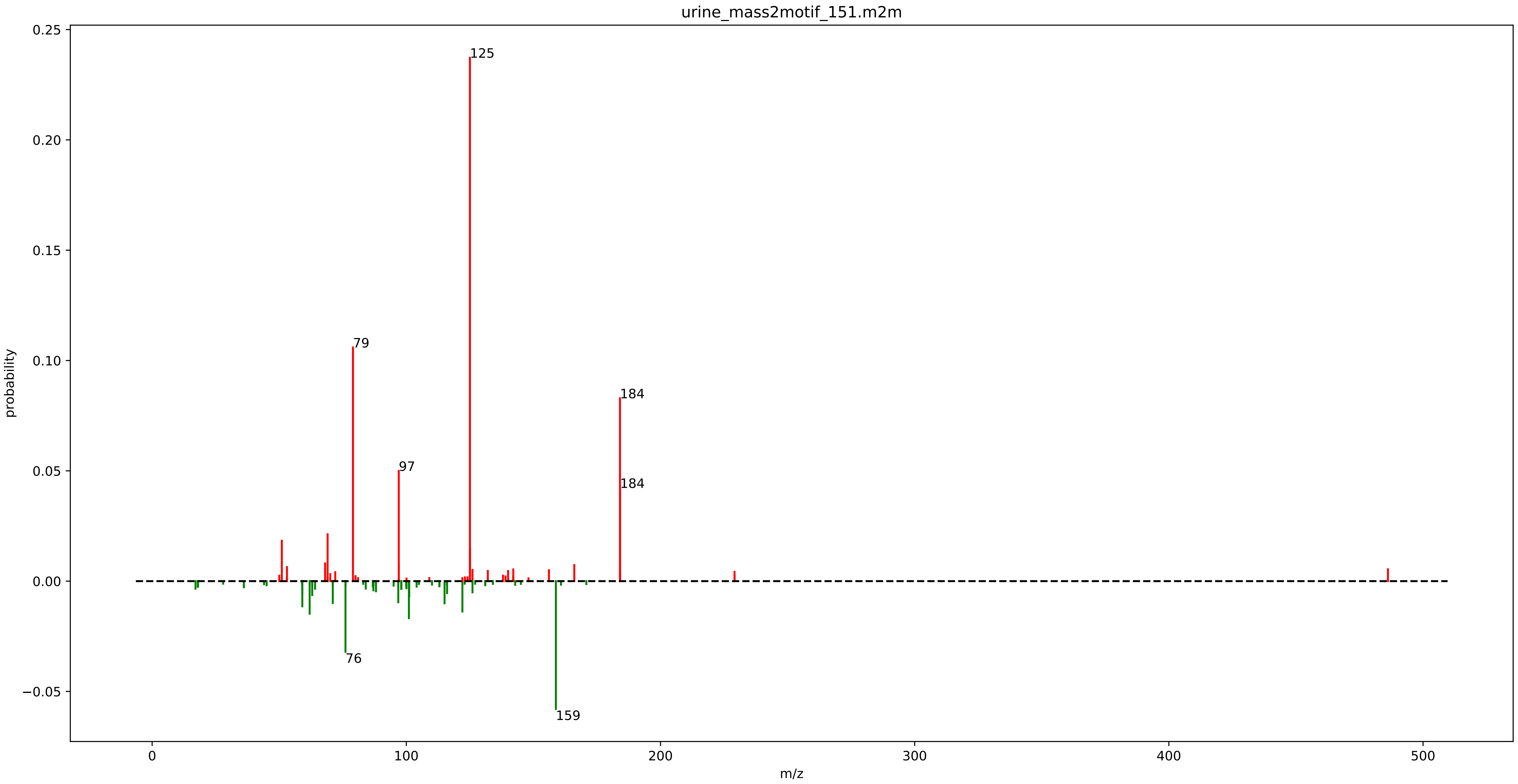
urine_mass2motif_151.m2m
probability
m/z
0.25
0.20
0.15
0.10
0.05
0.00
−0.05
0
100
200
300
400
500
125
79
184
97
184
76
159

urine_mass2motif_151.m2m

fragment_125.0225	: 0.24
fragment_79.0175	: 0.11
fragment_184.0625	: 0.08
loss_158.8375	: 0.06
fragment_97.0275	: 0.05
fragment_184.0975	: 0.04
loss_76.0625	: 0.03
fragment_69.0325	: 0.02
fragment_51.0225	: 0.02
loss_101.0125	: 0.02
fragment_125.0775	: 0.01
loss_61.9725	: 0.01
loss_122.0675	: 0.01
fragment_125.0475	: 0.01
loss_59.0825	: 0.01
loss_115.0425	: 0.01
loss_71.0725	: 0.01
loss_96.8375	: 0.01
fragment_68.0475	: 0.01
fragment_97.0475	: 0.01
fragment_166.0875	: 0.01
loss_101.1475	: 0.01
fragment_184.1075	: 0.01
fragment_53.0375	: 0.01
loss_62.9775	: 0.01
loss_59.0925	: 0.01
loss_116.0425	: 0.01
fragment_486.1925	: 0.01
fragment_142.0875	: 0.01
fragment_126.0275	: 0.01
loss_126.0325	: 0.01

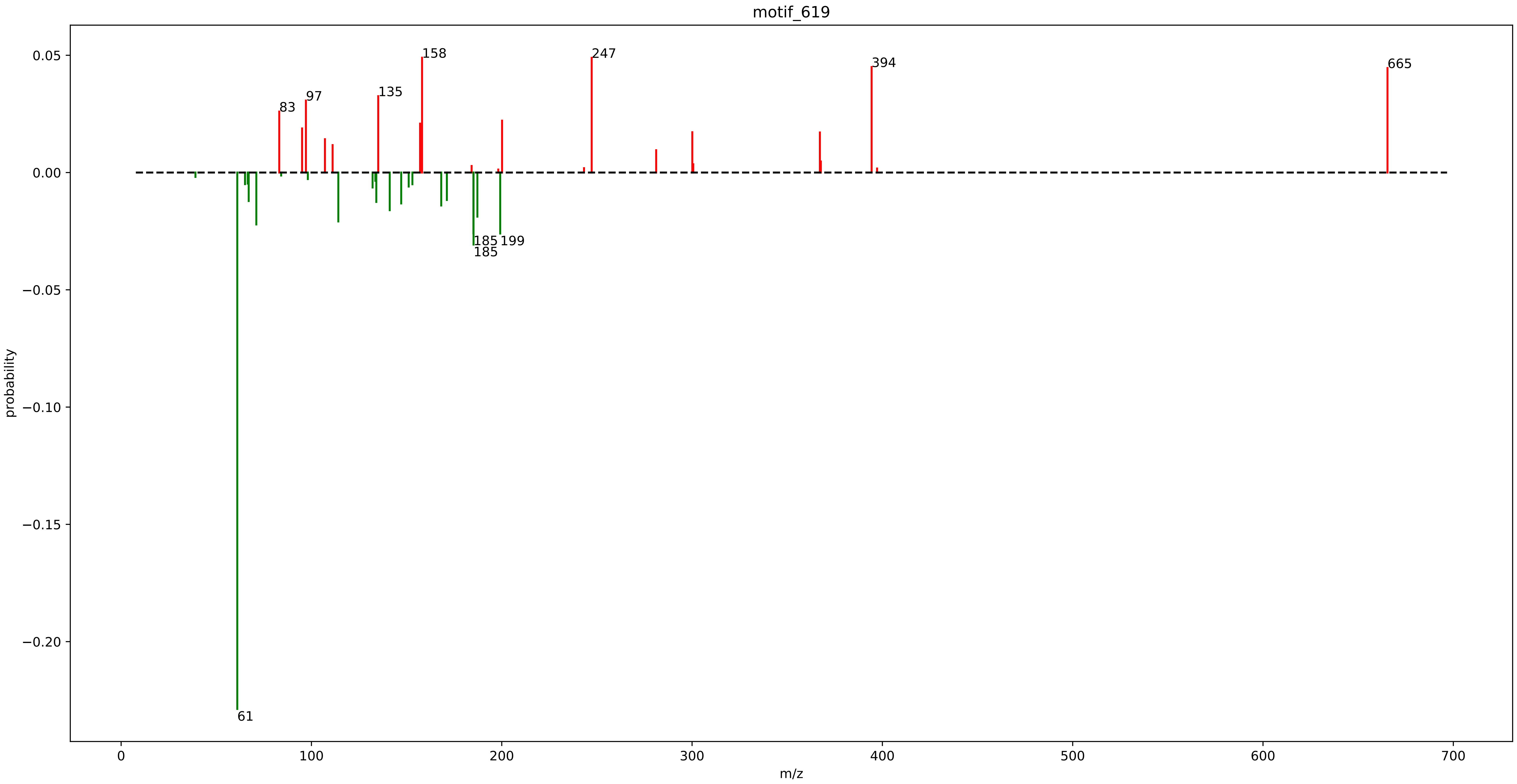

motif_619
0.05
0.00
−0.05
−0.10
−0.15
−0.20
probability
83
97
135
158
247
394
665
185
185
199
61
0
100
200
300
400
500
600
700
m/z

motif_619

loss_61.0225	: 0.23
fragment_158.1175	: 0.05
fragment_247.2425	: 0.05
fragment_394.3325	: 0.04
fragment_665.4025	: 0.04
fragment_135.0825	: 0.03
fragment_97.0975	: 0.03
loss_185.1775	: 0.03
fragment_83.0825	: 0.03
loss_199.1925	: 0.03
loss_185.1025	: 0.03
fragment_200.1625	: 0.02
loss_71.0375	: 0.02
fragment_157.0725	: 0.02
loss_114.1275	: 0.02
loss_187.1925	: 0.02
fragment_95.0825	: 0.02
fragment_300.1125	: 0.02
fragment_367.1475	: 0.02
loss_141.1375	: 0.02
fragment_107.0825	: 0.01
loss_168.1875	: 0.01
loss_147.1625	: 0.01
loss_134.0725	: 0.01
loss_67.0325	: 0.01
loss_171.1625	: 0.01
fragment_111.1175	: 0.01
fragment_281.1375	: 0.01
loss_132.1225	: 0.01
loss_151.0975	: 0.01

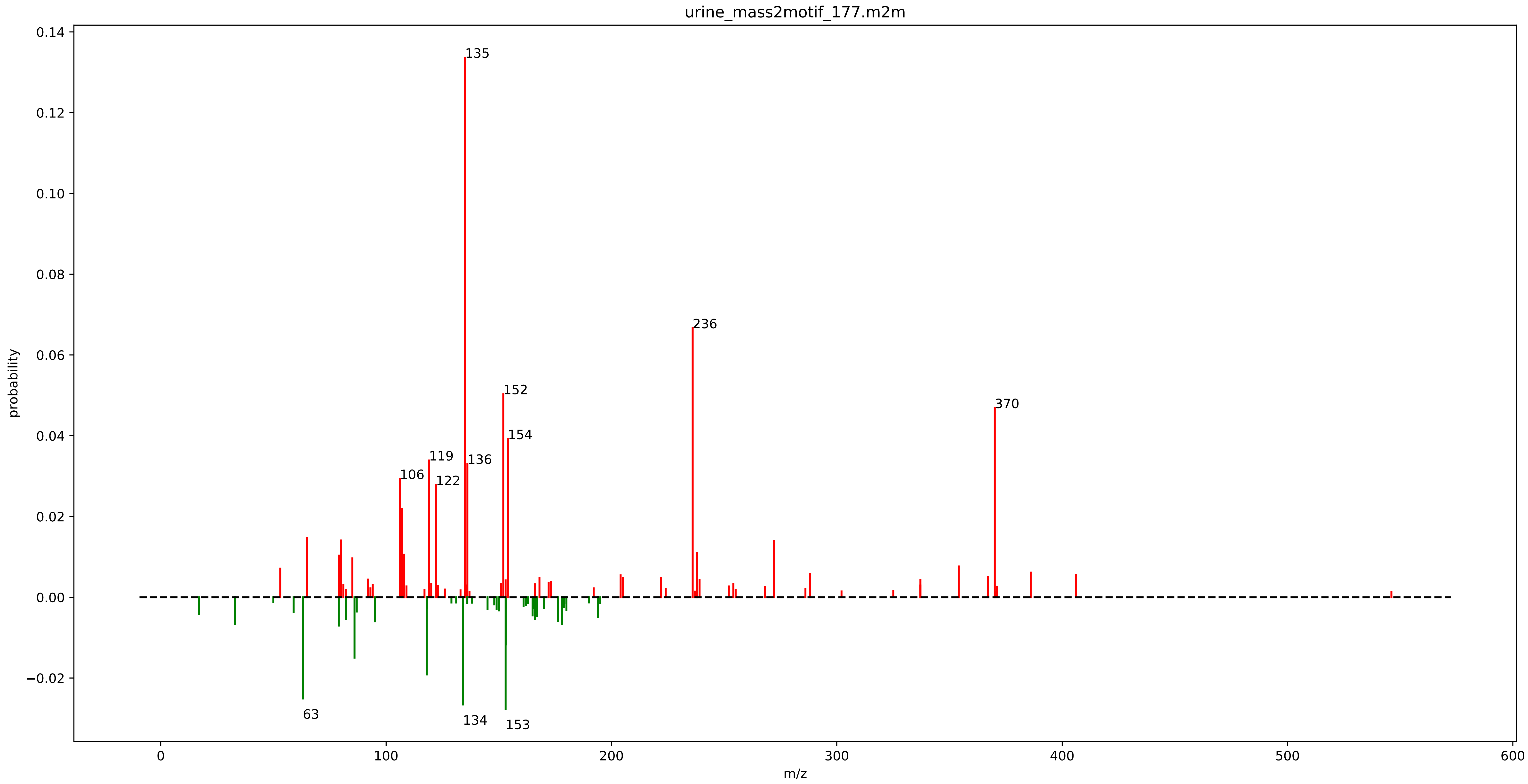
urine_mass2motif_177.m2m
probability
m/z
0.14
0.12
0.10
0.08
0.06
0.04
0.02
0.00
−0.02
0
100
200
300
400
500
600
135
236
152
370
154
119
136
106
122
63
134
153

urine_mass2motif_177.m2m

fragment_135.0525	: 0.13
fragment_236.0325	: 0.07
fragment_152.0175	: 0.05
fragment_370.0825	: 0.05
fragment_154.0325	: 0.04
fragment_119.0575	: 0.03
fragment_136.0775	: 0.03
fragment_106.0675	: 0.03
fragment_122.0625	: 0.03
loss_153.0225	: 0.03
loss_134.0625	: 0.03
loss_63.0375	: 0.03
fragment_107.0625	: 0.02
loss_118.0625	: 0.02
loss_85.9875	: 0.01
fragment_65.0375	: 0.01
fragment_80.0475	: 0.01
fragment_272.0875	: 0.01
loss_153.1275	: 0.01
fragment_107.0725	: 0.01
fragment_238.0525	: 0.01
fragment_108.0825	: 0.01
fragment_79.0525	: 0.01
fragment_85.0275	: 0.01
fragment_354.0875	: 0.01
loss_134.1475	: 0.01
fragment_53.0375	: 0.01
loss_79.0175	: 0.01
loss_32.9825	: 0.01
fragment_108.0425	: 0.01
loss_178.0325	: 0.01
fragment_386.0825	: 0.01
loss_95.0125	: 0.01
loss_176.1875	: 0.01
fragment_288.0775	: 0.01
fragment_406.1025	: 0.01
fragment_204.0625	: 0.01
loss_82.1525	: 0.01
loss_166.0175	: 0.01

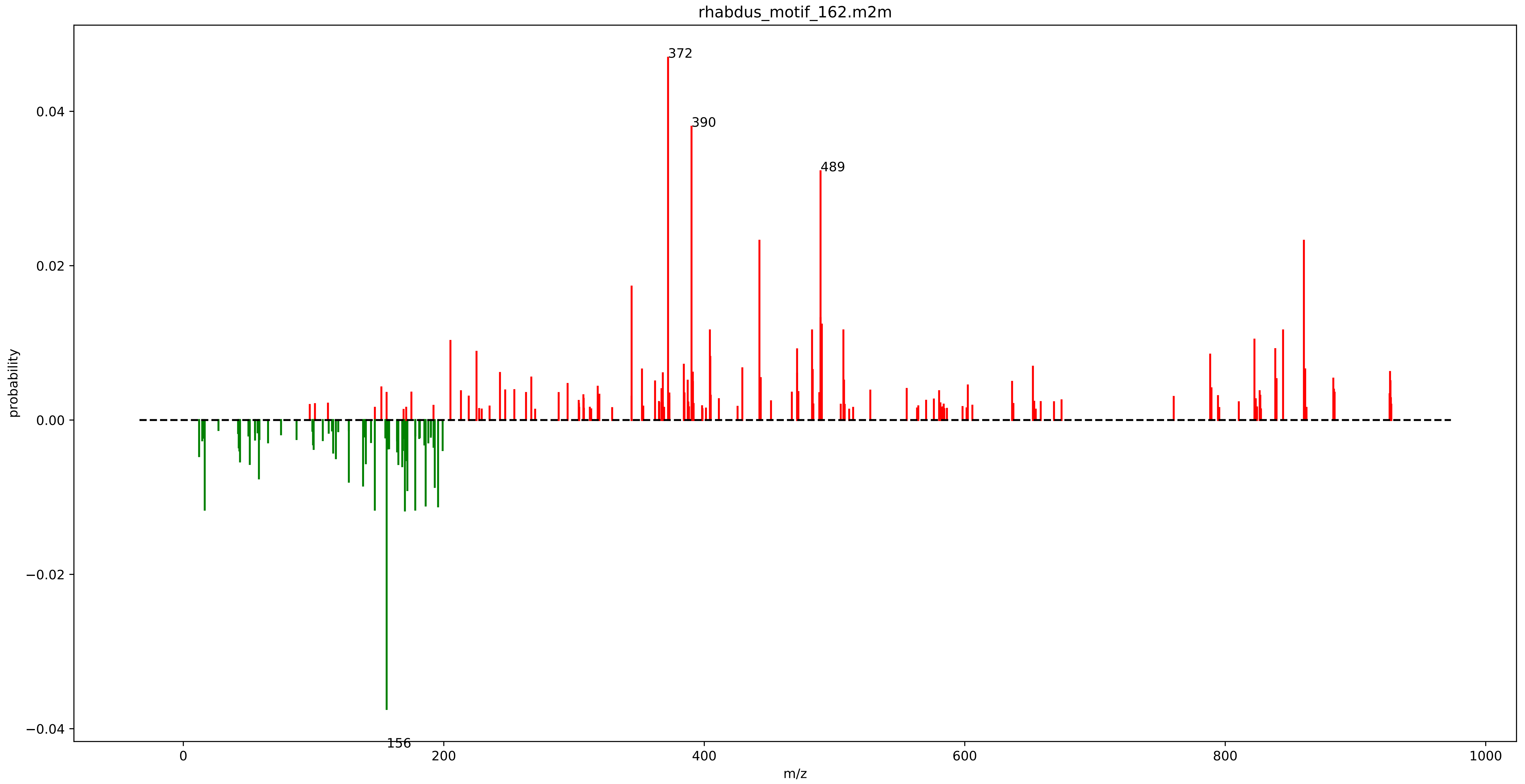

rhabdus_motif_162.m2m
372
390
489
156
0.04
0.02
0.00
−0.02
−0.04
probability
0
200
400
600
800
1000
m/z

rhabdus_motif_162.m2m

fragment_372.1925 : 0.05
fragment_390.2025 : 0.04
loss_156.1225 : 0.04
fragment_489.2675 : 0.03
fragment_860.3925 : 0.02
fragment_442.3475 : 0.02
fragment_344.1975 : 0.02
fragment_489.2725 : 0.01
fragment_490.2725 : 0.01
loss_170.1425 : 0.01
fragment_506.8125 : 0.01
fragment_404.3025 : 0.01
fragment_844.4025 : 0.01
fragment_482.7475 : 0.01
loss_147.0425 : 0.01
loss_16.4325 : 0.01
loss_178.0825 : 0.01
loss_195.6125 : 0.01
loss_186.0775 : 0.01
fragment_822.4175 : 0.01

fragment_205.0975 : 0.01
fragment_838.4125 : 0.01
fragment_471.2575 : 0.01
loss_172.0375 : 0.01
fragment_225.1225 : 0.01
loss_193.0575 : 0.01
loss_138.0175 : 0.01
fragment_788.4325 : 0.01
fragment_404.6375 : 0.01
loss_127.0975 : 0.01
loss_58.0475 : 0.01
fragment_384.2975 : 0.01
fragment_652.3325 : 0.01
fragment_429.2125 : 0.01
fragment_861.3975 : 0.01
fragment_352.1675 : 0.01
fragment_483.2525 : 0.01
fragment_926.5325 : 0.01
fragment_391.2025 : 0.01
fragment_243.1325 : 0.01

fragment_368.1625 : 0.01
fragment_471.2625 : 0.01
loss_168.0875 : 0.01
loss_165.1125 : 0.01
loss_51.0425 : 0.01
loss_140.1625 : 0.01
fragment_267.1925 : 0.01
fragment_443.3525 : 0.01
loss_43.5375 : 0.01
fragment_882.9825 : 0.01
fragment_839.4175 : 0.01
loss_171.1275 : 0.01
fragment_507.3175 : 0.01
fragment_387.2725 : 0.01
fragment_926.8725 : 0.01
fragment_362.2075 : 0.01

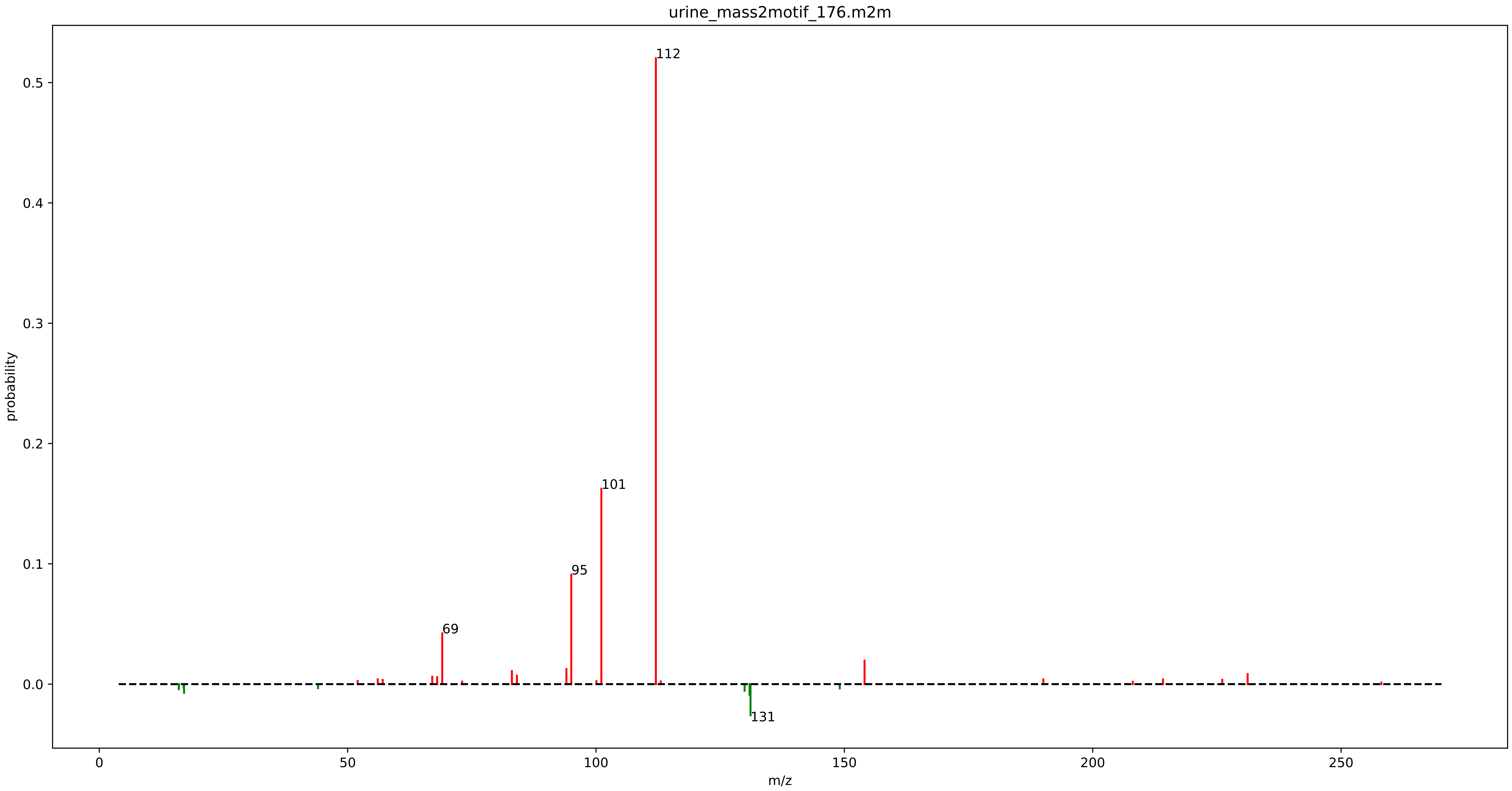

urine_mass2motif_176.m2m
probability
m/z
0.5
0.4
0.3
0.2
0.1
0.0
0
50
100
150
200
250
112
101
95
69
131

urine_mass2motif_176.m2m

fragment_112.0525	: 0.52
fragment_101.0775	: 0.16
fragment_95.0225	: 0.09
fragment_69.0425	: 0.04
loss_131.1075	: 0.03
fragment_154.0625	: 0.02
fragment_94.0425	: 0.01
fragment_83.0675	: 0.01
loss_130.9125	: 0.01
fragment_101.0725	: 0.01
fragment_231.1875	: 0.01
loss_17.0625	: 0.01
fragment_84.0775	: 0.01
fragment_67.0275	: 0.01
fragment_68.0125	: 0.01
loss_129.9125	: 0.01

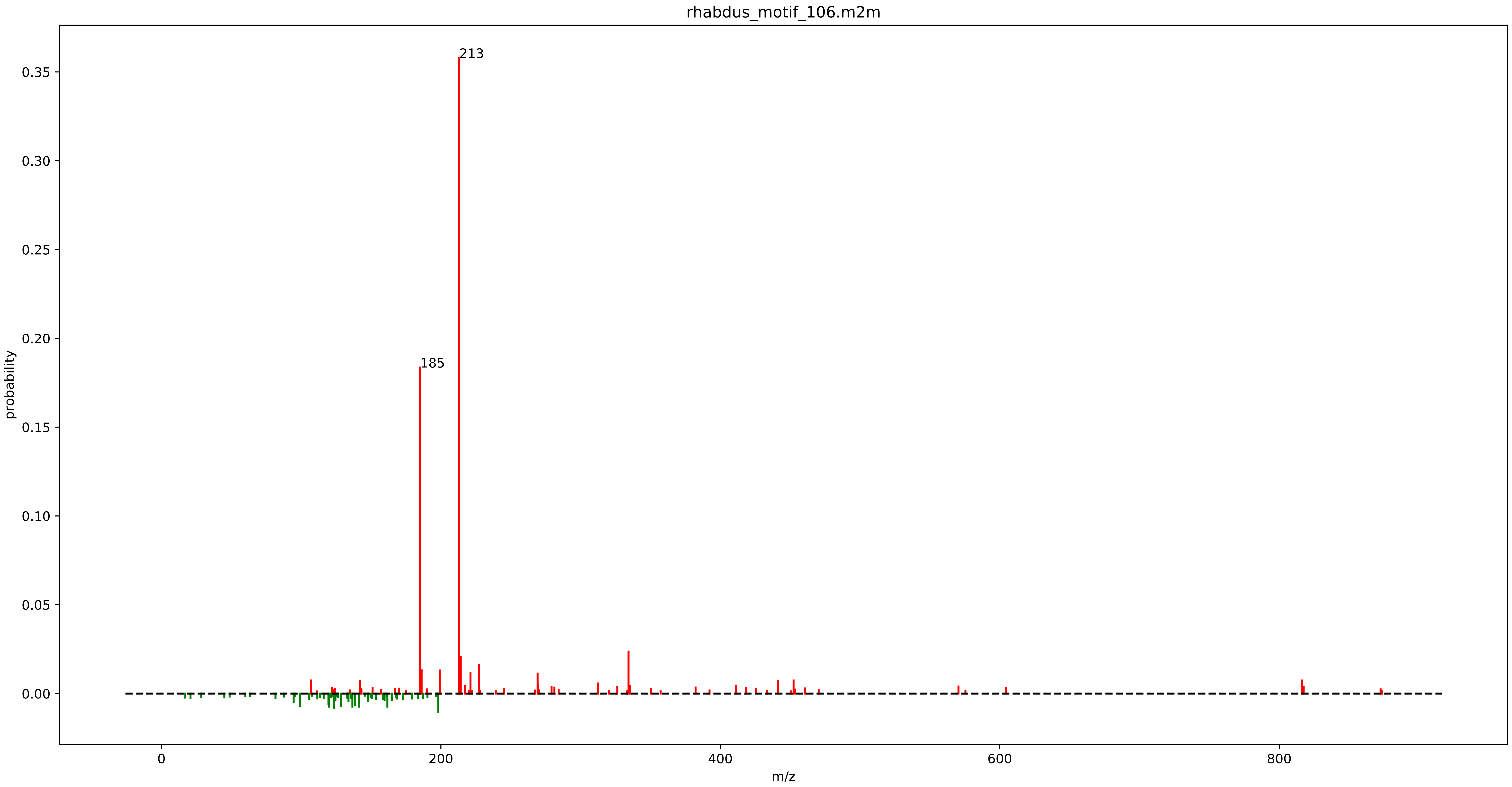
rhabdus_motif_106.m2m
213
185
probability
0.35
0.30
0.25
0.20
0.15
0.10
0.05
0.00
0
200
400
600
800
m/z

rhabdus_motif_106.m2m

fragment_213.1575	: 0.36
fragment_185.1625	: 0.18
fragment_334.2725	: 0.02
fragment_214.1625	: 0.02
fragment_227.1775	: 0.02
fragment_186.1675	: 0.01
fragment_199.1575	: 0.01
fragment_221.1625	: 0.01
fragment_269.1875	: 0.01
loss_198.1375	: 0.01
loss_123.5825	: 0.01
fragment_452.3875	: 0.01
loss_161.6875	: 0.01
loss_141.5775	: 0.01
fragment_107.0525	: 0.01
loss_136.6425	: 0.01
loss_119.8475	: 0.01
fragment_816.4675	: 0.01
fragment_441.2925	: 0.01
fragment_142.0825	: 0.01
loss_128.5525	: 0.01
loss_99.0675	: 0.01
loss_138.5625	: 0.01
loss_119.5325	: 0.01
fragment_312.2275	: 0.01
fragment_269.7025	: 0.01

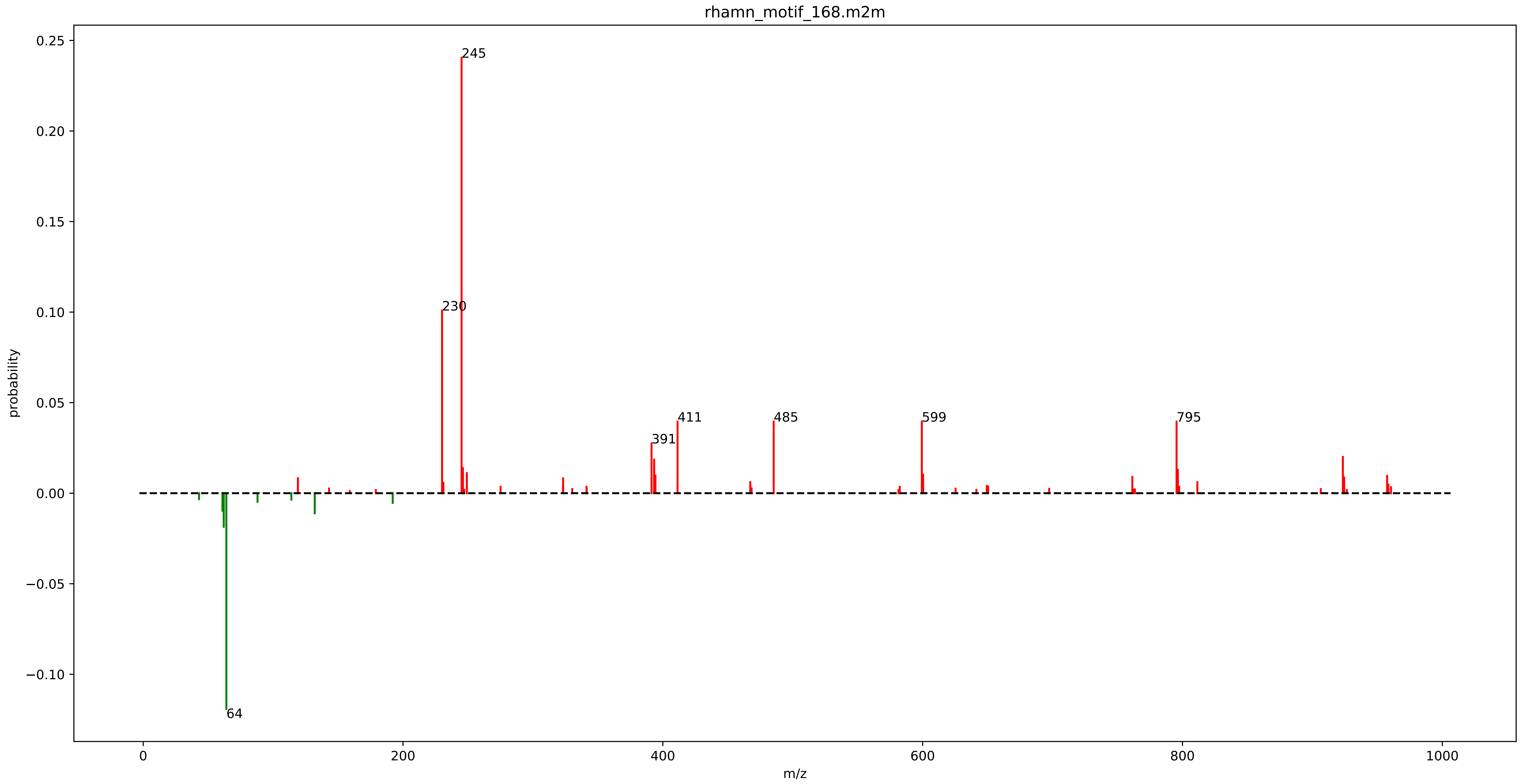
rhamn_motif_168.m2m
probability
m/z
0.25
0.20
0.15
0.10
0.05
0.00
−0.05
−0.10
0
200
400
600
800
1000
245
230
391
411
485
599
795
64

rhamn_motif_168.m2m

fragment_245.0825	: 0.24
loss_64.0175	: 0.12
fragment_230.0575	: 0.10
fragment_485.3225	: 0.04
fragment_411.3275	: 0.04
fragment_599.3625	: 0.04
fragment_795.4475	: 0.04
fragment_391.2975	: 0.03
fragment_923.4625	: 0.02
fragment_393.3025	: 0.02
loss_62.0125	: 0.02
fragment_393.3225	: 0.01
loss_61.9925	: 0.01
fragment_246.0875	: 0.01
fragment_796.4525	: 0.01
loss_132.0325	: 0.01
fragment_249.1425	: 0.01
fragment_246.0825	: 0.01
fragment_600.3675	: 0.01
fragment_394.3125	: 0.01
loss_61.0025	: 0.01
fragment_957.5075	: 0.01
fragment_761.4125	: 0.01
fragment_924.4725	: 0.01
fragment_119.0925	: 0.01
loss_132.0775	: 0.01
fragment_323.2425	: 0.01
fragment_467.3025	: 0.01
fragment_811.4475	: 0.01
fragment_231.0575	: 0.01
fragment_923.4875	: 0.01
loss_192.0675	: 0.01
fragment_231.0625	: 0.01
fragment_467.3175	: 0.01

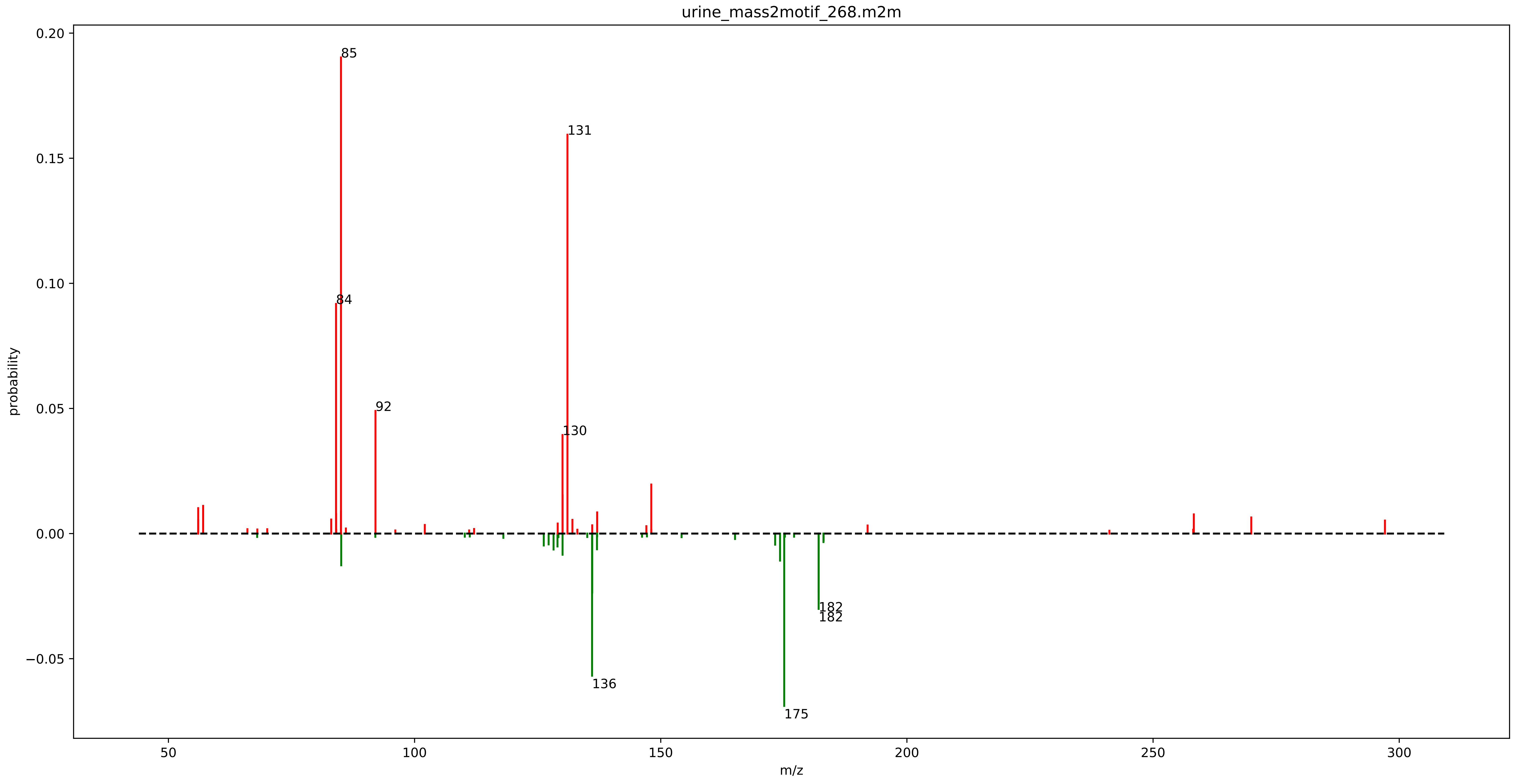

urine_mass2motif_268.m2m
probability
m/z
0.20
0.15
0.10
0.05
0.00
−0.05
50
100
150
200
250
300
85
84
92
131
130
136
175
182
182

urine_mass2motif_268.m2m

fragment_85.0475	: 0.19
fragment_131.0525	: 0.16
fragment_84.0425	: 0.09
loss_175.0775	: 0.07
loss_136.0525	: 0.06
fragment_92.0575	: 0.05
fragment_130.0475	: 0.04
loss_182.0675	: 0.03
loss_182.0775	: 0.03
loss_136.0775	: 0.02
loss_182.0575	: 0.02
fragment_148.0775	: 0.02
fragment_130.0675	: 0.02
loss_85.0975	: 0.01
fragment_57.0525	: 0.01
loss_174.2325	: 0.01
fragment_56.0475	: 0.01
fragment_85.0425	: 0.01
fragment_137.0825	: 0.01
loss_130.0575	: 0.01
fragment_84.0625	: 0.01
fragment_258.2775	: 0.01
fragment_269.9475	: 0.01
loss_128.2275	: 0.01
loss_137.0475	: 0.01
fragment_83.0625	: 0.01
fragment_132.0575	: 0.01
fragment_56.0575	: 0.01
fragment_297.0975	: 0.01
loss_129.0225	: 0.01

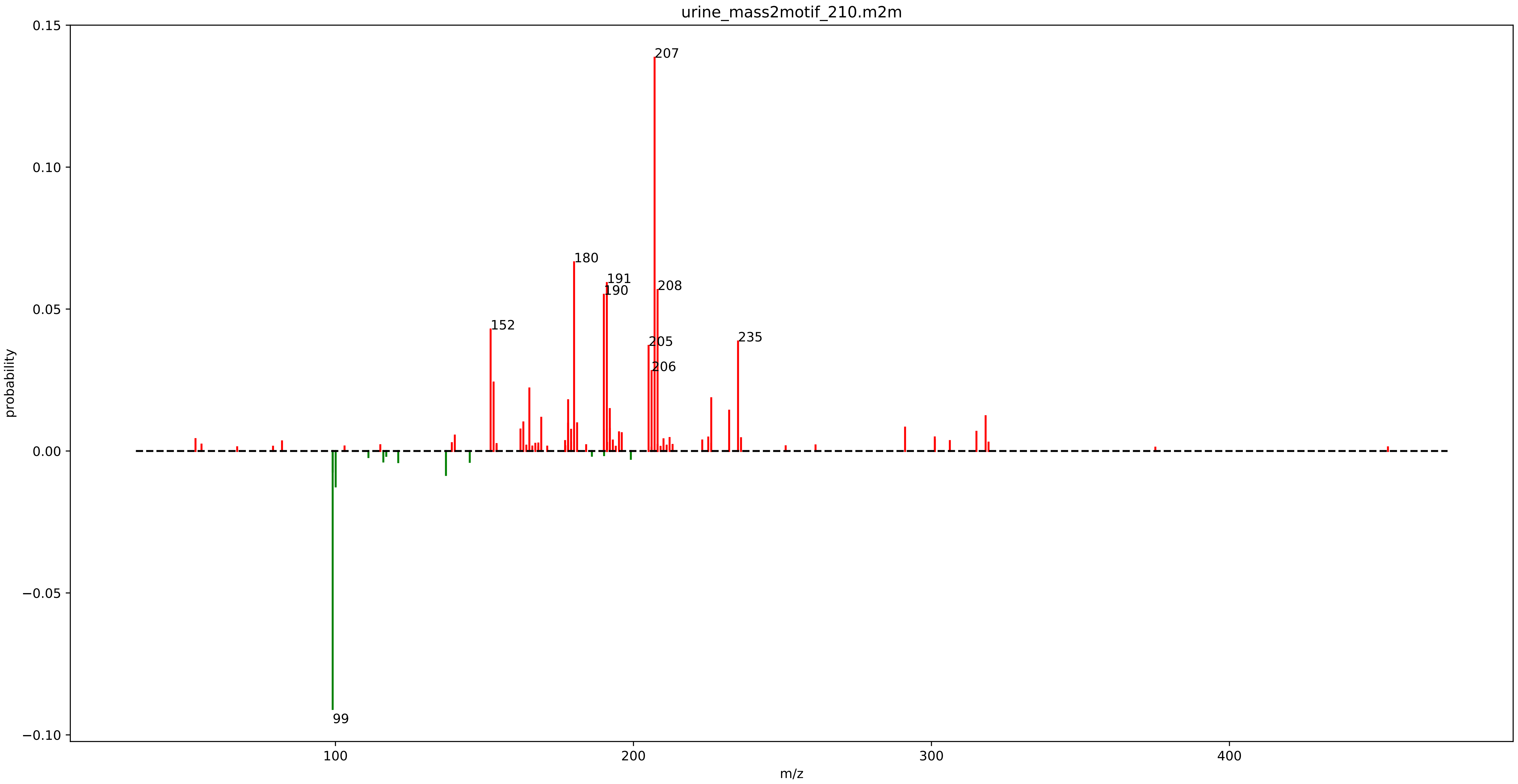

urine_mass2motif_210.m2m
probability
m/z
0.15
0.10
0.05
0.00
−0.05
−0.10
100
200
300
400
207
180
191
190
208
152
205
206
235
99

urine_mass2motif_210.m2m

fragment_207.0925	: 0.14
loss_99.0675	: 0.09
fragment_180.0775	: 0.07
fragment_191.0725	: 0.06
fragment_208.0975	: 0.06
fragment_190.0625	: 0.06
fragment_152.0675	: 0.04
fragment_235.0975	: 0.04
fragment_205.0775	: 0.04
fragment_206.0825	: 0.03
fragment_153.0675	: 0.02
fragment_165.0675	: 0.02
fragment_226.1075	: 0.02
fragment_178.0775	: 0.02
fragment_192.0675	: 0.01
fragment_232.1125	: 0.01
loss_100.0725	: 0.01
fragment_318.1775	: 0.01
fragment_169.0625	: 0.01
fragment_163.0525	: 0.01
fragment_181.0925	: 0.01
fragment_178.0625	: 0.01
loss_137.0825	: 0.01
fragment_291.1475	: 0.01
fragment_192.0775	: 0.01
fragment_162.0925	: 0.01
fragment_179.0725	: 0.01
loss_99.0775	: 0.01
fragment_190.0875	: 0.01
fragment_315.0975	: 0.01
fragment_195.1475	: 0.01
fragment_196.0775	: 0.01
fragment_140.0525	: 0.01

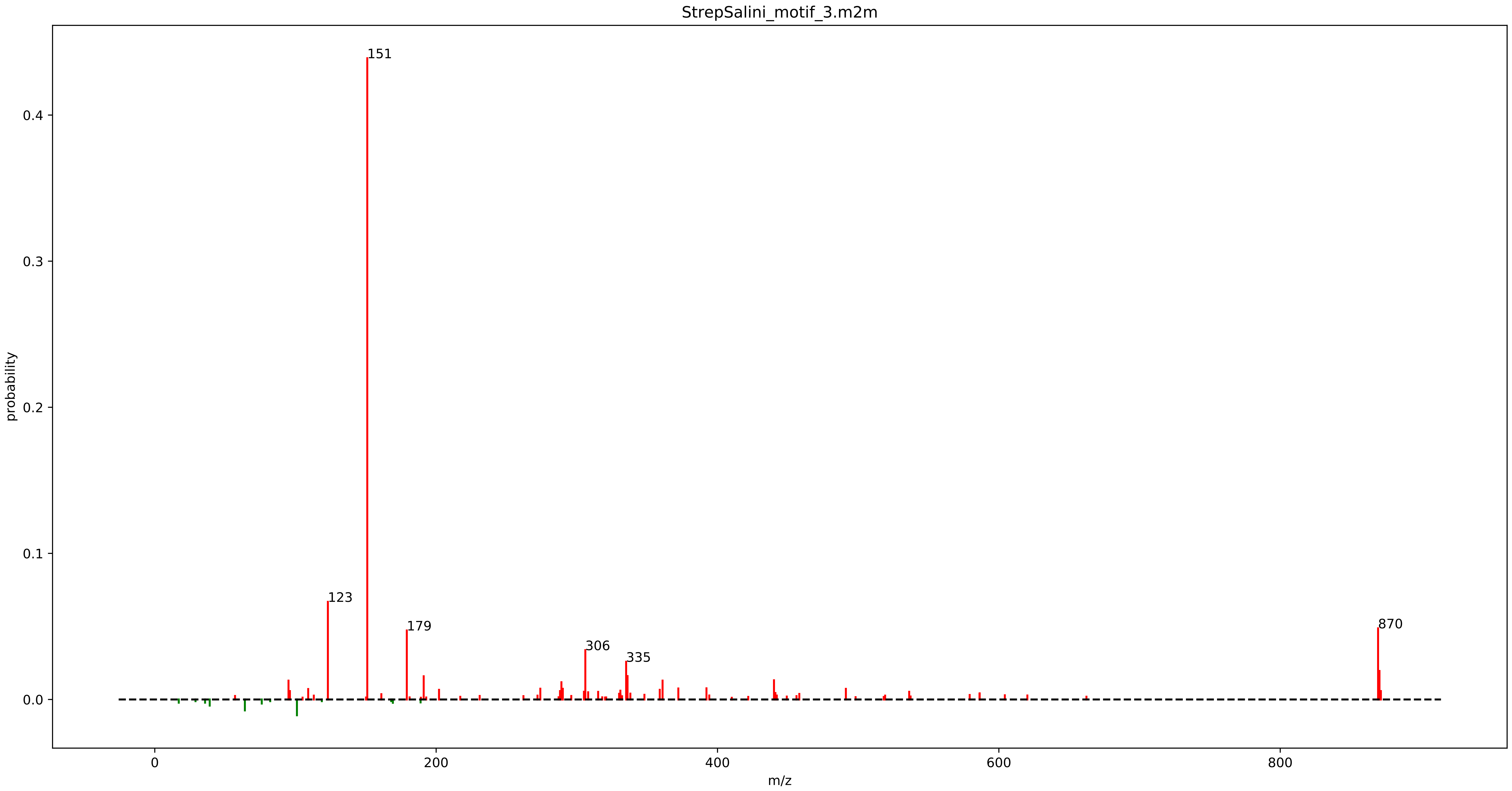

StrepSalini_motif_3.m2m
151
123
179
306
335
870
probability
0.0
0.1
0.2
0.3
0.4
m/z
0
200
400
600
800

StrepSalini_motif_3.m2m

fragment_151.0500	: 0.44
fragment_123.0500	: 0.07
fragment_869.5500	: 0.05
fragment_179.1500	: 0.05
fragment_306.0500	: 0.03
fragment_335.0500	: 0.03
fragment_870.5500	: 0.02
fragment_336.0500	: 0.02
fragment_191.1500	: 0.02
fragment_440.1500	: 0.01
fragment_360.9500	: 0.01
fragment_95.0500	: 0.01
fragment_289.0500	: 0.01
loss_101.0500	: 0.01
fragment_392.1500	: 0.01
fragment_372.1500	: 0.01
loss_64.0475	: 0.01
fragment_274.0500	: 0.01
fragment_290.0500	: 0.01
fragment_491.2500	: 0.01
fragment_109.0525	: 0.01
fragment_358.9500	: 0.01
fragment_202.0500	: 0.01
fragment_330.9500	: 0.01
fragment_871.5500	: 0.01
fragment_96.0500	: 0.01
fragment_288.0500	: 0.01
fragment_305.0500	: 0.01
fragment_536.2500	: 0.01
fragment_315.1500	: 0.01

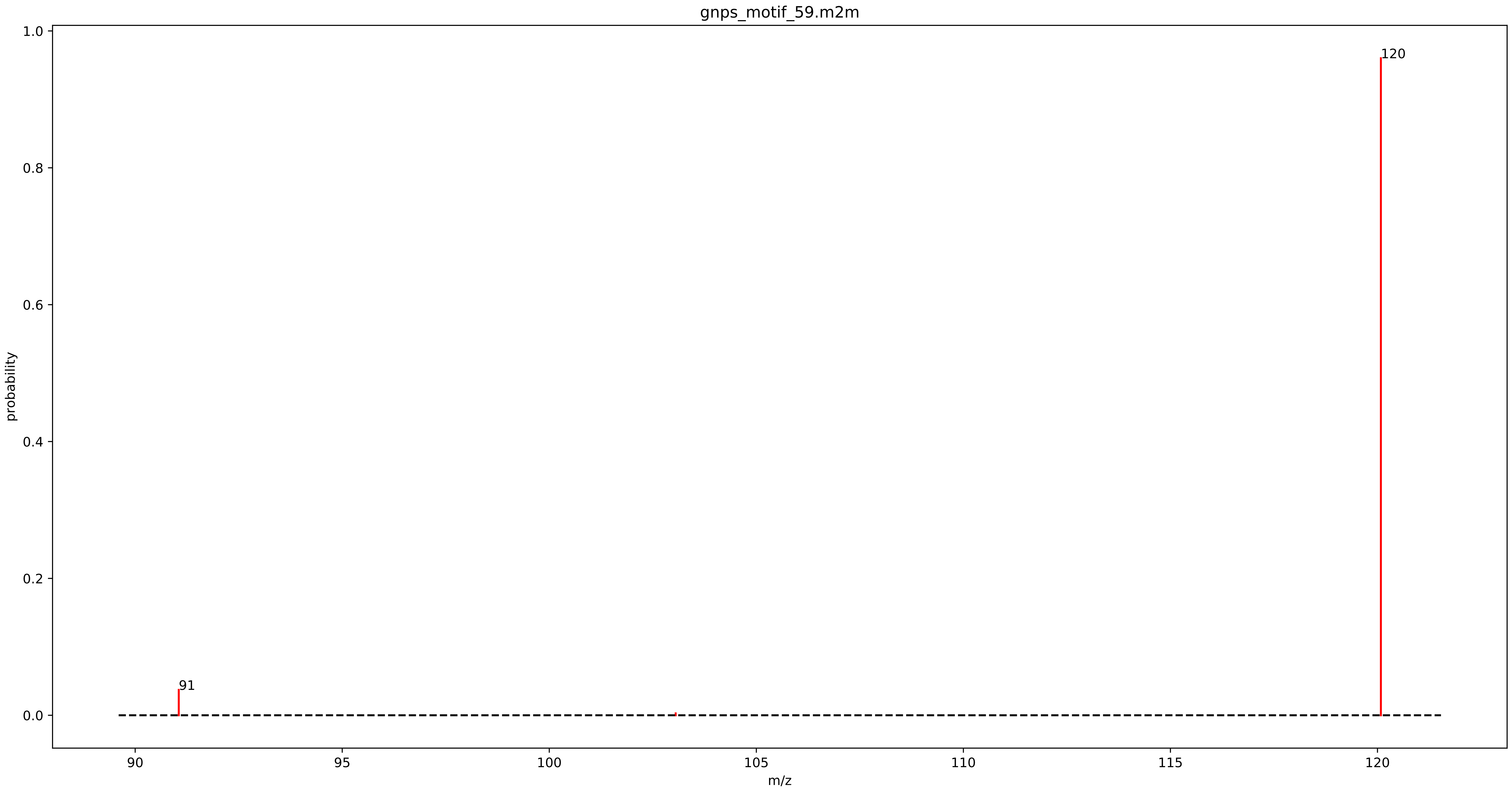

gnps_motif_59.m2m
probability
m/z
1.0
0.8
0.6
0.4
0.2
0.0
90
95
100
105
110
115
120
91
120

gnps_motif_59.m2m

fragment_120.0825	: 0.96
fragment_91.0525	: 0.04

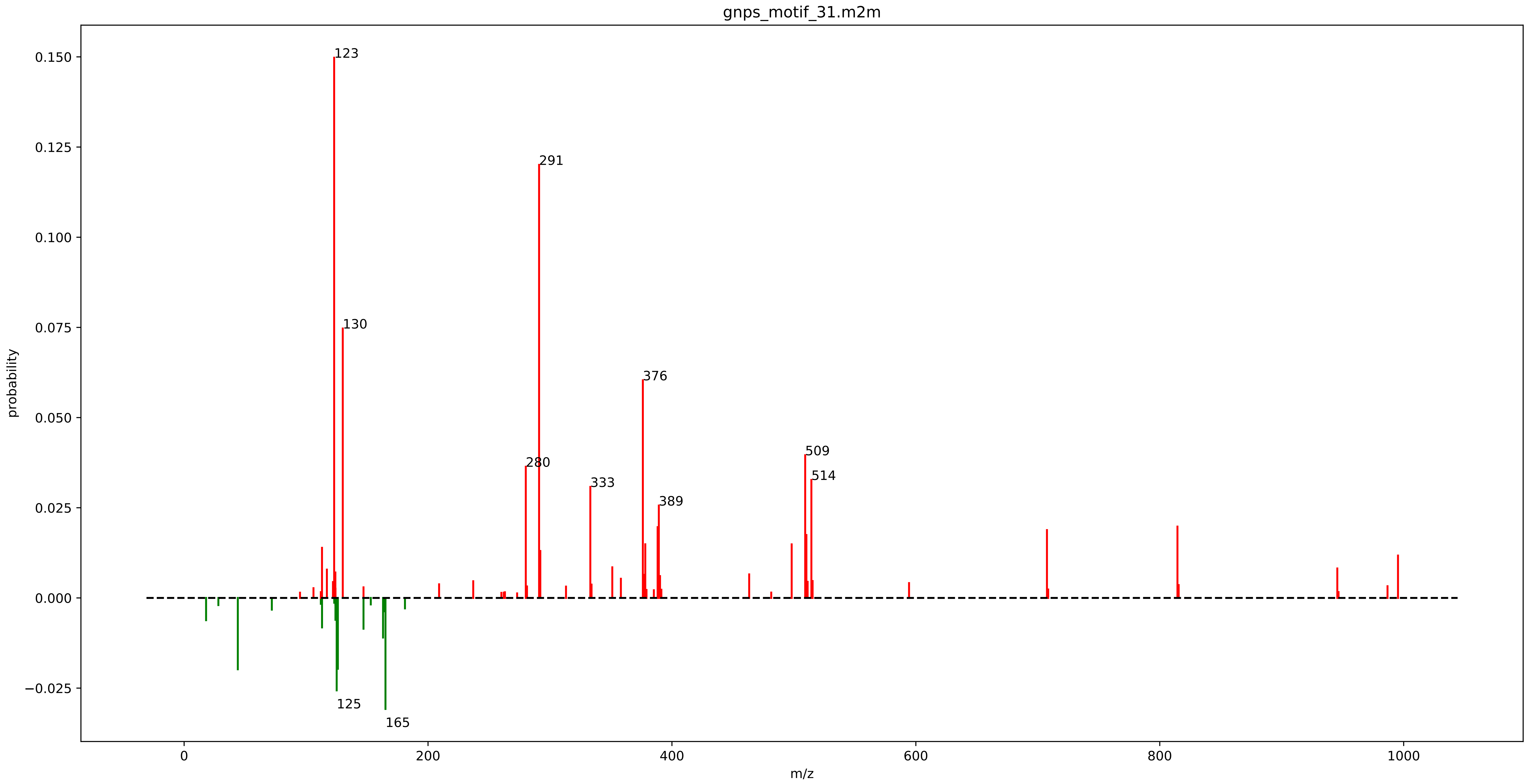

gnps_motif_31.m2m
probability
m/z
0.150
0.125
0.100
0.075
0.050
0.025
0.000
−0.025
0
200
400
600
800
1000
123
291
130
376
280
333
389
509
514
125
165

gnps_motif_31.m2m

fragment_123.0225	: 0.15
fragment_291.0625	: 0.12
fragment_130.0875	: 0.07
fragment_376.1475	: 0.06
fragment_509.2575	: 0.04
fragment_280.1525	: 0.04
fragment_514.3375	: 0.03
fragment_333.0775	: 0.03
loss_165.1025	: 0.03
loss_125.0975	: 0.03
fragment_389.2375	: 0.03
fragment_814.5025	: 0.02
loss_44.0275	: 0.02
loss_126.0775	: 0.02
fragment_388.2575	: 0.02
fragment_707.5025	: 0.02
fragment_510.2575	: 0.02
fragment_378.1425	: 0.01
fragment_498.1775	: 0.01
fragment_113.0575	: 0.01
fragment_292.0725	: 0.01
fragment_995.3475	: 0.01
loss_163.1125	: 0.01
loss_147.0875	: 0.01
fragment_351.0875	: 0.01
fragment_945.5825	: 0.01
loss_113.0875	: 0.01
fragment_117.0675	: 0.01
fragment_124.0275	: 0.01
fragment_463.3325	: 0.01
fragment_377.1525	: 0.01
loss_18.0075	: 0.01
loss_124.0875	: 0.01
fragment_390.2425	: 0.01
fragment_358.1375	: 0.01

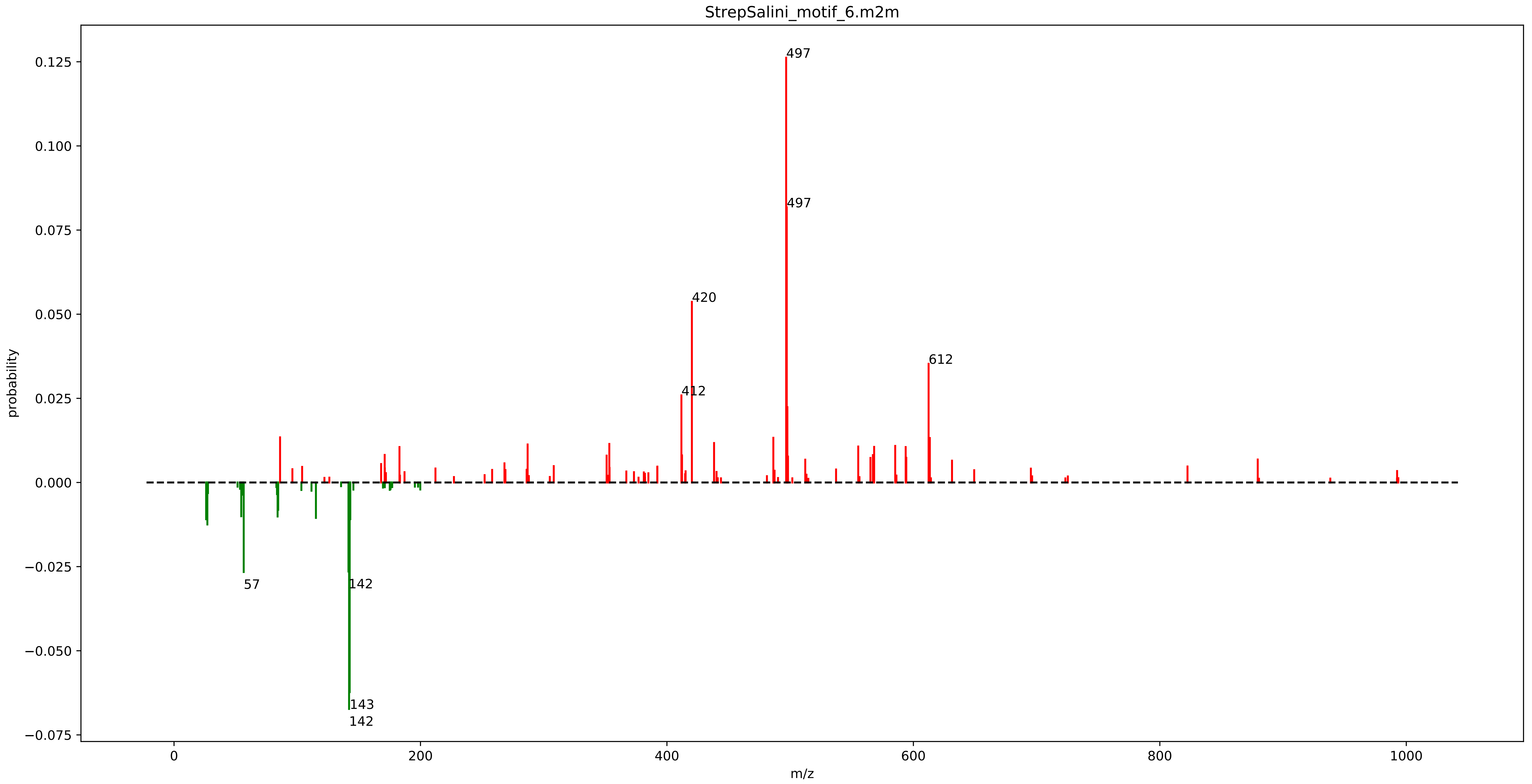

StrepSalini_motif_6.m2m
probability
m/z
0.125
0.100
0.075
0.050
0.025
0.000
−0.025
−0.050
−0.075
0
200
400
600
800
1000
497
497
420
412
612
57
142
143
142

StrepSalini_motif_6.m2m

fragment_496.7500	: 0.13	fragment_555.2500	: 0.01
fragment_497.2500	: 0.08	fragment_568.1500	: 0.01
loss_142.0500	: 0.07	fragment_182.9500	: 0.01
loss_142.5500	: 0.06	loss_115.1500	: 0.01
fragment_420.2500	: 0.05	fragment_593.7500	: 0.01
fragment_612.3500	: 0.04	loss_84.0500	: 0.01
loss_56.5500	: 0.03	loss_54.5500	: 0.01
loss_141.5500	: 0.03	fragment_170.9500	: 0.01
fragment_411.7500	: 0.03	fragment_567.1500	: 0.01
fragment_497.7500	: 0.02	loss_84.5500	: 0.01
fragment_86.0500	: 0.01	fragment_412.2500	: 0.01
fragment_486.3500	: 0.01	fragment_351.0500	: 0.01
fragment_613.3500	: 0.01	fragment_498.2500	: 0.01
loss_27.0500	: 0.01	fragment_594.2500	: 0.01
fragment_438.2500	: 0.01	fragment_565.1500	: 0.01
fragment_353.2500	: 0.01	fragment_879.4500	: 0.01
fragment_286.9500	: 0.01	fragment_512.2500	: 0.01
loss_26.0500	: 0.01	fragment_631.3500	: 0.01
loss_143.0500	: 0.01	fragment_268.1525	: 0.01
fragment_585.2500	: 0.01	fragment_168.0500	: 0.01

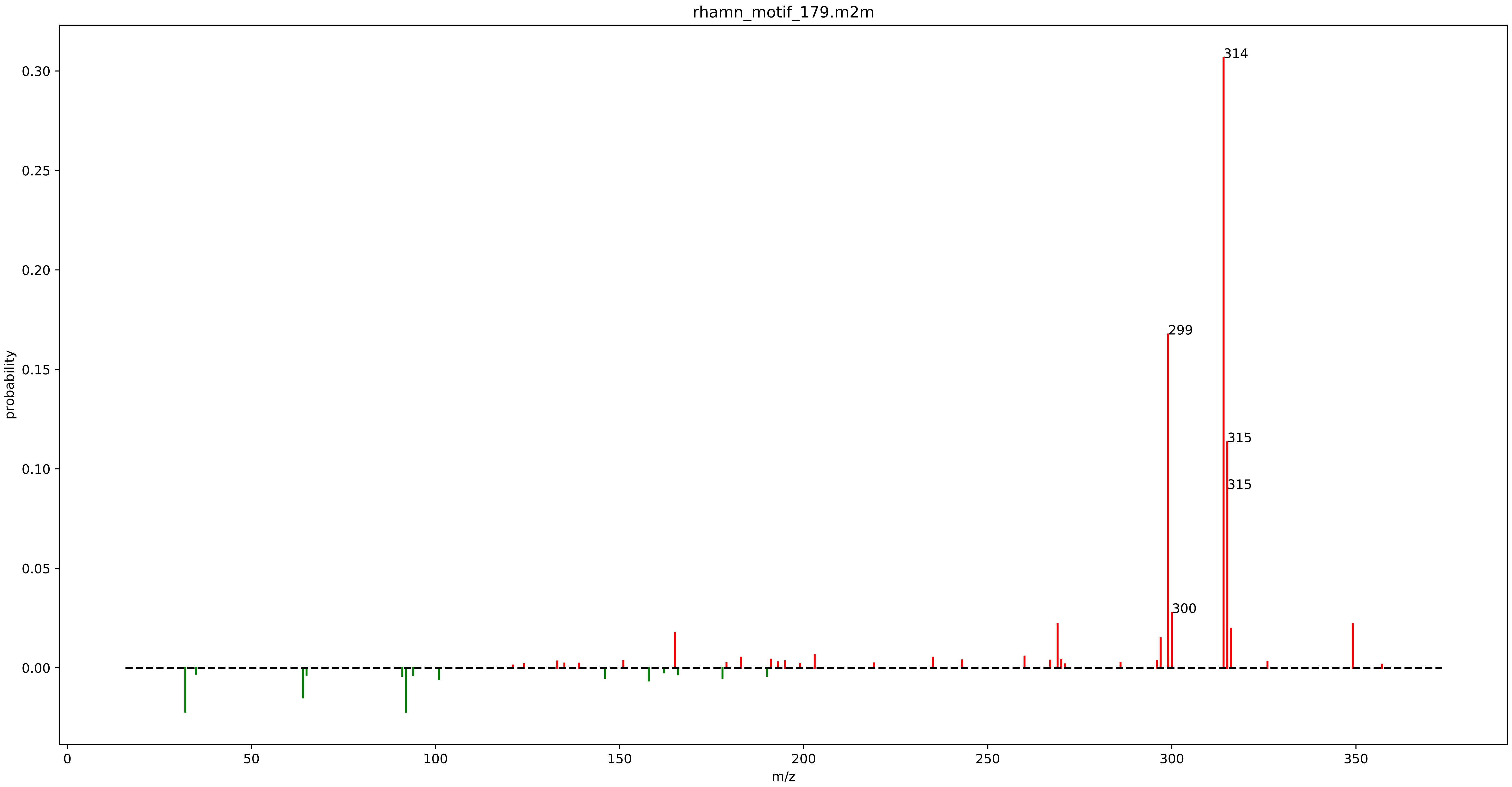

rhamn_motif_179.m2m
probability
m/z
0.30
0.25
0.20
0.15
0.10
0.05
0.00
0
50
100
150
200
250
300
350
314
299
315
315
300

rhamn_motif_179.m2m

fragment_314.0425	: 0.31
fragment_299.0175	: 0.17
fragment_315.0475	: 0.11
fragment_315.0525	: 0.09
fragment_300.0225	: 0.03
loss_91.9625	: 0.02
fragment_268.9625	: 0.02
fragment_349.1275	: 0.02
loss_32.0275	: 0.02
fragment_316.0525	: 0.02
fragment_165.0175	: 0.02
loss_63.9725	: 0.01
fragment_296.9525	: 0.01
loss_157.9325	: 0.01
fragment_202.9925	: 0.01
fragment_259.9825	: 0.01
loss_100.9425	: 0.01
fragment_299.0275	: 0.01
fragment_182.9825	: 0.01
loss_177.9425	: 0.01
fragment_235.0625	: 0.01
loss_146.0925	: 0.01

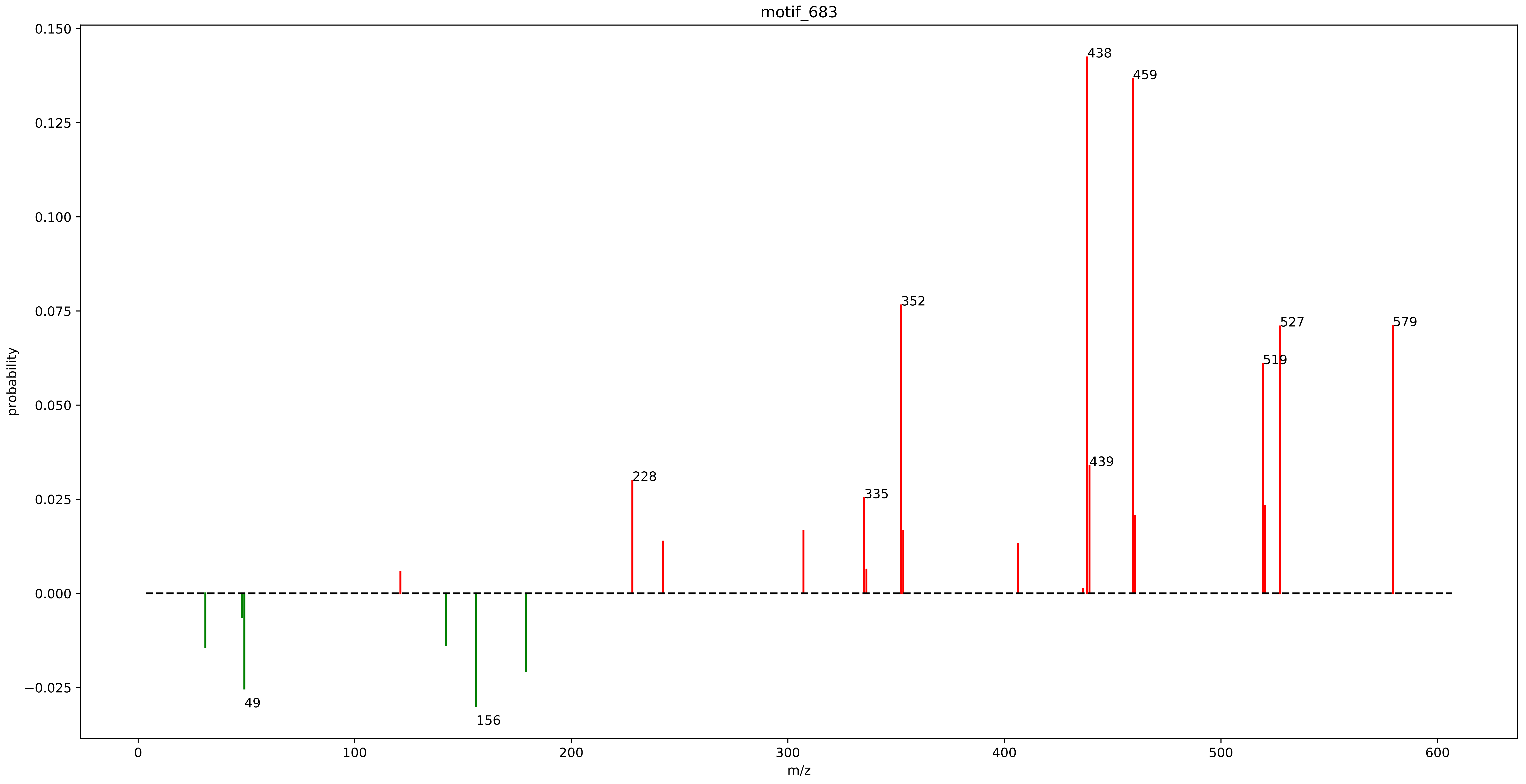
motif_683
probability
m/z
0.150
0.125
0.100
0.075
0.050
0.025
0.000
−0.025
0
100
200
300
400
500
600
438
459
352
527
579
519
439
228
335
49
156

motif_683

fragment_438.2825	: 0.14
fragment_459.3225	: 0.14
fragment_352.3225	: 0.08
fragment_579.3675	: 0.07
fragment_527.2925	: 0.07
fragment_519.3425	: 0.06
fragment_439.2875	: 0.03
fragment_228.1975	: 0.03
loss_156.1475	: 0.03
loss_49.0525	: 0.03
fragment_335.2925	: 0.03
fragment_520.3475	: 0.02
fragment_460.3275	: 0.02
loss_179.0575	: 0.02
fragment_353.3225	: 0.02
fragment_307.2225	: 0.02
loss_31.0225	: 0.01
loss_142.1325	: 0.01
fragment_242.2125	: 0.01
fragment_406.2575	: 0.01
fragment_336.2975	: 0.01
loss_48.0475	: 0.01
fragment_121.0975	: 0.01

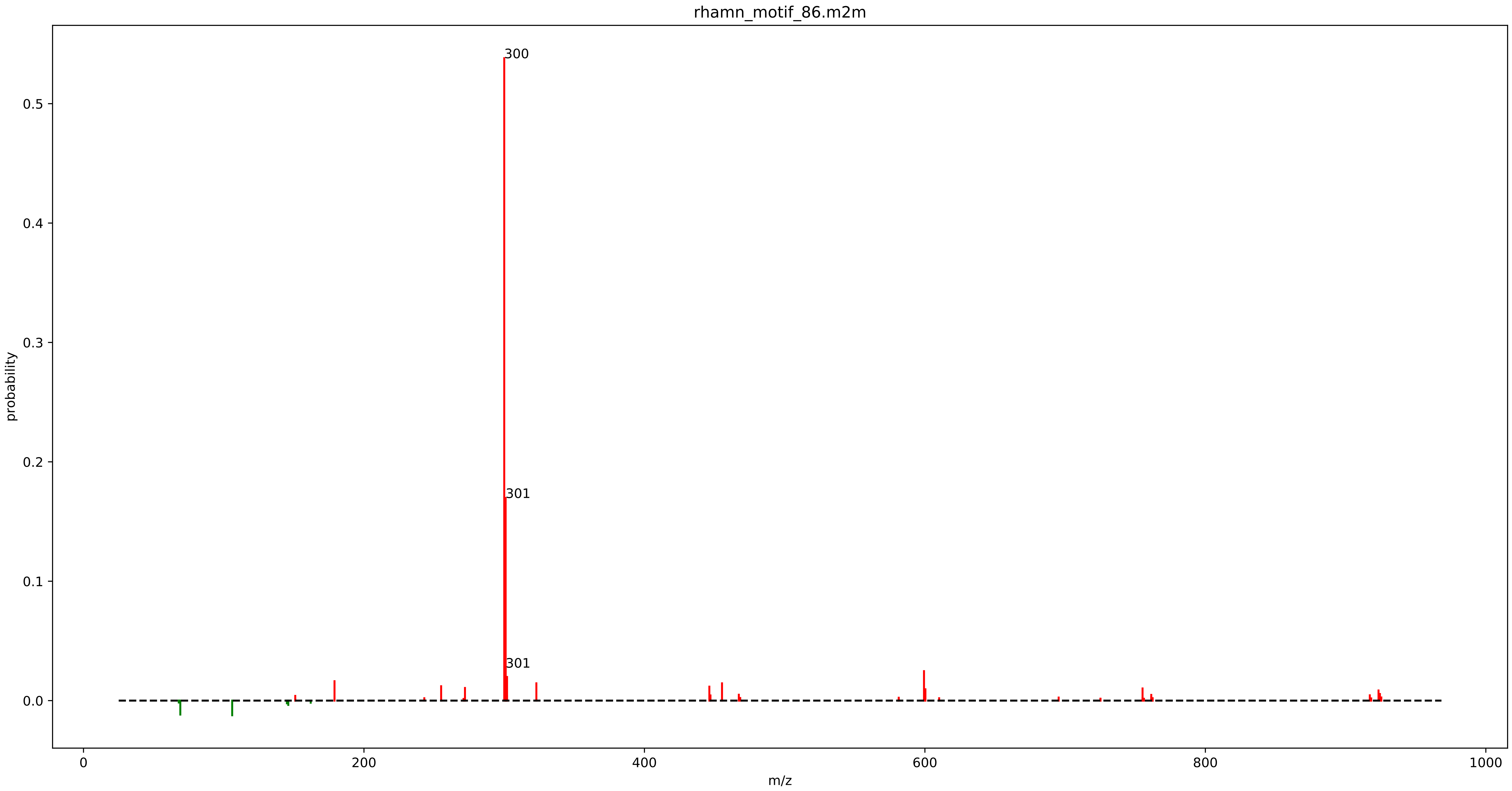

rhamn_motif_86.m2m
300
301
301
probability
0.0
0.1
0.2
0.3
0.4
0.5
m/z
0
200
400
600
800
1000

rhamn_motif_86.m2m

fragment_300.0275	: 0.54
fragment_301.0325	: 0.17
fragment_301.0375	: 0.03
fragment_599.3575	: 0.02
fragment_302.0375	: 0.02
fragment_178.9975	: 0.02
fragment_322.9425	: 0.01
fragment_455.3225	: 0.01
loss_106.0275	: 0.01
fragment_255.0275	: 0.01
fragment_446.2925	: 0.01
loss_69.0425	: 0.01
fragment_272.0325	: 0.01
fragment_755.2025	: 0.01
fragment_600.3625	: 0.01
fragment_923.4675	: 0.01
fragment_301.0275	: 0.01
fragment_924.4725	: 0.01

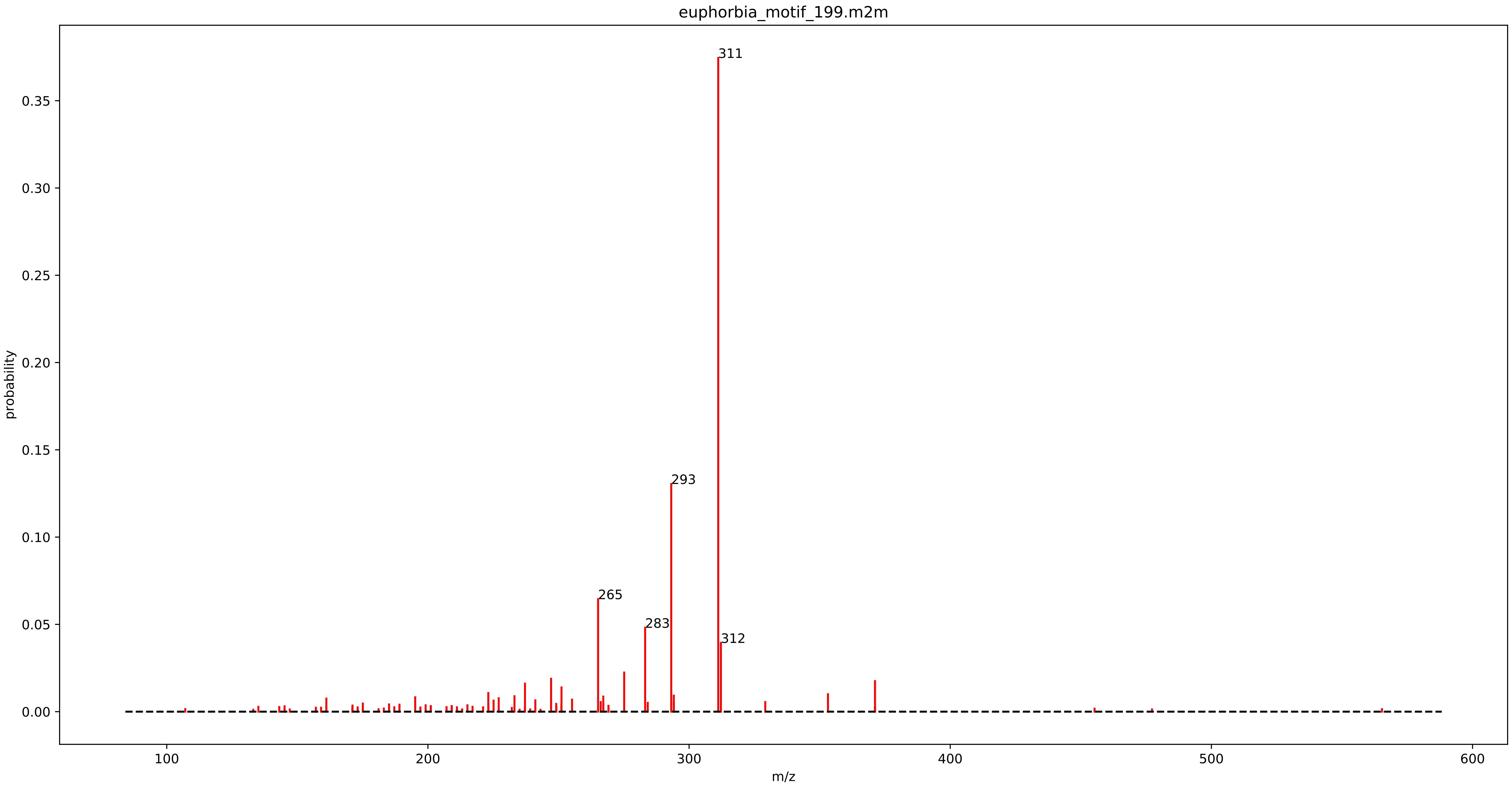
euphorbia_motif_199.m2m
probability
m/z
311
293
265
283
312
0.00
0.05
0.10
0.15
0.20
0.25
0.30
0.35
100
200
300
400
500
600

euphorbia_motif_199.m2m

fragment_311.1625	: 0.37
fragment_293.1525	: 0.13
fragment_265.1575	: 0.06
fragment_283.1675	: 0.05
fragment_312.1675	: 0.04
fragment_275.1425	: 0.02
fragment_247.1475	: 0.02
fragment_371.1825	: 0.02
fragment_237.1625	: 0.02
fragment_251.1425	: 0.01
fragment_223.1125	: 0.01
fragment_353.1725	: 0.01
fragment_294.1575	: 0.01
fragment_233.1325	: 0.01
fragment_267.1375	: 0.01
fragment_195.1175	: 0.01
fragment_227.1075	: 0.01
fragment_161.0975	: 0.01
fragment_255.1725	: 0.01
fragment_241.1225	: 0.01
fragment_225.1275	: 0.01
fragment_329.1725	: 0.01
fragment_266.1625	: 0.01
fragment_294.1525	: 0.01
fragment_284.1725	: 0.01

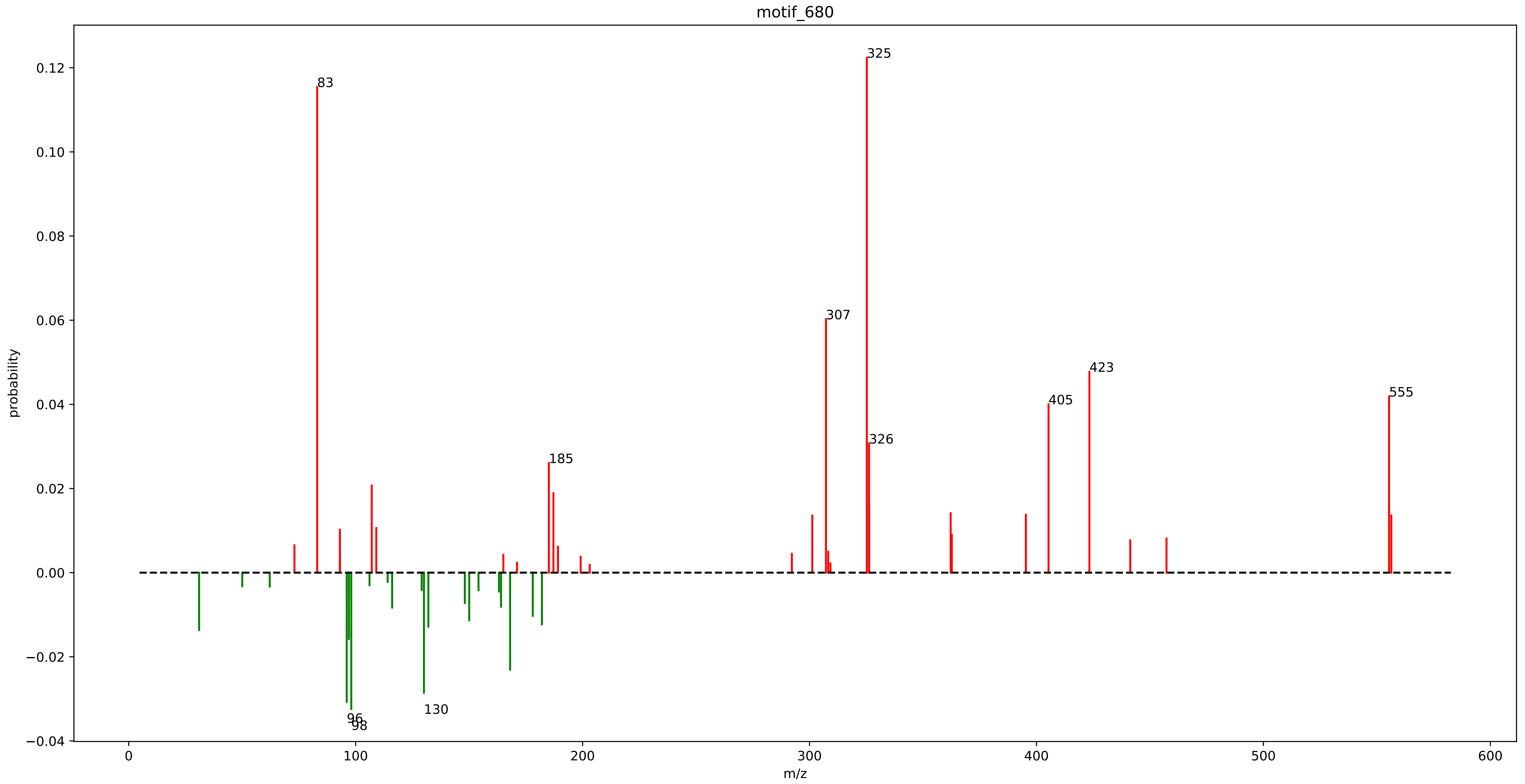

motif_680
probability
m/z
0.12
0.10
0.08
0.06
0.04
0.02
0.00
−0.02
−0.04
0
100
200
300
400
500
600
83
325
307
423
405
555
326
185
96
98
130

motif_680

fragment_325.2525	: 0.12
fragment_83.0475	: 0.12
fragment_307.2425	: 0.06
fragment_423.3275	: 0.05
fragment_555.3675	: 0.04
fragment_405.3175	: 0.04
loss_98.0725	: 0.03
loss_96.0675	: 0.03
fragment_326.2175	: 0.03
loss_130.0975	: 0.03
fragment_185.1325	: 0.03
loss_168.0625	: 0.02
fragment_107.0825	: 0.02
fragment_187.1475	: 0.02
fragment_326.2575	: 0.02
loss_97.0325	: 0.02
fragment_362.1925	: 0.01
fragment_395.3325	: 0.01
fragment_556.3725	: 0.01
loss_31.0175	: 0.01
fragment_301.2175	: 0.01
loss_132.0375	: 0.01
loss_182.0775	: 0.01
loss_150.0475	: 0.01
fragment_109.0975	: 0.01
loss_178.0425	: 0.01
fragment_93.0675	: 0.01
fragment_362.6975	: 0.01
loss_116.0825	: 0.01
fragment_457.2975	: 0.01
loss_164.0675	: 0.01
fragment_441.3375	: 0.01
loss_148.1075	: 0.01
fragment_73.0275	: 0.01
fragment_189.1625	: 0.01
loss_97.0675	: 0.01

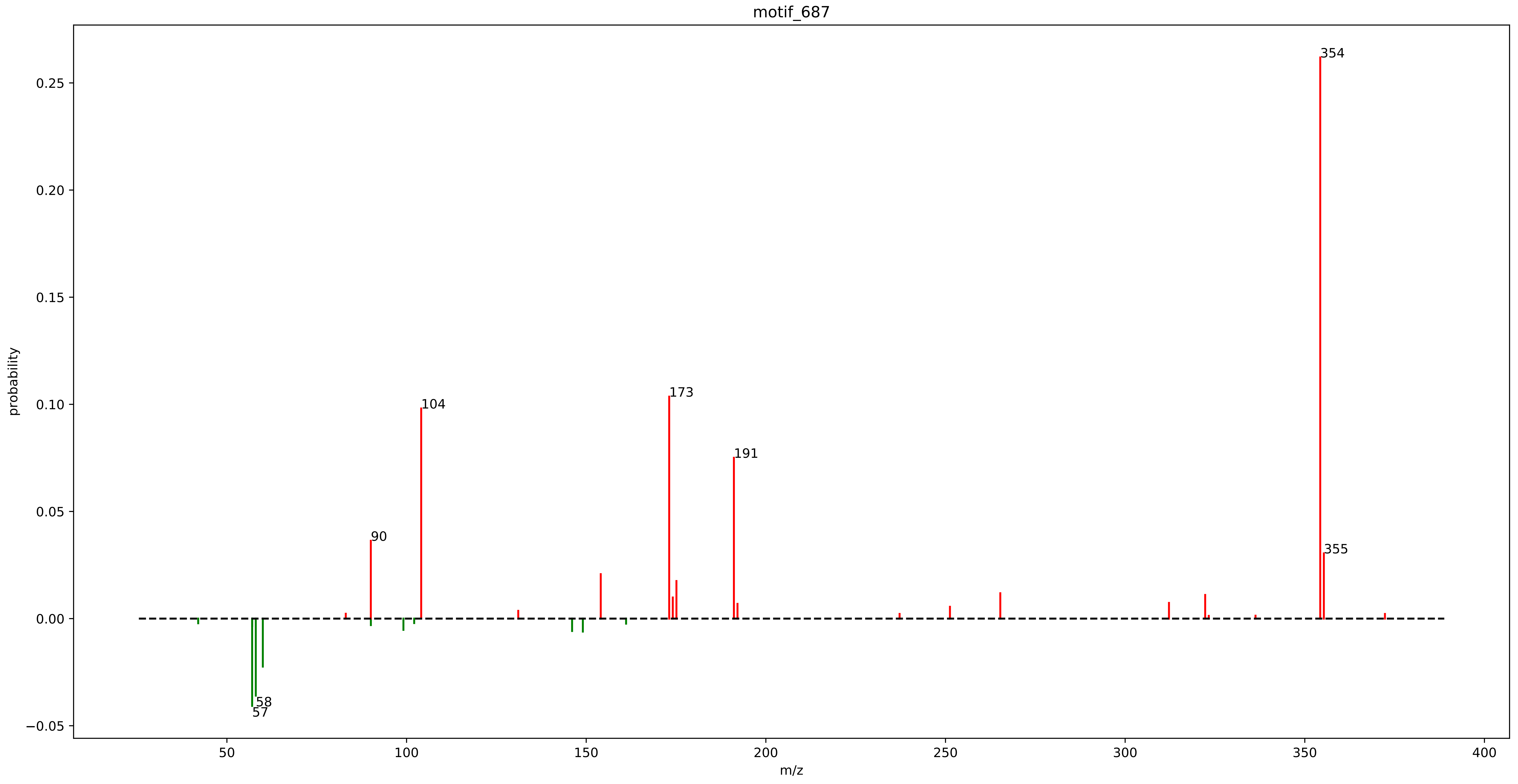

motif_687
probability
m/z
0.25
0.20
0.15
0.10
0.05
0.00
−0.05
50
100
150
200
250
300
350
400
354
173
104
191
90
355
58
57

motif_687

fragment_354.2975	: 0.26
fragment_173.0975	: 0.10
fragment_104.0725	: 0.10
fragment_191.1075	: 0.08
loss_57.0225	: 0.04
fragment_90.0525	: 0.04
loss_58.0375	: 0.04
fragment_355.3025	: 0.03
loss_59.9975	: 0.02
fragment_154.0475	: 0.02
fragment_175.1125	: 0.02
fragment_265.2525	: 0.01
fragment_322.2725	: 0.01
fragment_174.0975	: 0.01
fragment_312.1975	: 0.01
fragment_192.1075	: 0.01
loss_149.0675	: 0.01
loss_146.0725	: 0.01
fragment_251.2375	: 0.01
loss_99.1275	: 0.01

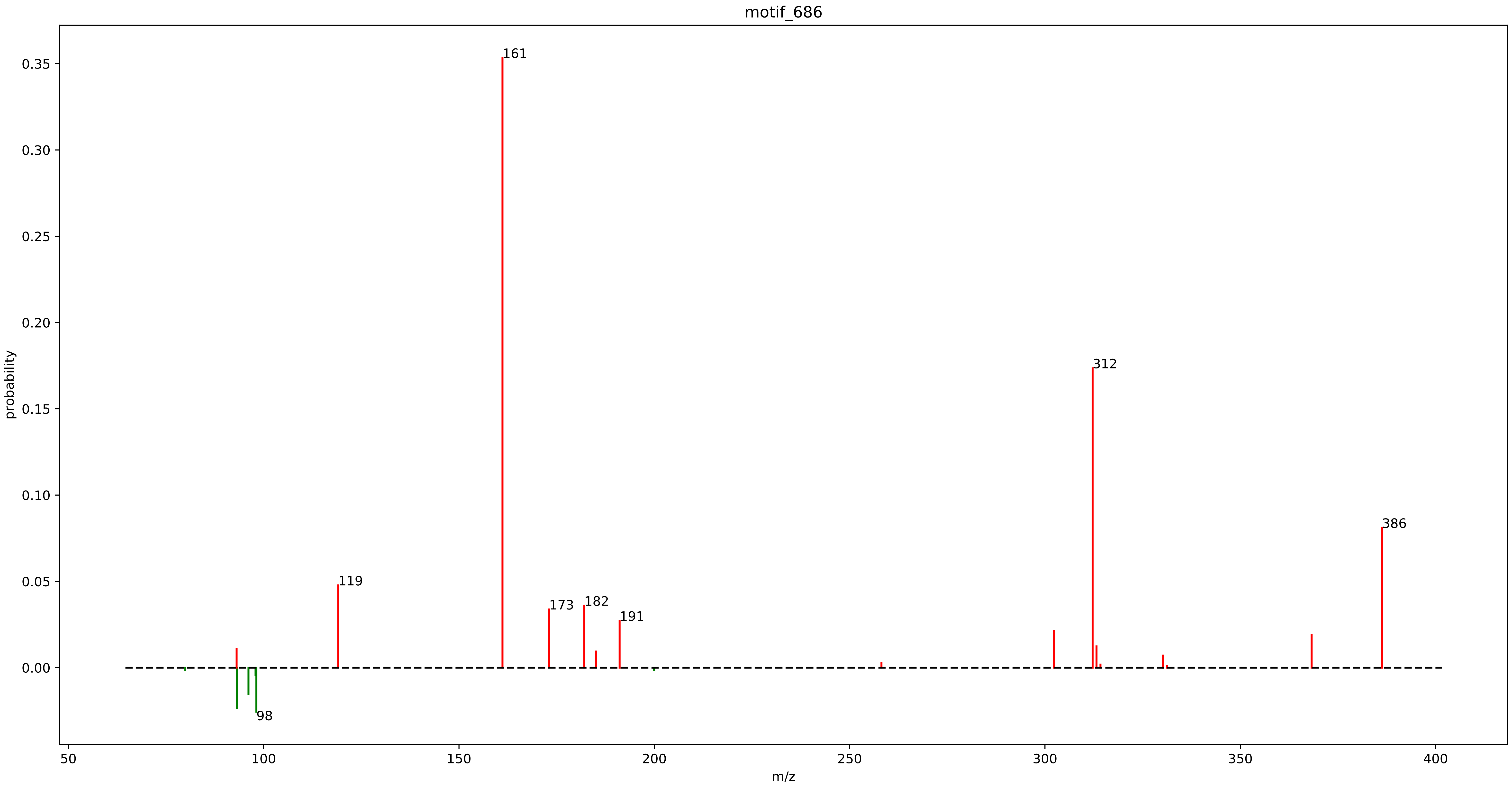

motif_686
0.35
0.30
0.25
0.20
0.15
0.10
0.05
0.00
probability
161
312
386
119
173
182
191
98
50
100
150
200
250
300
350
400
m/z

motif_686

fragment_161.1325	: 0.35
fragment_312.1975	: 0.17
fragment_386.2675	: 0.08
fragment_119.0825	: 0.05
fragment_182.0825	: 0.04
fragment_173.0975	: 0.03
fragment_191.1075	: 0.03
loss_98.1275	: 0.03
loss_93.1125	: 0.02
fragment_302.2475	: 0.02
fragment_368.2575	: 0.02
loss_96.1125	: 0.02
fragment_313.1975	: 0.01
fragment_93.0675	: 0.01
fragment_185.1325	: 0.01
fragment_330.2025	: 0.01

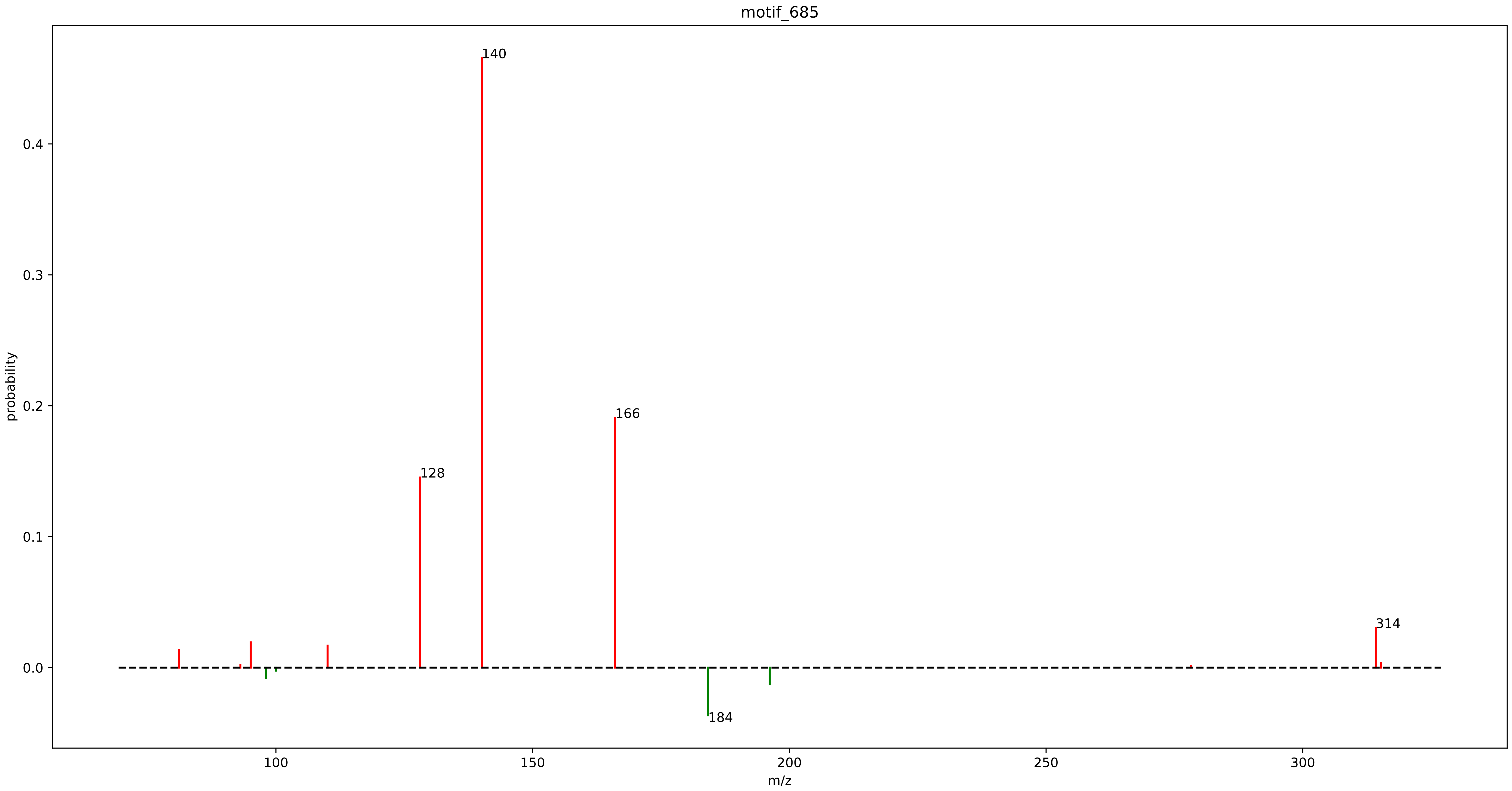

motif_685
140
166
128
314
184
0.4
0.3
0.2
0.1
0.0
probability
100
150
200
250
300
m/z

motif_685

fragment_140.0725	: 0.47
fragment_166.0875	: 0.19
fragment_128.0725	: 0.15
loss_184.1825	: 0.04
fragment_314.2125	: 0.03
fragment_95.0825	: 0.02
fragment_110.0575	: 0.02
fragment_81.0675	: 0.01
loss_196.1825	: 0.01
loss_98.0675	: 0.01

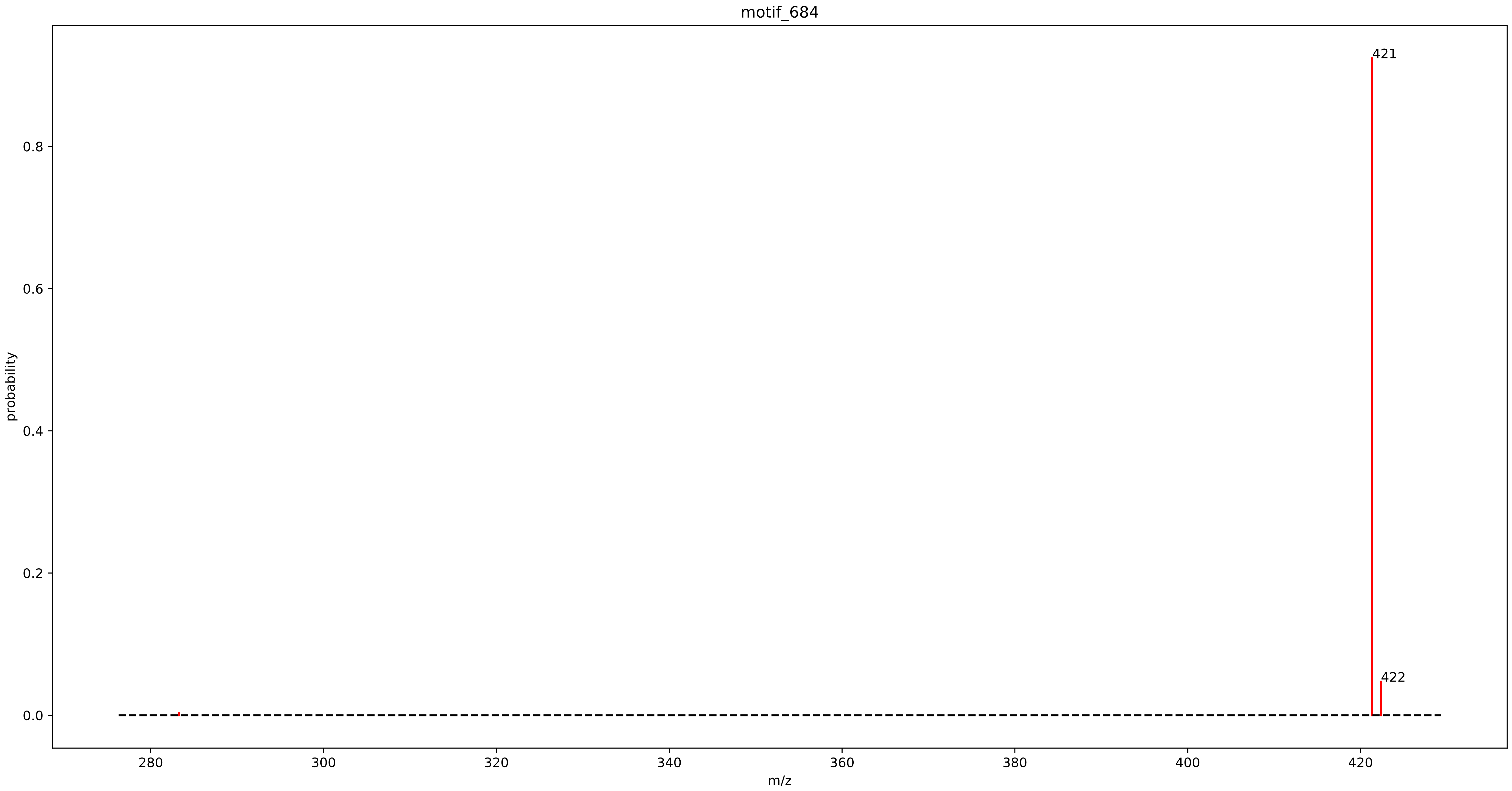
motif_684
probability
m/z
421
422
0.0
0.2
0.4
0.6
0.8
280
300
320
340
360
380
400
420

motif_684

fragment_421.3475	: 0.92
fragment_422.3525	: 0.05

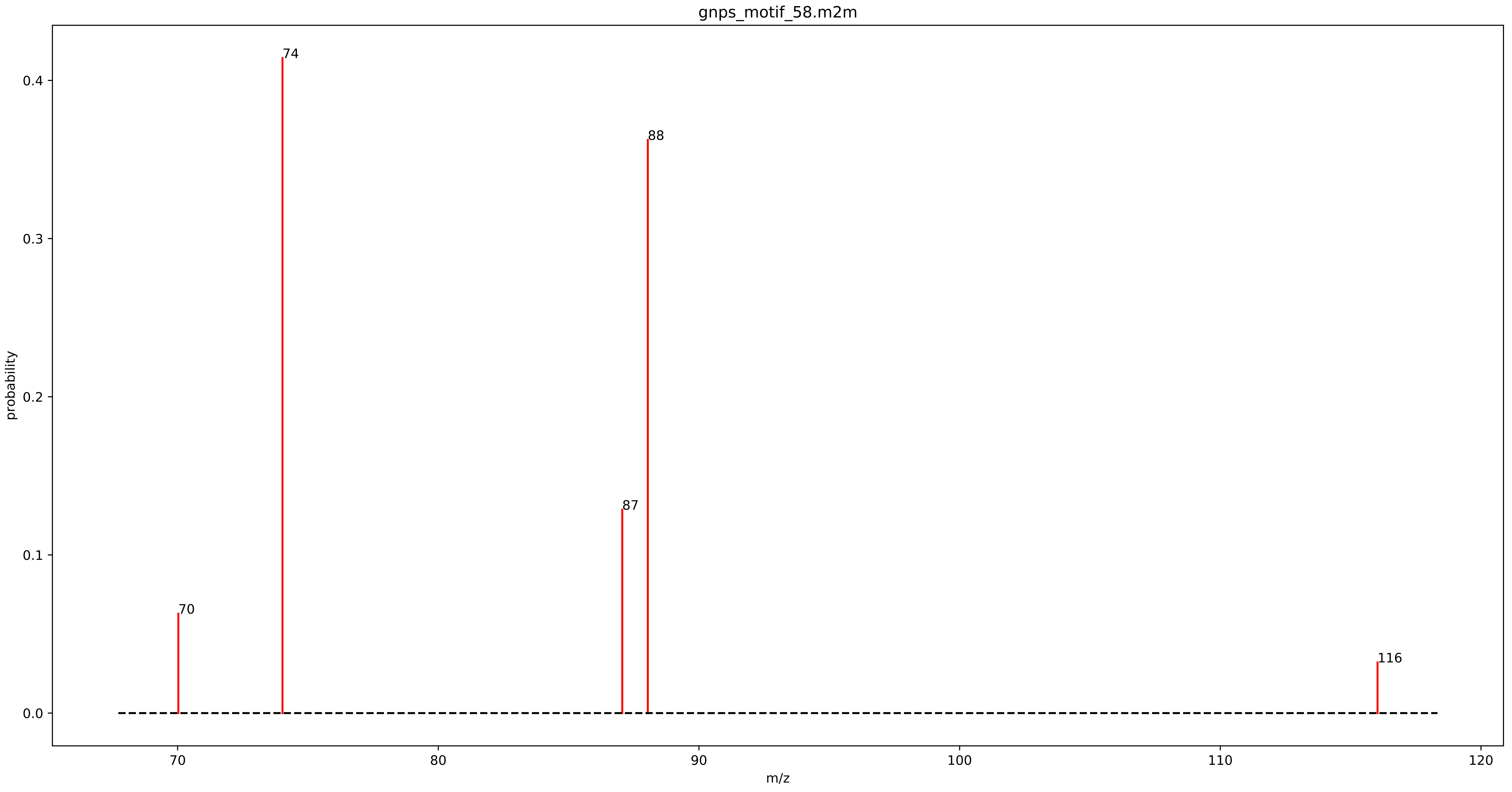

gnps_motif_58.m2m
74
88
87
70
116
0.4
0.3
0.2
0.1
0.0
probability
70
80
90
100
110
120
m/z

gnps_motif_58.m2m

fragment_74.0225	: 0.41
fragment_88.0375	: 0.36
fragment_87.0575	: 0.13
fragment_70.0275	: 0.06
fragment_116.0325	: 0.03

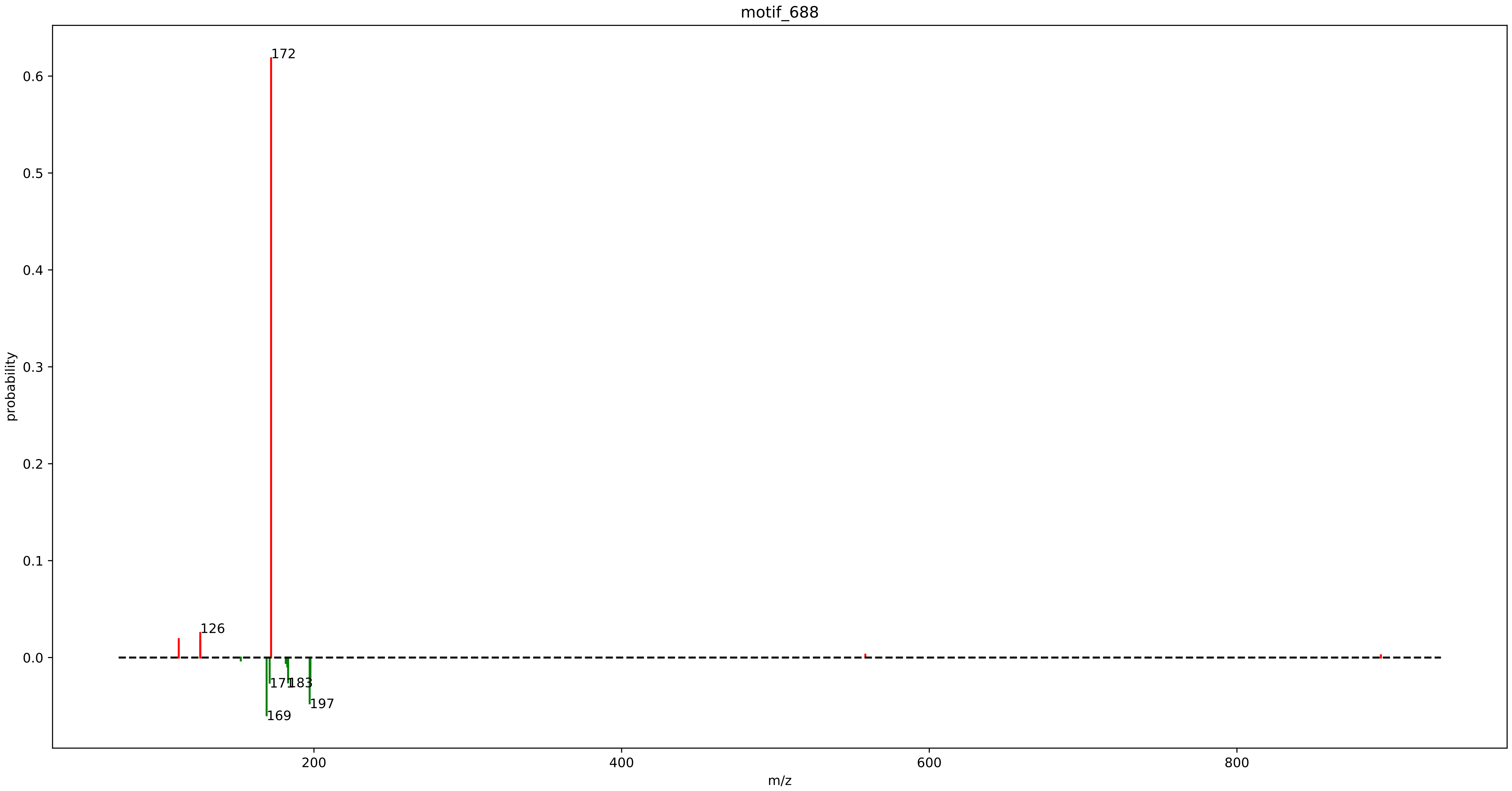

motif_688
172
126
171
183
197
169
0.6
0.5
0.4
0.3
0.2
0.1
0.0
probability
200
400
600
800
m/z

motif_688

fragment_172.1325	: 0.62
loss_169.2175	: 0.06
loss_197.2125	: 0.05
loss_171.1975	: 0.03
loss_183.1975	: 0.03
fragment_126.0875	: 0.03
loss_197.6925	: 0.02
fragment_112.1125	: 0.02
fragment_126.0975	: 0.02
loss_182.6875	: 0.01
loss_197.6825	: 0.01
loss_181.6775	: 0.01

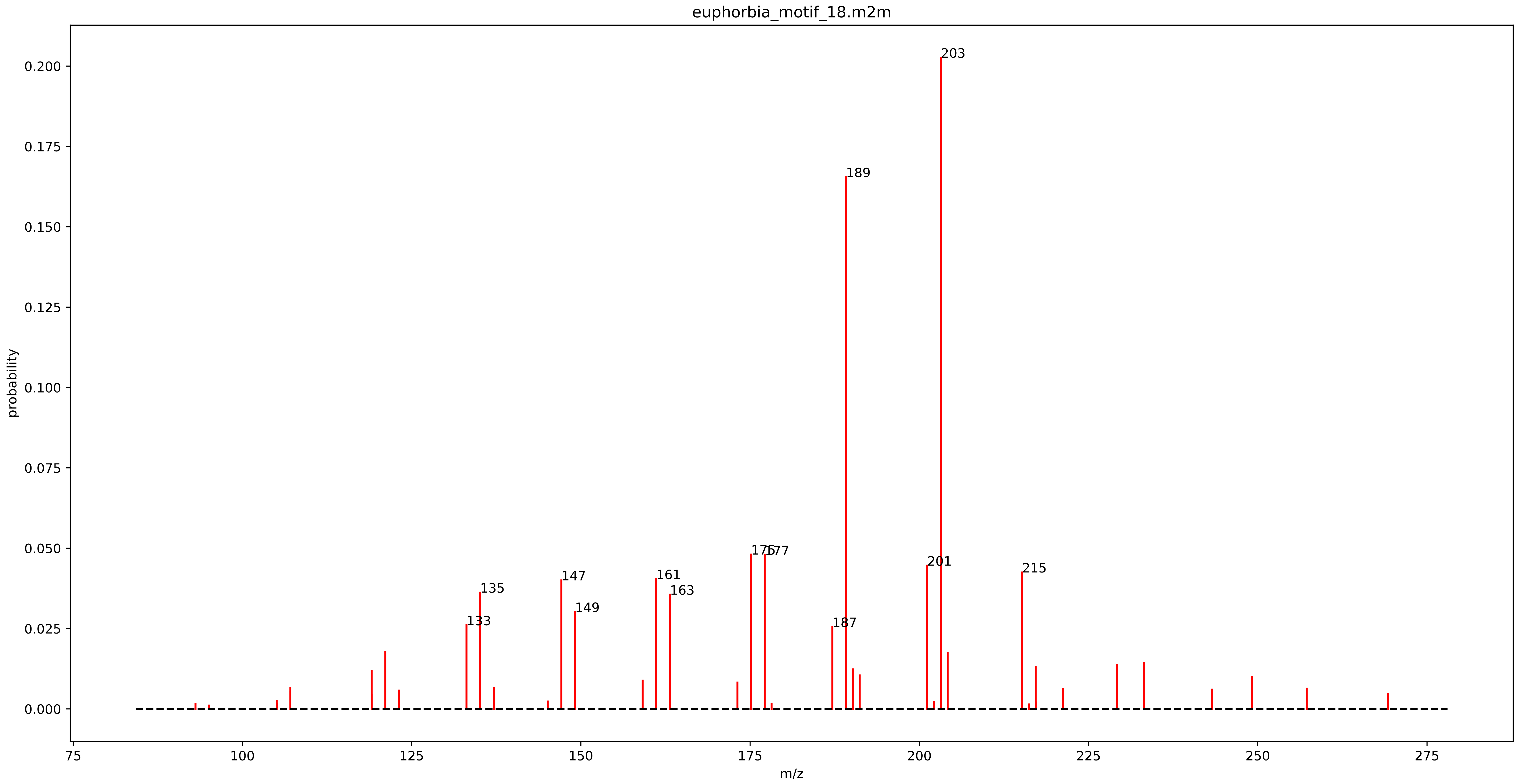

euphorbia_motif_18.m2m
probability
m/z
0.000
0.025
0.050
0.075
0.100
0.125
0.150
0.175
0.200
75
100
125
150
175
200
225
250
275
203
189
175
177
201
215
147
161
135
163
149
133
187

euphorbia_motif_18.m2m

fragment_203.1775	: 0.20
fragment_189.1625	: 0.17
fragment_175.1475	: 0.05
fragment_177.1625	: 0.05
fragment_201.1625	: 0.04
fragment_215.1775	: 0.04
fragment_161.1325	: 0.04
fragment_147.1175	: 0.04
fragment_135.1175	: 0.04
fragment_163.1475	: 0.04
fragment_149.1325	: 0.03
fragment_133.1025	: 0.03
fragment_187.1475	: 0.03
fragment_121.1025	: 0.02
fragment_204.1825	: 0.02
fragment_233.1875	: 0.01
fragment_229.1925	: 0.01
fragment_217.1925	: 0.01
fragment_190.1675	: 0.01
fragment_119.0825	: 0.01
fragment_191.1775	: 0.01
fragment_249.1825	: 0.01
fragment_159.1175	: 0.01
fragment_173.1325	: 0.01
fragment_137.1325	: 0.01
fragment_107.0875	: 0.01
fragment_257.2225	: 0.01
fragment_221.1875	: 0.01
fragment_243.2125	: 0.01
fragment_123.1175	: 0.01
fragment_107.0825	: 0.01

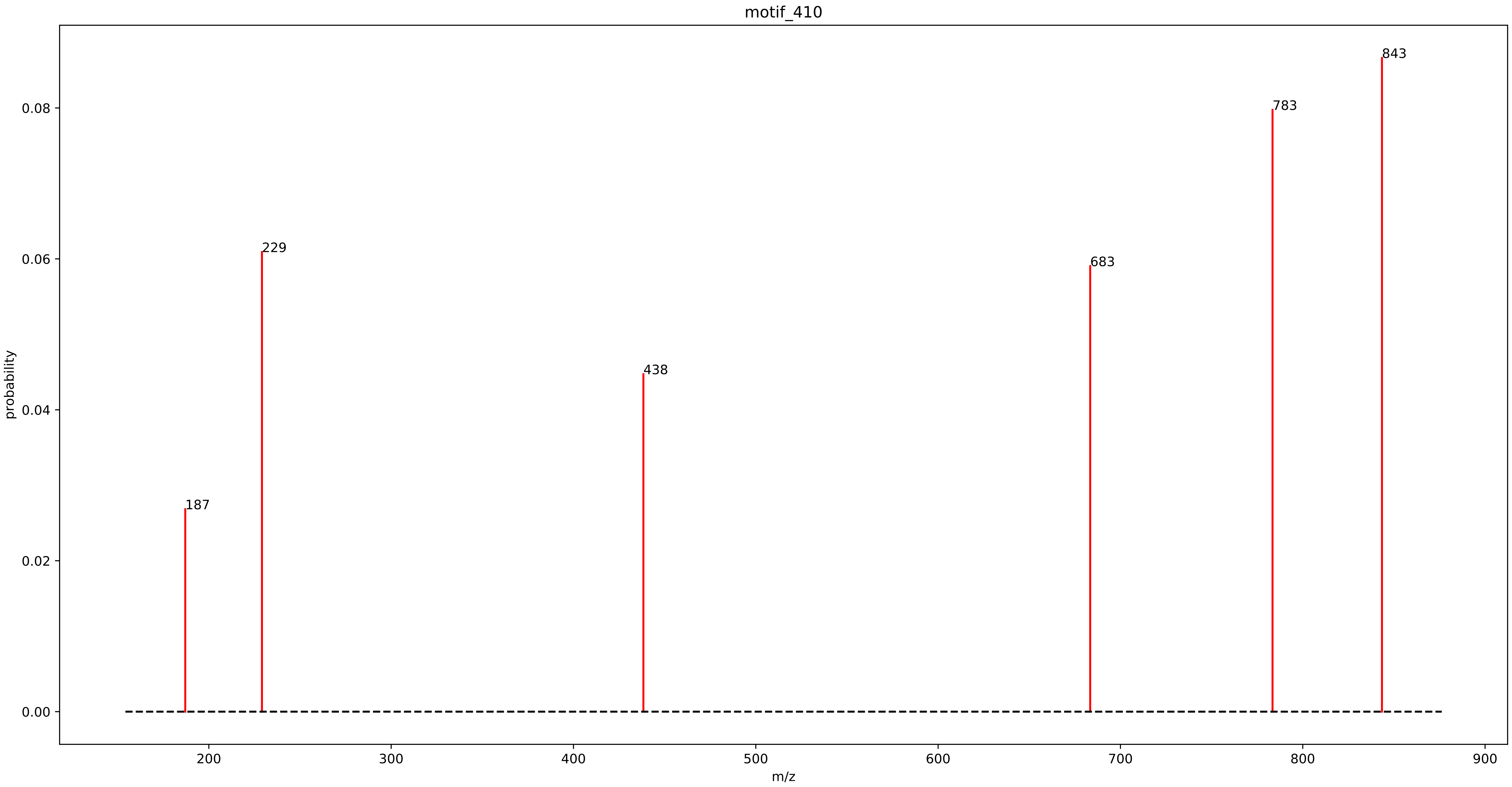

motif_410
probability
m/z
187
229
438
683
783
843
0.00
0.02
0.04
0.06
0.08
200
300
400
500
600
700
800
900

motif_410

fragment_843.4425	: 0.09
fragment_783.4175	: 0.08
fragment_229.1325	: 0.06
fragment_683.4175	: 0.06
fragment_438.3425	: 0.04
fragment_187.0625	: 0.03

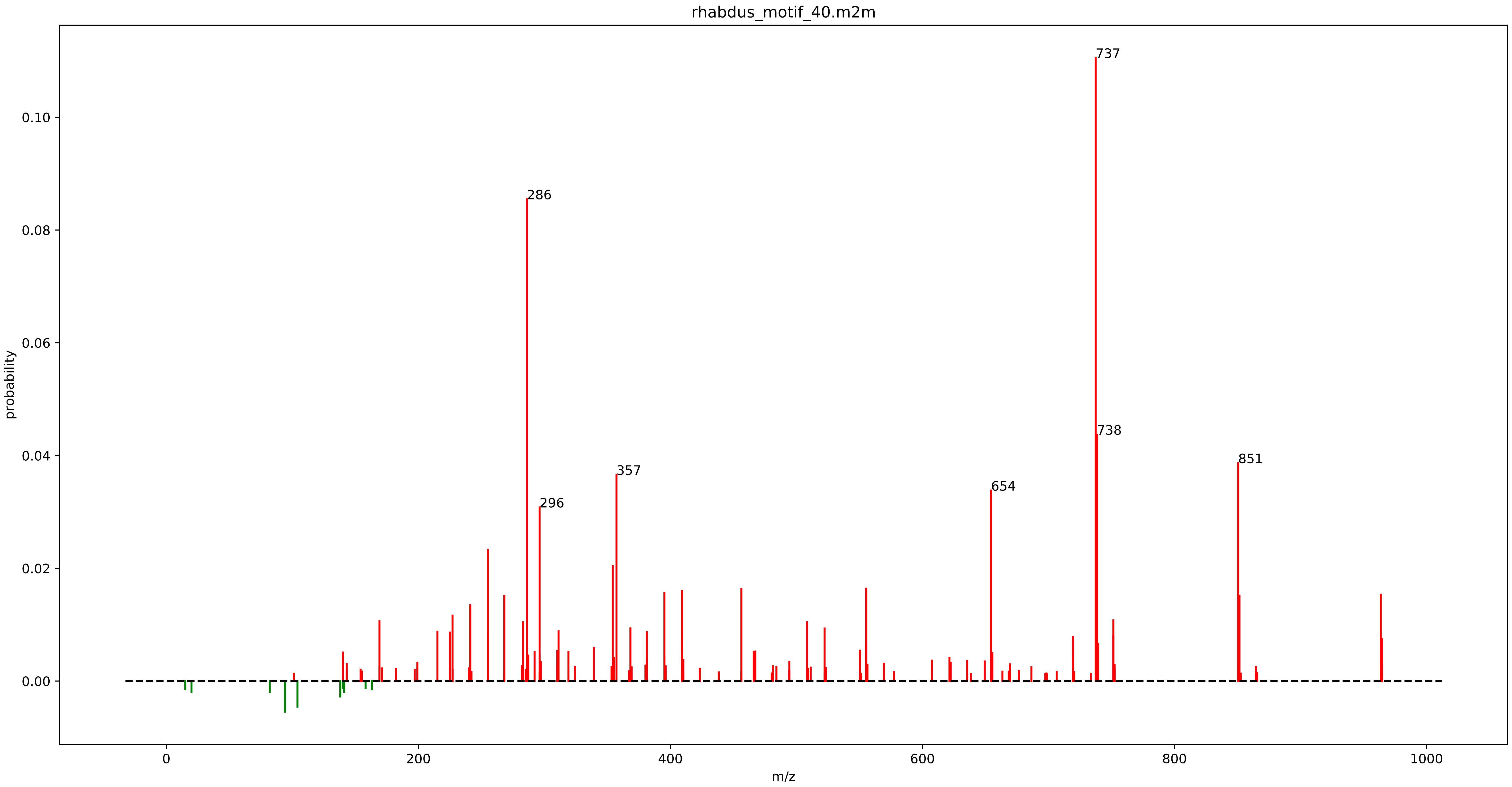

rhabdus_motif_40.m2m
probability
m/z
0.10
0.08
0.06
0.04
0.02
0.00
0
200
400
600
800
1000
737
286
738
851
357
654
296

rhabdus_motif_40.m2m

fragment_737.4575 : 0.11
fragment_286.1775 : 0.09
fragment_738.4575 : 0.04
fragment_850.5375 : 0.04
fragment_357.2125 : 0.04
fragment_654.4175 : 0.03
fragment_296.1625 : 0.03
fragment_255.1125 : 0.02
fragment_354.2375 : 0.02
fragment_555.3475 : 0.02
fragment_456.2825 : 0.02
fragment_409.2425 : 0.02
fragment_395.2275 : 0.02
fragment_963.6225 : 0.02
fragment_851.5425 : 0.02
fragment_268.1675 : 0.02
fragment_241.1525 : 0.01
fragment_227.1175 : 0.01
fragment_751.4675 : 0.01
fragment_169.0975 : 0.01
fragment_508.3125 : 0.01
fragment_283.1075 : 0.01
fragment_368.2525 : 0.01
fragment_522.3275 : 0.01
fragment_311.2075 : 0.01
fragment_215.1375 : 0.01
fragment_381.2475 : 0.01
fragment_225.1225 : 0.01
fragment_255.1675 : 0.01
fragment_751.4725 : 0.01
fragment_719.4475 : 0.01
fragment_963.6175 : 0.01
fragment_964.6225 : 0.01
fragment_409.2775 : 0.01
fragment_555.3525 : 0.01
fragment_739.4625 : 0.01
fragment_339.2025 : 0.01
loss_94.0425 : 0.01
fragment_550.3575 : 0.01
fragment_310.2125 : 0.01
fragment_467.3225 : 0.01
fragment_466.0775 : 0.01
fragment_319.0525 : 0.01
fragment_292.2225 : 0.01
fragment_140.1025 : 0.01
fragment_409.2475 : 0.01

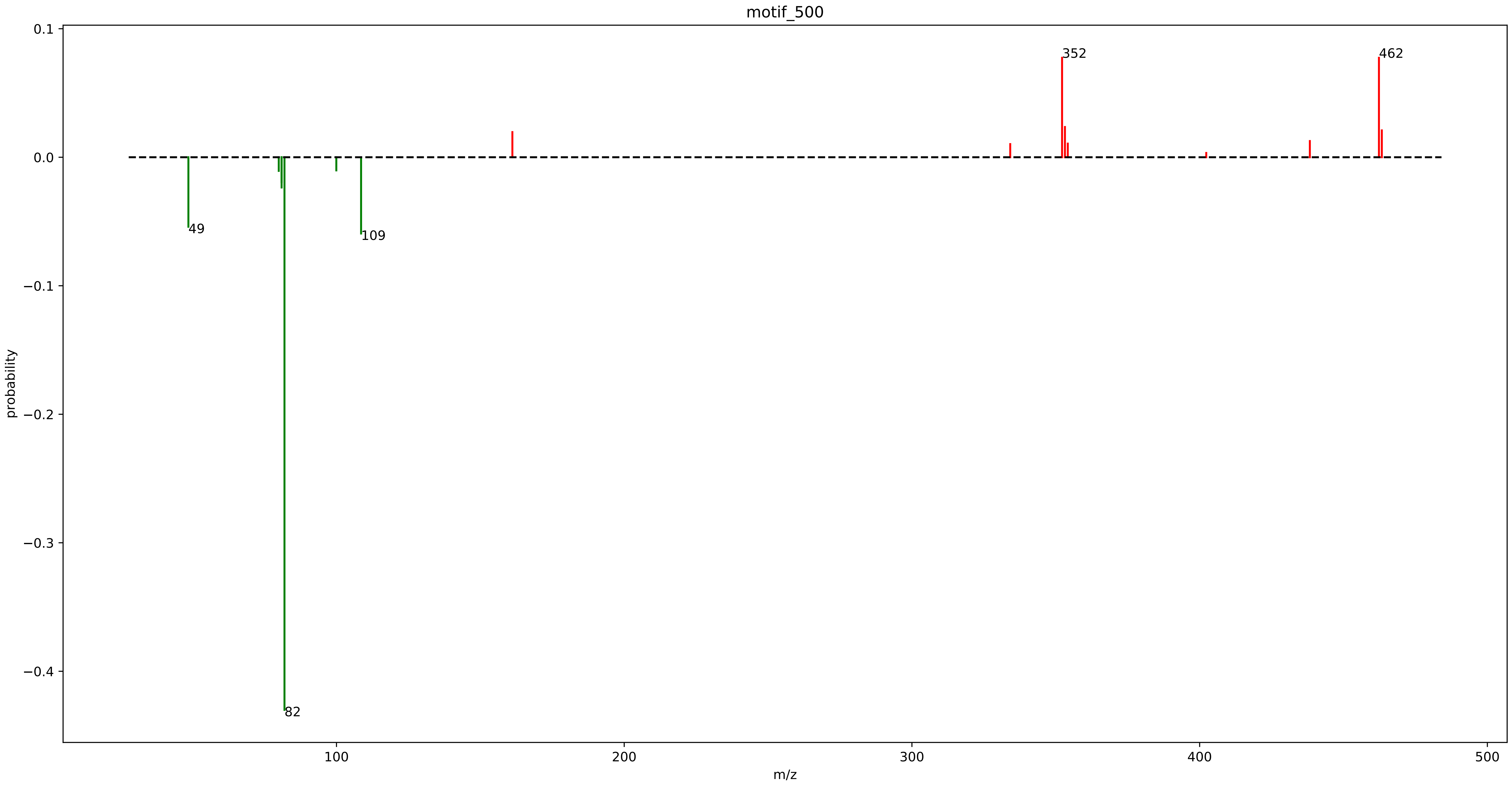
motif_500
probability
m/z
0.1
0.0
−0.1
−0.2
−0.3
−0.4
100
200
300
400
500
352
462
49
82
109

motif_500

loss_81.9225	: 0.43
fragment_352.1825	: 0.08
fragment_462.3175	: 0.08
loss_108.5475	: 0.06
loss_48.5275	: 0.05
loss_80.9225	: 0.02
fragment_353.1825	: 0.02
fragment_463.3225	: 0.02
fragment_161.1325	: 0.02
loss_80.9175	: 0.02
fragment_438.3025	: 0.01
fragment_354.1725	: 0.01
loss_79.9325	: 0.01
loss_99.9425	: 0.01
fragment_334.1675	: 0.01

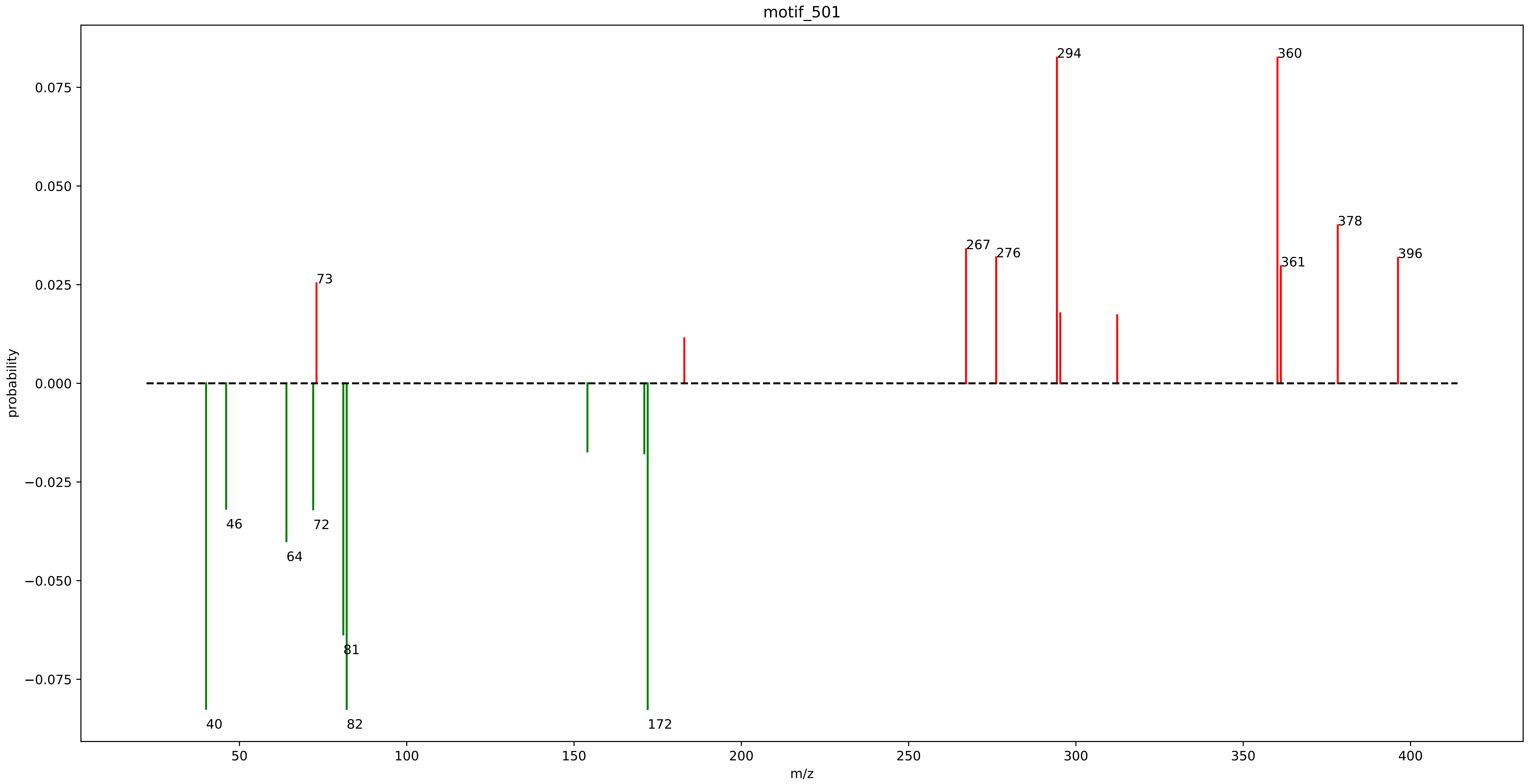
motif_501
probability
m/z
0.075
0.050
0.025
0.000
−0.025
−0.050
−0.075
50
100
150
200
250
300
350
400
73
267
276
294
360
361
378
396
40
46
64
72
81
82
172

motif_501

fragment_294.3175	: 0.08
loss_172.0125	: 0.08
fragment_360.2375	: 0.08
loss_82.0525	: 0.08
loss_40.0275	: 0.08
loss_81.0475	: 0.06
fragment_378.2475	: 0.04
loss_64.0425	: 0.04
fragment_267.1575	: 0.03
loss_72.0425	: 0.03
fragment_276.1625	: 0.03
loss_46.0275	: 0.03
fragment_396.2575	: 0.03
fragment_361.2375	: 0.03
fragment_73.0275	: 0.03
loss_171.0075	: 0.02
fragment_295.3175	: 0.02
fragment_312.3275	: 0.02
loss_154.0025	: 0.02
fragment_182.9625	: 0.01

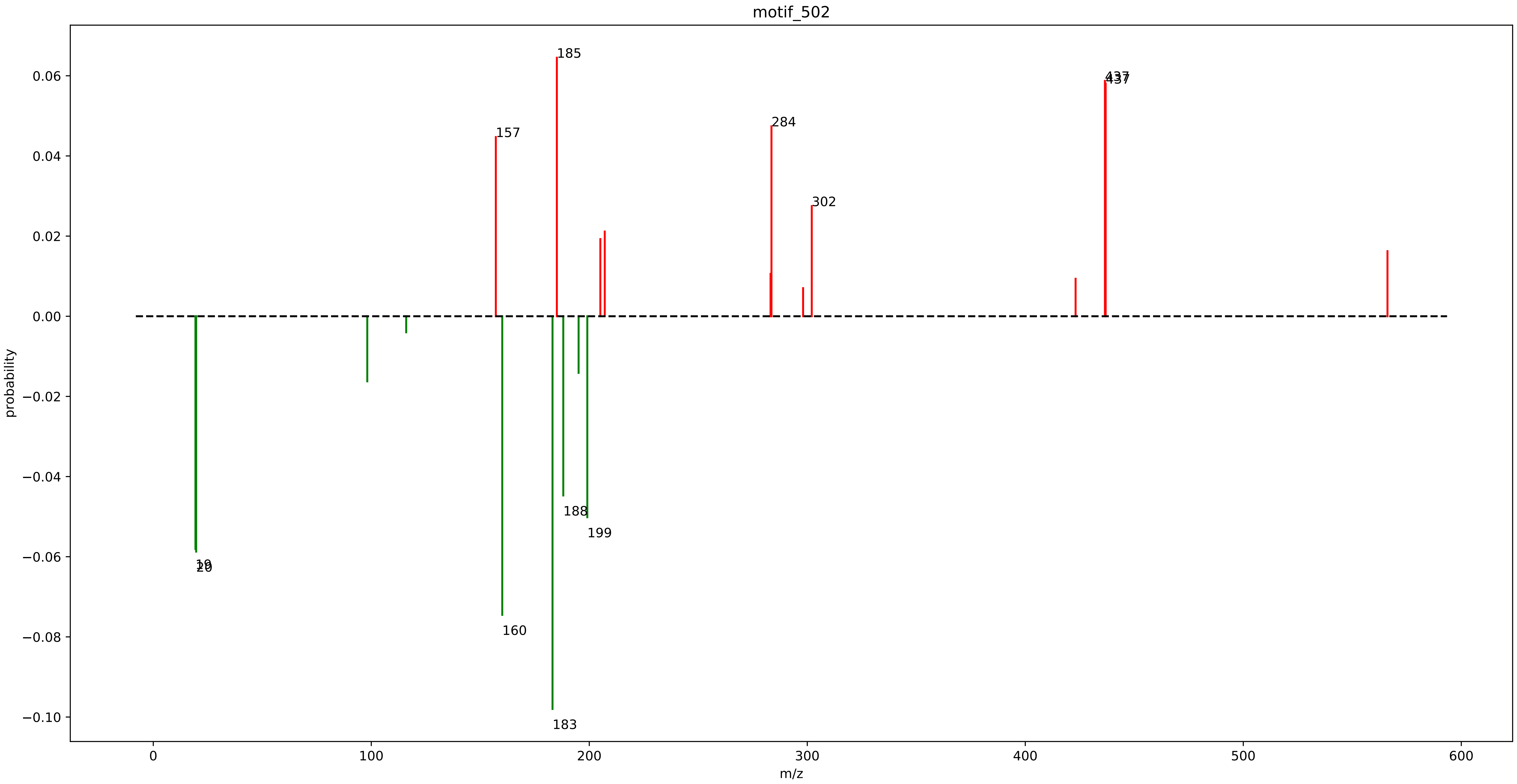
motif_502
185
437
437
157
284
302
188
199
19
20
160
183
0.06
0.04
0.02
0.00
−0.02
−0.04
−0.06
−0.08
−0.10
probability
0
100
200
300
400
500
600
m/z

motif_502

loss_183.1375	: 0.10
loss_160.0825	: 0.07
fragment_185.1275	: 0.06
loss_19.6775	: 0.06
fragment_436.5375	: 0.06
fragment_436.8725	: 0.06
loss_19.3425	: 0.06
loss_199.0975	: 0.05
fragment_283.5725	: 0.05
loss_188.0775	: 0.04
fragment_157.1325	: 0.04
fragment_302.0525	: 0.03
fragment_207.0975	: 0.02
fragment_205.0775	: 0.02
loss_98.1675	: 0.02
fragment_566.1425	: 0.02
loss_195.1025	: 0.01
fragment_283.1475	: 0.01
fragment_423.0775	: 0.01
fragment_298.0875	: 0.01

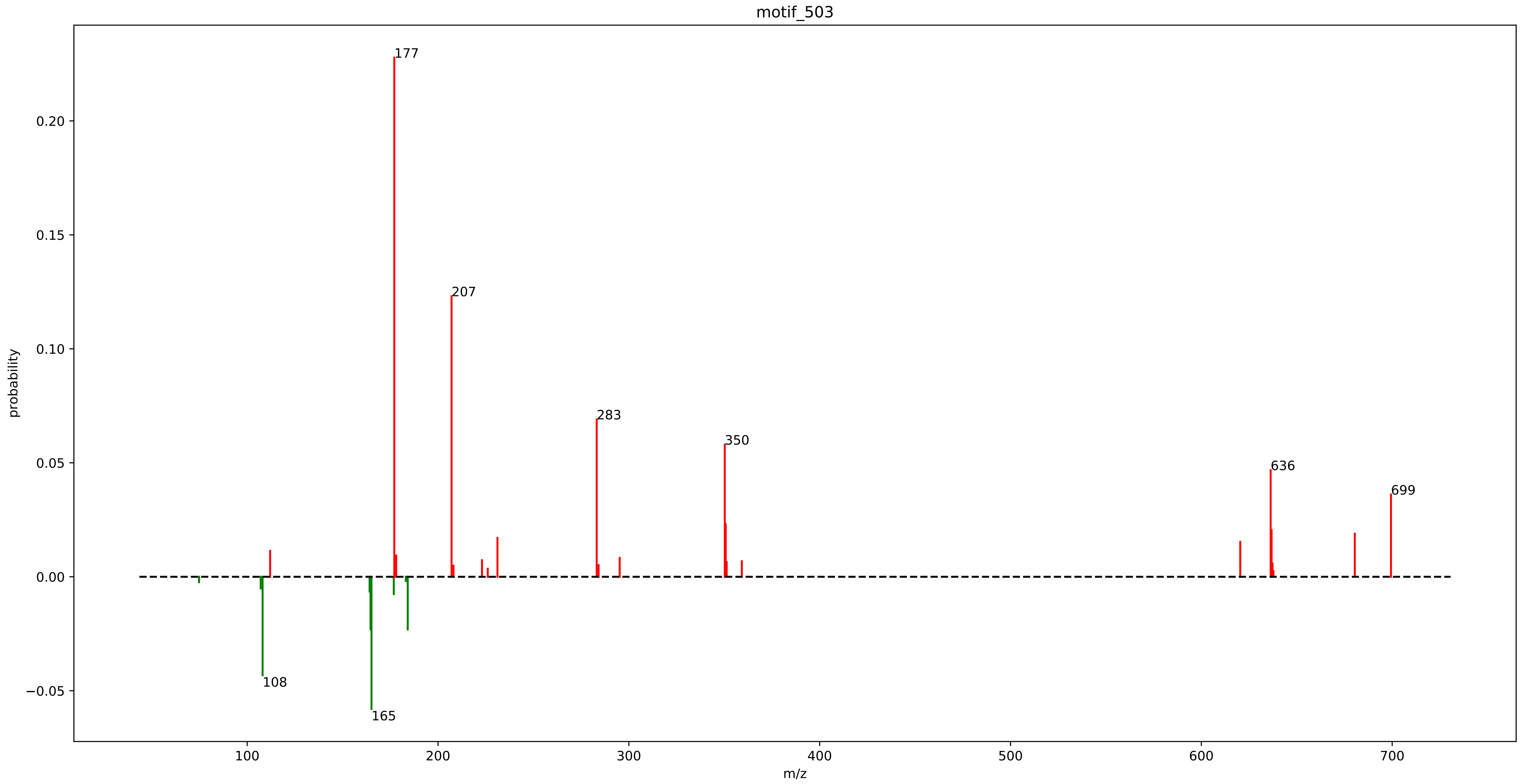

motif_503
probability
m/z
0.20
0.15
0.10
0.05
0.00
−0.05
100
200
300
400
500
600
700
177
207
283
350
636
699
108
165

motif_503

fragment_177.0875	: 0.23
fragment_207.0975	: 0.12
fragment_283.1525	: 0.07
loss_165.1375	: 0.06
fragment_350.2575	: 0.06
fragment_636.3075	: 0.05
loss_108.0775	: 0.04
fragment_699.3775	: 0.04
loss_184.1275	: 0.02
loss_164.6325	: 0.02
fragment_350.7575	: 0.02
fragment_636.8075	: 0.02
fragment_680.4075	: 0.02
fragment_231.1425	: 0.02
fragment_620.3825	: 0.02
fragment_112.0275	: 0.01
fragment_178.0875	: 0.01
fragment_295.1925	: 0.01
loss_176.8125	: 0.01
fragment_223.0475	: 0.01
fragment_359.2025	: 0.01
fragment_351.2625	: 0.01
loss_164.1325	: 0.01
fragment_637.3075	: 0.01
fragment_284.1525	: 0.01
loss_107.0725	: 0.01

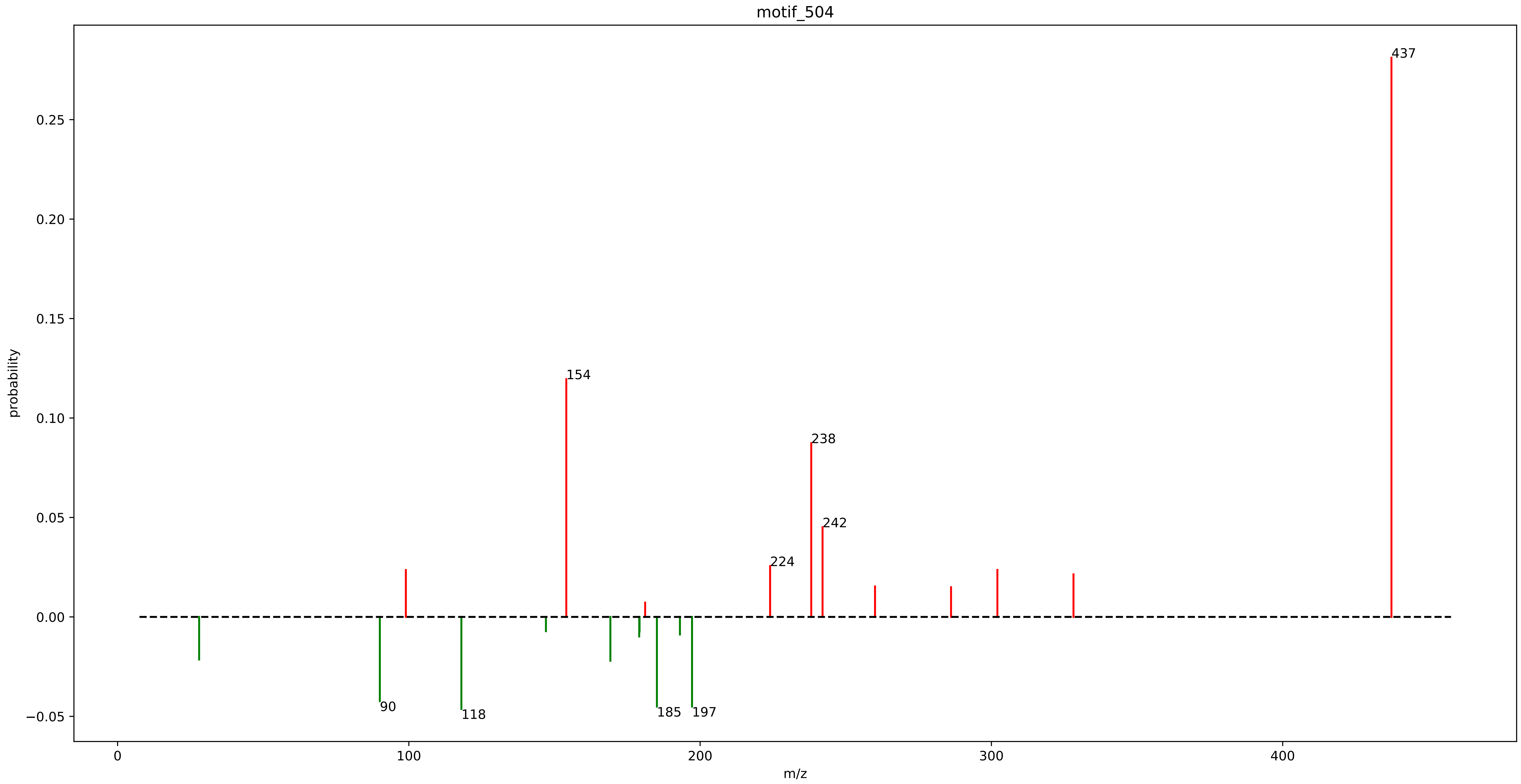

motif_504
probability
m/z
0.25
0.20
0.15
0.10
0.05
0.00
−0.05
0
100
200
300
400
437
154
238
242
224
90
118
185
197

motif_504

fragment_437.3375	: 0.28
fragment_154.0525	: 0.12
fragment_238.1475	: 0.09
loss_118.0325	: 0.05
fragment_242.0325	: 0.05
loss_185.1625	: 0.05
loss_197.2225	: 0.05
loss_90.0375	: 0.04
fragment_224.0225	: 0.03
fragment_302.0525	: 0.02
fragment_98.9725	: 0.02
loss_169.1875	: 0.02
loss_27.9975	: 0.02
fragment_328.1775	: 0.02
fragment_260.0425	: 0.02
fragment_286.1425	: 0.01
loss_179.0875	: 0.01
loss_193.0675	: 0.01
loss_179.2075	: 0.01
loss_147.0625	: 0.01
fragment_181.1225	: 0.01

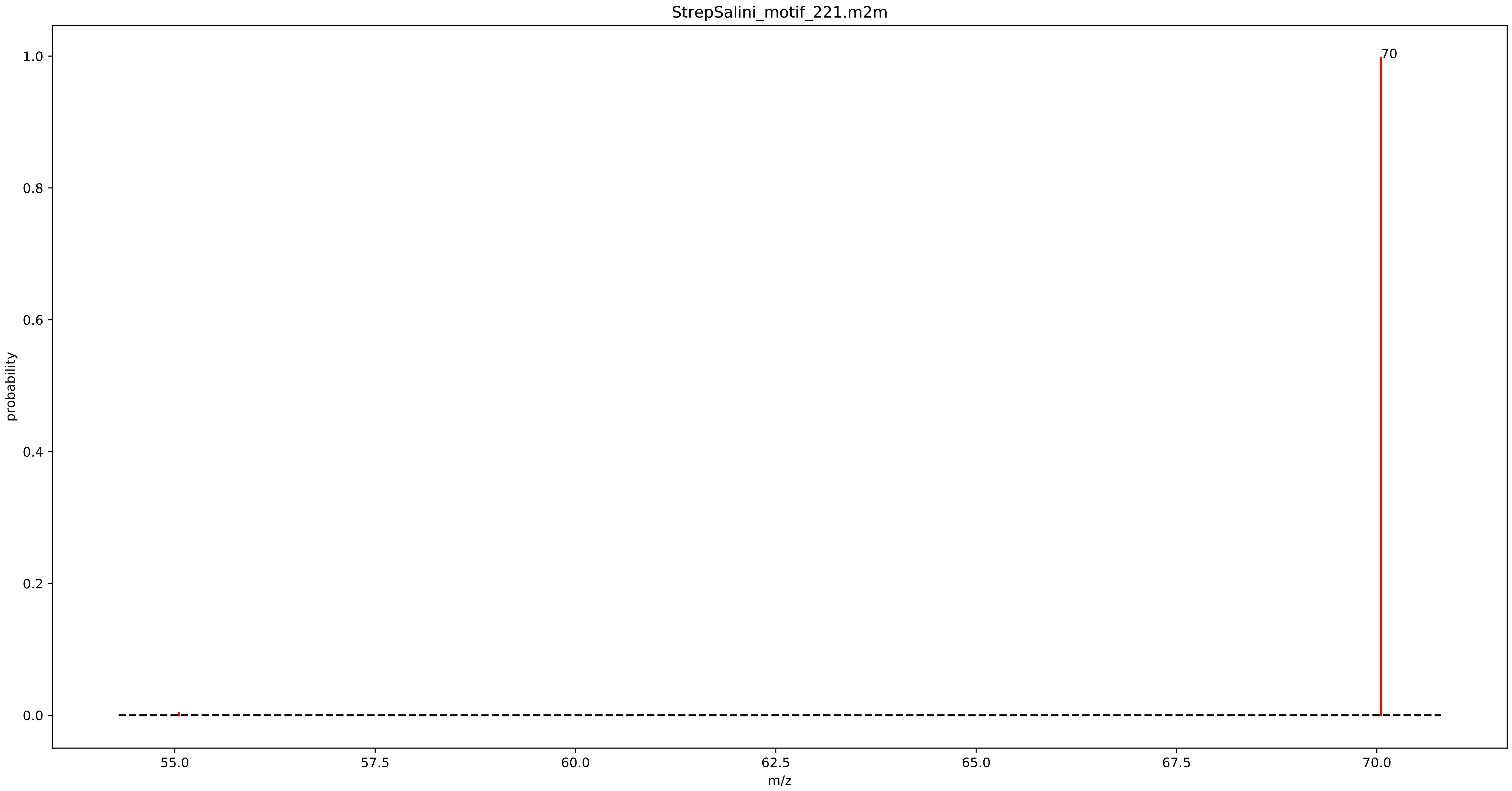

StrepSalini_motif_221.m2m
probability
1.0
0.8
0.6
0.4
0.2
0.0
55.0
57.5
60.0
62.5
65.0
67.5
70.0
m/z
70

StrepSalini_motif_221.m2m

fragment_70.0500 : 1.00

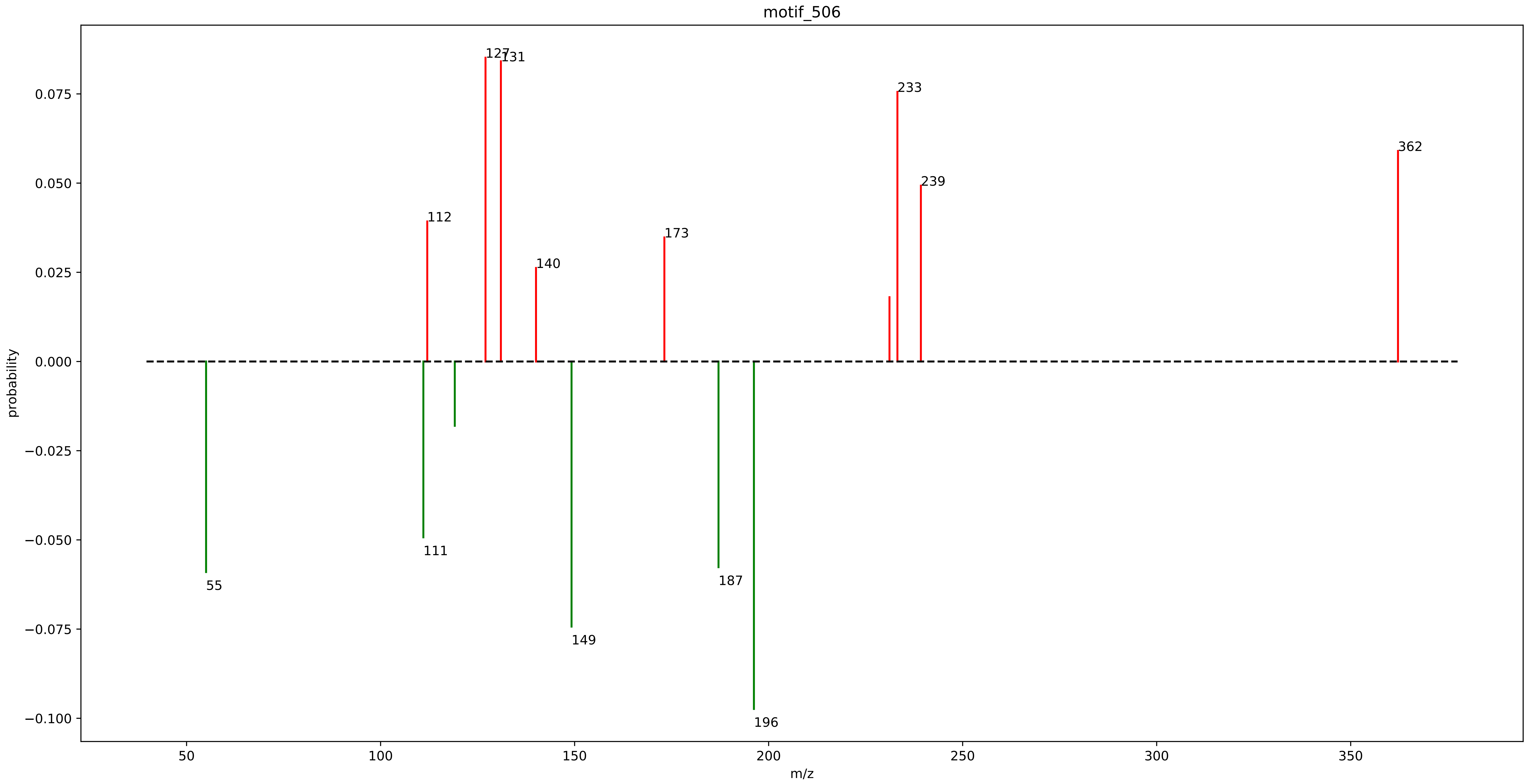

motif_506
probability
m/z
0.075
0.050
0.025
0.000
−0.025
−0.050
−0.075
−0.100
50
100
150
200
250
300
350
127
131
233
362
239
112
173
140
55
111
149
187
196

motif_506

loss_196.2075	: 0.10
fragment_127.0375	: 0.09
fragment_131.0025	: 0.08
fragment_233.1875	: 0.08
loss_149.2075	: 0.07
loss_55.0275	: 0.06
fragment_362.1925	: 0.06
loss_187.0825	: 0.06
fragment_239.2375	: 0.05
loss_111.0225	: 0.05
fragment_112.0275	: 0.04
fragment_173.1325	: 0.03
fragment_140.0525	: 0.03
loss_119.1175	: 0.02
fragment_231.1425	: 0.02

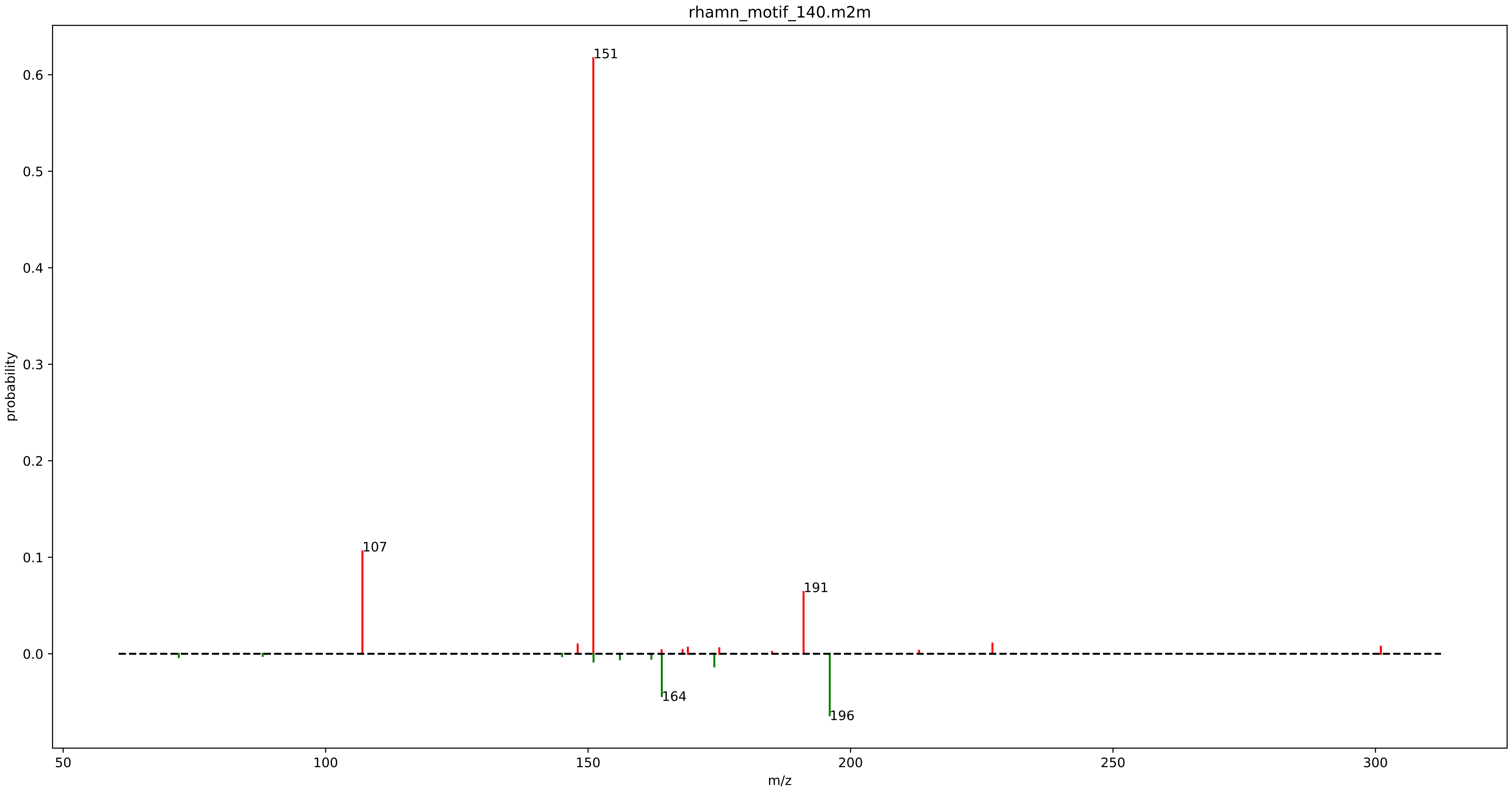

rhamn_motif_140.m2m
probability
m/z
0.6
0.5
0.4
0.3
0.2
0.1
0.0
50
100
150
200
250
300
151
107
191
164
196

rhamn_motif_140.m2m

fragment_151.0025	: 0.62
fragment_107.0125	: 0.11
fragment_191.0525	: 0.06
loss_196.0375	: 0.06
loss_164.0475	: 0.04
loss_174.0525	: 0.01
fragment_227.0325	: 0.01
fragment_148.0175	: 0.01
loss_151.0375	: 0.01
fragment_301.0375	: 0.01
fragment_169.0075	: 0.01
loss_156.0675	: 0.01
fragment_175.0025	: 0.01
loss_162.0575	: 0.01
fragment_301.0225	: 0.01

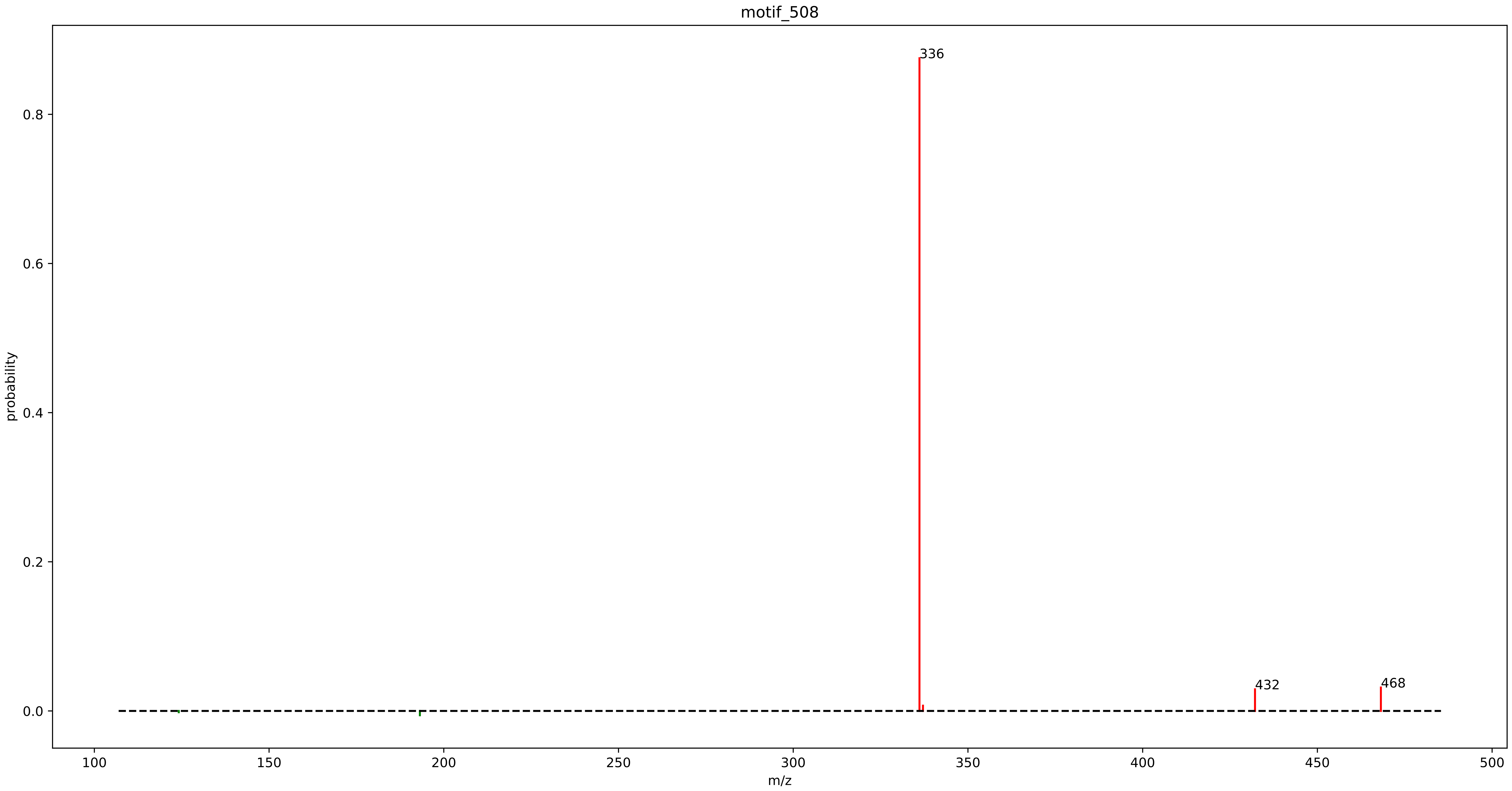

motif_508
336
432
468
probability
m/z
0.0
0.2
0.4
0.6
0.8
100
150
200
250
300
350
400
450
500

motif_508

fragment_336.1275	: 0.88
fragment_468.1725	: 0.03
fragment_432.1475	: 0.03
fragment_337.1325	: 0.01
loss_193.1575	: 0.01

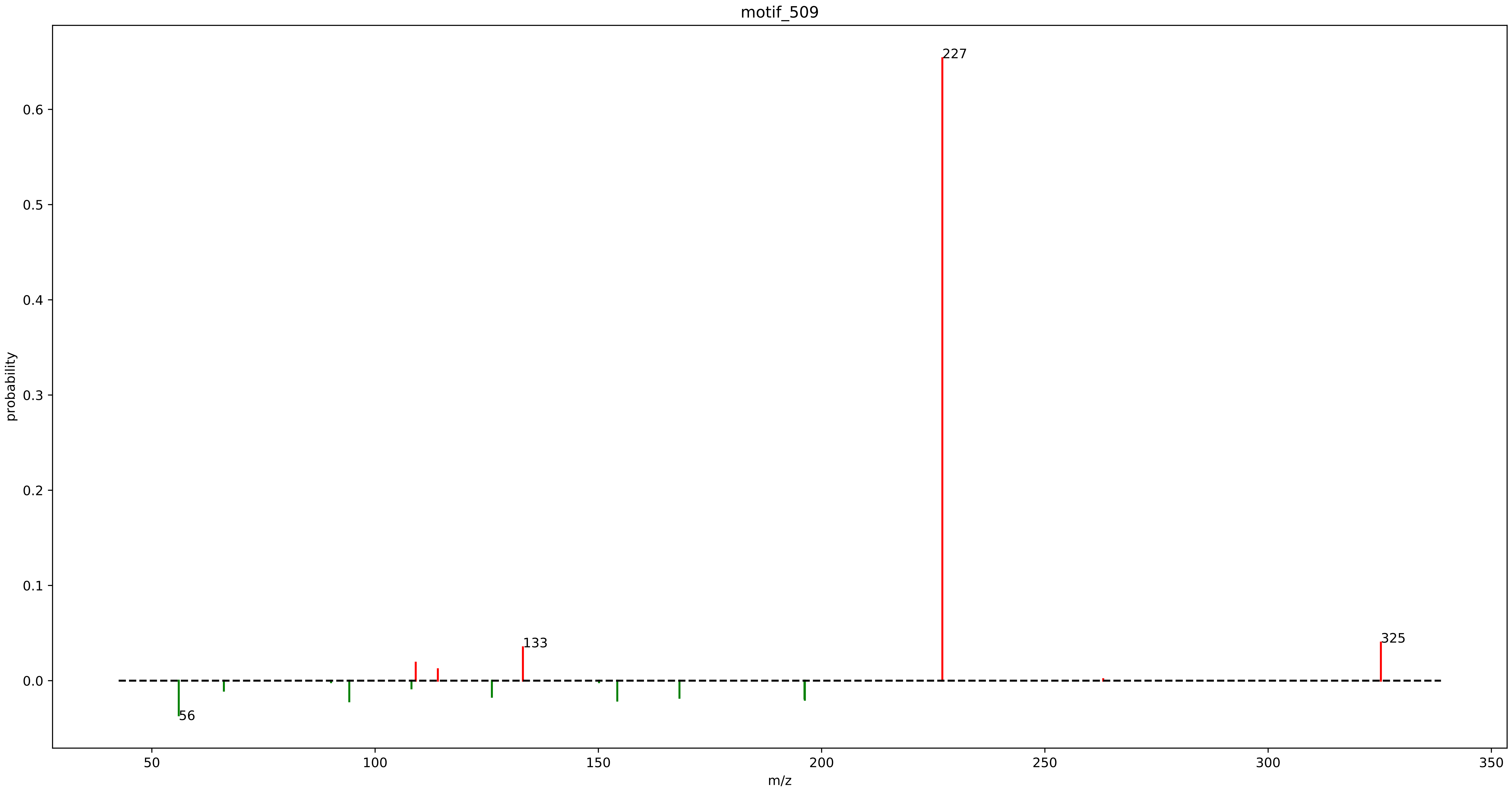

motif_509
227
133
325
56
probability
m/z
0.0
0.1
0.2
0.3
0.4
0.5
0.6
50
100
150
200
250
300
350

motif_509

fragment_227.0375	: 0.65
fragment_325.2525	: 0.04
loss_56.0275	: 0.04
fragment_133.1025	: 0.04
loss_94.2175	: 0.02
loss_154.2375	: 0.02
loss_196.2475	: 0.02
fragment_109.0975	: 0.02
loss_196.1625	: 0.02
loss_168.1625	: 0.02
loss_126.1525	: 0.02
fragment_114.0525	: 0.01
loss_66.1325	: 0.01
loss_108.1425	: 0.01

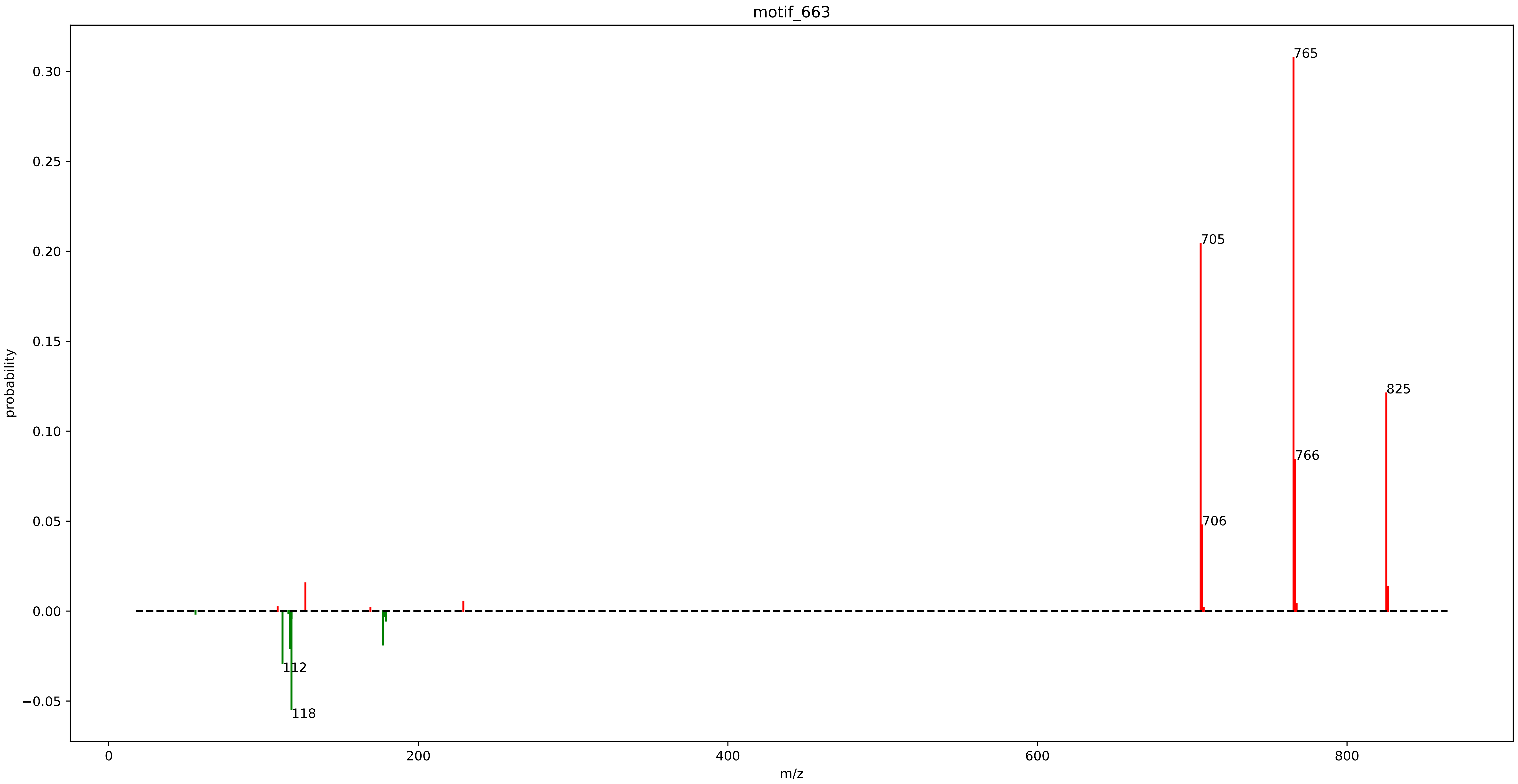

motif_663
probability
m/z
0.30
0.25
0.20
0.15
0.10
0.05
0.00
−0.05
0
200
400
600
800
765
705
825
766
706
112
118

motif_663

fragment_765.4175	: 0.31
fragment_705.3975	: 0.20
fragment_825.4425	: 0.12
fragment_766.4225	: 0.08
loss_118.0225	: 0.05
fragment_706.4025	: 0.05
loss_112.2275	: 0.03
loss_117.0175	: 0.02
loss_177.0525	: 0.02
fragment_127.0375	: 0.02
fragment_826.4425	: 0.01
loss_179.0575	: 0.01
fragment_229.0775	: 0.01

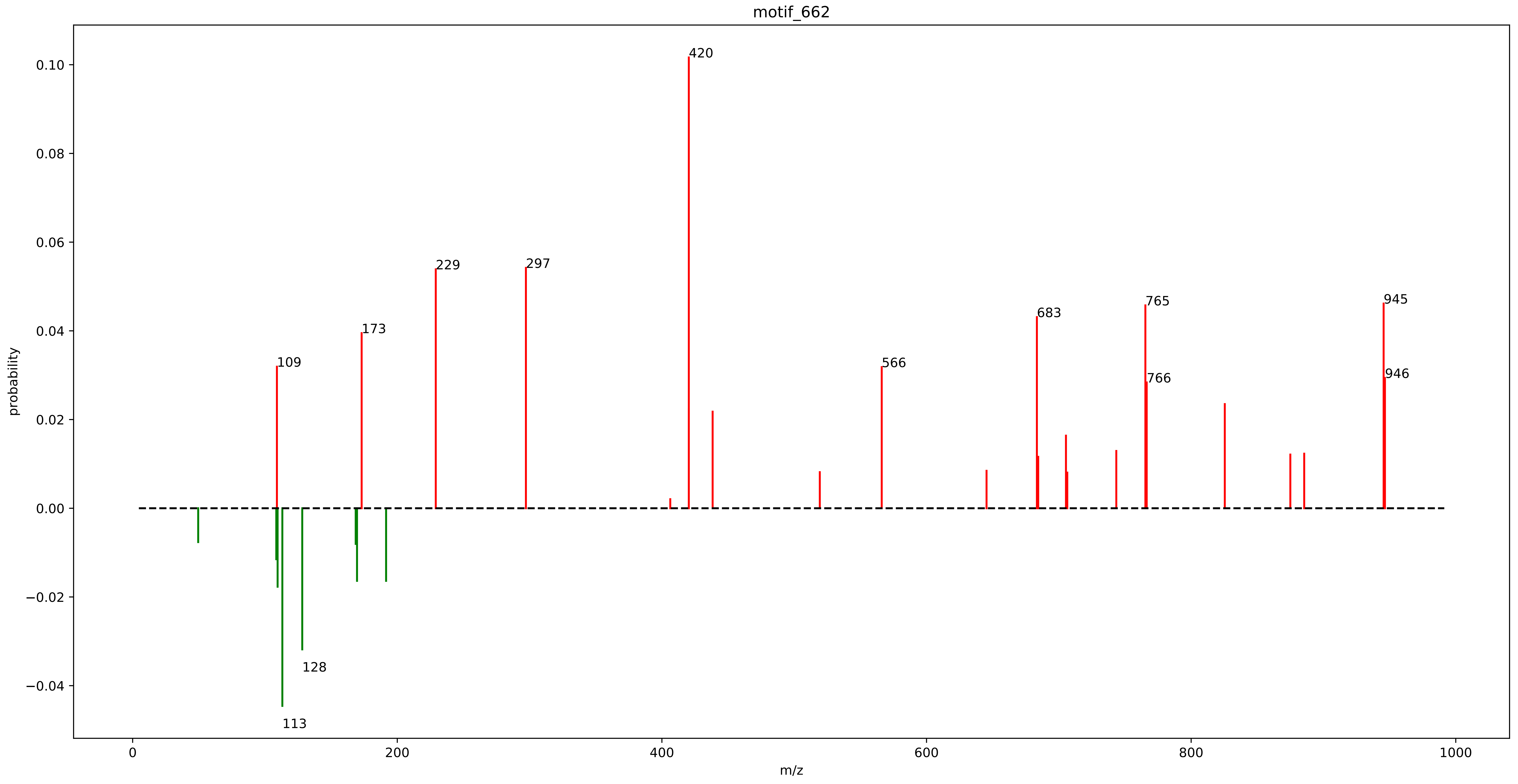

motif_662
probability
m/z
0.10
0.08
0.06
0.04
0.02
0.00
−0.02
−0.04
0
200
400
600
800
1000
420
229
297
173
109
566
683
765
766
945
946
128
113

motif_662

fragment_420.3425	: 0.10
fragment_297.2225	: 0.05
fragment_229.0875	: 0.05
fragment_945.4675	: 0.05
fragment_765.4075	: 0.05
loss_113.1375	: 0.04
fragment_683.4175	: 0.04
fragment_173.0675	: 0.04
fragment_109.0325	: 0.03
fragment_566.1375	: 0.03
loss_128.1775	: 0.03
fragment_946.4725	: 0.03
fragment_766.4075	: 0.03
fragment_825.4275	: 0.02
fragment_420.3325	: 0.02
fragment_438.3425	: 0.02
loss_109.5525	: 0.02
loss_191.5475	: 0.02
fragment_705.3875	: 0.02
loss_169.5775	: 0.02
fragment_743.4375	: 0.01
fragment_885.4525	: 0.01
fragment_874.9675	: 0.01
fragment_684.4175	: 0.01
loss_108.5525	: 0.01
fragment_645.3625	: 0.01
fragment_519.3325	: 0.01
fragment_706.3875	: 0.01
loss_168.5725	: 0.01
loss_49.5325	: 0.01

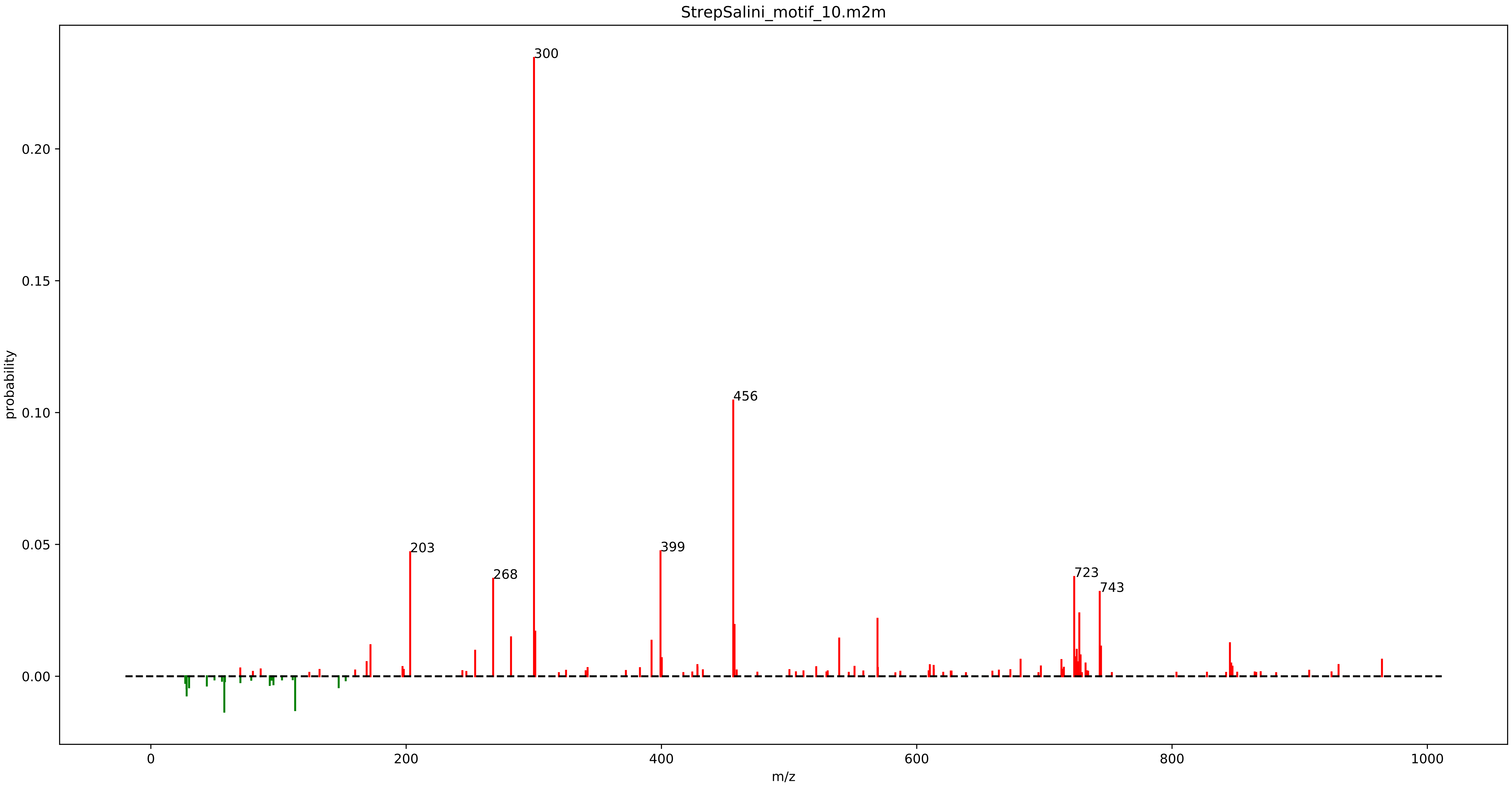

StrepSalini_motif_10.m2m
probability
m/z
300
456
203
399
268
723
743
0.20
0.15
0.10
0.05
0.00
0
200
400
600
800
1000

StrepSalini_motif_10.m2m

fragment_300.1525	: 0.23
fragment_456.2500	: 0.10
fragment_399.2500	: 0.05
fragment_203.1500	: 0.05
fragment_723.3500	: 0.04
fragment_268.1525	: 0.04
fragment_743.3500	: 0.03
fragment_727.3500	: 0.02
fragment_569.2500	: 0.02
fragment_457.2500	: 0.02
fragment_301.1500	: 0.02
fragment_282.1500	: 0.01
fragment_539.2500	: 0.01
fragment_392.2500	: 0.01
loss_57.5500	: 0.01
loss_113.0500	: 0.01
fragment_845.3500	: 0.01
fragment_172.0500	: 0.01
fragment_744.3500	: 0.01
fragment_725.3500	: 0.01
fragment_254.0500	: 0.01
fragment_728.3500	: 0.01
loss_28.0500	: 0.01
fragment_724.3500	: 0.01
fragment_400.2500	: 0.01
fragment_964.4500	: 0.01
fragment_681.3500	: 0.01
fragment_713.3500	: 0.01
fragment_169.0500	: 0.01
fragment_726.3500	: 0.01

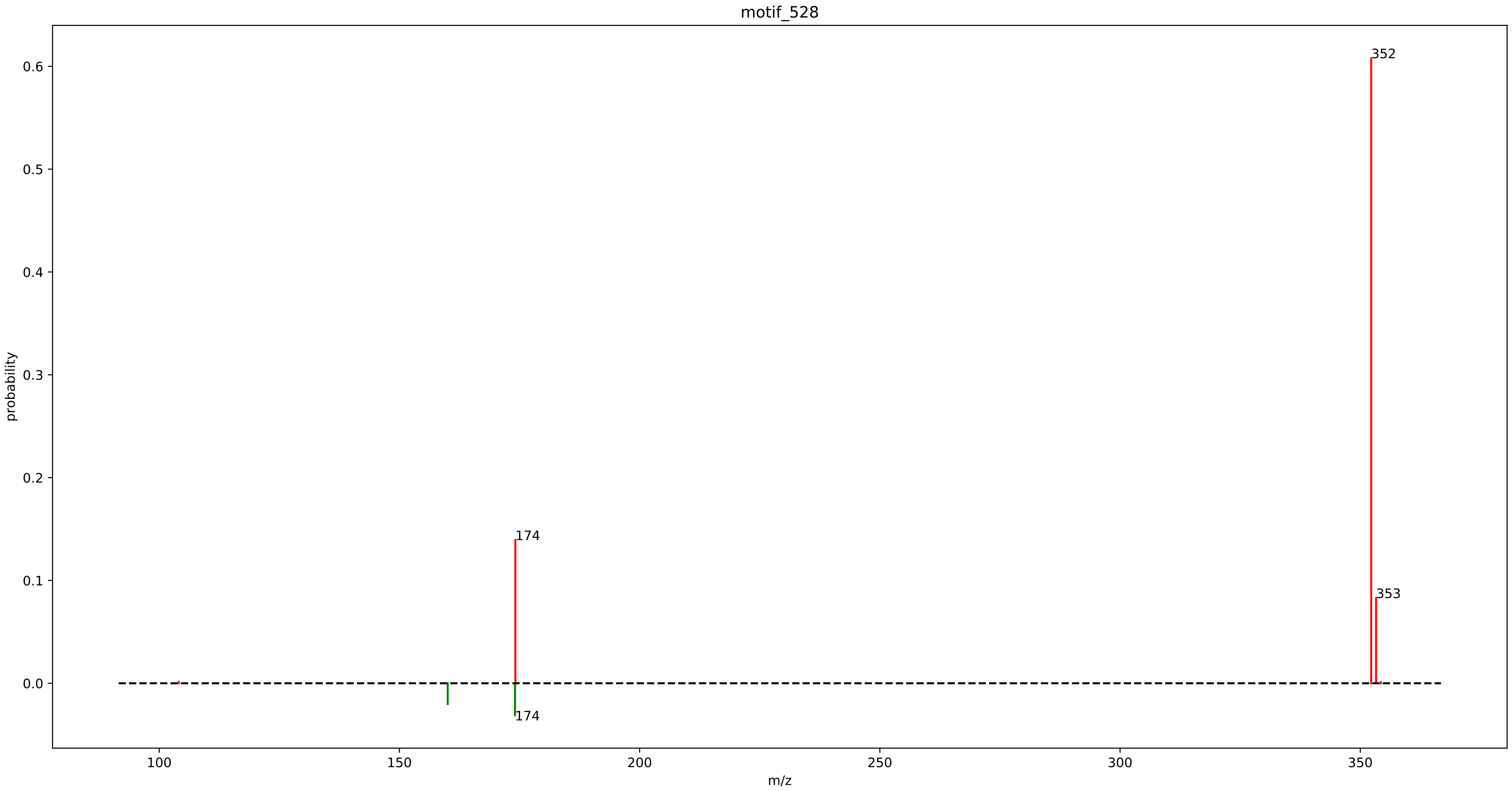

motif_528
352
174
353
174
probability
m/z
0.0
0.1
0.2
0.3
0.4
0.5
0.6
100
150
200
250
300
350

motif_528

fragment_352.2825	: 0.61
fragment_174.1275	: 0.14
fragment_353.2875	: 0.08
loss_174.0625	: 0.03
loss_160.0475	: 0.02

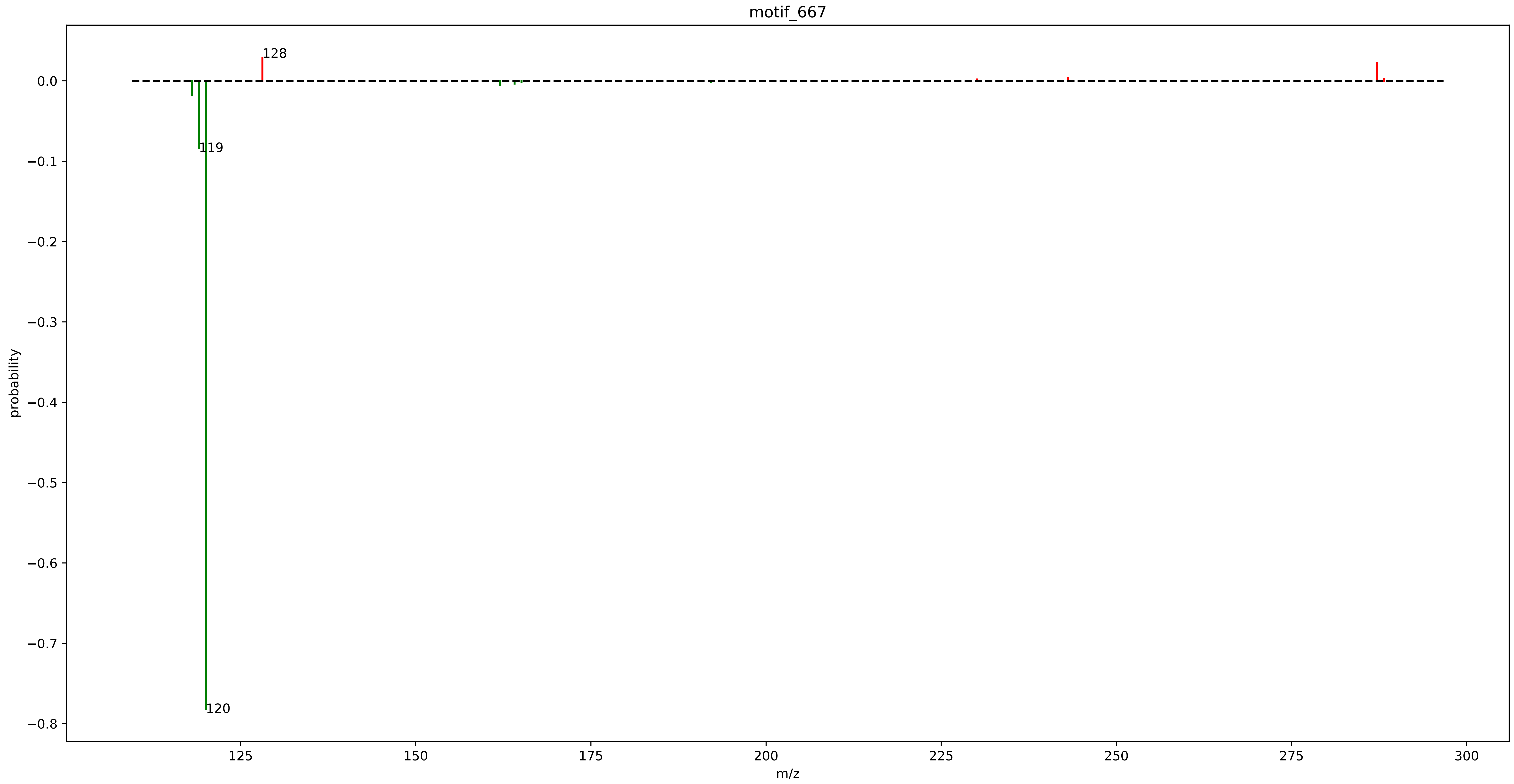

motif_667
128
119
120
0.0
−0.1
−0.2
−0.3
−0.4
−0.5
−0.6
−0.7
−0.8
probability
125
150
175
200
225
250
275
300
m/z

motif_667

loss_120.0375	: 0.78
loss_119.0375	: 0.08
fragment_128.1075	: 0.03
fragment_287.1975	: 0.02
loss_118.0325	: 0.02
loss_162.0475	: 0.01

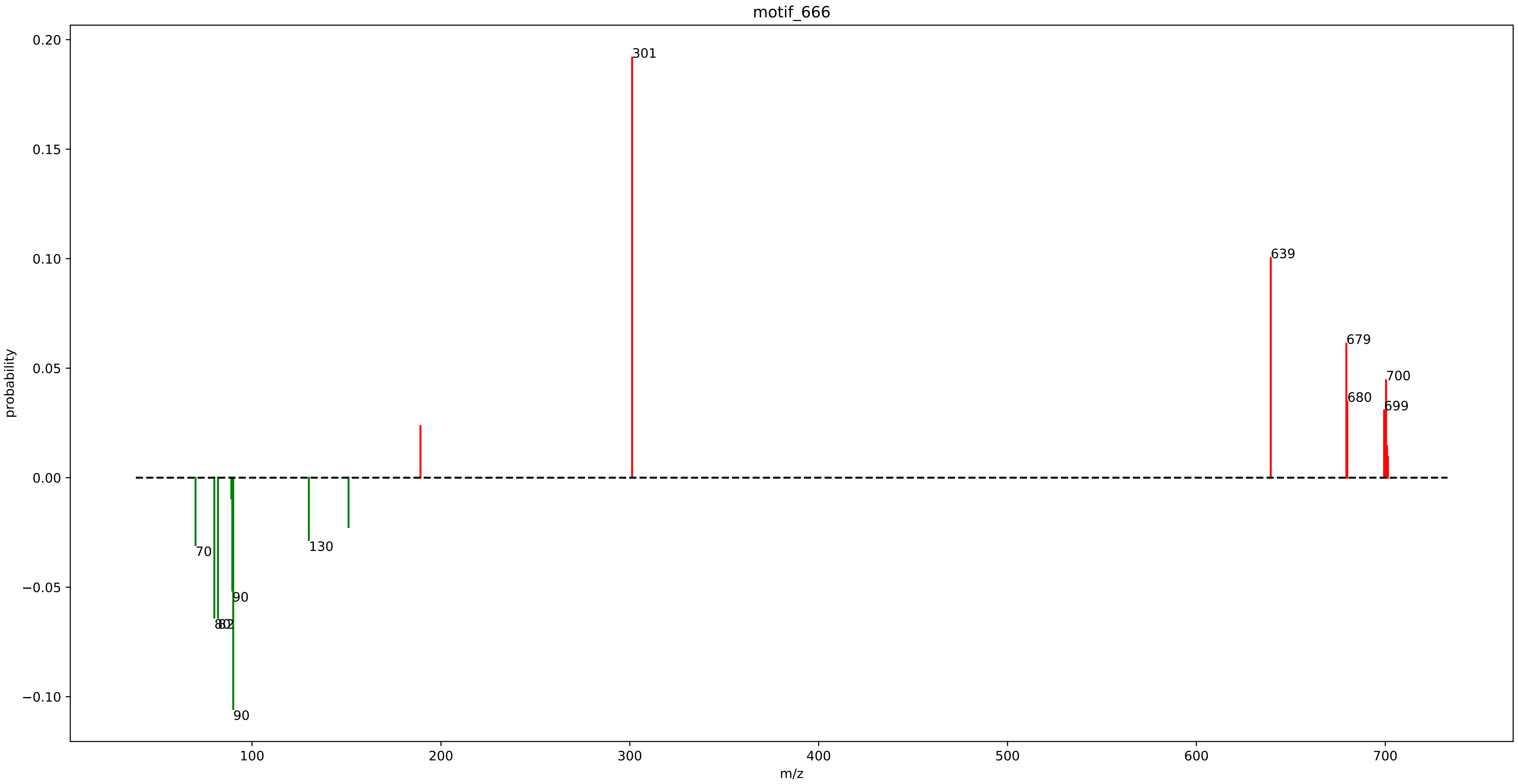

motif_666
probability
m/z
0.20
0.15
0.10
0.05
0.00
−0.05
−0.10
100
200
300
400
500
600
700
301
639
679
700
680
699
70
130
90
80
82
90

motif_666

fragment_301.2175	: 0.19
loss_90.0275	: 0.11
fragment_639.3575	: 0.10
loss_81.9775	: 0.06
loss_80.0225	: 0.06
fragment_679.3975	: 0.06
loss_89.5275	: 0.05
fragment_700.4025	: 0.04
fragment_679.8975	: 0.03
loss_70.0475	: 0.03
fragment_699.3775	: 0.03
loss_130.0675	: 0.03
fragment_189.1625	: 0.02
loss_151.0725	: 0.02
fragment_700.9025	: 0.01
fragment_701.4075	: 0.01
loss_89.0225	: 0.01

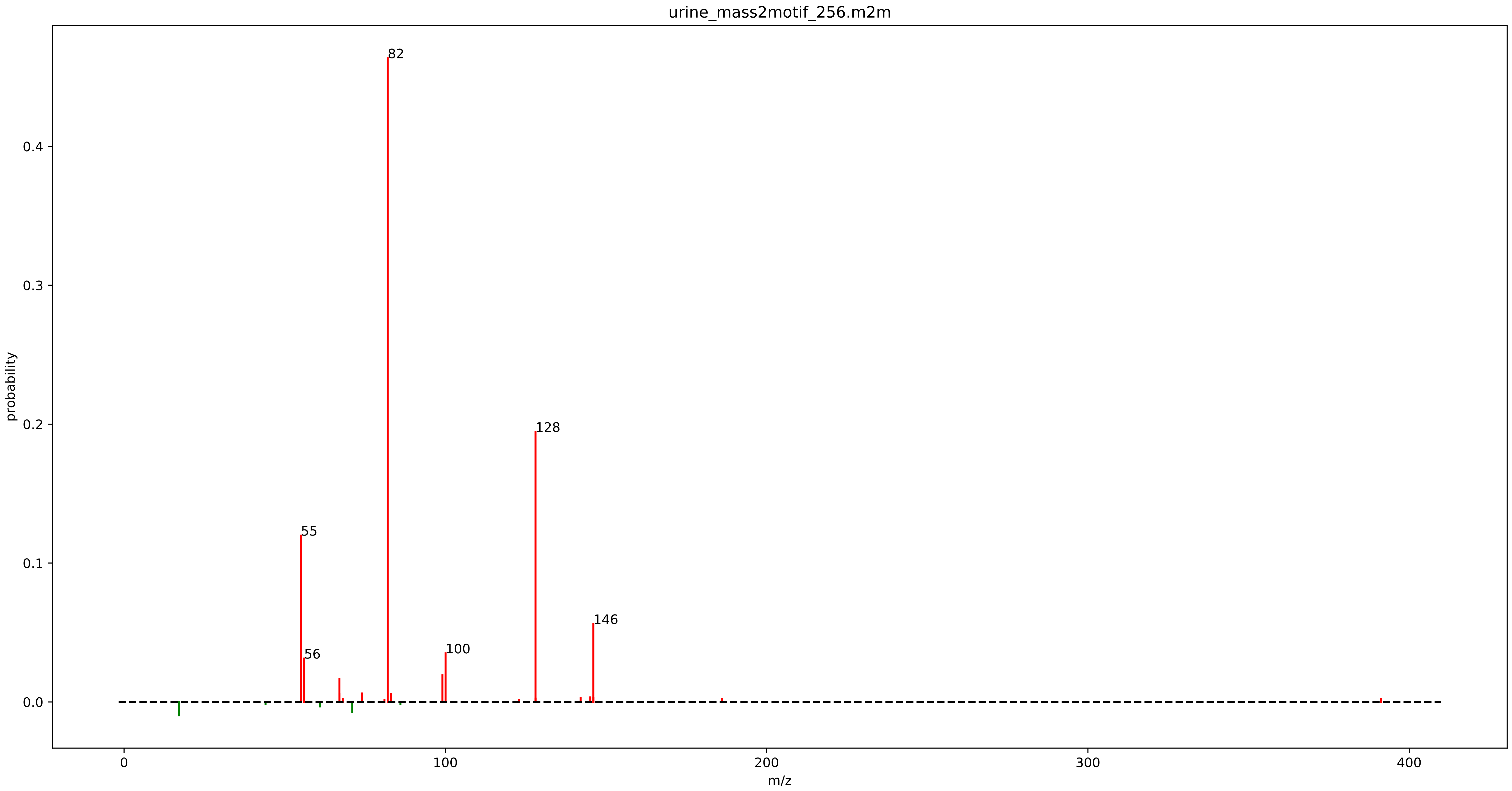
urine_mass2motif_256.m2m
probability
m/z
82
128
55
146
100
56
0.0
0.1
0.2
0.3
0.4
0
100
200
300
400

urine_mass2motif_256.m2m

fragment_82.0675	: 0.46
fragment_128.0725	: 0.19
fragment_55.0525	: 0.12
fragment_146.0825	: 0.06
fragment_100.0775	: 0.03
fragment_56.0475	: 0.03
fragment_99.0925	: 0.02
fragment_67.0425	: 0.02
loss_17.0275	: 0.01
loss_71.0275	: 0.01
fragment_74.0225	: 0.01
fragment_83.0725	: 0.01
fragment_67.0525	: 0.01

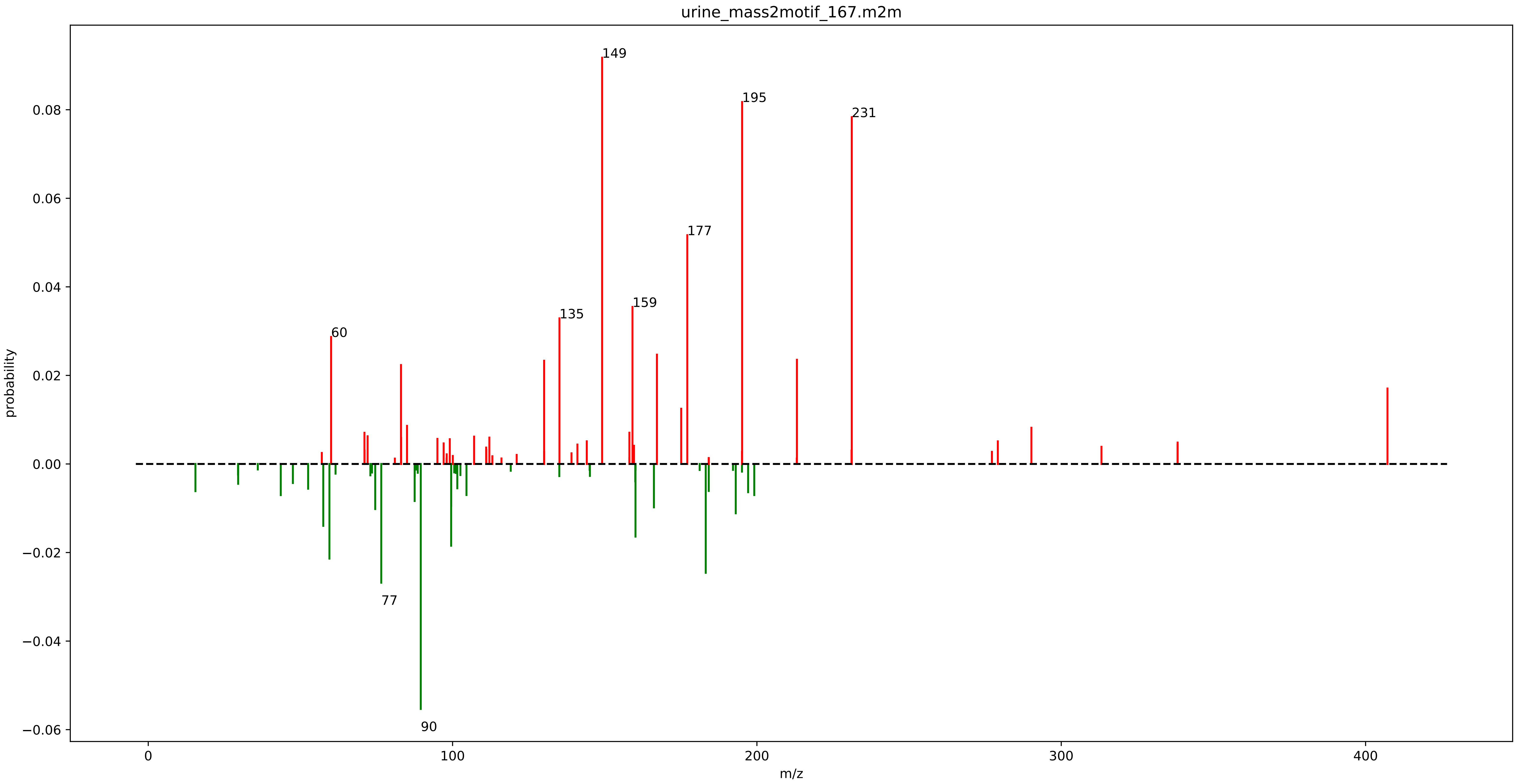

urine_mass2motif_167.m2m
probability
m/z
149
195
231
177
135
159
60
77
90
0.08
0.06
0.04
0.02
0.00
−0.02
−0.04
−0.06
0
100
200
300
400

urine_mass2motif_167.m2m

fragment_149.1325	: 0.09
fragment_195.1375	: 0.08
fragment_231.1625	: 0.08
loss_89.5475	: 0.06
fragment_177.1275	: 0.05
fragment_159.1175	: 0.04
fragment_135.1175	: 0.03
fragment_60.0825	: 0.03
loss_76.5625	: 0.03
fragment_167.1425	: 0.02
loss_183.1875	: 0.02
fragment_213.1475	: 0.02
fragment_130.0775	: 0.02
fragment_83.0475	: 0.02
loss_59.5325	: 0.02
loss_99.5275	: 0.02
fragment_407.1925	: 0.02
loss_160.1025	: 0.02
loss_57.5175	: 0.01
fragment_175.1225	: 0.01
loss_193.0475	: 0.01
loss_74.5825	: 0.01
loss_166.1575	: 0.01
loss_99.5525	: 0.01
fragment_85.0275	: 0.01
loss_87.5275	: 0.01
fragment_290.1975	: 0.01
fragment_158.0975	: 0.01
fragment_71.0475	: 0.01
loss_43.5425	: 0.01
loss_199.1275	: 0.01
loss_104.5575	: 0.01
loss_197.1075	: 0.01
fragment_72.0725	: 0.01
fragment_107.0725	: 0.01
loss_15.5075	: 0.01
loss_184.1725	: 0.01
fragment_112.0875	: 0.01
fragment_83.0875	: 0.01
fragment_95.0125	: 0.01
fragment_99.0825	: 0.01
loss_52.5375	: 0.01
loss_101.5475	: 0.01
fragment_144.0925	: 0.01
fragment_279.1575	: 0.01

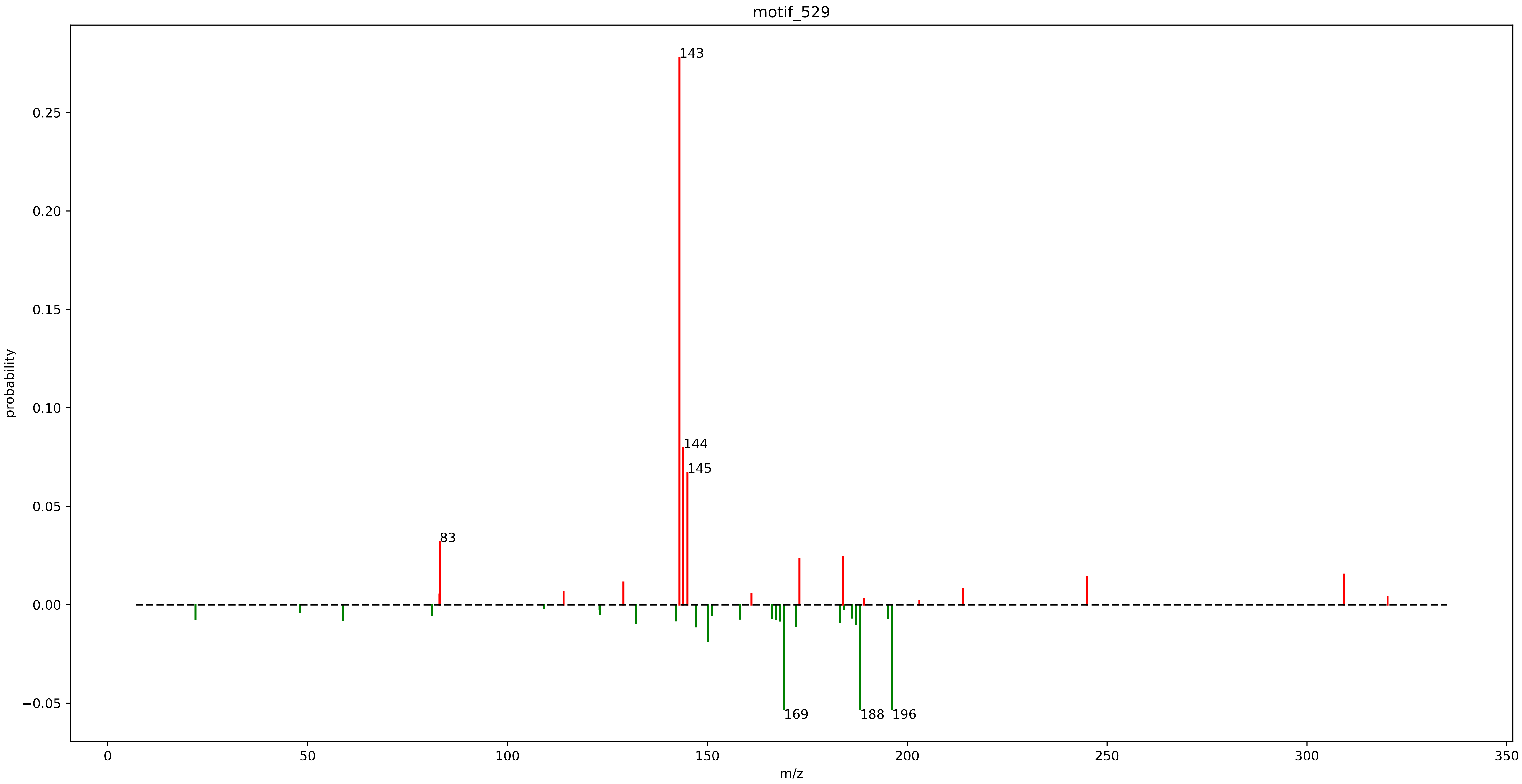
motif_529
143
144
145
83
169
188
196
probability
m/z
0.25
0.20
0.15
0.10
0.05
0.00
−0.05
0
50
100
150
200
250
300
350

motif_529

fragment_143.0175	: 0.28
fragment_144.0225	: 0.08
fragment_145.0175	: 0.07
loss_188.1875	: 0.05
loss_196.1825	: 0.05
loss_169.1725	: 0.05
fragment_83.0475	: 0.03
fragment_184.0425	: 0.02
fragment_173.0275	: 0.02
loss_150.1475	: 0.02
fragment_309.2575	: 0.02
fragment_245.0475	: 0.01
fragment_129.0025	: 0.01
loss_147.1575	: 0.01
loss_172.1525	: 0.01
loss_187.1825	: 0.01
loss_132.1375	: 0.01
loss_183.1675	: 0.01
loss_183.1525	: 0.01
loss_168.1725	: 0.01
fragment_214.0575	: 0.01
loss_142.1425	: 0.01
loss_58.9225	: 0.01
loss_21.9475	: 0.01
loss_167.1775	: 0.01
loss_158.1775	: 0.01
loss_166.1775	: 0.01
loss_195.1825	: 0.01
loss_186.1875	: 0.01
fragment_114.0525	: 0.01
fragment_161.0275	: 0.01
loss_151.1625	: 0.01
fragment_82.9975	: 0.01
loss_81.1225	: 0.01

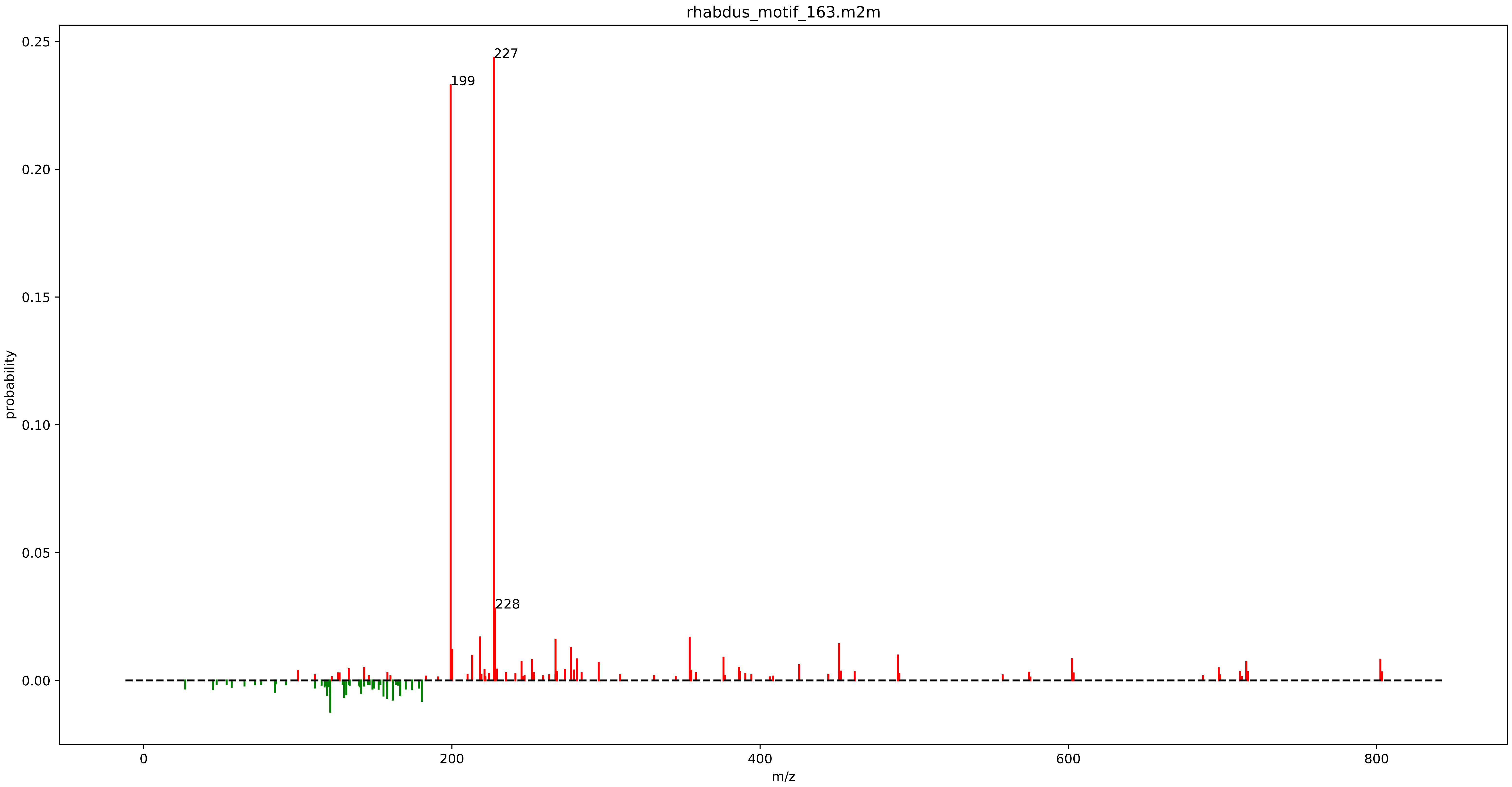

rhabdus_motif_163.m2m
probability
m/z
0.25
0.20
0.15
0.10
0.05
0.00
0
200
400
600
800
199
227
228

rhabdus_motif_163.m2m

fragment_227.1775	: 0.24
fragment_199.1775	: 0.23
fragment_228.1775	: 0.03
fragment_218.1525	: 0.02
fragment_354.2775	: 0.02
fragment_267.2325	: 0.02
fragment_451.3275	: 0.01
fragment_277.1875	: 0.01
loss_121.0875	: 0.01
fragment_200.1825	: 0.01
fragment_489.3075	: 0.01
fragment_213.1725	: 0.01
fragment_376.2225	: 0.01
fragment_602.3925	: 0.01
fragment_281.2225	: 0.01
loss_180.4775	: 0.01
fragment_252.1125	: 0.01
fragment_802.5025	: 0.01
loss_161.6175	: 0.01
fragment_245.1825	: 0.01
fragment_715.4775	: 0.01
fragment_295.2375	: 0.01
loss_158.1025	: 0.01
loss_130.1075	: 0.01
fragment_425.3475	: 0.01
loss_155.6275	: 0.01
loss_166.4625	: 0.01
loss_119.0575	: 0.01
loss_131.4775	: 0.01

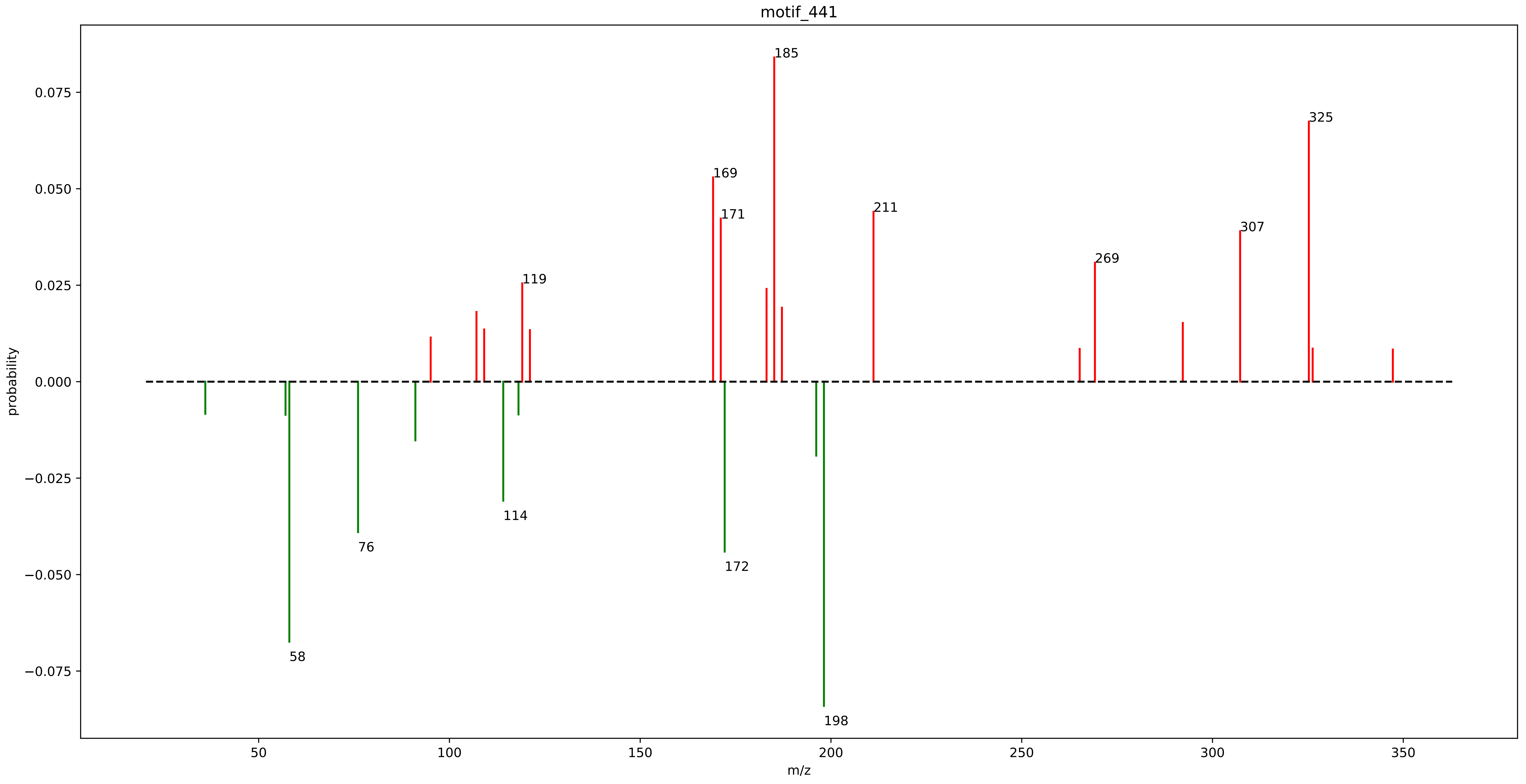

motif_441
probability
m/z
0.075
0.050
0.025
0.000
−0.025
−0.050
−0.075
50
100
150
200
250
300
350
185
325
169
171
211
307
269
119
114
76
58
172
198

motif_441

loss_198.1575	: 0.08
fragment_185.1325	: 0.08
fragment_325.2525	: 0.07
loss_58.0375	: 0.07
fragment_169.1025	: 0.05
loss_172.1425	: 0.04
fragment_211.1475	: 0.04
fragment_171.1175	: 0.04
fragment_307.2425	: 0.04
loss_76.0525	: 0.04
fragment_269.1875	: 0.03
loss_114.1075	: 0.03
fragment_119.0825	: 0.03
fragment_183.1175	: 0.02
loss_196.1425	: 0.02
fragment_187.1475	: 0.02
fragment_107.0825	: 0.02
fragment_292.2175	: 0.02
loss_91.0725	: 0.02
fragment_109.1025	: 0.01
fragment_121.0975	: 0.01
fragment_95.0825	: 0.01
loss_57.0375	: 0.01
fragment_326.2575	: 0.01
loss_118.0975	: 0.01
fragment_265.1925	: 0.01
loss_36.0175	: 0.01
fragment_347.2725	: 0.01

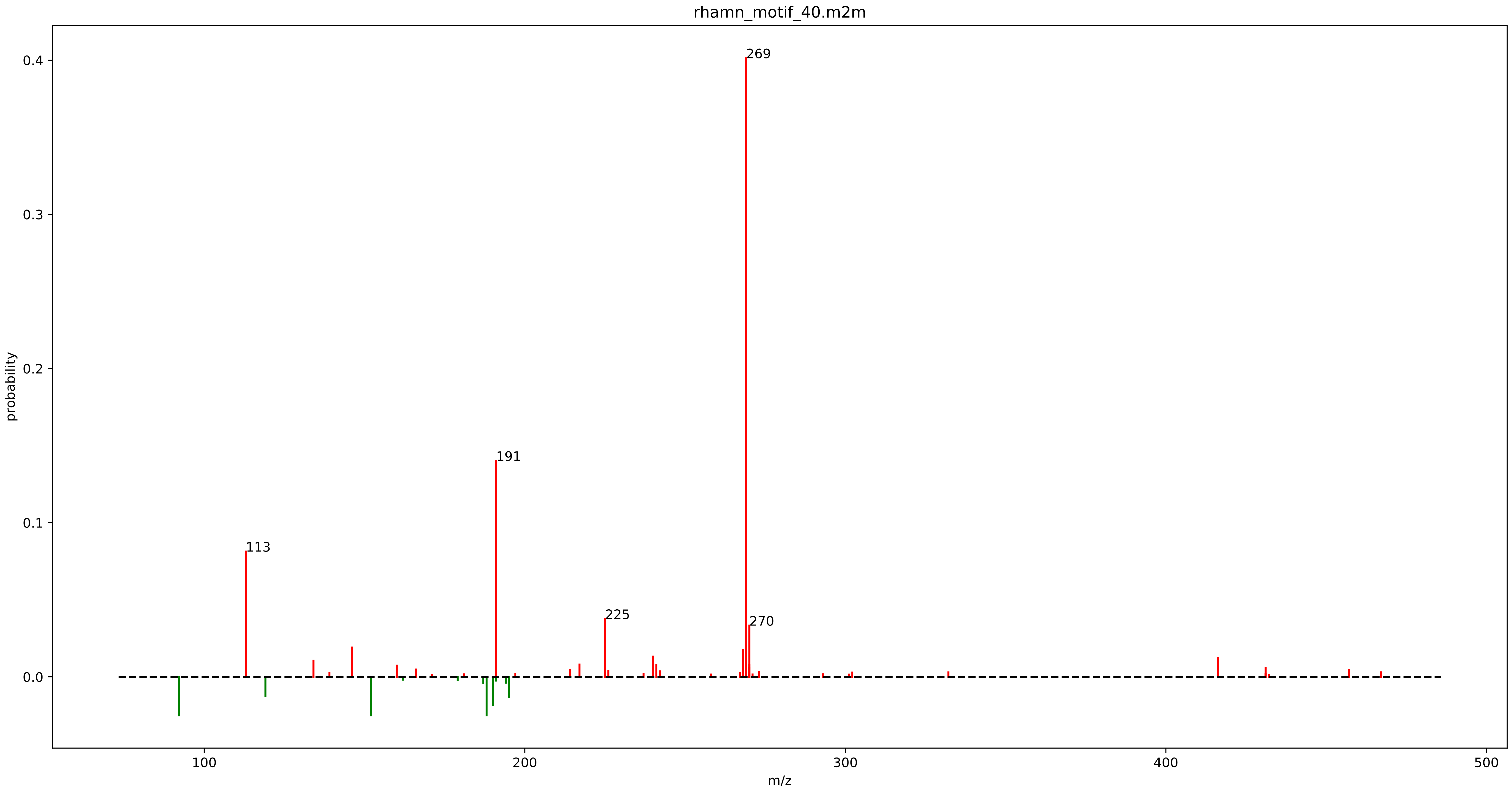
rhamn_motif_40.m2m
probability
m/z
0.4
0.3
0.2
0.1
0.0
100
200
300
400
500
269
191
113
225
270

rhamn_motif_40.m2m

fragment_269.0425	: 0.40
fragment_191.0825	: 0.14
fragment_112.9825	: 0.08
fragment_225.0525	: 0.04
fragment_270.0475	: 0.03
loss_151.9525	: 0.02
loss_92.0475	: 0.02
loss_188.0725	: 0.02
fragment_146.0625	: 0.02
loss_190.0475	: 0.02
fragment_268.0375	: 0.02
fragment_240.0425	: 0.01
loss_195.0875	: 0.01
fragment_416.1925	: 0.01
loss_119.0975	: 0.01
fragment_134.0625	: 0.01
fragment_217.0625	: 0.01
fragment_270.0525	: 0.01
fragment_241.0475	: 0.01
fragment_160.0325	: 0.01
fragment_431.1025	: 0.01
fragment_270.0425	: 0.01

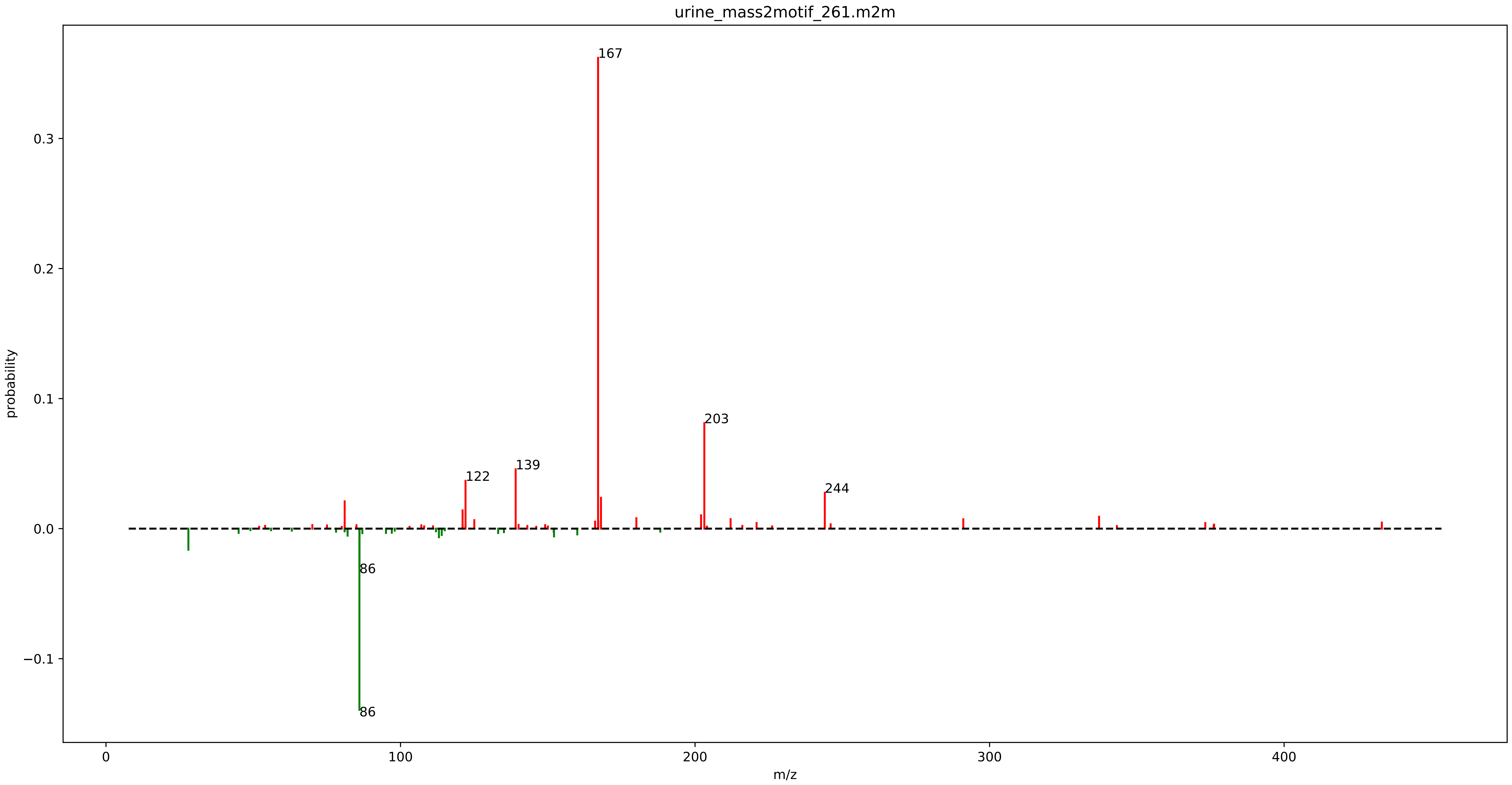
urine_mass2motif_261.m2m
probability
m/z
167
203
139
122
244
86
86
0.3
0.2
0.1
0.0
−0.1
0
100
200
300
400

urine_mass2motif_261.m2m

fragment_167.0775	: 0.36
loss_86.0475	: 0.14
fragment_203.1525	: 0.08
fragment_139.0975	: 0.05
fragment_122.0725	: 0.04
loss_86.0725	: 0.03
fragment_244.0525	: 0.03
fragment_168.0625	: 0.02
fragment_81.0425	: 0.02
loss_27.9825	: 0.02
fragment_121.0775	: 0.01
fragment_202.0425	: 0.01
fragment_337.1725	: 0.01
fragment_180.0775	: 0.01
fragment_212.0725	: 0.01
fragment_291.0775	: 0.01
loss_113.0375	: 0.01
fragment_125.0525	: 0.01
loss_152.1175	: 0.01
fragment_166.0675	: 0.01
fragment_167.1525	: 0.01
loss_82.0325	: 0.01

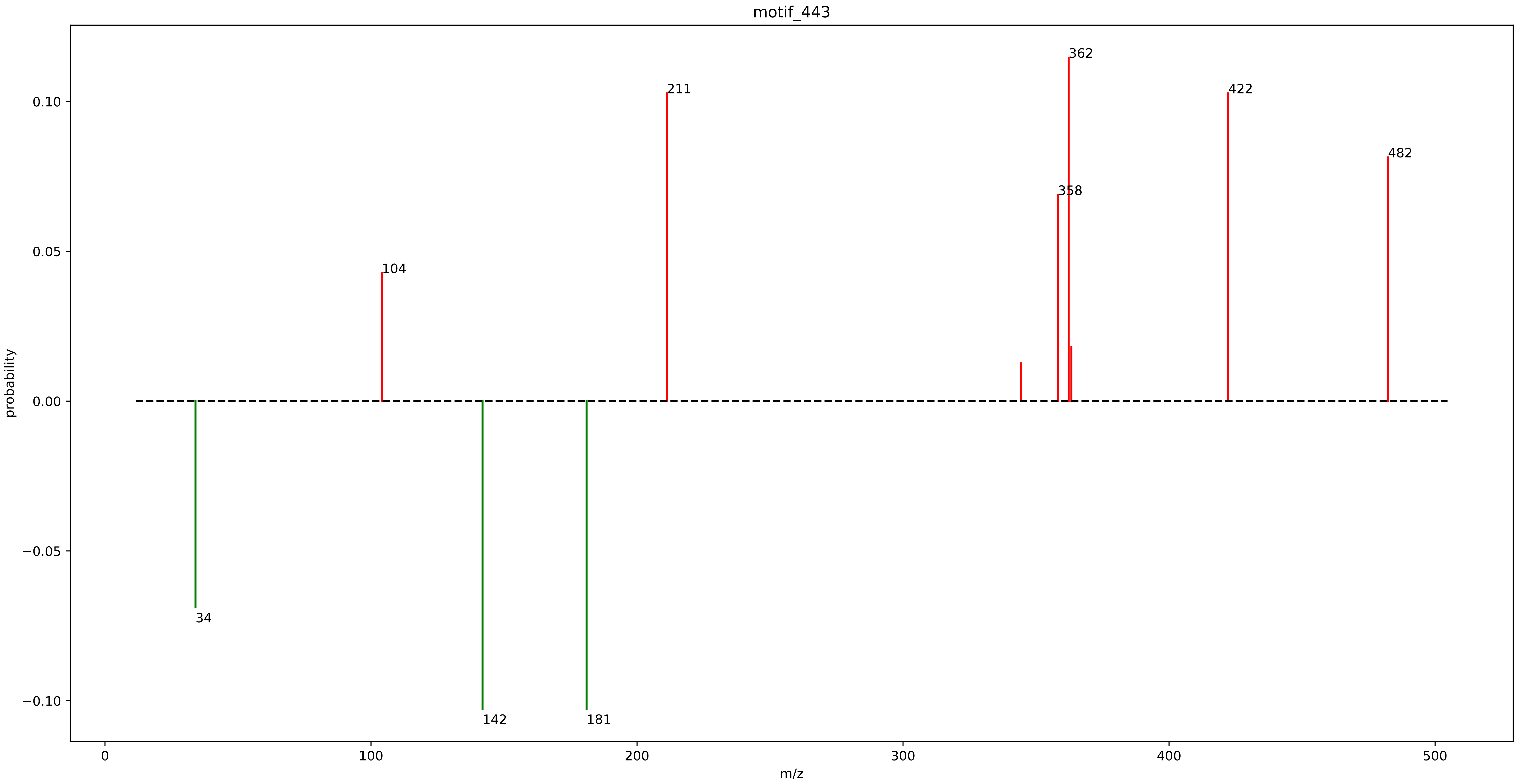

motif_443
probability
m/z
0.10
0.05
0.00
−0.05
−0.10
0
100
200
300
400
500
104
211
362
358
422
482
34
142
181

motif_443

fragment_362.2675	: 0.11
loss_181.0325	: 0.10
fragment_211.2075	: 0.10
loss_141.9275	: 0.10
fragment_422.2775	: 0.10
fragment_482.2875	: 0.08
loss_34.0225	: 0.07
fragment_358.2125	: 0.07
fragment_104.0675	: 0.04
fragment_363.2725	: 0.02
fragment_344.2575	: 0.01

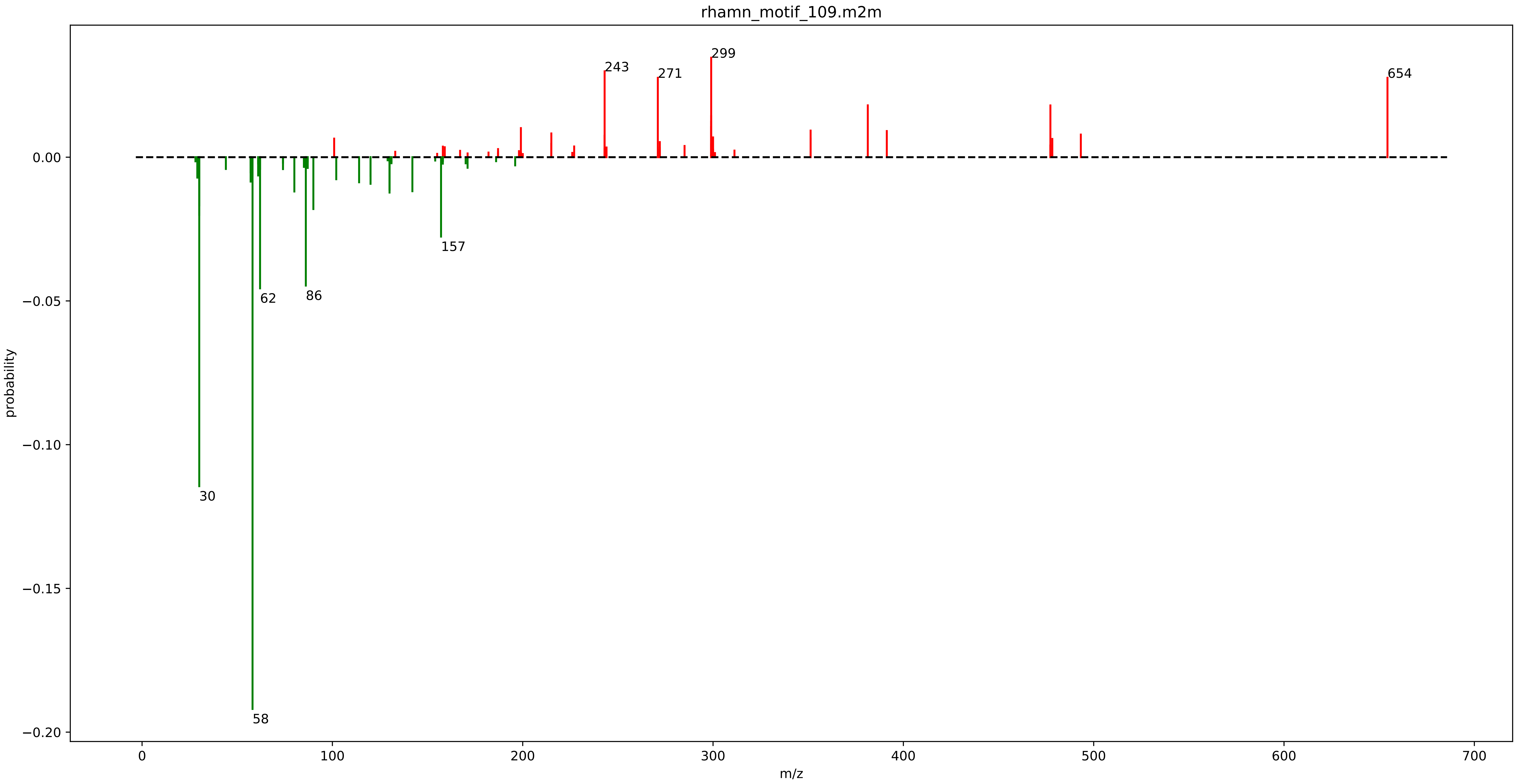

rhamn_motif_109.m2m
probability
m/z
0.00
−0.05
−0.10
−0.15
−0.20
0
100
200
300
400
500
600
700
30
58
62
86
157
243
271
299
654

rhamn_motif_109.m2m

loss_58.0425	: 0.19
loss_30.0475	: 0.11
loss_61.9975	: 0.05
loss_86.0375	: 0.04
fragment_299.0225	: 0.03
fragment_243.0275	: 0.03
loss_157.0875	: 0.03
fragment_270.9875	: 0.03
fragment_654.3575	: 0.03
loss_30.0425	: 0.02
loss_90.0025	: 0.02
fragment_381.3075	: 0.02
fragment_477.2525	: 0.02
loss_30.0525	: 0.02
fragment_299.0175	: 0.01
fragment_299.0125	: 0.01
loss_130.0225	: 0.01
loss_80.0125	: 0.01
loss_142.0275	: 0.01
loss_130.0275	: 0.01
fragment_298.9875	: 0.01
fragment_199.0425	: 0.01
loss_120.0475	: 0.01
fragment_351.2625	: 0.01
fragment_391.2975	: 0.01
loss_114.0325	: 0.01
loss_57.0425	: 0.01
fragment_215.0375	: 0.01
fragment_493.2425	: 0.01
loss_102.0325	: 0.01
fragment_242.9925	: 0.01
loss_29.0425	: 0.01
loss_29.0475	: 0.01
fragment_300.0175	: 0.01
loss_120.0325	: 0.01
fragment_351.2775	: 0.01
fragment_100.9275	: 0.01
fragment_478.2575	: 0.01
loss_60.9925	: 0.01
loss_57.0325	: 0.01
fragment_272.0375	: 0.01
loss_86.0325	: 0.01

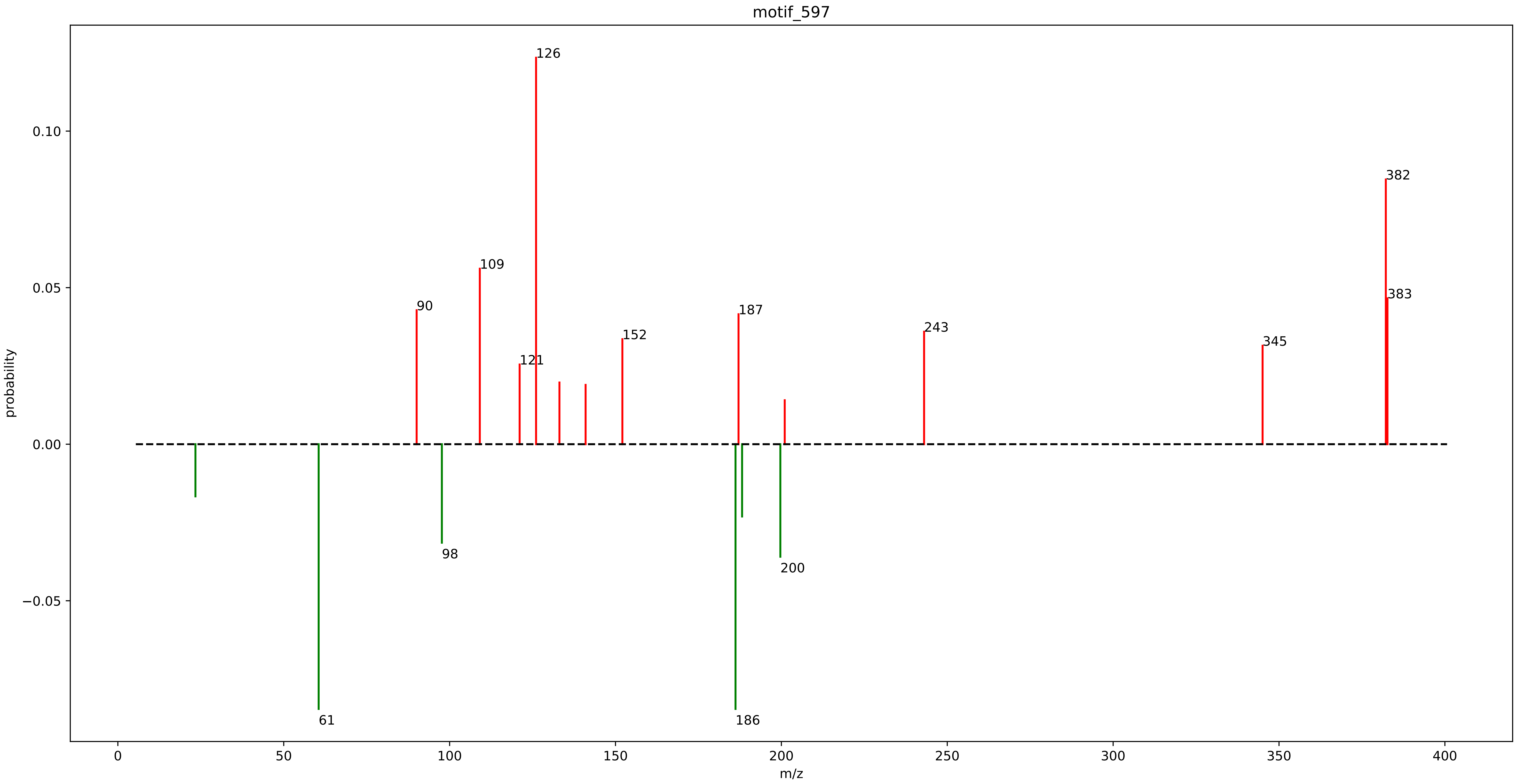

motif_597
126
382
109
383
90
187
152
243
345
121
98
200
61
186
0.10
0.05
0.00
−0.05
probability
0
50
100
150
200
250
300
350
400
m/z

motif_597

fragment_126.0575	: 0.12
fragment_382.1925	: 0.08
loss_60.5225	: 0.08
loss_186.1775	: 0.08
fragment_109.0975	: 0.06
fragment_382.6925	: 0.05
fragment_90.0575	: 0.04
fragment_187.0825	: 0.04
loss_199.6925	: 0.04
fragment_243.0175	: 0.04
fragment_152.0725	: 0.03
loss_97.6625	: 0.03
fragment_345.0475	: 0.03
fragment_121.1025	: 0.03
loss_188.1575	: 0.02
fragment_133.1025	: 0.02
fragment_140.9875	: 0.02
loss_23.3825	: 0.02
fragment_201.0075	: 0.01

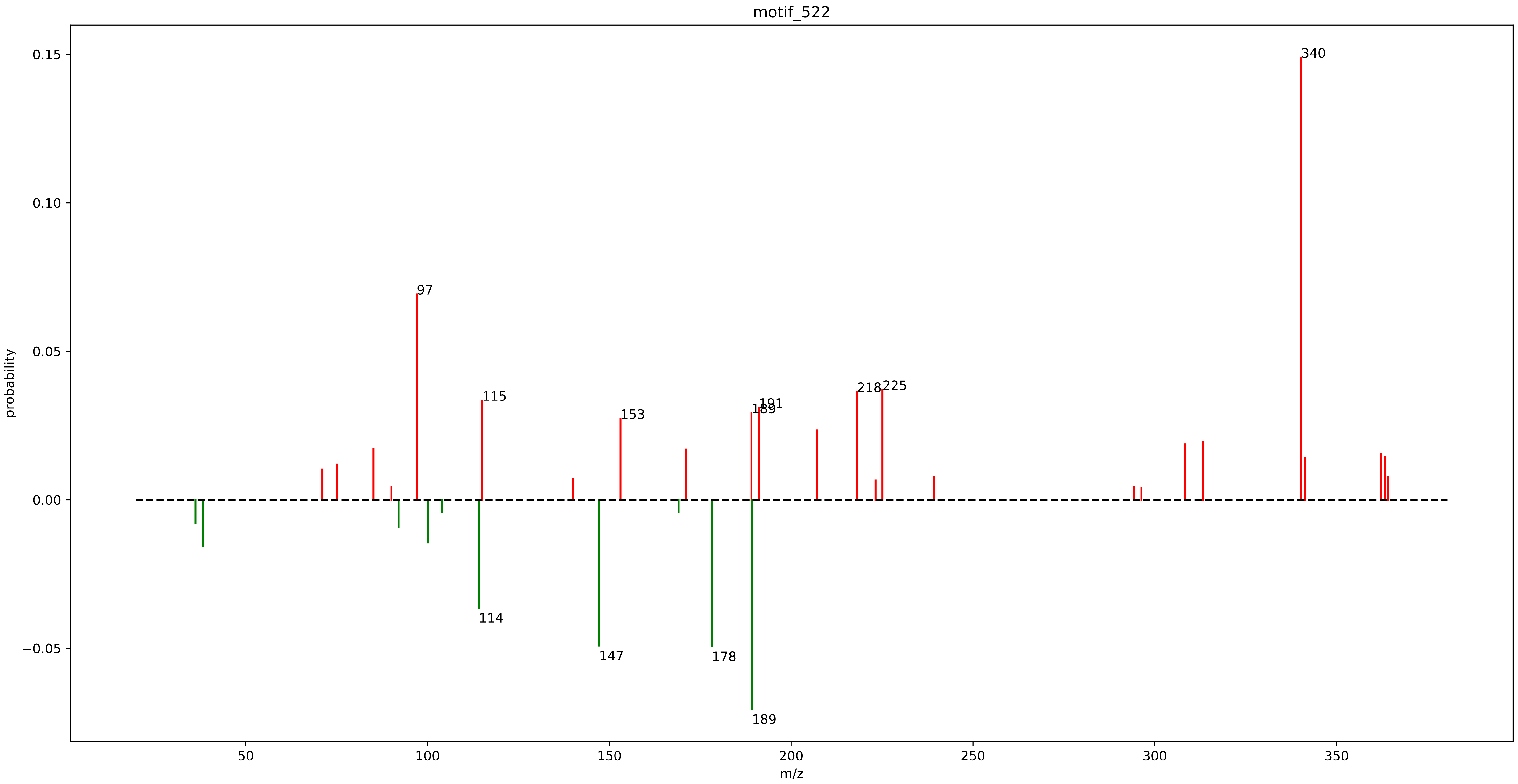

motif_522
probability
m/z
0.15
0.10
0.05
0.00
−0.05
50
100
150
200
250
300
350
340
97
115
153
189
191
218
225
114
147
178
189

motif_522

fragment_340.2825	: 0.15
loss_189.2025	: 0.07
fragment_97.0275	: 0.07
loss_178.1725	: 0.05
loss_147.1925	: 0.05
fragment_225.0975	: 0.04
loss_114.1025	: 0.04
fragment_218.1175	: 0.04
fragment_115.0375	: 0.03
fragment_191.0875	: 0.03
fragment_189.0725	: 0.03
fragment_153.0525	: 0.03
fragment_207.0875	: 0.02
fragment_313.3075	: 0.02
fragment_308.2575	: 0.02
fragment_85.1025	: 0.02
fragment_171.0625	: 0.02
fragment_362.1275	: 0.02
loss_38.1775	: 0.02
fragment_363.2625	: 0.01
loss_100.0975	: 0.01
fragment_341.2875	: 0.01
fragment_75.0425	: 0.01
fragment_71.0825	: 0.01
loss_92.0475	: 0.01
fragment_239.2725	: 0.01
fragment_364.1275	: 0.01
loss_36.1775	: 0.01
fragment_140.0475	: 0.01
fragment_223.2075	: 0.01

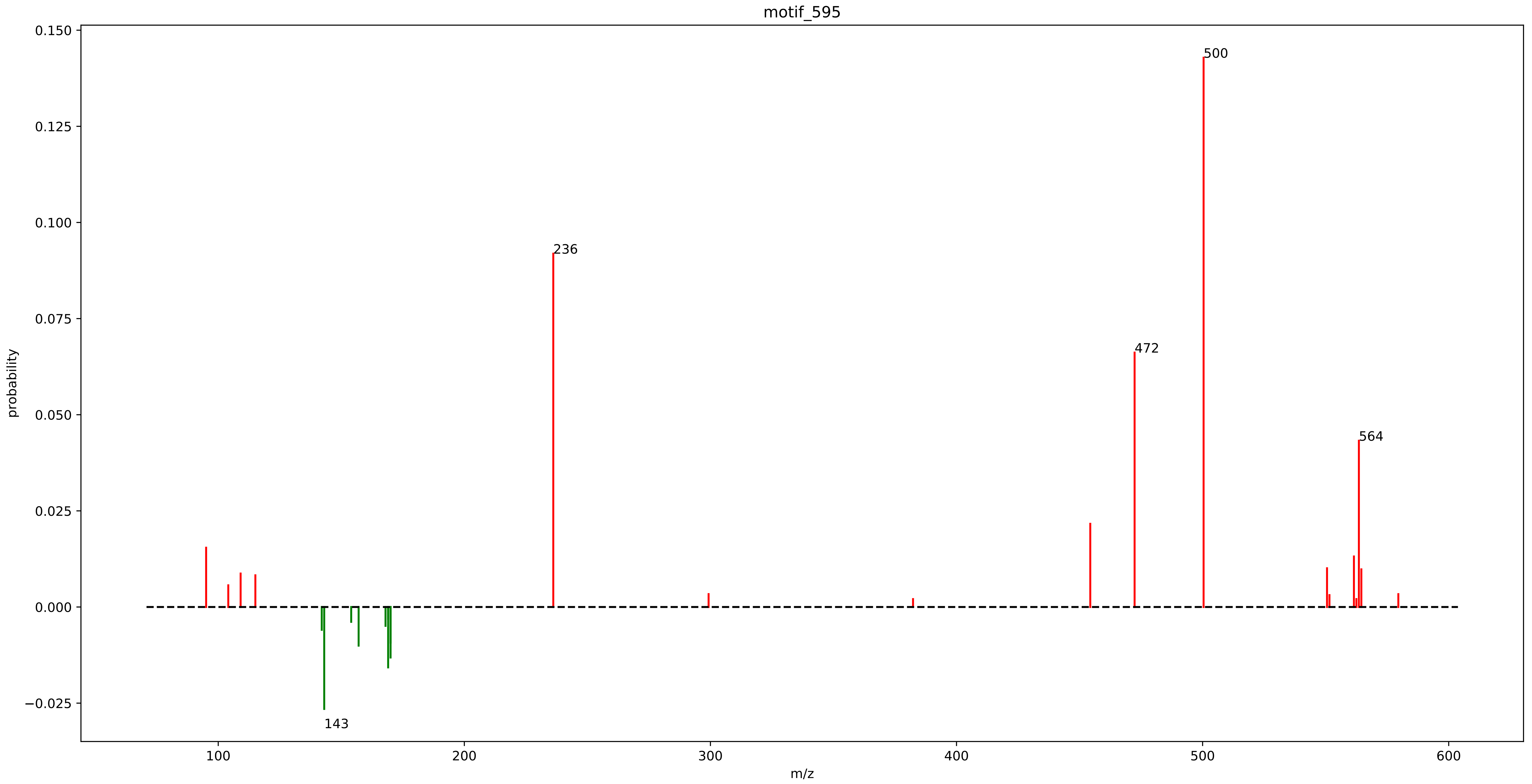
motif_595
probability
m/z
0.150
0.125
0.100
0.075
0.050
0.025
0.000
−0.025
100
200
300
400
500
600
500
236
472
564
143

motif_595

fragment_500.3975	: 0.14
fragment_236.1475	: 0.09
fragment_472.3625	: 0.07
fragment_563.5025	: 0.04
loss_143.0675	: 0.03
fragment_454.3525	: 0.02
loss_169.0425	: 0.02
fragment_95.0825	: 0.02
fragment_561.4875	: 0.01
loss_170.0325	: 0.01
fragment_550.5575	: 0.01
loss_157.0475	: 0.01
fragment_564.5075	: 0.01
loss_170.0275	: 0.01
fragment_109.0975	: 0.01
fragment_115.0875	: 0.01
loss_142.0625	: 0.01
fragment_104.0825	: 0.01

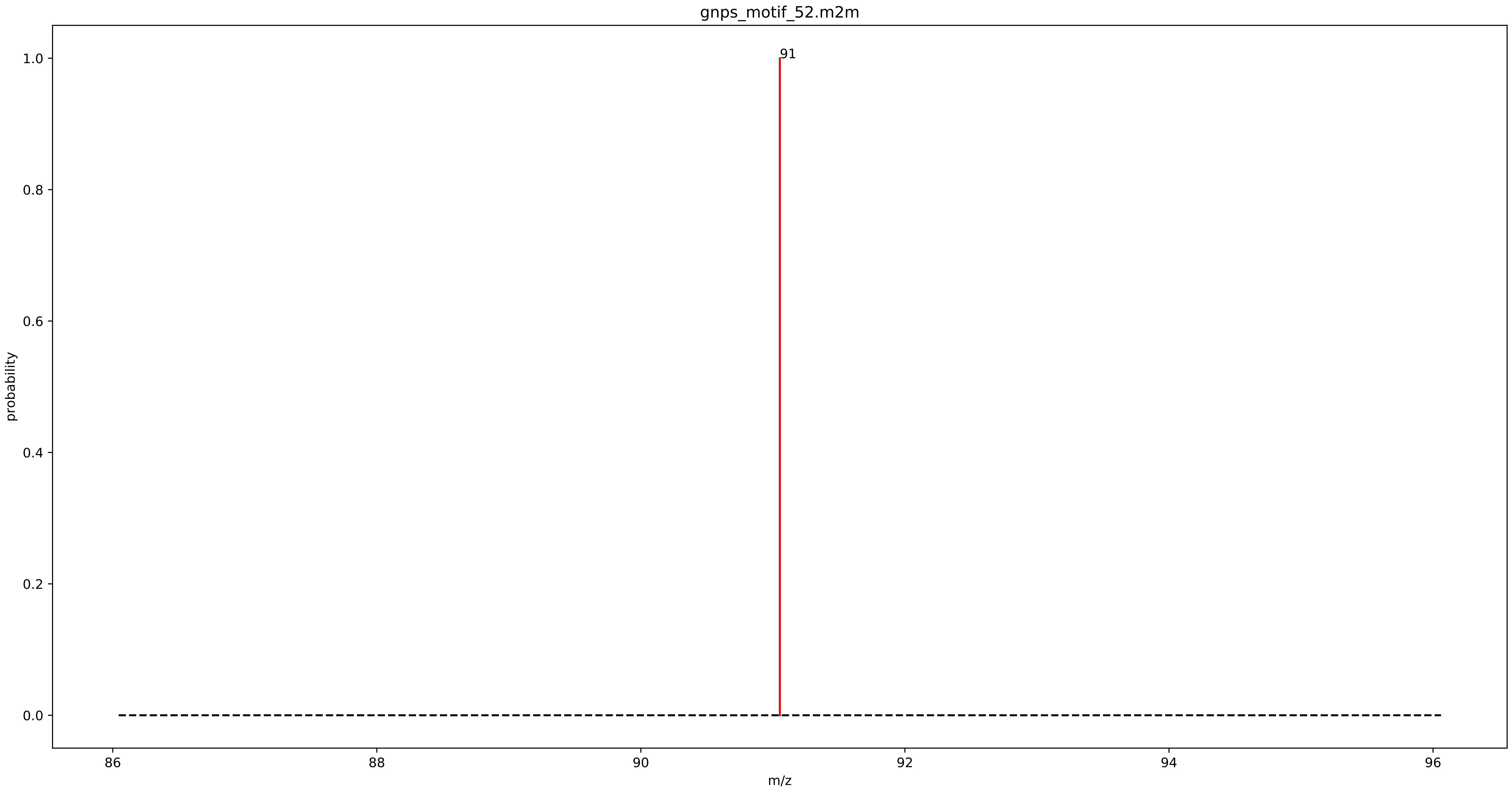

gnps_motif_52.m2m
91
probability
1.0
0.8
0.6
0.4
0.2
0.0
86
88
90
92
94
96
m/z

gnps_motif_52.m2m

fragment_91.0525 : 1.00

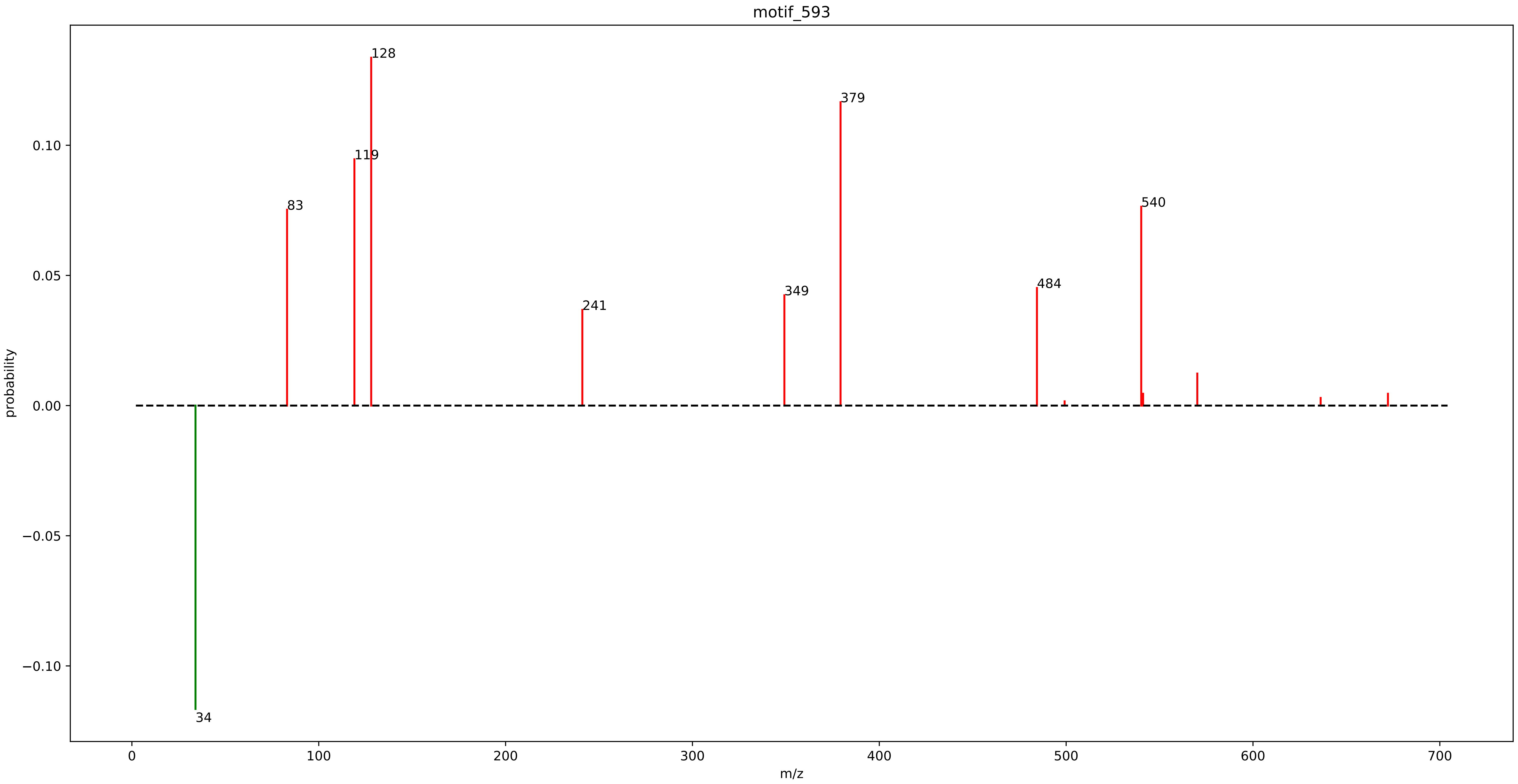

motif_593
128
379
119
83
540
484
349
241
34
probability
0.10
0.05
0.00
−0.05
−0.10
0
100
200
300
400
500
600
700
m/z

motif_593

fragment_128.0725	: 0.13
loss_34.0375	: 0.12
fragment_379.2675	: 0.12
fragment_119.0825	: 0.09
fragment_540.1925	: 0.08
fragment_83.0475	: 0.08
fragment_484.3775	: 0.05
fragment_349.2025	: 0.04
fragment_241.0725	: 0.04
fragment_570.2025	: 0.01

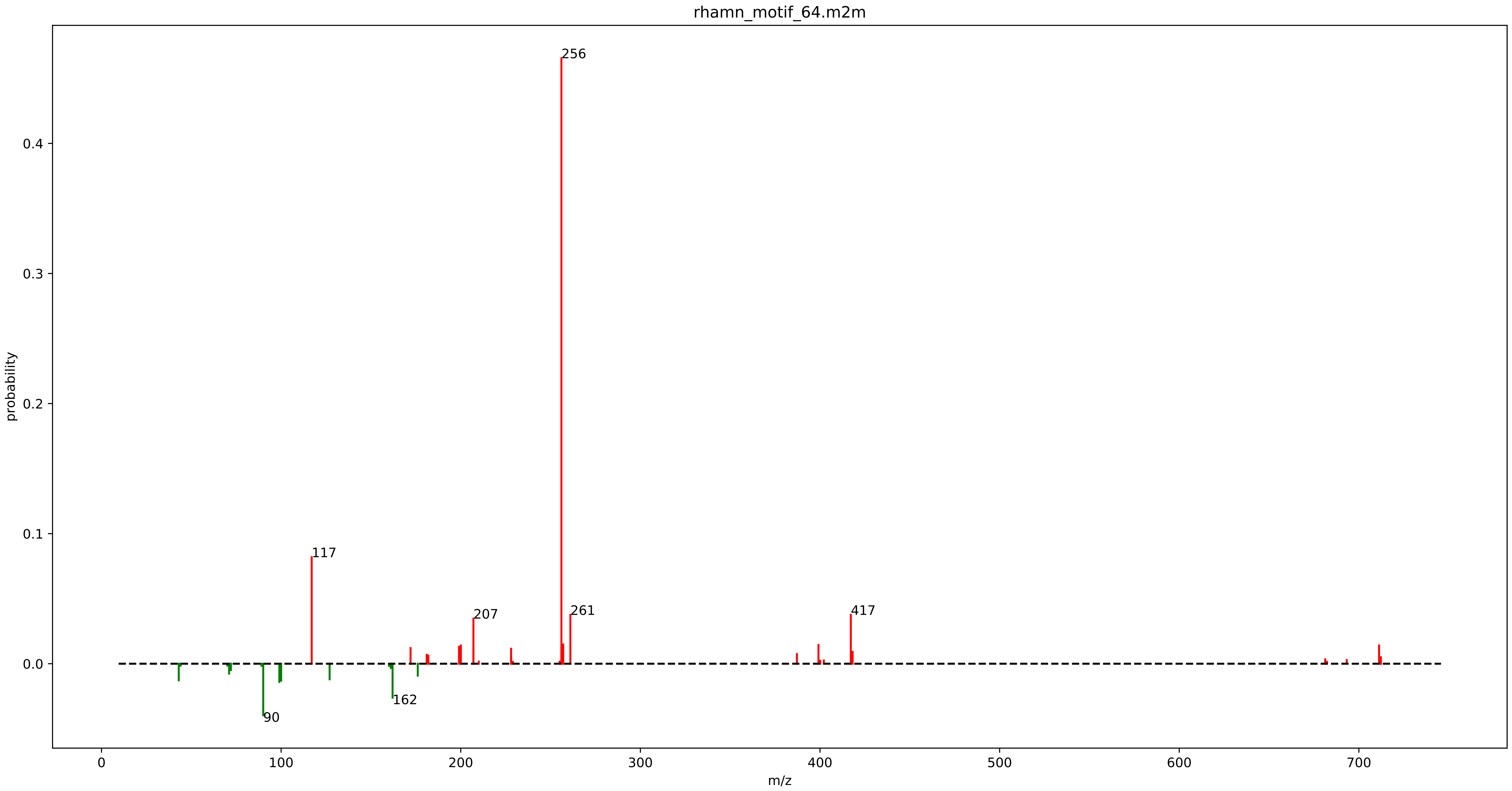

rhamn_motif_64.m2m
probability
m/z
0.4
0.3
0.2
0.1
0.0
0
100
200
300
400
500
600
700
256
117
207
261
417
90
162

rhamn_motif_64.m2m

fragment_256.0375	: 0.47
fragment_117.0175	: 0.08
loss_90.0325	: 0.04
fragment_261.0375	: 0.04
fragment_417.1575	: 0.04
fragment_207.0525	: 0.03
loss_162.0475	: 0.03
fragment_257.0375	: 0.01
fragment_399.1475	: 0.01
fragment_711.2525	: 0.01
loss_98.9975	: 0.01
fragment_200.0575	: 0.01
fragment_199.0325	: 0.01
loss_100.0225	: 0.01
loss_43.0125	: 0.01
fragment_256.0425	: 0.01
fragment_207.0425	: 0.01
loss_126.9975	: 0.01
fragment_172.0575	: 0.01
fragment_228.0375	: 0.01
loss_176.0725	: 0.01
fragment_418.1625	: 0.01
loss_71.0175	: 0.01
fragment_387.1425	: 0.01
fragment_181.0525	: 0.01
fragment_181.9675	: 0.01
fragment_228.0425	: 0.01

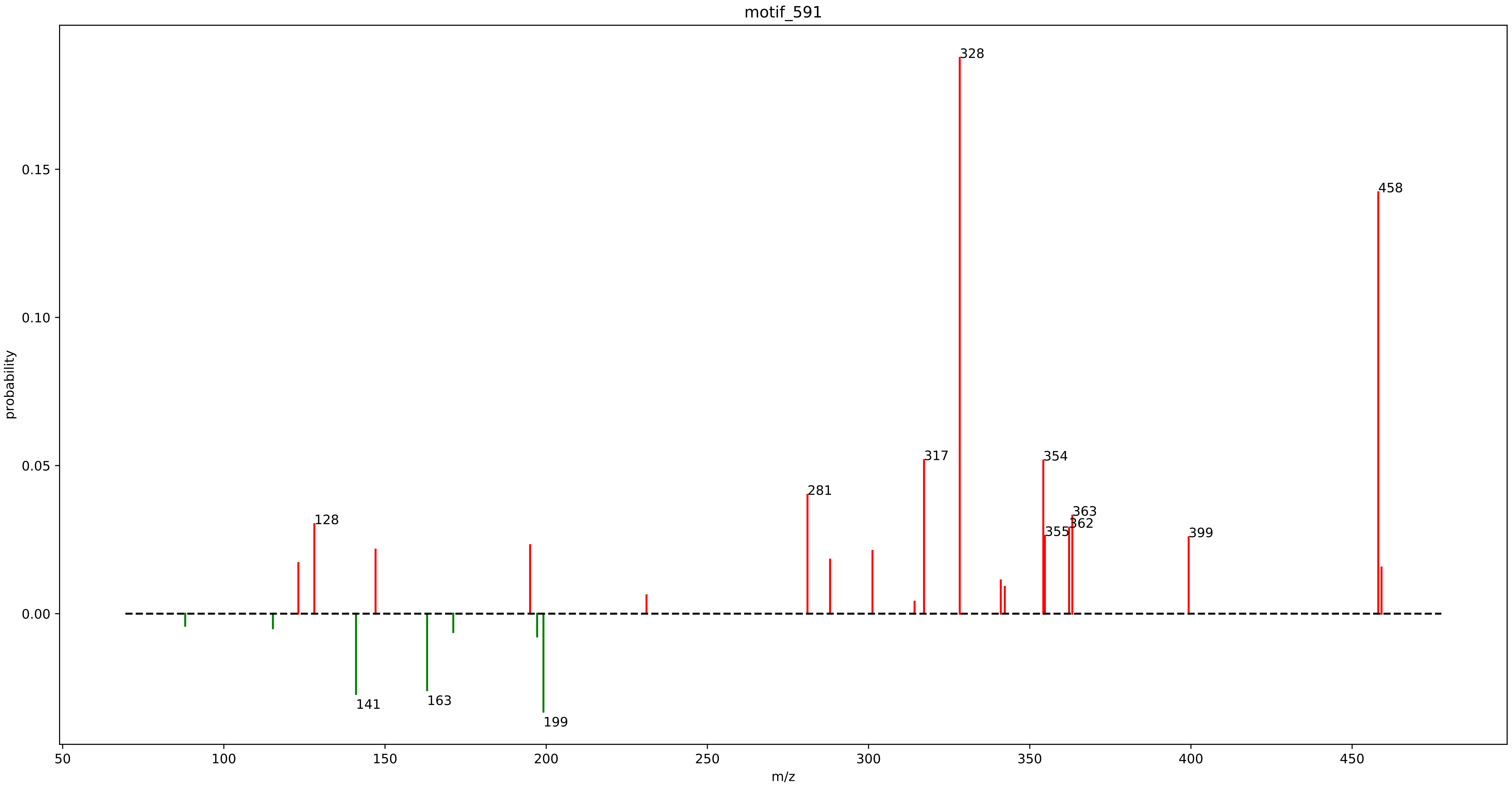
motif_591
probability
m/z
0.15
0.10
0.05
0.00
50
100
150
200
250
300
350
400
450
328
458
317
354
281
363
362
355
399
128
141
163
199

motif_591

fragment_328.2825	: 0.19
fragment_458.1175	: 0.14
fragment_317.2075	: 0.05
fragment_354.2025	: 0.05
fragment_281.0525	: 0.04
loss_199.1425	: 0.03
fragment_363.2075	: 0.03
fragment_128.0725	: 0.03
fragment_362.2025	: 0.03
loss_141.0025	: 0.03
fragment_354.7025	: 0.03
fragment_399.2925	: 0.03
loss_163.0575	: 0.03
fragment_195.0025	: 0.02
fragment_147.0675	: 0.02
fragment_301.2125	: 0.02
fragment_288.0625	: 0.02
fragment_123.1175	: 0.02
fragment_459.1225	: 0.02
fragment_341.0175	: 0.01
fragment_342.2625	: 0.01
loss_197.1875	: 0.01
loss_171.1725	: 0.01
fragment_231.1125	: 0.01

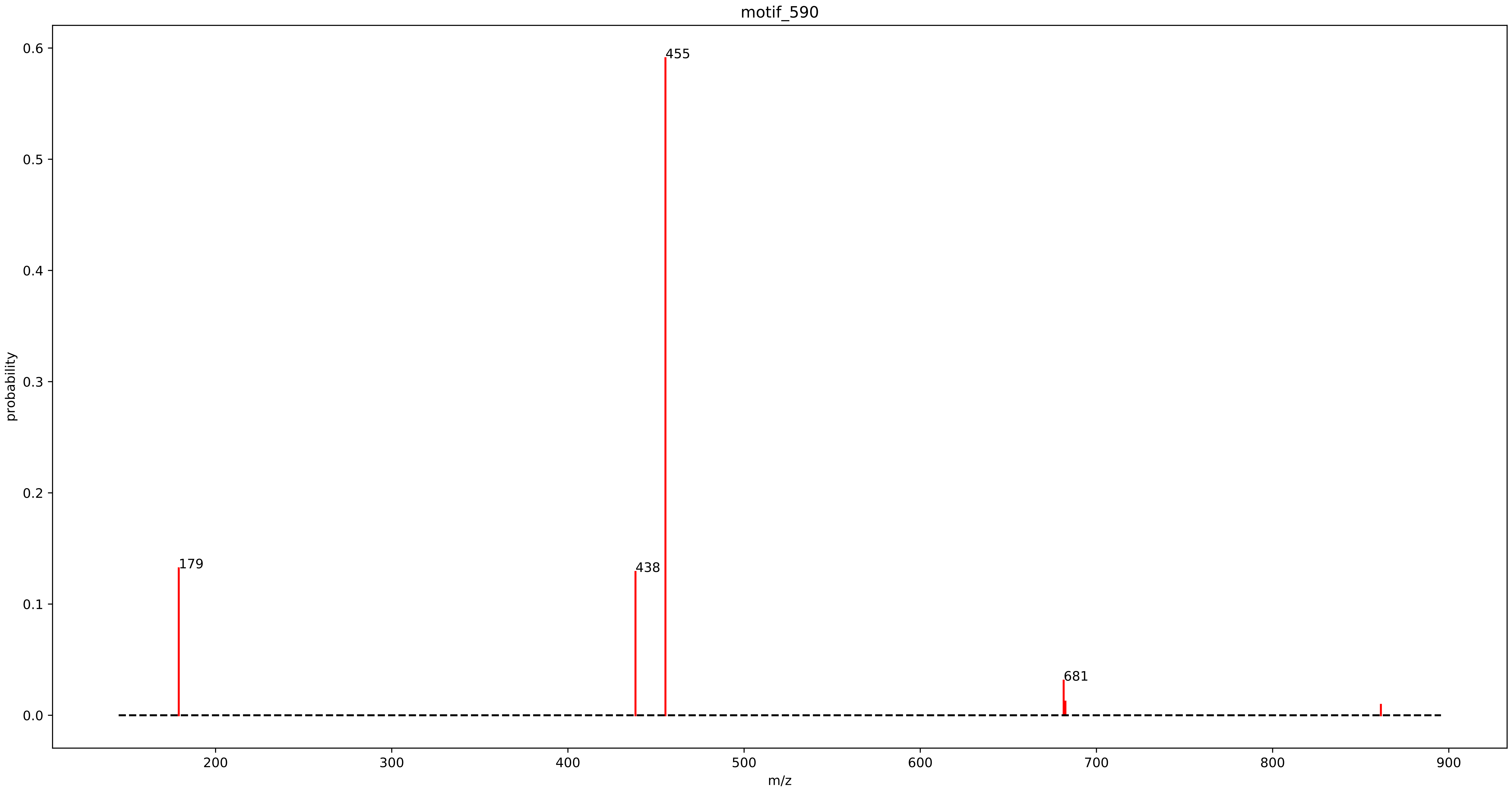

motif_590
probability
m/z
455
179
438
681
0.0
0.1
0.2
0.3
0.4
0.5
0.6
200
300
400
500
600
700
800
900

motif_590

fragment_455.3525	: 0.59
fragment_179.1075	: 0.13
fragment_438.3475	: 0.13
fragment_681.3875	: 0.03
fragment_682.3875	: 0.01
fragment_861.4525	: 0.01

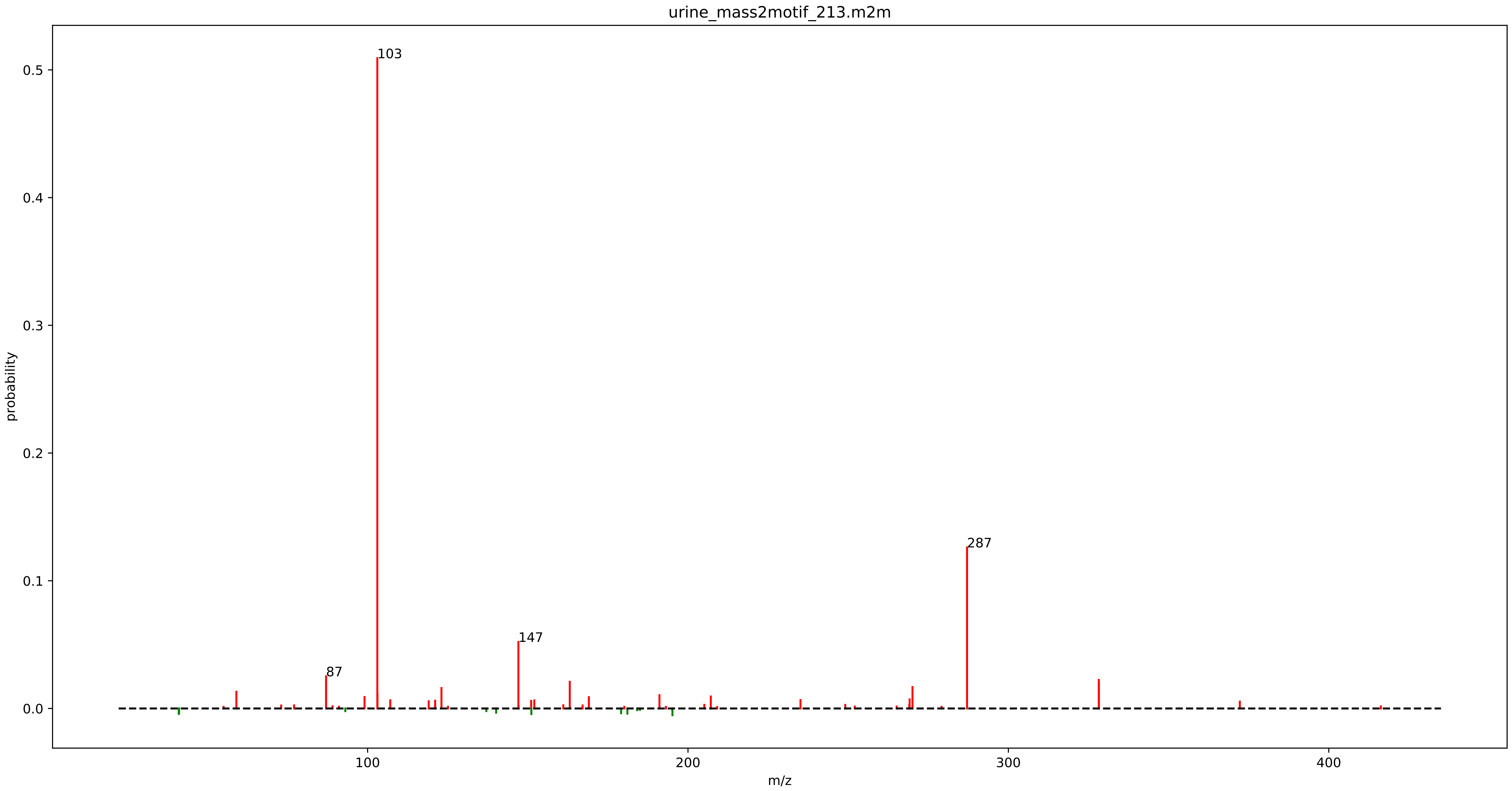

urine_mass2motif_213.m2m
probability
m/z
103
287
147
87
0.0
0.1
0.2
0.3
0.4
0.5
100
200
300
400

urine_mass2motif_213.m2m

fragment_103.0375	: 0.51
fragment_287.1025	: 0.13
fragment_147.0675	: 0.05
fragment_87.0425	: 0.03
fragment_328.2125	: 0.02
fragment_163.0975	: 0.02
fragment_328.2475	: 0.02
fragment_270.0725	: 0.02
fragment_123.0525	: 0.02
fragment_59.0475	: 0.01
fragment_103.0775	: 0.01
fragment_191.0925	: 0.01
fragment_207.1225	: 0.01
fragment_99.0425	: 0.01
fragment_169.0625	: 0.01
fragment_269.1775	: 0.01
fragment_235.1175	: 0.01
fragment_107.0725	: 0.01
fragment_152.0325	: 0.01
fragment_121.0875	: 0.01
fragment_151.0475	: 0.01
fragment_119.0725	: 0.01
fragment_372.2375	: 0.01
loss_195.1475	: 0.01

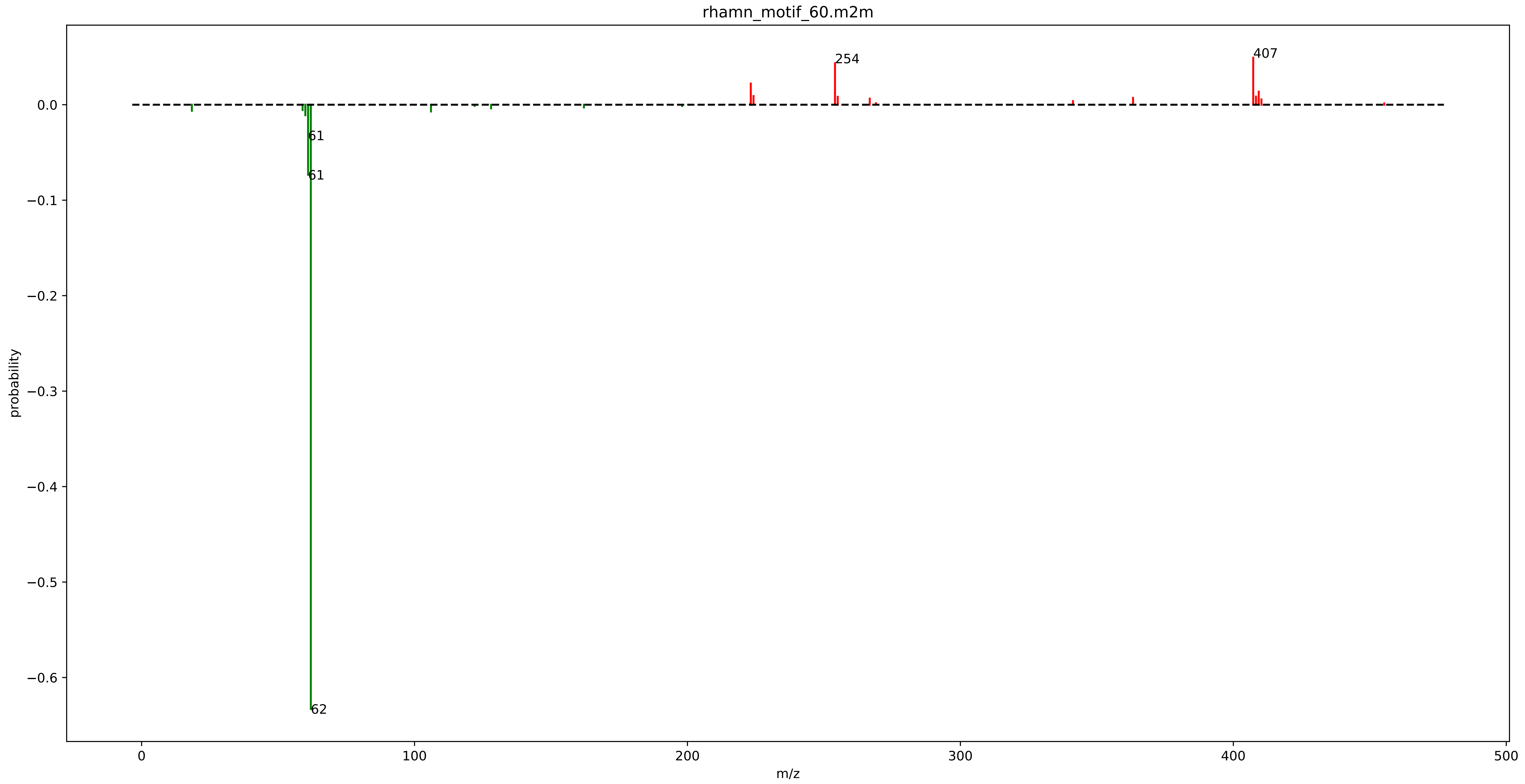

rhamn_motif_60.m2m
probability
m/z
0.0
−0.1
−0.2
−0.3
−0.4
−0.5
−0.6
0
100
200
300
400
500
254
407
61
61
62

rhamn_motif_60.m2m

loss_62.0025	: 0.63
loss_60.9975	: 0.07
fragment_407.2925	: 0.05
fragment_254.0575	: 0.04
loss_60.9925	: 0.03
fragment_223.2025	: 0.02
fragment_409.3075	: 0.01
loss_61.9925	: 0.01
fragment_223.2175	: 0.01
loss_59.9875	: 0.01
fragment_224.2125	: 0.01
fragment_408.3025	: 0.01
fragment_255.0575	: 0.01
loss_106.0275	: 0.01
fragment_363.2675	: 0.01
loss_18.4175	: 0.01
fragment_266.7925	: 0.01
fragment_409.2825	: 0.01
loss_60.0125	: 0.01
loss_58.9875	: 0.01
fragment_410.3075	: 0.01

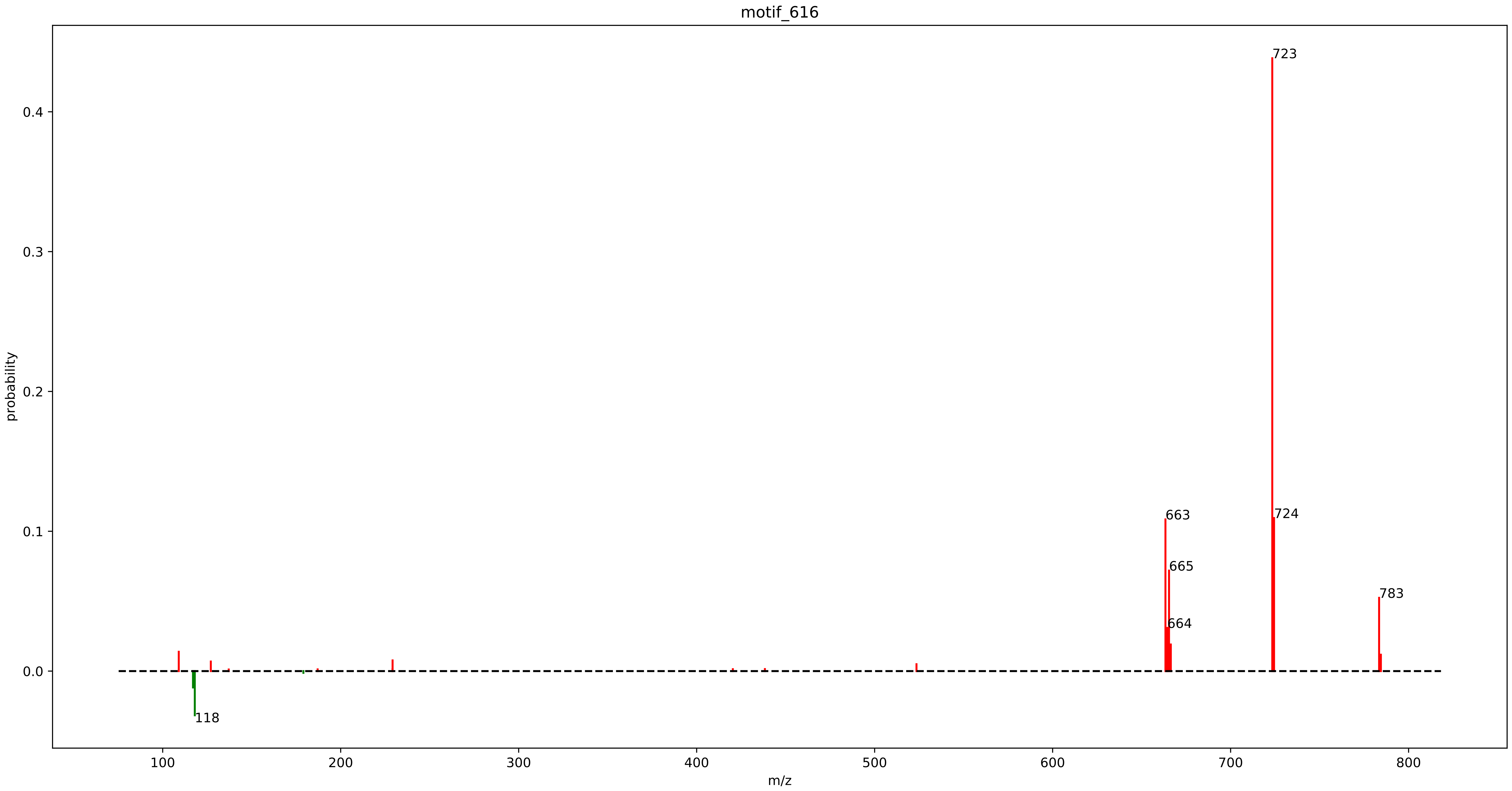

motif_616
probability
m/z
0.4
0.3
0.2
0.1
0.0
100
200
300
400
500
600
700
800
723
663
724
665
783
664
118

motif_616

fragment_723.4075	: 0.44
fragment_724.4125	: 0.11
fragment_663.3875	: 0.11
fragment_665.4025	: 0.07
fragment_783.4275	: 0.05
loss_118.0275	: 0.03
fragment_664.3925	: 0.03
fragment_666.4075	: 0.02
fragment_109.0275	: 0.01
fragment_784.4325	: 0.01
loss_117.0225	: 0.01
fragment_664.3875	: 0.01
fragment_229.1625	: 0.01
fragment_127.0375	: 0.01

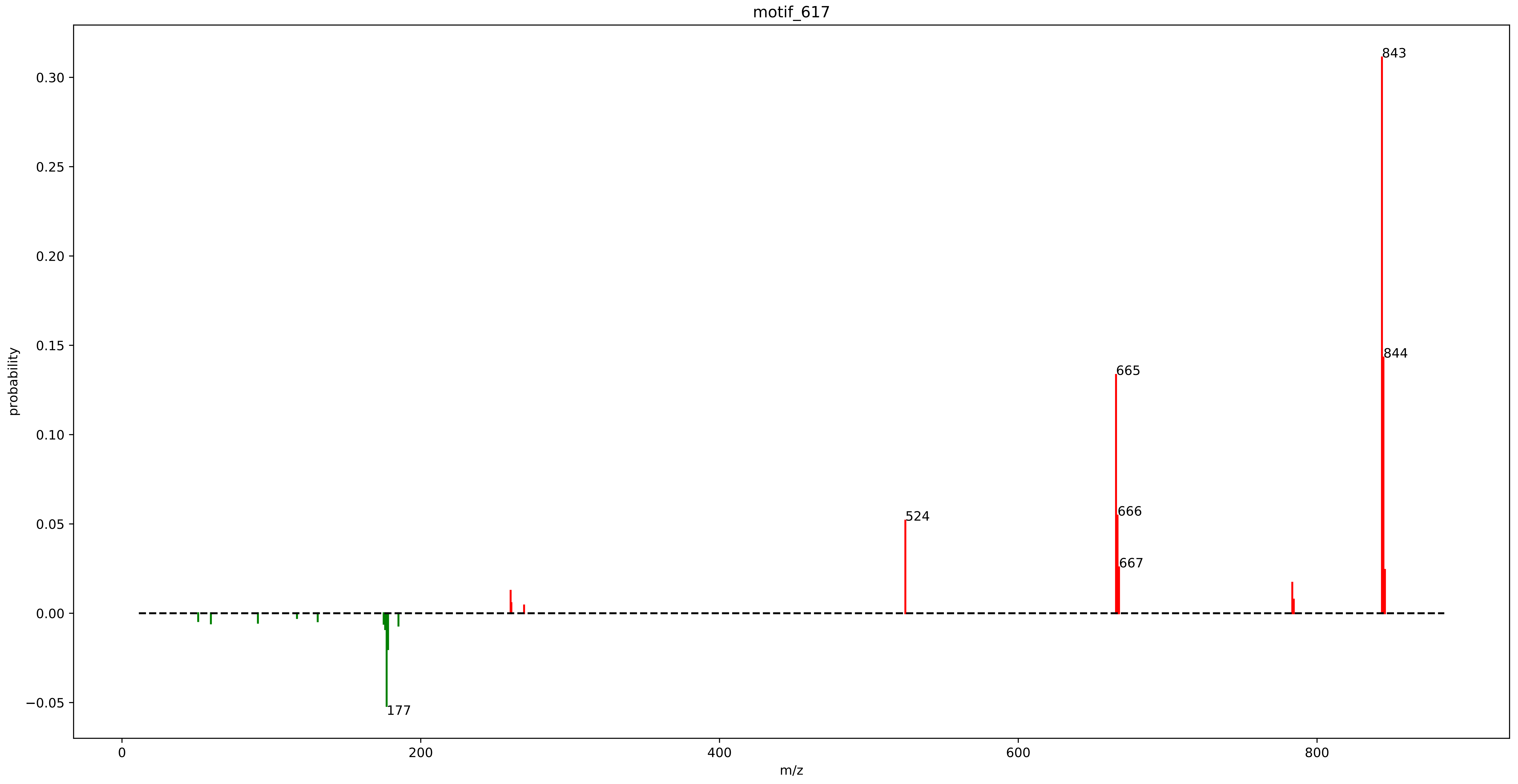

motif_617
probability
m/z
0.30
0.25
0.20
0.15
0.10
0.05
0.00
−0.05
0
200
400
600
800
843
844
665
666
667
524
177

motif_617

fragment_843.4525	: 0.31
fragment_844.4525	: 0.14
fragment_665.4375	: 0.13
fragment_666.4425	: 0.05
loss_177.1675	: 0.05
fragment_524.4025	: 0.05
fragment_667.4425	: 0.03
fragment_845.4575	: 0.02
loss_178.1175	: 0.02
fragment_783.4275	: 0.02
fragment_260.1675	: 0.01
loss_176.1675	: 0.01
fragment_784.4325	: 0.01
loss_185.0725	: 0.01
loss_175.1725	: 0.01
fragment_260.6725	: 0.01
loss_59.5175	: 0.01
loss_90.9925	: 0.01

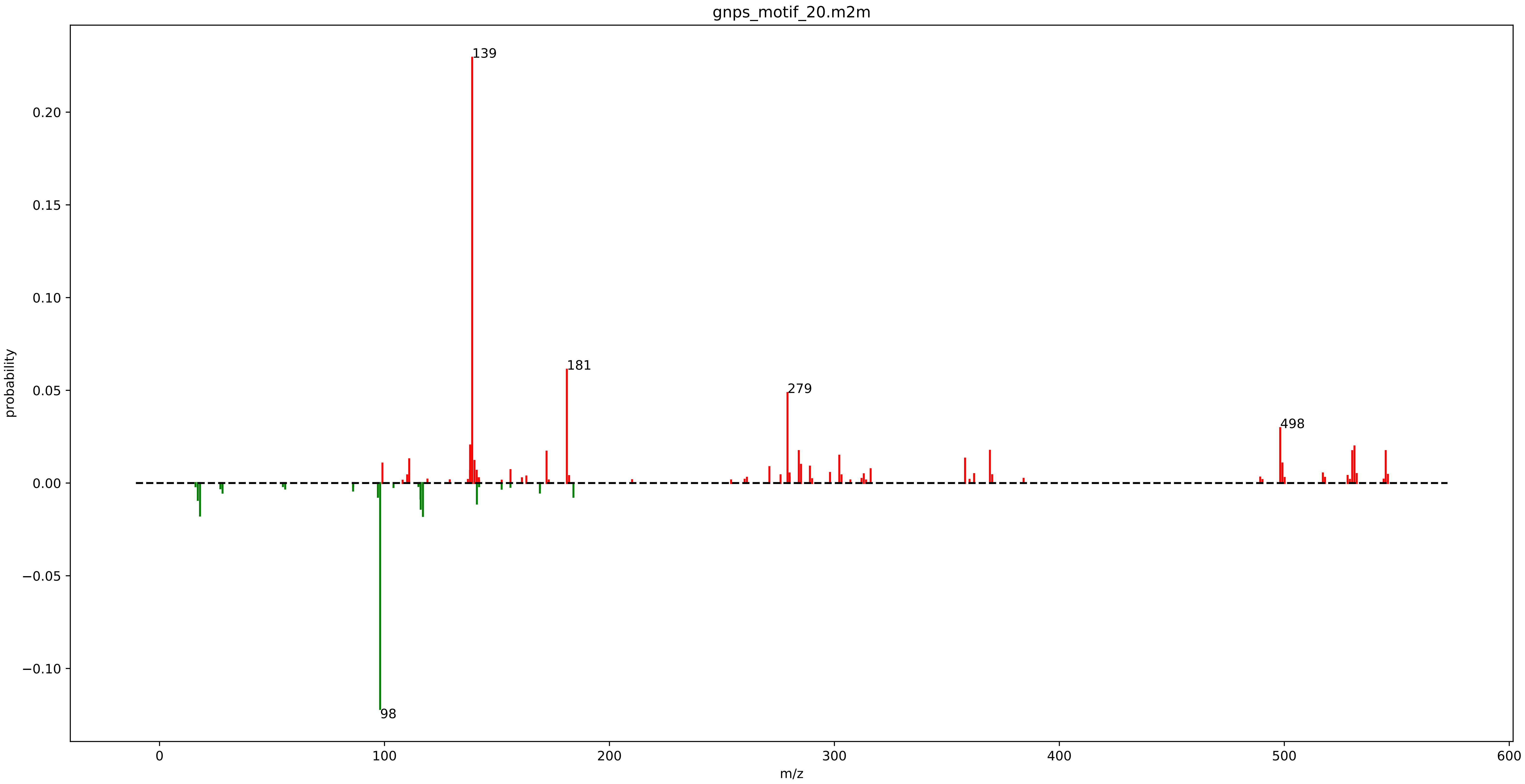

gnps_motif_20.m2m
probability
m/z
139
181
279
498
98
0.20
0.15
0.10
0.05
0.00
−0.05
−0.10
0
100
200
300
400
500
600

gnps_motif_20.m2m

fragment_138.9925	: 0.23
loss_98.0725	: 0.12
fragment_181.0725	: 0.06
fragment_279.1475	: 0.05
fragment_498.1775	: 0.03
fragment_138.0675	: 0.02
fragment_531.1875	: 0.02
loss_117.0925	: 0.02
loss_18.0075	: 0.02
fragment_369.1325	: 0.02
fragment_545.0825	: 0.02
fragment_284.1675	: 0.02
fragment_530.1925	: 0.02
fragment_172.0425	: 0.02
fragment_302.1775	: 0.01
loss_116.0925	: 0.01
fragment_358.0825	: 0.01
fragment_110.9975	: 0.01
fragment_139.9975	: 0.01
loss_141.0775	: 0.01
fragment_499.1775	: 0.01
fragment_99.0825	: 0.01
fragment_285.1675	: 0.01
loss_17.0075	: 0.01
fragment_289.1075	: 0.01
fragment_271.1125	: 0.01
loss_116.0025	: 0.01
fragment_316.1075	: 0.01
loss_97.0725	: 0.01
loss_183.9925	: 0.01
fragment_156.0225	: 0.01
fragment_140.9925	: 0.01
fragment_138.0125	: 0.01
fragment_298.0625	: 0.01
loss_28.0175	: 0.01
fragment_517.1375	: 0.01
fragment_280.0575	: 0.01
loss_169.0725	: 0.01

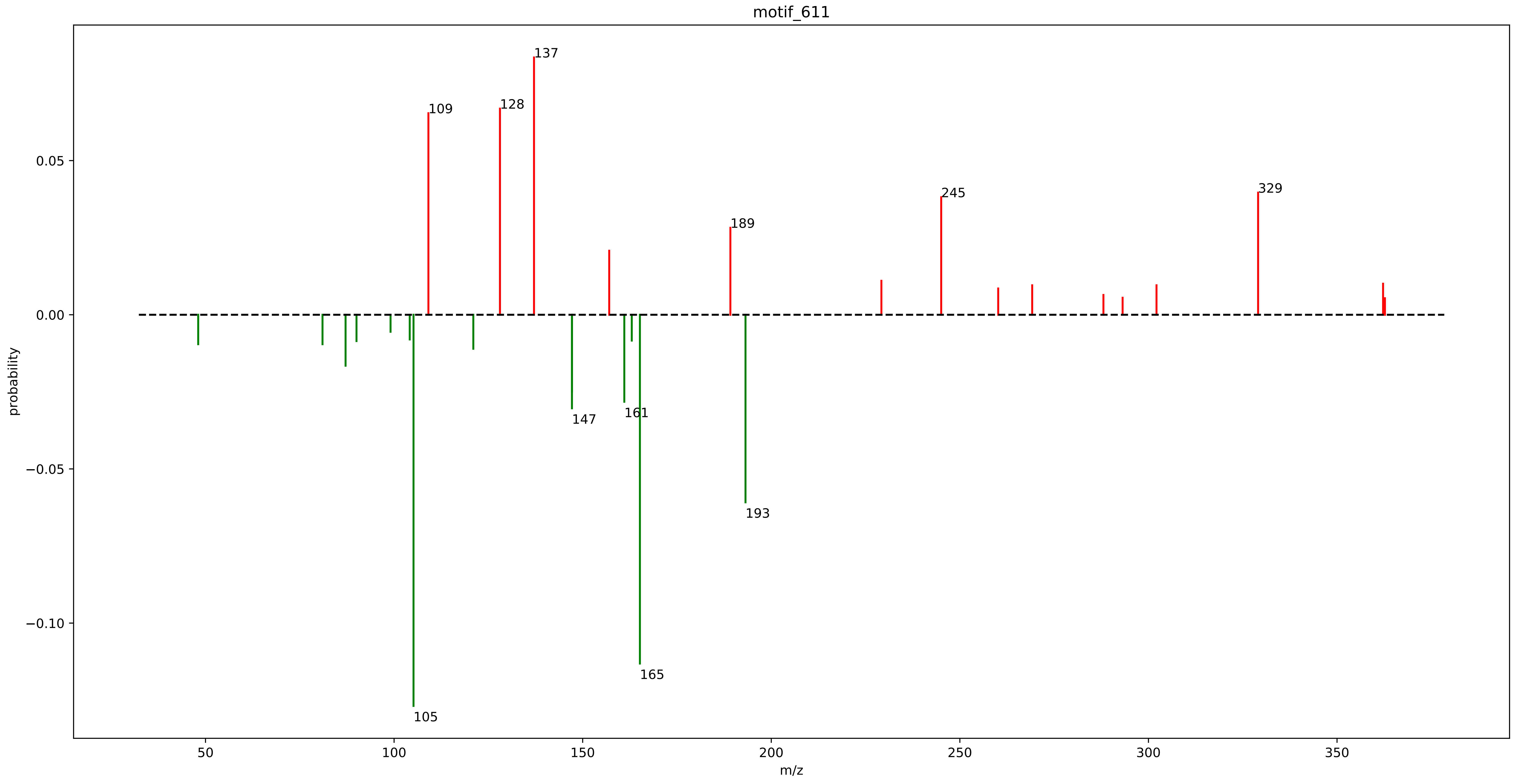
motif_611
probability
m/z
0.05
0.00
−0.05
−0.10
50
100
150
200
250
300
350
109
128
137
189
245
329
105
147
161
165
193

motif_611

loss_105.1475	: 0.13
loss_165.1725	: 0.11
fragment_137.0975	: 0.08
fragment_128.0725	: 0.07
fragment_109.0975	: 0.07
loss_193.1675	: 0.06
fragment_329.0725	: 0.04
fragment_245.0525	: 0.04
loss_147.1575	: 0.03
loss_161.0375	: 0.03
fragment_189.1625	: 0.03
loss_147.1625	: 0.02
fragment_157.0325	: 0.02
loss_87.1375	: 0.02
loss_121.0025	: 0.01
fragment_229.1975	: 0.01
fragment_362.2025	: 0.01
loss_48.0625	: 0.01
fragment_302.1375	: 0.01
fragment_269.1725	: 0.01
loss_81.0225	: 0.01
loss_90.0325	: 0.01
fragment_260.1675	: 0.01
loss_163.0175	: 0.01
loss_104.1475	: 0.01
fragment_288.0625	: 0.01
fragment_293.1475	: 0.01
loss_99.0625	: 0.01
fragment_362.7025	: 0.01

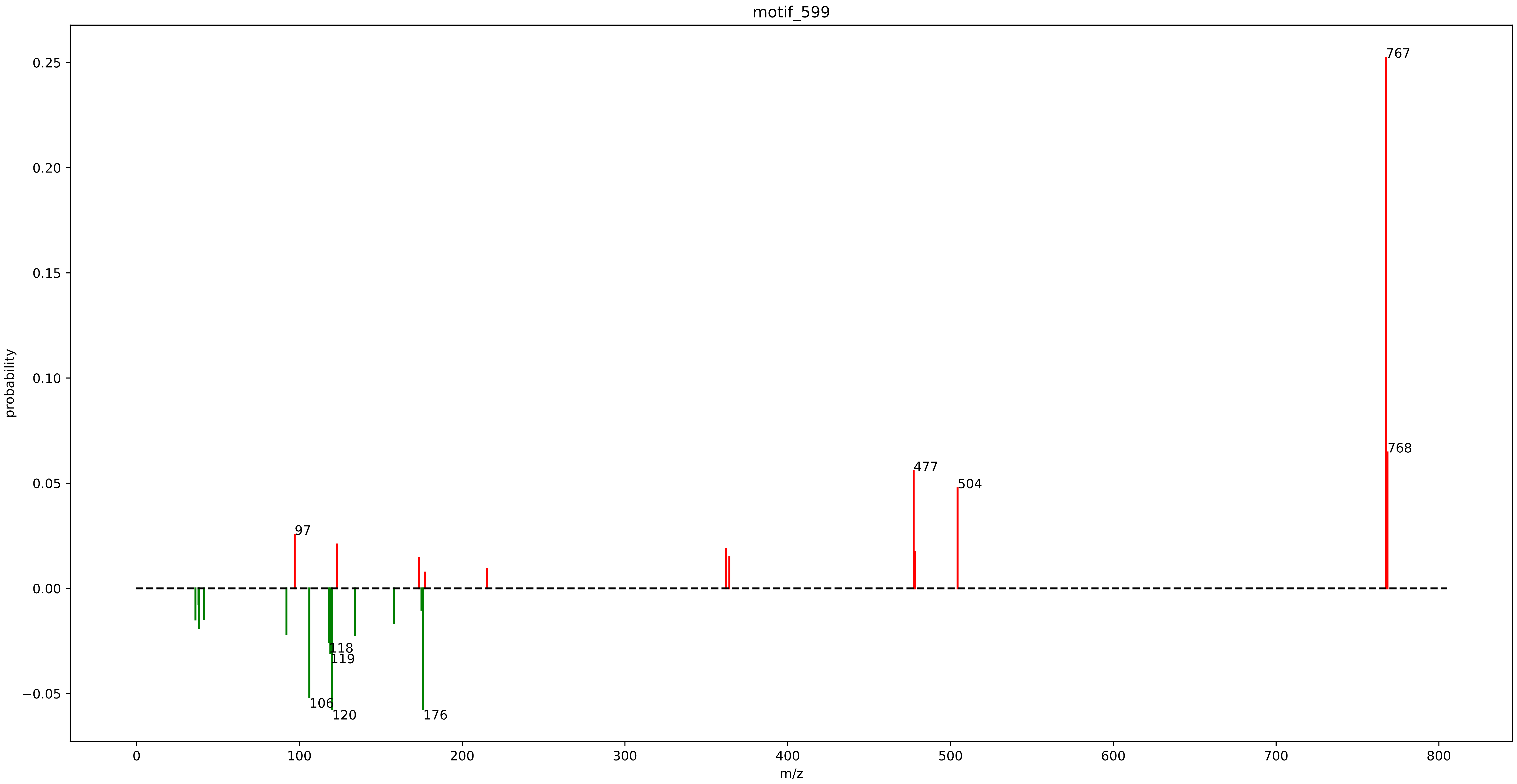

motif_599
probability
m/z
767
768
477
504
97
118
119
106
120
176
0.25
0.20
0.15
0.10
0.05
0.00
−0.05
0
100
200
300
400
500
600
700
800

motif_599

fragment_767.4225	: 0.25
fragment_768.4225	: 0.06
loss_120.1125	: 0.06
loss_176.0275	: 0.06
fragment_477.3325	: 0.06
loss_106.0975	: 0.05
fragment_504.3425	: 0.05
loss_119.0375	: 0.03
loss_118.0975	: 0.03
fragment_97.1025	: 0.03
loss_134.1325	: 0.02
loss_92.0825	: 0.02
fragment_123.1175	: 0.02
fragment_362.1275	: 0.02
loss_38.1525	: 0.02
fragment_478.3375	: 0.02
loss_158.0175	: 0.02
fragment_364.1275	: 0.01
loss_36.1575	: 0.01
fragment_173.6125	: 0.01
loss_41.5875	: 0.01
loss_175.0225	: 0.01
fragment_215.1775	: 0.01
loss_38.0375	: 0.01
fragment_177.1625	: 0.01

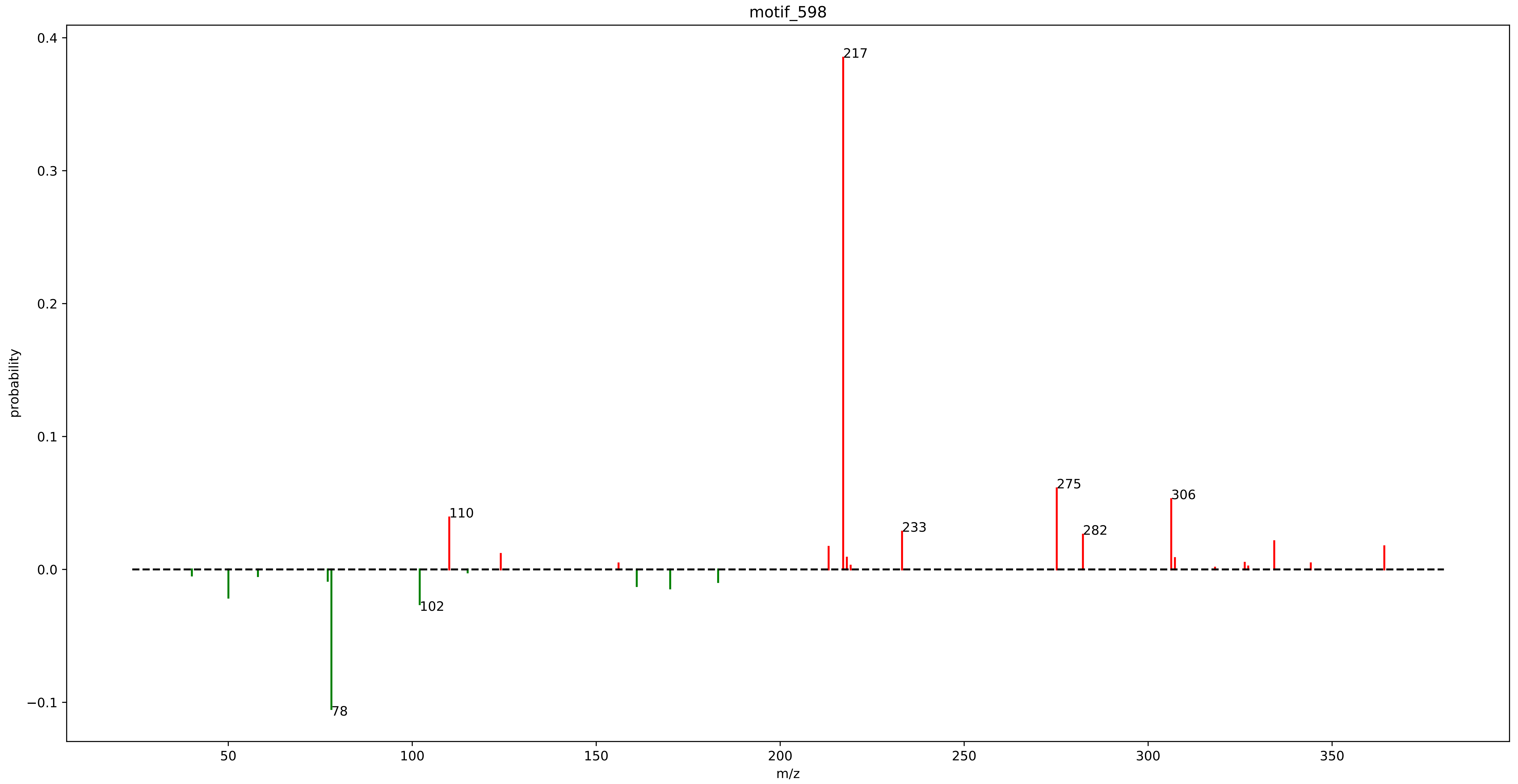

motif_598
0.4
0.3
0.2
0.1
0.0
−0.1
probability
217
275
306
110
233
282
102
78
50
100
150
200
250
300
350
m/z

motif_598

fragment_217.1175	: 0.39
loss_78.0275	: 0.10
fragment_275.1625	: 0.06
fragment_306.2775	: 0.05
fragment_110.0575	: 0.04
fragment_233.1175	: 0.03
loss_102.0275	: 0.03
fragment_282.2775	: 0.03
fragment_334.2725	: 0.02
loss_50.0325	: 0.02
fragment_364.1875	: 0.02
fragment_213.1625	: 0.02
loss_170.1025	: 0.01
loss_161.0125	: 0.01
fragment_124.0575	: 0.01
loss_183.1325	: 0.01
fragment_218.1175	: 0.01
fragment_307.2825	: 0.01
loss_77.0275	: 0.01

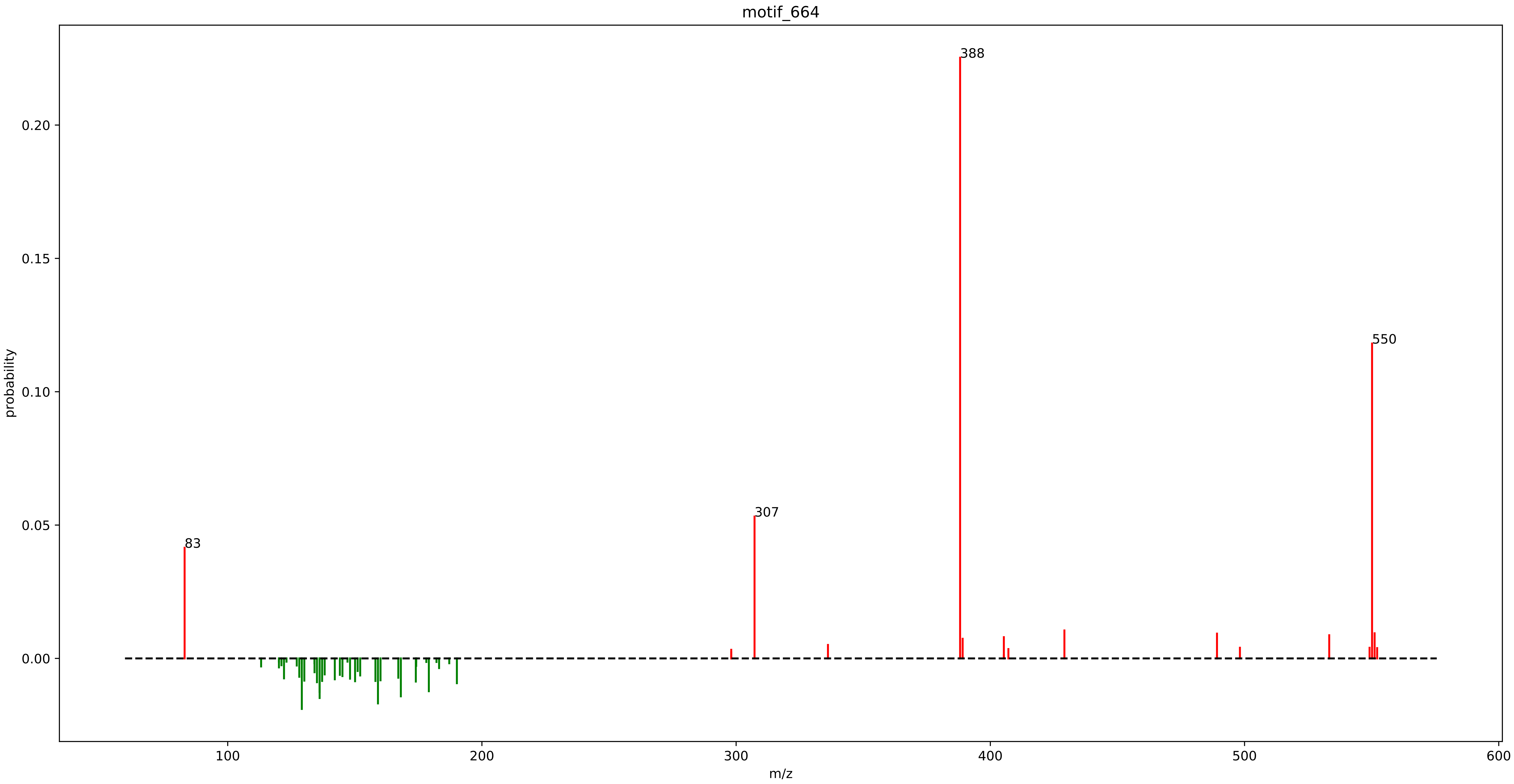

motif_664
388
550
307
83
probability
0.20
0.15
0.10
0.05
0.00
100
200
300
400
500
600
m/z

motif_664

fragment_388.1075	: 0.23
fragment_550.1625	: 0.12
fragment_307.2425	: 0.05
fragment_83.0475	: 0.04
loss_129.1525	: 0.02
loss_159.1075	: 0.02
loss_136.1625	: 0.01
loss_168.1125	: 0.01
loss_136.1475	: 0.01
loss_179.1225	: 0.01
fragment_429.1375	: 0.01
fragment_551.1675	: 0.01
loss_190.1525	: 0.01
fragment_489.1625	: 0.01
loss_135.1125	: 0.01
fragment_533.3125	: 0.01
loss_174.0025	: 0.01
loss_150.1025	: 0.01
loss_158.1125	: 0.01
loss_137.1425	: 0.01
loss_130.1425	: 0.01
loss_160.1125	: 0.01
fragment_405.3175	: 0.01
loss_142.1225	: 0.01
loss_148.1225	: 0.01
loss_122.1475	: 0.01
fragment_389.1125	: 0.01
loss_167.1075	: 0.01
loss_128.1525	: 0.01
loss_145.1375	: 0.01
loss_152.1175	: 0.01
loss_144.1175	: 0.01
loss_138.1475	: 0.01
loss_134.1525	: 0.01
fragment_336.1275	: 0.01

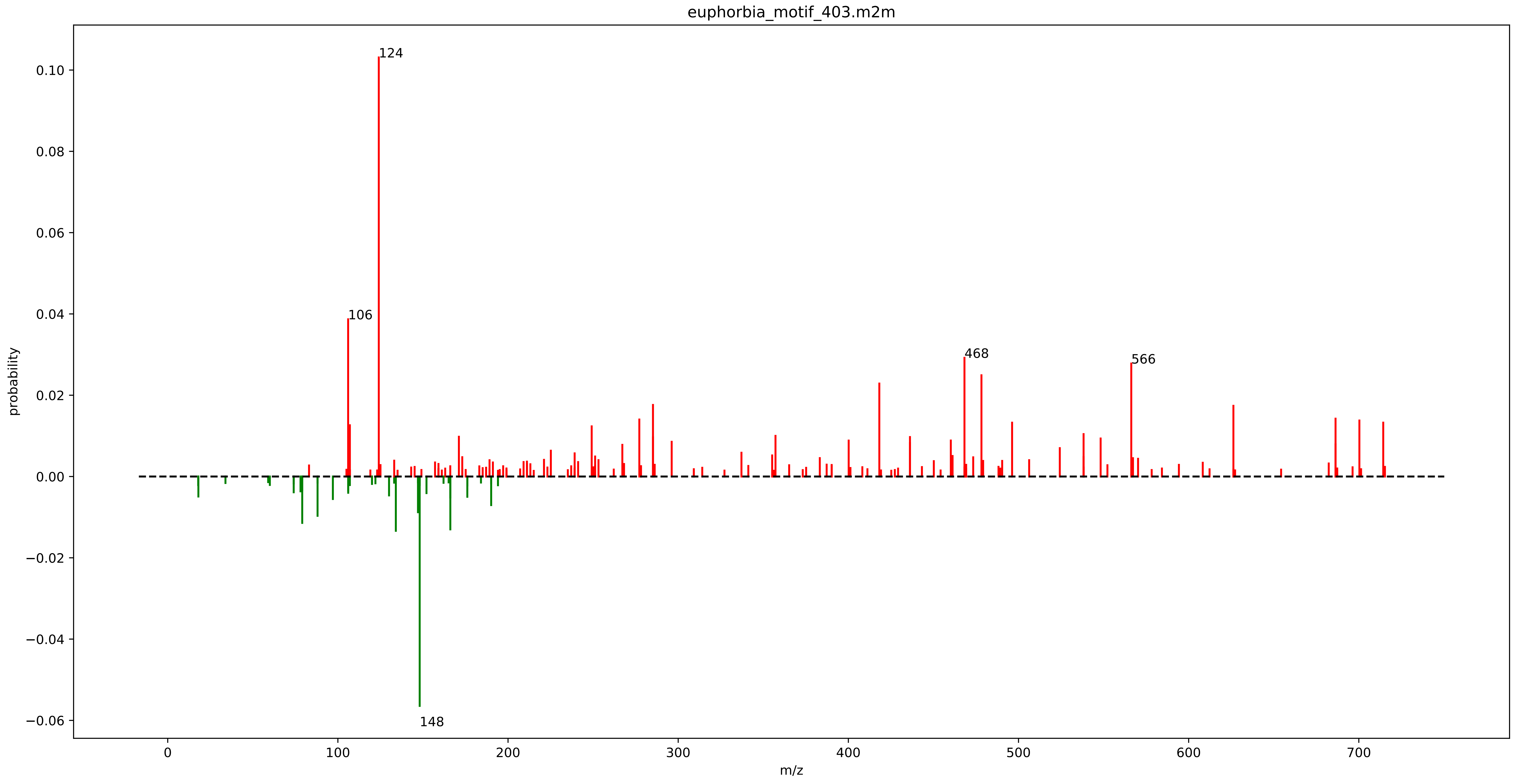

euphorbia_motif_403.m2m
probability
m/z
124
106
468
566
148
0.10
0.08
0.06
0.04
0.02
0.00
−0.02
−0.04
−0.06
0
100
200
300
400
500
600
700

euphorbia_motif_403.m2m

fragment_124.0375 : 0.10
loss_148.0775 : 0.06
fragment_106.0275 : 0.04
fragment_468.2375 : 0.03
fragment_566.2725 : 0.03
fragment_478.2225 : 0.02
fragment_418.2025 : 0.02
fragment_285.1825 : 0.02
fragment_626.2925 : 0.02
fragment_686.3175 : 0.01
fragment_277.1575 : 0.01
fragment_700.3325 : 0.01
loss_134.0625 : 0.01
fragment_714.3475 : 0.01
fragment_496.2325 : 0.01
loss_166.0875 : 0.01
fragment_107.0475 : 0.01
fragment_249.1625 : 0.01
loss_79.0475 : 0.01
fragment_538.2425 : 0.01
fragment_357.1825 : 0.01
fragment_171.1175 : 0.01
fragment_436.2125 : 0.01
loss_88.0575 : 0.01
fragment_285.1875 : 0.01
fragment_548.2625 : 0.01
fragment_460.2125 : 0.01
fragment_400.1925 : 0.01
loss_147.0725 : 0.01
fragment_296.1725 : 0.01
fragment_436.2075 : 0.01
fragment_686.3125 : 0.01
fragment_267.1725 : 0.01
loss_190.0875 : 0.01
fragment_524.2625 : 0.01
fragment_225.1625 : 0.01
fragment_337.1775 : 0.01
fragment_239.1425 : 0.01
loss_97.0575 : 0.01
fragment_355.2225 : 0.01
loss_166.0825 : 0.01
fragment_461.2525 : 0.01

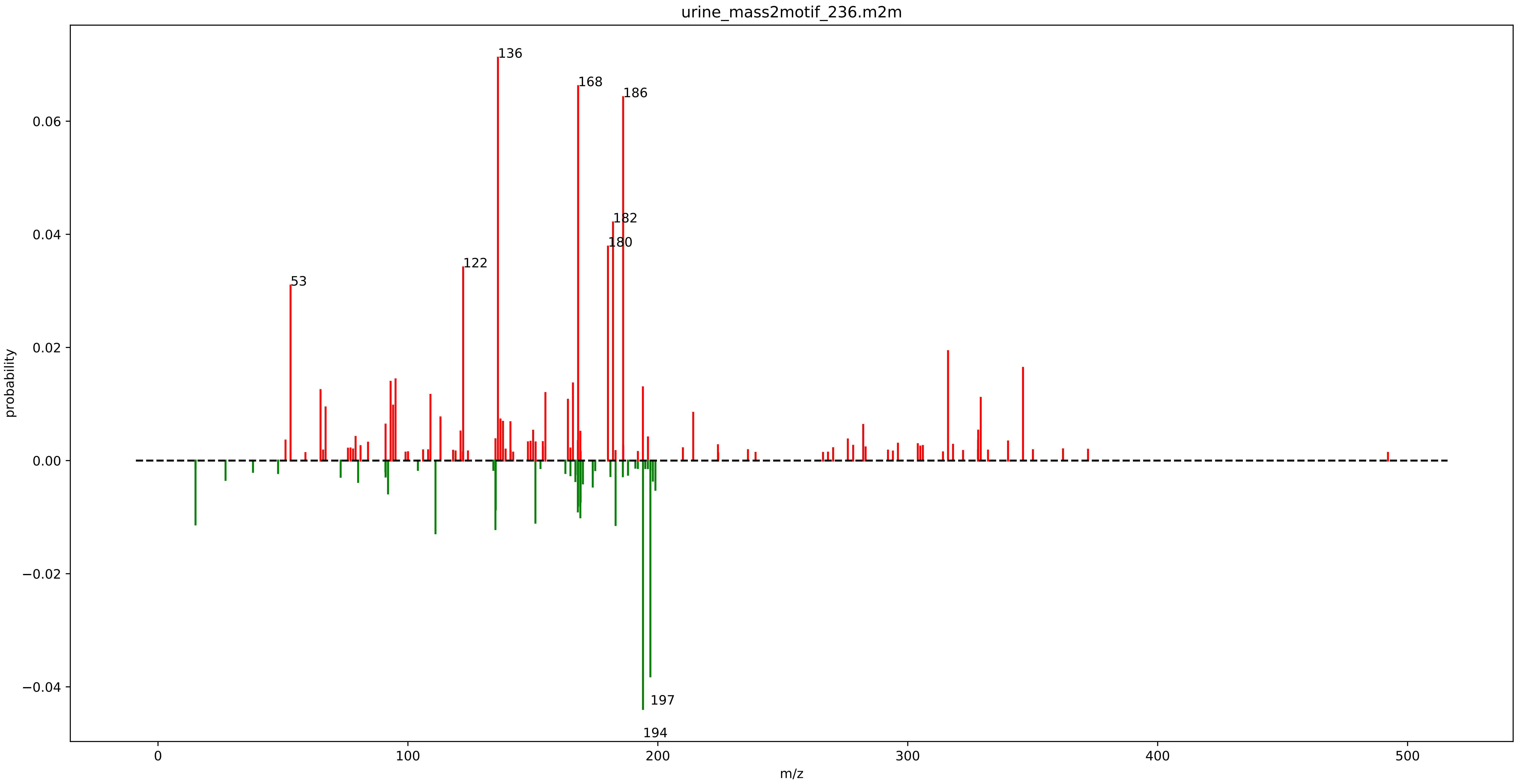
urine_mass2motif_236.m2m
probability
m/z
0.06
0.04
0.02
0.00
−0.02
−0.04
0
100
200
300
400
500
136
168
186
182
180
122
53
197
194

urine_mass2motif_236.m2m

fragment_136.0225	: 0.07
fragment_168.1025	: 0.07
fragment_186.1125	: 0.06
loss_194.0675	: 0.04
fragment_182.0625	: 0.04
loss_197.0125	: 0.04
fragment_180.0475	: 0.04
fragment_122.0975	: 0.03
fragment_53.0375	: 0.03
fragment_316.1125	: 0.02
fragment_346.1225	: 0.02
fragment_95.0475	: 0.01
fragment_93.0675	: 0.01
fragment_166.0325	: 0.01
fragment_194.0275	: 0.01
loss_111.0325	: 0.01
fragment_65.0375	: 0.01
loss_135.0175	: 0.01
fragment_155.0175	: 0.01
fragment_109.0125	: 0.01
loss_183.1075	: 0.01
loss_15.0075	: 0.01
fragment_329.2075	: 0.01
loss_151.0125	: 0.01
fragment_164.0175	: 0.01
loss_169.0025	: 0.01
fragment_94.0675	: 0.01
fragment_67.0525	: 0.01
loss_167.9975	: 0.01
loss_135.1275	: 0.01
fragment_214.1525	: 0.01
loss_168.1075	: 0.01
fragment_113.0175	: 0.01
fragment_137.0275	: 0.01
loss_169.1075	: 0.01
fragment_137.0075	: 0.01
fragment_138.0375	: 0.01
fragment_141.0125	: 0.01
fragment_91.0525	: 0.01
fragment_282.1675	: 0.01
loss_92.0475	: 0.01
fragment_328.1775	: 0.01
fragment_150.0925	: 0.01
loss_199.0325	: 0.01
fragment_121.0625	: 0.01
fragment_169.0025	: 0.01

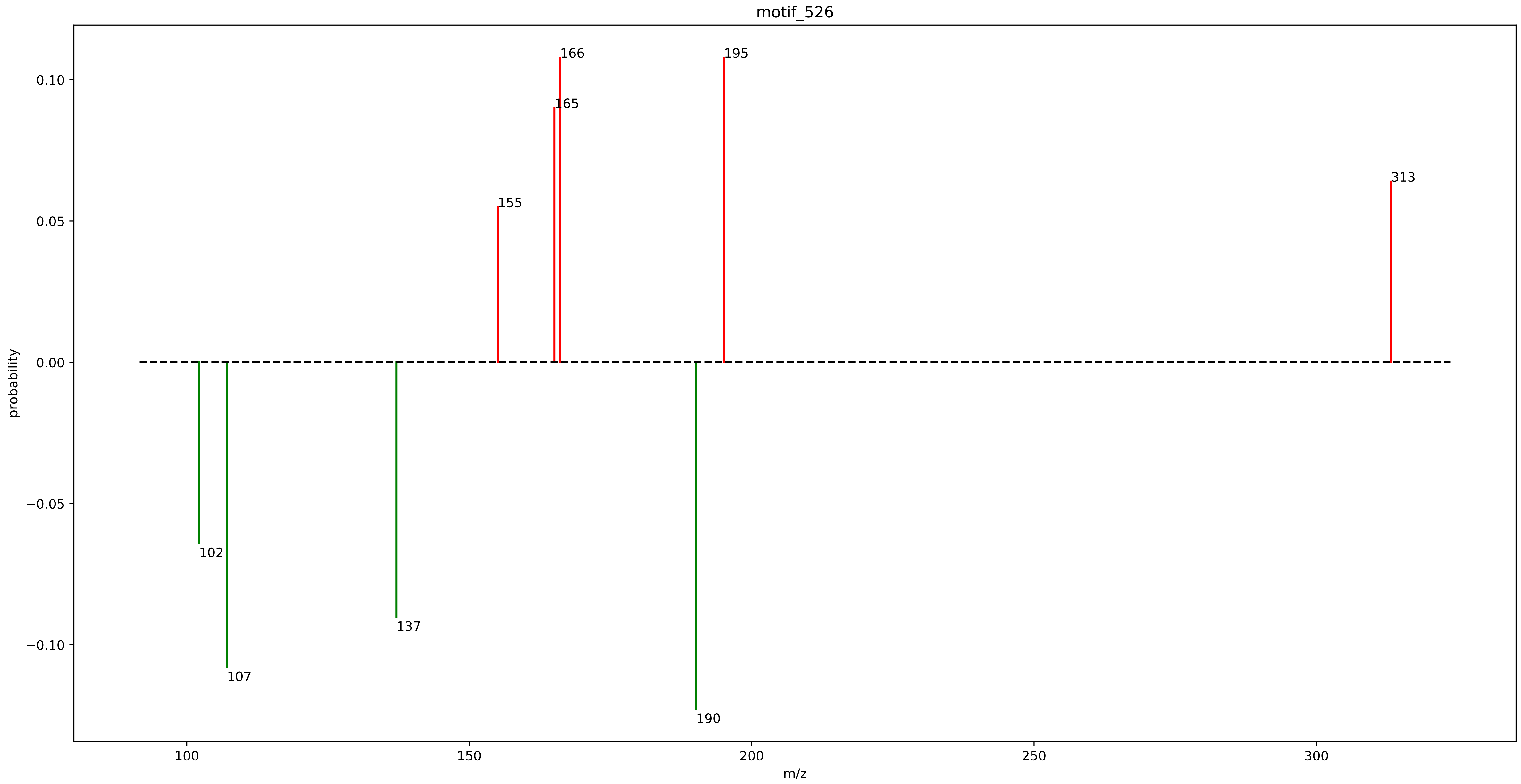
motif_526
166
195
165
313
155
102
107
137
190
0.10
0.05
0.00
−0.05
−0.10
probability
100
150
200
250
300
m/z

motif_526

loss_190.1725	: 0.12
loss_107.1075	: 0.11
fragment_195.0975	: 0.11
fragment_166.0875	: 0.11
fragment_165.0875	: 0.09
loss_137.1175	: 0.09
loss_102.1625	: 0.06
fragment_313.1975	: 0.06
fragment_155.0525	: 0.05

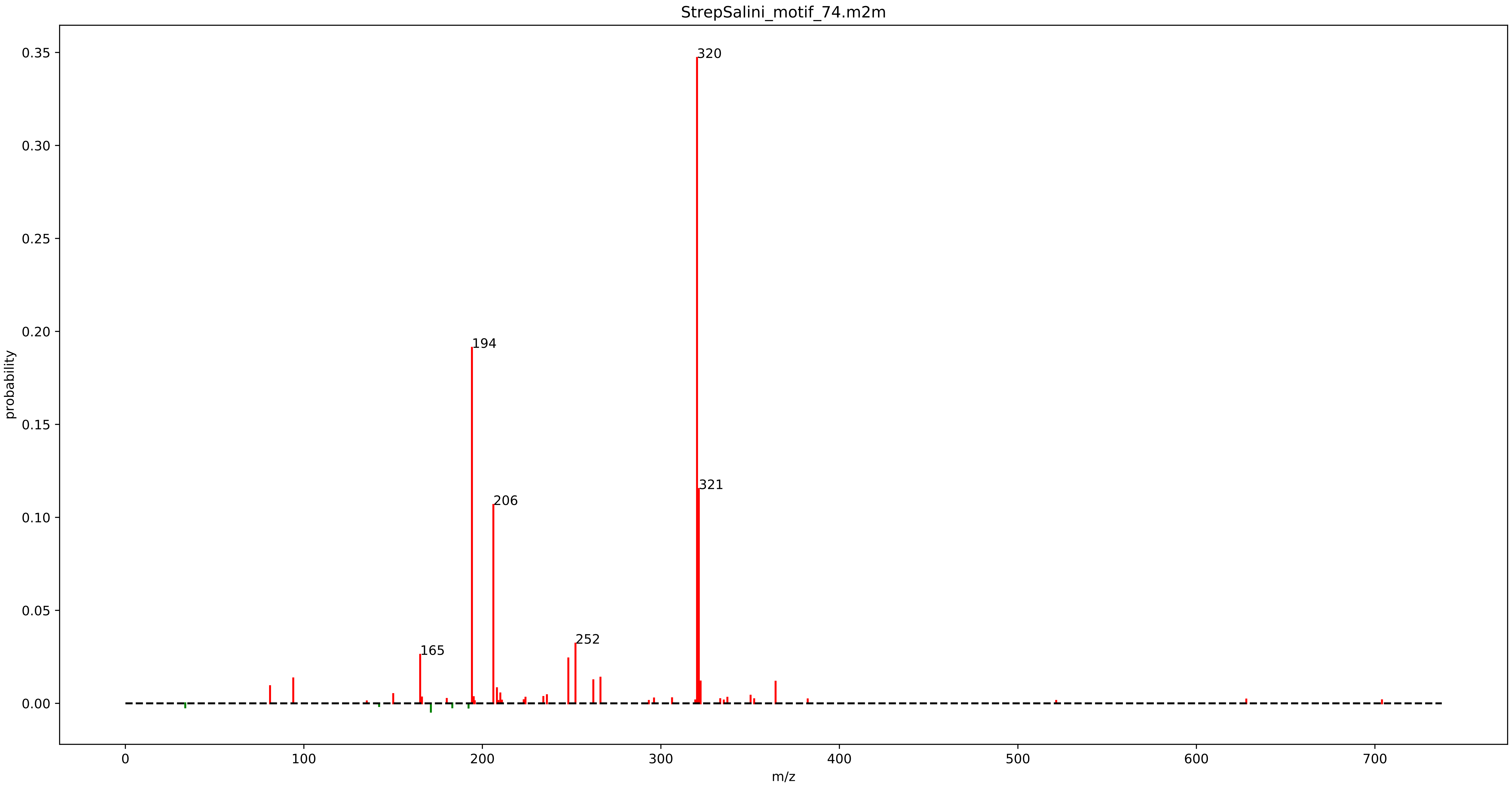
StrepSalini_motif_74.m2m
probability
m/z
0.35
0.30
0.25
0.20
0.15
0.10
0.05
0.00
0
100
200
300
400
500
600
700
320
194
206
321
252
165

StrepSalini_motif_74.m2m

fragment_320.2500	: 0.35
fragment_194.1500	: 0.19
fragment_321.2500	: 0.12
fragment_206.1500	: 0.11
fragment_252.1500	: 0.03
fragment_165.1500	: 0.03
fragment_248.1500	: 0.02
fragment_266.1500	: 0.01
fragment_94.0500	: 0.01
fragment_262.1500	: 0.01
fragment_322.2500	: 0.01
fragment_364.2500	: 0.01
fragment_81.0500	: 0.01
fragment_208.1500	: 0.01
fragment_210.0500	: 0.01

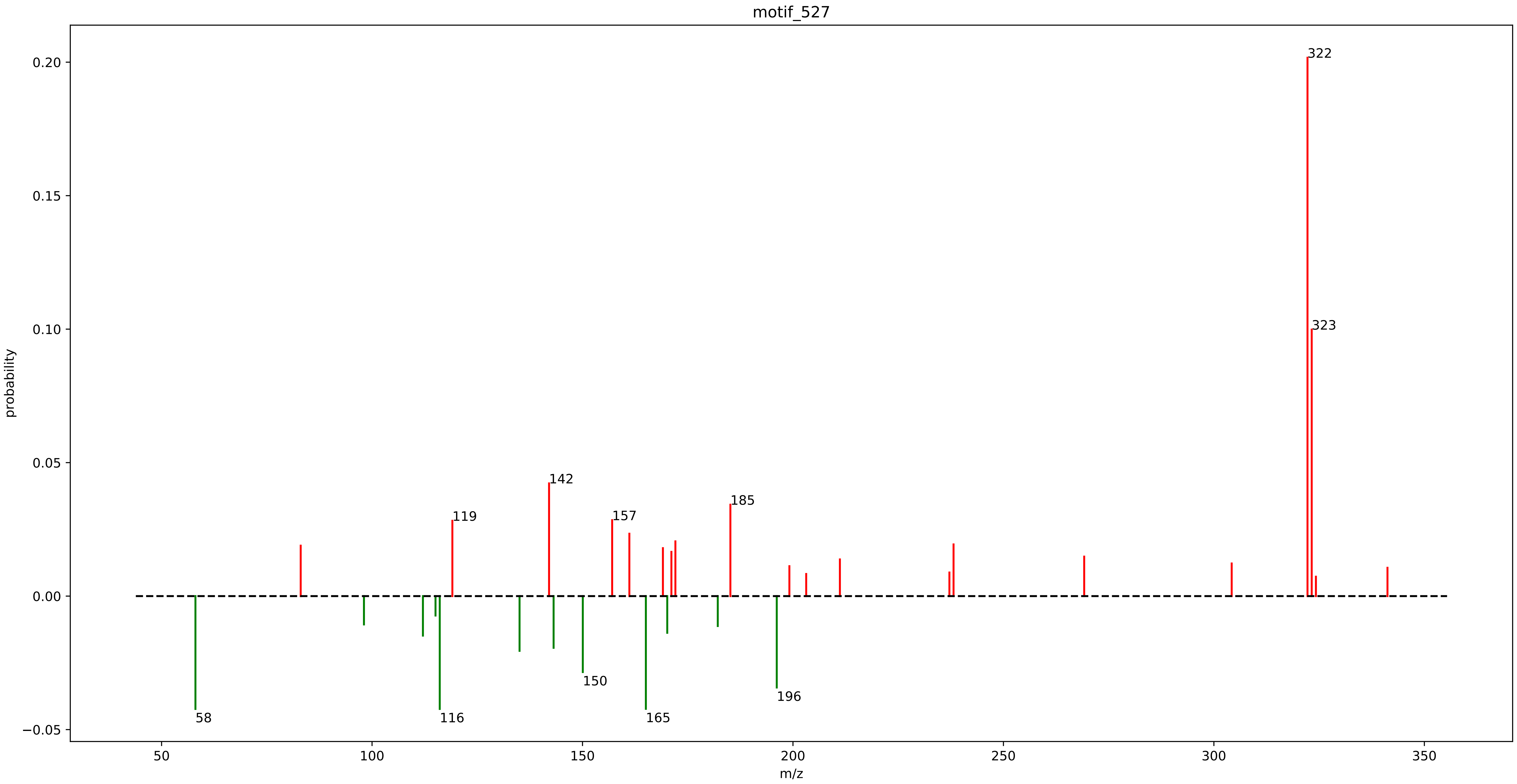
motif_527
probability
m/z
0.20
0.15
0.10
0.05
0.00
−0.05
50
100
150
200
250
300
350
322
323
142
185
119
157
58
116
150
165
196

motif_527

fragment_322.2375	: 0.20
fragment_323.2375	: 0.10
loss_58.0425	: 0.04
loss_116.0825	: 0.04
fragment_142.0525	: 0.04
loss_165.0525	: 0.04
loss_196.1475	: 0.03
fragment_185.1325	: 0.03
loss_150.0675	: 0.03
fragment_157.0425	: 0.03
fragment_119.0825	: 0.03
fragment_161.1325	: 0.02
loss_135.0425	: 0.02
fragment_172.0675	: 0.02
fragment_238.1475	: 0.02
loss_143.1325	: 0.02
fragment_83.0475	: 0.02
fragment_169.1025	: 0.02
fragment_171.1175	: 0.02
loss_112.0875	: 0.01
fragment_269.1875	: 0.01
loss_170.1325	: 0.01
fragment_211.1475	: 0.01
fragment_304.2275	: 0.01
fragment_199.1475	: 0.01
loss_182.1325	: 0.01
loss_98.0775	: 0.01
fragment_341.2375	: 0.01
fragment_237.1625	: 0.01
fragment_203.1425	: 0.01
fragment_324.2375	: 0.01
loss_115.0775	: 0.01

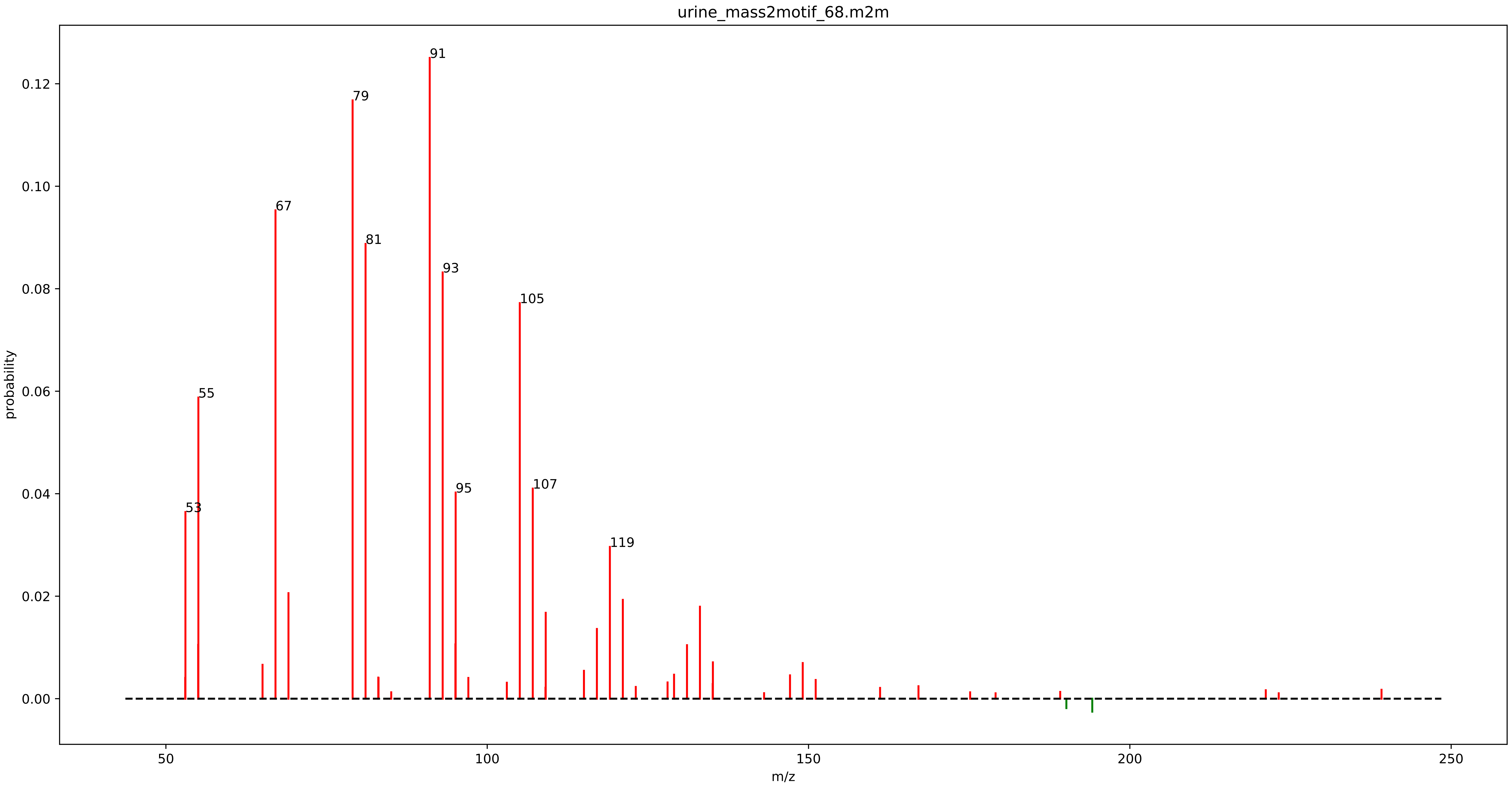

urine_mass2motif_68.m2m
probability
m/z
0.12
0.10
0.08
0.06
0.04
0.02
0.00
50
100
150
200
250
91
79
67
81
93
105
55
95
107
53
119

urine_mass2motif_68.m2m

fragment_91.0525	: 0.13
fragment_79.0525	: 0.12
fragment_67.0525	: 0.10
fragment_81.0675	: 0.09
fragment_93.0675	: 0.08
fragment_105.0675	: 0.08
fragment_55.0525	: 0.06
fragment_107.0875	: 0.04
fragment_95.0875	: 0.04
fragment_53.0375	: 0.04
fragment_119.0875	: 0.03
fragment_69.0725	: 0.02
fragment_121.1025	: 0.02
fragment_133.1025	: 0.02
fragment_109.1025	: 0.02
fragment_117.0675	: 0.01
fragment_95.0475	: 0.01
fragment_55.0175	: 0.01
fragment_131.0875	: 0.01
fragment_135.1175	: 0.01
fragment_149.0975	: 0.01
fragment_65.0375	: 0.01
fragment_115.0525	: 0.01

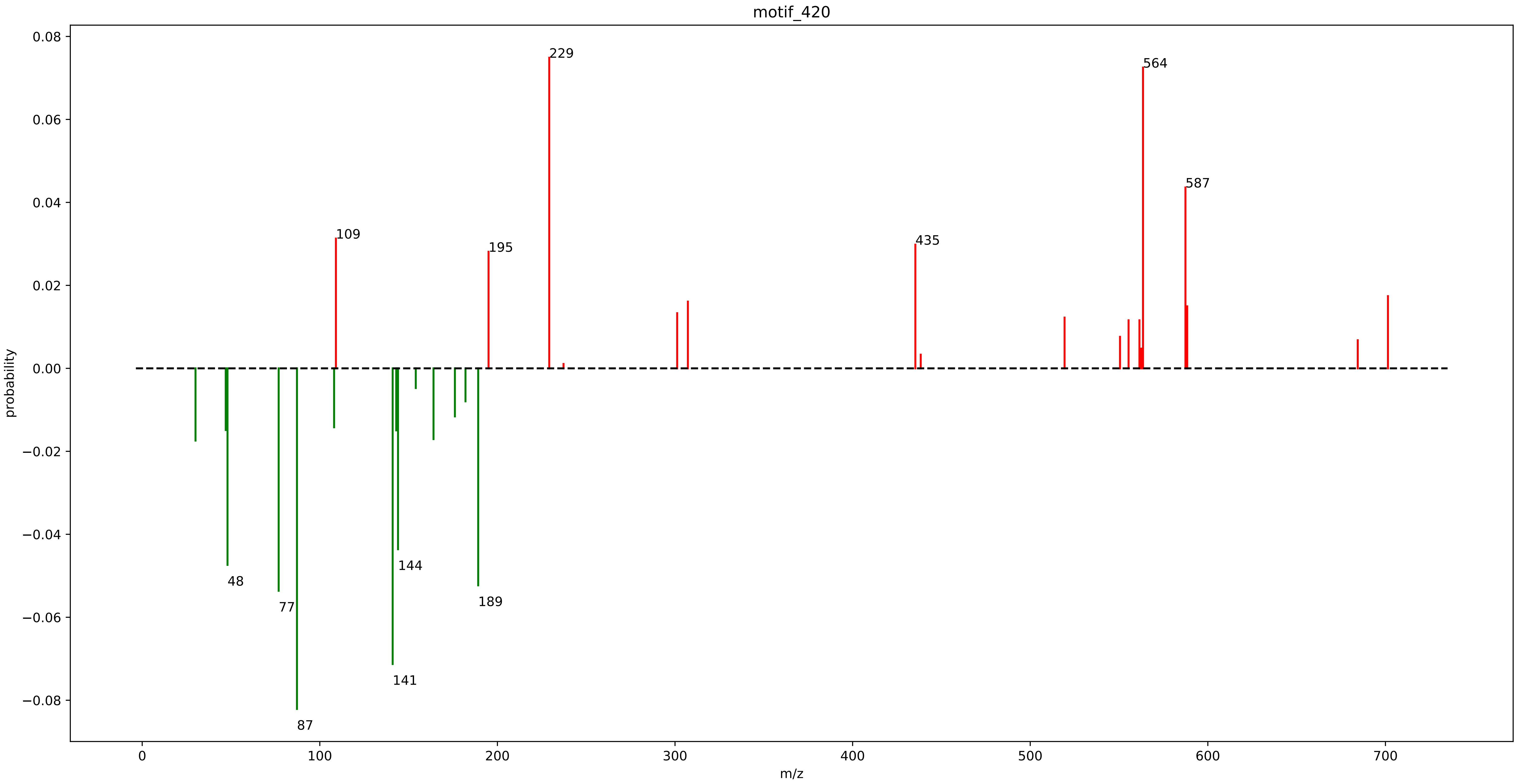
motif_420
probability
m/z
0.08
0.06
0.04
0.02
0.00
−0.02
−0.04
−0.06
−0.08
0
100
200
300
400
500
600
700
229
564
587
109
195
435
48
77
87
141
144
189

motif_420

loss_87.1375	: 0.08
fragment_229.1775	: 0.07
fragment_563.5025	: 0.07
loss_141.0125	: 0.07
loss_76.8325	: 0.05
loss_189.1725	: 0.05
loss_48.0275	: 0.05
fragment_587.3975	: 0.04
loss_144.0325	: 0.04
fragment_109.0725	: 0.03
fragment_435.3275	: 0.03
fragment_195.0025	: 0.03
fragment_701.4125	: 0.02
loss_30.0125	: 0.02
loss_164.0225	: 0.02
fragment_307.2425	: 0.02
loss_143.0275	: 0.01
fragment_588.3975	: 0.01
loss_47.0225	: 0.01
loss_108.0475	: 0.01
fragment_301.2125	: 0.01
fragment_519.3325	: 0.01
fragment_561.4875	: 0.01
loss_176.0575	: 0.01
fragment_555.3675	: 0.01
loss_182.0275	: 0.01
fragment_550.5575	: 0.01
fragment_684.4025	: 0.01

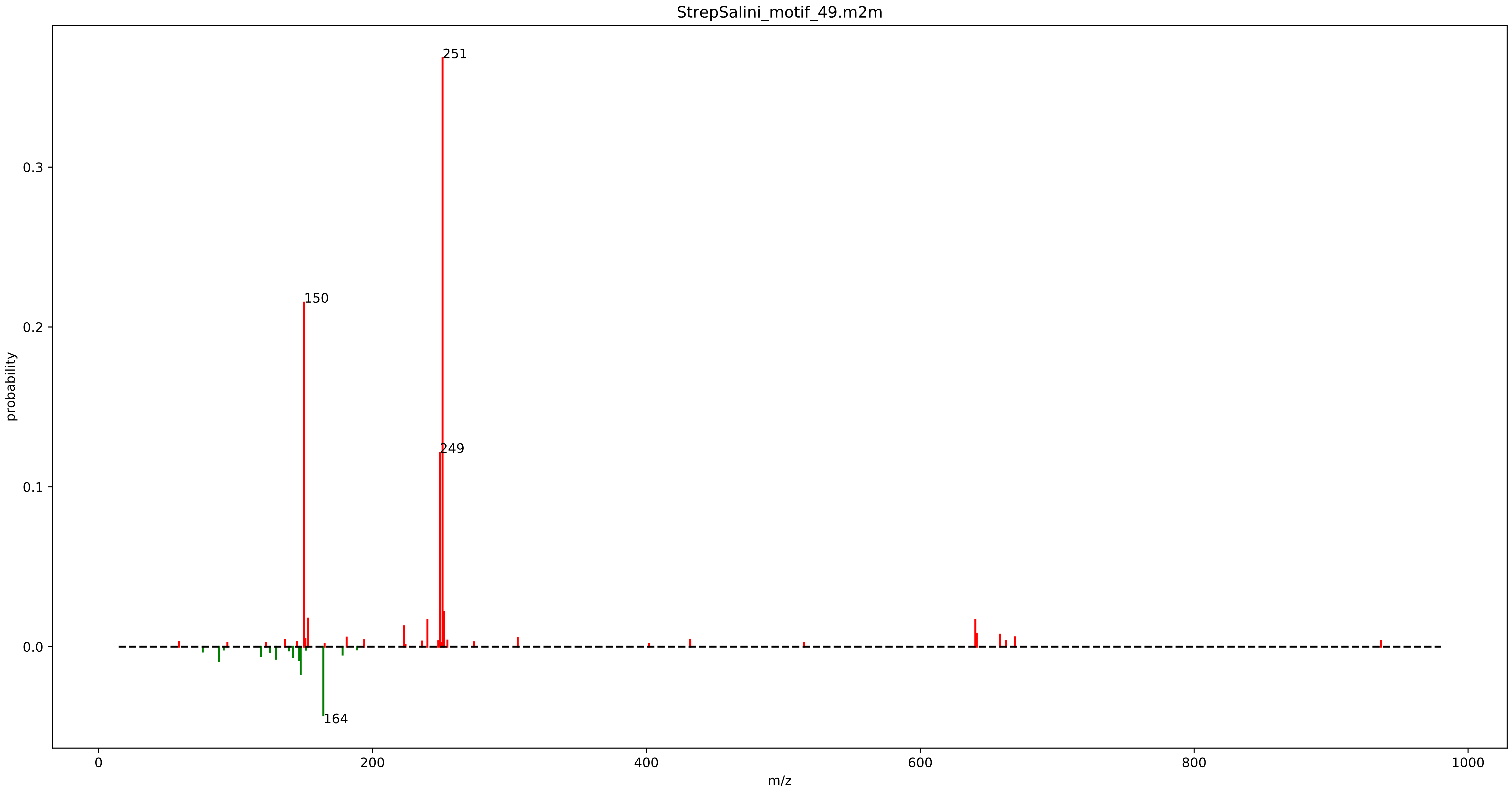

StrepSalini_motif_49.m2m
probability
m/z
251
150
249
164
0.3
0.2
0.1
0.0
0
200
400
600
800
1000

StrepSalini_motif_49.m2m

fragment_251.1500	: 0.37
fragment_150.0500	: 0.22
fragment_249.0500	: 0.12
loss_164.1500	: 0.04
fragment_252.1500	: 0.02
fragment_153.0525	: 0.02
loss_147.5500	: 0.02
fragment_640.2500	: 0.02
fragment_240.1500	: 0.02
fragment_223.1525	: 0.01
loss_88.0525	: 0.01
loss_146.5500	: 0.01
fragment_641.2500	: 0.01
fragment_658.2500	: 0.01
loss_129.5500	: 0.01
loss_142.1500	: 0.01
loss_118.5500	: 0.01
fragment_669.2500	: 0.01
fragment_181.1500	: 0.01
fragment_306.0500	: 0.01

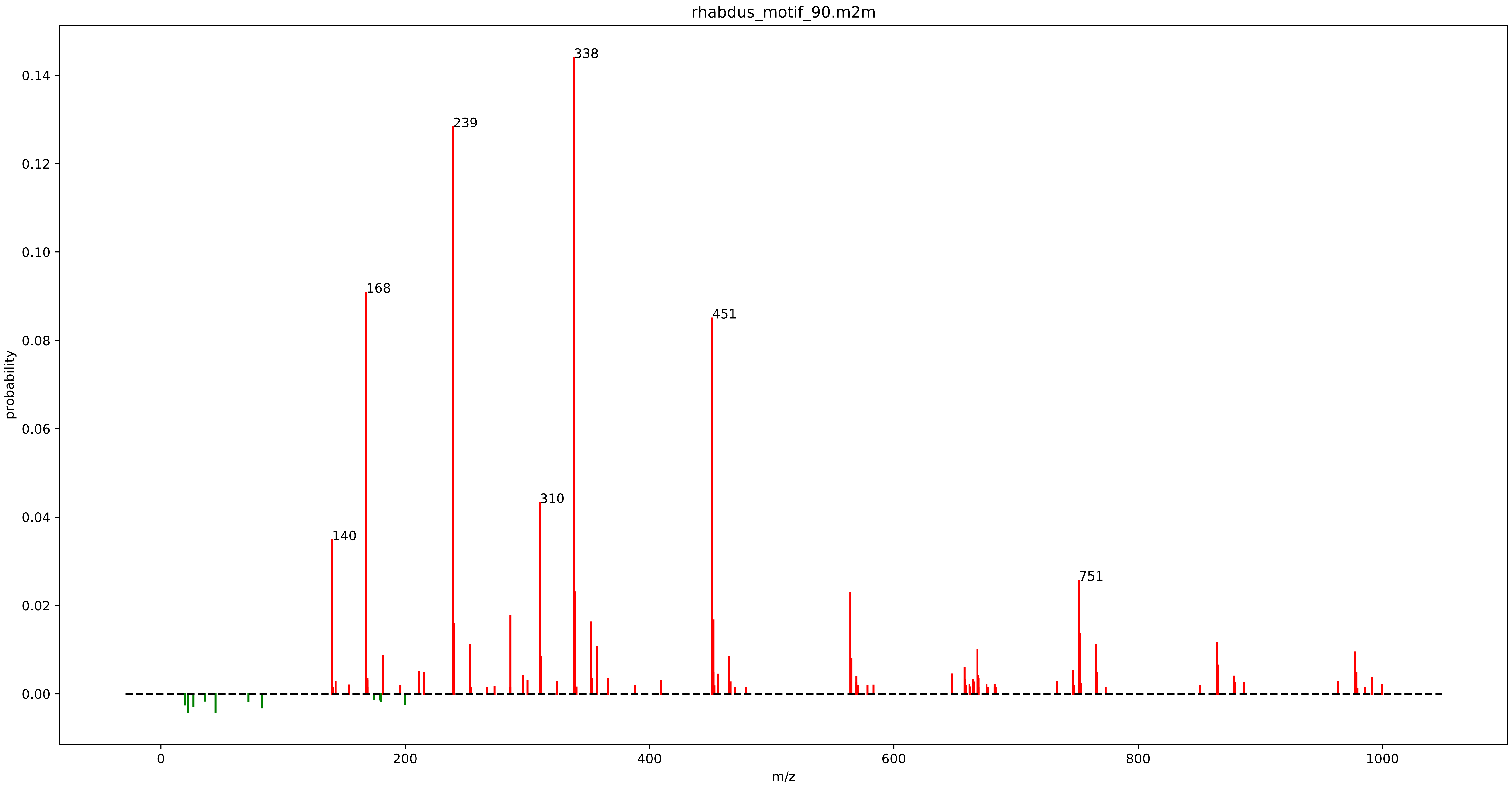

rhabdus_motif_90.m2m
probability
m/z
0.14
0.12
0.10
0.08
0.06
0.04
0.02
0.00
0
200
400
600
800
1000
338
239
168
451
310
140
751

rhabdus_motif_90.m2m

fragment_338.2075	: 0.14
fragment_239.1375	: 0.13
fragment_168.1025	: 0.09
fragment_451.2925	: 0.08
fragment_310.2125	: 0.04
fragment_140.1025	: 0.03
fragment_751.4725	: 0.03
fragment_339.2075	: 0.02
fragment_564.3775	: 0.02
fragment_286.1775	: 0.02
fragment_452.2925	: 0.02
fragment_352.2225	: 0.02
fragment_240.1425	: 0.02
fragment_752.4725	: 0.01
fragment_864.5525	: 0.01
fragment_765.4875	: 0.01
fragment_253.1525	: 0.01
fragment_357.2125	: 0.01
fragment_668.4325	: 0.01
fragment_977.6375	: 0.01
fragment_182.1175	: 0.01
fragment_465.3075	: 0.01
fragment_311.2125	: 0.01
fragment_310.2075	: 0.01
fragment_565.3775	: 0.01
fragment_865.5575	: 0.01
fragment_657.9225	: 0.01
fragment_746.4825	: 0.01
fragment_339.2125	: 0.01

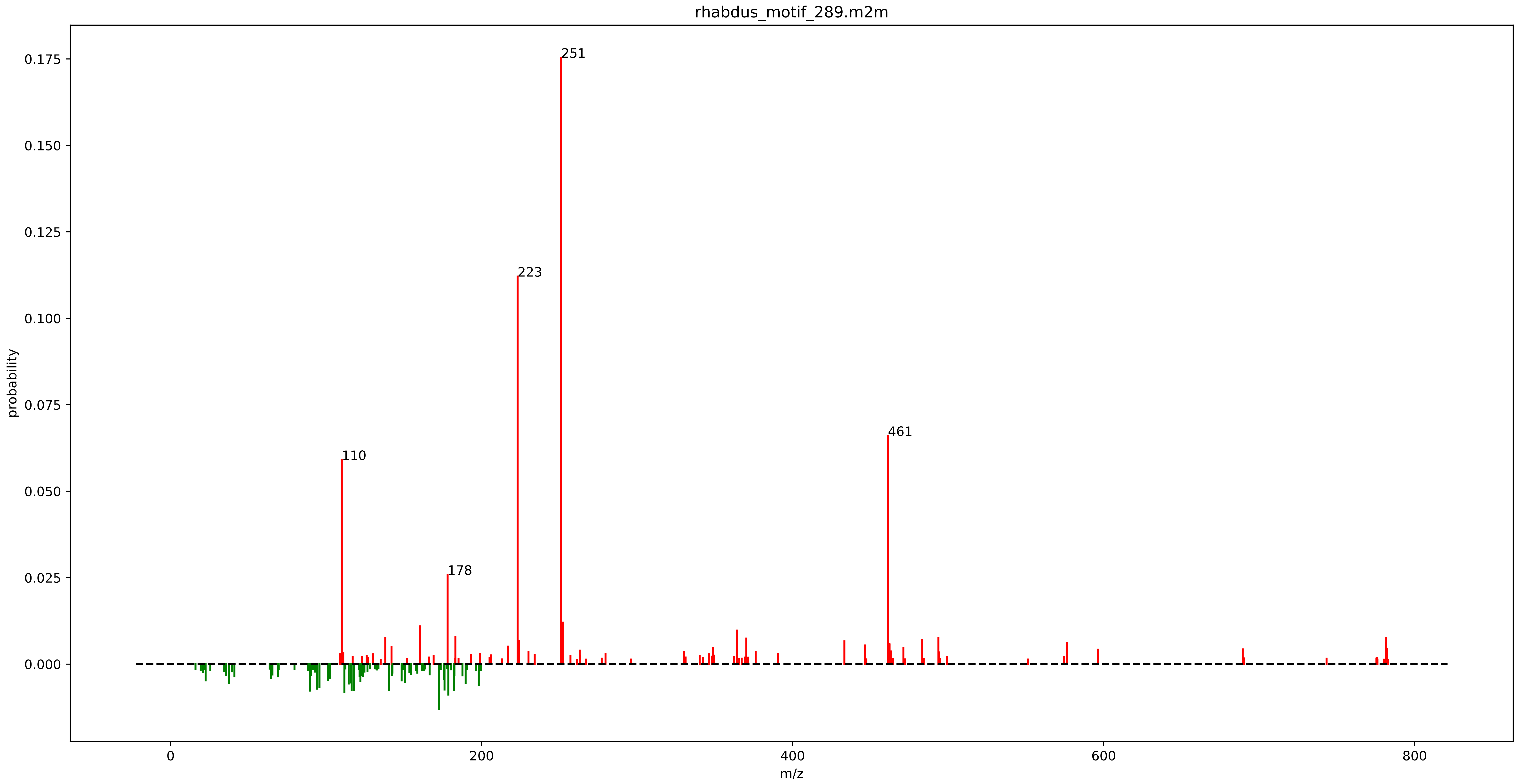

rhabdus_motif_289.m2m
probability
m/z
0.175
0.150
0.125
0.100
0.075
0.050
0.025
0.000
0
200
400
600
800
251
223
461
110
178

rhabdus_motif_289.m2m

fragment_251.1475	: 0.18
fragment_223.1525	: 0.11
fragment_461.2875	: 0.07
fragment_110.0725	: 0.06
fragment_178.1325	: 0.03
loss_172.6075	: 0.01
fragment_252.1525	: 0.01
fragment_160.6075	: 0.01
fragment_364.2325	: 0.01
loss_178.6075	: 0.01
loss_111.7725	: 0.01
fragment_183.1475	: 0.01
loss_89.7775	: 0.01
fragment_138.0675	: 0.01
loss_117.7625	: 0.01
fragment_781.7425	: 0.01
loss_182.1475	: 0.01
loss_116.4425	: 0.01
fragment_493.7575	: 0.01
loss_140.6375	: 0.01
fragment_370.2325	: 0.01
loss_176.1475	: 0.01
loss_94.0725	: 0.01
fragment_483.3225	: 0.01
fragment_224.1575	: 0.01
loss_94.4475	: 0.01
loss_95.7675	: 0.01
fragment_433.2925	: 0.01
fragment_781.4075	: 0.01
fragment_576.3475	: 0.01
loss_198.1475	: 0.01
fragment_462.2875	: 0.01
loss_114.5375	: 0.01
fragment_462.2925	: 0.01
loss_37.5275	: 0.01
loss_189.7175	: 0.01
fragment_446.4325	: 0.01
loss_116.0675	: 0.01
loss_150.6125	: 0.01
fragment_217.1275	: 0.01

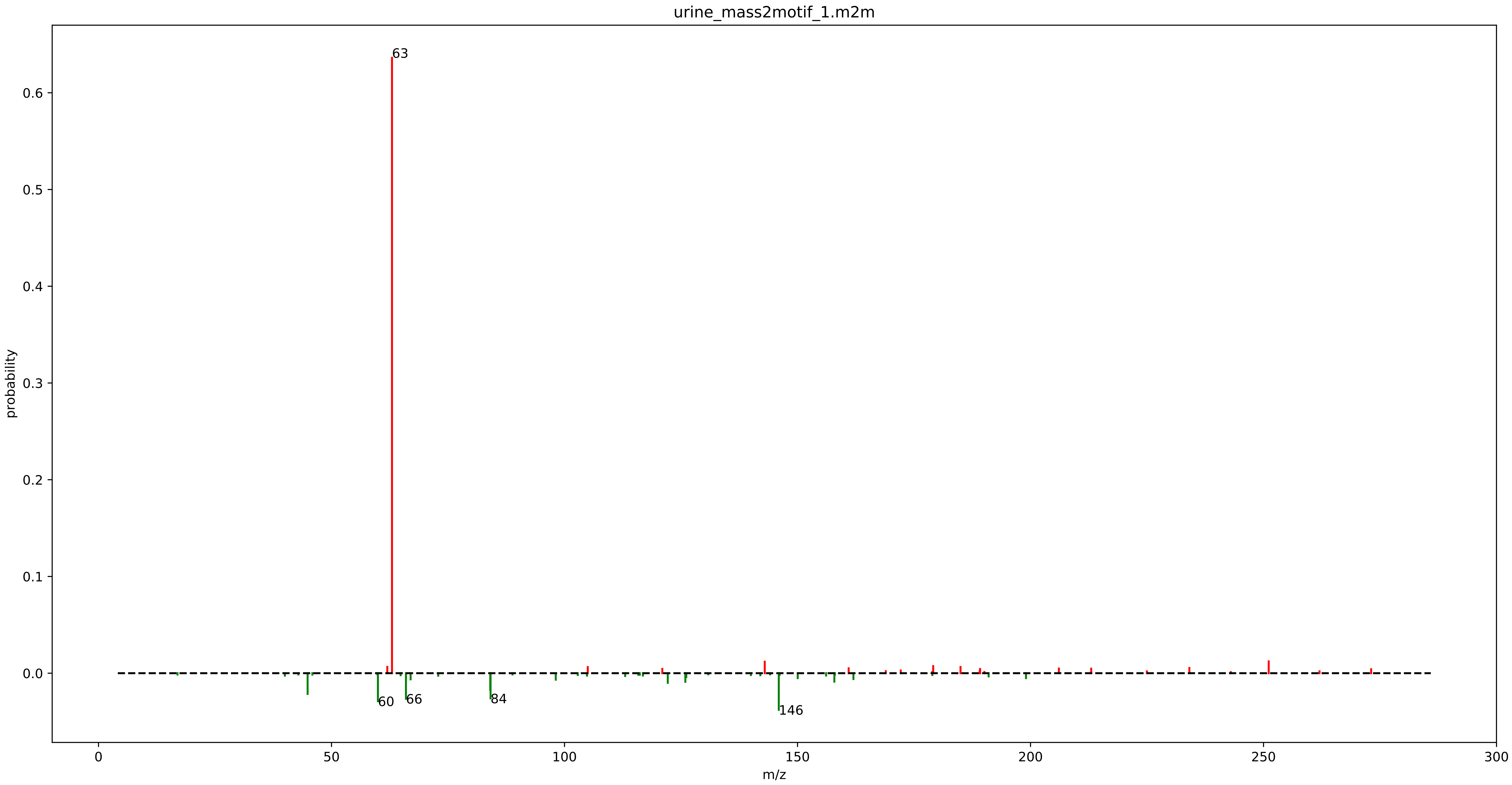

urine_mass2motif_1.m2m
probability
m/z
63
60
66
84
146
0.6
0.5
0.4
0.3
0.2
0.1
0.0
0
50
100
150
200
250
300

urine_mass2motif_1.m2m

fragment_62.9825	: 0.64
loss_145.9825	: 0.04
loss_59.9575	: 0.03
loss_65.9725	: 0.03
loss_84.1325	: 0.03
loss_44.8725	: 0.02
loss_84.0675	: 0.02
fragment_251.1125	: 0.01
fragment_142.9675	: 0.01
loss_122.1575	: 0.01
loss_125.9275	: 0.01
loss_157.9075	: 0.01
fragment_179.1125	: 0.01
loss_98.1225	: 0.01
fragment_61.9725	: 0.01
fragment_184.9775	: 0.01
fragment_104.9925	: 0.01
loss_66.9775	: 0.01
loss_161.9975	: 0.01
fragment_234.0875	: 0.01
loss_199.0325	: 0.01
loss_150.0425	: 0.01
fragment_160.9775	: 0.01

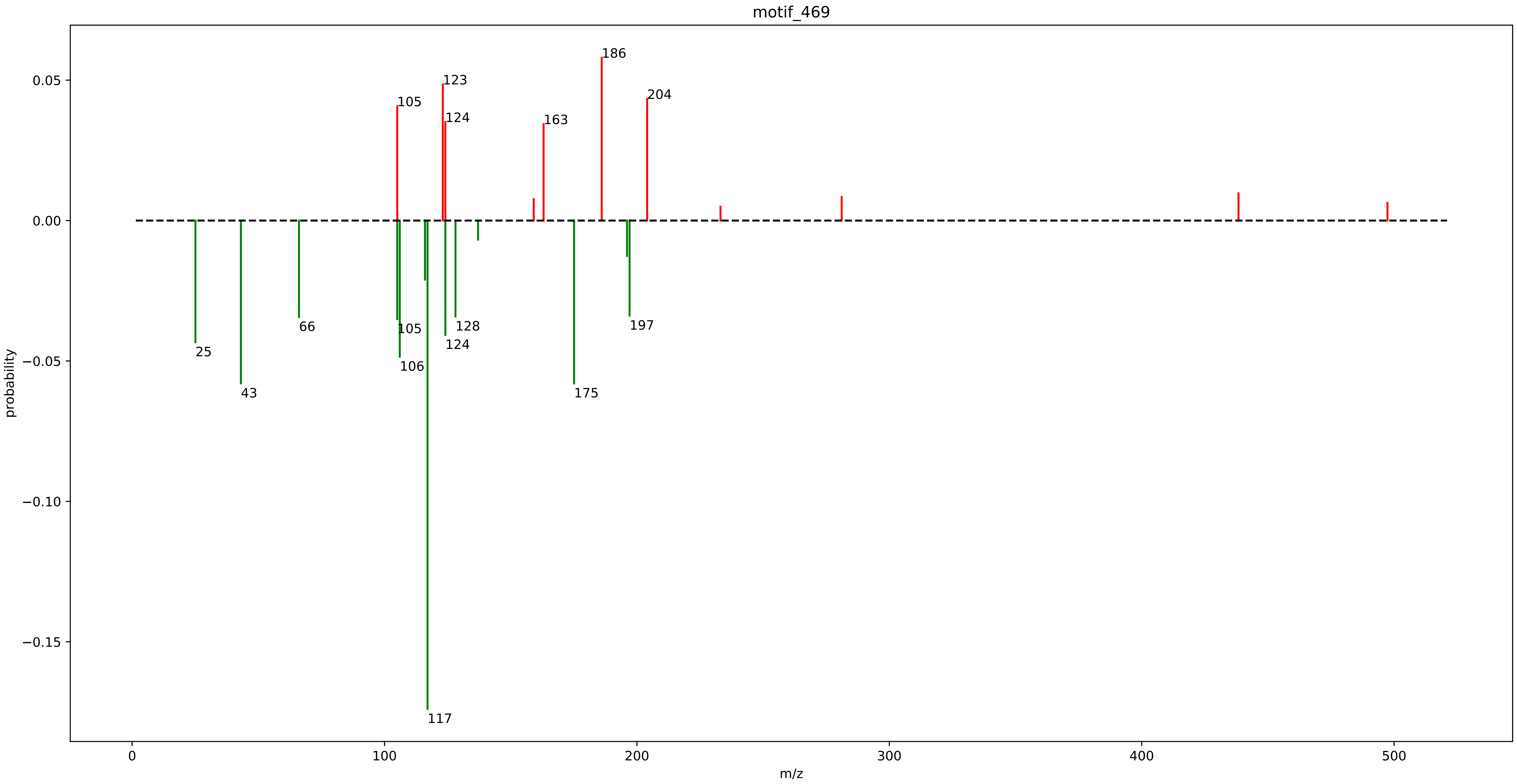

motif_469
probability
m/z
0.05
0.00
−0.05
−0.10
−0.15
0
100
200
300
400
500
186
123
204
105
124
163
25
43
66
105
106
117
124
128
175
197

motif_469

loss_117.0325	: 0.17
fragment_186.0375	: 0.06
loss_43.0925	: 0.06
loss_175.1025	: 0.06
fragment_123.0875	: 0.05
loss_106.0425	: 0.05
loss_25.0825	: 0.04
fragment_204.0475	: 0.04
fragment_105.0325	: 0.04
loss_124.0975	: 0.04
fragment_124.0975	: 0.04
loss_105.0325	: 0.04
loss_66.1075	: 0.03
fragment_163.0225	: 0.03
loss_128.1075	: 0.03
loss_197.0875	: 0.03
loss_116.0275	: 0.02
loss_196.0825	: 0.01
fragment_438.3475	: 0.01
fragment_281.1125	: 0.01
fragment_159.1175	: 0.01
loss_137.0625	: 0.01
fragment_497.3625	: 0.01

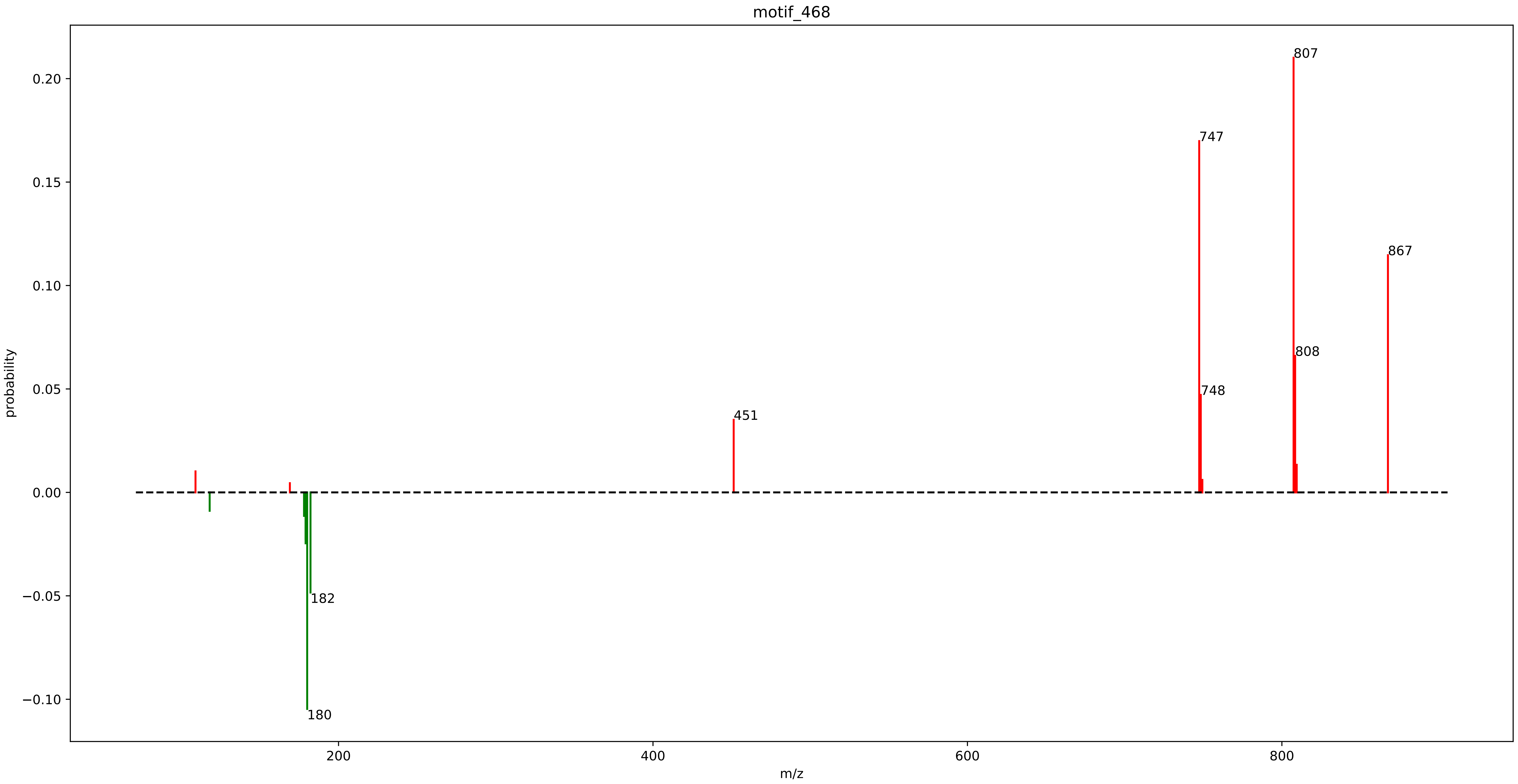
motif_468
probability
m/z
0.20
0.15
0.10
0.05
0.00
−0.05
−0.10
200
400
600
800
807
747
867
808
748
451
182
180

motif_468

fragment_807.4275	: 0.21
fragment_747.4075	: 0.17
fragment_867.4525	: 0.11
loss_180.0575	: 0.10
fragment_808.4325	: 0.07
loss_182.1675	: 0.05
fragment_748.4125	: 0.05
fragment_451.3225	: 0.04
loss_179.0525	: 0.02
loss_179.0575	: 0.02
fragment_809.4375	: 0.01
loss_178.0525	: 0.01
fragment_109.0275	: 0.01
loss_118.0325	: 0.01
fragment_749.4175	: 0.01
fragment_749.4125	: 0.01

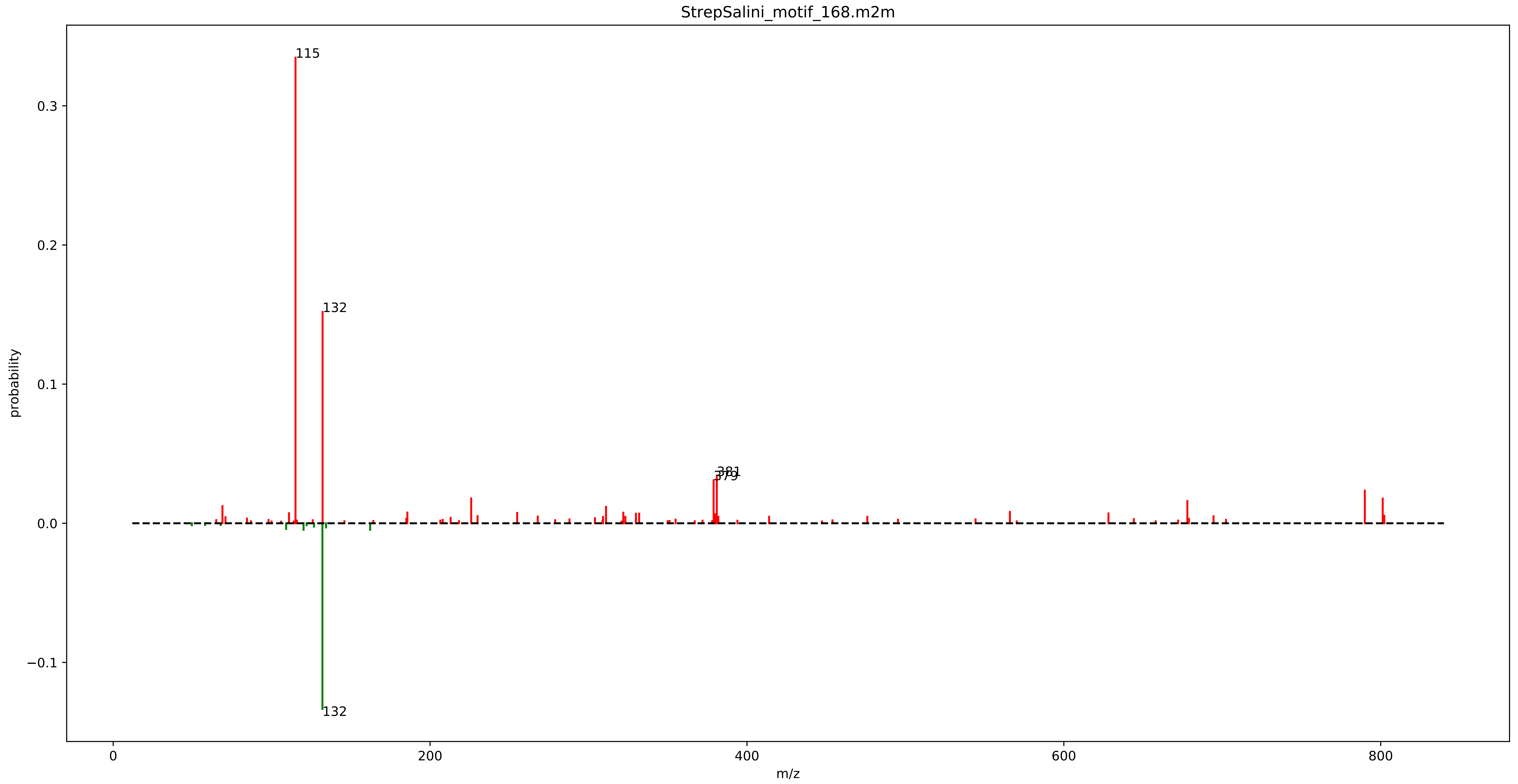

StrepSalini_motif_168.m2m
probability
m/z
115
132
381
379
132
0.3
0.2
0.1
0.0
−0.1
0
200
400
600
800

StrepSalini_motif_168.m2m

fragment_115.0500	: 0.33
fragment_132.1500	: 0.15
loss_132.0500	: 0.13
fragment_380.9500	: 0.03
fragment_378.9500	: 0.03
fragment_789.9500	: 0.02
fragment_225.9500	: 0.02
fragment_801.2500	: 0.02
fragment_677.9500	: 0.02
fragment_68.9500	: 0.01
fragment_311.0500	: 0.01
fragment_565.9500	: 0.01
fragment_185.6500	: 0.01
fragment_321.9500	: 0.01
fragment_254.9500	: 0.01
fragment_110.9500	: 0.01
fragment_628.1500	: 0.01
fragment_331.9500	: 0.01
fragment_329.9500	: 0.01
fragment_379.9500	: 0.01
fragment_802.2500	: 0.01
fragment_229.9500	: 0.01
fragment_694.4500	: 0.01

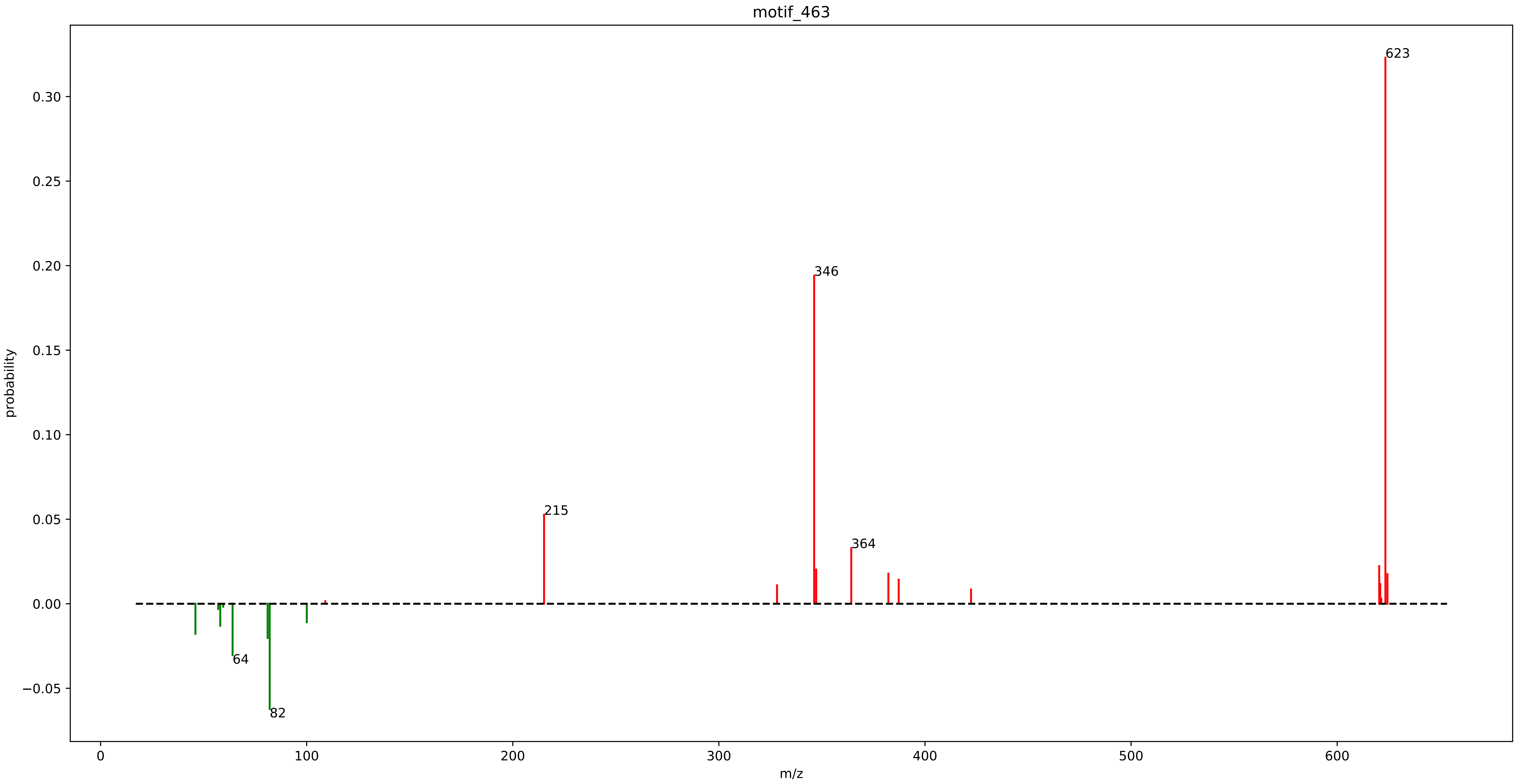
motif_463
probability
m/z
0.30
0.25
0.20
0.15
0.10
0.05
0.00
−0.05
0
100
200
300
400
500
600
623
346
215
364
64
82

motif_463

fragment_623.3925	: 0.32
fragment_346.2225	: 0.19
loss_82.0525	: 0.06
fragment_215.1775	: 0.05
fragment_364.2325	: 0.03
loss_64.0425	: 0.03
fragment_620.3825	: 0.02
loss_81.0475	: 0.02
fragment_347.2225	: 0.02
loss_46.0275	: 0.02
fragment_382.2425	: 0.02
fragment_624.3975	: 0.02
fragment_387.2475	: 0.01
loss_58.0725	: 0.01
fragment_620.8875	: 0.01
loss_100.0625	: 0.01
fragment_328.2125	: 0.01
fragment_422.3475	: 0.01

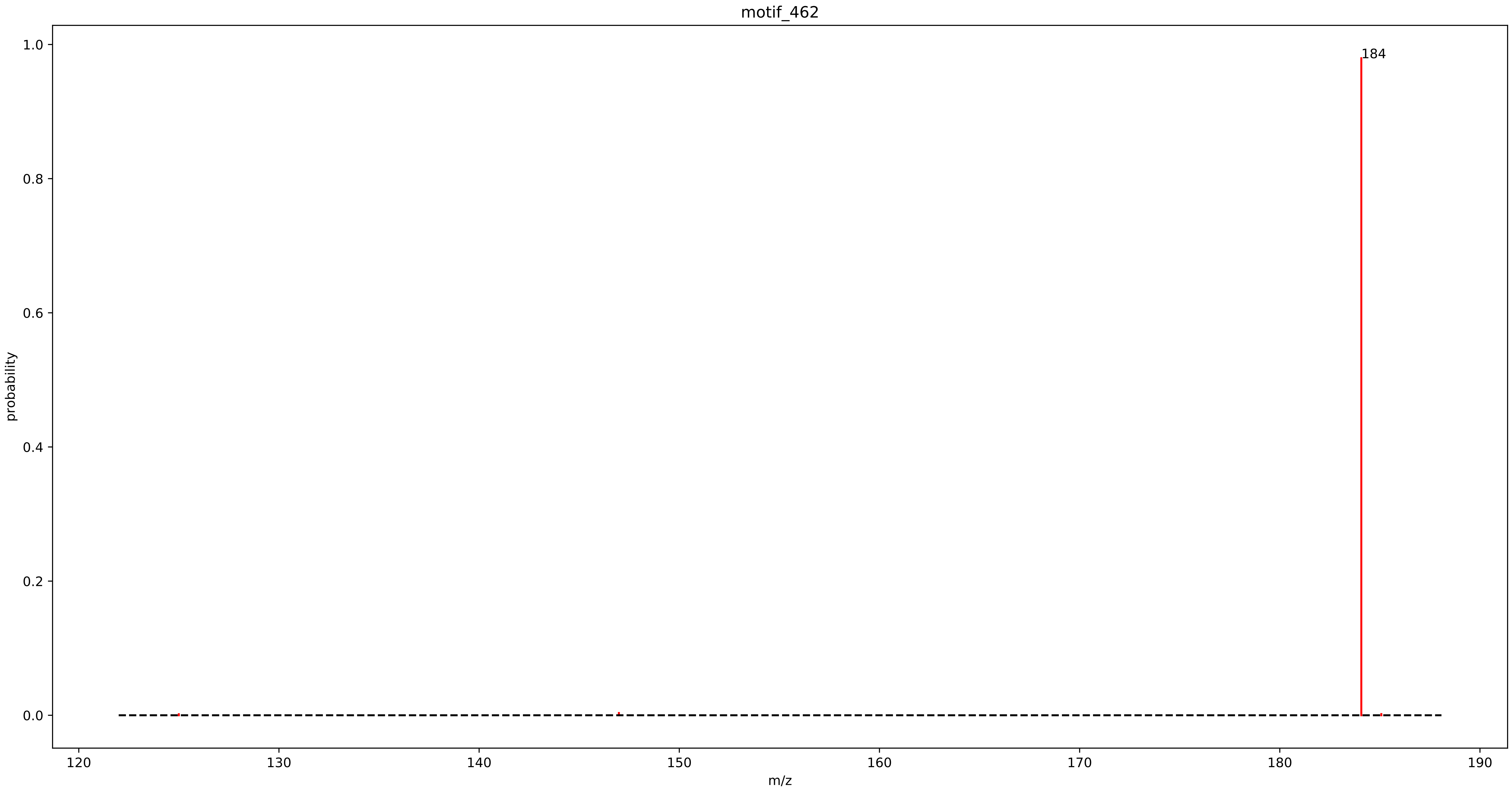
motif_462
184
probability
m/z
1.0
0.8
0.6
0.4
0.2
0.0
120
130
140
150
160
170
180
190

motif_462

fragment_184.0725 : 0.98

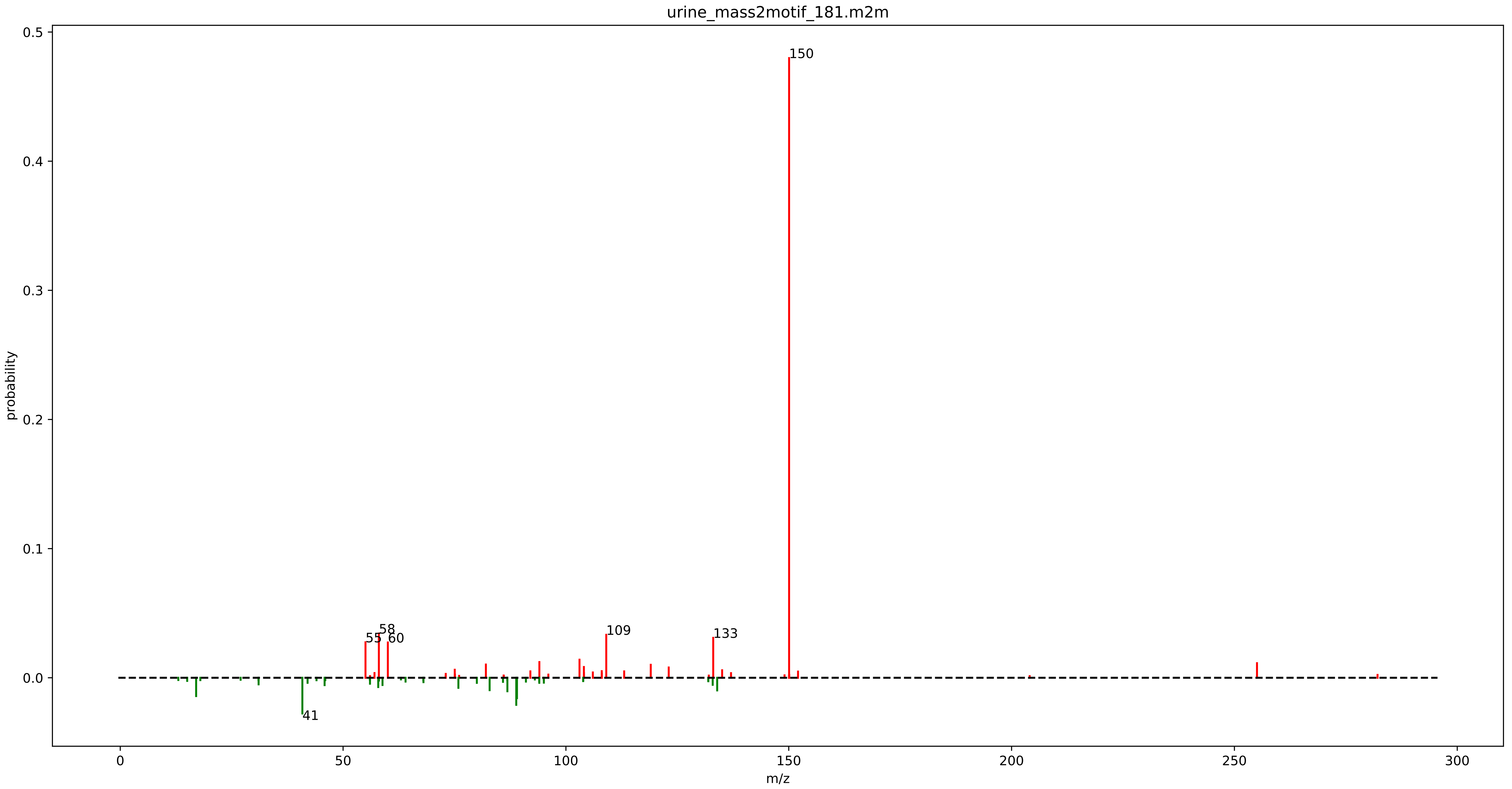

urine_mass2motif_181.m2m
probability
m/z
0.5
0.4
0.3
0.2
0.1
0.0
0
50
100
150
200
250
300
150
58
55
60
109
133
41

urine_mass2motif_181.m2m

fragment_150.0775 : 0.48
fragment_58.0275 : 0.03
fragment_109.0525 : 0.03
fragment_133.0525 : 0.03
loss_40.8625 : 0.03
fragment_55.0275 : 0.03
fragment_60.0425 : 0.03
loss_88.8575 : 0.02
loss_89.0175 : 0.02
loss_17.0275 : 0.01
fragment_103.0475 : 0.01
fragment_94.0425 : 0.01
fragment_255.0675 : 0.01
loss_86.8675 : 0.01
fragment_82.0425 : 0.01
fragment_119.0375 : 0.01
loss_133.9425 : 0.01
loss_82.8825 : 0.01
fragment_104.0325 : 0.01
fragment_123.0675 : 0.01
loss_75.8725 : 0.01
loss_57.8875 : 0.01
fragment_75.0575 : 0.01
fragment_135.0525 : 0.01
loss_45.8525 : 0.01
loss_58.8475 : 0.01
loss_132.9375 : 0.01
fragment_108.0425 : 0.01
loss_31.0425 : 0.01

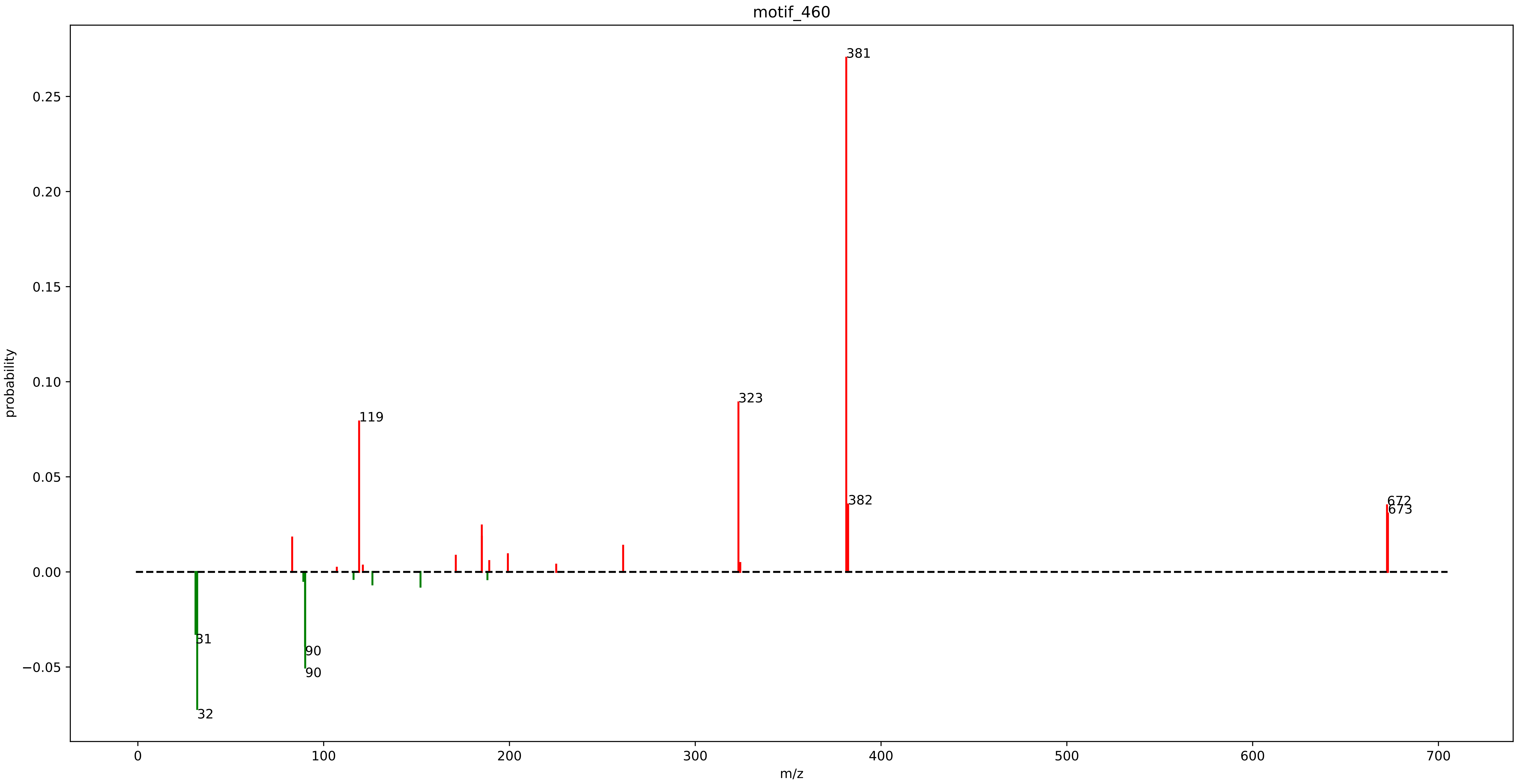

motif_460
probability
m/z
0.25
0.20
0.15
0.10
0.05
0.00
−0.05
0
100
200
300
400
500
600
700
381
323
119
382
672
673
31
90
90
32

motif_460

fragment_381.2775	: 0.27
fragment_323.2375	: 0.09
fragment_119.0825	: 0.08
loss_31.9375	: 0.07
loss_90.0675	: 0.05
loss_89.9825	: 0.04
fragment_382.2825	: 0.04
fragment_672.3175	: 0.04
loss_31.0225	: 0.03
fragment_672.8175	: 0.03
fragment_185.1125	: 0.02
fragment_185.1325	: 0.02
fragment_83.0475	: 0.02
fragment_261.1825	: 0.01
fragment_199.1475	: 0.01
fragment_171.1175	: 0.01
loss_152.1175	: 0.01
loss_126.2425	: 0.01
fragment_189.1275	: 0.01

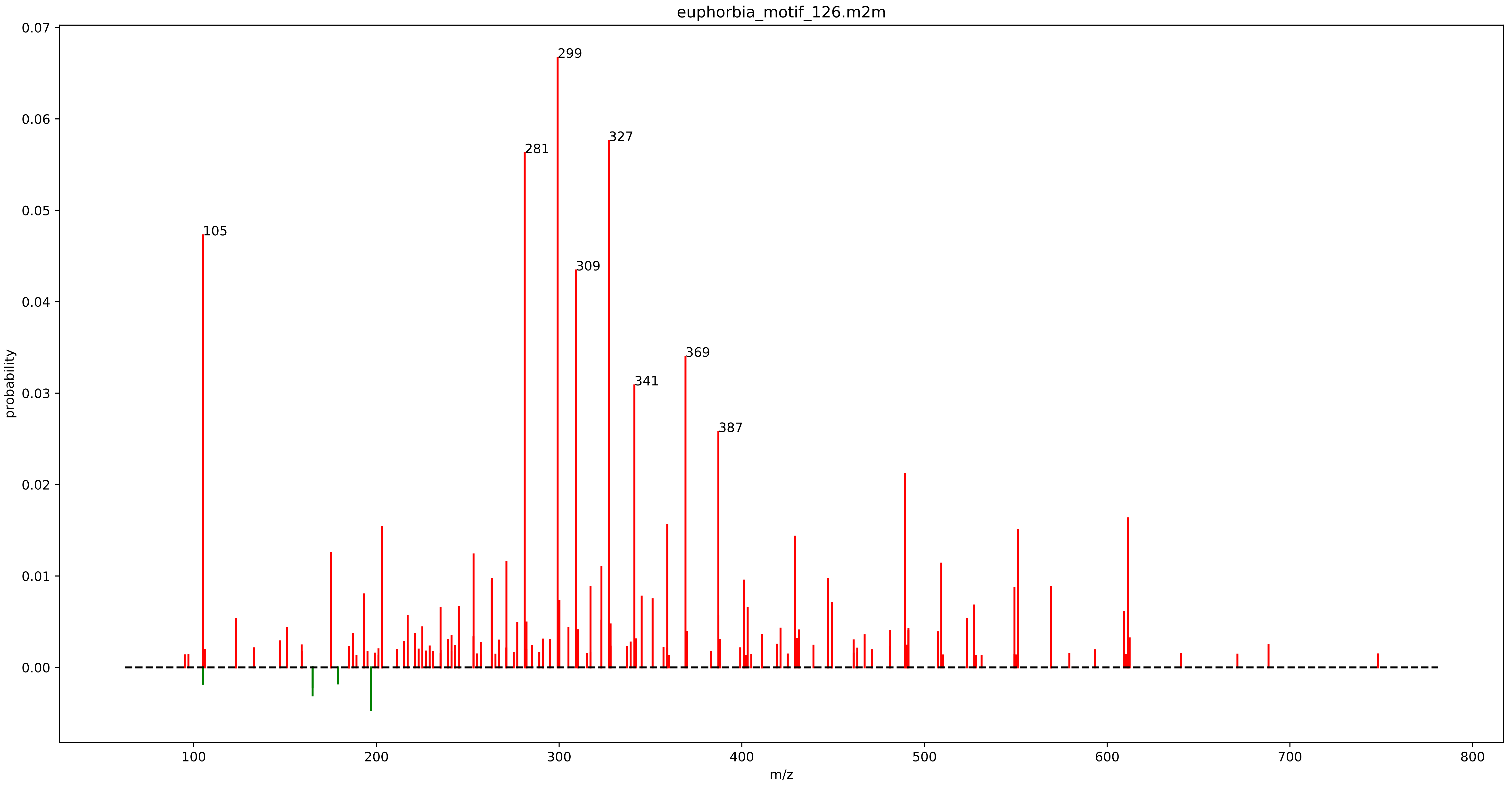
euphorbia_motif_126.m2m
probability
m/z
0.07
0.06
0.05
0.04
0.03
0.02
0.01
0.00
100
200
300
400
500
600
700
800
105
281
299
309
327
341
369
387

euphorbia_motif_126.m2m

fragment_299.1625	: 0.07	fragment_263.1425	: 0.01	fragment_523.2325	: 0.01
fragment_327.1575	: 0.06	fragment_447.2025	: 0.01	fragment_123.0775	: 0.01
fragment_281.1525	: 0.06	fragment_401.1925	: 0.01	fragment_323.1125	: 0.01
fragment_105.0325	: 0.05	fragment_263.0925	: 0.01	fragment_245.0825	: 0.01
fragment_309.1475	: 0.04	fragment_317.1725	: 0.01		
fragment_369.1675	: 0.03	fragment_569.2375	: 0.01		
fragment_341.1725	: 0.03	fragment_549.2325	: 0.01		
fragment_387.1775	: 0.03	fragment_193.0875	: 0.01		
fragment_489.2125	: 0.02	fragment_345.1675	: 0.01		
fragment_611.2475	: 0.02	fragment_351.1575	: 0.01		
fragment_359.1825	: 0.02	fragment_300.1675	: 0.01		
fragment_203.0675	: 0.02	fragment_449.1925	: 0.01		
fragment_551.2275	: 0.02	fragment_527.2275	: 0.01		
fragment_429.1925	: 0.01	fragment_245.0775	: 0.01		
fragment_429.1875	: 0.01	fragment_235.0975	: 0.01		
fragment_175.0725	: 0.01	fragment_403.1875	: 0.01		
fragment_253.1575	: 0.01	fragment_609.2675	: 0.01		
fragment_271.1675	: 0.01	fragment_401.1975	: 0.01		
fragment_509.2175	: 0.01	fragment_217.0875	: 0.01		
fragment_323.1625	: 0.01	fragment_387.1825	: 0.01		

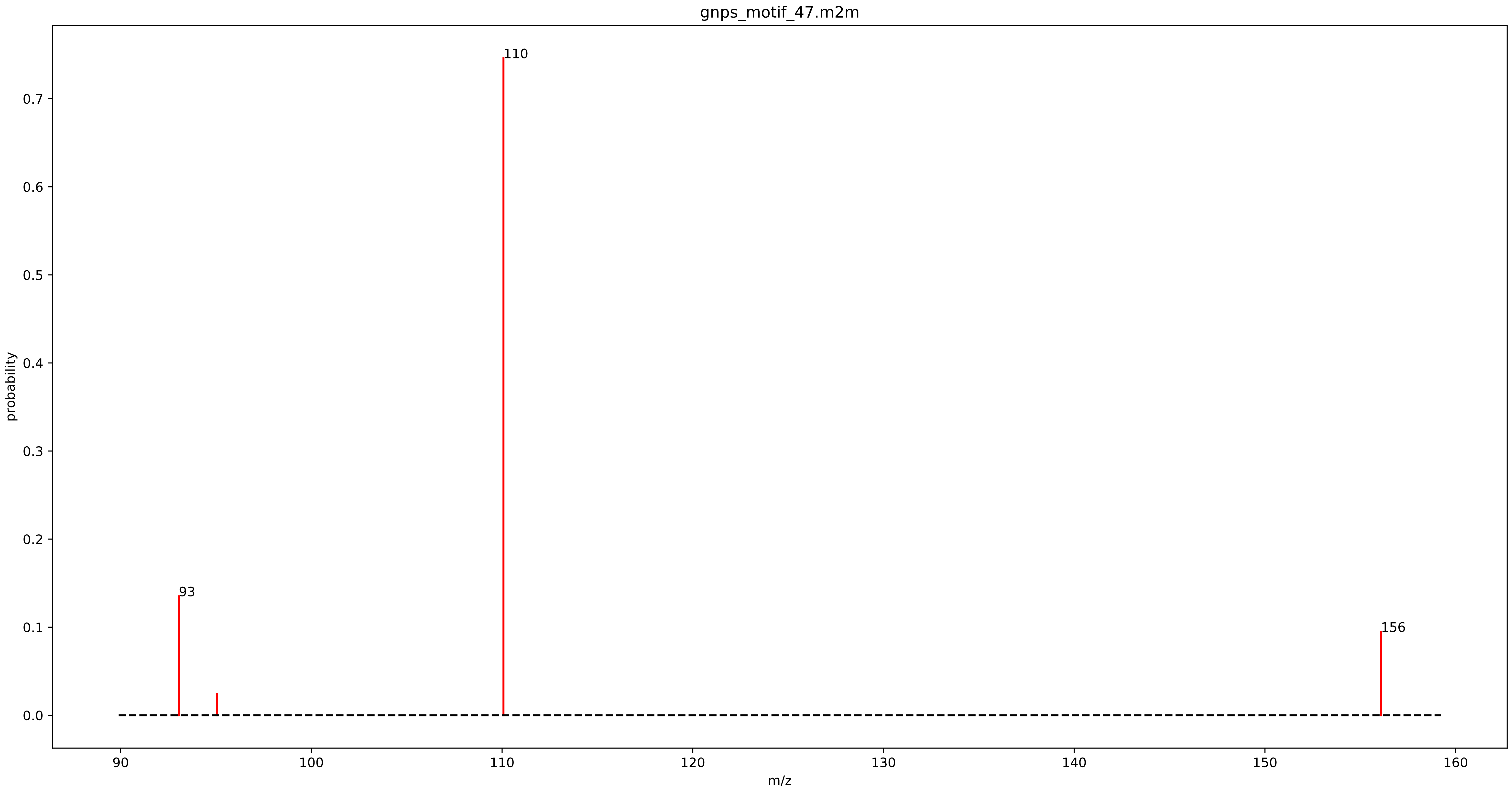

gnps_motif_47.m2m
probability
m/z
0.0
0.1
0.2
0.3
0.4
0.5
0.6
0.7
90
100
110
120
130
140
150
160
93
110
156

gnps_motif_47.m2m

fragment_110.0725	: 0.75
fragment_93.0475	: 0.14
fragment_156.0775	: 0.09
fragment_95.0625	: 0.02

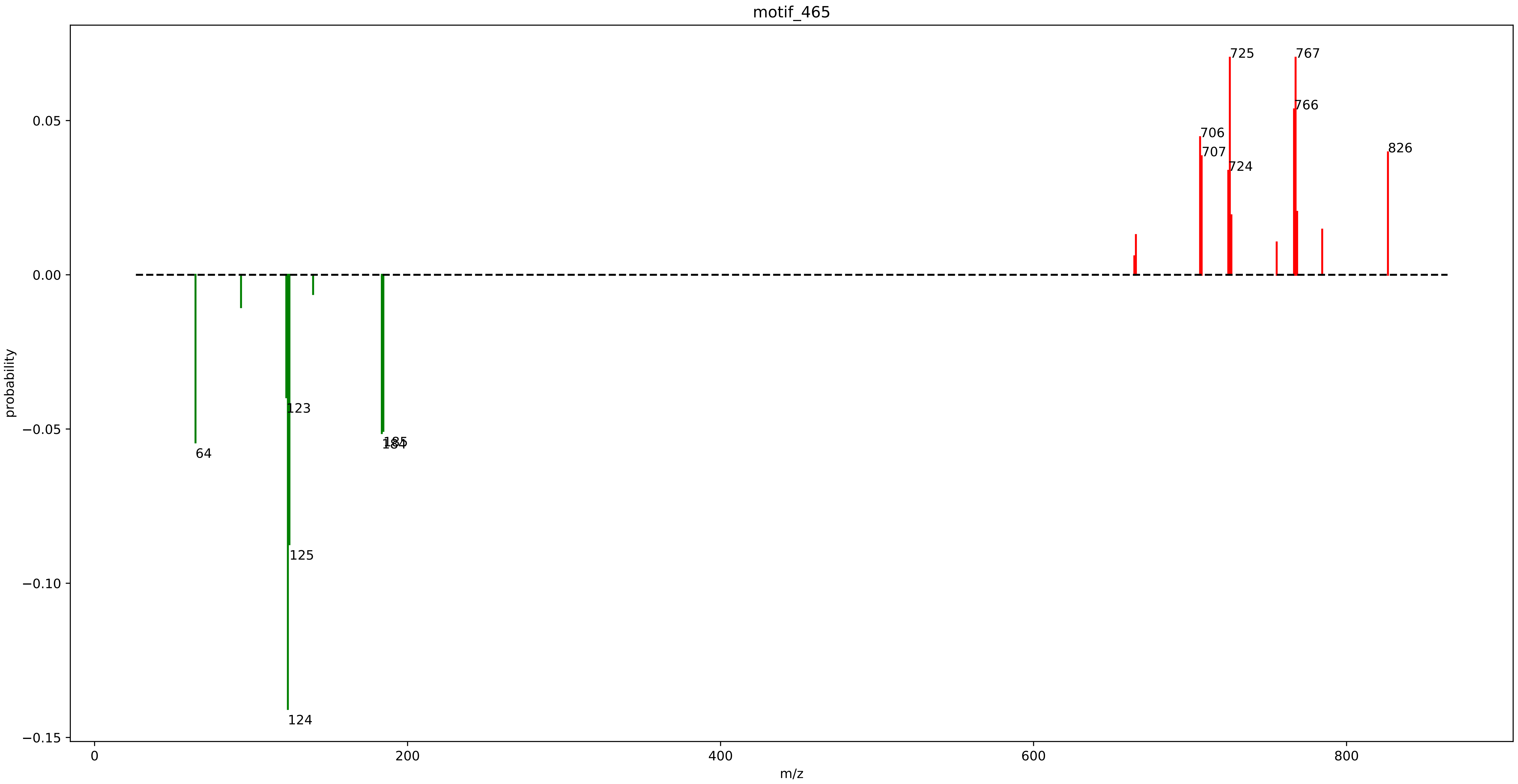

motif_465
725
767
766
706
707
724
826
0.05
0.00
−0.05
−0.10
−0.15
probability
123
64
185
184
125
124
0
200
400
600
800
m/z

motif_465

loss_123.5125	: 0.14
loss_124.5175	: 0.09
fragment_767.4275	: 0.07
fragment_725.4125	: 0.07
loss_64.4925	: 0.05
fragment_766.4225	: 0.05
loss_183.5325	: 0.05
loss_184.5375	: 0.05
fragment_706.4025	: 0.04
fragment_826.4425	: 0.04
loss_122.5075	: 0.04
fragment_707.4075	: 0.04
fragment_724.4125	: 0.03
fragment_768.4275	: 0.02
fragment_726.4175	: 0.02
fragment_784.4325	: 0.01
fragment_665.3925	: 0.01
loss_93.5825	: 0.01
fragment_755.3475	: 0.01
loss_139.6375	: 0.01
fragment_664.3875	: 0.01

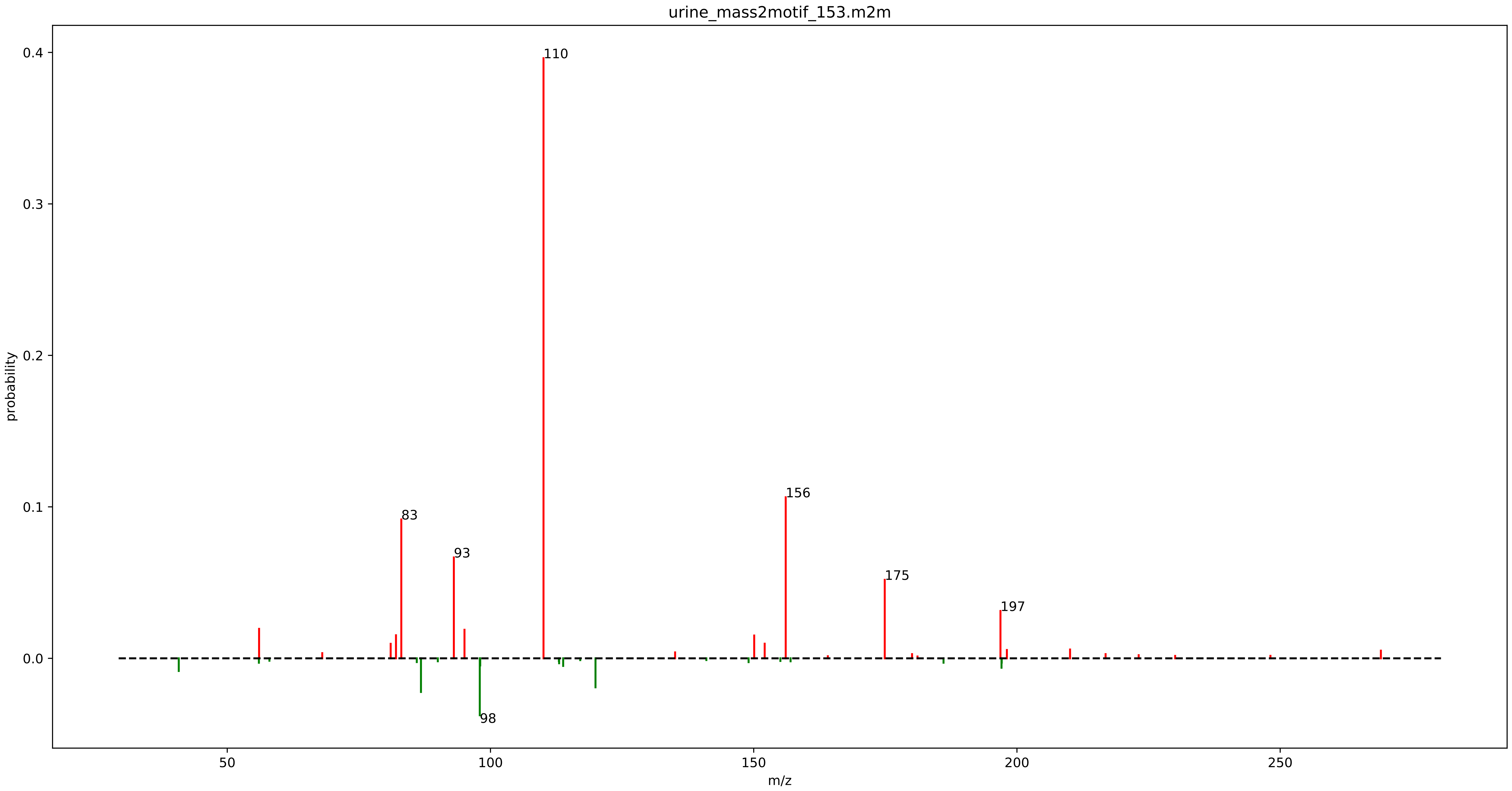
urine_mass2motif_153.m2m
probability
m/z
0.4
0.3
0.2
0.1
0.0
50
100
150
200
250
110
156
83
93
175
197
98

urine_mass2motif_153.m2m

fragment_110.0725	: 0.40
fragment_156.0775	: 0.11
fragment_83.0625	: 0.09
fragment_93.0425	: 0.07
fragment_174.8875	: 0.05
loss_97.9675	: 0.04
fragment_196.8675	: 0.03
loss_86.7975	: 0.02
fragment_56.0475	: 0.02
loss_119.9475	: 0.02
fragment_95.0625	: 0.02
fragment_82.0525	: 0.02
fragment_150.0925	: 0.02
fragment_152.0825	: 0.01
fragment_81.0425	: 0.01
loss_40.7925	: 0.01
loss_197.0675	: 0.01
fragment_210.0875	: 0.01
fragment_198.0875	: 0.01
fragment_269.1275	: 0.01
loss_113.8075	: 0.01

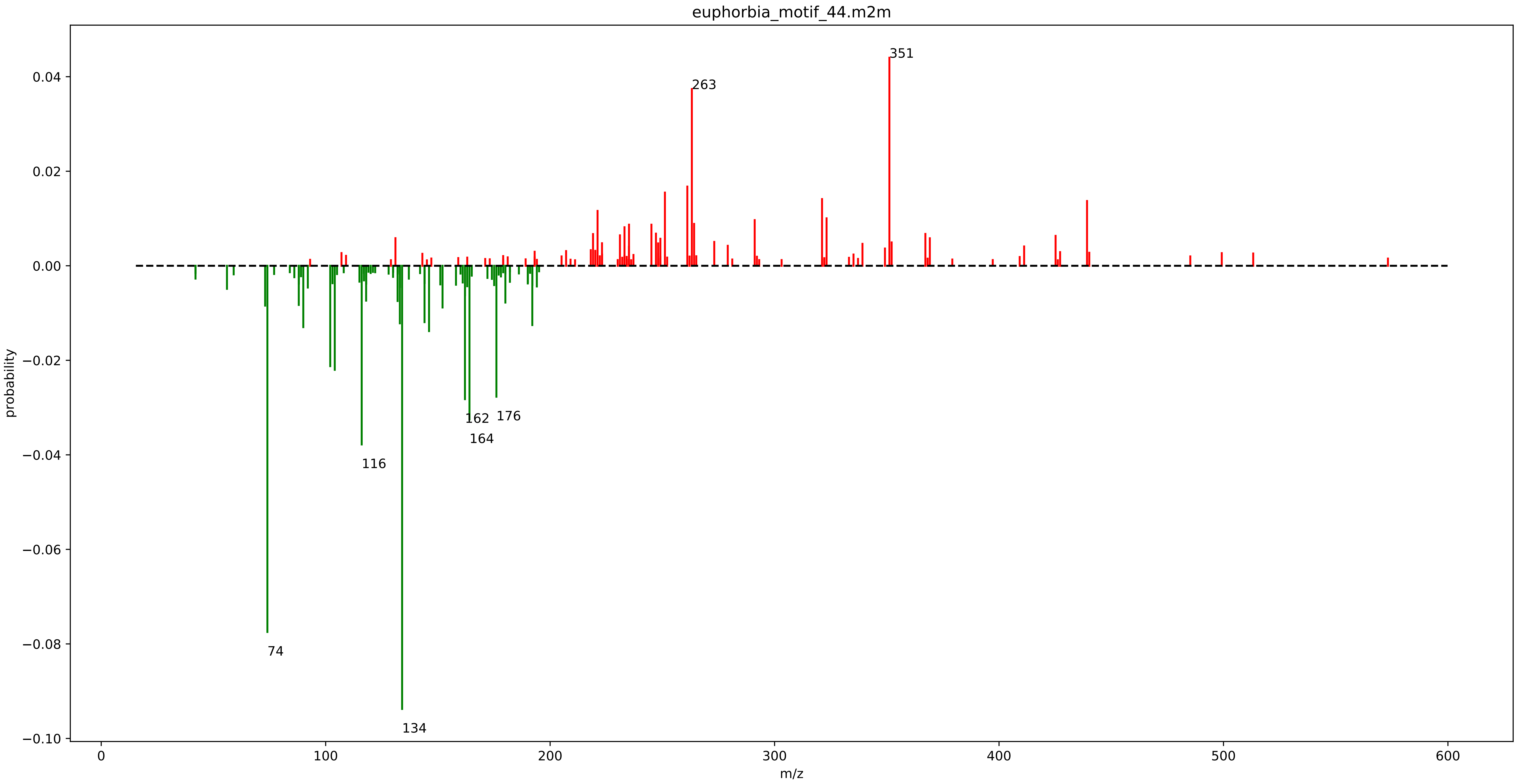

euphorbia_motif_44.m2m
probability
m/z
351
263
74
134
116
162
164
176
0.04
0.02
0.00
−0.02
−0.04
−0.06
−0.08
−0.10
0
100
200
300
400
500
600

euphorbia_motif_44.m2m

loss_134.0575	: 0.09
loss_74.0375	: 0.08
fragment_351.1575	: 0.04
loss_116.0475	: 0.04
fragment_263.1425	: 0.04
loss_164.0675	: 0.03
loss_162.0525	: 0.03
loss_176.0675	: 0.03
loss_104.0475	: 0.02
loss_102.0325	: 0.02
fragment_261.1275	: 0.02
fragment_251.1425	: 0.02
fragment_321.1475	: 0.01
loss_146.0575	: 0.01
fragment_439.2125	: 0.01
loss_90.0325	: 0.01
loss_192.0625	: 0.01
loss_133.0525	: 0.01
loss_144.0425	: 0.01
fragment_221.1325	: 0.01
fragment_323.1625	: 0.01
fragment_291.1375	: 0.01
fragment_264.1475	: 0.01
loss_152.0675	: 0.01
fragment_235.1475	: 0.01
fragment_245.1325	: 0.01
loss_73.0325	: 0.01
loss_88.0175	: 0.01
fragment_233.1325	: 0.01
loss_180.0625	: 0.01
loss_132.0425	: 0.01
loss_118.0275	: 0.01
fragment_247.1475	: 0.01
fragment_367.1875	: 0.01
fragment_219.1175	: 0.01
fragment_231.1025	: 0.01
fragment_425.1975	: 0.01
loss_162.0875	: 0.01
fragment_131.0875	: 0.01
fragment_369.1675	: 0.01
fragment_249.1225	: 0.01
fragment_273.1275	: 0.01

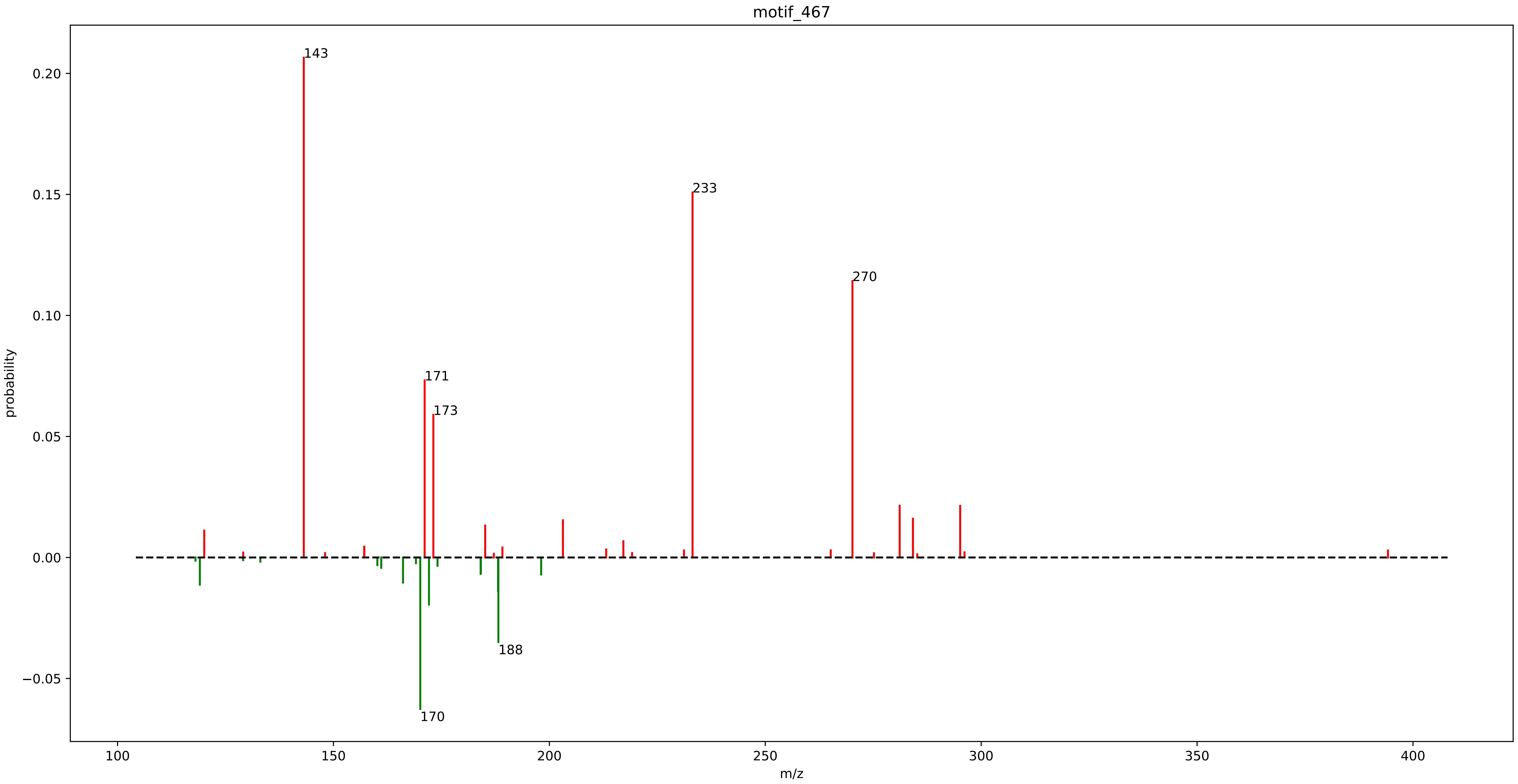

motif_467
probability
m/z
143
233
270
171
173
188
170
0.20
0.15
0.10
0.05
0.00
−0.05
100
150
200
250
300
350
400

motif_467

fragment_143.1175	: 0.21
fragment_233.1475	: 0.15
fragment_270.1825	: 0.11
fragment_171.1125	: 0.07
loss_170.1025	: 0.06
fragment_173.1275	: 0.06
loss_188.1925	: 0.03
fragment_281.1125	: 0.02
fragment_295.1275	: 0.02
loss_172.1175	: 0.02
fragment_284.1975	: 0.02
fragment_203.1375	: 0.02
loss_188.1125	: 0.01
fragment_185.1325	: 0.01
loss_119.0575	: 0.01
fragment_120.0725	: 0.01
loss_166.1075	: 0.01
loss_198.0975	: 0.01
loss_184.0825	: 0.01
fragment_217.1275	: 0.01
loss_184.1375	: 0.01

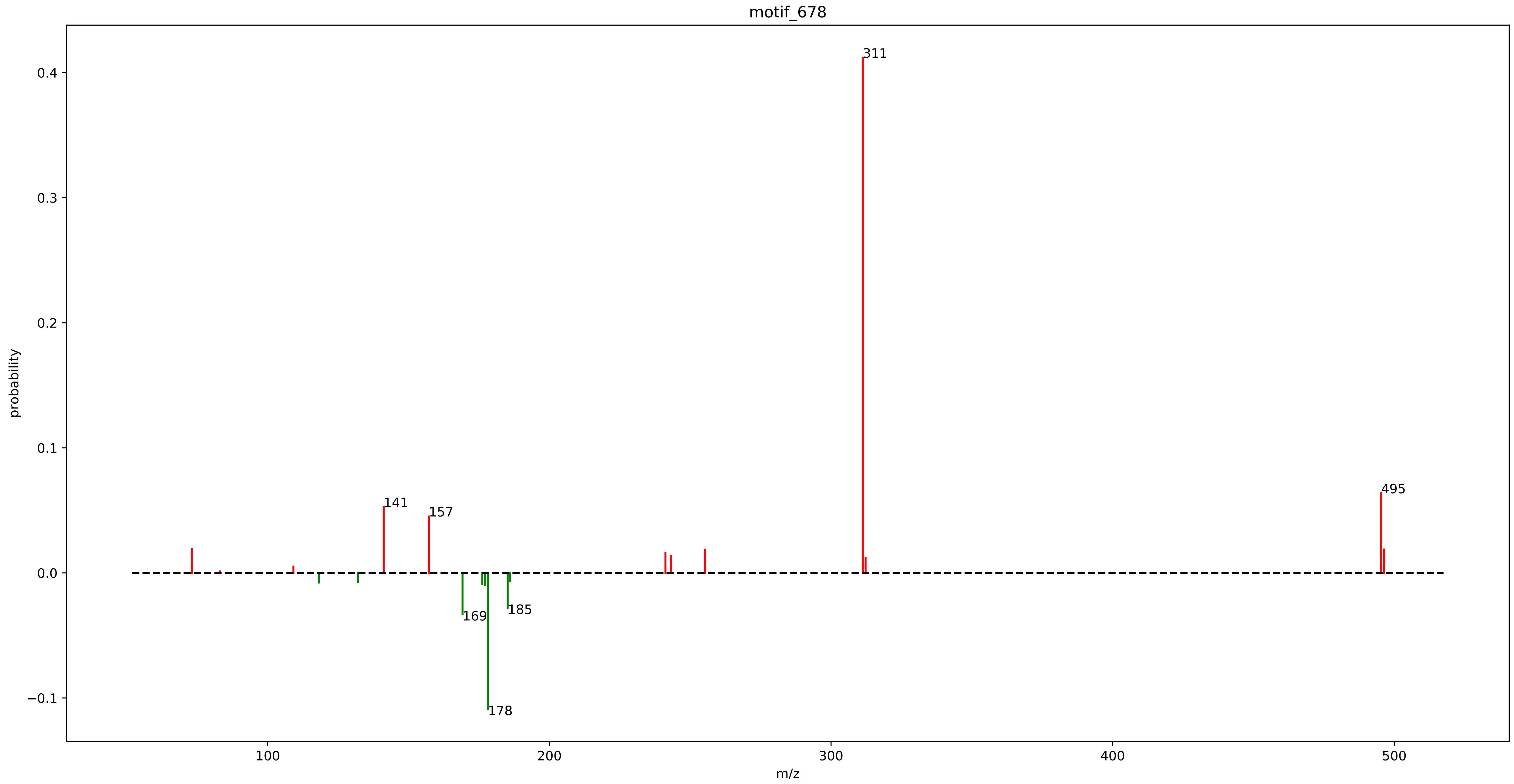

motif_678
311
495
141
157
169
185
178
0.4
0.3
0.2
0.1
0.0
−0.1
probability
100
200
300
400
500
m/z

motif_678

fragment_311.2725	: 0.41
loss_178.1775	: 0.11
fragment_495.3425	: 0.06
fragment_141.1375	: 0.05
fragment_157.1675	: 0.05
loss_169.1575	: 0.03
loss_185.1875	: 0.03
fragment_73.0275	: 0.02
fragment_496.3475	: 0.02
fragment_255.2125	: 0.02
fragment_241.1975	: 0.02
fragment_243.2125	: 0.01
fragment_312.2775	: 0.01
loss_177.1775	: 0.01
loss_176.1825	: 0.01
loss_118.1575	: 0.01
loss_132.0325	: 0.01
loss_186.0825	: 0.01

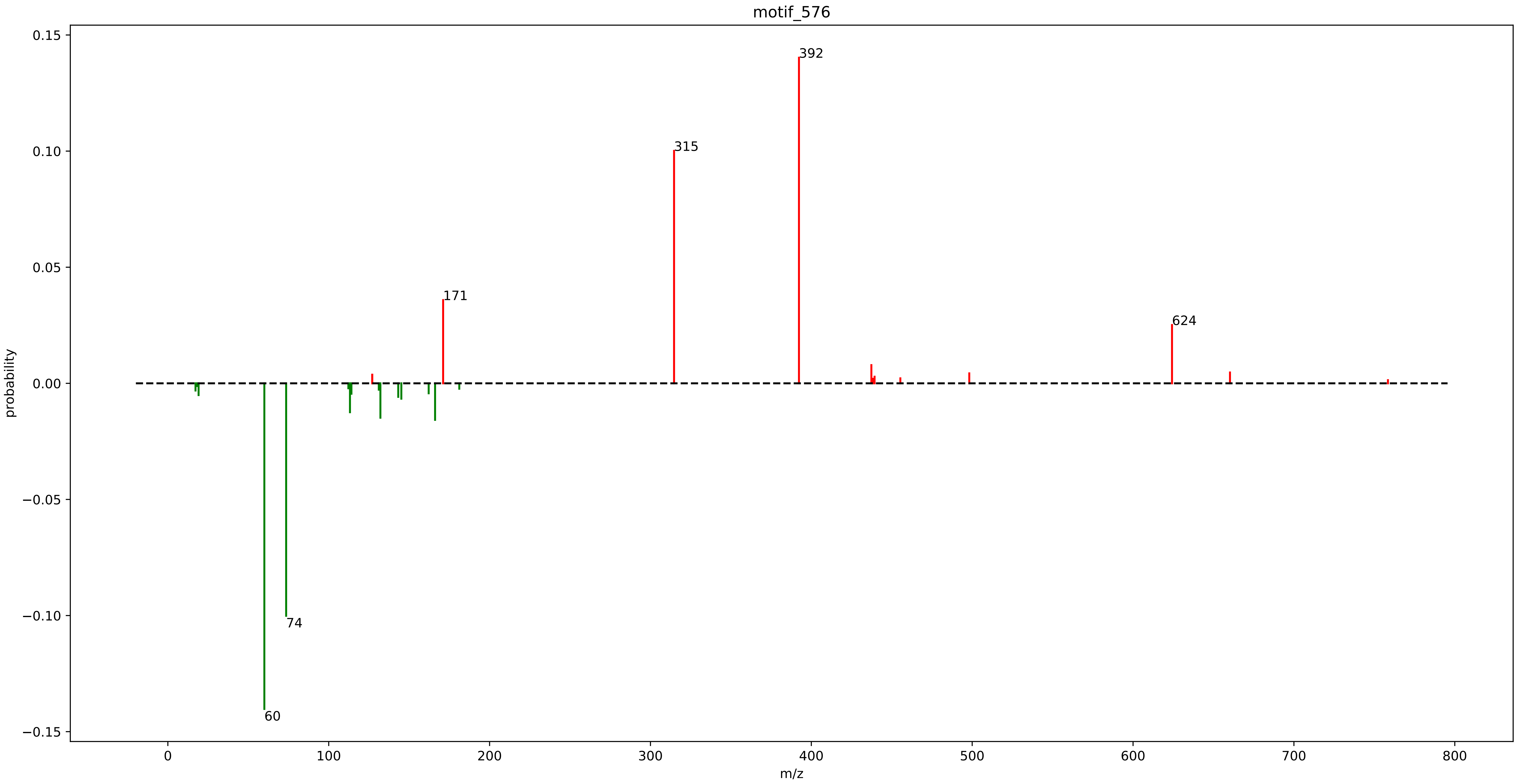
motif_576
392
315
171
624
74
60
0.15
0.10
0.05
0.00
−0.05
−0.10
−0.15
probability
0
100
200
300
400
500
600
700
800
m/z

motif_576

loss_59.9825	: 0.14
fragment_392.3175	: 0.14
fragment_314.6725	: 0.10
loss_73.5325	: 0.10
fragment_171.1125	: 0.04
fragment_624.2175	: 0.03
loss_166.1125	: 0.02
loss_132.1275	: 0.01
loss_113.2075	: 0.01
fragment_437.3275	: 0.01
loss_145.1375	: 0.01
loss_143.2175	: 0.01
loss_19.1025	: 0.01

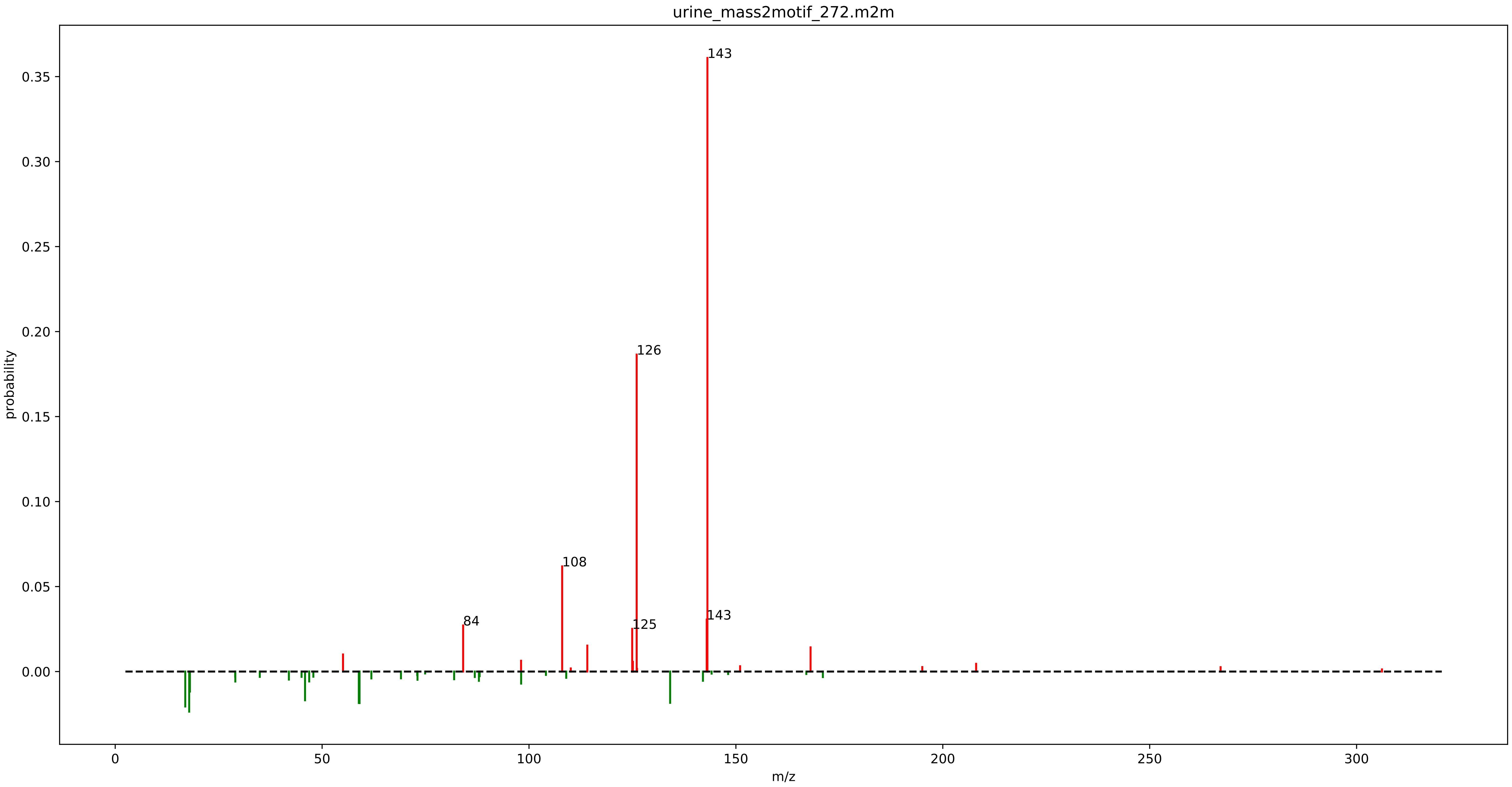

urine_mass2motif_272.m2m
probability
m/z
0.35
0.30
0.25
0.20
0.15
0.10
0.05
0.00
0
50
100
150
200
250
300
143
126
108
84
125
143

urine_mass2motif_272.m2m

fragment_143.1175	: 0.36
fragment_126.0225	: 0.19
fragment_108.0125	: 0.06
fragment_142.9475	: 0.03
fragment_84.0825	: 0.03
fragment_124.9375	: 0.03
loss_17.8775	: 0.02
loss_16.9275	: 0.02
loss_59.0475	: 0.02
loss_58.8675	: 0.02
loss_134.1075	: 0.02
loss_45.8725	: 0.02
fragment_114.0925	: 0.02
fragment_168.0425	: 0.01
loss_18.0575	: 0.01
fragment_55.0525	: 0.01
fragment_143.0825	: 0.01
loss_18.0125	: 0.01
loss_98.0975	: 0.01
fragment_98.0825	: 0.01
loss_29.0275	: 0.01
loss_46.8775	: 0.01
fragment_125.1075	: 0.01
loss_87.8925	: 0.01
loss_142.0375	: 0.01

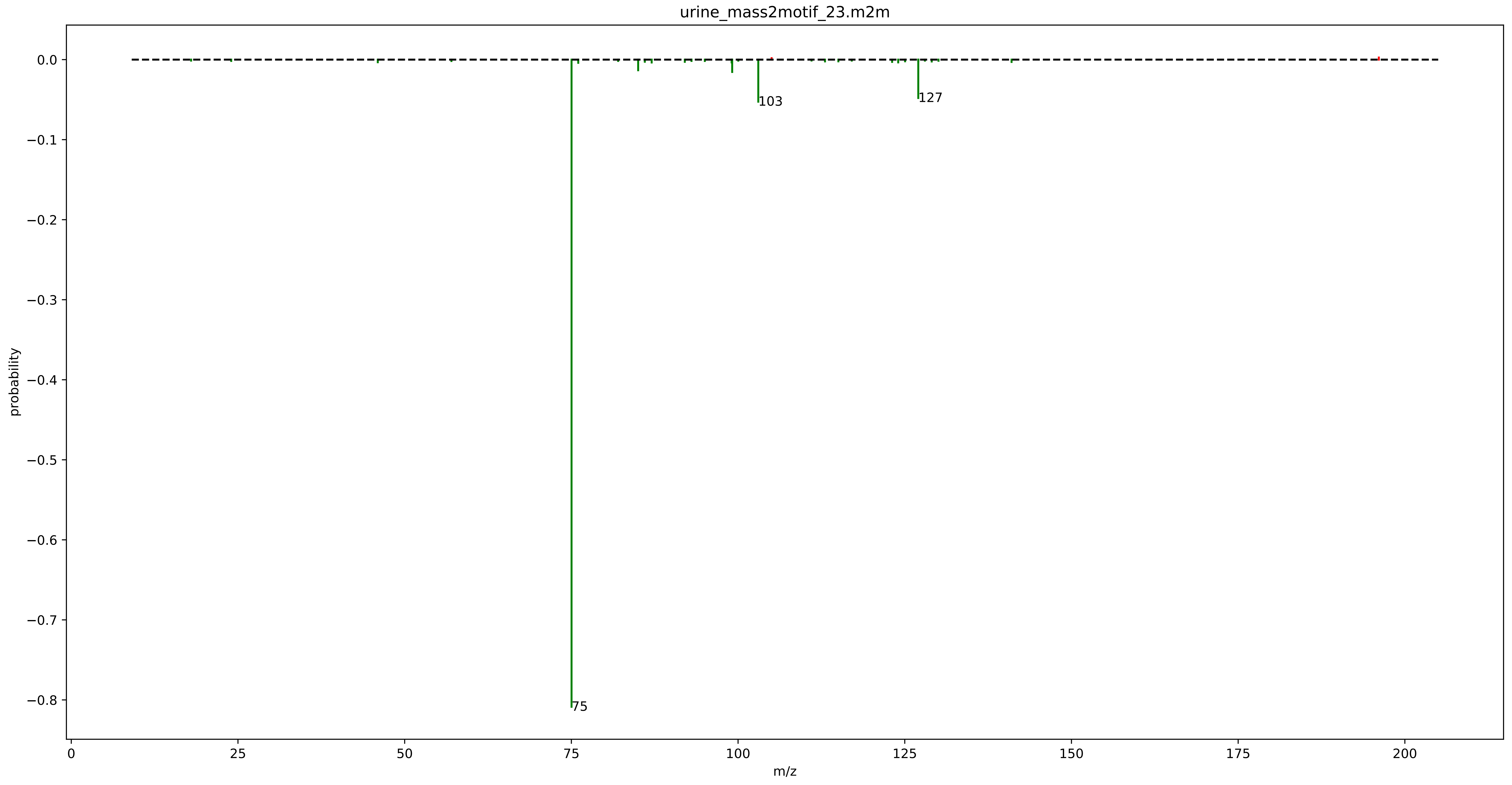

urine_mass2motif_23.m2m
probability
m/z
0.0
−0.1
−0.2
−0.3
−0.4
−0.5
−0.6
−0.7
−0.8
0
25
50
75
100
125
150
175
200
75
103
127

urine_mass2motif_23.m2m

loss_75.0275	: 0.81
loss_103.0325	: 0.05
loss_127.0275	: 0.05
loss_99.1175	: 0.02
loss_85.0175	: 0.01

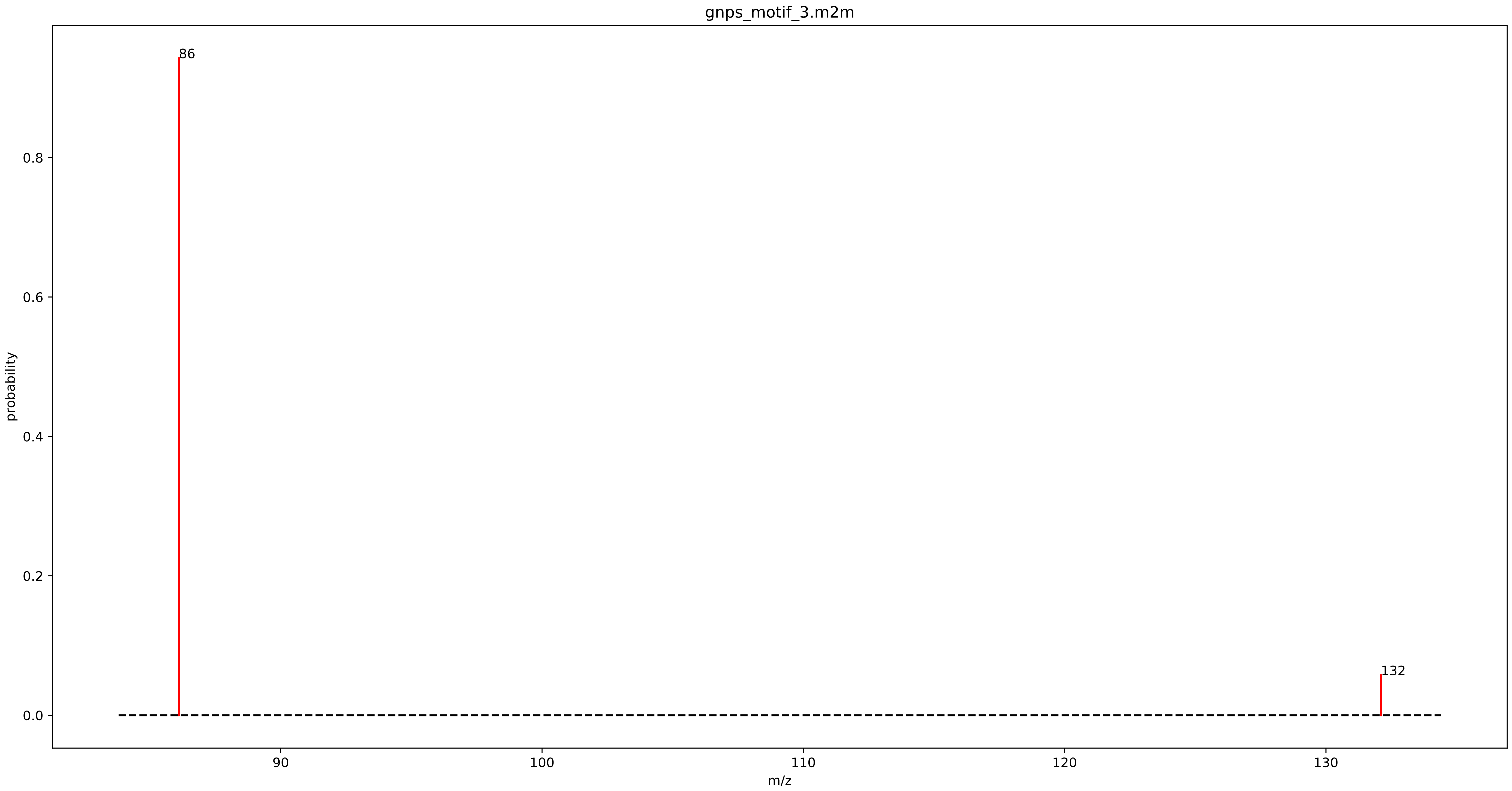
gnps_motif_3.m2m
86
132
0.0
0.2
0.4
0.6
0.8
probability
90
100
110
120
130
m/z

gnps_motif_3.m2m

fragment_86.0975	: 0.94
fragment_132.1025	: 0.06

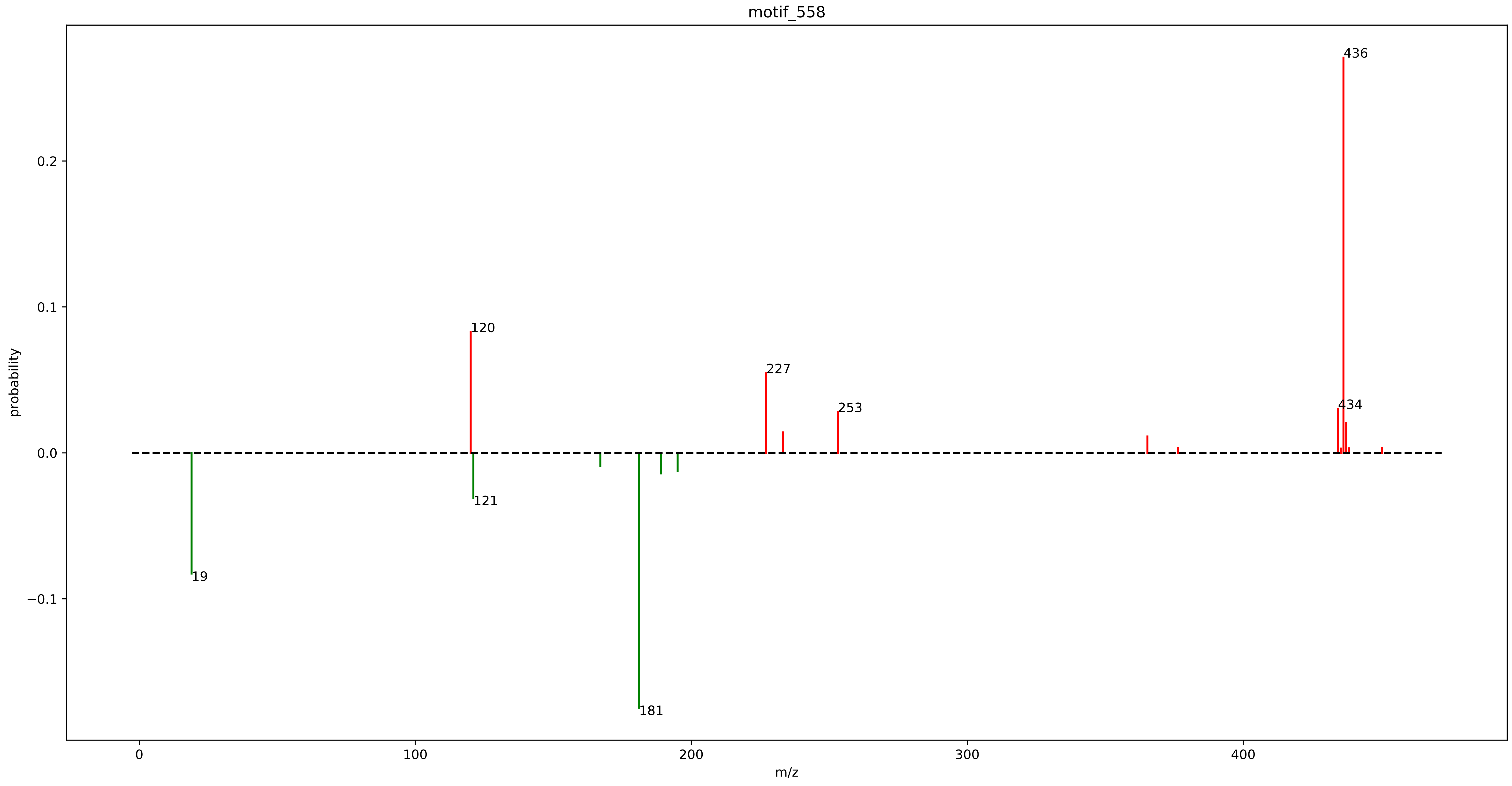

motif_558
probability
m/z
0.2
0.1
0.0
−0.1
0
100
200
300
400
436
120
227
253
434
121
19
181

motif_558

fragment_436.3025	: 0.27
loss_181.0725	: 0.17
fragment_120.0725	: 0.08
loss_18.9475	: 0.08
fragment_227.1725	: 0.05
loss_121.0525	: 0.03
fragment_434.3225	: 0.03
fragment_253.1175	: 0.03
fragment_437.3075	: 0.02
fragment_233.1625	: 0.01
loss_189.0625	: 0.01
loss_195.0525	: 0.01
fragment_365.2675	: 0.01
loss_167.0575	: 0.01

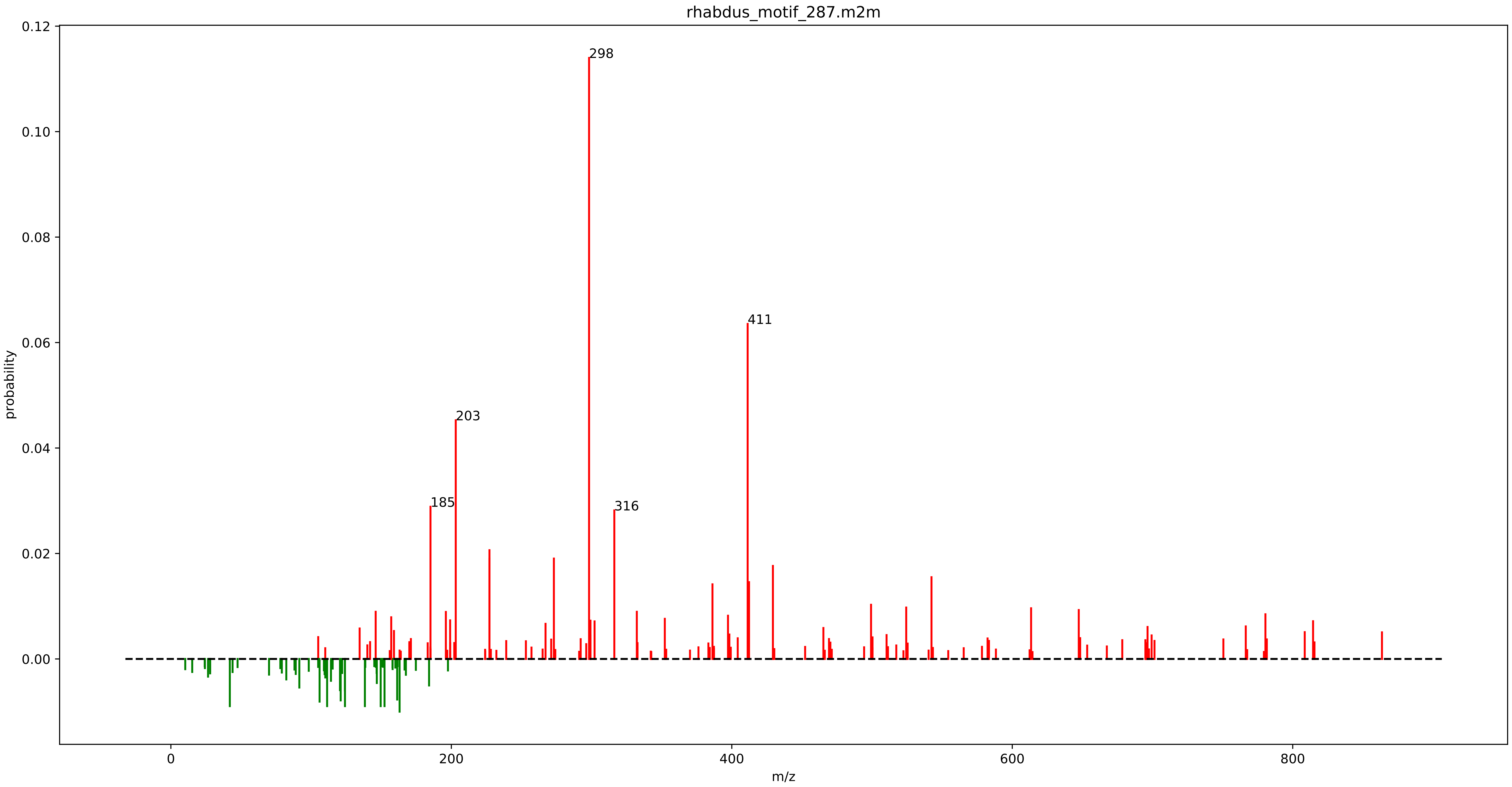

rhabdus_motif_287.m2m
probability
m/z
0.12
0.10
0.08
0.06
0.04
0.02
0.00
0
200
400
600
800
298
411
203
185
316

rhabdus_motif_287.m2m

fragment_298.2125	: 0.11
fragment_411.2975	: 0.06
fragment_203.1375	: 0.05
fragment_185.1275	: 0.03
fragment_316.2225	: 0.03
fragment_227.1775	: 0.02
fragment_273.1225	: 0.02
fragment_227.1725	: 0.02
fragment_429.3075	: 0.02
fragment_542.3925	: 0.02
fragment_412.2975	: 0.01
fragment_386.2075	: 0.01
fragment_499.2925	: 0.01
loss_163.0975	: 0.01
fragment_524.3775	: 0.01
fragment_613.4275	: 0.01
fragment_524.3825	: 0.01
fragment_647.4125	: 0.01
fragment_146.0375	: 0.01
loss_42.0325	: 0.01
fragment_332.2625	: 0.01
loss_124.1425	: 0.01
loss_149.6075	: 0.01
loss_111.4125	: 0.01
loss_138.3475	: 0.01
loss_152.3525	: 0.01
fragment_196.0875	: 0.01
fragment_780.5225	: 0.01
fragment_397.2825	: 0.01
loss_106.0425	: 0.01
fragment_157.1325	: 0.01
loss_121.0875	: 0.01
loss_161.3775	: 0.01
fragment_352.2225	: 0.01
fragment_199.1425	: 0.01
fragment_299.2175	: 0.01
fragment_814.5075	: 0.01
fragment_302.1275	: 0.01
fragment_267.1675	: 0.01
fragment_766.5075	: 0.01
fragment_696.4675	: 0.01
loss_124.0875	: 0.01
loss_120.5825	: 0.01
fragment_465.3075	: 0.01
fragment_134.6025	: 0.01
loss_91.6025	: 0.01
fragment_159.0875	: 0.01
fragment_808.5525	: 0.01
fragment_863.6325	: 0.01
loss_184.1175	: 0.01

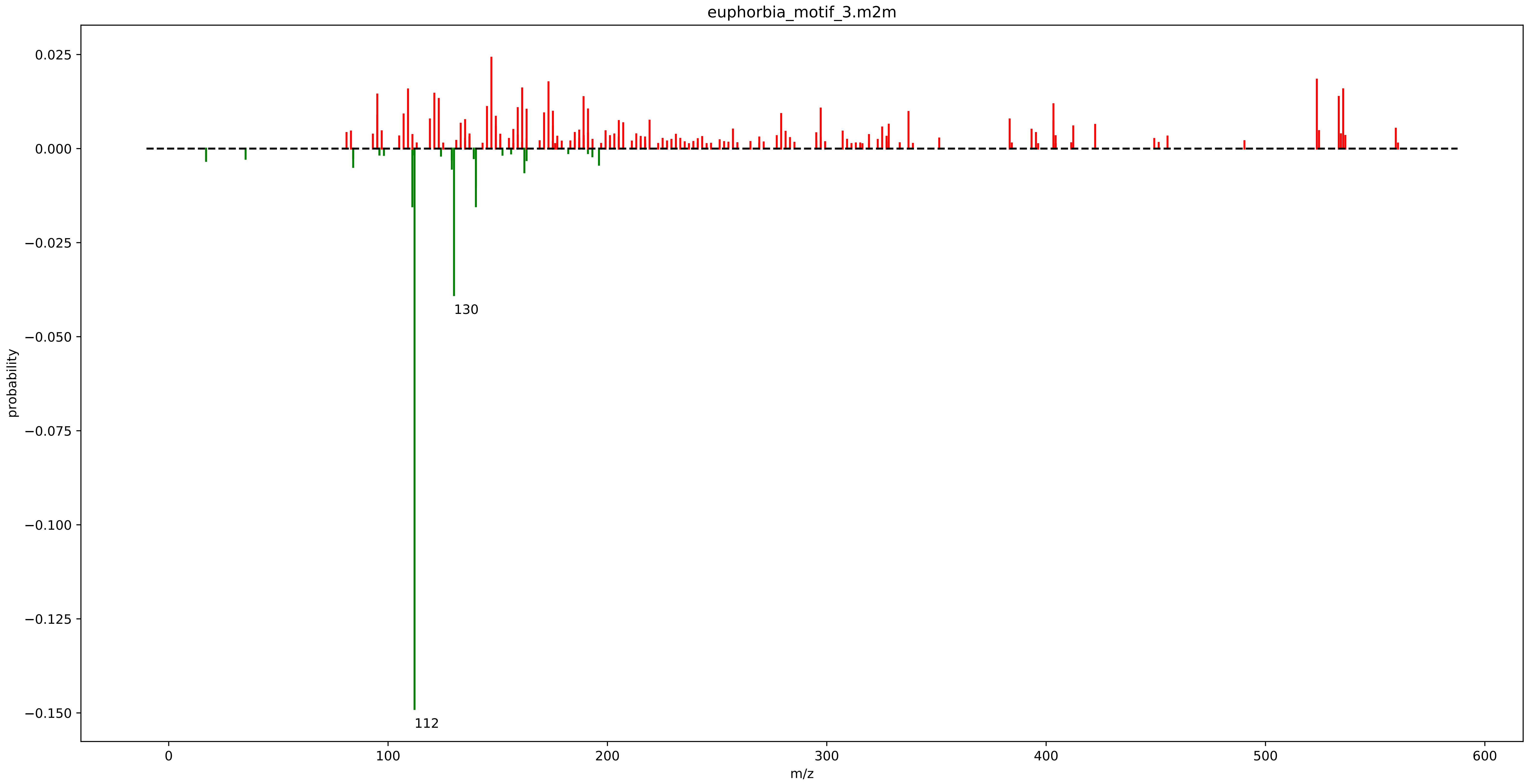

euphorbia_motif_3.m2m
probability
m/z
0.025
0.000
−0.025
−0.050
−0.075
−0.100
−0.125
−0.150
0
100
200
300
400
500
600
130
112

euphorbia_motif_3.m2m

loss_112.0575	: 0.15
loss_130.0675	: 0.04
fragment_147.1125	: 0.02
fragment_523.4075	: 0.02
fragment_173.1275	: 0.02
fragment_161.1275	: 0.02
fragment_535.4075	: 0.02
fragment_109.0975	: 0.02
loss_111.0575	: 0.02
loss_140.0475	: 0.02
fragment_121.0975	: 0.01
fragment_95.0825	: 0.01
fragment_533.3925	: 0.01
fragment_189.1225	: 0.01
fragment_123.1125	: 0.01
fragment_403.3275	: 0.01
fragment_145.0975	: 0.01
fragment_159.1125	: 0.01
fragment_297.2175	: 0.01
fragment_191.1425	: 0.01
fragment_163.1425	: 0.01
loss_140.0525	: 0.01
fragment_175.1475	: 0.01
fragment_337.2475	: 0.01
fragment_171.1125	: 0.01
fragment_279.2075	: 0.01
fragment_107.0825	: 0.01
fragment_95.0475	: 0.01
fragment_149.1325	: 0.01
fragment_119.0825	: 0.01
fragment_383.3625	: 0.01
fragment_135.1125	: 0.01
fragment_219.2075	: 0.01
fragment_205.1925	: 0.01
fragment_403.3325	: 0.01
fragment_207.2075	: 0.01
fragment_133.0975	: 0.01
fragment_328.2475	: 0.01
fragment_422.3425	: 0.01
loss_162.1425	: 0.01
fragment_412.3575	: 0.01
fragment_175.1425	: 0.01
fragment_149.1275	: 0.01
fragment_325.2475	: 0.01
loss_129.0625	: 0.01
fragment_559.4075	: 0.01
fragment_257.2225	: 0.01
fragment_393.3475	: 0.01

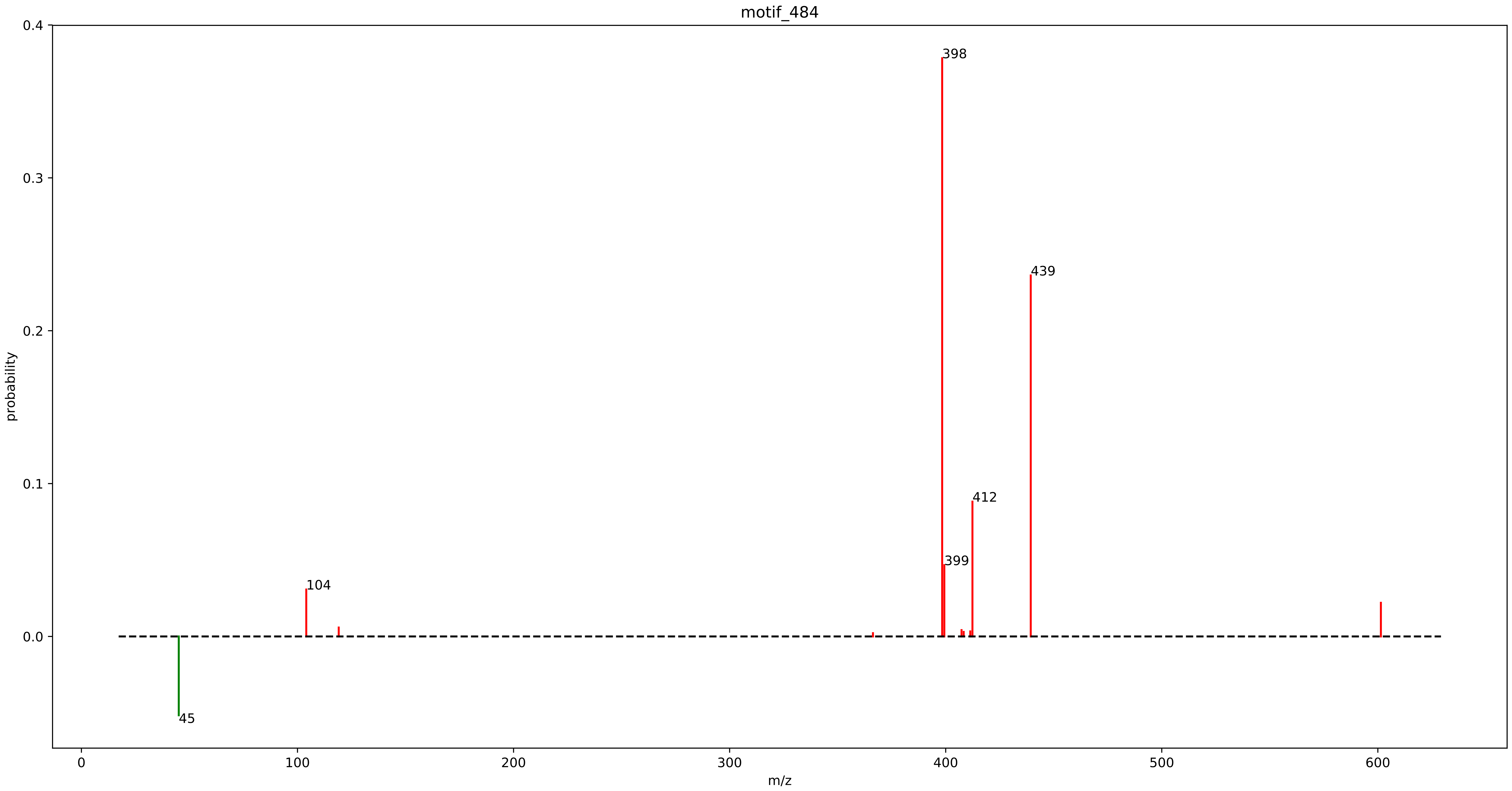

motif_484
probability
m/z
0.4
0.3
0.2
0.1
0.0
0
100
200
300
400
500
600
398
439
412
399
104
45

motif_484

fragment_398.3625	: 0.38
fragment_439.3575	: 0.24
fragment_412.3775	: 0.09
loss_45.0575	: 0.05
fragment_399.3675	: 0.05
fragment_104.0675	: 0.03
fragment_601.4075	: 0.02
fragment_119.0875	: 0.01

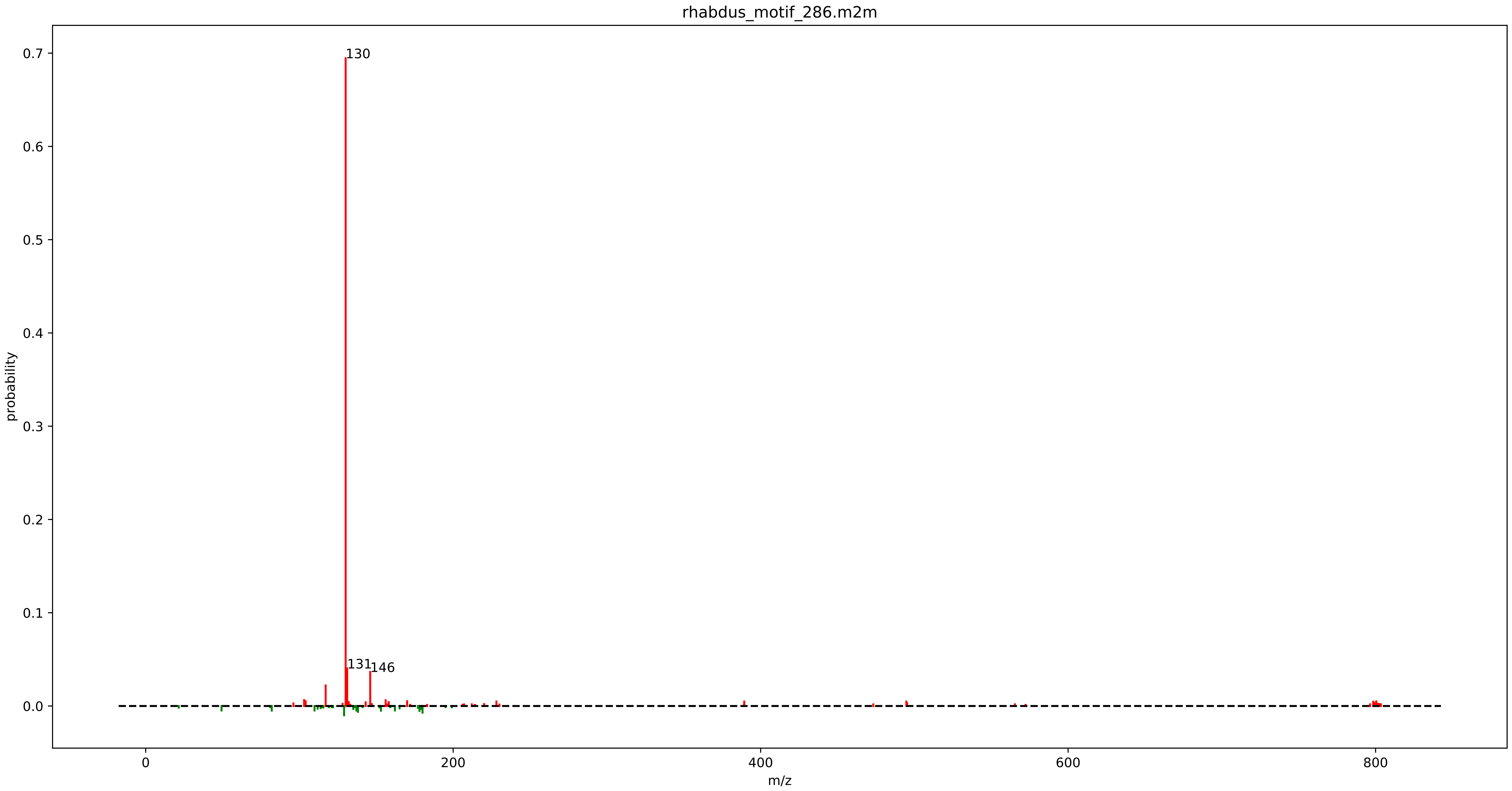

rhabdus_motif_286.m2m
probability
m/z
130
131
146
0.0
0.1
0.2
0.3
0.4
0.5
0.6
0.7
0
200
400
600
800

rhabdus_motif_286.m2m

fragment_130.0625	: 0.69
fragment_131.0675	: 0.04
fragment_146.0575	: 0.04
fragment_117.0575	: 0.02
loss_129.0575	: 0.01
loss_180.0775	: 0.01
fragment_103.0525	: 0.01
loss_138.0675	: 0.01
fragment_156.1025	: 0.01
loss_178.0975	: 0.01
fragment_170.0575	: 0.01
fragment_104.0525	: 0.01
loss_153.0425	: 0.01

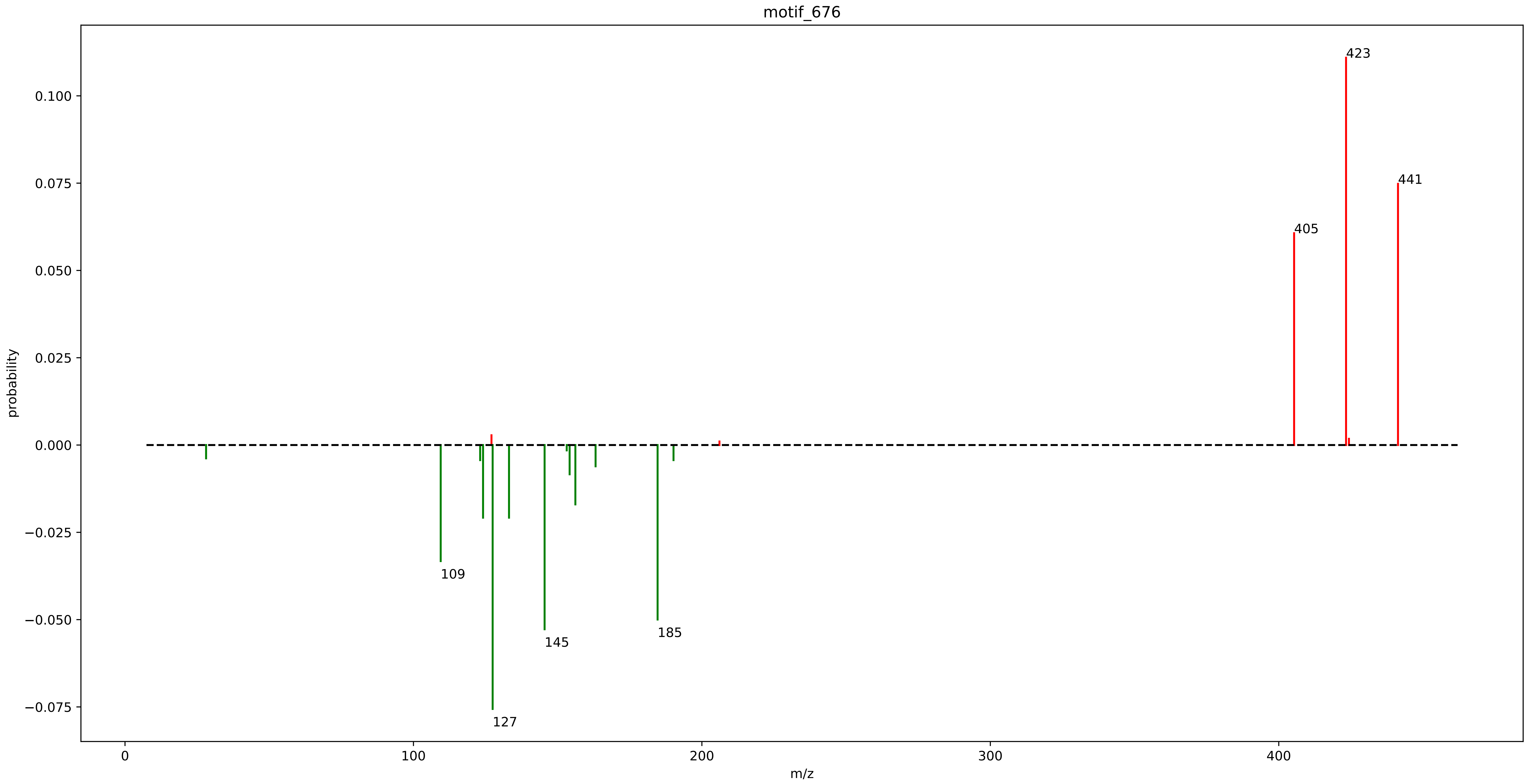

motif_676
423
441
405
109
127
145
185
0.100
0.075
0.050
0.025
0.000
−0.025
−0.050
−0.075
probability
0
100
200
300
400
m/z

motif_676

fragment_423.3275	: 0.11
loss_127.4575	: 0.08
fragment_441.3375	: 0.07
fragment_405.3175	: 0.06
loss_145.4675	: 0.05
loss_184.6425	: 0.05
loss_109.4475	: 0.03
loss_124.1325	: 0.02
loss_133.1325	: 0.02
loss_156.1375	: 0.02
loss_154.1425	: 0.01
loss_163.1425	: 0.01

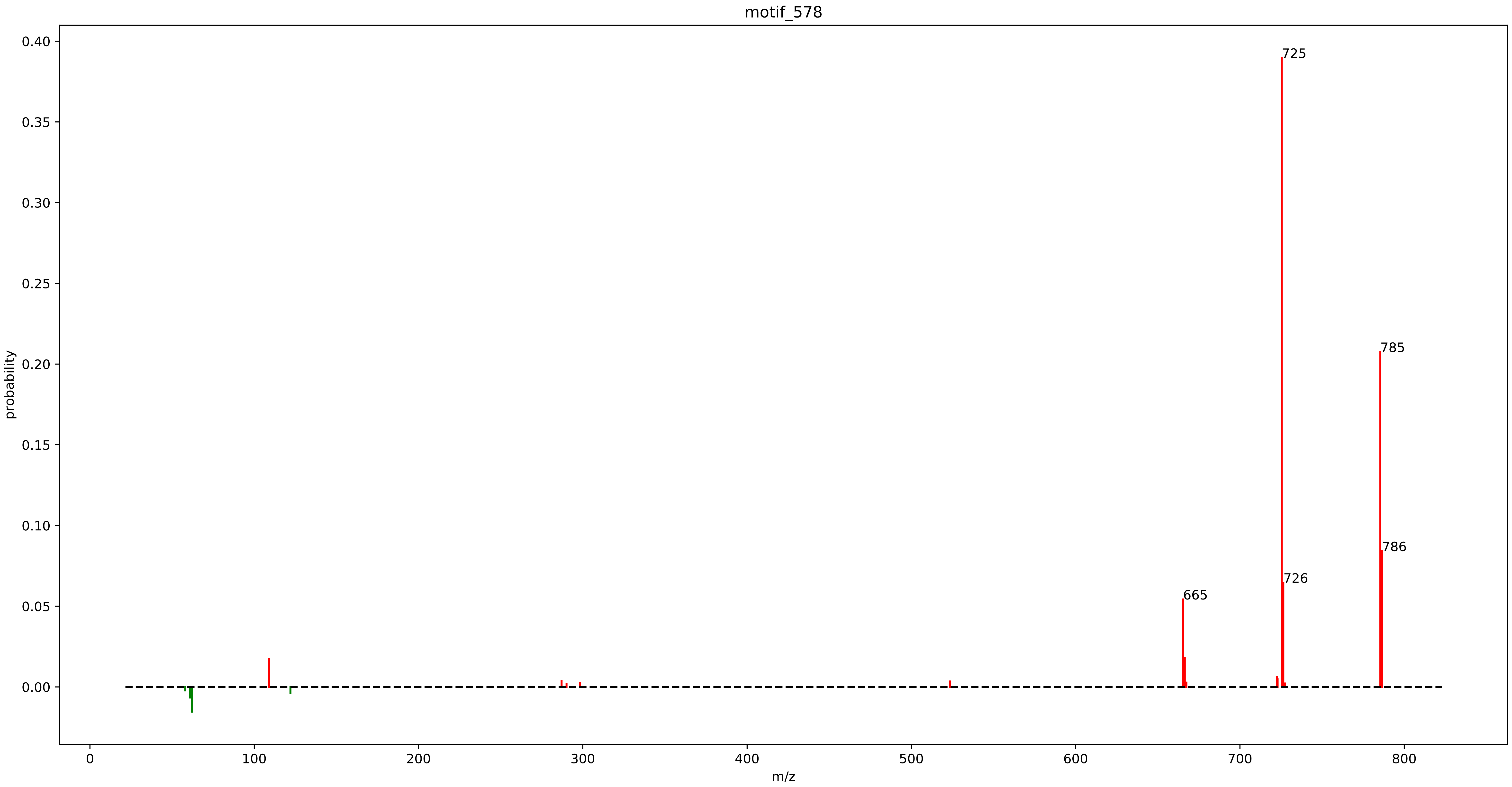

motif_578
probability
m/z
725
785
786
726
665
0.40
0.35
0.30
0.25
0.20
0.15
0.10
0.05
0.00
0
100
200
300
400
500
600
700
800

motif_578

fragment_725.4225	: 0.39
fragment_785.4475	: 0.21
fragment_786.4475	: 0.08
fragment_726.4275	: 0.06
fragment_665.4025	: 0.05
fragment_666.4075	: 0.02
fragment_109.0425	: 0.02
loss_62.0325	: 0.02
loss_61.0325	: 0.01
fragment_722.4175	: 0.01

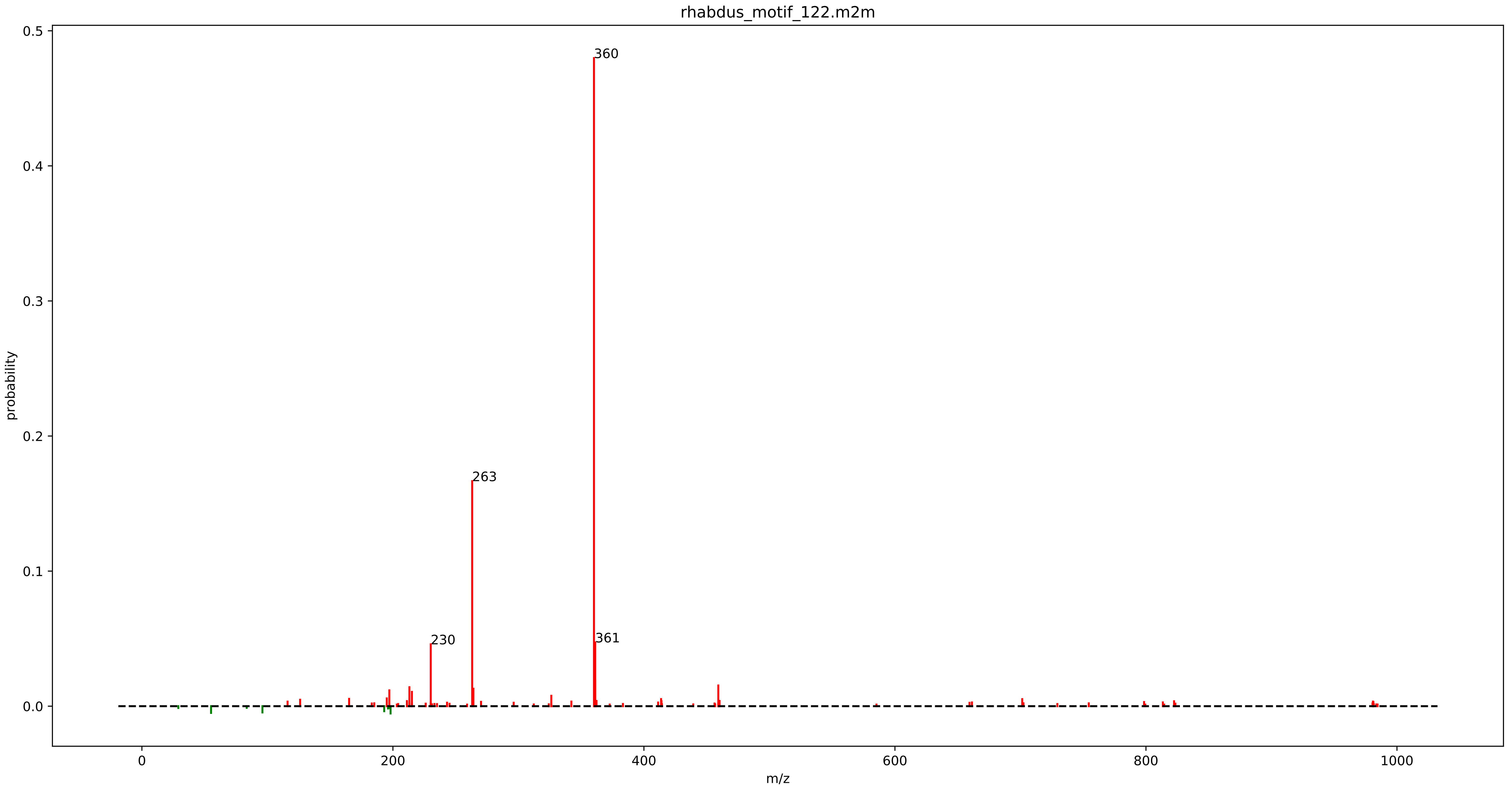

rhabdus_motif_122.m2m
probability
m/z
360
263
230
361
0.5
0.4
0.3
0.2
0.1
0.0
0
200
400
600
800
1000

rhabdus_motif_122.m2m

fragment_360.1925	: 0.48
fragment_263.1375	: 0.17
fragment_361.1975	: 0.05
fragment_230.1225	: 0.05
fragment_361.1925	: 0.02
fragment_459.2625	: 0.02
fragment_213.1275	: 0.01
fragment_264.1425	: 0.01
fragment_197.1275	: 0.01
fragment_215.1375	: 0.01
fragment_326.2025	: 0.01
fragment_195.1125	: 0.01
fragment_165.1025	: 0.01
loss_198.1375	: 0.01
fragment_413.6475	: 0.01
fragment_701.4075	: 0.01

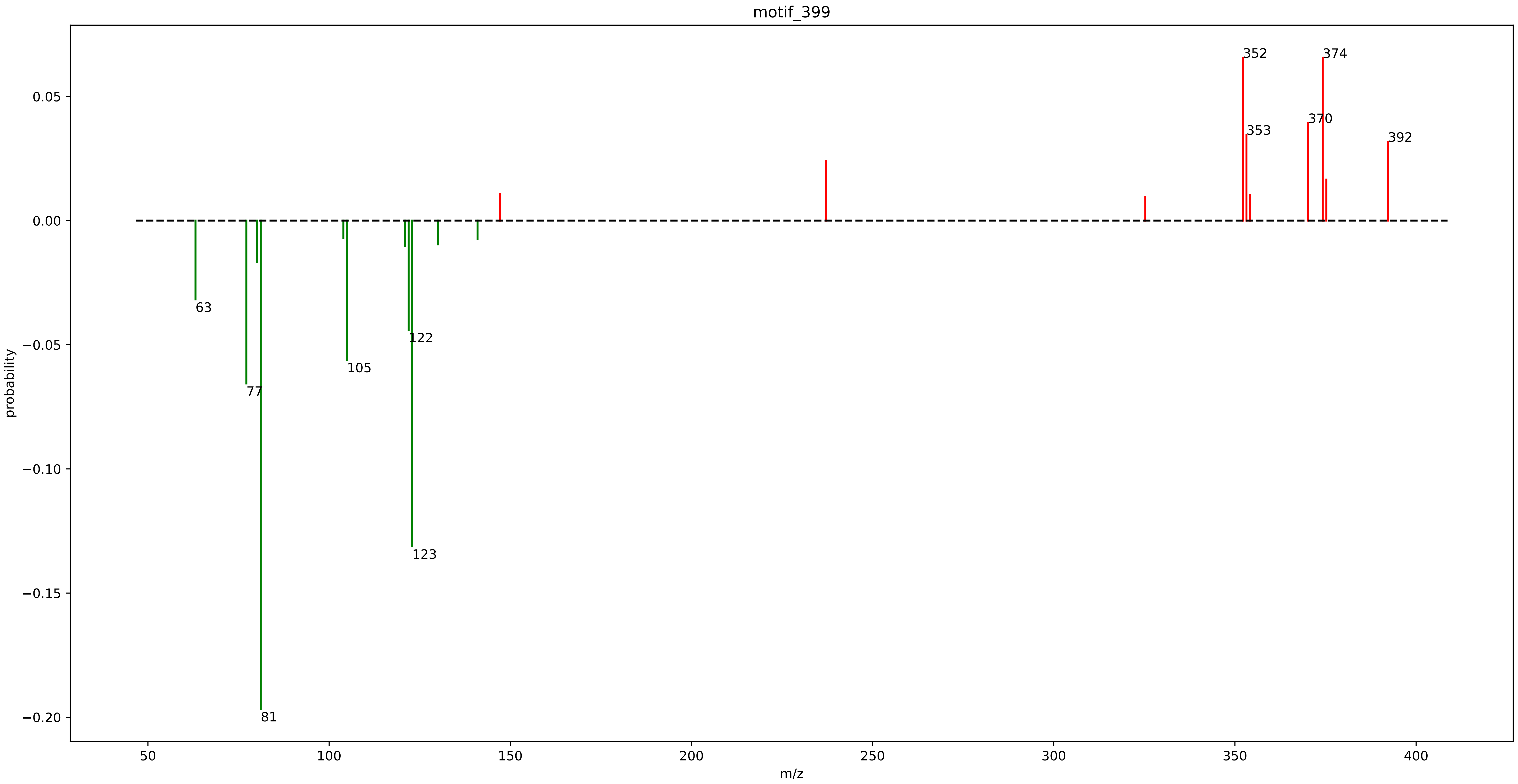
motif_399
probability
m/z
0.05
0.00
−0.05
−0.10
−0.15
−0.20
50
100
150
200
250
300
350
400
352
374
370
353
392
63
122
105
77
123
81

motif_399

loss_81.1325	: 0.20
loss_122.9475	: 0.13
fragment_352.1775	: 0.07
loss_77.1625	: 0.07
fragment_374.2175	: 0.07
loss_104.9375	: 0.06
loss_121.9475	: 0.04
fragment_370.1875	: 0.04
fragment_353.1775	: 0.03
fragment_392.2275	: 0.03
loss_63.1225	: 0.03
fragment_237.1825	: 0.02
fragment_375.2225	: 0.02
loss_80.1325	: 0.02
fragment_147.1175	: 0.01
fragment_354.1725	: 0.01
loss_120.9475	: 0.01
loss_130.0975	: 0.01
fragment_325.2525	: 0.01
loss_140.9575	: 0.01
loss_103.9325	: 0.01

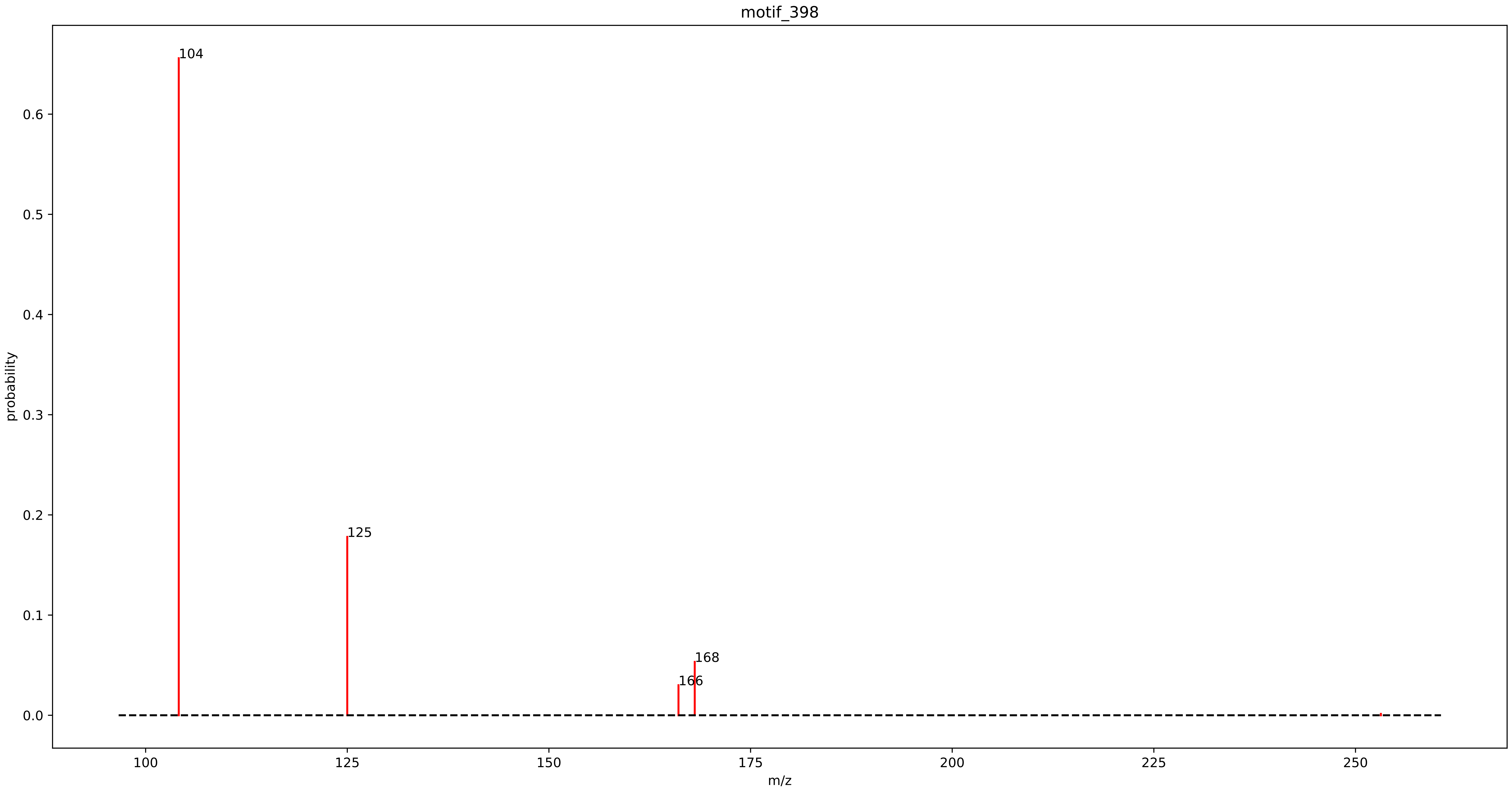

motif_398
probability
m/z
0.0
0.1
0.2
0.3
0.4
0.5
0.6
100
125
150
175
200
225
250
104
125
166
168

motif_398

fragment_104.1075	: 0.66
fragment_124.9975	: 0.18
fragment_168.0775	: 0.05
fragment_166.0625	: 0.03

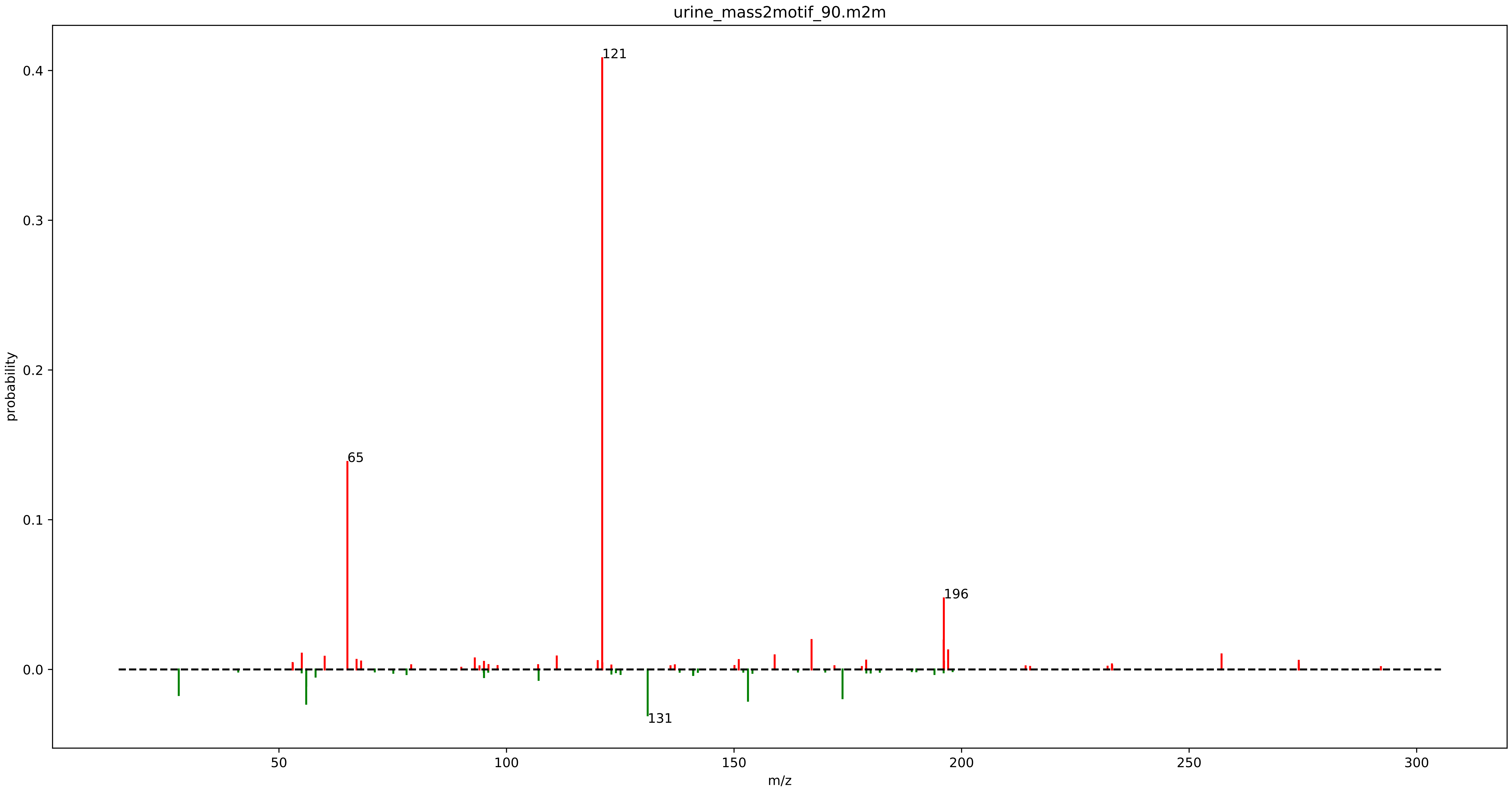

urine_mass2motif_90.m2m
probability
m/z
0.4
0.3
0.2
0.1
0.0
50
100
150
200
250
300
121
65
196
131

urine_mass2motif_90.m2m

fragment_121.0275	: 0.41
fragment_65.0375	: 0.14
fragment_196.0975	: 0.05
loss_131.0225	: 0.03
loss_55.9875	: 0.02
loss_153.0625	: 0.02
fragment_167.0325	: 0.02
fragment_196.0625	: 0.02
loss_173.8375	: 0.02
loss_27.9825	: 0.02
fragment_197.0425	: 0.01
loss_56.0025	: 0.01
fragment_55.0175	: 0.01
fragment_257.1275	: 0.01
fragment_158.9225	: 0.01
fragment_111.0425	: 0.01
fragment_60.0425	: 0.01
fragment_93.0325	: 0.01
loss_107.0575	: 0.01
fragment_67.0525	: 0.01
fragment_151.0375	: 0.01
fragment_179.0325	: 0.01
fragment_274.0925	: 0.01
fragment_120.0425	: 0.01
fragment_196.1325	: 0.01
fragment_68.0475	: 0.01
loss_95.0575	: 0.01
fragment_95.0475	: 0.01

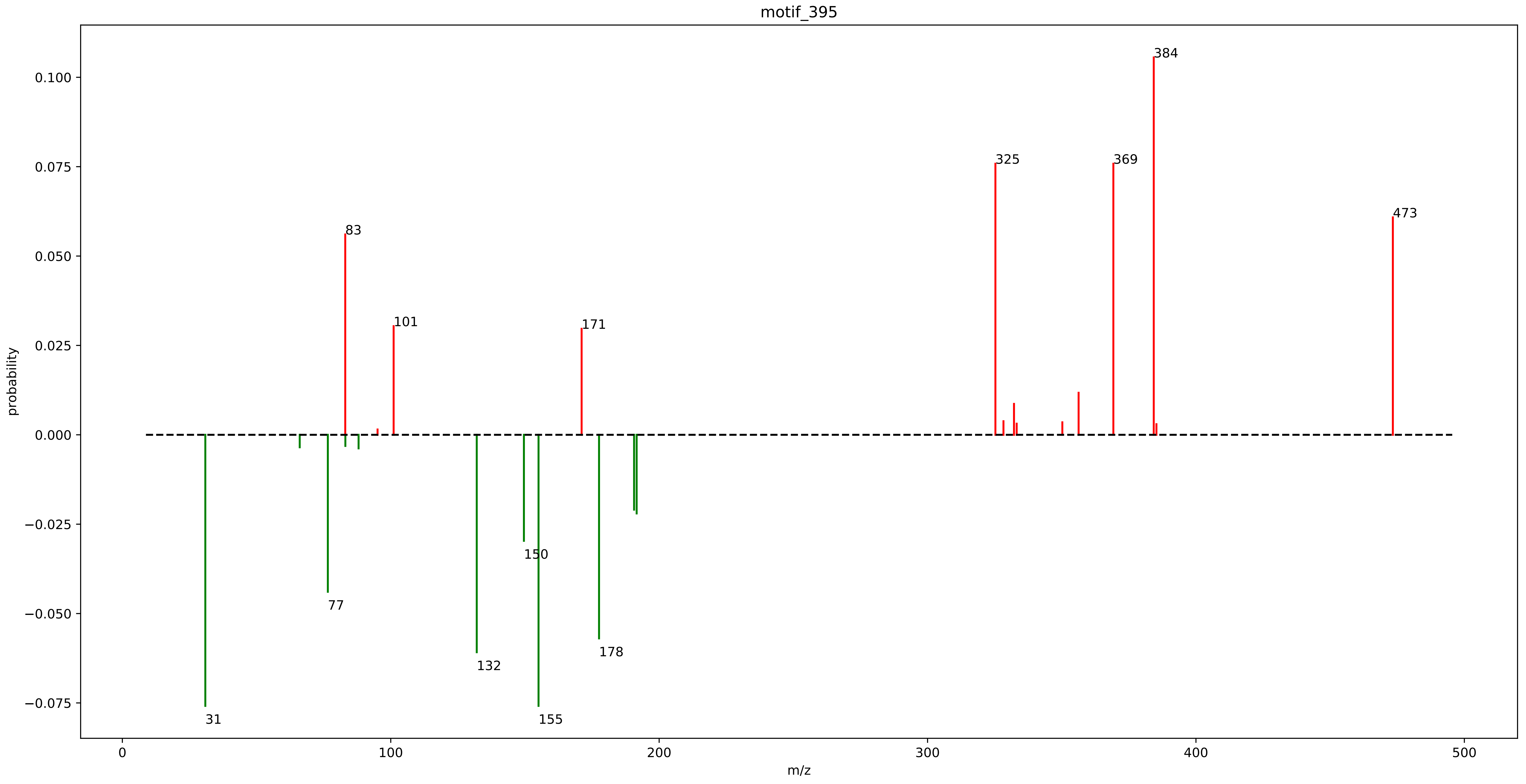

motif_395
probability
m/z
0.100
0.075
0.050
0.025
0.000
−0.025
−0.050
−0.075
0
100
200
300
400
500
384
325
369
473
83
101
171
150
77
178
132
31
155

motif_395

fragment_384.2725	: 0.11
fragment_369.2275	: 0.08
loss_30.9125	: 0.08
fragment_325.2825	: 0.08
loss_155.0575	: 0.08
fragment_473.3625	: 0.06
loss_132.0375	: 0.06
loss_177.6025	: 0.06
fragment_83.0475	: 0.06
loss_76.5575	: 0.04
fragment_101.0725	: 0.03
loss_149.6125	: 0.03
fragment_171.1125	: 0.03
loss_191.6175	: 0.02
loss_190.6575	: 0.02
fragment_356.2775	: 0.01
fragment_332.2075	: 0.01

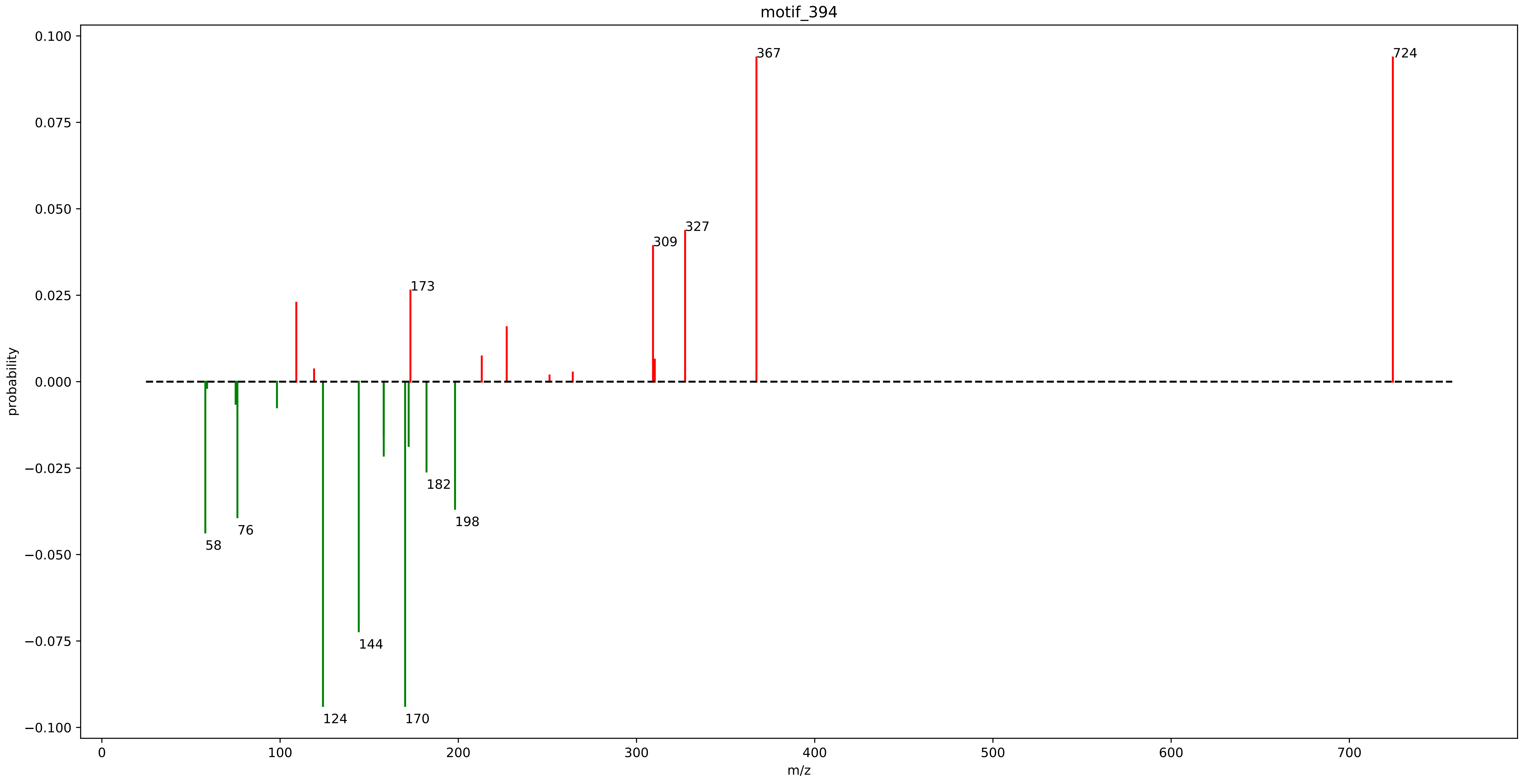

motif_394
0.100
0.075
0.050
0.025
0.000
−0.025
−0.050
−0.075
−0.100
probability
0
100
200
300
400
500
600
700
m/z
367
724
309
327
173
58
76
124
144
170
182
198

motif_394

fragment_367.2975	: 0.09
loss_124.0675	: 0.09
fragment_724.4125	: 0.09
loss_170.1625	: 0.09
loss_144.1475	: 0.07
loss_58.0425	: 0.04
fragment_327.2675	: 0.04
fragment_309.2575	: 0.04
loss_76.0525	: 0.04
loss_198.1625	: 0.04
fragment_173.1325	: 0.03
loss_182.1625	: 0.03
fragment_109.0975	: 0.02
loss_158.1625	: 0.02
loss_172.1475	: 0.02
fragment_227.1625	: 0.02
loss_158.1425	: 0.02
loss_98.2475	: 0.01
fragment_213.1625	: 0.01
loss_75.0475	: 0.01
fragment_310.2625	: 0.01

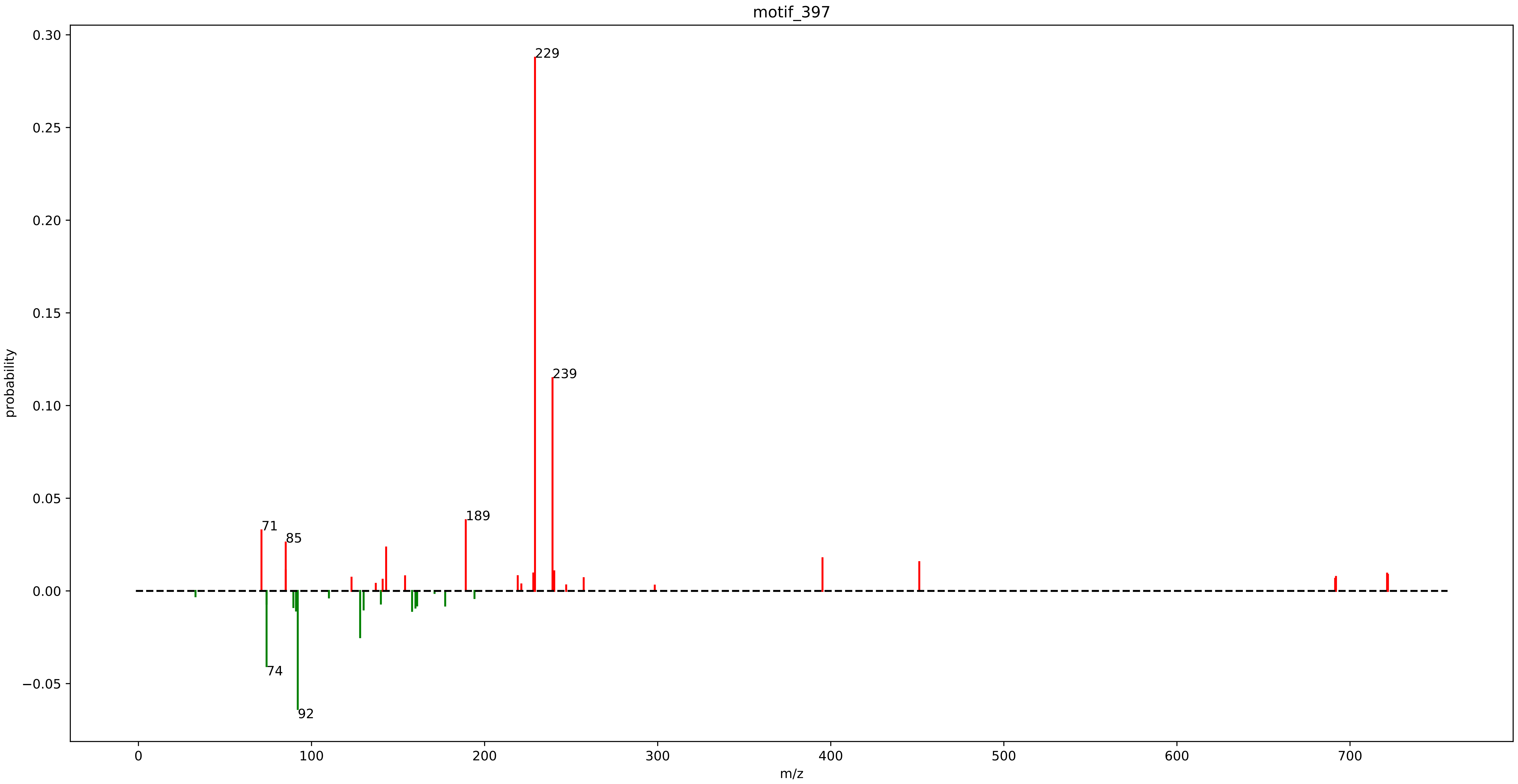

motif_397
probability
m/z
229
239
189
71
85
74
92

motif_397

fragment_229.1175	: 0.29
fragment_239.2375	: 0.11
loss_92.0475	: 0.06
loss_74.0325	: 0.04
fragment_189.1225	: 0.04
fragment_71.0825	: 0.03
fragment_85.0975	: 0.03
loss_128.0925	: 0.02
fragment_143.1075	: 0.02
fragment_395.2175	: 0.02
fragment_451.1425	: 0.02
fragment_85.1025	: 0.01
loss_158.1025	: 0.01
fragment_240.2375	: 0.01
loss_91.0425	: 0.01
loss_130.1075	: 0.01
fragment_228.1325	: 0.01
fragment_721.4075	: 0.01
loss_160.0475	: 0.01
loss_89.5225	: 0.01
fragment_721.9125	: 0.01
fragment_219.1475	: 0.01
loss_177.2325	: 0.01
fragment_154.0475	: 0.01
loss_161.0325	: 0.01
fragment_691.8975	: 0.01
fragment_123.1175	: 0.01
fragment_257.2475	: 0.01
loss_74.0375	: 0.01
loss_140.0775	: 0.01
fragment_691.3975	: 0.01
fragment_141.1025	: 0.01

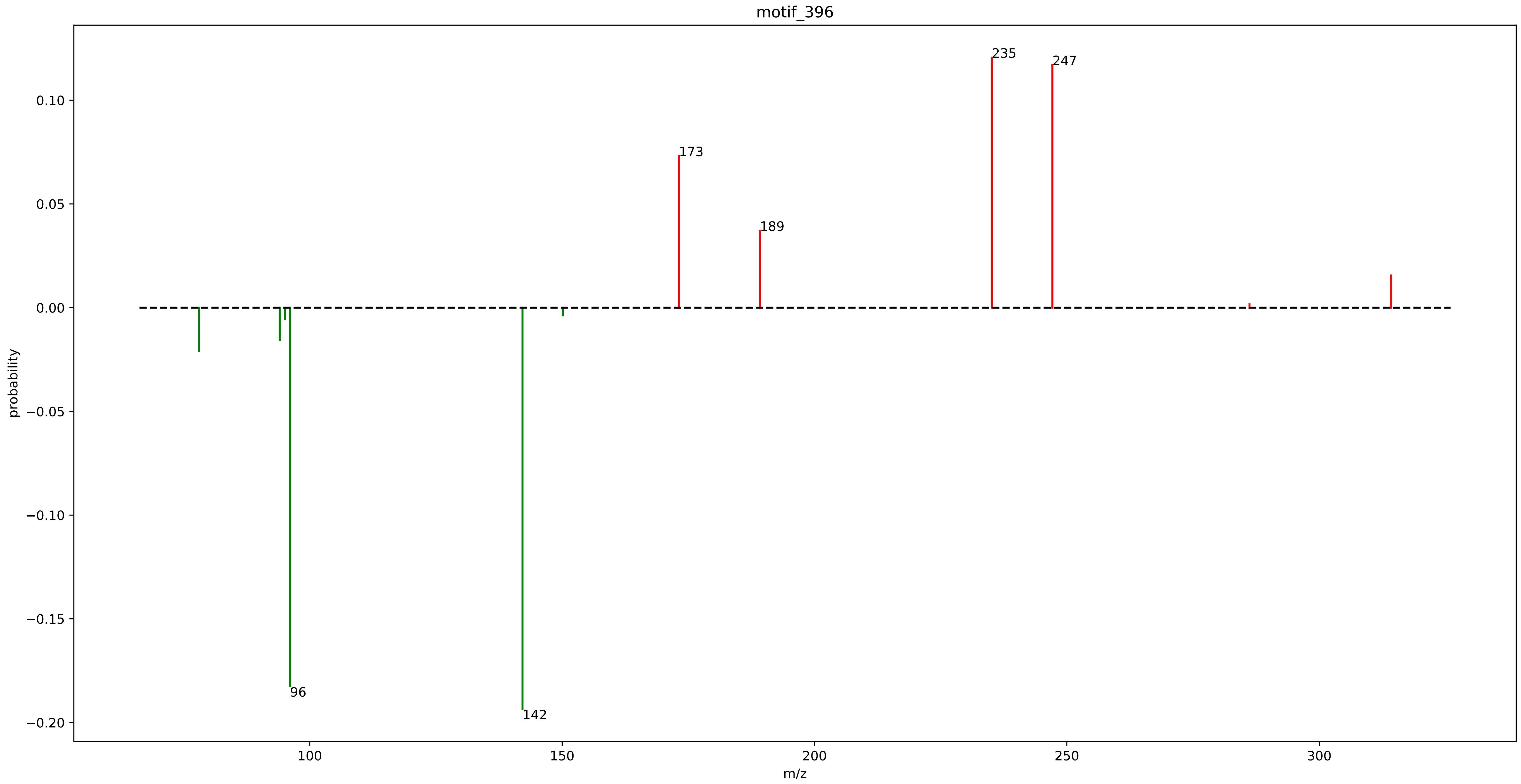

motif_396
235
247
173
189
96
142
0.10
0.05
0.00
−0.05
−0.10
−0.15
−0.20
probability
100
150
200
250
300
m/z

motif_396

loss_142.1475	: 0.19
loss_96.0725	: 0.18
fragment_235.1275	: 0.12
fragment_247.1275	: 0.12
fragment_173.1275	: 0.07
fragment_189.1625	: 0.04
loss_78.0625	: 0.02
loss_94.0575	: 0.02
fragment_314.2125	: 0.02
loss_95.0725	: 0.01

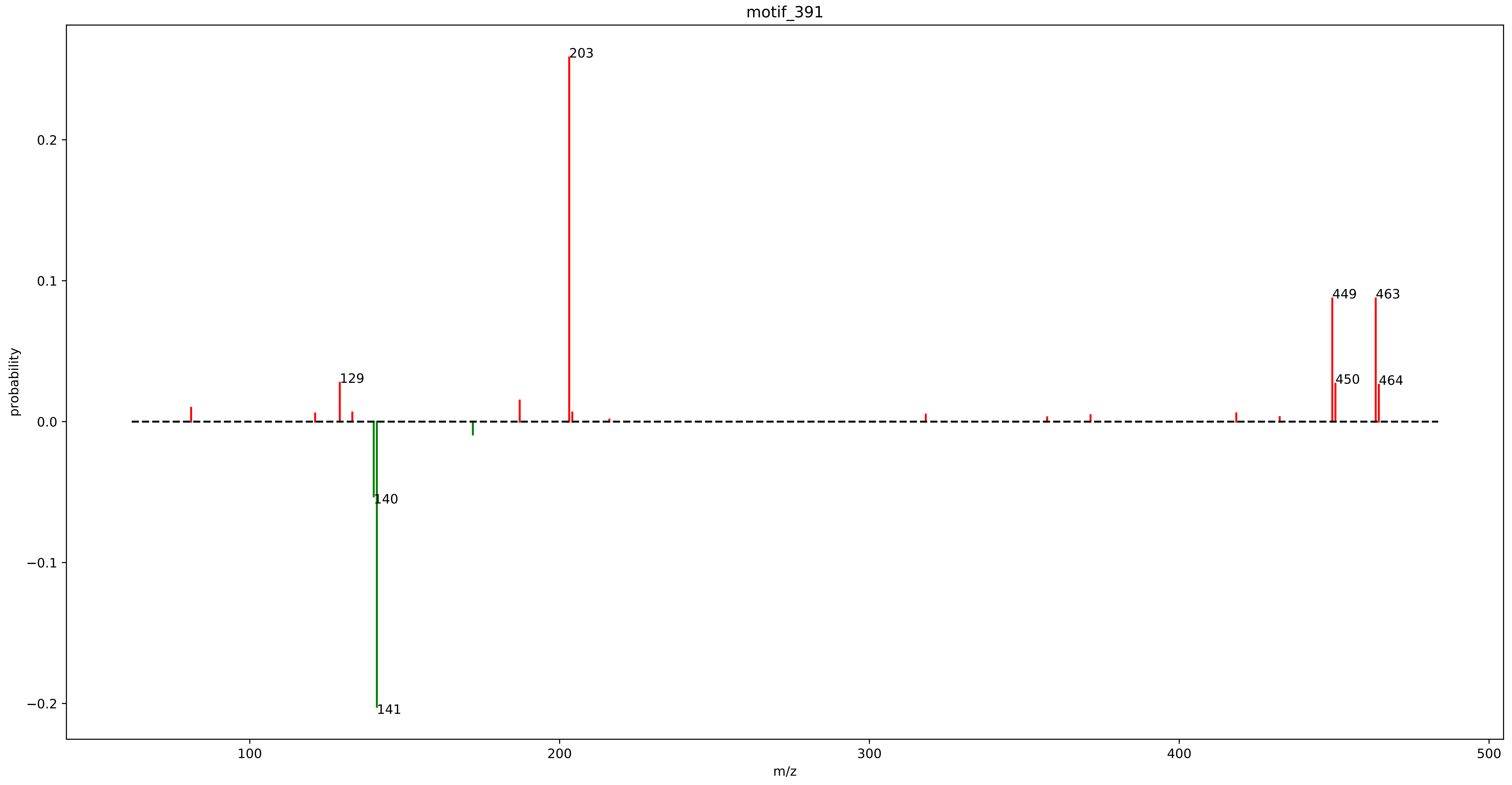

motif_391
203
129
449
463
450
464
140
141
0.2
0.1
0.0
−0.1
−0.2
probability
100
200
300
400
500
m/z

motif_391

fragment_203.1025	: 0.26
loss_141.0175	: 0.20
fragment_449.3975	: 0.09
fragment_463.4125	: 0.09
loss_140.0125	: 0.05
fragment_129.0525	: 0.03
fragment_450.4025	: 0.03
fragment_464.4175	: 0.03
fragment_187.1225	: 0.01
fragment_81.0675	: 0.01
loss_172.0125	: 0.01
fragment_204.1025	: 0.01
fragment_133.1025	: 0.01
fragment_418.4025	: 0.01
fragment_121.0975	: 0.01

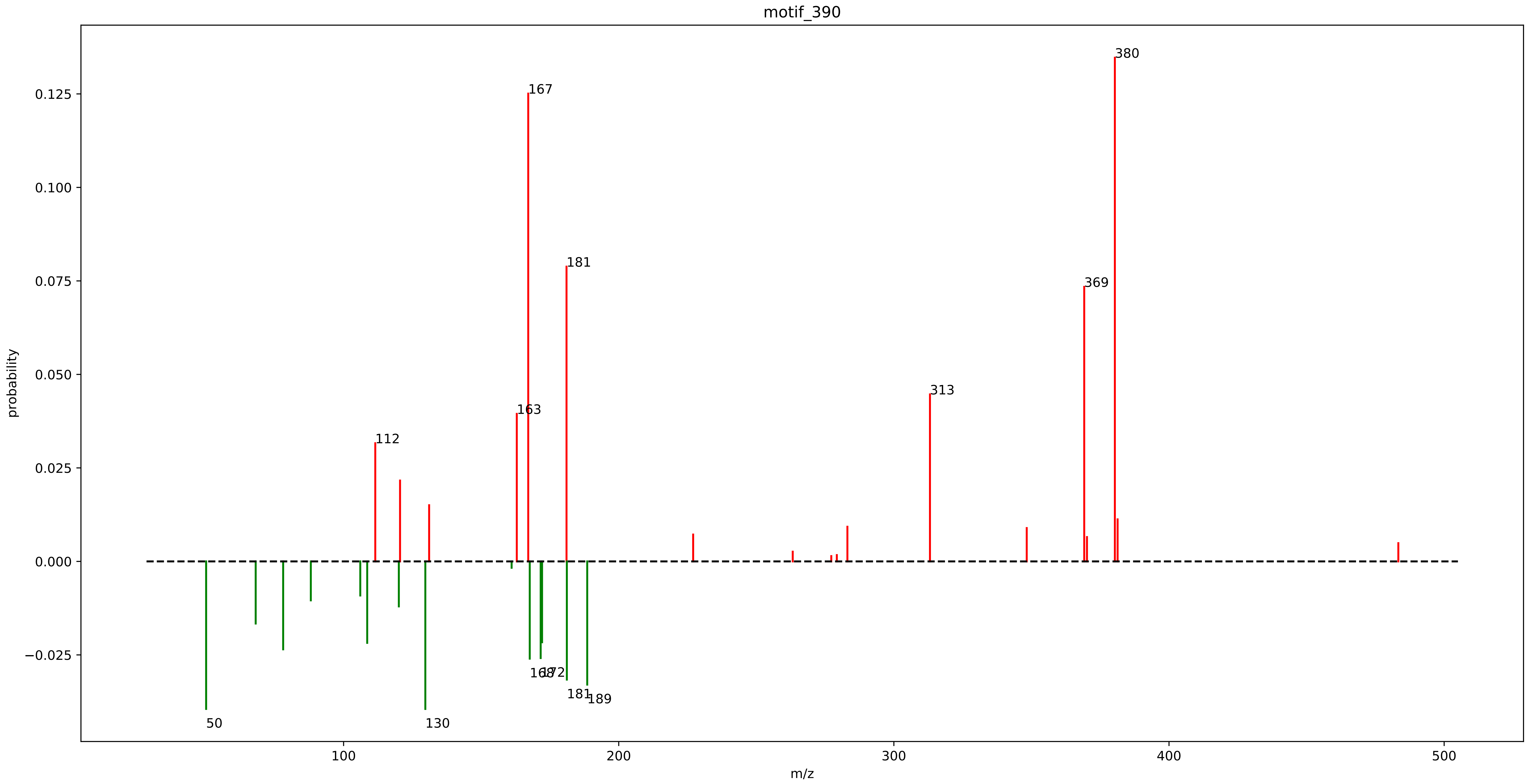

motif_390
probability
m/z
0.125
0.100
0.075
0.050
0.025
0.000
−0.025
100
200
300
400
500
380
167
181
369
313
163
112
50
130
168
172
181
189

motif_390

fragment_380.3175	: 0.13
fragment_167.1075	: 0.13
fragment_181.0225	: 0.08
fragment_369.1825	: 0.07
fragment_313.1175	: 0.04
loss_129.7075	: 0.04
fragment_162.9475	: 0.04
loss_50.0375	: 0.04
loss_188.5475	: 0.03
loss_181.1425	: 0.03
fragment_111.5125	: 0.03
loss_167.6525	: 0.03
loss_171.6275	: 0.03
loss_78.0325	: 0.02
loss_108.5825	: 0.02
loss_172.1375	: 0.02
fragment_120.5175	: 0.02
loss_68.0475	: 0.02
fragment_131.0875	: 0.02
loss_120.0775	: 0.01
fragment_381.3175	: 0.01
loss_88.0875	: 0.01
fragment_283.1075	: 0.01
loss_106.0625	: 0.01
fragment_348.2875	: 0.01
fragment_227.0475	: 0.01
fragment_370.1825	: 0.01

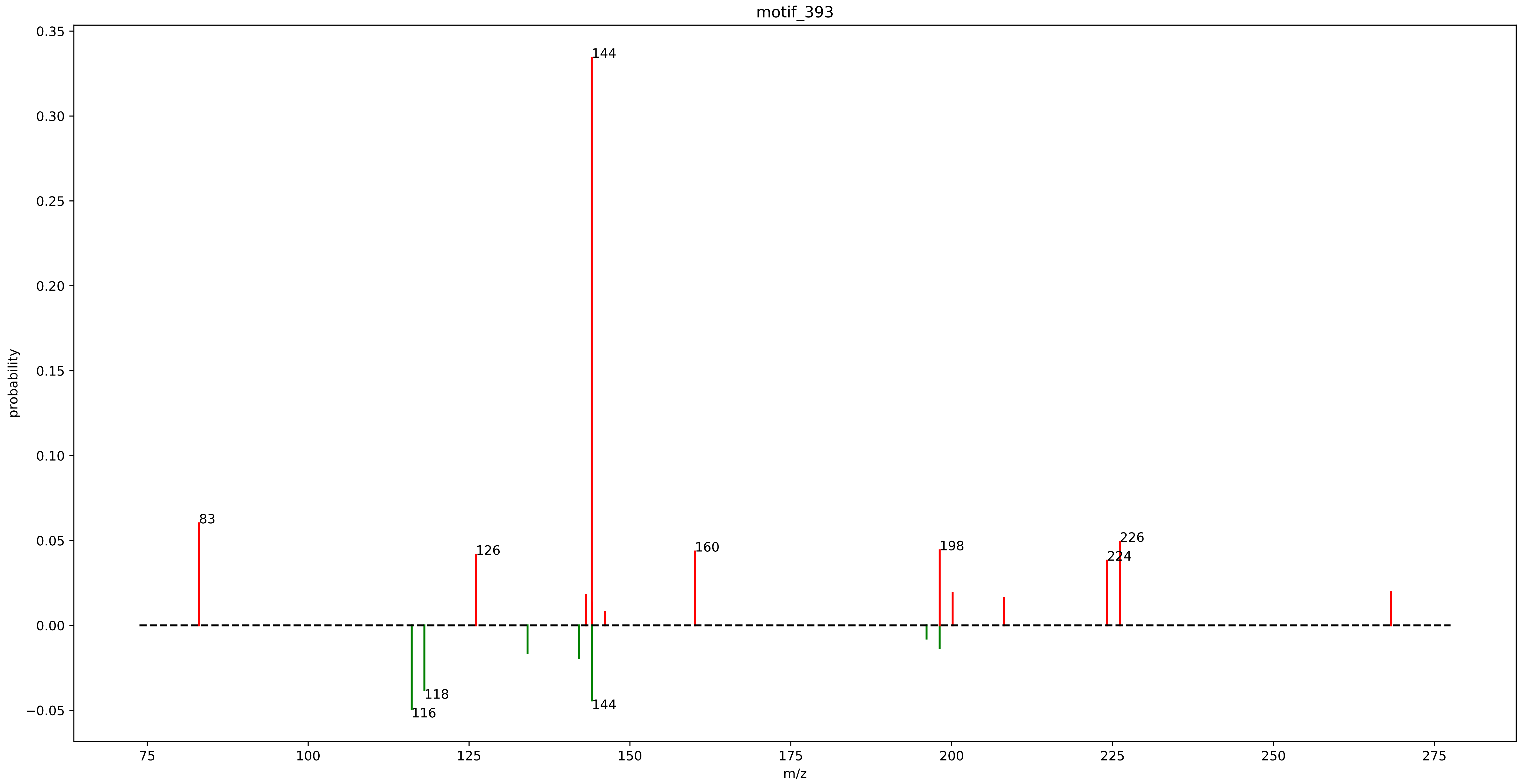

motif_393
probability
m/z
0.35
0.30
0.25
0.20
0.15
0.10
0.05
0.00
−0.05
75
100
125
150
175
200
225
250
275
144
83
126
160
198
226
224
118
116
144

motif_393

fragment_144.0625	: 0.33
fragment_83.0475	: 0.06
loss_116.0825	: 0.05
fragment_226.1225	: 0.05
fragment_198.1275	: 0.04
loss_144.0775	: 0.04
fragment_160.0975	: 0.04
fragment_126.0575	: 0.04
loss_118.0625	: 0.04
fragment_224.1425	: 0.04
fragment_268.2625	: 0.02
fragment_200.1425	: 0.02
loss_142.0625	: 0.02
fragment_143.1275	: 0.02
loss_134.0925	: 0.02
fragment_208.1125	: 0.02
loss_198.1225	: 0.01
fragment_144.0825	: 0.01
fragment_146.1175	: 0.01
loss_196.0875	: 0.01

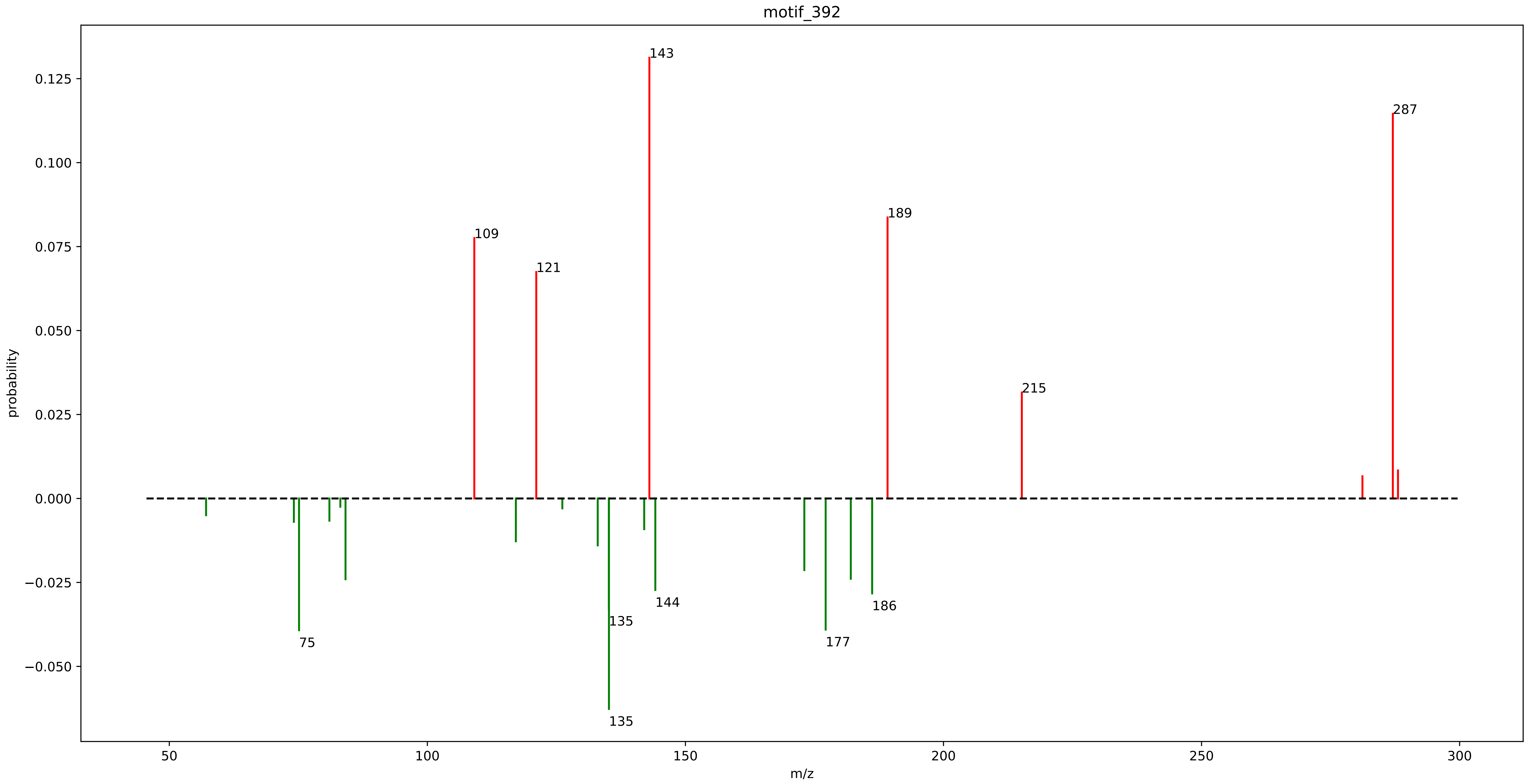

motif_392
probability
m/z
0.125
0.100
0.075
0.050
0.025
0.000
−0.025
−0.050
50
100
150
200
250
300
109
121
143
189
215
287
75
135
135
144
177
186

motif_392

fragment_143.0175	: 0.13
fragment_287.0625	: 0.11
fragment_189.1625	: 0.08
fragment_109.0975	: 0.08
fragment_121.1025	: 0.07
loss_135.1825	: 0.06
loss_75.1375	: 0.04
loss_177.1725	: 0.04
loss_135.1625	: 0.03
fragment_215.1775	: 0.03
loss_186.1775	: 0.03
loss_144.1675	: 0.03
loss_84.1425	: 0.02
loss_182.0425	: 0.02
loss_173.0375	: 0.02
loss_133.0075	: 0.01
loss_117.1475	: 0.01
loss_142.0125	: 0.01
fragment_288.0625	: 0.01
loss_74.1375	: 0.01
loss_81.0225	: 0.01
fragment_281.1725	: 0.01

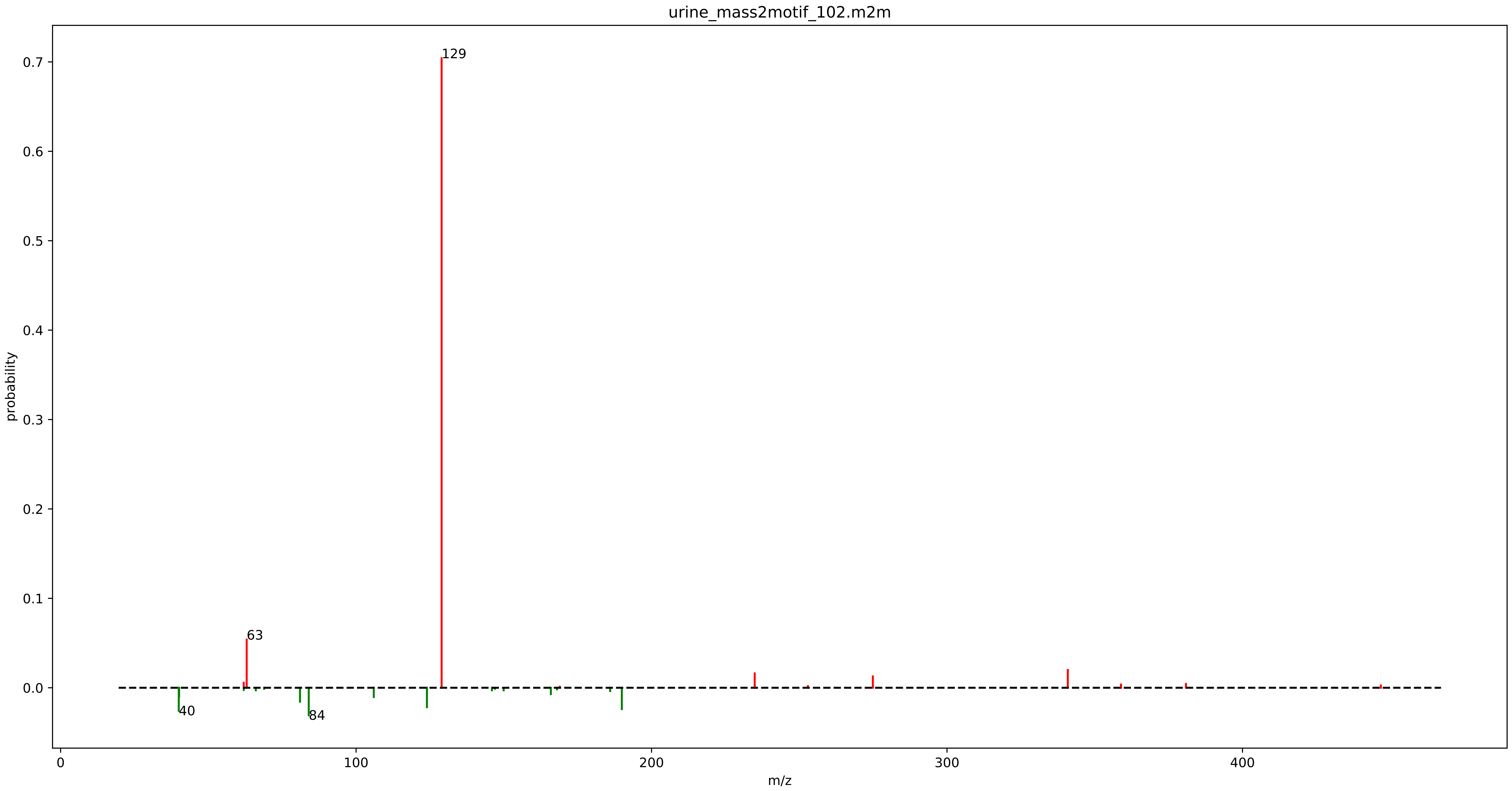
urine_mass2motif_102.m2m
probability
m/z
0.7
0.6
0.5
0.4
0.3
0.2
0.1
0.0
0
100
200
300
400
129
63
40
84

urine_mass2motif_102.m2m

fragment_128.9525	: 0.70
fragment_62.9825	: 0.05
loss_83.9825	: 0.03
loss_39.9925	: 0.03
loss_189.9475	: 0.02
loss_123.9775	: 0.02
fragment_340.8825	: 0.02
fragment_234.9175	: 0.02
loss_81.0225	: 0.02
fragment_274.9075	: 0.01
loss_105.9975	: 0.01
loss_40.1075	: 0.01
loss_165.9175	: 0.01
loss_124.0025	: 0.01
fragment_61.9725	: 0.01

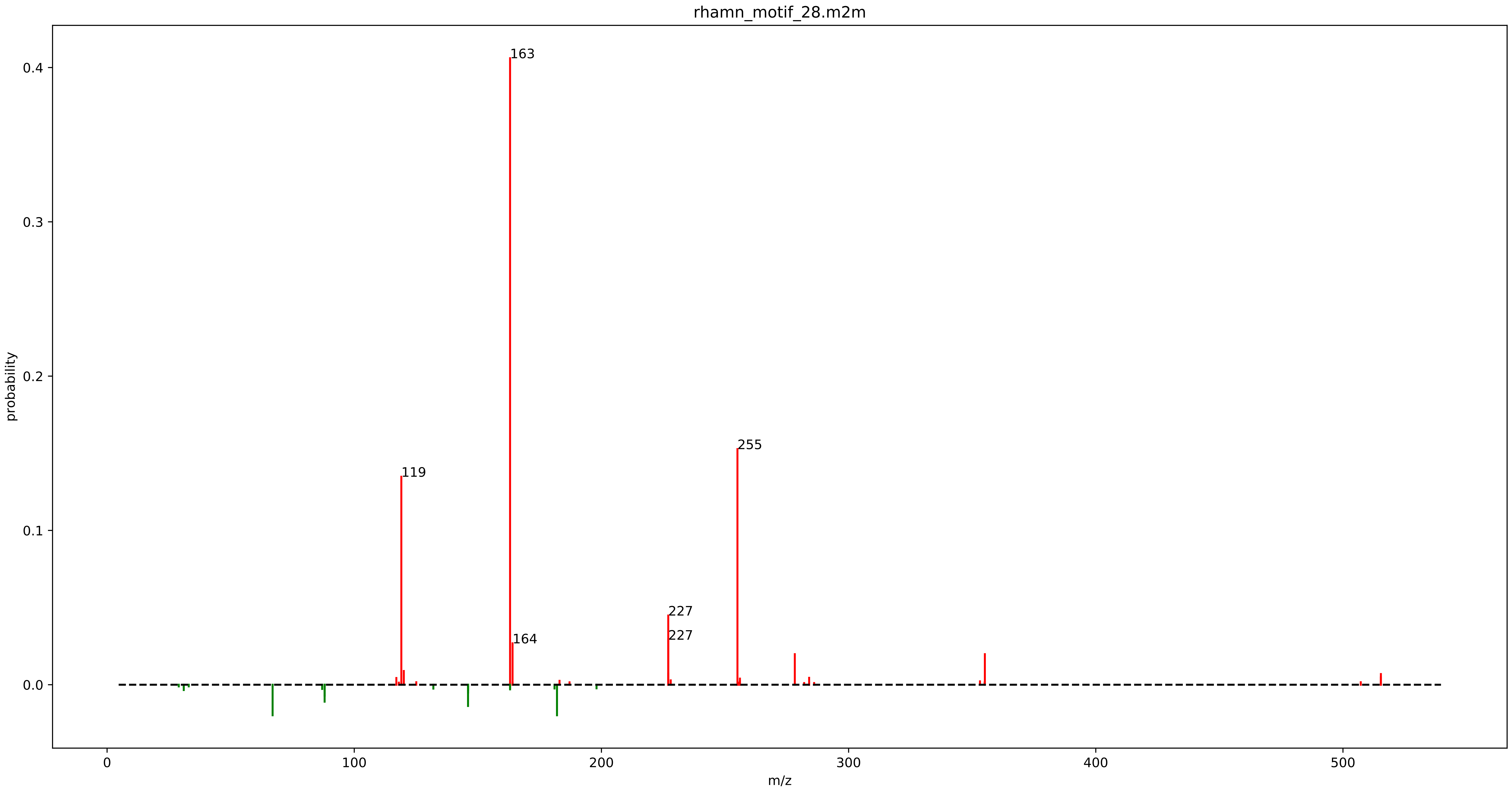
rhamn_motif_28.m2m
probability
m/z
0.4
0.3
0.2
0.1
0.0
0
100
200
300
400
500
163
119
255
227
227
164

rhamn_motif_28.m2m

fragment_163.0375	: 0.41
fragment_255.0275	: 0.15
fragment_119.0475	: 0.13
fragment_227.0325	: 0.04
fragment_227.0375	: 0.03
fragment_164.0425	: 0.03
fragment_355.1225	: 0.02
loss_66.9675	: 0.02
loss_182.0225	: 0.02
fragment_278.2325	: 0.02
loss_146.0325	: 0.01
loss_88.0125	: 0.01
fragment_120.0525	: 0.01
fragment_227.0275	: 0.01
loss_88.0225	: 0.01
fragment_515.3425	: 0.01
loss_146.0525	: 0.01

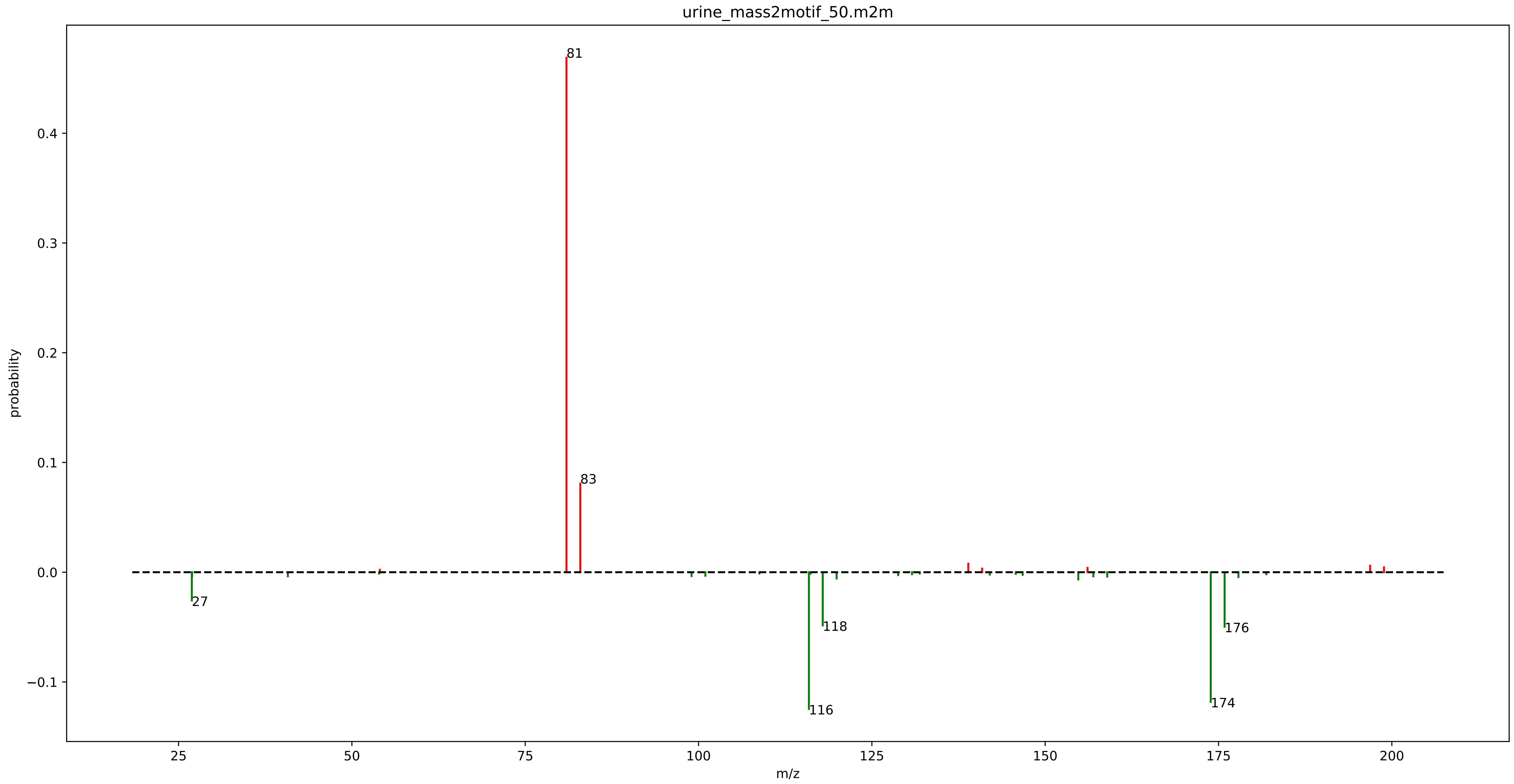

urine_mass2motif_50.m2m
probability
m/z
0.4
0.3
0.2
0.1
0.0
−0.1
25
50
75
100
125
150
175
200
81
83
27
116
118
174
176

urine_mass2motif_50.m2m

fragment_80.9475	: 0.47
loss_115.9175	: 0.12
loss_173.8775	: 0.12
fragment_82.9425	: 0.08
loss_175.8725	: 0.05
loss_117.9125	: 0.05
loss_26.9125	: 0.03
fragment_138.9075	: 0.01
loss_154.7725	: 0.01
fragment_196.8675	: 0.01
loss_119.9125	: 0.01

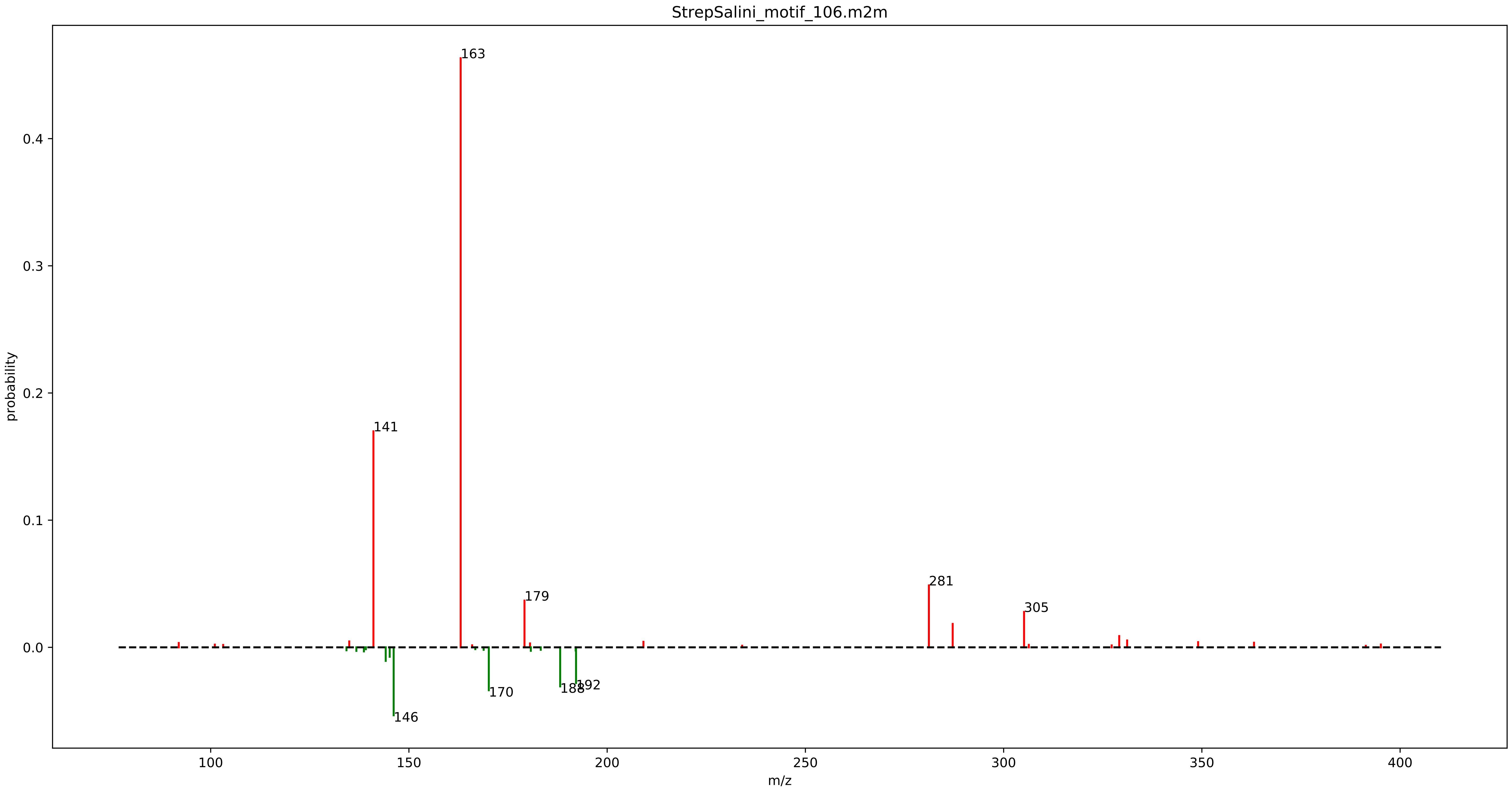

StrepSalini_motif_106.m2m
probability
m/z
163
141
179
281
305
146
170
188
192
0.4
0.3
0.2
0.1
0.0
100
150
200
250
300
350
400

StrepSalini_motif_106.m2m

fragment_163.0500	: 0.46
fragment_141.0500	: 0.17
loss_146.1500	: 0.05
fragment_281.1500	: 0.05
fragment_179.1500	: 0.04
loss_170.1525	: 0.03
loss_188.1500	: 0.03
loss_192.1525	: 0.03
fragment_305.1525	: 0.03
fragment_287.1500	: 0.02
loss_144.1500	: 0.01
fragment_329.1500	: 0.01
loss_145.1500	: 0.01
fragment_331.1500	: 0.01

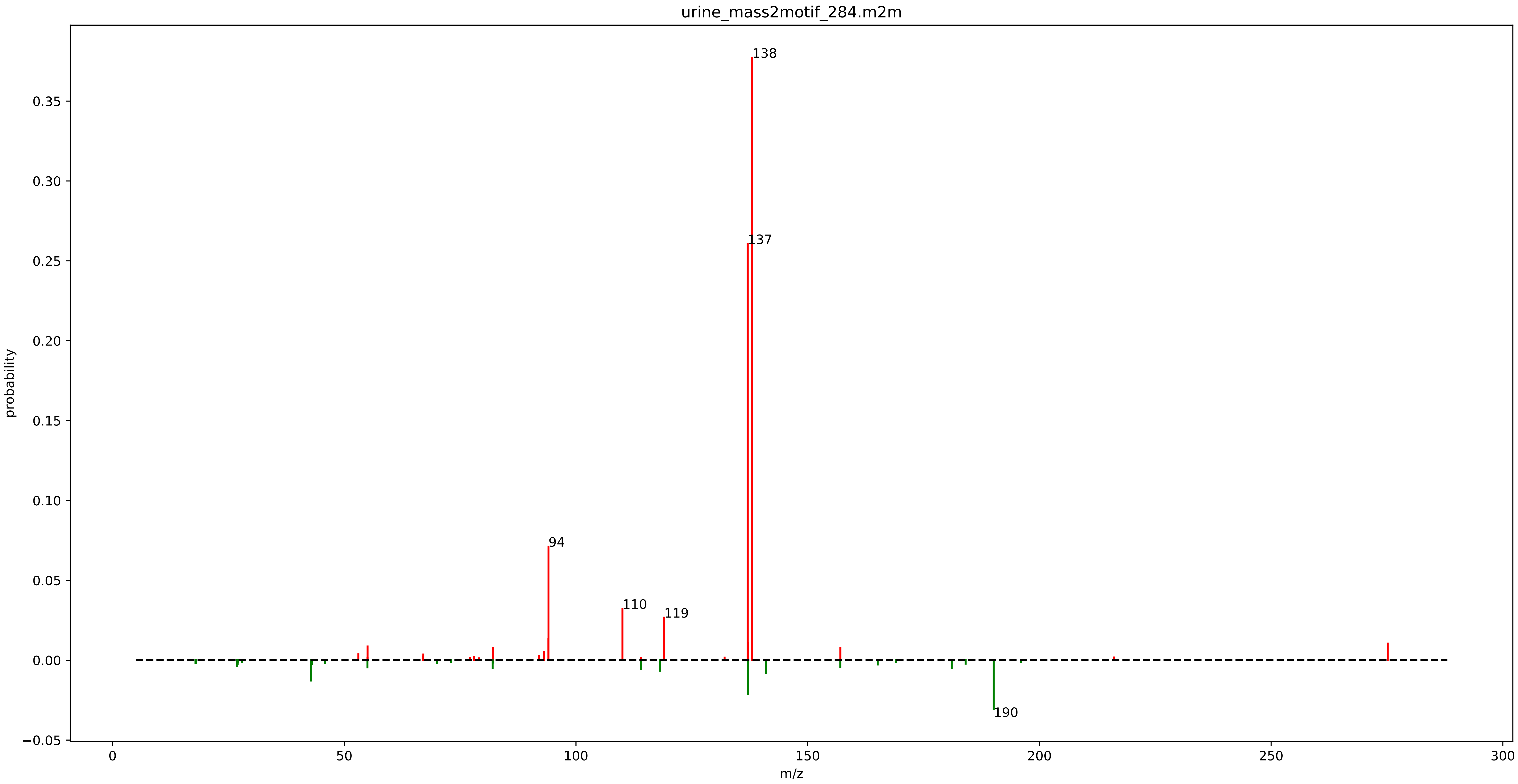

urine_mass2motif_284.m2m
138
137
94
110
119
190
probability
0.35
0.30
0.25
0.20
0.15
0.10
0.05
0.00
−0.05
0
50
100
150
200
250
300
m/z

urine_mass2motif_284.m2m

fragment_138.0525	: 0.38
fragment_137.0475	: 0.26
fragment_94.0675	: 0.07
fragment_110.0325	: 0.03
loss_190.1325	: 0.03
fragment_119.0375	: 0.03
loss_137.1025	: 0.02
fragment_94.0425	: 0.01
loss_42.8675	: 0.01
fragment_137.0725	: 0.01
fragment_275.1575	: 0.01
fragment_55.0275	: 0.01
loss_141.0375	: 0.01
fragment_157.0525	: 0.01
fragment_82.0425	: 0.01
fragment_137.1075	: 0.01
loss_118.1125	: 0.01
loss_114.0825	: 0.01
fragment_93.0575	: 0.01

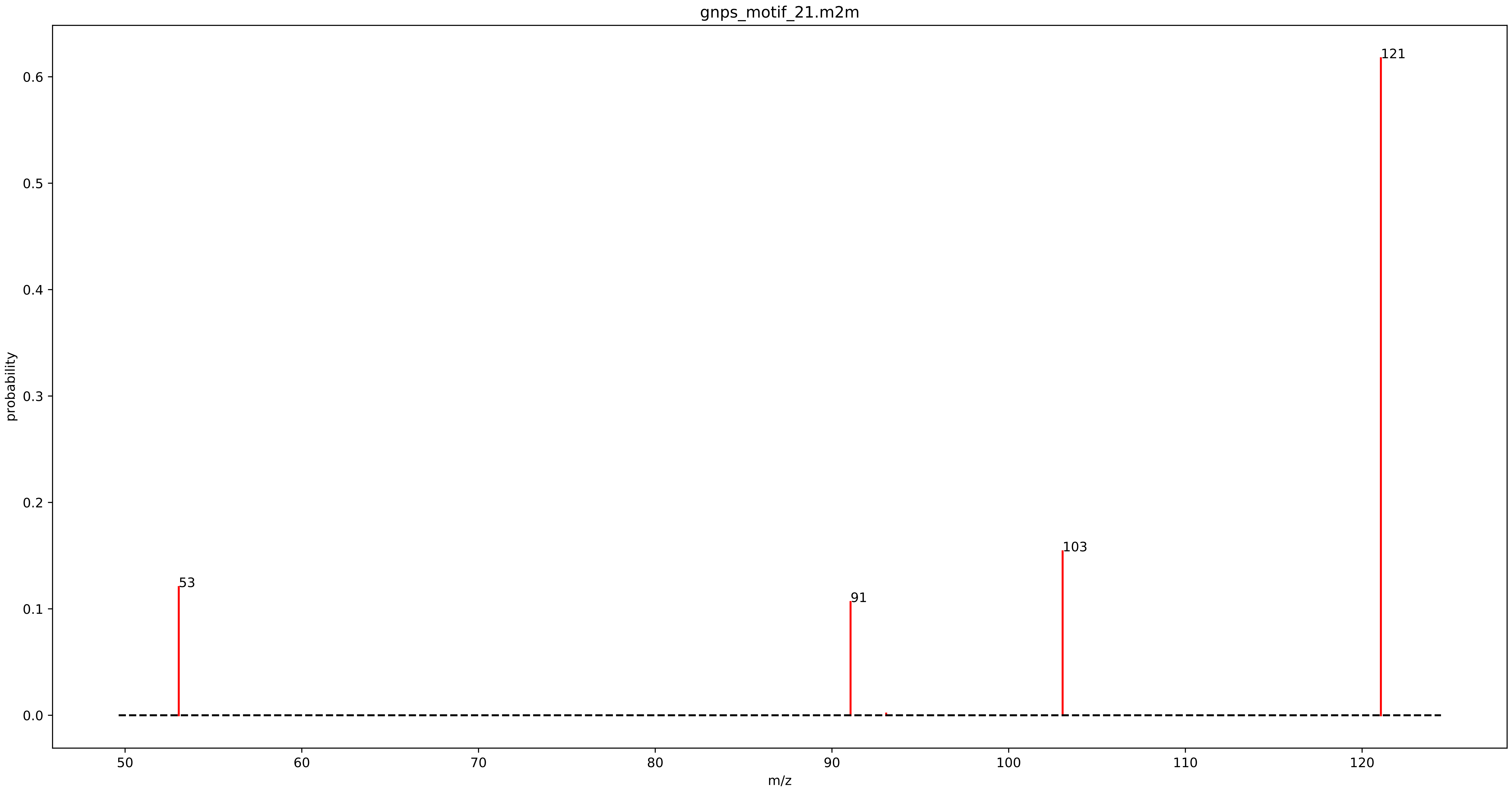

gnps_motif_21.m2m
probability
m/z
0.0
0.1
0.2
0.3
0.4
0.5
0.6
50
60
70
80
90
100
110
120
53
91
103
121

gnps_motif_21.m2m

fragment_121.0625	: 0.62
fragment_103.0525	: 0.15
fragment_53.0375	: 0.12
fragment_91.0525	: 0.11

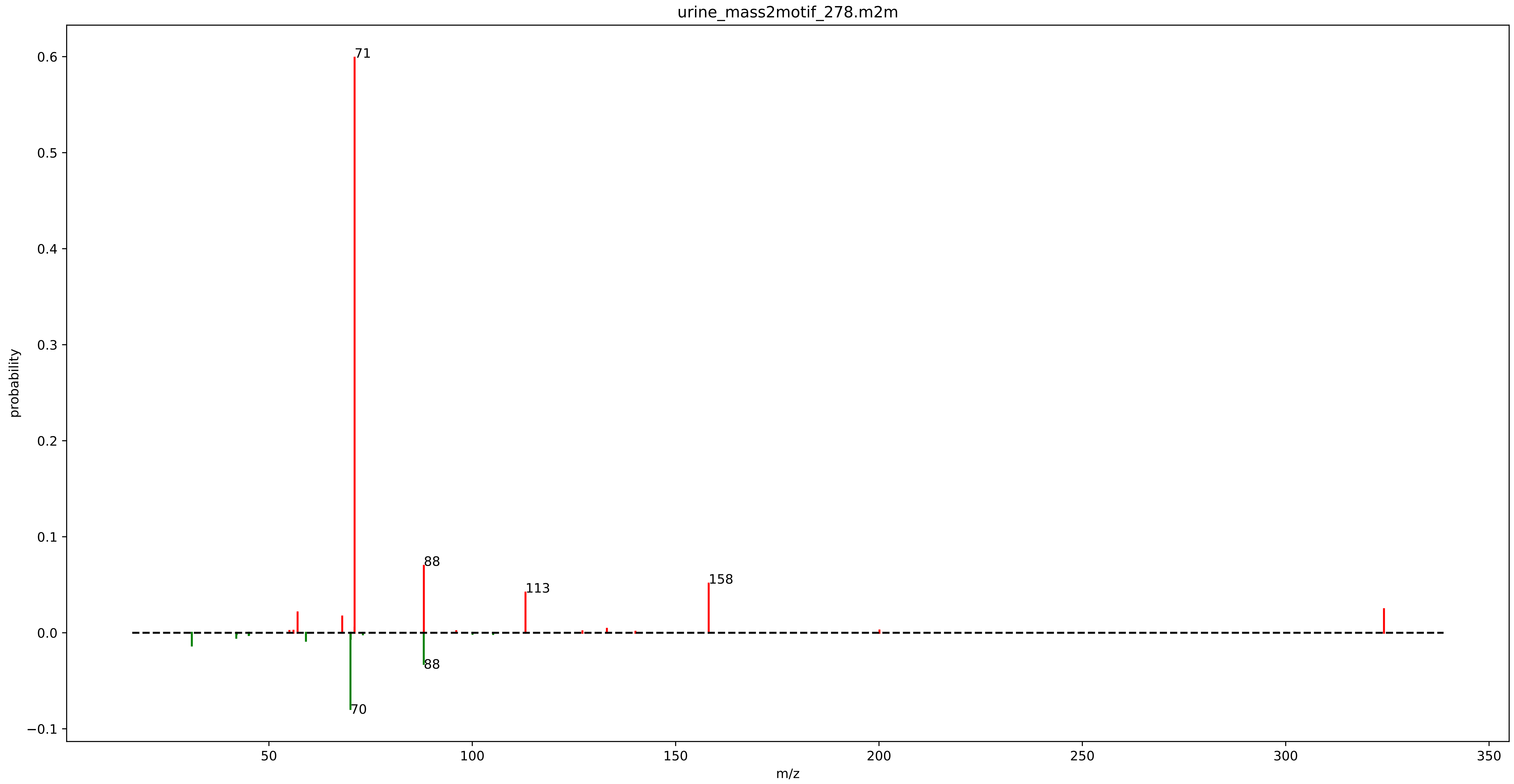

urine_mass2motif_278.m2m
probability
m/z
0.6
0.5
0.4
0.3
0.2
0.1
0.0
−0.1
50
100
150
200
250
300
350
71
88
113
158
88
70

urine_mass2motif_278.m2m

fragment_71.0625	: 0.60
loss_70.0475	: 0.08
fragment_88.0875	: 0.07
fragment_158.1275	: 0.05
fragment_113.0825	: 0.04
loss_88.0675	: 0.03
fragment_324.1625	: 0.02
fragment_57.0475	: 0.02
fragment_68.0225	: 0.02
loss_31.0425	: 0.01
loss_59.1025	: 0.01
loss_70.1025	: 0.01
fragment_71.0475	: 0.01
loss_41.9725	: 0.01

urine_mass2motif_83.m2m

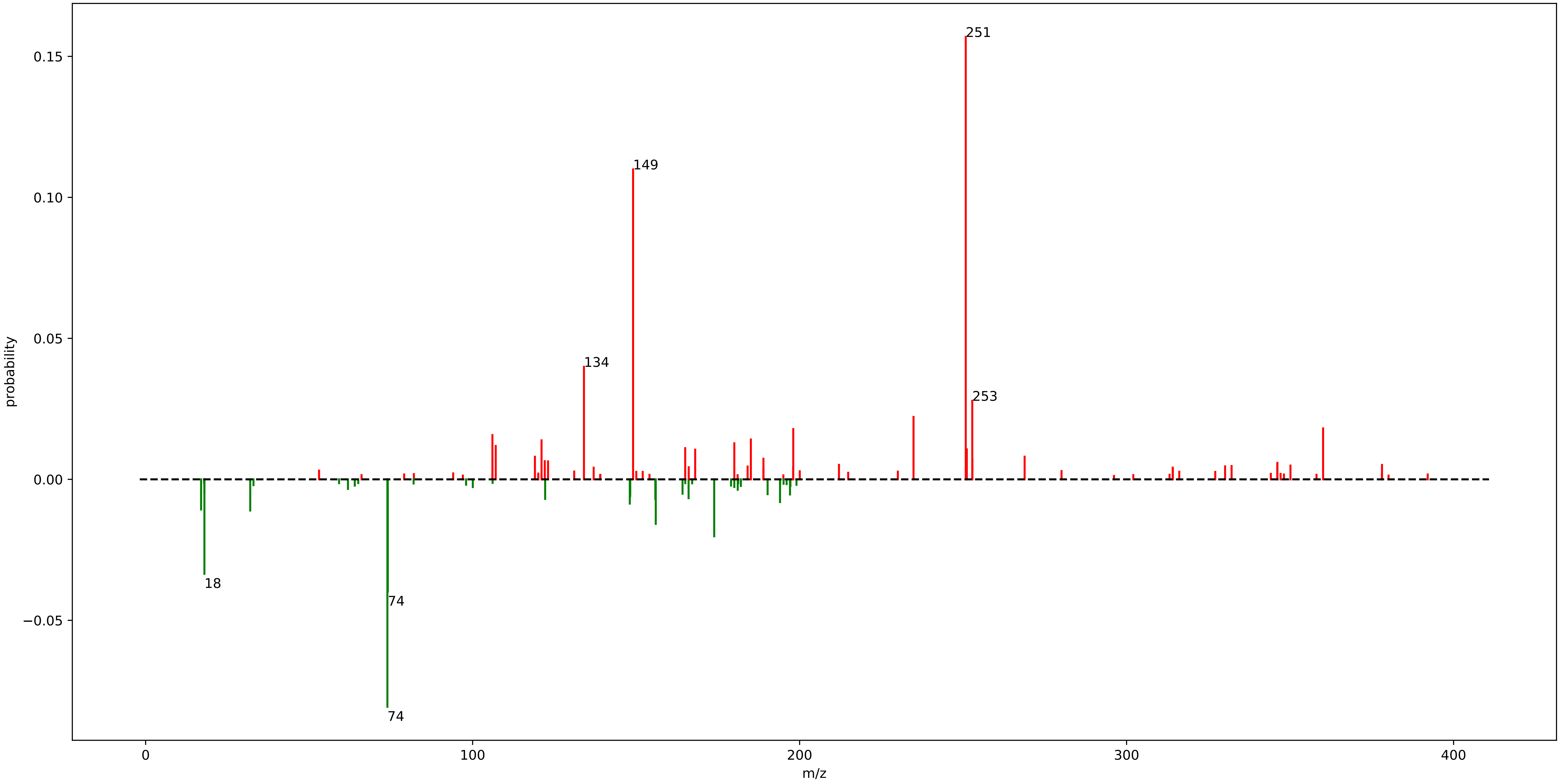

urine_mass2motif_83.m2m

fragment_250.8075 : 0.16
fragment_149.0725 : 0.11
loss_73.9325 : 0.08
fragment_134.0475 : 0.04
loss_74.0575 : 0.04
loss_17.9725 : 0.03
fragment_252.8075 : 0.03
fragment_234.8375 : 0.02
loss_173.8875 : 0.02
fragment_360.0975 : 0.02
fragment_198.0575 : 0.02
loss_156.0075 : 0.02
fragment_106.0525 : 0.02
fragment_185.0925 : 0.01
fragment_121.0775 : 0.01
fragment_180.0325 : 0.01
fragment_107.0625 : 0.01
fragment_165.0125 : 0.01
loss_31.9875 : 0.01
loss_16.9625 : 0.01
fragment_251.1375 : 0.01
fragment_168.0625 : 0.01
loss_148.0675 : 0.01
loss_194.0125 : 0.01
fragment_268.8175 : 0.01
fragment_119.0575 : 0.01
fragment_106.0675 : 0.01
fragment_188.9375 : 0.01
fragment_252.8425 : 0.01
loss_122.1825 : 0.01
loss_155.8775 : 0.01
loss_166.0475 : 0.01
fragment_122.0625 : 0.01
fragment_123.0675 : 0.01
loss_148.1625 : 0.01
fragment_346.1225 : 0.01
fragment_346.0875 : 0.01
loss_197.0675 : 0.01
loss_148.2025 : 0.01
loss_190.2125 : 0.01
fragment_212.0375 : 0.01
fragment_378.1125 : 0.01
loss_164.1975 : 0.01

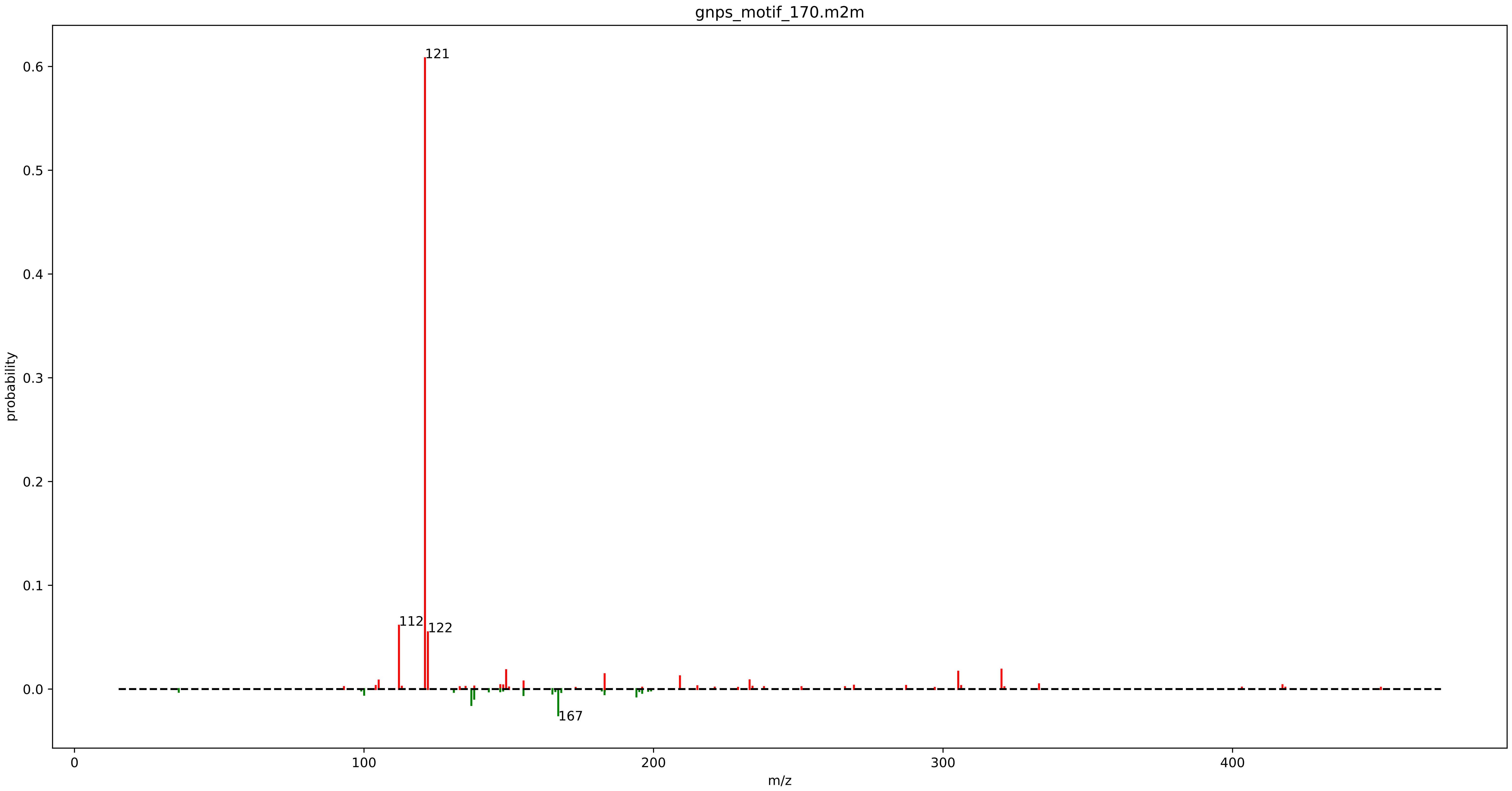
gnps_motif_170.m2m
probability
m/z
121
112
122
167
0.6
0.5
0.4
0.3
0.2
0.1
0.0
0
100
200
300
400

gnps_motif_170.m2m

fragment_121.0675	: 0.61
fragment_112.0775	: 0.06
fragment_122.0675	: 0.05
loss_167.0925	: 0.03
fragment_320.1975	: 0.02
fragment_149.0975	: 0.02
fragment_305.2475	: 0.02
loss_137.0825	: 0.02
fragment_183.1125	: 0.01
fragment_209.1275	: 0.01
loss_138.0675	: 0.01
fragment_233.1925	: 0.01
fragment_105.0725	: 0.01
fragment_155.1175	: 0.01
loss_194.1025	: 0.01
loss_155.0925	: 0.01
loss_100.0525	: 0.01

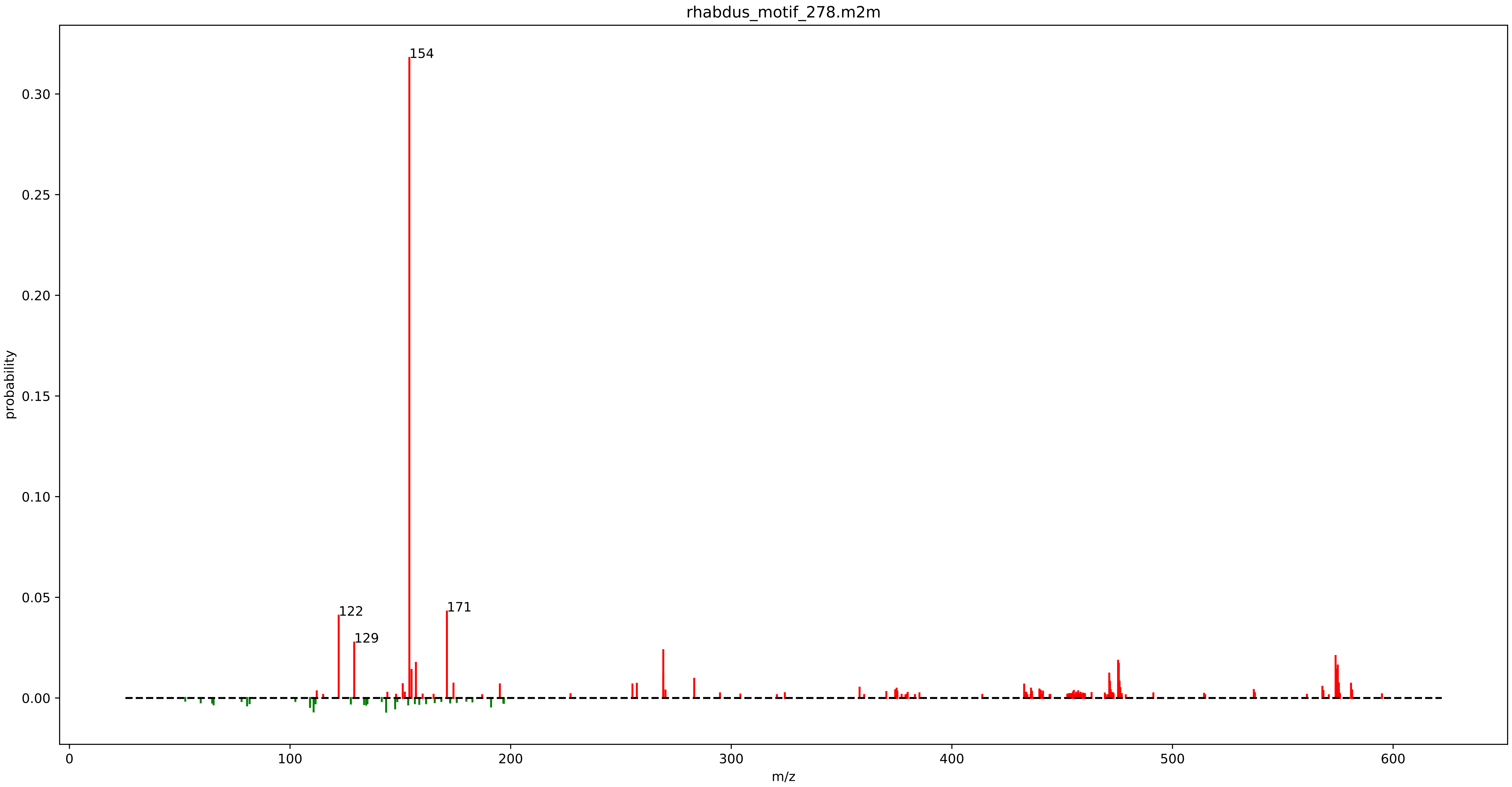

rhabdus_motif_278.m2m
probability
m/z
154
122
129
171
0.30
0.25
0.20
0.15
0.10
0.05
0.00
0
100
200
300
400
500
600

rhabdus_motif_278.m2m

fragment_154.0875	: 0.32
fragment_171.1125	: 0.04
fragment_122.0575	: 0.04
fragment_129.0875	: 0.03
fragment_269.1875	: 0.02
fragment_573.9225	: 0.02
fragment_475.3375	: 0.02
fragment_157.0625	: 0.02
fragment_475.6725	: 0.02
fragment_574.9275	: 0.02
fragment_574.4225	: 0.01
fragment_155.0875	: 0.01
fragment_471.3375	: 0.01
fragment_269.1825	: 0.01
fragment_283.2025	: 0.01
fragment_476.0075	: 0.01
fragment_471.6725	: 0.01
fragment_575.4275	: 0.01
fragment_174.0925	: 0.01
fragment_580.9275	: 0.01
fragment_257.1975	: 0.01
fragment_151.0875	: 0.01
loss_143.5575	: 0.01
fragment_195.1125	: 0.01
fragment_255.2075	: 0.01
fragment_475.3325	: 0.01
fragment_432.7575	: 0.01
loss_110.6875	: 0.01
fragment_567.9225	: 0.01
loss_147.6425	: 0.01
fragment_358.1625	: 0.01
